# A probabilistic generative model for quantification of DNA modifications enables analysis of demethylation pathways

**DOI:** 10.1186/s13059-016-0911-6

**Published:** 2016-03-14

**Authors:** Tarmo Äijö, Yun Huang, Henrik Mannerström, Lukas Chavez, Ageliki Tsagaratou, Anjana Rao, Harri Lähdesmäki

**Affiliations:** Department of Computer Science, Aalto University School of Science, Aalto, FI-00076 Finland; Present address: Simons Center for Data Analysis, Simons Foundation, New York, NY 10010 USA; La Jolla Institute for Allergy and Immunology, La Jolla, CA 92037 USA; Sanford Consortium for Regenerative Medicine, La Jolla, CA 92037 USA; Present address: Institute of Biosciences & Technology, Texas A&M University Health Science Center, 2121 W. Holcombe Blvd, Houston, TX 77030 USA; Present address: Division of Pediatric Neurooncology, German Cancer Research Center (DKFZ), Heidelberg, 69120 Germany; Department of Pharmacology and Moores Cancer Center, University of California, La Jolla, CA 92037 USA; Turku Centre for Biotechnology, University of Turku and Åbo Akademi University, Turku, FI-20520 Finland; Department of Signaling and Gene Expression, La Jolla Institute for Allergy and Immunology, 9420 Athena Circle, San Diego, CA 92037 USA

**Keywords:** DNA methylation, Bayesian analysis, Hierarchical modeling, TET proteins, 5-methylcytosine oxidation, Bisulfite sequencing, BS-seq/oxBS-seq/TAB-seq/fCAB-seq/CAB-seq/redBS-seq/MAB-seq

## Abstract

We present a generative model, Lux, to quantify DNA methylation modifications from any combination of bisulfite sequencing approaches, including reduced, oxidative, TET-assisted, chemical-modification assisted, and methylase-assisted bisulfite sequencing data. Lux models all cytosine modifications (C, 5mC, 5hmC, 5fC, and 5caC) simultaneously together with experimental parameters, including bisulfite conversion and oxidation efficiencies, as well as various chemical labeling and protection steps. We show that Lux improves the quantification and comparison of cytosine modification levels and that Lux can process any oxidized methylcytosine sequencing data sets to quantify all cytosine modifications. Analysis of targeted data from Tet2-knockdown embryonic stem cells and T cells during development demonstrates DNA modification quantification at unprecedented detail, quantifies active demethylation pathways and reveals 5hmC localization in putative regulatory regions.

**Electronic supplementary material:**

The online version of this article (doi:10.1186/s13059-016-0911-6) contains supplementary material, which is available to authorized users.

## Background

Many biological processes, including X-chromosome inactivation [1], gene imprinting [2] and genomic instability [3] are controlled by cytosine methylation, the most widely studied epigenetic modification of DNA [4]. In mammals, the bulk of DNA methylation in somatic cells occurs as 5-methylcytosine (5mC), typically in a CpG sequence context. DNA methylation is dynamically altered during normal development and abnormal changes have been described in disease [5]. For instance, DNA methylation is thought to contribute to cancer development by diminishing genome stability and suppressing the expression of tumor-suppressor genes [6]. Comparison of different cell types, including human embryonic stem cells and fetal fibroblasts [7], has revealed differential methylation at tissue-specific enhancers in various mouse [8] and human [9] tissues, linking methylation to cell development and differentiation [7–9]. DNA methylation has also been mechanistically linked to splicing regulation through inhibition of CTCF binding [10]. DNA methylation is also generally believed to have a repressive effect at regulatory regions, although transcriptional regulators can also selectively bind methylated and unmethylated DNA [11]. Finally, DNA methylation has been observed to accumulate during mammalian brain development [12] and decrease during aging [13]. For all these reasons, it is important to quantify 5mC changes accurately during embryonic development, cell differentiation and oncogenesis.

Proteins of the TET (Ten-eleven translocation) family were shown to be dioxygenases that converted 5mC to 5-hydroxymethylcytosine (5hmC), 5-formylcytosine (5fC) and 5-carboxylcytosine (5caC) [14, 15]. These oxidized methylcytosine (oxi-mC) species have multiple functions as intermediates in DNA demethylation (5mC → C) as well as stable epigenetic marks that recruit chromatin regulators and interact with RNA polymerase [16–20]. However, the discovery that oxi-mC modifications occur naturally in mammalian DNA has complicated the analysis of DNA methylation. Initially, affinity-based methods were used to map the location of 5mC and 5hmC in genomic DNA, including immunoprecipitation of methylated and hydroxymethylated DNA using antibodies to 5-methylcytosine (MeDIP), 5-hydroxymethylcytosine (hMeDIP), or cytosine 5-methylenesulfonate (CMS, the adduct formed by reaction of sodium bisulfite with 5hmC [21–23]), or biotinylation of 5hmC using sodium periodate (GLIB) [24] or click chemistry [25]. However, there are many obvious advantages to mapping 5mC and oxi-mC at single base resolution. First, compared with affinity-based methods, which show a strong density bias [26], single-base resolution methods are more sensitive at detecting 5hmC in regions of low density CpGs. Second, if performed at high sequence coverage, single-base resolution methods are more sensitive at detecting minor and dynamic changes of oxi-mC, which are likely to be important in many different biological processes. Third, single-base resolution methods can detect localized dynamic changes of oxi-mC, such as oscillating distribution of 5hmC around CTCF binding sites and its correlation with nucleosome positioning [27, 28]. Fourth, single-base resolution methods can detect strand-specific modifications that might be associated with transcriptional activity.

For many years, the most widely used method for quantification of DNA methylation at a single-base level was bisulfite sequencing (BS-seq), the gold standard for methylation profiling [7–9, 12, 29]. Unlike affinity-based approaches, BS-seq provides methylation information at the single-nucleotide resolution by introducing single nucleotide changes into DNA sequence in a methylation-dependent manner [30]. Briefly, treatment of genomic DNA with sodium bisulfite results in rapid deamination of unmodified cytosine to uracil, which is read as thymine after PCR amplification and sequencing (C → T conversion). In contrast, 5mC is deaminated much more slowly, and so remains unconverted and is read as C. Unfortunately, bisulfite sequencing has proved inadequate to detect oxi-mCs: 5hmC reacts with sodium bisulfite to form a new adduct, cytosine 5-methylenesulfonate [21], that is resistant to deamination like 5mC, whereas 5fC and 5caC are prone to deamination like unmodified C. Thus, bisulfite sequencing cannot distinguish 5mC and 5hmC, which are both read as C after PCR amplification, nor can it distinguish unmodified C from 5fC or 5caC, which are all read as T [31].

The previously reported contradictory functions of 5mC in gene regulation [7] are partly due to the inability of BS-seq to distinguish 5hmC from 5mC. To overcome the limitations of BS-seq, oxBS-seq (oxidative bisulfite sequencing) [32] and TAB-seq (Tet-assisted bisulfite sequencing) [28] have been developed to differentiate 5hmC from 5mC at a single nucleotide level. Both techniques use oxidation; KRuO_4_ oxidizes 5hmC to 5fC in oxBS-seq [32], whereas in TAB-seq, 5hmC is protected by β-glucosyltransferase and recombinant mouse *Tet1* is used to oxidize 5mC to 5caC [28]. Importantly, oxBS-seq and TAB-seq have to be combined with BS-seq in order to distinguish C, 5mc and 5hmC and to quantify their levels. Recently, several new sequencing protocols have been developed to quantify further oxidized methylcytosines in DNA (reviewed in [33]). In fCAB-seq (5fC chemical modification-assisted bisulfite sequencing) [34], *O*-ethylhydroxylamine (Et-ONH_2_) modifies 5fC and protects it from deamination by sodium bisulfite, whereas NaBH_4_ reduces 5fC to 5hmC in redBS-seq (reduced bisulfite sequencing) [35]. Subsequent sequencing of modified or reduced 5fC in fCAB-seq and redBS-seq, respectively, reads 5fC similarly with 5mC and 5hmC. Thus, quantification of 5fC becomes possible when fCAB-seq or redBS-seq data are combined with the standard BS-seq data obtained from the same sample. Similarly with fCAB-seq, in CAB-seq (chemical modification-assisted bisulfite sequencing) [36] 1-ethyl-3-[3-dimethylaminopropyl]-carbodiimide hydrochloride (EDC) selectively protects 5caC from deamination during bisulfite treatment and quantification of 5caC requires CAB-seq to be combined with BS-seq data. In MAB-seq (M.SssI methylase-assisted bisulfite sequencing) [37], unmethylated C is methylated with the bacterial DNA CpG methyltransferase M.SssI. Sequencing of the M.Sssl and sodium bisulfite treated DNA then discriminates 5fC and 5caC from other DNA methylation modifications. All aforementioned methods are challenging and sensitive to variation in various experimental steps, often resulting in sample-specific biases. Moreover, although TAB-seq or oxBS-seq selectively detect 5hmC and 5mC, respectively, in all other methods several modifications are convoluted and, thus, the underlying true modification levels, or proportions, need to be computationally inferred from a combination of these data sets.

Various computational methods exist for analyzing BS-seq data from Sanger and high-throughput sequencing — for instance, QUMA [38], BISMA [39], methylKit [40], GBSA [41], BSmooth [42], MOABS [43], a Bayesian hierarchical model [44], MethylSeekR [45], and RadMeth [46]. These methods provide means to quantify levels of methylation, visualize data and detect differential methylation. Depending on the biological question, the quantification of methylation is done either at individual cytosines, in sliding window fashion or for predefined genomic regions, such as promoters, CpG islands or shores. Earlier methods (e.g., QUMA, methylKit) make no specific statistical assumptions about data characteristics, whereas BSmooth models the distribution of converted and unconverted cytosine counts with binomial distribution, which was extended to a hierarchical beta-binomial model in MOABS and other methods [43–46] to account for biological variation. Different measures have been proposed for calling differential methylation — for instance, Fisher’s exact test [7] on the counts of converted and unconverted cytosines, Mann–Whitney *U*-test [38] or a modified *t*-test [42] on methylation profiles, and the credible methylation difference metric calculated between methylation level distributions [43]. Although these methods are applicable for analyzing BS-seq/oxBS-seq data separately, they lack support for integrative analysis of different methylation states (the percentages of which need to add up to 100 %) from BS-seq and oxBS-seq data. Consequently, the previously proposed methods use a naïve integration, such as subtraction, of the individual methylation state estimates, which is prone to erroneous estimates. Recently, the MLML method was published, which provides consistent methylation (non-negative and adds up to 100 %) estimates from BS-seq, oxBS-seq, and TAB-seq data using the expectation maximization algorithm [47]. However, no method exists to analyze other oxi-mC-seq data (other than simple subtraction of read counts) and, importantly, previous methods do not take into account experiment-specific variation in the biochemistry. These non-ideal experimental parameters include, e.g., bisulfite conversion, oxidation efficiencies, chemical labeling and protection steps and sequencing errors, and their experimental significance has been demonstrated [28, 32, 34, 37]. Notable exceptions include the computational methods introduced in [12, 28, 32] which use the binomial test together with a conversion inefficiency parameter to quantify the significance (i.e., *p* value) of 5mC > 0 and 5hmC > 0. However, the use of these early methods is limited as they provide neither a way to accurately quantify cytosine modification levels nor a method to assess differential methylation.

To study active demethylation and to characterize unknown functions of oxi-mC species, a rigorous statistical analysis of BS-seq and oxi-mC-seq data is needed for accurate quantification of different cytosine modifications and detection of differential methylation between conditions. To fill this gap we present an integrative hierarchical model, Lux, which is inspired by the aforementioned measurement processes. This probabilistic generative model enables accurate and unbiased quantification of different cytosine modifications and differential methylation at individual cytosines or loci, with or without replicates, while taking imperfect and sample-specific experimental parameters into account. Full Bayesian inference quantifies the effect of the uncertainties in data and parameters to the final estimates. Lux is applicable for analyzing any number and combination of BS-seq and oxi-mC-seq data sets from whole genome, reduced representation or targeted experiments, and provides the most accurate methylome estimates when samples are spiked-in with stretches of unmethylated and methylated (5mC, 5hmC, 5fC, and/or 5caC) control DNAs. These features were benchmarked extensively on real and simulated data, including BS-seq, oxBS-seq, TAB-seq, and fCAB-seq. We also show that the statistical framework is easily extended for other existing data types, such as CAB-seq, redBS-seq, and MAB-seq, as well as upcoming derivatives of traditional bisulfite sequencing. A platform-independent implementation of Lux is released under MIT license at https://github.com/tare/Lux/ and as Additional files 1 and 2.

## Results and discussion

### Method overview

**Fig. 1:**
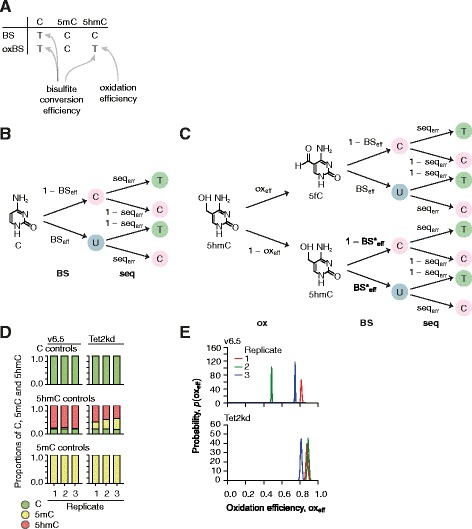
The effect of imperfect bisulfite conversion and oxidation efficiencies on BS-seq and oxBS-seq assays. **a** The read-outs for C, 5mC and 5hmC in BS-seq and oxBS-seq assays. The *arrows* indicate which read-outs are affected by bisulfite conversion and/or oxidation efficiencies. **b** The bisulfite conversion of C followed by sequencing. The four possible scenarios of sequencing “C” or “T” are expressed in terms of BS_eff_ and seq_err_. Oxidation does not have an effect on C so this model also applies to the oxBS-seq measurement of C. **c** The oxidation of 5hmC followed by bisulfite treatment and sequencing. The eight possible scenarios of sequencing “C” or “T” are expressed, stated in terms of BS_eff_, BS*_eff_, ox_eff_ and seq_err_. Under bisulfite treatment, without the preceding oxidation step, 5hmC and 5mC react in the same way. **d** The posterior mean methylation proportions across the control loci in v6.5 (*left panel*) and Tet2kd (*right panel*) samples. The different replicates are in the columns. The bars show the arithmetic means of the posterior means of the individual cytosines. The one-sided error bars (mean – standard deviation is depicted) show the standard deviations. **e** Posterior distributions of oxidation efficiencies across biological conditions (v6.5 in *top panel*; Tet2kd in *bottom panel*) and replicates. Kernel density estimates with the Gaussian kernel (the bandwidth obtained with Scott’s rule) are shown

We first describe how Lux can be applied to simultaneously analyze C (together with 5fC and 5caC), 5mC and 5hmC from BS-seq and oxBS-seq data, and later extend Lux to other data types. BS-seq and oxBS-seq provide partially orthogonal, but convoluted, information on methylation status (Fig. 1a) as BS-seq reads discriminate 5mC and 5hmC from C whereas oxBS-seq reads discriminate 5mC from C and 5hmC. Thus, together they provide the data required for quantifying levels of C, 5mC and 5hmC. Two straightforward approaches for quantifying 5hmC levels from BS-seq and oxBS-seq data calculate the difference in proportions of unconverted cytosines [32] or the difference of separately estimated proportions [43], respectively, resulting in unconstrained maximum likelihood estimates (termed as frequency method; see Additional file 3). Unfortunately, both approaches can lead to erroneous estimates, such as negative values for 5hmC, because the cytosine modification levels are tightly interconnected. Moreover, the read-outs from BS-seq and oxBS-seq assays depend on the efficiencies of bisulfite conversion and oxidation (Fig. 1a).

We considered the following experimental parameters: bisulfite conversion (BS_eff_), inaccurate bisulfite conversion (BS*_eff_; Figure S1a in Additional file 4), oxidation (ox_eff_) efficiencies and sequencing errors (seq_err_). To quantify the C, 5mC and 5hmC proportions from BS-seq and oxBS-seq data while taking into account the experimental parameters; we formulated a probabilistic generative model (see "Materials and methods"). First, for each cytosine modification, we write the probabilities of the BS-seq and oxBS-seq outcomes in the terms of experimental parameters BS_eff_, BS*_eff_, ox_eff_ and seq_err_ (Fig. 1b, c; Figure S1b, c in Additional file 4). Next, we modeled cytosine-specific methylation states (C, 5mC and 5hmC) with cytosine-specific probabilities θ = [p(C), p(5mC), p(5hmC)] (Σθ = 1) and weighted the probabilities of the BS-seq and oxBS-seq outcomes with the proportions in θ (Additional file 4: Figure S1d; see "Materials and methods"). Consequently, the BS-seq and oxBS-seq outcomes are Bernoulli distributed with the aforementioned weighted and summed success probabilities; moreover, the frequencies of the sequencing read-outs are binomially distributed (Figure S2 in Additional file 4). The capability of analyzing data from repeated biological experiments in Lux is implemented by adding a hierarchical level for modeling biological variation between the replicate-specific proportions θ_i_ from the common proportions μ (Figure S2 in Additional file 4; see "Materials and methods"). The statistical model is described in more detail in "Materials and methods" and in full detail in Additional file 3.

Bayesian inference of the model yields posterior distributions of the model parameters conditioned on data (see "Materials and methods"). This starts by specifying prior distributions on the model parameters (Figure S2 in Additional file 4; Table S1 in Additional file 5; see "Materials and methods"). The model inference was implemented in Stan, which utilizes the Hamiltonian MCMC strategy (HMC) with the No-U-turn sampler (NUTS) for estimating posterior distributions with fast convergence [48] (see "Materials and methods"). In practice, Lux with the model suitable for distributed computing (Figure S2b in Additional file 4) is able to analyze approximately 15,000 cytosines for a single replicate in an hour on a single core (Figure S2c in Additional file 4). Notice that run time requirement with respect to replicates increases sub-linearly. Thus, as demonstrated in this study by utilizing a computing cluster, one can analyze all cytosines in a CpG context in mammalian genomes in several hours, therefore rendering Lux applicable for integrative analysis of oxi-mC data with or without replicates in a genome-wide setting.

### Estimation of experimental parameters

We focused on 14 previously studied genomic loci covering approximately 2000 cytosines in wild-type (v6.5) and *Tet2* knockdown (Tet2kd) v6.5 embryonic stem cells [49] and carried out targeted BS and oxBS sequencing with three biological replicates. Ten of the selected loci were highly statistically significantly differentially methylated and had varying methylation states based on the previous mapped 5hmC and 5mC methylomes [49] obtained using CMS-IP (cytosine-5-methylenesulfonate immunoprecipitation) and MeDIP (methylated DNA immunoprecipitation) antibody techniques. Four of the loci showed no differential methylation [49]. The obtained high coverage (median cytosine coverage 2042×) data sets on the selected loci provided an ideal backdrop for assessing the applicability of Lux, and for comparing Lux’s accuracy with that of other methods. To estimate the bisulfite conversion rates and oxidation efficiencies, the sequencing libraries were spiked with stretches of unmethylated, methylated and hydroxymethylated DNAs (see "Materials and methods"). The Cs and 5mCs in the control DNA are close to 100 % unmethylated and methylated, respectively, while 5hmC has ~90 % purity, reflecting the purity of the 5hmCTP obtained from the manufacturer [28]. This prior knowledge was plugged into the model through the prior distributions (Table S1 in Additional file 5; Figure S2a in Additional file 4; see "Materials and methods"). Next the model was conditioned on the data and the posterior distributions of the methylation states of the control DNA (Fig. 1d) and experimental parameters (oxidation efficiencies shown in Fig. 1e; others are listed in Table S2 in Additional file 5) were derived (see "Materials and methods"). C and 5mC controls were close to ideal, whereas 5hmC controls had more experimental variation, presumably because of the impure dhmCTP mix and experimental challenges (Fig. 1d). The small standard deviations of the estimates demonstrate the identifiability of the experimental parameters. The experimental variation in the parameters (ox_eff_ from 0.48 to 0.89) emphasizes the importance of considering them while estimating methylation levels and comparing methylation levels among samples (Fig. 1e). In addition to impure dhmCTP mixes, the amount of 5hmC in the genomic DNA might affect the oxidation efficiency, and thus the higher oxidation efficiency in Tet2kd cells might be due to the reduced amount of 5hmC in Tet2kd cells compared with that in v6.5 cells. Importantly, 5hmC estimates would be underestimated in the v6.5 samples if the sample-specific oxidization efficiency was not taken into account, as implemented in Lux. As expected, other parameters were close to ideal (Table S2 in Additional file 5).

In addition, we carried out an in silico experiment to enable a more controlled evaluation (Figure S3a in Additional file 4). Briefly, we studied the identifiability of the model by testing different settings of experimental parameters, number of control cytosines, and coverage levels. The simulation results also demonstrate a good identifiability of the experimental parameters since they can be estimated even from a single control cytosine (Figure S3b in Additional file 4). Moreover, simulation results suggest that with 20 control cytosines per methylation modification, the experimental parameters can be accurately estimated and their accuracy saturates at 48× coverage (Figure S3c in Additional file 4).

### Estimation of methylation levels

**Fig. 2:**
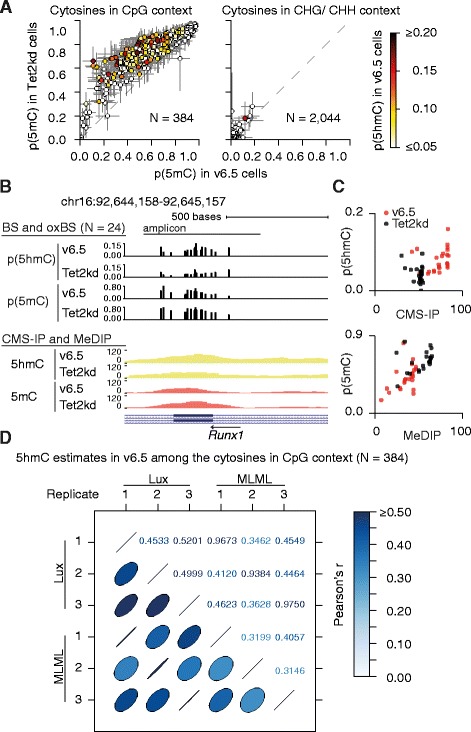
Estimating proportions of DNA methylation modifications. **a** Scatter plot representations of the observed changes in 5mC levels (estimated over three replicates) between wild-type and *Tet2* knock-down cells across all the cytosines in the 14 genomic loci studied. The estimated posterior means and their standard deviations are illustrated. The cytosines are grouped based on their sequence context. The coloring of the circles depicts the estimated 5hmC levels in v6.5. **b** The estimated posterior means of 5hmC and 5mC levels from three replicates per condition are depicted in the bar charts (*top panel*). Only the cytosines in a CpG context and the designed amplicon are depicted. Read density profiles from affinity-based measurements of 5hmC and 5mC levels across the locus chr16:92,644,158-92,645,157 are shown (*bottom panel*). **c** Scatter plot representations of 5hmC levels estimated using CMS-IP and BS-/oxBS-seq (*top panel*) and 5mC using MeDIP and BS-/oxBS-seq (*bottom panel*) measurements. The conditions are set apart by the color. Random jitter has been added to the scatter plot points to avoid overlapping points. **d** A pair-wise comparison between the Lux and MLML replicate-specific 5hmC level estimates. Only the cytosines in a CpG context are considered. The Pearson’s correlation coefficients are shown

The methylation statuses of all the cytosines with at least 10× coverage across all six samples (N = 2428) were estimated (Table S3 in Additional file 5) simultaneously with the estimation of experimental parameters. As expected, there was wide variation in the DNA methylation levels of cytosines in a CpG context (left panel in Fig. 2a), but no 5mC or 5hmC in cytosines located in a non-CpG (CHG/CHH) context (right panel in Fig. 2a). Because TET family proteins oxidize 5mC to 5hmC, 5fC and 5caC, a Tet2kd is expected to block this demethylation pathway and increase the level of 5mC. Notably, 5mC levels for 179 out of 384 cytosines were increased in Tet2kd cells (p_Tet2kd_(5mC) − p_v6.5_(5mC) > 0.1) (Fig. 2a). These cytosines were also highly marked by 5hmC in v6.5 cells (Fig. 2a), suggesting the expected scenario in which *Tet2* depletion resulted in loss of 5hmC and concomitantly increased 5mC. However, the demethylation process is reduced rather than entirely blocked in *Tet2* knock-down mESCs, either because of incomplete depletion and residual TET2 activity or compensatory activity of other TET enzymes, most likely TET1 [49]. More generally, we observed an inverse correlation between 5mC and 5hmC levels (Figure S4 in Additional file 4) reflecting the inherent relationships between cytosine modifications in the active demethylation pathway.

To confirm Lux’s ability to estimate methylation levels, we compared our estimates with published 5hmC and 5mC methylome maps [49] obtained using CMS-IP and MeDIP antibody techniques, respectively. For example, in a methylated locus (*Runx1*), Lux estimated qualitatively similar 5mC and 5hmC levels from BS- and oxBS-seq data before and after *Tet2* depletion (Fig. 2b, top). However, detailed analysis of individual CpGs showed the expected loss of 5hmC and gain of 5mC in *Tet2*-depleted relative to parental v6.5 embryonic stem cells (Fig. 2c), confirming previous conclusions from comparisons of CMS-IP and MeDIP peaks (Fig. 2b, bottom) [49]. Additionally, as a negative control, no 5mC or 5hmC were detected within the tested unmethylated loci (Figure S5 in Additional file 4). Results on other loci are similar (data not shown).

A recently published method, MLML, provides consistent methylation estimates by calculating the constrained maximum likelihood estimates using the expectation maximization algorithm [47]. To study the differences between Lux and MLML, we analyzed our BS-seq and oxBS-seq data using MLML and then compared the results with the ones obtained using Lux. First, the obtained C and 5mC level estimates correlate well between biological replicates for both of the two methods (Figure S6a in Additional file 4), although 5mC estimates from Lux correlate slightly better between biological replicates. Next we analyze all cytosines in a CpG context using Lux and observe that 5hmC correlations between replicates range from 0.45 to 0.52, which are a bit low but still higher than those from the MLML method (from 0.35 to 0.41) (Fig. 2d). MLML’s poor performance is likely affected by several biological and methodological factors, particularly the lack of experimental parameters in the MLML model. While Lux incorporates experiment-specific experimental parameters in estimating methylation modifications, these are not included in the MLML model, which together with variation in the oxidation efficiencies (Fig. 1e) can explain MLML’s lower 5hmC correlation values.

We further validated Lux’s performance on BS-seq and oxBS-seq data from Booth et al. [35]. We analyzed the BS-seq and oxBS-seq libraries (two biological replicates) while assuming 1) ideal experimental parameters (BS_eff_ = 1, ox_eff_ = 1, BS^*^_eff_ = 0, seq_err_ = 0) or 2) non-ideal experimental parameters (BS_eff_ = 0.99, ox_eff_ = 0.85, BS^*^_eff_ = 0.001, seq_err_ = 0.001) (see "Materials and methods"). Then we compared the resultant 5mC and 5hmC level estimates to glucMS-qPCR measurements (measured using a bisulfite-free and restriction enzyme-based assay) from [35]. We carried out the same comparison for the BS-seq and oxBS-seq analysis method from [35]. As expected, the Lux estimates obtained with the ideal experimental parameters have a slightly better correlation with the glucMS-qPCR measurements than the Booth et al. estimates (Table S4 in Additional file 5), particularly for 5hmC levels (0.54 versus 0.57). When the non-ideal experimental parameters are incorporated into the Lux analysis, we observe more accurate quantification of methylation levels (Table S4 in Additional file 5). Although the correlation of 5mC levels remains practically unchanged, the correlation of 5hmC levels increases from 0.57 (*p* = 0.007) to 0.63 (*p* = 0.002). Moreover, comparison of the correlation measures between Lux and the method from Booth et al. [35] shows a marked improvement for 5hmC quantification; correlation increases from 0.54 (*p* = 0.012) to 0.63 (*p* = 0.002).

Additionally, we carried out an in silico experiment simulating a replicate-free experiment to study the effect of sequencing coverage on the Lux and MLML estimates [47], thus providing guidelines for experiments (Figure S6b in Additional file 4). In short, we compared the Lux and MLML estimates of methylation levels using simulated data from different methylation level/coverage settings with controls. The results further demonstrate the importance of both integrative analysis of all cytosine modifications simultaneously and accounting for the experimental parameters in estimating C, 5mC and 5hmC levels because the MLML estimates are consistently biased, i.e., the medians are deviated from the true values (Figure S6c in Additional file 4). Notably, this holds for both hypo- and hyper-5mC (methylation levels of p(C) = 0.8, p(5mC) = 0.1, and p(5hmC) = 0.1 and p(C) = 0.1, p(5mC) = 0.8, and p(5hmC) = 0.1) and situations with high but realistic 5hmC levels (p(5hmC) = 0.3), commonly observed in various applications. Note in particular that ignoring the experimental parameters results in consistent underestimation of the already less abundant 5hmC species levels. Lux also has a small bias for low sequencing depths due to the prior distribution used in Bayesian analysis. Importantly, the more experimental data one has, the smaller the bias in Lux estimates becomes, i.e., Lux provides consistent methylation level estimates. The user can also adjust the strength of the prior; a less informative prior produces less bias, whereas a stronger prior produces less variance for low sequencing depth. In this simulation experiment, approximately 48× coverage is enough for Lux to produce accurate methylation proportion estimates. Supposedly, a higher sequencing depth is needed when the 5hmC level is lower and/or the experimental parameters are impaired. We also simulated biological replicates to gain information on the effect of replicates on methylation level estimates (Figure S7a, b in Additional file 4). As expected, the accuracy of estimates is commensurate with the number of replicates and more replicates are needed as the divergence between the distribution of interest and prior increases (Figure S7c–e in Additional file 4). Overall, these results demonstrate that Lux is able to infer biological variation from BS- and oxBS-seq data, which is essential in detecting differential methylation.

### Detection of differential methylation

Next we describe how Lux identifies differential methylation between conditions A and B. Briefly, two hypotheses, or models, are formulated (see "Materials and methods"): the null hypothesis H_0_ where Δμ = μ_A_ − μ_B_ = 0 (no differential methylation); and the alternative hypothesis H_1_ where Δμ = μ_A_ − μ_B_ ≠ 0 (differential methylation). In a Bayesian setting the data support for the hypothesis H_1_ over H_0_ can be quantified using the Bayes factor (BF; see "Materials and methods"). Here the BFs are approximated using the Savage-Dickey density ratio approach, which has recently been used, e.g., in detecting alternative splicing by Katz et al. [50]. The Savage-Dickey formulation involves calculation of the ratio BF ≃ p(Δμ = 0|H_1_)/p(Δμ = 0|H_1_,D). Succinctly, the term in the numerator is calculated from the prior distributions of μ for which we derive a closed-form solution, and the denominator is calculated from the posterior for which we use samples from the HMC sampler (see "Materials and methods").

**Fig. 3:**
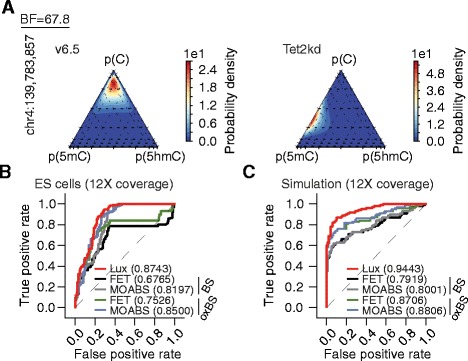
Identifying differentially methylated cytosines. **a** A ternary plot representation of the posterior distribution of the methylation proportions (estimated over three replicates) for the cytosine at chr4:139,783,857 in v6.5 (*left panel*) and Tet2kd (*right panel*) samples. **b** A comparison of Lux, FET, and MOABS in detecting differential methylation. For this purpose we down-sampled the full data set to 12× coverage for each of the three replicates. BS-seq and oxBS-seq data sets were analyzed separately with FET and MOABS for differential methylation. All the covered cytosines in a CpG context (N = 384) were divided into sets of differentially (N = 252) and similarly (N = 132) methylated cytosines based on independent CMS-IP and MeDIP loci-level information (see "Materials and methods"). The ROC curves of the methods are calculated based on the differential methylation analysis results. The curves of different methods (Lux, FET, and MOABS) and data types (BS-seq/oxBS-seq) are distinguished with different colors. The AUC values are listed in the figure key. **c** Same as (**b**) but here the methods are compared using simulated data (see also Figure S8e in Additional file 4)

We compared Lux, MOABS, and FET for detecting differential methylation on real data. We divided all the covered cytosines in a CpG context (N = 384) into sets of differentially (N = 252) and similarly (N = 132) methylated cytosines based on independent CMS-IP and MeDIP loci-level information (see "Materials and methods"). Obviously, CMS-IP and MeDIP do not give information on methylation at a single-nucleotide resolution level; however, in many cases the methylation of nearby CpG sites is highly correlated. Since we consider short loci, presumably the obtained ground-truth sets are largely correct and, importantly, obtained using a method independent of BS-seq and oxBS-seq protocols. The use of FET and MOABS require that replicates are pooled and BS-seq and oxBS-seq data are analyzed separately (see "Materials and methods"). To investigate the effect of coverage, we analyzed the data using either the full data set or reduced data sets down-sampled to either 12× or 30× coverage. The cytosines were listed in descending order of evidence for differential methylation (descending and ascending order of *p* values and BFs, respectively), and the methods were compared using the area under the curve (AUC) of receiver operating characteristic (ROC) curves (Fig. 3c; Figure S8c in Additional file 4). Lux provided better performance on the 12× down-sampled data with realistic coverage (AUC = 0.8743) than MOABS (AUC_BS_ = 0.8197, AUC_oxBS_ = 0.8500) or FET (AUC_BS_ = 0.6765, AUC_oxBS_ = 0.7526). The results were highly similar on the 30× down-sampled data (Lux AUC = 0.8748; MOABS AUC_BS_ = 0.8197, AUC_oxBS_ = 0.8500; and FET AUC_BS_ = 0.6765, AUC_oxBS_ = 0.7526; Figure S8c in Additional file 4). As expected, the results of the methods on the full coverage data set were close, but Lux still provided the best performance; the AUC values were 0.8728 for Lux, AUC_BS_ = 0.7446 and AUC_oxBS_ = 0.7697 for FET, and AUC_BS_ = 0.8678 and AUC_oxBS_ = 0.8576 for MOABS. Additionally, we tested the binomial test with conversion efficiency method [32] on our data (see "Materials and methods"). The performance of the method was poorer than those of Lux, MOABS, and FET, which is expected since the method is primarily designed for the detection of methylation (Figure S8d in Additional file 4). These results collectively show that the integrative analysis of BS-seq/oxBS-seq data and the model-based analysis of replicates by Lux result in improved performance on data with realistic sequencing coverage.

From the ten differentially methylated and four non-differentially methylated loci covered by our targeted experiments, we identified that 30 individual cytosines, regardless of sequence context, were differentially methylated between v6.5 and Tet2kd cells (BF > 1, i.e., the posterior probability of H_1_ exceeds that of H_0_, corresponding to ‘weak evidence’). Eight of the cytosines had at least ‘substantial evidence’ (BF > 3) for differential methylation (Figure S9a in Additional file 4; Table S3 in Additional file 5). For comparison, FET and MOABS are very non-conservative, as FET detected 464 (BS-seq) and 788 (oxBS-seq) and MOABS 226 (BS-seq) and 316 (oxBS-seq) differentially methylated cytosines (Benjamini-Hochberg false discovery rate (FDR) < 0.01). Although Lux is more conservative in reporting significant differential methylation, nevertheless, ROC analysis confirms that Lux is more accurate in discriminating differential methylation from non-differential methylation (Fig. 3c; Figure S8c in Additional file 4). The changes detected by Lux were supported by antibody-based approaches (Figure S10 in Additional file 4). Notably, the amount of evidence for differential methylation decreases significantly when ideal experimental parameters (i.e., BS_eff_ = 1, BS*_eff_ = 0, ox_eff_ = 1, and seq_err_ = 0) are used in the model (Figure S9b in Additional file 4), thus further emphasizing the importance of accounting for the experimental parameters. The condition-specific posterior distributions of the methylation levels for the top hits from the loci chr4:139,783,236–139,784,235 and chr15:61,868,386–61,869,385 show the expected pattern of TET2-dependent demethylation, i.e., increased 5mC and decreased 5hmC levels in Tet2kd samples (Fig. 3b; Figure S10a, c in Additional file 4). Intriguingly, these loci reside in the vicinity of a promoter of a long non-coding gene, *Pvt1* (plasmacytoma variant translocation 1; Figure S10c in Additional file 4) and an intronic enhancer within *Igsf21* (immunoglobin superfamily, member 21; Figure S10a in Additional file 4) identified in mESCs [51]. Unexpectedly, the cytosine having the third highest BF, chr15:100,300,108, shows unaffected 5mC (p_v6.5_(5mC) = 0.23/p_Tet2kd_(5mC) = 0.20) but increased 5hmC upon Tet2 knock down (p_v6.5_(5hmC) = 0.02/p_Tet2kd_(5hmC) = 0.34) (Figure S10b in Additional file 4). Possibly, the downstream demethylation pathway (5hmC → C) is dependent on TET2. In conclusion, detection of modest changes caused by an individual enzyme (TET2) requires primarily biological replicates but not exceedingly deep sequencing per sample (Figure S8f in Additional file 4) and consideration of experimental parameters (Figure S9b in Additional file 4), whereas near complete methylation (p(5mC) = 0.95) and unmethylation (p(C) = 0.95) can be distinguished from each other without biological replicates even with a low sequencing coverage (Figure S8g in Additional file 4).

Additionally, to guide experimental design in future studies, we applied Lux, Fisher’s exact test and MOABS to in silico data with realistic genome-wide coverage (12×) and varying number of replicates. First, as desired, Lux does not detect differential methylation between identical conditions and the detection sensitivity of differential methylation increases together with the number of replicates and the magnitude of differential methylation (Figure S8a, b in Additional file 4). Second, consistent with our results on real data, we observed that Lux (AUC = 0.9443) outperformed FET (AUC_BS_ = 0.7919, AUC_oxBS_ = 0.8706) and MOABS (AUC_BS_ = 0.8001, AUC_oxBS_ = 0.8806) in discriminating differential methylation from nondifferential methylation (Fig. 3d). For the amount of biological variation and differential methylation used in our simulations, strong evidence (BF > 10) for differential methylation is typically obtained with two or more replicates. Taken together with results from real data (Fig. 3; Figures S8f, g, S9 and S10 in Additional file 4) we expect that three biological replicates with only modest sequencing coverage are sufficient to detect larger differential methylation changes in controlled molecular biology studies. As methylation modification level changes in disease studies can be modest, our results support the use of larger sample sizes even at the price of sequencing coverage.

**Fig. 4:**
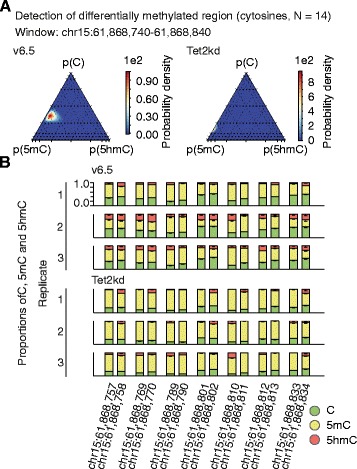
Detecting differentially methylated regions. **a** The loci-level methylation is estimated using the 14 cytosines in a CpG context in wild-type (*left panel*) and knockdown samples (*right panel*). The estimated posterior distributions obtained from the three biological replicates are shown. **b** The posterior means of the methylation modifications of individual cytosines in a CpG context across the studied window and the three biological replicates are depicted. The one-sided error bars (mean − standard deviation) show the standard deviations

To gain more statistical power for managing biological variation one can move from the individual cytosine level to the loci level [7]. In Lux, this is implemented by assuming that the methylation levels of cytosines within a locus follow the same μ distribution while allowing variation between individual cytosines within a locus (Figure S11a in Additional file 4; see "Materials and methods"). We scanned the 14 loci with window-length 100 bp and 50 bp step size (Table S5 in Additional file 5; see "Materials and methods"). Altogether, we identified 16 windows from six different loci having BF > 1; as expected, 14 out of these 16 windows exhibited increased 5mC and decreased 5hmC levels in Tet2kd compared with v6.5 cells. As an example, this approach led to posterior distributions on the locus chr15:61,868,740–61,868,840 having great kurtosis (BF > 1e16; Fig. 4a) even though the individual cytosines, of which only two have BF > 1, are variably methylated across the locus and between biological replicates (Fig. 4b). In other words, the proposed loci level analysis scheme achieves greater sensitivity for detecting modest changes in methylation, which is an anticipated feature of studies without biological replicates or with large biological variability. Additionally, comparison of these loci-level differential methylation analysis results with the independent CMS-IP and MeDIP validation data shows that Lux is more accurate in detecting differential methylation than MOABS (Figure S11b in Additional file 4).

### DNA demethylation dynamics during mouse T-cell development

To further demonstrate the applicability of Lux for analyzing dynamic DNA methylation/demethylation changes during T-cell development, we measured DNA modifications for five loci in double positive (DP), CD4 single positive (SP) and naïve CD4 T cells using targeted BS and oxBS sequencing. The five loci distributed in *Il6ra*, *Prkcq*, *Zbtb7b* (two loci), and *Pax5* were selected because they are important for mouse T-cell development and 5hmC levels were dynamically changed during mouse T-cell development based on an antibody-based assay [52]. The resulting methylome snapshots of three biological replicates enabled us to study DNA methylation through three consecutive developmental stages during mouse T-cell development at single-base resolution.

The sequencing libraries were spiked with stretches of unmethylated, methylated and hydroxymethylated DNAs as described previously (see "Materials and methods"). Strikingly, when estimating the experimental parameters as described above, each cytosine in the hydroxymethylated control DNA was estimated to be lowly hydroxymethylated (p(5hmC) ≃ 0.1) contrary to our prior belief of ~90 % purity of the 5hmCTP mix (Figure S12a in Additional file 4; Table S6 in Additional file 5). To confirm the impurity of the 5hmCTP mix, we performed a dotblot assay to quantify the 5hmC level in the new spike-in 5hmC-containing oligonucleotides used in this study (Figure S12b in Additional file 4). Indeed, the dotblot assay results suggest a ~10-fold decrease of 5hmC levels in the new 5hmC spike-in control. Lux took the impurity of the 5hmCTP mix into account automatically through integrative analysis of all modifications and all spike-in controls and updated the prior distributions in light of the experimental data (Figure S12a in Additional file 4); hence, the resulting experimental parameter estimates were in the expected range (Table S7 in Additional file 5). For instance, the posterior mean of ox_eff_ varied from 0.86 to 0.94 (Figure S12c in Additional file 4). Importantly, none of the existing tools would be able to correct these kinds of biases in the control data and/or experimental parameters.

Next, we estimated the methylation status of all of the cytosines with at least 10× coverage across all nine samples, that is, we analyzed 423 cytosines (64 are in a CpG context; Table S7 in Additional file 5). We first repeated the same correlation analysis between biological replicates as for the embryonic stem cell data above. The correlations for C and 5mC levels are again very high for both Lux and MLML (data not shown). Interestingly, in our T-cell data the 5hmC correlations (Figure S12d in Additional file 4) are also notably higher compared with the v6.5 data (Fig. 2d). Importantly, Lux achieves consistently higher correlation values than MLML, although the amount of increase is smaller than in the embryonic stem cell data. For T-cell data, where oxidation efficiencies are consistently good (Figure S12c in Additional file 4), MLML is able to estimate consistent 5hmC levels between replicates, whereas for the embryonic stem cell data, where oxidation efficiencies exhibit more variation (Fig. 1e), 5hmC estimates from MLML are less consistent. The Lux method, in turn, provides more consistent 5hmC estimates both for the embryonic stem cell and T-cell data. Overall, our results reveal that Lux is notably more consistent across biological replicates than previous methods, thus suggesting that utilization of experimental parameters improves the quantification of cytosine modification levels.

**Fig. 5:**
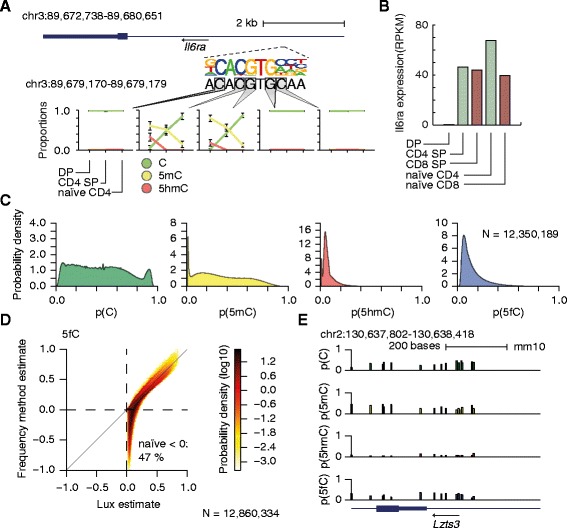
Demethylation in *Il6ra* during mouse T-cell development and analysis of genome-wide BS-seq, TAB-seq, and fCAB-seq data. **a** A locus close to the 3’ UTR of *Il6ra* (*top*) with the canonical E-box motif (*middle*) is depicted. The posterior means of methylation proportions of the cytosines within the locus are visualized in DP, CD4 SP, and naïve CD4 cells (*bottom*). The error bars (mean ± standard deviation is depicted) show the standard deviations of the posterior distributions. The different methylation modifications are distinguished with different colors (p(C) in *green*, p(5mC) in *yellow*, and p(5hmC) in *red*). **b** The bar plot shows the gene expression (RPKM) of *Il6ra* in DP, CD4 SP, CD8 SP, naïve CD4, and naïve CD8 cells. **c** The marginal distributions of the posterior means of θ among the common cytosines. The panels, from left to right, correspond to the marginal distributions of the posteriors means of p(C), p(5mC), p(5hmC) and p(5fC). **d** Comparison of the Lux and frequency method estimates (5fC_frequency_ = N_BS,T_/N_BS_ − N_fCAB,T_/N_fCAB_) of the 5fC levels. Almost half (47 %) of the frequency method estimates are negative. **e** The estimated posterior means of C, 5mC, 5hmC, and 5fC levels (from *top* to *bottom*) across the locus chr2:130,637,802 − 130,638,418 are depicted in the bar charts

We next detected differentially methylated cytosines in a CpG context between any two cell types and identified 18, 29, and 17 differentially methylated cytosines (BF > 1) from the comparisons of DP versus CD4 SP, DP versus naïve CD4, and CD4 SP versus naïve CD4, respectively. Altogether, 30 unique differentially methylated cytosines were identified (Figure S12e in Additional file 4; Table S6 in Additional file 5). The rest of the cytosines were mostly methylated in the three stages of development (average 5mC levels are 0.85, 0.84, and 0.77 in DP, CD4 SP, and naïve CD4 cells, respectively; Table S6 in Additional file 5). The average 5mC level of differentially methylated cytosines decreases during the transitions from DP (0.71) to CD4 SP (0.59) and further to naïve CD4 (0.32) (Figure S12e in Additional file 4; Table S6 in Additional file 5). Simultaneously, the average 5hmC level peaks in DP cells (0.23, 0.08, and 0.02 in DP, CD4 SP, and naïve CD4 cells, respectively), supporting the role of oxi-mC species in the demethylation pathway (Figure S12e in Additional file 4; Table S6 in Additional file 5). Collectively, we detected gradual loss of 5mC during the transition from DP stage to naïve CD4 stage within the three loci, which are important in mouse T-cell development. Interestingly, one of the CpG dinucleotides that lost 5mC resides within a detected canonical E-box motif occurrence in *Il6ra* (Fig. 5a). *Il6ra* is not expressed in DP cells but it is highly expressed in CD4/CD8 SP and naïve CD4/CD8 cells (Fig. 5b) [52–54]. As many transcription factors binding the canonical E-box motif are expressed during T-cell development [55], and as DNA methylation is known to alter DNA conformation and conformational changes in turn alter binding to E-box motifs [56], it is plausible that this locus is occupied by one or more readers of 5mC in DP cells and/or readers of oxi-mC or unmodified cytosine in CD4 SP and naïve CD4 cells.

### Integrative analysis of genome-wide BS-seq, TAB-seq, and fCAB-seq data

To further demonstrate the applicability of Lux in a genome-wide setting, we analyzed recently published BS-seq, TAB-seq, fCAB-seq data sets from two-cell embryos [57]. Notably, the introduction of fCAB-seq allows the identification of 5fC, and consequently the cytosine-specific probability vector θ = [p(C), p(5mC), p(5hmC), p(5fC)] (Σθ = 1) is four-dimensional. First, we derive the required statistical model by stating the propagated probabilities of the possible outcomes of the BS-seq, TAB-seq and fCAB-seq assays (Additional file 3; Figure S13 in Additional file 4). Besides the aforementioned bisulfite conversion (BS_eff_) and inaccurate bisulfite conversion (BS*_eff_) efficiencies and sequencing error (seq_err_), here we considered labeling (lab_eff_), oxidation (ox_eff_), and protection (pro_eff_) efficiencies involved in TAB-seq and fCAB-seq assays (Figure S13 in Additional file 4). First, we confirmed using an in silico simulation approach that we can simultaneously identify experimental parameters and methylation levels accurately from the data (Figure S14a in Additional file 4). Indeed, our simulations with different realistic methylation level/coverage settings demonstrate Lux’s ability to produce consistent (i.e., unbiased) methylation level estimates with notably smaller variance than the frequency method estimator. As expected, the frequency estimator produces often negative methylation level estimates in the cases of low 5mC (hypo-5mC) and/or 5fC (hypo-5fC). This is an important point because cytosines with negative estimates are typically ignored from downstream analysis.

Next, we estimated the four methylation modification levels of the common (N = 12,350,189), maternal (N = 477,179) and paternal (N = 32,966) cytosines in a CpG context with at least 10× coverage (Fig. 5c; Figure S14b in Additional file 4; see "Materials and methods"). The experimental values were set based on the values reported in Wang et al. [57] (see "Materials and methods"). As reported previously, the 5hmC and 5fC levels are modest in general (Fig. 5c; Figure S14b in Additional file 4); for instance, 79 % and 46 % of the common cytosines are lowly hydroxided (p(5hmC) ≤ 0.1) and/or formylated (p(5fC) ≤ 0.1), respectively (Fig. 5c). However, for some cytosines 5hmC and 5fC modification levels can reach up to 0.3 and 0.6, respectively. Intriguingly, the distributions of the C and 5mC levels differ between the common and maternal cytosines (Fig. 5c; Figure S14b, top in Additional file 4) as 10 % and 22 % of the common and maternal cytosines are methylated (p(C) ≤ 0.1), respectively. Lux automatically quantifies the amount of uncertainty in estimated cytosine modification levels for each cytosine via the full posterior distribution. As expected, the standard deviations of the estimated posterior distributions of methylation levels decrease when the sequencing coverage increases (Figure S14c in Additional file 4). Notably, almost half of the considered cytosines had negative 5mC or 5fC levels and would thus be ignored (or truncated to zero) when the frequency method estimator is used (Fig. 5d; see Figure S14d in Additional file 4 for the 5mC and 5hmC comparisons). As expected, the similarity between the Lux and frequency method estimates improves when cytosines with negative frequency method estimates are ignored, but simultaneously almost half of the data is also ignored (Figure S14e in Additional file 4), whereas Lux provides estimates of methylation modification levels which are both consistent (sum up to one) and take into account the experiment-specific variation in biochemistry, i.e., non-ideal experimental parameters. Finally, we visualized the estimated C, 5mC, 5hmC, and 5fC levels across the locus discussed in Wang et al. [57] (Fig. 5e). Note that Lux can estimate all the four different modification levels (instead of 5mC, 5fC and 5mC + 5hmC + 5fC) and that the methylation levels of each cytosine sum up to one.

### Applicability of Lux to analyze other derivatives of traditional bisulfite sequencing data

**Fig. 6:**
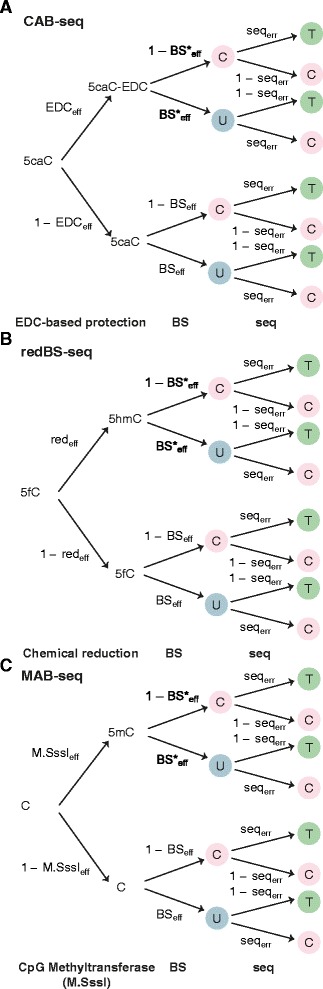
The effect of experimental parameters on CAB-seq, redBS-seq and MAB-seq read-outs. **a** The experimental steps of CAB-seq depending on 1-ethyl-3-[3-dimethylaminopropyl]-carbodiimide hydrochloride (EDC) on 5caC are stated in terms of BS_eff_, BS*_eff_, EDC_eff_, and seq_err_. **b** The experimental steps of redBS-seq on 5fC are stated in terms of BS_eff_, BS*_eff_, red_eff_, and seq_err_. **c** The experimental steps of MAB-seq on 5fC and 5caC are stated in terms of BS_eff_, BS*_eff_, M.Sssl_eff_, and seq_err_

Above we described how Lux can analyze BS-seq, oxBS-seq, TAB-seq and fCAB-seq data together with their experimental parameters. Importantly, Lux is also applicable for the analysis of CAB-seq, redBS-seq and MAB-seq data with minor changes. Another bisulfite-based technique, termed CAB-seq, was recently published for detecting 5caC at nucleotide resolution [36], making it possible to differentiate C, 5mC, 5hmC and 5caC. This requires an integration of CAB-seq data with BS-seq and oxBS-seq/TAB-seq data, which is easily implemented in Lux by defining the generative model for the outcomes of a CAB-seq experiment in terms of its related experimental parameters (Fig. 6a; Additional file 3) and combining that with the likelihood functions of BS-seq and oxBS-seq/TAB-seq data. An additional bisulfite-based technique, redBS-seq, has been developed for detecting 5fC at individual cytosine sites. Interestingly, Booth et al. [35] reported that almost 30 % of the 5fC estimates obtained using the frequency method estimator were negative, which were then discarded from the subsequent analysis. This problem can only be resolved by using the integrative analysis of all cytosines as implemented in Lux. Similar to CAB-seq, Lux can be straightforwardly extended to redBS-seq and MAB-seq data (Fig. 6b, c; Additional file 3). More generally, the hierarchical framework implemented in Lux can be extended to process data from various sequencing assays with sequential, error-prone experimental steps [33].

## Conclusions

Here, we present a unified statistical framework, Lux, for analyzing BS-seq and oxi-mC-seq data sets. Lux provides several major improvements and extensions compared with existing methods; for instance, it integrates BS-seq and oxi-mC-seq measurements, models bisulfite conversion and oxidation efficiencies, various chemical labeling and protection steps and sequencing errors, and analyzes data from replicated experiments. No previous computational analysis methods exist which would have the above-mentioned functionalities. Lux’s performance on detecting experimental parameters, methylation levels, biological variation and differential methylation was assessed extensively on real and simulated data on various realistic methylation levels ((C, 5mC, 5hmC): (0.8, 0.1, 0.1), (0.1, 0.8, 0.1), (0.6, 0.1, 0.3), (0.1, 0.6, 0.3), (0.7, 0.25, 0.05), (0.2, 0.7, 0.1); (C, 5mC, 5hmC, 5fC): (0.8, 0.1, 0.05, 0.05), (0.1, 0.8, 0.05, 0.05), (0.6, 0.1, 0.15, 0.15), and (0.1, 0.6, 0.15, 0.15) corresponding to different scenarios of strong hyper- and hypomethylation, weak hyper- and hypomethylation, as well as active demethylation (see also Figure S14b in Additional file 4)). Through Bayesian inference, experimental parameters and their associated uncertainties propagate to the final estimates of methylation levels, which makes it possible to compare samples with different experimental parameters in a meaningful and statistically justified manner. We have shown how the accuracy and sensitivity of methylation estimates and the detection of differential methylation are improved compared with previous methods when the non-ideal, sample-specific experimental parameters and replicates are taken into account. Our results support the previous guidelines for sequencing depth requirements for discriminating completely methylated cytosines from completely unmethylated cytosines. Importantly, we further examined the detectability of endogenous levels of 5hmC and demonstrated the importance of biological replicates as well as experimental parameters in detecting subtle changes in 5hmC or other cytosine modifications.

Our detailed analysis of selected genomic loci revealed TET2-dependent demethylation of individual cytosines occurring at promoters and enhancers in mESCs. Moreover, we identified progressive loss of 5mC, leading to production of 5fC, 5caC or unmodified cytosine (these three cytosine species are experimentally indistinguishable in our experimental conditions) in genes known to be important for mouse T-cell development. The observed effect of TET2 was only partial, suggesting that demethylation is controlled in parallel by multiple enzymes. As reported previously, 5mC and 5hmC were only observed at cytosines in a CpG context. Our analysis of biological replicates illustrated the stochastic nature of demethylation. The observed stochasticity emphasizes the importance of biological replicates, especially when the focus is on studying differential methylation of individual cytosines. For instance, the inclusion of the exon 5 of *Ptprc* during lymphocyte activation is governed by methylation status [10]. Low levels of DNA methylation on exon 5 allow CTCF binding and cause RNA polymerase II pausing, thus resulting in exclusion of exon 5 [10]. Previous studies suggested that the interaction between CTCF and DNA is affected by CpG methylation, even at a single CpG site. Although antibody-based techniques can be useful in detecting larger methylated loci, their resolution is limited to the range of hundreds of nucleotides. Furthermore, quantification and comparison of absolute proportions of different cytosine modifications from immunoprecipitation data are challenging, whereas Lux automatically estimates absolute proportions of Cs, 5mCs, and 5hmCs from BS-seq and oxBS-seq data at single nucleotide resolution.

Deciphering the active demethylation pathway will require deconvolution of the effects of individual enzymes to understand their orchestrated action. Moreover, it will be intriguing to shed light on the interplay between transcription factor binding and methylation using DNase footprinting or ChIP-exo and BS-seq/oxi-mC-seq approaches, respectively. In addition, the interaction between DNA and other transcriptional factors can be affected by DNA methylation at a single CpG site, such as C/EBP [58]. It will be very interesting to examine how oxidized 5mCs (5hmC, 5fC, and 5caC) affect the DNA binding capability of transcriptional factors to regulate gene expression. Although 5hmC, 5fC, and 5caC binding proteins have been identified in mESCs and neural progenitor cells, many transcriptional factors have their own binding motif which might not be captured with the approach of Spruijt et al. [59]. In combination with the enrichment of certain transcription factors, single-base resolution mapping of oxi-mC and Lux analysis will provide insights into the effect of DNA modifications on DNA binding of transcriptional factors either genome-wide or at the loci-specific scale. In addition, understanding the role and importance of 5hmC and other further oxidized cytosine modifications in transcription will require temporal approaches for measuring active transcription, such as nascent-seq, and the capability of detecting temporal changes in methylation levels at high resolution. In conclusion, all of the aforementioned and many additional future research questions will benefit greatly from Lux’s unique features of accounting for sample-specific variation in experimental parameters when quantifying all cytosine modification levels from replicated BS-seq and oxi-mC-seq data sets. All of Lux’s functionality described above is implemented in the Lux software, which has been made freely available.

## Materials and methods

### Embryonic stem cell culture and genomic DNA isolation

mESCs (v6.5) were cultured in Knockout DMEM (Invitrogen) with 20 % embryonic stem cell qualified fetal bovine serum (Germini Bio-product), 2 mM L-glutamine, 0.1 mM 2-mercaptoethanol, 0.1 mM nonessential amino acids, 50 units/ml penicillin/streptomycin and 1000 U/ml ESGRO (LIF; Chemicon). Tet2 was stably knocked down in v6.5 cells using electroporation with pSUPER-puro-Tet2shRNA (320V, 250F) followed by 1.5 μg/ml puromycin selection for 7–10 days [60]. Genomic DNA was isolated with the DNeasy blood and tissue kit (Qiagen) by following the manufacturer’s instructions. Three independent cultures of wild-type and Tet2kd samples were used.

### Validation of Tet2 knockdown in mESCs

Tet2 knockdown efficiency was measured by quantitative PCR (qPCR) and western blot [49]. For qPCR, total RNA was isolated with an RNeasy kit (Qiagen, Chatsworth, CA, USA) and cDNA was made using SuperScript III reverse transcriptase (Invitrogen). qPCR was performed using FastStart Universal SYBR Green Master mix (Roche, Mannheim, Germany) on a StepOnePlus real-time PCR system (Applied Biosystems, Foster City, CA, USA). Gene expression was normalized to *Gapdh*. Primers used for qPCR are listed below:

- *Tet1* forward: GAGCCTGTTCCTCGATGTGG
- *Tet1* reverse: CAACCCACCTGAGGCTGTT
- *Tet2* forward: AACCTGGCTACTGTCATTGCTCCA
- *Tet2* reverse: ATGTTCTGCTGGTCTCTGTGGGAA
- *Gapdh* forward: GTGTTCCTACCCCCAATGTGT
- *Gapdh* reverse: ATTGTCATACCAGGAAATGAGCTT

For western blot, nuclear proteins from parental and *Tet2* knock-down mESCs were extracted as previously described [61]. Nuclear protein (30 μg) was loaded on 4–12 % Bis-Tris gels (Invitrogen) and transferred to nitrocellulose membrane. Tet2 was detected using anti-Tet2 (Abcam) antibodies. Loading control, beta-actin, was detected using anti-beta actin from Abcam.

### Mice

We used 4–6-week-old female C57BL/6 mice obtained from Jackson labs for cell isolation. The mice were housed in a pathogen-free animal facility in the La Jolla Institute for Allergy and Immunology and were used according to protocols approved by the Institutional Animal Care and use Committee (IACUC).

### Preparation of thymocyte subsets

Subsets of thymocytes were isolated by cell sorting as previously described [54], after cell surface staining using CD4 (GK1.5), CD8 (53–6.7), CD3ε (145-2C11), and CD24 (M1/69) (all from Biolegend). DP cells were CD4+ CD8 int/hi; CD4 SP cells were CD4CD3 hi, CD24 int/lo. Peripheral subsets were isolated after pooling spleen and lymph nodes. T cells were enriched by negative isolation using Dynabeads (Dynabeads untouched mouse T cells, 11413D, Invitrogen). After surface staining for CD4 (GK1.5), CD8 (53–6.7), CD62L (MEL-14), CD25 (PC61) and CD44 (IM7), naïve CD4 + CD62LhiCD25-CD44lo were obtained by sorting (BD FACS Aria). Three cell isolations from independent mice were prepared for each of the three thymocyte subsets.

### Synthesis of cytosine-, 5mC- and 5hmC-containing control oligonucleotides

Spike-in cytosine-, 5mC- and 5hmC-containing control oligonucleotides were synthesized using unmethylated lambda DNA (Promega) as template by PCR containing dCTP, dmCTP (5mC) or dhmCTP (5hmC), respectively. Regular dCTP was purchased from Promega, and dmCTP and dhmCTP were purchased from Zymo Research. PCR primers for control oligonucleotides are listed below:

- C control oligo forward: ATTGTATGTATTGGTTTATTG
- C control oligo reverse: TTATCACATTCAAACATTAAT
- 5mC control oligo forward: TAGATAGTAAATATAATGTGAGA
- 5mC control oligo reverse: ATAAATCATCAACAAAACACAA
- 5hmC control oligo forward: GTTTTTTTGAATAATAAATGTTA
- 5hmC control oligo reverse: TTTATCACCTCTAAAATATATCA

PCR was performed using REDtaq DNA polymerase (Sigma) by following the manufacturer’s instructions.

### BS-seq and oxBS-seq

Purified genomic DNA with spike-in control oligonucleotides (1:50) was divided into two parts. One part was directly treated with sodium bisulfite, while the other was treated with KRuO4 to oxidize 5hmC to 5fC, followed by bisulfite treatment. oxBS experiments were performed by following the procedures described by Booth et al. [32, 62]. Briefly, up to 1 μg ethanol-precipitated genomic DNA was purified by Micro Bio-Spin column (SSC buffer; Bio-Rad) and denatured in 24 μl 0.05M NaOH at 37 °C for 30 min. Denatured genomic DNA was snap-cooled on ice for 5 min and followed by adding 1 μl KRuO4 solution (15 mM in 0.05M NaOH). The reaction was performed on ice for 1 h with gentle flicks every 10–15 min. Next, reacted genomic DNA was purified by Micro Bio-Spin column (SSC buffer; Bio-Rad). Bisulfite reaction was performed using the MethylCode bisulfite conversion kit (Invitrogen) by following the manufacturer’s Instructions. Loci-specific primers against bisulfite-treated genomic DNA were designed through the online MethPrimer software. Regions of interest were amplified using oxBS- and BS-treated genomic DNA as templates by using the PyroMark PCR kit (Qiagen) and further purified by AmpuXP beads (Beckman coulter) in 96-well PCR plates. To prepare libraries compatible with MiSeq, the concentration of each amplicon was quantified by Nanodrop and normalized to desired concentrations. In each condition, normalized amplicons were pooled together and followed by illumina library preparation using TruSeq DNA library preparation kit (Illumina). Prepared libraries were amplified for four cycles and purified by two rounds of AmpuXP beads to remove the primer dimmers. The quality of libraries was examined by Bioanalyzer (Agilent) and then subjected to sequencing on MiSeq.

### Preprocessing of BS-seq and oxBS-seq data

First the sequencing adapters were removed from the reads when encountered. Bismark v0.7.12 [63] was used to align the BS and oxBS reads against the mm9 reference genome and lambda phage DNA simultaneously. The alignment was done using the paired-end Bowtie 2 [64] backend with the following parameters: -I 0 -X 2000 -N 0. The “bismark_methylation_extractor” script distributed with the Bismark aligner was used to extract the number of unconverted and converted read-outs for each cytosine with the following parameters: --paired-end --CX --cutoff 10 --no_overlap --bedGraph --counts. The cytosines having at least ten read-outs across all six samples were taken into account. The control cytosines located on the Watson strand were used in the analysis.

### Derivation of the statistical model

We first describe the statistical model to quantify C, 5mC and 5hmC from BS-seq and oxBS-seq data and later extend Lux to other oxi-mC species and data types. For a given cytosine, we use a Dirichlet random variable of order three to model proportions of different cytosine methylations θ = [p(C), p(5mC), p(5hmC)] (Σθ = 1) simultaneously and, for a given value of θ, we define BS-seq and oxBS-seq likelihoods to have binomial distributions. Thus, our model could be described as Dirichlet-binomial^2^, where the binomial squared refers to the two binomial distributions used in modeling BS-seq and oxBS-seq data (Figure S2a in Additional file 4). Our model can also be viewed as an extension of a previously presented beta-binomial model, which is inadequate for simultaneous analysis of BS-seq and oxBS-seq data: MOABS [43] uses the beta distribution to model separately the probabilities p(C) + p(5mC or 5hmC) = 1 (BS-seq) and p(C or 5hmC) + p(5mC) = 1 (oxBS-seq). Our generalization makes it possible to analyze BS-seq and oxBS-seq data (and later any number and combination of BS-seq and/or oxi-mC-seq data sets) together and correctly deconvolve the proportions of different cytosine modifications. This procedure is explained in detail below.

To take into account the bisulfite conversion (BS_eff_), inaccurate bisulfite conversion (BS*_eff_) and oxidation (ox_eff_) efficiencies as well as sequencing errors (seq_err_), we have to define their effects on the BS-seq and oxBS-seq read-outs. Motivated by the chemical steps involved in BS-seq and oxBS-seq experiments, we define the effects of BS_eff_, BS*_eff_, ox_eff_ and seq_err_ on each of the cytosine modifications (C, 5mC, 5hmC; Fig. 1b, c; Figure S1b in Additional file 4) and derive the BS-seq- and oxBS-seq-specific emission probabilities (propagated probabilities; Figure S1c in Additional file 4). That is, we define the probability of observing “C” in a BS-seq experiment given that the nucleotide is unmethylated, p_BS_(“C”|C), as:

$$ {\mathrm{p}}_{\mathrm{BS}}\left(``\mathrm{C}"\Big|\mathrm{C}\right) = \left(1-\mathrm{B}{\mathrm{S}}_{\mathrm{eff}}\right)\left(1-\mathrm{s}\mathrm{e}{\mathrm{q}}_{\mathrm{err}}\right) + \mathrm{B}{\mathrm{S}}_{\mathrm{eff}}\mathrm{s}\mathrm{e}{\mathrm{q}}_{\mathrm{err}}, $$

and similarly for the other cases:

$$ \begin{array}{l}{\mathrm{p}}_{\mathrm{BS}}\left(``\mathrm{C}"\Big|5\mathrm{m}\mathrm{C}\right) = \left(1-\mathrm{B}\mathrm{S}{*}_{\mathrm{eff}}\right)\left(1-\mathrm{s}\mathrm{e}{\mathrm{q}}_{\mathrm{err}}\right) + \mathrm{B}\mathrm{S}{*}_{\mathrm{eff}}\mathrm{s}\mathrm{e}{\mathrm{q}}_{\mathrm{err}}\hfill \\ {}{\mathrm{p}}_{\mathrm{BS}}\left(``\mathrm{C}"\Big|5\mathrm{h}\mathrm{m}\mathrm{C}\right) = \left(1-\mathrm{B}\mathrm{S}{*}_{\mathrm{eff}}\right)\left(1-\mathrm{s}\mathrm{e}{\mathrm{q}}_{\mathrm{err}}\right) + \mathrm{B}\mathrm{S}{*}_{\mathrm{eff}}\mathrm{s}\mathrm{e}{\mathrm{q}}_{\mathrm{err}}\hfill \\ {}{\mathrm{p}}_{\mathrm{oxBS}}\left(``\mathrm{C}"\Big|\mathrm{C}\right) = \left(1-\mathrm{B}{\mathrm{S}}_{\mathrm{eff}}\right)\left(1-\mathrm{s}\mathrm{e}{\mathrm{q}}_{\mathrm{err}}\right) + \mathrm{B}{\mathrm{S}}_{\mathrm{eff}}\mathrm{s}\mathrm{e}{\mathrm{q}}_{\mathrm{err}}\hfill \\ {}{\mathrm{p}}_{\mathrm{oxBS}}\left(``\mathrm{C}"\Big|5\mathrm{m}\mathrm{C}\right) = \left(1-\mathrm{B}\mathrm{S}{*}_{\mathrm{eff}}\right)\left(1-\mathrm{s}\mathrm{e}{\mathrm{q}}_{\mathrm{err}}\right) + \mathrm{B}\mathrm{S}{*}_{\mathrm{eff}}\mathrm{s}\mathrm{e}{\mathrm{q}}_{\mathrm{err}}\hfill \\ {}{\mathrm{p}}_{\mathrm{oxBS}}\left(``\mathrm{C}"\Big|5\mathrm{h}\mathrm{m}\mathrm{C}\right) = \mathrm{o}{\mathrm{x}}_{\mathrm{eff}}\left[\left(1-\mathrm{B}{\mathrm{S}}_{\mathrm{eff}}\right)\left(1-\mathrm{s}\mathrm{e}{\mathrm{q}}_{\mathrm{err}}\right) + \mathrm{B}{\mathrm{S}}_{\mathrm{eff}}\mathrm{s}\mathrm{e}{\mathrm{q}}_{\mathrm{err}}\right] + \left(1-\mathrm{o}{\mathrm{x}}_{\mathrm{eff}}\right)\left[\left(1-\mathrm{B}\mathrm{S}{*}_{\mathrm{eff}}\right)\left(1-\mathrm{s}\mathrm{e}{\mathrm{q}}_{\mathrm{err}}\right) + \mathrm{B}\mathrm{S}{*}_{\mathrm{eff}}\mathrm{s}\mathrm{e}{\mathrm{q}}_{\mathrm{err}}\right].\hfill \end{array} $$

We follow the standard practice and ignore “A” and “G” read-outs as the reads containing these read-outs are discarded during the mapping (their impact on the estimates would be negligible), and, consequently, the probability of the complementary events, i.e., reading “T” instead of “C”, are one minus the aforementioned probabilities. Parameters BS_eff_, BS*_eff_, ox_eff_ and seq_err_ are shared across cytosines but, importantly, specific for each biological experiment.

In practice, BS-seq and oxBS-seq experiments are carried out for a collection of cells, which comprise a cytosine population. Consequently, the probability of sequencing a “C” (for a given cytosine) in BS-seq experiment is obtained by weighting the above emission probabilities with the (unknown) cytosine proportions, θ = [p(C), p(5hmC), p(5hmC)] (Figure S1d in Additional file 4):

$$ \begin{array}{l}{\mathrm{p}}_{\mathrm{BS}}\left(``\mathrm{C}"\right) = \mathrm{p}\left(\mathrm{C}\right){\mathrm{p}}_{\mathrm{BS}}\left(``\mathrm{C}"\Big|\mathrm{C}\right) + \mathrm{p}\left(5\mathrm{m}\mathrm{C}\right){\mathrm{p}}_{\mathrm{BS}}\left(``\mathrm{C}"\Big|5\mathrm{m}\mathrm{C}\right) + \mathrm{p}\left(5\mathrm{h}\mathrm{m}\mathrm{C}\right){\mathrm{p}}_{\mathrm{BS}}\left(``\mathrm{C}"\Big|5\mathrm{h}\mathrm{m}\mathrm{C}\right)\hfill \\ {}{\mathrm{p}}_{\mathrm{oxBS}}\left(``\mathrm{C}"\right) = \mathrm{p}\left(\mathrm{C}\right){\mathrm{p}}_{\mathrm{oxBS}}\left(``\mathrm{C}"\Big|\mathrm{C}\right) + \mathrm{p}\left(5\mathrm{m}\mathrm{C}\right){\mathrm{p}}_{\mathrm{oxBS}}\left(``\mathrm{C}"\Big|5\mathrm{m}\mathrm{C}\right) + \mathrm{p}\left(5\mathrm{h}\mathrm{m}\mathrm{C}\right){\mathrm{p}}_{\mathrm{oxBS}}\left(``\mathrm{C}"\Big|5\mathrm{h}\mathrm{m}\mathrm{C}\right).\hfill \end{array} $$

In other words, p_BS_(“C”) and p_oxBS_(“C”) are the probabilities of obtaining “C” in a single BS-seq and oxBS-seq draw, respectively, from a cytosine population with proportions p(C), p(5hmC) and p(5hmC). Thus, individual “C” and “T” read-outs from BS-seq and oxBS-seq are Bernoulli distributed where the probabilities of observing “C” are p_BS_(“C”) and p_oxBS_(“C”), respectively. Consequently, the counts of “C” read-outs, N_BS,C_ and N_oxBS,C_, from N_BS_ BS-seq and N_oxBS_ oxBS-seq draws, respectively, are binomially distributed (Figure S2a in Additional file 4).

Because BS-seq and oxBS-seq data are conditionally independent given model parameters, the likelihood of data D = (D_BS_,D_oxBS_) for a single cytosine is the product of the BS-seq and oxBS-seq likelihoods, p(D_BS_|θ,BS_eff_,BS*_eff_,seq_err_) and p(D_oxBS_|θ,BS_eff_,BS*_eff_,ox_eff_,seq_err_). Thus, under the binomial model the likelihood function has the following form:

$$ \begin{array}{l}\mathrm{p}\left({\mathrm{D}}_{\mathrm{BS}},{\mathrm{D}}_{\mathrm{oxBS}}\Big|\uptheta, \mathrm{B}{\mathrm{S}}_{\mathrm{eff}},\mathrm{B}\mathrm{S}{*}_{\mathrm{eff}},\mathrm{o}{\mathrm{x}}_{\mathrm{eff}},\mathrm{s}\mathrm{e}{\mathrm{q}}_{\mathrm{err}}\right)\hfill \\ {}=\mathrm{p}\left({\mathrm{D}}_{\mathrm{BS}}\Big|\uptheta, \mathrm{B}{\mathrm{S}}_{\mathrm{eff}},\mathrm{B}\mathrm{S}{*}_{\mathrm{eff}},\mathrm{s}\mathrm{e}{\mathrm{q}}_{\mathrm{err}}\right)\ \mathrm{p}\left({\mathrm{D}}_{\mathrm{oxBS}}\Big|\uptheta, \mathrm{B}{\mathrm{S}}_{\mathrm{eff}},\mathrm{B}\mathrm{S}{*}_{\mathrm{eff}},\mathrm{o}{\mathrm{x}}_{\mathrm{eff}},\mathrm{s}\mathrm{e}{\mathrm{q}}_{\mathrm{err}}\right)\hfill \\ {}=\kern0.5em \left(\begin{array}{c}\hfill {\mathrm{N}}_{\mathrm{BS}}\hfill \\ {}\hfill {\mathrm{N}}_{\mathrm{BS},\mathrm{C}}\hfill \end{array}\right){\mathrm{p}}_{\mathrm{BS}}{\left("C"\right)}^{{\mathrm{N}}_{\mathrm{BS},\mathrm{C}}}{\left(1-{\mathrm{p}}_{\mathrm{BS}}\left("C"\right)\right)}^{{\mathrm{N}}_{\mathrm{BS}}\hbox{-} {\mathrm{N}}_{\mathrm{BS},\mathrm{C}}}\left(\begin{array}{c}\hfill {\mathrm{N}}_{\mathrm{oxBS}}\hfill \\ {}\hfill {\mathrm{N}}_{\mathrm{oxBS},\mathrm{C}}\hfill \end{array}\right){\mathrm{p}}_{\mathrm{oxBS}}{\left("C"\right)}^{{\mathrm{N}}_{\mathrm{oxBS},\mathrm{C}}}{\left(1-{\mathrm{p}}_{\mathrm{oxBS}}\left("C"\right)\right)}^{{\mathrm{N}}_{\mathrm{oxBS}}-{\mathrm{N}}_{\mathrm{oxBS},\mathrm{C}}}.\hfill \end{array} $$

The complete likelihood is obtained by multiplying the likelihoods of all cytosines in the studied regions and in the control oligonucleotides.

Biological variation is modeled hierarchically (see also Figure S2a in Additional file 4) by defining a condition-specific mean μ for methylation proportions, and μ is assigned a Dirichlet prior with hyperparameters α = (0.8, 0.8, 0.8), where α was selected to increase sensitivity of the estimation even with low sequencing coverage. The effect of α on estimation is studied systematically in Figure S15a in Additional file 4. The sensitivity of the methylation estimation is greater and “bias” is smaller (i.e., fewer data are needed to update the posterior), when the values of the elements of α decrease. On the other hand, estimates have larger variance when more a sensitive parameter is used. Thus, in the cases of relatively high coverage we recommend the use of the default value of α.

Replicate specific methylation proportions θ are defined to follow Dir(gμ + 1) distribution, where g represents biological variation around μ and was given a gamma prior with the shape parameter a = 2 and rate parameter b = 2/6. The vector 1 is added in order to prevent concentration of the probability mass in a few components. The presented statistical model is described in detail in Additional file 3.

### Prior and hyperprior definitions

The knowledge on the purity of spike-in controls was incorporated in the model through Dirichlet priors. The parameters of the priors α_C_, α_5mC_ and α_5hmC_ were defined so that they reflected expected and previously reported purities of the dNTP, 5mC dNTP and 5hmC dNTP mixes (Table S1 in Additional file 5) [28].

The probability model of the experimental parameters BS_eff_, BS*_eff_, ox_eff_ and seq_err_ is defined as a hierarchical structure. Each experiment has its own set of parameter values which are drawn from their corresponding prior distributions. The shapes of the prior distributions are in turn controlled by corresponding hyperpriors which are defined by the user.

As the parameters BS_eff_, BS*_eff_, ox_eff_ and seq_err_ represent probabilities, an intuitive way of eliciting the prior knowledge would be by defining Beta distributions through pseudo-counts or by specifying means and standard deviations. However, as the mean and standard deviation of each parameter depend on the experimental setup, we modeled them with hyperparameters. For each of the parameters, the hyperparameter specifying the mean models the expected value of that parameter in each experiment, and the parameter specifying the standard deviation models the spread of the values over separate experiments.

When implementing the hierarchical probabilistic model of the experimental parameters BS_eff_, BS*_eff_, ox_eff_ and seq_err_ and their respective hyperparameters, we decided not to use the straightforward Beta parameterization but instead use normal distributions and transformations of normal distributions. This enables us to use noncentered parameterizations (NCPs) [65], which gives a significantly faster sampler than one implemented with Beta distributions. The parameters BS_eff_, BS*_eff_, ox_eff_ and seq_err_ are modeled with logistic-normal distributions [66]. The unconstrained expected values of the corresponding distributions are modeled with normal distributions and the corresponding standard deviations with log-normal distributions (see Figure S2a in Additional file 4; Table S1 in Additional file 5). The values of the hyperhyperparameters were selected so that they will produce distributions reflecting our prior knowledge on BS_eff_, BS*_eff_, ox_eff_ and seq_err_; that is, BS_eff_ should be close to 1, ox_eff_ should be around 0.95 and BS^*^_eff_ and seq_err_ should be close to zero. The estimation procedure is not sensitive to the selection of the values of the hyperhyperparameters (Figure S15b in Additional file 4). The default values should be applicable for most of the cases. For a more detailed description, see Additional file 3.

### MCMC estimation of posterior distributions

After assigning priors and hyperpriors for the model parameters, the next step is to condition the model on data and derive posterior distribution of the model parameters. We use the Hamiltonian Monte Carlo (HMC) strategy with the No-U-turn (NUTS) sampler [48] to sample the posterior distributions. NUTS as implemented in Stan v2.2.0 [67] was used in all the analyses with the following settings: method = sample algorithm = hmc engine = nuts max_depth = 8 stepsize = 0.02. The default numbers of warm-up (1000) and sampling (1000) iterations were run. The chains were initialized with values sampled from the priors. The convergence of the MCMC chains was monitored using the built-in Gelman and Rubin's convergence diagnostic, the potential scale reduction factor [68].

### Detection of differential methylation

Differential methylation between two conditions is quantified by assessing the difference in the posterior distributions of μ in conditions A and B. For this, we define Δμ = μ_A_ − μ_B_, where the difference is taken element-wise. In addition, the null hypothesis H_0_ and alternative hypothesis H_1_ are defined as Δμ = 0 and Δμ ≠ 0, respectively. The BF is a measure of the evidence in the data D in support of H_1_ over H_0_ BF = p(D|H_1_)/p(D|H_0_). The calculation of the BF requires evaluation of the marginal likelihoods of the data, which unfortunately do not have closed-form solutions. We resort to the Savage-Dickey density ratio for approximating the BF as BF ≃ p(Δμ = 0|H_1_)/p(Δμ = 0|D,H_1_). Next we will go through how we calculated the numerator and denominator.

The value of the probability density function of the difference of two independent Dirichlet random variables at the origin (0, 0, 0) can be solved analytically (see Additional file 3). Thus, if μ_1_, μ_2_ ~ Dir((0.8, 0.8, 0.8)), then p(Δμ = 0|H_1_) = p_μ1-μ2_((0, 0, 0)) ≃ 2.19712.

To calculate the value p(Δμ = 0|H_1_,D), we use two MCMC chains containing posterior samples of μ_A_ and μ_B_, corresponding to the conditions A and B, and estimate the empirical posterior distribution of the difference Δμ. Here the estimation was done using a standard kernel density estimation approach with the Gaussian kernel (the routine scipy.stats.gaussian_kde in SciPy [69]). The density is estimated based on all the pair-wise differences calculated between the samples of the two chains; in the case of N samples per chain there are altogether N^2^ differences used in the kernel density estimation. The bandwidth of the kernel was selected to be one-fourth of the estimate given by Scott’s rule [70]. The scaling factor of ¼ for the bandwidth was included to improve the accuracy of the kernel density estimates. The accuracy of the kernel density estimation was assessed in the following way: 1) sample data from two known Dirichlet distributions; 2) calculate the kernel density estimate for the difference between the two Dirichlet distributed variables using the sampled data; and 3) compare the estimate with the true value obtained using the analytical formula.

We systematically studied the effect of α on the detection of differential methylation (Figure S15c in Additional file 4). Small α values result in more sensitive differential detection estimation and larger BF values (Figure S15c in Additional file 4). Note that the increase in the absolute value of BF is mainly due to the denominator term, which is calculated based on the prior in the Savage-Dickey estimator. In the case of the Jeffreys non-informative prior (α = (0.5,0.5,0.5); which would produce most sensitive methylation estimates), the Savage-Dickey density ratio is not applicable because the denominator calculated based on the prior is always 0.

### Detection of differential methylation at the locus level

At the locus level, Lux accounts for two types of variability: variability between individual cytosines within a locus, and variability in individual cytosine methylation levels between biological replicates. This is achieved by introducing an additional level to the Lux model (Figure S11 in Additional file 4). That is, variation in methylation across a locus is modeled hierarchically by first defining a condition-specific mean μ for methylation proportions in a locus, and μ is assigned a Dirichlet prior with hyperparameters α = (0.8, 0.8, 0.8), where α was selected to increase sensitivity of the estimation even with low sequencing coverage. Methylation proportions ν over individual cytosines within a locus are defined to follow Dir(gμ + 1) distribution, where g represents biological variation around μ and was given a gamma prior with the shape parameter a = 2 and rate parameter b = 2/6. The vector 1 is added in order to prevent concentration of the probability mass in a few components. Finally, replicate-specific methylation proportions θ are defined to follow Dir(fν +1) distribution, where f represents variation around ν and was given a gamma prior with the shape parameter a = 2 and rate parameter b = 2/6. Differential methylation between two conditions is quantified as described above by assessing the difference in the posterior distributions of μ in conditions A and B.

To scan our loci, we used a scanning window approach with window-length 100 bp and step-size 50 bp. In our analysis we only considered those cytosines which were in a CpG context. Moreover, we ignored those windows which had less than three cytosines, as those are better quantified using cytosine-level analysis.

### Defining differentially and similarly methylated cytosines

To compare Lux, MOABS, and FET in detecting differential methylation we have to define sets of differentially and similarly methylated cytosines. This was done by detecting ten top scoring loci and four low scoring loci showing differential 5mC and/or 5hmC levels based on independent CMS-IP and MeDIP measurements [49]. The detection of loci with differential 5mC and 5hmC was done using the MEDIPS tool [71] with 300 bp windows (*p* value < 1e-4). All the covered cytosines in a CpG context (N = 384) were divided into sets of differentially (N = 252) and similarly (N = 132) methylated cytosines based on the aforementioned loci-level information. The same procedure was carried out while defining differentially and similarly methylated windows in Figure S11b in Additional file 4.

DNA modification-sensitive assays like MeDIP and CMS-IP are known to have a CpG density bias. Especially regions with low CpG densities will result in only moderate signals, even when fully methylated. Various methods have been proposed to transform MeDIP-seq-derived count data into beta-like absolute methylation values by correcting for CpG densities [71–74]. However, any experiment-independent bias, like local CpG density, affects each sample the same way. Therefore, no normalization of CpG density or other experiment-independent factors needs to be performed when differential methylation at a fixed region and between samples is calculated. For validation of our method, we have focused on genomic regions identified as differentially methylated (MeDIP) and differentially hydroxymethylated (CMS-IP) comparing conditions and the selected regions all have balanced and elevated CpG densities. Although MeDIP and CMS-IP do not provide information on the single nucleotide level, they have been used to detect differential 5mC and 5hmC successfully. Moreover, it has also been reported that in many cases the methylation levels of several nearby CpG sites are highly correlated. Finally, MeDIP and CMS-IP are independent techniques from BS-seq and oxBS-seq and, thus, provide orthogonal information.

### Using MOABS

To compare Lux with MOABS in detecting differential methylation we first downloaded the MOABS (v.1.2.7) binaries from https://code.google.com/p/moabs/. We generated necessary input files (in the BED format) containing information about methylation calls as described in the MOABS user guide (v.1.2.2). Then, we carried out differential methylation analysis of individual cytosines between two conditions with (“mcomp --doDmrScan = 0 -r c1_r1.bed,c1_r2.bed,c1_r3.bed -r c2_r1.bed,c2_r2.bed,c3_r3.bed -m c1.bed c2.bed -c c1_vs_v2.txt”) or without replicates (“mcomp --doDmrScan 0 -r c1.bed -r c2.bed -c c1_vs_c2.txt”) using the mcomp module as described in the user guide. To carry out differential methylation of windows, we used mcomp (“mcomp -r c1_r1.bed,c1_r2.bed,c1_r3.bed -r c2_r1.bed,c2_r2.bed,c3_r3.bed -m c1.bed c2.bed -c c1_vs_v2.txt” and “mcomp -c c1_vs_v2.txt -f window.bed”) and the obtained *p* values were used. Based on the user guide, mcomp does not support simultaneous analysis of BS-seq and oxBS-seq data, and thus we analyzed BS-seq and oxBS-seq data separately.

### Binomial test with conversion efficiency

We used the binomial test with the conversion efficiencies (BS_Eff_ = 0.99) as described in the supplement of [32] to quantify the presence of 5mC and 5hmC for each CpG. Since [32] does not provide a way to handle replicates, we combined the replicate-specific *p* values using Fisher’s method. We used this strategy to analyze both wild-type and knockout conditions separately. The obtained *p* values therefore provide a proxy for the amount of 5mC and 5hmC; low *p* values correspond to high amounts of cytosine modifications. Using a *p* value threshold we can decide the presence of 5mC and/or 5hmC in both conditions and call a difference in methylation modification levels, which we defined by using the minimum of the two *p* values. Finally, by sliding the *p* value threshold from 0 to 1 we can then generate the ROC graph and the AUC score as illustrated in Figure S8d in Additional file 4.

### Simulation of data

The counts of unconverted read-outs out of N read-outs from BS-seq and oxBS-seq experiments are assumed to be binomially distributed random variables with the derived emission probabilities. The experimental parameters and methylation levels are varied as indicated.

Downsampling was done by sampling data from binomial distributions defined by the parameters estimated from the complete data. That is, for a given cytosine and BS-seq experiment we calculated the fraction of unconverted read-outs, N_BS,C_/N_BS_. This value was used as the success probability parameter, i.e., the probability of observing “C”. Using the defined binomial distribution, we sampled a number of “C” read-outs out of N read-outs. The same procedure was used for oxBS-seq but in that case we calculated the fractions N_oxBS,C_/N_oxBS_.

### Kernel density estimation in the open two-dimensional simplex

A kernel density estimator was applied to data prior to ternary plotting. To deal with compositional data correctly we utilized a published method based on the use of the isometric log-ratio normal kernel (iln) [75].

### Comparison with glucMC-qPCR data

The raw BS-seq and oxBS-seq data sets were downloaded from the European Molecular Biology Laboratory-European Bioinformatics Institute ArrayExpress Archive (E-MTAB-1042). Bismark v0.7.12 [63] was used to align the BS and oxBS reads against the mm9 reference genome. The alignment was done using the single-end Bowtie 2 [64] backend with the following parameters: -N 1 –L 20. The “bismark_methylation_extractor” script distributed with the Bismark aligner was used to extract the number of unconverted and converted read-outs for each cytosine with the following parameters: --cutoff 5 --bedGraph --counts. The PCR primers given in [32] were aligned against the mm9 reference genome and the locations of the CCGG sites within the loci were extracted. The methylation levels of the second cytosine within the CCGG sites were estimated using Lux (α = (0.8, 0.8, 0.8)). The Booth et al. estimates and glucMS-qPCR measurements were taken from [32].

### Integrative analysis of BS-seq, TAB-seq, and fCAB-seq data

First, we derived the statistical model for the simultaneous and integrative analysis of BS-seq, TAB-seq, and fCAB-seq data. The derivation of BS-seq/TAB-seq/fCAB-seq model followed the same principle as the aforementioned derivation of the BS-seq/oxBS-seq model. Briefly, for a given cytosine, we used a Dirichlet random variable of order four to model proportions of different cytosine methylations θ = [p(C), p(5mC), p(5hmC), p(5fC)] (Σθ = 1) simultaneously. Similarly as in the derivation of the BS-seq/oxBS-seq model, we define the effects of BS_eff_, BS*_eff_, lab_eff_, ox_eff_, pro_eff_ and seq_err_ on each of the cytosine modification (C, 5mC, 5hmC, 5fC) and the BS-seq/TAB-seq/fCAB-seq read-outs (Additional file 3; Figure S13 in Additional file 4). Then we derive the BS-seq-, TAB-seq-, and fCAB-seq-specific emission probabilities (propagated probabilities; Additional file 3). Finally, for given a value of θ, we define BS-seq, TAB-seq, and fCAB-seq likelihoods to have binomial distributions as in the BS-seq/oxBS-seq model. Consequently, we can define the complete likelihood function as in the case of the BS-seq/oxBS-seq model.

The preprocessed BS-seq, TAB-seq, and fCAB-seq data sets (GSM1386021, GSM1386028, and GSM1386029) were downloaded from the Gene Expression Omnibus (GEO) database. We limited our analysis to the cytosines (common, maternal, and paternal) on the positive strand because no preprocessed BS-seq data were available for the cytosines on the negative strand. Moreover, we only considered the cytosines (N = 12,860,334) with ≥10× coverage in all three experiments (BS-seq, TAB-seq, and fCAB-seq).

Because no controls were available for all the experimental parameters, we set the values of the experimental parameters to the values reported in the original study [57], i.e., BS_eff_ = 0.99, lab_eff_ = 0.95, ox_eff_ = 0.95, and pro_eff_ = 0.8. Moreover, we assumed that BS*_eff_ = 0.001 and seq_err_ = 0.001. Finally, given the relatively low sequencing coverage in the genome-wide data, we assigned the Jeffreys prior for μ, i.e., μ ~ Dir(α), where α = (0.5, 0.5, 0.5, 0.5).

Next, as with the BS-seq/oxBS-seq model, we used the HMC sampling scheme to estimate the posterior distributions of μ and θ for each of the considered cytosines given the read count data and the values of the experimental parameters.

### Integrative analysis of other derivatives of traditional bisulfite sequencing data

Besides BS-seq, oxBS-seq, TAB-seq and fCAB-seq, Lux can be easily extended to analyze and quantify other oxi-mC-seq data. The main experimental steps and the corresponding parameters for CAB-seq, redBS-seq and MAB-seq are shown in Fig. 6. Details of the propagated probabilities, which are needed to compute the likelihood are shown in Additional file 3.

### Availability of software implementation

A platform-independent implementation of Lux is released under MIT license at https://github.com/tare/Lux/ and as Additional files 1 and 2. We recommend to get the latest version from the GitHub repository.

### Availability of experimental data

The data sets supporting the results of this article are available in the GEO repository under accession number GSE68576.

## Additional files

Additional file 1: A Stan implementation of the Lux method with documentation and a preprocessed data set. (TBZ2 9 kb) Additional file 2: A small example data set. (TBZ2 8 kb) Additional file 3: Methods document. (PDF 138 kb) Additional file 4: Figures S1 to S15 with legends. (PDF 8537 kb) Additional file 5: Tables S1 to S7 with legends. (PDF 4341 kb)

## References

[1] Csankovszki G, Nagy A, Jaenisch R (2001). Synergism of Xist RNA, DNA methylation, and histone hypoacetylation in maintaining X chromosome inactivation. J Cell Biol.

[2] Li E, Beard C, Jaenisch R (1993). Role for DNA methylation in genomic imprinting. Nature.

[3] Chen RZ, Pettersson U, Beard C, Jackson-Grusby L, Jaenisch R (1998). DNA hypomethylation leads to elevated mutation rates. Nature.

[4] Smith ZD, Meissner A (2013). DNA methylation: roles in mammalian development. Nat Rev Genet.

[5] Bergman Y, Cedar H (2013). DNA methylation dynamics in health and disease. Nat Struct Mol Biol.

[6] Kulis M, Esteller M (2010). DNA methylation and cancer. Adv Genet..

[7] Lister R, Pelizzola M, Dowen RH, Hawkins RD, Hon G, Tonti-Filippini J (2009). Human DNA methylomes at base resolution show widespread epigenomic differences. Nature.

[8] Hon GC, Rajagopal N, Shen Y, McCleary DF, Yue F, Dang MD (2013). Epigenetic memory at embryonic enhancers identified in DNA methylation maps from adult mouse tissues. Nat Genet.

[9] Ziller MJ, Gu H, Muller F, Donaghey J, Tsai LT, Kohlbacher O (2013). Charting a dynamic DNA methylation landscape of the human genome. Nature.

[10] Shukla S, Kavak E, Gregory M, Imashimizu M, Shutinoski B, Kashlev M (2011). CTCF-promoted RNA polymerase II pausing links DNA methylation to splicing. Nature.

[11] Hu S, Wan J, Su Y, Song Q, Zeng Y, Nguyen HN (2013). DNA methylation presents distinct binding sites for human transcription factors. Elife..

[12] Lister R, Mukamel EA, Nery JR, Urich M, Puddifoot CA, Johnson ND (2013). Global epigenomic reconfiguration during mammalian brain development. Science.

[13] Heyn H, Li N, Ferreira HJ, Moran S, Pisano DG, Gomez A (2012). Distinct DNA methylomes of newborns and centenarians. Proc Natl Acad Sci U S A.

[14] Tahiliani M, Koh KP, Shen Y, Pastor WA, Bandukwala H, Brudno Y (2009). Conversion of 5-methylcytosine to 5-hydroxymethylcytosine in mammalian DNA by MLL partner TET1. Science.

[15] Ito S, Shen L, Dai Q, Wu SC, Collins LB, Swenberg JA (2011). Tet proteins can convert 5-methylcytosine to 5-formylcytosine and 5-carboxylcytosine. Science.

[16] Pastor WA, Aravind L, Rao A (2013). TETonic shift: biological roles of TET proteins in DNA demethylation and transcription. Nat Rev Mol Cell Biol.

[17] Kohli RM, Zhang Y (2013). TET enzymes, TDG and the dynamics of DNA demethylation. Nature.

[18] Bachman M, Uribe-Lewis S, Yang X, Williams M, Murrell A, Balasubramanian S (2014). 5-Hydroxymethylcytosine is a predominantly stable DNA modification. Nat Chem.

[19] Bachman M, Uribe-Lewis S, Yang X, Burgess HE, Iurlaro M, Reik W (2015). 5-Formylcytosine can be a stable DNA modification in mammals. Nat Chem Biol.

[20] Wang L, Zhou Y, Xu L, Xiao R, Lu X, Chen L (2015). Molecular basis for 5-carboxycytosine recognition by RNA polymerase II elongation complex. Nature.

[21] Ko M, Huang Y, Jankowska AM, Pape UJ, Tahiliani M, Bandukwala HS (2010). Impaired hydroxylation of 5-methylcytosine in myeloid cancers with mutant TET2. Nature.

[22] Pastor WA, Pape UJ, Huang Y, Henderson HR, Lister R, Ko M (2011). Genome-wide mapping of 5-hydroxymethylcytosine in embryonic stem cells. Nature.

[23] Huang Y, Pastor WA, Zepeda-Martinez JA, Rao A (2012). The anti-CMS technique for genome-wide mapping of 5-hydroxymethylcytosine. Nat Protoc.

[24] Pastor WA, Huang Y, Henderson HR, Agarwal S, Rao A (2012). The GLIB technique for genome-wide mapping of 5-hydroxymethylcytosine. Nat Protoc.

[25] Song CX, Szulwach KE, Fu Y, Dai Q, Yi C, Li X (2011). Selective chemical labeling reveals the genome-wide distribution of 5-hydroxymethylcytosine. Nat Biotechnol.

[26] Robinson MD, Stirzaker C, Statham AL, Coolen MW, Song JZ, Nair SS (2010). Evaluation of affinity-based genome-wide DNA methylation data: effects of CpG density, amplification bias, and copy number variation. Genome Res.

[27] Chodavarapu RK, Feng S, Bernatavichute YV, Chen PY, Stroud H, Yu Y (2010). Relationship between nucleosome positioning and DNA methylation. Nature.

[28] Yu M, Hon GC, Szulwach KE, Song CX, Zhang L, Kim A (2012). Base-resolution analysis of 5-hydroxymethylcytosine in the mammalian genome. Cell.

[29] Rein T, DePamphilis ML, Zorbas H (1998). Identifying 5-methylcytosine and related modifications in DNA genomes. Nucleic Acids Res.

[30] Frommer M, McDonald LE, Millar DS, Collis CM, Watt F, Grigg GW (1992). A genomic sequencing protocol that yields a positive display of 5-methylcytosine residues in individual DNA strands. Proc Natl Acad Sci U S A.

[31] Huang Y, Pastor WA, Shen Y, Tahiliani M, Liu DR, Rao A (2010). The behaviour of 5-hydroxymethylcytosine in bisulfite sequencing. PLoS One.

[32] Booth MJ, Branco MR, Ficz G, Oxley D, Krueger F, Reik W (2012). Quantitative sequencing of 5-methylcytosine and 5-hydroxymethylcytosine at single-base resolution. Science.

[33] Plongthongkum N, Diep DH, Zhang K (2014). Advances in the profiling of DNA modifications: cytosine methylation and beyond. Nat Rev Genet.

[34] Song CX, Szulwach KE, Dai Q, Fu Y, Mao SQ, Lin L (2013). Genome-wide profiling of 5-formylcytosine reveals its roles in epigenetic priming. Cell.

[35] Booth MJ, Marsico G, Bachman M, Beraldi D, Balasubramanian S (2014). Quantitative sequencing of 5-formylcytosine in DNA at single-base resolution. Nat Chem.

[36] Lu X, Song CX, Szulwach K, Wang Z, Weidenbacher P, Jin P (2013). Chemical modification-assisted bisulfite sequencing (CAB-Seq) for 5-carboxylcytosine detection in DNA. J Am Chem Soc.

[37] Wu H, Wu X, Shen L, Zhang Y (2014). Single-base resolution analysis of active DNA demethylation using methylase-assisted bisulfite sequencing. Nat Biotechnol.

[38] Kumaki Y, Oda M, Okano M (2008). QUMA: quantification tool for methylation analysis. Nucleic Acids Res.

[39] Rohde C, Zhang Y, Reinhardt R, Jeltsch A (2010). BISMA--fast and accurate bisulfite sequencing data analysis of individual clones from unique and repetitive sequences. BMC Bioinformatics..

[40] Akalin A, Kormaksson M, Li S, Garrett-Bakelman FE, Figueroa ME, Melnick A (2012). methylKit: a comprehensive R package for the analysis of genome-wide DNA methylation profiles. Genome Biol.

[41] Benoukraf T, Wongphayak S, Hadi LH, Wu M, Soong R (2013). GBSA: a comprehensive software for analysing whole genome bisulfite sequencing data. Nucleic Acids Res.

[42] Hansen KD, Langmead B, Irizarry RA (2012). BSmooth: from whole genome bisulfite sequencing reads to differentially methylated regions. Genome Biol.

[43] Sun D, Xi Y, Rodriguez B, Park HJ, Tong P, Meong M (2014). MOABS: model based analysis of bisulfite sequencing data. Genome Biol.

[44] Feng H, Conneely KN, Wu H (2014). A Bayesian hierarchical model to detect differentially methylated loci from single nucleotide resolution sequencing data. Nucleic Acids Res.

[45] Burger L, Gaidatzis D, Schubeler D, Stadler MB (2013). Identification of active regulatory regions from DNA methylation data. Nucleic Acids Res.

[46] Dolzhenko E, Smith AD (2014). Using beta-binomial regression for high-precision differential methylation analysis in multifactor whole-genome bisulfite sequencing experiments. BMC Bioinformatics..

[47] Qu J, Zhou M, Song Q, Hong EE, Smith AD (2013). MLML: consistent simultaneous estimates of DNA methylation and hydroxymethylation. Bioinformatics.

[48] Hoffman MD, Gelman A. The No-U-Turn Sampler: adaptively setting path lengths in Hamiltonian Monte Carlo. J Mach Learn Res. 2013, in press.

[49] Huang Y, Chavez L, Chang X, Wang X, Pastor WA, Kang J (2014). Distinct roles of the methylcytosine oxidases Tet1 and Tet2 in mouse embryonic stem cells. Proc Natl Acad Sci U S A.

[50] Katz Y, Wang ET, Airoldi EM, Burge CB (2010). Analysis and design of RNA sequencing experiments for identifying isoform regulation. Nat Methods.

[51] Shen Y, Yue F, McCleary DF, Ye Z, Edsall L, Kuan S (2012). A map of the cis-regulatory sequences in the mouse genome. Nature.

[52] Tsagaratou A, Aijo T, Lio CW, Yue X, Huang Y, Jacobsen SE (2014). Dissecting the dynamic changes of 5-hydroxymethylcytosine in T-cell development and differentiation. Proc Natl Acad Sci U S A.

[53] Zhang JA, Mortazavi A, Williams BA, Wold BJ, Rothenberg EV (2012). Dynamic transformations of genome-wide epigenetic marking and transcriptional control establish T cell identity. Cell.

[54] Kirigin FF, Lindstedt K, Sellars M, Ciofani M, Low SL, Jones L (2012). Dynamic microRNA gene transcription and processing during T cell development. J Immunol.

[55] Rothenberg EV, Taghon T (2005). Molecular genetics of T cell development. Annu Rev Immunol..

[56] Gordan R, Shen N, Dror I, Zhou T, Horton J, Rohs R (2013). Genomic regions flanking E-box binding sites influence DNA binding specificity of bHLH transcription factors through DNA shape. Cell Rep.

[57] Wang L, Zhang J, Duan J, Gao X, Zhu W, Lu X (2014). Programming and inheritance of parental DNA methylomes in mammals. Cell.

[58] Rishi V, Bhattacharya P, Chatterjee R, Rozenberg J, Zhao J, Glass K (2010). CpG methylation of half-CRE sequences creates C/EBPalpha binding sites that activate some tissue-specific genes. Proc Natl Acad Sci U S A.

[59] Spruijt CG, Gnerlich F, Smits AH, Pfaffeneder T, Jansen PW, Bauer C (2013). Dynamic readers for 5-(hydroxy)methylcytosine and its oxidized derivatives. Cell.

[60] Koh KP, Yabuuchi A, Rao S, Huang Y, Cunniff K, Nardone J (2011). Tet1 and Tet2 regulate 5-hydroxymethylcytosine production and cell lineage specification in mouse embryonic stem cells. Cell Stem Cell.

[61] Ko M, An J, Bandukwala HS, Chavez L, Aijo T, Pastor WA (2013). Modulation of TET2 expression and 5-methylcytosine oxidation by the CXXC domain protein IDAX. Nature.

[62] Booth MJ, Ost TW, Beraldi D, Bell NM, Branco MR, Reik W (2013). Oxidative bisulfite sequencing of 5-methylcytosine and 5-hydroxymethylcytosine. Nat Protoc.

[63] Krueger F, Andrews SR (2011). Bismark: a flexible aligner and methylation caller for Bisulfite-Seq applications. Bioinformatics.

[64] Langmead B, Salzberg SL (2012). Fast gapped-read alignment with Bowtie 2. Nat Methods.

[65] Papaspiliopoulos O, Roberts GO, Sköld M (2007). A general framework for the parametrization of hierarchical models. Stat Sci.

[66] Aitchison J, Shen SM (1980). Logistic-normal distributions: some properties and uses. Biometrika.

[67] Bob Carpenter, Andrew Gelman, Matt Hoffman, Daniel Lee, Ben Goodrich, Michael Betancourt, Michael A. Brubaker, Jiqiang Guo, Peter Li, and Allen Riddell. 2016. Stan: A probabilistic programming language. Journal of Statistical Software (in press). 10.18637/jss.v076.i01 PMC9788645 36568334

[68] Gelman A, Rubin DB (1992). Inference from iterative simulation using multiple sequences. Stat Sci.

[69] Jones E, Oliphant E, Peterson P, et al. SciPy: Open Source Scientific Tools for Python, 2001, http://www.scipy.org/[Online; accessed 2016-03-06].

[70] Scott DW. Multivariate density estimation: theory, practice, and visualization. New York: Wiley; 2009.

[71] Chavez L, Jozefczuk J, Grimm C, Dietrich J, Timmermann B, Lehrach H (2010). Computational analysis of genome-wide DNA methylation during the differentiation of human embryonic stem cells along the endodermal lineage. Genome Res.

[72] Down TA, Rakyan VK, Turner DJ, Flicek P, Li H, Kulesha E (2008). A Bayesian deconvolution strategy for immunoprecipitation-based DNA methylome analysis. Nat Biotechnol.

[73] Riebler A, Menigatti M, Song JZ, Statham AL, Stirzaker C, Mahmud N (2014). BayMeth: improved DNA methylation quantification for affinity capture sequencing data using a flexible Bayesian approach. Genome Biol.

[74] Lienhard M, Grimm C, Morkel M, Herwig R, Chavez L (2014). MEDIPS: genome-wide differential coverage analysis of sequencing data derived from DNA enrichment experiments. Bioinformatics.

[75] Chacón JE, Mateu-Figueras G, Martín-Fernández JA (2011). Gaussian kernels for density estimation with compositional data. Comput Geosci.

