# Supplementary material for: A probabilistic generative model for quantification of DNA modifications enables analysis of demethylation pathways
Source: Genome Biol. 2016 Mar 14;17:49. doi: 10.1186/s13059-016-0911-6 (PMC4792102; doi:10.1186/s13059-016-0911-6)
Supplement: Additional file 3: — Methods document. (PDF 138 kb) [file 13059_2016_911_MOESM3_ESM.pdf]

A probabilistic model for accurate quantification of DNA methylation modifications.

Supplemental Methods

Tarmo Äijö, Yun Huang, Henrik Mannerström, Lukas Chavez, Ageliki Tsagaratou, Anjana Rao, and Harri Lähdesmäki

March 11, 2016

On the statistical model of Lux

In this section we go through the statistical model of Lux in detail. The graphical model of Lux is depicted in **Suppl. Fig. 2** and the values of the hyperprior parameters are also listed in **Suppl. Table 1**.

Parameters

In this subsection we describe the model parameters.

$$\theta^{\text{control},i,j} = \left(p^{\text{control},i,j}(\text{“C”}),\, p^{\text{control},i,j}(\text{“5mC”}),\, p^{\text{control},i,j}(\text{“5hmC”})\right),$$

$$i = 1, \ldots, N_{\text{control cytosines}},\; j = 1, \ldots, N_{\text{replicates}}$$

Description

Represents the proportions of the considered cytosine modifications for control cytosines ($\forall i, j : \sum \theta^{\text{control},i,j} = 1$). Note that we model control cytosines indepedently across replicates, i.e. there is no hierarchical level for modeling biological variation on them.

Distribution

$$\theta^{\text{control},i,j} \sim \text{Dir}(\alpha^{\text{control},i,j})$$

$\theta^{i,j} = \left(p^{i,j}(\text{“C”}),\, p^{i,j}(\text{“5mC”}),\, p^{i,j}(\text{“5hmC”})\right),\, i = 1, \ldots, N_{\text{cytosines}},\, j = 1, \ldots, N_{\text{replicates}}$

Description

Represents the proportions of the considered cytosine modifications for noncontrol cytosines ($\forall i, j : \sum \theta^{i,j} = 1$).

Distribution

$\theta^{i,j} \sim \text{Dir}(g^i \mu_\theta^i + \mathbf{1})$

$\text{BS}_{\text{eff}}^{j},\, j = 1, \ldots, N_{\text{replicates}}$

Description

Represents the probability of successful bisulphite conversion, i.e. conversion of unmethylated cytosine to uracil.

Distribution

Is defined by parameters $\mu_{\text{BS}_{\text{eff}}}$ and $\sigma_{\text{BS}_{\text{eff}}}$ such that $\text{logit}(\text{BS}_{\text{eff}}^{j}) \sim \mathcal{N}(\mu_{\text{BS}_{\text{eff}}}, \sigma_{\text{BS}_{\text{eff}}})$.

$\text{BS}_{\text{eff}}^{*,j},\, j = 1, \ldots, N_{\text{replicates}}$

Description

Represents the probability of inaccurate bisulphite conversion, i.e. conversion of 5mC or 5hmC to uracil.

Distribution

Is defined by parameters $\mu_{\text{BS}_{\text{eff}}^*}$ and $\sigma_{\text{BS}_{\text{eff}}^*}$ such that $\text{logit}(\text{BS}_{\text{eff}}^{*,j}) \sim \mathcal{N}(\mu_{\text{BS}_{\text{eff}}^*}, \sigma_{\text{BS}_{\text{eff}}^*})$.

$\text{ox}_{\text{eff}}^{j},\, j = 1, \ldots, N_{\text{replicates}}$

Description

Represents the probability of successful oxidation, i.e. oxidation of 5hmC to 5fC.

Distribution

Is defined by parameters $\mu_{\text{ox}_{\text{eff}}}$ and $\sigma_{\text{ox}_{\text{eff}}}$ such that $\text{logit}(\text{ox}_{\text{eff}}^{j}) \sim \mathcal{N}(\mu_{\text{ox}_{\text{eff}}}, \sigma_{\text{ox}_{\text{eff}}})$.

$\text{seq}_{\text{err}}^{j}$, $j = 1, \ldots, N_{\text{replicates}}$

Description

Represents the probability of sequencing error, i.e. sequencing C instead of T or vice versa.

Distribution

Is defined by parameters $\mu_{\text{seq}_{\text{err}}}$ and $\sigma_{\text{seq}_{\text{err}}}$ such that $\text{logit}(\text{seq}_{\text{err}}^{j}) \sim \mathcal{N}(\mu_{\text{seq}_{\text{err}}}, \sigma_{\text{seq}_{\text{err}}})$.

Hyperparameters

In this subsection we describe the hyperparameters of the model.

$\alpha^{\text{control},i,j} = (\alpha_{\text{C}}^{\text{control},i,j}, \alpha_{\text{5mC}}^{\text{control},i,j}, \alpha_{\text{5hmC}}^{\text{control},i,j})$, $i = 1, \ldots, N_{\text{control cytosines}}$, $j = 1, \ldots, N_{\text{replicates}}$

Description

Defines the priors for the proportions of the considered cytosine modifications for control cytosines, i.e. $\theta^{\text{control},i,j} \sim \text{Dir}(\alpha^{\text{control},i,j})$.

Value In the case of the C, 5mC and 5hmC control cytosines the values are $\alpha_{\text{C}}^{\text{control},i,j} = (1000,\ 1,\ 1)$, $\alpha_{\text{5mC}}^{\text{control},i,j} = (1,\ 1000,\ 1)$, and $\alpha_{\text{5hmC}}^{\text{control},i,j} = (6,\ 2,\ 72)$, respectively, for all i and j.

g^{i}, $i = 1, \ldots, N_{\text{cytosines}}$

Description

Controls the variance of the replicate-specific proportions of cytosine modifications for noncontrol cytosines $\theta_{i,j}$.

Distribution

$g^{j} \sim \text{Gamma}(\psi_g^1,\ \psi_g^2)$, where $\psi_g^1 = 2$, $\psi_g^2 = 2/6$

μ_θ^i, $i = 1, \ldots, N_{\text{cytosines}}$

Description

Represents the proportions of the considered cytosine modifications for noncontrol cytosines across replicates.

Distribution

$\mu_\theta^i \sim \text{Dir}(\alpha)$, where $\alpha = (0.8,\, 0.8,\, 0.8)$

$\mu_{\text{BS}_{\text{eff}}}$

Description

Controls the expected value of logit(BS_{eff}) across replicates.

Distribution

$\mu_{\text{BS}_{\text{eff}}} \sim \mathcal{N}(\psi^{\mu,\mu}_{\text{BS}_{\text{eff}}}, \psi^{\mu,\sigma}_{\text{BS}_{\text{eff}}})$, where $\psi^{\mu,\mu}_{\text{BS}_{\text{eff}}} = 2$ and $\psi^{\mu,\sigma}_{\text{BS}_{\text{eff}}} = 1.29$

$\sigma_{\text{BS}_{\text{eff}}}$

Description

Controls the standard devation of logit(BS_{eff}) across replicates.

Distribution

$\sigma_{\text{BS}_{\text{eff}}} \sim \ln\mathcal{N}(\psi^{\sigma,\mu}_{\text{BS}_{\text{eff}}}, \psi^{\sigma,\sigma}_{\text{BS}_{\text{eff}}})$, where $\psi^{\sigma,\mu}_{\text{BS}_{\text{eff}}} = 0.4$ and $\psi^{\sigma,\sigma}_{\text{BS}_{\text{eff}}} = 0.5$

$\mu_{\text{BS}^*_{\text{eff}}}$

Description

Controls the expected value of logit$(\text{BS}^*_{\text{eff}})$ across replicates.

Distribution

$\mu_{\text{BS}^*_{\text{eff}}} \sim \mathcal{N}(\psi^{\mu,\mu}_{\text{BS}^*_{\text{eff}}}, \psi^{\mu,\sigma}_{\text{BS}^*_{\text{eff}}})$, where $\psi^{\mu,\mu}_{\text{BS}^*_{\text{eff}}} = -3$ and $\psi^{\mu,\sigma}_{\text{BS}^*_{\text{eff}}} = 1.29$

$\sigma_{\text{BS}^*_{\text{eff}}}$

Description

Controls the standard deviation of logit$(\text{BS}^*_{\text{eff}})$ across replicates.

Distribution

$\sigma_{\text{BS}^*_{\text{eff}}} \sim \ln\mathcal{N}(\psi^{\sigma,\mu}_{\text{BS}^*_{\text{eff}}}, \psi^{\sigma,\sigma}_{\text{BS}^*_{\text{eff}}})$, where $\psi^{\sigma,\mu}_{\text{BS}^*_{\text{eff}}} = 0.4$ and $\psi^{\sigma,\sigma}_{\text{BS}^*_{\text{eff}}} = 0.5$

$\mu_{\text{ox}_{\text{eff}}}$

Description

Controls the expected value of logit(ox_{eff}) across replicates.

Distribution

$\mu_{\text{ox}_{\text{eff}}} \sim \mathcal{N}(\psi^{\mu,\mu}_{\text{ox}_{\text{eff}}}, \psi^{\mu,\sigma}_{\text{ox}_{\text{eff}}})$, where $\psi^{\mu,\mu}_{\text{ox}_{\text{eff}}} = 2$ and $\psi^{\mu,\sigma}_{\text{ox}_{\text{eff}}} = 1.29$

$\sigma_{\text{ox}_{\text{eff}}}$

Description

Controls the standard deviation of logit(ox_{eff}) across replicates.

Distribution

$\sigma_{\text{ox}_{\text{eff}}} \sim \ln\mathcal{N}(\psi^{\sigma,\mu}_{\text{ox}_{\text{eff}}}, \psi^{\sigma,\sigma}_{\text{ox}_{\text{eff}}})$, where $\psi^{\sigma,\mu}_{\text{ox}_{\text{eff}}} = 0.4$ and $\psi^{\sigma,\sigma}_{\text{ox}_{\text{eff}}} = 0.5$

$\mu_{\text{seq}_{\text{err}}}$

Description

Controls the expected value of logit$(\text{seq}_{\text{err}})$ across replicates.

Distribution

$\mu_{\text{seq}_{\text{err}}} \sim \mathcal{N}(\psi^{\mu,\mu}_{\text{seq}_{\text{err}}}, \psi^{\mu,\sigma}_{\text{seq}_{\text{err}}})$, where $\psi^{\mu,\mu}_{\text{seq}_{\text{err}}} = -3$ and $\psi^{\mu,\sigma}_{\text{seq}_{\text{err}}} = 1.29$

$\sigma_{\text{seq}_{\text{err}}}$

Description

Controls the standard deviation of $\text{logit}(\text{seq}_{\text{err}})$ across replicates.

Distribution

$\sigma_{\text{seq}_{\text{err}}} \sim \ln\mathcal{N}(\psi^{\sigma,\mu}_{\text{seq}_{\text{err}}}, \psi^{\sigma,\sigma}_{\text{seq}_{\text{err}}})$, where $\psi^{\sigma,\mu}_{\text{seq}_{\text{err}}} = 0.4$ and $\psi^{\sigma,\sigma}_{\text{seq}_{\text{err}}} = 0.5$

Data

In this subsection we describe the data.

$N^{\text{control},i,j}_{\text{BS,C}}$, $i = 1, \ldots, N_{\text{control cytosines}}$, $j = 1, \ldots, N_{\text{replicates}}$

Description

The count of "C" read-outs for the ith control cytosine in the jth BS-seq experiment.

$N^{\text{control},i,j}_{\text{BS}}$, $i = 1, \ldots, N_{\text{control cytosines}}$, $j = 1, \ldots, N_{\text{replicates}}$

Description

The count of total read-outs for the ith control cytosine in the jth BS-seq experiment.

$N^{\text{control},i,j}_{\text{oxBS,C}}$, $i = 1, \ldots, N_{\text{control cytosines}}$, $j = 1, \ldots, N_{\text{replicates}}$

Description

The count of "C" read-outs for the ith control cytosine in the jth oxBS-seq experiment.

$N^{\text{control},i,j}_{\text{oxBS}}$, $i = 1, \ldots, N_{\text{control cytosines}}$, $j = 1, \ldots, N_{\text{replicates}}$

Description

The count of total read-outs for the ith control cytosine in the jth oxBS-seq experiment.

$N^{i,j}_{\text{BS,C}}$, $i = 1, \ldots, N_{\text{cytosines}}$, $j = 1, \ldots, N_{\text{replicates}}$

Description

The count of "C" read-outs for the ith noncontrol cytosine in the jth BS-seq experiment.

$N_{\text{BS}}^{i,j},\ i=1,\dots,N_{\text{cytosines}},\ j=1,\dots,N_{\text{replicates}}$

Description

The count of total read-outs for the ith noncontrol cytosine in the jth BS-seq experiment.

$N_{\text{oxBS,C}}^{i,j},\ i=1,\dots,N_{\text{cytosines}},\ j=1,\dots,N_{\text{replicates}}$

Description

The count of "C" read-outs for the ith noncontrol cytosine in the jth oxBS-seq experiment.

$N_{\text{oxBS}}^{i,j},\ i=1,\dots,N_{\text{cytosines}},\ j=1,\dots,N_{\text{replicates}}$

Description

The count of total read-outs for the ith noncontrol cytosine in the jth oxBS-seq experiment.

Likelihood

In this subsection we start with the combination of BS-seq and oxBS-seq data sets and state the likelihood in the general form, i.e. for replicates, and control and noncontrol cytosines. First, let us denote

$$\mathcal{D}^{\text{control}} = \left\{N_{\text{BS,C}}^{\text{control},i,j}, N_{\text{BS}}^{\text{control},i,j}, N_{\text{oxBS,C}}^{\text{control},i,j}, N_{\text{oxBS}}^{\text{control},i,j}\right\}_{\substack{i=1,\dots,N_{\text{control cytosines}} \\ j=1,\dots,N_{\text{replicates}}}},$$

$$\mathcal{D} = \left\{N_{\text{BS,C}}^{i,j}, N_{\text{BS}}^{i,j}, N_{\text{oxBS,C}}^{i,j}, N_{\text{oxBS}}^{i,j}\right\}_{\substack{i=1,\dots,N_{\text{cytosines}} \\ j=1,\dots,N_{\text{replicates}}}},$$

$$\Theta^{\text{control}} = \left\{\theta^{\text{control},i,j}\right\}_{\substack{i=1,\dots,N_{\text{control cytosines}} \\ j=1,\dots,N_{\text{replicates}}}},$$

$$\Theta = \left\{\theta^{i,j}\right\}_{\substack{i=1,\dots,N_{\text{cytosines}} \\ j=1,\dots,N_{\text{replicates}}}},$$

and

$$\Omega = \left\{\text{BS}_{\text{eff}}^{j}, \text{BS}_{\text{eff}}^{*,j}, \text{ox}_{\text{eff}}^{j}, \text{seq}_{\text{err}}^{j}\right\}_{j=1,\dots,N_{\text{replicates}}}.$$

Then the likelihood of the data $\mathcal{D}^{\text{control}}$ and $\mathcal{D}$ under the binomial model, assuming conditional independency, and given the parameters Θ^{control}, Θ, and Ω is

$$\begin{aligned}
&p(\mathcal{D}^{\text{control}}, \mathcal{D}|\Theta^{\text{control}}, \Theta, \Omega) = p(\mathcal{D}^{\text{control}}|\Theta^{\text{control}}, \Omega)p(\mathcal{D}|\Theta, \Omega) \\
&= \prod_{j=1}^{N_{\text{replicates}}} \left[\left(\prod_{i=1}^{N_{\text{control cytosines}}} p(\mathcal{D}^{\text{control}}|\Theta^{\text{control}}, \Omega) \right) \left(\prod_{i=1}^{N_{\text{cytosines}}} p(\mathcal{D}|\Theta, \Omega \right) \right] \\
&= \prod_{j=1}^{N_{\text{replicates}}} \left[\left(\prod_{i=1}^{N_{\text{control cytosines}}} \binom{N_{\text{BS}}^{\text{control},i,j}}{N_{\text{BS,C}}^{\text{control},i,j}} p_{\text{BS}}^{\text{control},i,j}(\text{“C”})^{N_{\text{BS,C}}^{\text{control},i,j}} \left(1 - p_{\text{BS}}^{\text{control},i,j}(\text{“C”})\right)^{N_{\text{BS}}^{\text{control},i,j} - N_{\text{BS,C}}^{\text{control},i,j}} \right. \right. \\
&\left. \binom{N_{\text{oxBS}}^{\text{control},i,j}}{N_{\text{oxBS,C}}^{\text{control},i,j}} p_{\text{oxBS}}^{\text{control},i,j}(\text{“C”})^{N_{\text{oxBS,C}}^{\text{control},i,j}} \left(1 - p_{\text{oxBS}}^{\text{control},i,j}(\text{“C”})\right)^{N_{\text{oxBS}}^{\text{control},i,j} - N_{\text{oxBS,C}}^{\text{control},i,j}} \right) \\
&\left(\prod_{i=1}^{N_{\text{cytosines}}} \binom{N_{\text{BS}}^{i,j}}{N_{\text{BS,C}}^{i,j}} p_{\text{BS}}^{i,j}(\text{“C”})^{N_{\text{BS,C}}^{i,j}} \left(1 - p_{\text{BS}}^{i,j}(\text{“C”})\right)^{N_{\text{BS}}^{i,j} - N_{\text{BS,C}}^{i,j}} \right. \\
&\left. \left. \binom{N_{\text{oxBS}}^{i,j}}{N_{\text{oxBS,C}}^{i,j}} p_{\text{oxBS}}^{i,j}(\text{“C”})^{N_{\text{oxBS,C}}^{i,j}} \left(1 - p_{\text{oxBS}}^{i,j}(\text{“C”})\right)^{N_{\text{oxBS}}^{i,j} - N_{\text{oxBS,C}}^{i,j}} \right) \right],
\end{aligned} \tag{1}$$

where

$$\begin{aligned}
p_{\text{BS}}^{\text{control},i,j}(\text{“C”}) =& p^{\text{control},i,j}(\text{“C”}) \left[(1 - \text{BS}_{\text{eff}}^{j})(1 - \text{seq}_{\text{err}}^{j}) + \text{BS}_{\text{eff}}^{j}\text{seq}_{\text{err}}^{j} \right] + \\
& p^{\text{control},i,j}(\text{“5mC”}) \left[(1 - \text{BS}_{\text{eff}}^{*,j})(1 - \text{seq}_{\text{err}}^{j}) + \text{BS}_{\text{eff}}^{*,j}\text{seq}_{\text{err}}^{j} \right] + \\
& p^{\text{control},i,j}(\text{“5hmC”}) \left[(1 - \text{BS}_{\text{eff}}^{*,j})(1 - \text{seq}_{\text{err}}^{j}) + \text{BS}_{\text{eff}}^{*,j}\text{seq}_{\text{err}}^{j} \right],
\end{aligned} \tag{2}$$

$$\begin{aligned}
p_{\text{oxBS}}^{\text{control},i,j}(\text{“C”}) =& p^{\text{control},i,j}(\text{“C”}) \left[(1 - \text{BS}_{\text{eff}}^{j})(1 - \text{seq}_{\text{err}}^{j}) + \text{BS}_{\text{eff}}^{j}\text{seq}_{\text{err}}^{j} \right] + \\
& p^{\text{control},i,j}(\text{“5mC”}) \left[(1 - \text{BS}_{\text{eff}}^{*,j})(1 - \text{seq}_{\text{err}}^{j}) + \text{BS}_{\text{eff}}^{*,j}\text{seq}_{\text{err}}^{j} \right] + \\
& p^{\text{control},i,j}(\text{“5hmC”}) \left[\text{ox}_{\text{eff}}^{j}[(1 - \text{BS}_{\text{eff}}^{j})(1 - \text{seq}_{\text{err}}^{j}) + \text{BS}_{\text{eff}}^{j}\text{seq}_{\text{err}}^{j}] + \right. \\
& \left. (1 - \text{ox}_{\text{eff}}^{j})[(1 - \text{BS}_{\text{eff}}^{*,j})(1 - \text{seq}_{\text{err}}^{j}) + \text{BS}_{\text{eff}}^{*,j}\text{seq}_{\text{err}}^{j}] \right],
\end{aligned} \tag{3}$$

$$\begin{aligned}
p_{\text{BS}}^{i,j}(\text{“C”}) =& p^{i,j}(\text{“C”}) \left[(1 - \text{BS}_{\text{eff}}^{j})(1 - \text{seq}_{\text{err}}^{j}) + \text{BS}_{\text{eff}}^{j}\text{seq}_{\text{err}}^{j} \right] + \\
& p^{i,j}(\text{“5mC”}) \left[(1 - \text{BS}_{\text{eff}}^{*,j})(1 - \text{seq}_{\text{err}}^{j}) + \text{BS}_{\text{eff}}^{*,j}\text{seq}_{\text{err}}^{j} \right] + \\
& p^{i,j}(\text{“5hmC”}) \left[(1 - \text{BS}_{\text{eff}}^{*,j})(1 - \text{seq}_{\text{err}}^{j}) + \text{BS}_{\text{eff}}^{*,j}\text{seq}_{\text{err}}^{j} \right],
\end{aligned} \tag{4}$$

and

$$
\begin{aligned}
p^{i,j}_{\text{oxBS}}(\text{“C”}) =& p^{i,j}(\text{“C”}) \left[(1-\text{BS}^{j}_{\text{eff}})(1-\text{seq}^{j}_{\text{err}}) + \text{BS}^{j}_{\text{eff}}\text{seq}^{j}_{\text{err}}\right] + \\
& p^{i,j}(\text{“5mC”}) \left[(1-\text{BS}^{*,j}_{\text{eff}})(1-\text{seq}^{j}_{\text{err}}) + \text{BS}^{*,j}_{\text{eff}}\text{seq}^{j}_{\text{err}}\right] + \\
& p^{i,j}(\text{“5hmC”}) \Big[\text{ox}^{j}_{\text{eff}}[(1-\text{BS}^{j}_{\text{eff}})(1-\text{seq}^{j}_{\text{err}}) + \text{BS}^{j}_{\text{eff}}\text{seq}^{j}_{\text{err}}] + \\
& (1-\text{ox}^{j}_{\text{eff}})[(1-\text{BS}^{*,j}_{\text{eff}})(1-\text{seq}^{j}_{\text{err}}) + \text{BS}^{*,j}_{\text{eff}}\text{seq}^{j}_{\text{err}}]\Big] .
\end{aligned}
\qquad (5)
$$

These propagated probabilities Equations (2), (3), (4), and (5) are derived in **Fig. 1b-c** and **Suppl. Fig. 1**.

Integrative analysis of BS-seq, TAB-seq, fCAB-seq, CAB-seq, redBS-seq, and MAB-seq data

Let BS_{eff}, $\text{BS}^{*}_{\text{eff}}$, lab_{eff}, ox_{eff}, pro_{eff}, EDC_{eff}, red_{eff}, $\text{M.SssI}_{\text{eff}}$ and seq_{err} denote bisulphite conversion efficiency, inaccurate bisulphite conversion efficiency, labeling efficiency, oxidation efficiency, Et-ONH_2 protection efficiency, EDC protection efficiency, NaBH_4 reduction efficiency, M.SssI efficiency and sequencing error, respectively. All the parameters have the same prior distributions and hyper parameters as described at the beginning of this document. Furthermore, let i and j denote cytosine and replicate, respectively. In the following subsections we state the emission probabilities, $p_{\text{BS}}(\text{“C”})$, $p_{\text{TAB}}(\text{“C”})$, $p_{\text{fCAB}}(\text{“C”})$, $p_{\text{CAB}}(\text{“C”})$, $p_{\text{redBS}}(\text{“C”})$ and $p_{\text{MAB}}(\text{“C”})$, in the terms of the aforementioned experimental parameters and methylation proportions,
$\theta = [p(\text{“C”}), p(\text{“5mC”}), p(\text{“5hmC”}), p(\text{“5fC”}), p(\text{“5caC”})]$.

BS-seq

$$
\begin{aligned}
p^{i,j}_{\text{BS}}(\text{“C”}) =& p^{i,j}(\text{“C”}) \underbrace{\left[(1-\text{BS}^{j}_{\text{eff}})(1-\text{seq}^{j}_{\text{err}})+\text{BS}^{j}_{\text{eff}}\text{seq}^{j}_{\text{err}}\right]}_{=p^{j}_{\text{BS}}(\text{“C”}|\text{C})} + \\
& p^{i,j}(\text{“5mC”}) \underbrace{\left[(1-\text{BS}^{*,j}_{\text{eff}})(1-\text{seq}^{j}_{\text{err}})+\text{BS}^{*,j}_{\text{eff}}\text{seq}^{j}_{\text{err}}\right]}_{=p^{j}_{\text{BS}}(\text{“C”}|\text{5mC})} + \\
& p^{i,j}(\text{“5hmC”}) \underbrace{\left[(1-\text{BS}^{*,j}_{\text{eff}})(1-\text{seq}^{j}_{\text{err}})+\text{BS}^{*,j}_{\text{eff}}\text{seq}^{j}_{\text{err}}\right]}_{=p^{j}_{\text{BS}}(\text{“C”}|\text{5hmC})} + \\
& p^{i,j}(\text{“5fC”}) \underbrace{\left[(1-\text{BS}^{j}_{\text{eff}})(1-\text{seq}^{j}_{\text{err}})+\text{BS}^{j}_{\text{eff}}\text{seq}^{j}_{\text{err}}\right]}_{=p^{j}_{\text{BS}}(\text{“C”}|\text{5fC})} + \\
& p^{i,j}(\text{“5caC”}) \underbrace{\left[(1-\text{BS}^{j}_{\text{eff}})(1-\text{seq}^{j}_{\text{err}})+\text{BS}^{j}_{\text{eff}}\text{seq}^{j}_{\text{err}}\right]}_{=p^{j}_{\text{BS}}(\text{“C”}|\text{5caC})} .
\end{aligned}
\tag{6}
$$

TAB-seq

$$
\begin{aligned}
p^{i,j}_{\text{TAB}}(\text{“C”}) =& p^{i,j}(\text{“C”}) \underbrace{\left[(1-\text{BS}^{j}_{\text{eff}})(1-\text{seq}^{j}_{\text{err}})+\text{BS}^{j}_{\text{eff}}\text{seq}^{j}_{\text{err}}\right]}_{=p^{j}_{\text{TAB}}(\text{“C”}|\text{C})} + \\
& p^{i,j}(\text{“5mC”}) \underbrace{\left[\begin{array}{l} \text{ox}^{j}_{\text{eff}}\left[(1-\text{BS}^{j}_{\text{eff}})(1-\text{seq}^{j}_{\text{err}})+\text{BS}^{j}_{\text{eff}}\text{seq}^{j}_{\text{err}}\right] \\ (1-\text{ox}^{j}_{\text{eff}})\left[(1-\text{BS}^{*,j}_{\text{eff}})(1-\text{seq}^{j}_{\text{err}})+\text{BS}^{*,j}_{\text{eff}}\text{seq}^{j}_{\text{err}}\right] \end{array}\right]}_{=p^{j}_{\text{TAB}}(\text{“C”}|\text{5mC})} + \\
& p^{i,j}(\text{“5hmC”}) \underbrace{\left[\begin{array}{l} \text{lab}^{j}_{\text{eff}}\left[(1-\text{BS}^{*,j}_{\text{eff}})(1-\text{seq}^{j}_{\text{err}})+\text{BS}^{*,j}_{\text{eff}}\text{seq}^{j}_{\text{err}}\right] + \\ (1-\text{lab}^{j}_{\text{eff}})\left[\text{ox}^{j}_{\text{eff}}\left[(1-\text{BS}^{j}_{\text{eff}})(1-\text{seq}^{j}_{\text{err}})+\text{BS}^{j}_{\text{eff}}\text{seq}^{j}_{\text{err}}\right]+(1-\text{ox}^{j}_{\text{eff}})\left[(1-\text{BS}^{*,j}_{\text{eff}})(1-\text{seq}^{j}_{\text{err}})+\text{BS}^{*,j}_{\text{eff}}\text{seq}^{j}_{\text{err}}\right]\right] \end{array}\right]}_{=p^{j}_{\text{TAB}}(\text{“C”}|\text{5hmC})} + \\
& p^{i,j}(\text{“5fC”}) \underbrace{\left[\begin{array}{l} \text{ox}^{j}_{\text{eff}}\left[(1-\text{BS}^{j}_{\text{eff}})(1-\text{seq}^{j}_{\text{err}})+\text{BS}^{j}_{\text{eff}}\text{seq}^{j}_{\text{err}}\right]+ \\ (1-\text{ox}^{j}_{\text{eff}})\left[(1-\text{BS}^{j}_{\text{eff}})(1-\text{seq}^{j}_{\text{err}})+\text{BS}^{j}_{\text{eff}}\text{seq}^{j}_{\text{err}}\right] \end{array}\right]}_{=p^{j}_{\text{TAB}}(\text{“C”}|\text{5fC})} + \\
& p^{i,j}(\text{“5caC”}) \underbrace{\left[\begin{array}{l} \text{ox}^{j}_{\text{eff}}\left[(1-\text{BS}^{j}_{\text{eff}})(1-\text{seq}^{j}_{\text{err}})+\text{BS}^{j}_{\text{eff}}\text{seq}^{j}_{\text{err}}\right]+ \\ (1-\text{ox}^{j}_{\text{eff}})\left[(1-\text{BS}^{j}_{\text{eff}})(1-\text{seq}^{j}_{\text{err}})+\text{BS}^{j}_{\text{eff}}\text{seq}^{j}_{\text{err}}\right] \end{array}\right]}_{=p^{j}_{\text{TAB}}(\text{“C”}|\text{5caC})} .
\end{aligned}
\tag{7}
$$

fCAB-seq

$$\begin{aligned}
p^{i,j}_{\text{fCAB}}(\text{“C”}) =& p^{i,j}(\text{“C”}) \underbrace{\left[(1-\text{BS}^{j}_{\text{eff}})(1-\text{seq}^{j}_{\text{err}}) + \text{BS}^{j}_{\text{eff}}\text{seq}^{j}_{\text{err}}\right]}_{=p^{j}_{\text{fCAB}}(\text{“C”}|\text{C})} + \\
& p^{i,j}(\text{“5mC”}) \underbrace{\left[(1-\text{BS}^{*,j}_{\text{eff}})(1-\text{seq}^{j}_{\text{err}}) + \text{BS}^{*,j}_{\text{eff}}\text{seq}^{j}_{\text{err}}\right]}_{=p^{j}_{\text{fCAB}}(\text{“C”}|\text{5mC})} + \\
& p^{i,j}(\text{“5hmC”}) \underbrace{\left[(1-\text{BS}^{*,j}_{\text{eff}})(1-\text{seq}^{j}_{\text{err}}) + \text{BS}^{*,j}_{\text{eff}}\text{seq}^{j}_{\text{err}}\right]}_{=p^{j}_{\text{fCAB}}(\text{“C”}|\text{5hmC})} + \\
& p^{i,j}(\text{“5fC”}) \underbrace{\begin{bmatrix}\text{pro}^{j}_{\text{eff}}\left[(1-\text{BS}^{*,j}_{\text{eff}})(1-\text{seq}^{j}_{\text{err}}) + \text{BS}^{*,j}_{\text{eff}}\text{seq}^{j}_{\text{err}}\right] + \\ (1-\text{pro}^{j}_{\text{eff}})\left[(1-\text{BS}^{j}_{\text{eff}})(1-\text{seq}^{j}_{\text{err}}) + \text{BS}^{j}_{\text{eff}}\text{seq}^{j}_{\text{err}}\right]\end{bmatrix}}_{=p^{j}_{\text{fCAB}}(\text{“C”}|\text{5fC})} + \\
& p^{i,j}(\text{“5caC”}) \underbrace{\left[(1-\text{BS}^{j}_{\text{eff}})(1-\text{seq}^{j}_{\text{err}}) + \text{BS}^{j}_{\text{eff}}\text{seq}^{j}_{\text{err}}\right]}_{=p^{j}_{\text{fCAB}}(\text{“C”}|\text{5caC})}.
\end{aligned} \tag{8}$$

CAB-seq

$$\begin{aligned}
p^{i,j}_{\text{CAB}}(\text{“C”}) =& p^{i,j}(\text{“C”}) \underbrace{\left[(1-\text{BS}^{j}_{\text{eff}})(1-\text{seq}^{j}_{\text{err}}) + \text{BS}^{j}_{\text{eff}}\text{seq}^{j}_{\text{err}}\right]}_{=p^{j}_{\text{CAB}}(\text{“C”}|\text{C})} + \\
& p^{i,j}(\text{“5mC”}) \underbrace{\left[(1-\text{BS}^{*,j}_{\text{eff}})(1-\text{seq}^{j}_{\text{err}}) + \text{BS}^{*,j}_{\text{eff}}\text{seq}^{j}_{\text{err}}\right]}_{=p^{j}_{\text{CAB}}(\text{“C”}|\text{5mC})} + \\
& p^{i,j}(\text{“5hmC”}) \underbrace{\left[(1-\text{BS}^{*,j}_{\text{eff}})(1-\text{seq}^{j}_{\text{err}}) + \text{BS}^{*,j}_{\text{eff}}\text{seq}^{j}_{\text{err}}\right]}_{=p^{j}_{\text{CAB}}(\text{“C”}|\text{5hmC})} + \\
& p^{i,j}(\text{“5fC”}) \underbrace{\left[(1-\text{BS}^{j}_{\text{eff}})(1-\text{seq}^{j}_{\text{err}}) + \text{BS}^{j}_{\text{eff}}\text{seq}^{j}_{\text{err}}\right]}_{=p^{j}_{\text{CAB}}(\text{“C”}|\text{5fC})} + \\
& p^{i,j}(\text{“5caC”}) \underbrace{\begin{bmatrix}\text{EDC}^{j}_{\text{eff}}\left[(1-\text{BS}^{*,j}_{\text{eff}})(1-\text{seq}^{j}_{\text{err}}) + \text{BS}^{*,j}_{\text{eff}}\text{seq}^{j}_{\text{err}}\right] + \\ (1-\text{EDC}^{j}_{\text{eff}})\left[(1-\text{BS}^{j}_{\text{eff}})(1-\text{seq}^{j}_{\text{err}}) + \text{BS}^{j}_{\text{eff}}\text{seq}^{j}_{\text{err}}\right]\end{bmatrix}}_{=p^{j}_{\text{CAB}}(\text{“C”}|\text{5fC})} +
\end{aligned} \tag{9}$$

redBS-seq

$$
\begin{aligned}
p^{i,j}_{\text{redBS}}(\text{“C”}) =& p^{i,j}(\text{“C”}) \underbrace{\left[(1-\text{BS}^{j}_{\text{eff}})(1-\text{seq}^{j}_{\text{err}}) + \text{BS}^{j}_{\text{eff}}\text{seq}^{j}_{\text{err}}\right]}_{=p^{j}_{\text{redBS}}(\text{“C”}|\text{C})} + \\
& p^{i,j}(\text{“5mC”}) \underbrace{\left[(1-\text{BS}^{*,j}_{\text{eff}})(1-\text{seq}^{j}_{\text{err}}) + \text{BS}^{*,j}_{\text{eff}}\text{seq}^{j}_{\text{err}}\right]}_{=p^{j}_{\text{redBS}}(\text{“C”}|\text{5mC})} + \\
& p^{i,j}(\text{“5hmC”}) \underbrace{\left[(1-\text{BS}^{*,j}_{\text{eff}})(1-\text{seq}^{j}_{\text{err}}) + \text{BS}^{*,j}_{\text{eff}}\text{seq}^{j}_{\text{err}}\right]}_{=p^{j}_{\text{redBS}}(\text{“C”}|\text{5hmC})} + \\
& p^{i,j}(\text{“5fC”}) \underbrace{\begin{bmatrix}\text{red}^{j}_{\text{eff}}\left[(1-\text{BS}^{*,j}_{\text{eff}})(1-\text{seq}^{j}_{\text{err}})+\text{BS}^{*,j}_{\text{eff}}\text{seq}^{j}_{\text{err}}\right]+ \\ (1-\text{red}^{j}_{\text{eff}})\left[(1-\text{BS}^{j}_{\text{eff}})(1-\text{seq}^{j}_{\text{err}})+\text{BS}^{j}_{\text{eff}}\text{seq}^{j}_{\text{err}}\right]\end{bmatrix}}_{=p^{j}_{\text{redBS}}(\text{“C”}|\text{5fC})} + \\
& p^{i,j}(\text{“5caC”}) \underbrace{\left[(1-\text{BS}^{j}_{\text{eff}})(1-\text{seq}^{j}_{\text{err}}) + \text{BS}^{j}_{\text{eff}}\text{seq}^{j}_{\text{err}}\right]}_{=p^{j}_{\text{redBS}}(\text{“C”}|\text{5caC})}.
\end{aligned}
\tag{10}
$$

MAB-seq

$$
\begin{aligned}
p^{i,j}_{\text{MAB}}(\text{“C”}) =& p^{i,j}(\text{“C”}) \underbrace{\begin{bmatrix}\text{M.SssI}^{j}_{\text{eff}}\left[(1-\text{BS}^{*,j}_{\text{eff}})(1-\text{seq}^{j}_{\text{err}})+\text{BS}^{*,j}_{\text{eff}}\text{seq}^{j}_{\text{err}}\right]+ \\ (1-\text{M.SssI}^{j}_{\text{eff}})\left[(1-\text{BS}^{j}_{\text{eff}})(1-\text{seq}^{j}_{\text{err}})+\text{BS}^{j}_{\text{eff}}\text{seq}^{j}_{\text{err}}\right]\end{bmatrix}}_{=p^{j}_{\text{MAB}}(\text{“C”}|\text{C})} + \\
& p^{i,j}(\text{“5mC”}) \underbrace{\left[(1-\text{BS}^{*,j}_{\text{eff}})(1-\text{seq}^{j}_{\text{err}}) + \text{BS}^{*,j}_{\text{eff}}\text{seq}^{j}_{\text{err}}\right]}_{=p^{j}_{\text{MAB}}(\text{“C”}|\text{5mC})} + \\
& p^{i,j}(\text{“5hmC”}) \underbrace{\left[(1-\text{BS}^{*,j}_{\text{eff}})(1-\text{seq}^{j}_{\text{err}}) + \text{BS}^{*,j}_{\text{eff}}\text{seq}^{j}_{\text{err}}\right]}_{=p^{j}_{\text{MAB}}(\text{“C”}|\text{5hmC})} + \\
& p^{i,j}(\text{“5fC” }) \underbrace{\left[(1-\text{BS}^{j}_{\text{eff}})(1-\text{seq}^{j}_{\text{err}}) + \text{BS}^{j}_{\text{eff}}\text{seq}^{j}_{\text{err}}\right]}_{=p^{j}_{\text{MAB}}(\text{“C”}|\text{5fC})} + \\
& p^{i,j}(\text{“5caC” }) \underbrace{\left[(1-\text{BS}^{j}_{\text{eff}})(1-\text{seq}^{j}_{\text{err}}) + \text{BS}^{j}_{\text{eff}}\text{seq}^{j}_{\text{err}}\right]}_{=p^{j}_{\text{MAB}}(\text{“C”}|\text{5caC})}.
\end{aligned}
\tag{11}
$$

For a particular combination of different BS-seq and “oxi-mC”-seq data sets, the likelihood will be computed as in Equation (1) using the emission probabilities defined in Equations (6-11).

On the difference of two independent Dirichlet random variables

Let X_1 and X_2 be independent Dirichlet random variables of order 3 with parameters α_1 and α_2, respectively. Consequently, the probability density functions of X_1 and X_2 are

$$f_{X_j}(\mathbf{x}_j;\alpha_j) = \frac{1}{\mathsf{B}(\alpha_j)} \prod_{i=1}^{3} x_{j,i}^{\alpha_{j,i}-1}, \tag{12}$$

where $\mathsf{B}(\alpha_j) = \left(\prod_{i=1}^{3} \Gamma(\alpha_{j,i})\right) / \left(\Gamma\left(\sum_{i=1}^{3} \alpha_{j,i}\right)\right)$ and $j \in \{1,\, 2\}$.

Alternatively, because the following statement $\sum_{i=1}^{3} x_{j,i} = 1$ holds Equation (12) can be equivalently stated as

$$f_{X_j}(\mathbf{x}_j;\alpha_j) = \frac{1}{\mathsf{B}(\alpha_j)} x_{j,1}^{\alpha_{j,1}-1} x_{j,2}^{\alpha_{j,2}-1} (1 - x_{j,1} - x_{j,2})^{\alpha_{j,3}-1}, \tag{13}$$

where $j \in \{1,\, 2\}$.

Now let us consider the difference of the random variables X_1 and X_2, i.e., the random variable $Y = X_1 - X_2$. The probability density function of Y is the convolution of the probability density functions of X_1 and X_2

$$f_Y(\mathbf{y}) = \int_S f_{X_1}(\mathbf{x};\alpha) f_{X_2}(\mathbf{x}-\mathbf{y};\alpha)\ \mathsf{d}\,\mathbf{x} \tag{14}$$

Let us follow the procedure described by Wolpert and Wolf to solve the integral. If we denote $h_i(x_i) = x_i^{\alpha_{1,i}-1}(x_i - y_i)^{\alpha_{2,i}-1}$, then Equation (14) can be stated as

$$\begin{aligned} f_Y(\mathbf{y}) &= \int_S f_{X_1}(\mathbf{x};\alpha) f_{X_2}(\mathbf{x}-\mathbf{y};\alpha)\ \mathsf{d}\,\mathbf{x} \\ &= \frac{1}{\mathsf{B}(\alpha_1)} \frac{1}{\mathsf{B}(\alpha_2)} \int_0^1 \int_0^{1-x_1} h_1(x_1) h_2(x_2) h_3(1 - x_1 - x_2)\ \mathsf{d}\,x_2\ \mathsf{d}\,x_1 \\ &= \frac{1}{\mathsf{B}(\alpha_1)} \frac{1}{\mathsf{B}(\alpha_2)} \int_0^1 h_1(x_1) \int_0^{1-x_1} h_2(x_2) h_3(1 - x_1 - x_2)\ \mathsf{d}\,x_2\ \mathsf{d}\,x_1. \end{aligned} \tag{15}$$

Now let us define τ_1, τ_2 and τ_3 recursively as $\tau_1 = \sum_{i=1}^{3} x_i = 1$ and $\tau_k = \tau_{k-1} - x_{k-1}$, then $\tau_k =$

$\tau_1 - \sum_{i=1}^{k-1} x_i$. Thus, the integral of Equation (15) can be equivalently stated as

$$
\begin{aligned}
f_Y(\mathbf{y}) &= \frac{1}{\mathsf{B}(\alpha_1)} \frac{1}{\mathsf{B}(\alpha_2)} \int_0^{\tau_1} h_1(x_1) \int_0^{\tau_2} h_2(x_2) h_3(\tau_2 - x_2) \,\mathsf{d}\, x_2 \,\mathsf{d}\, x_1 \\
&= \frac{1}{\mathsf{B}(\alpha_1)} \frac{1}{\mathsf{B}(\alpha_2)} \int_0^{\tau_1} h_1(x_1) \left(h_2 * h_3\right)(\tau_2) \,\mathsf{d}\, x_1 \\
&= \frac{1}{\mathsf{B}(\alpha_1)} \frac{1}{\mathsf{B}(\alpha_2)} \int_0^{\tau_1} h_1(x_1) \left(h_2 * h_3\right)(\tau_1 - x_1) \,\mathsf{d}\, x_1 \\
&= \frac{1}{\mathsf{B}(\alpha_1)} \frac{1}{\mathsf{B}(\alpha_2)} \left(h_1 * h_2 * h_3\right)(\tau_1) \\
&= \frac{1}{\mathsf{B}(\alpha_1)} \frac{1}{\mathsf{B}(\alpha_2)} \left(h_1(x_1) * h_2(x_2) * h_3(x_3)\right)(1).
\end{aligned}
\tag{16}
$$

We know that the Laplace transformation has the property $\mathcal{L}\left[h_1(x_1) * h_2(x_2) * h_3(x_3)\right] = \prod_{i=1}^{3} \mathcal{L}\left[h_i(x_i)\right]$. Since we are only interested on the probability density at the origin, we may assume $y_i = 0,\ i \in \{1,\ 2,\ 3\}$, then

$$
\mathcal{L}_{x_i}\left[h_i(x_i)\right] = \mathcal{L}_{x_i}\left[x_i^{\alpha_{1,i}-1} x_i^{\alpha_{2,i}-1}\right] = s^{-\alpha_{1,i}-\alpha_{2,i}+1}\Gamma\left(\alpha_{1,i} + \alpha_{2,i} - 1\right), \tag{17}
$$

where $i \in \{1,\ 2,\ 3\}$. Then by applying the inverse Laplace transformation to $\prod_{i=1}^{3} \mathcal{L}\left[h_i(x_i)\right]$ we get

$$
\begin{aligned}
\mathcal{L}^{-1}\left[\prod_{i=1}^{3} \mathcal{L}[h_i(x_i)]\right] &= \prod_{i=1}^{3} \Gamma(\alpha_{1,i} + \alpha_{2,i} - 1) \mathcal{L}^{-1}\left[s^{\sum_{i=1}^{3}(-\alpha_{1,i}-\alpha_{2,i}+1)}\right](1) \\
&= \frac{\prod_{i=1}^{3} \Gamma(\alpha_{1,i} + \alpha_{2,i} - 1)}{\Gamma\left(\sum_{i=1}^{3}(\alpha_{1,i} + \alpha_{2,i} - 1)\right)}.
\end{aligned}
\tag{18}
$$

Finally, we obtain the probability density at the origin of the difference of two Dirichlet random variables with the concentration parameters α_1 and α_2

$$
\begin{aligned}
f_Y(\mathbf{0}) &= \frac{1}{\mathsf{B}(\alpha_1)} \frac{1}{\mathsf{B}(\alpha_2)} \frac{\prod_{i=1}^{3} \Gamma(\alpha_{1,i} + \alpha_{2,i} - 1)}{\Gamma\left(\sum_{i=1}^{3}(\alpha_{1,i} + \alpha_{2,i} - 1)\right)} \\
&= \frac{\Gamma(\sum_{i=1}^{3} \alpha_{1,i})}{\prod_{i=1}^{3} \Gamma(\alpha_{1,i})} \frac{\Gamma(\sum_{i=1}^{3} \alpha_{2,i})}{\prod_{i=1}^{3} \Gamma(\alpha_{2,i})} \frac{\prod_{i=1}^{3} \Gamma(\alpha_{1,i} + \alpha_{2,i} - 1)}{\Gamma\left(\sum_{i=1}^{3}(\alpha_{1,i} + \alpha_{2,i} - 1)\right)}.
\end{aligned}
\tag{19}
$$

On the approximation of the beta distribution with the reparameterized versions

The Beta distribution has very intuitive properties when specifying the distribution of the parameter p of a Bernoulli trial Be(p) or a binomial experiment B(n, p). For example, to define the prior knowledge of

n trials with k successes, we use a prior of $p \sim \text{Beta}(k, n-k)$. However, when implementing a sampler for a hierarchical probability model, the Beta distribution has the disadvantage of being restricted to the $[0, 1]$ support and lacking a noncentered parametrization. As shown in Papaspiliopoulos et al. (2007), the noncentered parametrization enables significantly better sampling in hierarchical models. In the Stan sampler, variables with restricted support are automatically transformed into unrestricted equivalents in order to remove boundaries that would need to be treated separately (Stan Development Team (2014)). Currently, however, there is no automatic noncentering transformation. Therefore, to model the parameter p of a binomial experiment $\text{B}(n, p)$, we chose to use a logistic-normal distribution (Aitchison and Shen (1980)) that has a noncentered variant. To enjoy the best of both worlds, we initially elicit the required priors as Beta distributions and then choose the logistic-normal distribution that best approximates them.

The best approximation P of a distribution Q in the information theoretic sense is the one that minimizes the Kullback–Liebler distance, or discrepancy (Bernardo and Smith (2000) page 76), from Q to P.

$$\delta(P \mid Q) = \text{E}_Q\left[\log \frac{Q}{P}\right]. \tag{20}$$

Given the logistic map L and its inverse L^{-1}

$$\text{L}(x) = \frac{1}{1+e^{-x}} \qquad \text{and} \qquad \text{L}^{-1}(y) = \log\left(\frac{y}{1-y}\right), \tag{21}$$

the logistic-normal distribution with parameters μ and σ is defined as

$$\text{LN}(x\,; \mu, \sigma) = \frac{1}{x(1-x)}\frac{1}{\sqrt{2\pi\sigma^2}} \exp\left[-\frac{1}{2}\frac{\left(\text{L}^{-1}(x)-\mu\right)^2}{\sigma^2}\right]. \tag{22}$$

The Beta distribution with parameters α and β is defined as

$$\text{Beta}(x\,; \alpha, \beta) = \frac{x^{\alpha-1}(1-x)^{\beta-1}}{\text{B}(\alpha, \beta)}. \tag{23}$$

The discrepancy from LN$(x\,;\mu,\sigma)$ to Beta$(x\,;\alpha,\beta)$ is

$$\begin{aligned}
\delta\left(\mathsf{LN}(x)\,|\,\mathsf{Beta}(x)\right) &= \int_0^1 \log\left(\frac{\mathsf{Beta}(x\,;\alpha,\beta)}{\mathsf{LN}(x\,;\mu,\sigma)}\right)\mathsf{Beta}(x\,;\alpha,\beta)\,dx \\
&= \mathsf{E}\left[\log\left(\frac{\mathsf{Beta}(x\,;\alpha,\beta)}{\mathsf{LN}(x\,;\mu,\sigma)}\right)\right] \\
&= \mathsf{E}\log\left[\frac{x^{\alpha}(1-x)^{\beta}}{B(\alpha,\beta)}\sqrt{2\pi\sigma^2}\,\mathsf{exp}\left(\frac{1}{2}\frac{\left(\mathsf{L}^{-1}(x)-\mu\right)^2}{\sigma^2}\right)\right] \\
&= \mathsf{E}\left[\alpha\log x+\beta\log(1-x)-\log B(\alpha,\beta)+\log\sqrt{2\pi\sigma^2}+\frac{1}{2}\frac{\left(\mathsf{L}^{-1}(x)-\mu\right)^2}{\sigma^2}\right].
\end{aligned} \tag{24}$$

where all the expectations are taken with respect to the Beta$(x\,;\alpha,\beta)$ distribution. To find the values of the parameters μ and σ that minimize the discrepancy, we solve the roots of the derivatives:

$$\begin{aligned}
D_\mu\,\delta\left(\mathsf{LN}(x)\,|\,\mathsf{Beta}(x)\right) &= D_\mu\ \mathsf{E}\left[\frac{1}{2}\frac{\left(\mathsf{L}^{-1}(x)-\mu\right)^2}{\sigma^2}\right] \\
&= \frac{1}{2\sigma^2}\,\mathsf{E}\left[D_\mu\left(\mathsf{L}^{-1}(x)-\mu\right)^2\right] \\
&= \frac{1}{2\sigma^2}\,\mathsf{E}\left[-2(\mathsf{L}^{-1}(x)-\mu)\right] \\
&= -\frac{1}{\sigma^2}\,\mathsf{E}\left[\mathsf{L}^{-1}(x)\right]+\frac{1}{\sigma^2}\,\mathsf{E}[\mu] \\
&= -\frac{1}{\sigma^2}\left[\psi(\alpha)-\psi(\beta)\right]+\frac{1}{\sigma^2}\mu \\
&= 0,
\end{aligned} \tag{25}$$

where ψ is the digamma function. Equation (25) uses the identities in (28). The solution of the equation is $\mu=\psi(\alpha)-\psi(\beta)$.

$$
\begin{aligned}
D_{\sigma^2}\,\delta\left(\mathsf{LN}(x)\,|\,\mathsf{Beta}(x)\right) &= D_{\sigma^2}\ \mathsf{E}\left[\log\sqrt{2\pi\sigma^2}+\frac{1}{2}\frac{\left(\mathsf{L}^{-1}(x)-\mu\right)^2}{\sigma^2}\right] \\
&= \mathsf{E}\left[\frac{1}{2}D_{\sigma^2}\log 2\pi\sigma^2+\frac{1}{2}D_{\sigma^2}\frac{\left(\mathsf{L}^{-1}(x)-\mu\right)^2}{\sigma^2}\right] \\
&= \mathsf{E}\left[\frac{1}{2}\sigma^{-2}-\frac{1}{2}\sigma^{-4}\left(\mathsf{L}^{-1}(x)-\mu\right)^2\right] \\
&= \frac{1}{2}\sigma^{-2}-\frac{1}{2}\sigma^{-4}\,\mathsf{E}\left[\left(\mathsf{L}^{-1}(x)-\mu\right)^2\right] \\
&= \frac{1}{2}\sigma^{-2}-\frac{1}{2}\sigma^{-4}\,\mathsf{E}\left[(\log x-\log(1-x)-\mu)^2\right] \\
&= \frac{1}{2}\sigma^{-2}-\frac{1}{2}\sigma^{-4}\,\mathsf{E}\Big[\log^2 x+\log^2(1-x)+\mu^2 \\
&\quad -2\log x\log(1-x)-2\mu\log x+2\mu\log(1-x)\Big] \\
&= \frac{1}{2}\sigma^{-2}-\frac{1}{2}\sigma^{-4}\Big[(\psi(\alpha)-\psi(\alpha+\beta))^2+\psi_1(\alpha)-\psi_1(\alpha+\beta) \\
&\quad +(\psi(\beta)-\psi(\alpha+\beta))^2+\psi_1(\beta)-\psi_1(\alpha+\beta) \\
&\quad +\mu^2 \\
&\quad -2(\psi(\alpha)-\psi(\alpha+\beta))(\psi(\beta)-\psi(\alpha+\beta))+2\psi_1(\alpha+\beta) \\
&\quad -2\mu\psi(\alpha)+2\mu\psi(\alpha+\beta)+2\mu\psi(\beta)-2\mu\psi(\alpha+\beta)\Big] \\
&= \frac{1}{2}\sigma^{-2}-\frac{1}{2}\sigma^{-4}\Big[\psi_1(\alpha)+\psi_1(\beta) \\
&\quad +\psi^2(\alpha)+\psi^2(\alpha+\beta)-2\psi(\alpha)\psi(\alpha+\beta) \\
&\quad +\psi^2(\beta)+\psi^2(\alpha+\beta)-2\psi(\beta)\psi(\alpha+\beta) \\
&\quad +\psi^2(\alpha)+\psi^2(\beta)-2\psi(\alpha)\psi(\beta) \\
&\quad -2\psi(\alpha)\psi(\beta)+2\psi(\alpha)\psi(\alpha+\beta)+2\psi(\beta)\psi(\alpha+\beta)-2\psi^2(\alpha+\beta) \\
&\quad -2\psi^2(\alpha)+2\psi(\beta)\psi(\alpha)-2\psi^2(\beta)+2\psi(\alpha)\psi(\beta)\Big] \\
&= \frac{1}{2}\sigma^{-2}-\frac{1}{2}\sigma^{-4}\Big[\psi_1(\alpha)+\psi_1(\beta)\Big] \\
&= 0,
\end{aligned}
\tag{26}
$$

where ψ_1 is the trigamma function. Equation (26) uses the identities in (28). The solution of the equation is $\sigma^2=\psi_1(\alpha)+\psi_1(\beta)$.

Conclusion

We have shown that to approximate, in the K–L sense, a Beta distribution as defined in (23) with a logistic-normal distribution as defined in (22) one should choose the parameters as follows:

$$\begin{aligned} \mu &= \psi(\alpha) - \psi(\beta), \\ \sigma^2 &= \psi_1(\alpha) + \psi_1(\beta). \end{aligned} \tag{27}$$

Logarithmic moments of the Beta distribution

$$\begin{aligned} \mathsf{E}[\ln(X)] &= \psi(\alpha) - \psi(\alpha+\beta), \\ \mathsf{E}[\ln(1-X)] &= \psi(\beta) - \psi(\alpha+\beta), \\ \mathsf{E}\left[\ln^2(X)\right] &= (\psi(\alpha) - \psi(\alpha+\beta))^2 + \psi_1(\alpha) - \psi_1(\alpha+\beta), \\ \mathsf{E}\left[\ln^2(1-X)\right] &= (\psi(\beta) - \psi(\alpha+\beta))^2 + \psi_1(\beta) - \psi_1(\alpha+\beta), \\ \mathsf{E}\left[\ln(X)\ln(1-X)\right] &= (\psi(\alpha) - \psi(\alpha+\beta))(\psi(\beta) - \psi(\alpha+\beta)) - \psi_1(\alpha+\beta). \end{aligned} \tag{28}$$

Maximum likelihood estimators

In this section we will consider frequentist estimation for methylation levels from BS-seq/oxBS-seq and BS-seq/TAB-seq/fCAB-seq data. We show that the estimators considered in Booth (2012) and in Wang et al. (2014) for BS-seq/oxBS-seq and BS-seq/TAB-seq/fCAB-seq data, respectively, correspond to (unconstrained) maximum likelihood estimators.

BS-seq and oxBS-seq

Let us consider a single cytosine for the sake of notation simplicity. Assume the proportions of unmethylated C (C), methylated C (5mC), and hydroxymethylated C (5hmC) are governed by the probabilities p(“C”), p(“mC”), and p(“5hmC”). Morever, let $\theta = (p(\text{“C”}), p(\text{“5mC”}), p(\text{“5hmC”}))^{\mathsf{T}}$ denote a vector composed by the these unknown probabilities.

Using the conversion chart and θ, we can state the probabilities of the BS-seq and oxBS-seq

outcomes with as follows

$$\begin{aligned}p_{\text{BS}}(\text{"C"}|\theta) =& 1 - p(\text{"C"}) = p(\text{"5mC"}) + p(\text{"5hmC"})\\ p_{\text{BS}}(\text{"T"}|\theta) =& p(\text{"C"}) = 1 - p(\text{"5mC"}) + p(\text{"5hmC"})\\ p_{\text{oxBS}}(\text{"C"}|\theta) =& p(\text{"5mC"}) = 1 - p(\text{"C"}) + p(\text{"5hmC"})\\ p_{\text{oxBS}}(\text{"T"}|\theta) =& 1 - p(\text{"5mC"}) = p(\text{"C"}) + p(\text{"5hmC"}).\end{aligned} \tag{29}$$

Let us denote BS-seq and oxBS-seq data as follows

$$\mathcal{D} = \left\{N_{\text{BS,C}}, N_{\text{BS}}, N_{\text{oxBS,C}}, N_{\text{oxBS}}\right\}, \tag{30}$$

where $N_{\text{BS,C}}$ and $N_{\text{oxBS,C}}$ are the numbers of C reads out of $N_{\text{BS}} > 0$ (C reads and T reads from BS-seq) and $N_{\text{oxBS}} > 0$ (C reads and T reads from oxBS-seq) total reads, respectively.

Then the likelihood of θ, given $\mathcal{D}$ is

$$\begin{aligned}\mathcal{L}(\theta|\mathcal{D}) \propto\ & p(\text{"C"})^{N_{\text{BS}}-N_{\text{BS,C}}} (1 - p(\text{"C"}))^{N_{\text{BS,C}}}\\ & (1 - p(\text{"5mC"}))^{N_{\text{oxBS}}-N_{\text{oxBS,C}}}\, p(\text{"5mC"})^{N_{\text{oxBS,C}}},\end{aligned} \tag{31}$$

with the following constraints $p(\text{"C"}) + p(\text{"5mC"}) + p(\text{"5hmC"}) = 1$ (and $p(\text{"C"}),\ p(\text{"5mC"}),\ p(\text{"5hmC"}) \in [0,\ 1]$).

Next, let us calculate the gradient

$$\nabla\mathcal{L}(\theta|\mathcal{D}) = \begin{cases} \frac{\partial\mathcal{L}(\theta|\mathcal{D})}{\partial p(\text{"C"})} = & p(\text{"C"})^{N_{\text{BS}}-N_{\text{BS,C}}-1}\left(-(1-p(\text{"C"}))^{N_{\text{BS,C}}-1}\right)\\ & (1-p(\text{"5mC"}))^{N_{\text{oxBS}}-N_{\text{oxBS,C}}} p(\text{"5mC"})^{N_{\text{oxBS,C}}}\\ & \left((N_{\text{BS}} - N_{\text{BS,C}})(p(\text{"C"}) - 1) + N_{\text{BS,C}}\, p(\text{"C"})\right)\\ \frac{\partial\mathcal{L}(\theta|\mathcal{D})}{\partial p(\text{"5mC"})} = & p(\text{"C"})^{N_{\text{BS}}-N_{\text{BS,C}}}\left(-(1-p(\text{"C"}))^{N_{\text{BS,C}}}\right)\\ & (1-p(\text{"5mC"}))^{N_{\text{oxBS}}-N_{\text{oxBS,C}}-1} p(\text{"5mC"})^{N_{\text{oxBS,C}}-1}\\ & \left((N_{\text{oxBS}} - N_{\text{oxBS,C}})p(\text{"5mC"}) + N_{\text{oxBS,C}}(p(\text{"5mC"}) - 1)\right)\end{cases}. \tag{32}$$

Then we set $\nabla\mathcal{L}(\theta|\mathcal{D}) = \mathbf{0}$. We will not consider trivial solutions. The nontrivial solution is $p(\text{"C"}) = (N_{\text{BS}} - N_{\text{BS,C}})/N_{\text{BS}} \wedge p(\text{"5mC"}) = N_{\text{oxBS,C}}/N_{\text{oxBS}}$.

Note that we have assumed that $p(\text{"C"}) + p(\text{"5mC"}) + p(\text{"5hmC"}) = 1$. Then we can state the

(unconstrained) maximum likelihood estimator (MLE) of θ

$$\hat{\theta} = \begin{pmatrix} \hat{p}(\text{“C”}) \\ \hat{p}(\text{“5mC”}) \\ \hat{p}(\text{“5hmC”}) \end{pmatrix} = \begin{pmatrix} (N_{\text{BS}} - N_{\text{BS,C}})/N_{\text{BS}} \\ N_{\text{oxBS,C}}/N_{\text{oxBS}} \\ 1 - (N_{\text{BS}} - N_{\text{BS,C}})/N_{\text{BS}} - N_{\text{oxBS,C}}/N_{\text{oxBS}} \end{pmatrix} = \begin{pmatrix} N_{\text{BS,T}}/N_{\text{BS}} \\ N_{\text{oxBS,C}}/N_{\text{oxBS}} \\ 1 - N_{\text{BS,T}}/N_{\text{BS}} - N_{\text{oxBS,C}}/N_{\text{oxBS}} \end{pmatrix}. \tag{33}$$

BS-seq, TAB-seq and fCAB-seq

Similarly as in the previous section, let us assume the proportions of C, 5mC, 5hmC, and 5fC are governed by the probabilities $p(\text{“C”})$, $p(\text{“mC”})$, $p(\text{“5hmC”})$ and $p(\text{“5fC”})$. Morever, let $\theta = (p(\text{“C”}), p(\text{“5mC”}), p(\text{“5hmC”}, p(\text{“5fC”}))^{\text{T}}$ denote a vector composed by the these unknown probabilities.

Using the conversion chart and θ, we can state the probabilities of the BS-seq, TAB-seq, and fCAB-seq outcomes as follows

$$\begin{aligned} p_{\text{BS}}(\text{“C”}|\theta) =& 1 - p(\text{“C”}) - p(\text{“5fC”}) = p(\text{“5mC”}) + p(\text{“5hmC”}) \\ p_{\text{BS}}(\text{“T”}|\theta) =& p(\text{“C”}) + p(\text{“5fC”}) = 1 - p(\text{“5mC”}) - p(\text{“5hmC”}) \\ p_{\text{TAB}}(\text{“C”}|\theta) =& p(\text{“5hmC”}) = 1 - p(\text{“C”}) - p(\text{“5mC”}) - p(\text{“5fC”}) \\ p_{\text{TAB}}(\text{“T”}|\theta) =& 1 - p(\text{“5hmC”}) = p(\text{“C”}) + p(\text{“5mC”}) + p(\text{“5fC”}) \\ p_{\text{fCAB}}(\text{“C”}|\theta) =& p(\text{“5mC”}) + p(\text{“5hmC”}) + p(\text{“5fC”}) = 1 - p(\text{“C”}) \\ p_{\text{fCAB}}(\text{“T”}|\theta) =& 1 - p(\text{“5mC”}) - p(\text{“5hmC”}) - p(\text{“5fC”}) = p(\text{“C”}). \end{aligned} \tag{34}$$

Let us denote BS-seq, TAB-seq, and fCAB-seq data as follows

$$\mathcal{D} = \left\{N_{\text{BS,C}}, N_{\text{BS}}, N_{\text{TAB,C}}, N_{\text{TAB}}, N_{\text{fCAB,C}}, N_{\text{fCAB}}\right\}, \tag{35}$$

where $N_{\text{BS,C}}$, $N_{\text{TAB,C}}$ and $N_{\text{fCAB,C}}$ are the numbers of C reads out of $N_{\text{BS}} > 0$ (C reads and T reads from BS-seq), $N_{\text{TAB}} > 0$ (C reads and T reads from TAB-seq), and $N_{\text{fCAB}} > 0$ (C reads and T reads from fCAB-seq) total reads, respectively.

Then the likelihood of θ, given $\mathcal{D}$ is

$$\begin{aligned}\mathcal{L}(\theta|\mathcal{D}) = & (p(\text{“C”}) + p(\text{“5fC”}))^{N_{\text{BS}}-N_{\text{BS,C}}} (1 - p(\text{“C”}) - p(\text{“5fC”}))^{N_{\text{BS,C}}} \\ & (1 - p(\text{“5hmC”}))^{N_{\text{TAB}}-N_{\text{TAB,C}}} p(\text{“5hmC”})^{N_{\text{TAB,C}}} \\ & p(\text{“C”})^{N_{\text{fCAB}}-N_{\text{fCAB,C}}} (1 - p(\text{“C”}))^{N_{\text{fCAB,C}}} ,\end{aligned} \tag{36}$$

with the following constraints $p(\text{“C”}) + p(\text{“5mC”}) + p(\text{“5hmC”}) + p(\text{“5fC”}) = 1$ and $p(\text{“C”}),\ p(\text{“5mC”}),\ p(\text{“5hmC”}),\ p(\text{“5fC”}) \in [0,\ 1]$.

Next, let us calculate the gradient

$$\nabla\mathcal{L}(\theta|\mathcal{D}) = \begin{cases} \frac{\partial\mathcal{L}(\theta|\mathcal{D})}{\partial p(\text{“C”})} = & (1 - p(\text{“5hmC”}))^{N_{\text{TAB}}-N_{\text{TAB,C}}} p(\text{“5hmC”})^{N_{\text{TAB,C}}} \\ & p(\text{“C”})^{N_{\text{fCAB}}-N_{\text{fCAB,C}}-1} (1 - p(\text{“C”}))^{N_{\text{fCAB,C}}-1} \\ & (p(\text{“5fC”}) + p(\text{“C”}))^{N_{\text{BS}}-N_{\text{BS,C}}-1} (-p(\text{“5fC”}) - p(\text{“C”}) + 1)^{N_{\text{BS,C}}-1} \\ & \Big((N_{\text{BS}} - N_{\text{BS,C}})(p(\text{“C”}) - 1)p(\text{“C”})(p(\text{“5fC”}) + p(\text{“C”}) - 1) + \\ & (p(\text{“5fC”}) + p(\text{“C”}))\Big(N_{\text{BS,C}}(p(\text{“C”}) - 1)p(\text{“C”}) + \\ & (p(\text{“5fC”}) + p(\text{“C”}) - 1)\big((N_{\text{fCAB}} - N_{\text{fCAB,C}})(p(\text{“C”}) - 1) + N_{\text{fCAB,C}} p(\text{“C”})\big)\Big)\Big) \\ \frac{\partial\mathcal{L}(\theta|\mathcal{D})}{\partial p(\text{“5hmC”})} = & (1 - p(\text{“5hmC”}))^{N_{\text{TAB}}-N_{\text{TAB,C}}-1} p(\text{“5hmC”})^{N_{\text{TAB,C}}-1} \\ & p(\text{“C”})^{N_{\text{fCAB}}-N_{\text{fCAB,C}}} \big(-(1 - p(\text{“C”}))^{N_{\text{fCAB,C}}}\big) \\ & (p(\text{“5fC”}) + p(\text{“C”}))^{N_{\text{BS}}-N_{\text{BS,C}}} (-p(\text{“5fC”}) - p(\text{“C”}) + 1)^{N_{\text{BS,C}}} \\ & \big((N_{\text{TAB}} - N_{\text{TAB,C}}) p(\text{“5hmC”}) + N_{\text{TAB,C}}(p(\text{“5hmC”}) - 1)\big) \\ \frac{\partial\mathcal{L}(\theta|\mathcal{D})}{\partial p(\text{“5fC”})} = & (1 - p(\text{“5hmC”}))^{N_{\text{TAB}}-N_{\text{TAB,C}}} p(\text{“5hmC”})^{N_{\text{TAB,C}}} \\ & p(\text{“C”})^{N_{\text{fCAB}}-N_{\text{fCAB,C}}} \big(-(1 - p(\text{“C”}))^{N_{\text{fCAB,C}}}\big) \\ & (p(\text{“5fC”}) + p(\text{“C”}))^{N_{\text{BS}}-N_{\text{BS,C}}-1} (-p(\text{“5fC”}) - p(\text{“C”}) + 1)^{N_{\text{BS,C}}-1} \\ & \big((N_{\text{BS}} - N_{\text{BS,C}})(p(\text{“5fC”}) + p(\text{“C”}) - 1) + N_{\text{BS,C}}(p(\text{“5fC”}) + p(\text{“C”}))\big) \end{cases} . \tag{37}$$

Next, let us set $\nabla\mathcal{L}(\theta|\mathcal{D}) = \mathbf{0}$. Moreover, let us skip the trivial solutions.

Firstly, we observe that $\frac{\partial\mathcal{L}(\theta|\mathcal{D})}{\partial p(\text{“5hmC”})} = 0$ when $p(\text{“5hmC”}) = N_{\text{TAB,C}}/N_{\text{TAB}}$.

Secondly, we observe that $\frac{\partial\mathcal{L}(\theta|\mathcal{D})}{\partial p(\text{“5fC”})} = 0$ when $p(\text{“5fC”}) + p(\text{“5C”}) = (N_{\text{BS}} - N_{\text{BS,C}})/N_{\text{BS}}$. Then, using the identity $1 - p(\text{“C”}) - p(\text{“5fC”}) = p(\text{“5mC”}) + p(\text{“5hmC”})$ we get $p(\text{“5mC”}) = 1 - (N_{\text{BS}} - N_{\text{BS,C}})/N_{\text{BS}} - N_{\text{TAB,C}}/N_{\text{TAB}} = N_{\text{BS,C}}/N_{\text{BS}} - N_{\text{TAB,C}}/N_{\text{TAB}}$.

Then, let us check when the following term is zero

$$
\begin{aligned}
&(N_{\text{BS}} - N_{\text{BS,C}})(p(\text{“C”}) - 1)p(\text{“C”})(p(\text{“5fC”}) + p(\text{“C”}) - 1)+ \\
&(p(\text{“5fC”}) + p(\text{“C”}))\Big(N_{\text{BS,C}}(p(\text{“C”}) - 1)p(\text{“C”})+ \\
&(p(\text{“5fC”}) + p(\text{“C”}) - 1)\big((N_{\text{fCAB}} - N_{\text{fCAB,C}})(p(\text{“C”}) - 1) + N_{\text{fCAB,C}}p(\text{“C”})\big)\Big) = 0
\end{aligned} \tag{38}
$$

Next, let us make the substitution $p(\text{“C”}) + p(\text{“5fC”}) = (N_{\text{BS}} - N_{\text{BS,C}})/N_{\text{BS}} = N_{\text{BS,T}}/N_{\text{BS}}$

$$
\begin{aligned}
&(N_{\text{BS}} - N_{\text{BS,C}})(p(\text{“C”}) - 1)p(\text{“C”})(N_{\text{BS,T}}/N_{\text{BS}} - 1)+ \\
&N_{\text{BS,T}}/N_{\text{BS}}\Big(N_{\text{BS,C}}(p(\text{“C”}) - 1)p(\text{“C”})+ \\
&(N_{\text{BS,T}}/N_{\text{BS}} - 1)\underbrace{\big((N_{\text{fCAB}} - N_{\text{fCAB,C}})(p(\text{“C”}) - 1) + N_{\text{fCAB,C}}p(\text{“C”})\big)}_{=N_{\text{fCAB}}p(\text{“C”}) - N_{\text{fCAB,T}}}\Big) = 0
\end{aligned} \tag{39}
$$

Let us then rewrite some of the terms and simplify

$$
\begin{aligned}
&-(N_{\text{BS,T}}/N_{\text{BS}})N_{\text{BS,C}}(p(\text{“C”}) - 1)p(\text{“C”})+ \\
&(N_{\text{BS,T}}/N_{\text{BS}})N_{\text{BS,C}}(p(\text{“C”}) - 1)p(\text{“C”})- \\
&(N_{\text{BS,T}}/N_{\text{BS}})(N_{\text{BS,C}}/N_{\text{BS}})(N_{\text{fCAB}}p(\text{“C”}) - N_{\text{fCAB,T}}) = 0,
\end{aligned} \tag{40}
$$

which can be simplied to

$$
N_{\text{BS,T}}N_{\text{BS,C}}p(\text{“C”}) = N_{\text{BS,T}}N_{\text{BS,C}}(N_{\text{fCAB,T}}/N_{\text{fCAB}}). \tag{41}
$$

Then by assuming $N_{\text{BS,T}}N_{\text{BS,C}} \neq 0$ we get

$$
p(\text{“C”}) = N_{\text{fCAB,T}}/N_{\text{fCAB}}. \tag{42}
$$

By combining $p(\text{“C”}) = N_{\text{fCAB,T}}/N_{\text{fCAB}}$ and $p(\text{“5fC”}) + p(\text{“5C”}) = (N_{\text{BS}} - N_{\text{BS,C}})/N_{\text{BS}}$

$$
p(\text{“5fC”}) = (N_{\text{BS}} - N_{\text{BS,C}})/N_{\text{BS}} - N_{\text{fCAB,T}}/N_{\text{fCAB}}. \tag{43}
$$

Finally, we can state the (unconstrained) MLE of θ

$$\hat{\theta} = \begin{pmatrix} \hat{p}(\text{“C”}) \\ \hat{p}(\text{“5mC”}) \\ \hat{p}(\text{“5hmC”}) \\ \hat{p}(\text{“5fC”}) \end{pmatrix} = \begin{pmatrix} (N_{\text{fCAB}} - N_{\text{fCAB,C}})/N_{\text{fCAB}} \\ N_{\text{BS,C}}/N_{\text{BS}} - N_{\text{TAB,C}}/N_{\text{TAB}} \\ N_{\text{TAB,C}}/N_{\text{TAB}} \\ (N_{\text{BS}} - N_{\text{BS,C}})/N_{\text{BS}} - (N_{\text{fCAB}} - N_{\text{fCAB,C}})/N_{\text{fCAB}} \end{pmatrix} = \begin{pmatrix} N_{\text{fCAB,T}}/N_{\text{fCAB}} \\ N_{\text{BS,C}}/N_{\text{BS}} - N_{\text{TAB,C}}/N_{\text{TAB}} \\ N_{\text{TAB,C}}/N_{\text{TAB}} \\ N_{\text{BS,T}}/N_{\text{BS}} - N_{\text{fCAB,T}}/N_{\text{fCAB}} \end{pmatrix}. \tag{44}$$

References

J. Aitchison and S. M. Shen. Logistic-normal distributions: Some properties and uses. *Biometrika*, 67(2):261–272, 1980. ISSN 00063444. doi: 10.1093/biomet/67.2.261. URL `http://www.jstor.org/stable/2335470`.

J. M. Bernardo and A. F. M. Smith. *Bayesian Theory*. Wiley, 2000. ISBN 978-0-471-49464-5. doi: 10.1002/9780470316870.

M. Booth. Quantitative sequencing of 5-methylcytosine and 5-hydroxymethylcytosine at single-base resolution. *Science*, 336(6083):934–937, 2012. doi: 10.1126/science.1220671.

O. Papaspiliopoulos, G. O. Roberts, and M. Sköld. A general framework for the parametrization of hierarchical models. *Statistical Science*, 22(1):59–73, 02 2007. doi: 10.1214/088342307000000014. URL `http://dx.doi.org/10.1214/088342307000000014`.

Stan Development Team. Stan: A c++ library for probability and sampling, version 2.2, 2014. URL `http://mc-stan.org/`.

L. Wang, J. Zhang, J. Duan, X. Gao, W. Zhu, X. Lu, L. Yang, J. Zhang, G. Li, W. Ci, W. Li, Q. Zhou, N. Aluru, F. Tang, C. He, X. Huang, and J. Liu. Programming and inheritance of parental dna methylomes in mammals. *Cell*, 157(7):979–991, 2014. doi: 10.1016/j.cell.2014.05.029. URL `http://dx.doi.org/10.1016/j.cell.2014.05.029`.

D. Wolpert and D. Wolf. Estimating functions of probability distributions from a finite set of samples. *Physical Review E*, 52(6):6841–6854, Dec. 1995. ISSN 1063-651X. doi: 10.1103/physreve.52.6841. URL `http://dx.doi.org/10.1103/physreve.52.6841`.
