# Supplementary material for: A probabilistic generative model for quantification of DNA modifications enables analysis of demethylation pathways
Source: Genome Biol. 2016 Mar 14;17:49. doi: 10.1186/s13059-016-0911-6 (PMC4792102; doi:10.1186/s13059-016-0911-6)
Supplement: Additional file 4: — Figures S1 to S15 with legends. (PDF 8537 kb) [file 13059_2016_911_MOESM4_ESM.pdf]

Supplemental Figure 1. The effect of bisulphite conversion and oxidation efficiencies and sequencing error on BS-seq and oxBS-seq read-outs. (**a**) The conversion of 5mC or 5hmC to uracil upon bisulphite treatment is termed as inaccurate bisulphite conversion. (**b**) The bisulphite conversion of 5mC followed by sequencing stated in terms of BS^*_{eff} and seq_{err}. Oxidation does not have an effect on 5mC, hence the same model applies to 5hmC-seq measurement of 5mC. (**c**) The outcomes the BS-seq (*left*) and oxBS-seq (*right*) assays when applied to C (*top panel*), 5mC (*middle panel*) or 5hmC (*bottom panel*) are stated in terms of BS_{eff}, BS^*_{eff}, ox_{eff} and seq_{err}. (**d**) The outcomes of the BS-seq (on *left*) and oxBS-seq (on *right*) assays when applied to a population of cells with (unknown) cytosine modification proportions are stated in terms of BS_{eff}, BS^*_{eff}, ox_{eff}, seq_{err}, p(C), p(5mC) and p(5hmC).

Supplemental Figure 2. The graphical model of Lux represented with the plate notation. In the plate notation, the grey and white circles are used to represent observed variables and latent variables, respectively. The grey squares represent fixed parameters. (**a**) The model in which the values of the experimental parameters are estimated simultaneously with methylation levels. The variables g and μ together define a distribution for methylation modifications over biological replicates so that the replicate-specific methylation modifications θ_i are distributed according to that distribution. The replicate-specific bisulphite conversion and oxidation efficiencies together with the replicate-specific methylation patterns generate the observable data. The distributions of the variables and their conditional relationships are listed below the plate model. (**b**) The model in which the bisulphite conversion and oxidation efficiencies are given fixed values estimated beforehand. This model is computationally more attractive for large-scale estimation tasks than the model presented in (**a**). (**c**) A runtime analysis of the model presented in (**b**) with different number of samples. The depicted times are actual CPU times (user+system). The case of 12 BS-seq and 12 oxBS-seq reads was considered.

Supplemental Figure 3. An *in silico* study on estimating experimental parameters. (**a**) The model in the plate notation for generating data. Either a single or 20 control cytosines are generated for each of the cytosine modification. The experimental parameter values and the cytosine modification patterns are randomly generated from beta and Dirichlet distributions, respectively, using the indicated parameter values. (**b**) Observed data is generated for a single cytosine per cytosine modification. The estimation performance is studied as a function of the number of BS-seq and oxBS-seq read-outs. The boxplots are derived from 100 random simulations. The solid and dashed red lines depict the mean and standard deviations of the random experimental variables, respectively. (**c**) Same as (**b**) but here 20 control cytosines are generated per cytosine modification.

Supplemental Figure 4. The effect of *Tet2* knockdown on 5mC and 5hmC levels. The scatter plot shows the changes in 5mC (x-axis) and 5hmC (y-axis) levels upon *Tet2* knockdown. Only the cytosines in CpG context (N=384) are considered (see also **Fig. 2a**). The cytosines are represented by the points in the scatter plot. The Pearson's r and Spearman's ρ are shown (exact permutation test for testing the null hypothesis that there is no correlation, two tailed, $^*p < 2.2\times10^{-16}$).

Supplemental Figure 5. Estimating proportions of DNA methylation modifications. (**a**) The bar charts show estimated posterior means of 5hmC and 5mC proportions using three replicates per condition (on *top*). Only the cytosines in the CpG context are depicted. The designed amplicon is depicted. Read density profiles from affinity-based measurements of 5hmC and 5mC levels across the locus chrX:7,476,154-7,477,153 are shown (on *bottom*). (**b**)

Scatter plot representation of 5hmC levels estimated using CMS-IP and BS-/ oxBS-seq (on *top*) and 5mC using MeDIP and BS-/ oxBS-seq (on *bottom*) measurements. The conditions are set apart by the color. Random jitter is added to the scatter plot points to avoid overlapping points.

Supplemental Figure 6. Comparison of Lux and MLML in estimating methylation levels. (**a**) A pair-wise comparison between the Lux and MLML replicate-specific C (on *top*) and 5mC (on *bottom*) level estimates. Only the cytosines in CpG context are considered. The Pearson's correlation coefficients are shown. (**b**) The model in the plate notation for generating data. Altogether, 20 control cytosines are generated for each of the three cytosine modifications. Each control cytosine has 96 BS-seq and 96 oxBS-seq read-outs. The experimental parameter values and the cytosine modification patterns are randomly generated from beta and Dirichlet distributions, respectively, using the indicated parameter values. Observable data is generated for each of the defined methylation pattern θ while the number of BS-seq and oxBS-seq read-outs are varied from 6 to 192. (**c**) The estimates of the different methylation modifications are in the columns (p(C) on *left*; p(5mC) on *middle*; p(5hmC) on *right*). The Lux estimates (on *left* in each column) and MLML estimates (on *right* in each column) are shown. The red lines depict the true methylation levels. The boxplots are derived from 100 random simulations.

Supplemental Figure 7. An *in silico* study on analyzing methylation levels over biological experiments. (**a**) The model in the plate notation for generating data. Altogether, 20 control cytosines are generated for each of the three cytosine modifications. Observable data is generated for a given condition with predefined methylation pattern distribution (**α**s) with or without replicates. Each cytosine (control and non-control) has 96 BS-seq and 96 oxBS-seq read-outs. (**b**) The process of generating (**c**) and (**d**). Two different settings are considered: with more (on *left*) or less (on *right*) biological variation between replicates. First, the replicate-

specific methylation levels and experimental parameters are sampled from beta and Dirichlet distributions, respectively, and then these are used for generating observed data. Given the data, the posterior distribution of methylation proportions is estimated simultaneously together with the experimental parameters. The aforementioned procedure is repeated 100 times. (**c**) The results when the distribution with more biological variation (on *left* in (**b**)) is used to sample replicate-specific methylation levels. To allow a comparison between the ground truth and the estimates the following procedure was used. For each of the 100 simulations, we estimate the posterior mean of the parameter $g\mu+\mathbf{1}$ reflecting variability in θs. Using the estimated parameter we define the distribution $\mathrm{Dir}(g\mu+\mathbf{1})$. The ternary plots show the average of these distributions over 100 random simulations. (**d**) Same as (**c**) but here the replicate-specific methylation levels are sampled from the distribution with less biological variation (on *right* in (**b**)). (**e**) The difference between the estimated distributions and the true distributions is quantified using the Kullback-Leibler divergence, i.e., how much information is lost if we use our estimated distribution instead of the true distribution (an asymmetric measure) as a function of the number of replicates. The Kullback-Leibler divergence[2] is calculated between the true distribution with known parameters, $\mathrm{Dir}(\boldsymbol{\alpha})$, and estimated distribution with the posterior mean of $g\mu+\mathbf{1}$, $\mathrm{Dir}(g\mu+\mathbf{1})$. The boxplots are derived from 100 random simulations.

Supplemental Figure 8. Detecting differential methylation using real and *in silico* data. (**a**) The model in the plate notation for generating data. Altogether, 20 control cytosines are generated for each of the three cytosine modifications. Observable data is generated for each of the condition with predefined methylation pattern distribution ($\boldsymbol{\alpha}$s) with or without replicates. Each non-control cytosine has 12 BS-seq and 12 oxBS-seq read-outs, whereas each control cytosine has 96 BS-seq and 96 oxBS-seq read-outs. The experimental parameter values are randomly generated from beta distributions using the indicated parameter values. (**b**) The

differential methylation between conditions A and B is detected by quantifying evidence in the data for the alternative hypothesis H_1: $\Delta\mu=\mu_A-\mu_B\neq0$ over the null hypothesis H_0: $\Delta\mu=\mu_A-\mu_B=0$ (see *Methods*). The evidence is measured using the Bayes factor (BF), which is approximated using the Savage-Dickey density ratio $p(\Delta\mu=0|H_1)/p(\Delta\mu=0|H_1,D)$ (see *Methods*). The evidence for differential methylation ($\log_{10}$ BF) between a pair of samples (row/ column) is studied as a function of the number of replicates. Outliers are not depicted. (**c**) A comparison of Lux, FET, and MOABS in detecting differential methylation. For this purpose we down-sampled the full data set to 30X coverage for each of the three replicates. BS-seq and oxBS-seq data sets were analyzed separately with FET and MOABS for differential methylation. All the covered cytosines in CpG context (N=384) were divided into sets of differentially (N=252) and similarly (N=132) methylated cytosines based on independent CMS-IP and MeDIP loci-level information (see *Methods*). The ROC curves of the methods are calculated based on the differential methylation analysis results. The curves of different methods (Lux, FET, and MOABS) and data types (BS-seq/ oxBS-seq) are distinguished with different colors. The AUC values are listed in the figure legend. (**d**) A comparison of Lux and the method [1] (see *Methods*) for sequencing coverages 12X, 30X and "full data" using the sequencing data from WT and Tet2 KO mESCs. For comparison with other previous methods these graphs can also be compared directly with **Fig. 3b** and (**c**). (**e**) A comparison of Lux, FET, and MOABS in detecting differential methylation. For this purpose we simulated data from variably differentially methylated (N=100) and similarly methylated cytosines (N=100) with three replicates and variable bisulphite conversion and oxidation efficiencies and controls. For each noncontrol cytosine 12 BS-seq and 12 oxBS-seq read-outs are generated, whereas each control cytosine (20 per cytosine modification) has 96 BS-seq and 96 oxBS-seq read-outs. Then, the goal is to discriminate the differentially methylated cytosines from similarly methylated cytosines. Each cytosine is analyzed separately. In the FET and MOABS analyses the BS-seq and oxBS-seq data are analyzed separately and no control data is taken into account. (**f**) As in (**b**) but here the detection of differential

methylation is studied between methylated (p(5mC)=0.97 in v6.5) and unmethylated (p(C)=0.96 in Tetkd) cytosines at chr4:136,547,567 and chr8:120,115,720, respectively. Only the case of one replicate is considered. (**g**) As in (**f**) but here the analysis is done for the cytosine at chr4:139,783,857. The effect of the number of reads and replicates on Bayes factor is studied. The boxplots are derived from 100 randomly subsampled data sets.

Supplemental Figure 9. Differentially methylated CpGs and effect of modeling nonideal experimental parameters. (**a**) The Bayes factors of the top 100 (on *top*) or all the cytosines (on *bottom*) in descending order. The red line denotes the base line (BF=1). (**b**) The effect of assuming the ideal experimental parameters on the Bayes factors of the top ranked cytosines. The values of the ideal experimental parameters are listed. The one-sample *t*-test was used to test the null hypothesis that the population mean is equal to zero. The test statistic and two-tailed p-value are listed, indicating statistically significantly lower BFs when ideal parameter values are used.

Supplemental Figure 10. Examples of differentially methylated cytosines and 5mC and 5hmC maps obtained using antibody-based approaches. (**a**) Read density profiles of affinity-based measurements of 5hmC and 5mC levels across the locus chr4:139,783,236-139,784,235 are shown. The designed amplicon and the covered CpG sites are depicted. The cytosine marked with the red arrow is depicted in **Fig. 3a**. (**b**) As in (**a**) but here the focus in on the locus chr15:100,299,817-100,300,816 (on *left*). The posterior methylation patterns of a differentially methylated cytosine in the v6.5 and Tet2kd conditions are depicted (on *right*; marked with the red arrow on *left*). (**c**) As in (**b**) but here the focus in on the locus chr15:61,868,386-61,869,385.

Supplemental Figure 11. Locus-based analysis using Lux. (**a**) The graphical model of Lux for locus-based analysis represented with the plate notation. In the plate notation, the grey and white circles are used to represent observed variables and latent variables, respectively. The grey squares represent fixed parameters. Variation in methylation across a locus is modeled hierarchically by first defining a condition specific mean μ for methylation proportions in a locus, and μ is assigned a Dirichlet prior with hyperparameters α=(0.8, 0.8, 0.8). Methylation proportions v over individual cytosines within a locus are defined to follow Dir(gμ+**1**) distribution, where g represents biological variation around μ and was given a gamma prior with the shape parameter a=2 and rate parameter b=2/6. The vector **1** is added in order to prevent concentration of the probability mass in a few components. Finally, replicate specific methylation proportions θ are defined to follow Dir(fv +**1**) distribution, where f represents variation around v and was given a gamma prior with the shape parameter a=2 and rate parameter b=2/6. The rest of the model is presented in **Suppl. Fig. 2a**. (**b**) A comparison of Lux and MOABS in detecting differential methylation at loci level. In this comparison, we used the full v6.5/ Tet2kd data set. BS-seq and oxBS-seq data sets were analyzed separately with MOABS. All the analyzed windows were divided into sets of differentially and non-differentially methylated windows based on independent CMS-IP and MeDIP loci-level information (see *Methods*). The ROC curves of the methods are calculated based on the differential methylation analysis results. The curves of different methods (Lux and MOABS) and data types (BS-seq/ oxBS-seq) are distinguished with different colors. The AUC values are listed in the figure legend.

Supplemental Figure 12. Analysis of methylation dynamics during mouse T-cell development. (**a**). The posterior mean methylation proportions across the control loci in DP (*left panel*), CD4 SP (*middle panel*), and naïve CD4 (*right panel*) samples. The different replicates

are in the columns. The bars show the arithmetic means of the posterior means of the individual cytosines. The one-sided error bars (mean – standard deviation is depicted) show the standard deviations. (**b**) Dotblot assay to quantify 5hmC levels in the new spike-in 5hmC control used in the T-cell development study (*top panel*). Known amounts of 5hmC oligonucleotides were used[3] to generate the standard curve. The 5hmC levels in the spike-in control used in T-cell development study were determined according to the standard curve. 5hmC levels were quantified based on the dotblot assay results shown above (*bottom panel*). (**c**) Posterior distributions of oxidation efficiencies across biological conditions (DP in *top panel*; CD4 SP in *bottom panel*; naïve CD4 in *bottom panel*) and replicates. Kernel density estimates with the Gaussian kernel (the bandwidth obtained with Scott's rule) are shown. (**d**) A pair-wise comparison between the Lux and MLML replicate-specific 5hmC level estimates in DP, CD4 SP, and naïve CD4 cells. Only the cytosines in CpG context are considered. The Pearson's correlation coefficients are shown. (**e**) Differentially methylated cytosines in CpG context in *Prkcq*, *Zbtb7b*, and *Il6ra* are shown. The posterior means of methylation proportions of the individual cytosines are visualized in DP, CD4 SP, and naïve CD4 cell stages. The error bars (mean ± standard deviation is depicted) show the standard deviations of posterior distributions. The different methylation modifications are distinguished with different colors (p(C) in green, p(5mC) in yellow, and p(5hmC) in red).

Supplemental Figure 13. The effect of experimental parameters on BS-seq, TAB-seq and fCAB-seq read-outs. The experimental steps of BS-seq (on *left*), TAB-seq (on *middle*), and fCAB-seq (on *right*) on C (*first* row), 5mC (*second* row), 5hmC (*third* row), and 5fC (*fourth* row) are considered. For instance, in the bottom right corner the experimental steps of fCAB-seq on 5fC are stated in terms of BS_{eff}, BS^*_{eff}, pro_{eff}, and seq_{err}.

Supplemental Figure 14. Analysis of genome-wide BS-seq, TAB-seq, and fCAB-seq data. (**a**) Altogether, 20 control cytosines are generated for each of the four cytosine modifications. Each control cytosine has 96 BS-seq, 96 TAB-seq, and 96 fCAB-seq read-outs. The experimental parameter values and the cytosine modification patterns are randomly generated from beta and Dirichlet distributions (the means are reported), respectively. Observable data is generated for each of the defined methylation pattern θ while the number of BS-seq, TAB-seq, and fCAB-seq read-outs are varied from 6 to 192. The four different methylation patterns are on the rows. The estimates of the different methylation modifications are in the columns (p(C) on *first*; p(5mC) on *second*; p(5hmC) on *third*; p(5fC) on *fourth*). The Lux estimates (on *left* in each column) and frequency method estimates (*Suppl. Methods*; on *right* in each column) are shown. The red lines depict the true methylation levels. The boxplots are derived from 100 random simulations. (**b**) The marginal distributions of the posterior means of θ among the maternal (on *top*) and paternal (at *bottom*) cytosines. The panels, from left to right, correspond to the marginal distributions of the posteriors means of p(C), p(5mC), p(5hmC) and p(5fC). (**c**) The relationship of the standard deviations of the estimated posterior distributions (i.e., uncertainty in the estimated methylation modification level) and sequencing coverage studied using density plot representations. (**d**) The comparison of the Lux and frequency method of the 5mC ($5mC_{frequency}=N_{BS,C}/N_{BS}-N_{TAB,C}/N_{TAB}$; on *left*) and 5hmC ($5hmC_{frequency}=N_{TAB,C}/N_{TAB}$; on *right*) levels. (**e**) The comparison of the Lux and frequency method estimates of the 5mC (on *left*), 5hmC (on *middle*), and 5fC ($5fC_{frequency}=N_{BS,T}/N_{BS}-N_{fCAB,T}/N_{fCAB}$; on *right*) levels. As in (**d**) but here the cytosines with negative $5mC_{frequency}$ or $5fC_{frequency}$ are ignored.

Supplemental Figure 15. A sensitivity analysis of the model to the selection of the hyperhyperparameter values. (**a**) The model in the plate notation for generating data is described in **Suppl. Fig. 6b**. Altogether, 20 control cytosines are generated for each of the three

cytosine modifications. Each control cytosine has 96 BS-seq and 96 oxBS-seq read-outs. The experimental parameter values and the cytosine modification patterns are randomly generated from beta and Dirichlet distributions, respectively, using the indicated parameter values. Observable data is generated for each of the defined methylation pattern θ while the number of BS-seq and oxBS-seq read-outs are varied from 6 to 192. The effect of different choices of α on the estimates are studied. The estimates of the different methylation modifications are in the columns (p(C) on *left*; p(5mC) on *middle*; p(5hmC) on *right*). The red lines depict the true methylation levels. The boxplots are derived from 100 random simulations. (**b**) As in (**a**) but here the effects of the hyperhyperparameters related to the experimental parameters BS_{eff}, BS^*_{eff}, ox_{eff} and seq_{err} are studied. Briefly, we carried out an experiment where we let the values of the hyperhyperparameters to vary extensively and compared the results with those obtained with the original hyperhyperparameter values (listed in **Suppl. Table 1**). That is, for each of the eight hyperhyperparameter related to BS_{eff}, BS^*_{eff}, ox_{eff} and seq_{err} (the upper right corner of **Suppl. Fig. 2a**) values are sampled uniformly from the interval [0.5x, 1.5x] (where x is the original value listed in **Suppl. Table 1**). The difference between the estimates with different number of reads is studied. (**c**) The detection of differential methylation between two conditions with different values of α is studied as a function of the number of replicates. The data are generated as described in **Suppl. Fig. 8**.

REFERENCES

1. Booth MJ, Branco MR, Ficz G, Oxley D, Krueger F, Reik W, Balasubramanian S: **Quantitative sequencing of 5-methylcytosine and 5-hydroxymethylcytosine at single-base resolution.** *Science* 2012, **336**(6083)**:**934-937.

2. Penny WD: **Kullback-liebler divergences of normal, gamma, dirichlet and wishart densities.** *Wellcome Department of Cognitive Neurology* 2001, **:**.

3. Huang Y, Pastor WA, Shen Y, Tahiliani M, Liu DR, Rao A: **The behaviour of 5-hydroxymethylcytosine in bisulfite sequencing.** *PLoS One* 2010, **5**(1)**:**e8888.

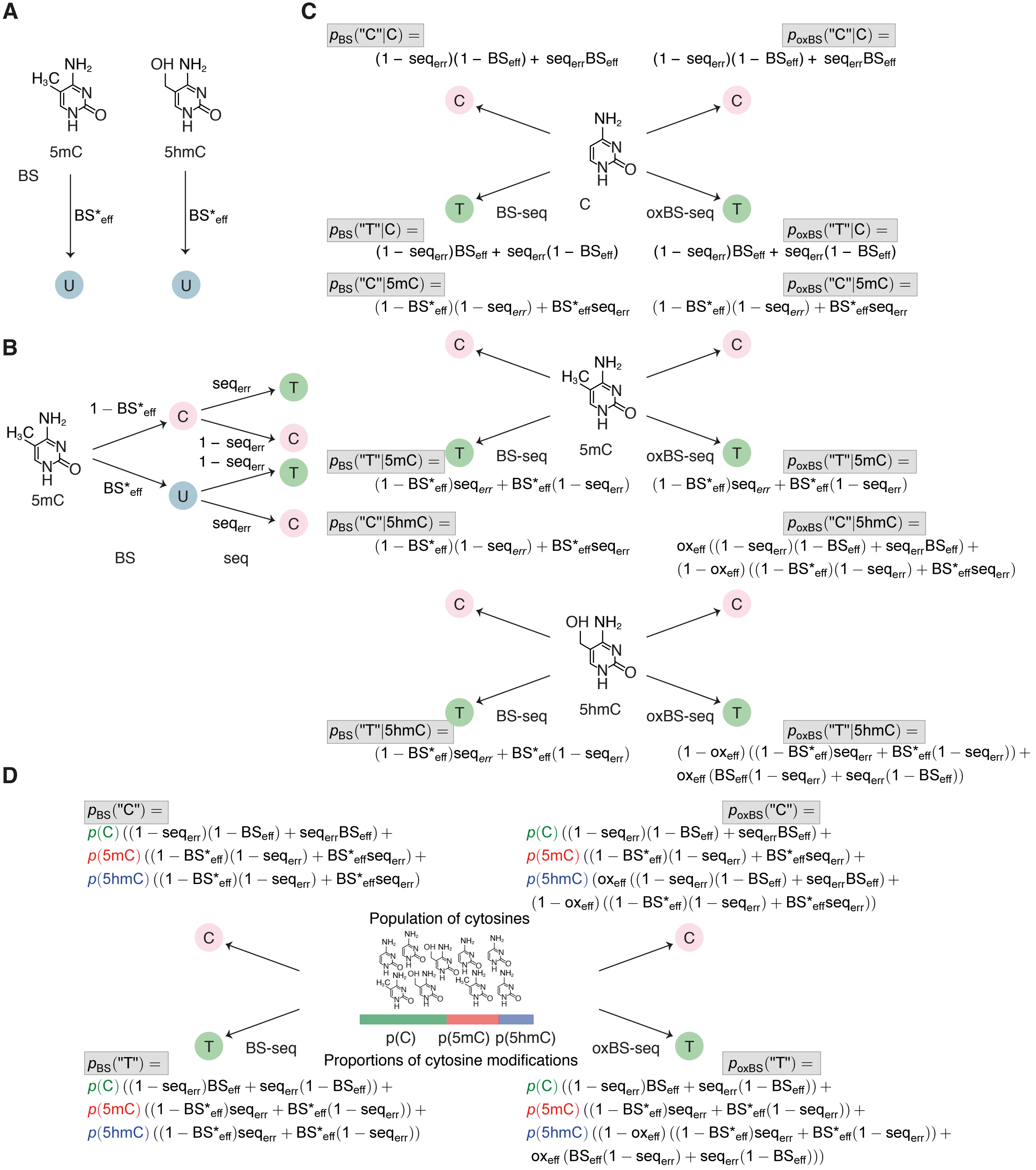

Supplemental Figure 1

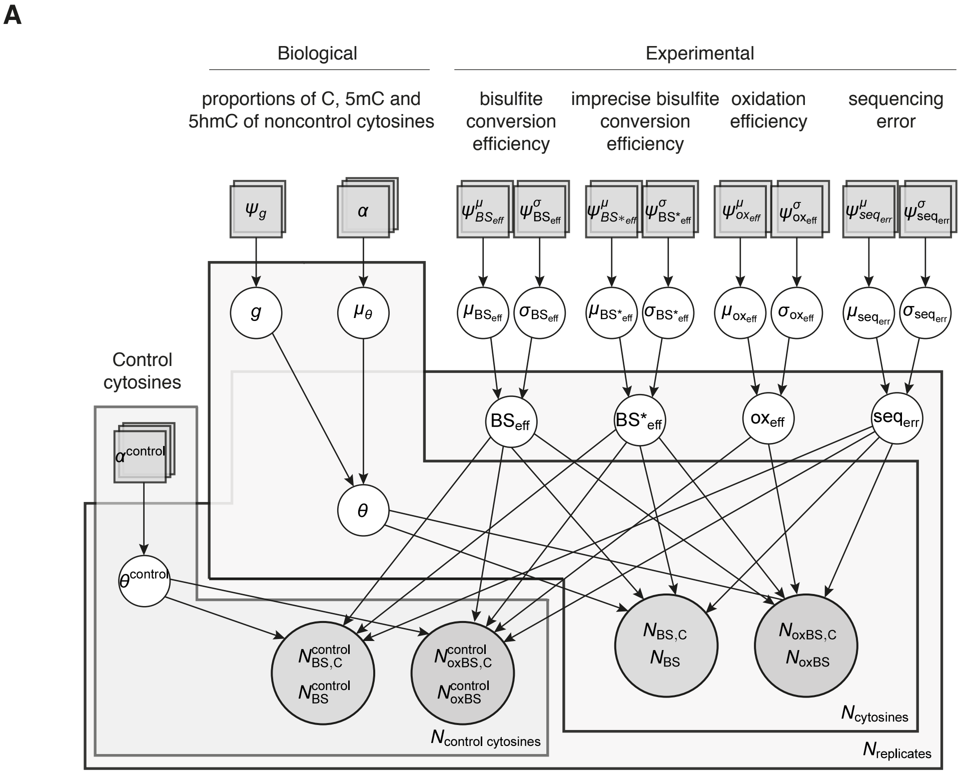

$$\theta^{\text{control}} \sim \text{Dir}(\alpha^{\text{control}})$$
$$N^{\text{control}}_{\text{BS,C}} \sim \text{Binom}(N^{\text{control}}_{\text{BS}}, p^{\text{control}}_{\text{BS}}(``\text{C}"))$$
$$N^{\text{control}}_{\text{oxBS,C}} \sim \text{Binom}(N^{\text{control}}_{\text{oxBS}}, p^{\text{control}}_{\text{oxBS}}(``\text{C}"))$$

$$g \sim \text{Gamma}(\psi_g^1, \psi_g^2)$$
$$\mu_\theta \sim \text{Dir}(\alpha)$$
$$\theta \sim \text{Dir}(g\mu_\theta + 1)$$
$$N_{\text{BS,C}} \sim \text{Binom}(N_{\text{BS}}, p_{\text{BS}}(``\text{C}"))$$
$$N_{\text{oxBS,C}} \sim \text{Binom}(N_{\text{oxBS}}, p_{\text{oxBS}}(``\text{C}"))$$
$$\text{BS}_{\text{eff}} = \text{logit}^{-1}(\mu_{\text{BS}_{\text{eff}}} + \sigma_{\text{BS}_{\text{eff}}} r_{\text{BS}_{\text{eff}}})$$
$$\text{BS*}_{\text{eff}} = \text{logit}^{-1}(\mu_{\text{BS*}_{\text{eff}}} + \sigma_{\text{BS*}_{\text{eff}}} r_{\text{BS*}_{\text{eff}}})$$
$$\text{ox}_{\text{eff}} = \text{logit}^{-1}(\mu_{\text{ox}_{\text{eff}}} + \sigma_{\text{ox}_{\text{eff}}} r_{\text{ox}_{\text{eff}}})$$
$$\text{seq}_{\text{err}} = \text{logit}^{-1}(\mu_{\text{seq}_{\text{err}}} + \sigma_{\text{seq}_{\text{err}}} r_{\text{seq}_{\text{err}}})$$

$$\mu_{\text{BS}_{\text{eff}}} \sim \mathcal{N}(\psi^{\mu,\mu}_{\text{BS}_{\text{eff}}}, \psi^{\mu,\sigma}_{\text{BS}_{\text{eff}}})$$
$$\sigma_{\text{BS}_{\text{eff}}} \sim \ln\mathcal{N}(\psi^{\sigma,\mu}_{\text{BS}_{\text{eff}}}, \psi^{\sigma,\sigma}_{\text{BS}_{\text{eff}}})$$
$$r_{\text{BS}_{\text{eff}}} \sim \mathcal{N}(0,1)$$
$$\mu_{\text{ox}_{\text{eff}}} \sim \mathcal{N}(\psi^{\mu,\mu}_{\text{ox}_{\text{eff}}}, \psi^{\mu,\sigma}_{\text{ox}_{\text{eff}}})$$
$$\sigma_{\text{ox}_{\text{eff}}} \sim \ln\mathcal{N}(\psi^{\sigma,\mu}_{\text{ox}_{\text{eff}}}, \psi^{\sigma,\sigma}_{\text{ox}_{\text{eff}}})$$
$$r_{\text{ox}_{\text{eff}}} \sim \mathcal{N}(0,1)$$
$$\mu_{\text{BS*}_{\text{eff}}} \sim \mathcal{N}(\psi^{\mu,\mu}_{\text{BS*}_{\text{eff}}}, \psi^{\mu,\sigma}_{\text{BS*}_{\text{eff}}})$$
$$\sigma_{\text{BS*}_{\text{eff}}} \sim \ln\mathcal{N}(\psi^{\sigma,\mu}_{\text{BS*}_{\text{eff}}}, \psi^{\sigma,\sigma}_{\text{BS*}_{\text{eff}}})$$
$$r_{\text{BS*}_{\text{eff}}} \sim \mathcal{N}(0,1)$$
$$\mu_{\text{seq}_{\text{err}}} \sim \mathcal{N}(\psi^{\mu,\mu}_{\text{seq}_{\text{err}}}, \psi^{\mu,\sigma}_{\text{seq}_{\text{err}}})$$
$$\sigma_{\text{seq}_{\text{err}}} \sim \ln\mathcal{N}(\psi^{\sigma,\mu}_{\text{seq}_{\text{err}}}, \psi^{\sigma,\sigma}_{\text{seq}_{\text{err}}})$$
$$r_{\text{seq}_{\text{err}}} \sim \mathcal{N}(0,1)$$

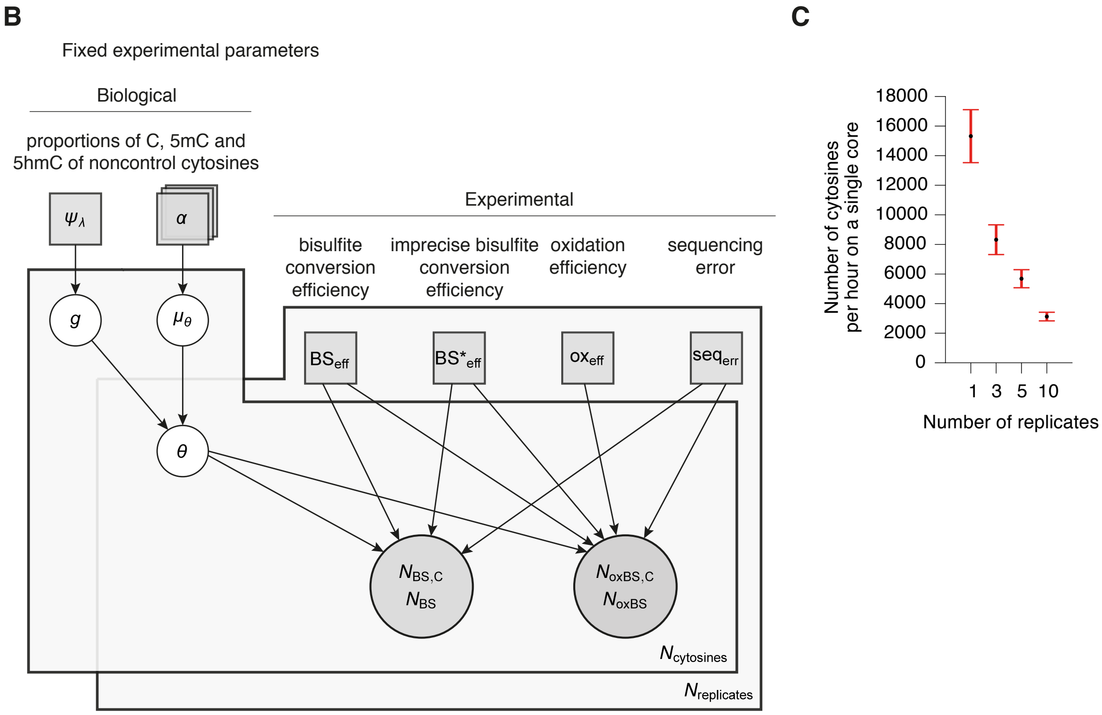

Supplemental Figure 2

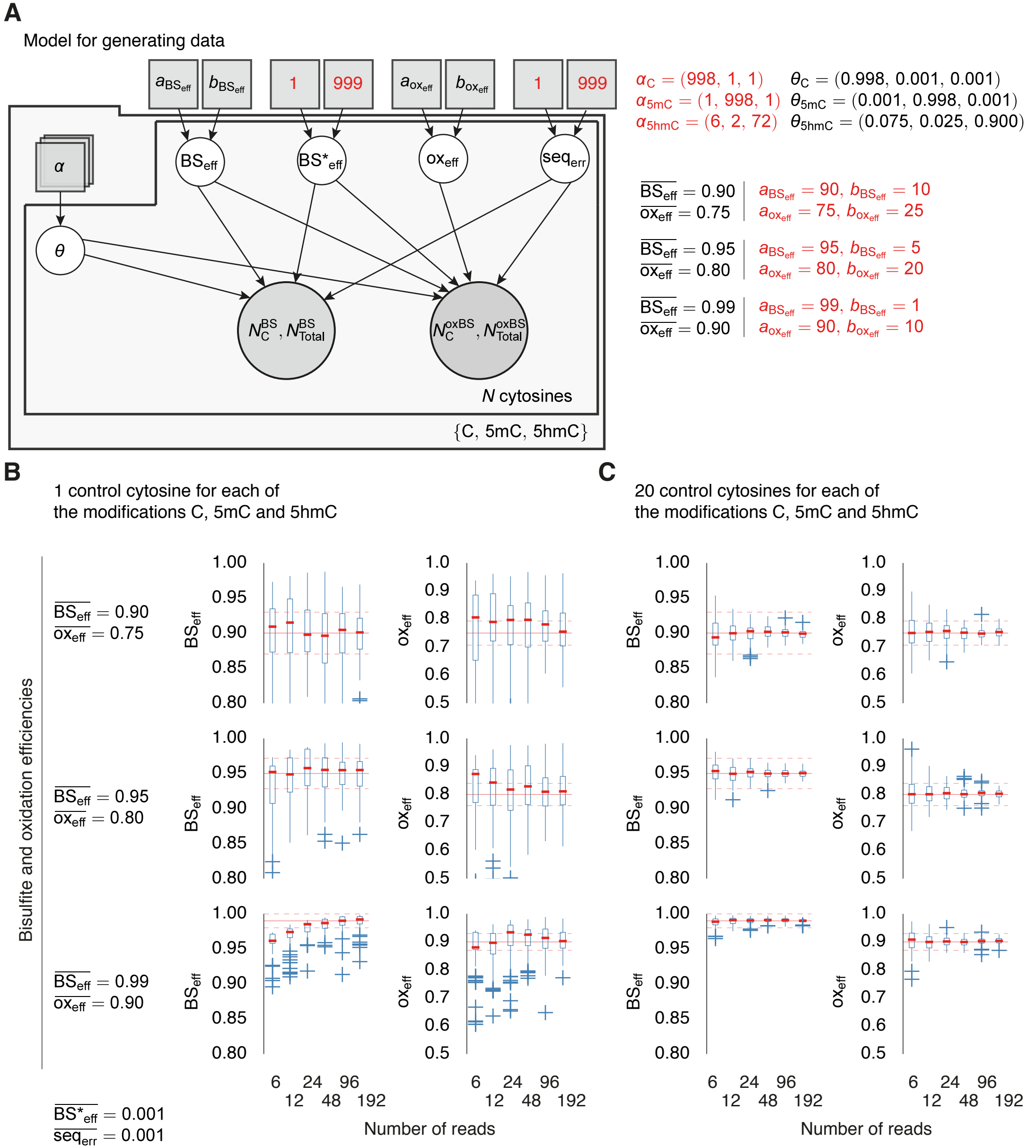

Supplemental Figure 3

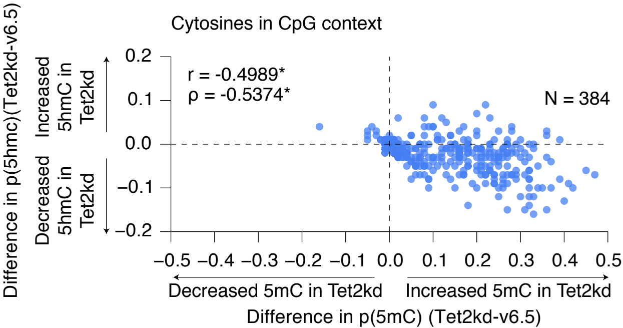

Supplemental Figure 4

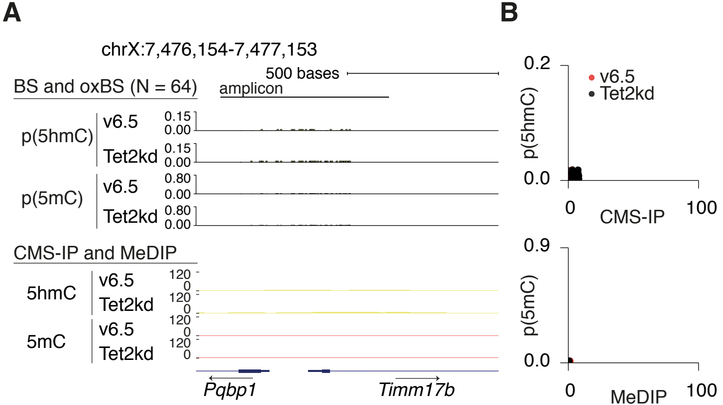

Supplemental Figure 5

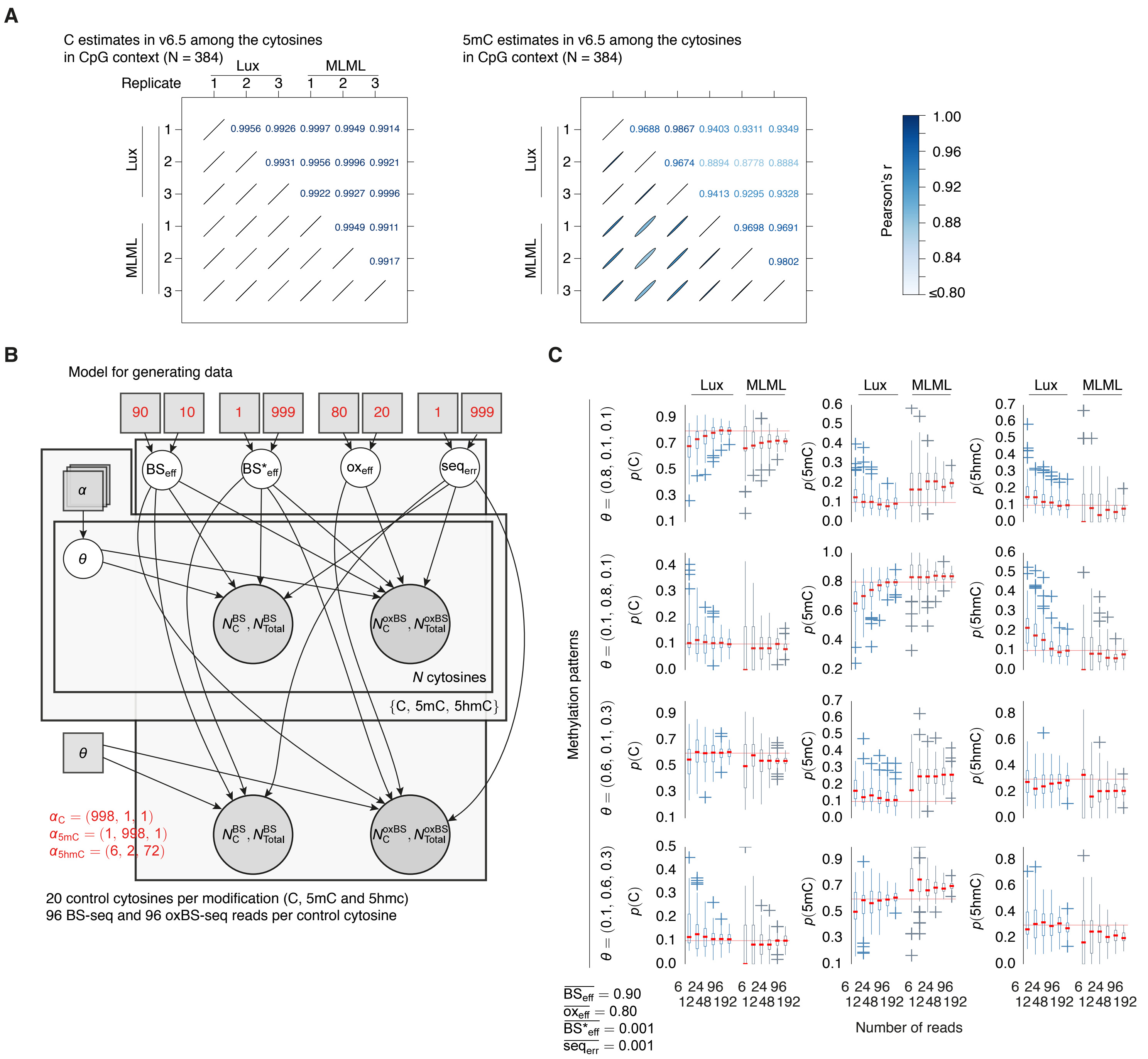

Supplemental Figure 6

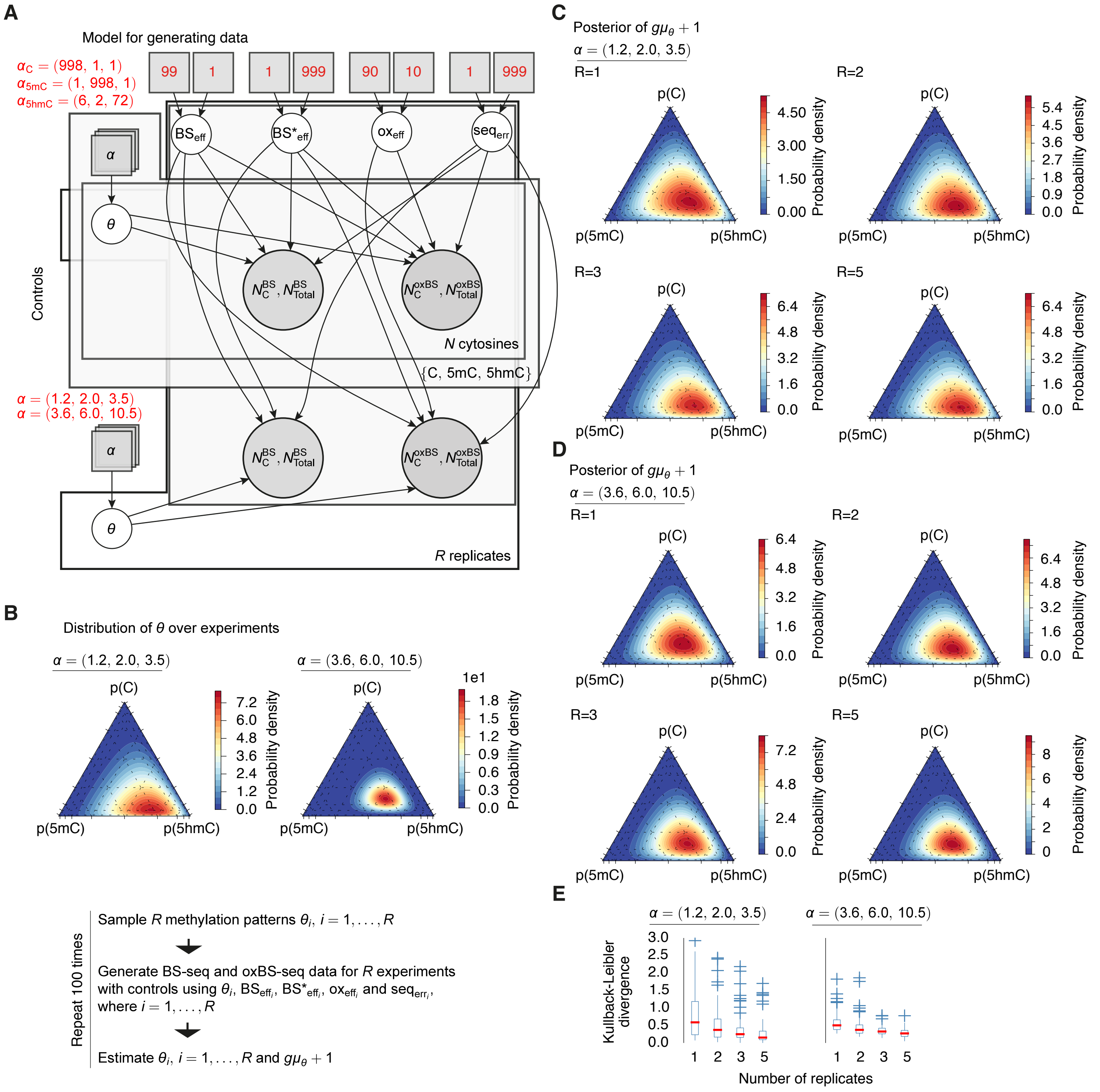

Supplemental Figure 7

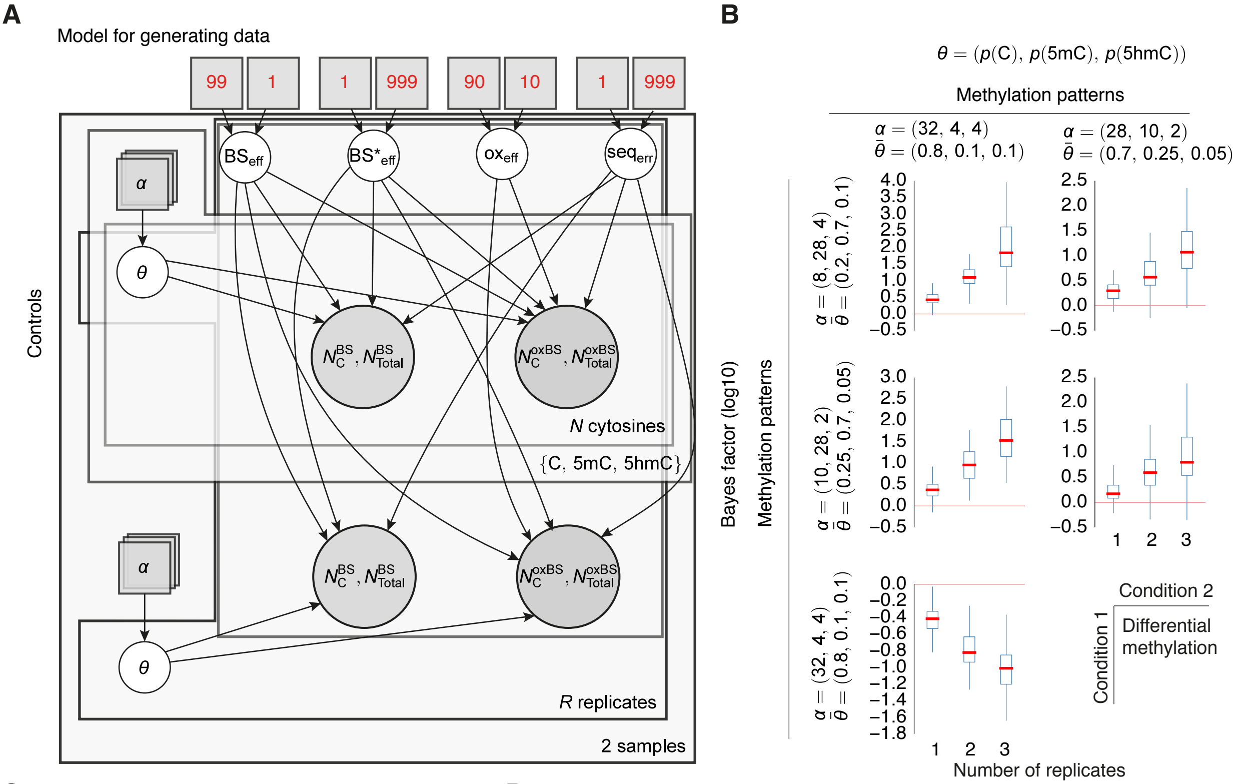

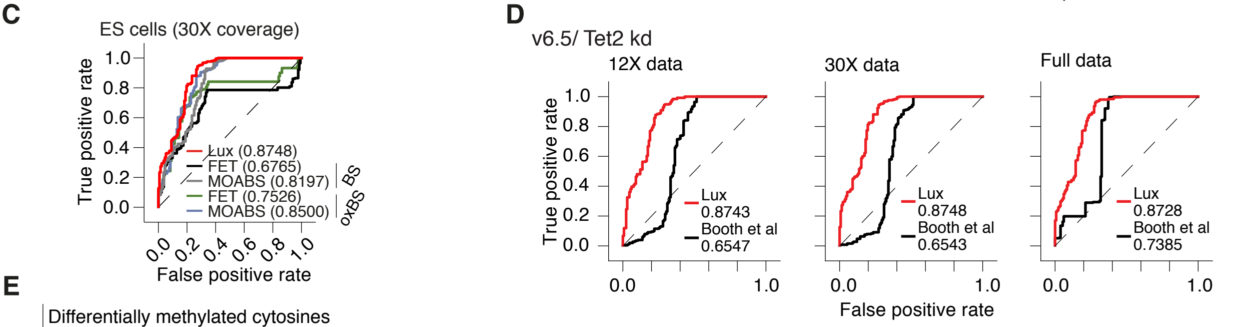

E

Repeat 100 times

Differentially methylated cytosines

Sample $\mu_1, \mu_2 \sim \mathrm{Dir}(0.5, 0.5, 0.5)$ so that $\|\mu_1 - \mu_2\|_1 \geq 0.4$

Sample $\theta_1^j \sim \mathrm{Dir}(10\mu_1)$ and $\theta_2^j \sim \mathrm{Dir}(10\mu_2)$, where $j \in \{1, 2, 3\}$

Sample $BS_{eff}^{i,j} \sim \mathrm{Beta}(19.8, 0.2)$, $ox_{eff}^{i,j} \sim \mathrm{Beta}(12, 3)$, $BS_{eff}^{*,i,j} \sim \mathrm{Beta}(1, 999)$, and $seq_{err}^{i,j} \sim \mathrm{Beta}(1, 999)$, where $i \in \{1, 2\}$ and $j \in \{1, 2, 3\}$

Sample $N_{BS,C}^{i,j}$ and $N_{oxBS,C}^{i,j}$ given $N_{BS}^{i,j} = 12$ and $N_{oxBS}^{i,j} = 12$ and sampled θ_i^j, $BS_{eff}^{i,j}$, $ox_{eff}^{i,j}$, $BS_{eff}^{*,i,j}$, and $seq_{err}^{i,j}$, where $i \in \{1, 2\}$ and $j \in \{1, 2, 3\}$

Repeat 100 times

Nondifferentially methylated cytosines

Sample $\mu \sim \mathrm{Dir}(0.5, 0.5, 0.5)$

Sample $\theta_1^j \sim \mathrm{Dir}(10\mu)$ and $\theta_2^j \sim \mathrm{Dir}(10\mu)$, where $j \in \{1, 2, 3\}$

Sample $BS_{eff}^{i,j} \sim \mathrm{Beta}(19.8, 0.2)$, $ox_{eff}^{i,j} \sim \mathrm{Beta}(12, 3)$, $BS_{eff}^{*,i,j} \sim \mathrm{Beta}(1, 999)$, and $seq_{err}^{i,j} \sim \mathrm{Beta}(1, 999)$, where $i \in \{1, 2\}$ and $j \in \{1, 2, 3\}$

Sample $N_{BS,C}^{i,j}$ and $N_{oxBS,C}^{i,j}$ given $N_{BS}^{i,j} = 12$ and $N_{oxBS}^{i,j} = 12$ and sampled θ_i^j, $BS_{eff}^{i,j}$, $ox_{eff}^{i,j}$, $BS_{eff}^{*,i,j}$, and $seq_{err}^{i,j}$, where $i \in \{1, 2\}$ and $j \in \{1, 2, 3\}$

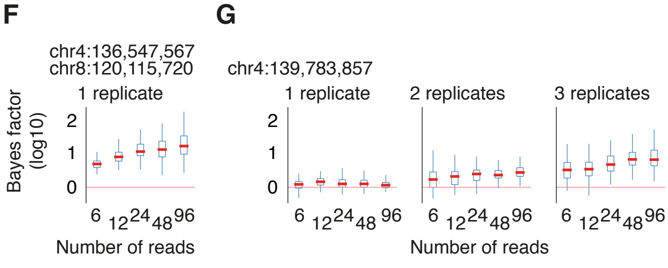

Supplemental Figure 8

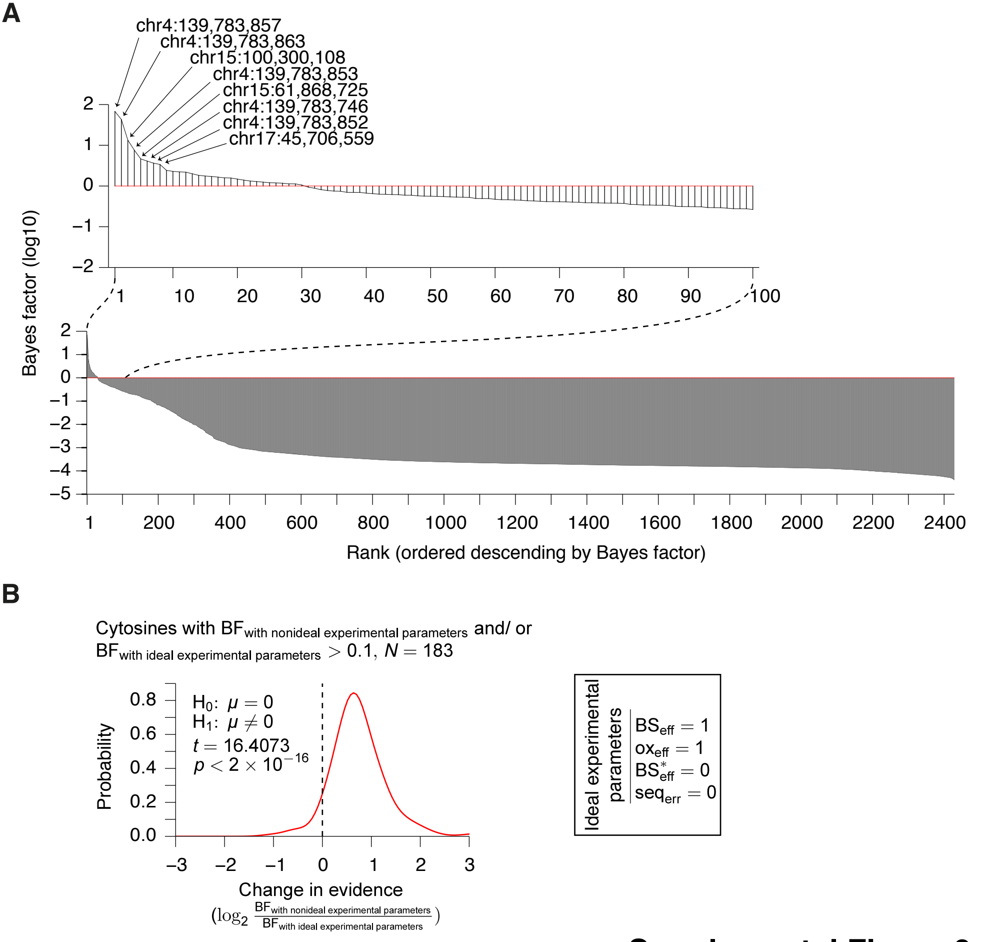

Supplemental Figure 9

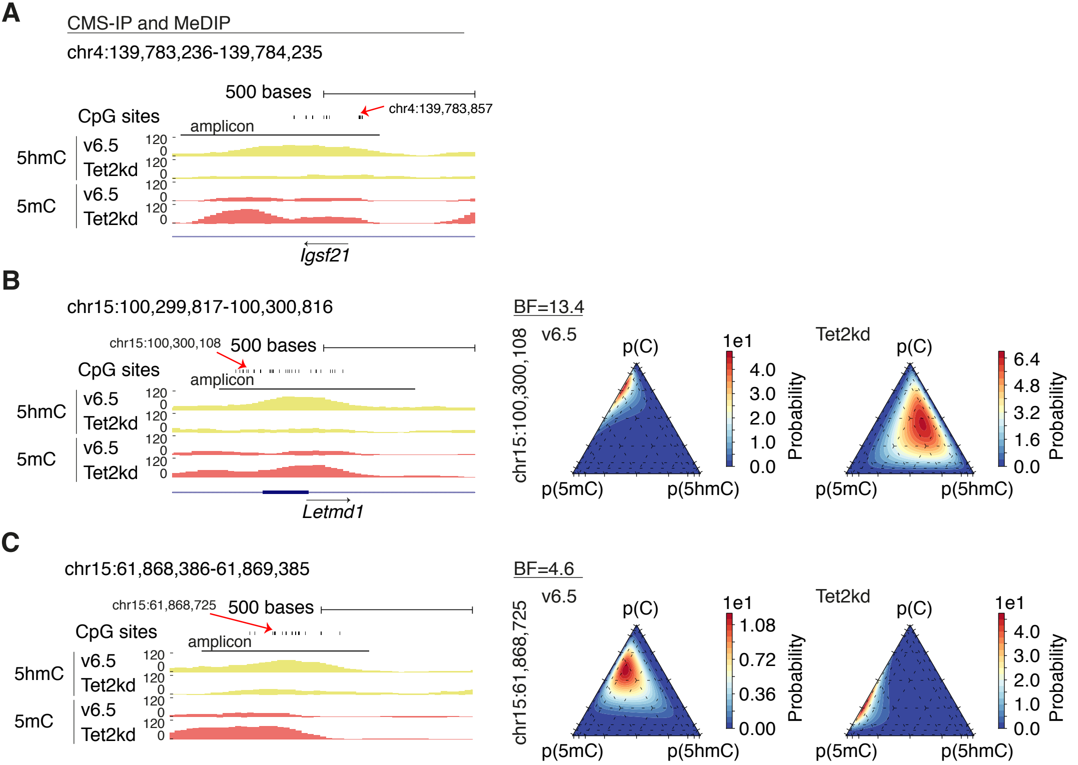

Supplemental Figure 10

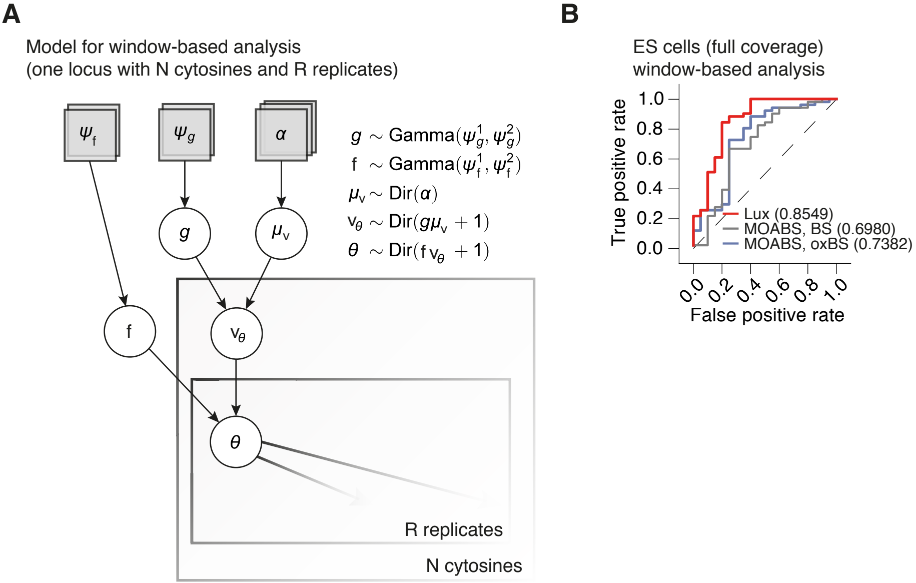

Supplemental Figure 11

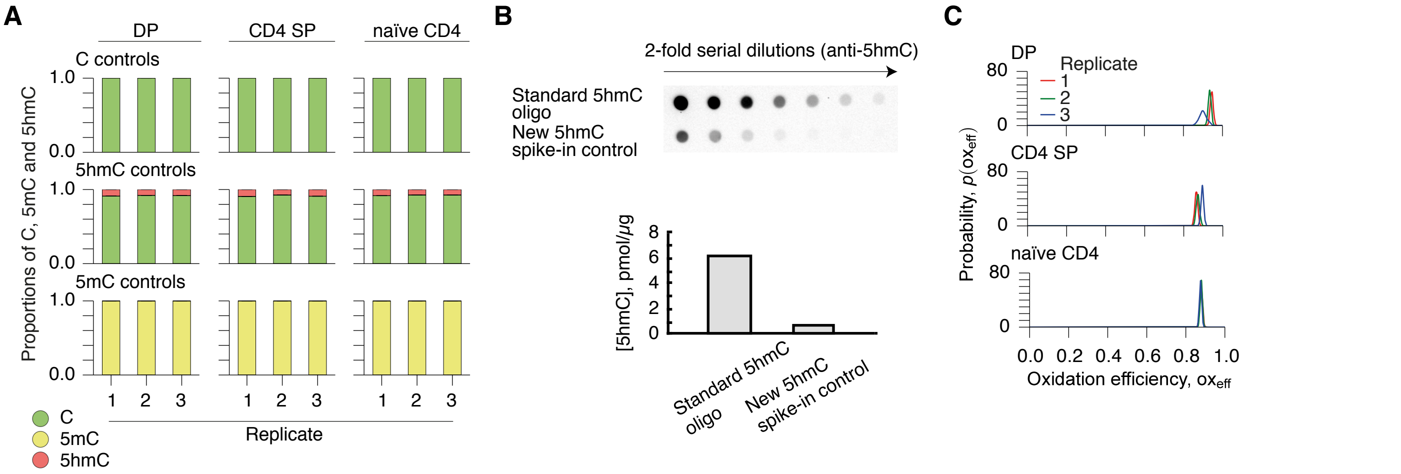

D

5hmC correlations (Pearson)

DP

r	Lux Rep1	Lux Rep2	Lux Rep3	MLML Rep1	MLML Rep2	MLML Rep3
Rep1	1.000	0.976	0.970	1.000	0.963	0.953
Rep2		1.000	0.984		1.000	0.981
Rep3			1.000			1.000

CD4 SP

r	Lux Rep1	Lux Rep2	Lux Rep3	MLML Rep1	MLML Rep2	MLML Rep3
Rep1	1.000	0.970	0.945	1.000	0.960	0.931
Rep2		1.000	0.964		1.000	0.950
Rep3			1.000			1.000

naïve CD4

r	Lux Rep1	Lux Rep2	Lux Rep3	MLML Rep1	MLML Rep2	MLML Rep3
Rep1	1.000	0.555	0.626	1.000	0.441	0.557
Rep2		1.000	0.954		1.000	0.933
Rep3			1.000			1.000

N=64 (Number of cytosines in CpG context)

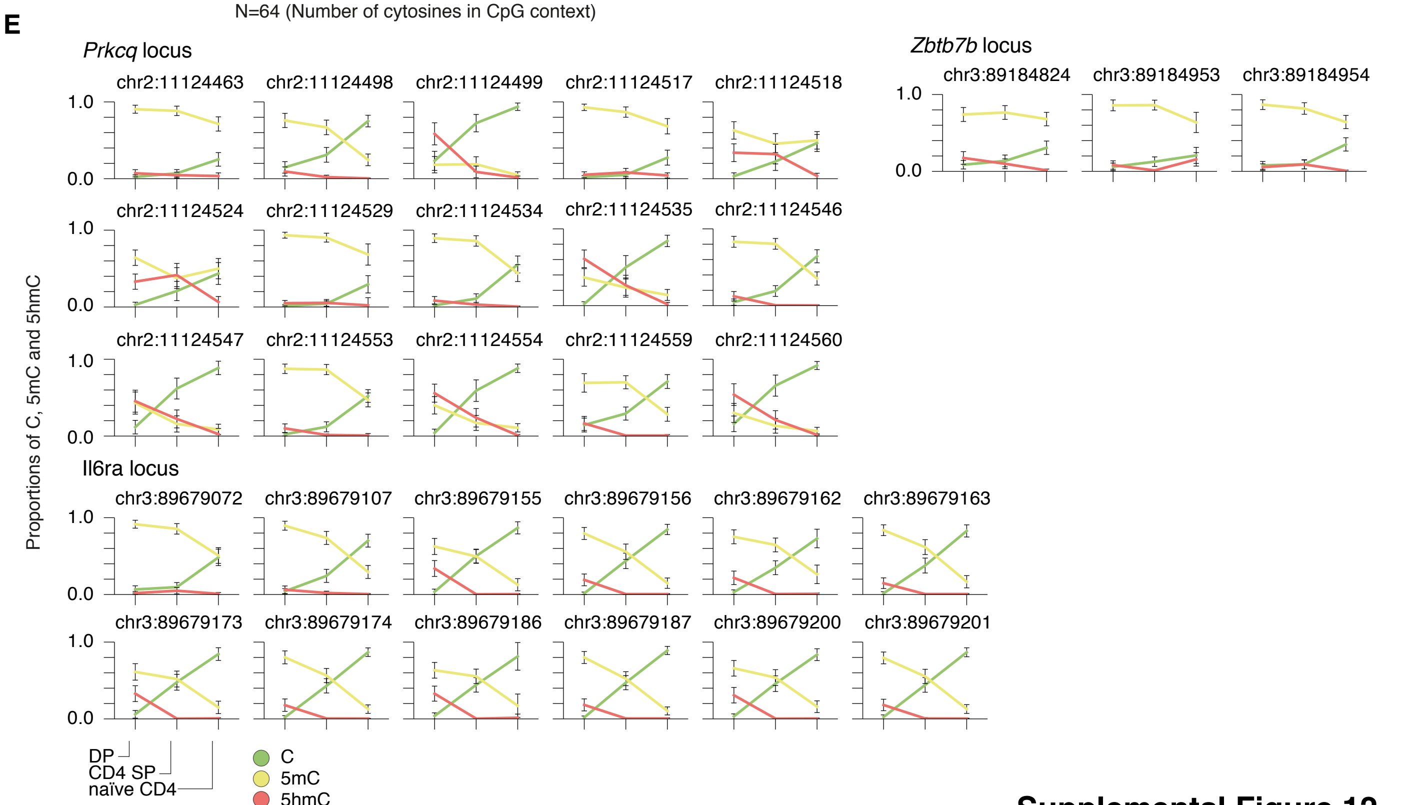

Supplemental Figure 12

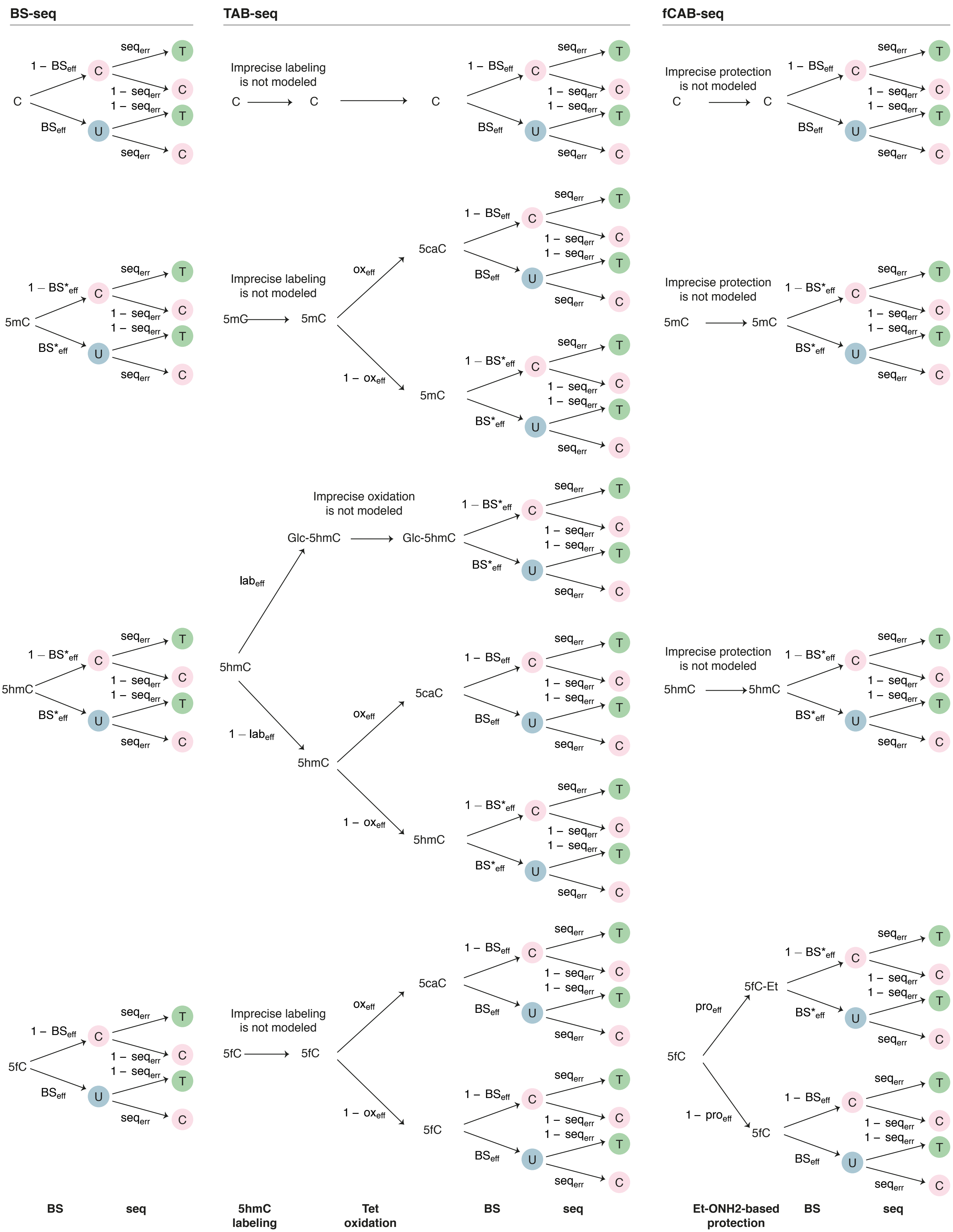

Supplemental Figure 13

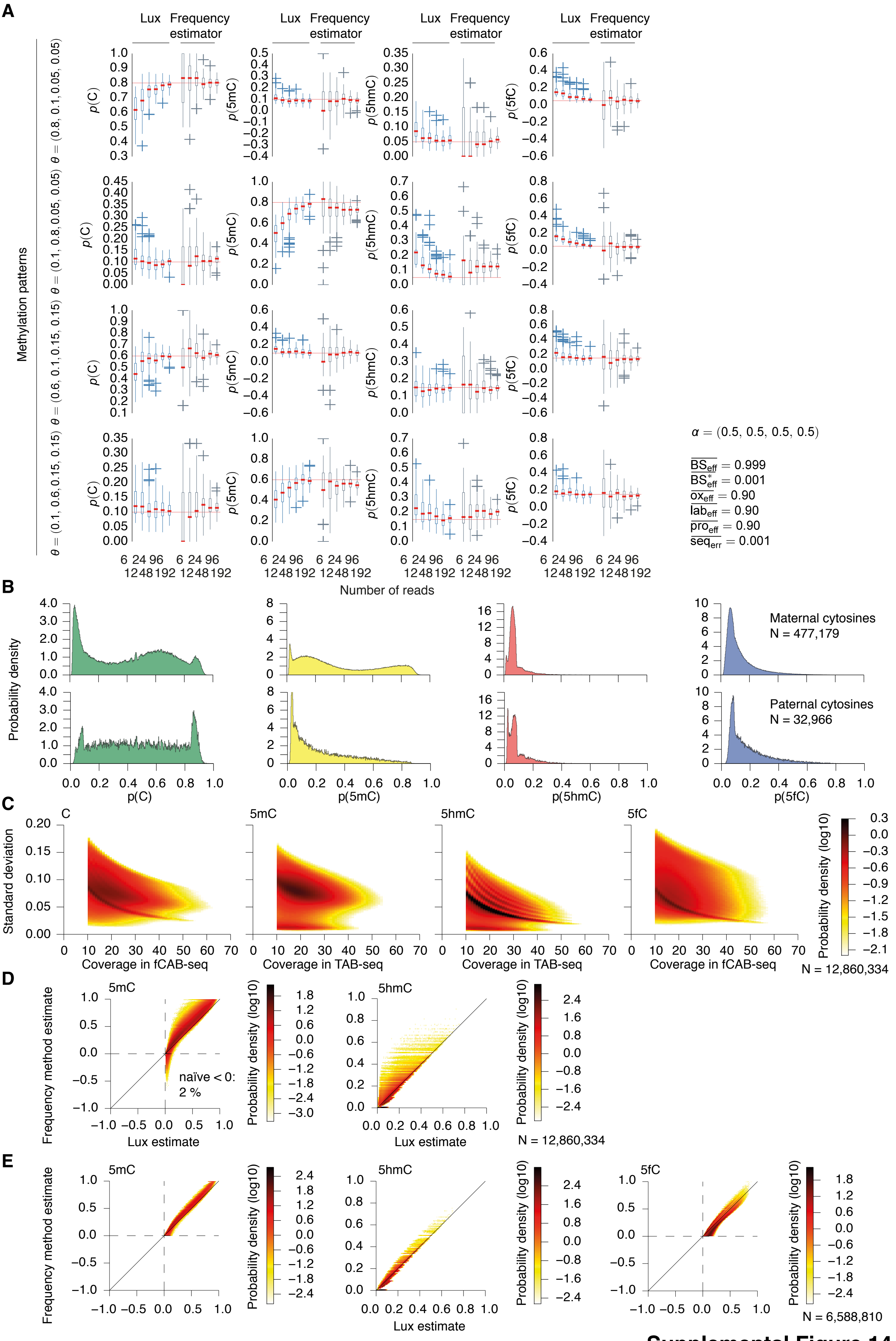

Supplemental Figure 14

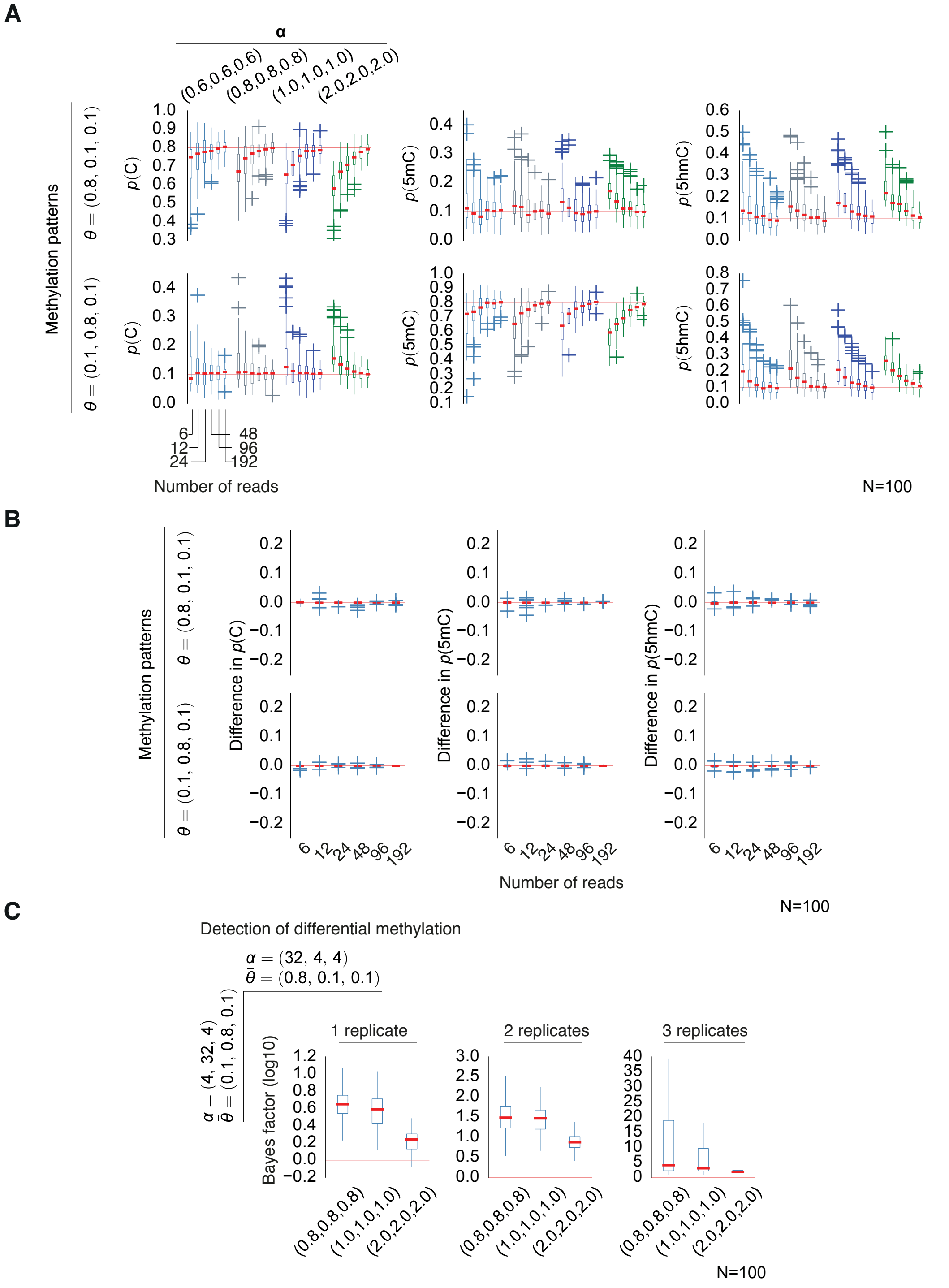

Supplemental Figure 15
