# Supplementary material for: A probabilistic generative model for quantification of DNA modifications enables analysis of demethylation pathways
Source: Genome Biol. 2016 Mar 14;17:49. doi: 10.1186/s13059-016-0911-6 (PMC4792102; doi:10.1186/s13059-016-0911-6)
Supplement: Additional file 5: — Tables S1 to S7 with legends. (PDF 4341 kb) [file 13059_2016_911_MOESM5_ESM.pdf]

Supplemental Table 1: The values of the fixed hyperparameters in the model are listed (**Suppl. Fig. 2a**). These parameters define the Dirichlet priors for control and wild-type cytosines, and the hyperpriors for the experimental parameters.

Supplemental Table 2: The posterior means and the standard deviations of the experimental parameters BS_{eff}, BS^{*}_{eff} and seq_{err} are listed per condition (v6.5 and Tet2kd) and replicate.

Supplemental Table 3: The numbers of converted and unconverted cytosines and the obtained Lux estimates are listed (v6.5 and Tet2kd).

Supplemental Table 4: GlucMS-qPCR validation for methylation level estimates. Pearson correlation values between glucMS-qPCR measurements and methylation level estimates obtained using 1) the Booth et al. method [32], 2) Lux with ideal experimental parameters, and 3) Lux with non-ideal experimental parameters. Significance (p-value) of the correlation is shown in parenthesis, i.e., p-values for testing the hypothesis of no correlation against the alternative that there is a nonzero correlation.

Supplemental Table 5: The results from the locus level analysis using the window-based analysis method. The considered windows, number of cytosines per window, and Bayes factors are listed.

Supplemental Table 6: The numbers of converted and unconverted cytosines and the obtained Lux estimates are listed (DP, CD4 SP, and naïve CD4).

Supplemental Table 7: The posterior means and the standard deviations of the experimental parameters BS_{eff}, BS^*_{eff} and seq_{err} are listed per condition (DP, CD4 SP, and naïve CD4) and replicate.

		α	
	Wild-type	(0.8, 0.8, 0.8)	Dirichlet
Controls	100% C	(1000, 1, 1)	
	100% 5mC	(1, 1000, 1)	
	~90% 5hmC	(6, 2, 72)	

Ψ_g^1 , Ψ_g^2	2, 2/6
$\Psi^{\mu,\mu}_{BS_{eff}}, \Psi^{\mu,\sigma}_{BS_{eff}}$	2, 1.29
$\Psi^{\mu,\mu}_{BS^*_{eff}}, \Psi^{\mu,\sigma}_{BS^*_{eff}}$	-3, 1.29
$\Psi^{\mu,\mu}_{ox_{eff}}, \Psi^{\mu,\sigma}_{ox_{eff}}$	2, 1.29
$\Psi^{\mu,\mu}_{seq_{err}}, \Psi^{\mu,\sigma}_{seq_{err}}$	-3, 1.29
$\Psi^{\sigma,\mu}_{BS_{eff}}, \Psi^{\sigma,\sigma}_{BS_{eff}}$	0.4, 0.5
$\Psi^{\sigma,\mu}_{BS^*_{eff}}, \Psi^{\sigma,\sigma}_{BS^*_{eff}}$	0.4, 0.5
$\Psi^{\sigma,\mu}_{ox_{eff}}, \Psi^{\sigma,\sigma}_{ox_{eff}}$	0.4, 0.5
$\Psi^{\sigma,\mu}_{seq_{err}}, \Psi^{\sigma,\sigma}_{seq_{err}}$	0.4, 0.5

Supplemental Table 1

		Posterior mean ± standard deviation		
		BS_{eff}	BS^*_{eff}	seq_{err}
v6.5	Replicate 1	1.0e0 ± 3.1e-7	1.1e-2 ± 2.0e-4	1.4e-7 ± 3.0e-7
	Replicate 2	1.0e0 ± 3.2e-7	5.6e-3 ± 3.5e-4	1.5e-7 ± 2.9e-7
	Replicate 3	1.0e0 ± 3.4e-7	2.0e-3 ± 1.3e-4	1.3e-7 ± 3.0e-7
Tet2kd	Replicate 1	1.0e0 ± 3.4e-7	3.9e-3 ± 2.2e-4	1.7e-7 ± 2.7e-7
	Replicate 2	1.0e0 ± 3.2e-7	4.6e-3 ± 3.3e-4	1.4e-7 ± 2.8e-7
	Replicate 3	1.0e0 ± 3.3e-7	2.1e-3 ± 1.8e-4	1.4e-7 ± 3.0e-7

Supplemental Table 2

Supplemental Table 3

v6.5 controls

Number of C's and T's

			v65bs1		v65oxbs1		v65bs2		v65oxbs2		v65bs3		v65oxbs3	
**Chromosome**	**Position**		**C to C**	**C to T**	**C to C**	**C to T**	**C to C**	**C to T**	**C to C**	**C to T**	**C to C**	**C to T**	**C to C**	**C to T**
Lambda_ctrl	22924	C	28	11306	19	9202	7	4189	20	4079	16	6242	34	14757
Lambda_ctrl	22928	C	29	11290	25	9176	7	4182	17	4080	8	6239	45	14726
Lambda_ctrl	22933	C	27	11313	17	9210	9	4188	15	4089	8	6252	24	14771
Lambda_ctrl	22938	C	20	11319	30	9198	14	4182	14	4089	11	6249	31	14764
Lambda_ctrl	22939	C	17	11319	17	9205	8	4186	16	4086	8	6247	25	14764
Lambda_ctrl	22944	C	16	11328	32	9194	13	4182	18	4086	10	6251	31	14761
Lambda_ctrl	22946	C	24	11318	22	9210	7	4190	16	4089	10	6251	20	14779
Lambda_ctrl	22953	C	13	11332	21	9211	6	4191	18	4088	10	6253	14	14786
Lambda_ctrl	22954	C	10	11333	16	9216	6	4190	17	4089	8	6254	20	14781
Lambda_ctrl	22963	C	24	11315	19	9207	6	4191	16	4086	8	6253	28	14768
Lambda_ctrl	22964	C	20	11325	13	9219	8	4189	14	4091	14	6248	21	14778
Lambda_ctrl	22970	C	26	11314	20	9210	5	4191	17	4088	10	6249	34	14763
Lambda_ctrl	22985	C	25	11310	25	9201	6	4182	15	4087	6	6254	31	14762
Lambda_ctrl	22987	C	21	11322	14	9216	5	4190	17	4089	9	6252	27	14771
Lambda_ctrl	22991	C	16	11327	22	9210	4	4192	16	4090	12	6250	27	14773
Lambda_ctrl	22994	C	24	11317	21	9209	13	4182	17	4087	15	6246	30	14766
Lambda_ctrl	22997	C	30	11311	21	9209	17	4179	16	4088	13	6248	46	14750
Lambda_ctrl	23002	C	35	11308	33	9199	9	4187	15	4090	12	6251	40	14758
Lambda_ctrl	23004	C	42	11301	29	9200	9	4186	21	4084	15	6246	41	14758
Lambda_ctrl	23005	C	12	11332	23	9209	10	4185	19	4086	15	6248	27	14772
Lambda_ctrl	23021	C	23	11320	29	9203	11	4186	18	4087	11	6251	30	14771
Lambda_ctrl	23024	C	41	11302	41	9189	8	4188	15	4091	15	6248	42	14757
Lambda_ctrl	23030	C	31	11311	30	9200	13	4184	20	4086	13	6250	30	14767
Lambda_ctrl	23033	C	23	11320	18	9214	16	4181	15	4091	12	6251	28	14772
Lambda_ctrl	23054	C	26	11302	10	9214	5	4188	12	4089	10	6243	24	14746
Lambda_ctrl	23075	C	34	10844	21	8953	6	3981	13	3941	10	5891	32	14144
Lambda_ctrl	23789	C	37843	4600	288	1037	19540	1610	961	1116	33879	2452	2378	7871
Lambda_ctrl	23794	C	35775	7811	262	1216	17429	4152	964	1159	30606	6555	2243	8133
Lambda_ctrl	23798	C	37498	6368	304	1209	18543	3212	972	1161	32182	5328	2440	7980
Lambda_ctrl	23817	C	37407	6540	320	1203	18869	2985	974	1166	32573	5207	2434	8017
Lambda_ctrl	23823	C	37348	6605	318	1205	18642	3219	971	1168	32362	5433	2439	8016
Lambda_ctrl	23827	C	37654	6240	310	1207	18877	2965	975	1157	32621	5143	2428	7965
Lambda_ctrl	23837	C	37510	6455	310	1213	18676	3197	993	1147	32384	5427	2493	7966
Lambda_ctrl	23843	C	35998	7959	304	1219	17935	3938	1005	1134	31067	6742	2465	7994
Lambda_ctrl	23855	C	38050	5910	315	1207	19003	2870	1006	1134	32977	4828	2582	7875
Lambda_ctrl	23860	C	39336	4619	335	1188	19634	2237	993	1145	33833	3973	2658	7797
Lambda_ctrl	23863	C	39193	4763	312	1210	19624	2247	1000	1139	33778	4027	2637	7817
Lambda_ctrl	23871	C	39072	4880	318	1204	19419	2449	980	1159	33646	4161	2542	7909
Lambda_ctrl	23877	C	38842	5116	306	1217	19369	2501	986	1153	33553	4252	2489	7967
Lambda_ctrl	23885	C	31700	12259	298	1225	15369	6505	965	1175	27206	10606	2312	8147

Number of C's and total read outs

v65bs1		v65oxbs1		v65bs2		v65oxbs2		v65bs3		v65oxbs3	
**C**	**Total**	**C**	**Total**	**C**	**Total**	**C**	**Total**	**C**	**Total**	**C**	**Total**
28	11334	19	9221	7	4196	20	4099	16	6258	34	14791
29	11319	25	9201	7	4189	17	4097	8	6247	45	14771
27	11340	17	9227	9	4197	15	4104	8	6260	24	14795
20	11339	30	9228	14	4196	14	4103	11	6260	31	14795
17	11336	17	9222	8	4194	16	4102	8	6255	25	14789
16	11344	32	9226	13	4195	18	4104	10	6261	31	14792
24	11342	22	9232	7	4197	16	4105	10	6261	20	14799
13	11345	21	9232	6	4197	18	4106	10	6263	14	14800
10	11343	16	9232	6	4196	17	4106	8	6262	20	14801
24	11339	19	9226	6	4197	16	4102	8	6261	28	14796
20	11345	13	9232	8	4197	14	4105	14	6262	21	14799
26	11340	20	9230	5	4196	17	4105	10	6259	34	14797
25	11335	25	9226	6	4188	15	4102	6	6260	31	14793
21	11343	14	9230	5	4195	17	4106	9	6261	27	14798
16	11343	22	9232	4	4196	16	4106	12	6262	27	14800
24	11341	21	9230	13	4195	17	4104	15	6261	30	14796
30	11341	21	9230	17	4196	16	4104	13	6261	46	14796
35	11343	33	9232	9	4196	15	4105	12	6263	40	14798
42	11343	29	9229	9	4195	21	4105	15	6261	41	14799
12	11344	23	9232	10	4195	19	4105	15	6263	27	14799
23	11343	29	9232	11	4197	18	4105	11	6262	30	14801
41	11343	41	9230	8	4196	15	4106	15	6263	42	14799
31	11342	30	9230	13	4197	20	4106	13	6263	30	14797
23	11343	18	9232	16	4197	15	4106	12	6263	28	14800
26	11328	10	9224	5	4193	12	4101	10	6253	24	14770
34	10878	21	8974	6	3987	13	3954	10	5901	32	14176
37843	42443	288	1325	19540	21150	961	2077	33879	36331	2378	10249
35775	43586	262	1478	17429	21581	964	2123	30606	37161	2243	10376
37498	43866	304	1513	18543	21755	972	2133	32182	37510	2440	10420
37407	43947	320	1523	18869	21854	974	2140	32573	37780	2434	10451
37348	43953	318	1523	18642	21861	971	2139	32362	37795	2439	10455
37654	43894	310	1517	18877	21842	975	2132	32621	37764	2428	10393
37510	43965	310	1523	18676	21873	993	2140	32384	37811	2493	10459
35998	43957	304	1523	17935	21873	1005	2139	31067	37809	2465	10459
38050	43960	315	1522	19003	21873	1006	2140	32977	37805	2582	10457
39336	43955	335	1523	19634	21871	993	2138	33833	37806	2658	10455
39193	43956	312	1522	19624	21871	1000	2139	33778	37805	2637	10454
39072	43952	318	1522	19419	21868	980	2139	33646	37807	2542	10451
38842	43958	306	1523	19369	21870	986	2139	33553	37805	2489	10456
31700	43959	298	1523	15369	21874	965	2140	27206	37812	2312	10459

Lambda_ctrl	23893	C	33795	10154	285	1221	16869	4991	955	1160	29336	8455	2242	8010
Lambda_ctrl	23896	C	31014	12946	277	1246	15166	6709	948	1192	27028	10785	2297	8161
Lambda_ctrl	23900	C	29999	13957	294	1229	14830	7042	947	1192	25758	12046	2301	8161
Lambda_ctrl	23901	C	26821	17132	267	1255	12901	8968	952	1188	22911	14892	2253	8202
Lambda_ctrl	23902	C	29198	14756	300	1223	14662	7209	977	1163	25811	11994	2440	8020
Lambda_ctrl	23916	C	37242	6711	336	1186	18759	3113	1008	1131	32353	5446	2684	7775
Lambda_ctrl	23923	C	36054	7672	293	1227	18048	3703	956	1177	31286	6303	2366	8054
Lambda_ctrl	23925	C	36478	7411	291	1232	18214	3617	968	1169	31715	5991	2301	8137
Lambda_ctrl	23927	C	33304	8912	277	1229	16617	4400	932	1161	28637	7705	2271	8015
Lambda_ctrl	23928	C	34569	8960	309	1212	17539	4156	963	1163	30248	7212	2401	8012
Lambda_ctrl	23943	C	38681	4913	299	1200	19547	2235	1008	1125	33732	3893	2608	7798
Lambda_ctrl	23947	C	38040	5728	293	1201	19013	2739	947	1185	32832	4678	2371	8010
Lambda_ctrl	23954	C	36518	6344	269	1002	18408	3122	948	1145	32202	4899	2453	7807
Lambda_ctrl	47359	C	82243	579	78997	796	20919	48	17497	31	139809	208	20616	36
Lambda_ctrl	47367	C	84074	1126	80369	1225	20975	96	17519	39	140149	391	20651	56
Lambda_ctrl	47377	C	84253	978	80500	1118	20914	164	17416	145	139677	905	20545	167
Lambda_ctrl	47387	C	84284	979	80610	1048	20897	197	17394	172	139683	1024	20543	176
Lambda_ctrl	47392	C	84139	1129	80371	1296	20905	197	17379	190	139583	1185	20498	222
Lambda_ctrl	47394	C	84250	1032	80493	1192	20877	227	17390	184	139597	1193	20496	227
Lambda_ctrl	47398	C	84116	1175	80305	1380	20867	240	17348	235	139436	1395	20441	285
Lambda_ctrl	47404	C	84079	1219	80327	1361	20887	223	17370	211	139561	1291	20465	265
Lambda_ctrl	47409	C	84138	1157	80302	1374	20822	287	17336	246	139228	1611	20416	311
Lambda_ctrl	47411	C	84227	1073	80461	1233	20861	248	17340	242	139370	1477	20439	290
Lambda_ctrl	47413	C	84250	1049	80441	1253	20874	236	17329	254	139423	1425	20411	318
Lambda_ctrl	47419	C	84237	1051	80433	1249	20813	293	17284	296	139324	1515	20422	306
Lambda_ctrl	47428	C	84170	1119	80343	1338	20852	255	17303	275	139406	1424	20436	292
Lambda_ctrl	47440	C	84311	980	80660	1026	20889	216	17354	228	139293	1549	20452	275
Lambda_ctrl	47443	C	84269	1017	80446	1242	20867	243	17299	282	139195	1649	20394	336
Lambda_ctrl	47444	C	84163	1126	80353	1331	20838	273	17278	303	139073	1763	20377	353
Lambda_ctrl	47449	C	84412	862	80670	1005	20854	251	17324	250	139326	1477	20459	264
Lambda_ctrl	47450	C	84327	967	80641	1046	20863	244	17357	219	139424	1415	20450	275
Lambda_ctrl	47461	C	84280	1013	80464	1223	20896	212	17331	249	139366	1474	20457	271
Lambda_ctrl	47462	C	84272	1011	80458	1231	20848	262	17322	258	139264	1574	20384	339
Lambda_ctrl	47465	C	84191	1105	80376	1309	20860	248	17297	281	139151	1683	20412	313
Lambda_ctrl	47466	C	84243	1044	80475	1208	20872	234	17332	251	139340	1508	20407	321
Lambda_ctrl	47475	C	84257	1034	80516	1164	20871	238	17364	217	139275	1568	20408	322
Lambda_ctrl	47481	C	84007	1269	80245	1407	20844	263	17261	315	138786	2032	20370	355
Lambda_ctrl	47483	C	84137	1153	80348	1334	20860	250	17341	239	139050	1788	20383	343
Lambda_ctrl	47492	C	84216	1003	80544	1111	20889	202	17343	232	139325	1442	20462	259
Lambda_ctrl	47498	C	84062	1135	80271	1345	20856	228	17294	268	139144	1591	20397	312
Lambda_ctrl	47507	C	84022	1186	80317	1318	20810	270	17333	226	139055	1675	20407	308
Lambda_ctrl	47512	C	84141	1069	80427	1209	20865	216	17335	218	139262	1429	20470	237
Lambda_ctrl	47516	C	84091	1113	80369	1267	20846	236	17304	243	139069	1619	20442	267
Lambda_ctrl	47521	C	84134	1048	80195	1408	20871	206	17321	219	138903	1746	20443	260
Lambda_ctrl	47525	C	84179	972	80310	1265	20860	200	17340	186	139006	1570	20444	236
Lambda_ctrl	47526	C	84071	1006	80192	1313	20862	189	17310	214	138941	1600	20455	215

33795	43949	285	1506	16869	21860	955	2115	29336	37791	2242	10252
31014	43960	277	1523	15166	21875	948	2140	27028	37813	2297	10458
29999	43956	294	1523	14830	21872	947	2139	25758	37804	2301	10462
26821	43953	267	1522	12901	21869	952	2140	22911	37803	2253	10455
29198	43954	300	1523	14662	21871	977	2140	25811	37805	2440	10460
37242	43953	336	1522	18759	21872	1008	2139	32353	37799	2684	10459
36054	43726	293	1520	18048	21751	956	2133	31286	37589	2366	10420
36478	43889	291	1523	18214	21831	968	2137	31715	37706	2301	10438
33304	42216	277	1506	16617	21017	932	2093	28637	36342	2271	10286
34569	43529	309	1521	17539	21695	963	2126	30248	37460	2401	10413
38681	43594	299	1499	19547	21782	1008	2133	33732	37625	2608	10406
38040	43768	293	1494	19013	21752	947	2132	32832	37510	2371	10381
36518	42862	269	1271	18408	21530	948	2093	32202	37101	2453	10260
82243	82822	78997	79793	20919	20967	17497	17528	1E+05	140017	20616	20652
84074	85200	80369	81594	20975	21071	17519	17558	1E+05	140540	20651	20707
84253	85231	80500	81618	20914	21078	17416	17561	1E+05	140582	20545	20712
84284	85263	80610	81658	20897	21094	17394	17566	1E+05	140707	20543	20719
84139	85268	80371	81667	20905	21102	17379	17569	1E+05	140768	20498	20720
84250	85282	80493	81685	20877	21104	17390	17574	1E+05	140790	20496	20723
84116	85291	80305	81685	20867	21107	17348	17583	1E+05	140831	20441	20726
84079	85298	80327	81688	20887	21110	17370	17581	1E+05	140852	20465	20730
84138	85295	80302	81676	20822	21109	17336	17582	1E+05	140839	20416	20727
84227	85300	80461	81694	20861	21109	17340	17582	1E+05	140847	20439	20729
84250	85299	80441	81694	20874	21110	17329	17583	1E+05	140848	20411	20729
84237	85288	80433	81682	20813	21106	17284	17580	1E+05	140839	20422	20728
84170	85289	80343	81681	20852	21107	17303	17578	1E+05	140830	20436	20728
84311	85291	80660	81686	20889	21105	17354	17582	1E+05	140842	20452	20727
84269	85286	80446	81688	20867	21110	17299	17581	1E+05	140844	20394	20730
84163	85289	80353	81684	20838	21111	17278	17581	1E+05	140836	20377	20730
84412	85274	80670	81675	20854	21105	17324	17574	1E+05	140803	20459	20723
84327	85294	80641	81687	20863	21107	17357	17576	1E+05	140839	20450	20725
84280	85293	80464	81687	20896	21108	17331	17580	1E+05	140840	20457	20728
84272	85283	80458	81689	20848	21110	17322	17580	1E+05	140838	20384	20723
84191	85296	80376	81685	20860	21108	17297	17578	1E+05	140834	20412	20725
84243	85287	80475	81683	20872	21106	17332	17583	1E+05	140848	20407	20728
84257	85291	80516	81680	20871	21109	17364	17581	1E+05	140843	20408	20730
84007	85276	80245	81652	20844	21107	17261	17576	1E+05	140818	20370	20725
84137	85290	80348	81682	20860	21110	17341	17580	1E+05	140838	20383	20726
84216	85219	80544	81655	20889	21091	17343	17575	1E+05	140767	20462	20721
84062	85197	80271	81616	20856	21084	17294	17562	1E+05	140735	20397	20709
84022	85208	80317	81635	20810	21080	17333	17559	1E+05	140730	20407	20715
84141	85210	80427	81636	20865	21081	17335	17553	1E+05	140691	20470	20707
84091	85204	80369	81636	20846	21082	17304	17547	1E+05	140688	20442	20709
84134	85182	80195	81603	20871	21077	17321	17540	1E+05	140649	20443	20703
84179	85151	80310	81575	20860	21060	17340	17526	1E+05	140576	20444	20680
84071	85077	80192	81505	20862	21051	17310	17524	1E+05	140541	20455	20670

Lambda_ctrl	47528	C	83904	1112	80050	1402	20827	210	17313	196	138726	1714	20427	238
Lambda_ctrl	47529	C	83778	1164	79899	1479	20805	217	17307	199	138626	1749	20426	228

83904	85016	80050	81452	20827	21037	17313	17509	1E+05	140440	20427	20665
83778	84942	79899	81378	20805	21022	17307	17506	1E+05	140375	20426	20654

Tet2kd controls

Number of C's and T's

			t2kdbs1		t2kdoxbs1		t2kdbs2		t2kdoxbs2		t2kdbs3		t2kdoxbs3	
**Chromosome**	**Position**		**C to C**	**C to T**	**C to C**	**C to T**	**C to C**	**C to T**	**C to C**	**C to T**	**C to C**	**C to T**	**C to C**	**C to T**
Lambda_ctrl	22924	C	11	4923	22	9746	20	10645	12	4542	6	3030	19	8652
Lambda_ctrl	22928	C	10	4916	24	9731	15	10639	10	4541	8	3032	14	8645
Lambda_ctrl	22933	C	5	4931	16	9760	9	10664	3	4559	2	3043	18	8655
Lambda_ctrl	22938	C	11	4923	23	9750	16	10658	6	4560	10	3041	13	8658
Lambda_ctrl	22939	C	8	4925	19	9752	14	10655	8	4554	11	3038	18	8654
Lambda_ctrl	22944	C	7	4925	24	9750	14	10656	6	4556	9	3033	21	8651
Lambda_ctrl	22946	C	10	4926	19	9756	6	10670	10	4555	6	3046	15	8663
Lambda_ctrl	22953	C	5	4931	18	9759	12	10664	10	4557	4	3049	10	8669
Lambda_ctrl	22954	C	5	4930	15	9763	13	10663	8	4559	6	3047	13	8667
Lambda_ctrl	22963	C	3	4930	18	9754	16	10657	7	4555	7	3046	21	8656
Lambda_ctrl	22964	C	7	4929	16	9763	9	10666	8	4559	19	3032	11	8670
Lambda_ctrl	22970	C	8	4927	18	9759	10	10664	10	4557	6	3046	10	8666
Lambda_ctrl	22985	C	9	4922	21	9751	12	10660	14	4549	10	3040	18	8654
Lambda_ctrl	22987	C	5	4931	9	9766	9	10667	8	4558	8	3043	10	8669
Lambda_ctrl	22991	C	4	4932	10	9768	14	10663	10	4557	5	3047	14	8662
Lambda_ctrl	22994	C	10	4923	25	9750	15	10658	15	4550	10	3042	24	8650
Lambda_ctrl	22997	C	15	4920	28	9748	30	10643	7	4559	9	3042	17	8659
Lambda_ctrl	23002	C	10	4926	17	9760	14	10661	10	4557	11	3041	18	8660
Lambda_ctrl	23004	C	17	4917	29	9747	27	10647	10	4556	5	3047	15	8663
Lambda_ctrl	23005	C	6	4929	18	9761	17	10659	11	4556	6	3045	15	8662
Lambda_ctrl	23021	C	7	4929	20	9760	15	10660	7	4562	11	3040	24	8652
Lambda_ctrl	23024	C	9	4926	30	9747	22	10654	13	4557	14	3037	27	8648
Lambda_ctrl	23030	C	9	4926	20	9759	18	10658	12	4558	7	3045	20	8657
Lambda_ctrl	23033	C	5	4931	21	9759	10	10665	9	4561	6	3045	11	8665
Lambda_ctrl	23054	C	6	4915	20	9756	13	10654	7	4558	4	3043	12	8641
Lambda_ctrl	23075	C	13	4656	18	9571	20	10210	9	4492	6	2957	21	8222
Lambda_ctrl	23789	C	30680	2927	1752	3493	22073	1701	1345	2007	37202	1930	5537	6454
Lambda_ctrl	23794	C	28629	5943	1737	3613	19789	4531	1347	2063	33692	6759	5531	6676
Lambda_ctrl	23798	C	29981	4867	1814	3561	21191	3292	1381	2042	36173	4867	5719	6532
Lambda_ctrl	23817	C	30088	4956	1812	3577	21417	3266	1389	2037	36447	5151	5750	6540
Lambda_ctrl	23823	C	29957	5096	1801	3592	21245	3443	1365	2063	36022	5593	5750	6543
Lambda_ctrl	23827	C	30166	4857	1767	3585	21526	3139	1374	2039	36208	5391	5739	6502
Lambda_ctrl	23837	C	30034	5020	1799	3598	21141	3557	1386	2042	35978	5664	5808	6488
Lambda_ctrl	23843	C	28833	6227	1798	3597	20450	4245	1409	2018	34672	6971	5741	6555
Lambda_ctrl	23855	C	30560	4498	1820	3577	21523	3173	1404	2024	36465	5183	5833	6460
Lambda_ctrl	23860	C	31491	3565	1850	3546	22227	2471	1428	2000	37442	4196	5851	6440
Lambda_ctrl	23863	C	31217	3835	1857	3537	22118	2576	1432	1994	37395	4246	5898	6389
Lambda_ctrl	23871	C	31115	3934	1834	3559	22202	2486	1386	2041	37193	4443	5865	6424
Lambda_ctrl	23877	C	31079	3967	1829	3565	21916	2775	1382	2045	37143	4498	5815	6471
Lambda_ctrl	23885	C	25084	9971	1714	3683	17201	7497	1362	2066	29426	12218	5664	6626

Number of C's and total read outs

t2kdbs1		t2kdoxbs1		t2kdbs2		t2kdoxbs2		t2kdbs3		t2kdoxbs3	
C	Total	C	Total	C	Total	C	Total	C	Total	C	Total
11	4934	22	9768	20	10665	12	4554	6	3036	19	8671
10	4926	24	9755	15	10654	10	4551	8	3040	14	8659
5	4936	16	9776	9	10673	3	4562	2	3045	18	8673
11	4934	23	9773	16	10674	6	4566	10	3051	13	8671
8	4933	19	9771	14	10669	8	4562	11	3049	18	8672
7	4932	24	9774	14	10670	6	4562	9	3042	21	8672
10	4936	19	9775	6	10676	10	4565	6	3052	15	8678
5	4936	18	9777	12	10676	10	4567	4	3053	10	8679
5	4935	15	9778	13	10676	8	4567	6	3053	13	8680
3	4933	18	9772	16	10673	7	4562	7	3053	21	8677
7	4936	16	9779	9	10675	8	4567	19	3051	11	8681
8	4935	18	9777	10	10674	10	4567	6	3052	10	8676
9	4931	21	9772	12	10672	14	4563	10	3050	18	8672
5	4936	9	9775	9	10676	8	4566	8	3051	10	8679
4	4936	10	9778	14	10677	10	4567	5	3052	14	8676
10	4933	25	9775	15	10673	15	4565	10	3052	24	8674
15	4935	28	9776	30	10673	7	4566	9	3051	17	8676
10	4936	17	9777	14	10675	10	4567	11	3052	18	8678
17	4934	29	9776	27	10674	10	4566	5	3052	15	8678
6	4935	18	9779	17	10676	11	4567	6	3051	15	8677
7	4936	20	9780	15	10675	7	4569	11	3051	24	8676
9	4935	30	9777	22	10676	13	4570	14	3051	27	8675
9	4935	20	9779	18	10676	12	4570	7	3052	20	8677
5	4936	21	9780	10	10675	9	4570	6	3051	11	8676
6	4921	20	9776	13	10667	7	4565	4	3047	12	8653
13	4669	18	9589	20	10230	9	4501	6	2963	21	8243
30680	33607	1752	5245	22073	23774	1345	3352	37202	39132	5537	11991
28629	34572	1737	5350	19789	24320	1347	3410	33692	40451	5531	12207
29981	34848	1814	5375	21191	24483	1381	3423	36173	41040	5719	12251
30088	35044	1812	5389	21417	24683	1389	3426	36447	41598	5750	12290
29957	35053	1801	5393	21245	24688	1365	3428	36022	41615	5750	12293
30166	35023	1767	5352	21526	24665	1374	3413	36208	41599	5739	12241
30034	35054	1799	5397	21141	24698	1386	3428	35978	41642	5808	12296
28833	35060	1798	5395	20450	24695	1409	3427	34672	41643	5741	12296
30560	35058	1820	5397	21523	24696	1404	3428	36465	41648	5833	12293
31491	35056	1850	5396	22227	24698	1428	3428	37442	41638	5851	12291
31217	35052	1857	5394	22118	24694	1432	3426	37395	41641	5898	12287
31115	35049	1834	5393	22202	24688	1386	3427	37193	41636	5865	12289
31079	35046	1829	5394	21916	24691	1382	3427	37143	41641	5815	12286
25084	35055	1714	5397	17201	24698	1362	3428	29426	41644	5664	12290

Lambda_ctrl	23893	C	26850	8186	1711	3581	18747	5945	1336	2051	31663	9970	5644	6471
Lambda_ctrl	23896	C	24114	10941	1717	3679	16505	8192	1325	2102	28296	13348	5611	6684
Lambda_ctrl	23900	C	24030	11030	1728	3669	16721	7977	1340	2087	28257	13384	5654	6640
Lambda_ctrl	23901	C	21111	13942	1704	3690	14603	10089	1329	2098	24718	16921	5646	6647
Lambda_ctrl	23902	C	23847	11208	1775	3621	16912	7779	1371	2057	28880	12761	5777	6516
Lambda_ctrl	23916	C	29832	5222	1847	3550	20911	3776	1391	2036	35486	6150	5820	6473
Lambda_ctrl	23923	C	28881	5990	1723	3652	20167	4358	1362	2052	34241	7152	5641	6595
Lambda_ctrl	23925	C	29152	5838	1710	3675	20353	4270	1338	2084	34639	6908	5614	6661
Lambda_ctrl	23927	C	26506	7145	1686	3615	18578	5004	1328	2050	31644	8269	5368	6621
Lambda_ctrl	23928	C	27874	6861	1732	3634	19611	4833	1330	2074	33598	7634	5641	6566
Lambda_ctrl	23943	C	31014	3764	1826	3543	22075	2466	1398	2014	37781	3657	5821	6429
Lambda_ctrl	23947	C	30412	4382	1734	3634	21462	3040	1345	2064	37293	4122	5635	6603
Lambda_ctrl	23954	C	29066	4996	1745	3480	20885	3255	1315	2038	36759	4093	5509	6557
Lambda_ctrl	47359	C	45600	82	19516	43	26994	36	29149	105	54088	53	281605	1799
Lambda_ctrl	47367	C	46242	183	19595	62	27131	61	29287	211	54170	89	283048	3391
Lambda_ctrl	47377	C	46053	389	19463	195	27006	179	29193	314	53991	282	283065	3583
Lambda_ctrl	47387	C	46109	386	19449	215	27000	209	29167	355	53962	374	283419	3511
Lambda_ctrl	47392	C	46129	396	19378	295	26949	258	29154	377	53942	411	283106	3917
Lambda_ctrl	47394	C	46123	409	19419	256	26953	267	29193	340	53918	448	283275	3815
Lambda_ctrl	47398	C	46035	511	19364	316	26946	269	29091	447	53851	525	282722	4452
Lambda_ctrl	47404	C	46099	455	19414	268	26935	287	29128	414	53831	553	283093	4100
Lambda_ctrl	47409	C	46057	492	19380	297	26896	324	29069	473	53795	588	282585	4595
Lambda_ctrl	47411	C	46069	481	19410	273	26950	271	29113	427	53806	574	282922	4261
Lambda_ctrl	47413	C	46057	497	19383	299	26943	278	29124	419	53790	591	282890	4306
Lambda_ctrl	47419	C	46042	507	19384	296	26941	275	29100	438	53723	654	282637	4537
Lambda_ctrl	47428	C	46003	546	19428	253	26940	276	29115	427	53831	542	282843	4316
Lambda_ctrl	47440	C	46049	500	19440	241	26928	292	29141	395	53795	585	283315	3866
Lambda_ctrl	47443	C	46036	511	19379	303	26893	328	29071	463	53724	655	282650	4532
Lambda_ctrl	47444	C	45983	567	19352	328	26866	354	29026	513	53686	692	282462	4702
Lambda_ctrl	47449	C	46065	475	19435	236	26928	288	29170	356	53802	572	283278	3836
Lambda_ctrl	47450	C	46077	472	19434	246	26971	247	29135	405	53791	585	283385	3780
Lambda_ctrl	47461	C	46102	446	19418	261	26929	287	29110	433	53841	538	282962	4211
Lambda_ctrl	47462	C	46064	488	19357	321	26931	288	29111	429	53754	623	282897	4270
Lambda_ctrl	47465	C	46012	537	19357	323	26925	294	29089	451	53726	650	282648	4520
Lambda_ctrl	47466	C	46064	488	19360	319	26915	299	29110	432	53797	577	282848	4301
Lambda_ctrl	47475	C	46022	531	19429	253	26929	289	29096	444	53760	622	282932	4250
Lambda_ctrl	47481	C	45929	612	19338	337	26828	380	29033	497	53550	814	281723	5381
Lambda_ctrl	47483	C	45963	587	19381	302	26926	296	29050	489	53655	726	282402	4753
Lambda_ctrl	47492	C	46046	471	19419	245	26952	255	29122	400	53838	502	283080	3952
Lambda_ctrl	47498	C	45966	544	19363	295	26928	270	29048	471	53700	588	282508	4486
Lambda_ctrl	47507	C	45935	569	19350	294	26896	289	29066	441	53634	636	282603	4359
Lambda_ctrl	47512	C	46028	464	19380	251	26911	265	29154	347	53712	495	283101	3826
Lambda_ctrl	47516	C	45909	573	19354	278	26881	285	29054	447	53720	477	282399	4517
Lambda_ctrl	47521	C	45871	577	19301	311	26874	287	29036	454	53619	545	282120	4691
Lambda_ctrl	47525	C	45863	503	19326	264	26915	236	29038	435	53608	491	281909	4716
Lambda_ctrl	47526	C	45778	527	19349	223	26916	228	29088	373	53645	438	282045	4429

26850	35036	1711	5292	18747	24692	1336	3387	31663	41633	5644	12115
24114	35055	1717	5396	16505	24697	1325	3427	28296	41644	5611	12295
24030	35060	1728	5397	16721	24698	1340	3427	28257	41641	5654	12294
21111	35053	1704	5394	14603	24692	1329	3427	24718	41639	5646	12293
23847	35055	1775	5396	16912	24691	1371	3428	28880	41641	5777	12293
29832	35054	1847	5397	20911	24687	1391	3427	35486	41636	5820	12293
28881	34871	1723	5375	20167	24525	1362	3414	34241	41393	5641	12236
29152	34990	1710	5385	20353	24623	1338	3422	34639	41547	5614	12275
26506	33651	1686	5301	18578	23582	1328	3378	31644	39913	5368	11989
27874	34735	1732	5366	19611	24444	1330	3404	33598	41232	5641	12207
31014	34778	1826	5369	22075	24541	1398	3412	37781	41438	5821	12250
30412	34794	1734	5368	21462	24502	1345	3409	37293	41415	5635	12238
29066	34062	1745	5225	20885	24140	1315	3353	36759	40852	5509	12066
45600	45682	19516	19559	26994	27030	29149	29254	54088	54141	281605	283404
46242	46425	19595	19657	27131	27192	29287	29498	54170	54259	283048	286439
46053	46442	19463	19658	27006	27185	29193	29507	53991	54273	283065	286648
46109	46495	19449	19664	27000	27209	29167	29522	53962	54336	283419	286930
46129	46525	19378	19673	26949	27207	29154	29531	53942	54353	283106	287023
46123	46532	19419	19675	26953	27220	29193	29533	53918	54366	283275	287090
46035	46546	19364	19680	26946	27215	29091	29538	53851	54376	282722	287174
46099	46554	19414	19682	26935	27222	29128	29542	53831	54384	283093	287193
46057	46549	19380	19677	26896	27220	29069	29542	53795	54383	282585	287180
46069	46550	19410	19683	26950	27221	29113	29540	53806	54380	282922	287183
46057	46554	19383	19682	26943	27221	29124	29543	53790	54381	282890	287196
46042	46549	19384	19680	26941	27216	29100	29538	53723	54377	282637	287174
46003	46549	19428	19681	26940	27216	29115	29542	53831	54373	282843	287159
46049	46549	19440	19681	26928	27220	29141	29536	53795	54380	283315	287181
46036	46547	19379	19682	26893	27221	29071	29534	53724	54379	282650	287182
45983	46550	19352	19680	26866	27220	29026	29539	53686	54378	282462	287164
46065	46540	19435	19671	26928	27216	29170	29526	53802	54374	283278	287114
46077	46549	19434	19680	26971	27218	29135	29540	53791	54376	283385	287165
46102	46548	19418	19679	26929	27216	29110	29543	53841	54379	282962	287173
46064	46552	19357	19678	26931	27219	29111	29540	53754	54377	282897	287167
46012	46549	19357	19680	26925	27219	29089	29540	53726	54376	282648	287168
46064	46552	19360	19679	26915	27214	29110	29542	53797	54374	282848	287149
46022	46553	19429	19682	26929	27218	29096	29540	53760	54382	282932	287182
45929	46541	19338	19675	26828	27208	29033	29530	53550	54364	281723	287104
45963	46550	19381	19683	26926	27222	29050	29539	53655	54381	282402	287155
46046	46517	19419	19664	26952	27207	29122	29522	53838	54340	283080	287032
45966	46510	19363	19658	26928	27198	29048	29519	53700	54288	282508	286994
45935	46504	19350	19644	26896	27185	29066	29507	53634	54270	282603	286962
46028	46492	19380	19631	26911	27176	29154	29501	53712	54207	283101	286927
45909	46482	19354	19632	26881	27166	29054	29501	53720	54197	282399	286916
45871	46448	19301	19612	26874	27161	29036	29490	53619	54164	282120	286811
45863	46366	19326	19590	26915	27151	29038	29473	53608	54099	281909	286625
45778	46305	19349	19572	26916	27144	29088	29461	53645	54083	282045	286474

Lambda_ctrl	47528	C	45696	586	19291	274	26815	295	29006	428	53568	464	281768	4490
Lambda_ctrl	47529	C	45622	571	19243	303	26829	263	28972	440	53471	487	281318	4766

45696	46282	19291	19565	26815	27110	29006	29434	53568	54032	281768	286258
45622	46193	19243	19546	26829	27092	28972	29412	53471	53958	281318	286084

v6.5 wild type cytosines

Number of C's and T's

			v65bs1		v65oxbs1		v65bs2		v65oxbs2		v65bs3		v65oxbs3	
**Chromosome**	**Position**		**C to C**	**C to T**	**C to C**	**C to T**	**C to C**	**C to T**	**C to C**	**C to T**	**C to C**	**C to T**	**C to C**	**C to T**
chr11	3525591	G	0	1054	1	1587	0	676	0	513	0	966	0	769
chr11	3525594	G	0	1059	0	1606	0	679	0	518	0	972	0	776
chr11	3525596	G	1	1061	0	1620	0	685	0	527	0	980	0	779
chr11	3525598	C	5	8587	2	8414	2	6999	0	3124	2	5323	1	7998
chr11	3525599	C	3	10517	3	10246	2	8793	0	3884	2	6646	2	9891
chr11	3525603	C	1	10750	3	10544	0	9005	2	3991	0	6769	0	10113
chr11	3525605	C	0	10950	2	10756	2	9138	0	4064	0	6871	0	10278
chr11	3525606	C	2	11707	2	11767	2	9576	0	4383	1	7226	3	10866
chr11	3525608	G	0	983	0	1471	0	635	0	483	0	920	0	681
chr11	3525609	G	0	1124	0	1709	0	724	0	550	0	1037	1	778
chr11	3525611	G	14	1153	43	1727	9	731	4	560	9	1054	5	795
chr11	3525612	G	6	1167	1	1793	3	738	1	564	2	1064	1	800
chr11	3525615	G	0	1177	2	1803	2	742	1	566	0	1065	39	763
chr11	3525618	G	3	1174	0	1805	4	740	0	567	2	1065	1	801
chr11	3525619	G	2	1171	26	1776	3	739	6	559	2	1064	3	799
chr11	3525620	C	6	14144	4	14805	3	11335	0	5453	2	8536	4	12923
chr11	3525623	G	6	1171	0	1805	0	744	0	567	3	1064	0	803
chr11	3525624	G	3	1174	6	1799	1	743	0	567	0	1067	1	802
chr11	3525628	G	8	1170	1	1802	0	744	1	564	2	1064	2	801
chr11	3525629	C	21	14333	19	14986	40	11359	3	5473	15	8573	17	13039
chr11	3525633	G	4	1171	0	1807	0	744	0	568	1	1067	3	800
chr11	3525635	G	1	1175	4	1803	1	744	3	565	0	1070	1	801
chr11	3525636	C	30	14338	43	14994	23	11379	7	5472	23	8568	25	13045
chr11	3525638	G	19	1159	3	1805	7	739	7	561	9	1062	0	803
chr11	3525639	C	186	14178	27	15007	56	11339	96	5380	62	8527	192	12877
chr11	3525642	G	13	1162	4	1799	4	743	2	565	2	1069	5	795
chr11	3525643	C	7746	6543	8262	6717	6190	5144	2247	3199	4871	3694	6346	6659
chr11	3525644	G	626	534	894	878	401	338	190	368	593	465	428	359
chr11	3525645	C	51	14317	34	15000	18	11379	12	5463	37	8558	34	13033
chr11	3525647	G	12	1164	3	1802	9	737	3	564	4	1065	0	803
chr11	3525649	C	39	14337	15	15032	30	11378	9	5470	12	8587	25	13052
chr11	3525657	C	27	14357	34	15015	15	11396	11	5471	20	8583	21	13060
chr11	3525660	G	0	1182	2	1807	0	751	0	568	0	1073	33	771
chr11	3525663	G	8	1176	4	1805	3	749	2	566	4	1069	3	801
chr11	3525665	G	5	1179	6	1803	2	750	2	567	4	1069	4	799
chr11	3525666	C	36	14350	24	15026	42	11369	15	5470	25	8580	80	13005
chr11	3525668	C	8775	5616	9554	5503	7250	4168	2778	2706	5512	3095	7747	5340
chr11	3525669	G	743	445	1077	730	449	300	321	247	682	391	518	285
chr11	3525670	C	40	14344	32	15013	77	11332	14	5466	21	8578	51	13023
chr11	3525672	C	154	14241	12	15050	21	11398	59	5425	20	8586	51	13040

Number of C's and total read outs

v65bs1		v65oxbs1		v65bs2		v65oxbs2		v65bs3		v65oxbs3	
**C**	**Total**	**C**	**Total**	**C**	**Total**	**C**	**Total**	**C**	**Total**	**C**	**Total**
0	1054	1	1588	0	676	0	513	0	966	0	769
0	1059	0	1606	0	679	0	518	0	972	0	776
1	1062	0	1620	0	685	0	527	0	980	0	779
5	8592	2	8416	2	7001	0	3124	2	5325	1	7999
3	10520	3	10249	2	8795	0	3884	2	6648	2	9893
1	10751	3	10547	0	9005	2	3993	0	6769	0	10113
0	10950	2	10758	2	9140	0	4064	0	6871	0	10278
2	11709	2	11769	2	9578	0	4383	1	7227	3	10869
0	983	0	1471	0	635	0	483	0	920	0	681
0	1124	0	1709	0	724	0	550	0	1037	1	779
14	1167	43	1770	9	740	4	564	9	1063	5	800
6	1173	1	1794	3	741	1	565	2	1066	1	801
0	1177	2	1805	2	744	1	567	0	1065	39	802
3	1177	0	1805	4	744	0	567	2	1067	1	802
2	1173	26	1802	3	742	6	565	2	1066	3	802
6	14150	4	14809	3	11338	0	5453	2	8538	4	12927
6	1177	0	1805	0	744	0	567	3	1067	0	803
3	1177	6	1805	1	744	0	567	0	1067	1	803
8	1178	1	1803	0	744	1	565	2	1066	2	803
21	14354	19	15005	40	11399	3	5476	15	8588	17	13056
4	1175	0	1807	0	744	0	568	1	1068	3	803
1	1176	4	1807	1	745	3	568	0	1070	1	802
30	14368	43	15037	23	11402	7	5479	23	8591	25	13070
19	1178	3	1808	7	746	7	568	9	1071	0	803
186	14364	27	15034	56	11395	96	5476	62	8589	192	13069
13	1175	4	1803	4	747	2	567	2	1071	5	800
7746	14289	8262	14979	6190	11334	2247	5446	4871	8565	6346	13005
626	1160	894	1772	401	739	190	558	593	1058	428	787
51	14368	34	15034	18	11397	12	5475	37	8595	34	13067
12	1176	3	1805	9	746	3	567	4	1069	0	803
39	14376	15	15047	30	11408	9	5479	12	8599	25	13077
27	14384	34	15049	15	11411	11	5482	20	8603	21	13081
0	1182	2	1809	0	751	0	568	0	1073	33	804
8	1184	4	1809	3	752	2	568	4	1073	3	804
5	1184	6	1809	2	752	2	569	4	1073	4	803
36	14386	24	15050	42	11411	15	5485	25	8605	80	13085
8775	14391	9554	15057	7250	11418	2778	5484	5512	8607	7747	13087
743	1188	1077	1807	449	749	321	568	682	1073	518	803
40	14384	32	15045	77	11409	14	5480	21	8599	51	13074
154	14395	12	15062	21	11419	59	5484	20	8606	51	13091

chr11	3525674	C	6687	7708	7261	7800	5382	6039	2115	3371	4150	4459	5594	7497
chr11	3525675	G	529	655	827	979	365	382	224	342	554	516	403	397
chr11	3525678	C	64	14332	16	15047	33	11390	8	5478	22	8586	133	12959
chr11	3525680	C	9861	4536	9407	5652	7874	3549	2966	2519	5698	2911	8332	4758
chr11	3525681	G	764	423	1183	627	520	230	370	200	758	316	522	281
chr11	3525686	C	16	14384	15	15042	36	11387	5	5483	15	8598	23	13073
chr11	3525688	G	4	1185	5	1810	4	748	1	569	1	1074	1	803
chr11	3525689	G	2	1187	4	1812	1	751	0	570	1	1074	4	800
chr11	3525692	C	21	14389	62	15004	26	11407	12	5476	45	8575	21	13071
chr11	3525693	C	59	14354	13	15060	35	11396	15	5477	37	8587	80	13024
chr11	3525695	G	3	1189	1	1816	1	752	1	572	1	1074	1	804
chr11	3525696	G	3	1189	2	1815	0	754	1	571	2	1073	2	803
chr11	3525699	C	22	14394	21	15058	17	11418	5	5487	8	8619	71	13032
chr11	3525702	G	3	1189	0	1814	1	753	0	573	1	1075	4	802
chr11	3525703	G	3	1190	0	1817	1	753	3	571	1	1074	1	805
chr11	3525705	C	79	14342	36	15047	13	11427	83	5411	34	8592	32	13076
chr11	3525708	G	12	1179	4	1814	4	750	4	571	14	1061	7	799
chr11	3525710	C	27	14393	15	15071	18	11424	9	5485	8	8627	19	13093
chr11	3525711	C	211	14212	319	14764	91	11349	72	5424	70	8568	58	13053
chr11	3525713	G	2	1190	1	1816	2	753	0	575	2	1074	0	807
chr11	3525714	G	5	1189	2	1816	0	755	1	574	0	1076	1	806
chr11	3525716	C	7433	6993	8214	6872	6042	5401	2485	3010	4389	4249	6404	6711
chr11	3525717	G	723	471	969	849	440	316	284	290	662	414	575	230
chr11	3525718	G	1	1194	1	1817	0	755	2	573	4	1071	3	804
chr11	3525719	G	4	1190	2	1814	5	751	0	575	6	1070	2	804
chr11	3525720	C	42	14347	23	15025	31	11383	7	5482	45	8578	90	12997
chr11	3525722	G	2	1193	20	1798	1	754	1	575	1	1076	1	805
chr11	3525723	C	89	14340	22	15065	29	11418	15	5481	12	8624	20	13099
chr11	3525725	G	0	1197	1	1818	0	755	0	575	3	1075	1	806
chr11	3525726	C	27	8383	20	9081	43	6597	5	3252	18	5156	19	7877
chr11	3525729	C	97	14324	251	14828	82	11353	8	5483	86	8553	116	12994
chr11	3525731	C	18	14421	26	15070	22	11428	9	5488	15	8628	36	13086
chr11	3525732	C	50	14389	32	15063	10	11442	43	5453	27	8614	35	13087
chr11	3525733	C	27	14411	20	15077	17	11435	14	5484	11	8635	19	13103
chr11	3525734	C	28	14413	30	15067	13	11439	23	5474	11	8635	40	13080
chr11	3525735	C	10	14430	15	15074	14	11435	5	5493	15	8630	21	13099
chr11	3525738	G	2	505	0	654	0	330	0	227	5	403	3	261
chr11	3525742	C	11114	3289	10188	4883	8474	2958	3931	1559	6627	2003	9984	3105
chr11	3525743	G	251	127	317	95	180	67	98	70	231	64	117	46
chr11	3525744	C	44	14361	39	15004	53	11375	113	5367	26	8599	36	13052
chr11	3525746	C	18	14427	21	15079	12	11445	10	5490	12	8637	21	13103
chr11	3525753	C	3	4088	6	5030	5	2788	0	1637	13	2153	3	3346
chr11	3525755	C	5	3974	5	4898	3	2703	2	1592	3	2102	8	3224
chr11	3525756	C	1778	2151	1993	2861	1236	1422	707	859	924	1160	1344	1828
chr11	3525757	G	149	192	132	179	93	142	51	87	125	149	88	101
chr11	3525758	G	0	342	0	312	0	239	0	137	1	273	1	187

6687	14395	7261	15061	5382	11421	2115	5486	4150	8609	5594	13091
529	1184	827	1806	365	747	224	566	554	1070	403	800
64	14396	16	15063	33	11423	8	5486	22	8608	133	13092
9861	14397	9407	15059	7874	11423	2966	5485	5698	8609	8332	13090
764	1187	1183	1810	520	750	370	570	758	1074	522	803
16	14400	15	15057	36	11423	5	5488	15	8613	23	13096
4	1189	5	1815	4	752	1	570	1	1075	1	804
2	1189	4	1816	1	752	0	570	1	1075	4	804
21	14410	62	15066	26	11433	12	5488	45	8620	21	13092
59	14413	13	15073	35	11431	15	5492	37	8624	80	13104
3	1192	1	1817	1	753	1	573	1	1075	1	805
3	1192	2	1817	0	754	1	572	2	1075	2	805
22	14416	21	15079	17	11435	5	5492	8	8627	71	13103
3	1192	0	1814	1	754	0	573	1	1076	4	806
3	1193	0	1817	1	754	3	574	1	1075	1	806
79	14421	36	15083	13	11440	83	5494	34	8626	32	13108
12	1191	4	1818	4	754	4	575	14	1075	7	806
27	14420	15	15086	18	11442	9	5494	8	8635	19	13112
211	14423	319	15083	91	11440	72	5496	70	8638	58	13111
2	1192	1	1817	2	755	0	575	2	1076	0	807
5	1194	2	1818	0	755	1	575	0	1076	1	807
7433	14426	8214	15086	6042	11443	2485	5495	4389	8638	6404	13115
723	1194	969	1818	440	756	284	574	662	1076	575	805
1	1195	1	1818	0	755	2	575	4	1075	3	807
4	1194	2	1816	5	756	0	575	6	1076	2	806
42	14389	23	15048	31	11414	7	5489	45	8623	90	13087
2	1195	20	1818	1	755	1	576	1	1077	1	806
89	14429	22	15087	29	11447	15	5496	12	8636	20	13119
0	1197	1	1819	0	755	0	575	3	1078	1	807
27	8410	20	9101	43	6640	5	3257	18	5174	19	7896
97	14421	251	15079	82	11435	8	5491	86	8639	116	13110
18	14439	26	15096	22	11450	9	5497	15	8643	36	13122
50	14439	32	15095	10	11452	43	5496	27	8641	35	13122
27	14438	20	15097	17	11452	14	5498	11	8646	19	13122
28	14441	30	15097	13	11452	23	5497	11	8646	40	13120
10	14440	15	15089	14	11449	5	5498	15	8645	21	13120
2	507	0	654	0	330	0	227	5	408	3	264
11114	14403	10188	15071	8474	11432	3931	5490	6627	8630	9984	13089
251	378	317	412	180	247	98	168	231	295	117	163
44	14405	39	15043	53	11428	113	5480	26	8625	36	13088
18	14445	21	15100	12	11457	10	5500	12	8649	21	13124
3	4091	6	5036	5	2793	0	1637	13	2166	3	3349
5	3979	5	4903	3	2706	2	1594	3	2105	8	3232
1778	3929	1993	4854	1236	2658	707	1566	924	2084	1344	3172
149	341	132	311	93	235	51	138	125	274	88	189
0	342	0	312	0	239	0	137	1	274	1	188

chr11	3525759	G	0	334	1	315	0	232	0	137	0	270	0	188
chr11	3525762	C	7	1815	2	2294	1	1188	1	678	1	936	2	1266
chr11	3525765	G	2	544	2	611	1	374	0	234	1	456	0	366
chr11	3525766	G	1	566	0	633	2	383	0	240	0	466	1	376
chr11	3525768	G	2	581	0	665	1	393	2	247	3	482	1	395
chr11	3525769	G	1	590	1	681	1	400	0	254	3	496	1	401
chr11	3525770	G	0	604	0	694	2	410	1	260	0	501	1	414
chr11	3525771	G	1	616	1	742	0	424	1	271	0	521	0	432
chr11	3525774	C	104	14358	38	15047	65	11415	11	5504	58	8598	19	13101
chr11	3525776	G	1	889	2	1284	0	591	0	445	1	826	1	665
chr11	3525777	C	79	14387	81	15021	64	11418	10	5507	28	8616	80	13045
chr11	3525779	C	41	14444	39	15069	26	11466	12	5512	17	8638	25	13109
chr11	3525780	C	36	14450	25	15090	13	11477	5	5518	23	8629	20	13121
chr11	3525781	C	7371	7109	7633	7476	5716	5774	2527	2997	4271	4378	6540	6600
chr11	3525782	G	483	498	637	763	307	325	220	263	475	448	440	267
chr11	3525783	G	1	979	2	1401	2	630	0	483	0	921	4	703
chr11	3525786	G	4	977	6	1401	1	642	1	488	2	924	2	708
chr11	3525788	G	1	980	1	1409	2	641	0	490	1	929	3	709
chr11	3525789	C	21	14455	32	15071	20	11465	12	5505	13	8630	25	13109
chr11	3525790	C	7386	7089	7856	7242	6184	5289	2609	2908	4494	4141	6792	6329
chr11	3525791	G	517	454	799	621	336	303	224	263	513	419	445	275
chr11	3525792	G	1	975	1	1436	1	640	1	490	0	933	1	723
chr11	3525793	G	5	968	3	1435	3	633	1	482	3	932	2	723
chr11	3525794	C	43	14405	36	15038	84	11375	3	5511	52	8574	14	13105
chr11	3525797	C	24	14434	28	15056	20	11437	5	5503	5	8629	25	13094
chr11	3525800	C	23	14436	119	14965	25	11430	7	5503	19	8616	24	13094
chr11	3525801	C	417	14046	538	14546	430	11026	81	5428	299	8337	141	12978
chr11	3525803	C	144	14282	31	15013	217	11213	124	5378	86	8521	258	12834
chr11	3525805	C	64	14385	31	15040	33	11415	40	5465	25	8602	59	13049
chr11	3525807	C	36	14409	33	15046	21	11426	12	5494	8	8617	29	13075
chr11	3525809	G	6	960	5	1435	0	610	1	467	5	912	1	719
chr11	3525812	C	26	14419	74	14993	12	11435	6	5499	10	8614	26	13078
chr11	3525816	G	0	962	1	1433	1	604	1	462	0	914	4	712
chr11	3525817	C	22	14413	13	15045	37	11408	10	5494	18	8598	25	13062
chr11	3525819	C	14	14422	40	15028	23	11423	5	5500	15	8603	24	13067
chr11	3525820	C	21	14413	16	15050	62	11382	5	5501	11	8609	29	13068
chr11	3525821	C	9022	5407	9175	5887	7175	4264	3243	2261	5529	3091	8218	4871
chr11	3525822	G	604	349	993	438	379	217	282	181	579	331	502	211
chr11	3525823	C	8978	5447	9037	6016	6804	4634	3028	2473	5389	3223	7606	5474
chr11	3525824	G	660	427	1043	681	409	247	306	203	632	357	499	258
chr11	3525825	C	31	14390	26	15041	38	11396	9	5494	33	8586	126	12956
chr11	3525827	G	6	1071	24	1696	2	648	1	508	9	969	4	745
chr11	3525828	C	68	14360	22	15038	39	11400	10	5494	32	8583	31	13056
chr11	3525831	G	8	1066	16	1700	9	638	0	505	4	971	6	742
chr11	3525834	G	2	1070	7	1708	2	645	0	507	2	974	1	746
chr11	3525835	G	2	1069	1	1713	1	645	0	505	2	971	1	746

0	334	1	316	0	232	0	137	0	270	0	188
7	1822	2	2296	1	1189	1	679	1	937	2	1268
2	546	2	613	1	375	0	234	1	457	0	366
1	567	0	633	2	385	0	240	0	466	1	377
2	583	0	665	1	394	2	249	3	485	1	396
1	591	1	682	1	401	0	254	3	499	1	402
0	604	0	694	2	412	1	261	0	501	1	415
1	617	1	743	0	424	1	272	0	521	0	432
104	14462	38	15085	65	11480	11	5515	58	8656	19	13120
1	890	2	1286	0	591	0	445	1	827	1	666
79	14466	81	15102	64	11482	10	5517	28	8644	80	13125
41	14485	39	15108	26	11492	12	5524	17	8655	25	13134
36	14486	25	15115	13	11490	5	5523	23	8652	20	13141
7371	14480	7633	15109	5716	11490	2527	5524	4271	8649	6540	13140
483	981	637	1400	307	632	220	483	475	923	440	707
1	980	2	1403	2	632	0	483	0	921	4	707
4	981	6	1407	1	643	1	489	2	926	2	710
1	981	1	1410	2	643	0	490	1	930	3	712
21	14476	32	15103	20	11485	12	5517	13	8643	25	13134
7386	14475	7856	15098	6184	11473	2609	5517	4494	8635	6792	13121
517	971	799	1420	336	639	224	487	513	932	445	720
1	976	1	1437	1	641	1	491	0	933	1	724
5	973	3	1438	3	636	1	483	3	935	2	725
43	14448	36	15074	84	11459	3	5514	52	8626	14	13119
24	14458	28	15084	20	11457	5	5508	5	8634	25	13119
23	14459	119	15084	25	11455	7	5510	19	8635	24	13118
417	14463	538	15084	430	11456	81	5509	299	8636	141	13119
144	14426	31	15044	217	11430	124	5502	86	8607	258	13092
64	14449	31	15071	33	11448	40	5505	25	8627	59	13108
36	14445	33	15079	21	11447	12	5506	8	8625	29	13104
6	966	5	1440	0	610	1	468	5	917	1	720
26	14445	74	15067	12	11447	6	5505	10	8624	26	13104
0	962	1	1434	1	605	1	463	0	914	4	716
22	14435	13	15058	37	11445	10	5504	18	8616	25	13087
14	14436	40	15068	23	11446	5	5505	15	8618	24	13091
21	14434	16	15066	62	11444	5	5506	11	8620	29	13097
9022	14429	9175	15062	7175	11439	3243	5504	5529	8620	8218	13089
604	953	993	1431	379	596	282	463	579	910	502	713
8978	14425	9037	15053	6804	11438	3028	5501	5389	8612	7606	13080
660	1087	1043	1724	409	656	306	509	632	989	499	757
31	14421	26	15067	38	11434	9	5503	33	8619	126	13082
6	1077	24	1720	2	650	1	509	9	978	4	749
68	14428	22	15060	39	11439	10	5504	32	8615	31	13087
8	1074	16	1716	9	647	0	505	4	975	6	748
2	1072	7	1715	2	647	0	507	2	976	1	747
2	1071	1	1714	1	646	0	505	2	973	1	747

chr11	3525837	C	43	14385	29	15031	15	11419	11	5491	13	8597	35	13055
chr11	3525840	C	23	14400	22	15037	8	11422	2	5500	44	8563	16	13070
chr11	3525842	C	8014	6402	6342	8712	6299	5123	2957	2543	4738	3864	7158	5923
chr11	3525843	G	620	437	1022	682	385	255	233	264	603	362	493	247
chr11	3525844	G	3	1053	3	1698	0	639	1	497	2	964	1	739
chr11	3525846	C	26	14392	33	15025	35	11388	43	5454	21	8584	42	13039
chr11	3525848	G	9	1041	39	1656	5	630	0	495	12	945	7	725
chr11	3525849	C	53	14366	16	15041	35	11390	4	5497	63	8538	9	13072
chr11	3525852	G	0	1046	4	1689	0	633	0	494	0	958	8	722
chr11	3525853	G	4	1039	1	1691	0	633	0	493	1	955	1	729
chr11	3525854	C	11793	2598	11367	3657	9197	2220	4097	1396	6946	1645	10441	2621
chr11	3525855	G	837	204	1275	414	503	130	382	113	769	187	628	102
chr11	3525856	C	10369	4041	10156	4878	7980	3438	3953	1539	6279	2314	9033	4035
chr11	3525857	G	737	299	1173	510	480	147	336	156	671	269	567	155
chr11	3525858	C	8447	5711	7350	7487	6477	4815	2993	2415	5118	3335	6946	5882
chr11	3525859	G	613	422	988	691	363	262	262	229	541	397	476	244
chr11	3525860	G	4	1029	2	1676	3	620	0	489	1	937	1	719
chr11	3525861	G	0	1033	0	1678	0	621	0	489	0	936	1	719
chr11	3525862	G	1	1031	2	1675	1	620	1	488	2	932	2	718
chr11	3525863	G	0	1031	2	1674	2	619	0	489	0	934	2	718
chr11	3525865	C	66	14331	36	14992	24	11372	11	5476	23	8552	33	13026
chr11	3525866	C	18	14376	22	15011	20	11370	12	5479	12	8562	22	13034
chr11	3525867	C	8278	6095	7742	7260	6534	4848	2589	2898	4860	3702	6673	6363
chr11	3525868	G	553	453	932	734	322	284	279	207	536	395	492	221
chr11	3525870	G	1	1005	2	1669	1	610	0	487	2	924	3	706
chr11	3525872	C	110	14275	7	15023	95	11288	83	5406	37	8516	113	12923
chr11	3525874	G	1	995	1	1667	0	608	5	478	0	919	9	697
chr11	3525876	C	5098	8884	5052	9596	4198	6783	1803	3558	2970	5325	4443	8207
chr11	3525877	G	399	586	683	974	229	367	148	331	325	589	323	376
chr11	3525879	C	20	14347	25	14982	18	11323	8	5472	12	8507	24	12963
chr11	3525880	C	39	14328	20	14994	18	11326	13	5467	10	8513	22	12964
chr11	3525882	G	3	971	1	1651	4	578	1	467	3	901	1	690
chr11	3525884	C	26	14332	19	14980	20	11309	6	5468	11	8494	32	12937
chr11	3525887	C	6650	7688	5722	9257	4731	6586	2184	3288	3698	4789	5815	7138
chr11	3525888	G	420	544	744	898	230	345	151	314	387	502	380	302
chr11	3525890	C	3933	10393	3288	11678	2741	8573	1563	3911	2065	6415	3260	9688
chr11	3525891	G	207	753	418	1219	132	441	69	396	215	673	222	455
chr11	3525893	C	7573	6656	6890	7977	6031	5218	2474	2979	4333	4096	6276	6598
chr11	3525894	G	469	482	861	772	283	286	191	270	440	441	427	245
chr11	3525897	C	128	14038	26	14745	119	11100	118	5313	59	8353	9	12840
chr11	3525899	C	51	14064	21	14615	46	11127	4	5401	22	8360	5	12787
chr11	3525902	C	2	14090	5	14589	3	11195	1	5407	3	8393	5	12822
chr11	3525903	C	3	14086	3	14591	1	11194	0	5410	0	8395	2	12825
chr11	3525905	G	12	905	16	1582	1	542	11	440	13	819	2	639
chr11	3525907	G	2	885	2	1564	2	514	0	436	1	816	0	616
chr11	3525909	C	5	13517	3	13797	3	10904	2	5201	2	8183	7	12505

43	14428	29	15060	15	11434	11	5502	13	8610	35	13090
23	14423	22	15059	8	11430	2	5502	44	8607	16	13086
8014	14416	6342	15054	6299	11422	2957	5500	4738	8602	7158	13081
620	1057	1022	1704	385	640	233	497	603	965	493	740
3	1056	3	1701	0	639	1	498	2	966	1	740
26	14418	33	15058	35	11423	43	5497	21	8605	42	13081
9	1050	39	1695	5	635	0	495	12	957	7	732
53	14419	16	15057	35	11425	4	5501	63	8601	9	13081
0	1046	4	1693	0	633	0	494	0	958	8	730
4	1043	1	1692	0	633	0	493	1	956	1	730
11793	14391	11367	15024	9197	11417	4097	5493	6946	8591	10441	13062
837	1041	1275	1689	503	633	382	495	769	956	628	730
10369	14410	10156	15034	7980	11418	3953	5492	6279	8593	9033	13068
737	1036	1173	1683	480	627	336	492	671	940	567	722
8447	14158	7350	14837	6477	11292	2993	5408	5118	8453	6946	12828
613	1035	988	1679	363	625	262	491	541	938	476	720
4	1033	2	1678	3	623	0	489	1	938	1	720
0	1033	0	1678	0	621	0	489	0	936	1	720
1	1032	2	1677	1	621	1	489	2	934	2	720
0	1031	2	1676	2	621	0	489	0	934	2	720
66	14397	36	15028	24	11396	11	5487	23	8575	33	13059
18	14394	22	15033	20	11390	12	5491	12	8574	22	13056
8278	14373	7742	15002	6534	11382	2589	5487	4860	8562	6673	13036
553	1006	932	1666	322	606	279	486	536	931	492	713
1	1006	2	1671	1	611	0	487	2	926	3	709
110	14385	7	15030	95	11383	83	5489	37	8553	113	13036
1	996	1	1668	0	608	5	483	0	919	9	706
5098	13982	5052	14648	4198	10981	1803	5361	2970	8295	4443	12650
399	985	683	1657	229	596	148	479	325	914	323	699
20	14367	25	15007	18	11341	8	5480	12	8519	24	12987
39	14367	20	15014	18	11344	13	5480	10	8523	22	12986
3	974	1	1652	4	582	1	468	3	904	1	691
26	14358	19	14999	20	11329	6	5474	11	8505	32	12969
6650	14338	5722	14979	4731	11317	2184	5472	3698	8487	5815	12953
420	964	744	1642	230	575	151	465	387	889	380	682
3933	14326	3288	14966	2741	11314	1563	5474	2065	8480	3260	12948
207	960	418	1637	132	573	69	465	215	888	222	677
7573	14229	6890	14867	6031	11249	2474	5453	4333	8429	6276	12874
469	951	861	1633	283	569	191	461	440	881	427	672
128	14166	26	14771	119	11219	118	5431	59	8412	9	12849
51	14115	21	14636	46	11173	4	5405	22	8382	5	12792
2	14092	5	14594	3	11198	1	5408	3	8396	5	12827
3	14089	3	14594	1	11195	0	5410	0	8395	2	12827
12	917	16	1598	1	543	11	451	13	832	2	641
2	887	2	1566	2	516	0	436	1	817	0	616
5	13522	3	13800	3	10907	2	5203	2	8185	7	12512

chr11	3525910	C	2	13325	6	13535	4	10784	1	5124	2	8099	3	12399
chr11	3525912	C	5	13167	5	13333	6	10686	3	5062	1	8030	2	12294
chr11	3525915	C	5	13078	4	13191	4	10638	2	5013	3	7995	5	12233
chr11	3525917	G	5	615	0	1182	3	336	6	312	6	593	0	419
chr11	3525918	G	1	611	0	1161	0	348	1	314	0	585	0	413
chr11	3525923	C	1	12590	1	12564	3	10368	0	4822	4	7795	1	11882
chr11	3525928	G	3	206	1	432	1	91	0	76	0	143	2	92
chr11	3525935	G	5	171	2	385	1	70	0	65	0	109	0	81
chr11	3525948	g	0	159	0	325	0	63	0	55	0	94	0	56
chr11	3525950	g	0	156	0	325	0	63	0	55	0	93	0	55
chr11	3525954	g	0	153	0	321	0	63	0	55	0	92	0	51
chr11	3525958	g	0	152	0	321	0	63	0	54	0	92	0	51
chr11	3525964	g	0	152	0	321	0	63	0	55	0	92	0	51
chr11	3525968	g	0	152	0	320	0	63	0	55	0	92	0	51
chr15	61868624	C	1	1420	2	1715	1	1099	0	894	0	831	0	3035
chr15	61868625	C	0	1441	0	1733	1	1114	0	898	0	840	1	3060
chr15	61868632	C	0	1511	0	1794	0	1151	0	926	1	866	0	3123
chr15	61868634	C	0	1519	1	1802	0	1156	0	932	0	874	0	3141
chr15	61868635	C	0	1522	1	1809	0	1181	1	945	0	895	1	3165
chr15	61868644	C	0	1587	0	1882	1	1301	0	1041	0	983	0	3324
chr15	61868647	C	0	1598	0	1891	0	1313	0	1051	0	999	1	3341
chr15	61868648	C	0	1602	1	1894	0	1315	0	1053	0	1001	0	3346
chr15	61868649	C	1321	337	1453	469	1120	232	815	274	883	138	2680	724
chr15	61868651	C	7	1864	6	2343	2	1361	1	1113	8	1017	13	3391
chr15	61868655	C	3	1885	5	2361	0	1369	1	1120	2	1028	2	3422
chr15	61868656	C	24	1866	1	2366	9	1362	4	1119	11	1022	49	3377
chr15	61868658	C	36	1866	76	2296	52	1327	56	1077	31	1003	167	3273
chr15	61868663	C	5	1908	24	2358	0	1385	4	1140	2	1041	5	3468
chr15	61868665	C	3	1927	3	2385	2	1390	2	1147	0	1049	7	3483
chr15	61868666	C	1298	633	1421	968	1012	380	608	541	780	269	2198	1296
chr15	61868668	G	1	4297	2	5944	0	744	0	1535	0	578	0	1202
chr15	61868672	C	8	1929	5	2390	2	1393	5	1148	0	1051	3	3501
chr15	61868673	C	4	1933	2	2393	0	1393	1	1152	1	1050	6	3498
chr15	61868674	C	6	1930	9	2382	0	1395	1	1151	2	1050	43	3464
chr15	61868676	C	2	1935	3	2391	1	1395	0	1154	5	1047	6	3503
chr15	61868679	G	1	4752	2	6658	1	797	0	1677	0	634	0	1285
chr15	61868680	G	2	4755	6	6655	0	800	1	1675	0	634	0	1285
chr15	61868683	C	1	1936	0	2396	7	1396	1	1155	3	1054	5	3525
chr15	61868685	G	1	4775	1	6692	1	809	1	1689	0	643	1	1295
chr15	61868687	G	0	4783	1	6702	1	813	0	1690	0	648	1	1297
chr15	61868689	C	3	1933	6	2390	2	1401	1	1157	3	1055	8	3522
chr15	61868692	C	4	1934	9	2387	2	1402	1	1158	4	1053	5	3527
chr15	61868693	C	4	1935	11	2385	1	1403	5	1154	5	1054	3	3529
chr15	61868695	C	3	1934	0	2397	12	1392	6	1153	6	1053	42	3491
chr15	61868698	C	1	1938	1	2396	2	1402	4	1155	2	1059	4	3531
chr15	61868702	C	12	1925	2	2395	7	1398	16	1140	10	1052	1	3528

2	13327	6	13541	4	10788	1	5125	2	8101	3	12402
5	13172	5	13338	6	10692	3	5065	1	8031	2	12296
5	13083	4	13195	4	10642	2	5015	3	7998	5	12238
5	620	0	1182	3	339	6	318	6	599	0	419
1	612	0	1161	0	348	1	315	0	585	0	413
1	12591	1	12565	3	10371	0	4822	4	7799	1	11883
3	209	1	433	1	92	0	76	0	143	2	94
5	176	2	387	1	71	0	65	0	109	0	81
0	159	0	325	0	63	0	55	0	94	0	56
0	156	0	325	0	63	0	55	0	93	0	55
0	153	0	321	0	63	0	55	0	92	0	51
0	152	0	321	0	63	0	54	0	92	0	51
0	152	0	321	0	63	0	55	0	92	0	51
0	152	0	320	0	63	0	55	0	92	0	51
1	1421	2	1717	1	1100	0	894	0	831	0	3035
0	1441	0	1733	1	1115	0	898	0	840	1	3061
0	1511	0	1794	0	1151	0	926	1	867	0	3123
0	1519	1	1803	0	1156	0	932	0	874	0	3141
0	1522	1	1810	0	1181	1	946	0	895	1	3166
0	1587	0	1882	1	1302	0	1041	0	983	0	3324
0	1598	0	1891	0	1313	0	1051	0	999	1	3342
0	1602	1	1895	0	1315	0	1053	0	1001	0	3346
1321	1658	1453	1922	1120	1352	815	1089	883	1021	2680	3404
7	1871	6	2349	2	1363	1	1114	8	1025	13	3404
3	1888	5	2366	0	1369	1	1121	2	1030	2	3424
24	1890	1	2367	9	1371	4	1123	11	1033	49	3426
36	1902	76	2372	52	1379	56	1133	31	1034	167	3440
5	1913	24	2382	0	1385	4	1144	2	1043	5	3473
3	1930	3	2388	2	1392	2	1149	0	1049	7	3490
1298	1931	1421	2389	1012	1392	608	1149	780	1049	2198	3494
1	4298	2	5946	0	744	0	1535	0	578	0	1202
8	1937	5	2395	2	1395	5	1153	0	1051	3	3504
4	1937	2	2395	0	1393	1	1153	1	1051	6	3504
6	1936	9	2391	0	1395	1	1152	2	1052	43	3507
2	1937	3	2394	1	1396	0	1154	5	1052	6	3509
1	4753	2	6660	1	798	0	1677	0	634	0	1285
2	4757	6	6661	0	800	1	1676	0	634	0	1285
1	1937	0	2396	7	1403	1	1156	3	1057	5	3530
1	4776	1	6693	1	810	1	1690	0	643	1	1296
0	4783	1	6703	1	814	0	1690	0	648	1	1298
3	1936	6	2396	2	1403	1	1158	3	1058	8	3530
4	1938	9	2396	2	1404	1	1159	4	1057	5	3532
4	1939	11	2396	1	1404	5	1159	5	1059	3	3532
3	1937	0	2397	12	1404	6	1159	6	1059	42	3533
1	1939	1	2397	2	1404	4	1159	2	1061	4	3535
12	1937	2	2397	7	1405	16	1156	10	1062	1	3529

chr15	61868706	C	8	1939	5	2396	9	1455	1	1196	6	1077	12	3542
chr15	61868707	C	4	1951	2	2409	2	1467	6	1198	4	1084	3	3565
chr15	61868708	C	7	1949	2	2409	2	1468	1	1206	1	1089	4	3568
chr15	61868709	C	6	1951	10	2400	3	1466	2	1204	2	1089	9	3564
chr15	61868713	C	3	1958	9	2403	2	1480	1	1210	2	1102	7	3580
chr15	61868715	G	15	4853	26	6801	2	825	1	1712	4	664	4	1311
chr15	61868716	G	28	4838	11	6816	2	826	3	1710	0	668	7	1309
chr15	61868719	G	28	4841	17	6811	10	818	3	1710	5	663	12	1305
chr15	61868722	G	25	4846	14	6819	1	825	4	1710	2	666	1	1311
chr15	61868723	C	7	1957	6	2408	3	1499	1	1221	3	1121	6	3583
chr15	61868725	C	932	1040	1007	1414	720	783	390	835	593	531	1525	2081
chr15	61868726	G	2204	2613	2540	4240	432	386	659	1036	328	337	491	818
chr15	61868729	G	10	4864	12	6824	2	830	3	1711	1	671	7	1314
chr15	61868730	C	4	1970	9	2413	9	1499	4	1229	2	1131	6	3601
chr15	61868731	C	1323	647	1481	941	1032	479	611	623	826	309	1896	1709
chr15	61868732	G	2820	2056	3525	3312	518	313	921	792	419	253	666	654
chr15	61868733	C	1246	724	1373	1049	883	626	602	629	731	403	2086	1519
chr15	61868734	G	2875	1939	3662	3119	570	253	1009	679	452	209	694	615
chr15	61868735	C	5	1968	19	2407	6	1506	4	1234	5	1130	13	3599
chr15	61868738	G	9	4869	7	6835	7	825	7	1707	3	669	4	1319
chr15	61868740	C	1	1973	5	2425	2	1513	1	1237	0	1140	4	3611
chr15	61868746	G	16	4861	5	6834	0	830	3	1711	2	669	4	1322
chr15	61868747	G	11	4867	11	6839	1	840	3	1714	1	679	4	1340
chr15	61868750	C	11	1968	8	2425	8	1511	0	1237	8	1137	8	3614
chr15	61868753	G	11	4873	10	6844	1	839	0	1718	3	677	3	1339
chr15	61868755	G	9	4876	38	6820	4	836	1	1716	0	681	3	1339
chr15	61868757	C	1280	699	1465	975	938	587	623	615	766	381	2008	1613
chr15	61868758	G	2906	1979	3233	3619	515	325	823	895	420	261	665	677
chr15	61868760	G	4	4883	10	6849	2	839	2	1715	0	681	3	1340
chr15	61868761	C	218	1762	285	2152	187	1340	60	1180	138	1012	333	3291
chr15	61868763	C	5	1980	4	2439	6	1535	2	1249	4	1154	8	3628
chr15	61868764	C	6	1978	7	2436	5	1537	6	1245	2	1156	3	3631
chr15	61868766	G	17	4871	10	6850	9	833	0	1718	4	677	1	1342
chr15	61868769	C	1353	630	1568	874	1069	472	695	556	842	315	2212	1420
chr15	61868770	G	3149	1738	3932	2926	601	240	946	772	473	208	726	618
chr15	61868771	G	7	4880	8	6853	1	841	2	1716	0	681	1	1343
chr15	61868773	G	23	4865	11	6848	3	839	1	1717	3	678	5	1339
chr15	61868774	C	9	1975	6	2431	4	1538	4	1245	1	1156	5	3629
chr15	61868776	G	130	4756	91	6764	17	827	42	1675	13	671	30	1319
chr15	61868777	G	11	4876	11	6849	4	839	3	1716	2	680	5	1345
chr15	61868781	G	32	4856	8	6850	2	842	2	1716	1	682	2	1348
chr15	61868786	C	21	1962	45	2395	15	1524	12	1236	18	1140	95	3538
chr15	61868788	G	22	4873	9	6860	4	845	25	1694	0	684	2	1348
chr15	61868789	C	1580	399	1789	646	1226	312	925	321	968	190	2603	1027
chr15	61868790	G	3630	1182	4876	1832	645	194	1211	485	512	154	939	373
chr15	61868795	G	10	4890	17	6857	2	847	2	1716	1	684	1	1351

8	1947	5	2401	9	1464	1	1197	6	1083	12	3554
4	1955	2	2411	2	1469	6	1204	4	1088	3	3568
7	1956	2	2411	2	1470	1	1207	1	1090	4	3572
6	1957	10	2410	3	1469	2	1206	2	1091	9	3573
3	1961	9	2412	2	1482	1	1211	2	1104	7	3587
15	4868	26	6827	2	827	1	1713	4	668	4	1315
28	4866	11	6827	2	828	3	1713	0	668	7	1316
28	4869	17	6828	10	828	3	1713	5	668	12	1317
25	4871	14	6833	1	826	4	1714	2	668	1	1312
7	1964	6	2414	3	1502	1	1222	3	1124	6	3589
932	1972	1007	2421	720	1503	390	1225	593	1124	1525	3606
2204	4817	2540	6780	432	818	659	1695	328	665	491	1309
10	4874	12	6836	2	832	3	1714	1	672	7	1321
4	1974	9	2422	9	1508	4	1233	2	1133	6	3607
1323	1970	1481	2422	1032	1511	611	1234	826	1135	1896	3605
2820	4876	3525	6837	518	831	921	1713	419	672	666	1320
1246	1970	1373	2422	883	1509	602	1231	731	1134	2086	3605
2875	4814	3662	6781	570	823	1009	1688	452	661	694	1309
5	1973	19	2426	6	1512	4	1238	5	1135	13	3612
9	4878	7	6842	7	832	7	1714	3	672	4	1323
1	1974	5	2430	2	1515	1	1238	0	1140	4	3615
16	4877	5	6839	0	830	3	1714	2	671	4	1326
11	4878	11	6850	1	841	3	1717	1	680	4	1344
11	1979	8	2433	8	1519	0	1237	8	1145	8	3622
11	4884	10	6854	1	840	0	1718	3	680	3	1342
9	4885	38	6858	4	840	1	1717	0	681	3	1342
1280	1979	1465	2440	938	1525	623	1238	766	1147	2008	3621
2906	4885	3233	6852	515	840	823	1718	420	681	665	1342
4	4887	10	6859	2	841	2	1717	0	681	3	1343
218	1980	285	2437	187	1527	60	1240	138	1150	333	3624
5	1985	4	2443	6	1541	2	1251	4	1158	8	3636
6	1984	7	2443	5	1542	6	1251	2	1158	3	3634
17	4888	10	6860	9	842	0	1718	4	681	1	1343
1353	1983	1568	2442	1069	1541	695	1251	842	1157	2212	3632
3149	4887	3932	6858	601	841	946	1718	473	681	726	1344
7	4887	8	6861	1	842	2	1718	0	681	1	1344
23	4888	11	6859	3	842	1	1718	3	681	5	1344
9	1984	6	2437	4	1542	4	1249	1	1157	5	3634
130	4886	91	6855	17	844	42	1717	13	684	30	1349
11	4887	11	6860	4	843	3	1719	2	682	5	1350
32	4888	8	6858	2	844	2	1718	1	683	2	1350
21	1983	45	2440	15	1539	12	1248	18	1158	95	3633
22	4895	9	6869	4	849	25	1719	0	684	2	1350
1580	1979	1789	2435	1226	1538	925	1246	968	1158	2603	3630
3630	4812	4876	6708	645	839	1211	1696	512	666	939	1312
10	4900	17	6874	2	849	2	1718	1	685	1	1352

chr15	61868801	C	1027	953	1162	1272	806	731	497	751	644	514	1832	1802
chr15	61868802	G	2358	2542	2883	3995	449	402	681	1039	340	346	534	816
chr15	61868804	G	6	4895	10	6869	3	849	2	1720	3	685	2	1350
chr15	61868805	G	19	4883	10	6866	7	845	2	1720	0	688	3	1348
chr15	61868810	C	1517	460	1734	698	1213	321	819	428	950	207	2780	844
chr15	61868811	G	3360	1549	4514	2373	633	222	1223	505	505	181	894	459
chr15	61868812	C	1245	733	1483	950	1011	524	716	530	810	344	1971	1654
chr15	61868813	G	2955	1958	3646	3241	511	345	968	763	423	265	729	622
chr15	61868815	G	15	4909	21	6876	1	858	1	1734	3	684	3	1351
chr15	61868816	C	8	1953	3	2410	6	1513	4	1231	1	1144	7	3581
chr15	61868817	C	4	1973	3	2434	0	1531	12	1236	1	1156	18	3611
chr15	61868821	C	43	1933	5	2431	7	1523	10	1235	15	1141	78	3547
chr15	61868823	C	150	1827	168	2266	119	1411	48	1199	106	1050	278	3349
chr15	61868825	C	5	1972	4	2431	2	1529	0	1247	4	1152	12	3614
chr15	61868826	C	5	1972	4	2431	7	1522	0	1247	4	1152	25	3602
chr15	61868828	G	1	581	2	811	0	195	0	206	1	174	0	230
chr15	61868829	C	2	1977	3	2433	4	1525	1	1245	4	1153	8	3616
chr15	61868830	C	4	1975	26	2410	2	1529	3	1245	2	1155	7	3621
chr15	61868833	C	1046	929	1197	1238	868	661	580	667	720	435	1981	1643
chr15	61868834	G	323	336	388	587	117	87	87	134	96	83	99	157
chr15	61868837	C	9	1969	6	2426	0	1532	3	1245	5	1152	19	3605
chr15	61868841	G	11	4909	39	6860	7	843	0	1721	1	683	2	1354
chr15	61868842	C	13	1963	3	2432	6	1525	3	1244	9	1148	21	3604
chr15	61868844	G	8	4912	12	6889	1	847	1	1720	3	679	2	1353
chr15	61868846	G	10	4909	12	6888	2	847	3	1718	1	682	3	1352
chr15	61868847	G	3	4914	11	6886	2	847	7	1713	0	683	2	1352
chr15	61868848	C	4	1969	5	2428	7	1523	2	1244	3	1148	5	3613
chr15	61868853	G	31	4871	14	6871	2	828	1	1711	9	655	4	1337
chr15	61868854	C	37	1938	48	2383	20	1508	6	1240	27	1123	77	3542
chr15	61868856	C	3	1971	4	2431	0	1529	3	1241	4	1146	16	3606
chr15	61868861	G	9	4847	2	6847	1	809	0	1682	5	651	0	1332
chr15	61868863	G	19	4855	12	6857	1	814	0	1689	2	657	4	1330
chr15	61868865	G	15	4857	15	6851	1	813	0	1688	4	654	6	1327
chr15	61868866	G	6	4869	8	6852	2	812	5	1683	2	656	3	1330
chr15	61868870	G	24	4847	14	6845	7	802	13	1670	1	653	8	1325
chr15	61868872	G	215	4653	148	6713	42	767	43	1640	17	637	36	1296
chr15	61868878	C	10	1946	8	2387	2	1518	0	1240	4	1143	9	3600
chr15	61868879	C	7	1944	5	2384	0	1520	2	1239	1	1146	7	3601
chr15	61868880	C	7	1938	9	2357	3	1517	3	1238	3	1143	8	3600
chr15	61868882	G	11	4853	6	6843	3	798	1	1678	0	647	0	1320
chr15	61868885	C	1048	792	1074	1153	841	658	511	690	712	427	1680	1911
chr15	61868886	G	2439	2422	3071	3767	424	378	729	950	330	317	552	768
chr15	61868887	G	8	4853	29	6814	5	799	0	1680	2	646	2	1320
chr15	61868889	C	0	1621	0	2177	0	1194	0	1053	0	971	1	3126
chr15	61868890	C	0	1618	0	2173	0	1195	0	1052	0	971	1	3125
chr15	61868893	G	7	4845	12	6824	1	797	7	1671	1	645	3	1315

1027	1980	1162	2434	806	1537	497	1248	644	1158	1832	3634
2358	4900	2883	6878	449	851	681	1720	340	686	534	1350
6	4901	10	6879	3	852	2	1722	3	688	2	1352
19	4902	10	6876	7	852	2	1722	0	688	3	1351
1517	1977	1734	2432	1213	1534	819	1247	950	1157	2780	3624
3360	4909	4514	6887	633	855	1223	1728	505	686	894	1353
1245	1978	1483	2433	1011	1535	716	1246	810	1154	1971	3625
2955	4913	3646	6887	511	856	968	1731	423	688	729	1351
15	4924	21	6897	1	859	1	1735	3	687	3	1354
8	1961	3	2413	6	1519	4	1235	1	1145	7	3588
4	1977	3	2437	0	1531	12	1248	1	1157	18	3629
43	1976	5	2436	7	1530	10	1245	15	1156	78	3625
150	1977	168	2434	119	1530	48	1247	106	1156	278	3627
5	1977	4	2435	2	1531	0	1247	4	1156	12	3626
5	1977	4	2435	7	1529	0	1247	4	1156	25	3627
1	582	2	813	0	195	0	206	1	175	0	230
2	1979	3	2436	4	1529	1	1246	4	1157	8	3624
4	1979	26	2436	2	1531	3	1248	2	1157	7	3628
1046	1975	1197	2435	868	1529	580	1247	720	1155	1981	3624
323	659	388	975	117	204	87	221	96	179	99	256
9	1978	6	2432	0	1532	3	1248	5	1157	19	3624
11	4920	39	6899	7	850	0	1721	1	684	2	1356
13	1976	3	2435	6	1531	3	1247	9	1157	21	3625
8	4920	12	6901	1	848	1	1721	3	682	2	1355
10	4919	12	6900	2	849	3	1721	1	683	3	1355
3	4917	11	6897	2	849	7	1720	0	683	2	1354
4	1973	5	2433	7	1530	2	1246	3	1151	5	3618
31	4902	14	6885	2	830	1	1712	9	664	4	1341
37	1975	48	2431	20	1528	6	1246	27	1150	77	3619
3	1974	4	2435	0	1529	3	1244	4	1150	16	3622
9	4856	2	6849	1	810	0	1682	5	656	0	1332
19	4874	12	6869	1	815	0	1689	2	659	4	1334
15	4872	15	6866	1	814	0	1688	4	658	6	1333
6	4875	8	6860	2	814	5	1688	2	658	3	1333
24	4871	14	6859	7	809	13	1683	1	654	8	1333
215	4868	148	6861	42	809	43	1683	17	654	36	1332
10	1956	8	2395	2	1520	0	1240	4	1147	9	3609
7	1951	5	2389	0	1520	2	1241	1	1147	7	3608
7	1945	9	2366	3	1520	3	1241	3	1146	8	3608
11	4864	6	6849	3	801	1	1679	0	647	0	1320
1048	1840	1074	2227	841	1499	511	1201	712	1139	1680	3591
2439	4861	3071	6838	424	802	729	1679	330	647	552	1320
8	4861	29	6843	5	804	0	1680	2	648	2	1322
0	1621	0	2177	0	1194	0	1053	0	971	1	3127
0	1618	0	2173	0	1195	0	1052	0	971	1	3126
7	4852	12	6836	1	798	7	1678	1	646	3	1318

chr15	61868897	G	41	4784	7	6793	0	784	9	1659	1	630	3	1292
chr15	61868898	G	8	4834	14	6815	1	789	4	1668	3	633	1	1300
chr15	61868900	G	14	4828	24	6801	5	781	4	1666	1	633	7	1289
chr15	61868901	G	2	4839	11	6814	0	772	2	1666	1	622	1	1282
chr15	61868904	G	4	4835	15	6801	2	763	0	1664	1	616	1	1269
chr15	61868905	C	1	1462	0	1963	0	1035	0	925	0	824	0	2858
chr15	61868907	G	86	4737	143	6663	12	729	30	1626	17	594	26	1232
chr15	61868908	C	0	1450	0	1928	0	1023	0	910	0	815	0	2840
chr15	61868910	G	8	4826	20	6794	0	760	1	1661	0	613	4	1258
chr15	61868912	C	0	1351	0	1820	0	959	1	853	0	733	0	2665
chr15	61868914	G	17	4816	43	6767	5	734	1	1656	1	610	8	1249
chr15	61868915	G	12	4819	8	6802	1	738	8	1649	1	610	1	1256
chr15	61868917	G	42	4789	22	6789	6	733	15	1641	10	599	4	1250
chr15	61868918	G	7	4823	28	6780	0	738	0	1656	1	609	3	1250
chr15	61868929	G	5	4815	12	6794	2	736	1	1651	4	605	5	1247
chr15	61868933	G	6	4816	6	6796	2	735	1	1654	0	607	3	1247
chr15	61868935	G	9	4814	15	6787	4	734	8	1647	1	605	1	1250
chr15	61868939	G	7	4814	9	6791	1	734	1	1652	1	603	1	1249
chr15	61868944	G	3	4808	6	6787	3	732	1	1651	0	603	3	1244
chr15	61868948	G	2770	2042	2735	4061	500	234	645	1007	343	260	643	604
chr15	61868961	G	2	4692	1	6584	0	732	1	1642	0	597	0	1242
chr15	61868969	G	1	4624	1	6493	0	722	0	1626	0	583	0	1228
chr15	61868972	G	0	4577	1	6405	1	718	1	1604	0	580	0	1220
chr15	61868975	G	0	4546	0	6353	1	717	0	1598	1	576	1	1213
chr15	61868976	G	2	4536	0	6343	0	716	0	1596	0	570	0	1206
chr15	61868979	G	0	4532	0	6339	1	716	0	1594	0	568	0	1205
chr15	61868980	G	0	4528	0	6333	1	714	0	1594	0	565	0	1207
chr15	100299982	C	0	170	1	158	0	145	0	28	0	120	0	234
chr15	100299985	C	0	216	0	196	0	177	0	35	0	150	0	301
chr15	100299986	C	0	218	0	199	0	176	0	35	0	151	0	304
chr15	100299987	C	1	218	1	198	0	178	0	36	0	153	0	306
chr15	100299991	C	0	230	0	208	0	182	0	39	0	159	1	319
chr15	100299992	C	0	233	0	212	0	183	0	40	0	160	0	322
chr15	100299993	C	0	236	0	212	0	184	0	41	0	161	0	325
chr15	100299995	G	2	4709	3	4917	1	2137	0	1179	1	1543	0	2446
chr15	100299997	G	3	4718	0	4930	0	2141	0	1181	0	1548	0	2446
chr15	100299998	C	0	294	0	254	0	262	0	68	0	206	2	424
chr15	100300001	G	1	4718	0	4923	0	2141	0	1181	0	1546	0	2445
chr15	100300002	G	0	4722	0	4932	0	2143	0	1181	1	1547	0	2447
chr15	100300003	G	2	4717	0	4926	0	2142	0	1178	0	1547	0	2447
chr15	100300008	C	0	304	0	273	0	276	0	71	0	214	1	459
chr15	100300010	G	3	4724	4	4957	7	2138	6	1176	4	1544	4	2442
chr15	100300013	G	6	4723	4	4960	3	2142	0	1182	3	1545	5	2441
chr15	100300015	G	11	4715	9	4953	5	2141	1	1181	2	1547	7	2440
chr15	100300018	C	1	355	0	359	1	275	0	73	2	213	0	474
chr15	100300020	G	5	4728	65	4904	8	2139	1	1183	3	1549	1	2451

41	4825	7	6800	0	784	9	1668	1	631	3	1295
8	4842	14	6829	1	790	4	1672	3	636	1	1301
14	4842	24	6825	5	786	4	1670	1	634	7	1296
2	4841	11	6825	0	772	2	1668	1	623	1	1283
4	4839	15	6816	2	765	0	1664	1	617	1	1270
1	1463	0	1963	0	1035	0	925	0	824	0	2858
86	4823	143	6806	12	741	30	1656	17	611	26	1258
0	1450	0	1928	0	1023	0	910	0	815	0	2840
8	4834	20	6814	0	760	1	1662	0	613	4	1262
0	1351	0	1820	0	959	1	854	0	733	0	2665
17	4833	43	6810	5	739	1	1657	1	611	8	1257
12	4831	8	6810	1	739	8	1657	1	611	1	1257
42	4831	22	6811	6	739	15	1656	10	609	4	1254
7	4830	28	6808	0	738	0	1656	1	610	3	1253
5	4820	12	6806	2	738	1	1652	4	609	5	1252
6	4822	6	6802	2	737	1	1655	0	607	3	1250
9	4823	15	6802	4	738	8	1655	1	606	1	1251
7	4821	9	6800	1	735	1	1653	1	604	1	1250
3	4811	6	6793	3	735	1	1652	0	603	3	1247
2770	4812	2735	6796	500	734	645	1652	343	603	643	1247
2	4694	1	6585	0	732	1	1643	0	597	0	1242
1	4625	1	6494	0	722	0	1626	0	583	0	1228
0	4577	1	6406	1	719	1	1605	0	580	0	1220
0	4546	0	6353	1	718	0	1598	1	577	1	1214
2	4538	0	6343	0	716	0	1596	0	570	0	1206
0	4532	0	6339	1	717	0	1594	0	568	0	1205
0	4528	0	6333	1	715	0	1594	0	565	0	1207
0	170	1	159	0	145	0	28	0	120	0	234
0	216	0	196	0	177	0	35	0	150	0	301
0	218	0	199	0	176	0	35	0	151	0	304
1	219	1	199	0	178	0	36	0	153	0	306
0	230	0	208	0	182	0	39	0	159	1	320
0	233	0	212	0	183	0	40	0	160	0	322
0	236	0	212	0	184	0	41	0	161	0	325
2	4711	3	4920	1	2138	0	1179	1	1544	0	2446
3	4721	0	4930	0	2141	0	1181	0	1548	0	2446
0	294	0	254	0	262	0	68	0	206	2	426
1	4719	0	4923	0	2141	0	1181	0	1546	0	2445
0	4722	0	4932	0	2143	0	1181	1	1548	0	2447
2	4719	0	4926	0	2142	0	1178	0	1547	0	2447
0	304	0	273	0	276	0	71	0	214	1	460
3	4727	4	4961	7	2145	6	1182	4	1548	4	2446
6	4729	4	4964	3	2145	0	1182	3	1548	5	2446
11	4726	9	4962	5	2146	1	1182	2	1549	7	2447
1	356	0	359	1	276	0	73	2	215	0	474
5	4733	65	4969	8	2147	1	1184	3	1552	1	2452

chr15	100300021	G	4	4730	12	4956	4	2145	0	1184	4	1548	1	2450
chr15	100300022	C	2	354	0	359	1	275	0	73	0	214	1	472
chr15	100300026	C	46	310	14	345	25	251	4	69	33	182	45	429
chr15	100300027	G	455	4278	568	4404	240	1908	60	1122	185	1367	258	2191
chr15	100300028	C	3	353	2	354	0	275	1	72	2	212	0	473
chr15	100300030	C	1	355	1	358	0	275	0	73	1	214	0	474
chr15	100300032	C	1	355	1	358	0	276	0	73	1	214	0	474
chr15	100300034	G	19	4719	11	4968	1	2150	2	1182	2	1554	26	2424
chr15	100300038	C	4	352	0	359	0	276	1	72	0	215	1	473
chr15	100300039	C	41	315	20	339	25	251	10	63	26	189	46	428
chr15	100300040	G	470	4271	321	4660	267	1884	119	1066	217	1339	218	2240
chr15	100300044	G	7	4738	6	4975	7	2144	6	1178	2	1556	5	2454
chr15	100300049	C	0	356	0	359	0	277	0	73	0	215	0	474
chr15	100300050	C	17	339	1	358	17	260	0	73	21	194	64	410
chr15	100300051	G	231	4511	311	4665	151	1996	66	1119	128	1428	186	2263
chr15	100300053	G	16	4736	7	4982	4	2152	1	1187	4	1562	2	2460
chr15	100300054	G	9	4743	5	4984	10	2145	1	1187	4	1561	3	2458
chr15	100300055	G	7	4746	5	4984	3	2153	1	1187	3	1564	7	2455
chr15	100300056	G	6	4746	9	4980	6	2149	1	1186	2	1565	4	2458
chr15	100300058	C	0	356	0	359	0	277	0	73	0	216	1	474
chr15	100300059	C	0	356	0	358	0	277	0	73	0	216	2	473
chr15	100300062	C	99	257	123	236	83	194	19	54	65	151	142	333
chr15	100300063	G	1232	3527	1264	3727	589	1568	275	912	519	1050	556	1911
chr15	100300066	C	0	356	0	359	0	277	0	73	0	216	1	474
chr15	100300067	C	41	315	25	334	43	234	12	61	34	182	59	416
chr15	100300068	G	625	4139	534	4464	310	1849	159	1027	250	1321	285	2182
chr15	100300072	G	9	4757	3	4995	0	2158	1	1186	3	1567	3	2463
chr15	100300073	G	3	4761	10	4984	8	2151	3	1184	4	1566	2	2466
chr15	100300074	C	1	353	2	355	0	276	0	73	0	213	0	473
chr15	100300077	C	0	356	0	359	0	277	0	73	0	216	2	473
chr15	100300080	C	0	355	24	334	0	275	0	73	0	214	0	471
chr15	100300082	C	1	355	1	358	0	277	0	73	0	216	0	475
chr15	100300083	C	1	355	2	357	0	277	0	73	0	216	2	473
chr15	100300086	C	142	214	164	195	108	169	27	46	91	125	149	326
chr15	100300087	G	1857	2913	1860	3141	820	1341	414	775	720	853	990	1478
chr15	100300088	C	0	356	0	359	0	276	0	73	0	216	2	473
chr15	100300090	G	6	4767	7	4992	6	2156	1	1189	3	1570	7	2462
chr15	100300094	G	7	4764	5	4996	1	2160	3	1187	4	1569	1	2469
chr15	100300096	C	2	355	1	358	0	278	0	73	0	216	4	471
chr15	100300098	C	0	357	1	358	0	278	0	73	0	216	0	475
chr15	100300102	G	4	4770	5	4998	2	2161	2	1188	6	1568	1	2468
chr15	100300103	G	11	4763	35	4967	6	2156	1	1189	2	1572	8	2462
chr15	100300105	C	3	354	0	359	0	278	0	73	1	216	0	474
chr15	100300108	C	90	267	130	229	85	193	20	52	46	170	108	366
chr15	100300109	G	1407	3367	1367	3639	644	1521	245	945	562	1012	601	1869
chr15	100300112	G	6	4768	9	5000	1	2164	0	1189	1	1572	0	2471

4	4734	12	4968	4	2149	0	1184	4	1552	1	2451
2	356	0	359	1	276	0	73	0	214	1	473
46	356	14	359	25	276	4	73	33	215	45	474
455	4733	568	4972	240	2148	60	1182	185	1552	258	2449
3	356	2	356	0	275	1	73	2	214	0	473
1	356	1	359	0	275	0	73	1	215	0	474
1	356	1	359	0	276	0	73	1	215	0	474
19	4738	11	4979	1	2151	2	1184	2	1556	26	2450
4	356	0	359	0	276	1	73	0	215	1	474
41	356	20	359	25	276	10	73	26	215	46	474
470	4741	321	4981	267	2151	119	1185	217	1556	218	2458
7	4745	6	4981	7	2151	6	1184	2	1558	5	2459
0	356	0	359	0	277	0	73	0	215	0	474
17	356	1	359	17	277	0	73	21	215	64	474
231	4742	311	4976	151	2147	66	1185	128	1556	186	2449
16	4752	7	4989	4	2156	1	1188	4	1566	2	2462
9	4752	5	4989	10	2155	1	1188	4	1565	3	2461
7	4753	5	4989	3	2156	1	1188	3	1567	7	2462
6	4752	9	4989	6	2155	1	1187	2	1567	4	2462
0	356	0	359	0	277	0	73	0	216	1	475
0	356	0	358	0	277	0	73	0	216	2	475
99	356	123	359	83	277	19	73	65	216	142	475
1232	4759	1264	4991	589	2157	275	1187	519	1569	556	2467
0	356	0	359	0	277	0	73	0	216	1	475
41	356	25	359	43	277	12	73	34	216	59	475
625	4764	534	4998	310	2159	159	1186	250	1571	285	2467
9	4766	3	4998	0	2158	1	1187	3	1570	3	2466
3	4764	10	4994	8	2159	3	1187	4	1570	2	2468
1	354	2	357	0	276	0	73	0	213	0	473
0	356	0	359	0	277	0	73	0	216	2	475
0	355	24	358	0	275	0	73	0	214	0	471
1	356	1	359	0	277	0	73	0	216	0	475
1	356	2	359	0	277	0	73	0	216	2	475
142	356	164	359	108	277	27	73	91	216	149	475
1857	4770	1860	5001	820	2161	414	1189	720	1573	990	2468
0	356	0	359	0	276	0	73	0	216	2	475
6	4773	7	4999	6	2162	1	1190	3	1573	7	2469
7	4771	5	5001	1	2161	3	1190	4	1573	1	2470
2	357	1	359	0	278	0	73	0	216	4	475
0	357	1	359	0	278	0	73	0	216	0	475
4	4774	5	5003	2	2163	2	1190	6	1574	1	2469
11	4774	35	5002	6	2162	1	1190	2	1574	8	2470
3	357	0	359	0	278	0	73	1	217	0	474
90	357	130	359	85	278	20	72	46	216	108	474
1407	4774	1367	5006	644	2165	245	1190	562	1574	601	2470
6	4774	9	5009	1	2165	0	1189	1	1573	0	2471

chr15	100300115	G	11	4766	13	4999	10	2155	1	1189	4	1570	3	2467
chr15	100300116	G	7	4771	13	4998	6	2158	7	1183	3	1571	3	2467
chr15	100300118	C	2	356	0	359	1	277	0	73	0	217	2	473
chr15	100300121	C	0	358	0	359	1	277	0	73	0	217	0	474
chr15	100300125	G	12	4776	18	4991	6	2166	3	1189	8	1564	2	2465
chr15	100300126	C	1	357	0	360	0	278	0	73	1	215	0	475
chr15	100300129	C	7	351	0	360	4	274	0	73	0	218	1	474
chr15	100300131	C	1	351	1	347	1	273	0	71	0	213	0	469
chr15	100300132	C	1	357	0	360	0	278	0	72	0	217	0	474
chr15	100300136	C	1	164	0	193	0	133	0	42	0	103	0	185
chr15	100300138	C	0	165	0	190	1	132	0	41	0	101	0	183
chr15	100300139	C	90	73	89	100	73	60	22	19	37	62	83	99
chr15	100300140	G	74	101	55	106	76	115	33	63	84	97	62	94
chr15	100300143	G	1	170	1	148	1	190	0	92	1	182	2	155
chr15	100300144	G	1	169	0	148	0	191	1	90	0	185	0	156
chr15	100300145	C	92	31	111	51	68	29	25	9	45	30	80	52
chr15	100300146	G	115	56	108	41	116	76	51	41	110	76	102	54
chr15	100300147	G	1	172	0	150	0	190	0	92	3	180	2	156
chr15	100300149	C	39	48	42	93	16	38	3	10	21	30	20	47
chr15	100300150	G	69	95	33	119	65	109	22	64	58	111	48	107
chr15	100300152	G	0	167	1	164	1	173	1	88	1	166	0	155
chr15	100300155	G	0	176	0	266	0	176	0	90	0	168	2	163
chr15	100300161	G	1	433	2	925	0	212	0	110	1	185	0	223
chr15	100300171	G	156	353	314	672	90	191	33	127	76	162	92	180
chr15	100300173	G	6	539	2	1014	0	297	0	168	1	244	0	286
chr15	100300174	G	0	551	3	1031	2	295	0	170	0	246	0	286
chr15	100300176	G	20	679	25	1284	2	344	0	202	8	253	4	314
chr15	100300182	G	1	1221	4	1799	2	595	2	329	1	429	2	576
chr15	100300187	G	9	4788	7	5013	6	2149	15	1173	5	1560	1	2464
chr15	100300189	G	13	4777	18	5000	9	2135	2	1182	5	1553	9	2455
chr15	100300191	G	1916	2845	1960	3042	822	1248	410	736	652	831	998	1430
chr15	100300198	G	1953	2805	1771	3227	856	1213	416	729	681	797	966	1458
chr15	100300199	G	5	4756	10	4988	5	2064	0	1145	3	1477	4	2422
chr15	100300203	G	2180	2578	2127	2871	965	1103	421	724	755	724	994	1430
chr15	100300211	G	647	4099	415	4572	275	1784	88	1055	260	1207	294	2122
chr15	100300218	G	15	4735	4	4987	8	2053	0	1143	6	1462	13	2405
chr15	100300219	G	8	4742	7	4986	2	2059	1	1142	0	1468	7	2412
chr15	100300220	G	7	4741	18	4977	3	2058	1	1142	4	1463	11	2408
chr15	100300221	G	12	4739	7	4986	5	2056	2	1140	1	1464	5	2411
chr15	100300223	G	1024	3726	1244	3749	483	1577	174	969	396	1072	586	1831
chr15	100300235	G	1808	2936	1716	3264	857	1201	380	762	564	900	822	1585
chr15	100300243	G	7	4736	8	4976	3	2052	0	1142	4	1458	4	2403
chr15	100300263	G	22	4680	1	4963	6	2022	0	1130	3	1448	1	2371
chr15	100300267	G	3	4723	11	4972	2	2034	0	1133	5	1449	0	2379
chr15	100300268	G	10	4717	7	4979	1	2035	0	1133	3	1452	4	2374
chr15	100300274	G	1447	3277	1550	3434	694	1339	368	765	617	836	885	1490

11	4777	13	5012	10	2165	1	1190	4	1574	3	2470
7	4778	13	5011	6	2164	7	1190	3	1574	3	2470
2	358	0	359	1	278	0	73	0	217	2	475
0	358	0	359	1	278	0	73	0	217	0	474
12	4788	18	5009	6	2172	3	1192	8	1572	2	2467
1	358	0	360	0	278	0	73	1	216	0	475
7	358	0	360	4	278	0	73	0	218	1	475
1	352	1	348	1	274	0	71	0	213	0	469
1	358	0	360	0	278	0	72	0	217	0	474
1	165	0	193	0	133	0	42	0	103	0	185
0	165	0	190	1	133	0	41	0	101	0	183
90	163	89	189	73	133	22	41	37	99	83	182
74	175	55	161	76	191	33	96	84	181	62	156
1	171	1	149	1	191	0	92	1	183	2	157
1	170	0	148	0	191	1	91	0	185	0	156
92	123	111	162	68	97	25	34	45	75	80	132
115	171	108	149	116	192	51	92	110	186	102	156
1	173	0	150	0	190	0	92	3	183	2	158
39	87	42	135	16	54	3	13	21	51	20	67
69	164	33	152	65	174	22	86	58	169	48	155
0	167	1	165	1	174	1	89	1	167	0	155
0	176	0	266	0	176	0	90	0	168	2	165
1	434	2	927	0	212	0	110	1	186	0	223
156	509	314	986	90	281	33	160	76	238	92	272
6	545	2	1016	0	297	0	168	1	245	0	286
0	551	3	1034	2	297	0	170	0	246	0	286
20	699	25	1309	2	346	0	202	8	261	4	318
1	1222	4	1803	2	597	2	331	1	430	2	578
9	4797	7	5020	6	2155	15	1188	5	1565	1	2465
13	4790	18	5018	9	2144	2	1184	5	1558	9	2464
1916	4761	1960	5002	822	2070	410	1146	652	1483	998	2428
1953	4758	1771	4998	856	2069	416	1145	681	1478	966	2424
5	4761	10	4998	5	2069	0	1145	3	1480	4	2426
2180	4758	2127	4998	965	2068	421	1145	755	1479	994	2424
647	4746	415	4987	275	2059	88	1143	260	1467	294	2416
15	4750	4	4991	8	2061	0	1143	6	1468	13	2418
8	4750	7	4993	2	2061	1	1143	0	1468	7	2419
7	4748	18	4995	3	2061	1	1143	4	1467	11	2419
12	4751	7	4993	5	2061	2	1142	1	1465	5	2416
1024	4750	1244	4993	483	2060	174	1143	396	1468	586	2417
1808	4744	1716	4980	857	2058	380	1142	564	1464	822	2407
7	4743	8	4984	3	2055	0	1142	4	1462	4	2407
22	4702	1	4964	6	2028	0	1130	3	1451	1	2372
3	4726	11	4983	2	2036	0	1133	5	1454	0	2379
10	4727	7	4986	1	2036	0	1133	3	1455	4	2378
1447	4724	1550	4984	694	2033	368	1133	617	1453	885	2375

chr15	100300279	C	0	65	0	120	0	29	0	10	0	24	0	41
chr15	100300283	G	1064	3643	1211	3757	553	1475	223	900	436	1007	621	1738
chr15	100300285	G	15	4702	11	4968	8	2022	2	1125	7	1439	38	2324
chr15	100300288	G	9	4627	13	4910	6	2003	5	1105	6	1409	9	2321
chr15	100300289	C	0	70	0	124	0	35	0	14	0	28	0	54
chr15	100300290	C	0	71	0	124	0	35	0	15	0	28	0	55
chr15	100300291	C	40	32	71	54	15	20	12	3	21	8	31	26
chr15	100300292	G	2326	2391	2812	2167	1127	900	484	641	928	516	1414	945
chr15	100300296	C	0	80	0	133	0	39	0	18	0	31	0	61
chr15	100300298	G	15	4700	5	4970	11	2015	2	1121	6	1435	9	2347
chr15	100300299	C	0	102	1	145	0	47	0	21	3	39	0	91
chr15	100300301	C	0	152	0	176	0	79	0	32	1	60	2	172
chr15	100300304	C	0	172	0	195	0	95	0	38	0	77	1	215
chr15	100300306	G	47	4658	4	4851	16	2005	11	1107	14	1422	40	2303
chr15	100300307	C	121	57	130	68	75	28	27	11	61	18	107	121
chr15	100300308	G	3310	1344	2816	1610	1594	422	745	371	1220	213	1606	722
chr15	100300312	G	1	4442	0	4156	0	1982	0	1099	0	1414	0	2283
chr15	100300314	C	2	356	1	358	0	259	0	73	0	194	21	454
chr15	100300316	C	7	351	29	330	10	249	0	73	8	185	3	471
chr15	100300318	G	0	4401	0	4110	0	1952	1	1088	0	1389	0	2256
chr15	100300320	C	0	357	0	359	0	259	0	73	0	194	0	476
chr15	100300322	G	3	4354	2	4084	0	1910	1	1047	0	1357	0	2226
chr15	100300324	G	0	4322	0	4047	0	1889	0	1039	0	1350	0	2211
chr15	100300326	G	4	4292	1	3993	0	1881	0	1032	0	1347	0	2201
chr15	100300330	G	2	4136	0	3704	1	1837	0	1002	1	1323	1	2159
chr15	100300331	C	3	355	0	359	0	258	0	73	0	194	1	473
chr15	100300338	C	0	358	1	358	1	256	0	73	0	193	1	474
chr15	100300339	C	215	141	196	162	156	101	40	33	143	49	215	259
chr15	100300344	C	1	357	1	357	0	257	0	73	1	192	1	474
chr15	100300345	C	295	62	282	75	211	46	54	18	151	42	334	137
chr15	100300347	C	3	355	0	357	0	257	0	73	2	191	1	473
chr15	100300350	C	0	358	3	355	4	253	0	73	1	190	3	472
chr15	100300353	C	294	60	290	68	216	40	64	9	169	24	320	150
chr15	100300355	C	0	358	1	357	2	252	0	73	0	193	0	473
chr15	100300371	C	0	339	1	340	0	247	0	71	0	184	2	445
chr15	100300374	C	1	353	0	358	0	255	0	73	3	187	24	445
chr15	100300376	C	0	355	1	357	0	254	0	72	0	190	1	468
chr15	100300379	C	1	354	0	358	1	253	0	73	1	189	2	467
chr15	100300380	C	247	108	228	128	156	98	37	36	126	64	222	247
chr15	100300383	C	0	355	1	357	0	253	0	73	0	189	1	468
chr15	100300394	C	2	352	0	356	0	252	0	72	0	187	2	467
chr15	100300402	C	0	353	0	353	0	246	0	68	0	185	1	464
chr15	100300406	C	0	351	1	351	1	245	0	69	0	184	0	465
chr15	100300414	C	7	343	0	348	3	243	0	69	2	182	1	464
chr15	100300416	C	0	350	1	348	1	245	0	69	0	184	13	452
chr15	100300419	C	0	349	0	349	0	244	0	68	0	184	5	457

0	65	0	120	0	29	0	10	0	24	0	41
1064	4707	1211	4968	553	2028	223	1123	436	1443	621	2359
15	4717	11	4979	8	2030	2	1127	7	1446	38	2362
9	4636	13	4923	6	2009	5	1110	6	1415	9	2330
0	70	0	124	0	35	0	14	0	28	0	54
0	71	0	124	0	35	0	15	0	28	0	55
40	72	71	125	15	35	12	15	21	29	31	57
2326	4717	2812	4979	1127	2027	484	1125	928	1444	1414	2359
0	80	0	133	0	39	0	18	0	31	0	61
15	4715	5	4975	11	2026	2	1123	6	1441	9	2356
0	102	1	146	0	47	0	21	3	42	0	91
0	152	0	176	0	79	0	32	1	61	2	174
0	172	0	195	0	95	0	38	0	77	1	216
47	4705	4	4855	16	2021	11	1118	14	1436	40	2343
121	178	130	198	75	103	27	38	61	79	107	228
3310	4654	2816	4426	1594	2016	745	1116	1220	1433	1606	2328
1	4443	0	4156	0	1982	0	1099	0	1414	0	2283
2	358	1	359	0	259	0	73	0	194	21	475
7	358	29	359	10	259	0	73	8	193	3	474
0	4401	0	4110	0	1952	1	1089	0	1389	0	2256
0	357	0	359	0	259	0	73	0	194	0	476
3	4357	2	4086	0	1910	1	1048	0	1357	0	2226
0	4322	0	4047	0	1889	0	1039	0	1350	0	2211
4	4296	1	3994	0	1881	0	1032	0	1347	0	2201
2	4138	0	3704	1	1838	0	1002	1	1324	1	2160
3	358	0	359	0	258	0	73	0	194	1	474
0	358	1	359	1	257	0	73	0	193	1	475
215	356	196	358	156	257	40	73	143	192	215	474
1	358	1	358	0	257	0	73	1	193	1	475
295	357	282	357	211	257	54	72	151	193	334	471
3	358	0	357	0	257	0	73	2	193	1	474
0	358	3	358	4	257	0	73	1	191	3	475
294	354	290	358	216	256	64	73	169	193	320	470
0	358	1	358	2	254	0	73	0	193	0	473
0	339	1	341	0	247	0	71	0	184	2	447
1	354	0	358	0	255	0	73	3	190	24	469
0	355	1	358	0	254	0	72	0	190	1	469
1	355	0	358	1	254	0	73	1	190	2	469
247	355	228	356	156	254	37	73	126	190	222	469
0	355	1	358	0	253	0	73	0	189	1	469
2	354	0	356	0	252	0	72	0	187	2	469
0	353	0	353	0	246	0	68	0	185	1	465
0	351	1	352	1	246	0	69	0	184	0	465
7	350	0	348	3	246	0	69	2	184	1	465
0	350	1	349	1	246	0	69	0	184	13	465
0	349	0	349	0	244	0	68	0	184	5	462

chr15	100300420	C	12	336	3	346	6	237	4	64	8	176	8	454
chr15	100300422	C	1	345	0	347	0	244	0	68	0	183	1	458
chr15	100300423	C	1	347	0	346	0	244	0	68	0	183	2	459
chr15	100300425	C	0	348	0	347	0	244	0	68	0	183	1	460
chr15	100300434	C	0	317	0	260	0	244	0	66	0	182	1	444
chr15	100300435	C	0	317	0	260	0	244	0	66	1	181	0	446
chr15	100300455	C	0	213	0	186	0	179	0	39	0	131	0	279
chr15	100300460	C	0	204	0	182	0	169	0	39	0	127	0	261
chr15	100300461	C	0	202	0	180	0	166	0	35	0	126	0	260
chr16	92644291	G	0	242	0	81	0	143	0	24	0	390	0	553
chr16	92644300	G	0	269	0	87	0	153	0	26	0	418	0	592
chr16	92644309	G	0	377	0	114	0	254	0	39	2	1094	0	1051
chr16	92644311	G	0	378	0	114	0	257	0	39	0	1103	0	1058
chr16	92644318	G	0	471	0	163	0	387	0	50	11	2093	5	1738
chr16	92644322	G	0	480	0	169	0	395	0	51	5	2224	2	1799
chr16	92644323	G	1	480	0	171	0	395	1	50	5	2242	6	1800
chr16	92644327	G	2	484	0	171	1	398	0	51	7	2254	1	1812
chr16	92644328	G	3	483	0	170	1	398	0	51	4	2259	1	1811
chr16	92644331	G	4	482	0	171	1	399	0	51	4	2262	2	1810
chr16	92644336	G	307	165	96	74	239	155	23	27	1418	831	996	796
chr16	92644337	G	1	485	0	171	3	398	0	51	3	2270	2	1813
chr16	92644339	G	5	481	0	171	2	399	0	51	28	2244	6	1810
chr16	92644346	G	0	486	1	170	4	397	0	51	18	2255	4	1812
chr16	92644348	G	407	75	143	27	334	62	42	8	1853	387	1372	422
chr16	92644352	G	2	485	1	170	3	396	0	51	13	2257	13	1802
chr16	92644353	G	0	488	2	169	0	401	0	51	5	2270	1	1815
chr16	92644354	G	0	488	0	171	1	400	0	51	10	2266	4	1812
chr16	92644361	G	0	488	0	171	5	396	0	51	12	2267	10	1807
chr16	92644366	G	2	486	0	171	1	399	0	51	5	2275	2	1816
chr16	92644370	G	1	487	0	171	1	400	1	50	15	2266	11	1809
chr16	92644372	G	1	487	3	168	5	396	0	51	5	2274	5	1815
chr16	92644373	G	2	485	0	171	0	400	0	51	4	2276	3	1817
chr16	92644374	G	2	486	0	171	0	401	1	50	11	2269	14	1806
chr16	92644377	G	1	487	0	171	4	397	0	51	6	2274	7	1813
chr16	92644379	G	8	479	1	170	5	395	0	51	24	2258	14	1806
chr16	92644380	G	2	485	0	171	0	400	0	51	3	2279	3	1817
chr16	92644381	G	1	487	0	171	2	399	0	51	7	2275	13	1805
chr16	92644386	G	229	257	86	85	210	190	16	35	1117	1171	882	940
chr16	92644388	G	0	488	3	168	2	397	0	51	2	2284	0	1822
chr16	92644389	G	0	488	0	171	3	398	0	51	2	2286	2	1820
chr16	92644390	G	0	488	0	171	0	401	0	51	3	2284	5	1817
chr16	92644391	G	1	487	0	171	2	399	0	51	11	2277	6	1816
chr16	92644402	G	0	493	1	171	3	398	1	50	3	2293	9	1815
chr16	92644408	G	0	493	1	171	1	400	0	51	5	2295	9	1817
chr16	92644414	G	0	493	1	171	1	400	0	51	6	2294	11	1814
chr16	92644416	G	1	492	1	171	1	400	0	51	10	2290	2	1824

12	348	3	349	6	243	4	68	8	184	8	462
1	346	0	347	0	244	0	68	0	183	1	459
1	348	0	346	0	244	0	68	0	183	2	461
0	348	0	347	0	244	0	68	0	183	1	461
0	317	0	260	0	244	0	66	0	182	1	445
0	317	0	260	0	244	0	66	1	182	0	446
0	213	0	186	0	179	0	39	0	131	0	279
0	204	0	182	0	169	0	39	0	127	0	261
0	202	0	180	0	166	0	35	0	126	0	260
0	242	0	81	0	143	0	24	0	390	0	553
0	269	0	87	0	153	0	26	0	418	0	592
0	377	0	114	0	254	0	39	2	1096	0	1051
0	378	0	114	0	257	0	39	0	1103	0	1058
0	471	0	163	0	387	0	50	11	2104	5	1743
0	480	0	169	0	395	0	51	5	2229	2	1801
1	481	0	171	0	395	1	51	5	2247	6	1806
2	486	0	171	1	399	0	51	7	2261	1	1813
3	486	0	170	1	399	0	51	4	2263	1	1812
4	486	0	171	1	400	0	51	4	2266	2	1812
307	472	96	170	239	394	23	50	1418	2249	996	1792
1	486	0	171	3	401	0	51	3	2273	2	1815
5	486	0	171	2	401	0	51	28	2272	6	1816
0	486	1	171	4	401	0	51	18	2273	4	1816
407	482	143	170	334	396	42	50	1853	2240	1372	1794
2	487	1	171	3	399	0	51	13	2270	13	1815
0	488	2	171	0	401	0	51	5	2275	1	1816
0	488	0	171	1	401	0	51	10	2276	4	1816
0	488	0	171	5	401	0	51	12	2279	10	1817
2	488	0	171	1	400	0	51	5	2280	2	1818
1	488	0	171	1	401	1	51	15	2281	11	1820
1	488	3	171	5	401	0	51	5	2279	5	1820
2	487	0	171	0	400	0	51	4	2280	3	1820
2	488	0	171	0	401	1	51	11	2280	14	1820
1	488	0	171	4	401	0	51	6	2280	7	1820
8	487	1	171	5	400	0	51	24	2282	14	1820
2	487	0	171	0	400	0	51	3	2282	3	1820
1	488	0	171	2	401	0	51	7	2282	13	1818
229	486	86	171	210	400	16	51	1117	2288	882	1822
0	488	3	171	2	399	0	51	2	2286	0	1822
0	488	0	171	3	401	0	51	2	2288	2	1822
0	488	0	171	0	401	0	51	3	2287	5	1822
1	488	0	171	2	401	0	51	11	2288	6	1822
0	493	1	172	3	401	1	51	3	2296	9	1824
0	493	1	172	1	401	0	51	5	2300	9	1826
0	493	1	172	1	401	0	51	6	2300	11	1825
1	493	1	172	1	401	0	51	10	2300	2	1826

chr16	92644417	G	1	492	0	172	1	400	0	51	6	2293	3	1823
chr16	92644420	G	2	492	3	169	0	401	0	51	19	2281	39	1788
chr16	92644422	G	3	492	2	170	1	399	0	51	6	2296	1	1825
chr16	92644423	G	0	495	0	172	0	400	0	51	2	2300	4	1824
chr16	92644424	G	0	495	0	172	1	400	0	51	1	2298	6	1822
chr16	92644430	G	2	494	0	173	1	400	1	50	2	2298	3	1825
chr16	92644431	G	1	492	0	173	1	400	0	51	10	2290	5	1824
chr16	92644432	C	0	400	0	305	0	114	0	156	0	245	0	168
chr16	92644434	G	3	493	0	173	1	400	0	51	28	2274	13	1817
chr16	92644436	G	8	488	2	171	13	388	2	49	77	2224	76	1751
chr16	92644437	G	0	496	0	173	1	400	1	50	6	2293	1	1827
chr16	92644439	G	2	494	0	173	0	401	0	51	25	2277	30	1800
chr16	92644441	G	5	491	0	173	0	401	0	51	13	2289	2	1828
chr16	92644442	G	1	495	1	172	0	401	0	51	2	2300	2	1828
chr16	92644445	C	0	420	0	321	0	118	0	160	0	255	0	173
chr16	92644448	G	0	496	0	173	2	398	0	51	8	2294	4	1826
chr16	92644449	G	1	495	0	173	1	400	0	51	3	2297	7	1822
chr16	92644451	G	0	496	1	172	1	400	0	51	12	2289	8	1822
chr16	92644452	G	2	494	0	172	1	399	0	51	6	2294	6	1821
chr16	92644453	C	0	420	0	318	0	117	0	160	0	255	0	174
chr16	92644454	C	1	419	0	321	0	118	0	160	0	256	0	176
chr16	92644455	C	138	282	102	219	34	84	43	117	89	167	46	130
chr16	92644456	G	165	330	69	102	150	250	16	35	836	1462	565	1260
chr16	92644458	G	0	496	0	172	2	397	0	51	2	2299	2	1828
chr16	92644459	G	0	496	0	172	3	396	0	51	3	2297	6	1823
chr16	92644460	G	0	496	0	172	0	399	0	51	4	2295	7	1822
chr16	92644463	G	0	496	0	172	1	398	0	51	15	2286	25	1805
chr16	92644464	G	0	496	0	172	1	398	0	51	5	2295	4	1825
chr16	92644465	G	2	494	2	170	3	396	0	51	10	2289	28	1798
chr16	92644466	C	244	176	194	127	63	55	76	84	154	102	90	86
chr16	92644467	G	310	186	96	76	243	156	29	22	1394	907	1008	822
chr16	92644469	G	1	494	0	172	1	397	0	51	16	2280	5	1822
chr16	92644470	G	2	494	1	170	0	399	0	51	5	2295	2	1827
chr16	92644471	G	2	494	0	172	3	396	0	51	4	2295	2	1826
chr16	92644472	G	1	495	0	172	1	398	0	51	10	2289	3	1825
chr16	92644473	G	1	495	1	171	2	397	0	51	7	2292	3	1824
chr16	92644474	C	2	418	0	321	0	118	0	159	0	256	1	175
chr16	92644476	G	11	484	5	167	3	395	1	50	23	2277	37	1791
chr16	92644477	G	0	496	1	171	1	398	0	50	4	2295	2	1827
chr16	92644478	G	0	495	0	172	2	397	0	50	5	2292	3	1826
chr16	92644480	G	1	495	1	171	1	398	0	50	7	2292	5	1824
chr16	92644481	G	1	495	0	172	4	395	0	50	3	2296	5	1822
chr16	92644483	G	21	475	10	162	22	377	3	47	174	2124	132	1695
chr16	92644484	C	218	203	145	176	60	58	69	91	120	137	85	91
chr16	92644485	G	278	217	87	85	212	187	24	26	1285	1012	1018	810
chr16	92644486	G	3	493	1	171	1	398	0	50	10	2289	8	1821

1	493	0	172	1	401	0	51	6	2299	3	1826
2	494	3	172	0	401	0	51	19	2300	39	1827
3	495	2	172	1	400	0	51	6	2302	1	1826
0	495	0	172	0	400	0	51	2	2302	4	1828
0	495	0	172	1	401	0	51	1	2299	6	1828
2	496	0	173	1	401	1	51	2	2300	3	1828
1	493	0	173	1	401	0	51	10	2300	5	1829
0	400	0	305	0	114	0	156	0	245	0	168
3	496	0	173	1	401	0	51	28	2302	13	1830
8	496	2	173	13	401	2	51	77	2301	76	1827
0	496	0	173	1	401	1	51	6	2299	1	1828
2	496	0	173	0	401	0	51	25	2302	30	1830
5	496	0	173	0	401	0	51	13	2302	2	1830
1	496	1	173	0	401	0	51	2	2302	2	1830
0	420	0	321	0	118	0	160	0	255	0	173
0	496	0	173	2	400	0	51	8	2302	4	1830
1	496	0	173	1	401	0	51	3	2300	7	1829
0	496	1	173	1	401	0	51	12	2301	8	1830
2	496	0	172	1	400	0	51	6	2300	6	1827
0	420	0	318	0	117	0	160	0	255	0	174
1	420	0	321	0	118	0	160	0	256	0	176
138	420	102	321	34	118	43	160	89	256	46	176
165	495	69	171	150	400	16	51	836	2298	565	1825
0	496	0	172	2	399	0	51	2	2301	2	1830
0	496	0	172	3	399	0	51	3	2300	6	1829
0	496	0	172	0	399	0	51	4	2299	7	1829
0	496	0	172	1	399	0	51	15	2301	25	1830
0	496	0	172	1	399	0	51	5	2300	4	1829
2	496	2	172	3	399	0	51	10	2299	28	1826
244	420	194	321	63	118	76	160	154	256	90	176
310	496	96	172	243	399	29	51	1394	2301	1008	1830
1	495	0	172	1	398	0	51	16	2296	5	1827
2	496	1	171	0	399	0	51	5	2300	2	1829
2	496	0	172	3	399	0	51	4	2299	2	1828
1	496	0	172	1	399	0	51	10	2299	3	1828
1	496	1	172	2	399	0	51	7	2299	3	1827
2	420	0	321	0	118	0	159	0	256	1	176
11	495	5	172	3	398	1	51	23	2300	37	1828
0	496	1	172	1	399	0	50	4	2299	2	1829
0	495	0	172	2	399	0	50	5	2297	3	1829
1	496	1	172	1	399	0	50	7	2299	5	1829
1	496	0	172	4	399	0	50	3	2299	5	1827
21	496	10	172	22	399	3	50	174	2298	132	1827
218	421	145	321	60	118	69	160	120	257	85	176
278	495	87	172	212	399	24	50	1285	2297	1018	1828
3	496	1	172	1	399	0	50	10	2299	8	1829

chr16	92644487	G	1	492	0	172	1	398	0	50	13	2284	2	1827
chr16	92644488	C	1	420	0	321	0	118	3	157	1	255	0	177
chr16	92644490	G	3	493	0	172	5	393	0	50	11	2286	13	1814
chr16	92644492	C	0	420	0	319	0	118	0	160	1	255	0	176
chr16	92644493	C	0	421	1	320	0	118	0	159	0	257	0	177
chr16	92644494	C	0	421	2	319	0	118	1	159	1	256	0	177
chr16	92644496	C	1	420	0	321	0	118	5	155	1	256	0	177
chr16	92644499	G	1	494	1	170	0	398	0	50	3	2293	2	1824
chr16	92644500	G	3	492	0	171	2	396	1	49	7	2287	5	1820
chr16	92644501	C	0	421	0	322	0	118	0	160	3	253	1	176
chr16	92644502	C	220	201	155	166	58	60	70	90	115	142	61	116
chr16	92644503	G	281	214	87	84	222	175	26	24	1307	989	786	1036
chr16	92644505	G	3	492	0	171	3	395	0	50	4	2293	5	1819
chr16	92644506	C	304	117	210	112	82	36	91	69	163	94	102	75
chr16	92644507	G	362	133	109	62	280	116	29	21	1587	706	1118	701
chr16	92644508	C	0	421	0	322	0	118	3	156	1	257	0	178
chr16	92644509	C	257	163	135	187	67	51	70	90	133	124	84	92
chr16	92644510	G	265	230	95	76	205	191	19	31	1235	1060	849	972
chr16	92644511	C	7	414	5	317	2	116	7	153	2	257	0	179
chr16	92644513	G	2	493	0	171	2	394	0	50	10	2286	2	1820
chr16	92644514	C	1	420	1	321	0	118	1	159	0	258	3	174
chr16	92644516	G	2	493	1	170	1	395	0	50	10	2287	6	1816
chr16	92644517	C	15	406	6	316	0	118	3	156	3	255	3	176
chr16	92644519	C	0	421	0	322	0	118	0	159	0	258	0	179
chr16	92644520	C	16	405	0	322	0	118	0	158	5	253	6	173
chr16	92644525	C	8	413	0	322	0	119	0	159	1	257	0	179
chr16	92644527	C	0	421	6	316	0	119	0	159	2	256	0	179
chr16	92644529	G	0	494	0	171	1	395	0	50	7	2283	1	1819
chr16	92644531	G	1	494	1	169	1	394	0	50	4	2288	6	1815
chr16	92644532	C	0	421	1	322	0	119	0	159	1	257	0	179
chr16	92644533	C	222	199	128	195	45	74	73	86	102	157	68	111
chr16	92644534	G	224	271	84	86	198	197	21	29	1061	1227	705	1111
chr16	92644535	C	2	419	3	321	0	119	0	159	1	258	0	178
chr16	92644537	C	205	216	147	176	54	65	75	84	122	137	70	109
chr16	92644538	G	250	215	65	100	162	213	20	28	1023	1137	678	1033
chr16	92644539	G	8	487	1	170	0	395	1	49	23	2268	11	1806
chr16	92644544	G	0	495	1	170	0	395	0	50	6	2284	4	1817
chr16	92644545	G	0	495	1	169	0	395	0	50	5	2287	5	1817
chr16	92644547	C	0	421	1	323	0	119	0	159	1	258	0	179
chr16	92644551	C	0	420	0	325	1	118	1	157	1	258	0	179
chr16	92644553	C	0	421	0	325	0	117	0	159	0	259	0	179
chr16	92644554	C	1	420	0	325	1	118	0	159	0	259	0	179
chr16	92644555	C	190	231	123	202	47	72	61	98	105	154	48	131
chr16	92644556	G	209	273	62	104	169	215	15	34	936	1326	645	1153
chr16	92644557	G	0	473	0	164	0	383	0	49	1	2232	1	1779
chr16	92644558	G	0	473	0	164	1	382	0	49	0	2229	0	1777

1	493	0	172	1	399	0	50	13	2297	2	1829
1	421	0	321	0	118	3	160	1	256	0	177
3	496	0	172	5	398	0	50	11	2297	13	1827
0	420	0	319	0	118	0	160	1	256	0	176
0	421	1	321	0	118	0	159	0	257	0	177
0	421	2	321	0	118	1	160	1	257	0	177
1	421	0	321	0	118	5	160	1	257	0	177
1	495	1	171	0	398	0	50	3	2296	2	1826
3	495	0	171	2	398	1	50	7	2294	5	1825
0	421	0	322	0	118	0	160	3	256	1	177
220	421	155	321	58	118	70	160	115	257	61	177
281	495	87	171	222	397	26	50	1307	2296	786	1822
3	495	0	171	3	398	0	50	4	2297	5	1824
304	421	210	322	82	118	91	160	163	257	102	177
362	495	109	171	280	396	29	50	1587	2293	1118	1819
0	421	0	322	0	118	3	159	1	258	0	178
257	420	135	322	67	118	70	160	133	257	84	176
265	495	95	171	205	396	19	50	1235	2295	849	1821
7	421	5	322	2	118	7	160	2	259	0	179
2	495	0	171	2	396	0	50	10	2296	2	1822
1	421	1	322	0	118	1	160	0	258	3	177
2	495	1	171	1	396	0	50	10	2297	6	1822
15	421	6	322	0	118	3	159	3	258	3	179
0	421	0	322	0	118	0	159	0	258	0	179
16	421	0	322	0	118	0	158	5	258	6	179
8	421	0	322	0	119	0	159	1	258	0	179
0	421	6	322	0	119	0	159	2	258	0	179
0	494	0	171	1	396	0	50	7	2290	1	1820
1	495	1	170	1	395	0	50	4	2292	6	1821
0	421	1	323	0	119	0	159	1	258	0	179
222	421	128	323	45	119	73	159	102	259	68	179
224	495	84	170	198	395	21	50	1061	2288	705	1816
2	421	3	324	0	119	0	159	1	259	0	178
205	421	147	323	54	119	75	159	122	259	70	179
250	465	65	165	162	375	20	48	1023	2160	678	1711
8	495	1	171	0	395	1	50	23	2291	11	1817
0	495	1	171	0	395	0	50	6	2290	4	1821
0	495	1	170	0	395	0	50	5	2292	5	1822
0	421	1	324	0	119	0	159	1	259	0	179
0	420	0	325	1	119	1	158	1	259	0	179
0	421	0	325	0	117	0	159	0	259	0	179
1	421	0	325	1	119	0	159	0	259	0	179
190	421	123	325	47	119	61	159	105	259	48	179
209	482	62	166	169	384	15	49	936	2262	645	1798
0	473	0	164	0	383	0	49	1	2233	1	1780
0	473	0	164	1	383	0	49	0	2229	0	1777

chr16	92644559	C	0	419	0	323	0	120	0	159	0	257	0	178
chr16	92644562	G	0	473	0	161	0	381	0	48	0	2228	0	1776
chr16	92644563	G	0	473	0	161	0	383	0	48	1	2225	0	1776
chr16	92644565	C	0	421	0	325	0	120	1	159	0	259	1	178
chr16	92644567	G	0	473	0	161	0	379	0	48	0	2219	0	1771
chr16	92644570	C	0	421	0	325	0	121	0	160	2	257	0	179
chr16	92644573	C	0	422	0	325	0	121	1	159	1	258	0	179
chr16	92644576	G	0	472	0	161	1	379	0	48	0	2182	0	1745
chr16	92644580	C	10	413	0	325	2	119	0	160	2	257	0	179
chr16	92644582	G	0	465	0	156	0	375	0	48	0	2152	0	1717
chr16	92644583	C	1	122	1	142	0	84	0	95	1	164	0	114
chr16	92644584	C	51	67	47	91	28	54	32	63	57	109	29	87
chr16	92644590	C	1	103	0	132	0	81	0	91	1	157	1	109
chr16	92644591	C	1	102	0	130	0	81	0	90	1	156	0	110
chr16	92644603	C	1	87	0	109	0	68	0	75	0	123	0	102
chr16	92644604	C	32	54	41	67	20	48	27	45	32	89	35	64
chr16	92644639	c	1	352	0	266	0	68	0	110	0	161	0	134
chr16	92644663	C	0	375	1	271	0	67	0	100	0	158	0	135
chr16	92644670	C	126	224	80	183	26	39	17	78	56	96	37	98
chr16	92644686	C	0	325	0	223	0	59	0	84	0	143	0	126
chr16	92644691	C	0	330	0	217	0	49	0	80	0	115	0	103
chr16	92644696	C	0	285	0	173	0	43	0	67	0	102	0	88
chr16	92644700	C	2	320	2	200	0	37	0	68	1	94	0	69
chr16	92644701	C	3	319	4	197	2	35	1	68	3	92	2	66
chr16	92644702	C	7	315	8	192	0	37	2	67	4	91	1	68
chr16	92644703	C	49	273	6	193	4	33	2	67	12	83	18	51
chr16	92644705	C	13	309	6	192	1	35	2	67	7	88	6	63
chr16	92644706	C	16	306	17	180	0	36	3	66	2	91	4	63
chr16	92644707	C	53	269	74	125	15	21	20	49	14	81	6	62
chr16	92644709	C	135	187	12	186	14	22	24	44	35	60	29	39
chr16	92644711	C	5	316	21	179	1	35	1	67	3	92	1	67
chr16	92644712	C	2	319	0	200	0	36	0	68	0	95	0	68
chr16	92644713	C	1	320	1	199	0	36	2	66	3	92	0	68
chr16	92644716	C	76	244	71	128	5	31	21	47	15	80	0	68
chr16	92644718	C	2	317	1	198	0	36	0	68	0	94	0	68
chr16	92644719	C	1	320	0	199	0	36	0	68	0	94	1	67
chr16	92644720	C	0	321	0	199	0	36	0	68	2	92	0	68
chr16	92644721	C	0	321	1	198	0	36	0	68	0	94	0	68
chr16	92644722	C	1	320	1	198	0	36	9	58	0	94	0	68
chr16	92644731	C	0	319	1	197	0	36	0	66	0	94	0	68
chr16	92644740	C	1	317	0	199	0	36	0	66	0	94	0	67
chr16	92644745	C	0	318	1	197	0	36	0	66	0	93	1	67
chr16	92644746	C	0	318	0	198	0	36	0	66	0	94	0	68
chr16	92644747	C	0	318	1	197	0	36	0	66	0	93	0	68
chr16	92644748	C	1	317	0	198	0	36	0	66	0	94	0	68
chr16	92644754	C	10	308	1	195	1	35	0	66	0	94	0	68

0	419	0	323	0	120	0	159	0	257	0	178
0	473	0	161	0	381	0	48	0	2228	0	1776
0	473	0	161	0	383	0	48	1	2226	0	1776
0	421	0	325	0	120	1	160	0	259	1	179
0	473	0	161	0	379	0	48	0	2219	0	1771
0	421	0	325	0	121	0	160	2	259	0	179
0	422	0	325	0	121	1	160	1	259	0	179
0	472	0	161	1	380	0	48	0	2182	0	1745
10	423	0	325	2	121	0	160	2	259	0	179
0	465	0	156	0	375	0	48	0	2152	0	1717
1	123	1	143	0	84	0	95	1	165	0	114
51	118	47	138	28	82	32	95	57	166	29	116
1	104	0	132	0	81	0	91	1	158	1	110
1	103	0	130	0	81	0	90	1	157	0	110
1	88	0	109	0	68	0	75	0	123	0	102
32	86	41	108	20	68	27	72	32	121	35	99
1	353	0	266	0	68	0	110	0	161	0	134
0	375	1	272	0	67	0	100	0	158	0	135
126	350	80	263	26	65	17	95	56	152	37	135
0	325	0	223	0	59	0	84	0	143	0	126
0	330	0	217	0	49	0	80	0	115	0	103
0	285	0	173	0	43	0	67	0	102	0	88
2	322	2	202	0	37	0	68	1	95	0	69
3	322	4	201	2	37	1	69	3	95	2	68
7	322	8	200	0	37	2	69	4	95	1	69
49	322	6	199	4	37	2	69	12	95	18	69
13	322	6	198	1	36	2	69	7	95	6	69
16	322	17	197	0	36	3	69	2	93	4	67
53	322	74	199	15	36	20	69	14	95	6	68
135	322	12	198	14	36	24	68	35	95	29	68
5	321	21	200	1	36	1	68	3	95	1	68
2	321	0	200	0	36	0	68	0	95	0	68
1	321	1	200	0	36	2	68	3	95	0	68
76	320	71	199	5	36	21	68	15	95	0	68
2	319	1	199	0	36	0	68	0	94	0	68
1	321	0	199	0	36	0	68	0	94	1	68
0	321	0	199	0	36	0	68	2	94	0	68
0	321	1	199	0	36	0	68	0	94	0	68
1	321	1	199	0	36	9	67	0	94	0	68
0	319	1	198	0	36	0	66	0	94	0	68
1	318	0	199	0	36	0	66	0	94	0	67
0	318	1	198	0	36	0	66	0	93	1	68
0	318	0	198	0	36	0	66	0	94	0	68
0	318	1	198	0	36	0	66	0	93	0	68
1	318	0	198	0	36	0	66	0	94	0	68
10	318	1	196	1	36	0	66	0	94	0	68

chr16	92644758	C	0	317	1	197	0	36	0	66	0	93	0	67
chr16	92644761	C	0	310	0	191	0	36	0	66	0	91	0	67
chr16	92644762	C	0	311	0	191	0	36	0	66	0	93	0	67
chr16	92644771	C	0	306	0	191	0	35	0	65	0	90	0	67
chr16	92644772	C	0	307	0	191	0	35	0	65	0	90	0	66
chr16	92644777	C	0	293	0	182	0	33	0	64	0	88	0	62
chr16	92644781	C	0	274	0	164	0	31	0	59	0	87	0	49
chr16	92644782	C	0	274	0	162	0	31	0	59	0	86	0	49
chr17	45706386	C	0	1475	0	376	0	1218	0	297	0	1059	0	1123
chr17	45706403	C	0	2521	0	640	0	2024	0	556	0	1537	0	1796
chr17	45706405	C	0	2522	0	641	0	2026	0	556	0	1536	0	1798
chr17	45706410	C	0	2529	0	644	0	2038	0	558	1	1538	1	1803
chr17	45706411	C	1814	723	516	232	1544	500	373	189	1336	206	1303	505
chr17	45706414	C	10	2573	3	783	5	2039	4	560	1	1541	4	1807
chr17	45706418	C	7	2575	0	786	1	2045	1	564	2	1542	3	1807
chr17	45706419	C	4	2581	1	786	6	2045	4	562	3	1542	5	1808
chr17	45706439	C	6	2584	2	784	3	2067	0	568	4	1546	4	1818
chr17	45706440	C	2	2590	0	789	4	2067	2	567	1	1551	3	1820
chr17	45706442	C	5	2587	1	788	5	2067	3	566	8	1545	0	1823
chr17	45706449	C	3	2579	1	788	1	2065	3	565	1	1551	2	1814
chr17	45706452	C	0	2592	0	790	2	2073	2	567	2	1553	0	1823
chr17	45706458	G	0	2982	0	4759	0	536	0	586	0	766	1	1001
chr17	45706461	C	26	2569	2	788	23	2058	0	569	9	1550	19	1808
chr17	45706463	C	5	2591	1	790	3	2108	1	569	1	1586	2	1835
chr17	45706464	C	5	2592	2	789	0	2113	0	570	5	1585	2	1834
chr17	45706467	G	0	3175	0	5109	0	561	0	613	1	819	0	1052
chr17	45706469	C	9	2589	0	791	4	2107	0	570	2	1589	5	1832
chr17	45706470	C	10	2588	4	787	2	2111	6	565	6	1587	6	1831
chr17	45706472	C	39	2559	1	789	35	2079	2	568	20	1574	17	1820
chr17	45706474	G	1	3261	0	5233	0	578	0	633	1	852	2	1075
chr17	45706475	C	6	2592	0	790	3	2092	1	568	2	1578	8	1826
chr17	45706476	C	11	2589	1	790	18	2100	6	565	11	1591	24	1815
chr17	45706478	G	0	3263	0	5238	0	580	0	633	0	853	0	1078
chr17	45706479	C	6	2590	2	791	7	2110	0	570	6	1603	5	1833
chr17	45706481	C	1618	985	409	384	1377	746	373	198	1035	573	1062	779
chr17	45706482	G	2316	887	3317	1744	454	118	402	213	678	150	691	357
chr17	45706483	G	56	3269	54	5239	6	578	6	629	10	849	3	1079
chr17	45706485	G	13	3317	7	5292	2	580	0	635	3	852	3	1078
chr17	45706488	G	9	3327	7	5295	1	587	1	636	4	870	2	1091
chr17	45706490	G	30	3308	57	5246	5	583	6	631	10	864	15	1078
chr17	45706491	C	18	2581	2	789	7	2109	0	569	5	1604	3	1830
chr17	45706493	C	3	2602	1	793	1	2125	1	571	2	1612	3	1841
chr17	45706494	C	40	2565	6	788	29	2098	5	567	18	1597	89	1756
chr17	45706496	G	18	3322	4	5307	1	586	5	633	6	867	7	1084
chr17	45706497	G	5	3335	9	5303	4	584	0	638	0	873	2	1091
chr17	45706498	C	31	2575	12	782	35	2096	3	569	25	1592	15	1829

0	317	1	198	0	36	0	66	0	93	0	67
0	310	0	191	0	36	0	66	0	91	0	67
0	311	0	191	0	36	0	66	0	93	0	67
0	306	0	191	0	35	0	65	0	90	0	67
0	307	0	191	0	35	0	65	0	90	0	66
0	293	0	182	0	33	0	64	0	88	0	62
0	274	0	164	0	31	0	59	0	87	0	49
0	274	0	162	0	31	0	59	0	86	0	49
0	1475	0	376	0	1218	0	297	0	1059	0	1123
0	2521	0	640	0	2024	0	556	0	1537	0	1796
0	2522	0	641	0	2026	0	556	0	1536	0	1798
0	2529	0	644	0	2038	0	558	1	1539	1	1804
1814	2537	516	748	1544	2044	373	562	1336	1542	1303	1808
10	2583	3	786	5	2044	4	564	1	1542	4	1811
7	2582	0	786	1	2046	1	565	2	1544	3	1810
4	2585	1	787	6	2051	4	566	3	1545	5	1813
6	2590	2	786	3	2070	0	568	4	1550	4	1822
2	2592	0	789	4	2071	2	569	1	1552	3	1823
5	2592	1	789	5	2072	3	569	8	1553	0	1823
3	2582	1	789	1	2066	3	568	1	1552	2	1816
0	2592	0	790	2	2075	2	569	2	1555	0	1823
0	2982	0	4759	0	536	0	586	0	766	1	1002
26	2595	2	790	23	2081	0	569	9	1559	19	1827
5	2596	1	791	3	2111	1	570	1	1587	2	1837
5	2597	2	791	0	2113	0	570	5	1590	2	1836
0	3175	0	5109	0	561	0	613	1	820	0	1052
9	2598	0	791	4	2111	0	570	2	1591	5	1837
10	2598	4	791	2	2113	6	571	6	1593	6	1837
39	2598	1	790	35	2114	2	570	20	1594	17	1837
1	3262	0	5233	0	578	0	633	1	853	2	1077
6	2598	0	790	3	2095	1	569	2	1580	8	1834
11	2600	1	791	18	2118	6	571	11	1602	24	1839
0	3263	0	5238	0	580	0	633	0	853	0	1078
6	2596	2	793	7	2117	0	570	6	1609	5	1838
1618	2603	409	793	1377	2123	373	571	1035	1608	1062	1841
2316	3203	3317	5061	454	572	402	615	678	828	691	1048
56	3325	54	5293	6	584	6	635	10	859	3	1082
13	3330	7	5299	2	582	0	635	3	855	3	1081
9	3336	7	5302	1	588	1	637	4	874	2	1093
30	3338	57	5303	5	588	6	637	10	874	15	1093
18	2599	2	791	7	2116	0	569	5	1609	3	1833
3	2605	1	794	1	2126	1	572	2	1614	3	1844
40	2605	6	794	29	2127	5	572	18	1615	89	1845
18	3340	4	5311	1	587	5	638	6	873	7	1091
5	3340	9	5312	4	588	0	638	0	873	2	1093
31	2606	12	794	35	2131	3	572	25	1617	15	1844

chr17	45706500	C	5	2591	1	791	13	2105	0	570	4	1605	3	1832
chr17	45706503	C	4	2603	1	796	1	2131	1	570	9	1613	4	1849
chr17	45706506	G	20	3319	3	5311	4	584	0	638	1	873	0	1093
chr17	45706508	C	981	1627	203	594	825	1310	201	371	663	962	562	1290
chr17	45706509	G	1337	2004	2252	3053	197	389	227	412	440	433	440	650
chr17	45706512	G	12	3329	4	5310	3	585	9	630	3	871	11	1082
chr17	45706515	C	8	2605	2	797	0	2138	1	571	2	1629	3	1855
chr17	45706518	G	29	3314	44	5268	4	584	2	637	13	862	17	1076
chr17	45706519	C	1991	622	607	192	1681	457	435	137	1268	365	1291	567
chr17	45706520	G	2750	579	4260	1038	467	119	489	145	730	138	884	201
chr17	45706521	G	5	3336	12	5299	3	585	0	639	3	872	4	1088
chr17	45706524	G	9	3341	15	5301	3	585	0	641	3	873	3	1091
chr17	45706526	C	7	2609	1	796	3	2134	2	569	1	1639	5	1854
chr17	45706527	C	4	2616	2	797	3	2136	0	572	0	1642	6	1855
chr17	45706530	C	35	2586	11	789	9	2132	7	565	20	1629	25	1837
chr17	45706532	G	1	3357	11	5310	2	585	3	640	6	873	4	1094
chr17	45706533	G	7	3350	4	5316	1	587	1	642	0	879	2	1098
chr17	45706536	C	7	2612	0	797	3	2210	1	572	6	1756	3	1872
chr17	45706539	G	10	3349	20	5304	2	587	1	643	12	867	9	1091
chr17	45706540	C	4	597	1	280	5	460	0	166	1	349	3	372
chr17	45706542	C	1	586	0	276	1	459	0	158	1	346	0	364
chr17	45706544	G	8	3355	26	5297	3	586	6	638	1	879	15	1085
chr17	45706545	C	10	541	1	266	9	440	2	152	6	338	7	338
chr17	45706547	G	75	3287	122	5199	11	581	5	643	13	869	21	1079
chr17	45706548	C	5	324	1	224	7	300	0	77	7	284	0	199
chr17	45706550	G	37	3327	77	5249	2	589	15	633	9	874	1	1102
chr17	45706551	C	0	165	0	182	0	233	0	25	0	280	0	120
chr17	45706554	C	1	163	0	183	0	232	0	25	1	289	0	120
chr17	45706556	C	0	163	1	182	1	230	0	26	0	285	0	115
chr17	45706557	C	0	163	0	182	0	230	0	25	0	294	0	117
chr17	45706558	C	67	94	52	130	103	125	12	13	130	162	27	89
chr17	45706559	G	1514	1848	2038	3282	263	332	187	457	458	418	452	649
chr17	45706560	G	4	3363	7	5326	1	594	4	644	2	881	2	1103
chr17	45706561	G	3	3364	4	5331	3	590	0	648	1	880	1	1104
chr17	45706563	G	8	3359	36	5300	2	590	0	648	2	881	8	1097
chr17	45706566	G	32	3334	5	5333	2	593	1	647	5	879	1	1105
chr17	45706569	C	0	132	1	88	2	220	0	20	2	290	0	110
chr17	45706571	G	16	3349	14	5325	5	590	0	648	3	881	2	1106
chr17	45706572	G	14	3353	10	5326	0	596	1	648	3	885	3	1106
chr17	45706573	G	9	3358	15	5322	0	596	2	647	1	887	6	1103
chr17	45706574	G	12	3354	13	5325	1	595	3	646	1	886	2	1107
chr17	45706575	C	0	423	0	154	2	408	0	109	1	368	0	352
chr17	45706577	G	11	3357	7	5334	3	594	4	646	1	886	1	1108
chr17	45706578	G	11	3357	10	5331	0	597	1	649	5	883	1	1109
chr17	45706580	C	1	443	2	166	1	437	0	126	0	381	2	371
chr17	45706581	C	1	447	0	171	2	445	2	125	0	388	0	378

5	2596	1	792	13	2118	0	570	4	1609	3	1835
4	2607	1	797	1	2132	1	571	9	1622	4	1853
20	3339	3	5314	4	588	0	638	1	874	0	1093
981	2608	203	797	825	2135	201	572	663	1625	562	1852
1337	3341	2252	5305	197	586	227	639	440	873	440	1090
12	3341	4	5314	3	588	9	639	3	874	11	1093
8	2613	2	799	0	2138	1	572	2	1631	3	1858
29	3343	44	5312	4	588	2	639	13	875	17	1093
1991	2613	607	799	1681	2138	435	572	1268	1633	1291	1858
2750	3329	4260	5298	467	586	489	634	730	868	884	1085
5	3341	12	5311	3	588	0	639	3	875	4	1092
9	3350	15	5316	3	588	0	641	3	876	3	1094
7	2616	1	797	3	2137	2	571	1	1640	5	1859
4	2620	2	799	3	2139	0	572	0	1642	6	1861
35	2621	11	800	9	2141	7	572	20	1649	25	1862
1	3358	11	5321	2	587	3	643	6	879	4	1098
7	3357	4	5320	1	588	1	643	0	879	2	1100
7	2619	0	797	3	2213	1	573	6	1762	3	1875
10	3359	20	5324	2	589	1	644	12	879	9	1100
4	601	1	281	5	465	0	166	1	350	3	375
1	587	0	276	1	460	0	158	1	347	0	364
8	3363	26	5323	3	589	6	644	1	880	15	1100
10	551	1	267	9	449	2	154	6	344	7	345
75	3362	122	5321	11	592	5	648	13	882	21	1100
5	329	1	225	7	307	0	77	7	291	0	199
37	3364	77	5326	2	591	15	648	9	883	1	1103
0	165	0	182	0	233	0	25	0	280	0	120
1	164	0	183	0	232	0	25	1	290	0	120
0	163	1	183	1	231	0	26	0	285	0	115
0	163	0	182	0	230	0	25	0	294	0	117
67	161	52	182	103	228	12	25	130	292	27	116
1514	3362	2038	5320	263	595	187	644	458	876	452	1101
4	3367	7	5333	1	595	4	648	2	883	2	1105
3	3367	4	5335	3	593	0	648	1	881	1	1105
8	3367	36	5336	2	592	0	648	2	883	8	1105
32	3366	5	5338	2	595	1	648	5	884	1	1106
0	132	1	89	2	222	0	20	2	292	0	110
16	3365	14	5339	5	595	0	648	3	884	2	1108
14	3367	10	5336	0	596	1	649	3	888	3	1109
9	3367	15	5337	0	596	2	649	1	888	6	1109
12	3366	13	5338	1	596	3	649	1	887	2	1109
0	423	0	154	2	410	0	109	1	369	0	352
11	3368	7	5341	3	597	4	650	1	887	1	1109
11	3368	10	5341	0	597	1	650	5	888	1	1110
1	444	2	168	1	438	0	126	0	381	2	373
1	448	0	171	2	447	2	127	0	388	0	378

chr17	45706583	C	287	220	99	92	284	215	75	85	277	175	199	237
chr17	45706584	G	2216	1157	3135	2202	381	218	331	319	623	266	671	440
chr17	45706585	G	5	3369	6	5335	0	599	3	648	1	889	2	1112
chr17	45706587	G	16	3361	10	5337	0	600	1	652	5	885	12	1101
chr17	45706589	C	3	842	1	270	0	780	1	258	1	651	0	672
chr17	45706592	C	0	851	1	282	0	782	0	261	0	655	2	681
chr17	45706593	C	5	852	0	286	3	781	0	261	6	649	0	683
chr17	45706595	C	21	851	0	296	32	755	6	258	24	632	7	687
chr17	45706597	C	2	991	1	327	6	884	2	285	1	731	3	759
chr17	45706598	C	507	733	141	273	451	662	127	208	412	481	373	576
chr17	45706599	G	1356	2014	1827	3512	226	354	253	382	380	500	446	659
chr17	45706600	C	1074	1203	261	446	840	1124	221	303	736	802	703	933
chr17	45706601	G	2000	1381	3094	2252	288	311	338	320	553	340	652	466
chr17	45706602	G	1	3382	6	5342	1	600	0	658	2	893	0	1118
chr17	45706608	G	2	605	1	817	0	213	0	233	0	254	0	227
chr17	45706611	G	1	449	0	585	0	129	0	144	0	183	0	171
chr17	45706613	G	2	380	0	435	0	123	0	141	1	161	1	154
chr17	45706614	G	2	354	2	409	0	119	1	135	0	154	0	149
chr17	45706617	C	11	2622	1	802	3	2229	0	578	14	1756	13	1878
chr17	45706619	C	5	2624	0	804	2	2232	0	578	2	1767	0	1891
chr17	45706621	G	2	267	0	260	1	76	0	105	0	116	1	105
chr17	45706622	G	0	258	4	248	1	76	1	103	0	116	1	106
chr17	45706626	C	15	2616	1	802	9	2223	0	576	17	1750	10	1878
chr17	45706628	G	1	246	0	227	0	79	0	103	0	109	0	103
chr17	45706629	C	8	2619	3	800	3	2225	0	576	2	1764	6	1881
chr17	45706631	C	1481	1132	425	377	1279	945	310	266	1088	678	958	925
chr17	45706632	G	112	91	99	102	45	30	60	42	76	35	57	44
chr17	45706634	C	6	2624	8	794	9	2222	1	576	3	1763	3	1885
chr17	45706636	C	32	2597	13	788	61	2164	4	573	37	1728	49	1834
chr17	45706638	G	0	199	0	175	0	95	0	112	1	118	0	105
chr17	45706639	C	4	2626	2	799	3	2228	0	577	8	1758	4	1883
chr17	45706641	G	3	196	1	173	3	95	2	108	4	118	0	109
chr17	45706643	C	3	2629	2	800	1	2229	0	576	5	1761	3	1884
chr17	45706644	C	14	2618	1	801	15	2214	0	576	14	1751	24	1862
chr17	45706647	G	0	192	0	168	0	98	1	108	0	120	0	110
chr17	45706650	G	2	193	4	164	2	97	0	107	0	121	0	113
chr17	45706653	C	39	2588	1	800	28	2200	0	575	25	1740	28	1858
chr17	45706655	C	9	2618	0	801	8	2218	10	565	9	1754	2	1883
chr17	45706657	C	1277	1260	323	450	1077	1061	245	315	884	797	845	983
chr17	45706658	G	82	113	71	101	32	66	41	55	62	56	47	62
chr17	45706659	G	0	193	0	171	0	96	0	97	0	116	0	109
chr17	45706660	C	2049	512	549	233	1743	425	433	135	1385	338	1312	543
chr17	45706661	G	137	55	129	42	74	29	62	38	90	29	77	31
chr17	45706662	G	1	186	1	169	1	97	0	98	0	115	0	108
chr17	45706664	C	9	2616	2	796	2	2224	2	572	6	1757	12	1870
chr17	45706668	C	28	2596	3	792	10	2214	2	572	17	1744	5	1877

287	507	99	191	284	499	75	160	277	452	199	436
2216	3373	3135	5337	381	599	331	650	623	889	671	1111
5	3374	6	5341	0	599	3	651	1	890	2	1114
16	3377	10	5347	0	600	1	653	5	890	12	1113
3	845	1	271	0	780	1	259	1	652	0	672
0	851	1	283	0	782	0	261	0	655	2	683
5	857	0	286	3	784	0	261	6	655	0	683
21	872	0	296	32	787	6	264	24	656	7	694
2	993	1	328	6	890	2	287	1	732	3	762
507	1240	141	414	451	1113	127	335	412	893	373	949
1356	3370	1827	5339	226	580	253	635	380	880	446	1105
1074	2277	261	707	840	1964	221	524	736	1538	703	1636
2000	3381	3094	5346	288	599	338	658	553	893	652	1118
1	3383	6	5348	1	601	0	658	2	895	0	1118
2	607	1	818	0	213	0	233	0	254	0	227
1	450	0	585	0	129	0	144	0	183	0	171
2	382	0	435	0	123	0	141	1	162	1	155
2	356	2	411	0	119	1	136	0	154	0	149
11	2633	1	803	3	2232	0	578	14	1770	13	1891
5	2629	0	804	2	2234	0	578	2	1769	0	1891
2	269	0	260	1	77	0	105	0	116	1	106
0	258	4	252	1	77	1	104	0	116	1	107
15	2631	1	803	9	2232	0	576	17	1767	10	1888
1	247	0	227	0	79	0	103	0	109	0	103
8	2627	3	803	3	2228	0	576	2	1766	6	1887
1481	2613	425	802	1279	2224	310	576	1088	1766	958	1883
112	203	99	201	45	75	60	102	76	111	57	101
6	2630	8	802	9	2231	1	577	3	1766	3	1888
32	2629	13	801	61	2225	4	577	37	1765	49	1883
0	199	0	175	0	95	0	112	1	119	0	105
4	2630	2	801	3	2231	0	577	8	1766	4	1887
3	199	1	174	3	98	2	110	4	122	0	109
3	2632	2	802	1	2230	0	576	5	1766	3	1887
14	2632	1	802	15	2229	0	576	14	1765	24	1886
0	192	0	168	0	98	1	109	0	120	0	110
2	195	4	168	2	99	0	107	0	121	0	113
39	2627	1	801	28	2228	0	575	25	1765	28	1886
9	2627	0	801	8	2226	10	575	9	1763	2	1885
1277	2537	323	773	1077	2138	245	560	884	1681	845	1828
82	195	71	172	32	98	41	96	62	118	47	109
0	193	0	171	0	96	0	97	0	116	0	109
2049	2561	549	782	1743	2168	433	568	1385	1723	1312	1855
137	192	129	171	74	103	62	100	90	119	77	108
1	187	1	170	1	98	0	98	0	115	0	108
9	2625	2	798	2	2226	2	574	6	1763	12	1882
28	2624	3	795	10	2224	2	574	17	1761	5	1882

chr17	45706674	G	2	3310	2	5282	0	516	0	566	1	849	0	1089
chr17	45706677	G	63	3239	131	5149	2	514	0	565	16	832	14	1073
chr17	45706680	G	6	3298	79	5199	1	516	0	564	2	844	8	1080
chr17	45706683	C	7	2441	2	756	4	2078	1	535	7	1647	5	1776
chr17	45706686	C	7	2616	1	795	3	2220	3	571	7	1747	5	1878
chr17	45706689	C	4	2620	2	794	10	2214	1	573	5	1755	6	1876
chr17	45706692	C	6	2617	1	794	7	2217	1	573	6	1747	4	1879
chr17	45706695	G	88	3203	32	5243	8	507	7	553	22	820	14	1066
chr17	45706698	C	5	2616	3	793	8	2215	1	573	4	1747	1	1881
chr17	45706700	C	6	2613	0	796	5	2216	0	574	3	1746	1	1882
chr17	45706706	G	11	3283	10	5265	0	514	1	557	2	837	3	1079
chr17	45706707	G	10	3279	8	5266	2	512	3	553	1	838	1	1080
chr17	45706709	C	1086	1531	295	501	954	1268	223	349	857	892	780	1102
chr17	45706710	G	1455	1779	2463	2679	198	296	255	288	390	426	436	617
chr17	45706713	G	4	3287	5	5265	0	511	1	554	6	831	4	1074
chr17	45706714	G	2	3289	4	5266	1	510	2	553	4	833	1	1077
chr17	45706715	G	6	3284	6	5261	0	510	0	555	3	833	5	1073
chr17	45706717	G	27	3115	297	4681	7	474	1	528	7	765	22	1009
chr17	45706718	C	6	2601	5	777	4	2212	2	571	2	1741	7	1871
chr17	45706719	C	1	2608	0	766	2	2216	1	571	3	1743	0	1876
chr17	45706720	C	1641	942	421	329	1391	825	350	216	1179	563	1038	837
chr17	45706721	G	1883	1396	3260	2005	327	181	321	228	540	291	621	447
chr17	45706722	C	6	2580	1	739	5	2208	1	569	0	1742	4	1871
chr17	45706723	C	1291	1250	279	453	1184	1023	249	317	946	789	807	1066
chr17	45706724	G	1718	1550	2767	2486	289	210	270	273	493	332	504	558
chr17	45706725	G	5	3279	0	5263	0	511	0	552	0	833	1	1068
chr17	45706726	G	4	3278	8	5254	1	510	2	549	1	831	2	1067
chr17	45706727	G	6	3277	8	5254	1	510	1	551	4	829	5	1065
chr17	45706731	G	12	3265	20	5228	2	506	1	549	3	826	0	1065
chr17	45706734	G	7	3272	10	5251	2	503	1	545	3	825	1	1063
chr17	45706736	G	72	3196	198	5055	13	481	7	527	23	801	28	1024
chr17	45706737	C	0	1887	0	572	2	1626	0	334	0	1326	0	1295
chr17	45706741	G	5	3263	3	5245	3	492	1	532	2	822	1	1053
chr17	45706742	C	1	1864	0	558	0	1605	0	327	0	1311	0	1281
chr17	45706743	C	0	1862	0	555	1	1605	0	327	0	1308	0	1280
chr17	45706744	C	0	1861	0	550	0	1602	0	326	0	1308	0	1277
chr17	45706746	G	13	3250	2	5252	4	487	5	523	5	810	6	1042
chr17	45706757	G	2	3260	11	5237	0	492	4	523	3	808	10	1031
chr17	45706768	G	0	3258	12	5230	1	490	0	527	4	810	0	1043
chr17	45706770	G	7	3254	8	5236	1	490	1	526	1	813	2	1041
chr17	45706773	G	2	3259	12	5232	1	490	0	527	1	813	1	1041
chr17	45706775	G	22	3218	14	5212	6	483	0	522	3	806	20	1014
chr17	45706778	G	106	3140	67	5164	4	484	13	513	22	785	20	1020
chr17	45706783	G	7	3246	4	5233	0	486	8	518	1	811	1	1041
chr17	45706803	G	0	3238	0	5220	1	483	0	525	0	806	0	1039
chr17	45706804	G	1	3236	0	5219	1	483	1	525	0	806	0	1039

2	3312	2	5284	0	516	0	566	1	850	0	1089
63	3302	131	5280	2	516	0	565	16	848	14	1087
6	3304	79	5278	1	517	0	564	2	846	8	1088
7	2448	2	758	4	2082	1	536	7	1654	5	1781
7	2623	1	796	3	2223	3	574	7	1754	5	1883
4	2624	2	796	10	2224	1	574	5	1760	6	1882
6	2623	1	795	7	2224	1	574	6	1753	4	1883
88	3291	32	5275	8	515	7	560	22	842	14	1080
5	2621	3	796	8	2223	1	574	4	1751	1	1882
6	2619	0	796	5	2221	0	574	3	1749	1	1883
11	3294	10	5275	0	514	1	558	2	839	3	1082
10	3289	8	5274	2	514	3	556	1	839	1	1081
1086	2617	295	796	954	2222	223	572	857	1749	780	1882
1455	3234	2463	5142	198	494	255	543	390	816	436	1053
4	3291	5	5270	0	511	1	555	6	837	4	1078
2	3291	4	5270	1	511	2	555	4	837	1	1078
6	3290	6	5267	0	510	0	555	3	836	5	1078
27	3142	297	4978	7	481	1	529	7	772	22	1031
6	2607	5	782	4	2216	2	573	2	1743	7	1878
1	2609	0	766	2	2218	1	572	3	1746	0	1876
1641	2583	421	750	1391	2216	350	566	1179	1742	1038	1875
1883	3279	3260	5265	327	508	321	549	540	831	621	1068
6	2586	1	740	5	2213	1	570	0	1742	4	1875
1291	2541	279	732	1184	2207	249	566	946	1735	807	1873
1718	3268	2767	5253	289	499	270	543	493	825	504	1062
5	3284	0	5263	0	511	0	552	0	833	1	1069
4	3282	8	5262	1	511	2	551	1	832	2	1069
6	3283	8	5262	1	511	1	552	4	833	5	1070
12	3277	20	5248	2	508	1	550	3	829	0	1065
7	3279	10	5261	2	505	1	546	3	828	1	1064
72	3268	198	5253	13	494	7	534	23	824	28	1052
0	1887	0	572	2	1628	0	334	0	1326	0	1295
5	3268	3	5248	3	495	1	533	2	824	1	1054
1	1865	0	558	0	1605	0	327	0	1311	0	1281
0	1862	0	555	1	1606	0	327	0	1308	0	1280
0	1861	0	550	0	1602	0	326	0	1308	0	1277
13	3263	2	5254	4	491	5	528	5	815	6	1048
2	3262	11	5248	0	492	4	527	3	811	10	1041
0	3258	12	5242	1	491	0	527	4	814	0	1043
7	3261	8	5244	1	491	1	527	1	814	2	1043
2	3261	12	5244	1	491	0	527	1	814	1	1042
22	3240	14	5226	6	489	0	522	3	809	20	1034
106	3246	67	5231	4	488	13	526	22	807	20	1040
7	3253	4	5237	0	486	8	526	1	812	1	1042
0	3238	0	5220	1	484	0	525	0	806	0	1039
1	3237	0	5219	1	484	1	526	0	806	0	1039

chr17	45706808	G	0	3232	0	5213	0	484	0	526	1	805	0	1039
chr17	45706809	G	0	3233	0	5215	0	483	0	526	0	805	0	1037
chr17	45706813	G	1	3231	1	5213	1	480	1	521	0	802	0	1038
chr17	45706820	G	0	3201	0	5169	0	475	0	517	0	793	0	1029
chr19	24277358	c	1	2138	0	1867	0	1109	0	371	0	954	0	1605
chr19	24277364	c	0	2218	0	1913	0	1121	0	384	0	961	0	1631
chr19	24277365	c	0	2249	0	1930	0	1137	0	395	0	964	0	1647
chr19	24277367	c	0	2267	0	1943	0	1144	0	399	0	974	0	1658
chr19	24277368	c	0	2282	0	1949	0	1160	0	410	0	981	1	1667
chr19	24277394	c	5	2466	2	2141	3	1219	3	433	2	1021	4	1724
chr19	24277395	c	7	2465	3	2139	2	1220	0	436	2	1018	11	1717
chr19	24277396	c	0	2472	3	2140	4	1217	0	436	2	1021	1	1730
chr19	24277403	c	5	2466	1	2143	3	1219	0	437	3	1022	5	1729
chr19	24277409	c	10	2459	6	2137	2	1220	1	436	1	1025	4	1732
chr19	24277416	c	3	2471	3	2142	4	1218	3	434	2	1023	6	1731
chr19	24277417	c	5	2469	7	2138	5	1218	0	437	16	1010	10	1728
chr19	24277420	c	2	2473	5	2140	6	1216	0	437	1	1025	12	1727
chr19	24277425	c	0	2475	0	2145	2	1221	0	437	2	1022	5	1734
chr19	24277433	c	2	2471	0	2146	0	1224	1	436	3	1021	6	1734
chr19	24277441	c	9	2464	4	2140	5	1222	1	436	11	1014	2	1739
chr19	24277451	c	16	2459	0	2147	3	1224	1	436	3	1026	1	1741
chr19	24277455	c	3	2473	32	2116	6	1222	4	433	0	1030	0	1742
chr19	24277465	g	1	3551	4	9777	0	838	0	452	0	731	0	1296
chr19	24277466	c	3	2474	2	2141	4	1226	0	437	2	1029	3	1737
chr19	24277468	g	0	3580	2	9899	0	845	0	454	0	738	0	1312
chr19	24277469	g	0	3608	3	9964	0	849	0	454	0	740	1	1314
chr19	24277472	c	6	2470	109	2039	5	1223	1	436	6	1024	2	1740
chr19	24277474	c	39	2438	1	2146	16	1213	9	427	8	1024	20	1721
chr19	24277476	g	1	3790	9	10448	1	878	1	466	1	772	0	1366
chr19	24277477	c	3	2473	19	2127	3	1226	0	437	2	1028	5	1736
chr19	24277479	g	1	3804	0	10504	0	881	0	469	0	777	1	1372
chr19	24277480	c	4	1410	4	1211	3	685	0	248	0	585	2	975
chr19	24277481	c	3	2113	13	1786	1	1025	2	371	1	866	7	1482
chr19	24277485	c	3	2473	4	2143	4	1226	2	435	3	1032	17	1725
chr19	24277486	c	12	2466	16	2132	3	1227	2	435	3	1032	8	1734
chr19	24277487	c	3	2474	3	2145	1	1229	1	436	2	1032	4	1737
chr19	24277488	c	2	2476	3	2145	1	1229	1	436	1	1034	2	1740
chr19	24277489	c	15	2463	22	2125	2	1228	0	437	3	1032	3	1739
chr19	24277494	c	17	2452	10	2137	3	1225	9	428	3	1029	10	1728
chr19	24277496	c	1492	959	1147	978	742	470	235	201	596	430	846	879
chr19	24277497	g	2268	1574	5768	4781	558	327	277	193	464	313	825	554
chr19	24277502	g	11	3832	65	10487	6	879	1	469	2	775	8	1371
chr19	24277503	c	11	2455	7	2136	7	1220	6	432	5	1028	7	1734
chr19	24277506	c	13	1600	5	1351	6	840	0	301	8	713	17	1082
chr19	24277508	g	16	3832	19	10538	4	881	1	469	2	776	3	1378
chr19	24277510	c	1	570	2	429	1	369	0	132	0	334	1	314

0	3232	0	5213	0	484	0	526	1	806	0	1039
0	3233	0	5215	0	483	0	526	0	805	0	1037
1	3232	1	5214	1	481	1	522	0	802	0	1038
0	3201	0	5169	0	475	0	517	0	793	0	1029
1	2139	0	1867	0	1109	0	371	0	954	0	1605
0	2218	0	1913	0	1121	0	384	0	961	0	1631
0	2249	0	1930	0	1137	0	395	0	964	0	1647
0	2267	0	1943	0	1144	0	399	0	974	0	1658
0	2282	0	1949	0	1160	0	410	0	981	1	1668
5	2471	2	2143	3	1222	3	436	2	1023	4	1728
7	2472	3	2142	2	1222	0	436	2	1020	11	1728
0	2472	3	2143	4	1221	0	436	2	1023	1	1731
5	2471	1	2144	3	1222	0	437	3	1025	5	1734
10	2469	6	2143	2	1222	1	437	1	1026	4	1736
3	2474	3	2145	4	1222	3	437	2	1025	6	1737
5	2474	7	2145	5	1223	0	437	16	1026	10	1738
2	2475	5	2145	6	1222	0	437	1	1026	12	1739
0	2475	0	2145	2	1223	0	437	2	1024	5	1739
2	2473	0	2146	0	1224	1	437	3	1024	6	1740
9	2473	4	2144	5	1227	1	437	11	1025	2	1741
16	2475	0	2147	3	1227	1	437	3	1029	1	1742
3	2476	32	2148	6	1228	4	437	0	1030	0	1742
1	3552	4	9781	0	838	0	452	0	731	0	1296
3	2477	2	2143	4	1230	0	437	2	1031	3	1740
0	3580	2	9901	0	845	0	454	0	738	0	1312
0	3608	3	9967	0	849	0	454	0	740	1	1315
6	2476	109	2148	5	1228	1	437	6	1030	2	1742
39	2477	1	2147	16	1229	9	436	8	1032	20	1741
1	3791	9	10457	1	879	1	467	1	773	0	1366
3	2476	19	2146	3	1229	0	437	2	1030	5	1741
1	3805	0	10504	0	881	0	469	0	777	1	1373
4	1414	4	1215	3	688	0	248	0	585	2	977
3	2116	13	1799	1	1026	2	373	1	867	7	1489
3	2476	4	2147	4	1230	2	437	3	1035	17	1742
12	2478	16	2148	3	1230	2	437	3	1035	8	1742
3	2477	3	2148	1	1230	1	437	2	1034	4	1741
2	2478	3	2148	1	1230	1	437	1	1035	2	1742
15	2478	22	2147	2	1230	0	437	3	1035	3	1742
17	2469	10	2147	3	1228	9	437	3	1032	10	1738
1492	2451	1147	2125	742	1212	235	436	596	1026	846	1725
2268	3842	5768	10549	558	885	277	470	464	777	825	1379
11	3843	65	10552	6	885	1	470	2	777	8	1379
11	2466	7	2143	7	1227	6	438	5	1033	7	1741
13	1613	5	1356	6	846	0	301	8	721	17	1099
16	3848	19	10557	4	885	1	470	2	778	3	1381
1	571	2	431	1	370	0	132	0	334	1	315

chr19	24277511	c	47	509	42	371	38	327	11	114	22	309	24	285
chr19	24277513	g	5	3847	138	10400	0	888	0	472	1	783	0	1387
chr19	24277514	g	8	3844	24	10530	0	888	0	472	3	781	3	1384
chr19	24277516	c	1	494	1	376	2	349	1	115	0	318	0	286
chr19	24277517	c	0	477	1	367	3	338	0	112	0	316	1	280
chr19	24277518	c	0	465	0	362	0	336	0	111	1	314	0	275
chr19	24277520	c	397	51	298	55	278	29	96	7	260	47	215	48
chr19	24277521	g	3175	608	8350	2023	736	136	360	106	665	113	1131	241
chr19	24277523	c	0	445	0	340	0	301	0	98	0	280	0	244
chr19	24277524	c	4	440	1	338	4	295	0	98	2	276	8	233
chr19	24277528	c	374	56	303	32	261	29	86	9	254	19	205	29
chr19	24277529	g	3382	433	8525	1929	758	118	407	61	709	71	1214	162
chr19	24277531	g	9	3844	59	10497	10	879	1	472	3	782	5	1384
chr19	24277532	g	11	3845	24	10537	3	887	0	472	3	783	3	1386
chr19	24277534	c	257	54	190	42	237	29	77	6	218	42	186	20
chr19	24277535	g	3339	507	8582	1958	768	120	391	81	685	99	1155	231
chr19	24277537	c	253	32	142	40	236	28	75	8	220	39	166	40
chr19	24277538	g	3480	377	8676	1883	785	106	394	79	701	83	1173	214
chr19	24277540	G	11	3848	27	10534	3	888	0	474	0	785	3	1386
chr19	24277542	C	0	311	4	203	0	266	0	83	3	255	1	206
chr19	24277545	g	7	3857	15	10550	1	890	2	472	0	786	4	1385
chr19	24277548	g	13	3839	20	10546	4	875	0	465	2	782	8	1377
chr19	24277550	c	280	30	167	43	227	29	70	13	230	19	173	29
chr19	24277551	g	3488	351	9028	1500	788	84	410	53	697	83	1223	155
chr19	24277553	c	3	305	1	211	5	255	4	80	5	246	4	198
chr19	24277555	c	3	303	2	211	0	260	0	88	0	255	0	203
chr19	24277558	g	6	3846	13	10556	0	881	0	465	6	777	1	1383
chr19	24277560	c	379	58	300	48	266	33	89	21	228	43	225	35
chr19	24277561	g	3370	481	8805	1769	782	100	403	62	696	88	1180	205
chr19	24277563	c	538	67	451	75	321	44	121	14	287	43	329	59
chr19	24277564	g	3424	431	8931	1633	777	105	407	57	679	105	1143	243
chr19	24277566	c	5	2455	2	2136	5	1206	2	437	2	996	7	1729
chr19	24277569	c	27	2435	19	2123	33	1178	0	440	31	967	43	1691
chr19	24277573	g	1	3855	86	10478	0	882	0	465	2	781	1	1383
chr19	24277576	g	40	3818	77	10497	13	869	4	461	5	779	16	1369
chr19	24277579	g	38	3820	217	10357	8	874	3	462	9	775	14	1372
chr19	24277582	g	26	3831	15	10559	8	874	1	464	4	780	1	1384
chr19	24277585	g	25	3834	8	10568	5	878	1	464	4	780	5	1382
chr19	24277588	g	59	3805	40	10536	20	864	5	461	4	776	23	1364
chr19	24277589	c	2180	175	1833	216	1099	56	374	52	903	52	1582	64
chr19	24277590	g	3557	306	9386	1192	814	70	420	46	710	70	1242	149
chr19	24277592	c	2279	172	1881	252	1124	73	399	34	927	62	1532	193
chr19	24277593	g	3473	399	9087	1512	780	105	407	60	682	103	1201	190
chr19	24277595	c	2205	238	1851	283	1112	82	379	55	896	84	1515	210
chr19	24277596	g	3487	389	8357	2247	786	101	398	70	709	74	1169	222
chr19	24277598	c	27	2421	14	2113	1	1187	1	433	5	974	22	1699

47	556	42	413	38	365	11	125	22	331	24	309
5	3852	138	10538	0	888	0	472	1	784	0	1387
8	3852	24	10554	0	888	0	472	3	784	3	1387
1	495	1	377	2	351	1	116	0	318	0	286
0	477	1	368	3	341	0	112	0	316	1	281
0	465	0	362	0	336	0	111	1	315	0	275
397	448	298	353	278	307	96	103	260	307	215	263
3175	3783	8350	10373	736	872	360	466	665	778	1131	1372
0	445	0	340	0	301	0	98	0	280	0	244
4	444	1	339	4	299	0	98	2	278	8	241
374	430	303	335	261	290	86	95	254	273	205	234
3382	3815	8525	10454	758	876	407	468	709	780	1214	1376
9	3853	59	10556	10	889	1	473	3	785	5	1389
11	3856	24	10561	3	890	0	472	3	786	3	1389
257	311	190	232	237	266	77	83	218	260	186	206
3339	3846	8582	10540	768	888	391	472	685	784	1155	1386
253	285	142	182	236	264	75	83	220	259	166	206
3480	3857	8676	10559	785	891	394	473	701	784	1173	1387
11	3859	27	10561	3	891	0	474	0	785	3	1389
0	311	4	207	0	266	0	83	3	258	1	207
7	3864	15	10565	1	891	2	474	0	786	4	1389
13	3852	20	10566	4	879	0	465	2	784	8	1385
280	310	167	210	227	256	70	83	230	249	173	202
3488	3839	9028	10528	788	872	410	463	697	780	1223	1378
3	308	1	212	5	260	4	84	5	251	4	202
3	306	2	213	0	260	0	88	0	255	0	203
6	3852	13	10569	0	881	0	465	6	783	1	1384
379	437	300	348	266	299	89	110	228	271	225	260
3370	3851	8805	10574	782	882	403	465	696	784	1180	1385
538	605	451	526	321	365	121	135	287	330	329	388
3424	3855	8931	10564	777	882	407	464	679	784	1143	1386
5	2460	2	2138	5	1211	2	439	2	998	7	1736
27	2462	19	2142	33	1211	0	440	31	998	43	1734
1	3856	86	10564	0	882	0	465	2	783	1	1384
40	3858	77	10574	13	882	4	465	5	784	16	1385
38	3858	217	10574	8	882	3	465	9	784	14	1386
26	3857	15	10574	8	882	1	465	4	784	1	1385
25	3859	8	10576	5	883	1	465	4	784	5	1387
59	3864	40	10576	20	884	5	466	4	780	23	1387
2180	2355	1833	2049	1099	1155	374	426	903	955	1582	1646
3557	3863	9386	10578	814	884	420	466	710	780	1242	1391
2279	2451	1881	2133	1124	1197	399	433	927	989	1532	1725
3473	3872	9087	10599	780	885	407	467	682	785	1201	1391
2205	2443	1851	2134	1112	1194	379	434	896	980	1515	1725
3487	3876	8357	10604	786	887	398	468	709	783	1169	1391
27	2448	14	2127	1	1188	1	434	5	979	22	1721

chr19	24277600	C	30	2413	16	2105	23	1155	0	433	20	956	24	1695
chr19	24277604	C	12	2427	0	2122	21	1160	1	430	3	971	14	1703
chr19	24277606	G	11	3874	32	10578	3	893	0	474	4	793	0	1397
chr19	24277607	G	7	3885	24	10592	2	895	0	474	2	796	1	1397
chr19	24277608	G	14	3879	117	10494	3	895	0	474	5	791	2	1395
chr19	24277610	G	85	3808	403	10211	30	869	10	465	14	784	35	1363
chr19	24277612	C	19	2294	18	2034	8	1071	2	393	19	887	34	1613
chr19	24277614	C	10	2295	56	1991	4	1065	0	393	19	881	23	1618
chr19	24277617	G	3	491	1	1024	1	159	0	141	1	155	0	179
chr19	24277619	C	3	2287	47	1987	1	1045	2	384	7	802	3	1607
chr19	24277622	C	6	2261	54	1969	4	1005	0	378	0	863	10	1593
chr19	24277623	C	1	2264	5	2017	7	1003	0	379	2	860	0	1602
chr19	24277625	C	11	2246	11	2006	1	1007	2	370	5	856	6	1592
chr19	24277627	G	1	270	0	419	0	105	1	100	3	104	0	114
chr19	24277631	C	4	2235	8	1998	6	985	1	369	0	851	5	1582
chr19	24277635	C	5	2230	1	2004	8	976	2	368	1	775	4	1568
chr19	24277636	C	6	2225	24	1981	21	961	8	362	6	769	40	1531
chr19	24277641	C	1	2214	6	1982	2	971	4	362	1	771	5	1559
chr19	24277642	C	77	2135	3	1982	57	915	20	344	58	713	70	1494
chr19	24277644	C	190	2018	186	1797	107	860	8	357	110	662	37	1525
chr19	24277653	C	9	2195	44	1936	3	960	0	366	2	768	2	1559
chr19	24277654	C	12	2192	7	1975	2	959	1	365	8	762	13	1547
chr19	24277664	G	50	3733	199	10262	10	803	1	349	15	702	9	1366
chr19	24277668	G	17	3761	20	10420	0	812	13	334	5	711	9	1366
chr19	24277674	G	152	3626	251	10176	13	799	9	336	22	694	10	1365
chr19	24277675	G	8	3770	20	10405	3	808	3	343	8	708	2	1373
chr19	24277677	C	0	2201	3	1978	2	960	1	364	1	768	1	1558
chr19	24277681	G	23	3755	119	10295	4	808	10	336	5	708	7	1366
chr19	24277686	G	32	3747	85	10332	8	803	14	331	2	712	9	1364
chr19	24277690	C	1937	265	1749	224	868	94	323	42	688	82	1405	152
chr19	24277691	G	3324	451	9216	1202	688	117	301	38	618	91	1166	202
chr19	24277692	C	0	2171	0	1947	1	959	0	363	0	767	1	1549
chr19	24277694	C	0	2175	0	1951	0	962	0	364	0	769	0	1555
chr19	24277696	G	20	3755	47	10361	3	801	0	339	7	702	5	1360
chr19	24277698	G	6	3761	81	10304	2	801	0	338	2	705	5	1358
chr19	24277699	C	0	2173	0	1946	0	962	0	361	1	768	0	1555
chr19	24277702	G	14	3754	13	10389	5	799	1	338	3	705	7	1357
chr19	24277705	G	27	3721	410	9989	20	771	3	323	11	693	20	1335
chr19	24277707	G	115	3634	483	9914	22	770	5	321	11	691	32	1320
chr19	24277708	G	13	3736	17	10379	2	790	1	325	5	698	3	1350
chr19	24277709	G	5	3744	14	10386	0	791	0	326	0	704	3	1351
chr19	24277713	C	0	1929	0	1687	0	878	0	314	0	709	0	1411
chr19	24277714	C	0	1896	1	1654	0	864	0	310	0	703	0	1380
chr19	24277726	G	16	3728	14	10373	4	787	1	322	1	699	4	1346
chr19	24277731	G	12	3731	25	10363	3	788	0	323	4	696	2	1348
chr19	24277732	G	9	3731	16	10372	1	788	0	321	8	691	2	1338

30	2443	16	2121	23	1178	0	433	20	976	24	1719
12	2439	0	2122	21	1181	1	431	3	974	14	1717
11	3885	32	10610	3	896	0	474	4	797	0	1397
7	3892	24	10616	2	897	0	474	2	798	1	1398
14	3893	117	10611	3	898	0	474	5	796	2	1397
85	3893	403	10614	30	899	10	475	14	798	35	1398
19	2313	18	2052	8	1079	2	395	19	906	34	1647
10	2305	56	2047	4	1069	0	393	19	900	23	1641
3	494	1	1025	1	160	0	141	1	156	0	179
3	2290	47	2034	1	1046	2	386	7	809	3	1610
6	2267	54	2023	4	1009	0	378	0	863	10	1603
1	2265	5	2022	7	1010	0	379	2	862	0	1602
11	2257	11	2017	1	1008	2	372	5	861	6	1598
1	271	0	419	0	105	1	101	3	107	0	114
4	2239	8	2006	6	991	1	370	0	851	5	1587
5	2235	1	2005	8	984	2	370	1	776	4	1572
6	2231	24	2005	21	982	8	370	6	775	40	1571
1	2215	6	1988	2	973	4	366	1	772	5	1564
77	2212	3	1985	57	972	20	364	58	771	70	1564
190	2208	186	1983	107	967	8	365	110	772	37	1562
9	2204	44	1980	3	963	0	366	2	770	2	1561
12	2204	7	1982	2	961	1	366	8	770	13	1560
50	3783	199	10461	10	813	1	350	15	717	9	1375
17	3778	20	10440	0	812	13	347	5	716	9	1375
152	3778	251	10427	13	812	9	345	22	716	10	1375
8	3778	20	10425	3	811	3	346	8	716	2	1375
0	2201	3	1981	2	962	1	365	1	769	1	1559
23	3778	119	10414	4	812	10	346	5	713	7	1373
32	3779	85	10417	8	811	14	345	2	714	9	1373
1937	2202	1749	1973	868	962	323	365	688	770	1405	1557
3324	3775	9216	10418	688	805	301	339	618	709	1166	1368
0	2171	0	1947	1	960	0	363	0	767	1	1550
0	2175	0	1951	0	962	0	364	0	769	0	1555
20	3775	47	10408	3	804	0	339	7	709	5	1365
6	3767	81	10385	2	803	0	338	2	707	5	1363
0	2173	0	1946	0	962	0	361	1	769	0	1555
14	3768	13	10402	5	804	1	339	3	708	7	1364
27	3748	410	10399	20	791	3	326	11	704	20	1355
115	3749	483	10397	22	792	5	326	11	702	32	1352
13	3749	17	10396	2	792	1	326	5	703	3	1353
5	3749	14	10400	0	791	0	326	0	704	3	1354
0	1929	0	1687	0	878	0	314	0	709	0	1411
0	1896	1	1655	0	864	0	310	0	703	0	1380
16	3744	14	10387	4	791	1	323	1	700	4	1350
12	3743	25	10388	3	791	0	323	4	700	2	1350
9	3740	16	10388	1	789	0	321	8	699	2	1340

chr19	24277739	g	3512	228	9144	1243	746	45	294	29	645	54	1195	152
chr19	24277741	g	3572	162	9628	756	752	33	307	12	663	33	1251	78
chr19	24277745	g	94	3637	157	10206	12	772	7	312	16	678	41	1287
chr19	24277754	g	31	3699	6	10369	7	778	5	313	9	687	26	1302
chr19	24277757	g	12	3716	7	10366	8	777	5	313	3	693	0	1326
chr19	24277758	g	7	3717	12	10361	1	782	0	318	0	696	1	1324
chr19	24277761	g	21	3705	15	10357	4	781	1	317	4	691	5	1320
chr19	24277775	g	1	3715	1	10364	0	779	0	313	0	693	0	1318
chr19	24277778	g	1	3719	1	10355	0	784	0	318	0	694	0	1322
chr19	24277779	g	0	3722	2	10353	0	785	0	318	0	694	0	1321
chr19	24277783	g	2	3717	3	10348	0	784	0	318	1	693	0	1323
chr4	136547319	G	1	6726	1	7766	0	2143	0	2337	0	5030	0	2612
chr4	136547320	G	1	6737	3	7794	1	2145	0	2342	0	5037	1	2613
chr4	136547321	G	2	6753	1	7808	2	2145	0	2347	1	5039	0	2622
chr4	136547327	G	1	6789	3	7866	1	2154	0	2369	3	5055	0	2630
chr4	136547332	G	3	6788	2	7877	1	2156	0	2367	1	5064	0	2638
chr4	136547333	G	0	6795	1	7883	0	2158	0	2369	0	5067	1	2639
chr4	136547338	G	13	6830	7	7966	4	2158	4	2371	14	5056	8	2634
chr4	136547340	G	18	6832	91	7893	4	2159	6	2369	6	5065	4	2640
chr4	136547344	G	30	6826	44	7950	3	2160	7	2370	10	5063	16	2629
chr4	136547349	G	21	6842	13	7985	5	2165	1	2380	5	5077	5	2650
chr4	136547354	G	65	6799	12	7991	17	2153	11	2370	45	5037	31	2622
chr4	136547358	G	28	6837	21	7986	4	2166	2	2379	26	5057	8	2647
chr4	136547359	G	29	6836	8	7999	1	2169	2	2380	19	5064	2	2651
chr4	136547364	G	8	6856	19	7985	3	2167	2	2382	15	5062	5	2653
chr4	136547365	G	11	6858	12	7997	7	2163	2	2381	7	5074	6	2648
chr4	136547374	G	11	6890	56	7963	4	2189	5	2404	39	5109	1	2698
chr4	136547376	G	44	6857	484	7538	5	2188	19	2387	38	5109	39	2658
chr4	136547377	C	2	2244	0	1898	0	562	0	304	0	521	0	1174
chr4	136547378	C	1	2253	0	1912	0	561	0	304	0	530	0	1182
chr4	136547381	G	18	6885	20	8000	4	2190	9	2403	6	5143	11	2690
chr4	136547383	G	521	6385	840	7183	95	2101	117	2295	408	4743	158	2544
chr4	136547385	G	161	6745	216	7807	57	2140	40	2372	163	4990	68	2635
chr4	136547386	G	54	6852	10	8019	5	2192	3	2409	18	5133	9	2694
chr4	136547387	C	1	2293	1	1949	0	567	0	305	0	530	0	1189
chr4	136547388	C	0	2300	0	1953	0	566	0	309	0	533	0	1192
chr4	136547390	G	60	6837	6	8016	11	2183	32	2379	46	5099	39	2663
chr4	136547392	G	11	6901	23	8007	5	2192	4	2406	15	5138	7	2698
chr4	136547393	G	28	6883	22	8005	6	2191	38	2372	11	5144	8	2696
chr4	136547394	C	2	2302	0	1955	0	567	0	309	0	533	0	1195
chr4	136547396	G	196	6718	317	7712	51	2143	135	2276	227	4927	136	2570
chr4	136547399	G	42	6872	45	7985	36	2161	20	2392	47	5108	30	2677
chr4	136547402	G	186	6730	651	7382	55	2142	51	2361	155	4999	99	2606
chr4	136547405	G	132	6792	429	7609	22	2175	59	2354	107	5048	43	2664
chr4	136547408	C	2118	186	1766	189	526	41	297	12	511	22	1047	149
chr4	136547409	G	6696	242	7738	316	2142	61	2318	101	5012	146	2604	106

3512	3740	9144	10387	746	791	294	323	645	699	1195	1347
3572	3734	9628	10384	752	785	307	319	663	696	1251	1329
94	3731	157	10363	12	784	7	319	16	694	41	1328
31	3730	6	10375	7	785	5	318	9	696	26	1328
12	3728	7	10373	8	785	5	318	3	696	0	1326
7	3724	12	10373	1	783	0	318	0	696	1	1325
21	3726	15	10372	4	785	1	318	4	695	5	1325
1	3716	1	10365	0	779	0	313	0	693	0	1318
1	3720	1	10356	0	784	0	318	0	694	0	1322
0	3722	2	10355	0	785	0	318	0	694	0	1321
2	3719	3	10351	0	784	0	318	1	694	0	1323
1	6727	1	7767	0	2143	0	2337	0	5030	0	2612
1	6738	3	7797	1	2146	0	2342	0	5037	1	2614
2	6755	1	7809	2	2147	0	2347	1	5040	0	2622
1	6790	3	7869	1	2155	0	2369	3	5058	0	2630
3	6791	2	7879	1	2157	0	2367	1	5065	0	2638
0	6795	1	7884	0	2158	0	2369	0	5067	1	2640
13	6843	7	7973	4	2162	4	2375	14	5070	8	2642
18	6850	91	7984	4	2163	6	2375	6	5071	4	2644
30	6856	44	7994	3	2163	7	2377	10	5073	16	2645
21	6863	13	7998	5	2170	1	2381	5	5082	5	2655
65	6864	12	8003	17	2170	11	2381	45	5082	31	2653
28	6865	21	8007	4	2170	2	2381	26	5083	8	2655
29	6865	8	8007	1	2170	2	2382	19	5083	2	2653
8	6864	19	8004	3	2170	2	2384	15	5077	5	2658
11	6869	12	8009	7	2170	2	2383	7	5081	6	2654
11	6901	56	8019	4	2193	5	2409	39	5148	1	2699
44	6901	484	8022	5	2193	19	2406	38	5147	39	2697
2	2246	0	1898	0	562	0	304	0	521	0	1174
1	2254	0	1912	0	561	0	304	0	530	0	1182
18	6903	20	8020	4	2194	9	2412	6	5149	11	2701
521	6906	840	8023	95	2196	117	2412	408	5151	158	2702
161	6906	216	8023	57	2197	40	2412	163	5153	68	2703
54	6906	10	8029	5	2197	3	2412	18	5151	9	2703
1	2294	1	1950	0	567	0	305	0	530	0	1189
0	2300	0	1953	0	566	0	309	0	533	0	1192
60	6897	6	8022	11	2194	32	2411	46	5145	39	2702
11	6912	23	8030	5	2197	4	2410	15	5153	7	2705
28	6911	22	8027	6	2197	38	2410	11	5155	8	2704
2	2304	0	1955	0	567	0	309	0	533	0	1195
196	6914	317	8029	51	2194	135	2411	227	5154	136	2706
42	6914	45	8030	36	2197	20	2412	47	5155	30	2707
186	6916	651	8033	55	2197	51	2412	155	5154	99	2705
132	6924	429	8038	22	2197	59	2413	107	5155	43	2707
2118	2304	1766	1955	526	567	297	309	511	533	1047	1196
6696	6938	7738	8054	2142	2203	2318	2419	5012	5158	2604	2710

chr4	136547412	C	5	2298	3	1952	2	564	0	309	9	524	1	1195
chr4	136547414	G	1313	5636	1897	6166	406	1800	397	2028	1013	4148	570	2141
chr4	136547416	G	95	6817	243	7787	38	2154	63	2350	138	4988	90	2594
chr4	136547417	G	13	6937	12	8056	2	2202	3	2421	13	5146	27	2685
chr4	136547420	G	6	6947	12	8058	0	2210	8	2418	6	5160	2	2713
chr4	136547421	G	23	6926	31	8042	5	2205	5	2421	10	5157	16	2699
chr4	136547423	G	980	5973	1242	6832	207	2005	242	2187	705	4462	248	2468
chr4	136547425	G	595	6355	1081	6962	230	1985	345	2087	643	4518	329	2384
chr4	136547426	C	11	2286	30	1927	1	566	1	308	1	532	13	1183
chr4	136547428	G	10	6956	14	8069	10	2208	4	2442	22	5151	4	2715
chr4	136547429	G	17	6948	12	8071	9	2211	3	2448	11	5162	1	2715
chr4	136547431	G	637	6338	723	7366	142	2080	210	2248	464	4712	244	2474
chr4	136547432	G	10	6970	7	8085	2	2224	1	2462	14	5167	5	2715
chr4	136547435	G	292	6674	867	7188	90	2134	139	2324	220	4946	76	2628
chr4	136547436	G	11	6987	29	8080	7	2228	6	2473	8	5184	3	2721
chr4	136547438	G	106	6894	72	8044	17	2222	82	2402	65	5131	42	2682
chr4	136547440	G	20	7008	96	8037	1	2248	1	2510	5	5214	5	2725
chr4	136547441	G	11	7042	10	8153	3	2268	4	2540	9	5235	3	2733
chr4	136547442	C	7	2297	3	1954	1	567	1	308	2	532	2	1195
chr4	136547443	C	2010	293	1642	316	500	68	288	21	452	83	1048	149
chr4	136547444	G	6716	372	7730	452	2137	162	2385	178	4941	315	2550	193
chr4	136547447	G	14	7080	5	8183	1	2303	10	2565	5	5265	3	2743
chr4	136547448	G	25	7068	12	8178	6	2298	4	2573	8	5261	5	2741
chr4	136547449	C	2	2300	10	1947	0	568	1	309	0	535	1	1196
chr4	136547452	G	9	7114	6	8206	6	2335	4	2619	5	5306	5	2760
chr4	136547453	C	4	2299	2	1958	0	568	0	310	3	532	1	1196
chr4	136547454	C	2177	127	1673	287	510	58	299	11	516	19	1119	78
chr4	136547455	G	6874	283	7431	799	2207	158	2464	200	5071	283	2621	153
chr4	136547456	G	21	7139	8	8229	5	2366	23	2648	23	5340	21	2761
chr4	136547459	G	9	7150	59	8176	3	2367	8	2665	25	5332	8	2757
chr4	136547460	C	6	2205	3	1911	0	548	0	300	0	506	3	1148
chr4	136547461	C	3	2301	2	1960	1	567	0	310	0	535	0	1198
chr4	136547462	C	3	2301	2	1960	1	567	0	310	1	534	16	1181
chr4	136547466	C	0	2303	3	1958	0	568	0	309	1	534	1	1197
chr4	136547467	C	3	2303	1	1960	3	564	0	310	2	533	17	1181
chr4	136547469	C	179	2126	46	1916	34	534	3	307	11	524	34	1162
chr4	136547471	G	22	7197	31	8258	5	2448	6	2736	9	5638	8	2839
chr4	136547473	C	5	2299	4	1957	0	567	1	307	1	534	0	1198
chr4	136547474	C	23	2283	1	1963	0	568	0	310	2	533	2	1196
chr4	136547475	C	12	2294	0	1964	0	567	0	310	3	532	0	1197
chr4	136547478	C	93	2214	3	1961	34	534	15	295	11	525	53	1145
chr4	136547480	G	15	7199	13	8271	7	2444	19	2719	26	5610	6	2839
chr4	136547482	C	11	2296	2	1962	0	568	0	310	2	534	0	1198
chr4	136547487	G	16	7190	5	8273	10	2440	2	2736	14	5620	4	2842
chr4	136547488	G	8	7198	20	8257	2	2446	9	2730	17	5616	1	2842
chr4	136547489	C	11	2288	12	1945	1	566	2	308	0	535	4	1195

5	2303	3	1955	2	566	0	309	9	533	1	1196
1313	6949	1897	8063	406	2206	397	2425	1013	5161	570	2711
95	6912	243	8030	38	2192	63	2413	138	5126	90	2684
13	6950	12	8068	2	2204	3	2424	13	5159	27	2712
6	6953	12	8070	0	2210	8	2426	6	5166	2	2715
23	6949	31	8073	5	2210	5	2426	10	5167	16	2715
980	6953	1242	8074	207	2212	242	2429	705	5167	248	2716
595	6950	1081	8043	230	2215	345	2432	643	5161	329	2713
11	2297	30	1957	1	567	1	309	1	533	13	1196
10	6966	14	8083	10	2218	4	2446	22	5173	4	2719
17	6965	12	8083	9	2220	3	2451	11	5173	1	2716
637	6975	723	8089	142	2222	210	2458	464	5176	244	2718
10	6980	7	8092	2	2226	1	2463	14	5181	5	2720
292	6966	867	8055	90	2224	139	2463	220	5166	76	2704
11	6998	29	8109	7	2235	6	2479	8	5192	3	2724
106	7000	72	8116	17	2239	82	2484	65	5196	42	2724
20	7028	96	8133	1	2249	1	2511	5	5219	5	2730
11	7053	10	8163	3	2271	4	2544	9	5244	3	2736
7	2304	3	1957	1	568	1	309	2	534	2	1197
2010	2303	1642	1958	500	568	288	309	452	535	1048	1197
6716	7088	7730	8182	2137	2299	2385	2563	4941	5256	2550	2743
14	7094	5	8188	1	2304	10	2575	5	5270	3	2746
25	7093	12	8190	6	2304	4	2577	8	5269	5	2746
2	2302	10	1957	0	568	1	310	0	535	1	1197
9	7123	6	8212	6	2341	4	2623	5	5311	5	2765
4	2303	2	1960	0	568	0	310	3	535	1	1197
2177	2304	1673	1960	510	568	299	310	516	535	1119	1197
6874	7157	7431	8230	2207	2365	2464	2664	5071	5354	2621	2774
21	7160	8	8237	5	2371	23	2671	23	5363	21	2782
9	7159	59	8235	3	2370	8	2673	25	5357	8	2765
6	2211	3	1914	0	548	0	300	0	506	3	1151
3	2304	2	1962	1	568	0	310	0	535	0	1198
3	2304	2	1962	1	568	0	310	1	535	16	1197
0	2303	3	1961	0	568	0	309	1	535	1	1198
3	2306	1	1961	3	567	0	310	2	535	17	1198
179	2305	46	1962	34	568	3	310	11	535	34	1196
22	7219	31	8289	5	2453	6	2742	9	5647	8	2847
5	2304	4	1961	0	567	1	308	1	535	0	1198
23	2306	1	1964	0	568	0	310	2	535	2	1198
12	2306	0	1964	0	567	0	310	3	535	0	1197
93	2307	3	1964	34	568	15	310	11	536	53	1198
15	7214	13	8284	7	2451	19	2738	26	5636	6	2845
11	2307	2	1964	0	568	0	310	2	536	0	1198
16	7206	5	8278	10	2450	2	2738	14	5634	4	2846
8	7206	20	8277	2	2448	9	2739	17	5633	1	2843
11	2299	12	1957	1	567	2	310	0	535	4	1199

chr4	136547492	G	164	7043	37	8243	25	2423	16	2723	76	5557	63	2780
chr4	136547494	C	8	2299	3	1963	1	570	0	311	3	535	1	1201
chr4	136547495	C	13	2296	4	1961	8	563	2	309	1	537	5	1196
chr4	136547496	C	2	2306	1	1964	0	571	11	300	1	537	0	1202
chr4	136547497	C	2158	149	1802	164	524	45	299	12	476	61	1121	81
chr4	136547498	G	6764	436	7674	599	2273	169	2476	261	5210	421	2580	261
chr4	136547499	G	12	7184	13	8259	17	2425	1	2735	10	5618	11	2831
chr4	136547501	G	386	6811	954	7316	158	2284	148	2587	444	5184	231	2609
chr4	136547503	G	126	7065	196	8070	16	2427	16	2718	67	5560	21	2819
chr4	136547504	G	11	7184	12	8256	0	2443	7	2728	13	5616	5	2835
chr4	136547505	G	18	7178	11	8258	7	2435	7	2728	4	5625	4	2835
chr4	136547507	C	7	2305	4	1962	2	570	0	311	1	538	8	1194
chr4	136547508	C	11	2301	2	1964	0	572	9	302	1	538	0	1203
chr4	136547510	C	36	2276	1	1965	7	565	0	311	0	538	6	1197
chr4	136547512	C	7	2305	3	1963	1	571	2	308	1	538	1	1202
chr4	136547515	G	10	7173	5	8252	6	2435	4	2726	8	5617	10	2825
chr4	136547516	C	15	2297	7	1961	4	568	4	307	1	538	5	1198
chr4	136547517	C	2173	140	1822	146	525	47	289	22	516	23	1009	193
chr4	136547518	G	6585	598	7550	714	2268	172	2465	268	5214	410	2568	268
chr4	136547519	G	34	7149	12	8251	2	2439	9	2724	5	5621	3	2832
chr4	136547520	G	28	7156	16	8245	9	2431	14	2717	16	5608	6	2829
chr4	136547524	G	10	7167	18	8240	3	2436	4	2726	26	5596	6	2828
chr4	136547525	C	2	295	1	270	0	120	1	76	0	104	0	174
chr4	136547527	C	147	9	142	17	74	5	56	4	71	1	92	8
chr4	136547528	G	6785	395	7487	768	2303	138	2566	166	5257	365	2706	128
chr4	136547531	C	126	11	123	10	71	7	54	5	63	2	77	8
chr4	136547532	G	6755	424	7956	298	2321	120	2583	150	5424	197	2596	237
chr4	136547534	C	0	116	0	121	0	77	0	56	0	65	0	83
chr4	136547536	G	146	7030	65	8184	54	2384	39	2694	72	5547	45	2783
chr4	136547537	G	11	7165	20	8224	7	2432	4	2729	5	5617	3	2830
chr4	136547538	G	23	7153	53	8197	3	2437	4	2729	10	5611	5	2828
chr4	136547539	C	111	3	116	6	68	1	55	3	57	6	73	7
chr4	136547540	G	6910	264	7784	464	2335	104	2550	182	5333	289	2699	134
chr4	136547543	C	0	113	0	121	0	69	0	58	4	62	0	82
chr4	136547545	C	105	7	121	1	62	5	56	1	63	2	70	10
chr4	136547546	G	6835	338	7753	496	2340	96	2494	237	5382	236	2690	142
chr4	136547547	G	22	7144	19	8218	2	2431	4	2726	9	5604	5	2822
chr4	136547548	C	107	5	115	7	65	0	55	2	63	1	78	4
chr4	136547549	G	6754	420	7782	466	2304	132	2620	111	5402	218	2729	102
chr4	136547551	C	0	112	0	123	0	62	0	51	0	58	0	80
chr4	136547553	C	1	108	0	122	1	57	1	47	1	54	0	80
chr4	136547555	G	27	7145	58	8186	12	2424	6	2723	30	5587	12	2815
chr4	136547558	C	3	108	2	118	0	53	2	41	7	49	0	78
chr4	136547560	G	135	7035	70	8171	25	2410	9	2720	109	5506	82	2742
chr4	136547561	G	11	7156	12	8226	6	2429	3	2725	32	5580	15	2810
chr4	136547562	C	0	120	0	134	1	55	1	50	0	59	0	79

164	7207	37	8280	25	2448	16	2739	76	5633	63	2843
8	2307	3	1966	1	571	0	311	3	538	1	1202
13	2309	4	1965	8	571	2	311	1	538	5	1201
2	2308	1	1965	0	571	11	311	1	538	0	1202
2158	2307	1802	1966	524	569	299	311	476	537	1121	1202
6764	7200	7674	8273	2273	2442	2476	2737	5210	5631	2580	2841
12	7196	13	8272	17	2442	1	2736	10	5628	11	2842
386	7197	954	8270	158	2442	148	2735	444	5628	231	2840
126	7191	196	8266	16	2443	16	2734	67	5627	21	2840
11	7195	12	8268	0	2443	7	2735	13	5629	5	2840
18	7196	11	8269	7	2442	7	2735	4	5629	4	2839
7	2312	4	1966	2	572	0	311	1	539	8	1202
11	2312	2	1966	0	572	9	311	1	539	0	1203
36	2312	1	1966	7	572	0	311	0	538	6	1203
7	2312	3	1966	1	572	2	310	1	539	1	1203
10	7183	5	8257	6	2441	4	2730	8	5625	10	2835
15	2312	7	1968	4	572	4	311	1	539	5	1203
2173	2313	1822	1968	525	572	289	311	516	539	1009	1202
6585	7183	7550	8264	2268	2440	2465	2733	5214	5624	2568	2836
34	7183	12	8263	2	2441	9	2733	5	5626	3	2835
28	7184	16	8261	9	2440	14	2731	16	5624	6	2835
10	7177	18	8258	3	2439	4	2730	26	5622	6	2834
2	297	1	271	0	120	1	77	0	104	0	174
147	156	142	159	74	79	56	60	71	72	92	100
6785	7180	7487	8255	2303	2441	2566	2732	5257	5622	2706	2834
126	137	123	133	71	78	54	59	63	65	77	85
6755	7179	7956	8254	2321	2441	2583	2733	5424	5621	2596	2833
0	116	0	121	0	77	0	56	0	65	0	83
146	7176	65	8249	54	2438	39	2733	72	5619	45	2828
11	7176	20	8244	7	2439	4	2733	5	5622	3	2833
23	7176	53	8250	3	2440	4	2733	10	5621	5	2833
111	114	116	122	68	69	55	58	57	63	73	80
6910	7174	7784	8248	2335	2439	2550	2732	5333	5622	2699	2833
0	113	0	121	0	69	0	58	4	66	0	82
105	112	121	122	62	67	56	57	63	65	70	80
6835	7173	7753	8249	2340	2436	2494	2731	5382	5618	2690	2832
22	7166	19	8237	2	2433	4	2730	9	5613	5	2827
107	112	115	122	65	65	55	57	63	64	78	82
6754	7174	7782	8248	2304	2436	2620	2731	5402	5620	2729	2831
0	112	0	123	0	62	0	51	0	58	0	80
1	109	0	122	1	58	1	48	1	55	0	80
27	7172	58	8244	12	2436	6	2729	30	5617	12	2827
3	111	2	120	0	53	2	43	7	56	0	78
135	7170	70	8241	25	2435	9	2729	109	5615	82	2824
11	7167	12	8238	6	2435	3	2728	32	5612	15	2825
0	120	0	134	1	56	1	51	0	59	0	79

chr4	136547564	C	111	9	116	16	56	0	46	4	53	5	77	3
chr4	136547565	G	6856	303	7623	614	2303	131	2606	123	5405	209	2685	138
chr4	136547566	C	104	16	123	15	56	0	47	5	56	1	80	5
chr4	136547567	G	6945	214	7796	441	2328	106	2575	153	5420	192	2740	82
chr4	136547570	C	0	135	0	142	0	57	0	52	0	57	2	89
chr4	136547576	C	0	142	0	146	0	55	0	48	0	59	0	90
chr4	136547577	C	0	143	0	147	0	55	0	48	0	60	0	89
chr4	136547578	C	2	142	1	146	1	54	0	48	0	60	0	90
chr4	136547580	G	40	7093	227	7990	34	2392	4	2719	58	5539	25	2786
chr4	136547581	C	2	161	0	169	0	56	0	55	0	72	0	108
chr4	136547585	C	155	12	168	11	55	0	53	8	74	6	107	6
chr4	136547586	G	6307	822	6972	1231	2152	271	2480	244	5004	592	2566	243
chr4	136547587	C	4	164	2	176	0	55	0	61	0	80	0	113
chr4	136547589	C	10	161	7	170	2	52	2	59	0	81	1	113
chr4	136547591	G	1	7105	1	8170	1	2413	1	2706	2	5580	0	2802
chr4	136547592	G	0	7104	3	8165	0	2412	0	2705	0	5576	0	2799
chr4	136547594	G	0	7094	1	8157	0	2406	0	2702	1	5565	0	2792
chr4	136547596	G	0	7089	1	8153	1	2407	0	2702	1	5562	0	2790
chr4	136547598	G	1	7076	0	8139	0	2403	0	2700	0	5561	0	2789
chr4	136547600	G	0	7076	1	8131	0	2404	1	2698	1	5557	1	2783
chr4	136547602	G	0	7074	0	8131	0	2404	2	2696	0	5553	1	2785
chr4	136547604	G	2	7058	3	8111	0	2402	0	2697	3	5537	1	2774
chr4	136547605	C	2	142	0	155	0	35	0	57	0	66	0	100
chr4	136547607	C	2	134	5	143	1	25	0	50	0	60	3	87
chr4	136547611	C	0	133	0	145	0	21	0	50	1	56	0	88
chr4	136547612	C	0	133	0	145	0	21	0	50	0	57	0	87
chr4	136547616	C	0	147	0	161	0	22	0	50	0	58	0	91
chr4	136547618	C	0	149	0	165	0	22	0	50	0	61	0	91
chr4	136547624	C	5	149	0	159	1	21	0	51	0	61	1	84
chr4	136547626	C	2	149	0	158	1	20	2	49	0	59	4	79
chr4	136547628	C	0	151	0	158	0	21	0	51	0	57	0	84
chr4	136547629	C	2	150	0	159	0	21	1	50	0	58	0	84
chr4	136547632	C	4	155	2	162	0	23	0	55	0	59	0	89
chr4	136547636	C	1	291	0	279	1	55	0	71	2	86	0	180
chr4	136547641	C	4	2244	4	1881	5	489	0	263	0	486	3	1140
chr4	136547642	C	2	2247	2	1882	0	493	0	262	2	484	0	1143
chr4	136547645	C	6	2238	0	1881	0	493	4	257	1	485	3	1139
chr4	136547653	C	45	2197	51	1829	4	490	1	260	5	480	14	1126
chr4	136547655	C	57	2184	41	1837	10	484	9	252	11	474	66	1072
chr4	136547657	C	56	2184	89	1790	13	480	0	260	46	438	27	1111
chr4	136547659	C	5	2231	0	1877	1	493	0	259	1	483	2	1135
chr4	136547660	C	0	2241	9	1871	4	490	1	258	0	484	1	1137
chr4	136547671	C	9	2231	2	1874	1	493	0	259	5	479	2	1136
chr4	136547673	C	15	2224	1	1871	5	489	2	257	1	482	22	1115
chr4	136547677	C	2	2237	17	1857	0	494	1	258	5	478	2	1136
chr4	136547684	C	47	2188	1	1865	15	479	0	256	3	479	2	1131

111	120	116	132	56	56	46	50	53	58	77	80
6856	7159	7623	8237	2303	2434	2606	2729	5405	5614	2685	2823
104	120	123	138	56	56	47	52	56	57	80	85
6945	7159	7796	8237	2328	2434	2575	2728	5420	5612	2740	2822
0	135	0	142	0	57	0	52	0	57	2	91
0	142	0	146	0	55	0	48	0	59	0	90
0	143	0	147	0	55	0	48	0	60	0	89
2	144	1	147	1	55	0	48	0	60	0	90
40	7133	227	8217	34	2426	4	2723	58	5597	25	2811
2	163	0	169	0	56	0	55	0	72	0	108
155	167	168	179	55	55	53	61	74	80	107	113
6307	7129	6972	8203	2152	2423	2480	2724	5004	5596	2566	2809
4	168	2	178	0	55	0	61	0	80	0	113
10	171	7	177	2	54	2	61	0	81	1	114
1	7106	1	8171	1	2414	1	2707	2	5582	0	2802
0	7104	3	8168	0	2412	0	2705	0	5576	0	2799
0	7094	1	8158	0	2406	0	2702	1	5566	0	2792
0	7089	1	8154	1	2408	0	2702	1	5563	0	2790
1	7077	0	8139	0	2403	0	2700	0	5561	0	2789
0	7076	1	8132	0	2404	1	2699	1	5558	1	2784
0	7074	0	8131	0	2404	2	2698	0	5553	1	2786
2	7060	3	8114	0	2402	0	2697	3	5540	1	2775
2	144	0	155	0	35	0	57	0	66	0	100
2	136	5	148	1	26	0	50	0	60	3	90
0	133	0	145	0	21	0	50	1	57	0	88
0	133	0	145	0	21	0	50	0	57	0	87
0	147	0	161	0	22	0	50	0	58	0	91
0	149	0	165	0	22	0	50	0	61	0	91
5	154	0	159	1	22	0	51	0	61	1	85
2	151	0	158	1	21	2	51	0	59	4	83
0	151	0	158	0	21	0	51	0	57	0	84
2	152	0	159	0	21	1	51	0	58	0	84
4	159	2	164	0	23	0	55	0	59	0	89
1	292	0	279	1	56	0	71	2	88	0	180
4	2248	4	1885	5	494	0	263	0	486	3	1143
2	2249	2	1884	0	493	0	262	2	486	0	1143
6	2244	0	1881	0	493	4	261	1	486	3	1142
45	2242	51	1880	4	494	1	261	5	485	14	1140
57	2241	41	1878	10	494	9	261	11	485	66	1138
56	2240	89	1879	13	493	0	260	46	484	27	1138
5	2236	0	1877	1	494	0	259	1	484	2	1137
0	2241	9	1880	4	494	1	259	0	484	1	1138
9	2240	2	1876	1	494	0	259	5	484	2	1138
15	2239	1	1872	5	494	2	259	1	483	22	1137
2	2239	17	1874	0	494	1	259	5	483	2	1138
47	2235	1	1866	15	494	0	256	3	482	2	1133

chr4	136547689	C	26	2207	3	1856	6	488	1	254	14	467	1	1133
chr4	136547690	C	8	2226	6	1859	1	493	0	256	2	480	1	1132
chr4	136547692	C	48	2187	0	1865	19	474	11	245	0	481	2	1127
chr4	136547696	C	2	2232	2	1862	0	494	1	255	2	479	0	1133
chr4	136547711	C	3	2217	0	1849	1	491	0	251	1	477	2	1127
chr4	136547720	C	19	2174	5	1808	2	483	0	235	1	470	3	1108
chr4	136547722	C	16	2177	52	1762	2	482	3	233	8	459	1	1108
chr4	136547725	C	11	2175	4	1806	8	475	5	230	0	466	41	1066
chr4	136547729	C	2012	164	1593	204	452	28	221	12	439	25	1005	95
chr4	136547731	C	33	2144	75	1722	10	473	6	228	43	418	1	1099
chr4	136547733	C	38	2122	48	1731	14	467	17	207	8	441	33	1052
chr4	136547737	C	31	2124	9	1765	2	479	0	221	1	445	6	1074
chr4	136547738	C	3	2153	4	1770	3	476	0	221	4	442	1	1079
chr4	136547740	C	35	2118	1	1774	6	475	0	220	20	425	8	1072
chr4	136547742	C	75	2077	49	1724	28	453	4	215	11	434	46	1034
chr4	136547746	C	13	2136	2	1772	0	481	1	218	2	443	1	1077
chr4	136547748	C	89	2064	24	1749	64	417	13	206	57	388	29	1051
chr4	136547751	C	1	2149	3	1769	2	479	0	219	1	444	0	1078
chr4	136547752	C	8	2144	1	1772	1	479	0	219	3	442	7	1073
chr4	136547753	C	3	2146	0	1772	2	479	1	218	1	444	1	1079
chr4	136547754	C	69	2081	2	1769	11	470	8	211	5	439	75	1003
chr4	136547757	C	4	2147	5	1767	9	472	0	219	0	444	0	1079
chr4	136547759	C	31	2119	0	1771	11	470	0	218	0	444	0	1078
chr4	136547764	C	2014	136	1632	137	456	25	204	15	429	15	993	85
chr4	136547772	C	0	2129	1	1752	0	480	0	217	0	439	0	1074
chr4	136547774	C	0	2128	1	1748	0	480	0	217	0	438	0	1072
chr4	136547777	C	2	2122	2	1745	1	479	0	216	0	437	0	1073
chr4	136547788	C	0	1969	0	1607	0	443	0	191	0	406	0	968
chr4	139783570	c	0	4728	1	6630	0	3734	0	3192	0	2705	0	3624
chr4	139783571	c	0	4752	1	6644	1	3738	0	3200	0	2708	0	3629
chr4	139783578	c	0	4768	0	6660	1	3746	0	3202	0	2709	0	3630
chr4	139783585	c	0	4769	0	6642	0	3744	0	3199	0	2708	1	3622
chr4	139783587	c	1	4781	4	6675	0	3756	0	3210	0	2714	1	3634
chr4	139783588	c	4	4780	5	6674	1	3757	0	3211	1	2713	1	3634
chr4	139783589	c	6	4780	9	6666	0	3758	8	3203	3	2712	5	3629
chr4	139783590	c	19	4768	88	6593	9	3751	2	3208	1	2714	1	3633
chr4	139783592	g	1	6231	1	7800	5	4062	0	3355	1	2335	1	1790
chr4	139783596	c	2	4787	8	6672	4	3755	4	3207	2	2708	6	3629
chr4	139783598	c	5	4790	4	6684	4	3758	2	3213	6	2709	2	3637
chr4	139783601	g	4	6278	4	7874	0	4084	1	3378	0	2346	0	1806
chr4	139783602	g	2	6284	4	7877	4	4079	1	3376	1	2347	0	1807
chr4	139783603	g	2	6288	0	7883	1	4086	0	3379	0	2348	0	1808
chr4	139783605	c	3	4797	1	6696	10	3761	5	3213	12	2703	27	3613
chr4	139783607	g	0	6293	1	7879	0	4088	0	3379	0	2350	0	1808
chr4	139783608	c	3	4761	19	6640	9	3734	4	3193	2	2693	3	3612
chr4	139783611	c	7	4788	12	6674	15	3757	27	3188	1	2710	6	3628

26	2233	3	1859	6	494	1	255	14	481	1	1134
8	2234	6	1865	1	494	0	256	2	482	1	1133
48	2235	0	1865	19	493	11	256	0	481	2	1129
2	2234	2	1864	0	494	1	256	2	481	0	1133
3	2220	0	1849	1	492	0	251	1	478	2	1129
19	2193	5	1813	2	485	0	235	1	471	3	1111
16	2193	52	1814	2	484	3	236	8	467	1	1109
11	2186	4	1810	8	483	5	235	0	466	41	1107
2012	2176	1593	1797	452	480	221	233	439	464	1005	1100
33	2177	75	1797	10	483	6	234	43	461	1	1100
38	2160	48	1779	14	481	17	224	8	449	33	1085
31	2155	9	1774	2	481	0	221	1	446	6	1080
3	2156	4	1774	3	479	0	221	4	446	1	1080
35	2153	1	1775	6	481	0	220	20	445	8	1080
75	2152	49	1773	28	481	4	219	11	445	46	1080
13	2149	2	1774	0	481	1	219	2	445	1	1078
89	2153	24	1773	64	481	13	219	57	445	29	1080
1	2150	3	1772	2	481	0	219	1	445	0	1078
8	2152	1	1773	1	480	0	219	3	445	7	1080
3	2149	0	1772	2	481	1	219	1	445	1	1080
69	2150	2	1771	11	481	8	219	5	444	75	1078
4	2151	5	1772	9	481	0	219	0	444	0	1079
31	2150	0	1771	11	481	0	218	0	444	0	1078
2014	2150	1632	1769	456	481	204	219	429	444	993	1078
0	2129	1	1753	0	480	0	217	0	439	0	1074
0	2128	1	1749	0	480	0	217	0	438	0	1072
2	2124	2	1747	1	480	0	216	0	437	0	1073
0	1969	0	1607	0	443	0	191	0	406	0	968
0	4728	1	6631	0	3734	0	3192	0	2705	0	3624
0	4752	1	6645	1	3739	0	3200	0	2708	0	3629
0	4768	0	6660	1	3747	0	3202	0	2709	0	3630
0	4769	0	6642	0	3744	0	3199	0	2708	1	3623
1	4782	4	6679	0	3756	0	3210	0	2714	1	3635
4	4784	5	6679	1	3758	0	3211	1	2714	1	3635
6	4786	9	6675	0	3758	8	3211	3	2715	5	3634
19	4787	88	6681	9	3760	2	3210	1	2715	1	3634
1	6232	1	7801	5	4067	0	3355	1	2336	1	1791
2	4789	8	6680	4	3759	4	3211	2	2710	6	3635
5	4795	4	6688	4	3762	2	3215	6	2715	2	3639
4	6282	4	7878	0	4084	1	3379	0	2346	0	1806
2	6286	4	7881	4	4083	1	3377	1	2348	0	1807
2	6290	0	7883	1	4087	0	3379	0	2348	0	1808
3	4800	1	6697	10	3771	5	3218	12	2715	27	3640
0	6293	1	7880	0	4088	0	3379	0	2350	0	1808
3	4764	19	6659	9	3743	4	3197	2	2695	3	3615
7	4795	12	6686	15	3772	27	3215	1	2711	6	3634

chr4	139783612	c	4	4798	12	6686	6	3769	16	3203	4	2712	3	3637
chr4	139783613	c	4	4798	2	6697	33	3743	4	3214	6	2710	17	3621
chr4	139783616	g	28	6286	10	7919	7	4084	4	3380	4	2347	4	1803
chr4	139783618	G	71	6243	32	7899	35	4055	32	3354	24	2329	27	1780
chr4	139783621	G	2	6317	18	7921	10	4084	4	3385	2	2351	3	1806
chr4	139783622	G	35	6283	27	7910	10	4083	6	3383	9	2344	12	1797
chr4	139783623	C	32	4775	3	6696	13	3765	45	3173	7	2711	40	3606
chr4	139783625	G	16	6309	26	7917	6	4087	12	3378	13	2340	3	1806
chr4	139783629	G	8	6330	73	7874	8	4094	4	3390	11	2356	3	1809
chr4	139783632	G	25	6311	9	7942	6	4108	22	3375	8	2368	6	1822
chr4	139783633	G	5	6339	11	7946	1	4119	5	3397	4	2377	6	1824
chr4	139783634	G	15	6332	16	7947	9	4113	8	3397	7	2372	4	1827
chr4	139783635	G	26	6325	14	7951	6	4119	24	3381	8	2376	7	1825
chr4	139783636	G	10	6344	21	7943	5	4117	4	3401	5	2379	4	1830
chr4	139783638	C	3129	1683	3680	3025	2458	1323	2208	1016	1802	918	2047	1604
chr4	139783639	G	4400	1937	4872	3074	2780	1338	2085	1298	1661	713	1117	700
chr4	139783640	C	15	4797	9	6698	52	3728	6	3217	8	2712	25	3624
chr4	139783642	G	30	6333	15	7966	11	4122	7	3401	5	2380	7	1829
chr4	139783645	C	2	4813	1	6706	2	3780	4	3220	10	2712	30	3629
chr4	139783647	G	11	5964	22	7431	15	3869	9	3177	4	2258	18	1706
chr4	139783649	G	12	6357	36	7952	21	4116	12	3396	13	2386	3	1838
chr4	139783650	G	10	6357	17	7973	16	4123	3	3407	6	2392	3	1836
chr4	139783652	G	28	6342	106	7886	24	4118	24	3386	18	2374	8	1834
chr4	139783653	G	24	6347	15	7979	8	4138	5	3405	7	2390	5	1839
chr4	139783654	G	26	6345	19	7977	12	4130	4	3405	9	2387	3	1840
chr4	139783655	G	5	6367	18	7978	21	4125	8	3403	6	2392	8	1836
chr4	139783657	C	1	4826	7	6705	6	3777	4	3221	0	2726	5	3653
chr4	139783660	C	22	4804	4	6709	7	3779	1	3223	7	2718	2	3656
chr4	139783661	C	45	4782	2	6711	2	3784	0	3225	2	2724	12	3646
chr4	139783665	C	16	4813	13	6706	4	3779	11	3213	6	2719	7	3651
chr4	139783666	C	9	4820	8	6711	16	3769	6	3219	2	2726	3	3656
chr4	139783667	C	8	4821	9	6710	7	3777	1	3223	8	2720	4	3655
chr4	139783668	C	9	4817	17	6703	1	3783	4	3221	0	2727	1	3658
chr4	139783672	G	9	6407	32	8016	4	4176	9	3423	4	2416	4	1856
chr4	139783673	C	16	4812	15	6707	27	3756	7	3218	6	2721	15	3644
chr4	139783675	G	11	6405	5	8047	8	4174	4	3432	8	2414	13	1850
chr4	139783676	C	19	4805	14	6697	7	3775	5	3211	10	2714	16	3637
chr4	139783677	C	3029	1802	3949	2776	2461	1327	1900	1324	1740	988	2016	1643
chr4	139783678	G	4048	2347	4055	3970	2579	1597	1869	1561	1508	913	1060	798
chr4	139783680	C	6	4834	9	6718	7	3784	4	3225	1	2732	12	3652
chr4	139783682	G	20	6400	6	8047	7	4183	2	3438	12	2415	11	1852
chr4	139783684	C	21	4815	59	6662	4	3786	4	3223	2	2731	4	3673
chr4	139783685	C	35	4804	22	6703	17	3777	6	3221	7	2729	7	3672
chr4	139783687	C	4	4838	3	6727	4	3789	4	3224	4	2734	3	3676
chr4	139783690	C	104	4737	82	6649	43	3751	82	3148	43	2694	75	3605
chr4	139783693	G	7	6416	25	8030	12	4186	11	3430	5	2423	0	1863

4	4802	12	6698	6	3775	16	3219	4	2716	3	3640
4	4802	2	6699	33	3776	4	3218	6	2716	17	3638
28	6314	10	7929	7	4091	4	3384	4	2351	4	1807
71	6314	32	7931	35	4090	32	3386	24	2353	27	1807
2	6319	18	7939	10	4094	4	3389	2	2353	3	1809
35	6318	27	7937	10	4093	6	3389	9	2353	12	1809
32	4807	3	6699	13	3778	45	3218	7	2718	40	3646
16	6325	26	7943	6	4093	12	3390	13	2353	3	1809
8	6338	73	7947	8	4102	4	3394	11	2367	3	1812
25	6336	9	7951	6	4114	22	3397	8	2376	6	1828
5	6344	11	7957	1	4120	5	3402	4	2381	6	1830
15	6347	16	7963	9	4122	8	3405	7	2379	4	1831
26	6351	14	7965	6	4125	24	3405	8	2384	7	1832
10	6354	21	7964	5	4122	4	3405	5	2384	4	1834
3129	4812	3680	6705	2458	3781	2208	3224	1802	2720	2047	3651
4400	6337	4872	7946	2780	4118	2085	3383	1661	2374	1117	1817
15	4812	9	6707	52	3780	6	3223	8	2720	25	3649
30	6363	15	7981	11	4133	7	3408	5	2385	7	1836
2	4815	1	6707	2	3782	4	3224	10	2722	30	3659
11	5975	22	7453	15	3884	9	3186	4	2262	18	1724
12	6369	36	7988	21	4137	12	3408	13	2399	3	1841
10	6367	17	7990	16	4139	3	3410	6	2398	3	1839
28	6370	106	7992	24	4142	24	3410	18	2392	8	1842
24	6371	15	7994	8	4146	5	3410	7	2397	5	1844
26	6371	19	7996	12	4142	4	3409	9	2396	3	1843
5	6372	18	7996	21	4146	8	3411	6	2398	8	1844
1	4827	7	6712	6	3783	4	3225	0	2726	5	3658
22	4826	4	6713	7	3786	1	3224	7	2725	2	3658
45	4827	2	6713	2	3786	0	3225	2	2726	12	3658
16	4829	13	6719	4	3783	11	3224	6	2725	7	3658
9	4829	8	6719	16	3785	6	3225	2	2728	3	3659
8	4829	9	6719	7	3784	1	3224	8	2728	4	3659
9	4826	17	6720	1	3784	4	3225	0	2727	1	3659
9	6416	32	8048	4	4180	9	3432	4	2420	4	1860
16	4828	15	6722	27	3783	7	3225	6	2727	15	3659
11	6416	5	8052	8	4182	4	3436	8	2422	13	1863
19	4824	14	6711	7	3782	5	3216	10	2724	16	3653
3029	4831	3949	6725	2461	3788	1900	3224	1740	2728	2016	3659
4048	6395	4055	8025	2579	4176	1869	3430	1508	2421	1060	1858
6	4840	9	6727	7	3791	4	3229	1	2733	12	3664
20	6420	6	8053	7	4190	2	3440	12	2427	11	1863
21	4836	59	6721	4	3790	4	3227	2	2733	4	3677
35	4839	22	6725	17	3794	6	3227	7	2736	7	3679
4	4842	3	6730	4	3793	4	3228	4	2738	3	3679
104	4841	82	6731	43	3794	82	3230	43	2737	75	3680
7	6423	25	8055	12	4198	11	3441	5	2428	0	1863

chr4	139783694	C	114	4731	175	6560	52	3746	258	2973	57	2682	93	3587
chr4	139783696	C	142	4705	29	6708	138	3662	116	3116	62	2677	30	3650
chr4	139783698	C	16	4835	50	6687	20	3780	4	3229	8	2732	5	3675
chr4	139783699	C	3845	1006	4636	2102	2982	818	2343	891	2189	548	2614	1055
chr4	139783700	G	4538	1800	5062	2860	2938	1201	2052	1349	1797	603	1260	576
chr4	139783701	C	10	4842	8	6731	2	3802	9	3228	7	2732	9	3671
chr4	139783702	C	1	4853	6	6736	5	3800	1	3237	2	2738	2	3679
chr4	139783705	G	17	6409	12	8045	3	4199	16	3425	6	2426	2	1865
chr4	139783706	C	6	4846	14	6730	12	3794	39	3199	7	2734	19	3665
chr4	139783710	C	16	4837	10	6737	3	3803	6	3233	5	2739	2	3682
chr4	139783711	C	77	4779	215	6534	25	3783	3	3235	23	2722	60	3630
chr4	139783713	G	8	6423	12	8045	2	4202	6	3441	8	2431	10	1858
chr4	139783719	G	2	6440	4	8056	20	4190	16	3429	4	2435	2	1868
chr4	139783720	C	10	173	0	201	3	163	1	103	1	94	4	100
chr4	139783722	C	0	177	1	195	0	160	0	100	0	94	0	103
chr4	139783724	C	0	165	0	186	1	150	0	94	1	88	0	102
chr4	139783727	G	36	6409	15	8042	14	4208	25	3423	13	2434	8	1859
chr4	139783728	C	2	158	3	163	1	133	1	82	0	84	0	98
chr4	139783730	C	5	152	1	157	1	126	4	82	4	74	1	101
chr4	139783732	C	2	155	3	150	1	121	2	81	0	85	1	110
chr4	139783736	C	94	63	85	77	88	41	48	42	65	27	63	60
chr4	139783737	G	3526	2900	3431	4598	2168	2045	1447	1999	1294	1154	899	966
chr4	139783740	C	1	157	2	169	1	137	0	95	0	91	0	125
chr4	139783741	C	0	191	0	183	1	173	0	110	0	115	0	155
chr4	139783742	C	1	197	8	180	4	178	0	117	0	125	1	165
chr4	139783744	C	0	204	0	193	1	199	0	119	0	134	0	174
chr4	139783745	C	114	103	95	99	97	110	53	74	97	113	121	138
chr4	139783746	G	144	178	133	229	137	159	57	108	90	131	48	100
chr4	139783748	C	0	226	0	202	0	208	0	135	1	215	1	259
chr4	139783749	C	1	225	1	205	1	209	1	135	0	220	0	262
chr4	139783753	G	2	565	0	630	1	374	0	258	0	285	0	190
chr4	139783754	C	2949	1953	3936	2815	2456	1400	1545	1722	1644	1130	2081	1652
chr4	139783755	G	3402	3044	3205	4856	2125	2108	1471	1989	1295	1176	854	1028
chr4	139783756	G	18	6440	11	8071	9	4227	6	3459	5	2470	6	1875
chr4	139783759	G	153	6306	56	8022	81	4152	34	3428	39	2434	46	1831
chr4	139783760	G	7	6452	10	8072	6	4223	2	3460	3	2469	3	1877
chr4	139783763	G	12	6447	14	8065	9	4218	8	3453	10	2465	3	1875
chr4	139783764	C	28	4881	2	6761	2	3855	23	3246	10	2774	2	3747
chr4	139783766	C	20	4886	122	6640	3	3855	3	3262	4	2778	5	3744
chr4	139783768	G	50	6399	6	8066	44	4181	61	3396	12	2448	2	1870
chr4	139783771	G	14	6445	26	8054	7	4221	8	3451	7	2461	7	1869
chr4	139783772	G	17	6441	24	8054	10	4219	8	3450	5	2466	7	1870
chr4	139783773	C	5	4892	16	6742	8	3840	4	3257	8	2772	13	3735
chr4	139783774	C	34	4863	56	6696	4	3843	5	3258	8	2770	42	3706
chr4	139783776	C	208	4685	134	6613	126	3717	79	3179	61	2715	126	3619
chr4	139783778	C	8	4881	9	6736	6	3832	2	3255	6	2769	8	3738

114	4845	175	6735	52	3798	258	3231	57	2739	93	3680
142	4847	29	6737	138	3800	116	3232	62	2739	30	3680
16	4851	50	6737	20	3800	4	3233	8	2740	5	3680
3845	4851	4636	6738	2982	3800	2343	3234	2189	2737	2614	3669
4538	6338	5062	7922	2938	4139	2052	3401	1797	2400	1260	1836
10	4852	8	6739	2	3804	9	3237	7	2739	9	3680
1	4854	6	6742	5	3805	1	3238	2	2740	2	3681
17	6426	12	8057	3	4202	16	3441	6	2432	2	1867
6	4852	14	6744	12	3806	39	3238	7	2741	19	3684
16	4853	10	6747	3	3806	6	3239	5	2744	2	3684
77	4856	215	6749	25	3808	3	3238	23	2745	60	3690
8	6431	12	8057	2	4204	6	3447	8	2439	10	1868
2	6442	4	8060	20	4210	16	3445	4	2439	2	1870
10	183	0	201	3	166	1	104	1	95	4	104
0	177	1	196	0	160	0	100	0	94	0	103
0	165	0	186	1	151	0	94	1	89	0	102
36	6445	15	8057	14	4222	25	3448	13	2447	8	1867
2	160	3	166	1	134	1	83	0	84	0	98
5	157	1	158	1	127	4	86	4	78	1	102
2	157	3	153	1	122	2	83	0	85	1	111
94	157	85	162	88	129	48	90	65	92	63	123
3526	6426	3431	8029	2168	4213	1447	3446	1294	2448	899	1865
1	158	2	171	1	138	0	95	0	91	0	125
0	191	0	183	1	174	0	110	0	115	0	155
1	198	8	188	4	182	0	117	0	125	1	166
0	204	0	193	1	200	0	119	0	134	0	174
114	217	95	194	97	207	53	127	97	210	121	259
144	322	133	362	137	296	57	165	90	221	48	148
0	226	0	202	0	208	0	135	1	216	1	260
1	226	1	206	1	210	1	136	0	220	0	262
2	567	0	630	1	375	0	258	0	285	0	190
2949	4902	3936	6751	2456	3856	1545	3267	1644	2774	2081	3733
3402	6446	3205	8061	2125	4233	1471	3460	1295	2471	854	1882
18	6458	11	8082	9	4236	6	3465	5	2475	6	1881
153	6459	56	8078	81	4233	34	3462	39	2473	46	1877
7	6459	10	8082	6	4229	2	3462	3	2472	3	1880
12	6459	14	8079	9	4227	8	3461	10	2475	3	1878
28	4909	2	6763	2	3857	23	3269	10	2784	2	3749
20	4906	122	6762	3	3858	3	3265	4	2782	5	3749
50	6449	6	8072	44	4225	61	3457	12	2460	2	1872
14	6459	26	8080	7	4228	8	3459	7	2468	7	1876
17	6458	24	8078	10	4229	8	3458	5	2471	7	1877
5	4897	16	6758	8	3848	4	3261	8	2780	13	3748
34	4897	56	6752	4	3847	5	3263	8	2778	42	3748
208	4893	134	6747	126	3843	79	3258	61	2776	126	3745
8	4889	9	6745	6	3838	2	3257	6	2775	8	3746

chr4	139783779	C	18	4871	24	6722	5	3835	7	3248	13	2761	11	3732
chr4	139783781	C	40	4847	14	6726	26	3810	89	3164	7	2765	43	3700
chr4	139783783	C	6	4884	7	6739	7	3832	4	3251	6	2768	7	3738
chr4	139783785	G	19	6432	6	8069	10	4192	10	3445	7	2448	5	1869
chr4	139783787	G	50	6401	33	8041	37	4164	92	3363	22	2433	8	1865
chr4	139783790	G	10	6435	9	8058	7	4188	24	3425	2	2451	5	1864
chr4	139783791	C	47	4836	6	6729	50	3782	65	3185	23	2748	5	3734
chr4	139783793	C	10	4874	61	6675	13	3816	9	3245	3	2768	8	3736
chr4	139783794	C	6	4877	6	6731	6	3823	2	3251	10	2760	4	3741
chr4	139783796	G	12	6433	26	8043	10	4167	16	3433	10	2436	9	1853
chr4	139783800	C	6	4876	20	6713	27	3800	5	3242	8	2760	5	3737
chr4	139783801	C	14	4867	10	6727	6	3820	2	3249	7	2763	2	3742
chr4	139783802	C	66	4817	13	6723	43	3785	103	3147	20	2750	69	3674
chr4	139783804	C	13	4868	32	6700	16	3812	6	3242	7	2760	9	3734
chr4	139783805	C	8	4875	22	6711	7	3821	5	3244	3	2766	7	3737
chr4	139783808	G	13	6425	6	8060	5	4169	2	3446	4	2435	1	1857
chr4	139783810	C	10	4870	25	6707	8	3814	12	3236	4	2762	32	3708
chr4	139783812	G	80	6356	40	8022	48	4123	38	3408	23	2415	25	1829
chr4	139783814	G	19	6418	27	8034	11	4162	3	3441	5	2432	9	1845
chr4	139783817	C	12	4863	29	6699	13	3807	11	3234	8	2757	4	3734
chr4	139783818	C	5	4871	13	6715	28	3794	5	3240	5	2760	8	3730
chr4	139783819	C	10	4863	12	6716	6	3815	5	3239	4	2760	4	3732
chr4	139783822	C	6	4863	16	6709	6	3814	5	3238	7	2750	8	3726
chr4	139783823	C	8	4862	11	6715	5	3817	8	3234	3	2758	16	3720
chr4	139783824	C	5	4868	18	6705	5	3816	6	3237	9	2752	7	3729
chr4	139783826	G	15	6413	11	8047	68	4091	30	3411	18	2413	8	1844
chr4	139783829	G	12	6416	18	8038	7	4153	10	3430	1	2430	1	1850
chr4	139783830	C	33	4838	4	6715	9	3805	11	3231	32	2725	68	3666
chr4	139783832	C	9	4860	18	6693	7	3805	35	3204	6	2751	9	3724
chr4	139783833	C	4	4865	4	6710	3	3807	4	3236	3	2755	9	3724
chr4	139783836	C	18	4848	13	6699	7	3804	10	3229	4	2751	9	3723
chr4	139783840	G	7	6410	16	8041	6	4147	3	3436	5	2419	2	1850
chr4	139783841	C	15	4849	10	6701	6	3802	6	3232	6	2751	9	3722
chr4	139783843	C	79	4785	134	6579	72	3737	5	3235	23	2734	40	3689
chr4	139783845	G	6	6411	12	8043	8	4148	5	3436	2	2421	2	1846
chr4	139783847	C	14	4850	46	6667	26	3784	4	3236	23	2734	31	3698
chr4	139783849	G	13	6392	16	8022	9	4140	6	3431	10	2410	7	1837
chr4	139783850	C	11	4850	10	6703	5	3804	4	3235	5	2749	7	3721
chr4	139783851	C	11	4850	14	6696	6	3804	6	3232	4	2753	4	3725
chr4	139783852	C	1744	3118	1990	4720	1448	2358	846	2393	976	1780	1156	2572
chr4	139783853	G	1873	4525	1744	6296	1130	3017	628	2809	776	1641	462	1380
chr4	139783854	G	13	6379	9	8029	5	4139	8	3427	5	2411	6	1835
chr4	139783855	C	10	4851	12	6699	7	3800	2	3238	11	2746	5	3723
chr4	139783856	C	1210	3601	1730	4908	1146	2615	677	2527	787	1933	1065	2633
chr4	139783857	G	1653	4733	1485	6545	1104	3038	554	2878	759	1655	422	1415
chr4	139783860	C	4	4857	7	6702	4	3802	2	3236	4	2753	5	3723

18	4889	24	6746	5	3840	7	3255	13	2774	11	3743
40	4887	14	6740	26	3836	89	3253	7	2772	43	3743
6	4890	7	6746	7	3839	4	3255	6	2774	7	3745
19	6451	6	8075	10	4202	10	3455	7	2455	5	1874
50	6451	33	8074	37	4201	92	3455	22	2455	8	1873
10	6445	9	8067	7	4195	24	3449	2	2453	5	1869
47	4883	6	6735	50	3832	65	3250	23	2771	5	3739
10	4884	61	6736	13	3829	9	3254	3	2771	8	3744
6	4883	6	6737	6	3829	2	3253	10	2770	4	3745
12	6445	26	8069	10	4177	16	3449	10	2446	9	1862
6	4882	20	6733	27	3827	5	3247	8	2768	5	3742
14	4881	10	6737	6	3826	2	3251	7	2770	2	3744
66	4883	13	6736	43	3828	103	3250	20	2770	69	3743
13	4881	32	6732	16	3828	6	3248	7	2767	9	3743
8	4883	22	6733	7	3828	5	3249	3	2769	7	3744
13	6438	6	8066	5	4174	2	3448	4	2439	1	1858
10	4880	25	6732	8	3822	12	3248	4	2766	32	3740
80	6436	40	8062	48	4171	38	3446	23	2438	25	1854
19	6437	27	8061	11	4173	3	3444	5	2437	9	1854
12	4875	29	6728	13	3820	11	3245	8	2765	4	3738
5	4876	13	6728	28	3822	5	3245	5	2765	8	3738
10	4873	12	6728	6	3821	5	3244	4	2764	4	3736
6	4869	16	6725	6	3820	5	3243	7	2757	8	3734
8	4870	11	6726	5	3822	8	3242	3	2761	16	3736
5	4873	18	6723	5	3821	6	3243	9	2761	7	3736
15	6428	11	8058	68	4159	30	3441	18	2431	8	1852
12	6428	18	8056	7	4160	10	3440	1	2431	1	1851
33	4871	4	6719	9	3814	11	3242	32	2757	68	3734
9	4869	18	6711	7	3812	35	3239	6	2757	9	3733
4	4869	4	6714	3	3810	4	3240	3	2758	9	3733
18	4866	13	6712	7	3811	10	3239	4	2755	9	3732
7	6417	16	8057	6	4153	3	3439	5	2424	2	1852
15	4864	10	6711	6	3808	6	3238	6	2757	9	3731
79	4864	134	6713	72	3809	5	3240	23	2757	40	3729
6	6417	12	8055	8	4156	5	3441	2	2423	2	1848
14	4864	46	6713	26	3810	4	3240	23	2757	31	3729
13	6405	16	8038	9	4149	6	3437	10	2420	7	1844
11	4861	10	6713	5	3809	4	3239	5	2754	7	3728
11	4861	14	6710	6	3810	6	3238	4	2757	4	3729
1744	4862	1990	6710	1448	3806	846	3239	976	2756	1156	3728
1873	6398	1744	8040	1130	4147	628	3437	776	2417	462	1842
13	6392	9	8038	5	4144	8	3435	5	2416	6	1841
10	4861	12	6711	7	3807	2	3240	11	2757	5	3728
1210	4811	1730	6638	1146	3761	677	3204	787	2720	1065	3698
1653	6386	1485	8030	1104	4142	554	3432	759	2414	422	1837
4	4861	7	6709	4	3806	2	3238	4	2757	5	3728

chr4	139783862	C	1362	3497	1951	4755	1276	2532	801	2437	859	1897	1001	2728
chr4	139783863	G	2244	4126	1606	6392	1319	2805	635	2791	877	1530	511	1319
chr4	139783864	G	9	6378	22	8007	9	4127	5	3427	4	2408	5	1832
chr4	139783866	G	27	6362	17	8011	12	4126	12	3418	4	2409	3	1834
chr4	139783869	G	33	6342	17	7995	12	4116	10	3417	3	2407	4	1828
chr4	139783870	C	4	4852	8	6700	19	3790	3	3236	11	2745	9	3719
chr4	139783872	G	16	6365	5	8020	3	4126	2	3425	4	2408	9	1824
chr4	139783873	G	21	6361	16	8005	9	4122	8	3416	4	2407	3	1830
chr4	139783875	G	182	6197	289	7728	95	4036	38	3381	61	2343	37	1794
chr4	139783876	C	11	4845	57	6651	28	3780	16	3222	20	2733	8	3719
chr4	139783878	G	0	6374	1	8008	0	4125	0	3419	0	2405	1	1828
chr4	139783884	C	0	4844	0	6704	0	3803	1	3234	0	2748	0	3712
chr4	139783885	C	0	4844	1	6702	0	3804	0	3233	0	2748	0	3715
chr4	139783888	G	1	6367	1	7991	0	4116	0	3416	0	2400	0	1824
chr4	139783891	C	2	4840	4	6696	1	3800	0	3233	0	2744	2	3709
chr4	139783894	G	0	6350	1	7978	0	4107	1	3405	0	2391	0	1819
chr4	139783896	C	0	4822	1	6682	0	3793	1	3224	0	2656	1	3617
chr4	139783897	C	0	4819	1	6678	0	3793	1	3222	0	2657	0	3618
chr4	139783898	C	1	4815	0	6678	0	3789	0	3220	0	2658	1	3616
chr4	139783900	G	0	6177	1	7794	0	4062	0	3340	0	2350	0	1793
chr4	139783901	G	0	6149	1	7765	0	4046	1	3327	0	2345	0	1785
chr4	139783902	G	0	6136	3	7737	0	4037	0	3324	0	2330	0	1780
chr4	139783904	C	1	4722	0	6536	0	3722	0	3169	0	2622	0	3582
chr5	64987289	G	0	1668	0	1964	2	678	0	326	0	864	0	981
chr5	64987290	G	3	1669	0	1969	0	682	0	326	0	864	0	981
chr5	64987294	G	0	1677	0	1979	0	683	0	326	1	865	0	984
chr5	64987296	G	0	1677	0	1980	0	683	0	326	0	866	0	984
chr5	64987302	G	1	1676	1	1981	0	683	0	326	0	866	1	983
chr5	64987307	G	0	1679	1	1982	0	683	0	327	0	867	0	983
chr5	64987310	G	19	1662	4	1981	8	676	10	318	11	857	7	977
chr5	64987316	G	14	1665	23	1963	9	675	1	328	5	863	3	982
chr5	64987317	G	2	1678	5	1983	1	683	0	329	1	868	3	982
chr5	64987319	G	3	1678	5	1985	3	681	1	328	4	865	0	986
chr5	64987320	C	0	1481	0	1990	0	272	0	142	1	460	1	2147
chr5	64987323	G	1	1681	1	1989	5	680	1	328	1	868	1	986
chr5	64987325	C	0	2228	0	3001	0	397	0	220	0	667	0	3084
chr5	64987327	G	5	1677	3	1987	2	682	0	329	2	867	2	986
chr5	64987329	C	0	2231	1	3005	0	402	0	222	0	668	0	3094
chr5	64987330	C	0	2237	0	3017	0	404	0	224	0	669	0	3106
chr5	64987333	G	25	1658	29	1962	7	678	2	327	13	856	6	982
chr5	64987338	G	4	1681	5	1986	2	683	0	329	2	867	5	984
chr5	64987339	G	1	1685	10	1982	2	683	0	329	4	865	5	984
chr5	64987340	G	6	1680	3	1988	2	683	1	328	4	865	3	986
chr5	64987342	G	61	1626	78	1916	28	658	34	295	29	841	32	956
chr5	64987343	C	0	2249	2	3037	0	408	0	229	0	679	2	3142
chr5	64987345	G	39	1648	5	1990	16	670	1	328	17	853	24	965

1362	4859	1951	6706	1276	3808	801	3238	859	2756	1001	3729
2244	6370	1606	7998	1319	4124	635	3426	877	2407	511	1830
9	6387	22	8029	9	4136	5	3432	4	2412	5	1837
27	6389	17	8028	12	4138	12	3430	4	2413	3	1837
33	6375	17	8012	12	4128	10	3427	3	2410	4	1832
4	4856	8	6708	19	3809	3	3239	11	2756	9	3728
16	6381	5	8025	3	4129	2	3427	4	2412	9	1833
21	6382	16	8021	9	4131	8	3424	4	2411	3	1833
182	6379	289	8017	95	4131	38	3419	61	2404	37	1831
11	4856	57	6708	28	3808	16	3238	20	2753	8	3727
0	6374	1	8009	0	4125	0	3419	0	2405	1	1829
0	4844	0	6704	0	3803	1	3235	0	2748	0	3712
0	4844	1	6703	0	3804	0	3233	0	2748	0	3715
1	6368	1	7992	0	4116	0	3416	0	2400	0	1824
2	4842	4	6700	1	3801	0	3233	0	2744	2	3711
0	6350	1	7979	0	4107	1	3406	0	2391	0	1819
0	4822	1	6683	0	3793	1	3225	0	2656	1	3618
0	4819	1	6679	0	3793	1	3223	0	2657	0	3618
1	4816	0	6678	0	3789	0	3220	0	2658	1	3617
0	6177	1	7795	0	4062	0	3340	0	2350	0	1793
0	6149	1	7766	0	4046	1	3328	0	2345	0	1785
0	6136	3	7740	0	4037	0	3324	0	2330	0	1780
1	4723	0	6536	0	3722	0	3169	0	2622	0	3582
0	1668	0	1964	2	680	0	326	0	864	0	981
3	1672	0	1969	0	682	0	326	0	864	0	981
0	1677	0	1979	0	683	0	326	1	866	0	984
0	1677	0	1980	0	683	0	326	0	866	0	984
1	1677	1	1982	0	683	0	326	0	866	1	984
0	1679	1	1983	0	683	0	327	0	867	0	983
19	1681	4	1985	8	684	10	328	11	868	7	984
14	1679	23	1986	9	684	1	329	5	868	3	985
2	1680	5	1988	1	684	0	329	1	869	3	985
3	1681	5	1990	3	684	1	329	4	869	0	986
0	1481	0	1990	0	272	0	142	1	461	1	2148
1	1682	1	1990	5	685	1	329	1	869	1	987
0	2228	0	3001	0	397	0	220	0	667	0	3084
5	1682	3	1990	2	684	0	329	2	869	2	988
0	2231	1	3006	0	402	0	222	0	668	0	3094
0	2237	0	3017	0	404	0	224	0	669	0	3106
25	1683	29	1991	7	685	2	329	13	869	6	988
4	1685	5	1991	2	685	0	329	2	869	5	989
1	1686	10	1992	2	685	0	329	4	869	5	989
6	1686	3	1991	2	685	1	329	4	869	3	989
61	1687	78	1994	28	686	34	329	29	870	32	988
0	2249	2	3039	0	408	0	229	0	679	2	3144
39	1687	5	1995	16	686	1	329	17	870	24	989

chr5	64987346	G	0	1687	0	1993	0	686	1	328	0	870	2	987
chr5	64987349	C	3	2261	6	3051	1	410	0	230	1	681	6	3147
chr5	64987350	C	4	2260	4	3053	0	411	0	230	0	682	5	3149
chr5	64987354	G	1	1687	3	1997	0	690	2	327	2	870	1	990
chr5	64987357	G	2	1687	5	1997	2	689	1	328	3	871	1	989
chr5	64987358	C	1	2266	4	3051	2	410	0	230	1	681	3	3152
chr5	64987359	C	3	2264	0	3057	0	412	0	230	2	680	2	3154
chr5	64987363	C	3	2264	7	3050	7	405	6	224	0	681	8	3146
chr5	64987367	C	631	1636	854	2204	104	308	58	172	205	477	639	2519
chr5	64987368	G	481	1209	494	1508	242	449	113	217	308	569	265	727
chr5	64987376	C	4	2262	5	3054	0	412	0	230	1	682	9	3149
chr5	64987377	C	529	1738	898	2162	115	297	60	170	221	462	664	2494
chr5	64987378	G	480	1209	501	1499	216	475	98	232	299	579	247	744
chr5	64987380	C	9	2255	2	3050	1	411	0	230	2	679	9	3143
chr5	64987383	C	3	2265	1	3061	0	412	0	231	3	681	6	3154
chr5	64987384	C	9	2258	22	3040	3	409	3	228	0	684	16	3144
chr5	64987386	G	14	1678	12	1991	4	689	1	328	8	870	12	980
chr5	64987389	C	3	2266	2	3063	0	412	0	231	1	683	7	3156
chr5	64987391	G	9	1676	3	1999	9	683	0	329	7	872	2	989
chr5	64987393	G	6	1687	3	2000	0	693	1	329	1	879	7	986
chr5	64987402	G	2	1693	3	2001	5	690	0	332	0	883	0	994
chr5	64987407	C	0	2274	14	3051	0	412	1	230	2	686	2	3162
chr5	64987412	G	8	1693	17	1994	3	699	0	334	8	878	1	999
chr5	64987414	G	6	1696	5	2007	0	702	11	323	3	883	1	999
chr5	64987417	C	2	2274	2	3067	1	412	1	230	1	688	0	3166
chr5	64987419	C	1225	1050	1480	1589	215	199	138	92	411	278	1649	1517
chr5	64987420	G	980	734	1018	1005	457	254	207	132	588	306	607	406
chr5	64987421	G	3	1715	2	2022	2	708	2	339	1	894	4	1009
chr5	64987422	C	1268	1016	1570	1504	223	192	125	105	420	269	1602	1568
chr5	64987423	G	1369	342	1484	528	603	105	260	80	737	152	721	286
chr5	64987424	G	6	1712	6	2018	5	707	0	340	7	887	3	1010
chr5	64987425	G	3	1717	29	1995	4	708	1	339	1	896	3	1011
chr5	64987426	G	2	1718	3	2021	1	713	3	338	4	893	3	1013
chr5	64987427	G	0	1720	5	2019	0	714	1	340	2	895	1	1015
chr5	64987431	G	62	1658	64	1961	45	667	21	320	43	854	48	968
chr5	64987434	G	3	1718	3	2022	1	714	1	343	2	898	3	1015
chr5	64987435	G	1	1718	5	2018	3	712	0	344	13	887	1	1017
chr5	64987438	G	0	94	1	107	0	80	2	33	0	95	1	77
chr5	64987440	G	2	96	3	106	2	83	1	41	2	98	3	82
chr5	64987442	C	1412	817	1636	1372	249	159	111	114	516	156	1687	1425
chr5	64987443	G	83	17	69	37	81	17	31	12	80	22	60	26
chr5	64987445	C	1191	1083	1378	1662	228	188	133	98	432	256	1410	1735
chr5	64987446	G	62	39	73	38	57	48	25	22	65	41	49	42
chr5	64987448	C	1391	859	1638	1382	251	160	133	92	473	199	1717	1384
chr5	64987449	G	72	34	71	47	70	41	29	19	73	37	68	26
chr5	64987451	C	1504	768	1588	1457	255	159	134	99	478	207	1818	1324

0	1687	0	1993	0	686	1	329	0	870	2	989
3	2264	6	3057	1	411	0	230	1	682	6	3153
4	2264	4	3057	0	411	0	230	0	682	5	3154
1	1688	3	2000	0	690	2	329	2	872	1	991
2	1689	5	2002	2	691	1	329	3	874	1	990
1	2267	4	3055	2	412	0	230	1	682	3	3155
3	2267	0	3057	0	412	0	230	2	682	2	3156
3	2267	7	3057	7	412	6	230	0	681	8	3154
631	2267	854	3058	104	412	58	230	205	682	639	3158
481	1690	494	2002	242	691	113	330	308	877	265	992
4	2266	5	3059	0	412	0	230	1	683	9	3158
529	2267	898	3060	115	412	60	230	221	683	664	3158
480	1689	501	2000	216	691	98	330	299	878	247	991
9	2264	2	3052	1	412	0	230	2	681	9	3152
3	2268	1	3062	0	412	0	231	3	684	6	3160
9	2267	22	3062	3	412	3	231	0	684	16	3160
14	1692	12	2003	4	693	1	329	8	878	12	992
3	2269	2	3065	0	412	0	231	1	684	7	3163
9	1685	3	2002	9	692	0	329	7	879	2	991
6	1693	3	2003	0	693	1	330	1	880	7	993
2	1695	3	2004	5	695	0	332	0	883	0	994
0	2274	14	3065	0	412	1	231	2	688	2	3164
8	1701	17	2011	3	702	0	334	8	886	1	1000
6	1702	5	2012	0	702	11	334	3	886	1	1000
2	2276	2	3069	1	413	1	231	1	689	0	3166
1225	2275	1480	3069	215	414	138	230	411	689	1649	3166
980	1714	1018	2023	457	711	207	339	588	894	607	1013
3	1718	2	2024	2	710	2	341	1	895	4	1013
1268	2284	1570	3074	223	415	125	230	420	689	1602	3170
1369	1711	1484	2012	603	708	260	340	737	889	721	1007
6	1718	6	2024	5	712	0	340	7	894	3	1013
3	1720	29	2024	4	712	1	340	1	897	3	1014
2	1720	3	2024	1	714	3	341	4	897	3	1016
0	1720	5	2024	0	714	1	341	2	897	1	1016
62	1720	64	2025	45	712	21	341	43	897	48	1016
3	1721	3	2025	1	715	1	344	2	900	3	1018
1	1719	5	2023	3	715	0	344	13	900	1	1018
0	94	1	108	0	80	2	35	0	95	1	78
2	98	3	109	2	85	1	42	2	100	3	85
1412	2229	1636	3008	249	408	111	225	516	672	1687	3112
83	100	69	106	81	98	31	43	80	102	60	86
1191	2274	1378	3040	228	416	133	231	432	688	1410	3145
62	101	73	111	57	105	25	47	65	106	49	91
1391	2250	1638	3020	251	411	133	225	473	672	1717	3101
72	106	71	118	70	111	29	48	73	110	68	94
1504	2272	1588	3045	255	414	134	233	478	685	1818	3142

chr5	64987452	G	81	36	92	36	76	39	36	16	84	29	76	24
chr5	64987454	C	1796	459	1984	1050	314	94	175	56	577	100	2015	1120
chr5	64987455	G	90	30	97	26	88	27	46	8	88	28	81	24
chr5	64987458	C	13	2138	0	2919	3	393	2	217	3	640	30	3020
chr5	64987463	G	0	122	1	132	1	127	0	56	0	119	1	109
chr5	64987464	C	5	2271	5	3048	4	411	0	232	1	685	4	3153
chr5	64987465	C	1835	458	2080	1003	328	93	186	49	595	97	2063	1117
chr5	64987466	G	92	33	84	51	90	38	43	15	85	36	72	40
chr5	64987467	G	1	121	0	130	0	119	0	54	1	103	0	106
chr5	64987468	C	6	2008	6	2775	0	372	0	216	2	596	7	2862
chr5	64987469	C	1911	376	2469	602	352	70	196	39	605	85	2607	567
chr5	64987470	G	117	13	113	27	109	20	48	7	79	36	95	16
chr5	64987475	C	2	204	1	267	3	74	2	53	1	133	2	241
chr5	64987478	G	0	134	0	141	2	134	0	56	0	126	1	110
chr5	64987481	G	11	113	6	122	8	110	4	47	7	97	1	103
chr5	64987483	G	0	125	5	124	5	112	0	53	9	94	2	101
chr5	64987484	C	14	306	16	441	0	93	2	61	6	148	14	270
chr5	64987486	C	6	319	3	451	0	95	1	64	0	153	1	286
chr5	64987488	C	1	325	2	450	0	95	1	64	0	151	0	288
chr5	64987490	G	0	161	0	171	1	139	1	58	0	108	0	121
chr5	64987491	C	2	317	1	444	2	94	0	61	0	153	0	268
chr5	64987492	C	1	317	0	445	0	95	0	60	1	153	3	266
chr5	64987494	G	2	1761	12	2050	3	761	0	365	5	901	1	1043
chr5	64987496	G	4	1764	4	2068	0	766	1	363	3	903	4	1046
chr5	64987501	C	2	353	1	507	0	116	0	63	0	179	1	378
chr5	64987505	C	2	402	3	584	1	126	1	66	1	193	6	443
chr5	64987507	G	7	1760	4	2065	1	766	0	364	0	904	4	1042
chr5	64987508	G	3	1764	1	2070	2	763	1	363	5	899	5	1041
chr5	64987511	C	1	483	0	714	0	144	0	75	0	221	0	603
chr5	64987512	C	6	858	7	1220	0	193	0	102	1	320	0	1165
chr5	64987514	C	5	2267	1	3063	1	413	0	219	4	662	2	3172
chr5	64987516	C	6	2269	3	3067	0	414	0	218	3	655	3	3175
chr5	64987524	C	3	2271	21	3048	3	409	0	218	2	654	7	3166
chr5	64987525	C	1268	1004	1622	1444	247	166	120	98	446	209	1575	1597
chr5	64987526	G	979	783	1135	932	435	331	200	162	533	369	579	463
chr5	64987528	G	3	1758	7	2057	2	764	0	363	2	899	2	1039
chr5	64987529	C	18	2253	23	3037	1	407	1	217	0	655	32	3138
chr5	64987534	C	2	2268	2	3056	2	405	0	218	3	650	6	3162
chr5	64987536	G	23	1739	24	2040	16	749	9	354	11	890	12	1028
chr5	64987537	C	34	2236	2	3055	2	405	2	216	9	643	42	3129
chr5	64987539	G	10	1751	17	2044	1	764	3	360	7	893	4	1036
chr5	64987540	C	1	2267	2	3055	1	405	1	217	2	650	6	3163
chr5	64987545	G	3	1752	3	2061	1	764	2	361	2	897	2	1036
chr5	64987547	G	10	1750	3	2060	1	765	2	360	4	896	4	1035
chr5	64987548	C	2	2265	5	3053	1	405	0	218	2	650	11	3156
chr5	64987551	G	4	1755	1	2063	7	758	7	355	6	892	4	1035

81	117	92	128	76	115	36	52	84	113	76	100
1796	2255	1984	3034	314	408	175	231	577	677	2015	3135
90	120	97	123	88	115	46	54	88	116	81	105
13	2151	0	2919	3	396	2	219	3	643	30	3050
0	122	1	133	1	128	0	56	0	119	1	110
5	2276	5	3053	4	415	0	232	1	686	4	3157
1835	2293	2080	3083	328	421	186	235	595	692	2063	3180
92	125	84	135	90	128	43	58	85	121	72	112
1	122	0	130	0	119	0	54	1	104	0	106
6	2014	6	2781	0	372	0	216	2	598	7	2869
1911	2287	2469	3071	352	422	196	235	605	690	2607	3174
117	130	113	140	109	129	48	55	79	115	95	111
2	206	1	268	3	77	2	55	1	134	2	243
0	134	0	141	2	136	0	56	0	126	1	111
11	124	6	128	8	118	4	51	7	104	1	104
0	125	5	129	5	117	0	53	9	103	2	103
14	320	16	457	0	93	2	63	6	154	14	284
6	325	3	454	0	95	1	65	0	153	1	287
1	326	2	452	0	95	1	65	0	151	0	288
0	161	0	171	1	140	1	59	0	108	0	121
2	319	1	445	2	96	0	61	0	153	0	268
1	318	0	445	0	95	0	60	1	154	3	269
2	1763	12	2062	3	764	0	365	5	906	1	1044
4	1768	4	2072	0	766	1	364	3	906	4	1050
2	355	1	508	0	116	0	63	0	179	1	379
2	404	3	587	1	127	1	67	1	194	6	449
7	1767	4	2069	1	767	0	364	0	904	4	1046
3	1767	1	2071	2	765	1	364	5	904	5	1046
1	484	0	714	0	144	0	75	0	221	0	603
6	864	7	1227	0	193	0	102	1	321	0	1165
5	2272	1	3064	1	414	0	219	4	666	2	3174
6	2275	3	3070	0	414	0	218	3	658	3	3178
3	2274	21	3069	3	412	0	218	2	656	7	3173
1268	2272	1622	3066	247	413	120	218	446	655	1575	3172
979	1762	1135	2067	435	766	200	362	533	902	579	1042
3	1761	7	2064	2	766	0	363	2	901	2	1041
18	2271	23	3060	1	408	1	218	0	655	32	3170
2	2270	2	3058	2	407	0	218	3	653	6	3168
23	1762	24	2064	16	765	9	363	11	901	12	1040
34	2270	2	3057	2	407	2	218	9	652	42	3171
10	1761	17	2061	1	765	3	363	7	900	4	1040
1	2268	2	3057	1	406	1	218	2	652	6	3169
3	1755	3	2064	1	765	2	363	2	899	2	1038
10	1760	3	2063	1	766	2	362	4	900	4	1039
2	2267	5	3058	1	406	0	218	2	652	11	3167
4	1759	1	2064	7	765	7	362	6	898	4	1039

chr5	64987553	G	3	1757	2	2062	3	762	1	361	5	894	1	1039
chr5	64987554	G	6	1754	3	2059	0	765	0	362	1	898	3	1036
chr5	64987555	C	1617	644	2126	924	289	112	150	66	504	142	2141	1017
chr5	64987556	G	1163	596	1408	654	541	223	251	111	656	242	658	380
chr5	64987558	G	3	1756	20	2042	0	765	1	361	3	895	1	1039
chr5	64987559	C	6	2257	12	3026	1	403	0	214	3	638	6	3149
chr5	64987561	C	1	2260	34	3002	1	402	1	213	2	636	11	3147
chr5	64987563	C	10	2249	2	3029	1	400	3	210	0	638	12	3143
chr5	64987565	G	3	1757	21	2043	4	758	1	361	7	889	1	1038
chr5	64987569	C	7	2244	5	3018	0	397	1	210	4	623	1	3152
chr5	64987571	G	2	1757	4	2059	3	756	0	362	3	894	0	1038
chr5	64987574	G	3	1756	2	2061	0	759	2	359	1	897	1	1038
chr5	64987581	C	6	2215	10	2989	0	391	0	202	4	606	14	3130
chr5	64987583	C	6	2213	5	2992	0	390	0	202	3	605	6	3133
chr5	64987587	G	1	1754	2	2057	2	752	0	361	2	889	1	1032
chr5	64987591	G	4	1751	7	2052	5	750	1	360	2	887	2	1030
chr5	64987595	G	1	1752	2	2054	1	753	0	361	2	889	2	1028
chr5	64987596	C	11	2205	3	2987	1	387	1	201	0	604	7	3132
chr5	64987604	C	50	2155	14	2971	5	380	0	197	19	580	21	3109
chr5	64987607	C	2	2203	3	2981	1	383	0	197	0	600	6	3123
chr5	64987610	G	0	1753	12	2043	1	751	0	358	5	883	1	1029
chr5	64987611	G	3	1750	0	2054	1	751	1	356	0	889	0	1030
chr5	64987614	C	5	2176	25	2932	0	380	0	194	2	589	3	3082
chr5	64987615	C	8	2191	8	2972	0	383	2	195	1	596	10	3115
chr5	64987616	C	18	2178	3	2974	7	376	2	195	9	588	27	3100
chr5	64987618	C	4	2190	9	2966	1	380	1	196	3	587	23	3104
chr5	64987623	G	0	1750	0	2047	0	748	0	356	0	886	0	1028
chr5	64987624	C	8	2177	8	2944	0	378	1	196	7	576	16	3095
chr5	64987626	C	6	2167	24	2887	3	371	2	194	5	577	12	3050
chr5	64987631	G	0	1745	0	2046	0	748	0	354	0	884	0	1027
chr5	64987632	C	33	2089	104	2673	7	361	12	182	12	566	13	3031
chr5	64987634	G	2	1741	0	2046	0	748	0	355	0	882	0	1027
chr5	64987635	C	0	2037	0	2695	0	341	0	176	0	541	0	2977
chr5	64987637	C	0	2038	0	2698	0	348	0	175	0	550	0	2980
chr5	64987640	C	0	2037	0	2698	0	348	0	175	0	551	1	2977
chr5	64987641	C	2	2035	1	2696	0	348	0	175	0	551	0	2979
chr5	64987643	G	0	1685	0	1987	0	723	0	342	0	872	0	994
chr5	64987644	G	0	1434	1	1695	1	594	0	301	0	758	0	864
chr5	64987649	C	0	1986	1	2611	0	323	0	166	0	507	2	2872
chr5	64987654	C	0	1962	1	2580	0	314	0	164	0	499	1	2822
chr5	64987656	C	0	1928	0	2530	0	306	0	163	0	489	1	2779
chr5	64987657	C	0	1917	1	2513	0	304	0	160	0	487	0	2767
chr5	64987658	C	0	1896	0	2489	0	298	0	159	0	475	0	2745
chr5	64987661	C	0	1866	0	2445	0	293	0	154	0	464	0	2687
chr8	89376778	G	0	3689	1	5422	0	1480	0	505	0	806	0	1457
chr8	89376779	G	0	3714	0	5463	0	1494	0	509	0	813	0	1465

3	1760	2	2064	3	765	1	362	5	899	1	1040
6	1760	3	2062	0	765	0	362	1	899	3	1039
1617	2261	2126	3050	289	401	150	216	504	646	2141	3158
1163	1759	1408	2062	541	764	251	362	656	898	658	1038
3	1759	20	2062	0	765	1	362	3	898	1	1040
6	2263	12	3038	1	404	0	214	3	641	6	3155
1	2261	34	3036	1	403	1	214	2	638	11	3158
10	2259	2	3031	1	401	3	213	0	638	12	3155
3	1760	21	2064	4	762	1	362	7	896	1	1039
7	2251	5	3023	0	397	1	211	4	627	1	3153
2	1759	4	2063	3	759	0	362	3	897	0	1038
3	1759	2	2063	0	759	2	361	1	898	1	1039
6	2221	10	2999	0	391	0	202	4	610	14	3144
6	2219	5	2997	0	390	0	202	3	608	6	3139
1	1755	2	2059	2	754	0	361	2	891	1	1033
4	1755	7	2059	5	755	1	361	2	889	2	1032
1	1753	2	2056	1	754	0	361	2	891	2	1030
11	2216	3	2990	1	388	1	202	0	604	7	3139
50	2205	14	2985	5	385	0	197	19	599	21	3130
2	2205	3	2984	1	384	0	197	0	600	6	3129
0	1753	12	2055	1	752	0	358	5	888	1	1030
3	1753	0	2054	1	752	1	357	0	889	0	1030
5	2181	25	2957	0	380	0	194	2	591	3	3085
8	2199	8	2980	0	383	2	197	1	597	10	3125
18	2196	3	2977	7	383	2	197	9	597	27	3127
4	2194	9	2975	1	381	1	197	3	590	23	3127
0	1750	0	2047	0	748	0	356	0	886	0	1028
8	2185	8	2952	0	378	1	197	7	583	16	3111
6	2173	24	2911	3	374	2	196	5	582	12	3062
0	1745	0	2046	0	748	0	354	0	884	0	1027
33	2122	104	2777	7	368	12	194	12	578	13	3044
2	1743	0	2046	0	748	0	355	0	882	0	1027
0	2037	0	2695	0	341	0	176	0	541	0	2977
0	2038	0	2698	0	348	0	175	0	550	0	2980
0	2037	0	2698	0	348	0	175	0	551	1	2978
2	2037	1	2697	0	348	0	175	0	551	0	2979
0	1685	0	1987	0	723	0	342	0	872	0	994
0	1434	1	1696	1	595	0	301	0	758	0	864
0	1986	1	2612	0	323	0	166	0	507	2	2874
0	1962	1	2581	0	314	0	164	0	499	1	2823
0	1928	0	2530	0	306	0	163	0	489	1	2780
0	1917	1	2514	0	304	0	160	0	487	0	2767
0	1896	0	2489	0	298	0	159	0	475	0	2745
0	1866	0	2445	0	293	0	154	0	464	0	2687
0	3689	1	5423	0	1480	0	505	0	806	0	1457
0	3714	0	5463	0	1494	0	509	0	813	0	1465

chr8	89376781	G	0	3787	1	5556	1	1513	0	516	0	815	0	1490
chr8	89376783	G	1	3922	2	5747	1	1569	1	535	0	842	0	1540
chr8	89376785	C	0	4521	0	5353	0	1363	0	4476	0	710	0	832
chr8	89376788	C	1	4526	0	5355	0	1365	1	4477	0	711	0	831
chr8	89376790	c	0	4529	0	5357	0	1366	0	4485	0	711	0	832
chr8	89376791	c	1	4530	2	5370	0	1366	1	4494	0	713	0	833
chr8	89376793	g	1	5443	2	8256	0	2081	0	778	1	1132	0	2052
chr8	89376794	g	0	5449	1	8264	0	2082	0	778	0	1135	0	2053
chr8	89376795	c	2	4540	0	5387	0	1366	0	4509	0	713	0	837
chr8	89376796	c	0	4544	1	5386	0	1367	0	4511	0	713	1	836
chr8	89376798	g	1	5471	0	8283	0	2092	0	781	1	1148	1	2067
chr8	89376799	c	1	4547	0	5389	0	1366	0	4511	1	711	2	835
chr8	89376805	g	12	5527	8	8312	3	2096	0	791	2	1157	0	2118
chr8	89376807	c	5	4549	5	5394	0	1368	6	4511	1	720	2	838
chr8	89376808	c	5	4548	12	5387	4	1365	6	4511	1	720	2	838
chr8	89376811	c	15	4534	11	5385	2	1365	12	4502	2	720	0	839
chr8	89376815	g	43	5501	77	8259	14	2086	0	792	10	1158	1	2132
chr8	89376818	g	12	5537	15	8327	1	2101	2	791	4	1162	12	2118
chr8	89376819	c	4	4554	6	5394	3	1365	5	4513	1	723	4	836
chr8	89376820	c	12	4547	4	5402	4	1364	6	4512	3	721	4	836
chr8	89376823	c	2301	2254	2632	2766	726	642	2214	2302	376	347	376	464
chr8	89376824	g	2778	2761	3772	4555	961	1140	334	457	702	475	993	1155
chr8	89376827	g	18	5544	9	8342	7	2099	0	799	4	1185	7	2159
chr8	89376829	c	13	4549	4	5402	6	1363	13	4503	3	722	2	839
chr8	89376830	c	11	4553	10	5396	2	1367	8	4509	2	724	3	838
chr8	89376831	c	3	4562	12	5395	0	1369	10	4509	0	725	3	838
chr8	89376833	g	12	5558	10	8348	5	2105	0	798	1	1192	9	2164
chr8	89376836	c	5	4562	8	5400	2	1367	11	4510	2	724	1	841
chr8	89376839	c	11	4558	6	5403	3	1365	14	4507	0	726	1	841
chr8	89376840	c	29	4540	27	5381	4	1364	10	4514	2	724	3	839
chr8	89376842	g	6	5567	12	8353	4	2107	0	801	2	1196	3	2173
chr8	89376844	c	4	4565	7	5402	2	1378	23	4504	0	747	3	860
chr8	89376845	c	21	4550	9	5403	8	1375	7	4521	4	746	1	861
chr8	89376847	c	19	4553	84	5330	4	1379	5	4524	4	747	6	856
chr8	89376849	g	20	5552	10	8356	14	2096	1	800	3	1196	17	2163
chr8	89376850	c	11	4531	43	5322	1	1375	15	4473	2	747	0	860
chr8	89376851	c	6	4568	3	5413	0	1385	6	4523	2	751	2	860
chr8	89376852	c	18	4555	14	5400	0	1385	8	4521	2	751	2	861
chr8	89376855	c	6	4566	11	5401	2	1370	13	4513	1	732	0	843
chr8	89376856	c	11	4560	43	5372	5	1367	74	4448	4	729	1	842
chr8	89376857	c	3690	886	4135	1281	1181	204	3434	1096	601	151	664	199
chr8	89376858	g	4059	1511	6080	2280	1506	605	582	220	927	269	1607	574
chr8	89376859	c	66	4507	9	5403	26	1346	64	4460	7	725	11	832
chr8	89376861	c	9	4565	12	5402	0	1384	17	4514	4	749	1	863
chr8	89376862	c	93	4485	112	5305	17	1368	133	4398	8	745	7	857
chr8	89376865	c	22	4557	9	5407	5	1380	47	4484	0	756	1	861

0	3787	1	5557	1	1514	0	516	0	815	0	1490
1	3923	2	5749	1	1570	1	536	0	842	0	1540
0	4521	0	5353	0	1363	0	4476	0	710	0	832
1	4527	0	5355	0	1365	1	4478	0	711	0	831
0	4529	0	5357	0	1366	0	4485	0	711	0	832
1	4531	2	5372	0	1366	1	4495	0	713	0	833
1	5444	2	8258	0	2081	0	778	1	1133	0	2052
0	5449	1	8265	0	2082	0	778	0	1135	0	2053
2	4542	0	5387	0	1366	0	4509	0	713	0	837
0	4544	1	5387	0	1367	0	4511	0	713	1	837
1	5472	0	8283	0	2092	0	781	1	1149	1	2068
1	4548	0	5389	0	1366	0	4511	1	712	2	837
12	5539	8	8320	3	2099	0	791	2	1159	0	2118
5	4554	5	5399	0	1368	6	4517	1	721	2	840
5	4553	12	5399	4	1369	6	4517	1	721	2	840
15	4549	11	5396	2	1367	12	4514	2	722	0	839
43	5544	77	8336	14	2100	0	792	10	1168	1	2133
12	5549	15	8342	1	2102	2	793	4	1166	12	2130
4	4558	6	5400	3	1368	5	4518	1	724	4	840
12	4559	4	5406	4	1368	6	4518	3	724	4	840
2301	4555	2632	5398	726	1368	2214	4516	376	723	376	840
2778	5539	3772	8327	961	2101	334	791	702	1177	993	2148
18	5562	9	8351	7	2106	0	799	4	1189	7	2166
13	4562	4	5406	6	1369	13	4516	3	725	2	841
11	4564	10	5406	2	1369	8	4517	2	726	3	841
3	4565	12	5407	0	1369	10	4519	0	725	3	841
12	5570	10	8358	5	2110	0	798	1	1193	9	2173
5	4567	8	5408	2	1369	11	4521	2	726	1	842
11	4569	6	5409	3	1368	14	4521	0	726	1	842
29	4569	27	5408	4	1368	10	4524	2	726	3	842
6	5573	12	8365	4	2111	0	801	2	1198	3	2176
4	4569	7	5409	2	1380	23	4527	0	747	3	863
21	4571	9	5412	8	1383	7	4528	4	750	1	862
19	4572	84	5414	4	1383	5	4529	4	751	6	862
20	5572	10	8366	14	2110	1	801	3	1199	17	2180
11	4542	43	5365	1	1376	15	4488	2	749	0	860
6	4574	3	5416	0	1385	6	4529	2	753	2	862
18	4573	14	5414	0	1385	8	4529	2	753	2	863
6	4572	11	5412	2	1372	13	4526	1	733	0	843
11	4571	43	5415	5	1372	74	4522	4	733	1	843
3690	4576	4135	5416	1181	1385	3434	4530	601	752	664	863
4059	5570	6080	8360	1506	2111	582	802	927	1196	1607	2181
66	4573	9	5412	26	1372	64	4524	7	732	11	843
9	4574	12	5414	0	1384	17	4531	4	753	1	864
93	4578	112	5417	17	1385	133	4531	8	753	7	864
22	4579	9	5416	5	1385	47	4531	0	756	1	862

chr8	89376867	c	6	4572	5	5413	5	1380	10	4520	5	752	0	864
chr8	89376868	c	13	4567	5	5412	2	1383	3	4530	1	756	2	864
chr8	89376869	c	3302	1277	3298	2116	1019	366	2909	1625	537	221	509	355
chr8	89376870	g	3601	1972	4591	3773	1331	779	479	323	824	383	1285	910
chr8	89376872	g	20	5558	33	8336	18	2091	1	802	6	1203	7	2192
chr8	89376873	c	23	4556	6	5413	5	1380	10	4523	1	756	1	866
chr8	89376875	c	34	4549	35	5385	8	1377	5	4531	6	752	7	860
chr8	89376877	g	17	5563	51	8316	1	2110	2	800	4	1208	9	2198
chr8	89376879	c	122	4464	94	5326	38	1347	86	4450	13	745	23	847
chr8	89376881	c	9	4578	19	5398	2	1383	5	4533	0	759	0	871
chr8	89376882	c	21	4565	54	5366	7	1379	17	4520	5	754	1	870
chr8	89376884	c	47	4540	106	5314	13	1373	14	4524	6	753	4	867
chr8	89376886	g	3	5581	9	8361	4	2108	2	800	4	1210	3	2207
chr8	89376887	g	5	5580	19	8344	1	2109	1	803	1	1212	2	2208
chr8	89376889	g	434	5154	622	7749	202	1910	45	759	109	1107	215	1995
chr8	89376891	g	106	5481	153	8219	31	2080	28	776	27	1189	40	2169
chr8	89376892	c	4370	224	4819	602	1341	45	4219	324	722	42	825	51
chr8	89376893	g	5051	532	7632	735	1953	156	722	78	1125	89	1992	214
chr8	89376894	c	31	4561	52	5370	14	1372	40	4504	6	758	21	856
chr8	89376897	c	12	4584	0	5426	1	1386	3	4542	2	762	3	874
chr8	89376898	c	6	4590	14	5412	3	1384	25	4520	2	762	1	876
chr8	89376902	c	213	4384	227	5202	66	1321	263	4282	36	732	34	843
chr8	89376904	G	10	5581	60	8314	3	2108	3	801	1	1216	4	2208
chr8	89376906	C	9	4586	18	5405	4	1384	10	4535	0	768	1	875
chr8	89376911	C	3975	625	4619	811	1231	159	3924	626	663	105	735	144
chr8	89376912	G	4679	915	6640	1744	1767	348	658	146	1046	171	1838	374
chr8	89376916	G	18	5579	41	8345	16	2099	4	800	4	1213	24	2186
chr8	89376917	C	8	4590	7	5424	1	1388	5	4544	2	766	1	878
chr8	89376925	C	55	4543	52	5381	7	1383	111	4438	4	764	2	877
chr8	89376926	C	3818	780	4003	1430	1196	193	3656	893	628	139	682	196
chr8	89376927	G	4416	1179	6054	2324	1654	461	584	219	977	238	1713	496
chr8	89376928	G	6	2344	6	3279	0	815	0	358	1	492	1	840
chr8	89376930	C	66	4532	52	5377	24	1365	47	4497	11	757	10	869
chr8	89376932	C	47	4548	37	5387	7	1379	3	4525	3	762	0	878
chr8	89376935	C	9	4584	8	5413	0	1383	8	4520	4	761	3	874
chr8	89376943	G	1	985	2	824	0	422	0	107	1	203	4	314
chr8	89376948	C	12	4582	8	5416	3	1381	4	4522	2	762	0	877
chr8	89376950	G	0	1089	0	1004	2	449	0	110	1	209	1	339
chr8	89376952	C	7	4585	4	5419	9	1375	0	4525	1	763	1	876
chr8	89376956	C	20	4569	55	5368	3	1379	19	4507	3	761	2	875
chr8	89376959	G	24	5560	27	8360	7	2076	1	778	3	1194	4	2207
chr8	89376962	G	8	5571	7	8378	1	2076	1	764	2	1181	4	2204
chr8	89376964	G	79	5501	124	8260	21	2059	3	774	17	1180	28	2184
chr8	89376965	G	17	5563	16	8364	1	2074	2	774	0	1196	7	2205
chr8	89376966	C	13	4575	10	5410	2	1379	13	4510	1	762	1	873
chr8	89376967	C	23	4563	9	5411	1	1379	5	4518	3	760	3	874

6	4578	5	5418	5	1385	10	4530	5	757	0	864
13	4580	5	5417	2	1385	3	4533	1	757	2	866
3302	4579	3298	5414	1019	1385	2909	4534	537	758	509	864
3601	5573	4591	8364	1331	2110	479	802	824	1207	1285	2195
20	5578	33	8369	18	2109	1	803	6	1209	7	2199
23	4579	6	5419	5	1385	10	4533	1	757	1	867
34	4583	35	5420	8	1385	5	4536	6	758	7	867
17	5580	51	8367	1	2111	2	802	4	1212	9	2207
122	4586	94	5420	38	1385	86	4536	13	758	23	870
9	4587	19	5417	2	1385	5	4538	0	759	0	871
21	4586	54	5420	7	1386	17	4537	5	759	1	871
47	4587	106	5420	13	1386	14	4538	6	759	4	871
3	5584	9	8370	4	2112	2	802	4	1214	3	2210
5	5585	19	8363	1	2110	1	804	1	1213	2	2210
434	5588	622	8371	202	2112	45	804	109	1216	215	2210
106	5587	153	8372	31	2111	28	804	27	1216	40	2209
4370	4594	4819	5421	1341	1386	4219	4543	722	764	825	876
5051	5583	7632	8367	1953	2109	722	800	1125	1214	1992	2206
31	4592	52	5422	14	1386	40	4544	6	764	21	877
12	4596	0	5426	1	1387	3	4545	2	764	3	877
6	4596	14	5426	3	1387	25	4545	2	764	1	877
213	4597	227	5429	66	1387	263	4545	36	768	34	877
10	5591	60	8374	3	2111	3	804	1	1217	4	2212
9	4595	18	5423	4	1388	10	4545	0	768	1	876
3975	4600	4619	5430	1231	1390	3924	4550	663	768	735	879
4679	5594	6640	8384	1767	2115	658	804	1046	1217	1838	2212
18	5597	41	8386	16	2115	4	804	4	1217	24	2210
8	4598	7	5431	1	1389	5	4549	2	768	1	879
55	4598	52	5433	7	1390	111	4549	4	768	2	879
3818	4598	4003	5433	1196	1389	3656	4549	628	767	682	878
4416	5595	6054	8378	1654	2115	584	803	977	1215	1713	2209
6	2350	6	3285	0	815	0	358	1	493	1	841
66	4598	52	5429	24	1389	47	4544	11	768	10	879
47	4595	37	5424	7	1386	3	4528	3	765	0	878
9	4593	8	5421	0	1383	8	4528	4	765	3	877
1	986	2	826	0	422	0	107	1	204	4	318
12	4594	8	5424	3	1384	4	4526	2	764	0	877
0	1089	0	1004	2	451	0	110	1	210	1	340
7	4592	4	5423	9	1384	0	4525	1	764	1	877
20	4589	55	5423	3	1382	19	4526	3	764	2	877
24	5584	27	8387	7	2083	1	779	3	1197	4	2211
8	5579	7	8385	1	2077	1	765	2	1183	4	2208
79	5580	124	8384	21	2080	3	777	17	1197	28	2212
17	5580	16	8380	1	2075	2	776	0	1196	7	2212
13	4588	10	5420	2	1381	13	4523	1	763	1	874
23	4586	9	5420	1	1380	5	4523	3	763	3	877

chr8	89376968	C	8	4580	1	5417	2	1380	3	4518	4	758	2	875
chr8	89376971	G	18	5547	8	8369	2	2059	1	759	1	1176	7	2199
chr8	89376973	G	116	5445	29	8346	22	2037	4	755	20	1155	32	2174
chr8	89376974	C	11	4571	26	5387	3	1377	48	4470	4	755	3	873
chr8	89376976	C	3249	1333	3521	1887	1008	368	3203	1314	518	239	597	274
chr8	89376977	G	3773	1786	5683	2689	1374	680	540	219	848	329	1559	646
chr8	89376981	C	14	4561	17	5383	6	1371	3	4508	0	756	1	873
chr8	89376986	C	22	4549	13	5389	3	1373	9	4496	0	756	2	868
chr8	89376990	G	20	5530	32	8333	8	2035	1	754	2	1168	11	2189
chr8	89376991	C	223	4344	335	5066	80	1293	244	4255	38	708	43	821
chr8	89376993	C	26	4541	12	5387	3	1369	4	4493	2	745	2	861
chr8	89376994	C	15	4552	6	5394	6	1367	5	4492	2	744	3	859
chr8	89376999	G	23	5434	67	8209	13	1948	9	726	13	1133	17	2177
chr8	89377002	G	55	5400	210	8068	36	1920	0	734	21	1125	24	2170
chr8	89377009	G	9	5440	1	8274	3	1948	0	732	6	1138	1	2189
chr8	89377012	G	13	5437	31	8244	14	1937	1	731	3	1141	11	2179
chr8	89377013	C	8	4543	6	5386	1	1370	9	4467	2	736	2	859
chr8	89377023	C	10	4533	7	5372	3	1367	3	4463	1	737	3	857
chr8	89377026	G	10	5431	13	8248	4	1947	0	730	5	1139	6	2180
chr8	89377027	C	8	4529	43	5336	4	1366	5	4460	1	737	0	858
chr8	89377028	C	5	4532	6	5372	4	1366	10	4456	2	736	7	852
chr8	89377030	G	11	5429	9	8247	3	1941	11	720	3	1140	12	2174
chr8	89377037	G	17	5386	4	8210	5	1926	3	724	2	1127	9	2163
chr8	89377038	G	5	5430	11	8237	2	1938	3	726	2	1136	2	2180
chr8	89377042	G	2	5435	13	8235	1	1940	2	726	3	1135	5	2176
chr8	89377045	C	0	4492	0	5295	0	1367	0	4448	0	737	0	857
chr8	89377047	C	1	4485	1	5286	0	1366	0	4445	0	736	0	857
chr8	89377049	G	14	5418	33	8216	3	1936	0	727	2	1135	4	2177
chr8	89377050	G	11	5421	17	8228	6	1933	0	727	1	1135	3	2177
chr8	89377051	G	11	5417	19	8218	3	1936	0	725	1	1130	6	2169
chr8	89377052	C	0	4450	1	5249	0	1357	0	4374	0	727	1	842
chr8	89377053	C	0	4414	1	5201	0	1352	0	4326	0	724	0	840
chr8	89377055	C	0	4390	2	5182	0	1346	0	4302	0	723	0	838
chr8	89377057	C	1	4376	0	5167	1	1343	0	4292	0	719	0	837
chr8	89377058	C	1	4374	0	5168	0	1345	0	4291	0	719	1	835
chr8	89377060	G	51	5333	32	8187	30	1897	21	702	15	1109	34	2131
chr8	89377062	G	23	5358	49	8166	3	1916	2	721	7	1116	3	2158
chr8	89377069	G	20	5353	13	8198	2	1914	0	722	1	1121	2	2159
chr8	89377070	G	10	5362	12	8200	2	1914	0	723	0	1123	2	2159
chr8	89377076	G	4548	719	6690	1405	1659	252	660	63	1031	91	1904	250
chr8	89377081	G	0	5020	0	8034	0	1886	0	722	0	1118	0	2138
chr8	89377091	G	0	4759	1	7719	0	1712	0	697	0	1070	0	2039
chr8	89377093	G	1	4757	3	7701	1	1707	0	698	0	1069	0	2041
chr8	89377094	G	0	4759	1	7708	0	1708	0	699	1	1069	0	2040
chr8	89377095	G	0	4760	0	7709	0	1708	0	699	0	1070	0	2043
chr8	120115553	G	1	9663	4	15849	0	2925	0	1948	0	1884	0	3645

8	4588	1	5418	2	1382	3	4521	4	762	2	877
18	5565	8	8377	2	2061	1	760	1	1177	7	2206
116	5561	29	8375	22	2059	4	759	20	1175	32	2206
11	4582	26	5413	3	1380	48	4518	4	759	3	876
3249	4582	3521	5408	1008	1376	3203	4517	518	757	597	871
3773	5559	5683	8372	1374	2054	540	759	848	1177	1559	2205
14	4575	17	5400	6	1377	3	4511	0	756	1	874
22	4571	13	5402	3	1376	9	4505	0	756	2	870
20	5550	32	8365	8	2043	1	755	2	1170	11	2200
223	4567	335	5401	80	1373	244	4499	38	746	43	864
26	4567	12	5399	3	1372	4	4497	2	747	2	863
15	4567	6	5400	6	1373	5	4497	2	746	3	862
23	5457	67	8276	13	1961	9	735	13	1146	17	2194
55	5455	210	8278	36	1956	0	734	21	1146	24	2194
9	5449	1	8275	3	1951	0	732	6	1144	1	2190
13	5450	31	8275	14	1951	1	732	3	1144	11	2190
8	4551	6	5392	1	1371	9	4476	2	738	2	861
10	4543	7	5379	3	1370	3	4466	1	738	3	860
10	5441	13	8261	4	1951	0	730	5	1144	6	2186
8	4537	43	5379	4	1370	5	4465	1	738	0	858
5	4537	6	5378	4	1370	10	4466	2	738	7	859
11	5440	9	8256	3	1944	11	731	3	1143	12	2186
17	5403	4	8214	5	1931	3	727	2	1129	9	2172
5	5435	11	8248	2	1940	3	729	2	1138	2	2182
2	5437	13	8248	1	1941	2	728	3	1138	5	2181
0	4492	0	5295	0	1367	0	4448	0	737	0	857
1	4486	1	5287	0	1366	0	4445	0	736	0	857
14	5432	33	8249	3	1939	0	727	2	1137	4	2181
11	5432	17	8245	6	1939	0	727	1	1136	3	2180
11	5428	19	8237	3	1939	0	725	1	1131	6	2175
0	4450	1	5250	0	1357	0	4374	0	727	1	843
0	4414	1	5202	0	1352	0	4326	0	724	0	840
0	4390	2	5184	0	1346	0	4302	0	723	0	838
1	4377	0	5167	1	1344	0	4292	0	719	0	837
1	4375	0	5168	0	1345	0	4291	0	719	1	836
51	5384	32	8219	30	1927	21	723	15	1124	34	2165
23	5381	49	8215	3	1919	2	723	7	1123	3	2161
20	5373	13	8211	2	1916	0	722	1	1122	2	2161
10	5372	12	8212	2	1916	0	723	0	1123	2	2161
4548	5267	6690	8095	1659	1911	660	723	1031	1122	1904	2154
0	5020	0	8034	0	1886	0	722	0	1118	0	2138
0	4759	1	7720	0	1712	0	697	0	1070	0	2039
1	4758	3	7704	1	1708	0	698	0	1069	0	2041
0	4759	1	7709	0	1708	0	699	1	1070	0	2040
0	4760	0	7709	0	1708	0	699	0	1070	0	2043
1	9664	4	15853	0	2925	0	1948	0	1884	0	3645

chr8	120115555	G	2	9699	7	15947	1	2943	1	1955	0	1902	0	3666
chr8	120115560	G	0	9725	2	15988	0	2961	0	1973	0	1920	0	3680
chr8	120115562	G	1	9728	6	16005	0	2964	0	1976	0	1923	0	3685
chr8	120115563	G	8	9744	31	16084	6	2962	0	1980	3	1922	8	3680
chr8	120115567	G	120	9635	160	15969	49	2923	20	1961	22	1903	69	3622
chr8	120115568	G	32	9732	64	16082	6	2965	8	1973	7	1918	2	3689
chr8	120115569	G	15	9749	25	16122	4	2967	4	1978	2	1922	2	3688
chr8	120115570	G	11	9750	30	16121	6	2966	11	1971	1	1924	6	3685
chr8	120115575	G	28	9735	31	16131	2	2975	1	1981	2	1925	8	3685
chr8	120115582	G	27	9752	29	16161	3	2978	3	1981	3	1930	4	3694
chr8	120115584	G	33	9749	22	16172	16	2967	5	1980	2	1935	14	3682
chr8	120115588	G	8937	790	14194	1920	2753	217	1711	263	1779	148	3147	538
chr8	120115589	G	19	9760	73	16126	15	2969	1	1983	5	1934	7	3695
chr8	120115593	G	77	9710	23	16184	15	2973	14	1970	8	1934	14	3690
chr8	120115594	G	9	9780	12	16198	0	2990	12	1973	2	1940	5	3700
chr8	120115595	G	24	9763	34	16180	7	2984	3	1981	4	1942	8	3695
chr8	120115599	G	9192	592	14627	1590	2826	166	1840	143	1836	113	3361	345
chr8	120115602	G	44	9754	85	16144	16	2978	8	1977	16	1934	25	3682
chr8	120115604	G	141	9657	105	16126	53	2942	37	1948	23	1929	66	3641
chr8	120115606	C	0	1124	0	1738	0	663	0	464	0	486	0	1161
chr8	120115608	G	668	9133	1051	15183	198	2797	131	1854	166	1786	240	3467
chr8	120115610	G	34	9770	116	16124	24	2973	15	1972	19	1933	24	3685
chr8	120115611	C	0	1122	0	1743	0	666	0	466	0	487	0	1163
chr8	120115614	G	120	9684	266	15984	22	2978	42	1949	38	1916	36	3674
chr8	120115616	C	0	1128	0	1746	0	665	0	466	0	487	1	1164
chr8	120115619	G	52	9827	229	16020	5	3150	8	2043	10	2096	8	3770
chr8	120115620	G	25	9848	71	16179	8	3139	4	2044	8	2094	9	3760
chr8	120115621	C	0	1077	0	1681	0	645	0	438	0	464	0	1135
chr8	120115623	C	0	1129	0	1749	0	666	0	466	0	487	0	1167
chr8	120115624	C	0	1129	0	1749	0	666	0	466	0	487	0	1167
chr8	120115625	C	0	1129	0	1749	0	666	0	466	0	487	0	1167
chr8	120115627	C	1	1126	2	1747	2	664	0	466	0	486	2	1165
chr8	120115629	G	193	9693	187	16085	57	3114	37	2022	33	2077	60	3721
chr8	120115631	G	40	9854	27	16245	15	3176	1	2066	9	2110	4	3785
chr8	120115632	G	16	9881	26	16249	8	3184	8	2060	8	2112	9	3780
chr8	120115634	C	0	1129	0	1751	1	667	0	467	2	485	14	1152
chr8	120115638	G	21	10147	100	16185	9	3842	7	2388	2	2519	7	3968
chr8	120115639	G	34	10140	19	16266	4	3856	3	2395	3	2521	29	3947
chr8	120115643	C	21	1108	1	1750	3	665	2	465	0	486	1	1166
chr8	120115645	C	52	1077	30	1721	37	631	12	455	16	471	64	1103
chr8	120115647	G	61	10144	36	16255	18	3883	11	2454	15	2543	12	3974
chr8	120115652	G	39	10168	100	16186	19	3885	11	2456	12	2551	29	3952
chr8	120115653	G	22	10190	28	16266	9	3898	3	2465	5	2562	10	3973
chr8	120115654	G	12	10202	49	16230	7	3902	4	2466	5	2563	10	3976
chr8	120115655	C	11	1120	9	1739	4	664	0	466	2	486	8	1159
chr8	120115657	C	4	1127	13	1737	0	668	7	460	1	487	16	1152

2	9701	7	15954	1	2944	1	1956	0	1902	0	3666
0	9725	2	15990	0	2961	0	1973	0	1920	0	3680
1	9729	6	16011	0	2964	0	1976	0	1923	0	3685
8	9752	31	16115	6	2968	0	1980	3	1925	8	3688
120	9755	160	16129	49	2972	20	1981	22	1925	69	3691
32	9764	64	16146	6	2971	8	1981	7	1925	2	3691
15	9764	25	16147	4	2971	4	1982	2	1924	2	3690
11	9761	30	16151	6	2972	11	1982	1	1925	6	3691
28	9763	31	16162	2	2977	1	1982	2	1927	8	3693
27	9779	29	16190	3	2981	3	1984	3	1933	4	3698
33	9782	22	16194	16	2983	5	1985	2	1937	14	3696
8937	9727	14194	16114	2753	2970	1711	1974	1779	1927	3147	3685
19	9779	73	16199	15	2984	1	1984	5	1939	7	3702
77	9787	23	16207	15	2988	14	1984	8	1942	14	3704
9	9789	12	16210	0	2990	12	1985	2	1942	5	3705
24	9787	34	16214	7	2991	3	1984	4	1946	8	3703
9192	9784	14627	16217	2826	2992	1840	1983	1836	1949	3361	3706
44	9798	85	16229	16	2994	8	1985	16	1950	25	3707
141	9798	105	16231	53	2995	37	1985	23	1952	66	3707
0	1124	0	1738	0	663	0	464	0	486	0	1161
668	9801	1051	16234	198	2995	131	1985	166	1952	240	3707
34	9804	116	16240	24	2997	15	1987	19	1952	24	3709
0	1122	0	1743	0	666	0	466	0	487	0	1163
120	9804	266	16250	22	3000	42	1991	38	1954	36	3710
0	1128	0	1746	0	665	0	466	0	487	1	1165
52	9879	229	16249	5	3155	8	2051	10	2106	8	3778
25	9873	71	16250	8	3147	4	2048	8	2102	9	3769
0	1077	0	1681	0	645	0	438	0	464	0	1135
0	1129	0	1749	0	666	0	466	0	487	0	1167
0	1129	0	1749	0	666	0	466	0	487	0	1167
0	1129	0	1749	0	666	0	466	0	487	0	1167
1	1127	2	1749	2	666	0	466	0	486	2	1167
193	9886	187	16272	57	3171	37	2059	33	2110	60	3781
40	9894	27	16272	15	3191	1	2067	9	2119	4	3789
16	9897	26	16275	8	3192	8	2068	8	2120	9	3789
0	1129	0	1751	1	668	0	467	2	487	14	1166
21	10168	100	16285	9	3851	7	2395	2	2521	7	3975
34	10174	19	16285	4	3860	3	2398	3	2524	29	3976
21	1129	1	1751	3	668	2	467	0	486	1	1167
52	1129	30	1751	37	668	12	467	16	487	64	1167
61	10205	36	16291	18	3901	11	2465	15	2558	12	3986
39	10207	100	16286	19	3904	11	2467	12	2563	29	3981
22	10212	28	16294	9	3907	3	2468	5	2567	10	3983
12	10214	49	16279	7	3909	4	2470	5	2568	10	3986
11	1131	9	1748	4	668	0	466	2	488	8	1167
4	1131	13	1750	0	668	7	467	1	488	16	1168

chr8	120115659	C	1040	91	1422	329	595	73	389	78	429	59	954	215
chr8	120115660	G	8963	1262	14362	1946	3371	541	2128	348	2232	345	3376	611
chr8	120115664	G	102	10120	186	16114	37	3882	6	2475	32	2548	25	3962
chr8	120115665	C	6	1128	18	1731	7	660	0	467	8	480	19	1150
chr8	120115669	C	1	1134	2	1749	1	668	0	467	1	488	5	1164
chr8	120115671	C	1037	98	1602	149	610	59	390	77	456	33	1044	125
chr8	120115672	G	9117	1115	14489	1826	3546	378	2108	380	2308	288	3368	623
chr8	120115673	G	22	10206	41	16275	13	3911	6	2481	10	2587	10	3978
chr8	120115674	C	13	1122	5	1745	3	664	4	463	3	486	23	1146
chr8	120115676	G	130	10099	280	16042	18	3908	10	2481	24	2574	30	3960
chr8	120115679	C	1094	42	1609	142	638	31	435	32	473	16	1120	49
chr8	120115680	G	9429	803	14689	1637	3628	295	2257	232	2430	168	3514	474
chr8	120115683	G	23	10211	52	16270	23	3906	15	2478	13	2586	5	3985
chr8	120115686	C	8	1127	84	1668	11	659	2	465	5	484	9	1160
chr8	120115688	C	1	1135	1	1751	2	668	3	464	2	487	3	1166
chr8	120115690	C	1028	108	1570	182	621	49	424	43	460	29	1062	107
chr8	120115691	G	814	123	830	106	1053	193	621	137	736	133	403	92
chr8	120115692	C	11	1124	5	1746	3	666	2	465	0	489	16	1152
chr8	120115695	G	1	925	3	865	2	1257	1	768	3	878	0	499
chr8	120115697	C	4	1132	4	1748	1	668	1	466	1	488	3	1166
chr8	120115698	C	1063	73	1600	152	632	37	413	54	454	35	1038	132
chr8	120115699	G	837	99	733	132	1123	141	667	104	789	94	434	69
chr8	120115702	G	26	2378	6	3612	7	1698	1	1078	4	1142	0	1112
chr8	120115705	C	0	1135	2	1750	2	667	5	462	1	488	3	1167
chr8	120115707	C	8	1128	14	1738	15	655	3	464	12	477	14	1156
chr8	120115709	G	27	10204	32	16286	10	3940	2	2507	7	2614	9	3988
chr8	120115710	C	4	1132	5	1746	0	670	0	467	3	486	0	1169
chr8	120115711	C	7	1129	14	1738	13	657	1	466	14	475	7	1163
chr8	120115713	G	80	10156	69	16261	20	3931	4	2506	16	2608	3	3994
chr8	120115716	C	16	1120	16	1736	13	657	3	464	8	481	33	1137
chr8	120115718	C	49	1087	69	1683	21	649	5	462	12	477	24	1146
chr8	120115720	C	73	1062	135	1616	42	628	17	450	41	448	80	1090
chr8	120115722	C	3	1133	12	1739	5	665	1	466	11	477	4	1166
chr8	120115725	G	39	10158	55	16217	23	3904	12	2457	11	2592	18	3959
chr8	120115726	C	9	1127	8	1743	5	665	4	463	12	477	3	1167
chr8	120115728	C	4	1132	9	1742	9	661	1	466	10	478	5	1165
chr8	120115730	C	4	1132	2	1750	6	664	2	465	9	480	21	1149
chr8	120115732	C	922	214	1317	435	553	117	364	103	402	87	942	227
chr8	120115733	G	7774	2440	11940	4373	2930	992	1824	638	2013	572	2807	1163
chr8	120115736	C	5	1131	3	1749	1	669	1	466	5	484	14	1156
chr8	120115741	C	9	1127	13	1739	3	667	3	464	13	476	17	1152
chr8	120115745	C	970	166	1398	354	563	107	378	89	434	54	927	243
chr8	120115746	G	8773	1440	13815	2492	3314	601	2068	395	2249	331	3331	641
chr8	120115751	G	38	10168	20	16285	13	3901	9	2452	12	2567	25	3944
chr8	120115752	C	28	1108	3	1749	5	665	3	464	6	483	82	1084
chr8	120115756	C	5	1131	2	1748	2	668	2	464	1	485	19	1149

1040	1131	1422	1751	595	668	389	467	429	488	954	1169
8963	10225	14362	16308	3371	3912	2128	2476	2232	2577	3376	3987
102	10222	186	16300	37	3919	6	2481	32	2580	25	3987
6	1134	18	1749	7	667	0	467	8	488	19	1169
1	1135	2	1751	1	669	0	467	1	489	5	1169
1037	1135	1602	1751	610	669	390	467	456	489	1044	1169
9117	10232	14489	16315	3546	3924	2108	2488	2308	2596	3368	3991
22	10228	41	16316	13	3924	6	2487	10	2597	10	3988
13	1135	5	1750	3	667	4	467	3	489	23	1169
130	10229	280	16322	18	3926	10	2491	24	2598	30	3990
1094	1136	1609	1751	638	669	435	467	473	489	1120	1169
9429	10232	14689	16326	3628	3923	2257	2489	2430	2598	3514	3988
23	10234	52	16322	23	3929	15	2493	13	2599	5	3990
8	1135	84	1752	11	670	2	467	5	489	9	1169
1	1136	1	1752	2	670	3	467	2	489	3	1169
1028	1136	1570	1752	621	670	424	467	460	489	1062	1169
814	937	830	936	1053	1246	621	758	736	869	403	495
11	1135	5	1751	3	669	2	467	0	489	16	1168
1	926	3	868	2	1259	1	769	3	881	0	499
4	1136	4	1752	1	669	1	467	1	489	3	1169
1063	1136	1600	1752	632	669	413	467	454	489	1038	1170
837	936	733	865	1123	1264	667	771	789	883	434	503
26	2404	6	3618	7	1705	1	1079	4	1146	0	1112
0	1135	2	1752	2	669	5	467	1	489	3	1170
8	1136	14	1752	15	670	3	467	12	489	14	1170
27	10231	32	16318	10	3950	2	2509	7	2621	9	3997
4	1136	5	1751	0	670	0	467	3	489	0	1169
7	1136	14	1752	13	670	1	467	14	489	7	1170
80	10236	69	16330	20	3951	4	2510	16	2624	3	3997
16	1136	16	1752	13	670	3	467	8	489	33	1170
49	1136	69	1752	21	670	5	467	12	489	24	1170
73	1135	135	1751	42	670	17	467	41	489	80	1170
3	1136	12	1751	5	670	1	467	11	488	4	1170
39	10197	55	16272	23	3927	12	2469	11	2603	18	3977
9	1136	8	1751	5	670	4	467	12	489	3	1170
4	1136	9	1751	9	670	1	467	10	488	5	1170
4	1136	2	1752	6	670	2	467	9	489	21	1170
922	1136	1317	1752	553	670	364	467	402	489	942	1169
7774	10214	11940	16313	2930	3922	1824	2462	2013	2585	2807	3970
5	1136	3	1752	1	670	1	467	5	489	14	1170
9	1136	13	1752	3	670	3	467	13	489	17	1169
970	1136	1398	1752	563	670	378	467	434	488	927	1170
8773	10213	13815	16307	3314	3915	2068	2463	2249	2580	3331	3972
38	10206	20	16305	13	3914	9	2461	12	2579	25	3969
28	1136	3	1752	5	670	3	467	6	489	82	1166
5	1136	2	1750	2	670	2	466	1	486	19	1168

chr8	120115758	C	3	1132	17	1730	5	663	6	460	10	477	1	1168
chr8	120115761	G	23	10184	69	16229	5	3906	9	2452	6	2573	13	3954
chr8	120115762	G	22	10174	31	16265	7	3894	2	2447	4	2573	4	3959
chr8	120115764	C	801	322	1168	571	473	193	320	142	363	121	776	388
chr8	120115765	G	7821	2366	12597	3674	2858	1043	1826	619	1951	624	2784	1174
chr8	120115767	G	25	10173	31	16264	9	3894	1	2447	12	2565	9	3953
chr8	120115770	C	4	925	6	1434	2	536	0	373	1	402	14	933
chr8	120115771	C	8	1021	39	1568	4	598	1	413	2	449	1	1052
chr8	120115775	C	45	1074	60	1677	26	628	33	423	31	447	34	1117
chr8	120115777	C	41	1079	45	1692	18	635	12	444	11	467	42	1107
chr8	120115778	C	31	1088	96	1636	20	631	19	436	11	463	30	1121
chr8	120115782	C	76	1042	40	1692	24	625	33	421	31	443	72	1080
chr8	120115783	C	62	1055	67	1666	9	640	10	445	13	461	98	1050
chr8	120115784	C	100	1012	185	1539	54	592	48	404	54	419	77	1073
chr8	120115786	C	20	1094	19	1713	9	641	8	446	8	464	46	1101
chr8	120115793	C	0	1118	4	1729	5	645	0	455	3	470	2	1149
chr8	120115794	C	894	214	1221	486	531	113	329	123	389	74	881	262
chr8	120115795	G	7022	3132	10436	5803	2679	1215	1595	845	1695	873	2479	1468
chr8	120115796	C	964	151	1339	389	554	93	364	89	420	50	939	208
chr8	120115797	G	7340	1416	11486	2191	2805	533	1705	372	1877	329	2717	626
chr8	120115801	G	17	10155	70	16195	14	3879	3	2437	3	2566	5	3946
chr8	120115803	G	49	10123	33	16230	44	3847	5	2435	16	2553	29	3923
chr8	120115805	G	431	9734	507	15746	138	3751	106	2334	98	2470	128	3822
chr8	120115807	G	129	10030	194	16063	59	3831	48	2390	53	2516	96	3854
chr8	120115810	C	2	1116	0	1732	0	648	0	454	0	473	17	1131
chr8	120115811	C	108	1007	156	1567	83	562	48	405	62	409	191	949
chr8	120115813	G	26	10133	17	16227	12	3873	11	2421	9	2552	4	3944
chr8	120115814	G	27	10132	18	16235	7	3875	6	2430	13	2552	12	3936
chr8	120115815	C	5	1113	1	1731	4	643	2	451	0	472	1	1145
chr8	120115816	C	3	1115	5	1727	4	643	5	447	3	469	1	1144
chr8	120115819	C	3	1112	0	1731	1	645	1	450	3	468	12	1133
chr8	120115820	C	1	1114	6	1725	2	644	0	451	0	471	1	1144
chr8	120115821	C	5	1109	2	1728	3	643	14	436	7	464	18	1125
chr8	120115823	G	21	10129	51	16190	13	3867	8	2421	5	2552	13	3928
chr8	120115824	G	16	10133	65	16172	2	3874	1	2429	2	2552	6	3933
chr8	120115825	C	0	1104	0	1705	0	643	0	451	0	471	0	1143
chr8	120115829	G	0	10132	2	16214	1	3866	0	2425	0	2551	0	3937
chr8	120115831	G	1	10021	7	16190	1	3726	1	2323	0	2505	1	3883
chr8	120115832	G	1	10022	2	16195	0	3727	0	2323	0	2504	0	3883
chr8	120115834	C	0	1004	0	1504	0	606	0	412	0	423	0	1041
chr8	120115837	C	0	771	0	1065	0	509	0	295	0	322	0	798
chr8	120115839	C	0	608	0	771	0	408	0	232	0	266	0	577
chr8	120115843	C	0	606	0	765	0	407	0	231	0	262	0	573
chr8	120115844	C	1	603	0	762	0	407	0	231	0	262	0	573
chr8	120115846	G	0	9926	1	16035	0	3698	0	2300	1	2479	0	3857
chr9	100498295	G	2	8995	2	13921	1	2705	0	3430	0	3332	1	2080

3	1135	17	1747	5	668	6	466	10	487	1	1169
23	10207	69	16298	5	3911	9	2461	6	2579	13	3967
22	10196	31	16296	7	3901	2	2449	4	2577	4	3963
801	1123	1168	1739	473	666	320	462	363	484	776	1164
7821	10187	12597	16271	2858	3901	1826	2445	1951	2575	2784	3958
25	10198	31	16295	9	3903	1	2448	12	2577	9	3962
4	929	6	1440	2	538	0	373	1	403	14	947
8	1029	39	1607	4	602	1	414	2	451	1	1053
45	1119	60	1737	26	654	33	456	31	478	34	1151
41	1120	45	1737	18	653	12	456	11	478	42	1149
31	1119	96	1732	20	651	19	455	11	474	30	1151
76	1118	40	1732	24	649	33	454	31	474	72	1152
62	1117	67	1733	9	649	10	455	13	474	98	1148
100	1112	185	1724	54	646	48	452	54	473	77	1150
20	1114	19	1732	9	650	8	454	8	472	46	1147
0	1118	4	1733	5	650	0	455	3	473	2	1151
894	1108	1221	1707	531	644	329	452	389	463	881	1143
7022	10154	10436	16239	2679	3894	1595	2440	1695	2568	2479	3947
964	1115	1339	1728	554	647	364	453	420	470	939	1147
7340	8756	11486	13677	2805	3338	1705	2077	1877	2206	2717	3343
17	10172	70	16265	14	3893	3	2440	3	2569	5	3951
49	10172	33	16263	44	3891	5	2440	16	2569	29	3952
431	10165	507	16253	138	3889	106	2440	98	2568	128	3950
129	10159	194	16257	59	3890	48	2438	53	2569	96	3950
2	1118	0	1732	0	648	0	454	0	473	17	1148
108	1115	156	1723	83	645	48	453	62	471	191	1140
26	10159	17	16244	12	3885	11	2432	9	2561	4	3948
27	10159	18	16253	7	3882	6	2436	13	2565	12	3948
5	1118	1	1732	4	647	2	453	0	472	1	1146
3	1118	5	1732	4	647	5	452	3	472	1	1145
3	1115	0	1731	1	646	1	451	3	471	12	1145
1	1115	6	1731	2	646	0	451	0	471	1	1145
5	1114	2	1730	3	646	14	450	7	471	18	1143
21	10150	51	16241	13	3880	8	2429	5	2557	13	3941
16	10149	65	16237	2	3876	1	2430	2	2554	6	3939
0	1104	0	1705	0	643	0	451	0	471	0	1143
0	10132	2	16216	1	3867	0	2425	0	2551	0	3937
1	10022	7	16197	1	3727	1	2324	0	2505	1	3884
1	10023	2	16197	0	3727	0	2323	0	2504	0	3883
0	1004	0	1504	0	606	0	412	0	423	0	1041
0	771	0	1065	0	509	0	295	0	322	0	798
0	608	0	771	0	408	0	232	0	266	0	577
0	606	0	765	0	407	0	231	0	262	0	573
1	604	0	762	0	407	0	231	0	262	0	573
0	9926	1	16036	0	3698	0	2300	1	2480	0	3857
2	8997	2	13923	1	2706	0	3430	0	3332	1	2081

chr9	100498302	G	0	9246	0	14277	0	2768	0	3508	0	3389	1	2131
chr9	100498306	G	2	9283	2	14337	1	2773	0	3517	0	3392	0	2135
chr9	100498307	G	3	9286	7	14337	0	2775	1	3516	1	3392	1	2136
chr9	100498311	G	1	9340	1	14407	1	2780	0	3528	0	3400	0	2138
chr9	100498324	G	16	9378	12	14453	13	2776	3	3531	3	3399	1	2138
chr9	100498333	G	28	9383	34	14450	8	2787	26	3509	24	3395	11	2134
chr9	100498334	G	33	9382	38	14450	6	2788	17	3518	20	3397	16	2129
chr9	100498338	G	99	9303	154	14317	30	2760	24	3504	27	3387	19	2123
chr9	100498340	G	42	9377	25	14469	6	2788	5	3530	30	3391	11	2134
chr9	100498341	G	17	9402	31	14464	6	2789	10	3525	3	3417	6	2139
chr9	100498351	G	23	9410	60	14449	13	2782	3	3535	9	3414	2	2145
chr9	100498353	G	53	9374	74	14428	25	2769	5	3533	26	3397	9	2136
chr9	100498354	G	38	9395	66	14439	7	2791	5	3533	6	3415	6	2140
chr9	100498355	G	13	9419	29	14479	5	2793	4	3535	10	3413	1	2146
chr9	100498357	G	209	9223	148	14366	35	2765	58	3482	59	3365	12	2135
chr9	100498358	G	48	9389	26	14487	7	2792	6	3536	8	3417	3	2145
chr9	100498367	G	4697	4796	6030	8541	1449	1381	1514	2058	1758	1690	848	1317
chr9	100498368	G	37	9463	29	14553	8	2822	25	3550	10	3444	7	2161
chr9	100498370	G	115	9402	372	14234	30	2806	46	3531	38	3417	15	2152
chr9	100498375	C	2	4984	2	6118	1	5524	0	3046	1	6841	1	3539
chr9	100498377	G	11	9531	61	14566	10	2835	2	3580	12	3451	9	2162
chr9	100498379	C	1	5076	3	6227	0	5621	0	3100	0	6985	0	3595
chr9	100498381	G	201	9326	135	14455	30	2815	92	3483	35	3425	22	2152
chr9	100498382	G	12	9537	24	14601	6	2852	7	3582	6	3472	1	2187
chr9	100498383	G	18	9535	29	14600	2	2858	4	3586	5	3474	1	2188
chr9	100498385	G	34	9518	91	14536	13	2842	2	3592	10	3464	3	2176
chr9	100498387	C	0	5181	1	6395	0	5738	0	3157	1	7135	0	3639
chr9	100498389	C	0	5181	2	6386	0	5731	0	3158	0	7130	0	3630
chr9	100498390	C	0	5184	1	6399	0	5742	0	3160	1	7137	0	3639
chr9	100498392	C	1	5185	0	6399	0	5740	0	3159	1	7135	0	3641
chr9	100498394	G	23	9547	12	14634	6	2860	23	3578	20	3469	6	2187
chr9	100498396	G	7	9567	24	14621	13	2855	19	3583	2	3488	4	2190
chr9	100498398	C	1	5186	0	6402	0	5745	0	3160	1	7143	2	3640
chr9	100498401	G	27	9552	50	14598	9	2885	23	3609	5	3508	1	2219
chr9	100498403	G	83	9493	50	14596	26	2870	8	3624	31	3479	13	2203
chr9	100498406	G	22	9564	24	14633	9	2889	12	3622	9	3511	6	2217
chr9	100498407	C	9	5190	7	6409	9	5743	4	3159	3	7155	9	3651
chr9	100498408	C	16	5189	4	6418	3	5753	17	3150	31	7132	4	3660
chr9	100498411	C	14	5191	10	6411	6	5749	16	3154	14	7152	4	3659
chr9	100498414	C	17	5186	4	6414	18	5739	5	3165	19	7144	7	3649
chr9	100498415	C	3812	1390	4235	2187	4415	1343	2115	1054	5550	1616	2615	1043
chr9	100498416	G	6899	2695	9028	5632	2132	779	2606	1040	2663	874	1446	788
chr9	100498421	C	4132	1079	4797	1629	4633	1126	2135	1038	5739	1434	2697	974
chr9	100498422	G	7258	2331	10082	4584	2305	606	2673	971	2824	715	1599	633
chr9	100498423	C	9	5201	8	6420	19	5745	6	3168	12	7165	9	3656
chr9	100498426	G	30	9486	90	14434	18	2905	5	3654	9	3541	14	2224

0	9246	0	14277	0	2768	0	3508	0	3389	1	2132
2	9285	2	14339	1	2774	0	3517	0	3392	0	2135
3	9289	7	14344	0	2775	1	3517	1	3393	1	2137
1	9341	1	14408	1	2781	0	3528	0	3400	0	2138
16	9394	12	14465	13	2789	3	3534	3	3402	1	2139
28	9411	34	14484	8	2795	26	3535	24	3419	11	2145
33	9415	38	14488	6	2794	17	3535	20	3417	16	2145
99	9402	154	14471	30	2790	24	3528	27	3414	19	2142
42	9419	25	14494	6	2794	5	3535	30	3421	11	2145
17	9419	31	14495	6	2795	10	3535	3	3420	6	2145
23	9433	60	14509	13	2795	3	3538	9	3423	2	2147
53	9427	74	14502	25	2794	5	3538	26	3423	9	2145
38	9433	66	14505	7	2798	5	3538	6	3421	6	2146
13	9432	29	14508	5	2798	4	3539	10	3423	1	2147
209	9432	148	14514	35	2800	58	3540	59	3424	12	2147
48	9437	26	14513	7	2799	6	3542	8	3425	3	2148
4697	9493	6030	14571	1449	2830	1514	3572	1758	3448	848	2165
37	9500	29	14582	8	2830	25	3575	10	3454	7	2168
115	9517	372	14606	30	2836	46	3577	38	3455	15	2167
2	4986	2	6120	1	5525	0	3046	1	6842	1	3540
11	9542	61	14627	10	2845	2	3582	12	3463	9	2171
1	5077	3	6230	0	5621	0	3100	0	6985	0	3595
201	9527	135	14590	30	2845	92	3575	35	3460	22	2174
12	9549	24	14625	6	2858	7	3589	6	3478	1	2188
18	9553	29	14629	2	2860	4	3590	5	3479	1	2189
34	9552	91	14627	13	2855	2	3594	10	3474	3	2179
0	5181	1	6396	0	5738	0	3157	1	7136	0	3639
0	5181	2	6388	0	5731	0	3158	0	7130	0	3630
0	5184	1	6400	0	5742	0	3160	1	7138	0	3639
1	5186	0	6399	0	5740	0	3159	1	7136	0	3641
23	9570	12	14646	6	2866	23	3601	20	3489	6	2193
7	9574	24	14645	13	2868	19	3602	2	3490	4	2194
1	5187	0	6402	0	5745	0	3160	1	7144	2	3642
27	9579	50	14648	9	2894	23	3632	5	3513	1	2220
83	9576	50	14646	26	2896	8	3632	31	3510	13	2216
22	9586	24	14657	9	2898	12	3634	9	3520	6	2223
9	5199	7	6416	9	5752	4	3163	3	7158	9	3660
16	5205	4	6422	3	5756	17	3167	31	7163	4	3664
14	5205	10	6421	6	5755	16	3170	14	7166	4	3663
17	5203	4	6418	18	5757	5	3170	19	7163	7	3656
3812	5202	4235	6422	4415	5758	2115	3169	5550	7166	2615	3658
6899	9594	9028	14660	2132	2911	2606	3646	2663	3537	1446	2234
4132	5211	4797	6426	4633	5759	2135	3173	5739	7173	2697	3671
7258	9589	10082	14666	2305	2911	2673	3644	2824	3539	1599	2232
9	5210	8	6428	19	5764	6	3174	12	7177	9	3665
30	9516	90	14524	18	2923	5	3659	9	3550	14	2238

chr9	100498427	G	26	9579	39	14634	8	2941	12	3682	9	3579	4	2256
chr9	100498428	G	48	9557	27	14646	8	2942	3	3693	10	3579	4	2254
chr9	100498430	G	98	9507	119	14550	25	2925	41	3655	32	3557	26	2233
chr9	100498431	G	28	9575	27	14646	7	2942	8	3686	4	3585	6	2253
chr9	100498433	C	15	5206	4	6433	19	5752	22	3163	20	7170	40	3638
chr9	100498437	G	20	9584	23	14646	2	2947	6	3690	6	3582	5	2255
chr9	100498439	G	88	9517	105	14568	15	2935	24	3672	21	3568	9	2251
chr9	100498441	C	8	5212	4	6435	5	5768	1	3185	20	7170	5	3673
chr9	100498442	C	11	5211	4	6438	10	5763	12	3175	49	7143	31	3650
chr9	100498444	C	23	5199	7	6434	21	5753	2	3187	18	7175	8	3674
chr9	100498446	G	63	9535	36	14626	15	2930	11	3682	29	3559	8	2250
chr9	100498447	G	15	9587	95	14576	4	2944	0	3695	5	3583	2	2257
chr9	100498450	C	2976	2249	3235	3214	3260	2522	1777	1413	4190	3006	2277	1410
chr9	100498451	G	5907	3695	7113	7554	1818	1127	2036	1658	2294	1292	1177	1079
chr9	100498453	G	10	9588	14	14654	10	2934	4	3688	10	3572	7	2250
chr9	100498455	G	42	9559	21	14643	3	2943	19	3674	6	3578	7	2248
chr9	100498457	G	53	9543	38	14622	12	2911	4	3675	5	3570	10	2233
chr9	100498458	C	13	5210	22	6429	12	5772	7	3182	17	7183	10	3676
chr9	100498460	G	237	9361	283	14374	65	2859	83	3595	61	3515	33	2210
chr9	100498462	G	10	9587	59	14603	5	2919	3	3675	5	3569	8	2236
chr9	100498464	C	15	5207	38	6408	86	5699	56	3131	69	7130	4	3687
chr9	100498466	C	3	5213	9	6441	4	5776	7	3181	11	7180	7	3678
chr9	100498467	C	24	5200	33	6415	33	5753	5	3185	15	7186	18	3677
chr9	100498469	G	152	9435	328	14308	54	2854	79	3591	58	3511	36	2200
chr9	100498470	C	79	5148	9	6445	67	5720	83	3107	59	7142	45	3652
chr9	100498472	G	23	9569	28	14629	10	2911	3	3672	12	3562	9	2233
chr9	100498473	G	14	9577	37	14618	11	2908	2	3672	4	3569	7	2236
chr9	100498474	C	18	5197	19	6425	39	5745	4	3182	33	7154	7	3726
chr9	100498477	G	52	9529	17	14610	6	2899	29	3640	11	3558	6	2229
chr9	100498478	C	14	5216	10	6441	19	5777	7	3186	19	7178	13	3721
chr9	100498479	C	6	5223	17	6439	7	5793	1	3193	17	7187	5	3733
chr9	100498482	G	51	9498	168	14405	22	2870	21	3629	28	3528	16	2206
chr9	100498483	G	8	9584	24	14621	2	2909	5	3668	4	3566	4	2233
chr9	100498485	G	28	9560	26	14623	17	2894	5	3667	14	3558	5	2233
chr9	100498486	G	22	9568	16	14634	7	2902	2	3671	4	3566	4	2234
chr9	100498487	G	30	9558	29	14621	13	2897	12	3661	15	3555	7	2231
chr9	100498490	C	4148	1086	4619	1842	4489	1315	2132	1064	5532	1682	2680	1072
chr9	100498491	G	7496	2095	9992	4658	2320	591	2816	855	2895	676	1608	628
chr9	100498493	C	25	5209	9	6450	20	5782	2	3194	28	7190	10	3739
chr9	100498496	G	65	9507	12	14610	24	2879	16	3650	14	3549	18	2215
chr9	100498497	C	29	5203	73	6385	42	5760	7	3186	28	7183	36	3721
chr9	100498499	G	73	9517	81	14568	17	2889	6	3666	26	3544	9	2225
chr9	100498501	C	10	5223	6	6455	8	5796	5	3191	11	7207	8	3749
chr9	100498502	C	26	5211	19	6441	11	5794	0	3195	19	7200	22	3734
chr9	100498503	C	12	5227	8	6456	28	5777	10	3185	14	7206	12	3746
chr9	100498505	C	3503	1734	3565	2897	3771	2032	1967	1230	4649	2569	2273	1486

26	9605	39	14673	8	2949	12	3694	9	3588	4	2260
48	9605	27	14673	8	2950	3	3696	10	3589	4	2258
98	9605	119	14669	25	2950	41	3696	32	3589	26	2259
28	9603	27	14673	7	2949	8	3694	4	3589	6	2259
15	5221	4	6437	19	5771	22	3185	20	7190	40	3678
20	9604	23	14669	2	2949	6	3696	6	3588	5	2260
88	9605	105	14673	15	2950	24	3696	21	3589	9	2260
8	5220	4	6439	5	5773	1	3186	20	7190	5	3678
11	5222	4	6442	10	5773	12	3187	49	7192	31	3681
23	5222	7	6441	21	5774	2	3189	18	7193	8	3682
63	9598	36	14662	15	2945	11	3693	29	3588	8	2258
15	9602	95	14671	4	2948	0	3695	5	3588	2	2259
2976	5225	3235	6449	3260	5782	1777	3190	4190	7196	2277	3687
5907	9602	7113	14667	1818	2945	2036	3694	2294	3586	1177	2256
10	9598	14	14668	10	2944	4	3692	10	3582	7	2257
42	9601	21	14664	3	2946	19	3693	6	3584	7	2255
53	9596	38	14660	12	2923	4	3679	5	3575	10	2243
13	5223	22	6451	12	5784	7	3189	17	7200	10	3686
237	9598	283	14657	65	2924	83	3678	61	3576	33	2243
10	9597	59	14662	5	2924	3	3678	5	3574	8	2244
15	5222	38	6446	86	5785	56	3187	69	7199	4	3691
3	5216	9	6450	4	5780	7	3188	11	7191	7	3685
24	5224	33	6448	33	5786	5	3190	15	7201	18	3695
152	9587	328	14636	54	2908	79	3670	58	3569	36	2236
79	5227	9	6454	67	5787	83	3190	59	7201	45	3697
23	9592	28	14657	10	2921	3	3675	12	3574	9	2242
14	9591	37	14655	11	2919	2	3674	4	3573	7	2243
18	5215	19	6444	39	5784	4	3186	33	7187	7	3733
52	9581	17	14627	6	2905	29	3669	11	3569	6	2235
14	5230	10	6451	19	5796	7	3193	19	7197	13	3734
6	5229	17	6456	7	5800	1	3194	17	7204	5	3738
51	9549	168	14573	22	2892	21	3650	28	3556	16	2222
8	9592	24	14645	2	2911	5	3673	4	3570	4	2237
28	9588	26	14649	17	2911	5	3672	14	3572	5	2238
22	9590	16	14650	7	2909	2	3673	4	3570	4	2238
30	9588	29	14650	13	2910	12	3673	15	3570	7	2238
4148	5234	4619	6461	4489	5804	2132	3196	5532	7214	2680	3752
7496	9591	9992	14650	2320	2911	2816	3671	2895	3571	1608	2236
25	5234	9	6459	20	5802	2	3196	28	7218	10	3749
65	9572	12	14622	24	2903	16	3666	14	3563	18	2233
29	5232	73	6458	42	5802	7	3193	28	7211	36	3757
73	9590	81	14649	17	2906	6	3672	26	3570	9	2234
10	5233	6	6461	8	5804	5	3196	11	7218	8	3757
26	5237	19	6460	11	5805	0	3195	19	7219	22	3756
12	5239	8	6464	28	5805	10	3195	14	7220	12	3758
3503	5237	3565	6462	3771	5803	1967	3197	4649	7218	2273	3759

chr9	100498506	G	6859	2726	8848	5796	2067	839	2492	1179	2567	1002	1326	909
chr9	100498508	C	27	5212	36	6435	18	5790	9	3188	14	7208	5	3774
chr9	100498510	G	25	9552	91	14544	2	2903	25	3643	7	3558	2	2229
chr9	100498511	C	14	5184	77	6350	70	5686	8	3173	42	7125	11	3740
chr9	100498514	C	24	5217	52	6419	45	5764	17	3179	72	7152	4	3784
chr9	100498516	C	11	5229	24	6447	14	5796	8	3189	5	7219	2	3794
chr9	100498517	C	249	4993	61	6410	156	5654	45	3152	233	6990	127	3669
chr9	100498519	C	56	5185	5	6468	7	5803	7	3190	43	7183	2	3795
chr9	100498521	G	16	9548	18	14605	5	2892	3	3660	8	3550	8	2225
chr9	100498522	C	18	5226	40	6429	16	5792	9	3188	49	7176	9	3784
chr9	100498525	C	5	1106	0	1338	7	1309	7	731	3	1659	0	892
chr9	100498528	G	17	9548	48	14565	7	2883	7	3648	7	3539	4	2222
chr9	100498529	G	26	9524	22	14582	5	2866	6	3642	9	3525	5	2206
chr9	100498531	G	142	9418	170	14437	62	2809	75	3573	66	3469	33	2177
chr9	100498533	G	191	9360	186	14416	24	2846	58	3586	69	3461	35	2173
chr9	100498536	G	30	9524	18	14585	6	2865	4	3640	4	3525	4	2204
chr9	100498539	C	1	161	0	151	1	212	1	119	0	282	2	288
chr9	100498541	G	30	9514	8	14589	8	2863	1	3641	6	3520	13	2191
chr9	100498545	C	0	158	0	152	1	204	0	113	1	277	1	288
chr9	100498546	C	1	162	1	153	0	207	0	114	0	280	0	288
chr9	100498547	C	0	168	1	164	0	207	0	116	0	281	0	285
chr9	100498548	C	122	46	88	77	133	71	81	37	184	93	152	130
chr9	100498549	G	5927	3573	7642	6914	1995	876	2336	1301	2549	975	1372	830
chr9	100498551	G	3	9404	4	14468	0	2868	0	3636	1	3519	1	2201
chr9	100498553	G	2	9401	1	14465	0	2868	2	3634	1	3518	1	2199
chr9	100498557	C	0	189	0	185	2	236	1	135	2	297	1	324
chr9	100498558	C	2	203	0	219	2	247	1	150	2	319	1	328
chr9	100498560	G	6	9357	1	14400	1	2861	0	3628	3	3503	0	2192
chr9	100498561	C	1	311	0	391	0	398	2	237	2	499	0	389
chr9	100498562	C	1	325	0	415	0	430	0	256	0	524	0	397
chr9	100498563	C	0	336	0	429	1	442	1	271	0	539	1	400
chr9	100498565	G	3	8941	0	13821	0	2807	0	3540	0	3465	0	2153
chr9	100498566	G	3	8901	3	13772	0	2799	0	3536	1	3460	0	2152
chr9	100498569	G	4	8744	0	13488	0	2784	0	3498	1	3442	0	2146
chr9	100498572	C	5	1575	12	1868	7	1727	0	930	12	2096	9	1051
chr9	100498574	G	5	6745	4	10456	2	2080	1	2687	1	2667	1	1646
chr9	100498575	C	15	5246	11	6470	5	5813	6	3204	20	7224	18	3828
chr9	100498576	C	4126	1133	4501	1978	4624	1192	2361	852	5793	1452	2784	1061
chr9	100498581	C	8	5246	5	6477	5	5810	2	3198	26	7212	3	3844
chr9	100498583	C	38	5220	9	6473	30	5785	28	3178	46	7193	18	3830
chr9	100498588	C	11	5247	18	6463	9	5804	5	3203	14	7219	3	3830
chr9	100498589	C	3689	1568	4120	2360	4000	1811	1808	1399	4900	2331	2524	1310
chr9	100498594	C	15	5237	5	6474	26	5785	2	3204	16	7206	15	3815
chr9	100498602	C	12	5236	10	6465	12	5793	6	3197	13	7203	8	3822
chr9	100498606	C	23	5223	5	6469	6	5798	2	3197	14	7203	8	3821
chr9	100498610	C	46	5193	4	6469	25	5770	4	3194	27	7180	17	3784

6859	9585	8848	14644	2067	2906	2492	3671	2567	3569	1326	2235
27	5239	36	6471	18	5808	9	3197	14	7222	5	3779
25	9577	91	14635	2	2905	25	3668	7	3565	2	2231
14	5198	77	6427	70	5756	8	3181	42	7167	11	3751
24	5241	52	6471	45	5809	17	3196	72	7224	4	3788
11	5240	24	6471	14	5810	8	3197	5	7224	2	3796
249	5242	61	6471	156	5810	45	3197	233	7223	127	3796
56	5241	5	6473	7	5810	7	3197	43	7226	2	3797
16	9564	18	14623	5	2897	3	3663	8	3558	8	2233
18	5244	40	6469	16	5808	9	3197	49	7225	9	3793
5	1111	0	1338	7	1316	7	738	3	1662	0	892
17	9565	48	14613	7	2890	7	3655	7	3546	4	2226
26	9550	22	14604	5	2871	6	3648	9	3534	5	2211
142	9560	170	14607	62	2871	75	3648	66	3535	33	2210
191	9551	186	14602	24	2870	58	3644	69	3530	35	2208
30	9554	18	14603	6	2871	4	3644	4	3529	4	2208
1	162	0	151	1	213	1	120	0	282	2	290
30	9544	8	14597	8	2871	1	3642	6	3526	13	2204
0	158	0	152	1	205	0	113	1	278	1	289
1	163	1	154	0	207	0	114	0	280	0	288
0	168	1	165	0	207	0	116	0	281	0	285
122	168	88	165	133	204	81	118	184	277	152	282
5927	9500	7642	14556	1995	2871	2336	3637	2549	3524	1372	2202
3	9407	4	14472	0	2868	0	3636	1	3520	1	2202
2	9403	1	14466	0	2868	2	3636	1	3519	1	2200
0	189	0	185	2	238	1	136	2	299	1	325
2	205	0	219	2	249	1	151	2	321	1	329
6	9363	1	14401	1	2862	0	3628	3	3506	0	2192
1	312	0	391	0	398	2	239	2	501	0	389
1	326	0	415	0	430	0	256	0	524	0	397
0	336	0	429	1	443	1	272	0	539	1	401
3	8944	0	13821	0	2807	0	3540	0	3465	0	2153
3	8904	3	13775	0	2799	0	3536	1	3461	0	2152
4	8748	0	13488	0	2784	0	3498	1	3443	0	2146
5	1580	12	1880	7	1734	0	930	12	2108	9	1060
5	6750	4	10460	2	2082	1	2688	1	2668	1	1647
15	5261	11	6481	5	5818	6	3210	20	7244	18	3846
4126	5259	4501	6479	4624	5816	2361	3213	5793	7245	2784	3845
8	5254	5	6482	5	5815	2	3200	26	7238	3	3847
38	5258	9	6482	30	5815	28	3206	46	7239	18	3848
11	5258	18	6481	9	5813	5	3208	14	7233	3	3833
3689	5257	4120	6480	4000	5811	1808	3207	4900	7231	2524	3834
15	5252	5	6479	26	5811	2	3206	16	7222	15	3830
12	5248	10	6475	12	5805	6	3203	13	7216	8	3830
23	5246	5	6474	6	5804	2	3199	14	7217	8	3829
46	5239	4	6473	25	5795	4	3198	27	7207	17	3801

chr9	100498617	C	10	5225	5	6467	11	5783	36	3158	10	7189	21	3779
chr9	100498624	C	10	5221	9	6457	11	5783	3	3186	18	7175	8	3786
chr9	100498626	C	6	5225	6	6458	7	5784	4	3186	12	7179	5	3790
chr9	100498638	C	7	5219	5	6448	17	5765	3	3181	12	7162	3	3782
chr9	100498639	C	28	5199	49	6409	37	5745	4	3181	71	7106	8	3777
chr9	100498649	C	9	5038	8	6219	7	5572	6	3053	13	6898	1	3685
chr9	100498650	C	8	5205	9	6434	15	5747	1	3176	19	7138	13	3770
chr9	100498654	C	32	5184	5	6434	11	5749	16	3159	10	7144	14	3764
chr9	100498655	C	29	5187	5	6438	10	5751	5	3172	20	7135	26	3752
chr9	100498660	c	11	5203	10	6433	23	5739	6	3166	11	7143	8	3764
chr9	100498672	c	29	5166	6	6421	45	5702	5	3160	25	7106	14	3753
chr9	100498674	c	22	5174	4	6429	15	5734	4	3163	18	7121	3	3760
chr9	100498676	c	4252	926	5260	1147	4750	987	2408	752	5698	1419	3207	551
chr9	100498678	c	7	5184	12	6418	14	5730	22	3142	13	7112	8	3751
chr9	100498679	c	35	5153	55	6374	13	5730	5	3160	29	7095	6	3753
chr9	100498686	c	19	5166	13	6410	15	5724	3	3158	11	7107	10	3743
chr9	100498687	c	12	5172	4	6419	1	5738	3	3158	7	7113	46	3708
chr9	100498688	c	51	5134	66	6358	82	5657	0	3161	87	7034	55	3703
chr9	100498691	c	4603	580	5550	873	5148	590	2738	421	6420	698	3382	374
chr9	100498693	c	15	5154	15	6371	8	5710	4	3147	18	7087	9	3741
chr9	100498694	c	10	5174	11	6410	7	5724	4	3156	12	7105	8	3750
chr9	100498697	c	0	5177	1	6418	0	5732	0	3157	1	7112	1	3757
chr9	100498703	c	0	5170	1	6403	1	5726	0	3151	3	7102	0	3751
chr9	100498709	c	3	5131	9	6333	11	5681	5	3128	8	7056	5	3738
chr9	100498718	c	0	4162	3	5192	1	4653	0	2583	2	5800	1	3215
chr9	100498720	c	0	3992	1	4997	1	4471	0	2491	0	5591	2	3107
chrX	7476281	G	2	4352	1	2694	0	1654	2	2938	1	3358	1	2269
chrX	7476282	G	0	4352	0	2698	1	1653	3	2937	3	3357	1	2271
chrX	7476287	G	1	4356	1	2697	1	1654	1	2947	2	3359	2	2272
chrX	7476289	G	0	4356	1	2698	0	1655	0	2948	0	3362	0	2275
chrX	7476302	G	4	4344	6	2702	1	1653	3	2943	6	3350	0	2275
chrX	7476305	G	8	4358	3	2711	1	1657	6	2946	7	3358	2	2276
chrX	7476306	G	8	4357	3	2710	6	1650	16	2939	6	3360	3	2272
chrX	7476307	G	5	4360	7	2706	3	1654	7	2947	7	3360	7	2272
chrX	7476313	G	4	4362	1	2713	1	1657	0	2956	2	3368	2	2277
chrX	7476314	G	5	4357	5	2708	1	1657	1	2952	2	3366	2	2277
chrX	7476321	G	8	4359	8	2704	5	1653	3	2953	9	3362	4	2274
chrX	7476329	G	4	4364	3	2712	3	1655	5	2954	7	3365	5	2276
chrX	7476330	G	11	4359	16	2700	5	1653	8	2948	2	3372	4	2273
chrX	7476337	G	9	4359	5	2710	1	1657	3	2956	4	3371	4	2279
chrX	7476348	G	4	4365	5	2713	4	1655	3	2958	15	3359	6	2276
chrX	7476349	G	8	4364	3	2715	4	1655	7	2952	8	3366	6	2276
chrX	7476351	G	6	4364	2	2716	0	1658	1	2960	2	3374	4	2277
chrX	7476361	G	4	4368	1	2718	0	1659	3	2957	4	3375	2	2282
chrX	7476362	G	5	4365	3	2715	4	1655	1	2960	4	3377	8	2277
chrX	7476365	G	9	4362	3	2715	3	1656	3	2958	6	3375	3	2282

10	5235	5	6472	11	5794	36	3194	10	7199	21	3800
10	5231	9	6466	11	5794	3	3189	18	7193	8	3794
6	5231	6	6464	7	5791	4	3190	12	7191	5	3795
7	5226	5	6453	17	5782	3	3184	12	7174	3	3785
28	5227	49	6458	37	5782	4	3185	71	7177	8	3785
9	5047	8	6227	7	5579	6	3059	13	6911	1	3686
8	5213	9	6443	15	5762	1	3177	19	7157	13	3783
32	5216	5	6439	11	5760	16	3175	10	7154	14	3778
29	5216	5	6443	10	5761	5	3177	20	7155	26	3778
11	5214	10	6443	23	5762	6	3172	11	7154	8	3772
29	5195	6	6427	45	5747	5	3165	25	7131	14	3767
22	5196	4	6433	15	5749	4	3167	18	7139	3	3763
4252	5178	5260	6407	4750	5737	2408	3160	5698	7117	3207	3758
7	5191	12	6430	14	5744	22	3164	13	7125	8	3759
35	5188	55	6429	13	5743	5	3165	29	7124	6	3759
19	5185	13	6423	15	5739	3	3161	11	7118	10	3753
12	5184	4	6423	1	5739	3	3161	7	7120	46	3754
51	5185	66	6424	82	5739	0	3161	87	7121	55	3758
4603	5183	5550	6423	5148	5738	2738	3159	6420	7118	3382	3756
15	5169	15	6386	8	5718	4	3151	18	7105	9	3750
10	5184	11	6421	7	5731	4	3160	12	7117	8	3758
0	5177	1	6419	0	5732	0	3157	1	7113	1	3758
0	5170	1	6404	1	5727	0	3151	3	7105	0	3751
3	5134	9	6342	11	5692	5	3133	8	7064	5	3743
0	4162	3	5195	1	4654	0	2583	2	5802	1	3216
0	3992	1	4998	1	4472	0	2491	0	5591	2	3109
2	4354	1	2695	0	1654	2	2940	1	3359	1	2270
0	4352	0	2698	1	1654	3	2940	3	3360	1	2272
1	4357	1	2698	1	1655	1	2948	2	3361	2	2274
0	4356	1	2699	0	1655	0	2948	0	3362	0	2275
4	4348	6	2708	1	1654	3	2946	6	3356	0	2275
8	4366	3	2714	1	1658	6	2952	7	3365	2	2278
8	4365	3	2713	6	1656	16	2955	6	3366	3	2275
5	4365	7	2713	3	1657	7	2954	7	3367	7	2279
4	4366	1	2714	1	1658	0	2956	2	3370	2	2279
5	4362	5	2713	1	1658	1	2953	2	3368	2	2279
8	4367	8	2712	5	1658	3	2956	9	3371	4	2278
4	4368	3	2715	3	1658	5	2959	7	3372	5	2281
11	4370	16	2716	5	1658	8	2956	2	3374	4	2277
9	4368	5	2715	1	1658	3	2959	4	3375	4	2283
4	4369	5	2718	4	1659	3	2961	15	3374	6	2282
8	4372	3	2718	4	1659	7	2959	8	3374	6	2282
6	4370	2	2718	0	1658	1	2961	2	3376	4	2281
4	4372	1	2719	0	1659	3	2960	4	3379	2	2284
5	4370	3	2718	4	1659	1	2961	4	3381	8	2285
9	4371	3	2718	3	1659	3	2961	6	3381	3	2285

chrX	7476371	G	7	4365	13	2704	8	1651	5	2956	7	3373	3	2281
chrX	7476374	G	6	4364	5	2714	2	1657	3	2959	5	3375	5	2278
chrX	7476382	G	4	4366	5	2714	0	1657	3	2959	2	3376	2	2285
chrX	7476383	G	6	4365	4	2714	3	1654	6	2955	10	3371	3	2284
chrX	7476387	G	4	4370	7	2711	0	1659	2	2961	9	3373	5	2284
chrX	7476388	G	4	4369	1	2718	1	1659	4	2959	3	3382	3	2284
chrX	7476391	G	1	4373	2	2713	1	1660	2	2960	4	3381	3	2287
chrX	7476393	G	9	4364	6	2712	1	1659	1	2962	3	3383	0	2290
chrX	7476394	G	8	4366	6	2714	6	1654	8	2953	2	3382	5	2286
chrX	7476402	G	1	4370	4	2718	2	1659	1	2962	2	3382	2	2291
chrX	7476403	G	22	4354	3	2720	2	1657	3	2958	18	3368	5	2290
chrX	7476407	G	8	4368	4	2720	2	1659	3	2960	3	3383	6	2286
chrX	7476415	G	7	4369	7	2718	10	1654	5	2958	3	3388	2	2294
chrX	7476417	G	8	4364	9	2712	4	1659	3	2959	7	3381	2	2290
chrX	7476419	G	6	4369	5	2720	1	1661	7	2955	6	3382	36	2260
chrX	7476421	G	4	4369	6	2721	11	1649	3	2959	6	3381	5	2290
chrX	7476424	G	0	195	0	129	0	113	1	145	2	169	0	123
chrX	7476427	G	1	124	0	84	0	93	0	98	0	131	1	100
chrX	7476428	G	0	122	0	84	1	92	0	96	0	131	0	102
chrX	7476435	G	0	112	0	73	0	93	1	84	0	136	1	106
chrX	7476439	G	1	108	0	73	0	94	0	86	0	136	0	107
chrX	7476440	C	4	25192	6	26970	9	22703	2	12338	10	51533	8	16522
chrX	7476442	G	0	108	0	74	0	93	0	85	0	137	0	108
chrX	7476444	G	0	109	0	72	0	95	0	85	2	137	0	106
chrX	7476447	G	0	90	0	65	0	71	0	61	0	118	0	89
chrX	7476450	C	1	25681	4	27470	0	23176	1	12572	3	52554	1	16739
chrX	7476451	C	3	25687	4	27481	2	23186	2	12576	2	52599	1	16740
chrX	7476456	G	0	80	0	56	0	69	0	55	0	127	0	92
chrX	7476459	C	1	25907	4	27700	1	23473	0	12738	5	53013	1	16981
chrX	7476460	C	1	25914	7	27704	5	23481	0	12745	6	53021	2	16990
chrX	7476461	C	8	25903	1	27712	2	23477	1	12734	8	53015	2	16983
chrX	7476464	G	0	82	0	61	0	70	0	55	0	124	0	91
chrX	7476465	G	0	82	1	61	0	69	0	57	0	124	0	92
chrX	7476467	C	51	26040	82	27780	75	23491	19	12768	128	53055	19	17053
chrX	7476470	C	89	26005	67	27794	46	23514	111	12675	274	52886	35	17024
chrX	7476471	G	0	83	0	61	0	69	0	57	0	127	0	91
chrX	7476474	C	38	26092	28	27878	47	23554	14	12795	115	53117	38	17058
chrX	7476476	G	1	83	0	61	0	65	0	54	0	125	1	86
chrX	7476477	C	60	25836	71	27570	38	23352	111	12604	207	52591	47	16902
chrX	7476478	G	0	82	1	60	1	66	0	52	0	124	0	84
chrX	7476479	C	57	26074	64	27847	55	23566	34	12784	152	53106	27	17100
chrX	7476480	C	41	26116	40	27895	66	23577	15	12819	98	53225	44	17098
chrX	7476482	C	24	26119	14	27907	16	23612	15	12812	46	53246	12	17123
chrX	7476484	G	0	83	0	58	0	67	1	51	0	126	0	86
chrX	7476486	G	0	84	1	57	0	64	0	51	0	120	0	82
chrX	7476487	C	45	26123	44	27905	94	23589	24	12815	80	53292	34	17141

7	4372	13	2717	8	1659	5	2961	7	3380	3	2284
6	4370	5	2719	2	1659	3	2962	5	3380	5	2283
4	4370	5	2719	0	1657	3	2962	2	3378	2	2287
6	4371	4	2718	3	1657	6	2961	10	3381	3	2287
4	4374	7	2718	0	1659	2	2963	9	3382	5	2289
4	4373	1	2719	1	1660	4	2963	3	3385	3	2287
1	4374	2	2715	1	1661	2	2962	4	3385	3	2290
9	4373	6	2718	1	1660	1	2963	3	3386	0	2290
8	4374	6	2720	6	1660	8	2961	2	3384	5	2291
1	4371	4	2722	2	1661	1	2963	2	3384	2	2293
22	4376	3	2723	2	1659	3	2961	18	3386	5	2295
8	4376	4	2724	2	1661	3	2963	3	3386	6	2292
7	4376	7	2725	10	1664	5	2963	3	3391	2	2296
8	4372	9	2721	4	1663	3	2962	7	3388	2	2292
6	4375	5	2725	1	1662	7	2962	6	3388	36	2296
4	4373	6	2727	11	1660	3	2962	6	3387	5	2295
0	195	0	129	0	113	1	146	2	171	0	123
1	125	0	84	0	93	0	98	0	131	1	101
0	122	0	84	1	93	0	96	0	131	0	102
0	112	0	73	0	93	1	85	0	136	1	107
1	109	0	73	0	94	0	86	0	136	0	107
4	25196	6	26976	9	22712	2	12340	10	51543	8	16530
0	108	0	74	0	93	0	85	0	137	0	108
0	109	0	72	0	95	0	85	2	139	0	106
0	90	0	65	0	71	0	61	0	118	0	89
1	25682	4	27474	0	23176	1	12573	3	52557	1	16740
3	25690	4	27485	2	23188	2	12578	2	52601	1	16741
0	80	0	56	0	69	0	55	0	127	0	92
1	25908	4	27704	1	23474	0	12738	5	53018	1	16982
1	25915	7	27711	5	23486	0	12745	6	53027	2	16992
8	25911	1	27713	2	23479	1	12735	8	53023	2	16985
0	82	0	61	0	70	0	55	0	124	0	91
0	82	1	62	0	69	0	57	0	124	0	92
51	26091	82	27862	75	23566	19	12787	128	53183	19	17072
89	26094	67	27861	46	23560	111	12786	274	53160	35	17059
0	83	0	61	0	69	0	57	0	127	0	91
38	26130	28	27906	47	23601	14	12809	115	53232	38	17096
1	84	0	61	0	65	0	54	0	125	1	87
60	25896	71	27641	38	23390	111	12715	207	52798	47	16949
0	82	1	61	1	67	0	52	0	124	0	84
57	26131	64	27911	55	23621	34	12818	152	53258	27	17127
41	26157	40	27935	66	23643	15	12834	98	53323	44	17142
24	26143	14	27921	16	23628	15	12827	46	53292	12	17135
0	83	0	58	0	67	1	52	0	126	0	86
0	84	1	58	0	64	0	51	0	120	0	82
45	26168	44	27949	94	23683	24	12839	80	53372	34	17175

chrX	7476490	G	1	83	0	56	0	61	1	50	0	117	0	82
chrX	7476492	G	1	82	0	56	0	61	0	50	0	120	0	83
chrX	7476493	G	0	83	0	57	0	61	0	50	0	119	0	83
chrX	7476494	G	0	81	0	57	0	60	0	50	1	114	0	82
chrX	7476495	C	67	26055	145	27746	64	23611	13	12833	154	53167	49	17132
chrX	7476496	G	0	65	0	54	0	40	0	41	1	101	0	71
chrX	7476500	C	29	26177	34	27958	21	23729	103	12771	260	53228	24	17205
chrX	7476501	G	0	78	0	61	0	76	0	51	0	135	0	97
chrX	7476502	G	0	79	0	61	0	76	0	50	0	137	0	97
chrX	7476504	G	0	79	0	61	1	75	0	48	1	137	1	99
chrX	7476505	C	122	26083	31	27944	59	23689	23	12854	169	53329	132	17091
chrX	7476506	G	0	68	0	49	0	61	0	37	0	118	1	84
chrX	7476507	G	0	78	0	62	0	77	1	47	1	140	0	102
chrX	7476508	G	0	78	0	60	1	76	0	49	0	140	0	103
chrX	7476510	G	0	77	0	57	0	64	0	46	0	122	0	96
chrX	7476511	G	0	64	0	52	0	47	0	35	1	115	0	86
chrX	7476512	G	0	63	0	53	0	44	0	37	0	110	1	84
chrX	7476513	G	0	64	0	53	0	49	0	36	0	117	0	88
chrX	7476514	G	0	65	0	53	0	49	0	36	0	117	0	89
chrX	7476516	C	17	26195	35	27961	55	23710	15	12874	134	53393	24	17217
chrX	7476517	G	0	65	0	54	0	49	0	33	0	110	0	88
chrX	7476518	G	0	68	0	55	0	50	0	37	0	113	1	87
chrX	7476519	G	0	68	0	55	0	50	0	38	0	111	1	87
chrX	7476520	G	0	67	0	55	0	50	0	37	1	112	0	87
chrX	7476521	G	0	67	0	55	0	50	0	38	0	113	0	86
chrX	7476522	G	0	72	0	57	0	58	0	37	0	129	0	91
chrX	7476524	G	0	72	0	59	0	59	0	37	0	130	0	90
chrX	7476526	G	0	75	0	62	0	63	0	40	0	136	1	92
chrX	7476528	C	36	26175	68	27927	35	23750	14	12876	97	53447	27	17219
chrX	7476530	C	35	26137	100	27845	61	23680	24	12856	199	53255	31	17189
chrX	7476531	G	1	80	2	63	0	66	0	48	1	133	0	89
chrX	7476532	C	54	26159	53	27945	65	23721	17	12880	146	53376	75	17168
chrX	7476533	C	64	26172	46	27970	31	23781	24	12880	125	53455	80	17195
chrX	7476534	C	42	26184	99	27888	67	23736	22	12880	174	53377	27	17246
chrX	7476535	G	0	81	1	69	0	67	0	51	0	135	0	90
chrX	7476536	C	85	25841	44	27704	102	23456	28	12743	161	52883	30	17064
chrX	7476537	G	0	81	1	69	0	72	0	51	0	138	0	89
chrX	7476538	C	44	26186	58	27957	37	23778	53	12853	175	53407	29	17241
chrX	7476539	C	37	26204	34	27983	33	23765	8	12894	55	53540	18	17263
chrX	7476541	C	65	26173	138	27877	33	23787	56	12857	123	53476	49	17234
chrX	7476542	C	45	26137	36	27928	82	23699	101	12787	112	53369	35	17210
chrX	7476543	G	0	88	1	75	0	77	0	59	1	146	0	98
chrX	7476545	C	53	26182	193	27824	47	23774	52	12860	129	53467	59	17229
chrX	7476547	G	1	429	0	313	0	168	3	267	0	354	0	241
chrX	7476548	C	103	26124	53	27948	84	23732	122	12779	210	53358	52	17221
chrX	7476549	G	14	4286	5	2711	4	1568	7	2876	8	3300	6	2243

1	84	0	56	0	61	1	51	0	117	0	82
1	83	0	56	0	61	0	50	0	120	0	83
0	83	0	57	0	61	0	50	0	119	0	83
0	81	0	57	0	60	0	50	1	115	0	82
67	26122	145	27891	64	23675	13	12846	154	53321	49	17181
0	65	0	54	0	40	0	41	1	102	0	71
29	26206	34	27992	21	23750	103	12874	260	53488	24	17229
0	78	0	61	0	76	0	51	0	135	0	97
0	79	0	61	0	76	0	50	0	137	0	97
0	79	0	61	1	76	0	48	1	138	1	100
122	26205	31	27975	59	23748	23	12877	169	53498	132	17223
0	68	0	49	0	61	0	37	0	118	1	85
0	78	0	62	0	77	1	48	1	141	0	102
0	78	0	60	1	77	0	49	0	140	0	103
0	77	0	57	0	64	0	46	0	122	0	96
0	64	0	52	0	47	0	35	1	116	0	86
0	63	0	53	0	44	0	37	0	110	1	85
0	64	0	53	0	49	0	36	0	117	0	88
0	65	0	53	0	49	0	36	0	117	0	89
17	26212	35	27996	55	23765	15	12889	134	53527	24	17241
0	65	0	54	0	49	0	33	0	110	0	88
0	68	0	55	0	50	0	37	0	113	1	88
0	68	0	55	0	50	0	38	0	111	1	88
0	67	0	55	0	50	0	37	1	113	0	87
0	67	0	55	0	50	0	38	0	113	0	86
0	72	0	57	0	58	0	37	0	129	0	91
0	72	0	59	0	59	0	37	0	130	0	90
0	75	0	62	0	63	0	40	0	136	1	93
36	26211	68	27995	35	23785	14	12890	97	53544	27	17246
35	26172	100	27945	61	23741	24	12880	199	53454	31	17220
1	81	2	65	0	66	0	48	1	134	0	89
54	26213	53	27998	65	23786	17	12897	146	53522	75	17243
64	26236	46	28016	31	23812	24	12904	125	53580	80	17275
42	26226	99	27987	67	23803	22	12902	174	53551	27	17273
0	81	1	70	0	67	0	51	0	135	0	90
85	25926	44	27748	102	23558	28	12771	161	53044	30	17094
0	81	1	70	0	72	0	51	0	138	0	89
44	26230	58	28015	37	23815	53	12906	175	53582	29	17270
37	26241	34	28017	33	23798	8	12902	55	53595	18	17281
65	26238	138	28015	33	23820	56	12913	123	53599	49	17283
45	26182	36	27964	82	23781	101	12888	112	53481	35	17245
0	88	1	76	0	77	0	59	1	147	0	98
53	26235	193	28017	47	23821	52	12912	129	53596	59	17288
1	430	0	313	0	168	3	270	0	354	0	241
103	26227	53	28001	84	23816	122	12901	210	53568	52	17273
14	4300	5	2716	4	1572	7	2883	8	3308	6	2249

chrX	7476550	C	39	26037	45	27763	42	23641	17	12799	99	53147	56	17107
chrX	7476551	C	34	26205	33	27981	65	23763	8	12902	105	53497	24	17266
chrX	7476553	G	3	4312	3	2719	1	1580	4	2891	6	3314	3	2256
chrX	7476554	G	8	4307	14	2707	6	1574	7	2884	5	3312	7	2251
chrX	7476555	C	58	26153	54	27923	71	23693	24	12877	109	53421	35	17239
chrX	7476556	C	65	26174	81	27936	31	23793	101	12815	268	53327	46	17243
chrX	7476557	G	0	4315	7	2716	3	1575	5	2889	3	3312	1	2254
chrX	7476558	G	4	4310	2	2720	8	1570	0	2893	12	3301	5	2248
chrX	7476560	C	61	26171	117	27886	40	23776	20	12881	151	53437	40	17247
chrX	7476562	C	22	26219	38	27983	58	23768	10	12905	96	53513	29	17263
chrX	7476564	G	5	4308	4	2719	4	1574	2	2889	8	3305	1	2254
chrX	7476565	G	8	4304	3	2720	2	1574	6	2886	4	3305	5	2250
chrX	7476566	C	57	25938	144	27619	64	23543	38	12738	249	52887	118	17018
chrX	7476567	G	4	4302	8	2710	2	1574	9	2876	6	3300	37	2213
chrX	7476568	C	78	26152	49	27962	41	23777	42	12866	152	53437	70	17208
chrX	7476569	C	25	26220	123	27899	25	23803	15	12902	105	53507	42	17247
chrX	7476572	G	4	4261	4	2648	1	1554	3	2855	2	3259	5	2207
chrX	7476573	G	7	4299	4	2714	4	1570	2	2887	4	3298	1	2248
chrX	7476575	G	2	4305	2	2717	2	1574	4	2885	6	3299	2	2246
chrX	7476576	G	9	4296	2	2715	2	1574	6	2881	9	3295	4	2242
chrX	7476578	G	5	4300	4	2714	5	1571	6	2882	1	3301	2	2245
chrX	7476581	C	132	26101	214	27801	129	23702	127	12789	254	53353	30	17256
chrX	7476582	G	10	4290	7	2708	1	1573	7	2879	8	3293	4	2242
chrX	7476583	C	69	26164	56	27951	48	23772	29	12880	260	53331	78	17211
chrX	7476585	C	90	26151	54	27968	59	23770	30	12885	129	53472	55	17232
chrX	7476586	G	5	4297	33	2685	6	1566	7	2877	0	3293	2	2238
chrX	7476587	C	65	25951	60	27733	48	23553	116	12681	178	52958	41	17079
chrX	7476588	G	3	4301	2	2716	4	1566	6	2879	6	3289	44	2195
chrX	7476591	C	50	26193	36	27980	32	23792	19	12891	93	53501	26	17255
chrX	7476592	C	79	26159	59	27959	63	23762	30	12886	74	53530	37	17243
chrX	7476595	G	1	4303	4	2713	1	1568	3	2881	2	3292	1	2238
chrX	7476596	G	7	4298	7	2709	5	1564	2	2883	4	3291	1	2239
chrX	7476599	C	93	26109	40	27955	67	23729	108	12797	332	53209	22	17224
chrX	7476600	G	9	4292	8	2709	1	1568	2	2882	11	3284	4	2234
chrX	7476601	G	9	4289	6	2708	4	1563	5	2875	4	3286	7	2231
chrX	7476602	C	80	26135	48	27944	39	23767	30	12877	100	53444	38	17207
chrX	7476603	G	6	4299	4	2711	0	1569	9	2876	5	3289	6	2234
chrX	7476606	G	4	4299	3	2711	1	1566	4	2878	6	3285	1	2235
chrX	7476607	G	5	4300	3	2713	2	1566	5	2878	5	3286	0	2239
chrX	7476608	G	5	4300	9	2707	6	1563	13	2869	14	3278	4	2233
chrX	7476609	C	139	26083	34	27967	35	23774	11	12897	114	53462	63	17184
chrX	7476611	C	71	26156	46	27957	35	23774	15	12891	70	53504	37	17217
chrX	7476613	G	10	4294	4	2710	4	1564	3	2880	7	3285	5	2234
chrX	7476614	G	20	4283	5	2708	4	1563	4	2878	6	3285	4	2234
chrX	7476615	C	59	26167	56	27949	95	23710	25	12872	193	53357	82	17146
chrX	7476618	C	26	26174	39	27948	121	23669	97	12803	234	53288	82	17151

39	26076	45	27808	42	23683	17	12816	99	53246	56	17163
34	26239	33	28014	65	23828	8	12910	105	53602	24	17290
3	4315	3	2722	1	1581	4	2895	6	3320	3	2259
8	4315	14	2721	6	1580	7	2891	5	3317	7	2258
58	26211	54	27977	71	23764	24	12901	109	53530	35	17274
65	26239	81	28017	31	23824	101	12916	268	53595	46	17289
0	4315	7	2723	3	1578	5	2894	3	3315	1	2255
4	4314	2	2722	8	1578	0	2893	12	3313	5	2253
61	26232	117	28003	40	23816	20	12901	151	53588	40	17287
22	26241	38	28021	58	23826	10	12915	96	53609	29	17292
5	4313	4	2723	4	1578	2	2891	8	3313	1	2255
8	4312	3	2723	2	1576	6	2892	4	3309	5	2255
57	25995	144	27763	64	23607	38	12776	249	53136	118	17136
4	4306	8	2718	2	1576	9	2885	6	3306	37	2250
78	26230	49	28011	41	23818	42	12908	152	53589	70	17278
25	26245	123	28022	25	23828	15	12917	105	53612	42	17289
4	4265	4	2652	1	1555	3	2858	2	3261	5	2212
7	4306	4	2718	4	1574	2	2889	4	3302	1	2249
2	4307	2	2719	2	1576	4	2889	6	3305	2	2248
9	4305	2	2717	2	1576	6	2887	9	3304	4	2246
5	4305	4	2718	5	1576	6	2888	1	3302	2	2247
132	26233	214	28015	129	23831	127	12916	254	53607	30	17286
10	4300	7	2715	1	1574	7	2886	8	3301	4	2246
69	26233	56	28007	48	23820	29	12909	260	53591	78	17289
90	26241	54	28022	59	23829	30	12915	129	53601	55	17287
5	4302	33	2718	6	1572	7	2884	0	3293	2	2240
65	26016	60	27793	48	23601	116	12797	178	53136	41	17120
3	4304	2	2718	4	1570	6	2885	6	3295	44	2239
50	26243	36	28016	32	23824	19	12910	93	53594	26	17281
79	26238	59	28018	63	23825	30	12916	74	53604	37	17280
1	4304	4	2717	1	1569	3	2884	2	3294	1	2239
7	4305	7	2716	5	1569	2	2885	4	3295	1	2240
93	26202	40	27995	67	23796	108	12905	332	53541	22	17246
9	4301	8	2717	1	1569	2	2884	11	3295	4	2238
9	4298	6	2714	4	1567	5	2880	4	3290	7	2238
80	26215	48	27992	39	23806	30	12907	100	53544	38	17245
6	4305	4	2715	0	1569	9	2885	5	3294	6	2240
4	4303	3	2714	1	1567	4	2882	6	3291	1	2236
5	4305	3	2716	2	1568	5	2883	5	3291	0	2239
5	4305	9	2716	6	1569	13	2882	14	3292	4	2237
139	26222	34	28001	35	23809	11	12908	114	53576	63	17247
71	26227	46	28003	35	23809	15	12906	70	53574	37	17254
10	4304	4	2714	4	1568	3	2883	7	3292	5	2239
20	4303	5	2713	4	1567	4	2882	6	3291	4	2238
59	26226	56	28005	95	23805	25	12897	193	53550	82	17228
26	26200	39	27987	121	23790	97	12900	234	53522	82	17233

chrX	7476619	G	8	4293	1	2710	1	1566	8	2875	6	3283	8	2231
chrX	7476620	G	6	4292	6	2705	2	1565	5	2876	9	3279	1	2238
chrX	7476621	G	8	4291	5	2708	14	1553	20	2863	6	3285	4	2235
chrX	7476623	C	28	26192	38	27962	20	23783	8	12891	63	53496	19	17207
chrX	7476625	G	5	4295	2	2711	0	1567	3	2879	4	3284	5	2234
chrX	7476627	C	43	26168	57	27928	47	23745	27	12866	108	53451	77	17138
chrX	7476628	C	51	26136	60	27902	34	23741	123	12763	307	53204	42	17157
chrX	7476629	G	6	4293	6	2706	2	1563	5	2876	6	3280	5	2234
chrX	7476630	C	64	26145	191	27802	81	23712	160	12739	119	53439	37	17172
chrX	7476631	G	7	4280	6	2700	4	1558	4	2874	6	3279	7	2231
chrX	7476632	C	34	26167	49	27935	57	23733	19	12861	159	53380	80	17113
chrX	7476633	G	5	4294	4	2709	5	1560	6	2875	4	3281	5	2234
chrX	7476635	C	60	26140	45	27929	35	23752	11	12882	193	53339	19	17179
chrX	7476636	G	19	4277	4	2706	3	1561	3	2874	4	3278	5	2232
chrX	7476637	C	65	26132	61	27901	49	23727	27	12862	141	53382	88	17100
chrX	7476639	C	23	26189	31	27956	90	23691	16	12880	73	53472	21	17174
chrX	7476641	G	8	4288	2	2711	5	1560	6	2874	14	3270	4	2234
chrX	7476642	G	9	4285	6	2702	2	1561	5	2870	4	3273	2	2235
chrX	7476643	C	46	26154	45	27927	39	23744	50	12838	291	53215	126	17046
chrX	7476644	G	3	4295	7	2704	5	1559	3	2877	9	3274	5	2232
chrX	7476645	G	13	4285	10	2701	2	1563	7	2873	4	3280	6	2232
chrX	7476647	C	28	26170	24	27951	25	23756	9	12876	69	53450	21	17158
chrX	7476650	C	57	26149	34	27942	55	23716	18	12871	280	53244	68	17111
chrX	7476651	G	6	4282	3	2692	0	1557	7	2866	3	3268	3	2224
chrX	7476652	G	4	4293	2	2705	3	1556	1	2878	3	3273	2	2228
chrX	7476653	G	6	4281	4	2696	2	1558	88	2786	6	3265	4	2220
chrX	7476655	G	8	4287	6	2703	6	1556	5	2873	9	3271	8	2224
chrX	7476656	G	47	4239	9	2697	1	1550	8	2864	10	3267	7	2224
chrX	7476657	C	43	26158	30	27946	27	23738	14	12863	195	53315	89	17081
chrX	7476658	G	3	4284	1	2704	3	1552	1	2875	3	3274	4	2223
chrX	7476659	G	6	4290	4	2705	2	1559	3	2874	6	3274	3	2228
chrX	7476660	G	14	4281	7	2701	10	1551	4	2874	6	3275	4	2228
chrX	7476661	C	37	26162	39	27926	36	23730	20	12865	186	53316	26	17140
chrX	7476662	G	49	4105	6	2614	2	1508	105	2684	4	3180	2	2175
chrX	7476663	G	9	4287	1	2707	5	1554	3	2873	10	3266	6	2222
chrX	7476664	G	20	4276	4	2705	3	1556	2	2874	14	3264	0	2227
chrX	7476665	G	3	4287	7	2701	2	1556	5	2870	4	3272	3	2224
chrX	7476668	G	11	4283	1	2707	9	1550	23	2850	4	3273	6	2221
chrX	7476669	G	8	4279	10	2695	3	1553	4	2870	5	3268	8	2218
chrX	7476670	C	28	26100	22	27868	25	23706	19	12861	74	53343	19	17103
chrX	7476672	G	11	4281	3	2706	1	1558	4	2872	13	3264	1	2227
chrX	7476673	G	0	4292	0	2709	0	1559	1	2876	1	3276	0	2228
chrX	7476674	G	1	4291	0	2709	1	1557	0	2877	0	3277	0	2228
chrX	7476675	G	1	4290	0	2707	0	1557	0	2877	0	3277	1	2224
chrX	7476676	C	3	25870	1	27523	2	23469	1	12828	7	53117	4	16981
chrX	7476678	G	0	4288	1	2705	1	1554	0	2874	1	3272	1	2224

8	4301	1	2711	1	1567	8	2883	6	3289	8	2239
6	4298	6	2711	2	1567	5	2881	9	3288	1	2239
8	4299	5	2713	14	1567	20	2883	6	3291	4	2239
28	26220	38	28000	20	23803	8	12899	63	53559	19	17226
5	4300	2	2713	0	1567	3	2882	4	3288	5	2239
43	26211	57	27985	47	23792	27	12893	108	53559	77	17215
51	26187	60	27962	34	23775	123	12886	307	53511	42	17199
6	4299	6	2712	2	1565	5	2881	6	3286	5	2239
64	26209	191	27993	81	23793	160	12899	119	53558	37	17209
7	4287	6	2706	4	1562	4	2878	6	3285	7	2238
34	26201	49	27984	57	23790	19	12880	159	53539	80	17193
5	4299	4	2713	5	1565	6	2881	4	3285	5	2239
60	26200	45	27974	35	23787	11	12893	193	53532	19	17198
19	4296	4	2710	3	1564	3	2877	4	3282	5	2237
65	26197	61	27962	49	23776	27	12889	141	53523	88	17188
23	26212	31	27987	90	23781	16	12896	73	53545	21	17195
8	4296	2	2713	5	1565	6	2880	14	3284	4	2238
9	4294	6	2708	2	1563	5	2875	4	3277	2	2237
46	26200	45	27972	39	23783	50	12888	291	53506	126	17172
3	4298	7	2711	5	1564	3	2880	9	3283	5	2237
13	4298	10	2711	2	1565	7	2880	4	3284	6	2238
28	26198	24	27975	25	23781	9	12885	69	53519	21	17179
57	26206	34	27976	55	23771	18	12889	280	53524	68	17179
6	4288	3	2695	0	1557	7	2873	3	3271	3	2227
4	4297	2	2707	3	1559	1	2879	3	3276	2	2230
6	4287	4	2700	2	1560	88	2874	6	3271	4	2224
8	4295	6	2709	6	1562	5	2878	9	3280	8	2232
47	4286	9	2706	1	1551	8	2872	10	3277	7	2231
43	26201	30	27976	27	23765	14	12877	195	53510	89	17170
3	4287	1	2705	3	1555	1	2876	3	3277	4	2227
6	4296	4	2709	2	1561	3	2877	6	3280	3	2231
14	4295	7	2708	10	1561	4	2878	6	3281	4	2232
37	26199	39	27965	36	23766	20	12885	186	53502	26	17166
49	4154	6	2620	2	1510	105	2789	4	3184	2	2177
9	4296	1	2708	5	1559	3	2876	10	3276	6	2228
20	4296	4	2709	3	1559	2	2876	14	3278	0	2227
3	4290	7	2708	2	1558	5	2875	4	3276	3	2227
11	4294	1	2708	9	1559	23	2873	4	3277	6	2227
8	4287	10	2705	3	1556	4	2874	5	3273	8	2226
28	26128	22	27890	25	23731	19	12880	74	53417	19	17122
11	4292	3	2709	1	1559	4	2876	13	3277	1	2228
0	4292	0	2709	0	1559	1	2877	1	3277	0	2228
1	4292	0	2709	1	1558	0	2877	0	3277	0	2228
1	4291	0	2707	0	1557	0	2877	0	3277	1	2225
3	25873	1	27524	2	23471	1	12829	7	53124	4	16985
0	4288	1	2706	1	1555	0	2874	1	3273	1	2225

chrX	7476679	C	6	25831	9	27479	5	23416	1	12802	11	52993	2	16964
chrX	7476681	G	0	4290	0	2705	0	1555	0	2874	1	3272	0	2224
chrX	7476683	G	0	4285	1	2700	0	1554	0	2869	0	3268	0	2223
chrX	7476685	C	8	25383	10	27028	9	22382	3	12215	24	50865	7	16402
chrX	7476688	G	0	4287	1	2702	0	1555	0	2871	0	3274	0	2225
chrX	7476690	C	3	25128	2	26743	2	22056	0	12042	7	50174	4	16195
chrX	7476691	C	2	25026	2	26624	0	21974	0	12000	8	49972	3	16122
chrX	7476692	C	2	24937	3	26521	4	21906	1	11965	4	49850	2	16072
chrX	7476694	G	0	4276	0	2696	1	1548	0	2861	0	3267	1	2221
chrX	7476696	C	3	24261	8	25827	0	21401	0	11663	6	48634	2	15623
chrX	7476697	C	0	24001	6	25528	6	21145	0	11534	3	48102	2	15449
chrX	7539243	G	0	2903	1	2522	0	1817	0	712	0	1440	0	1834
chrX	7539248	G	0	2971	1	2584	1	1862	0	730	0	1475	0	1873
chrX	7539250	G	1	3039	1	2677	0	1925	0	756	1	1519	1	1927
chrX	7539258	G	0	3211	0	2875	0	2030	0	813	0	1626	0	2074
chrX	7539275	G	9	3237	0	2890	40	2003	0	815	16	1618	3	2075
chrX	7539276	G	4	3244	2	2890	2	2041	0	816	2	1634	2	2079
chrX	7539286	G	9	3224	7	2879	11	2029	15	800	5	1623	0	2070
chrX	7539290	G	17	3229	4	2883	13	2029	0	816	6	1628	5	2075
chrX	7539302	G	4	3245	6	2886	3	2041	1	814	2	1635	1	2081
chrX	7539303	G	7	3241	8	2884	5	2041	1	816	5	1632	6	2076
chrX	7539304	G	5	3245	5	2887	3	2043	16	799	1	1637	3	2079
chrX	7539305	G	9	3239	8	2884	3	2041	0	817	2	1636	2	2080
chrX	7539311	G	2	3248	1	2893	1	2045	0	816	6	1632	3	2079
chrX	7539312	G	4	3246	25	2866	5	2041	2	815	2	1635	5	2075
chrX	7539315	G	3	3247	6	2887	3	2043	1	816	11	1627	5	2077
chrX	7539316	G	7	3244	9	2880	19	2028	1	816	2	1636	4	2077
chrX	7539324	G	6	3245	5	2887	17	2030	0	817	3	1634	2	2080
chrX	7539331	G	22	2690	2	2347	2	1710	1	675	1	1345	2	1767
chrX	7539332	G	14	3207	11	2848	5	2022	1	807	1	1613	5	2053
chrX	7539333	G	2	3249	6	2886	1	2047	4	813	7	1629	2	2079
chrX	7539334	G	7	3245	8	2885	1	2047	2	815	3	1633	3	2078
chrX	7539335	G	3	3249	6	2888	3	2044	5	812	5	1631	3	2078
chrX	7539338	G	21	3229	11	2883	3	2044	0	817	2	1634	9	2072
chrX	7539343	G	4	3250	2	2893	1	2048	0	817	6	1631	4	2077
chrX	7539344	G	6	3247	8	2888	4	2042	1	816	0	1636	6	2072
chrX	7539345	C	2	7375	1	8586	0	1836	0	2994	1	2668	0	2296
chrX	7539347	G	19	3233	5	2896	33	2017	42	779	5	1630	5	2077
chrX	7539348	C	3	8671	3	10111	1	2163	1	3618	1	3135	0	2631
chrX	7539350	G	26	3229	3	2900	48	2000	33	788	23	1614	25	2058
chrX	7539352	G	6	3251	6	2897	2	2046	1	819	1	1636	9	2075
chrX	7539353	C	1	8804	3	10247	1	2176	0	3663	0	3183	1	2653
chrX	7539355	G	71	3154	41	2846	18	2004	19	798	18	1609	1	2060
chrX	7539357	G	12	3248	0	2903	2	2044	6	815	3	1634	22	2062
chrX	7539359	G	16	3245	5	2898	7	2044	2	819	7	1633	4	2079
chrX	7539360	G	6	3255	5	2898	2	2049	1	820	2	1636	0	2084

6	25837	9	27488	5	23421	1	12803	11	53004	2	16966
0	4290	0	2705	0	1555	0	2874	1	3273	0	2224
0	4285	1	2701	0	1554	0	2869	0	3268	0	2223
8	25391	10	27038	9	22391	3	12218	24	50889	7	16409
0	4287	1	2703	0	1555	0	2871	0	3274	0	2225
3	25131	2	26745	2	22058	0	12042	7	50181	4	16199
2	25028	2	26626	0	21974	0	12000	8	49980	3	16125
2	24939	3	26524	4	21910	1	11966	4	49854	2	16074
0	4276	0	2696	1	1549	0	2861	0	3267	1	2222
3	24264	8	25835	0	21401	0	11663	6	48640	2	15625
0	24001	6	25534	6	21151	0	11534	3	48105	2	15451
0	2903	1	2523	0	1817	0	712	0	1440	0	1834
0	2971	1	2585	1	1863	0	730	0	1475	0	1873
1	3040	1	2678	0	1925	0	756	1	1520	1	1928
0	3211	0	2875	0	2030	0	813	0	1626	0	2074
9	3246	0	2890	40	2043	0	815	16	1634	3	2078
4	3248	2	2892	2	2043	0	816	2	1636	2	2081
9	3233	7	2886	11	2040	15	815	5	1628	0	2070
17	3246	4	2887	13	2042	0	816	6	1634	5	2080
4	3249	6	2892	3	2044	1	815	2	1637	1	2082
7	3248	8	2892	5	2046	1	817	5	1637	6	2082
5	3250	5	2892	3	2046	16	815	1	1638	3	2082
9	3248	8	2892	3	2044	0	817	2	1638	2	2082
2	3250	1	2894	1	2046	0	816	6	1638	3	2082
4	3250	25	2891	5	2046	2	817	2	1637	5	2080
3	3250	6	2893	3	2046	1	817	11	1638	5	2082
7	3251	9	2889	19	2047	1	817	2	1638	4	2081
6	3251	5	2892	17	2047	0	817	3	1637	2	2082
22	2712	2	2349	2	1712	1	676	1	1346	2	1769
14	3221	11	2859	5	2027	1	808	1	1614	5	2058
2	3251	6	2892	1	2048	4	817	7	1636	2	2081
7	3252	8	2893	1	2048	2	817	3	1636	3	2081
3	3252	6	2894	3	2047	5	817	5	1636	3	2081
21	3250	11	2894	3	2047	0	817	2	1636	9	2081
4	3254	2	2895	1	2049	0	817	6	1637	4	2081
6	3253	8	2896	4	2046	1	817	0	1636	6	2078
2	7377	1	8587	0	1836	0	2994	1	2669	0	2296
19	3252	5	2901	33	2050	42	821	5	1635	5	2082
3	8674	3	10114	1	2164	1	3619	1	3136	0	2631
26	3255	3	2903	48	2048	33	821	23	1637	25	2083
6	3257	6	2903	2	2048	1	820	1	1637	9	2084
1	8805	3	10250	1	2177	0	3663	0	3183	1	2654
71	3225	41	2887	18	2022	19	817	18	1627	1	2061
12	3260	0	2903	2	2046	6	821	3	1637	22	2084
16	3261	5	2903	7	2051	2	821	7	1640	4	2083
6	3261	5	2903	2	2051	1	821	2	1638	0	2084

chrX	7539361	G	14	3245	6	2895	8	2041	1	820	6	1633	5	2078
chrX	7539362	C	2	8814	4	10259	1	2195	2	3680	0	3198	0	2662
chrX	7539364	C	1	8839	2	10290	0	2199	0	3691	0	3210	1	2665
chrX	7539365	C	2	8838	2	10290	0	2199	1	3690	0	3210	0	2666
chrX	7539366	C	1	8838	1	10291	0	2199	0	3691	1	3208	1	2665
chrX	7539368	G	10	3247	5	2900	4	2043	0	820	22	1616	5	2079
chrX	7539370	G	13	1822	17	1489	11	1093	4	405	15	879	14	1110
chrX	7539371	G	13	2691	38	2286	6	1652	9	640	9	1349	16	1687
chrX	7539372	G	10	3073	54	2667	6	1940	4	773	10	1549	4	1958
chrX	7539374	G	31	3221	114	2784	12	2037	1	816	10	1625	3	2077
chrX	7539376	G	86	3180	6	2901	21	2032	2	820	29	1610	7	2078
chrX	7539379	G	17	3249	5	2905	15	2038	1	823	3	1638	5	2081
chrX	7539383	G	23	3240	5	2903	2	2051	3	821	3	1639	3	2080
chrX	7539384	G	2	3254	4	2896	3	2042	0	822	0	1641	3	2075
chrX	7539386	C	11	8846	13	10297	2	2202	9	3684	3	3217	5	2670
chrX	7539388	G	80	3183	74	2834	35	2020	32	792	38	1602	21	2066
chrX	7539389	G	22	3242	11	2903	1	2054	1	825	6	1634	12	2074
chrX	7539390	G	6	3247	3	2903	2	2047	1	824	2	1639	13	2068
chrX	7539394	G	0	1365	0	1353	1	892	1	390	0	690	3	877
chrX	7539396	C	129	8739	19	10302	20	2185	6	3690	53	3173	15	2662
chrX	7539398	G	0	351	0	392	0	232	0	106	0	199	1	247
chrX	7539399	C	83	8767	15	10277	7	2194	4	3687	12	3203	2	2650
chrX	7539401	C	11	8857	6	10318	4	2201	2	3697	3	3222	3	2675
chrX	7539402	C	26	8844	33	10292	5	2200	5	3693	10	3216	2	2677
chrX	7539403	C	12	8855	11	10315	2	2204	5	3693	4	3221	4	2676
chrX	7539404	C	36	8832	23	10303	6	2200	3	3696	4	3223	35	2646
chrX	7539405	C	7849	1019	7884	2443	2003	203	3360	339	2928	299	2213	468
chrX	7539406	G	99	10	80	24	61	3	31	4	52	10	45	9
chrX	7539410	G	2	101	0	95	0	60	0	28	2	54	1	46
chrX	7539411	C	34	8766	140	10115	27	2169	34	3639	37	3176	29	2634
chrX	7539413	C	96	8781	36	10295	32	2174	40	3660	32	3199	13	2669
chrX	7539415	G	0	102	0	90	0	61	0	25	0	54	0	43
chrX	7539416	C	29	8856	18	10318	14	2192	34	3666	12	3221	1	2680
chrX	7539417	C	8210	676	8731	1603	1976	231	3000	701	2824	407	2254	431
chrX	7539418	G	66	19	64	20	42	11	21	4	40	7	37	9
chrX	7539419	G	0	77	0	84	0	53	0	25	0	46	0	46
chrX	7539421	G	1	74	0	81	2	47	1	22	0	49	0	45
chrX	7539423	C	9	8868	70	10262	31	2178	3	3691	46	3189	30	2653
chrX	7539425	G	0	79	0	82	0	51	0	24	0	50	0	42
chrX	7539426	G	0	79	0	82	0	51	0	24	0	50	0	42
chrX	7539427	G	0	79	0	82	0	51	1	23	0	50	0	42
chrX	7539429	G	5	73	6	70	4	43	2	22	5	42	0	38
chrX	7539430	C	8026	866	9386	951	2006	204	3286	415	2953	284	2428	262
chrX	7539431	G	71	8	72	5	45	3	22	2	42	5	33	5
chrX	7539434	G	3	76	6	72	5	43	1	24	0	50	2	37
chrX	7539435	C	267	8624	25	10316	51	2158	82	3618	44	3197	73	2617

14	3259	6	2901	8	2049	1	821	6	1639	5	2083
2	8816	4	10263	1	2196	2	3682	0	3198	0	2662
1	8840	2	10292	0	2199	0	3691	0	3210	1	2666
2	8840	2	10292	0	2199	1	3691	0	3210	0	2666
1	8839	1	10292	0	2199	0	3691	1	3209	1	2666
10	3257	5	2905	4	2047	0	820	22	1638	5	2084
13	1835	17	1506	11	1104	4	409	15	894	14	1124
13	2704	38	2324	6	1658	9	649	9	1358	16	1703
10	3083	54	2721	6	1946	4	777	10	1559	4	1962
31	3252	114	2898	12	2049	1	817	10	1635	3	2080
86	3266	6	2907	21	2053	2	822	29	1639	7	2085
17	3266	5	2910	15	2053	1	824	3	1641	5	2086
23	3263	5	2908	2	2053	3	824	3	1642	3	2083
2	3256	4	2900	3	2045	0	822	0	1641	3	2078
11	8857	13	10310	2	2204	9	3693	3	3220	5	2675
80	3263	74	2908	35	2055	32	824	38	1640	21	2087
22	3264	11	2914	1	2055	1	826	6	1640	12	2086
6	3253	3	2906	2	2049	1	825	2	1641	13	2081
0	1365	0	1353	1	893	1	391	0	690	3	880
129	8868	19	10321	20	2205	6	3696	53	3226	15	2677
0	351	0	392	0	232	0	106	0	199	1	248
83	8850	15	10292	7	2201	4	3691	12	3215	2	2652
11	8868	6	10324	4	2205	2	3699	3	3225	3	2678
26	8870	33	10325	5	2205	5	3698	10	3226	2	2679
12	8867	11	10326	2	2206	5	3698	4	3225	4	2680
36	8868	23	10326	6	2206	3	3699	4	3227	35	2681
7849	8868	7884	10327	2003	2206	3360	3699	2928	3227	2213	2681
99	109	80	104	61	64	31	35	52	62	45	54
2	103	0	95	0	60	0	28	2	56	1	47
34	8800	140	10255	27	2196	34	3673	37	3213	29	2663
96	8877	36	10331	32	2206	40	3700	32	3231	13	2682
0	102	0	90	0	61	0	25	0	54	0	43
29	8885	18	10336	14	2206	34	3700	12	3233	1	2681
8210	8886	8731	10334	1976	2207	3000	3701	2824	3231	2254	2685
66	85	64	84	42	53	21	25	40	47	37	46
0	77	0	84	0	53	0	25	0	46	0	46
1	75	0	81	2	49	1	23	0	49	0	45
9	8877	70	10332	31	2209	3	3694	46	3235	30	2683
0	79	0	82	0	51	0	24	0	50	0	42
0	79	0	82	0	51	0	24	0	50	0	42
0	79	0	82	0	51	1	24	0	50	0	42
5	78	6	76	4	47	2	24	5	47	0	38
8026	8892	9386	10337	2006	2210	3286	3701	2953	3237	2428	2690
71	79	72	77	45	48	22	24	42	47	33	38
3	79	6	78	5	48	1	25	0	50	2	39
267	8891	25	10341	51	2209	82	3700	44	3241	73	2690

chrX	7539437	G	1	78	1	75	0	48	0	25	1	49	1	38
chrX	7539438	G	0	80	0	78	0	49	0	24	0	50	0	40
chrX	7539439	C	40	8826	12	10301	4	2204	10	3678	7	3231	7	2681
chrX	7539441	G	0	80	1	77	0	49	1	23	2	48	0	40
chrX	7539443	G	4	76	1	74	3	46	0	24	4	45	2	37
chrX	7539444	C	418	8448	331	9971	111	2095	212	3478	201	3037	140	2542
chrX	7539446	G	0	82	0	75	0	51	0	23	0	51	0	42
chrX	7539448	C	42	8856	15	10331	6	2207	6	3698	9	3235	6	2687
chrX	7539449	C	30	8866	159	10187	1	2212	50	3654	10	3234	5	2688
chrX	7539450	C	10	8888	12	10330	3	2209	1	3700	11	3232	11	2680
chrX	7539451	C	34	8867	15	10331	15	2199	2	3701	10	3232	5	2688
chrX	7539453	G	0	87	0	74	2	48	0	24	0	54	0	42
chrX	7539454	C	7659	1236	8888	1457	1958	258	3437	268	2755	490	2302	394
chrX	7539455	G	73	13	64	9	47	3	22	2	45	8	38	5
chrX	7539457	C	15	8892	21	10325	14	2201	42	3663	15	3233	3	2694
chrX	7539459	G	3	85	0	76	3	49	0	23	0	58	0	48
chrX	7539460	G	2	87	1	75	0	52	0	23	0	59	0	48
chrX	7539462	G	1	89	0	77	0	54	0	24	0	62	1	48
chrX	7539463	G	1	90	0	79	0	54	0	24	1	62	0	49
chrX	7539465	C	10	8899	138	10212	4	2217	10	3692	16	3232	23	2677
chrX	7539467	G	0	92	5	75	0	56	0	25	0	65	0	49
chrX	7539468	G	0	93	0	80	1	57	0	27	0	66	0	48
chrX	7539470	C	17	8891	31	10318	3	2226	14	3689	13	3239	14	2689
chrX	7539472	C	111	8802	144	10209	40	2190	147	3561	82	3172	22	2684
chrX	7539474	G	0	90	0	81	0	55	0	30	0	70	0	51
chrX	7539475	C	7	8902	14	10335	5	2226	6	3702	5	3244	5	2700
chrX	7539476	C	8191	724	9331	1019	2072	150	3372	333	2954	295	2492	209
chrX	7539477	G	86	9	80	7	52	8	29	1	64	4	46	6
chrX	7539478	C	20	8899	62	10291	30	2206	50	3658	34	3224	21	2689
chrX	7539480	C	15	8903	12	10347	6	2234	2	3705	15	3242	2	2706
chrX	7539481	C	7948	970	8618	1741	1999	241	2976	732	2946	311	2372	338
chrX	7539482	G	121	14	74	17	58	11	29	4	64	5	49	7
chrX	7539484	C	14	8909	24	10339	14	2226	10	3698	7	3252	7	2702
chrX	7539487	C	44	8879	12	10351	25	2214	4	3705	15	3245	4	2705
chrX	7539490	G	0	142	0	96	0	82	0	39	0	79	0	63
chrX	7539491	G	0	169	0	121	0	99	0	54	0	99	0	87
chrX	7539493	G	13	511	14	432	6	296	7	146	24	265	11	334
chrX	7539495	C	42	8873	14	10330	14	2229	8	3693	8	3253	10	2697
chrX	7539496	C	11	1771	8	2033	12	687	20	884	16	1057	15	685
chrX	7539499	G	3	803	0	713	2	462	3	218	0	435	2	510
chrX	7539502	C	252	67	205	110	300	88	173	64	424	193	235	114
chrX	7539503	G	2706	537	2287	578	1675	350	696	122	1360	278	1612	439
chrX	7539505	G	2	3299	1	2918	2	2065	1	837	11	1661	1	2101
chrX	7539508	G	16	3284	6	2912	1	2064	1	837	7	1665	1	2100
chrX	7539511	G	19	3279	4	2911	2	2061	0	838	9	1659	5	2094
chrX	7539512	C	0	211	0	212	4	297	0	151	3	453	3	258

1	79	1	76	0	48	0	25	1	50	1	39
0	80	0	78	0	49	0	24	0	50	0	40
40	8866	12	10313	4	2208	10	3688	7	3238	7	2688
0	80	1	78	0	49	1	24	2	50	0	40
4	80	1	75	3	49	0	24	4	49	2	39
418	8866	331	10302	111	2206	212	3690	201	3238	140	2682
0	82	0	75	0	51	0	23	0	51	0	42
42	8898	15	10346	6	2213	6	3704	9	3244	6	2693
30	8896	159	10346	1	2213	50	3704	10	3244	5	2693
10	8898	12	10342	3	2212	1	3701	11	3243	11	2691
34	8901	15	10346	15	2214	2	3703	10	3242	5	2693
0	87	0	74	2	50	0	24	0	54	0	42
7659	8895	8888	10345	1958	2216	3437	3705	2755	3245	2302	2696
73	86	64	73	47	50	22	24	45	53	38	43
15	8907	21	10346	14	2215	42	3705	15	3248	3	2697
3	88	0	76	3	52	0	23	0	58	0	48
2	89	1	76	0	52	0	23	0	59	0	48
1	90	0	77	0	54	0	24	0	62	1	49
1	91	0	79	0	54	0	24	1	63	0	49
10	8909	138	10350	4	2221	10	3702	16	3248	23	2700
0	92	5	80	0	56	0	25	0	65	0	49
0	93	0	80	1	58	0	27	0	66	0	48
17	8908	31	10349	3	2229	14	3703	13	3252	14	2703
111	8913	144	10353	40	2230	147	3708	82	3254	22	2706
0	90	0	81	0	55	0	30	0	70	0	51
7	8909	14	10349	5	2231	6	3708	5	3249	5	2705
8191	8915	9331	10350	2072	2222	3372	3705	2954	3249	2492	2701
86	95	80	87	52	60	29	30	64	68	46	52
20	8919	62	10353	30	2236	50	3708	34	3258	21	2710
15	8918	12	10359	6	2240	2	3707	15	3257	2	2708
7948	8918	8618	10359	1999	2240	2976	3708	2946	3257	2372	2710
121	135	74	91	58	69	29	33	64	69	49	56
14	8923	24	10363	14	2240	10	3708	7	3259	7	2709
44	8923	12	10363	25	2239	4	3709	15	3260	4	2709
0	142	0	96	0	82	0	39	0	79	0	63
0	169	0	121	0	99	0	54	0	99	0	87
13	524	14	446	6	302	7	153	24	289	11	345
42	8915	14	10344	14	2243	8	3701	8	3261	10	2707
11	1782	8	2041	12	699	20	904	16	1073	15	700
3	806	0	713	2	464	3	221	0	435	2	512
252	319	205	315	300	388	173	237	424	617	235	349
2706	3243	2287	2865	1675	2025	696	818	1360	1638	1612	2051
2	3301	1	2919	2	2067	1	838	11	1672	1	2102
16	3300	6	2918	1	2065	1	838	7	1672	1	2101
19	3298	4	2915	2	2063	0	838	9	1668	5	2099
0	211	0	212	4	301	0	151	3	456	3	261

chrX	7539514	C	1	327	0	312	0	372	0	211	2	597	1	341
chrX	7539517	C	1	317	0	304	2	343	0	188	1	536	0	310
chrX	7539518	C	0	332	0	324	1	379	0	208	1	615	2	343
chrX	7539519	C	1	327	1	322	3	376	0	209	0	614	0	344
chrX	7539522	C	0	313	1	292	0	352	1	188	0	548	0	314
chrX	7539523	C	1	321	1	296	0	354	0	187	2	550	2	314
chrX	7539525	G	4	3292	6	2907	5	2060	19	817	2	1666	1	2097
chrX	7539526	G	1	3297	5	2907	0	2064	2	834	2	1669	2	2096
chrX	7539528	C	0	317	0	297	0	322	0	163	1	497	0	290
chrX	7539531	C	0	331	0	298	0	300	0	161	0	493	1	288
chrX	7539532	C	2	334	0	305	0	261	0	151	1	452	3	265
chrX	7539534	G	6	3291	2	2911	2	2060	4	832	1	1670	0	2098
chrX	7539535	G	5	3291	6	2906	6	2056	1	835	5	1665	2	2096
chrX	7539536	G	8	3285	2	2902	4	2058	1	835	6	1661	6	2087
chrX	7539537	C	19	8835	18	10290	9	2059	6	3619	9	2961	6	2583
chrX	7539538	C	10	8839	11	10295	4	2064	3	3621	6	2965	4	2588
chrX	7539540	G	33	3033	95	2608	18	1910	30	746	17	1545	5	1946
chrX	7539541	G	1	3288	5	2896	7	2053	2	829	4	1661	6	2085
chrX	7539542	G	3	3294	5	2907	3	2059	0	834	4	1665	3	2094
chrX	7539545	G	2	3294	6	2905	3	2058	0	834	3	1665	3	2092
chrX	7539546	G	6	3290	7	2905	0	2061	1	834	4	1664	0	2097
chrX	7539547	G	6	3289	9	2901	4	2057	2	833	2	1667	6	2089
chrX	7539548	G	9	3283	5	2903	6	2052	1	833	7	1658	5	2089
chrX	7539549	C	65	8785	157	10136	4	2026	6	3594	15	2893	5	2543
chrX	7539552	C	33	8815	16	10271	2	2032	4	3597	1	2900	40	2511
chrX	7539553	C	74	8759	156	10122	4	2008	15	3572	21	2858	11	2529
chrX	7539555	C	5	8846	7	10287	5	2025	5	3593	1	2896	8	2540
chrX	7539558	G	4	3290	4	2908	0	2060	0	834	7	1658	2	2094
chrX	7539559	G	5	3286	4	2905	2	2056	1	832	8	1654	1	2094
chrX	7539560	C	7	8829	15	10267	6	2004	3	3587	20	2839	10	2510
chrX	7539562	G	15	3279	145	2761	30	2029	35	798	29	1633	57	2036
chrX	7539563	G	6	3288	4	2907	5	2053	1	833	3	1662	5	2091
chrX	7539564	G	31	3263	4	2906	0	2059	1	833	2	1663	1	2095
chrX	7539565	G	16	3273	8	2901	0	2056	4	829	7	1653	6	2083
chrX	7539566	C	86	8746	8	10269	4	1979	68	3497	16	2809	48	2456
chrX	7539568	C	80	8752	8	10273	15	1966	7	3560	19	2808	1	2506
chrX	7539570	C	117	8711	202	10072	46	1935	232	3333	69	2753	17	2489
chrX	7539572	G	4	3285	4	2905	4	2053	1	833	3	1661	2	2091
chrX	7539575	G	1	3286	0	2906	0	2056	0	831	2	1660	1	2093
chrX	7539577	G	20	3268	2	2906	20	2035	1	833	7	1656	13	2080
chrX	7539578	G	8	3282	4	2904	3	2052	0	834	4	1659	5	2088
chrX	7539579	C	6	8822	8	10265	1	1976	5	3558	26	2792	1	2499
chrX	7539581	G	4	3285	3	2905	2	2054	0	834	1	1662	12	2081
chrX	7539582	G	2	3287	3	2904	3	2052	0	834	1	1662	3	2089
chrX	7539583	G	6	3281	5	2903	14	2041	2	832	1	1661	8	2085
chrX	7539586	G	3	3285	2	2905	4	2051	13	821	2	1659	0	2091

1	328	0	312	0	372	0	211	2	599	1	342
1	318	0	304	2	345	0	188	1	537	0	310
0	332	0	324	1	380	0	208	1	616	2	345
1	328	1	323	3	379	0	209	0	614	0	344
0	313	1	293	0	352	1	189	0	548	0	314
1	322	1	297	0	354	0	187	2	552	2	316
4	3296	6	2913	5	2065	19	836	2	1668	1	2098
1	3298	5	2912	0	2064	2	836	2	1671	2	2098
0	317	0	297	0	322	0	163	1	498	0	290
0	331	0	298	0	300	0	161	0	493	1	289
2	336	0	305	0	261	0	151	1	453	3	268
6	3297	2	2913	2	2062	4	836	1	1671	0	2098
5	3296	6	2912	6	2062	1	836	5	1670	2	2098
8	3293	2	2904	4	2062	1	836	6	1667	6	2093
19	8854	18	10308	9	2068	6	3625	9	2970	6	2589
10	8849	11	10306	4	2068	3	3624	6	2971	4	2592
33	3066	95	2703	18	1928	30	776	17	1562	5	1951
1	3289	5	2901	7	2060	2	831	4	1665	6	2091
3	3297	5	2912	3	2062	0	834	4	1669	3	2097
2	3296	6	2911	3	2061	0	834	3	1668	3	2095
6	3296	7	2912	0	2061	1	835	4	1668	0	2097
6	3295	9	2910	4	2061	2	835	2	1669	6	2095
9	3292	5	2908	6	2058	1	834	7	1665	5	2094
65	8850	157	10293	4	2030	6	3600	15	2908	5	2548
33	8848	16	10287	2	2034	4	3601	1	2901	40	2551
74	8833	156	10278	4	2012	15	3587	21	2879	11	2540
5	8851	7	10294	5	2030	5	3598	1	2897	8	2548
4	3294	4	2912	0	2060	0	834	7	1665	2	2096
5	3291	4	2909	2	2058	1	833	8	1662	1	2095
7	8836	15	10282	6	2010	3	3590	20	2859	10	2520
15	3294	145	2906	30	2059	35	833	29	1662	57	2093
6	3294	4	2911	5	2058	1	834	3	1665	5	2096
31	3294	4	2910	0	2059	1	834	2	1665	1	2096
16	3289	8	2909	0	2056	4	833	7	1660	6	2089
86	8832	8	10277	4	1983	68	3565	16	2825	48	2504
80	8832	8	10281	15	1981	7	3567	19	2827	1	2507
117	8828	202	10274	46	1981	232	3565	69	2822	17	2506
4	3289	4	2909	4	2057	1	834	3	1664	2	2093
1	3287	0	2906	0	2056	0	831	2	1662	1	2094
20	3288	2	2908	20	2055	1	834	7	1663	13	2093
8	3290	4	2908	3	2055	0	834	4	1663	5	2093
6	8828	8	10273	1	1977	5	3563	26	2818	1	2500
4	3289	3	2908	2	2056	0	834	1	1663	12	2093
2	3289	3	2907	3	2055	0	834	1	1663	3	2092
6	3287	5	2908	14	2055	2	834	1	1662	8	2093
3	3288	2	2907	4	2055	13	834	2	1661	0	2091

chrX	7539588	G	219	3070	93	2814	85	1970	89	745	128	1533	149	1944
chrX	7539590	G	85	3192	84	2812	23	2019	37	793	47	1613	87	2000
chrX	7539591	C	37	8783	18	10248	11	1956	9	3552	13	2796	7	2488
chrX	7539592	C	7995	801	9246	965	1790	170	3170	373	2645	151	2234	250
chrX	7539593	G	2996	292	2466	439	1903	152	787	47	1490	171	1930	163
chrX	7539595	C	9	8810	27	10234	5	1961	6	3554	12	2799	7	2486
chrX	7539598	G	39	3244	62	2840	59	1988	1	832	19	1639	2	2090
chrX	7539599	C	77	8738	14	10242	9	1953	6	3543	9	2797	2	2485
chrX	7539600	C	14	8799	162	10094	6	1955	17	3534	12	2796	23	2467
chrX	7539604	G	4	3280	5	2901	4	2049	1	831	6	1654	6	2087
chrX	7539605	G	9	3256	5	2877	2	2039	2	823	2	1645	4	2074
chrX	7539606	C	33	8778	22	10226	3	1957	8	3537	6	2795	2	2484
chrX	7539607	C	7	8801	13	10234	3	1954	4	3539	4	2795	4	2483
chrX	7539608	C	18	8792	11	10239	10	1949	23	3521	10	2790	15	2472
chrX	7539610	G	7	3276	7	2896	0	2052	1	832	4	1654	4	2086
chrX	7539611	C	6	8798	13	10237	8	1950	4	3537	4	2782	6	2477
chrX	7539614	G	9	3272	7	2896	2	2048	0	833	6	1649	16	2073
chrX	7539615	C	41	8760	86	10159	26	1931	14	3526	19	2766	15	2465
chrX	7539620	G	8	3269	16	2887	4	2045	0	831	3	1651	18	2073
chrX	7539621	G	3	3277	14	2888	19	2030	1	830	6	1649	3	2088
chrX	7539623	G	147	3132	3	2899	110	1938	123	709	64	1589	47	2044
chrX	7539625	G	6	3273	11	2892	4	2044	3	829	3	1650	1	2089
chrX	7539626	C	25	8761	23	10195	3	1951	6	3524	10	2759	5	2474
chrX	7539627	C	18	8775	19	10214	4	1950	9	3523	10	2768	15	2466
chrX	7539628	C	19	8769	20	10215	0	1953	3	3529	5	2770	16	2465
chrX	7539629	C	12	8771	13	10221	7	1945	3	3529	0	2780	3	2477
chrX	7539630	C	83	8703	7	10228	4	1948	6	3525	36	2741	11	2466
chrX	7539632	G	0	3231	3	2889	0	2034	0	830	0	1652	0	2086
chrX	7539633	G	0	3233	1	2891	0	2034	0	830	0	1652	2	2084
chrX	7539638	C	53	8703	18	10179	6	1921	6	3517	3	2733	18	2419
chrX	7539639	C	43	8716	8	10192	0	1941	41	3480	20	2736	18	2437
chrX	7539640	C	46	8708	22	10174	20	1919	6	3514	53	2698	3	2449
chrX	7539642	C	16	8736	17	10171	7	1917	3	3516	9	2726	19	2414
chrX	7539643	C	205	8552	11	10182	29	1897	14	3507	12	2722	66	2368
chrX	7539647	C	49	8705	24	10159	8	1918	9	3509	7	2727	4	2430
chrX	7539649	G	1	2563	0	2254	0	1633	0	638	1	1280	1	1622
chrX	7539652	G	1	2497	0	2199	0	1608	0	625	0	1250	1	1576
chrX	7539655	C	19	8724	42	10136	12	1909	44	3473	35	2687	4	2427
chrX	7539664	C	0	8696	1	10130	0	1918	0	3514	1	2710	0	2425
chrX	7539666	C	1	8691	1	10124	0	1916	0	3512	0	2710	0	2423
chrX	7539667	C	0	8693	1	10128	0	1917	1	3513	0	2710	1	2422
chrX	7539675	C	2	8677	0	10119	0	1913	0	3513	0	2696	0	2416

219	3289	93	2907	85	2055	89	834	128	1661	149	2093
85	3277	84	2896	23	2042	37	830	47	1660	87	2087
37	8820	18	10266	11	1967	9	3561	13	2809	7	2495
7995	8796	9246	10211	1790	1960	3170	3543	2645	2796	2234	2484
2996	3288	2466	2905	1903	2055	787	834	1490	1661	1930	2093
9	8819	27	10261	5	1966	6	3560	12	2811	7	2493
39	3283	62	2902	59	2047	1	833	19	1658	2	2092
77	8815	14	10256	9	1962	6	3549	9	2806	2	2487
14	8813	162	10256	6	1961	17	3551	12	2808	23	2490
4	3284	5	2906	4	2053	1	832	6	1660	6	2093
9	3265	5	2882	2	2041	2	825	2	1647	4	2078
33	8811	22	10248	3	1960	8	3545	6	2801	2	2486
7	8808	13	10247	3	1957	4	3543	4	2799	4	2487
18	8810	11	10250	10	1959	23	3544	10	2800	15	2487
7	3283	7	2903	0	2052	1	833	4	1658	4	2090
6	8804	13	10250	8	1958	4	3541	4	2786	6	2483
9	3281	7	2903	2	2050	0	833	6	1655	16	2089
41	8801	86	10245	26	1957	14	3540	19	2785	15	2480
8	3277	16	2903	4	2049	0	831	3	1654	18	2091
3	3280	14	2902	19	2049	1	831	6	1655	3	2091
147	3279	3	2902	110	2048	123	832	64	1653	47	2091
6	3279	11	2903	4	2048	3	832	3	1653	1	2090
25	8786	23	10218	3	1954	6	3530	10	2769	5	2479
18	8793	19	10233	4	1954	9	3532	10	2778	15	2481
19	8788	20	10235	0	1953	3	3532	5	2775	16	2481
12	8783	13	10234	7	1952	3	3532	0	2780	3	2480
83	8786	7	10235	4	1952	6	3531	36	2777	11	2477
0	3231	3	2892	0	2034	0	830	0	1652	0	2086
0	3233	1	2892	0	2034	0	830	0	1652	2	2086
53	8756	18	10197	6	1927	6	3523	3	2736	18	2437
43	8759	8	10200	0	1941	41	3521	20	2756	18	2455
46	8754	22	10196	20	1939	6	3520	53	2751	3	2452
16	8752	17	10188	7	1924	3	3519	9	2735	19	2433
205	8757	11	10193	29	1926	14	3521	12	2734	66	2434
49	8754	24	10183	8	1926	9	3518	7	2734	4	2434
1	2564	0	2254	0	1633	0	638	1	1281	1	1623
1	2498	0	2199	0	1608	0	625	0	1250	1	1577
19	8743	42	10178	12	1921	44	3517	35	2722	4	2431
0	8696	1	10131	0	1918	0	3514	1	2711	0	2425
1	8692	1	10125	0	1916	0	3512	0	2710	0	2423
0	8693	1	10129	0	1917	1	3514	0	2710	1	2423
2	8679	0	10119	0	1913	0	3513	0	2696	0	2416

Tet2kd wild type cytosines

Number of C's and T's

			t2kdbs1		t2kdoxbs1		t2kdbs2		t2kdoxbs2		t2kdbs3		t2kdoxbs3	
**Chromosome**	**Position**		**C to C**	**C to T**	**C to C**	**C to T**	**C to C**	**C to T**	**C to C**	**C to T**	**C to C**	**C to T**	**C to C**	**C to T**
chr11	3525591	G	0	744	0	1085	0	1050	0	796	0	3426	0	1347
chr11	3525594	G	0	752	1	1099	0	1054	0	813	0	3449	2	1360
chr11	3525596	G	0	762	0	1113	0	1061	0	832	1	3468	0	1380
chr11	3525598	C	6	8682	0	1625	3	12567	4	4897	3	10439	2	15408
chr11	3525599	C	1	10587	0	2044	1	15375	0	5988	1	13337	2	19239
chr11	3525603	C	3	10840	0	2104	1	15692	2	6164	0	13594	3	19747
chr11	3525605	C	5	11014	0	2142	1	15901	1	6321	1	13765	3	20126
chr11	3525606	C	3	11635	1	2419	6	16780	1	7073	1	14405	3	21583
chr11	3525608	G	0	671	0	1032	0	939	0	812	0	3084	0	1239
chr11	3525609	G	0	767	0	1184	0	1069	0	936	0	3497	0	1451
chr11	3525611	G	4	795	117	1132	19	1088	70	907	19	3554	2	1529
chr11	3525612	G	3	797	3	1268	3	1106	5	981	4	3573	1	1540
chr11	3525615	G	2	800	4	1286	5	1102	0	1002	14	3563	3	1545
chr11	3525618	G	2	800	1	1289	7	1101	2	1002	8	3568	6	1545
chr11	3525619	G	3	796	2	1287	4	1103	3	1000	12	3564	8	1541
chr11	3525620	C	4	13716	1	3178	2	19751	2	9682	2	17225	9	27416
chr11	3525623	G	2	800	0	1291	0	1106	0	1005	17	3559	18	1533
chr11	3525624	G	6	797	0	1290	1	1107	1	1004	4	3573	3	1547
chr11	3525628	G	11	792	3	1288	3	1105	3	1002	9	3566	1	1550
chr11	3525629	C	26	13882	2	3319	70	19934	7	10002	24	17288	24	27650
chr11	3525633	G	2	803	3	1290	1	1107	1	1006	11	3568	1	1550
chr11	3525635	G	2	804	3	1291	7	1102	5	1002	11	3567	3	1547
chr11	3525636	C	53	13870	11	3327	54	19963	17	10011	43	17291	40	27655
chr11	3525638	G	9	795	18	1276	17	1091	1	1006	52	3525	84	1465
chr11	3525639	C	84	13837	1	3335	253	19762	16	10009	233	17103	752	26937
chr11	3525642	G	3	802	4	1291	2	1105	2	1004	9	3566	17	1534
chr11	3525643	C	9565	4309	1885	1439	14058	5886	5481	4503	12875	4421	20835	6788
chr11	3525644	G	566	223	796	479	698	401	740	238	2548	969	997	524
chr11	3525645	C	32	13893	20	3319	61	19957	25	9999	51	17293	48	27634
chr11	3525647	G	16	790	33	1259	19	1086	3	1003	51	3528	8	1545
chr11	3525649	C	21	13912	6	3336	28	19994	15	10015	55	17302	33	27658
chr11	3525657	C	60	13877	7	3336	34	19996	21	10012	41	17323	45	27655
chr11	3525660	G	2	805	26	1269	5	1105	1	1007	13	3572	2	1555
chr11	3525663	G	8	799	0	1297	5	1104	1	1008	6	3578	0	1556
chr11	3525665	G	2	805	6	1291	2	1108	3	1006	13	3573	3	1554
chr11	3525666	C	28	13914	6	3336	58	19976	18	10017	61	17318	61	27650
chr11	3525668	C	10581	3361	2399	946	15355	4679	7592	2449	13769	3617	19164	8547
chr11	3525669	G	652	155	973	321	840	265	778	226	2886	695	1151	403
chr11	3525670	C	88	13848	7	3335	63	19962	16	10010	68	17308	141	27559
chr11	3525672	C	74	13873	191	3154	16	20022	15	10026	36	17353	31	27687

Number of C's and total read outs

t2kdbs1		t2kdoxbs1		t2kdbs2		t2kdoxbs2		t2kdbs3		t2kdoxbs3	
C	Total	C	Total	C	Total	C	Total	C	Total	C	Total
0	744	0	1085	0	1050	0	796	0	3426	0	1347
0	752	1	1100	0	1054	0	813	0	3449	2	1362
0	762	0	1113	0	1061	0	832	1	3469	0	1380
6	8688	0	1625	3	12570	4	4901	3	10442	2	15410
1	10588	0	2044	1	15376	0	5988	1	13338	2	19241
3	10843	0	2104	1	15693	2	6166	0	13594	3	19750
5	11019	0	2142	1	15902	1	6322	1	13766	3	20129
3	11638	1	2420	6	16786	1	7074	1	14406	3	21586
0	671	0	1032	0	939	0	812	0	3084	0	1239
0	767	0	1184	0	1069	0	936	0	3497	0	1451
4	799	117	1249	19	1107	70	977	19	3573	2	1531
3	800	3	1271	3	1109	5	986	4	3577	1	1541
2	802	4	1290	5	1107	0	1002	14	3577	3	1548
2	802	1	1290	7	1108	2	1004	8	3576	6	1551
3	799	2	1289	4	1107	3	1003	12	3576	8	1549
4	13720	1	3179	2	19753	2	9684	2	17227	9	27425
2	802	0	1291	0	1106	0	1005	17	3576	18	1551
6	803	0	1290	1	1108	1	1005	4	3577	3	1550
11	803	3	1291	3	1108	3	1005	9	3575	1	1551
26	13908	2	3321	70	20004	7	10009	24	17312	24	27674
2	805	3	1293	1	1108	1	1007	11	3579	1	1551
2	806	3	1294	7	1109	5	1007	11	3578	3	1550
53	13923	11	3338	54	20017	17	10028	43	17334	40	27695
9	804	18	1294	17	1108	1	1007	52	3577	84	1549
84	13921	1	3336	253	20015	16	10025	233	17336	752	27689
3	805	4	1295	2	1107	2	1006	9	3575	17	1551
9565	13874	1885	3324	14058	19944	5481	9984	12875	17296	20835	27623
566	789	796	1275	698	1099	740	978	2548	3517	997	1521
32	13925	20	3339	61	20018	25	10024	51	17344	48	27682
16	806	33	1292	19	1105	3	1006	51	3579	8	1553
21	13933	6	3342	28	20022	15	10030	55	17357	33	27691
60	13937	7	3343	34	20030	21	10033	41	17364	45	27700
2	807	26	1295	5	1110	1	1008	13	3585	2	1557
8	807	0	1297	5	1109	1	1009	6	3584	0	1556
2	807	6	1297	2	1110	3	1009	13	3586	3	1557
28	13942	6	3342	58	20034	18	10035	61	17379	61	27711
10581	13942	2399	3345	15355	20034	7592	10041	13769	17386	19164	27711
652	807	973	1294	840	1105	778	1004	2886	3581	1151	1554
88	13936	7	3342	63	20025	16	10026	68	17376	141	27700
74	13947	191	3345	16	20038	15	10041	36	17389	31	27718

chr11	3525674	C	9142	4804	1851	1493	12751	7286	7009	3032	11489	5900	16969	10755
chr11	3525675	G	565	238	860	426	723	378	714	286	2477	1078	961	589
chr11	3525678	C	16	13932	3	3345	15	20026	5	10037	58	17341	23	27701
chr11	3525680	C	10841	3108	2630	718	15661	4380	7112	2931	13959	3443	20753	6975
chr11	3525681	G	638	167	1108	191	864	245	758	251	2945	637	1033	521
chr11	3525686	C	64	13887	3	3345	19	20018	9	10035	22	17387	24	27709
chr11	3525688	G	0	811	3	1298	5	1107	2	1008	18	3571	15	1544
chr11	3525689	G	2	809	11	1278	1	1112	0	1010	13	3577	3	1556
chr11	3525692	C	42	13916	5	3341	28	20017	12	10028	47	17377	45	27705
chr11	3525693	C	75	13888	34	3315	142	19909	5	10045	96	17336	23	27740
chr11	3525695	G	0	812	0	1300	5	1109	2	1006	11	3583	1	1560
chr11	3525696	G	1	811	1	1300	4	1110	3	1006	17	3579	1	1559
chr11	3525699	C	20	13945	3	3347	25	20029	12	10043	27	17414	37	27734
chr11	3525702	G	1	811	1	1300	1	1112	1	1009	6	3590	9	1554
chr11	3525703	G	2	810	1	1301	5	1109	2	1006	17	3578	1	1561
chr11	3525705	C	17	13957	7	3343	66	19997	12	10044	46	17411	49	27724
chr11	3525708	G	8	803	26	1272	14	1100	31	979	52	3544	18	1546
chr11	3525710	C	20	13957	5	3345	24	20038	13	10046	40	17423	34	27748
chr11	3525711	C	67	13908	60	3289	250	19809	351	9705	212	17251	58	27717
chr11	3525713	G	3	811	1	1301	2	1112	1	1010	20	3581	21	1546
chr11	3525714	G	3	811	3	1299	1	1115	4	1007	11	3587	2	1565
chr11	3525716	C	8953	5028	2269	1082	12469	7598	6499	3559	11432	6042	17580	10197
chr11	3525717	G	591	221	971	330	846	270	755	255	2756	842	1208	356
chr11	3525718	G	2	812	2	1300	7	1110	1	1010	6	3597	5	1562
chr11	3525719	G	3	810	0	1302	4	1113	1	1009	22	3578	4	1562
chr11	3525720	C	32	13914	5	3334	82	19932	17	10015	56	17378	98	27601
chr11	3525722	G	3	811	12	1290	7	1111	8	1000	16	3586	5	1562
chr11	3525723	C	59	13921	33	3319	72	19996	16	10044	47	17435	64	27713
chr11	3525725	G	4	808	1	1298	0	1116	1	1009	4	3594	1	1565
chr11	3525726	C	41	8239	59	2013	94	11573	6	5939	60	10378	39	15560
chr11	3525729	C	216	13763	6	3342	146	19914	286	9769	209	17261	125	27619
chr11	3525731	C	25	13964	1	3351	40	20036	20	10042	24	17464	38	27750
chr11	3525732	C	32	13959	7	3345	54	20018	28	10033	37	17451	69	27717
chr11	3525733	C	39	13954	2	3351	29	20042	14	10049	41	17451	32	27757
chr11	3525734	C	30	13963	3	3350	34	20043	24	10040	35	17456	48	27740
chr11	3525735	C	45	13946	3	3347	28	20048	12	10051	22	17473	23	27759
chr11	3525738	G	3	299	16	488	5	352	0	409	16	1220	11	663
chr11	3525742	C	11396	2577	2969	370	16722	3311	8544	1507	14790	2705	23284	4440
chr11	3525743	G	165	30	296	49	166	51	210	73	734	132	346	108
chr11	3525744	C	107	13850	4	3341	68	19962	16	10013	86	17361	74	27613
chr11	3525746	C	21	13983	7	3348	20	20059	23	10041	48	17472	34	27757
chr11	3525753	C	4	3474	81	1454	21	4965	3	4196	7	4567	12	8763
chr11	3525755	C	9	3371	2	1519	6	4834	6	4109	5	4482	11	8525
chr11	3525756	C	2234	1077	1057	460	3178	1599	2787	1307	3119	1312	5879	2540
chr11	3525757	G	124	72	199	70	156	86	114	64	692	294	261	140
chr11	3525758	G	1	194	0	268	0	247	0	177	4	981	0	404

9142	13946	1851	3344	12751	20037	7009	10041	11489	17389	16969	27724
565	803	860	1286	723	1101	714	1000	2477	3555	961	1550
16	13948	3	3348	15	20041	5	10042	58	17399	23	27724
10841	13949	2630	3348	15661	20041	7112	10043	13959	17402	20753	27728
638	805	1108	1299	864	1109	758	1009	2945	3582	1033	1554
64	13951	3	3348	19	20037	9	10044	22	17409	24	27733
0	811	3	1301	5	1112	2	1010	18	3589	15	1559
2	811	11	1289	1	1113	0	1010	13	3590	3	1559
42	13958	5	3346	28	20045	12	10040	47	17424	45	27750
75	13963	34	3349	142	20051	5	10050	96	17432	23	27763
0	812	0	1300	5	1114	2	1008	11	3594	1	1561
1	812	1	1301	4	1114	3	1009	17	3596	1	1560
20	13965	3	3350	25	20054	12	10055	27	17441	37	27771
1	812	1	1301	1	1113	1	1010	6	3596	9	1563
2	812	1	1302	5	1114	2	1008	17	3595	1	1562
17	13974	7	3350	66	20063	12	10056	46	17457	49	27773
8	811	26	1298	14	1114	31	1010	52	3596	18	1564
20	13977	5	3350	24	20062	13	10059	40	17463	34	27782
67	13975	60	3349	250	20059	351	10056	212	17463	58	27775
3	814	1	1302	2	1114	1	1011	20	3601	21	1567
3	814	3	1302	1	1116	4	1011	11	3598	2	1567
8953	13981	2269	3351	12469	20067	6499	10058	11432	17474	17580	27777
591	812	971	1301	846	1116	755	1010	2756	3598	1208	1564
2	814	2	1302	7	1117	1	1011	6	3603	5	1567
3	813	0	1302	4	1117	1	1010	22	3600	4	1566
32	13946	5	3339	82	20014	17	10032	56	17434	98	27699
3	814	12	1302	7	1118	8	1008	16	3602	5	1567
59	13980	33	3352	72	20068	16	10060	47	17482	64	27777
4	812	1	1299	0	1116	1	1010	4	3598	1	1566
41	8280	59	2072	94	11667	6	5945	60	10438	39	15599
216	13979	6	3348	146	20060	286	10055	209	17470	125	27744
25	13989	1	3352	40	20076	20	10062	24	17488	38	27788
32	13991	7	3352	54	20072	28	10061	37	17488	69	27786
39	13993	2	3353	29	20071	14	10063	41	17492	32	27789
30	13993	3	3353	34	20077	24	10064	35	17491	48	27788
45	13991	3	3350	28	20076	12	10063	22	17495	23	27782
3	302	16	504	5	357	0	409	16	1236	11	674
11396	13973	2969	3339	16722	20033	8544	10051	14790	17495	23284	27724
165	195	296	345	166	217	210	283	734	866	346	454
107	13957	4	3345	68	20030	16	10029	86	17447	74	27687
21	14004	7	3355	20	20079	23	10064	48	17520	34	27791
4	3478	81	1535	21	4986	3	4199	7	4574	12	8775
9	3380	2	1521	6	4840	6	4115	5	4487	11	8536
2234	3311	1057	1517	3178	4777	2787	4094	3119	4431	5879	8419
124	196	199	269	156	242	114	178	692	986	261	401
1	195	0	268	0	247	0	177	4	985	0	404

chr11	3525759	G	1	194	1	272	0	253	0	181	2	1003	1	393
chr11	3525762	C	1	1417	0	994	1	2039	2	2104	1	1944	9	3953
chr11	3525765	G	0	353	2	429	1	429	1	379	1	1519	26	640
chr11	3525766	G	2	363	1	442	2	439	0	394	2	1562	0	680
chr11	3525768	G	4	371	15	443	2	456	1	411	15	1586	1	704
chr11	3525769	G	1	380	0	469	0	469	1	418	2	1634	0	710
chr11	3525770	G	2	384	1	489	1	483	0	441	7	1667	1	727
chr11	3525771	G	3	399	1	505	3	497	1	461	6	1739	2	756
chr11	3525774	C	96	13937	4	3366	156	19909	58	10012	100	17482	145	27664
chr11	3525776	G	1	640	23	794	9	791	0	782	19	2658	1	1208
chr11	3525777	C	79	13958	54	3316	148	19913	39	10047	135	17422	84	27727
chr11	3525779	C	53	14010	4	3370	61	20026	16	10076	42	17544	124	27730
chr11	3525780	C	22	14038	2	3372	65	20021	13	10079	24	17558	32	27812
chr11	3525781	C	9861	4197	2373	997	13857	6222	6357	3734	13048	4522	20150	7691
chr11	3525782	G	498	181	530	336	639	249	659	184	2212	665	1013	323
chr11	3525783	G	0	678	3	866	3	886	0	843	6	2869	1	1334
chr11	3525786	G	2	679	2	874	5	887	0	844	23	2850	19	1322
chr11	3525788	G	1	679	3	872	0	892	1	843	7	2864	1	1339
chr11	3525789	C	24	14022	5	3361	28	20047	13	10073	23	17532	32	27801
chr11	3525790	C	9843	4194	2383	985	13802	6261	7062	3025	12793	4755	19056	8770
chr11	3525791	G	510	168	745	131	664	235	686	163	2287	595	952	399
chr11	3525792	G	1	679	0	891	0	904	2	854	5	2900	2	1353
chr11	3525793	G	5	672	4	896	3	904	2	859	11	2903	11	1341
chr11	3525794	C	60	13958	3	3359	77	19969	7	10071	107	17400	23	27770
chr11	3525797	C	18	14006	14	3346	75	19973	21	10046	37	17507	74	27717
chr11	3525800	C	22	14004	24	3338	36	20015	17	10053	25	17518	38	27752
chr11	3525801	C	543	13480	58	3304	612	19442	1157	8912	648	16898	219	27571
chr11	3525803	C	157	13828	10	3343	122	19882	112	9928	211	17276	143	27573
chr11	3525805	C	74	13945	8	3353	81	19961	38	10025	37	17498	71	27705
chr11	3525807	C	23	13996	9	3353	63	19971	13	10051	33	17478	50	27725
chr11	3525809	G	3	654	2	895	1	906	0	859	12	2847	66	1268
chr11	3525812	C	38	13974	7	3352	118	19918	11	10054	22	17478	113	27647
chr11	3525816	G	3	650	3	894	2	901	1	858	10	2834	10	1318
chr11	3525817	C	25	13979	3	3354	183	19842	16	10047	54	17429	45	27709
chr11	3525819	C	28	13980	4	3353	41	19987	13	10053	28	17466	66	27702
chr11	3525820	C	28	13980	5	3353	62	19967	244	9820	33	17458	35	27729
chr11	3525821	C	10809	3193	2401	946	15328	4695	7124	2932	13817	3665	21719	6030
chr11	3525822	G	531	113	820	70	708	192	721	140	2273	551	1070	249
chr11	3525823	C	10646	3354	1959	1395	14503	5510	6874	3183	13174	4287	21140	6586
chr11	3525824	G	588	141	953	268	803	259	770	195	2644	653	1154	263
chr11	3525825	C	47	13960	59	3294	111	19915	23	10037	58	17425	505	27245
chr11	3525827	G	1	725	1	1214	12	1044	3	960	20	3253	68	1342
chr11	3525828	C	46	13957	5	3351	59	19966	22	10040	70	17402	50	27711
chr11	3525831	G	7	715	3	1212	14	1035	0	963	47	3220	29	1373
chr11	3525834	G	1	722	5	1207	3	1045	2	960	12	3251	2	1400
chr11	3525835	G	2	721	2	1209	1	1047	2	960	11	3252	2	1399

1	195	1	273	0	253	0	181	2	1005	1	394
1	1418	0	994	1	2040	2	2106	1	1945	9	3962
0	353	2	431	1	430	1	380	1	1520	26	666
2	365	1	443	2	441	0	394	2	1564	0	680
4	375	15	458	2	458	1	412	15	1601	1	705
1	381	0	469	0	469	1	419	2	1636	0	710
2	386	1	490	1	484	0	441	7	1674	1	728
3	402	1	506	3	500	1	462	6	1745	2	758
96	14033	4	3370	156	20065	58	10070	100	17582	145	27809
1	641	23	817	9	800	0	782	19	2677	1	1209
79	14037	54	3370	148	20061	39	10086	135	17557	84	27811
53	14063	4	3374	61	20087	16	10092	42	17586	124	27854
22	14060	2	3374	65	20086	13	10092	24	17582	32	27844
9861	14058	2373	3370	13857	20079	6357	10091	13048	17570	20150	27841
498	679	530	866	639	888	659	843	2212	2877	1013	1336
0	678	3	869	3	889	0	843	6	2875	1	1335
2	681	2	876	5	892	0	844	23	2873	19	1341
1	680	3	875	0	892	1	844	7	2871	1	1340
24	14046	5	3366	28	20075	13	10086	23	17555	32	27833
9843	14037	2383	3368	13802	20063	7062	10087	12793	17548	19056	27826
510	678	745	876	664	899	686	849	2287	2882	952	1351
1	680	0	891	0	904	2	856	5	2905	2	1355
5	677	4	900	3	907	2	861	11	2914	11	1352
60	14018	3	3362	77	20046	7	10078	107	17507	23	27793
18	14024	14	3360	75	20048	21	10067	37	17544	74	27791
22	14026	24	3362	36	20051	17	10070	25	17543	38	27790
543	14023	58	3362	612	20054	1157	10069	648	17546	219	27790
157	13985	10	3353	122	20004	112	10040	211	17487	143	27716
74	14019	8	3361	81	20042	38	10063	37	17535	71	27776
23	14019	9	3362	63	20034	13	10064	33	17511	50	27775
3	657	2	897	1	907	0	859	12	2859	66	1334
38	14012	7	3359	118	20036	11	10065	22	17500	113	27760
3	653	3	897	2	903	1	859	10	2844	10	1328
25	14004	3	3357	183	20025	16	10063	54	17483	45	27754
28	14008	4	3357	41	20028	13	10066	28	17494	66	27768
28	14008	5	3358	62	20029	244	10064	33	17491	35	27764
10809	14002	2401	3347	15328	20023	7124	10056	13817	17482	21719	27749
531	644	820	890	708	900	721	861	2273	2824	1070	1319
10646	14000	1959	3354	14503	20013	6874	10057	13174	17461	21140	27726
588	729	953	1221	803	1062	770	965	2644	3297	1154	1417
47	14007	59	3353	111	20026	23	10060	58	17483	505	27750
1	726	1	1215	12	1056	3	963	20	3273	68	1410
46	14003	5	3356	59	20025	22	10062	70	17472	50	27761
7	722	3	1215	14	1049	0	963	47	3267	29	1402
1	723	5	1212	3	1048	2	962	12	3263	2	1402
2	723	2	1211	1	1048	2	962	11	3263	2	1401

chr11	3525837	C	38	13967	6	3350	49	19967	13	10047	67	17400	44	27707
chr11	3525840	C	49	13956	4	3352	26	19986	9	10048	33	17434	74	27682
chr11	3525842	C	9886	4111	2519	831	14064	5935	7709	2332	12590	4865	19791	7942
chr11	3525843	G	562	156	939	269	783	260	748	213	2524	708	1079	313
chr11	3525844	G	2	715	0	1205	2	1040	0	960	2	3223	2	1392
chr11	3525846	C	36	13965	4	3351	69	19935	17	10036	44	17408	27	27720
chr11	3525848	G	13	699	27	1171	10	1031	20	938	52	3155	18	1369
chr11	3525849	C	50	13952	20	3333	70	19930	247	9805	122	17323	28	27710
chr11	3525852	G	1	710	1	1198	1	1038	0	959	11	3182	1	1383
chr11	3525853	G	0	711	2	1196	4	1033	3	956	5	3185	2	1382
chr11	3525854	C	12194	1791	3101	239	17106	2871	8555	1472	15661	1770	22403	5309
chr11	3525855	G	630	78	996	201	862	175	777	182	2826	360	1158	224
chr11	3525856	C	11663	2322	2839	504	16399	3567	8671	1357	14616	2800	22744	4957
chr11	3525857	G	615	91	1016	182	880	152	780	175	2729	435	1066	310
chr11	3525858	C	10297	3416	2753	505	14562	5000	7729	2076	13262	3774	21810	5286
chr11	3525859	G	568	135	866	327	818	213	694	265	2526	623	1066	309
chr11	3525860	G	2	701	5	1188	0	1029	0	958	6	3139	3	1372
chr11	3525861	G	0	703	0	1192	0	1028	1	957	2	3145	16	1357
chr11	3525862	G	3	700	1	1191	1	1027	2	955	9	3135	2	1371
chr11	3525863	G	0	703	1	1190	3	1025	1	954	7	3132	2	1370
chr11	3525865	C	32	13956	1	3353	41	19904	28	10014	39	17338	59	27625
chr11	3525866	C	37	13951	6	3348	31	19904	44	9996	34	17338	45	27639
chr11	3525867	C	10753	3211	2209	1135	15047	4863	7623	2387	13573	3777	20347	7264
chr11	3525868	G	552	144	998	188	766	255	731	222	2596	514	1017	345
chr11	3525870	G	0	694	3	1186	1	1019	3	948	2	3099	1	1362
chr11	3525872	C	82	13894	1	3353	109	19799	60	9980	122	17210	40	27609
chr11	3525874	G	1	692	2	1185	2	1011	2	949	6	3073	1	1357
chr11	3525876	C	8153	5171	1990	1251	11234	7752	6415	3187	10563	5893	14607	11794
chr11	3525877	G	472	218	739	446	658	347	670	280	2066	994	846	500
chr11	3525879	C	20	13933	6	3344	29	19808	18	10006	51	17203	25	27537
chr11	3525880	C	53	13905	7	3340	50	19787	10	10013	42	17223	38	27532
chr11	3525882	G	2	681	0	1184	3	993	22	924	6	3015	0	1331
chr11	3525884	C	28	13925	4	3342	31	19784	46	9967	54	17158	52	27494
chr11	3525887	C	9771	4162	2572	761	14057	5716	7023	2977	12664	4504	19751	7750
chr11	3525888	G	544	136	885	294	738	248	628	317	2299	679	941	384
chr11	3525890	C	5529	8394	1248	2083	7834	11933	4038	5944	7370	9775	10345	17162
chr11	3525891	G	288	388	584	594	395	585	465	477	1219	1721	568	749
chr11	3525893	C	10097	3790	2345	934	14339	5276	6261	3633	12677	4377	20222	7140
chr11	3525894	G	495	178	727	445	707	269	686	255	2284	634	1002	302
chr11	3525897	C	122	13713	1	3231	144	19398	59	9776	148	16868	317	26936
chr11	3525899	C	11	13766	3	3047	43	19443	95	9655	63	16896	8	27078
chr11	3525902	C	3	13773	0	2956	0	19504	0	9711	2	16962	2	27120
chr11	3525903	C	1	13775	0	2957	3	19499	2	9708	1	16961	5	27116
chr11	3525905	G	16	625	24	1114	15	914	18	889	52	2643	58	1164
chr11	3525907	G	1	628	1	1118	0	902	0	889	6	2631	0	1184
chr11	3525909	C	9	13370	1	2691	9	19019	4	8975	2	16626	6	25825

38	14005	6	3356	49	20016	13	10060	67	17467	44	27751
49	14005	4	3356	26	20012	9	10057	33	17467	74	27756
9886	13997	2519	3350	14064	19999	7709	10041	12590	17455	19791	27733
562	718	939	1208	783	1043	748	961	2524	3232	1079	1392
2	717	0	1205	2	1042	0	960	2	3225	2	1394
36	14001	4	3355	69	20004	17	10053	44	17452	27	27747
13	712	27	1198	10	1041	20	958	52	3207	18	1387
50	14002	20	3353	70	20000	247	10052	122	17445	28	27738
1	711	1	1199	1	1039	0	959	11	3193	1	1384
0	711	2	1198	4	1037	3	959	5	3190	2	1384
12194	13985	3101	3340	17106	19977	8555	10027	15661	17431	22403	27712
630	708	996	1197	862	1037	777	959	2826	3186	1158	1382
11663	13985	2839	3343	16399	19966	8671	10028	14616	17416	22744	27701
615	706	1016	1198	880	1032	780	955	2729	3164	1066	1376
10297	13713	2753	3258	14562	19562	7729	9805	13262	17036	21810	27096
568	703	866	1193	818	1031	694	959	2526	3149	1066	1375
2	703	5	1193	0	1029	0	958	6	3145	3	1375
0	703	0	1192	0	1028	1	958	2	3147	16	1373
3	703	1	1192	1	1028	2	957	9	3144	2	1373
0	703	1	1191	3	1028	1	955	7	3139	2	1372
32	13988	1	3354	41	19945	28	10042	39	17377	59	27684
37	13988	6	3354	31	19935	44	10040	34	17372	45	27684
10753	13964	2209	3344	15047	19910	7623	10010	13573	17350	20347	27611
552	696	998	1186	766	1021	731	953	2596	3110	1017	1362
0	694	3	1189	1	1020	3	951	2	3101	1	1363
82	13976	1	3354	109	19908	60	10040	122	17332	40	27649
1	693	2	1187	2	1013	2	951	6	3079	1	1358
8153	13324	1990	3241	11234	18986	6415	9602	10563	16456	14607	26401
472	690	739	1185	658	1005	670	950	2066	3060	846	1346
20	13953	6	3350	29	19837	18	10024	51	17254	25	27562
53	13958	7	3347	50	19837	10	10023	42	17265	38	27570
2	683	0	1184	3	996	22	946	6	3021	0	1331
28	13953	4	3346	31	19815	46	10013	54	17212	52	27546
9771	13933	2572	3333	14057	19773	7023	10000	12664	17168	19751	27501
544	680	885	1179	738	986	628	945	2299	2978	941	1325
5529	13923	1248	3331	7834	19767	4038	9982	7370	17145	10345	27507
288	676	584	1178	395	980	465	942	1219	2940	568	1317
10097	13887	2345	3279	14339	19615	6261	9894	12677	17054	20222	27362
495	673	727	1172	707	976	686	941	2284	2918	1002	1304
122	13835	1	3232	144	19542	59	9835	148	17016	317	27253
11	13777	3	3050	43	19486	95	9750	63	16959	8	27086
3	13776	0	2956	0	19504	0	9711	2	16964	2	27122
1	13776	0	2957	3	19502	2	9710	1	16962	5	27121
16	641	24	1138	15	929	18	907	52	2695	58	1222
1	629	1	1119	0	902	0	889	6	2637	0	1184
9	13379	1	2692	9	19028	4	8979	2	16628	6	25831

chr11	3525910	C	3	13212	1	2613	5	18862	2	8734	7	16495	15	25379
chr11	3525912	C	3	13078	0	2566	5	18722	1	8552	4	16385	14	25050
chr11	3525915	C	1	13016	1	2536	5	18637	2	8440	7	16304	15	24844
chr11	3525917	G	4	436	7	872	7	673	1	626	18	1963	11	837
chr11	3525918	G	0	439	6	856	0	675	2	610	2	1960	2	830
chr11	3525923	C	3	12573	0	2346	1	18135	1	7994	0	15860	4	23880
chr11	3525928	G	1	130	2	448	2	246	1	167	5	718	1	197
chr11	3525935	G	5	102	0	428	3	217	0	152	13	614	15	137
chr11	3525948	g	0	91	0	367	0	187	0	124	0	532	0	111
chr11	3525950	g	0	90	0	365	0	185	0	124	0	530	0	109
chr11	3525954	g	0	88	0	361	1	181	0	120	0	526	0	107
chr11	3525958	g	0	88	0	360	0	182	0	120	0	523	0	107
chr11	3525964	g	0	88	0	360	0	182	0	120	0	522	0	107
chr11	3525968	g	0	88	0	359	0	182	0	120	0	523	0	106
chr15	61868624	C	0	1069	1	1041	1	2587	1	1022	1	4374	2	3193
chr15	61868625	C	0	1082	1	1054	1	2612	0	1038	0	4434	1	3233
chr15	61868632	C	0	1121	1	1089	0	2694	0	1087	1	4557	0	3375
chr15	61868634	C	0	1131	0	1096	0	2711	0	1102	1	4575	1	3406
chr15	61868635	C	1	1136	0	1099	0	2746	0	1106	0	4630	1	3472
chr15	61868644	C	0	1166	0	1134	1	2868	0	1153	0	4922	0	3836
chr15	61868647	C	1	1170	0	1137	0	2877	0	1162	0	4941	2	3859
chr15	61868648	C	0	1173	0	1141	0	2879	0	1162	2	4947	0	3870
chr15	61868649	C	1109	158	1064	101	2749	272	1128	119	4673	374	3538	550
chr15	61868651	C	1	1398	6	1494	5	3021	29	1496	17	5030	4	4285
chr15	61868655	C	2	1405	16	1497	3	3041	0	1535	8	5071	5	4324
chr15	61868656	C	13	1394	0	1515	15	3029	1	1534	33	5048	15	4318
chr15	61868658	C	57	1355	89	1438	132	2918	30	1519	228	4875	195	4150
chr15	61868663	C	4	1419	4	1526	5	3072	4	1559	27	5132	22	4361
chr15	61868665	C	1	1428	3	1532	4	3088	3	1564	3	5188	5	4391
chr15	61868666	C	1195	234	1292	248	2621	476	1241	327	4445	755	3488	914
chr15	61868668	G	1	920	5	4960	1	2541	1	2990	0	3900	2	2592
chr15	61868672	C	13	1415	42	1500	4	3097	1	1574	13	5209	3	4409
chr15	61868673	C	2	1426	54	1488	7	3094	2	1573	8	5216	7	4404
chr15	61868674	C	2	1427	4	1538	8	3092	5	1570	18	5206	25	4389
chr15	61868676	C	5	1422	3	1538	3	3102	2	1573	11	5216	28	4386
chr15	61868679	G	0	986	1	5574	0	2686	1	3244	1	4199	1	2804
chr15	61868680	G	0	986	2	5580	0	2691	2	3245	0	4203	1	2808
chr15	61868683	C	3	1427	2	1541	3	3104	5	1570	10	5234	10	4403
chr15	61868685	G	0	995	2	5611	0	2701	0	3263	0	4215	1	2836
chr15	61868687	G	0	999	0	5613	0	2703	2	3266	0	4225	2	2852
chr15	61868689	C	2	1428	7	1536	2	3108	2	1574	9	5241	6	4411
chr15	61868692	C	12	1420	3	1541	13	3098	71	1504	27	5226	4	4413
chr15	61868693	C	6	1426	1	1543	4	3106	2	1573	14	5247	10	4410
chr15	61868695	C	1	1431	1	1543	12	3100	2	1572	32	5229	4	4414
chr15	61868698	C	1	1432	1	1544	21	3092	1	1576	8	5260	4	4415
chr15	61868702	C	12	1421	2	1539	24	3089	3	1577	37	5233	8	4411

3	13215	1	2614	5	18867	2	8736	7	16502	15	25394
3	13081	0	2566	5	18727	1	8553	4	16389	14	25064
1	13017	1	2537	5	18642	2	8442	7	16311	15	24859
4	440	7	879	7	680	1	627	18	1981	11	848
0	439	6	862	0	675	2	612	2	1962	2	832
3	12576	0	2346	1	18136	1	7995	0	15860	4	23884
1	131	2	450	2	248	1	168	5	723	1	198
5	107	0	428	3	220	0	152	13	627	15	152
0	91	0	367	0	187	0	124	0	532	0	111
0	90	0	365	0	185	0	124	0	530	0	109
0	88	0	361	1	182	0	120	0	526	0	107
0	88	0	360	0	182	0	120	0	523	0	107
0	88	0	360	0	182	0	120	0	522	0	107
0	88	0	359	0	182	0	120	0	523	0	106
0	1069	1	1042	1	2588	1	1023	1	4375	2	3195
0	1082	1	1055	1	2613	0	1038	0	4434	1	3234
0	1121	1	1090	0	2694	0	1087	1	4558	0	3375
0	1131	0	1096	0	2711	0	1102	1	4576	1	3407
1	1137	0	1099	0	2746	0	1106	0	4630	1	3473
0	1166	0	1134	1	2869	0	1153	0	4922	0	3836
1	1171	0	1137	0	2877	0	1162	0	4941	2	3861
0	1173	0	1141	0	2879	0	1162	2	4949	0	3870
1109	1267	1064	1165	2749	3021	1128	1247	4673	5047	3538	4088
1	1399	6	1500	5	3026	29	1525	17	5047	4	4289
2	1407	16	1513	3	3044	0	1535	8	5079	5	4329
13	1407	0	1515	15	3044	1	1535	33	5081	15	4333
57	1412	89	1527	132	3050	30	1549	228	5103	195	4345
4	1423	4	1530	5	3077	4	1563	27	5159	22	4383
1	1429	3	1535	4	3092	3	1567	3	5191	5	4396
1195	1429	1292	1540	2621	3097	1241	1568	4445	5200	3488	4402
1	921	5	4965	1	2542	1	2991	0	3900	2	2594
13	1428	42	1542	4	3101	1	1575	13	5222	3	4412
2	1428	54	1542	7	3101	2	1575	8	5224	7	4411
2	1429	4	1542	8	3100	5	1575	18	5224	25	4414
5	1427	3	1541	3	3105	2	1575	11	5227	28	4414
0	986	1	5575	0	2686	1	3245	1	4200	1	2805
0	986	2	5582	0	2691	2	3247	0	4203	1	2809
3	1430	2	1543	3	3107	5	1575	10	5244	10	4413
0	995	2	5613	0	2701	0	3263	0	4215	1	2837
0	999	0	5613	0	2703	2	3268	0	4225	2	2854
2	1430	7	1543	2	3110	2	1576	9	5250	6	4417
12	1432	3	1544	13	3111	71	1575	27	5253	4	4417
6	1432	1	1544	4	3110	2	1575	14	5261	10	4420
1	1432	1	1544	12	3112	2	1574	32	5261	4	4418
1	1433	1	1545	21	3113	1	1577	8	5268	4	4419
12	1433	2	1541	24	3113	3	1580	37	5270	8	4419

chr15	61868706	C	4	1435	6	1538	15	3128	2	1575	12	5288	6	4427
chr15	61868707	C	7	1436	1	1545	6	3151	2	1579	18	5292	13	4432
chr15	61868708	C	2	1441	25	1521	2	3155	3	1578	12	5305	10	4437
chr15	61868709	C	1	1442	4	1542	9	3150	6	1575	11	5308	8	4438
chr15	61868713	C	6	1441	4	1540	9	3158	1	1580	20	5331	7	4445
chr15	61868715	G	8	1009	6	5725	12	2708	5	3312	29	4252	5	2898
chr15	61868716	G	1	1014	6	5723	5	2714	3	3313	6	4277	1	2903
chr15	61868719	G	7	1010	6	5723	44	2677	11	3307	85	4195	92	2812
chr15	61868722	G	0	1016	12	5725	8	2715	16	3300	26	4259	3	2907
chr15	61868723	C	1	1458	0	1547	1	3170	18	1565	10	5393	4	4456
chr15	61868725	C	985	479	1031	520	2117	1070	1139	450	3855	1569	2954	1528
chr15	61868726	G	671	339	3359	2298	1936	745	2110	1169	2955	1275	1810	1070
chr15	61868729	G	2	1014	8	5735	4	2721	9	3315	9	4277	8	2901
chr15	61868730	C	5	1467	8	1546	20	3168	26	1566	26	5419	34	4458
chr15	61868731	C	1180	294	1285	268	2530	660	1276	316	4544	910	3492	1008
chr15	61868732	G	775	241	4005	1730	2133	590	2421	902	3399	888	2295	611
chr15	61868733	C	1068	407	1126	429	2335	855	1307	284	4205	1254	3129	1368
chr15	61868734	G	798	207	4094	1535	2166	519	2562	707	3443	794	2319	548
chr15	61868735	C	5	1471	4	1551	13	3178	4	1586	17	5451	15	4485
chr15	61868738	G	12	1006	5	5737	18	2709	126	3200	24	4269	2	2912
chr15	61868740	C	4	1474	0	1557	19	3177	2	1591	11	5465	8	4496
chr15	61868746	G	1	1018	6	5738	8	2719	1	3324	9	4290	4	2910
chr15	61868747	G	6	1016	8	5743	6	2732	7	3327	20	4297	19	2923
chr15	61868750	C	5	1480	6	1554	16	3187	4	1594	31	5470	14	4498
chr15	61868753	G	1	1022	8	5741	10	2728	4	3330	8	4308	5	2938
chr15	61868755	G	1	1021	7	5748	11	2729	6	3329	18	4297	16	2926
chr15	61868757	C	1164	324	1220	341	2497	708	1239	361	4430	1083	3461	1053
chr15	61868758	G	795	230	3844	1905	2130	609	2391	940	3416	894	2152	790
chr15	61868760	G	4	1021	3	5752	4	2735	32	3303	16	4299	7	2937
chr15	61868761	C	194	1292	279	1280	388	2811	223	1374	701	4808	456	4041
chr15	61868763	C	3	1485	2	1562	3	3209	5	1598	10	5521	10	4520
chr15	61868764	C	4	1485	1	1562	6	3207	4	1600	24	5507	7	4522
chr15	61868766	G	7	1018	234	5521	15	2725	0	3335	28	4292	85	2860
chr15	61868769	C	1277	210	1396	168	2724	485	1383	218	4742	783	3631	895
chr15	61868770	G	829	195	4122	1636	2280	460	2689	646	3561	759	2303	636
chr15	61868771	G	1	1024	7	5747	5	2734	7	3328	10	4311	4	2941
chr15	61868773	G	4	1021	18	5741	17	2723	30	3306	29	4291	10	2938
chr15	61868774	C	5	1483	38	1523	10	3201	2	1601	27	5499	44	4482
chr15	61868776	G	26	1000	141	5617	99	2647	105	3233	197	4130	86	2866
chr15	61868777	G	0	1027	7	5754	5	2740	9	3327	8	4319	28	2926
chr15	61868781	G	11	1016	2	5761	7	2739	5	3335	18	4309	19	2934
chr15	61868786	C	45	1443	83	1478	75	3137	50	1553	151	5378	106	4421
chr15	61868788	G	2	1026	15	5754	16	2734	8	3335	10	4328	11	2949
chr15	61868789	C	1309	178	1383	175	2863	347	1361	239	5030	498	3800	723
chr15	61868790	G	879	136	4841	831	2424	282	2944	353	3843	434	2476	423
chr15	61868795	G	0	1028	9	5763	17	2734	9	3337	14	4328	7	2957

4	1439	6	1544	15	3143	2	1577	12	5300	6	4433
7	1443	1	1546	6	3157	2	1581	18	5310	13	4445
2	1443	25	1546	2	3157	3	1581	12	5317	10	4447
1	1443	4	1546	9	3159	6	1581	11	5319	8	4446
6	1447	4	1544	9	3167	1	1581	20	5351	7	4452
8	1017	6	5731	12	2720	5	3317	29	4281	5	2903
1	1015	6	5729	5	2719	3	3316	6	4283	1	2904
7	1017	6	5729	44	2721	11	3318	85	4280	92	2904
0	1016	12	5737	8	2723	16	3316	26	4285	3	2910
1	1459	0	1547	1	3171	18	1583	10	5403	4	4460
985	1464	1031	1551	2117	3187	1139	1589	3855	5424	2954	4482
671	1010	3359	5657	1936	2681	2110	3279	2955	4230	1810	2880
2	1016	8	5743	4	2725	9	3324	9	4286	8	2909
5	1472	8	1554	20	3188	26	1592	26	5445	34	4492
1180	1474	1285	1553	2530	3190	1276	1592	4544	5454	3492	4500
775	1016	4005	5735	2133	2723	2421	3323	3399	4287	2295	2906
1068	1475	1126	1555	2335	3190	1307	1591	4205	5459	3129	4497
798	1005	4094	5629	2166	2685	2562	3269	3443	4237	2319	2867
5	1476	4	1555	13	3191	4	1590	17	5468	15	4500
12	1018	5	5742	18	2727	126	3326	24	4293	2	2914
4	1478	0	1557	19	3196	2	1593	11	5476	8	4504
1	1019	6	5744	8	2727	1	3325	9	4299	4	2914
6	1022	8	5751	6	2738	7	3334	20	4317	19	2942
5	1485	6	1560	16	3203	4	1598	31	5501	14	4512
1	1023	8	5749	10	2738	4	3334	8	4316	5	2943
1	1022	7	5755	11	2740	6	3335	18	4315	16	2942
1164	1488	1220	1561	2497	3205	1239	1600	4430	5513	3461	4514
795	1025	3844	5749	2130	2739	2391	3331	3416	4310	2152	2942
4	1025	3	5755	4	2739	32	3335	16	4315	7	2944
194	1486	279	1559	388	3199	223	1597	701	5509	456	4497
3	1488	2	1564	3	3212	5	1603	10	5531	10	4530
4	1489	1	1563	6	3213	4	1604	24	5531	7	4529
7	1025	234	5755	15	2740	0	3335	28	4320	85	2945
1277	1487	1396	1564	2724	3209	1383	1601	4742	5525	3631	4526
829	1024	4122	5758	2280	2740	2689	3335	3561	4320	2303	2939
1	1025	7	5754	5	2739	7	3335	10	4321	4	2945
4	1025	18	5759	17	2740	30	3336	29	4320	10	2948
5	1488	38	1561	10	3211	2	1603	27	5526	44	4526
26	1026	141	5758	99	2746	105	3338	197	4327	86	2952
0	1027	7	5761	5	2745	9	3336	8	4327	28	2954
11	1027	2	5763	7	2746	5	3340	18	4327	19	2953
45	1488	83	1561	75	3212	50	1603	151	5529	106	4527
2	1028	15	5769	16	2750	8	3343	10	4338	11	2960
1309	1487	1383	1558	2863	3210	1361	1600	5030	5528	3800	4523
879	1015	4841	5672	2424	2706	2944	3297	3843	4277	2476	2899
0	1028	9	5772	17	2751	9	3346	14	4342	7	2964

chr15	61868801	C	1054	431	1172	388	2309	900	1119	482	4150	1370	3406	1120
chr15	61868802	G	726	303	3521	2253	2023	732	2230	1119	3085	1263	2023	943
chr15	61868804	G	1	1030	6	5769	1	2756	3	3346	7	4344	3	2967
chr15	61868805	G	2	1028	15	5759	11	2746	4	3347	7	4348	5	2967
chr15	61868810	C	1283	202	1443	116	2854	354	1327	275	4964	555	3554	960
chr15	61868811	G	883	148	4510	1266	2345	418	2990	362	3770	588	2613	365
chr15	61868812	C	1169	314	1334	225	2458	747	1230	374	4485	1035	3506	1008
chr15	61868813	G	757	278	3957	1820	2106	656	2720	632	3266	1096	2249	730
chr15	61868815	G	7	1029	6	5773	20	2745	1	3352	8	4357	1	2982
chr15	61868816	C	3	1467	3	1545	6	3164	6	1567	24	5430	10	4470
chr15	61868817	C	5	1478	1	1557	8	3198	2	1601	19	5501	10	4511
chr15	61868821	C	23	1460	30	1528	19	3185	68	1533	41	5476	50	4468
chr15	61868823	C	140	1341	135	1423	286	2918	193	1409	573	4946	278	4244
chr15	61868825	C	4	1477	3	1555	8	3196	1	1601	16	5502	32	4485
chr15	61868826	C	8	1475	2	1556	15	3188	1	1601	37	5481	7	4513
chr15	61868828	G	0	180	0	727	1	328	1	344	3	643	1	574
chr15	61868829	C	4	1477	5	1551	8	3196	5	1598	18	5503	6	4515
chr15	61868830	C	6	1477	3	1554	8	3195	2	1600	19	5504	10	4512
chr15	61868833	C	1105	374	1233	316	2345	851	1097	499	4142	1374	3411	1100
chr15	61868834	G	143	58	467	357	249	115	270	124	496	205	387	228
chr15	61868837	C	2	1481	3	1554	9	3196	5	1596	15	5508	10	4511
chr15	61868841	G	1	1033	5	5768	14	2747	8	3332	15	4336	6	2951
chr15	61868842	C	16	1468	3	1553	22	3182	3	1598	48	5475	2	4516
chr15	61868844	G	0	1032	9	5766	6	2753	24	3313	13	4333	2	2953
chr15	61868846	G	5	1027	13	5763	13	2745	9	3327	11	4338	13	2942
chr15	61868847	G	2	1028	14	5760	6	2751	5	3328	7	4338	3	2946
chr15	61868848	C	3	1477	4	1552	3	3195	5	1594	21	5484	9	4500
chr15	61868853	G	7	1013	97	5659	23	2725	4	3313	37	4273	3	2901
chr15	61868854	C	41	1440	127	1428	52	3150	112	1485	161	5349	26	4486
chr15	61868856	C	8	1472	0	1555	6	3197	3	1592	22	5491	50	4459
chr15	61868861	G	3	997	1	5718	1	2731	1	3287	5	4261	0	2865
chr15	61868863	G	4	1003	11	5725	5	2735	2	3294	21	4262	7	2869
chr15	61868865	G	3	1004	9	5725	16	2724	4	3292	17	4260	4	2872
chr15	61868866	G	2	1005	10	5720	17	2722	6	3287	12	4263	5	2870
chr15	61868870	G	4	999	9	5711	6	2728	4	3285	30	4233	5	2858
chr15	61868872	G	57	947	306	5413	176	2560	280	3005	153	4112	96	2767
chr15	61868878	C	4	1467	2	1529	5	3190	1	1541	16	5472	12	4435
chr15	61868879	C	1	1470	2	1521	6	3188	1	1532	16	5467	5	4435
chr15	61868880	C	5	1464	48	1465	19	3174	14	1505	27	5453	5	4417
chr15	61868882	G	7	994	32	5679	38	2683	6	3277	56	4169	1	2834
chr15	61868885	C	1061	363	1001	431	2269	895	958	402	4096	1349	2589	1647
chr15	61868886	G	637	364	3358	2348	1941	779	2336	945	2894	1324	1775	1067
chr15	61868887	G	3	997	8	5700	4	2718	8	3274	14	4205	6	2833
chr15	61868889	C	0	1224	0	1379	0	2513	0	1116	0	4982	0	3448
chr15	61868890	C	1	1222	0	1381	0	2516	0	1117	0	4983	1	3448
chr15	61868893	G	1	998	9	5693	6	2708	3	3277	11	4198	6	2828

1054	1485	1172	1560	2309	3209	1119	1601	4150	5520	3406	4526
726	1029	3521	5774	2023	2755	2230	3349	3085	4348	2023	2966
1	1031	6	5775	1	2757	3	3349	7	4351	3	2970
2	1030	15	5774	11	2757	4	3351	7	4355	5	2972
1283	1485	1443	1559	2854	3208	1327	1602	4964	5519	3554	4514
883	1031	4510	5776	2345	2763	2990	3352	3770	4358	2613	2978
1169	1483	1334	1559	2458	3205	1230	1604	4485	5520	3506	4514
757	1035	3957	5777	2106	2762	2720	3352	3266	4362	2249	2979
7	1036	6	5779	20	2765	1	3353	8	4365	1	2983
3	1470	3	1548	6	3170	6	1573	24	5454	10	4480
5	1483	1	1558	8	3206	2	1603	19	5520	10	4521
23	1483	30	1558	19	3204	68	1601	41	5517	50	4518
140	1481	135	1558	286	3204	193	1602	573	5519	278	4522
4	1481	3	1558	8	3204	1	1602	16	5518	32	4517
8	1483	2	1558	15	3203	1	1602	37	5518	7	4520
0	180	0	727	1	329	1	345	3	646	1	575
4	1481	5	1556	8	3204	5	1603	18	5521	6	4521
6	1483	3	1557	8	3203	2	1602	19	5523	10	4522
1105	1479	1233	1549	2345	3196	1097	1596	4142	5516	3411	4511
143	201	467	824	249	364	270	394	496	701	387	615
2	1483	3	1557	9	3205	5	1601	15	5523	10	4521
1	1034	5	5773	14	2761	8	3340	15	4351	6	2957
16	1484	3	1556	22	3204	3	1601	48	5523	2	4518
0	1032	9	5775	6	2759	24	3337	13	4346	2	2955
5	1032	13	5776	13	2758	9	3336	11	4349	13	2955
2	1030	14	5774	6	2757	5	3333	7	4345	3	2949
3	1480	4	1556	3	3198	5	1599	21	5505	9	4509
7	1020	97	5756	23	2748	4	3317	37	4310	3	2904
41	1481	127	1555	52	3202	112	1597	161	5510	26	4512
8	1480	0	1555	6	3203	3	1595	22	5513	50	4509
3	1000	1	5719	1	2732	1	3288	5	4266	0	2865
4	1007	11	5736	5	2740	2	3296	21	4283	7	2876
3	1007	9	5734	16	2740	4	3296	17	4277	4	2876
2	1007	10	5730	17	2739	6	3293	12	4275	5	2875
4	1003	9	5720	6	2734	4	3289	30	4263	5	2863
57	1004	306	5719	176	2736	280	3285	153	4265	96	2863
4	1471	2	1531	5	3195	1	1542	16	5488	12	4447
1	1471	2	1523	6	3194	1	1533	16	5483	5	4440
5	1469	48	1513	19	3193	14	1519	27	5480	5	4422
7	1001	32	5711	38	2721	6	3283	56	4225	1	2835
1061	1424	1001	1432	2269	3164	958	1360	4096	5445	2589	4236
637	1001	3358	5706	1941	2720	2336	3281	2894	4218	1775	2842
3	1000	8	5708	4	2722	8	3282	14	4219	6	2839
0	1224	0	1379	0	2513	0	1116	0	4982	0	3448
1	1223	0	1381	0	2516	0	1117	0	4983	1	3449
1	999	9	5702	6	2714	3	3280	11	4209	6	2834

chr15	61868897	G	0	977	3	5675	20	2680	30	3224	43	4120	16	2788
chr15	61868898	G	6	977	5	5696	6	2697	7	3265	12	4171	9	2808
chr15	61868900	G	2	975	13	5686	17	2677	4	3267	22	4146	8	2795
chr15	61868901	G	0	967	11	5686	4	2652	5	3261	7	4149	5	2771
chr15	61868904	G	0	956	35	5656	8	2626	4	3254	12	4119	18	2734
chr15	61868905	C	1	1110	0	1254	0	2280	0	964	1	4503	1	3029
chr15	61868907	G	17	928	160	5512	95	2501	26	3208	121	3950	98	2600
chr15	61868908	C	1	1092	0	1241	0	2261	0	956	1	4481	2	2999
chr15	61868910	G	2	949	3	5684	7	2622	6	3234	11	4086	34	2703
chr15	61868912	C	0	1026	1	1150	0	2090	2	879	0	4177	1	2774
chr15	61868914	G	2	938	6	5671	4	2587	7	3226	14	4042	12	2684
chr15	61868915	G	3	936	4	5674	9	2581	9	3222	8	4047	3	2694
chr15	61868917	G	15	925	3	5674	40	2546	136	3096	67	3984	17	2677
chr15	61868918	G	1	938	4	5670	2	2578	4	3228	4	4042	9	2681
chr15	61868929	G	1	937	12	5657	8	2573	4	3224	4	4038	13	2676
chr15	61868933	G	3	934	4	5663	5	2575	8	3215	6	4026	7	2668
chr15	61868935	G	2	934	5	5664	8	2572	9	3218	14	4026	4	2680
chr15	61868939	G	2	933	3	5658	6	2572	2	3219	13	4016	5	2665
chr15	61868944	G	3	932	8	5651	2	2572	5	3215	11	4014	8	2658
chr15	61868948	G	662	272	3603	2056	2016	557	2318	902	3121	903	1942	727
chr15	61868961	G	0	926	0	5409	0	2567	1	3196	0	4006	1	2646
chr15	61868969	G	0	918	0	5352	0	2552	0	3177	0	3968	0	2619
chr15	61868972	G	1	913	0	5295	0	2534	2	3143	0	3944	1	2597
chr15	61868975	G	1	908	0	5274	0	2523	1	3123	1	3920	1	2583
chr15	61868976	G	0	909	0	5268	0	2522	1	3106	0	3906	0	2560
chr15	61868979	G	0	906	1	5261	0	2513	0	3107	0	3898	2	2554
chr15	61868980	G	0	907	1	5254	0	2512	0	3100	0	3899	0	2551
chr15	100299982	C	0	160	0	25	0	810	0	62	1	983	0	97
chr15	100299985	C	0	186	0	27	0	1000	0	79	0	1264	0	124
chr15	100299986	C	0	188	0	28	0	1003	0	79	1	1262	0	128
chr15	100299987	C	0	188	0	28	0	1004	0	79	0	1269	0	129
chr15	100299991	C	0	196	0	31	0	1025	0	86	0	1279	0	136
chr15	100299992	C	0	197	0	31	0	1030	0	90	1	1281	0	144
chr15	100299993	C	0	198	0	31	0	1031	0	91	0	1285	0	143
chr15	100299995	G	2	3170	0	3726	1	2526	2	4108	4	10683	2	5355
chr15	100299997	G	1	3177	1	3735	0	2528	1	4118	1	10692	0	5360
chr15	100299998	C	0	243	0	37	1	1231	0	144	0	1522	0	344
chr15	100300001	G	1	3176	0	3730	0	2528	0	4120	1	10688	3	5352
chr15	100300002	G	0	3179	1	3735	0	2528	0	4120	1	10695	1	5361
chr15	100300003	G	1	3177	1	3732	0	2527	0	4116	3	10687	0	5357
chr15	100300008	C	0	261	0	44	1	1268	0	182	1	1528	0	402
chr15	100300010	G	2	3180	4	3784	2	2525	8	4119	24	10676	12	5352
chr15	100300013	G	2	3180	13	3776	3	2526	4	4123	10	10690	11	5353
chr15	100300015	G	7	3176	10	3777	4	2522	7	4120	18	10682	5	5357
chr15	100300018	C	0	287	1	63	2	1293	0	210	3	1534	0	423
chr15	100300020	G	9	3181	1	3793	11	2522	94	4044	44	10675	5	5366

0	977	3	5678	20	2700	30	3254	43	4163	16	2804
6	983	5	5701	6	2703	7	3272	12	4183	9	2817
2	977	13	5699	17	2694	4	3271	22	4168	8	2803
0	967	11	5697	4	2656	5	3266	7	4156	5	2776
0	956	35	5691	8	2634	4	3258	12	4131	18	2752
1	1111	0	1254	0	2280	0	964	1	4504	1	3030
17	945	160	5672	95	2596	26	3234	121	4071	98	2698
1	1093	0	1241	0	2261	0	956	1	4482	2	3001
2	951	3	5687	7	2629	6	3240	11	4097	34	2737
0	1026	1	1151	0	2090	2	881	0	4177	1	2775
2	940	6	5677	4	2591	7	3233	14	4056	12	2696
3	939	4	5678	9	2590	9	3231	8	4055	3	2697
15	940	3	5677	40	2586	136	3232	67	4051	17	2694
1	939	4	5674	2	2580	4	3232	4	4046	9	2690
1	938	12	5669	8	2581	4	3228	4	4042	13	2689
3	937	4	5667	5	2580	8	3223	6	4032	7	2675
2	936	5	5669	8	2580	9	3227	14	4040	4	2684
2	935	3	5661	6	2578	2	3221	13	4029	5	2670
3	935	8	5659	2	2574	5	3220	11	4025	8	2666
662	934	3603	5659	2016	2573	2318	3220	3121	4024	1942	2669
0	926	0	5409	0	2567	1	3197	0	4006	1	2647
0	918	0	5352	0	2552	0	3177	0	3968	0	2619
1	914	0	5295	0	2534	2	3145	0	3944	1	2598
1	909	0	5274	0	2523	1	3124	1	3921	1	2584
0	909	0	5268	0	2522	1	3107	0	3906	0	2560
0	906	1	5262	0	2513	0	3107	0	3898	2	2556
0	907	1	5255	0	2512	0	3100	0	3899	0	2551
0	160	0	25	0	810	0	62	1	984	0	97
0	186	0	27	0	1000	0	79	0	1264	0	124
0	188	0	28	0	1003	0	79	1	1263	0	128
0	188	0	28	0	1004	0	79	0	1269	0	129
0	196	0	31	0	1025	0	86	0	1279	0	136
0	197	0	31	0	1030	0	90	1	1282	0	144
0	198	0	31	0	1031	0	91	0	1285	0	143
2	3172	0	3726	1	2527	2	4110	4	10687	2	5357
1	3178	1	3736	0	2528	1	4119	1	10693	0	5360
0	243	0	37	1	1232	0	144	0	1522	0	344
1	3177	0	3730	0	2528	0	4120	1	10689	3	5355
0	3179	1	3736	0	2528	0	4120	1	10696	1	5362
1	3178	1	3733	0	2527	0	4116	3	10690	0	5357
0	261	0	44	1	1269	0	182	1	1529	0	402
2	3182	4	3788	2	2527	8	4127	24	10700	12	5364
2	3182	13	3789	3	2529	4	4127	10	10700	11	5364
7	3183	10	3787	4	2526	7	4127	18	10700	5	5362
0	287	1	64	2	1295	0	210	3	1537	0	423
9	3190	1	3794	11	2533	94	4138	44	10719	5	5371

chr15	100300021	G	6	3182	8	3787	12	2519	9	4129	48	10669	17	5349
chr15	100300022	C	1	286	0	63	1	1295	0	210	6	1530	0	423
chr15	100300026	C	69	217	6	58	262	1035	43	167	399	1143	78	345
chr15	100300027	G	699	2489	458	3333	536	1992	761	3377	2265	8445	949	4419
chr15	100300028	C	0	286	0	64	4	1290	0	209	2	1535	1	422
chr15	100300030	C	2	284	0	64	1	1296	0	209	1	1540	1	422
chr15	100300032	C	0	287	0	64	0	1298	0	210	0	1541	0	423
chr15	100300034	G	16	3175	22	3775	6	2528	4	4151	22	10722	8	5374
chr15	100300038	C	0	287	0	64	2	1297	0	210	4	1541	7	416
chr15	100300039	C	91	196	2	62	294	1004	43	167	384	1160	125	298
chr15	100300040	G	921	2270	873	2924	835	1702	1172	2984	3384	7361	1467	3917
chr15	100300044	G	6	3187	2	3797	4	2533	6	4151	12	10743	7	5377
chr15	100300049	C	1	286	0	64	1	1298	1	209	2	1544	0	422
chr15	100300050	C	68	219	11	53	344	958	100	110	300	1247	93	330
chr15	100300051	G	664	2523	533	3248	573	1959	800	3350	2605	8139	875	4503
chr15	100300053	G	5	3194	8	3798	3	2544	1	4165	19	10756	1	5394
chr15	100300054	G	2	3196	8	3798	5	2542	11	4156	21	10758	6	5389
chr15	100300055	G	8	3191	6	3798	11	2537	6	4160	17	10761	14	5383
chr15	100300056	G	16	3182	14	3792	6	2541	9	4157	25	10753	12	5383
chr15	100300058	C	0	287	0	64	0	1303	0	210	3	1547	0	423
chr15	100300059	C	2	285	0	64	1	1302	0	210	2	1549	1	422
chr15	100300062	C	172	115	33	31	595	708	121	89	817	734	110	313
chr15	100300063	G	1694	1510	1537	2270	1436	1112	2281	1896	5919	4868	2776	2629
chr15	100300066	C	2	285	0	64	1	1302	0	210	4	1547	0	423
chr15	100300067	C	111	176	13	51	396	907	53	157	467	1084	92	331
chr15	100300068	G	963	2241	1270	2538	815	1736	980	3198	3804	6985	989	4419
chr15	100300072	G	4	3202	2	3809	9	2542	1	4179	16	10775	7	5405
chr15	100300073	G	5	3201	4	3809	2	2548	5	4175	37	10752	7	5404
chr15	100300074	C	0	285	0	64	9	1282	1	207	6	1540	0	420
chr15	100300077	C	0	287	0	64	0	1303	1	208	3	1549	0	423
chr15	100300080	C	1	286	0	64	8	1290	0	208	12	1529	0	417
chr15	100300082	C	0	287	0	64	1	1303	0	209	2	1550	0	423
chr15	100300083	C	0	287	0	64	2	1303	0	210	1	1552	2	421
chr15	100300086	C	177	110	35	29	716	589	104	106	907	646	146	277
chr15	100300087	G	1963	1248	1670	2148	1602	948	2652	1531	6773	4023	2692	2730
chr15	100300088	C	0	286	0	64	2	1302	0	210	6	1546	3	420
chr15	100300090	G	6	3206	33	3787	9	2544	7	4178	19	10784	11	5410
chr15	100300094	G	5	3203	8	3811	5	2548	9	4177	31	10774	10	5412
chr15	100300096	C	1	286	0	64	1	1304	0	210	3	1551	0	423
chr15	100300098	C	0	287	0	64	3	1302	0	210	2	1553	1	422
chr15	100300102	G	4	3208	3	3820	1	2554	3	4184	16	10795	14	5410
chr15	100300103	G	11	3203	3	3817	3	2553	5	4181	23	10785	12	5415
chr15	100300105	C	0	287	0	64	3	1302	0	211	3	1551	4	419
chr15	100300108	C	154	132	18	46	720	582	42	169	864	691	135	288
chr15	100300109	G	1835	1378	1786	2038	1486	1071	2543	1649	6194	4619	2612	2816
chr15	100300112	G	6	3209	4	3823	3	2554	15	4179	25	10790	8	5423

6	3188	8	3795	12	2531	9	4138	48	10717	17	5366
1	287	0	63	1	1296	0	210	6	1536	0	423
69	286	6	64	262	1297	43	210	399	1542	78	423
699	3188	458	3791	536	2528	761	4138	2265	10710	949	5368
0	286	0	64	4	1294	0	209	2	1537	1	423
2	286	0	64	1	1297	0	209	1	1541	1	423
0	287	0	64	0	1298	0	210	0	1541	0	423
16	3191	22	3797	6	2534	4	4155	22	10744	8	5382
0	287	0	64	2	1299	0	210	4	1545	7	423
91	287	2	64	294	1298	43	210	384	1544	125	423
921	3191	873	3797	835	2537	1172	4156	3384	10745	1467	5384
6	3193	2	3799	4	2537	6	4157	12	10755	7	5384
1	287	0	64	1	1299	1	210	2	1546	0	422
68	287	11	64	344	1302	100	210	300	1547	93	423
664	3187	533	3781	573	2532	800	4150	2605	10744	875	5378
5	3199	8	3806	3	2547	1	4166	19	10775	1	5395
2	3198	8	3806	5	2547	11	4167	21	10779	6	5395
8	3199	6	3804	11	2548	6	4166	17	10778	14	5397
16	3198	14	3806	6	2547	9	4166	25	10778	12	5395
0	287	0	64	0	1303	0	210	3	1550	0	423
2	287	0	64	1	1303	0	210	2	1551	1	423
172	287	33	64	595	1303	121	210	817	1551	110	423
1694	3204	1537	3807	1436	2548	2281	4177	5919	10787	2776	5405
2	287	0	64	1	1303	0	210	4	1551	0	423
111	287	13	64	396	1303	53	210	467	1551	92	423
963	3204	1270	3808	815	2551	980	4178	3804	10789	989	5408
4	3206	2	3811	9	2551	1	4180	16	10791	7	5412
5	3206	4	3813	2	2550	5	4180	37	10789	7	5411
0	285	0	64	9	1291	1	208	6	1546	0	420
0	287	0	64	0	1303	1	209	3	1552	0	423
1	287	0	64	8	1298	0	208	12	1541	0	417
0	287	0	64	1	1304	0	209	2	1552	0	423
0	287	0	64	2	1305	0	210	1	1553	2	423
177	287	35	64	716	1305	104	210	907	1553	146	423
1963	3211	1670	3818	1602	2550	2652	4183	6773	10796	2692	5422
0	286	0	64	2	1304	0	210	6	1552	3	423
6	3212	33	3820	9	2553	7	4185	19	10803	11	5421
5	3208	8	3819	5	2553	9	4186	31	10805	10	5422
1	287	0	64	1	1305	0	210	3	1554	0	423
0	287	0	64	3	1305	0	210	2	1555	1	423
4	3212	3	3823	1	2555	3	4187	16	10811	14	5424
11	3214	3	3820	3	2556	5	4186	23	10808	12	5427
0	287	0	64	3	1305	0	211	3	1554	4	423
154	286	18	64	720	1302	42	211	864	1555	135	423
1835	3213	1786	3824	1486	2557	2543	4192	6194	10813	2612	5428
6	3215	4	3827	3	2557	15	4194	25	10815	8	5431

chr15	100300115	G	8	3206	9	3816	6	2550	9	4183	20	10792	10	5421
chr15	100300116	G	7	3206	6	3823	14	2543	6	4186	14	10803	9	5419
chr15	100300118	C	1	286	0	64	4	1305	0	211	7	1550	1	422
chr15	100300121	C	0	287	0	64	3	1305	0	211	10	1547	0	423
chr15	100300125	G	13	3209	6	3822	11	2546	6	4192	32	10801	22	5417
chr15	100300126	C	2	284	1	63	2	1305	1	210	2	1553	0	422
chr15	100300129	C	4	283	0	64	30	1279	0	210	39	1519	0	424
chr15	100300131	C	1	283	0	61	4	1286	0	209	6	1539	1	408
chr15	100300132	C	1	286	0	64	2	1307	0	211	0	1552	0	424
chr15	100300136	C	0	110	0	39	1	400	0	134	0	379	1	302
chr15	100300138	C	0	110	0	39	0	398	0	134	1	375	1	297
chr15	100300139	C	81	29	19	19	297	98	114	19	294	82	208	89
chr15	100300140	G	113	56	83	57	138	53	105	69	387	117	132	92
chr15	100300143	G	2	161	0	130	1	192	0	159	1	504	0	213
chr15	100300144	G	0	162	0	130	0	192	0	157	0	506	1	213
chr15	100300145	C	71	16	17	15	217	68	77	31	235	52	160	74
chr15	100300146	G	124	38	73	57	135	60	124	33	408	102	153	65
chr15	100300147	G	1	164	0	133	0	196	0	158	2	509	1	218
chr15	100300149	C	40	20	7	22	107	69	45	25	83	62	59	38
chr15	100300150	G	93	62	63	84	93	95	103	53	318	197	99	113
chr15	100300152	G	1	155	0	202	0	186	0	157	1	516	0	224
chr15	100300155	G	1	168	1	311	1	189	0	162	3	523	2	225
chr15	100300161	G	0	322	2	867	0	249	1	501	0	616	1	350
chr15	100300171	G	210	145	477	455	164	128	304	309	516	311	315	280
chr15	100300173	G	1	378	1	957	2	300	1	663	2	861	0	635
chr15	100300174	G	0	385	5	964	0	304	0	685	5	871	1	650
chr15	100300176	G	25	457	35	1156	8	322	27	811	43	959	9	793
chr15	100300182	G	4	794	5	1528	1	548	1	1218	4	2434	4	1385
chr15	100300187	G	5	3206	5	3833	5	2546	10	4181	14	10839	14	5417
chr15	100300189	G	12	3196	37	3789	12	2524	3	4184	46	10786	14	5392
chr15	100300191	G	2106	1069	2109	1729	1573	874	3006	1179	7310	3465	3037	2355
chr15	100300198	G	2153	1014	2167	1666	1724	719	2855	1330	7512	3256	3153	2230
chr15	100300199	G	5	3167	7	3828	4	2440	4	4183	25	10745	3	5382
chr15	100300203	G	2352	817	2359	1473	1862	581	3068	1116	8129	2636	3563	1819
chr15	100300211	G	1246	1916	1285	2544	900	1538	1240	2932	3643	7087	1896	3475
chr15	100300218	G	2	3161	75	3757	9	2430	110	4065	47	10686	7	5365
chr15	100300219	G	7	3157	10	3821	8	2431	8	4170	23	10710	7	5366
chr15	100300220	G	7	3156	3	3825	10	2429	11	4164	30	10700	11	5362
chr15	100300221	G	8	3155	12	3818	11	2426	7	4168	37	10694	34	5338
chr15	100300223	G	1480	1681	1584	2245	1144	1293	1851	2327	4773	5962	2151	3221
chr15	100300235	G	1820	1332	1880	1943	1490	946	2470	1701	6445	4271	2896	2467
chr15	100300243	G	11	3141	5	3816	8	2425	34	4133	26	10668	4	5357
chr15	100300263	G	2	3116	3	3801	0	2412	3	4120	35	10554	3	5320
chr15	100300267	G	5	3125	7	3817	6	2412	3	4149	13	10629	10	5337
chr15	100300268	G	4	3124	10	3814	7	2414	13	4140	23	10615	6	5337
chr15	100300274	G	1951	1178	1393	2431	1660	757	2717	1436	7094	3543	2895	2449

8	3214	9	3825	6	2556	9	4192	20	10812	10	5431
7	3213	6	3829	14	2557	6	4192	14	10817	9	5428
1	287	0	64	4	1309	0	211	7	1557	1	423
0	287	0	64	3	1308	0	211	10	1557	0	423
13	3222	6	3828	11	2557	6	4198	32	10833	22	5439
2	286	1	64	2	1307	1	211	2	1555	0	422
4	287	0	64	30	1309	0	210	39	1558	0	424
1	284	0	61	4	1290	0	209	6	1545	1	409
1	287	0	64	2	1309	0	211	0	1552	0	424
0	110	0	39	1	401	0	134	0	379	1	303
0	110	0	39	0	398	0	134	1	376	1	298
81	110	19	38	297	395	114	133	294	376	208	297
113	169	83	140	138	191	105	174	387	504	132	224
2	163	0	130	1	193	0	159	1	505	0	213
0	162	0	130	0	192	0	157	0	506	1	214
71	87	17	32	217	285	77	108	235	287	160	234
124	162	73	130	135	195	124	157	408	510	153	218
1	165	0	133	0	196	0	158	2	511	1	219
40	60	7	29	107	176	45	70	83	145	59	97
93	155	63	147	93	188	103	156	318	515	99	212
1	156	0	202	0	186	0	157	1	517	0	224
1	169	1	312	1	190	0	162	3	526	2	227
0	322	2	869	0	249	1	502	0	616	1	351
210	355	477	932	164	292	304	613	516	827	315	595
1	379	1	958	2	302	1	664	2	863	0	635
0	385	5	969	0	304	0	685	5	876	1	651
25	482	35	1191	8	330	27	838	43	1002	9	802
4	798	5	1533	1	549	1	1219	4	2438	4	1389
5	3211	5	3838	5	2551	10	4191	14	10853	14	5431
12	3208	37	3826	12	2536	3	4187	46	10832	14	5406
2106	3175	2109	3838	1573	2447	3006	4185	7310	10775	3037	5392
2153	3167	2167	3833	1724	2443	2855	4185	7512	10768	3153	5383
5	3172	7	3835	4	2444	4	4187	25	10770	3	5385
2352	3169	2359	3832	1862	2443	3068	4184	8129	10765	3563	5382
1246	3162	1285	3829	900	2438	1240	4172	3643	10730	1896	5371
2	3163	75	3832	9	2439	110	4175	47	10733	7	5372
7	3164	10	3831	8	2439	8	4178	23	10733	7	5373
7	3163	3	3828	10	2439	11	4175	30	10730	11	5373
8	3163	12	3830	11	2437	7	4175	37	10731	34	5372
1480	3161	1584	3829	1144	2437	1851	4178	4773	10735	2151	5372
1820	3152	1880	3823	1490	2436	2470	4171	6445	10716	2896	5363
11	3152	5	3821	8	2433	34	4167	26	10694	4	5361
2	3118	3	3804	0	2412	3	4123	35	10589	3	5323
5	3130	7	3824	6	2418	3	4152	13	10642	10	5347
4	3128	10	3824	7	2421	13	4153	23	10638	6	5343
1951	3129	1393	3824	1660	2417	2717	4153	7094	10637	2895	5344

chr15	100300279	C	0	27	0	35	0	75	0	54	0	141	1	72
chr15	100300283	G	1380	1739	970	2838	1251	1159	1518	2615	5550	5027	1835	3466
chr15	100300285	G	25	3100	8	3815	3	2408	26	4118	29	10570	9	5326
chr15	100300288	G	11	3062	16	3758	10	2367	59	4017	25	10399	10	5236
chr15	100300289	C	1	31	0	37	0	93	0	74	0	167	0	110
chr15	100300290	C	0	32	0	37	0	94	0	76	0	168	0	111
chr15	100300291	C	28	6	12	25	67	28	51	26	126	45	91	21
chr15	100300292	G	2450	672	2451	1369	2065	344	3170	975	8877	1714	3723	1606
chr15	100300296	C	0	41	0	39	0	111	0	81	0	184	0	137
chr15	100300298	G	13	3108	3	3812	11	2392	8	4134	36	10544	4	5320
chr15	100300299	C	1	53	5	37	2	140	0	102	7	218	1	194
chr15	100300301	C	0	94	0	48	1	273	0	132	0	344	0	279
chr15	100300304	C	1	114	0	51	2	343	0	145	0	422	0	300
chr15	100300306	G	25	3082	26	3608	15	2385	5	4124	80	10468	77	5231
chr15	100300307	C	103	20	32	20	301	73	122	28	356	93	251	57
chr15	100300308	G	2712	373	2502	727	2215	178	3418	673	9788	740	4086	1202
chr15	100300312	G	1	2946	0	3020	0	2339	0	3775	1	10450	0	5178
chr15	100300314	C	0	287	0	61	2	1247	0	217	7	1545	1	425
chr15	100300316	C	3	284	1	60	58	1191	1	216	67	1484	0	426
chr15	100300318	G	1	2924	0	2973	0	2319	1	3725	2	10371	1	5075
chr15	100300320	C	0	287	0	61	1	1248	0	217	2	1549	0	426
chr15	100300322	G	1	2898	0	2950	1	2287	1	3649	0	10203	2	4923
chr15	100300324	G	0	2877	1	2919	0	2278	0	3595	1	10166	1	4879
chr15	100300326	G	0	2858	0	2887	2	2274	2	3549	0	10133	1	4840
chr15	100300330	G	0	2751	1	2667	1	2236	1	3397	2	9989	1	4676
chr15	100300331	C	2	283	1	60	5	1243	0	217	21	1526	8	417
chr15	100300338	C	1	286	0	61	2	1245	1	216	0	1546	1	425
chr15	100300339	C	237	49	28	33	960	283	134	82	1289	253	383	42
chr15	100300344	C	1	284	0	61	1	1246	1	216	7	1539	1	425
chr15	100300345	C	253	32	35	26	1104	138	193	24	1397	145	379	46
chr15	100300347	C	1	285	0	61	6	1240	1	217	7	1538	0	426
chr15	100300350	C	1	286	0	61	7	1238	0	218	8	1536	0	426
chr15	100300353	C	257	29	56	4	1133	112	210	6	1431	113	326	97
chr15	100300355	C	2	285	0	61	4	1242	0	218	8	1536	2	424
chr15	100300371	C	0	273	1	60	1	1218	0	212	5	1504	4	413
chr15	100300374	C	0	287	0	61	4	1235	1	217	10	1521	5	418
chr15	100300376	C	1	286	1	59	2	1237	0	218	1	1527	0	423
chr15	100300379	C	0	287	0	61	2	1237	1	217	8	1519	0	423
chr15	100300380	C	218	69	28	33	983	253	146	69	1251	274	371	51
chr15	100300383	C	0	287	0	61	3	1232	0	218	2	1521	0	423
chr15	100300394	C	2	285	0	61	4	1225	0	215	4	1513	0	416
chr15	100300402	C	0	284	0	59	1	1218	2	207	3	1506	1	399
chr15	100300406	C	1	283	0	59	2	1217	0	206	3	1504	1	397
chr15	100300414	C	1	283	0	59	8	1210	0	205	4	1500	0	394
chr15	100300416	C	1	283	0	58	3	1214	1	203	0	1503	2	391
chr15	100300419	C	0	283	0	57	0	1216	0	202	2	1497	0	388

0	27	0	35	0	75	0	54	0	141	1	73
1380	3119	970	3808	1251	2410	1518	4133	5550	10577	1835	5301
25	3125	8	3823	3	2411	26	4144	29	10599	9	5335
11	3073	16	3774	10	2377	59	4076	25	10424	10	5246
1	32	0	37	0	93	0	74	0	167	0	110
0	32	0	37	0	94	0	76	0	168	0	111
28	34	12	37	67	95	51	77	126	171	91	112
2450	3122	2451	3820	2065	2409	3170	4145	8877	10591	3723	5329
0	41	0	39	0	111	0	81	0	184	0	137
13	3121	3	3815	11	2403	8	4142	36	10580	4	5324
1	54	5	42	2	142	0	102	7	225	1	195
0	94	0	48	1	274	0	132	0	344	0	279
1	115	0	51	2	345	0	145	0	422	0	300
25	3107	26	3634	15	2400	5	4129	80	10548	77	5308
103	123	32	52	301	374	122	150	356	449	251	308
2712	3085	2502	3229	2215	2393	3418	4091	9788	10528	4086	5288
1	2947	0	3020	0	2339	0	3775	1	10451	0	5178
0	287	0	61	2	1249	0	217	7	1552	1	426
3	287	1	61	58	1249	1	217	67	1551	0	426
1	2925	0	2973	0	2319	1	3726	2	10373	1	5076
0	287	0	61	1	1249	0	217	2	1551	0	426
1	2899	0	2950	1	2288	1	3650	0	10203	2	4925
0	2877	1	2920	0	2278	0	3595	1	10167	1	4880
0	2858	0	2887	2	2276	2	3551	0	10133	1	4841
0	2751	1	2668	1	2237	1	3398	2	9991	1	4677
2	285	1	61	5	1248	0	217	21	1547	8	425
1	287	0	61	2	1247	1	217	0	1546	1	426
237	286	28	61	960	1243	134	216	1289	1542	383	425
1	285	0	61	1	1247	1	217	7	1546	1	426
253	285	35	61	1104	1242	193	217	1397	1542	379	425
1	286	0	61	6	1246	1	218	7	1545	0	426
1	287	0	61	7	1245	0	218	8	1544	0	426
257	286	56	60	1133	1245	210	216	1431	1544	326	423
2	287	0	61	4	1246	0	218	8	1544	2	426
0	273	1	61	1	1219	0	212	5	1509	4	417
0	287	0	61	4	1239	1	218	10	1531	5	423
1	287	1	60	2	1239	0	218	1	1528	0	423
0	287	0	61	2	1239	1	218	8	1527	0	423
218	287	28	61	983	1236	146	215	1251	1525	371	422
0	287	0	61	3	1235	0	218	2	1523	0	423
2	287	0	61	4	1229	0	215	4	1517	0	416
0	284	0	59	1	1219	2	209	3	1509	1	400
1	284	0	59	2	1219	0	206	3	1507	1	398
1	284	0	59	8	1218	0	205	4	1504	0	394
1	284	0	58	3	1217	1	204	0	1503	2	393
0	283	0	57	0	1216	0	202	2	1499	0	388

chr15	100300420	C	8	276	1	56	61	1155	0	202	60	1439	0	389
chr15	100300422	C	0	283	0	56	3	1210	1	199	1	1492	0	387
chr15	100300423	C	0	284	0	56	1	1214	1	200	3	1494	0	388
chr15	100300425	C	0	284	0	56	0	1212	0	199	0	1495	0	386
chr15	100300434	C	0	267	0	26	0	1200	0	145	0	1485	0	334
chr15	100300435	C	0	267	0	26	0	1202	0	146	0	1484	0	333
chr15	100300455	C	0	181	0	12	0	947	0	74	1	1214	0	131
chr15	100300460	C	0	173	0	11	0	899	0	69	0	1183	0	119
chr15	100300461	C	0	172	0	11	0	889	0	69	1	1166	0	118
chr16	92644291	G	0	248	0	58	0	1121	0	52	1	1713	0	481
chr16	92644300	G	0	261	0	61	0	1196	0	55	0	1857	0	514
chr16	92644309	G	0	360	0	90	0	1733	0	84	0	5300	0	727
chr16	92644311	G	0	360	0	90	0	1744	0	84	0	5322	1	729
chr16	92644318	G	7	449	0	97	9	2367	0	106	90	11435	6	785
chr16	92644322	G	2	469	0	100	8	2430	0	108	24	11845	0	811
chr16	92644323	G	1	470	1	99	3	2440	0	110	29	11871	1	815
chr16	92644327	G	1	472	0	100	10	2432	0	109	48	11892	12	806
chr16	92644328	G	0	473	0	100	7	2436	0	110	30	11907	0	819
chr16	92644331	G	1	472	0	100	7	2438	1	109	30	11911	0	819
chr16	92644336	G	336	124	57	40	1750	633	81	28	8666	3199	545	248
chr16	92644337	G	0	473	1	99	3	2445	0	110	47	11907	8	812
chr16	92644339	G	3	470	2	98	16	2432	1	109	76	11880	7	813
chr16	92644346	G	3	470	0	100	16	2432	3	107	72	11885	4	816
chr16	92644348	G	382	85	84	14	2037	382	95	14	10144	1637	613	195
chr16	92644352	G	2	470	0	100	6	2440	0	110	43	11907	12	807
chr16	92644353	G	2	472	0	100	10	2439	0	110	14	11959	1	821
chr16	92644354	G	0	474	0	100	2	2447	0	110	11	11966	1	821
chr16	92644361	G	2	472	0	100	9	2441	5	105	56	11926	3	819
chr16	92644366	G	0	474	0	101	10	2441	0	110	33	11948	3	819
chr16	92644370	G	2	472	0	101	6	2444	1	110	37	11958	0	823
chr16	92644372	G	2	473	0	101	16	2433	1	110	19	11974	3	820
chr16	92644373	G	0	475	1	100	6	2445	0	111	31	11962	0	823
chr16	92644374	G	0	475	0	101	15	2435	0	111	57	11935	2	820
chr16	92644377	G	1	474	0	101	11	2440	0	111	58	11936	1	822
chr16	92644379	G	10	465	1	100	44	2407	1	110	192	11799	24	800
chr16	92644380	G	2	473	0	101	3	2448	0	111	23	11971	0	823
chr16	92644381	G	0	475	0	101	6	2446	0	111	14	11980	1	823
chr16	92644386	G	280	196	68	33	1419	1035	76	35	7303	4694	441	383
chr16	92644388	G	1	475	0	101	0	2454	0	111	16	11981	1	826
chr16	92644389	G	2	472	0	101	4	2448	0	111	27	11975	2	825
chr16	92644390	G	2	474	2	99	4	2449	0	111	17	11992	0	827
chr16	92644391	G	1	475	0	101	2	2452	0	111	38	11968	1	826
chr16	92644402	G	0	476	0	101	8	2447	0	111	37	11980	1	836
chr16	92644408	G	0	476	0	101	4	2453	0	111	18	12000	1	836
chr16	92644414	G	2	474	0	101	4	2453	0	111	17	12006	0	839
chr16	92644416	G	6	470	0	101	13	2442	0	111	31	11989	11	829

8	284	1	57	61	1216	0	202	60	1499	0	389
0	283	0	56	3	1213	1	200	1	1493	0	387
0	284	0	56	1	1215	1	201	3	1497	0	388
0	284	0	56	0	1212	0	199	0	1495	0	386
0	267	0	26	0	1200	0	145	0	1485	0	334
0	267	0	26	0	1202	0	146	0	1484	0	333
0	181	0	12	0	947	0	74	1	1215	0	131
0	173	0	11	0	899	0	69	0	1183	0	119
0	172	0	11	0	889	0	69	1	1167	0	118
0	248	0	58	0	1121	0	52	1	1714	0	481
0	261	0	61	0	1196	0	55	0	1857	0	514
0	360	0	90	0	1733	0	84	0	5300	0	727
0	360	0	90	0	1744	0	84	0	5322	1	730
7	456	0	97	9	2376	0	106	90	11525	6	791
2	471	0	100	8	2438	0	108	24	11869	0	811
1	471	1	100	3	2443	0	110	29	11900	1	816
1	473	0	100	10	2442	0	109	48	11940	12	818
0	473	0	100	7	2443	0	110	30	11937	0	819
1	473	0	100	7	2445	1	110	30	11941	0	819
336	460	57	97	1750	2383	81	109	8666	11865	545	793
0	473	1	100	3	2448	0	110	47	11954	8	820
3	473	2	100	16	2448	1	110	76	11956	7	820
3	473	0	100	16	2448	3	110	72	11957	4	820
382	467	84	98	2037	2419	95	109	10144	11781	613	808
2	472	0	100	6	2446	0	110	43	11950	12	819
2	474	0	100	10	2449	0	110	14	11973	1	822
0	474	0	100	2	2449	0	110	11	11977	1	822
2	474	0	100	9	2450	5	110	56	11982	3	822
0	474	0	101	10	2451	0	110	33	11981	3	822
2	474	0	101	6	2450	1	111	37	11995	0	823
2	475	0	101	16	2449	1	111	19	11993	3	823
0	475	1	101	6	2451	0	111	31	11993	0	823
0	475	0	101	15	2450	0	111	57	11992	2	822
1	475	0	101	11	2451	0	111	58	11994	1	823
10	475	1	101	44	2451	1	111	192	11991	24	824
2	475	0	101	3	2451	0	111	23	11994	0	823
0	475	0	101	6	2452	0	111	14	11994	1	824
280	476	68	101	1419	2454	76	111	7303	11997	441	824
1	476	0	101	0	2454	0	111	16	11997	1	827
2	474	0	101	4	2452	0	111	27	12002	2	827
2	476	2	101	4	2453	0	111	17	12009	0	827
1	476	0	101	2	2454	0	111	38	12006	1	827
0	476	0	101	8	2455	0	111	37	12017	1	837
0	476	0	101	4	2457	0	111	18	12018	1	837
2	476	0	101	4	2457	0	111	17	12023	0	839
6	476	0	101	13	2455	0	111	31	12020	11	840

chr16	92644417	G	4	472	0	101	7	2451	0	112	35	11992	3	837
chr16	92644420	G	3	473	2	99	12	2446	4	108	100	11911	1	836
chr16	92644422	G	2	474	0	101	4	2455	0	112	47	11984	0	841
chr16	92644423	G	0	476	0	101	2	2456	0	112	38	11996	7	835
chr16	92644424	G	3	473	2	99	5	2454	0	112	41	11994	13	829
chr16	92644430	G	3	474	0	101	8	2452	1	111	34	12007	4	842
chr16	92644431	G	2	475	0	101	4	2452	0	111	45	11982	10	836
chr16	92644432	C	0	171	0	387	0	188	0	303	0	373	0	1039
chr16	92644434	G	3	475	2	99	38	2425	0	113	165	11878	13	833
chr16	92644436	G	9	469	2	99	62	2399	7	106	317	11720	26	820
chr16	92644437	G	0	478	0	101	2	2460	0	113	34	12007	2	844
chr16	92644439	G	4	474	0	100	22	2441	0	113	141	11902	0	846
chr16	92644441	G	5	473	1	100	17	2446	1	112	109	11935	4	842
chr16	92644442	G	1	477	0	101	3	2459	0	113	30	12015	2	843
chr16	92644445	C	0	176	0	401	0	193	0	318	0	388	0	1075
chr16	92644448	G	3	475	1	100	2	2460	0	113	33	12009	1	843
chr16	92644449	G	0	478	2	99	4	2458	0	113	25	12018	0	843
chr16	92644451	G	0	478	0	101	10	2451	0	112	36	12004	1	840
chr16	92644452	G	0	478	1	100	8	2453	0	112	29	11995	2	837
chr16	92644453	C	0	176	0	400	0	192	0	320	0	389	0	1073
chr16	92644454	C	0	177	0	402	0	193	0	320	0	389	0	1076
chr16	92644455	C	77	100	235	167	103	90	146	174	198	191	486	580
chr16	92644456	G	258	220	52	49	1306	1150	72	40	6634	5395	370	469
chr16	92644458	G	1	477	0	101	4	2457	0	112	22	12015	0	841
chr16	92644459	G	1	477	0	101	1	2460	0	112	25	12017	2	839
chr16	92644460	G	3	475	0	101	6	2455	0	111	35	12003	0	841
chr16	92644463	G	4	474	0	101	16	2445	0	111	63	11979	0	841
chr16	92644464	G	1	477	0	101	8	2453	2	109	29	12008	5	834
chr16	92644465	G	2	476	0	101	9	2450	0	111	48	11986	1	839
chr16	92644466	C	111	67	296	106	139	54	258	62	237	153	653	426
chr16	92644467	G	317	161	70	31	1654	807	80	30	8362	3677	493	347
chr16	92644469	G	1	477	0	101	6	2452	1	109	37	11992	0	839
chr16	92644470	G	0	478	0	101	7	2454	0	110	28	12006	0	840
chr16	92644471	G	3	475	0	101	4	2456	0	110	29	12004	2	836
chr16	92644472	G	0	478	0	101	3	2457	0	110	66	11969	1	838
chr16	92644473	G	1	477	1	100	12	2448	0	110	23	12012	0	839
chr16	92644474	C	0	177	0	401	0	193	1	318	1	389	5	1074
chr16	92644476	G	6	472	0	101	51	2407	0	110	154	11878	11	827
chr16	92644477	G	1	476	0	101	3	2452	0	110	12	12021	0	839
chr16	92644478	G	2	476	1	100	11	2446	0	110	26	12004	0	839
chr16	92644480	G	2	476	1	100	15	2442	2	108	64	11968	1	838
chr16	92644481	G	0	478	0	101	1	2456	0	110	31	12001	2	837
chr16	92644483	G	35	443	7	94	147	2310	8	102	886	11142	63	775
chr16	92644484	C	113	65	278	125	123	71	193	127	232	159	609	470
chr16	92644485	G	317	161	67	34	1718	735	87	23	8215	3813	519	317
chr16	92644486	G	2	476	1	100	2	2454	2	108	21	12011	1	835

4	476	0	101	7	2458	0	112	35	12027	3	840
3	476	2	101	12	2458	4	112	100	12011	1	837
2	476	0	101	4	2459	0	112	47	12031	0	841
0	476	0	101	2	2458	0	112	38	12034	7	842
3	476	2	101	5	2459	0	112	41	12035	13	842
3	477	0	101	8	2460	1	112	34	12041	4	846
2	477	0	101	4	2456	0	111	45	12027	10	846
0	171	0	387	0	188	0	303	0	373	0	1039
3	478	2	101	38	2463	0	113	165	12043	13	846
9	478	2	101	62	2461	7	113	317	12037	26	846
0	478	0	101	2	2462	0	113	34	12041	2	846
4	478	0	100	22	2463	0	113	141	12043	0	846
5	478	1	101	17	2463	1	113	109	12044	4	846
1	478	0	101	3	2462	0	113	30	12045	2	845
0	176	0	401	0	193	0	318	0	388	0	1075
3	478	1	101	2	2462	0	113	33	12042	1	844
0	478	2	101	4	2462	0	113	25	12043	0	843
0	478	0	101	10	2461	0	112	36	12040	1	841
0	478	1	101	8	2461	0	112	29	12024	2	839
0	176	0	400	0	192	0	320	0	389	0	1073
0	177	0	402	0	193	0	320	0	389	0	1076
77	177	235	402	103	193	146	320	198	389	486	1066
258	478	52	101	1306	2456	72	112	6634	12029	370	839
1	478	0	101	4	2461	0	112	22	12037	0	841
1	478	0	101	1	2461	0	112	25	12042	2	841
3	478	0	101	6	2461	0	111	35	12038	0	841
4	478	0	101	16	2461	0	111	63	12042	0	841
1	478	0	101	8	2461	2	111	29	12037	5	839
2	478	0	101	9	2459	0	111	48	12034	1	840
111	178	296	402	139	193	258	320	237	390	653	1079
317	478	70	101	1654	2461	80	110	8362	12039	493	840
1	478	0	101	6	2458	1	110	37	12029	0	839
0	478	0	101	7	2461	0	110	28	12034	0	840
3	478	0	101	4	2460	0	110	29	12033	2	838
0	478	0	101	3	2460	0	110	66	12035	1	839
1	478	1	101	12	2460	0	110	23	12035	0	839
0	177	0	401	0	193	1	319	1	390	5	1079
6	478	0	101	51	2458	0	110	154	12032	11	838
1	477	0	101	3	2455	0	110	12	12033	0	839
2	478	1	101	11	2457	0	110	26	12030	0	839
2	478	1	101	15	2457	2	110	64	12032	1	839
0	478	0	101	1	2457	0	110	31	12032	2	839
35	478	7	101	147	2457	8	110	886	12028	63	838
113	178	278	403	123	194	193	320	232	391	609	1079
317	478	67	101	1718	2453	87	110	8215	12028	519	836
2	478	1	101	2	2456	2	110	21	12032	1	836

chr16	92644487	G	1	477	0	101	5	2447	0	110	14	12001	2	833
chr16	92644488	C	0	178	1	402	1	195	0	320	3	389	1	1078
chr16	92644490	G	4	474	4	97	4	2451	0	110	32	11996	1	835
chr16	92644492	C	0	178	1	400	0	197	0	319	0	391	2	1080
chr16	92644493	C	0	178	0	403	0	197	2	319	2	390	2	1080
chr16	92644494	C	1	177	0	403	1	196	0	321	1	390	1	1081
chr16	92644496	C	1	177	1	402	1	196	0	321	0	392	12	1070
chr16	92644499	G	0	478	0	101	2	2451	0	110	37	11983	0	835
chr16	92644500	G	0	478	0	101	3	2447	0	110	35	11982	0	833
chr16	92644501	C	0	179	1	402	1	196	0	320	0	392	3	1079
chr16	92644502	C	108	71	266	137	126	71	126	195	219	173	654	426
chr16	92644503	G	339	139	61	40	1772	681	71	39	8784	3234	536	298
chr16	92644505	G	0	478	0	101	7	2446	1	109	25	11988	3	832
chr16	92644506	C	143	36	288	115	155	42	262	58	292	100	803	279
chr16	92644507	G	369	108	71	30	1908	542	84	26	9629	2380	597	236
chr16	92644508	C	1	178	1	402	1	196	1	319	3	390	2	1081
chr16	92644509	C	114	65	251	151	148	49	169	149	230	161	693	384
chr16	92644510	G	314	164	58	43	1643	808	72	38	8438	3572	519	316
chr16	92644511	C	1	178	2	401	5	192	0	321	1	392	1	1083
chr16	92644513	G	4	474	0	101	7	2445	0	110	45	11965	0	835
chr16	92644514	C	0	180	0	403	0	197	1	320	1	391	1	1082
chr16	92644516	G	2	476	0	101	7	2444	0	110	58	11953	8	828
chr16	92644517	C	4	176	11	393	2	195	36	285	11	382	11	1072
chr16	92644519	C	0	180	1	402	0	196	0	321	0	393	2	1081
chr16	92644520	C	2	178	11	393	5	192	0	321	3	390	11	1072
chr16	92644525	C	0	180	0	405	0	197	0	321	2	391	1	1083
chr16	92644527	C	1	179	2	403	0	197	1	320	2	391	0	1084
chr16	92644529	G	4	473	0	101	7	2443	1	109	30	11969	4	830
chr16	92644531	G	1	477	0	101	11	2438	0	110	29	11971	4	828
chr16	92644532	C	0	180	1	404	0	197	0	322	1	391	2	1081
chr16	92644533	C	111	69	273	132	120	77	167	155	240	153	606	478
chr16	92644534	G	291	185	56	45	1400	1044	73	37	7195	4779	469	363
chr16	92644535	C	2	178	2	403	0	197	3	318	2	390	1	1083
chr16	92644537	C	111	69	285	120	122	75	227	95	228	166	613	470
chr16	92644538	G	280	176	61	36	1450	893	61	46	7046	4380	437	357
chr16	92644539	G	1	474	0	100	19	2423	1	109	188	11794	10	822
chr16	92644544	G	1	475	0	100	12	2433	0	110	35	11951	3	831
chr16	92644545	G	0	476	0	101	8	2440	0	110	42	11954	3	830
chr16	92644547	C	3	177	6	399	0	197	1	321	2	392	0	1085
chr16	92644551	C	1	179	76	329	4	193	0	323	1	393	1	1083
chr16	92644553	C	0	180	1	404	0	198	1	322	0	394	0	1085
chr16	92644554	C	0	180	0	405	2	196	0	323	0	394	0	1085
chr16	92644555	C	101	79	229	176	118	79	183	140	215	179	604	481
chr16	92644556	G	262	202	50	49	1364	1069	71	37	6973	4926	411	410
chr16	92644557	G	0	455	0	98	0	2411	0	108	1	11831	0	820
chr16	92644558	G	0	455	0	98	0	2409	0	108	3	11821	0	820

1	478	0	101	5	2452	0	110	14	12015	2	835
0	178	1	403	1	196	0	320	3	392	1	1079
4	478	4	101	4	2455	0	110	32	12028	1	836
0	178	1	401	0	197	0	319	0	391	2	1082
0	178	0	403	0	197	2	321	2	392	2	1082
1	178	0	403	1	197	0	321	1	391	1	1082
1	178	1	403	1	197	0	321	0	392	12	1082
0	478	0	101	2	2453	0	110	37	12020	0	835
0	478	0	101	3	2450	0	110	35	12017	0	833
0	179	1	403	1	197	0	320	0	392	3	1082
108	179	266	403	126	197	126	321	219	392	654	1080
339	478	61	101	1772	2453	71	110	8784	12018	536	834
0	478	0	101	7	2453	1	110	25	12013	3	835
143	179	288	403	155	197	262	320	292	392	803	1082
369	477	71	101	1908	2450	84	110	9629	12009	597	833
1	179	1	403	1	197	1	320	3	393	2	1083
114	179	251	402	148	197	169	318	230	391	693	1077
314	478	58	101	1643	2451	72	110	8438	12010	519	835
1	179	2	403	5	197	0	321	1	393	1	1084
4	478	0	101	7	2452	0	110	45	12010	0	835
0	180	0	403	0	197	1	321	1	392	1	1083
2	478	0	101	7	2451	0	110	58	12011	8	836
4	180	11	404	2	197	36	321	11	393	11	1083
0	180	1	403	0	196	0	321	0	393	2	1083
2	180	11	404	5	197	0	321	3	393	11	1083
0	180	0	405	0	197	0	321	2	393	1	1084
1	180	2	405	0	197	1	321	2	393	0	1084
4	477	0	101	7	2450	1	110	30	11999	4	834
1	478	0	101	11	2449	0	110	29	12000	4	832
0	180	1	405	0	197	0	322	1	392	2	1083
111	180	273	405	120	197	167	322	240	393	606	1084
291	476	56	101	1400	2444	73	110	7195	11974	469	832
2	180	2	405	0	197	3	321	2	392	1	1084
111	180	285	405	122	197	227	322	228	394	613	1083
280	456	61	97	1450	2343	61	107	7046	11426	437	794
1	475	0	100	19	2442	1	110	188	11982	10	832
1	476	0	100	12	2445	0	110	35	11986	3	834
0	476	0	101	8	2448	0	110	42	11996	3	833
3	180	6	405	0	197	1	322	2	394	0	1085
1	180	76	405	4	197	0	323	1	394	1	1084
0	180	1	405	0	198	1	323	0	394	0	1085
0	180	0	405	2	198	0	323	0	394	0	1085
101	180	229	405	118	197	183	323	215	394	604	1085
262	464	50	99	1364	2433	71	108	6973	11899	411	821
0	455	0	98	0	2411	0	108	1	11832	0	820
0	455	0	98	0	2409	0	108	3	11824	0	820

chr16	92644559	C	0	179	1	403	0	199	1	320	1	392	7	1070
chr16	92644562	G	0	455	0	98	0	2406	0	108	0	11809	0	820
chr16	92644563	G	0	455	0	98	0	2407	0	108	0	11802	0	819
chr16	92644565	C	0	180	0	405	1	198	0	323	1	398	1	1085
chr16	92644567	G	0	455	0	98	0	2406	0	108	2	11778	1	815
chr16	92644570	C	0	180	7	398	4	196	0	323	1	398	17	1067
chr16	92644573	C	0	180	2	403	2	198	2	321	1	398	2	1084
chr16	92644576	G	0	454	0	98	0	2394	0	107	2	11682	0	807
chr16	92644580	C	3	177	3	402	1	199	17	305	2	397	3	1084
chr16	92644582	G	0	449	0	96	0	2369	0	107	2	11585	0	799
chr16	92644583	C	0	114	1	117	0	97	2	151	0	305	1	639
chr16	92644584	C	60	53	43	71	51	45	78	67	150	153	313	319
chr16	92644590	C	1	112	1	94	1	90	1	137	1	289	2	594
chr16	92644591	C	0	112	0	95	0	91	0	138	1	288	1	595
chr16	92644603	C	0	94	0	85	0	80	0	119	0	228	1	488
chr16	92644604	C	53	41	30	53	32	47	59	60	128	98	259	221
chr16	92644639	c	0	123	0	331	0	148	0	256	0	244	3	774
chr16	92644663	C	0	127	0	350	0	156	0	257	1	237	1	756
chr16	92644670	C	53	73	164	169	75	75	63	181	107	127	250	483
chr16	92644686	C	1	115	2	308	0	149	1	228	0	218	3	597
chr16	92644691	C	0	95	0	310	0	126	0	223	0	163	1	560
chr16	92644696	C	1	77	0	224	0	98	0	188	0	134	1	418
chr16	92644700	C	1	65	3	302	1	109	0	184	0	113	9	467
chr16	92644701	C	1	65	2	303	2	109	2	183	3	110	9	467
chr16	92644702	C	1	64	9	297	1	110	7	176	5	106	18	453
chr16	92644703	C	5	61	15	290	1	110	3	181	10	101	27	441
chr16	92644705	C	2	64	16	290	2	107	8	176	4	106	49	421
chr16	92644706	C	2	63	8	296	2	106	8	175	7	104	50	412
chr16	92644707	C	18	47	54	247	13	94	74	109	31	77	67	399
chr16	92644709	C	33	32	130	175	67	42	57	125	33	75	40	424
chr16	92644711	C	1	64	30	274	1	108	2	180	0	108	10	457
chr16	92644712	C	1	64	2	303	0	109	1	181	1	107	6	459
chr16	92644713	C	5	60	0	305	0	109	1	180	0	108	5	462
chr16	92644716	C	11	54	9	295	16	93	1	180	19	88	18	449
chr16	92644718	C	0	65	0	304	1	108	0	181	0	107	1	464
chr16	92644719	C	0	65	2	302	1	108	2	178	1	106	2	464
chr16	92644720	C	0	65	0	302	1	108	0	180	0	106	2	464
chr16	92644721	C	0	65	0	304	1	108	1	179	0	107	5	461
chr16	92644722	C	0	64	0	304	0	108	0	179	0	106	1	464
chr16	92644731	C	1	63	0	304	1	107	1	179	1	104	4	460
chr16	92644740	C	0	64	0	303	0	108	1	179	0	104	0	464
chr16	92644745	C	0	64	2	300	1	107	0	178	1	103	0	462
chr16	92644746	C	0	64	0	302	0	108	0	180	0	103	0	464
chr16	92644747	C	0	64	0	302	0	108	0	180	0	103	0	464
chr16	92644748	C	0	64	0	302	0	108	0	180	0	103	3	461
chr16	92644754	C	0	64	0	302	0	108	1	179	2	101	3	461

0	179	1	404	0	199	1	321	1	393	7	1077
0	455	0	98	0	2406	0	108	0	11809	0	820
0	455	0	98	0	2407	0	108	0	11802	0	819
0	180	0	405	1	199	0	323	1	399	1	1086
0	455	0	98	0	2406	0	108	2	11780	1	816
0	180	7	405	4	200	0	323	1	399	17	1084
0	180	2	405	2	200	2	323	1	399	2	1086
0	454	0	98	0	2394	0	107	2	11684	0	807
3	180	3	405	1	200	17	322	2	399	3	1087
0	449	0	96	0	2369	0	107	2	11587	0	799
0	114	1	118	0	97	2	153	0	305	1	640
60	113	43	114	51	96	78	145	150	303	313	632
1	113	1	95	1	91	1	138	1	290	2	596
0	112	0	95	0	91	0	138	1	289	1	596
0	94	0	85	0	80	0	119	0	228	1	489
53	94	30	83	32	79	59	119	128	226	259	480
0	123	0	331	0	148	0	256	0	244	3	777
0	127	0	350	0	156	0	257	1	238	1	757
53	126	164	333	75	150	63	244	107	234	250	733
1	116	2	310	0	149	1	229	0	218	3	600
0	95	0	310	0	126	0	223	0	163	1	561
1	78	0	224	0	98	0	188	0	134	1	419
1	66	3	305	1	110	0	184	0	113	9	476
1	66	2	305	2	111	2	185	3	113	9	476
1	65	9	306	1	111	7	183	5	111	18	471
5	66	15	305	1	111	3	184	10	111	27	468
2	66	16	306	2	109	8	184	4	110	49	470
2	65	8	304	2	108	8	183	7	111	50	462
18	65	54	301	13	107	74	183	31	108	67	466
33	65	130	305	67	109	57	182	33	108	40	464
1	65	30	304	1	109	2	182	0	108	10	467
1	65	2	305	0	109	1	182	1	108	6	465
5	65	0	305	0	109	1	181	0	108	5	467
11	65	9	304	16	109	1	181	19	107	18	467
0	65	0	304	1	109	0	181	0	107	1	465
0	65	2	304	1	109	2	180	1	107	2	466
0	65	0	302	1	109	0	180	0	106	2	466
0	65	0	304	1	109	1	180	0	107	5	466
0	64	0	304	0	108	0	179	0	106	1	465
1	64	0	304	1	108	1	180	1	105	4	464
0	64	0	303	0	108	1	180	0	104	0	464
0	64	2	302	1	108	0	178	1	104	0	462
0	64	0	302	0	108	0	180	0	103	0	464
0	64	0	302	0	108	0	180	0	103	0	464
0	64	0	302	0	108	0	180	0	103	3	464
0	64	0	302	0	108	1	180	2	103	3	464

chr16	92644758	C	0	64	3	299	0	108	0	180	0	103	1	463
chr16	92644761	C	0	64	0	301	0	107	0	176	0	102	0	462
chr16	92644762	C	0	64	0	302	0	107	0	177	0	103	0	463
chr16	92644771	C	0	63	0	301	0	102	0	177	0	100	1	454
chr16	92644772	C	0	63	1	300	0	102	0	177	0	99	1	453
chr16	92644777	C	0	59	0	291	0	99	0	174	0	94	0	439
chr16	92644781	C	0	57	0	263	0	92	0	156	0	82	0	403
chr16	92644782	C	0	57	0	259	0	91	0	155	0	82	0	402
chr17	45706386	C	1	807	0	148	1	1239	0	645	0	640	0	2052
chr17	45706403	C	0	1328	0	253	0	1891	0	1225	0	1034	1	3900
chr17	45706405	C	0	1328	0	255	0	1890	0	1225	1	1033	0	3906
chr17	45706410	C	0	1330	0	256	0	1894	0	1234	0	1038	2	3925
chr17	45706411	C	1045	326	260	30	1581	314	935	357	867	173	3005	953
chr17	45706414	C	4	1367	1	300	3	1893	1	1305	6	1035	6	3961
chr17	45706418	C	1	1372	0	303	3	1895	4	1301	3	1039	11	3956
chr17	45706419	C	2	1372	1	302	1	1896	1	1308	1	1043	10	3969
chr17	45706439	C	3	1378	3	301	6	1905	2	1314	4	1055	9	3985
chr17	45706440	C	1	1383	2	302	3	1912	3	1315	3	1057	6	3991
chr17	45706442	C	2	1382	1	303	5	1910	4	1314	5	1056	7	3989
chr17	45706449	C	6	1375	1	302	1	1910	1	1310	1	1066	3	3988
chr17	45706452	C	2	1382	0	304	2	1913	1	1316	2	1067	3	3996
chr17	45706458	G	0	1725	2	3578	1	4884	0	1883	0	5485	0	3291
chr17	45706461	C	15	1369	0	304	36	1883	1	1317	21	1062	19	3984
chr17	45706463	C	3	1383	0	304	3	1943	5	1313	2	1127	5	4001
chr17	45706464	C	5	1381	0	304	4	1942	1	1317	3	1128	6	4000
chr17	45706467	G	0	1829	0	3823	2	5203	0	1993	1	5767	1	3521
chr17	45706469	C	6	1381	0	304	3	1943	1	1316	4	1128	8	3997
chr17	45706470	C	1	1385	0	304	5	1944	5	1311	6	1125	7	4000
chr17	45706472	C	24	1364	23	281	34	1915	1	1317	15	1117	69	3939
chr17	45706474	G	0	1884	1	3903	2	5355	0	2061	1	5927	2	3650
chr17	45706475	C	2	1382	0	304	4	1934	2	1315	4	1117	5	4004
chr17	45706476	C	7	1381	2	302	12	1949	0	1318	8	1143	3	4009
chr17	45706478	G	1	1884	1	3910	0	5362	0	2060	1	5927	0	3651
chr17	45706479	C	6	1384	1	303	7	1954	3	1316	6	1146	8	4006
chr17	45706481	C	1073	320	177	127	1503	457	947	374	876	280	3272	745
chr17	45706482	G	1612	249	3098	820	4591	719	1619	416	5183	644	2972	580
chr17	45706483	G	22	1883	74	4010	58	5356	36	2064	66	5890	46	3617
chr17	45706485	G	28	1877	112	3979	20	5397	3	2103	64	5903	9	3656
chr17	45706488	G	0	1917	6	4093	11	5414	3	2117	29	5987	10	3684
chr17	45706490	G	17	1898	8	4082	46	5378	1	2120	53	5961	106	3593
chr17	45706491	C	9	1383	1	302	8	1958	4	1316	2	1171	7	4001
chr17	45706493	C	3	1392	0	306	2	1971	4	1320	3	1183	5	4011
chr17	45706494	C	47	1348	2	304	79	1894	34	1290	35	1148	75	3940
chr17	45706496	G	5	1912	103	3984	16	5416	5	2116	14	6006	14	3685
chr17	45706497	G	3	1911	8	4094	16	5413	4	2119	17	6006	15	3685
chr17	45706498	C	17	1378	18	288	34	1942	0	1322	25	1185	5	4011

0	64	3	302	0	108	0	180	0	103	1	464
0	64	0	301	0	107	0	176	0	102	0	462
0	64	0	302	0	107	0	177	0	103	0	463
0	63	0	301	0	102	0	177	0	100	1	455
0	63	1	301	0	102	0	177	0	99	1	454
0	59	0	291	0	99	0	174	0	94	0	439
0	57	0	263	0	92	0	156	0	82	0	403
0	57	0	259	0	91	0	155	0	82	0	402
1	808	0	148	1	1240	0	645	0	640	0	2052
0	1328	0	253	0	1891	0	1225	0	1034	1	3901
0	1328	0	255	0	1890	0	1225	1	1034	0	3906
0	1330	0	256	0	1894	0	1234	0	1038	2	3927
1045	1371	260	290	1581	1895	935	1292	867	1040	3005	3958
4	1371	1	301	3	1896	1	1306	6	1041	6	3967
1	1373	0	303	3	1898	4	1305	3	1042	11	3967
2	1374	1	303	1	1897	1	1309	1	1044	10	3979
3	1381	3	304	6	1911	2	1316	4	1059	9	3994
1	1384	2	304	3	1915	3	1318	3	1060	6	3997
2	1384	1	304	5	1915	4	1318	5	1061	7	3996
6	1381	1	303	1	1911	1	1311	1	1067	3	3991
2	1384	0	304	2	1915	1	1317	2	1069	3	3999
0	1725	2	3580	1	4885	0	1883	0	5485	0	3291
15	1384	0	304	36	1919	1	1318	21	1083	19	4003
3	1386	0	304	3	1946	5	1318	2	1129	5	4006
5	1386	0	304	4	1946	1	1318	3	1131	6	4006
0	1829	0	3823	2	5205	0	1993	1	5768	1	3522
6	1387	0	304	3	1946	1	1317	4	1132	8	4005
1	1386	0	304	5	1949	5	1316	6	1131	7	4007
24	1388	23	304	34	1949	1	1318	15	1132	69	4008
0	1884	1	3904	2	5357	0	2061	1	5928	2	3652
2	1384	0	304	4	1938	2	1317	4	1121	5	4009
7	1388	2	304	12	1961	0	1318	8	1151	3	4012
1	1885	1	3911	0	5362	0	2060	1	5928	0	3651
6	1390	1	304	7	1961	3	1319	6	1152	8	4014
1073	1393	177	304	1503	1960	947	1321	876	1156	3272	4017
1612	1861	3098	3918	4591	5310	1619	2035	5183	5827	2972	3552
22	1905	74	4084	58	5414	36	2100	66	5956	46	3663
28	1905	112	4091	20	5417	3	2106	64	5967	9	3665
0	1917	6	4099	11	5425	3	2120	29	6016	10	3694
17	1915	8	4090	46	5424	1	2121	53	6014	106	3699
9	1392	1	303	8	1966	4	1320	2	1173	7	4008
3	1395	0	306	2	1973	4	1324	3	1186	5	4016
47	1395	2	306	79	1973	34	1324	35	1183	75	4015
5	1917	103	4087	16	5432	5	2121	14	6020	14	3699
3	1914	8	4102	16	5429	4	2123	17	6023	15	3700
17	1395	18	306	34	1976	0	1322	25	1210	5	4016

chr17	45706500	C	10	1382	0	302	5	1966	1	1317	4	1204	86	3906
chr17	45706503	C	3	1394	0	306	4	1977	4	1320	10	1211	85	3934
chr17	45706506	G	7	1912	5	4095	11	5417	3	2119	42	5980	9	3691
chr17	45706508	C	778	618	160	145	1108	873	580	744	682	545	2101	1920
chr17	45706509	G	1195	720	2405	1682	3310	2118	1402	716	3655	2358	2373	1312
chr17	45706512	G	11	1908	2	4101	10	5423	37	2086	51	5974	5	3696
chr17	45706515	C	4	1394	0	306	5	1977	4	1320	6	1233	3	4024
chr17	45706518	G	11	1908	3	4101	127	5311	2	2122	93	5931	57	3643
chr17	45706519	C	1161	239	226	79	1590	393	1052	274	1041	201	3162	865
chr17	45706520	G	1663	250	3117	974	4699	721	1827	286	5169	831	3048	640
chr17	45706521	G	6	1913	6	4099	9	5429	6	2117	17	6006	12	3689
chr17	45706524	G	8	1911	5	4100	48	5393	2	2122	39	5994	3	3704
chr17	45706526	C	5	1393	2	306	4	1980	3	1323	3	1244	3	4024
chr17	45706527	C	4	1396	1	307	5	1982	3	1324	4	1245	6	4024
chr17	45706530	C	26	1374	0	308	53	1936	0	1328	27	1225	44	3986
chr17	45706532	G	8	1915	15	4104	17	5436	6	2124	32	6018	9	3714
chr17	45706533	G	3	1920	4	4115	7	5445	5	2126	29	6022	9	3716
chr17	45706536	C	6	1402	0	307	2	2041	0	1327	2	1399	6	4023
chr17	45706539	G	15	1907	3	4115	66	5388	2	2131	43	6010	4	3722
chr17	45706540	C	5	307	1	111	1	345	2	468	2	514	14	1114
chr17	45706542	C	0	303	0	110	0	336	1	456	3	511	2	1101
chr17	45706544	G	7	1917	4	4114	19	5436	1	2133	17	6040	8	3724
chr17	45706545	C	7	274	0	107	7	317	0	435	9	503	32	1021
chr17	45706547	G	42	1883	107	4009	145	5304	67	2069	197	5855	22	3728
chr17	45706548	C	6	150	0	87	5	216	0	290	11	457	24	534
chr17	45706550	G	11	1914	133	3988	28	5429	5	2134	66	5998	4	3752
chr17	45706551	C	0	93	0	61	0	183	0	129	2	463	0	185
chr17	45706554	C	0	94	0	62	1	179	0	130	1	468	0	178
chr17	45706556	C	0	92	0	61	0	181	0	128	0	468	0	173
chr17	45706557	C	0	96	0	60	0	182	0	128	0	476	0	164
chr17	45706558	C	65	31	28	34	120	62	73	56	307	165	99	61
chr17	45706559	G	1326	597	1988	2119	3912	1534	1616	520	4349	1707	2409	1344
chr17	45706560	G	4	1924	109	4013	23	5441	5	2135	12	6059	4	3756
chr17	45706561	G	3	1925	2	4120	5	5460	0	2140	15	6056	2	3759
chr17	45706563	G	8	1920	2	4121	29	5434	3	2137	32	6041	5	3755
chr17	45706566	G	13	1915	5	4119	37	5429	1	2141	30	6045	3	3763
chr17	45706569	C	0	67	0	27	1	189	1	61	5	479	4	150
chr17	45706571	G	13	1917	10	4113	34	5431	1	2140	22	6057	13	3754
chr17	45706572	G	13	1917	32	4094	16	5452	4	2141	16	6065	3	3764
chr17	45706573	G	6	1925	17	4107	9	5459	8	2137	26	6060	7	3761
chr17	45706574	G	6	1927	11	4112	8	5458	3	2140	6	6076	5	3762
chr17	45706575	C	4	443	0	56	2	409	1	378	4	536	18	1167
chr17	45706577	G	2	1932	6	4117	10	5457	4	2138	12	6076	10	3761
chr17	45706578	G	1	1933	11	4114	13	5458	2	2143	15	6077	5	3769
chr17	45706580	C	0	478	0	59	1	428	0	405	1	545	0	1299
chr17	45706581	C	2	479	0	59	0	428	0	413	2	543	2	1317

10	1392	0	302	5	1971	1	1318	4	1208	86	3992
3	1397	0	306	4	1981	4	1324	10	1221	85	4019
7	1919	5	4100	11	5428	3	2122	42	6022	9	3700
778	1396	160	305	1108	1981	580	1324	682	1227	2101	4021
1195	1915	2405	4087	3310	5428	1402	2118	3655	6013	2373	3685
11	1919	2	4103	10	5433	37	2123	51	6025	5	3701
4	1398	0	306	5	1982	4	1324	6	1239	3	4027
11	1919	3	4104	127	5438	2	2124	93	6024	57	3700
1161	1400	226	305	1590	1983	1052	1326	1041	1242	3162	4027
1663	1913	3117	4091	4699	5420	1827	2113	5169	6000	3048	3688
6	1919	6	4105	9	5438	6	2123	17	6023	12	3701
8	1919	5	4105	48	5441	2	2124	39	6033	3	3707
5	1398	2	308	4	1984	3	1326	3	1247	3	4027
4	1400	1	308	5	1987	3	1327	4	1249	6	4030
26	1400	0	308	53	1989	0	1328	27	1252	44	4030
8	1923	15	4119	17	5453	6	2130	32	6050	9	3723
3	1923	4	4119	7	5452	5	2131	29	6051	9	3725
6	1408	0	307	2	2043	0	1327	2	1401	6	4029
15	1922	3	4118	66	5454	2	2133	43	6053	4	3726
5	312	1	112	1	346	2	470	2	516	14	1128
0	303	0	110	0	336	1	457	3	514	2	1103
7	1924	4	4118	19	5455	1	2134	17	6057	8	3732
7	281	0	107	7	324	0	435	9	512	32	1053
42	1925	107	4116	145	5449	67	2136	197	6052	22	3750
6	156	0	87	5	221	0	290	11	468	24	558
11	1925	133	4121	28	5457	5	2139	66	6064	4	3756
0	93	0	61	0	183	0	129	2	465	0	185
0	94	0	62	1	180	0	130	1	469	0	178
0	92	0	61	0	181	0	128	0	468	0	173
0	96	0	60	0	182	0	128	0	476	0	164
65	96	28	62	120	182	73	129	307	472	99	160
1326	1923	1988	4107	3912	5446	1616	2136	4349	6056	2409	3753
4	1928	109	4122	23	5464	5	2140	12	6071	4	3760
3	1928	2	4122	5	5465	0	2140	15	6071	2	3761
8	1928	2	4123	29	5463	3	2140	32	6073	5	3760
13	1928	5	4124	37	5466	1	2142	30	6075	3	3766
0	67	0	27	1	190	1	62	5	484	4	154
13	1930	10	4123	34	5465	1	2141	22	6079	13	3767
13	1930	32	4126	16	5468	4	2145	16	6081	3	3767
6	1931	17	4124	9	5468	8	2145	26	6086	7	3768
6	1933	11	4123	8	5466	3	2143	6	6082	5	3767
4	447	0	56	2	411	1	379	4	540	18	1185
2	1934	6	4123	10	5467	4	2142	12	6088	10	3771
1	1934	11	4125	13	5471	2	2145	15	6092	5	3774
0	478	0	59	1	429	0	405	1	546	0	1299
2	481	0	59	0	428	0	413	2	545	2	1319

chr17	45706583	C	369	145	43	21	362	110	319	145	436	124	1030	496
chr17	45706584	G	1589	344	3379	745	4547	924	1783	365	5035	1061	2920	852
chr17	45706585	G	5	1928	6	4120	5	5469	4	2145	22	6079	11	3762
chr17	45706587	G	6	1928	6	4120	27	5450	5	2145	36	6068	4	3772
chr17	45706589	C	1	676	0	92	0	702	0	639	2	621	1	2236
chr17	45706592	C	2	682	0	95	1	705	0	642	1	624	2	2250
chr17	45706593	C	2	684	1	96	0	707	0	645	5	619	45	2213
chr17	45706595	C	32	658	0	99	37	676	20	629	33	596	30	2245
chr17	45706597	C	1	741	0	115	4	782	3	697	4	670	3	2410
chr17	45706598	C	532	336	83	61	622	385	574	258	539	260	1912	793
chr17	45706599	G	1276	659	2293	1832	3638	1838	1291	820	3951	2151	2287	1440
chr17	45706600	C	793	452	143	138	1052	668	479	734	826	396	2063	1595
chr17	45706601	G	1440	501	2898	1231	4123	1358	1449	705	4524	1594	2512	1271
chr17	45706602	G	5	1934	5	4128	6	5478	3	2152	10	6107	3	3784
chr17	45706608	G	0	329	1	728	2	785	2	414	2	1039	0	888
chr17	45706611	G	0	263	1	577	1	601	0	313	1	816	0	667
chr17	45706613	G	0	228	0	472	1	498	0	267	2	706	0	570
chr17	45706614	G	0	219	1	445	0	473	0	257	6	677	1	550
chr17	45706617	C	9	1407	0	310	7	2052	2	1329	3	1425	5	4038
chr17	45706619	C	1	1415	0	311	14	2046	0	1333	3	1423	0	4041
chr17	45706621	G	0	153	0	360	0	287	0	184	2	493	1	378
chr17	45706622	G	2	146	1	354	1	274	0	179	2	486	3	364
chr17	45706626	C	23	1394	17	293	28	2031	2	1329	22	1402	0	4038
chr17	45706628	G	0	135	0	344	0	268	0	170	0	480	0	356
chr17	45706629	C	7	1409	0	310	4	2055	9	1321	5	1415	3	4032
chr17	45706631	C	955	451	237	73	1488	560	831	489	1000	420	2497	1515
chr17	45706632	G	85	32	140	65	174	51	111	26	334	131	230	119
chr17	45706634	C	4	1411	1	310	11	2046	14	1316	1	1420	87	3953
chr17	45706636	C	40	1372	4	306	69	1989	51	1278	46	1371	128	3905
chr17	45706638	G	0	116	2	167	2	222	1	125	1	462	0	361
chr17	45706639	C	10	1405	0	311	19	2039	3	1328	9	1411	4	4037
chr17	45706641	G	2	113	3	173	4	219	0	125	5	462	5	355
chr17	45706643	C	6	1409	1	309	4	2055	4	1326	3	1414	10	4028
chr17	45706644	C	13	1402	11	299	8	2050	2	1328	13	1403	5	4032
chr17	45706647	G	1	116	0	171	0	220	0	124	1	459	1	354
chr17	45706650	G	2	114	1	172	4	210	3	117	6	457	1	359
chr17	45706653	C	22	1392	0	310	47	2008	42	1287	43	1373	4	4032
chr17	45706655	C	4	1411	0	310	7	2050	1	1328	2	1414	5	4032
chr17	45706657	C	988	359	219	74	1439	504	783	492	985	369	2422	1477
chr17	45706658	G	80	29	96	84	149	62	64	52	295	152	205	141
chr17	45706659	G	0	109	1	178	2	210	0	116	2	446	0	347
chr17	45706660	C	1143	227	252	53	1704	299	975	339	1196	191	3288	673
chr17	45706661	G	93	17	150	26	183	30	103	15	371	77	259	93
chr17	45706662	G	0	108	0	165	1	213	0	113	0	437	0	336
chr17	45706664	C	4	1411	2	305	13	2042	1	1329	14	1401	4	4030
chr17	45706668	C	22	1393	1	306	33	2024	0	1327	28	1384	101	3930

369	514	43	64	362	472	319	464	436	560	1030	1526
1589	1933	3379	4124	4547	5471	1783	2148	5035	6096	2920	3772
5	1933	6	4126	5	5474	4	2149	22	6101	11	3773
6	1934	6	4126	27	5477	5	2150	36	6104	4	3776
1	677	0	92	0	702	0	639	2	623	1	2237
2	684	0	95	1	706	0	642	1	625	2	2252
2	686	1	97	0	707	0	645	5	624	45	2258
32	690	0	99	37	713	20	649	33	629	30	2275
1	742	0	115	4	786	3	700	4	674	3	2413
532	868	83	144	622	1007	574	832	539	799	1912	2705
1276	1935	2293	4125	3638	5476	1291	2111	3951	6102	2287	3727
793	1245	143	281	1052	1720	479	1213	826	1222	2063	3658
1440	1941	2898	4129	4123	5481	1449	2154	4524	6118	2512	3783
5	1939	5	4133	6	5484	3	2155	10	6117	3	3787
0	329	1	729	2	787	2	416	2	1041	0	888
0	263	1	578	1	602	0	313	1	817	0	667
0	228	0	472	1	499	0	267	2	708	0	570
0	219	1	446	0	473	0	257	6	683	1	551
9	1416	0	310	7	2059	2	1331	3	1428	5	4043
1	1416	0	311	14	2060	0	1333	3	1426	0	4041
0	153	0	360	0	287	0	184	2	495	1	379
2	148	1	355	1	275	0	179	2	488	3	367
23	1417	17	310	28	2059	2	1331	22	1424	0	4038
0	135	0	344	0	268	0	170	0	480	0	356
7	1416	0	310	4	2059	9	1330	5	1420	3	4035
955	1406	237	310	1488	2048	831	1320	1000	1420	2497	4012
85	117	140	205	174	225	111	137	334	465	230	349
4	1415	1	311	11	2057	14	1330	1	1421	87	4040
40	1412	4	310	69	2058	51	1329	46	1417	128	4033
0	116	2	169	2	224	1	126	1	463	0	361
10	1415	0	311	19	2058	3	1331	9	1420	4	4041
2	115	3	176	4	223	0	125	5	467	5	360
6	1415	1	310	4	2059	4	1330	3	1417	10	4038
13	1415	11	310	8	2058	2	1330	13	1416	5	4037
1	117	0	171	0	220	0	124	1	460	1	355
2	116	1	173	4	214	3	120	6	463	1	360
22	1414	0	310	47	2055	42	1329	43	1416	4	4036
4	1415	0	310	7	2057	1	1329	2	1416	5	4037
988	1347	219	293	1439	1943	783	1275	985	1354	2422	3899
80	109	96	180	149	211	64	116	295	447	205	346
0	109	1	179	2	212	0	116	2	448	0	347
1143	1370	252	305	1704	2003	975	1314	1196	1387	3288	3961
93	110	150	176	183	213	103	118	371	448	259	352
0	108	0	165	1	214	0	113	0	437	0	336
4	1415	2	307	13	2055	1	1330	14	1415	4	4034
22	1415	1	307	33	2057	0	1327	28	1412	101	4031

chr17	45706674	G	1	1906	7	4087	11	5420	0	2072	6	6015	3	3598
chr17	45706677	G	35	1867	188	3894	164	5262	33	2035	151	5862	119	3464
chr17	45706680	G	9	1892	3	4081	78	5348	2	2069	52	5962	11	3583
chr17	45706683	C	5	1327	2	291	10	1948	3	1218	1	1347	6	3780
chr17	45706686	C	10	1404	2	305	4	2047	3	1323	8	1389	13	4010
chr17	45706689	C	7	1408	1	306	6	2049	50	1275	5	1405	24	4001
chr17	45706692	C	4	1410	0	307	2	2049	1	1323	5	1392	6	4018
chr17	45706695	G	68	1827	65	4009	242	5177	63	2003	247	5748	14	3564
chr17	45706698	C	4	1408	1	306	7	2045	1	1324	11	1386	10	4014
chr17	45706700	C	3	1412	0	307	11	2040	2	1323	4	1392	1	4022
chr17	45706706	G	4	1896	5	4074	9	5416	2	2068	17	5981	10	3571
chr17	45706707	G	1	1895	10	4059	18	5400	2	2047	17	5975	8	3566
chr17	45706709	C	838	570	190	117	1293	756	783	539	972	422	2389	1632
chr17	45706710	G	1166	708	2115	1882	3313	2026	916	1096	3617	2269	2044	1470
chr17	45706713	G	3	1892	4	4069	14	5404	3	2063	22	5966	3	3565
chr17	45706714	G	2	1893	6	4067	4	5414	2	2065	18	5971	4	3565
chr17	45706715	G	7	1887	8	4065	16	5402	4	2061	13	5975	5	3564
chr17	45706717	G	21	1705	107	3685	111	4860	4	1873	88	5389	5	3220
chr17	45706718	C	6	1404	1	302	9	2041	5	1311	3	1385	13	3993
chr17	45706719	C	2	1407	2	298	1	2045	2	1319	1	1388	5	3995
chr17	45706720	C	1067	328	205	91	1561	483	1039	256	1085	299	3065	901
chr17	45706721	G	1421	466	2803	1269	3944	1467	1569	493	4427	1540	2580	975
chr17	45706722	C	10	1378	0	293	6	2031	3	1302	3	1376	5	3947
chr17	45706723	C	1061	317	175	116	1556	476	942	346	1065	313	2802	1103
chr17	45706724	G	1457	433	2725	1326	4299	1097	1445	610	4544	1401	2684	858
chr17	45706725	G	2	1892	5	4061	9	5401	2	2063	8	5963	46	3513
chr17	45706726	G	2	1892	5	4058	7	5403	1	2063	14	5955	6	3553
chr17	45706727	G	2	1892	9	4058	5	5404	3	2061	10	5957	5	3552
chr17	45706731	G	6	1882	7	4049	10	5387	2	2055	42	5906	9	3535
chr17	45706734	G	4	1882	5	4059	9	5393	8	2051	24	5925	9	3533
chr17	45706736	G	63	1821	186	3871	124	5267	6	2048	166	5760	170	3348
chr17	45706737	C	2	772	0	232	0	1498	0	731	1	1166	1	1890
chr17	45706741	G	9	1876	5	4053	10	5387	4	2046	13	5912	2	3519
chr17	45706742	C	0	765	0	228	1	1482	0	721	1	1155	0	1836
chr17	45706743	C	0	760	0	229	0	1482	0	723	0	1154	1	1834
chr17	45706744	C	0	758	0	228	1	1482	0	720	0	1152	0	1826
chr17	45706746	G	25	1859	77	3980	35	5351	2	2047	54	5839	163	3344
chr17	45706757	G	5	1877	5	4046	19	5362	5	2041	14	5865	2	3494
chr17	45706768	G	6	1872	2	4039	14	5360	4	2041	22	5851	7	3486
chr17	45706770	G	4	1875	66	3976	12	5362	3	2043	14	5858	4	3490
chr17	45706773	G	9	1869	3	4037	9	5363	4	2042	17	5854	3	3488
chr17	45706775	G	22	1846	2	4022	27	5322	1	2037	72	5783	11	3473
chr17	45706778	G	48	1828	175	3854	170	5183	58	1981	236	5619	129	3356
chr17	45706783	G	2	1874	6	4027	6	5352	1	2041	4	5852	0	3479
chr17	45706803	G	0	1873	1	4013	0	5347	1	2039	0	5844	0	3471
chr17	45706804	G	0	1873	1	4012	1	5346	0	2041	0	5843	1	3470

1	1907	7	4094	11	5431	0	2072	6	6021	3	3601
35	1902	188	4082	164	5426	33	2068	151	6013	119	3583
9	1901	3	4084	78	5426	2	2071	52	6014	11	3594
5	1332	2	293	10	1958	3	1221	1	1348	6	3786
10	1414	2	307	4	2051	3	1326	8	1397	13	4023
7	1415	1	307	6	2055	50	1325	5	1410	24	4025
4	1414	0	307	2	2051	1	1324	5	1397	6	4024
68	1895	65	4074	242	5419	63	2066	247	5995	14	3578
4	1412	1	307	7	2052	1	1325	11	1397	10	4024
3	1415	0	307	11	2051	2	1325	4	1396	1	4023
4	1900	5	4079	9	5425	2	2070	17	5998	10	3581
1	1896	10	4069	18	5418	2	2049	17	5992	8	3574
838	1408	190	307	1293	2049	783	1322	972	1394	2389	4021
1166	1874	2115	3997	3313	5339	916	2012	3617	5886	2044	3514
3	1895	4	4073	14	5418	3	2066	22	5988	3	3568
2	1895	6	4073	4	5418	2	2067	18	5989	4	3569
7	1894	8	4073	16	5418	4	2065	13	5988	5	3569
21	1726	107	3792	111	4971	4	1877	88	5477	5	3225
6	1410	1	303	9	2050	5	1316	3	1388	13	4006
2	1409	2	300	1	2046	2	1321	1	1389	5	4000
1067	1395	205	296	1561	2044	1039	1295	1085	1384	3065	3966
1421	1887	2803	4072	3944	5411	1569	2062	4427	5967	2580	3555
10	1388	0	293	6	2037	3	1305	3	1379	5	3952
1061	1378	175	291	1556	2032	942	1288	1065	1378	2802	3905
1457	1890	2725	4051	4299	5396	1445	2055	4544	5945	2684	3542
2	1894	5	4066	9	5410	2	2065	8	5971	46	3559
2	1894	5	4063	7	5410	1	2064	14	5969	6	3559
2	1894	9	4067	5	5409	3	2064	10	5967	5	3557
6	1888	7	4056	10	5397	2	2057	42	5948	9	3544
4	1886	5	4064	9	5402	8	2059	24	5949	9	3542
63	1884	186	4057	124	5391	6	2054	166	5926	170	3518
2	774	0	232	0	1498	0	731	1	1167	1	1891
9	1885	5	4058	10	5397	4	2050	13	5925	2	3521
0	765	0	228	1	1483	0	721	1	1156	0	1836
0	760	0	229	0	1482	0	723	0	1154	1	1835
0	758	0	228	1	1483	0	720	0	1152	0	1826
25	1884	77	4057	35	5386	2	2049	54	5893	163	3507
5	1882	5	4051	19	5381	5	2046	14	5879	2	3496
6	1878	2	4041	14	5374	4	2045	22	5873	7	3493
4	1879	66	4042	12	5374	3	2046	14	5872	4	3494
9	1878	3	4040	9	5372	4	2046	17	5871	3	3491
22	1868	2	4024	27	5349	1	2038	72	5855	11	3484
48	1876	175	4029	170	5353	58	2039	236	5855	129	3485
2	1876	6	4033	6	5358	1	2042	4	5856	0	3479
0	1873	1	4014	0	5347	1	2040	0	5844	0	3471
0	1873	1	4013	1	5347	0	2041	0	5843	1	3471

chr17	45706808	G	0	1873	1	4006	2	5341	0	2040	0	5840	0	3470
chr17	45706809	G	1	1868	0	4009	1	5341	0	2041	0	5840	0	3470
chr17	45706813	G	1	1872	0	4008	0	5342	0	2038	0	5835	0	3466
chr17	45706820	G	0	1854	1	3967	0	5295	0	2024	1	5789	0	3435
chr19	24277358	c	1	1194	0	452	0	1472	0	808	1	3740	0	1193
chr19	24277364	c	0	1223	0	474	1	1481	0	852	0	3781	0	1244
chr19	24277365	c	0	1235	0	481	1	1492	0	875	1	3795	0	1279
chr19	24277367	c	0	1243	0	483	1	1495	0	888	0	3803	0	1295
chr19	24277368	c	0	1249	0	487	0	1503	0	908	0	3817	0	1337
chr19	24277394	c	3	1322	2	567	3	1555	3	1043	14	3934	3	1411
chr19	24277395	c	2	1323	2	567	7	1551	1	1044	10	3938	11	1402
chr19	24277396	c	3	1324	1	568	3	1555	3	1043	6	3942	2	1411
chr19	24277403	c	3	1326	1	568	2	1557	0	1047	14	3940	3	1412
chr19	24277409	c	4	1325	0	569	3	1558	0	1046	16	3940	3	1413
chr19	24277416	c	5	1325	1	569	5	1556	1	1046	3	3958	0	1417
chr19	24277417	c	8	1323	4	566	8	1552	3	1044	14	3947	1	1416
chr19	24277420	c	6	1325	1	569	6	1555	1	1046	14	3945	32	1385
chr19	24277425	c	4	1327	0	570	7	1555	2	1045	16	3943	29	1388
chr19	24277433	c	3	1329	0	568	4	1557	0	1047	8	3948	1	1412
chr19	24277441	c	8	1323	0	570	7	1557	28	1018	25	3937	25	1389
chr19	24277451	c	2	1331	0	571	4	1562	29	1019	6	3961	0	1414
chr19	24277455	c	1	1332	2	569	1	1565	2	1046	14	3954	2	1413
chr19	24277465	g	1	358	0	6560	1	2225	0	4064	0	2231	0	2299
chr19	24277466	c	3	1330	1	570	2	1564	0	1048	8	3962	0	1414
chr19	24277468	g	0	364	1	6620	1	2235	0	4109	0	2252	0	2317
chr19	24277469	g	0	365	1	6652	0	2241	0	4125	0	2264	1	2330
chr19	24277472	c	3	1330	2	569	2	1564	17	1030	16	3955	4	1411
chr19	24277474	c	10	1323	0	571	8	1559	2	1046	34	3938	1	1413
chr19	24277476	g	0	372	3	7013	1	2295	3	4310	2	2347	2	2437
chr19	24277477	c	2	1327	0	569	6	1559	0	1046	6	3961	14	1398
chr19	24277479	g	0	373	0	7036	0	2300	0	4332	0	2361	1	2453
chr19	24277480	c	1	720	3	316	1	886	1	580	6	2335	0	796
chr19	24277481	c	2	1123	1	485	2	1340	5	876	9	3406	2	1164
chr19	24277485	c	8	1325	1	569	3	1565	3	1043	7	3967	9	1406
chr19	24277486	c	2	1330	0	571	2	1566	1	1047	8	3966	1	1413
chr19	24277487	c	2	1331	1	570	3	1564	2	1045	9	3965	18	1397
chr19	24277488	c	1	1332	0	571	1	1567	1	1047	6	3969	4	1411
chr19	24277489	c	3	1330	0	571	3	1565	0	1048	15	3960	4	1411
chr19	24277494	c	14	1310	1	568	5	1560	4	1042	27	3936	2	1407
chr19	24277496	c	744	569	376	189	843	711	566	472	2188	1733	665	742
chr19	24277497	g	229	145	4249	2833	1515	791	2278	2085	1516	855	1516	956
chr19	24277502	g	0	374	5	7080	6	2301	5	4360	12	2360	2	2472
chr19	24277503	c	21	1311	2	568	9	1558	4	1042	45	3922	19	1396
chr19	24277506	c	9	869	5	378	25	941	0	693	25	2386	12	949
chr19	24277508	g	1	373	10	7078	6	2305	7	4361	4	2369	4	2472
chr19	24277510	c	2	279	1	145	2	249	0	278	3	532	1	385

0	1873	1	4007	2	5343	0	2040	0	5840	0	3470
1	1869	0	4009	1	5342	0	2041	0	5840	0	3470
1	1873	0	4008	0	5342	0	2038	0	5835	0	3466
0	1854	1	3968	0	5295	0	2024	1	5790	0	3435
1	1195	0	452	0	1472	0	808	1	3741	0	1193
0	1223	0	474	1	1482	0	852	0	3781	0	1244
0	1235	0	481	1	1493	0	875	1	3796	0	1279
0	1243	0	483	1	1496	0	888	0	3803	0	1295
0	1249	0	487	0	1503	0	908	0	3817	0	1337
3	1325	2	569	3	1558	3	1046	14	3948	3	1414
2	1325	2	569	7	1558	1	1045	10	3948	11	1413
3	1327	1	569	3	1558	3	1046	6	3948	2	1413
3	1329	1	569	2	1559	0	1047	14	3954	3	1415
4	1329	0	569	3	1561	0	1046	16	3956	3	1416
5	1330	1	570	5	1561	1	1047	3	3961	0	1417
8	1331	4	570	8	1560	3	1047	14	3961	1	1417
6	1331	1	570	6	1561	1	1047	14	3959	32	1417
4	1331	0	570	7	1562	2	1047	16	3959	29	1417
3	1332	0	568	4	1561	0	1047	8	3956	1	1413
8	1331	0	570	7	1564	28	1046	25	3962	25	1414
2	1333	0	571	4	1566	29	1048	6	3967	0	1414
1	1333	2	571	1	1566	2	1048	14	3968	2	1415
1	359	0	6560	1	2226	0	4064	0	2231	0	2299
3	1333	1	571	2	1566	0	1048	8	3970	0	1414
0	364	1	6621	1	2236	0	4109	0	2252	0	2317
0	365	1	6653	0	2241	0	4125	0	2264	1	2331
3	1333	2	571	2	1566	17	1047	16	3971	4	1415
10	1333	0	571	8	1567	2	1048	34	3972	1	1414
0	372	3	7016	1	2296	3	4313	2	2349	2	2439
2	1329	0	569	6	1565	0	1046	6	3967	14	1412
0	373	0	7036	0	2300	0	4332	0	2361	1	2454
1	721	3	319	1	887	1	581	6	2341	0	796
2	1125	1	486	2	1342	5	881	9	3415	2	1166
8	1333	1	570	3	1568	3	1046	7	3974	9	1415
2	1332	0	571	2	1568	1	1048	8	3974	1	1414
2	1333	1	571	3	1567	2	1047	9	3974	18	1415
1	1333	0	571	1	1568	1	1048	6	3975	4	1415
3	1333	0	571	3	1568	0	1048	15	3975	4	1415
14	1324	1	569	5	1565	4	1046	27	3963	2	1409
744	1313	376	565	843	1554	566	1038	2188	3921	665	1407
229	374	4249	7082	1515	2306	2278	4363	1516	2371	1516	2472
0	374	5	7085	6	2307	5	4365	12	2372	2	2474
21	1332	2	570	9	1567	4	1046	45	3967	19	1415
9	878	5	383	25	966	0	693	25	2411	12	961
1	374	10	7088	6	2311	7	4368	4	2373	4	2476
2	281	1	146	2	251	0	278	3	535	1	386

chr19	24277511	c	20	255	3	137	16	229	45	227	46	472	24	355
chr19	24277513	g	0	374	9	7079	6	2306	5	4368	4	2373	17	2461
chr19	24277514	g	4	371	5	7084	5	2308	11	4363	8	2370	5	2473
chr19	24277516	c	0	252	0	124	2	231	0	231	0	491	2	325
chr19	24277517	c	1	243	0	119	0	228	0	217	1	487	1	312
chr19	24277518	c	0	237	0	117	0	226	0	210	1	479	0	302
chr19	24277520	c	204	23	99	13	185	31	173	19	403	55	224	39
chr19	24277521	g	317	54	5573	1394	1978	297	3624	674	1977	366	2185	266
chr19	24277523	c	0	221	2	104	0	203	0	183	1	435	1	244
chr19	24277524	c	6	215	7	98	2	202	4	179	1	431	7	237
chr19	24277528	c	191	24	92	11	174	23	163	12	387	35	207	23
chr19	24277529	g	343	32	5981	1022	2056	228	3803	526	2102	265	2218	246
chr19	24277531	g	2	376	13	7076	8	2305	11	4365	9	2373	3	2480
chr19	24277532	g	2	376	13	7082	5	2310	7	4367	9	2375	4	2477
chr19	24277534	c	136	30	34	16	130	37	74	8	289	50	180	25
chr19	24277535	g	314	64	6242	847	2020	295	3825	540	2069	312	2215	265
chr19	24277537	c	135	24	31	9	145	17	61	7	281	46	181	24
chr19	24277538	g	333	49	6316	779	2073	248	4092	282	2087	305	2227	257
chr19	24277540	G	0	381	25	7068	2	2322	5	4372	13	2380	5	2477
chr19	24277542	C	2	163	0	46	1	165	0	91	1	317	6	200
chr19	24277545	g	1	381	41	7056	4	2319	4	4376	8	2387	9	2478
chr19	24277548	g	1	379	6	7093	5	2310	10	4366	11	2378	3	2474
chr19	24277550	c	151	14	41	7	147	15	82	3	268	40	195	11
chr19	24277551	g	336	44	6285	781	2116	189	3831	532	2105	279	2170	296
chr19	24277553	c	6	159	0	47	2	160	5	82	5	298	0	208
chr19	24277555	c	1	165	0	48	3	161	0	91	1	306	1	215
chr19	24277558	g	1	379	92	7003	5	2310	6	4371	8	2382	3	2475
chr19	24277560	c	191	20	93	23	180	20	170	28	350	56	261	38
chr19	24277561	g	323	57	6360	734	2050	266	3904	474	2084	307	2215	264
chr19	24277563	c	257	32	140	31	248	40	260	47	583	86	362	56
chr19	24277564	g	322	58	6034	1063	2052	264	3850	529	2076	313	2179	300
chr19	24277566	c	3	1311	2	564	3	1553	38	1007	10	3934	2	1396
chr19	24277569	c	44	1270	33	535	37	1520	18	1027	95	3846	43	1354
chr19	24277573	g	1	379	61	7035	3	2312	72	4309	8	2381	1	2475
chr19	24277576	g	0	380	70	7030	14	2304	10	4372	12	2384	17	2462
chr19	24277579	g	2	378	73	7028	20	2298	78	4305	20	2376	6	2473
chr19	24277582	g	2	378	10	7091	8	2310	10	4372	29	2370	4	2476
chr19	24277585	g	1	380	8	7097	13	2307	6	4375	7	2391	6	2477
chr19	24277588	g	5	375	120	6984	37	2282	51	4331	33	2366	37	2448
chr19	24277589	c	1188	72	491	55	1379	92	969	33	3488	274	1242	82
chr19	24277590	g	331	51	6606	501	2134	189	4132	255	2138	266	2308	183
chr19	24277592	c	1237	74	496	71	1428	117	1024	16	3625	293	1283	104
chr19	24277593	g	335	46	6191	927	2127	199	3844	550	2074	331	2325	166
chr19	24277595	c	1189	117	505	58	1372	163	959	76	3527	389	1212	171
chr19	24277596	g	334	49	6287	832	2130	197	3885	513	2085	324	2310	185
chr19	24277598	c	2	1304	0	566	16	1520	2	1034	24	3890	1	1383

20	275	3	140	16	245	45	272	46	518	24	379
0	374	9	7088	6	2312	5	4373	4	2377	17	2478
4	375	5	7089	5	2313	11	4374	8	2378	5	2478
0	252	0	124	2	233	0	231	0	491	2	327
1	244	0	119	0	228	0	217	1	488	1	313
0	237	0	117	0	226	0	210	1	480	0	302
204	227	99	112	185	216	173	192	403	458	224	263
317	371	5573	6967	1978	2275	3624	4298	1977	2343	2185	2451
0	221	2	106	0	203	0	183	1	436	1	245
6	221	7	105	2	204	4	183	1	432	7	244
191	215	92	103	174	197	163	175	387	422	207	230
343	375	5981	7003	2056	2284	3803	4329	2102	2367	2218	2464
2	378	13	7089	8	2313	11	4376	9	2382	3	2483
2	378	13	7095	5	2315	7	4374	9	2384	4	2481
136	166	34	50	130	167	74	82	289	339	180	205
314	378	6242	7089	2020	2315	3825	4365	2069	2381	2215	2480
135	159	31	40	145	162	61	68	281	327	181	205
333	382	6316	7095	2073	2321	4092	4374	2087	2392	2227	2484
0	381	25	7093	2	2324	5	4377	13	2393	5	2482
2	165	0	46	1	166	0	91	1	318	6	206
1	382	41	7097	4	2323	4	4380	8	2395	9	2487
1	380	6	7099	5	2315	10	4376	11	2389	3	2477
151	165	41	48	147	162	82	85	268	308	195	206
336	380	6285	7066	2116	2305	3831	4363	2105	2384	2170	2466
6	165	0	47	2	162	5	87	5	303	0	208
1	166	0	48	3	164	0	91	1	307	1	216
1	380	92	7095	5	2315	6	4377	8	2390	3	2478
191	211	93	116	180	200	170	198	350	406	261	299
323	380	6360	7094	2050	2316	3904	4378	2084	2391	2215	2479
257	289	140	171	248	288	260	307	583	669	362	418
322	380	6034	7097	2052	2316	3850	4379	2076	2389	2179	2479
3	1314	2	566	3	1556	38	1045	10	3944	2	1398
44	1314	33	568	37	1557	18	1045	95	3941	43	1397
1	380	61	7096	3	2315	72	4381	8	2389	1	2476
0	380	70	7100	14	2318	10	4382	12	2396	17	2479
2	380	73	7101	20	2318	78	4383	20	2396	6	2479
2	380	10	7101	8	2318	10	4382	29	2399	4	2480
1	381	8	7105	13	2320	6	4381	7	2398	6	2483
5	380	120	7104	37	2319	51	4382	33	2399	37	2485
1188	1260	491	546	1379	1471	969	1002	3488	3762	1242	1324
331	382	6606	7107	2134	2323	4132	4387	2138	2404	2308	2491
1237	1311	496	567	1428	1545	1024	1040	3625	3918	1283	1387
335	381	6191	7118	2127	2326	3844	4394	2074	2405	2325	2491
1189	1306	505	563	1372	1535	959	1035	3527	3916	1212	1383
334	383	6287	7119	2130	2327	3885	4398	2085	2409	2310	2495
2	1306	0	566	16	1536	2	1036	24	3914	1	1384

chr19	24277600	C	32	1272	0	564	12	1522	3	1032	45	3865	3	1375
chr19	24277604	C	8	1294	0	564	10	1521	0	1033	41	3867	2	1373
chr19	24277606	G	2	392	18	7103	6	2328	26	4391	10	2425	5	2511
chr19	24277607	G	1	398	10	7113	5	2332	7	4410	5	2437	2	2519
chr19	24277608	G	0	399	17	7105	8	2328	8	4405	18	2422	13	2506
chr19	24277610	G	5	394	127	6993	62	2274	3	4412	41	2398	96	2425
chr19	24277612	C	4	1229	0	551	18	1460	9	1008	34	3800	3	1297
chr19	24277614	C	16	1215	0	551	12	1462	2	1013	34	3792	24	1274
chr19	24277617	G	0	98	2	746	0	235	1	509	3	422	0	463
chr19	24277619	C	3	1219	19	529	12	1441	2	1006	17	3736	3	1289
chr19	24277622	C	2	1203	1	545	1	1456	0	1008	5	3785	1	1266
chr19	24277623	C	4	1201	0	546	3	1454	1	1007	20	3768	11	1255
chr19	24277625	C	9	1191	2	544	8	1446	3	1004	19	3764	3	1255
chr19	24277627	G	0	81	8	312	1	156	0	263	4	289	1	308
chr19	24277631	C	6	1188	0	542	8	1438	4	994	14	3757	2	1235
chr19	24277635	C	7	1182	1	544	6	1427	4	996	9	3701	2	1234
chr19	24277636	C	5	1184	34	511	8	1427	2	997	34	3679	33	1203
chr19	24277641	C	3	1174	3	542	8	1418	19	977	15	3691	5	1210
chr19	24277642	C	54	1123	0	545	14	1411	102	891	35	3672	31	1187
chr19	24277644	C	91	1087	59	485	46	1379	3	993	140	3566	46	1171
chr19	24277653	C	7	1171	2	542	2	1421	4	993	6	3697	1	1215
chr19	24277654	C	3	1174	0	543	7	1416	2	995	8	3696	8	1207
chr19	24277664	G	2	360	10	6986	7	2286	9	4244	25	2291	5	2272
chr19	24277668	G	1	361	42	6929	16	2278	13	4241	9	2305	63	2211
chr19	24277674	G	6	356	244	6716	36	2257	12	4239	53	2256	54	2219
chr19	24277675	G	0	362	16	6947	3	2291	12	4238	12	2297	11	2262
chr19	24277677	C	4	1170	4	538	4	1418	0	996	8	3695	1	1214
chr19	24277681	G	1	361	10	6943	20	2273	2	4241	16	2290	24	2246
chr19	24277686	G	5	357	9	6945	20	2272	5	4245	17	2289	4	2266
chr19	24277690	C	1010	163	464	70	1239	180	694	296	3252	444	1075	140
chr19	24277691	G	296	64	6110	846	1960	315	3584	661	1909	391	2003	259
chr19	24277692	C	0	1167	0	528	0	1416	0	963	0	3696	0	1208
chr19	24277694	C	1	1166	0	529	0	1415	0	964	0	3696	0	1210
chr19	24277696	G	0	357	15	6939	7	2266	8	4234	6	2291	1	2260
chr19	24277698	G	1	353	9	6928	4	2270	10	4229	8	2285	4	2254
chr19	24277699	C	0	1166	0	527	0	1414	0	963	1	3692	0	1208
chr19	24277702	G	0	356	10	6932	15	2259	2	4243	9	2285	5	2256
chr19	24277705	G	2	341	90	6851	13	2246	63	4179	35	2243	1	2242
chr19	24277707	G	3	340	51	6887	58	2201	64	4176	35	2242	15	2225
chr19	24277708	G	0	341	7	6932	6	2253	10	4230	10	2268	3	2238
chr19	24277709	G	0	343	11	6928	2	2257	2	4239	4	2272	3	2240
chr19	24277713	C	0	1066	0	418	1	1325	0	775	0	3449	0	1032
chr19	24277714	C	0	1050	0	405	0	1304	0	756	0	3382	0	999
chr19	24277726	G	1	336	9	6923	8	2237	6	4229	7	2247	2	2238
chr19	24277731	G	0	337	25	6903	7	2238	6	4230	4	2248	3	2236
chr19	24277732	G	0	334	10	6920	0	2239	4	4214	4	2242	4	2227

32	1304	0	564	12	1534	3	1035	45	3910	3	1378
8	1302	0	564	10	1531	0	1033	41	3908	2	1375
2	394	18	7121	6	2334	26	4417	10	2435	5	2516
1	399	10	7123	5	2337	7	4417	5	2442	2	2521
0	399	17	7122	8	2336	8	4413	18	2440	13	2519
5	399	127	7120	62	2336	3	4415	41	2439	96	2521
4	1233	0	551	18	1478	9	1017	34	3834	3	1300
16	1231	0	551	12	1474	2	1015	34	3826	24	1298
0	98	2	748	0	235	1	510	3	425	0	463
3	1222	19	548	12	1453	2	1008	17	3753	3	1292
2	1205	1	546	1	1457	0	1008	5	3790	1	1267
4	1205	0	546	3	1457	1	1008	20	3788	11	1266
9	1200	2	546	8	1454	3	1007	19	3783	3	1258
0	81	8	320	1	157	0	263	4	293	1	309
6	1194	0	542	8	1446	4	998	14	3771	2	1237
7	1189	1	545	6	1433	4	1000	9	3710	2	1236
5	1189	34	545	8	1435	2	999	34	3713	33	1236
3	1177	3	545	8	1426	19	996	15	3706	5	1215
54	1177	0	545	14	1425	102	993	35	3707	31	1218
91	1178	59	544	46	1425	3	996	140	3706	46	1217
7	1178	2	544	2	1423	4	997	6	3703	1	1216
3	1177	0	543	7	1423	2	997	8	3704	8	1215
2	362	10	6996	7	2293	9	4253	25	2316	5	2277
1	362	42	6971	16	2294	13	4254	9	2314	63	2274
6	362	244	6960	36	2293	12	4251	53	2309	54	2273
0	362	16	6963	3	2294	12	4250	12	2309	11	2273
4	1174	4	542	4	1422	0	996	8	3703	1	1215
1	362	10	6953	20	2293	2	4243	16	2306	24	2270
5	362	9	6954	20	2292	5	4250	17	2306	4	2270
1010	1173	464	534	1239	1419	694	990	3252	3696	1075	1215
296	360	6110	6956	1960	2275	3584	4245	1909	2300	2003	2262
0	1167	0	528	0	1416	0	963	0	3696	0	1208
1	1167	0	529	0	1415	0	964	0	3696	0	1210
0	357	15	6954	7	2273	8	4242	6	2297	1	2261
1	354	9	6937	4	2274	10	4239	8	2293	4	2258
0	1166	0	527	0	1414	0	963	1	3693	0	1208
0	356	10	6942	15	2274	2	4245	9	2294	5	2261
2	343	90	6941	13	2259	63	4242	35	2278	1	2243
3	343	51	6938	58	2259	64	4240	35	2277	15	2240
0	341	7	6939	6	2259	10	4240	10	2278	3	2241
0	343	11	6939	2	2259	2	4241	4	2276	3	2243
0	1066	0	418	1	1326	0	775	0	3449	0	1032
0	1050	0	405	0	1304	0	756	0	3382	0	999
1	337	9	6932	8	2245	6	4235	7	2254	2	2240
0	337	25	6928	7	2245	6	4236	4	2252	3	2239
0	334	10	6930	0	2239	4	4218	4	2246	4	2231

chr19	24277739	g	298	37	6394	534	2073	170	3977	258	2039	207	2077	160
chr19	24277741	g	308	22	6457	470	2094	133	3978	235	2091	140	2163	58
chr19	24277745	g	11	319	64	6855	56	2168	75	4135	54	2176	77	2142
chr19	24277754	g	3	327	4	6921	29	2193	2	4207	31	2197	11	2207
chr19	24277757	g	0	330	8	6916	5	2218	6	4202	10	2216	13	2204
chr19	24277758	g	0	330	6	6914	9	2213	3	4201	5	2220	2	2213
chr19	24277761	g	2	328	6	6914	4	2218	4	4201	13	2212	2	2213
chr19	24277775	g	0	324	1	6914	0	2207	0	4195	0	2212	0	2209
chr19	24277778	g	0	330	0	6906	1	2216	0	4198	0	2219	0	2214
chr19	24277779	g	0	330	2	6909	0	2217	0	4197	0	2217	0	2214
chr19	24277783	g	0	330	0	6905	1	2213	3	4191	1	2217	0	2208
chr4	136547319	G	0	5178	1	5305	1	8654	0	4806	3	24677	2	12199
chr4	136547320	G	2	5175	1	5310	0	8656	2	4807	5	24682	5	12198
chr4	136547321	G	1	5185	0	5320	0	8667	1	4819	5	24705	7	12203
chr4	136547327	G	1	5198	2	5339	2	8688	1	4851	7	24730	5	12220
chr4	136547332	G	1	5203	2	5342	1	8695	0	4854	4	24754	0	12236
chr4	136547333	G	1	5203	0	5347	2	8695	0	4855	1	24760	1	12237
chr4	136547338	G	4	5211	7	5348	23	8682	5	4881	92	24676	37	12203
chr4	136547340	G	48	5167	4	5354	7	8698	39	4851	105	24665	14	12232
chr4	136547344	G	13	5205	6	5356	47	8660	4	4886	116	24660	20	12229
chr4	136547349	G	9	5215	98	5267	33	8682	5	4892	33	24762	27	12235
chr4	136547354	G	25	5201	255	5108	58	8660	4	4894	102	24689	122	12141
chr4	136547358	G	51	5177	8	5360	21	8698	43	4857	107	24696	287	11975
chr4	136547359	G	19	5212	9	5356	20	8699	39	4861	55	24744	20	12244
chr4	136547364	G	5	5228	15	5354	20	8703	9	4889	50	24752	27	12232
chr4	136547365	G	4	5228	12	5354	25	8696	12	4889	36	24755	19	12244
chr4	136547374	G	35	5238	11	5364	80	8689	128	4793	150	24738	19	12332
chr4	136547376	G	35	5240	11	5365	165	8606	1	4920	210	24687	196	12147
chr4	136547377	C	0	966	2	1562	0	1839	0	881	0	2661	1	1948
chr4	136547378	C	0	968	0	1566	1	1848	0	885	0	2670	0	1958
chr4	136547381	G	21	5256	11	5366	24	8751	10	4911	123	24773	36	12318
chr4	136547383	G	437	4838	279	5099	789	7986	132	4787	2067	22832	933	11424
chr4	136547385	G	216	5062	398	4977	311	8464	243	4677	681	24220	234	12125
chr4	136547386	G	28	5249	7	5370	58	8718	17	4905	62	24838	174	12183
chr4	136547387	C	0	980	0	1591	0	1863	0	896	1	2705	0	1978
chr4	136547388	C	1	981	1	1595	0	1868	0	897	0	2705	1	1982
chr4	136547390	G	126	5140	8	5361	129	8641	4	4919	233	24641	196	12149
chr4	136547392	G	10	5269	17	5363	20	8760	11	4916	61	24848	123	12236
chr4	136547393	G	6	5271	10	5363	48	8734	15	4912	150	24746	37	12318
chr4	136547394	C	0	979	0	1596	1	1869	0	898	0	2708	0	1985
chr4	136547396	G	384	4894	286	5094	715	8066	331	4597	1261	23647	1172	11189
chr4	136547399	G	86	5192	64	5317	82	8702	85	4844	271	24640	200	12160
chr4	136547402	G	238	5041	15	5366	415	8372	171	4758	678	24233	412	11948
chr4	136547405	G	77	5210	74	5309	108	8681	16	4918	250	24658	597	11762
chr4	136547408	C	889	92	1527	71	1752	121	825	73	2547	165	1901	87
chr4	136547409	G	5096	200	5141	253	8505	303	4817	130	23997	914	11869	493

298	335	6394	6928	2073	2243	3977	4235	2039	2246	2077	2237
308	330	6457	6927	2094	2227	3978	4213	2091	2231	2163	2221
11	330	64	6919	56	2224	75	4210	54	2230	77	2219
3	330	4	6925	29	2222	2	4209	31	2228	11	2218
0	330	8	6924	5	2223	6	4208	10	2226	13	2217
0	330	6	6920	9	2222	3	4204	5	2225	2	2215
2	330	6	6920	4	2222	4	4205	13	2225	2	2215
0	324	1	6915	0	2207	0	4195	0	2212	0	2209
0	330	0	6906	1	2217	0	4198	0	2219	0	2214
0	330	2	6911	0	2217	0	4197	0	2217	0	2214
0	330	0	6905	1	2214	3	4194	1	2218	0	2208
0	5178	1	5306	1	8655	0	4806	3	24680	2	12201
2	5177	1	5311	0	8656	2	4809	5	24687	5	12203
1	5186	0	5320	0	8667	1	4820	5	24710	7	12210
1	5199	2	5341	2	8690	1	4852	7	24737	5	12225
1	5204	2	5344	1	8696	0	4854	4	24758	0	12236
1	5204	0	5347	2	8697	0	4855	1	24761	1	12238
4	5215	7	5355	23	8705	5	4886	92	24768	37	12240
48	5215	4	5358	7	8705	39	4890	105	24770	14	12246
13	5218	6	5362	47	8707	4	4890	116	24776	20	12249
9	5224	98	5365	33	8715	5	4897	33	24795	27	12262
25	5226	255	5363	58	8718	4	4898	102	24791	122	12263
51	5228	8	5368	21	8719	43	4900	107	24803	287	12262
19	5231	9	5365	20	8719	39	4900	55	24799	20	12264
5	5233	15	5369	20	8723	9	4898	50	24802	27	12259
4	5232	12	5366	25	8721	12	4901	36	24791	19	12263
35	5273	11	5375	80	8769	128	4921	150	24888	19	12351
35	5275	11	5376	165	8771	1	4921	210	24897	196	12343
0	966	2	1564	0	1839	0	881	0	2661	1	1949
0	968	0	1566	1	1849	0	885	0	2670	0	1958
21	5277	11	5377	24	8775	10	4921	123	24896	36	12354
437	5275	279	5378	789	8775	132	4919	2067	24899	933	12357
216	5278	398	5375	311	8775	243	4920	681	24901	234	12359
28	5277	7	5377	58	8776	17	4922	62	24900	174	12357
0	980	0	1591	0	1863	0	896	1	2706	0	1978
1	982	1	1596	0	1868	0	897	0	2705	1	1983
126	5266	8	5369	129	8770	4	4923	233	24874	196	12345
10	5279	17	5380	20	8780	11	4927	61	24909	123	12359
6	5277	10	5373	48	8782	15	4927	150	24896	37	12355
0	979	0	1596	1	1870	0	898	0	2708	0	1985
384	5278	286	5380	715	8781	331	4928	1261	24908	1172	12361
86	5278	64	5381	82	8784	85	4929	271	24911	200	12360
238	5279	15	5381	415	8787	171	4929	678	24911	412	12360
77	5287	74	5383	108	8789	16	4934	250	24908	597	12359
889	981	1527	1598	1752	1873	825	898	2547	2712	1901	1988
5096	5296	5141	5394	8505	8808	4817	4947	23997	24911	11869	12362

chr4	136547412	C	4	978	10	1588	9	1865	1	897	24	2689	6	1982
chr4	136547414	G	1299	4009	778	4622	2201	6612	1517	3435	5128	19789	2970	9389
chr4	136547416	G	157	5125	57	5299	198	8572	8	4905	517	24242	291	11987
chr4	136547417	G	8	5304	8	5395	35	8784	9	4941	60	24850	38	12326
chr4	136547420	G	8	5308	12	5394	12	8813	2	4954	37	24885	42	12333
chr4	136547421	G	13	5302	8	5395	14	8814	11	4946	77	24843	22	12351
chr4	136547423	G	709	4614	1167	4239	1170	7658	759	4201	3413	21516	2305	10071
chr4	136547425	G	689	4633	600	4798	1155	7671	497	4461	2736	22176	1295	11065
chr4	136547426	C	6	975	1	1595	4	1868	9	890	15	2698	3	1985
chr4	136547428	G	23	5311	12	5402	13	8833	8	4961	76	24871	15	12373
chr4	136547429	G	7	5329	5	5411	32	8810	3	4966	22	24931	16	12376
chr4	136547431	G	432	4913	130	5292	675	8182	405	4569	1544	23411	900	11501
chr4	136547432	G	8	5348	8	5417	13	8856	12	4967	43	24917	28	12383
chr4	136547435	G	338	5011	207	5194	390	8449	325	4641	1245	23584	951	11420
chr4	136547436	G	15	5365	44	5393	55	8830	9	4990	90	24892	21	12410
chr4	136547438	G	49	5335	234	5205	91	8799	235	4771	204	24792	186	12266
chr4	136547440	G	9	5402	12	5437	14	8893	11	5013	93	24966	20	12476
chr4	136547441	G	12	5426	6	5457	7	8935	6	5034	65	25050	21	12527
chr4	136547442	C	0	982	5	1592	4	1872	1	897	11	2706	0	1989
chr4	136547443	C	846	135	1270	326	1605	270	818	81	2308	410	1833	157
chr4	136547444	G	5106	370	4874	600	8522	456	4763	301	23684	1486	11528	1070
chr4	136547447	G	10	5474	2	5480	24	8968	10	5068	77	25115	10	12607
chr4	136547448	G	8	5476	5	5477	11	8981	8	5070	45	25147	15	12605
chr4	136547449	C	2	979	5	1593	10	1865	4	895	9	2710	5	1985
chr4	136547452	G	11	5527	5	5508	18	9019	43	5074	52	25216	72	12668
chr4	136547453	C	2	980	1	1596	11	1866	0	899	18	2700	1	1988
chr4	136547454	C	919	63	1536	62	1723	154	821	77	2580	137	1883	107
chr4	136547455	G	5220	347	5091	433	8625	426	4773	363	23924	1367	12048	766
chr4	136547456	G	16	5561	125	5404	100	8959	6	5141	106	25215	115	12716
chr4	136547459	G	12	5569	11	5521	14	9039	5	5139	67	25229	20	12798
chr4	136547460	C	1	952	4	1539	4	1821	2	870	5	2606	2	1930
chr4	136547461	C	1	979	1	1594	8	1867	3	895	2	2717	0	1990
chr4	136547462	C	2	978	5	1590	1	1876	0	899	8	2708	2	1988
chr4	136547466	C	2	979	3	1593	1	1876	1	898	9	2709	2	1987
chr4	136547467	C	9	973	1	1596	6	1870	1	898	23	2696	0	1990
chr4	136547469	C	53	929	0	1597	135	1742	117	782	92	2627	374	1615
chr4	136547471	G	20	5638	21	5521	24	9103	5	5192	99	25458	21	13058
chr4	136547473	C	0	982	2	1595	3	1870	0	898	4	2716	4	1987
chr4	136547474	C	0	982	2	1596	1	1876	61	838	14	2705	0	1992
chr4	136547475	C	1	981	2	1597	0	1876	1	898	2	2718	98	1893
chr4	136547478	C	46	936	0	1599	112	1765	22	877	110	2609	178	1814
chr4	136547480	G	12	5637	9	5533	37	9085	46	5144	57	25457	86	12984
chr4	136547482	C	0	982	1	1598	7	1871	0	899	2	2717	1	1992
chr4	136547487	G	10	5635	7	5532	15	9106	2	5185	57	25437	105	12957
chr4	136547488	G	11	5632	6	5532	12	9105	8	5176	45	25443	20	13041
chr4	136547489	C	7	981	4	1593	2	1875	0	902	12	2718	2	1988

4	982	10	1598	9	1874	1	898	24	2713	6	1988
1299	5308	778	5400	2201	8813	1517	4952	5128	24917	2970	12359
157	5282	57	5356	198	8770	8	4913	517	24759	291	12278
8	5312	8	5403	35	8819	9	4950	60	24910	38	12364
8	5316	12	5406	12	8825	2	4956	37	24922	42	12375
13	5315	8	5403	14	8828	11	4957	77	24920	22	12373
709	5323	1167	5406	1170	8828	759	4960	3413	24929	2305	12376
689	5322	600	5398	1155	8826	497	4958	2736	24912	1295	12360
6	981	1	1596	4	1872	9	899	15	2713	3	1988
23	5334	12	5414	13	8846	8	4969	76	24947	15	12388
7	5336	5	5416	32	8842	3	4969	22	24953	16	12392
432	5345	130	5422	675	8857	405	4974	1544	24955	900	12401
8	5356	8	5425	13	8869	12	4979	43	24960	28	12411
338	5349	207	5401	390	8839	325	4966	1245	24829	951	12371
15	5380	44	5437	55	8885	9	4999	90	24982	21	12431
49	5384	234	5439	91	8890	235	5006	204	24996	186	12452
9	5411	12	5449	14	8907	11	5024	93	25059	20	12496
12	5438	6	5463	7	8942	6	5040	65	25115	21	12548
0	982	5	1597	4	1876	1	898	11	2717	0	1989
846	981	1270	1596	1605	1875	818	899	2308	2718	1833	1990
5106	5476	4874	5474	8522	8978	4763	5064	23684	25170	11528	12598
10	5484	2	5482	24	8992	10	5078	77	25192	10	12617
8	5484	5	5482	11	8992	8	5078	45	25192	15	12620
2	981	5	1598	10	1875	4	899	9	2719	5	1990
11	5538	5	5513	18	9037	43	5117	52	25268	72	12740
2	982	1	1597	11	1877	0	899	18	2718	1	1989
919	982	1536	1598	1723	1877	821	898	2580	2717	1883	1990
5220	5567	5091	5524	8625	9051	4773	5136	23924	25291	12048	12814
16	5577	125	5529	100	9059	6	5147	106	25321	115	12831
12	5581	11	5532	14	9053	5	5144	67	25296	20	12818
1	953	4	1543	4	1825	2	872	5	2611	2	1932
1	980	1	1595	8	1875	3	898	2	2719	0	1990
2	980	5	1595	1	1877	0	899	8	2716	2	1990
2	981	3	1596	1	1877	1	899	9	2718	2	1989
9	982	1	1597	6	1876	1	899	23	2719	0	1990
53	982	0	1597	135	1877	117	899	92	2719	374	1989
20	5658	21	5542	24	9127	5	5197	99	25557	21	13079
0	982	2	1597	3	1873	0	898	4	2720	4	1991
0	982	2	1598	1	1877	61	899	14	2719	0	1992
1	982	2	1599	0	1876	1	899	2	2720	98	1991
46	982	0	1599	112	1877	22	899	110	2719	178	1992
12	5649	9	5542	37	9122	46	5190	57	25514	86	13070
0	982	1	1599	7	1878	0	899	2	2719	1	1993
10	5645	7	5539	15	9121	2	5187	57	25494	105	13062
11	5643	6	5538	12	9117	8	5184	45	25488	20	13061
7	988	4	1597	2	1877	0	902	12	2730	2	1990

chr4	136547492	G	106	5538	207	5329	187	8930	216	4974	569	24915	208	12851
chr4	136547494	C	2	987	2	1596	1	1880	1	904	4	2734	4	1994
chr4	136547495	C	3	986	1	1598	8	1873	5	900	6	2732	7	1992
chr4	136547496	C	2	986	3	1597	5	1875	2	903	3	2735	2	1997
chr4	136547497	C	912	73	1370	229	1799	84	877	27	2448	276	1757	242
chr4	136547498	G	5246	393	4976	553	8436	674	4892	290	23577	1888	11917	1135
chr4	136547499	G	9	5629	3	5525	23	9084	10	5169	84	25377	19	13031
chr4	136547501	G	463	5172	451	5075	584	8524	203	4976	1860	23599	1399	11646
chr4	136547503	G	57	5577	79	5443	56	9049	64	5114	269	25177	222	12818
chr4	136547504	G	9	5627	9	5516	12	9095	8	5171	69	25379	23	13017
chr4	136547505	G	15	5618	9	5514	21	9084	9	5170	99	25355	61	12980
chr4	136547507	C	4	987	43	1557	5	1878	2	904	28	2716	3	1994
chr4	136547508	C	5	985	0	1601	2	1881	2	904	7	2738	3	1996
chr4	136547510	C	1	990	0	1600	9	1874	60	846	11	2734	1	1996
chr4	136547512	C	3	988	3	1598	5	1877	1	905	16	2728	1	1999
chr4	136547515	G	13	5617	9	5512	6	9090	6	5167	48	25361	9	13010
chr4	136547516	C	7	983	2	1598	21	1862	4	902	19	2727	9	1990
chr4	136547517	C	938	52	1558	43	1769	113	855	50	2638	108	1935	68
chr4	136547518	G	5324	306	4977	543	8421	674	4790	381	23702	1716	12066	963
chr4	136547519	G	4	5627	5	5517	16	9080	6	5164	34	25383	15	13014
chr4	136547520	G	18	5614	22	5500	46	9052	13	5160	85	25319	34	12994
chr4	136547524	G	21	5605	41	5477	15	9078	7	5165	70	25329	16	13005
chr4	136547525	C	0	177	0	206	0	201	1	146	1	440	1	346
chr4	136547527	C	120	9	106	2	115	10	99	2	274	7	224	23
chr4	136547528	G	5287	343	5144	377	8626	473	4872	301	23993	1402	12382	645
chr4	136547531	C	103	13	92	1	104	10	91	2	248	11	216	18
chr4	136547532	G	5434	196	5157	363	8744	352	5086	84	24447	947	12418	607
chr4	136547534	C	0	110	0	83	0	108	0	92	1	243	1	226
chr4	136547536	G	134	5490	31	5488	144	8951	221	4947	546	24825	68	12950
chr4	136547537	G	6	5621	8	5512	19	9076	4	5167	62	25332	20	13001
chr4	136547538	G	18	5609	7	5512	22	9070	14	5157	76	25318	28	12989
chr4	136547539	C	100	8	76	4	95	10	79	11	225	7	200	20
chr4	136547540	G	5401	225	5222	298	8660	433	5057	113	24361	1030	12525	492
chr4	136547543	C	0	106	4	75	0	105	0	90	5	225	0	221
chr4	136547545	C	99	6	78	2	101	5	78	12	220	7	197	19
chr4	136547546	G	5437	188	5196	321	8756	335	4887	278	24350	1029	12547	462
chr4	136547547	G	15	5607	5	5499	28	9047	36	5121	67	25281	21	12969
chr4	136547548	C	98	7	77	2	96	8	79	9	215	7	209	9
chr4	136547549	G	5293	329	4940	574	8662	430	4924	244	24050	1324	12600	408
chr4	136547551	C	0	95	0	80	0	106	1	87	0	200	0	206
chr4	136547553	C	2	84	0	76	4	96	0	89	7	175	11	185
chr4	136547555	G	57	5564	208	5307	198	8893	5	5161	429	24943	13	12994
chr4	136547558	C	3	80	8	66	6	89	0	83	13	157	6	185
chr4	136547560	G	112	5506	368	5148	186	8902	260	4905	457	24908	326	12677
chr4	136547561	G	6	5608	8	5506	23	9065	4	5160	144	25210	19	12975
chr4	136547562	C	3	80	0	79	0	91	0	91	1	180	3	199

106	5644	207	5536	187	9117	216	5190	569	25484	208	13059
2	989	2	1598	1	1881	1	905	4	2738	4	1998
3	989	1	1599	8	1881	5	905	6	2738	7	1999
2	988	3	1600	5	1880	2	905	3	2738	2	1999
912	985	1370	1599	1799	1883	877	904	2448	2724	1757	1999
5246	5639	4976	5529	8436	9110	4892	5182	23577	25465	11917	13052
9	5638	3	5528	23	9107	10	5179	84	25461	19	13050
463	5635	451	5526	584	9108	203	5179	1860	25459	1399	13045
57	5634	79	5522	56	9105	64	5178	269	25446	222	13040
9	5636	9	5525	12	9107	8	5179	69	25448	23	13040
15	5633	9	5523	21	9105	9	5179	99	25454	61	13041
4	991	43	1600	5	1883	2	906	28	2744	3	1997
5	990	0	1601	2	1883	2	906	7	2745	3	1999
1	991	0	1600	9	1883	60	906	11	2745	1	1997
3	991	3	1601	5	1882	1	906	16	2744	1	2000
13	5630	9	5521	6	9096	6	5173	48	25409	9	13019
7	990	2	1600	21	1883	4	906	19	2746	9	1999
938	990	1558	1601	1769	1882	855	905	2638	2746	1935	2003
5324	5630	4977	5520	8421	9095	4790	5171	23702	25418	12066	13029
4	5631	5	5522	16	9096	6	5170	34	25417	15	13029
18	5632	22	5522	46	9098	13	5173	85	25404	34	13028
21	5626	41	5518	15	9093	7	5172	70	25399	16	13021
0	177	0	206	0	201	1	147	1	441	1	347
120	129	106	108	115	125	99	101	274	281	224	247
5287	5630	5144	5521	8626	9099	4872	5173	23993	25395	12382	13027
103	116	92	93	104	114	91	93	248	259	216	234
5434	5630	5157	5520	8744	9096	5086	5170	24447	25394	12418	13025
0	110	0	83	0	108	0	92	1	244	1	227
134	5624	31	5519	144	9095	221	5168	546	25371	68	13018
6	5627	8	5520	19	9095	4	5171	62	25394	20	13021
18	5627	7	5519	22	9092	14	5171	76	25394	28	13017
100	108	76	80	95	105	79	90	225	232	200	220
5401	5626	5222	5520	8660	9093	5057	5170	24361	25391	12525	13017
0	106	4	79	0	105	0	90	5	230	0	221
99	105	78	80	101	106	78	90	220	227	197	216
5437	5625	5196	5517	8756	9091	4887	5165	24350	25379	12547	13009
15	5622	5	5504	28	9075	36	5157	67	25348	21	12990
98	105	77	79	96	104	79	88	215	222	209	218
5293	5622	4940	5514	8662	9092	4924	5168	24050	25374	12600	13008
0	95	0	80	0	106	1	88	0	200	0	206
2	86	0	76	4	100	0	89	7	182	11	196
57	5621	208	5515	198	9091	5	5166	429	25372	13	13007
3	83	8	74	6	95	0	83	13	170	6	191
112	5618	368	5516	186	9088	260	5165	457	25365	326	13003
6	5614	8	5514	23	9088	4	5164	144	25354	19	12994
3	83	0	79	0	91	0	91	1	181	3	202

chr4	136547564	C	79	3	72	6	86	5	82	2	159	15	184	17
chr4	136547565	G	5368	248	5407	105	8696	388	4912	249	24314	1030	12242	755
chr4	136547566	C	79	1	70	5	80	10	76	11	155	11	189	11
chr4	136547567	G	5327	285	5132	379	8747	339	4842	317	24409	937	12216	778
chr4	136547570	C	0	73	0	72	0	90	0	87	0	142	0	203
chr4	136547576	C	0	65	0	72	1	92	1	85	1	130	0	199
chr4	136547577	C	0	65	0	72	0	93	0	87	0	127	2	197
chr4	136547578	C	0	65	0	72	1	93	0	86	0	128	0	200
chr4	136547580	G	64	5537	7	5497	99	8959	6	5137	340	24902	291	12657
chr4	136547581	C	0	70	0	75	2	107	9	84	4	123	1	223
chr4	136547585	C	66	5	74	5	101	14	85	9	115	13	211	26
chr4	136547586	G	4917	678	4802	701	7977	1071	4295	847	22345	2875	10884	2065
chr4	136547587	C	0	71	0	78	3	112	0	94	0	128	0	237
chr4	136547589	C	0	71	0	78	3	113	0	95	3	124	30	207
chr4	136547591	G	1	5576	2	5494	2	9008	1	5123	2	25127	3	12918
chr4	136547592	G	0	5576	0	5496	3	9006	0	5123	3	25118	2	12920
chr4	136547594	G	1	5571	0	5489	2	8998	1	5116	6	25093	0	12915
chr4	136547596	G	1	5571	1	5487	3	8990	0	5116	4	25086	1	12911
chr4	136547598	G	0	5565	0	5482	1	8987	0	5109	4	25067	0	12903
chr4	136547600	G	0	5566	1	5481	0	8985	0	5107	1	25056	3	12900
chr4	136547602	G	1	5566	1	5473	2	8976	1	5100	1	25048	0	12889
chr4	136547604	G	0	5545	1	5463	1	8959	1	5095	2	24997	1	12869
chr4	136547605	C	0	46	0	62	0	91	0	68	0	106	0	191
chr4	136547607	C	2	38	0	56	1	87	13	46	4	89	3	168
chr4	136547611	C	0	38	0	55	0	87	2	58	0	90	0	169
chr4	136547612	C	0	38	0	55	0	88	0	60	0	90	0	170
chr4	136547616	C	0	41	0	65	0	91	0	66	0	92	0	181
chr4	136547618	C	0	41	1	64	0	91	0	67	0	92	0	183
chr4	136547624	C	0	40	1	64	3	91	0	65	2	101	1	185
chr4	136547626	C	0	40	0	65	1	89	0	65	0	102	0	180
chr4	136547628	C	0	40	0	64	0	91	0	66	0	102	0	180
chr4	136547629	C	0	40	3	62	1	89	0	66	2	100	0	181
chr4	136547632	C	0	44	0	70	1	96	1	74	0	110	1	194
chr4	136547636	C	1	92	0	180	2	180	2	114	2	212	0	298
chr4	136547641	C	2	891	4	1539	3	1813	1	826	5	2559	1	1814
chr4	136547642	C	0	894	1	1543	2	1814	1	826	4	2560	3	1809
chr4	136547645	C	9	884	0	1542	16	1798	0	826	17	2545	0	1813
chr4	136547653	C	9	883	2	1539	9	1800	0	825	22	2535	97	1714
chr4	136547655	C	18	875	55	1482	40	1767	2	822	98	2460	182	1625
chr4	136547657	C	21	871	93	1443	69	1736	77	744	122	2438	187	1618
chr4	136547659	C	3	890	4	1531	4	1801	1	823	9	2544	2	1806
chr4	136547660	C	2	891	4	1531	1	1805	0	824	1	2558	3	1804
chr4	136547671	C	4	887	3	1532	1	1805	0	822	8	2550	1	1804
chr4	136547673	C	7	885	4	1531	34	1772	2	818	24	2534	2	1802
chr4	136547677	C	3	889	1	1534	5	1800	0	821	7	2551	1	1801
chr4	136547684	C	26	863	3	1530	25	1778	15	804	30	2527	3	1793

79	82	72	78	86	91	82	84	159	174	184	201
5368	5616	5407	5512	8696	9084	4912	5161	24314	25344	12242	12997
79	80	70	75	80	90	76	87	155	166	189	200
5327	5612	5132	5511	8747	9086	4842	5159	24409	25346	12216	12994
0	73	0	72	0	90	0	87	0	142	0	203
0	65	0	72	1	93	1	86	1	131	0	199
0	65	0	72	0	93	0	87	0	127	2	199
0	65	0	72	1	94	0	86	0	128	0	200
64	5601	7	5504	99	9058	6	5143	340	25242	291	12948
0	70	0	75	2	109	9	93	4	127	1	224
66	71	74	79	101	115	85	94	115	128	211	237
4917	5595	4802	5503	7977	9048	4295	5142	22345	25220	10884	12949
0	71	0	78	3	115	0	94	0	128	0	237
0	71	0	78	3	116	0	95	3	127	30	237
1	5577	2	5496	2	9010	1	5124	2	25129	3	12921
0	5576	0	5496	3	9009	0	5123	3	25121	2	12922
1	5572	0	5489	2	9000	1	5117	6	25099	0	12915
1	5572	1	5488	3	8993	0	5116	4	25090	1	12912
0	5565	0	5482	1	8988	0	5109	4	25071	0	12903
0	5566	1	5482	0	8985	0	5107	1	25057	3	12903
1	5567	1	5474	2	8978	1	5101	1	25049	0	12889
0	5545	1	5464	1	8960	1	5096	2	24999	1	12870
0	46	0	62	0	91	0	68	0	106	0	191
2	40	0	56	1	88	13	59	4	93	3	171
0	38	0	55	0	87	2	60	0	90	0	169
0	38	0	55	0	88	0	60	0	90	0	170
0	41	0	65	0	91	0	66	0	92	0	181
0	41	1	65	0	91	0	67	0	92	0	183
0	40	1	65	3	94	0	65	2	103	1	186
0	40	0	65	1	90	0	65	0	102	0	180
0	40	0	64	0	91	0	66	0	102	0	180
0	40	3	65	1	90	0	66	2	102	0	181
0	44	0	70	1	97	1	75	0	110	1	195
1	93	0	180	2	182	2	116	2	214	0	298
2	893	4	1543	3	1816	1	827	5	2564	1	1815
0	894	1	1544	2	1816	1	827	4	2564	3	1812
9	893	0	1542	16	1814	0	826	17	2562	0	1813
9	892	2	1541	9	1809	0	825	22	2557	97	1811
18	893	55	1537	40	1807	2	824	98	2558	182	1807
21	892	93	1536	69	1805	77	821	122	2560	187	1805
3	893	4	1535	4	1805	1	824	9	2553	2	1808
2	893	4	1535	1	1806	0	824	1	2559	3	1807
4	891	3	1535	1	1806	0	822	8	2558	1	1805
7	892	4	1535	34	1806	2	820	24	2558	2	1804
3	892	1	1535	5	1805	0	821	7	2558	1	1802
26	889	3	1533	25	1803	15	819	30	2557	3	1796

chr4	136547689	C	1	887	76	1454	9	1790	1	817	87	2467	2	1791
chr4	136547690	C	0	889	18	1512	7	1794	2	816	11	2546	13	1783
chr4	136547692	C	25	864	0	1531	65	1736	1	817	33	2523	7	1788
chr4	136547696	C	1	888	1	1530	11	1788	1	814	9	2545	3	1791
chr4	136547711	C	1	882	1	1524	3	1793	0	806	6	2538	1	1781
chr4	136547720	C	2	874	3	1513	3	1781	3	794	15	2512	9	1733
chr4	136547722	C	16	860	12	1506	34	1752	1	793	29	2501	199	1538
chr4	136547725	C	13	862	0	1515	5	1779	3	788	13	2513	0	1732
chr4	136547729	C	817	51	1358	150	1620	160	758	22	2346	163	1458	263
chr4	136547731	C	21	849	103	1413	24	1752	81	706	111	2412	3	1715
chr4	136547733	C	18	850	8	1501	37	1725	19	763	30	2488	169	1527
chr4	136547737	C	2	865	3	1501	13	1744	5	777	10	2504	26	1664
chr4	136547738	C	1	866	2	1505	2	1757	1	781	12	2503	8	1683
chr4	136547740	C	11	856	61	1445	15	1744	17	763	89	2424	7	1684
chr4	136547742	C	41	825	25	1482	80	1677	110	671	49	2466	103	1584
chr4	136547746	C	7	859	1	1506	13	1742	0	780	5	2506	0	1687
chr4	136547748	C	40	826	102	1405	112	1645	19	761	172	2341	435	1252
chr4	136547751	C	3	862	1	1506	11	1747	2	777	12	2496	0	1681
chr4	136547752	C	2	863	0	1506	1	1757	1	779	7	2504	0	1685
chr4	136547753	C	1	865	0	1507	2	1756	0	779	4	2509	2	1683
chr4	136547754	C	42	823	2	1504	95	1661	16	763	48	2464	132	1552
chr4	136547757	C	5	861	0	1507	4	1753	3	776	22	2490	2	1683
chr4	136547759	C	4	861	2	1505	22	1734	1	777	27	2486	16	1667
chr4	136547764	C	845	21	1461	45	1631	123	727	50	2460	51	1553	128
chr4	136547772	C	0	862	2	1492	0	1748	0	770	0	2506	2	1667
chr4	136547774	C	0	862	1	1493	0	1747	0	770	0	2506	0	1667
chr4	136547777	C	1	860	2	1491	2	1745	1	767	1	2504	1	1661
chr4	136547788	C	0	803	0	1373	1	1641	0	708	0	2372	0	1527
chr4	139783570	c	0	1082	2	7955	1	2811	3	7417	0	3525	2	14079
chr4	139783571	c	0	1083	0	7971	1	2817	2	7440	1	3527	4	14103
chr4	139783578	c	1	1081	2	7969	0	2821	2	7450	0	3531	1	14101
chr4	139783585	c	0	1078	1	7956	0	2821	0	7436	0	3532	4	14079
chr4	139783587	c	1	1083	0	7978	2	2821	0	7469	1	3535	1	14116
chr4	139783588	c	0	1084	8	7973	3	2819	5	7465	3	3530	13	14106
chr4	139783589	c	0	1084	10	7972	2	2819	8	7463	2	3535	19	14097
chr4	139783590	c	4	1081	5	7982	10	2813	3	7468	14	3526	8	14111
chr4	139783592	g	2	6880	1	6560	0	10666	1	4517	2	8772	0	3074
chr4	139783596	c	0	1084	11	7967	5	2816	3	7462	5	3531	7	14106
chr4	139783598	c	0	1086	6	7986	1	2822	3	7474	5	3539	11	14115
chr4	139783601	g	4	6930	2	6606	1	10757	1	4543	3	8836	1	3084
chr4	139783602	g	2	6931	3	6607	6	10755	1	4546	3	8840	0	3085
chr4	139783603	g	1	6934	4	6608	1	10763	0	4549	4	8842	1	3089
chr4	139783605	c	15	1071	10	7988	12	2813	21	7466	21	3526	12	14116
chr4	139783607	g	1	6937	0	6611	3	10767	1	4551	0	8853	1	3093
chr4	139783608	c	1	1081	12	7916	2	2805	26	7408	6	3522	18	14020
chr4	139783611	c	2	1085	11	7980	7	2817	13	7450	9	3536	25	14083

1	888	76	1530	9	1799	1	818	87	2554	2	1793
0	889	18	1530	7	1801	2	818	11	2557	13	1796
25	889	0	1531	65	1801	1	818	33	2556	7	1795
1	889	1	1531	11	1799	1	815	9	2554	3	1794
1	883	1	1525	3	1796	0	806	6	2544	1	1782
2	876	3	1516	3	1784	3	797	15	2527	9	1742
16	876	12	1518	34	1786	1	794	29	2530	199	1737
13	875	0	1515	5	1784	3	791	13	2526	0	1732
817	868	1358	1508	1620	1780	758	780	2346	2509	1458	1721
21	870	103	1516	24	1776	81	787	111	2523	3	1718
18	868	8	1509	37	1762	19	782	30	2518	169	1696
2	867	3	1504	13	1757	5	782	10	2514	26	1690
1	867	2	1507	2	1759	1	782	12	2515	8	1691
11	867	61	1506	15	1759	17	780	89	2513	7	1691
41	866	25	1507	80	1757	110	781	49	2515	103	1687
7	866	1	1507	13	1755	0	780	5	2511	0	1687
40	866	102	1507	112	1757	19	780	172	2513	435	1687
3	865	1	1507	11	1758	2	779	12	2508	0	1681
2	865	0	1506	1	1758	1	780	7	2511	0	1685
1	866	0	1507	2	1758	0	779	4	2513	2	1685
42	865	2	1506	95	1756	16	779	48	2512	132	1684
5	866	0	1507	4	1757	3	779	22	2512	2	1685
4	865	2	1507	22	1756	1	778	27	2513	16	1683
845	866	1461	1506	1631	1754	727	777	2460	2511	1553	1681
0	862	2	1494	0	1748	0	770	0	2506	2	1669
0	862	1	1494	0	1747	0	770	0	2506	0	1667
1	861	2	1493	2	1747	1	768	1	2505	1	1662
0	803	0	1373	1	1642	0	708	0	2372	0	1527
0	1082	2	7957	1	2812	3	7420	0	3525	2	14081
0	1083	0	7971	1	2818	2	7442	1	3528	4	14107
1	1082	2	7971	0	2821	2	7452	0	3531	1	14102
0	1078	1	7957	0	2821	0	7436	0	3532	4	14083
1	1084	0	7978	2	2823	0	7469	1	3536	1	14117
0	1084	8	7981	3	2822	5	7470	3	3533	13	14119
0	1084	10	7982	2	2821	8	7471	2	3537	19	14116
4	1085	5	7987	10	2823	3	7471	14	3540	8	14119
2	6882	1	6561	0	10666	1	4518	2	8774	0	3074
0	1084	11	7978	5	2821	3	7465	5	3536	7	14113
0	1086	6	7992	1	2823	3	7477	5	3544	11	14126
4	6934	2	6608	1	10758	1	4544	3	8839	1	3085
2	6933	3	6610	6	10761	1	4547	3	8843	0	3085
1	6935	4	6612	1	10764	0	4549	4	8846	1	3090
15	1086	10	7998	12	2825	21	7487	21	3547	12	14128
1	6938	0	6611	3	10770	1	4552	0	8853	1	3094
1	1082	12	7928	2	2807	26	7434	6	3528	18	14038
2	1087	11	7991	7	2824	13	7463	9	3545	25	14108

chr4	139783612	c	1	1087	9	7989	1	2827	3	7485	6	3542	14	14114
chr4	139783613	c	0	1088	109	7889	16	2811	10	7476	11	3538	20	14107
chr4	139783616	g	25	6929	6	6649	26	10761	12	4555	16	8848	9	3086
chr4	139783618	G	72	6888	151	6507	204	10590	36	4532	164	8702	7	3089
chr4	139783621	G	6	6953	8	6652	26	10774	8	4561	30	8839	4	3095
chr4	139783622	G	25	6936	15	6643	33	10769	14	4555	30	8839	7	3091
chr4	139783623	C	4	1084	136	7863	18	2809	9	7488	41	3520	13	14121
chr4	139783625	G	42	6917	124	6538	39	10765	6	4562	49	8821	4	3096
chr4	139783629	G	50	6919	10	6657	60	10754	9	4578	59	8846	8	3121
chr4	139783632	G	40	6932	11	6661	42	10770	4	4594	75	8865	1	3150
chr4	139783633	G	12	6961	12	6666	30	10785	1	4614	16	8951	13	3162
chr4	139783634	G	13	6962	15	6665	21	10801	6	4613	27	8951	11	3171
chr4	139783635	G	18	6958	19	6660	28	10796	11	4610	19	8955	4	3178
chr4	139783636	G	16	6969	10	6676	16	10812	7	4616	17	8964	4	3182
chr4	139783638	C	829	263	6474	1535	2151	681	5617	1886	2675	894	9972	4179
chr4	139783639	G	5606	1343	5190	1459	8492	2289	3873	736	7356	1588	2733	434
chr4	139783640	C	9	1083	104	7903	34	2798	200	7304	42	3528	45	14106
chr4	139783642	G	21	6972	19	6675	50	10795	8	4625	31	8976	11	3184
chr4	139783645	C	7	1087	180	7832	3	2837	13	7489	22	3563	217	13933
chr4	139783647	G	31	6520	24	6228	23	10135	7	4312	44	8478	6	2952
chr4	139783649	G	36	6963	11	6693	39	10814	7	4650	56	8986	4	3212
chr4	139783650	G	18	6984	17	6690	32	10822	10	4648	16	9034	7	3212
chr4	139783652	G	40	6964	58	6649	64	10794	64	4582	53	8981	51	3163
chr4	139783653	G	20	6987	13	6697	44	10812	7	4653	27	9031	13	3212
chr4	139783654	G	58	6949	18	6687	29	10829	7	4638	34	9010	8	3213
chr4	139783655	G	10	6995	10	6704	29	10828	9	4656	27	9035	35	3192
chr4	139783657	C	0	1095	27	7985	6	2836	9	7491	7	3583	16	14138
chr4	139783660	C	4	1091	16	8000	20	2822	9	7494	25	3566	18	14138
chr4	139783661	C	1	1094	6	8013	14	2828	1	7499	14	3576	30	14125
chr4	139783665	C	6	1090	9	8013	5	2838	13	7486	11	3577	23	14127
chr4	139783666	C	1	1095	13	8010	7	2836	13	7488	6	3585	23	14135
chr4	139783667	C	1	1095	8	8014	4	2839	11	7492	6	3585	9	14145
chr4	139783668	C	1	1095	2	8020	2	2840	2	7497	2	3587	9	14148
chr4	139783672	G	17	7043	5	6743	29	10871	10	4690	18	9129	10	3256
chr4	139783673	C	6	1090	18	8004	12	2832	8	7490	15	3577	29	14125
chr4	139783675	G	28	7037	10	6741	61	10840	8	4697	51	9096	62	3204
chr4	139783676	C	7	1092	11	7991	4	2836	15	7464	11	3578	16	14114
chr4	139783677	C	869	226	6424	1603	2387	456	6124	1377	2929	666	10563	3594
chr4	139783678	G	5869	1165	5204	1521	9034	1832	4024	660	7865	1261	2702	554
chr4	139783680	C	1	1102	12	8018	13	2841	217	7294	16	3593	14	14163
chr4	139783682	G	18	7049	3	6751	37	10876	5	4705	26	9141	0	3272
chr4	139783684	C	0	1104	9	8018	2	2849	11	7489	2	3615	13	14153
chr4	139783685	C	3	1103	11	8023	14	2843	15	7496	14	3607	20	14154
chr4	139783687	C	2	1105	14	8019	5	2853	5	7507	4	3618	18	14160
chr4	139783690	C	21	1086	5	8027	90	2770	137	7376	82	3542	409	13768
chr4	139783693	G	17	7056	7	6754	26	10894	6	4707	14	9159	1	3277

1	1088	9	7998	1	2828	3	7488	6	3548	14	14128
0	1088	109	7998	16	2827	10	7486	11	3549	20	14127
25	6954	6	6655	26	10787	12	4567	16	8864	9	3095
72	6960	151	6658	204	10794	36	4568	164	8866	7	3096
6	6959	8	6660	26	10800	8	4569	30	8869	4	3099
25	6961	15	6658	33	10802	14	4569	30	8869	7	3098
4	1088	136	7999	18	2827	9	7497	41	3561	13	14134
42	6959	124	6662	39	10804	6	4568	49	8870	4	3100
50	6969	10	6667	60	10814	9	4587	59	8905	8	3129
40	6972	11	6672	42	10812	4	4598	75	8940	1	3151
12	6973	12	6678	30	10815	1	4615	16	8967	13	3175
13	6975	15	6680	21	10822	6	4619	27	8978	11	3182
18	6976	19	6679	28	10824	11	4621	19	8974	4	3182
16	6985	10	6686	16	10828	7	4623	17	8981	4	3186
829	1092	6474	8009	2151	2832	5617	7503	2675	3569	9972	14151
5606	6949	5190	6649	8492	10781	3873	4609	7356	8944	2733	3167
9	1092	104	8007	34	2832	200	7504	42	3570	45	14151
21	6993	19	6694	50	10845	8	4633	31	9007	11	3195
7	1094	180	8012	3	2840	13	7502	22	3585	217	14150
31	6551	24	6252	23	10158	7	4319	44	8522	6	2958
36	6999	11	6704	39	10853	7	4657	56	9042	4	3216
18	7002	17	6707	32	10854	10	4658	16	9050	7	3219
40	7004	58	6707	64	10858	64	4646	53	9034	51	3214
20	7007	13	6710	44	10856	7	4660	27	9058	13	3225
58	7007	18	6705	29	10858	7	4645	34	9044	8	3221
10	7005	10	6714	29	10857	9	4665	27	9062	35	3227
0	1095	27	8012	6	2842	9	7500	7	3590	16	14154
4	1095	16	8016	20	2842	9	7503	25	3591	18	14156
1	1095	6	8019	14	2842	1	7500	14	3590	30	14155
6	1096	9	8022	5	2843	13	7499	11	3588	23	14150
1	1096	13	8023	7	2843	13	7501	6	3591	23	14158
1	1096	8	8022	4	2843	11	7503	6	3591	9	14154
1	1096	2	8022	2	2842	2	7499	2	3589	9	14157
17	7060	5	6748	29	10900	10	4700	18	9147	10	3266
6	1096	18	8022	12	2844	8	7498	15	3592	29	14154
28	7065	10	6751	61	10901	8	4705	51	9147	62	3266
7	1099	11	8002	4	2840	15	7479	11	3589	16	14130
869	1095	6424	8027	2387	2843	6124	7501	2929	3595	10563	14157
5869	7034	5204	6725	9034	10866	4024	4684	7865	9126	2702	3256
1	1103	12	8030	13	2854	217	7511	16	3609	14	14177
18	7067	3	6754	37	10913	5	4710	26	9167	0	3272
0	1104	9	8027	2	2851	11	7500	2	3617	13	14166
3	1106	11	8034	14	2857	15	7511	14	3621	20	14174
2	1107	14	8033	5	2858	5	7512	4	3622	18	14178
21	1107	5	8032	90	2860	137	7513	82	3624	409	14177
17	7073	7	6761	26	10920	6	4713	14	9173	1	3278

chr4	139783694	C	46	1060	463	7573	121	2738	438	7069	178	3448	781	13398
chr4	139783696	C	33	1073	391	7649	87	2772	234	7277	159	3472	329	13847
chr4	139783698	C	6	1102	9	8033	1	2859	14	7499	8	3622	19	14159
chr4	139783699	C	981	127	6650	1393	2550	304	6483	1028	3170	448	12218	1964
chr4	139783700	G	5839	1129	5963	697	9116	1646	3859	772	7937	1118	2704	531
chr4	139783701	C	2	1106	11	8033	3	2856	12	7506	6	3626	23	14158
chr4	139783702	C	0	1108	13	8035	6	2854	6	7513	12	3623	15	14169
chr4	139783705	G	25	7051	4	6757	40	10882	25	4693	70	9120	14	3274
chr4	139783706	C	8	1101	6	8046	16	2845	5	7516	14	3626	141	14042
chr4	139783710	C	1	1108	9	8042	7	2853	21	7500	5	3635	11	14178
chr4	139783711	C	15	1095	2	8048	16	2847	38	7481	45	3601	211	13984
chr4	139783713	G	32	7053	6	6755	34	10896	11	4717	19	9191	4	3287
chr4	139783719	G	11	7074	4	6757	13	10923	0	4727	25	9190	9	3285
chr4	139783720	C	0	77	9	162	0	76	11	177	5	264	5	213
chr4	139783722	C	0	77	0	172	0	76	2	184	1	266	0	218
chr4	139783724	C	1	73	0	168	0	74	0	184	0	264	0	208
chr4	139783727	G	53	7055	15	6747	71	10874	106	4628	54	9177	1	3299
chr4	139783728	C	2	68	3	159	1	68	1	177	4	255	2	204
chr4	139783730	C	0	71	1	166	2	66	12	163	3	247	0	205
chr4	139783732	C	1	76	4	162	1	72	7	168	4	260	14	204
chr4	139783736	C	60	22	141	31	62	16	141	47	215	64	188	53
chr4	139783737	G	5198	1884	4860	1881	7884	2997	3625	1102	7123	2095	2418	883
chr4	139783740	C	0	84	1	171	0	79	0	197	3	280	3	241
chr4	139783741	C	2	101	0	180	1	100	0	207	2	315	0	277
chr4	139783742	C	0	105	0	182	3	103	6	208	2	329	11	283
chr4	139783744	C	0	120	0	185	0	119	0	228	1	344	1	321
chr4	139783745	C	188	78	146	40	159	66	188	62	432	175	292	127
chr4	139783746	G	272	110	213	96	250	120	230	129	660	266	314	144
chr4	139783748	C	0	265	0	201	2	224	1	251	1	612	0	426
chr4	139783749	C	0	267	1	201	1	226	1	251	2	617	2	433
chr4	139783753	G	1	622	3	553	2	688	11	500	4	1161	1	533
chr4	139783754	C	931	203	6990	1053	2388	491	5626	1931	2948	722	11330	2917
chr4	139783755	G	5173	1938	4909	1858	8085	2855	3549	1210	7174	2097	2541	787
chr4	139783756	G	16	7109	14	6765	16	10950	5	4760	26	9255	8	3324
chr4	139783759	G	176	6944	366	6410	292	10658	197	4558	246	9016	95	3231
chr4	139783760	G	17	7103	21	6758	28	10935	8	4749	24	9226	6	3322
chr4	139783763	G	24	7089	112	6663	107	10851	16	4745	35	9219	35	3293
chr4	139783764	C	1	1144	21	8044	10	2881	120	7446	5	3691	19	14266
chr4	139783766	C	4	1141	15	8042	11	2880	18	7545	9	3684	30	14248
chr4	139783768	G	133	6980	138	6636	100	10847	7	4750	120	9109	33	3286
chr4	139783771	G	21	7098	14	6764	26	10935	15	4749	29	9209	7	3315
chr4	139783772	G	19	7099	15	6762	29	10927	12	4751	28	9214	6	3315
chr4	139783773	C	1	1144	15	8038	6	2882	12	7538	6	3683	20	14249
chr4	139783774	C	9	1136	8	8041	30	2860	33	7512	32	3656	14	14254
chr4	139783776	C	34	1111	278	7765	175	2715	116	7424	177	3510	297	13956
chr4	139783778	C	1	1142	15	8023	8	2881	9	7527	5	3680	23	14227

46	1106	463	8036	121	2859	438	7507	178	3626	781	14179
33	1106	391	8040	87	2859	234	7511	159	3631	329	14176
6	1108	9	8042	1	2860	14	7513	8	3630	19	14178
981	1108	6650	8043	2550	2854	6483	7511	3170	3618	12218	14182
5839	6968	5963	6660	9116	10762	3859	4631	7937	9055	2704	3235
2	1108	11	8044	3	2859	12	7518	6	3632	23	14181
0	1108	13	8048	6	2860	6	7519	12	3635	15	14184
25	7076	4	6761	40	10922	25	4718	70	9190	14	3288
8	1109	6	8052	16	2861	5	7521	14	3640	141	14183
1	1109	9	8051	7	2860	21	7521	5	3640	11	14189
15	1110	2	8050	16	2863	38	7519	45	3646	211	14195
32	7085	6	6761	34	10930	11	4728	19	9210	4	3291
11	7085	4	6761	13	10936	0	4727	25	9215	9	3294
0	77	9	171	0	76	11	188	5	269	5	218
0	77	0	172	0	76	2	186	1	267	0	218
1	74	0	168	0	74	0	184	0	264	0	208
53	7108	15	6762	71	10945	106	4734	54	9231	1	3300
2	70	3	162	1	69	1	178	4	259	2	206
0	71	1	167	2	68	12	175	3	250	0	205
1	77	4	166	1	73	7	175	4	264	14	218
60	82	141	172	62	78	141	188	215	279	188	241
5198	7082	4860	6741	7884	10881	3625	4727	7123	9218	2418	3301
0	84	1	172	0	79	0	197	3	283	3	244
2	103	0	180	1	101	0	207	2	317	0	277
0	105	0	182	3	106	6	214	2	331	11	294
0	120	0	185	0	119	0	228	1	345	1	322
188	266	146	186	159	225	188	250	432	607	292	419
272	382	213	309	250	370	230	359	660	926	314	458
0	265	0	201	2	226	1	252	1	613	0	426
0	267	1	202	1	227	1	252	2	619	2	435
1	623	3	556	2	690	11	511	4	1165	1	534
931	1134	6990	8043	2388	2879	5626	7557	2948	3670	11330	14247
5173	7111	4909	6767	8085	10940	3549	4759	7174	9271	2541	3328
16	7125	14	6779	16	10966	5	4765	26	9281	8	3332
176	7120	366	6776	292	10950	197	4755	246	9262	95	3326
17	7120	21	6779	28	10963	8	4757	24	9250	6	3328
24	7113	112	6775	107	10958	16	4761	35	9254	35	3328
1	1145	21	8065	10	2891	120	7566	5	3696	19	14285
4	1145	15	8057	11	2891	18	7563	9	3693	30	14278
133	7113	138	6774	100	10947	7	4757	120	9229	33	3319
21	7119	14	6778	26	10961	15	4764	29	9238	7	3322
19	7118	15	6777	29	10956	12	4763	28	9242	6	3321
1	1145	15	8053	6	2888	12	7550	6	3689	20	14269
9	1145	8	8049	30	2890	33	7545	32	3688	14	14268
34	1145	278	8043	175	2890	116	7540	177	3687	297	14253
1	1143	15	8038	8	2889	9	7536	5	3685	23	14250

chr4	139783779	C	12	1131	141	7897	58	2832	6	7525	78	3606	254	14000
chr4	139783781	C	11	1132	199	7838	27	2858	18	7498	28	3653	29	14214
chr4	139783783	C	2	1141	10	8027	7	2880	9	7524	7	3675	12	14239
chr4	139783785	G	14	7093	12	6759	37	10909	6	4735	33	9143	6	3289
chr4	139783787	G	88	7016	165	6604	212	10740	104	4637	150	9026	11	3283
chr4	139783790	G	8	7088	12	6746	13	10925	8	4721	30	9122	6	3274
chr4	139783791	C	17	1123	671	7346	73	2813	92	7426	54	3620	19	14215
chr4	139783793	C	1	1138	22	8012	8	2880	17	7503	4	3670	23	14216
chr4	139783794	C	3	1138	12	8022	1	2887	6	7516	7	3669	18	14225
chr4	139783796	G	36	7061	194	6572	55	10891	71	4662	78	9044	13	3241
chr4	139783800	C	8	1132	16	8016	11	2876	14	7498	14	3656	34	14202
chr4	139783801	C	1	1138	12	8019	1	2885	14	7501	10	3662	21	14211
chr4	139783802	C	20	1119	361	7669	77	2807	135	7381	115	3555	754	13484
chr4	139783804	C	2	1137	17	8008	3	2881	14	7501	11	3656	32	14200
chr4	139783805	C	0	1137	17	8011	4	2882	8	7507	9	3659	27	14210
chr4	139783808	G	29	7054	6	6751	35	10907	107	4613	50	9032	6	3235
chr4	139783810	C	12	1127	13	8015	3	2879	13	7496	6	3658	36	14193
chr4	139783812	G	117	6965	220	6535	264	10677	236	4480	212	8857	58	3178
chr4	139783814	G	10	7073	4	6751	34	10905	103	4613	22	9043	27	3206
chr4	139783817	C	4	1134	19	8000	3	2879	20	7485	13	3644	32	14196
chr4	139783818	C	1	1136	9	8010	6	2877	11	7496	15	3642	7	14226
chr4	139783819	C	3	1135	13	8006	10	2872	9	7498	9	3644	31	14197
chr4	139783822	C	8	1121	22	7994	35	2846	21	7479	8	3626	271	13936
chr4	139783823	C	3	1132	7	8010	13	2869	9	7496	4	3644	31	14196
chr4	139783824	C	3	1132	16	7999	9	2872	15	7488	6	3643	25	14201
chr4	139783826	G	94	6985	3	6747	252	10685	143	4562	198	8840	23	3203
chr4	139783829	G	33	7046	16	6736	26	10914	9	4696	40	8996	9	3217
chr4	139783830	C	10	1118	10	8001	25	2852	162	7336	17	3618	190	14022
chr4	139783832	C	3	1124	12	7986	3	2873	12	7481	0	3631	34	14176
chr4	139783833	C	1	1125	10	7998	6	2871	3	7493	1	3631	11	14203
chr4	139783836	C	0	1123	13	7994	5	2871	12	7480	10	3614	12	14199
chr4	139783840	G	16	7053	11	6738	20	10918	4	4694	11	9015	7	3215
chr4	139783841	C	3	1120	7	7998	4	2871	18	7474	13	3610	42	14161
chr4	139783843	C	11	1113	23	7985	58	2818	8	7485	56	3564	214	13992
chr4	139783845	G	10	7057	174	6574	26	10910	10	4690	21	9004	6	3215
chr4	139783847	C	9	1114	98	7908	39	2836	2	7492	63	3555	98	14113
chr4	139783849	G	28	7025	12	6732	18	10905	8	4687	39	8959	5	3203
chr4	139783850	C	4	1116	18	7985	8	2867	14	7478	4	3604	32	14171
chr4	139783851	C	2	1121	24	7979	5	2871	12	7476	7	3610	24	14179
chr4	139783852	C	709	411	4959	3044	2018	854	4692	2797	2433	1184	9362	4841
chr4	139783853	G	4096	2955	3426	3315	6175	4750	3242	1445	6579	2419	1971	1236
chr4	139783854	G	6	7039	5	6735	12	10912	6	4678	24	8968	10	3194
chr4	139783855	C	0	1119	10	7997	3	2873	13	7481	5	3604	32	14173
chr4	139783856	C	680	415	4782	3013	1746	1065	3857	3487	2156	1393	7781	6090
chr4	139783857	G	4266	2773	4212	2525	6331	4587	2826	1861	6631	2355	1944	1253
chr4	139783860	C	1	1118	125	7881	1	2874	10	7483	5	3603	17	14187

12	1143	141	8038	58	2890	6	7531	78	3684	254	14254
11	1143	199	8037	27	2885	18	7516	28	3681	29	14243
2	1143	10	8037	7	2887	9	7533	7	3682	12	14251
14	7107	12	6771	37	10946	6	4741	33	9176	6	3295
88	7104	165	6769	212	10952	104	4741	150	9176	11	3294
8	7096	12	6758	13	10938	8	4729	30	9152	6	3280
17	1140	671	8017	73	2886	92	7518	54	3674	19	14234
1	1139	22	8034	8	2888	17	7520	4	3674	23	14239
3	1141	12	8034	1	2888	6	7522	7	3676	18	14243
36	7097	194	6766	55	10946	71	4733	78	9122	13	3254
8	1140	16	8032	11	2887	14	7512	14	3670	34	14236
1	1139	12	8031	1	2886	14	7515	10	3672	21	14232
20	1139	361	8030	77	2884	135	7516	115	3670	754	14238
2	1139	17	8025	3	2884	14	7515	11	3667	32	14232
0	1137	17	8028	4	2886	8	7515	9	3668	27	14237
29	7083	6	6757	35	10942	107	4720	50	9082	6	3241
12	1139	13	8028	3	2882	13	7509	6	3664	36	14229
117	7082	220	6755	264	10941	236	4716	212	9069	58	3236
10	7083	4	6755	34	10939	103	4716	22	9065	27	3233
4	1138	19	8019	3	2882	20	7505	13	3657	32	14228
1	1137	9	8019	6	2883	11	7507	15	3657	7	14233
3	1138	13	8019	10	2882	9	7507	9	3653	31	14228
8	1129	22	8016	35	2881	21	7500	8	3634	271	14207
3	1135	7	8017	13	2882	9	7505	4	3648	31	14227
3	1135	16	8015	9	2881	15	7503	6	3649	25	14226
94	7079	3	6750	252	10937	143	4705	198	9038	23	3226
33	7079	16	6752	26	10940	9	4705	40	9036	9	3226
10	1128	10	8011	25	2877	162	7498	17	3635	190	14212
3	1127	12	7998	3	2876	12	7493	0	3631	34	14210
1	1126	10	8008	6	2877	3	7496	1	3632	11	14214
0	1123	13	8007	5	2876	12	7492	10	3624	12	14211
16	7069	11	6749	20	10938	4	4698	11	9026	7	3222
3	1123	7	8005	4	2875	18	7492	13	3623	42	14203
11	1124	23	8008	58	2876	8	7493	56	3620	214	14206
10	7067	174	6748	26	10936	10	4700	21	9025	6	3221
9	1123	98	8006	39	2875	2	7494	63	3618	98	14211
28	7053	12	6744	18	10923	8	4695	39	8998	5	3208
4	1120	18	8003	8	2875	14	7492	4	3608	32	14203
2	1123	24	8003	5	2876	12	7488	7	3617	24	14203
709	1120	4959	8003	2018	2872	4692	7489	2433	3617	9362	14203
4096	7051	3426	6741	6175	10925	3242	4687	6579	8998	1971	3207
6	7045	5	6740	12	10924	6	4684	24	8992	10	3204
0	1119	10	8007	3	2876	13	7494	5	3609	32	14205
680	1095	4782	7795	1746	2811	3857	7344	2156	3549	7781	13871
4266	7039	4212	6737	6331	10918	2826	4687	6631	8986	1944	3197
1	1119	125	8006	1	2875	10	7493	5	3608	17	14204

chr4	139783862	C	648	470	4307	3695	1820	1053	3824	3665	2266	1340	8286	5910
chr4	139783863	G	4460	2554	3342	3375	6725	4165	3382	1294	6743	2205	2114	1067
chr4	139783864	G	32	6996	15	6718	16	10897	7	4671	27	8928	10	3176
chr4	139783866	G	20	7004	14	6716	26	10886	7	4672	18	8932	7	3181
chr4	139783869	G	20	6987	122	6588	43	10852	10	4657	66	8850	30	3151
chr4	139783870	C	3	1115	219	7784	0	2875	5	7488	9	3600	154	14044
chr4	139783872	G	14	6999	8	6716	8	10899	7	4667	11	8904	14	3160
chr4	139783873	G	14	7003	27	6699	19	10887	8	4668	23	8895	4	3170
chr4	139783875	G	205	6809	57	6664	351	10555	220	4451	326	8578	125	3036
chr4	139783876	C	28	1090	24	7973	29	2845	386	7099	68	3538	12	14188
chr4	139783878	G	2	7011	0	6718	1	10904	0	4663	3	8888	1	3151
chr4	139783884	C	0	1107	1	7994	0	2868	1	7470	2	3584	0	14180
chr4	139783885	C	0	1113	0	7994	0	2869	1	7472	1	3585	1	14177
chr4	139783888	G	0	6993	1	6704	2	10900	1	4651	2	8871	0	3142
chr4	139783891	C	0	1110	4	7984	3	2862	4	7461	4	3571	3	14163
chr4	139783894	G	0	6975	0	6695	1	10864	0	4642	0	8857	0	3141
chr4	139783896	C	0	944	4	7969	1	2741	3	7426	0	3278	5	14030
chr4	139783897	C	0	944	0	7966	0	2742	1	7429	0	3278	1	14033
chr4	139783898	C	0	943	0	7960	1	2741	3	7426	0	3276	1	14028
chr4	139783900	G	0	6797	0	6500	1	10644	0	4524	2	8692	0	3085
chr4	139783901	G	0	6765	0	6479	3	10602	0	4519	4	8659	0	3080
chr4	139783902	G	1	6751	0	6454	3	10570	1	4496	3	8646	0	3072
chr4	139783904	C	1	920	3	7823	1	2695	2	7263	0	3204	4	13811
chr5	64987289	G	0	633	0	1891	0	1119	2	1632	0	2168	1	1021
chr5	64987290	G	1	632	0	1893	0	1119	1	1635	0	2170	0	1021
chr5	64987294	G	0	634	2	1897	0	1120	0	1643	0	2174	1	1023
chr5	64987296	G	0	634	0	1899	0	1121	1	1643	0	2173	0	1024
chr5	64987302	G	0	635	0	1901	2	1119	0	1643	1	2175	0	1024
chr5	64987307	G	0	634	0	1898	0	1121	0	1642	0	2176	1	1025
chr5	64987310	G	7	628	0	1904	26	1094	1	1644	34	2144	17	1008
chr5	64987316	G	0	636	4	1902	4	1116	17	1629	16	2162	17	1008
chr5	64987317	G	1	635	2	1904	4	1117	3	1643	7	2171	3	1023
chr5	64987319	G	4	632	7	1898	5	1116	4	1642	15	2163	2	1024
chr5	64987320	C	0	377	1	1522	0	1131	1	847	1	1779	0	1019
chr5	64987323	G	1	635	0	1907	1	1120	5	1642	12	2168	0	1026
chr5	64987325	C	0	526	1	2340	0	1587	1	1249	1	2824	0	1492
chr5	64987327	G	4	633	1	1905	2	1119	2	1644	1	2181	2	1025
chr5	64987329	C	0	526	0	2346	0	1591	0	1254	0	2830	0	1497
chr5	64987330	C	0	527	1	2352	0	1595	0	1258	0	2840	0	1506
chr5	64987333	G	18	617	2	1908	31	1091	1	1646	67	2114	59	964
chr5	64987338	G	1	637	1	1909	3	1118	3	1644	7	2179	3	1023
chr5	64987339	G	1	637	2	1906	3	1119	9	1638	4	2181	1	1026
chr5	64987340	G	4	634	5	1904	2	1120	4	1644	9	2177	2	1024
chr5	64987342	G	17	622	120	1790	42	1080	2	1646	83	2104	17	1010
chr5	64987343	C	0	530	0	2374	0	1608	2	1266	0	2861	0	1527
chr5	64987345	G	13	626	104	1806	27	1095	14	1635	58	2128	30	996

648	1118	4307	8002	1820	2873	3824	7489	2266	3606	8286	14196
4460	7014	3342	6717	6725	10890	3382	4676	6743	8948	2114	3181
32	7028	15	6733	16	10913	7	4678	27	8955	10	3186
20	7024	14	6730	26	10912	7	4679	18	8950	7	3188
20	7007	122	6710	43	10895	10	4667	66	8916	30	3181
3	1118	219	8003	0	2875	5	7493	9	3609	154	14198
14	7013	8	6724	8	10907	7	4674	11	8915	14	3174
14	7017	27	6726	19	10906	8	4676	23	8918	4	3174
205	7014	57	6721	351	10906	220	4671	326	8904	125	3161
28	1118	24	7997	29	2874	386	7485	68	3606	12	14200
2	7013	0	6718	1	10905	0	4663	3	8891	1	3152
0	1107	1	7995	0	2868	1	7471	2	3586	0	14180
0	1113	0	7994	0	2869	1	7473	1	3586	1	14178
0	6993	1	6705	2	10902	1	4652	2	8873	0	3142
0	1110	4	7988	3	2865	4	7465	4	3575	3	14166
0	6975	0	6695	1	10865	0	4642	0	8857	0	3141
0	944	4	7973	1	2742	3	7429	0	3278	5	14035
0	944	0	7966	0	2742	1	7430	0	3278	1	14034
0	943	0	7960	1	2742	3	7429	0	3276	1	14029
0	6797	0	6500	1	10645	0	4524	2	8694	0	3085
0	6765	0	6479	3	10605	0	4519	4	8663	0	3080
1	6752	0	6454	3	10573	1	4497	3	8649	0	3072
1	921	3	7826	1	2696	2	7265	0	3204	4	13815
0	633	0	1891	0	1119	2	1634	0	2168	1	1022
1	633	0	1893	0	1119	1	1636	0	2170	0	1021
0	634	2	1899	0	1120	0	1643	0	2174	1	1024
0	634	0	1899	0	1121	1	1644	0	2173	0	1024
0	635	0	1901	2	1121	0	1643	1	2176	0	1024
0	634	0	1898	0	1121	0	1642	0	2176	1	1026
7	635	0	1904	26	1120	1	1645	34	2178	17	1025
0	636	4	1906	4	1120	17	1646	16	2178	17	1025
1	636	2	1906	4	1121	3	1646	7	2178	3	1026
4	636	7	1905	5	1121	4	1646	15	2178	2	1026
0	377	1	1523	0	1131	1	848	1	1780	0	1019
1	636	0	1907	1	1121	5	1647	12	2180	0	1026
0	526	1	2341	0	1587	1	1250	1	2825	0	1492
4	637	1	1906	2	1121	2	1646	1	2182	2	1027
0	526	0	2346	0	1591	0	1254	0	2830	0	1497
0	527	1	2353	0	1595	0	1258	0	2840	0	1506
18	635	2	1910	31	1122	1	1647	67	2181	59	1023
1	638	1	1910	3	1121	3	1647	7	2186	3	1026
1	638	2	1908	3	1122	9	1647	4	2185	1	1027
4	638	5	1909	2	1122	4	1648	9	2186	2	1026
17	639	120	1910	42	1122	2	1648	83	2187	17	1027
0	530	0	2374	0	1608	2	1268	0	2861	0	1527
13	639	104	1910	27	1122	14	1649	58	2186	30	1026

chr5	64987346	G	1	638	2	1907	4	1118	5	1643	4	2185	1	1026
chr5	64987349	C	1	537	7	2389	2	1619	3	1276	7	2858	4	1531
chr5	64987350	C	2	536	7	2389	7	1614	4	1275	7	2859	3	1532
chr5	64987354	G	1	638	49	1867	1	1124	0	1649	3	2187	1	1027
chr5	64987357	G	2	637	4	1915	2	1124	3	1646	9	2185	0	1027
chr5	64987358	C	0	537	3	2393	1	1622	1	1278	10	2858	6	1530
chr5	64987359	C	0	538	0	2396	3	1620	0	1279	3	2865	2	1534
chr5	64987363	C	1	537	1	2395	1	1622	3	1276	6	2861	40	1497
chr5	64987367	C	244	294	573	1822	700	923	581	698	1424	1446	535	1002
chr5	64987368	G	352	287	771	1147	546	583	820	831	1082	1122	552	477
chr5	64987376	C	0	539	2	2394	2	1621	1	1282	5	2865	0	1539
chr5	64987377	C	211	328	810	1586	661	962	469	814	1249	1621	542	997
chr5	64987378	G	317	324	739	1179	500	630	771	879	1061	1144	441	587
chr5	64987380	C	2	536	4	2384	5	1618	1	1280	8	2861	3	1534
chr5	64987383	C	2	537	6	2390	2	1622	2	1282	6	2867	1	1539
chr5	64987384	C	3	536	5	2391	2	1622	2	1282	8	2865	5	1535
chr5	64987386	G	1	639	2	1914	11	1120	5	1646	29	2179	5	1024
chr5	64987389	C	0	540	3	2392	5	1620	12	1272	1	2872	1	1540
chr5	64987391	G	4	635	3	1912	4	1123	5	1644	17	2187	1	1026
chr5	64987393	G	0	641	5	1912	3	1128	3	1650	8	2200	3	1026
chr5	64987402	G	4	640	67	1854	4	1130	0	1654	10	2209	0	1031
chr5	64987407	C	3	537	0	2396	4	1621	0	1283	5	2869	28	1516
chr5	64987412	G	6	642	5	1925	8	1128	4	1654	9	2212	21	1017
chr5	64987414	G	2	646	1	1936	0	1137	1	1658	9	2216	4	1035
chr5	64987417	C	1	541	2	2395	4	1625	2	1283	5	2870	3	1543
chr5	64987419	C	378	164	1357	1041	1139	488	802	484	2149	727	1056	491
chr5	64987420	G	515	137	1432	518	815	328	1307	366	1710	532	829	214
chr5	64987421	G	3	652	5	1948	2	1144	3	1672	7	2236	1	1041
chr5	64987422	C	345	198	1285	1112	1095	534	718	571	2050	825	1040	506
chr5	64987423	G	561	91	1636	309	986	156	1271	397	1893	346	899	136
chr5	64987424	G	4	653	6	1945	5	1141	2	1673	3	2245	1	1042
chr5	64987425	G	1	657	5	1948	0	1148	6	1669	9	2242	1	1044
chr5	64987426	G	2	658	5	1948	0	1149	6	1670	6	2247	3	1043
chr5	64987427	G	1	661	5	1949	1	1148	3	1673	5	2247	1	1045
chr5	64987431	G	30	629	156	1798	63	1086	102	1573	95	2153	41	1006
chr5	64987434	G	3	658	5	1950	4	1146	0	1675	4	2244	0	1048
chr5	64987435	G	4	655	1	1950	6	1143	1	1674	0	2246	18	1027
chr5	64987438	G	0	84	0	103	1	118	0	72	0	329	0	85
chr5	64987440	G	7	84	5	100	1	123	0	80	12	332	0	94
chr5	64987442	C	391	132	1681	658	1178	400	796	464	2154	640	1062	451
chr5	64987443	G	79	13	82	26	100	27	72	12	269	73	92	8
chr5	64987445	C	388	149	1515	876	1147	469	769	506	2092	741	1104	432
chr5	64987446	G	74	23	86	34	89	42	63	23	249	95	71	30
chr5	64987448	C	391	145	1483	881	1198	397	895	374	2226	581	1137	388
chr5	64987449	G	83	15	100	32	90	37	67	24	266	74	84	21
chr5	64987451	C	423	113	1632	753	1277	331	990	287	2260	580	1307	227

1	639	2	1909	4	1122	5	1648	4	2189	1	1027
1	538	7	2396	2	1621	3	1279	7	2865	4	1535
2	538	7	2396	7	1621	4	1279	7	2866	3	1535
1	639	49	1916	1	1125	0	1649	3	2190	1	1028
2	639	4	1919	2	1126	3	1649	9	2194	0	1027
0	537	3	2396	1	1623	1	1279	10	2868	6	1536
0	538	0	2396	3	1623	0	1279	3	2868	2	1536
1	538	1	2396	1	1623	3	1279	6	2867	40	1537
244	538	573	2395	700	1623	581	1279	1424	2870	535	1537
352	639	771	1918	546	1129	820	1651	1082	2204	552	1029
0	539	2	2396	2	1623	1	1283	5	2870	0	1539
211	539	810	2396	661	1623	469	1283	1249	2870	542	1539
317	641	739	1918	500	1130	771	1650	1061	2205	441	1028
2	538	4	2388	5	1623	1	1281	8	2869	3	1537
2	539	6	2396	2	1624	2	1284	6	2873	1	1540
3	539	5	2396	2	1624	2	1284	8	2873	5	1540
1	640	2	1916	11	1131	5	1651	29	2208	5	1029
0	540	3	2395	5	1625	12	1284	1	2873	1	1541
4	639	3	1915	4	1127	5	1649	17	2204	1	1027
0	641	5	1917	3	1131	3	1653	8	2208	3	1029
4	644	67	1921	4	1134	0	1654	10	2219	0	1031
3	540	0	2396	4	1625	0	1283	5	2874	28	1544
6	648	5	1930	8	1136	4	1658	9	2221	21	1038
2	648	1	1937	0	1137	1	1659	9	2225	4	1039
1	542	2	2397	4	1629	2	1285	5	2875	3	1546
378	542	1357	2398	1139	1627	802	1286	2149	2876	1056	1547
515	652	1432	1950	815	1143	1307	1673	1710	2242	829	1043
3	655	5	1953	2	1146	3	1675	7	2243	1	1042
345	543	1285	2397	1095	1629	718	1289	2050	2875	1040	1546
561	652	1636	1945	986	1142	1271	1668	1893	2239	899	1035
4	657	6	1951	5	1146	2	1675	3	2248	1	1043
1	658	5	1953	0	1148	6	1675	9	2251	1	1045
2	660	5	1953	0	1149	6	1676	6	2253	3	1046
1	662	5	1954	1	1149	3	1676	5	2252	1	1046
30	659	156	1954	63	1149	102	1675	95	2248	41	1047
3	661	5	1955	4	1150	0	1675	4	2248	0	1048
4	659	1	1951	6	1149	1	1675	0	2246	18	1045
0	84	0	103	1	119	0	72	0	329	0	85
7	91	5	105	1	124	0	80	12	344	0	94
391	523	1681	2339	1178	1578	796	1260	2154	2794	1062	1513
79	92	82	108	100	127	72	84	269	342	92	100
388	537	1515	2391	1147	1616	769	1275	2092	2833	1104	1536
74	97	86	120	89	131	63	86	249	344	71	101
391	536	1483	2364	1198	1595	895	1269	2226	2807	1137	1525
83	98	100	132	90	127	67	91	266	340	84	105
423	536	1632	2385	1277	1608	990	1277	2260	2840	1307	1534

chr5	64987452	G	79	20	113	20	103	24	72	26	279	58	90	14
chr5	64987454	C	456	82	1919	446	1390	213	1008	271	2473	355	1268	271
chr5	64987455	G	86	17	114	23	113	20	83	18	297	44	94	12
chr5	64987458	C	3	502	7	2266	4	1499	16	1180	7	2616	1	1453
chr5	64987463	G	0	105	0	142	1	126	0	100	2	329	0	108
chr5	64987464	C	0	536	4	2385	6	1599	2	1274	14	2829	39	1503
chr5	64987465	C	451	91	1649	765	1424	207	980	310	2523	359	1258	294
chr5	64987466	G	76	32	113	32	75	53	82	21	238	99	86	24
chr5	64987467	G	0	96	0	143	0	104	0	102	0	314	0	97
chr5	64987468	C	2	458	5	2195	8	1376	2	1146	4	2406	3	1326
chr5	64987469	C	468	75	1999	402	1417	209	995	289	2576	296	1311	231
chr5	64987470	G	78	26	130	20	78	41	91	11	248	78	82	27
chr5	64987475	C	2	87	3	224	1	163	1	127	8	425	4	249
chr5	64987478	G	0	105	0	153	0	128	0	106	0	330	0	112
chr5	64987481	G	6	82	1	152	3	89	5	101	15	261	1	93
chr5	64987483	G	3	86	7	148	2	90	8	100	6	263	9	85
chr5	64987484	C	3	112	13	487	5	213	11	246	16	412	9	348
chr5	64987486	C	2	115	3	508	2	216	0	259	5	421	1	354
chr5	64987488	C	1	115	1	507	0	217	1	257	1	420	1	352
chr5	64987490	G	0	89	3	192	0	96	0	153	2	277	0	116
chr5	64987491	C	2	114	1	495	0	214	0	250	0	443	3	347
chr5	64987492	C	0	116	0	494	0	213	0	252	0	441	2	348
chr5	64987494	G	2	667	64	1947	5	1110	45	1672	7	2175	1	1053
chr5	64987496	G	0	675	5	2014	0	1118	5	1719	8	2187	0	1058
chr5	64987501	C	0	125	0	554	1	250	1	283	0	459	0	470
chr5	64987505	C	5	138	2	616	0	281	1	327	9	486	11	512
chr5	64987507	G	3	669	5	2012	1	1113	1	1721	5	2184	4	1055
chr5	64987508	G	4	668	2	2016	0	1114	5	1716	5	2181	8	1051
chr5	64987511	C	0	153	1	728	0	327	1	385	0	513	0	565
chr5	64987512	C	0	239	0	1117	4	584	0	605	0	885	1	778
chr5	64987514	C	2	529	1	2399	9	1592	1	1279	18	2706	1	1502
chr5	64987516	C	1	529	0	2400	2	1594	1	1279	5	2708	1	1503
chr5	64987524	C	0	528	3	2397	2	1592	0	1276	3	2699	2	1499
chr5	64987525	C	358	170	1592	807	1074	517	847	428	1889	807	1042	458
chr5	64987526	G	430	242	1048	966	691	417	1070	648	1244	921	693	364
chr5	64987528	G	9	659	3	2010	4	1105	3	1713	5	2154	1	1056
chr5	64987529	C	10	517	3	2395	5	1589	9	1264	20	2668	20	1477
chr5	64987534	C	2	525	6	2391	6	1587	2	1271	6	2681	2	1493
chr5	64987536	G	11	659	140	1872	22	1085	1	1715	45	2106	38	1019
chr5	64987537	C	3	524	1	2395	26	1564	7	1266	37	2648	2	1494
chr5	64987539	G	5	663	6	1998	8	1097	4	1708	16	2130	1	1056
chr5	64987540	C	0	527	2	2394	1	1591	2	1270	4	2675	0	1494
chr5	64987545	G	0	668	3	2006	1	1107	1	1712	4	2145	4	1050
chr5	64987547	G	1	667	4	2006	7	1100	6	1708	17	2135	1	1051
chr5	64987548	C	1	524	2	2393	8	1581	3	1269	3	2670	6	1485
chr5	64987551	G	7	660	2	2006	10	1096	55	1660	16	2124	0	1053

79	99	113	133	103	127	72	98	279	337	90	104
456	538	1919	2365	1390	1603	1008	1279	2473	2828	1268	1539
86	103	114	137	113	133	83	101	297	341	94	106
3	505	7	2273	4	1503	16	1196	7	2623	1	1454
0	105	0	142	1	127	0	100	2	331	0	108
0	536	4	2389	6	1605	2	1276	14	2843	39	1542
451	542	1649	2414	1424	1631	980	1290	2523	2882	1258	1552
76	108	113	145	75	128	82	103	238	337	86	110
0	96	0	143	0	104	0	102	0	314	0	97
2	460	5	2200	8	1384	2	1148	4	2410	3	1329
468	543	1999	2401	1417	1626	995	1284	2576	2872	1311	1542
78	104	130	150	78	119	91	102	248	326	82	109
2	89	3	227	1	164	1	128	8	433	4	253
0	105	0	153	0	128	0	106	0	330	0	112
6	88	1	153	3	92	5	106	15	276	1	94
3	89	7	155	2	92	8	108	6	269	9	94
3	115	13	500	5	218	11	257	16	428	9	357
2	117	3	511	2	218	0	259	5	426	1	355
1	116	1	508	0	217	1	258	1	421	1	353
0	89	3	195	0	96	0	153	2	279	0	116
2	116	1	496	0	214	0	250	0	443	3	350
0	116	0	494	0	213	0	252	0	441	2	350
2	669	64	2011	5	1115	45	1717	7	2182	1	1054
0	675	5	2019	0	1118	5	1724	8	2195	0	1058
0	125	0	554	1	251	1	284	0	459	0	470
5	143	2	618	0	281	1	328	9	495	11	523
3	672	5	2017	1	1114	1	1722	5	2189	4	1059
4	672	2	2018	0	1114	5	1721	5	2186	8	1059
0	153	1	729	0	327	1	386	0	513	0	565
0	239	0	1117	4	588	0	605	0	885	1	779
2	531	1	2400	9	1601	1	1280	18	2724	1	1503
1	530	0	2400	2	1596	1	1280	5	2713	1	1504
0	528	3	2400	2	1594	0	1276	3	2702	2	1501
358	528	1592	2399	1074	1591	847	1275	1889	2696	1042	1500
430	672	1048	2014	691	1108	1070	1718	1244	2165	693	1057
9	668	3	2013	4	1109	3	1716	5	2159	1	1057
10	527	3	2398	5	1594	9	1273	20	2688	20	1497
2	527	6	2397	6	1593	2	1273	6	2687	2	1495
11	670	140	2012	22	1107	1	1716	45	2151	38	1057
3	527	1	2396	26	1590	7	1273	37	2685	2	1496
5	668	6	2004	8	1105	4	1712	16	2146	1	1057
0	527	2	2396	1	1592	2	1272	4	2679	0	1494
0	668	3	2009	1	1108	1	1713	4	2149	4	1054
1	668	4	2010	7	1107	6	1714	17	2152	1	1052
1	525	2	2395	8	1589	3	1272	3	2673	6	1491
7	667	2	2008	10	1106	55	1715	16	2140	0	1053

chr5	64987553	G	0	668	3	2007	2	1105	5	1710	7	2136	2	1051
chr5	64987554	G	2	666	4	2005	3	1103	5	1708	4	2138	1	1052
chr5	64987555	C	398	127	1714	673	1214	366	785	480	2128	535	1194	284
chr5	64987556	G	486	180	1472	535	769	337	1102	612	1453	684	824	227
chr5	64987558	G	4	661	3	2003	2	1105	3	1711	4	2132	4	1047
chr5	64987559	C	0	516	1	2378	2	1576	2	1258	12	2634	2	1466
chr5	64987561	C	4	512	22	2355	10	1566	0	1259	9	2627	8	1455
chr5	64987563	C	4	509	2	2373	14	1559	2	1256	10	2619	17	1440
chr5	64987565	G	1	660	2	2007	7	1100	2	1711	20	2111	2	1045
chr5	64987569	C	0	511	1	2370	0	1565	1	1253	6	2598	13	1433
chr5	64987571	G	0	662	0	2007	7	1100	1	1711	11	2115	2	1042
chr5	64987574	G	0	663	3	2004	1	1104	3	1711	3	2121	0	1041
chr5	64987581	C	4	496	1	2353	11	1526	0	1246	14	2542	29	1386
chr5	64987583	C	1	499	4	2350	7	1528	4	1240	15	2537	10	1404
chr5	64987587	G	0	656	4	1997	2	1094	4	1709	6	2101	9	1025
chr5	64987591	G	0	656	4	1997	4	1092	5	1708	6	2102	3	1031
chr5	64987595	G	1	655	1	1997	0	1095	2	1710	5	2100	2	1031
chr5	64987596	C	0	496	8	2340	4	1528	1	1243	10	2537	3	1400
chr5	64987604	C	2	492	85	2256	31	1500	20	1220	67	2472	2	1387
chr5	64987607	C	1	493	6	2337	2	1528	0	1239	2	2538	2	1386
chr5	64987610	G	2	653	3	1990	4	1092	3	1704	11	2094	0	1034
chr5	64987611	G	3	652	3	1987	1	1095	0	1710	5	2100	3	1031
chr5	64987614	C	0	492	3	2316	3	1512	4	1210	3	2513	6	1364
chr5	64987615	C	2	491	2	2331	4	1527	2	1233	9	2523	5	1375
chr5	64987616	C	4	489	5	2331	8	1523	3	1232	42	2491	17	1363
chr5	64987618	C	0	487	11	2323	5	1510	3	1230	3	2508	8	1361
chr5	64987623	G	0	654	1	1984	0	1094	0	1707	0	2101	0	1029
chr5	64987624	C	2	484	0	2306	7	1503	1	1222	11	2494	3	1357
chr5	64987626	C	2	479	4	2271	7	1500	2	1210	27	2477	3	1345
chr5	64987631	G	0	654	0	1982	0	1092	0	1707	0	2098	0	1029
chr5	64987632	C	15	445	52	1997	49	1444	17	1151	115	2382	20	1312
chr5	64987634	G	0	652	1	1980	0	1092	0	1705	0	2097	0	1029
chr5	64987635	C	0	443	2	1968	0	1436	0	1066	0	2448	0	1216
chr5	64987637	C	1	447	0	1968	0	1444	0	1067	1	2478	0	1224
chr5	64987640	C	0	448	0	1965	1	1442	0	1066	0	2478	0	1224
chr5	64987641	C	0	448	1	1965	0	1443	1	1064	0	2477	0	1224
chr5	64987643	G	0	645	0	1928	0	1081	1	1642	0	2058	0	987
chr5	64987644	G	1	583	0	1635	1	958	0	1426	0	1694	0	838
chr5	64987649	C	0	431	1	1886	0	1390	0	1019	1	2383	0	1087
chr5	64987654	C	0	422	0	1861	0	1367	0	1002	2	2350	0	1046
chr5	64987656	C	0	411	1	1816	0	1352	0	975	1	2325	0	1018
chr5	64987657	C	0	409	0	1798	1	1340	0	968	0	2316	1	1006
chr5	64987658	C	0	407	1	1770	0	1336	0	959	0	2304	0	1001
chr5	64987661	C	0	398	0	1743	0	1313	0	938	1	2288	0	982
chr8	89376778	G	0	499	2	3710	0	5843	0	4228	1	6465	0	1242
chr8	89376779	G	0	501	0	3753	0	5883	1	4263	2	6496	1	1254

0	668	3	2010	2	1107	5	1715	7	2143	2	1053
2	668	4	2009	3	1106	5	1713	4	2142	1	1053
398	525	1714	2387	1214	1580	785	1265	2128	2663	1194	1478
486	666	1472	2007	769	1106	1102	1714	1453	2137	824	1051
4	665	3	2006	2	1107	3	1714	4	2136	4	1051
0	516	1	2379	2	1578	2	1260	12	2646	2	1468
4	516	22	2377	10	1576	0	1259	9	2636	8	1463
4	513	2	2375	14	1573	2	1258	10	2629	17	1457
1	661	2	2009	7	1107	2	1713	20	2131	2	1047
0	511	1	2371	0	1565	1	1254	6	2604	13	1446
0	662	0	2007	7	1107	1	1712	11	2126	2	1044
0	663	3	2007	1	1105	3	1714	3	2124	0	1041
4	500	1	2354	11	1537	0	1246	14	2556	29	1415
1	500	4	2354	7	1535	4	1244	15	2552	10	1414
0	656	4	2001	2	1096	4	1713	6	2107	9	1034
0	656	4	2001	4	1096	5	1713	6	2108	3	1034
1	656	1	1998	0	1095	2	1712	5	2105	2	1033
0	496	8	2348	4	1532	1	1244	10	2547	3	1403
2	494	85	2341	31	1531	20	1240	67	2539	2	1389
1	494	6	2343	2	1530	0	1239	2	2540	2	1388
2	655	3	1993	4	1096	3	1707	11	2105	0	1034
3	655	3	1990	1	1096	0	1710	5	2105	3	1034
0	492	3	2319	3	1515	4	1214	3	2516	6	1370
2	493	2	2333	4	1531	2	1235	9	2532	5	1380
4	493	5	2336	8	1531	3	1235	42	2533	17	1380
0	487	11	2334	5	1515	3	1233	3	2511	8	1369
0	654	1	1985	0	1094	0	1707	0	2101	0	1029
2	486	0	2306	7	1510	1	1223	11	2505	3	1360
2	481	4	2275	7	1507	2	1212	27	2504	3	1348
0	654	0	1982	0	1092	0	1707	0	2098	0	1029
15	460	52	2049	49	1493	17	1168	115	2497	20	1332
0	652	1	1981	0	1092	0	1705	0	2097	0	1029
0	443	2	1970	0	1436	0	1066	0	2448	0	1216
1	448	0	1968	0	1444	0	1067	1	2479	0	1224
0	448	0	1965	1	1443	0	1066	0	2478	0	1224
0	448	1	1966	0	1443	1	1065	0	2477	0	1224
0	645	0	1928	0	1081	1	1643	0	2058	0	987
1	584	0	1635	1	959	0	1426	0	1694	0	838
0	431	1	1887	0	1390	0	1019	1	2384	0	1087
0	422	0	1861	0	1367	0	1002	2	2352	0	1046
0	411	1	1817	0	1352	0	975	1	2326	0	1018
0	409	0	1798	1	1341	0	968	0	2316	1	1007
0	407	1	1771	0	1336	0	959	0	2304	0	1001
0	398	0	1743	0	1313	0	938	1	2289	0	982
0	499	2	3712	0	5843	0	4228	1	6466	0	1242
0	501	0	3753	0	5883	1	4264	2	6498	1	1255

chr8	89376781	G	0	512	0	3827	1	5946	1	4340	1	6562	0	1276
chr8	89376783	G	0	546	1	4033	0	6135	1	4552	0	6795	0	1340
chr8	89376785	C	0	1138	1	5654	0	1961	0	2953	0	1085	1	5083
chr8	89376788	C	0	1138	1	5661	0	1962	0	2955	0	1086	0	5083
chr8	89376790	c	0	1138	0	5669	0	1964	1	2954	0	1086	1	5083
chr8	89376791	c	0	1139	1	5680	1	1966	0	2955	0	1086	1	5085
chr8	89376793	g	0	775	0	6968	2	8268	2	7330	1	8928	1	2091
chr8	89376794	g	0	776	1	6972	2	8278	0	7340	0	8935	1	2094
chr8	89376795	c	0	1141	2	5687	0	1970	1	2958	0	1089	0	5091
chr8	89376796	c	0	1142	0	5691	0	1970	0	2957	1	1088	1	5090
chr8	89376798	g	1	784	0	7011	2	8301	2	7377	0	8956	0	2101
chr8	89376799	c	0	1142	2	5692	0	1970	1	2958	0	1089	0	5092
chr8	89376805	g	1	818	6	7389	6	8384	9	7455	6	9040	31	2085
chr8	89376807	c	0	1146	12	5693	0	1975	4	2962	5	1095	5	5103
chr8	89376808	c	1	1145	54	5651	5	1970	4	2963	0	1100	10	5098
chr8	89376811	c	2	1144	13	5694	4	1971	5	2960	0	1101	11	5096
chr8	89376815	g	13	811	252	7154	87	8315	35	7433	73	8993	57	2063
chr8	89376818	g	0	821	14	7406	28	8385	12	7461	26	9046	3	2120
chr8	89376819	c	3	1144	5	5701	3	1973	5	2965	5	1097	8	5102
chr8	89376820	c	4	1143	8	5701	4	1973	2	2967	5	1097	8	5106
chr8	89376823	c	883	262	4289	1422	1438	538	2106	860	790	311	3922	1192
chr8	89376824	g	642	194	4902	2494	5943	2465	5012	2432	7043	2052	1430	691
chr8	89376827	g	1	841	9	7425	20	8421	10	7472	19	9127	5	2136
chr8	89376829	c	1	1146	4	5709	0	1977	5	2966	2	1104	2	5109
chr8	89376830	c	2	1146	12	5704	2	1975	9	2962	0	1106	7	5111
chr8	89376831	c	1	1147	10	5707	6	1970	3	2968	0	1105	8	5111
chr8	89376833	g	2	842	8	7436	12	8439	6	7476	30	9138	1	2141
chr8	89376836	c	0	1148	11	5709	2	1977	0	2973	4	1104	9	5113
chr8	89376839	c	6	1141	87	5635	8	1971	9	2963	4	1100	7	5116
chr8	89376840	c	4	1144	5	5717	9	1970	7	2966	4	1104	5	5120
chr8	89376842	g	2	846	7	7437	17	8441	5	7482	12	9187	21	2130
chr8	89376844	c	4	1163	10	5713	3	1999	4	2967	4	1143	3	5139
chr8	89376845	c	4	1164	10	5715	6	1999	4	2976	6	1142	11	5139
chr8	89376847	c	6	1162	85	5641	19	1986	3	2978	8	1143	57	5093
chr8	89376849	g	3	847	15	7433	38	8430	11	7479	44	9176	10	2143
chr8	89376850	c	2	1159	14	5676	10	1976	11	2955	4	1139	16	5093
chr8	89376851	c	1	1169	6	5720	0	2007	6	2980	2	1150	9	5140
chr8	89376852	c	6	1164	115	5609	8	1999	8	2977	8	1145	9	5142
chr8	89376855	c	7	1150	7	5720	10	1973	35	2947	2	1118	5	5125
chr8	89376856	c	9	1148	8	5717	6	1977	1	2979	4	1117	24	5102
chr8	89376857	c	1002	167	4882	846	1766	242	2505	481	927	225	4041	1109
chr8	89376858	g	747	100	6249	1189	6947	1522	5866	1618	7920	1305	1661	494
chr8	89376859	c	13	1143	61	5663	28	1956	99	2880	12	1108	79	5047
chr8	89376861	c	2	1168	6	5724	4	2004	6	2982	2	1150	11	5142
chr8	89376862	c	26	1144	33	5695	40	1969	18	2969	6	1147	191	4960
chr8	89376865	c	5	1165	2	5727	20	1989	7	2984	7	1144	7	5144

0	512	0	3827	1	5947	1	4341	1	6563	0	1276
0	546	1	4034	0	6135	1	4553	0	6795	0	1340
0	1138	1	5655	0	1961	0	2953	0	1085	1	5084
0	1138	1	5662	0	1962	0	2955	0	1086	0	5083
0	1138	0	5669	0	1964	1	2955	0	1086	1	5084
0	1139	1	5681	1	1967	0	2955	0	1086	1	5086
0	775	0	6968	2	8270	2	7332	1	8929	1	2092
0	776	1	6973	2	8280	0	7340	0	8935	1	2095
0	1141	2	5689	0	1970	1	2959	0	1089	0	5091
0	1142	0	5691	0	1970	0	2957	1	1089	1	5091
1	785	0	7011	2	8303	2	7379	0	8956	0	2101
0	1142	2	5694	0	1970	1	2959	0	1089	0	5092
1	819	6	7395	6	8390	9	7464	6	9046	31	2116
0	1146	12	5705	0	1975	4	2966	5	1100	5	5108
1	1146	54	5705	5	1975	4	2967	0	1100	10	5108
2	1146	13	5707	4	1975	5	2965	0	1101	11	5107
13	824	252	7406	87	8402	35	7468	73	9066	57	2120
0	821	14	7420	28	8413	12	7473	26	9072	3	2123
3	1147	5	5706	3	1976	5	2970	5	1102	8	5110
4	1147	8	5709	4	1977	2	2969	5	1102	8	5114
883	1145	4289	5711	1438	1976	2106	2966	790	1101	3922	5114
642	836	4902	7396	5943	8408	5012	7444	7043	9095	1430	2121
1	842	9	7434	20	8441	10	7482	19	9146	5	2141
1	1147	4	5713	0	1977	5	2971	2	1106	2	5111
2	1148	12	5716	2	1977	9	2971	0	1106	7	5118
1	1148	10	5717	6	1976	3	2971	0	1105	8	5119
2	844	8	7444	12	8451	6	7482	30	9168	1	2142
0	1148	11	5720	2	1979	0	2973	4	1108	9	5122
6	1147	87	5722	8	1979	9	2972	4	1104	7	5123
4	1148	5	5722	9	1979	7	2973	4	1108	5	5125
2	848	7	7444	17	8458	5	7487	12	9199	21	2151
4	1167	10	5723	3	2002	4	2971	4	1147	3	5142
4	1168	10	5725	6	2005	4	2980	6	1148	11	5150
6	1168	85	5726	19	2005	3	2981	8	1151	57	5150
3	850	15	7448	38	8468	11	7490	44	9220	10	2153
2	1161	14	5690	10	1986	11	2966	4	1143	16	5109
1	1170	6	5726	0	2007	6	2986	2	1152	9	5149
6	1170	115	5724	8	2007	8	2985	8	1153	9	5151
7	1157	7	5727	10	1983	35	2982	2	1120	5	5130
9	1157	8	5725	6	1983	1	2980	4	1121	24	5126
1002	1169	4882	5728	1766	2008	2505	2986	927	1152	4041	5150
747	847	6249	7438	6947	8469	5866	7484	7920	9225	1661	2155
13	1156	61	5724	28	1984	99	2979	12	1120	79	5126
2	1170	6	5730	4	2008	6	2988	2	1152	11	5153
26	1170	33	5728	40	2009	18	2987	6	1153	191	5151
5	1170	2	5729	20	2009	7	2991	7	1151	7	5151

chr8	89376867	c	2	1169	5	5723	4	2005	2	2988	1	1155	9	5144
chr8	89376868	c	1	1170	6	5724	9	1999	4	2987	1	1155	7	5146
chr8	89376869	c	997	174	4215	1515	1692	314	2185	806	908	248	4217	931
chr8	89376870	g	720	132	5848	1597	6750	1725	5519	1967	7653	1592	1584	574
chr8	89376872	g	4	848	29	7426	17	8462	19	7473	34	9224	7	2151
chr8	89376873	c	8	1163	59	5675	8	1999	16	2976	3	1158	4	5151
chr8	89376875	c	10	1161	92	5643	23	1988	51	2941	19	1144	1	5153
chr8	89376877	g	4	852	8	7448	18	8473	9	7488	45	9233	2	2160
chr8	89376879	c	20	1151	71	5666	49	1962	51	2941	26	1139	167	4988
chr8	89376881	c	2	1168	8	5728	3	2010	8	2985	1	1165	11	5145
chr8	89376882	c	2	1169	7	5732	2	2013	1	2992	4	1162	2	5157
chr8	89376884	c	16	1155	94	5647	22	1992	3	2990	11	1156	112	5044
chr8	89376886	g	4	853	10	7446	9	8490	11	7485	16	9277	2	2161
chr8	89376887	g	2	856	11	7444	23	8475	14	7484	22	9268	20	2141
chr8	89376889	g	96	762	912	6549	771	7728	937	6559	908	8385	125	2039
chr8	89376891	g	18	840	421	7039	199	8300	266	7232	230	9064	29	2136
chr8	89376892	c	1099	71	5358	394	1914	99	2772	220	1105	73	4958	202
chr8	89376893	g	816	40	7188	267	7846	650	6936	555	8661	630	2010	155
chr8	89376894	c	15	1156	4	5750	19	1995	107	2889	5	1173	71	5091
chr8	89376897	c	5	1166	3	5749	4	2011	3	2994	1	1177	6	5159
chr8	89376898	c	0	1171	8	5746	4	2010	2	2994	0	1178	59	5105
chr8	89376902	c	65	1105	613	5142	146	1868	198	2798	71	1112	194	4971
chr8	89376904	G	2	857	234	7229	35	8466	17	7480	43	9266	2	2165
chr8	89376906	C	2	1169	7	5747	4	2009	4	2993	4	1178	11	5147
chr8	89376911	C	1076	96	5469	292	1884	132	2687	312	1045	139	4593	574
chr8	89376912	G	779	80	6890	572	7404	1102	6951	556	8292	1024	1891	276
chr8	89376916	G	9	851	58	7404	31	8477	10	7497	57	9261	13	2154
chr8	89376917	C	5	1167	8	5751	7	2008	9	2991	5	1180	10	5157
chr8	89376925	C	6	1166	18	5743	14	2002	65	2934	7	1179	13	5152
chr8	89376926	C	1040	131	4960	801	1793	223	2381	619	1007	178	4470	696
chr8	89376927	G	755	103	6494	959	7100	1387	6476	1027	8110	1203	1795	373
chr8	89376928	G	0	404	7	4333	3	2936	2	3774	7	3154	2	1044
chr8	89376930	C	13	1158	28	5721	18	1997	68	2928	10	1176	239	4924
chr8	89376932	C	7	1162	4	5735	15	2000	3	2986	10	1174	9	5140
chr8	89376935	C	3	1164	109	5626	8	2007	76	2910	2	1180	9	5139
chr8	89376943	G	0	138	4	1721	0	665	3	1467	0	808	0	391
chr8	89376948	C	3	1166	5	5731	5	2010	3	2984	2	1180	5	5141
chr8	89376950	G	1	141	1	1922	3	781	1	1602	2	935	1	424
chr8	89376952	C	2	1166	3	5732	1	2013	3	2984	0	1179	7	5141
chr8	89376956	C	6	1163	3	5732	8	2007	62	2925	2	1177	55	5091
chr8	89376959	G	2	848	9	7448	13	8463	13	7485	20	9241	4	2134
chr8	89376962	G	1	843	8	7446	35	8435	12	7485	22	9227	3	2130
chr8	89376964	G	8	840	107	7351	112	8359	117	7378	95	9157	10	2122
chr8	89376965	G	0	849	11	7444	43	8423	16	7476	35	9213	0	2132
chr8	89376966	C	3	1162	13	5717	4	2006	5	2981	2	1174	17	5129
chr8	89376967	C	2	1163	11	5719	6	2006	5	2981	6	1170	12	5132

2	1171	5	5728	4	2009	2	2990	1	1156	9	5153
1	1171	6	5730	9	2008	4	2991	1	1156	7	5153
997	1171	4215	5730	1692	2006	2185	2991	908	1156	4217	5148
720	852	5848	7445	6750	8475	5519	7486	7653	9245	1584	2158
4	852	29	7455	17	8479	19	7492	34	9258	7	2158
8	1171	59	5734	8	2007	16	2992	3	1161	4	5155
10	1171	92	5735	23	2011	51	2992	19	1163	1	5154
4	856	8	7456	18	8491	9	7497	45	9278	2	2162
20	1171	71	5737	49	2011	51	2992	26	1165	167	5155
2	1170	8	5736	3	2013	8	2993	1	1166	11	5156
2	1171	7	5739	2	2015	1	2993	4	1166	2	5159
16	1171	94	5741	22	2014	3	2993	11	1167	112	5156
4	857	10	7456	9	8499	11	7496	16	9293	2	2163
2	858	11	7455	23	8498	14	7498	22	9290	20	2161
96	858	912	7461	771	8499	937	7496	908	9293	125	2164
18	858	421	7460	199	8499	266	7498	230	9294	29	2165
1099	1170	5358	5752	1914	2013	2772	2992	1105	1178	4958	5160
816	856	7188	7455	7846	8496	6936	7491	8661	9291	2010	2165
15	1171	4	5754	19	2014	107	2996	5	1178	71	5162
5	1171	3	5752	4	2015	3	2997	1	1178	6	5165
0	1171	8	5754	4	2014	2	2996	0	1178	59	5164
65	1170	613	5755	146	2014	198	2996	71	1183	194	5165
2	859	234	7463	35	8501	17	7497	43	9309	2	2167
2	1171	7	5754	4	2013	4	2997	4	1182	11	5158
1076	1172	5469	5761	1884	2016	2687	2999	1045	1184	4593	5167
779	859	6890	7462	7404	8506	6951	7507	8292	9316	1891	2167
9	860	58	7462	31	8508	10	7507	57	9318	13	2167
5	1172	8	5759	7	2015	9	3000	5	1185	10	5167
6	1172	18	5761	14	2016	65	2999	7	1186	13	5165
1040	1171	4960	5761	1793	2016	2381	3000	1007	1185	4470	5166
755	858	6494	7453	7100	8487	6476	7503	8110	9313	1795	2168
0	404	7	4340	3	2939	2	3776	7	3161	2	1046
13	1171	28	5749	18	2015	68	2996	10	1186	239	5163
7	1169	4	5739	15	2015	3	2989	10	1184	9	5149
3	1167	109	5735	8	2015	76	2986	2	1182	9	5148
0	138	4	1725	0	665	3	1470	0	808	0	391
3	1169	5	5736	5	2015	3	2987	2	1182	5	5146
1	142	1	1923	3	784	1	1603	2	937	1	425
2	1168	3	5735	1	2014	3	2987	0	1179	7	5148
6	1169	3	5735	8	2015	62	2987	2	1179	55	5146
2	850	9	7457	13	8476	13	7498	20	9261	4	2138
1	844	8	7454	35	8470	12	7497	22	9249	3	2133
8	848	107	7458	112	8471	117	7495	95	9252	10	2132
0	849	11	7455	43	8466	16	7492	35	9248	0	2132
3	1165	13	5730	4	2010	5	2986	2	1176	17	5146
2	1165	11	5730	6	2012	5	2986	6	1176	12	5144

chr8	89376968	C	8	1158	1	5728	9	2003	53	2932	1	1172	0	5145
chr8	89376971	G	3	834	13	7436	13	8444	6	7479	35	9200	2	2126
chr8	89376973	G	11	825	261	7184	90	8361	74	7405	118	9108	40	2086
chr8	89376974	C	1	1162	9	5717	12	1999	4	2973	4	1163	6	5131
chr8	89376976	C	877	282	4122	1600	1564	442	2165	811	828	333	3972	1164
chr8	89376977	G	663	173	6183	1258	6339	2114	5696	1786	7219	2006	1503	621
chr8	89376981	C	6	1157	9	5709	14	1994	15	2960	2	1160	7	5126
chr8	89376986	C	2	1159	13	5698	13	1992	1	2968	5	1154	6	5119
chr8	89376990	G	2	832	26	7412	29	8420	19	7457	45	9170	15	2099
chr8	89376991	C	50	1107	145	5563	113	1886	139	2824	55	1095	307	4812
chr8	89376993	C	3	1155	12	5691	7	1993	5	2954	5	1140	3	5114
chr8	89376994	C	1	1157	6	5694	3	1997	2	2958	4	1141	3	5112
chr8	89376999	G	6	821	5	7383	55	8359	8	7372	52	9136	20	2044
chr8	89377002	G	7	820	189	7200	131	8279	14	7360	113	9072	65	1999
chr8	89377009	G	5	819	371	7006	4	8398	4	7366	38	9142	1	2056
chr8	89377012	G	0	824	21	7360	41	8365	26	7344	33	9149	25	2031
chr8	89377013	C	1	1156	6	5670	3	1997	6	2946	4	1130	3	5109
chr8	89377023	C	2	1155	8	5649	2	1997	6	2943	4	1130	6	5104
chr8	89377026	G	0	820	15	7357	15	8388	13	7351	12	9161	3	2051
chr8	89377027	C	1	1155	14	5642	4	1995	3	2946	0	1134	8	5102
chr8	89377028	C	3	1153	5	5651	1	1997	4	2946	3	1129	54	5055
chr8	89377030	G	0	819	11	7354	25	8375	11	7346	15	9154	22	2030
chr8	89377037	G	3	811	6	7302	53	8297	9	7309	28	9066	3	2029
chr8	89377038	G	2	815	9	7345	9	8380	49	7299	13	9145	3	2046
chr8	89377042	G	3	815	10	7346	18	8372	15	7330	22	9132	0	2047
chr8	89377045	C	1	1149	0	5519	0	1996	0	2944	0	1131	2	5104
chr8	89377047	C	0	1151	1	5509	0	1996	1	2939	0	1128	2	5101
chr8	89377049	G	5	812	192	7161	12	8374	7	7338	34	9109	36	2010
chr8	89377050	G	0	817	11	7344	29	8354	11	7330	18	9130	1	2045
chr8	89377051	G	4	813	16	7337	14	8352	11	7327	23	9109	2	2041
chr8	89377052	C	1	1145	0	5473	0	1993	0	2934	1	1121	0	5086
chr8	89377053	C	0	1142	0	5430	0	1988	0	2925	0	1105	0	5069
chr8	89377055	C	0	1140	0	5408	1	1984	0	2922	0	1096	2	5063
chr8	89377057	C	0	1136	1	5383	0	1980	1	2917	0	1087	0	5053
chr8	89377058	C	0	1140	0	5382	0	1981	1	2918	0	1092	1	5059
chr8	89377060	G	11	802	402	6914	148	8206	154	7152	144	8964	11	2019
chr8	89377062	G	4	809	57	7256	69	8285	85	7213	32	9073	2	2026
chr8	89377069	G	0	813	8	7300	23	8325	122	7170	12	9087	4	2020
chr8	89377070	G	1	812	17	7291	13	8334	8	7284	12	9085	0	2024
chr8	89377076	G	760	51	6687	230	7659	662	6179	1021	8515	567	1828	188
chr8	89377081	G	0	802	1	6355	1	8255	1	6720	1	9041	0	1998
chr8	89377091	G	0	785	3	6168	0	8029	0	6212	0	8830	0	1834
chr8	89377093	G	1	784	0	6155	0	8031	1	6197	1	8833	0	1829
chr8	89377094	G	2	784	0	6155	1	8029	0	6199	1	8837	0	1830
chr8	89377095	G	0	786	1	6155	1	8032	1	6198	1	8838	0	1830
chr8	120115553	G	0	2511	2	12537	1	6349	0	7435	1	7325	1	5671

8	1166	1	5729	9	2012	53	2985	1	1173	0	5145
3	837	13	7449	13	8457	6	7485	35	9235	2	2128
11	836	261	7445	90	8451	74	7479	118	9226	40	2126
1	1163	9	5726	12	2011	4	2977	4	1167	6	5137
877	1159	4122	5722	1564	2006	2165	2976	828	1161	3972	5136
663	836	6183	7441	6339	8453	5696	7482	7219	9225	1503	2124
6	1163	9	5718	14	2008	15	2975	2	1162	7	5133
2	1161	13	5711	13	2005	1	2969	5	1159	6	5125
2	834	26	7438	29	8449	19	7476	45	9215	15	2114
50	1157	145	5708	113	1999	139	2963	55	1150	307	5119
3	1158	12	5703	7	2000	5	2959	5	1145	3	5117
1	1158	6	5700	3	2000	2	2960	4	1145	3	5115
6	827	5	7388	55	8414	8	7380	52	9188	20	2064
7	827	189	7389	131	8410	14	7374	113	9185	65	2064
5	824	371	7377	4	8402	4	7370	38	9180	1	2057
0	824	21	7381	41	8406	26	7370	33	9182	25	2056
1	1157	6	5676	3	2000	6	2952	4	1134	3	5112
2	1157	8	5657	2	1999	6	2949	4	1134	6	5110
0	820	15	7372	15	8403	13	7364	12	9173	3	2054
1	1156	14	5656	4	1999	3	2949	0	1134	8	5110
3	1156	5	5656	1	1998	4	2950	3	1132	54	5109
0	819	11	7365	25	8400	11	7357	15	9169	22	2052
3	814	6	7308	53	8350	9	7318	28	9094	3	2032
2	817	9	7354	9	8389	49	7348	13	9158	3	2049
3	818	10	7356	18	8390	15	7345	22	9154	0	2047
1	1150	0	5519	0	1996	0	2944	0	1131	2	5106
0	1151	1	5510	0	1996	1	2940	0	1128	2	5103
5	817	192	7353	12	8386	7	7345	34	9143	36	2046
0	817	11	7355	29	8383	11	7341	18	9148	1	2046
4	817	16	7353	14	8366	11	7338	23	9132	2	2043
1	1146	0	5473	0	1993	0	2934	1	1122	0	5086
0	1142	0	5430	0	1988	0	2925	0	1105	0	5069
0	1140	0	5408	1	1985	0	2922	0	1096	2	5065
0	1136	1	5384	0	1980	1	2918	0	1087	0	5053
0	1140	0	5382	0	1981	1	2919	0	1092	1	5060
11	813	402	7316	148	8354	154	7306	144	9108	11	2030
4	813	57	7313	69	8354	85	7298	32	9105	2	2028
0	813	8	7308	23	8348	122	7292	12	9099	4	2024
1	813	17	7308	13	8347	8	7292	12	9097	0	2024
760	811	6687	6917	7659	8321	6179	7200	8515	9082	1828	2016
0	802	1	6356	1	8256	1	6721	1	9042	0	1998
0	785	3	6171	0	8029	0	6212	0	8830	0	1834
1	785	0	6155	0	8031	1	6198	1	8834	0	1829
2	786	0	6155	1	8030	0	6199	1	8838	0	1830
0	786	1	6156	1	8033	1	6199	1	8839	0	1830
0	2511	2	12539	1	6350	0	7435	1	7326	1	5672

chr8	120115555	G	1	2524	2	12621	1	6377	4	7476	2	7386	1	5706
chr8	120115560	G	0	2547	2	12644	0	6400	0	7500	0	7509	0	5743
chr8	120115562	G	0	2552	3	12653	1	6404	0	7511	0	7512	3	5748
chr8	120115563	G	0	2555	20	12673	3	6405	15	7513	7	7513	8	5755
chr8	120115567	G	28	2526	143	12567	67	6344	146	7379	124	7400	5	5760
chr8	120115568	G	1	2554	22	12699	3	6411	14	7520	17	7510	10	5757
chr8	120115569	G	3	2552	30	12695	5	6412	7	7525	12	7519	8	5759
chr8	120115570	G	5	2550	19	12705	18	6400	12	7525	13	7520	31	5738
chr8	120115575	G	3	2554	12	12725	5	6414	6	7533	24	7511	9	5762
chr8	120115582	G	4	2553	196	12536	10	6415	10	7540	21	7534	8	5765
chr8	120115584	G	7	2554	36	12722	11	6414	36	7522	18	7550	13	5767
chr8	120115588	G	2376	171	10735	1954	5820	579	6855	667	6961	576	5176	585
chr8	120115589	G	9	2553	9	12739	25	6396	11	7545	24	7551	11	5772
chr8	120115593	G	15	2549	185	12578	17	6409	58	7504	52	7528	21	5764
chr8	120115594	G	6	2559	17	12747	11	6418	8	7554	11	7571	4	5784
chr8	120115595	G	7	2558	32	12734	9	6422	10	7553	11	7570	18	5767
chr8	120115599	G	2456	110	11512	1253	5963	467	6849	718	7124	460	5025	765
chr8	120115602	G	23	2548	22	12758	34	6402	9	7562	48	7545	15	5782
chr8	120115604	G	37	2534	283	12498	92	6344	84	7486	67	7530	41	5756
chr8	120115606	C	0	727	0	464	0	539	0	957	0	542	0	958
chr8	120115608	G	161	2410	656	12126	463	5975	664	6906	610	6989	328	5469
chr8	120115610	G	32	2539	259	12528	43	6398	16	7556	56	7548	88	5714
chr8	120115611	C	0	730	0	467	0	539	1	958	0	543	0	963
chr8	120115614	G	28	2545	343	12454	163	6282	244	7331	177	7435	202	5605
chr8	120115616	C	1	733	0	467	0	539	0	961	0	543	0	962
chr8	120115619	G	10	2703	27	12768	26	6533	25	7575	48	7938	18	5966
chr8	120115620	G	5	2703	29	12767	21	6532	10	7590	23	7917	12	5954
chr8	120115621	C	0	708	0	456	1	523	0	922	0	516	1	925
chr8	120115623	C	1	733	0	467	0	541	0	963	0	545	0	964
chr8	120115624	C	0	734	0	466	0	541	0	963	0	545	0	964
chr8	120115625	C	0	732	0	467	0	541	0	963	0	543	0	964
chr8	120115627	C	0	733	1	467	5	536	3	960	4	539	19	945
chr8	120115629	G	66	2651	105	12703	156	6408	240	7371	134	7883	82	5919
chr8	120115631	G	12	2718	10	12801	11	6567	7	7615	29	8012	22	5996
chr8	120115632	G	2	2728	19	12792	12	6568	13	7607	22	8022	9	6011
chr8	120115634	C	2	732	1	468	0	541	3	960	2	543	0	964
chr8	120115638	G	7	3287	23	12787	12	6888	15	7748	14	8804	6	6628
chr8	120115639	G	7	3289	21	12793	11	6896	12	7756	24	8808	11	6635
chr8	120115643	C	1	733	0	469	0	544	2	961	1	544	0	964
chr8	120115645	C	23	711	1	468	25	519	18	945	20	525	15	949
chr8	120115647	G	12	3317	22	12793	58	6873	59	7721	73	8848	50	6681
chr8	120115652	G	18	3312	25	12788	32	6899	14	7764	34	8897	14	6720
chr8	120115653	G	7	3325	39	12780	16	6919	9	7773	10	8927	13	6720
chr8	120115654	G	8	3325	23	12800	19	6916	22	7762	27	8920	9	6732
chr8	120115655	C	1	733	1	468	4	539	5	957	5	540	4	959
chr8	120115657	C	1	735	0	469	2	542	0	963	0	546	3	963

1	2525	2	12623	1	6378	4	7480	2	7388	1	5707
0	2547	2	12646	0	6400	0	7500	0	7509	0	5743
0	2552	3	12656	1	6405	0	7511	0	7512	3	5751
0	2555	20	12693	3	6408	15	7528	7	7520	8	5763
28	2554	143	12710	67	6411	146	7525	124	7524	5	5765
1	2555	22	12721	3	6414	14	7534	17	7527	10	5767
3	2555	30	12725	5	6417	7	7532	12	7531	8	5767
5	2555	19	12724	18	6418	12	7537	13	7533	31	5769
3	2557	12	12737	5	6419	6	7539	24	7535	9	5771
4	2557	196	12732	10	6425	10	7550	21	7555	8	5773
7	2561	36	12758	11	6425	36	7558	18	7568	13	5780
2376	2547	10735	12689	5820	6399	6855	7522	6961	7537	5176	5761
9	2562	9	12748	25	6421	11	7556	24	7575	11	5783
15	2564	185	12763	17	6426	58	7562	52	7580	21	5785
6	2565	17	12764	11	6429	8	7562	11	7582	4	5788
7	2565	32	12766	9	6431	10	7563	11	7581	18	5785
2456	2566	11512	12765	5963	6430	6849	7567	7124	7584	5025	5790
23	2571	22	12780	34	6436	9	7571	48	7593	15	5797
37	2571	283	12781	92	6436	84	7570	67	7597	41	5797
0	727	0	464	0	539	0	957	0	542	0	958
161	2571	656	12782	463	6438	664	7570	610	7599	328	5797
32	2571	259	12787	43	6441	16	7572	56	7604	88	5802
0	730	0	467	0	539	1	959	0	543	0	963
28	2573	343	12797	163	6445	244	7575	177	7612	202	5807
1	734	0	467	0	539	0	961	0	543	0	962
10	2713	27	12795	26	6559	25	7600	48	7986	18	5984
5	2708	29	12796	21	6553	10	7600	23	7940	12	5966
0	708	0	456	1	524	0	922	0	516	1	926
1	734	0	467	0	541	0	963	0	545	0	964
0	734	0	466	0	541	0	963	0	545	0	964
0	732	0	467	0	541	0	963	0	543	0	964
0	733	1	468	5	541	3	963	4	543	19	964
66	2717	105	12808	156	6564	240	7611	134	8017	82	6001
12	2730	10	12811	11	6578	7	7622	29	8041	22	6018
2	2730	19	12811	12	6580	13	7620	22	8044	9	6020
2	734	1	469	0	541	3	963	2	545	0	964
7	3294	23	12810	12	6900	15	7763	14	8818	6	6634
7	3296	21	12814	11	6907	12	7768	24	8832	11	6646
1	734	0	469	0	544	2	963	1	545	0	964
23	734	1	469	25	544	18	963	20	545	15	964
12	3329	22	12815	58	6931	59	7780	73	8921	50	6731
18	3330	25	12813	32	6931	14	7778	34	8931	14	6734
7	3332	39	12819	16	6935	9	7782	10	8937	13	6733
8	3333	23	12823	19	6935	22	7784	27	8947	9	6741
1	734	1	469	4	543	5	962	5	545	4	963
1	736	0	469	2	544	0	963	0	546	3	966

chr8	120115659	C	658	78	424	44	493	51	865	98	500	46	868	98
chr8	120115660	G	3016	328	11401	1434	6212	731	6724	1063	7940	1043	5728	1017
chr8	120115664	G	49	3295	35	12775	65	6881	9	7776	70	8927	87	6664
chr8	120115665	C	7	728	7	461	3	541	1	962	3	543	0	966
chr8	120115669	C	1	735	0	469	1	543	0	963	0	546	1	965
chr8	120115671	C	682	54	424	45	508	36	884	79	510	36	830	136
chr8	120115672	G	3022	332	10869	1970	6335	617	6893	902	8214	825	6039	735
chr8	120115673	G	13	3342	16	12816	12	6947	22	7774	25	9015	42	6731
chr8	120115674	C	6	730	4	465	4	538	6	957	2	543	5	961
chr8	120115676	G	74	3283	107	12734	87	6871	10	7786	154	8892	40	6737
chr8	120115679	C	717	19	402	67	533	11	901	61	530	15	849	114
chr8	120115680	G	3114	244	11929	915	6532	428	6972	825	8396	651	6289	489
chr8	120115683	G	20	3341	18	12824	42	6924	8	7790	44	9017	8	6779
chr8	120115686	C	16	720	3	465	9	534	4	959	7	539	17	949
chr8	120115688	C	1	735	0	469	0	543	3	960	3	543	0	965
chr8	120115690	C	673	62	445	24	504	38	894	69	508	38	831	135
chr8	120115691	G	843	149	627	59	776	126	491	124	1812	354	1219	223
chr8	120115692	C	5	731	0	469	0	543	1	960	8	537	1	962
chr8	120115695	G	3	1006	8	614	3	907	1	612	4	2197	1	1457
chr8	120115697	C	3	733	1	468	1	542	1	962	3	548	1	965
chr8	120115698	C	696	40	431	38	529	14	920	43	519	32	881	85
chr8	120115699	G	901	108	516	71	853	82	497	106	1932	255	1250	221
chr8	120115702	G	2	1365	2	2601	10	1822	3	1799	13	3239	2	2371
chr8	120115705	C	3	733	2	467	5	538	3	960	3	548	1	965
chr8	120115707	C	8	728	1	468	8	535	3	960	7	544	17	948
chr8	120115709	G	8	3359	31	12812	17	6946	23	7792	19	9103	24	6775
chr8	120115710	C	0	736	1	468	1	542	3	960	0	551	2	964
chr8	120115711	C	4	732	7	462	7	536	4	959	4	547	9	956
chr8	120115713	G	27	3341	285	12565	78	6893	62	7752	89	9032	92	6715
chr8	120115716	C	7	729	1	468	19	524	105	858	11	540	5	961
chr8	120115718	C	16	720	0	469	24	519	109	854	21	530	31	935
chr8	120115720	C	36	700	6	463	37	506	95	868	20	531	41	925
chr8	120115722	C	3	733	0	469	12	531	0	963	6	545	4	962
chr8	120115725	G	11	3327	22	12785	17	6917	64	7708	18	9056	4	6770
chr8	120115726	C	5	731	10	459	3	540	9	954	2	549	1	965
chr8	120115728	C	2	733	0	469	2	541	3	960	6	545	5	961
chr8	120115730	C	4	732	0	469	0	543	5	958	2	548	1	965
chr8	120115732	C	574	162	374	95	466	77	842	121	460	91	807	159
chr8	120115733	G	2681	662	10415	2431	5443	1491	6405	1368	7027	1995	5372	1352
chr8	120115736	C	3	733	0	469	4	539	9	954	3	547	3	963
chr8	120115741	C	15	721	0	469	30	512	12	950	22	529	22	944
chr8	120115745	C	629	107	365	104	481	62	833	129	484	67	831	135
chr8	120115746	G	2922	422	10561	2278	6144	785	6448	1320	7989	1029	5876	846
chr8	120115751	G	19	3321	11	12823	44	6886	16	7752	47	8966	66	6655
chr8	120115752	C	9	724	0	469	18	524	2	961	18	533	5	961
chr8	120115756	C	7	725	2	467	1	542	4	958	1	550	1	964

658	736	424	468	493	544	865	963	500	546	868	966
3016	3344	11401	12835	6212	6943	6724	7787	7940	8983	5728	6745
49	3344	35	12810	65	6946	9	7785	70	8997	87	6751
7	735	7	468	3	544	1	963	3	546	0	966
1	736	0	469	1	544	0	963	0	546	1	966
682	736	424	469	508	544	884	963	510	546	830	966
3022	3354	10869	12839	6335	6952	6893	7795	8214	9039	6039	6774
13	3355	16	12832	12	6959	22	7796	25	9040	42	6773
6	736	4	469	4	542	6	963	2	545	5	966
74	3357	107	12841	87	6958	10	7796	154	9046	40	6777
717	736	402	469	533	544	901	962	530	545	849	963
3114	3358	11929	12844	6532	6960	6972	7797	8396	9047	6289	6778
20	3361	18	12842	42	6966	8	7798	44	9061	8	6787
16	736	3	468	9	543	4	963	7	546	17	966
1	736	0	469	0	543	3	963	3	546	0	965
673	735	445	469	504	542	894	963	508	546	831	966
843	992	627	686	776	902	491	615	1812	2166	1219	1442
5	736	0	469	0	543	1	961	8	545	1	963
3	1009	8	622	3	910	1	613	4	2201	1	1458
3	736	1	469	1	543	1	963	3	551	1	966
696	736	431	469	529	543	920	963	519	551	881	966
901	1009	516	587	853	935	497	603	1932	2187	1250	1471
2	1367	2	2603	10	1832	3	1802	13	3252	2	2373
3	736	2	469	5	543	3	963	3	551	1	966
8	736	1	469	8	543	3	963	7	551	17	965
8	3367	31	12843	17	6963	23	7815	19	9122	24	6799
0	736	1	469	1	543	3	963	0	551	2	966
4	736	7	469	7	543	4	963	4	551	9	965
27	3368	285	12850	78	6971	62	7814	89	9121	92	6807
7	736	1	469	19	543	105	963	11	551	5	966
16	736	0	469	24	543	109	963	21	551	31	966
36	736	6	469	37	543	95	963	20	551	41	966
3	736	0	469	12	543	0	963	6	551	4	966
11	3338	22	12807	17	6934	64	7772	18	9074	4	6774
5	736	10	469	3	543	9	963	2	551	1	966
2	735	0	469	2	543	3	963	6	551	5	966
4	736	0	469	0	543	5	963	2	550	1	966
574	736	374	469	466	543	842	963	460	551	807	966
2681	3343	10415	12846	5443	6934	6405	7773	7027	9022	5372	6724
3	736	0	469	4	543	9	963	3	550	3	966
15	736	0	469	30	542	12	962	22	551	22	966
629	736	365	469	481	543	833	962	484	551	831	966
2922	3344	10561	12839	6144	6929	6448	7768	7989	9018	5876	6722
19	3340	11	12834	44	6930	16	7768	47	9013	66	6721
9	733	0	469	18	542	2	963	18	551	5	966
7	732	2	469	1	543	4	962	1	551	1	965

chr8	120115758	C	3	729	4	465	13	528	4	956	8	542	45	920
chr8	120115761	G	4	3336	22	12810	11	6916	18	7750	16	8990	11	6706
chr8	120115762	G	9	3328	29	12809	17	6904	13	7747	13	8991	43	6652
chr8	120115764	C	521	203	348	115	403	137	762	191	401	148	669	286
chr8	120115765	G	2677	656	9516	3306	5442	1466	5698	2055	7163	1830	5111	1589
chr8	120115767	G	10	3327	128	12709	21	6897	11	7749	26	8969	9	6697
chr8	120115770	C	1	589	1	386	1	439	0	729	2	451	2	774
chr8	120115771	C	1	663	4	429	4	493	8	834	2	499	2	862
chr8	120115775	C	47	670	15	452	42	495	16	934	39	502	62	885
chr8	120115777	C	25	692	11	456	22	515	24	927	15	527	41	905
chr8	120115778	C	29	683	13	454	28	491	17	932	23	492	55	886
chr8	120115782	C	66	643	101	362	35	483	17	933	25	486	28	910
chr8	120115783	C	24	687	13	453	7	512	93	857	9	503	53	885
chr8	120115784	C	60	647	44	420	84	432	211	732	65	444	81	856
chr8	120115786	C	6	704	6	460	2	518	7	942	6	501	12	925
chr8	120115793	C	3	706	0	466	1	518	0	947	1	505	3	936
chr8	120115794	C	578	118	395	60	462	46	735	195	438	56	751	176
chr8	120115795	G	2464	864	9471	3328	5129	1776	5483	2260	6468	2500	4722	1966
chr8	120115796	C	597	106	387	74	486	32	819	120	466	28	824	107
chr8	120115797	G	2451	408	9152	1786	5074	934	5426	1193	6593	1104	4965	697
chr8	120115801	G	4	3326	19	12797	17	6896	6	7746	23	8949	4	6691
chr8	120115803	G	45	3286	23	12794	90	6826	16	7734	77	8894	88	6606
chr8	120115805	G	146	3182	763	12050	370	6545	505	7244	356	8611	287	6407
chr8	120115807	G	66	3262	198	12608	107	6804	198	7547	207	8760	208	6483
chr8	120115810	C	1	707	1	462	1	516	2	943	6	494	4	933
chr8	120115811	C	94	613	26	437	86	429	216	724	79	416	112	824
chr8	120115813	G	12	3313	19	12781	12	6895	12	7728	19	8937	7	6678
chr8	120115814	G	7	3318	34	12778	18	6890	16	7729	43	8921	17	6674
chr8	120115815	C	1	704	1	457	1	514	1	941	1	497	4	931
chr8	120115816	C	4	694	0	452	0	512	1	939	1	497	2	933
chr8	120115819	C	2	693	0	441	0	511	1	938	2	494	2	932
chr8	120115820	C	5	690	1	435	0	511	1	938	0	496	2	932
chr8	120115821	C	1	693	4	410	8	503	1	938	3	493	6	928
chr8	120115823	G	0	3315	18	12790	15	6890	11	7724	24	8927	6	6676
chr8	120115824	G	28	3287	17	12787	34	6868	15	7717	33	8914	18	6662
chr8	120115825	C	0	666	0	364	0	511	0	916	0	496	0	927
chr8	120115829	G	2	3309	1	12801	1	6883	0	7728	1	8912	0	6665
chr8	120115831	G	0	3258	3	12796	0	6736	0	7690	1	8800	0	6555
chr8	120115832	G	0	3260	3	12794	0	6736	2	7686	1	8810	1	6555
chr8	120115834	C	1	570	0	263	0	454	0	712	0	446	0	773
chr8	120115837	C	0	364	0	159	0	274	0	373	0	265	0	449
chr8	120115839	C	0	248	0	113	0	163	0	226	0	150	0	283
chr8	120115843	C	0	246	0	112	0	160	0	224	0	147	0	281
chr8	120115844	C	0	246	0	111	0	160	0	222	0	147	0	281
chr8	120115846	G	1	3222	3	12662	3	6684	1	7623	2	8732	2	6482
chr9	100498295	G	2	8800	3	9435	0	9188	1	4264	1	12009	0	5049

3	732	4	469	13	541	4	960	8	550	45	965
4	3340	22	12832	11	6927	18	7768	16	9006	11	6717
9	3337	29	12838	17	6921	13	7760	13	9004	43	6695
521	724	348	463	403	540	762	953	401	549	669	955
2677	3333	9516	12822	5442	6908	5698	7753	7163	8993	5111	6700
10	3337	128	12837	21	6918	11	7760	26	8995	9	6706
1	590	1	387	1	440	0	729	2	453	2	776
1	664	4	433	4	497	8	842	2	501	2	864
47	717	15	467	42	537	16	950	39	541	62	947
25	717	11	467	22	537	24	951	15	542	41	946
29	712	13	467	28	519	17	949	23	515	55	941
66	709	101	463	35	518	17	950	25	511	28	938
24	711	13	466	7	519	93	950	9	512	53	938
60	707	44	464	84	516	211	943	65	509	81	937
6	710	6	466	2	520	7	949	6	507	12	937
3	709	0	466	1	519	0	947	1	506	3	939
578	696	395	455	462	508	735	930	438	494	751	927
2464	3328	9471	12799	5129	6905	5483	7743	6468	8968	4722	6688
597	703	387	461	486	518	819	939	466	494	824	931
2451	2859	9152	10938	5074	6008	5426	6619	6593	7697	4965	5662
4	3330	19	12816	17	6913	6	7752	23	8972	4	6695
45	3331	23	12817	90	6916	16	7750	77	8971	88	6694
146	3328	763	12813	370	6915	505	7749	356	8967	287	6694
66	3328	198	12806	107	6911	198	7745	207	8967	208	6691
1	708	1	463	1	517	2	945	6	500	4	937
94	707	26	463	86	515	216	940	79	495	112	936
12	3325	19	12800	12	6907	12	7740	19	8956	7	6685
7	3325	34	12812	18	6908	16	7745	43	8964	17	6691
1	705	1	458	1	515	1	942	1	498	4	935
4	698	0	452	0	512	1	940	1	498	2	935
2	695	0	441	0	511	1	939	2	496	2	934
5	695	1	436	0	511	1	939	0	496	2	934
1	694	4	414	8	511	1	939	3	496	6	934
0	3315	18	12808	15	6905	11	7735	24	8951	6	6682
28	3315	17	12804	34	6902	15	7732	33	8947	18	6680
0	666	0	364	0	511	0	916	0	496	0	927
2	3311	1	12802	1	6884	0	7728	1	8913	0	6665
0	3258	3	12799	0	6736	0	7690	1	8801	0	6555
0	3260	3	12797	0	6736	2	7688	1	8811	1	6556
1	571	0	263	0	454	0	712	0	446	0	773
0	364	0	159	0	274	0	373	0	265	0	449
0	248	0	113	0	163	0	226	0	150	0	283
0	246	0	112	0	160	0	224	0	147	0	281
0	246	0	111	0	160	0	222	0	147	0	281
1	3223	3	12665	3	6687	1	7624	2	8734	2	6484
2	8802	3	9438	0	9188	1	4265	1	12010	0	5049

chr9	100498302	G	3	9033	1	9694	0	9320	0	4350	0	12201	1	5166
chr9	100498306	G	1	9084	0	9736	0	9327	1	4355	1	12225	1	5177
chr9	100498307	G	2	9087	2	9737	1	9327	3	4353	4	12222	1	5176
chr9	100498311	G	3	9125	0	9773	0	9339	1	4365	2	12241	0	5181
chr9	100498324	G	19	9151	3	9850	42	9303	39	4342	48	12222	2	5193
chr9	100498333	G	29	9149	30	9844	54	9315	19	4370	190	12233	28	5184
chr9	100498334	G	17	9159	35	9843	50	9322	19	4373	168	12257	30	5183
chr9	100498338	G	60	9094	191	9672	60	9298	125	4258	153	12245	66	5135
chr9	100498340	G	36	9144	155	9717	43	9329	18	4374	187	12240	17	5195
chr9	100498341	G	28	9154	20	9865	29	9346	11	4382	31	12395	20	5190
chr9	100498351	G	56	9138	13	9888	42	9335	24	4371	56	12379	13	5200
chr9	100498353	G	34	9158	70	9835	42	9337	4	4391	37	12396	37	5175
chr9	100498354	G	30	9166	31	9872	13	9364	10	4386	42	12391	24	5191
chr9	100498355	G	24	9172	17	9892	19	9361	10	4385	30	12406	8	5208
chr9	100498357	G	155	9039	76	9834	154	9226	3	4393	215	12224	192	5025
chr9	100498358	G	26	9177	22	9890	19	9362	7	4393	27	12413	13	5202
chr9	100498367	G	5428	3849	5123	4856	5777	3622	2352	2144	7791	4720	2916	2430
chr9	100498368	G	20	9256	23	9969	44	9365	14	4489	35	12493	28	5320
chr9	100498370	G	66	9232	160	9848	121	9289	66	4435	118	12406	75	5273
chr9	100498375	C	2	3605	0	5207	0	1737	0	4275	2	2449	1	6480
chr9	100498377	G	16	9313	18	10011	34	9388	3	4520	23	12526	35	5334
chr9	100498379	C	0	3680	0	5304	0	1757	0	4352	1	2489	0	6601
chr9	100498381	G	88	9225	73	9926	176	9237	29	4487	263	12250	149	5224
chr9	100498382	G	23	9314	15	10023	20	9417	4	4543	22	12544	10	5393
chr9	100498383	G	18	9321	16	10023	15	9422	37	4514	20	12554	7	5400
chr9	100498385	G	44	9295	25	10010	46	9393	10	4537	74	12493	13	5380
chr9	100498387	C	0	3744	0	5426	0	1777	1	4461	0	2513	2	6750
chr9	100498389	C	0	3740	2	5410	1	1775	0	4459	0	2514	0	6754
chr9	100498390	C	1	3744	1	5430	0	1777	0	4466	1	2514	1	6756
chr9	100498392	C	1	3746	0	5432	0	1776	0	4467	0	2516	3	6756
chr9	100498394	G	42	9326	7	10057	22	9427	2	4565	43	12545	59	5355
chr9	100498396	G	41	9332	4	10067	16	9433	5	4565	28	12564	4	5415
chr9	100498398	C	2	3744	0	5436	1	1777	0	4469	0	2518	3	6760
chr9	100498401	G	34	9341	9	10074	53	9427	7	4609	52	12600	10	5496
chr9	100498403	G	138	9236	18	10066	60	9413	49	4566	185	12464	30	5470
chr9	100498406	G	62	9320	20	10078	40	9441	4	4615	45	12618	17	5500
chr9	100498407	C	1	3758	8	5439	2	1783	7	4470	5	2536	10	6767
chr9	100498408	C	12	3750	5	5448	3	1783	4	4475	6	2532	5	6783
chr9	100498411	C	15	3747	10	5445	4	1780	8	4472	6	2536	15	6774
chr9	100498414	C	6	3754	4	5449	4	1779	8	4470	5	2521	11	6774
chr9	100498415	C	2853	909	4233	1225	1383	400	2820	1661	1981	553	4822	1968
chr9	100498416	G	6950	2445	7110	2998	7386	2127	3444	1199	9821	2894	4122	1439
chr9	100498421	C	3078	689	4189	1274	1384	404	3267	1222	2069	485	5069	1734
chr9	100498422	G	7278	2116	7257	2848	7526	1990	3613	1028	10168	2545	4102	1455
chr9	100498423	C	14	3755	12	5449	5	1783	9	4482	9	2546	10	6789
chr9	100498426	G	65	9222	96	9930	130	9341	3	4667	91	12631	23	5602

3	9036	1	9695	0	9320	0	4350	0	12201	1	5167
1	9085	0	9736	0	9327	1	4356	1	12226	1	5178
2	9089	2	9739	1	9328	3	4356	4	12226	1	5177
3	9128	0	9773	0	9339	1	4366	2	12243	0	5181
19	9170	3	9853	42	9345	39	4381	48	12270	2	5195
29	9178	30	9874	54	9369	19	4389	190	12423	28	5212
17	9176	35	9878	50	9372	19	4392	168	12425	30	5213
60	9154	191	9863	60	9358	125	4383	153	12398	66	5201
36	9180	155	9872	43	9372	18	4392	187	12427	17	5212
28	9182	20	9885	29	9375	11	4393	31	12426	20	5210
56	9194	13	9901	42	9377	24	4395	56	12435	13	5213
34	9192	70	9905	42	9379	4	4395	37	12433	37	5212
30	9196	31	9903	13	9377	10	4396	42	12433	24	5215
24	9196	17	9909	19	9380	10	4395	30	12436	8	5216
155	9194	76	9910	154	9380	3	4396	215	12439	192	5217
26	9203	22	9912	19	9381	7	4400	27	12440	13	5215
5428	9277	5123	9979	5777	9399	2352	4496	7791	12511	2916	5346
20	9276	23	9992	44	9409	14	4503	35	12528	28	5348
66	9298	160	10008	121	9410	66	4501	118	12524	75	5348
2	3607	0	5207	0	1737	0	4275	2	2451	1	6481
16	9329	18	10029	34	9422	3	4523	23	12549	35	5369
0	3680	0	5304	0	1757	0	4352	1	2490	0	6601
88	9313	73	9999	176	9413	29	4516	263	12513	149	5373
23	9337	15	10038	20	9437	4	4547	22	12566	10	5403
18	9339	16	10039	15	9437	37	4551	20	12574	7	5407
44	9339	25	10035	46	9439	10	4547	74	12567	13	5393
0	3744	0	5426	0	1777	1	4462	0	2513	2	6752
0	3740	2	5412	1	1776	0	4459	0	2514	0	6754
1	3745	1	5431	0	1777	0	4466	1	2515	1	6757
1	3747	0	5432	0	1776	0	4467	0	2516	3	6759
42	9368	7	10064	22	9449	2	4567	43	12588	59	5414
41	9373	4	10071	16	9449	5	4570	28	12592	4	5419
2	3746	0	5436	1	1778	0	4469	0	2518	3	6763
34	9375	9	10083	53	9480	7	4616	52	12652	10	5506
138	9374	18	10084	60	9473	49	4615	185	12649	30	5500
62	9382	20	10098	40	9481	4	4619	45	12663	17	5517
1	3759	8	5447	2	1785	7	4477	5	2541	10	6777
12	3762	5	5453	3	1786	4	4479	6	2538	5	6788
15	3762	10	5455	4	1784	8	4480	6	2542	15	6789
6	3760	4	5453	4	1783	8	4478	5	2526	11	6785
2853	3762	4233	5458	1383	1783	2820	4481	1981	2534	4822	6790
6950	9395	7110	10108	7386	9513	3444	4643	9821	12715	4122	5561
3078	3767	4189	5463	1384	1788	3267	4489	2069	2554	5069	6803
7278	9394	7257	10105	7526	9516	3613	4641	10168	12713	4102	5557
14	3769	12	5461	5	1788	9	4491	9	2555	10	6799
65	9287	96	10026	130	9471	3	4670	91	12722	23	5625

chr9	100498427	G	34	9370	26	10097	23	9553	4	4713	45	12792	28	5685
chr9	100498428	G	25	9382	65	10059	33	9543	52	4666	39	12801	18	5696
chr9	100498430	G	95	9309	19	10104	89	9487	100	4615	162	12680	7	5708
chr9	100498431	G	28	9377	22	10104	24	9552	12	4705	49	12791	17	5696
chr9	100498433	C	11	3767	1	5474	4	1789	5	4501	9	2552	56	6760
chr9	100498437	G	24	9379	12	10109	49	9522	30	4688	20	12818	9	5700
chr9	100498439	G	53	9354	89	10036	60	9513	8	4709	106	12737	30	5683
chr9	100498441	C	2	3775	6	5467	5	1789	9	4498	5	2557	9	6808
chr9	100498442	C	24	3755	5	5472	12	1782	5	4505	12	2551	65	6755
chr9	100498444	C	11	3770	9	5470	9	1785	8	4503	7	2556	87	6733
chr9	100498446	G	50	9354	89	10032	68	9501	20	4694	83	12741	28	5676
chr9	100498447	G	10	9396	12	10111	13	9558	4	4711	17	12804	11	5694
chr9	100498450	C	2505	1279	3896	1584	1204	593	3003	1513	1745	819	4335	2495
chr9	100498451	G	6349	3056	5733	4385	6807	2765	2991	1717	9063	3749	3526	2179
chr9	100498453	G	18	9380	66	10043	21	9547	54	4649	21	12770	25	5676
chr9	100498455	G	25	9380	15	10099	44	9527	9	4701	50	12753	16	5688
chr9	100498457	G	24	9377	17	10095	17	9512	24	4663	37	12739	7	5656
chr9	100498458	C	9	3774	9	5470	4	1796	4	4515	4	2568	16	6820
chr9	100498460	G	190	9206	340	9770	234	9293	161	4523	328	12450	13	5649
chr9	100498462	G	21	9377	9	10103	33	9494	7	4676	25	12753	28	5630
chr9	100498464	C	50	3729	9	5471	24	1776	7	4508	20	2560	68	6762
chr9	100498466	C	5	3777	5	5475	6	1796	18	4498	7	2570	12	6826
chr9	100498467	C	16	3767	8	5474	7	1797	12	4509	11	2570	14	6826
chr9	100498469	G	246	9140	174	9929	173	9330	152	4509	248	12494	165	5468
chr9	100498470	C	33	3753	239	5247	28	1776	6	4517	27	2561	68	6774
chr9	100498472	G	50	9345	21	10090	19	9501	10	4670	27	12734	44	5604
chr9	100498473	G	19	9376	22	10086	23	9495	15	4663	27	12731	5	5639
chr9	100498474	C	12	3769	15	5464	5	1830	7	4509	6	2628	6	6838
chr9	100498477	G	34	9342	44	10049	16	9470	10	4650	53	12681	12	5621
chr9	100498478	C	11	3776	15	5473	11	1832	11	4514	1	2630	16	6844
chr9	100498479	C	10	3780	8	5484	4	1840	6	4520	3	2637	11	6852
chr9	100498482	G	53	9290	46	9994	89	9364	5	4642	87	12584	36	5579
chr9	100498483	G	18	9370	10	10087	15	9485	13	4654	17	12724	12	5621
chr9	100498485	G	33	9354	10	10088	43	9459	27	4641	55	12686	33	5601
chr9	100498486	G	13	9375	66	10033	15	9483	7	4660	19	12721	17	5617
chr9	100498487	G	53	9335	48	10048	35	9467	43	4623	29	12711	28	5603
chr9	100498490	C	2968	829	4498	996	1453	402	3284	1247	2098	560	5125	1747
chr9	100498491	G	7445	1944	6943	3155	7908	1592	3235	1431	10429	2309	4284	1347
chr9	100498493	C	11	3786	12	5487	5	1849	15	4517	6	2652	10	6861
chr9	100498496	G	75	9290	110	9962	103	9378	10	4644	135	12575	4	5615
chr9	100498497	C	21	3776	12	5486	10	1843	8	4525	14	2649	18	6853
chr9	100498499	G	70	9310	46	10048	67	9429	21	4641	117	12611	100	5528
chr9	100498501	C	4	3795	8	5494	1	1855	8	4526	5	2661	9	6865
chr9	100498502	C	9	3787	18	5482	7	1850	7	4528	10	2657	17	6856
chr9	100498503	C	10	3789	9	5492	4	1852	8	4526	4	2666	12	6862
chr9	100498505	C	2540	1260	3718	1785	1284	573	3053	1482	1817	854	4419	2458

34	9404	26	10123	23	9576	4	4717	45	12837	28	5713
25	9407	65	10124	33	9576	52	4718	39	12840	18	5714
95	9404	19	10123	89	9576	100	4715	162	12842	7	5715
28	9405	22	10126	24	9576	12	4717	49	12840	17	5713
11	3778	1	5475	4	1793	5	4506	9	2561	56	6816
24	9403	12	10121	49	9571	30	4718	20	12838	9	5709
53	9407	89	10125	60	9573	8	4717	106	12843	30	5713
2	3777	6	5473	5	1794	9	4507	5	2562	9	6817
24	3779	5	5477	12	1794	5	4510	12	2563	65	6820
11	3781	9	5479	9	1794	8	4511	7	2563	87	6820
50	9404	89	10121	68	9569	20	4714	83	12824	28	5704
10	9406	12	10123	13	9571	4	4715	17	12821	11	5705
2505	3784	3896	5480	1204	1797	3003	4516	1745	2564	4335	6830
6349	9405	5733	10118	6807	9572	2991	4708	9063	12812	3526	5705
18	9398	66	10109	21	9568	54	4703	21	12791	25	5701
25	9405	15	10114	44	9571	9	4710	50	12803	16	5704
24	9401	17	10112	17	9529	24	4687	37	12776	7	5663
9	3783	9	5479	4	1800	4	4519	4	2572	16	6836
190	9396	340	10110	234	9527	161	4684	328	12778	13	5662
21	9398	9	10112	33	9527	7	4683	25	12778	28	5658
50	3779	9	5480	24	1800	7	4515	20	2580	68	6830
5	3782	5	5480	6	1802	18	4516	7	2577	12	6838
16	3783	8	5482	7	1804	12	4521	11	2581	14	6840
246	9386	174	10103	173	9503	152	4661	248	12742	165	5633
33	3786	239	5486	28	1804	6	4523	27	2588	68	6842
50	9395	21	10111	19	9520	10	4680	27	12761	44	5648
19	9395	22	10108	23	9518	15	4678	27	12758	5	5644
12	3781	15	5479	5	1835	7	4516	6	2634	6	6844
34	9376	44	10093	16	9486	10	4660	53	12734	12	5633
11	3787	15	5488	11	1843	11	4525	1	2631	16	6860
10	3790	8	5492	4	1844	6	4526	3	2640	11	6863
53	9343	46	10040	89	9453	5	4647	87	12671	36	5615
18	9388	10	10097	15	9500	13	4667	17	12741	12	5633
33	9387	10	10098	43	9502	27	4668	55	12741	33	5634
13	9388	66	10099	15	9498	7	4667	19	12740	17	5634
53	9388	48	10096	35	9502	43	4666	29	12740	28	5631
2968	3797	4498	5494	1453	1855	3284	4531	2098	2658	5125	6872
7445	9389	6943	10098	7908	9500	3235	4666	10429	12738	4284	5631
11	3797	12	5499	5	1854	15	4532	6	2658	10	6871
75	9365	110	10072	103	9481	10	4654	135	12710	4	5619
21	3797	12	5498	10	1853	8	4533	14	2663	18	6871
70	9380	46	10094	67	9496	21	4662	117	12728	100	5628
4	3799	8	5502	1	1856	8	4534	5	2666	9	6874
9	3796	18	5500	7	1857	7	4535	10	2667	17	6873
10	3799	9	5501	4	1856	8	4534	4	2670	12	6874
2540	3800	3718	5503	1284	1857	3053	4535	1817	2671	4419	6877

chr9	100498506	G	6811	2562	6367	3718	7116	2372	3066	1595	9700	3019	4015	1608
chr9	100498508	C	4	3802	14	5491	4	1871	8	4529	6	2687	7	6874
chr9	100498510	G	33	9326	15	10060	42	9436	5	4651	41	12667	25	5591
chr9	100498511	C	35	3745	9	5437	12	1851	99	4384	26	2652	13	6816
chr9	100498514	C	39	3767	128	5380	29	1847	8	4529	49	2652	129	6748
chr9	100498516	C	1	3806	6	5502	0	1881	7	4530	3	2700	9	6874
chr9	100498517	C	143	3666	188	5322	60	1822	4	4535	97	2612	339	6544
chr9	100498519	C	20	3790	6	5502	19	1865	58	4481	20	2689	75	6808
chr9	100498521	G	12	9347	18	10035	12	9461	7	4645	27	12669	6	5601
chr9	100498522	C	19	3791	129	5383	6	1878	5	4532	9	2704	11	6874
chr9	100498525	C	4	735	2	1177	4	435	21	928	3	841	15	1593
chr9	100498528	G	21	9316	19	10017	21	9440	34	4610	27	12634	4	5594
chr9	100498529	G	26	9312	17	10011	20	9408	7	4615	32	12596	9	5550
chr9	100498531	G	186	9149	100	9933	247	9182	4	4620	230	12402	135	5423
chr9	100498533	G	192	9136	127	9900	190	9217	27	4587	236	12387	134	5422
chr9	100498536	G	16	9312	12	10012	24	9383	29	4586	9	12610	5	5547
chr9	100498539	C	0	157	1	193	1	201	1	156	2	441	0	306
chr9	100498541	G	43	9273	79	9938	30	9371	40	4566	34	12576	42	5502
chr9	100498545	C	0	158	0	188	0	202	0	156	2	443	2	301
chr9	100498546	C	0	159	0	189	0	201	1	158	1	444	0	303
chr9	100498547	C	0	167	0	201	1	203	1	160	1	435	0	300
chr9	100498548	C	114	51	155	47	142	54	73	88	294	133	208	89
chr9	100498549	G	5953	3328	5589	4367	7327	2067	3101	1499	9686	2902	3850	1683
chr9	100498551	G	0	9206	3	9782	0	9379	0	4590	1	12567	1	5530
chr9	100498553	G	0	9203	1	9781	1	9379	0	4590	1	12569	0	5532
chr9	100498557	C	0	175	1	211	1	256	0	188	1	489	0	344
chr9	100498558	C	2	183	2	229	3	258	0	206	3	491	1	373
chr9	100498560	G	4	9158	3	9721	2	9346	0	4574	6	12523	4	5512
chr9	100498561	C	0	294	0	376	2	284	2	344	1	527	1	559
chr9	100498562	C	0	300	8	383	2	287	0	370	0	542	0	591
chr9	100498563	C	1	308	1	401	3	291	1	378	2	543	2	606
chr9	100498565	G	2	8776	5	9322	2	9263	1	4494	4	12342	1	5404
chr9	100498566	G	3	8753	5	9279	1	9263	1	4488	4	12341	1	5398
chr9	100498569	G	0	8596	0	9087	0	9220	0	4454	1	12252	0	5345
chr9	100498572	C	5	1142	1	1672	7	620	4	1410	2	871	6	2125
chr9	100498574	G	2	6751	4	7039	4	7180	1	3508	2	9066	3	4138
chr9	100498575	C	7	3837	13	5501	9	1955	8	4534	10	2808	16	6889
chr9	100498576	C	3129	717	4517	999	1580	385	3419	1125	2284	535	5318	1586
chr9	100498581	C	10	3827	5	5510	0	1964	3	4540	5	2814	11	6891
chr9	100498583	C	25	3817	6	5509	20	1942	3	4540	15	2804	230	6676
chr9	100498588	C	6	3831	2	5507	5	1950	5	4534	4	2784	11	6888
chr9	100498589	C	2765	1072	4393	1115	1429	524	2700	1841	2000	788	4905	1988
chr9	100498594	C	26	3810	6	5498	14	1941	5	4536	9	2776	5	6891
chr9	100498602	C	10	3825	6	5496	6	1944	4	4536	5	2774	12	6880
chr9	100498606	C	13	3821	11	5488	10	1941	120	4417	11	2760	10	6878
chr9	100498610	C	22	3812	5	5491	4	1929	2	4533	8	2727	131	6740

6811	9373	6367	10085	7116	9488	3066	4661	9700	12719	4015	5623
4	3806	14	5505	4	1875	8	4537	6	2693	7	6881
33	9359	15	10075	42	9478	5	4656	41	12708	25	5616
35	3780	9	5446	12	1863	99	4483	26	2678	13	6829
39	3806	128	5508	29	1876	8	4537	49	2701	129	6877
1	3807	6	5508	0	1881	7	4537	3	2703	9	6883
143	3809	188	5510	60	1882	4	4539	97	2709	339	6883
20	3810	6	5508	19	1884	58	4539	20	2709	75	6883
12	9359	18	10053	12	9473	7	4652	27	12696	6	5607
19	3810	129	5512	6	1884	5	4537	9	2713	11	6885
4	739	2	1179	4	439	21	949	3	844	15	1608
21	9337	19	10036	21	9461	34	4644	27	12661	4	5598
26	9338	17	10028	20	9428	7	4622	32	12628	9	5559
186	9335	100	10033	247	9429	4	4624	230	12632	135	5558
192	9328	127	10027	190	9407	27	4614	236	12623	134	5556
16	9328	12	10024	24	9407	29	4615	9	12619	5	5552
0	157	1	194	1	202	1	157	2	443	0	306
43	9316	79	10017	30	9401	40	4606	34	12610	42	5544
0	158	0	188	0	202	0	156	2	445	2	303
0	159	0	189	0	201	1	159	1	445	0	303
0	167	0	201	1	204	1	161	1	436	0	300
114	165	155	202	142	196	73	161	294	427	208	297
5953	9281	5589	9956	7327	9394	3101	4600	9686	12588	3850	5533
0	9206	3	9785	0	9379	0	4590	1	12568	1	5531
0	9203	1	9782	1	9380	0	4590	1	12570	0	5532
0	175	1	212	1	257	0	188	1	490	0	344
2	185	2	231	3	261	0	206	3	494	1	374
4	9162	3	9724	2	9348	0	4574	6	12529	4	5516
0	294	0	376	2	286	2	346	1	528	1	560
0	300	8	391	2	289	0	370	0	542	0	591
1	309	1	402	3	294	1	379	2	545	2	608
2	8778	5	9327	2	9265	1	4495	4	12346	1	5405
3	8756	5	9284	1	9264	1	4489	4	12345	1	5399
0	8596	0	9087	0	9220	0	4454	1	12253	0	5345
5	1147	1	1673	7	627	4	1414	2	873	6	2131
2	6753	4	7043	4	7184	1	3509	2	9068	3	4141
7	3844	13	5514	9	1964	8	4542	10	2818	16	6905
3129	3846	4517	5516	1580	1965	3419	4544	2284	2819	5318	6904
10	3837	5	5515	0	1964	3	4543	5	2819	11	6902
25	3842	6	5515	20	1962	3	4543	15	2819	230	6906
6	3837	2	5509	5	1955	5	4539	4	2788	11	6899
2765	3837	4393	5508	1429	1953	2700	4541	2000	2788	4905	6893
26	3836	6	5504	14	1955	5	4541	9	2785	5	6896
10	3835	6	5502	6	1950	4	4540	5	2779	12	6892
13	3834	11	5499	10	1951	120	4537	11	2771	10	6888
22	3834	5	5496	4	1933	2	4535	8	2735	131	6871

chr9	100498617	C	3	3827	4	5496	2	1931	3	4528	3	2726	8	6860
chr9	100498624	C	7	3822	6	5487	7	1922	10	4520	9	2711	13	6846
chr9	100498626	C	6	3823	15	5479	2	1926	8	4520	7	2712	7	6854
chr9	100498638	C	15	3803	7	5474	6	1919	15	4505	5	2706	70	6780
chr9	100498639	C	18	3804	5	5477	6	1920	37	4486	17	2693	9	6842
chr9	100498649	C	6	3676	6	5195	3	1842	8	4343	2	2628	11	6578
chr9	100498650	C	3	3805	20	5445	9	1911	7	4507	3	2700	53	6774
chr9	100498654	C	6	3802	15	5448	5	1913	13	4498	10	2689	17	6807
chr9	100498655	C	7	3801	9	5456	3	1915	6	4506	9	2692	11	6812
chr9	100498660	c	8	3799	11	5453	4	1910	9	4501	6	2688	12	6810
chr9	100498672	c	6	3792	11	5433	6	1905	7	4489	16	2671	22	6767
chr9	100498674	c	7	3792	4	5446	3	1907	0	4493	14	2673	39	6754
chr9	100498676	c	3061	734	4290	1145	1519	387	3591	896	2138	547	4963	1809
chr9	100498678	c	5	3792	7	5437	2	1906	5	4481	10	2675	9	6775
chr9	100498679	c	7	3788	6	5434	2	1908	6	4483	11	2672	11	6772
chr9	100498686	c	10	3785	11	5425	1	1909	6	4484	5	2675	10	6766
chr9	100498687	c	4	3790	84	5347	6	1902	107	4379	1	2679	5	6773
chr9	100498688	c	53	3743	43	5395	21	1889	112	4378	28	2652	80	6698
chr9	100498691	c	3343	452	4647	789	1698	211	3697	789	2427	252	5383	1390
chr9	100498693	c	8	3773	9	5403	4	1903	4	4466	10	2658	15	6741
chr9	100498694	c	9	3786	10	5423	7	1903	12	4473	5	2670	17	6757
chr9	100498697	c	2	3791	2	5425	0	1908	0	4484	1	2673	1	6769
chr9	100498703	c	0	3783	1	5411	0	1906	0	4474	0	2673	1	6749
chr9	100498709	c	6	3761	8	5358	8	1890	10	4419	2	2659	15	6678
chr9	100498718	c	1	3092	1	4410	0	1638	1	3596	2	2408	1	5452
chr9	100498720	c	0	2970	1	4223	0	1587	0	3449	0	2341	2	5241
chrX	7476281	G	0	1630	0	2246	2	2939	0	1551	5	8115	1	6419
chrX	7476282	G	0	1630	0	2246	1	2940	0	1551	0	8122	2	6424
chrX	7476287	G	2	1630	3	2243	0	2939	1	1550	5	8120	6	6426
chrX	7476289	G	0	1632	0	2247	0	2941	0	1551	0	8126	2	6432
chrX	7476302	G	1	1632	3	2245	5	2930	2	1551	37	8084	10	6434
chrX	7476305	G	0	1634	3	2247	7	2937	1	1557	13	8127	15	6438
chrX	7476306	G	0	1634	6	2242	11	2930	5	1553	9	8129	18	6432
chrX	7476307	G	8	1626	2	2248	15	2928	4	1553	26	8114	12	6445
chrX	7476313	G	0	1634	2	2249	6	2939	1	1557	9	8138	4	6457
chrX	7476314	G	0	1633	5	2247	6	2939	0	1556	9	8136	9	6450
chrX	7476321	G	3	1630	6	2245	14	2933	7	1551	23	8122	15	6447
chrX	7476329	G	2	1634	4	2250	2	2949	1	1557	38	8109	7	6458
chrX	7476330	G	0	1636	7	2248	5	2945	3	1554	24	8125	9	6456
chrX	7476337	G	5	1633	3	2253	5	2946	2	1555	47	8106	13	6453
chrX	7476348	G	1	1636	0	2255	2	2949	0	1559	42	8110	28	6443
chrX	7476349	G	1	1637	2	2254	9	2940	3	1556	14	8140	17	6449
chrX	7476351	G	5	1631	3	2253	5	2945	1	1558	11	8144	12	6456
chrX	7476361	G	1	1636	2	2258	1	2950	1	1559	7	8153	7	6466
chrX	7476362	G	2	1635	2	2261	2	2948	0	1560	11	8150	18	6450
chrX	7476365	G	18	1619	6	2257	2	2950	1	1557	21	8140	5	6471

3	3830	4	5500	2	1933	3	4531	3	2729	8	6868
7	3829	6	5493	7	1929	10	4530	9	2720	13	6859
6	3829	15	5494	2	1928	8	4528	7	2719	7	6861
15	3818	7	5481	6	1925	15	4520	5	2711	70	6850
18	3822	5	5482	6	1926	37	4523	17	2710	9	6851
6	3682	6	5201	3	1845	8	4351	2	2630	11	6589
3	3808	20	5465	9	1920	7	4514	3	2703	53	6827
6	3808	15	5463	5	1918	13	4511	10	2699	17	6824
7	3808	9	5465	3	1918	6	4512	9	2701	11	6823
8	3807	11	5464	4	1914	9	4510	6	2694	12	6822
6	3798	11	5444	6	1911	7	4496	16	2687	22	6789
7	3799	4	5450	3	1910	0	4493	14	2687	39	6793
3061	3795	4290	5435	1519	1906	3591	4487	2138	2685	4963	6772
5	3797	7	5444	2	1908	5	4486	10	2685	9	6784
7	3795	6	5440	2	1910	6	4489	11	2683	11	6783
10	3795	11	5436	1	1910	6	4490	5	2680	10	6776
4	3794	84	5431	6	1908	107	4486	1	2680	5	6778
53	3796	43	5438	21	1910	112	4490	28	2680	80	6778
3343	3795	4647	5436	1698	1909	3697	4486	2427	2679	5383	6773
8	3781	9	5412	4	1907	4	4470	10	2668	15	6756
9	3795	10	5433	7	1910	12	4485	5	2675	17	6774
2	3793	2	5427	0	1908	0	4484	1	2674	1	6770
0	3783	1	5412	0	1906	0	4474	0	2673	1	6750
6	3767	8	5366	8	1898	10	4429	2	2661	15	6693
1	3093	1	4411	0	1638	1	3597	2	2410	1	5453
0	2970	1	4224	0	1587	0	3449	0	2341	2	5243
0	1630	0	2246	2	2941	0	1551	5	8120	1	6420
0	1630	0	2246	1	2941	0	1551	0	8122	2	6426
2	1632	3	2246	0	2939	1	1551	5	8125	6	6432
0	1632	0	2247	0	2941	0	1551	0	8126	2	6434
1	1633	3	2248	5	2935	2	1553	37	8121	10	6444
0	1634	3	2250	7	2944	1	1558	13	8140	15	6453
0	1634	6	2248	11	2941	5	1558	9	8138	18	6450
8	1634	2	2250	15	2943	4	1557	26	8140	12	6457
0	1634	2	2251	6	2945	1	1558	9	8147	4	6461
0	1633	5	2252	6	2945	0	1556	9	8145	9	6459
3	1633	6	2251	14	2947	7	1558	23	8145	15	6462
2	1636	4	2254	2	2951	1	1558	38	8147	7	6465
0	1636	7	2255	5	2950	3	1557	24	8149	9	6465
5	1638	3	2256	5	2951	2	1557	47	8153	13	6466
1	1637	0	2255	2	2951	0	1559	42	8152	28	6471
1	1638	2	2256	9	2949	3	1559	14	8154	17	6466
5	1636	3	2256	5	2950	1	1559	11	8155	12	6468
1	1637	2	2260	1	2951	1	1560	7	8160	7	6473
2	1637	2	2263	2	2950	0	1560	11	8161	18	6468
18	1637	6	2263	2	2952	1	1558	21	8161	5	6476

chrX	7476371	G	16	1619	1	2262	5	2948	2	1557	44	8118	14	6463
chrX	7476374	G	19	1618	5	2258	9	2943	6	1554	40	8125	8	6471
chrX	7476382	G	1	1636	1	2262	1	2952	2	1558	6	8159	7	6471
chrX	7476383	G	12	1625	3	2261	4	2950	1	1559	13	8152	21	6456
chrX	7476387	G	32	1606	8	2264	2	2954	11	1551	59	8113	11	6464
chrX	7476388	G	0	1638	2	2269	12	2943	2	1559	14	8158	10	6468
chrX	7476391	G	1	1637	6	2267	3	2953	1	1561	7	8167	4	6473
chrX	7476393	G	8	1630	1	2273	2	2952	0	1562	10	8159	6	6472
chrX	7476394	G	3	1635	2	2272	10	2946	0	1562	12	8158	9	6470
chrX	7476402	G	4	1636	1	2274	0	2957	2	1560	19	8160	6	6475
chrX	7476403	G	4	1636	5	2269	6	2953	3	1558	12	8166	17	6460
chrX	7476407	G	1	1639	4	2272	5	2952	3	1559	9	8166	13	6466
chrX	7476415	G	4	1639	4	2277	6	2952	1	1560	36	8158	9	6477
chrX	7476417	G	3	1640	7	2277	3	2953	2	1559	18	8169	9	6473
chrX	7476419	G	3	1637	3	2283	8	2952	3	1559	55	8139	18	6465
chrX	7476421	G	5	1637	6	2278	5	2955	2	1559	15	8172	9	6476
chrX	7476424	G	1	71	0	121	0	133	0	60	3	379	0	254
chrX	7476427	G	0	50	0	88	0	105	0	34	1	286	0	162
chrX	7476428	G	0	50	0	87	0	105	0	31	0	285	1	155
chrX	7476435	G	2	53	0	86	0	106	0	26	9	290	0	135
chrX	7476439	G	0	53	1	84	0	106	0	24	1	298	0	133
chrX	7476440	C	3	26966	7	18139	0	43	7	16780	10	57083	4	29244
chrX	7476442	G	0	53	0	84	0	105	0	24	0	303	0	131
chrX	7476444	G	0	53	0	81	0	107	0	24	1	305	1	126
chrX	7476447	G	0	44	0	78	0	90	0	23	0	283	0	116
chrX	7476450	C	2	27463	0	18494	0	44	1	17065	4	58041	0	29717
chrX	7476451	C	4	27467	1	18495	0	44	1	17075	11	58034	3	29710
chrX	7476456	G	0	43	0	80	0	86	0	15	0	269	0	100
chrX	7476459	C	3	27600	2	18620	0	45	0	17157	8	58218	3	30017
chrX	7476460	C	5	27601	2	18626	0	45	2	17161	7	58226	3	30030
chrX	7476461	C	1	27604	1	18623	0	45	5	17153	7	58228	9	30016
chrX	7476464	G	0	42	0	80	0	83	0	17	0	265	1	103
chrX	7476465	G	0	41	1	80	1	82	0	18	1	266	0	101
chrX	7476467	C	77	27639	41	18699	1	45	24	17202	128	58203	73	30079
chrX	7476470	C	263	27445	268	18473	0	46	155	17062	607	57699	693	29450
chrX	7476471	G	1	39	0	80	0	82	0	18	0	258	1	99
chrX	7476474	C	35	27706	22	18729	0	46	26	17215	145	58209	35	30138
chrX	7476476	G	0	41	0	82	0	80	0	18	1	264	1	96
chrX	7476477	C	78	27425	36	18517	0	46	53	17043	102	57720	564	29297
chrX	7476478	G	1	43	0	83	0	81	0	18	2	261	0	97
chrX	7476479	C	64	27679	110	18660	1	45	46	17206	121	58192	67	30126
chrX	7476480	C	48	27722	24	18764	0	46	23	17241	84	58306	51	30185
chrX	7476482	C	24	27728	19	18746	0	46	13	17242	37	58300	71	30135
chrX	7476484	G	0	44	0	83	0	86	0	18	0	270	1	98
chrX	7476486	G	0	42	0	82	0	83	0	17	0	265	0	99
chrX	7476487	C	51	27735	25	18771	0	46	33	17252	116	58287	50	30232

16	1635	1	2263	5	2953	2	1559	44	8162	14	6477
19	1637	5	2263	9	2952	6	1560	40	8165	8	6479
1	1637	1	2263	1	2953	2	1560	6	8165	7	6478
12	1637	3	2264	4	2954	1	1560	13	8165	21	6477
32	1638	8	2272	2	2956	11	1562	59	8172	11	6475
0	1638	2	2271	12	2955	2	1561	14	8172	10	6478
1	1638	6	2273	3	2956	1	1562	7	8174	4	6477
8	1638	1	2274	2	2954	0	1562	10	8169	6	6478
3	1638	2	2274	10	2956	0	1562	12	8170	9	6479
4	1640	1	2275	0	2957	2	1562	19	8179	6	6481
4	1640	5	2274	6	2959	3	1561	12	8178	17	6477
1	1640	4	2276	5	2957	3	1562	9	8175	13	6479
4	1643	4	2281	6	2958	1	1561	36	8194	9	6486
3	1643	7	2284	3	2956	2	1561	18	8187	9	6482
3	1640	3	2286	8	2960	3	1562	55	8194	18	6483
5	1642	6	2284	5	2960	2	1561	15	8187	9	6485
1	72	0	121	0	133	0	60	3	382	0	254
0	50	0	88	0	105	0	34	1	287	0	162
0	50	0	87	0	105	0	31	0	285	1	156
2	55	0	86	0	106	0	26	9	299	0	135
0	53	1	85	0	106	0	24	1	299	0	133
3	26969	7	18146	0	43	7	16787	10	57093	4	29248
0	53	0	84	0	105	0	24	0	303	0	131
0	53	0	81	0	107	0	24	1	306	1	127
0	44	0	78	0	90	0	23	0	283	0	116
2	27465	0	18494	0	44	1	17066	4	58045	0	29717
4	27471	1	18496	0	44	1	17076	11	58045	3	29713
0	43	0	80	0	86	0	15	0	269	0	100
3	27603	2	18622	0	45	0	17157	8	58226	3	30020
5	27606	2	18628	0	45	2	17163	7	58233	3	30033
1	27605	1	18624	0	45	5	17158	7	58235	9	30025
0	42	0	80	0	83	0	17	0	265	1	104
0	41	1	81	1	83	0	18	1	267	0	101
77	27716	41	18740	1	46	24	17226	128	58331	73	30152
263	27708	268	18741	0	46	155	17217	607	58306	693	30143
1	40	0	80	0	82	0	18	0	258	1	100
35	27741	22	18751	0	46	26	17241	145	58354	35	30173
0	41	0	82	0	80	0	18	1	265	1	97
78	27503	36	18553	0	46	53	17096	102	57822	564	29861
1	44	0	83	0	81	0	18	2	263	0	97
64	27743	110	18770	1	46	46	17252	121	58313	67	30193
48	27770	24	18788	0	46	23	17264	84	58390	51	30236
24	27752	19	18765	0	46	13	17255	37	58337	71	30206
0	44	0	83	0	86	0	18	0	270	1	99
0	42	0	82	0	83	0	17	0	265	0	99
51	27786	25	18796	0	46	33	17285	116	58403	50	30282

chrX	7476490	G	0	36	0	76	0	82	0	14	0	255	0	97
chrX	7476492	G	0	38	0	78	0	86	0	13	1	259	0	95
chrX	7476493	G	1	38	0	78	0	87	0	14	2	258	0	95
chrX	7476494	G	0	39	0	76	1	85	0	14	0	259	1	96
chrX	7476495	C	73	27671	270	18499	1	44	71	17178	286	57976	55	30205
chrX	7476496	G	0	38	0	75	0	77	0	13	0	245	0	93
chrX	7476500	C	168	27648	279	18555	0	46	32	17266	482	57958	394	29959
chrX	7476501	G	1	51	0	87	0	100	0	16	0	279	0	102
chrX	7476502	G	0	53	0	88	0	100	0	17	0	279	0	103
chrX	7476504	G	0	51	0	88	0	101	0	17	0	285	0	98
chrX	7476505	C	110	27698	30	18800	0	46	25	17272	229	58193	393	29964
chrX	7476506	G	0	39	1	78	0	84	0	17	4	222	0	77
chrX	7476507	G	0	54	1	90	0	104	0	18	2	286	0	101
chrX	7476508	G	0	55	1	90	1	104	0	18	1	290	0	101
chrX	7476510	G	0	47	1	80	0	97	0	14	0	271	1	96
chrX	7476511	G	0	48	0	83	1	84	0	16	1	262	0	93
chrX	7476512	G	0	46	0	85	0	82	0	15	0	262	1	90
chrX	7476513	G	0	51	0	83	0	85	0	15	1	271	0	92
chrX	7476514	G	0	50	0	84	0	86	0	15	0	273	0	92
chrX	7476516	C	120	27714	271	18567	1	45	201	17108	289	58172	381	30007
chrX	7476517	G	0	51	0	84	0	85	0	16	2	266	0	90
chrX	7476518	G	0	51	0	86	0	86	0	17	0	276	0	95
chrX	7476519	G	0	51	0	87	0	86	0	17	0	276	0	96
chrX	7476520	G	0	51	1	88	0	87	0	17	0	281	0	98
chrX	7476521	G	0	50	0	89	0	88	0	17	0	284	0	98
chrX	7476522	G	0	51	0	101	0	91	0	21	0	294	0	106
chrX	7476524	G	0	51	0	103	1	91	0	21	0	296	0	109
chrX	7476526	G	0	55	0	101	0	93	0	19	2	301	0	114
chrX	7476528	C	41	27801	48	18794	0	46	41	17258	100	58365	64	30351
chrX	7476530	C	203	27579	32	18776	0	46	32	17244	410	57953	40	30339
chrX	7476531	G	0	65	0	99	0	95	0	25	8	311	0	125
chrX	7476532	C	71	27763	42	18807	0	46	36	17273	143	58310	165	30265
chrX	7476533	C	52	27807	35	18821	0	46	30	17294	140	58348	56	30404
chrX	7476534	C	147	27701	288	18556	0	46	42	17274	413	58031	396	30049
chrX	7476535	G	0	63	0	107	0	98	0	26	9	314	0	131
chrX	7476536	C	93	27495	284	18362	0	45	42	17098	538	57303	511	29645
chrX	7476537	G	0	63	0	105	0	96	0	26	1	319	1	132
chrX	7476538	C	52	27803	31	18825	0	46	35	17291	127	58359	45	30408
chrX	7476539	C	37	27831	17	18841	0	46	20	17307	173	58318	37	30428
chrX	7476541	C	47	27820	45	18810	0	46	25	17305	119	58381	150	30320
chrX	7476542	C	204	27610	270	18557	0	46	205	17091	575	57815	597	29808
chrX	7476543	G	0	72	0	118	1	95	0	33	9	327	0	138
chrX	7476545	C	42	27825	28	18836	1	45	25	17308	138	58363	62	30409
chrX	7476547	G	2	169	1	299	1	253	1	166	0	729	2	777
chrX	7476548	C	202	27655	278	18578	1	45	206	17119	570	57897	410	30049
chrX	7476549	G	19	1585	8	2289	5	2881	4	1557	15	8026	16	6439

0	36	0	76	0	82	0	14	0	255	0	97
0	38	0	78	0	86	0	13	1	260	0	95
1	39	0	78	0	87	0	14	2	260	0	95
0	39	0	76	1	86	0	14	0	259	1	97
73	27744	270	18769	1	45	71	17249	286	58262	55	30260
0	38	0	75	0	77	0	13	0	245	0	93
168	27816	279	18834	0	46	32	17298	482	58440	394	30353
1	52	0	87	0	100	0	16	0	279	0	102
0	53	0	88	0	100	0	17	0	279	0	103
0	51	0	88	0	101	0	17	0	285	0	98
110	27808	30	18830	0	46	25	17297	229	58422	393	30357
0	39	1	79	0	84	0	17	4	226	0	77
0	54	1	91	0	104	0	18	2	288	0	101
0	55	1	91	1	105	0	18	1	291	0	101
0	47	1	81	0	97	0	14	0	271	1	97
0	48	0	83	1	85	0	16	1	263	0	93
0	46	0	85	0	82	0	15	0	262	1	91
0	51	0	83	0	85	0	15	1	272	0	92
0	50	0	84	0	86	0	15	0	273	0	92
120	27834	271	18838	1	46	201	17309	289	58461	381	30388
0	51	0	84	0	85	0	16	2	268	0	90
0	51	0	86	0	86	0	17	0	276	0	95
0	51	0	87	0	86	0	17	0	276	0	96
0	51	1	89	0	87	0	17	0	281	0	98
0	50	0	89	0	88	0	17	0	284	0	98
0	51	0	101	0	91	0	21	0	294	0	106
0	51	0	103	1	92	0	21	0	296	0	109
0	55	0	101	0	93	0	19	2	303	0	114
41	27842	48	18842	0	46	41	17299	100	58465	64	30415
203	27782	32	18808	0	46	32	17276	410	58363	40	30379
0	65	0	99	0	95	0	25	8	319	0	125
71	27834	42	18849	0	46	36	17309	143	58453	165	30430
52	27859	35	18856	0	46	30	17324	140	58488	56	30460
147	27848	288	18844	0	46	42	17316	413	58444	396	30445
0	63	0	107	0	98	0	26	9	323	0	131
93	27588	284	18646	0	45	42	17140	538	57841	511	30156
0	63	0	105	0	96	0	26	1	320	1	133
52	27855	31	18856	0	46	35	17326	127	58486	45	30453
37	27868	17	18858	0	46	20	17327	173	58491	37	30465
47	27867	45	18855	0	46	25	17330	119	58500	150	30470
204	27814	270	18827	0	46	205	17296	575	58390	597	30405
0	72	0	118	1	96	0	33	9	336	0	138
42	27867	28	18864	1	46	25	17333	138	58501	62	30471
2	171	1	300	1	254	1	167	0	729	2	779
202	27857	278	18856	1	46	206	17325	570	58467	410	30459
19	1604	8	2297	5	2886	4	1561	15	8041	16	6455

chrX	7476550	C	68	27649	32	18658	1	45	31	17193	69	58051	46	30227
chrX	7476551	C	30	27837	17	18846	0	46	14	17317	81	58411	131	30344
chrX	7476553	G	0	1611	3	2307	3	2896	0	1563	4	8064	11	6476
chrX	7476554	G	4	1606	5	2301	2	2894	3	1560	23	8039	15	6465
chrX	7476555	C	64	27776	44	18798	0	46	35	17277	135	58297	59	30392
chrX	7476556	C	243	27625	277	18584	1	45	195	17137	780	57716	595	29884
chrX	7476557	G	26	1584	3	2299	5	2889	0	1564	84	7981	10	6471
chrX	7476558	G	2	1609	2	2302	8	2887	1	1562	12	8041	8	6476
chrX	7476560	C	35	27826	43	18817	0	46	40	17286	169	58306	48	30421
chrX	7476562	C	33	27833	14	18852	0	46	18	17312	81	58418	41	30438
chrX	7476564	G	0	1611	2	2300	3	2893	2	1562	10	8048	8	6476
chrX	7476565	G	2	1609	2	2300	2	2894	6	1558	15	8037	15	6467
chrX	7476566	C	108	27512	36	18626	0	46	208	16975	486	57521	270	29922
chrX	7476567	G	14	1597	4	2297	4	2885	5	1553	28	8020	18	6454
chrX	7476568	C	53	27809	39	18817	1	45	49	17278	120	58360	73	30401
chrX	7476569	C	70	27801	37	18827	0	46	16	17315	77	58430	43	30433
chrX	7476572	G	0	1596	1	2267	2	2865	2	1543	35	7895	8	6316
chrX	7476573	G	1	1610	3	2294	3	2885	1	1561	8	8017	7	6466
chrX	7476575	G	1	1610	3	2295	5	2883	2	1562	11	8020	7	6469
chrX	7476576	G	2	1608	3	2295	6	2882	2	1561	18	8010	5	6472
chrX	7476578	G	2	1609	5	2293	3	2883	4	1556	12	8018	8	6464
chrX	7476581	C	272	27592	135	18726	2	44	304	17028	769	57727	578	29888
chrX	7476582	G	30	1581	8	2283	6	2880	3	1560	60	7964	13	6459
chrX	7476583	C	81	27782	41	18821	0	46	42	17285	122	58356	61	30406
chrX	7476585	C	134	27734	41	18824	1	45	205	17127	618	57882	614	29853
chrX	7476586	G	13	1592	8	2283	1	2881	3	1561	63	7939	13	6459
chrX	7476587	C	209	27482	49	18637	1	44	209	16963	795	57223	619	29551
chrX	7476588	G	18	1585	2	2293	5	2879	1	1563	70	7935	9	6464
chrX	7476591	C	62	27795	37	18822	0	46	33	17295	87	58399	48	30407
chrX	7476592	C	43	27824	33	18824	0	46	179	17149	142	58354	81	30374
chrX	7476595	G	1	1604	1	2292	4	2877	3	1560	8	7986	7	6467
chrX	7476596	G	0	1605	8	2286	2	2879	3	1561	12	7987	12	6463
chrX	7476599	C	255	27583	285	18558	1	45	198	17116	800	57630	397	29983
chrX	7476600	G	33	1571	4	2290	6	2872	1	1563	63	7934	138	6335
chrX	7476601	G	3	1601	3	2287	11	2865	5	1557	25	7959	12	6457
chrX	7476602	C	147	27682	46	18790	2	44	46	17262	511	57930	259	30127
chrX	7476603	G	12	1592	3	2289	5	2873	1	1562	67	7923	8	6466
chrX	7476606	G	2	1602	6	2284	4	2869	1	1560	4	7985	7	6461
chrX	7476607	G	6	1598	1	2291	7	2869	4	1560	11	7983	8	6464
chrX	7476608	G	3	1601	6	2287	2	2872	5	1557	33	7962	19	6451
chrX	7476609	C	50	27796	55	18791	0	46	25	17295	139	58333	34	30364
chrX	7476611	C	37	27808	20	18825	0	46	33	17289	121	58346	27	30367
chrX	7476613	G	2	1602	2	2290	8	2869	1	1562	31	7962	14	6454
chrX	7476614	G	2	1602	2	2289	7	2870	3	1560	16	7977	13	6451
chrX	7476615	C	63	27775	51	18786	0	46	39	17284	194	58257	56	30310
chrX	7476618	C	206	27611	272	18554	1	45	34	17275	781	57637	588	29784

68	27717	32	18690	1	46	31	17224	69	58120	46	30273
30	27867	17	18863	0	46	14	17331	81	58492	131	30475
0	1611	3	2310	3	2899	0	1563	4	8068	11	6487
4	1610	5	2306	2	2896	3	1563	23	8062	15	6480
64	27840	44	18842	0	46	35	17312	135	58432	59	30451
243	27868	277	18861	1	46	195	17332	780	58496	595	30479
26	1610	3	2302	5	2894	0	1564	84	8065	10	6481
2	1611	2	2304	8	2895	1	1563	12	8053	8	6484
35	27861	43	18860	0	46	40	17326	169	58475	48	30469
33	27866	14	18866	0	46	18	17330	81	58499	41	30479
0	1611	2	2302	3	2896	2	1564	10	8058	8	6484
2	1611	2	2302	2	2896	6	1564	15	8052	15	6482
108	27620	36	18662	0	46	208	17183	486	58007	270	30192
14	1611	4	2301	4	2889	5	1558	28	8048	18	6472
53	27862	39	18856	1	46	49	17327	120	58480	73	30474
70	27871	37	18864	0	46	16	17331	77	58507	43	30476
0	1596	1	2268	2	2867	2	1545	35	7930	8	6324
1	1611	3	2297	3	2888	1	1562	8	8025	7	6473
1	1611	3	2298	5	2888	2	1564	11	8031	7	6476
2	1610	3	2298	6	2888	2	1563	18	8028	5	6477
2	1611	5	2298	3	2886	4	1560	12	8030	8	6472
272	27864	135	18861	2	46	304	17332	769	58496	578	30466
30	1611	8	2291	6	2886	3	1563	60	8024	13	6472
81	27863	41	18862	0	46	42	17327	122	58478	61	30467
134	27868	41	18865	1	46	205	17332	618	58500	614	30467
13	1605	8	2291	1	2882	3	1564	63	8002	13	6472
209	27691	49	18686	1	45	209	17172	795	58018	619	30170
18	1603	2	2295	5	2884	1	1564	70	8005	9	6473
62	27857	37	18859	0	46	33	17328	87	58486	48	30455
43	27867	33	18857	0	46	179	17328	142	58496	81	30455
1	1605	1	2293	4	2881	3	1563	8	7994	7	6474
0	1605	8	2294	2	2881	3	1564	12	7999	12	6475
255	27838	285	18843	1	46	198	17314	800	58430	397	30380
33	1604	4	2294	6	2878	1	1564	63	7997	138	6473
3	1604	3	2290	11	2876	5	1562	25	7984	12	6469
147	27829	46	18836	2	46	46	17308	511	58441	259	30386
12	1604	3	2292	5	2878	1	1563	67	7990	8	6474
2	1604	6	2290	4	2873	1	1561	4	7989	7	6468
6	1604	1	2292	7	2876	4	1564	11	7994	8	6472
3	1604	6	2293	2	2874	5	1562	33	7995	19	6470
50	27846	55	18846	0	46	25	17320	139	58472	34	30398
37	27845	20	18845	0	46	33	17322	121	58467	27	30394
2	1604	2	2292	8	2877	1	1563	31	7993	14	6468
2	1604	2	2291	7	2877	3	1563	16	7993	13	6464
63	27838	51	18837	0	46	39	17323	194	58451	56	30366
206	27817	272	18826	1	46	34	17309	781	58418	588	30372

chrX	7476619	G	17	1585	4	2286	4	2873	5	1560	64	7927	17	6452
chrX	7476620	G	2	1601	9	2277	5	2869	3	1561	11	7974	12	6453
chrX	7476621	G	6	1595	4	2289	2	2875	2	1563	32	7958	12	6458
chrX	7476623	C	39	27805	16	18816	0	45	24	17297	121	58338	49	30322
chrX	7476625	G	5	1594	4	2289	12	2862	3	1560	13	7973	8	6463
chrX	7476627	C	54	27783	33	18799	0	46	45	17276	114	58323	67	30282
chrX	7476628	C	120	27691	47	18751	0	46	37	17262	550	57825	334	29969
chrX	7476629	G	7	1590	3	2290	5	2866	3	1561	13	7970	11	6452
chrX	7476630	C	166	27671	45	18778	0	46	44	17272	459	57969	591	29754
chrX	7476631	G	4	1593	6	2280	7	2863	23	1540	40	7936	17	6443
chrX	7476632	C	102	27729	31	18787	0	46	35	17270	361	58046	402	29913
chrX	7476633	G	2	1596	3	2290	11	2862	7	1558	60	7922	18	6449
chrX	7476635	C	84	27751	275	18538	0	46	21	17294	240	58187	449	29877
chrX	7476636	G	18	1579	5	2287	6	2864	5	1559	16	7958	15	6445
chrX	7476637	C	70	27756	51	18768	0	46	51	17263	155	58259	87	30229
chrX	7476639	C	73	27758	21	18802	0	46	23	17292	101	58335	28	30307
chrX	7476641	G	3	1595	5	2288	7	2866	5	1559	17	7956	16	6450
chrX	7476642	G	0	1597	7	2283	6	2865	1	1563	12	7956	15	6447
chrX	7476643	C	156	27669	32	18780	0	46	29	17278	614	57777	462	29829
chrX	7476644	G	4	1594	4	2287	17	2854	3	1561	64	7906	8	6457
chrX	7476645	G	0	1596	6	2285	7	2864	2	1562	14	7958	13	6451
chrX	7476647	C	41	27775	15	18801	0	46	13	17294	88	58307	37	30259
chrX	7476650	C	309	27514	272	18543	1	45	13	17298	812	57580	580	29713
chrX	7476651	G	12	1579	4	2278	5	2856	6	1552	63	7887	19	6423
chrX	7476652	G	0	1595	4	2283	3	2861	3	1560	5	7958	12	6448
chrX	7476653	G	2	1589	4	2278	6	2852	9	1549	20	7931	15	6433
chrX	7476655	G	5	1591	5	2285	3	2861	6	1557	22	7942	14	6447
chrX	7476656	G	4	1587	6	2276	7	2853	3	1556	27	7924	13	6435
chrX	7476657	C	91	27724	234	18554	0	46	23	17288	245	58139	388	29876
chrX	7476658	G	4	1590	7	2278	10	2848	0	1562	57	7895	16	6432
chrX	7476659	G	5	1591	2	2287	5	2857	9	1553	13	7947	9	6454
chrX	7476660	G	3	1593	7	2282	7	2855	3	1560	30	7927	12	6448
chrX	7476661	C	78	27738	81	18724	1	45	194	17094	413	57931	559	29705
chrX	7476662	G	17	1528	6	2206	4	2805	6	1500	57	7752	6	6173
chrX	7476663	G	2	1592	1	2287	5	2857	1	1560	7	7943	15	6445
chrX	7476664	G	2	1592	2	2285	2	2861	0	1563	7	7945	14	6447
chrX	7476665	G	4	1589	3	2285	4	2860	3	1560	11	7934	20	6436
chrX	7476668	G	1	1591	5	2282	12	2850	1	1561	9	7944	9	6449
chrX	7476669	G	5	1583	13	2271	6	2853	3	1559	17	7920	15	6435
chrX	7476670	C	48	27725	111	18621	0	45	11	17288	37	58313	22	30154
chrX	7476672	G	2	1592	1	2285	4	2858	2	1560	3	7947	13	6444
chrX	7476673	G	0	1594	0	2286	0	2861	0	1561	0	7948	1	6456
chrX	7476674	G	1	1592	1	2285	0	2861	0	1561	2	7946	0	6457
chrX	7476675	G	0	1594	0	2286	0	2861	1	1560	1	7944	2	6452
chrX	7476676	C	4	27494	3	18002	0	45	2	16963	4	58230	3	29609
chrX	7476678	G	0	1591	0	2285	0	2860	0	1560	2	7940	1	6453

17	1602	4	2290	4	2877	5	1565	64	7991	17	6469
2	1603	9	2286	5	2874	3	1564	11	7985	12	6465
6	1601	4	2293	2	2877	2	1565	32	7990	12	6470
39	27844	16	18832	0	45	24	17321	121	58459	49	30371
5	1599	4	2293	12	2874	3	1563	13	7986	8	6471
54	27837	33	18832	0	46	45	17321	114	58437	67	30349
120	27811	47	18798	0	46	37	17299	550	58375	334	30303
7	1597	3	2293	5	2871	3	1564	13	7983	11	6463
166	27837	45	18823	0	46	44	17316	459	58428	591	30345
4	1597	6	2286	7	2870	23	1563	40	7976	17	6460
102	27831	31	18818	0	46	35	17305	361	58407	402	30315
2	1598	3	2293	11	2873	7	1565	60	7982	18	6467
84	27835	275	18813	0	46	21	17315	240	58427	449	30326
18	1597	5	2292	6	2870	5	1564	16	7974	15	6460
70	27826	51	18819	0	46	51	17314	155	58414	87	30316
73	27831	21	18823	0	46	23	17315	101	58436	28	30335
3	1598	5	2293	7	2873	5	1564	17	7973	16	6466
0	1597	7	2290	6	2871	1	1564	12	7968	15	6462
156	27825	32	18812	0	46	29	17307	614	58391	462	30291
4	1598	4	2291	17	2871	3	1564	64	7970	8	6465
0	1596	6	2291	7	2871	2	1564	14	7972	13	6464
41	27816	15	18816	0	46	13	17307	88	58395	37	30296
309	27823	272	18815	1	46	13	17311	812	58392	580	30293
12	1591	4	2282	5	2861	6	1558	63	7950	19	6442
0	1595	4	2287	3	2864	3	1563	5	7963	12	6460
2	1591	4	2282	6	2858	9	1558	20	7951	15	6448
5	1596	5	2290	3	2864	6	1563	22	7964	14	6461
4	1591	6	2282	7	2860	3	1559	27	7951	13	6448
91	27815	234	18788	0	46	23	17311	245	58384	388	30264
4	1594	7	2285	10	2858	0	1562	57	7952	16	6448
5	1596	2	2289	5	2862	9	1562	13	7960	9	6463
3	1596	7	2289	7	2862	3	1563	30	7957	12	6460
78	27816	81	18805	1	46	194	17288	413	58344	559	30264
17	1545	6	2212	4	2809	6	1506	57	7809	6	6179
2	1594	1	2288	5	2862	1	1561	7	7950	15	6460
2	1594	2	2287	2	2863	0	1563	7	7952	14	6461
4	1593	3	2288	4	2864	3	1563	11	7945	20	6456
1	1592	5	2287	12	2862	1	1562	9	7953	9	6458
5	1588	13	2284	6	2859	3	1562	17	7937	15	6450
48	27773	111	18732	0	45	11	17299	37	58350	22	30176
2	1594	1	2286	4	2862	2	1562	3	7950	13	6457
0	1594	0	2286	0	2861	0	1561	0	7948	1	6457
1	1593	1	2286	0	2861	0	1561	2	7948	0	6457
0	1594	0	2286	0	2861	1	1561	1	7945	2	6454
4	27498	3	18005	0	45	2	16965	4	58234	3	29612
0	1591	0	2285	0	2860	0	1560	2	7942	1	6454

chrX	7476679	C	4	27452	4	17979	0	45	3	16930	6	58162	6	29576
chrX	7476681	G	1	1590	1	2285	0	2858	1	1559	1	7937	0	6451
chrX	7476683	G	0	1590	0	2283	0	2854	0	1558	2	7929	0	6446
chrX	7476685	C	12	26926	13	17694	0	44	5	16630	19	56558	14	28841
chrX	7476688	G	0	1591	1	2284	1	2857	0	1559	2	7939	1	6448
chrX	7476690	C	3	26659	1	17538	0	44	5	16472	5	56051	0	28554
chrX	7476691	C	10	26587	0	17461	0	43	1	16429	8	55883	5	28462
chrX	7476692	C	2	26518	1	17401	0	43	3	16387	6	55749	2	28410
chrX	7476694	G	0	1586	0	2279	0	2854	1	1550	0	7928	0	6434
chrX	7476696	C	6	25929	2	17004	0	42	1	16044	4	54512	6	27787
chrX	7476697	C	7	25679	9	16838	0	42	6	15880	8	53989	17	27497
chrX	7539243	G	2	2181	0	1879	0	3355	0	961	1	5000	2	2611
chrX	7539248	G	0	2240	0	1923	1	3432	0	975	0	5113	0	2660
chrX	7539250	G	0	2306	1	1975	0	3511	1	985	2	5239	0	2725
chrX	7539258	G	0	2420	0	2057	0	3677	0	1009	0	5481	1	2867
chrX	7539275	G	20	2419	1	2067	39	3645	8	1002	13	5495	87	2795
chrX	7539276	G	5	2436	5	2065	7	3679	0	1010	1	5506	7	2875
chrX	7539286	G	8	2419	3	2055	16	3654	0	1006	69	5419	1	2867
chrX	7539290	G	5	2436	1	2063	20	3666	1	1006	11	5489	3	2876
chrX	7539302	G	8	2433	2	2067	5	3684	5	1006	5	5506	2	2879
chrX	7539303	G	5	2436	7	2064	8	3684	3	1008	14	5495	4	2879
chrX	7539304	G	7	2434	9	2063	10	3682	2	1008	11	5498	7	2876
chrX	7539305	G	7	2434	3	2067	15	3676	3	1008	16	5493	9	2875
chrX	7539311	G	3	2439	2	2070	36	3656	3	1006	3	5510	12	2871
chrX	7539312	G	2	2441	2	2068	17	3676	2	1009	8	5504	4	2879
chrX	7539315	G	3	2440	3	2068	20	3673	3	1008	12	5501	2	2882
chrX	7539316	G	4	2439	4	2067	9	3685	2	1009	15	5498	4	2879
chrX	7539324	G	6	2438	2	2069	5	3689	1	1010	19	5493	5	2876
chrX	7539331	G	14	2004	4	1708	3	3071	2	854	19	4621	3	2144
chrX	7539332	G	10	2418	0	2054	2	3646	2	1008	20	5441	4	2830
chrX	7539333	G	1	2443	9	2063	3	3687	1	1010	5	5505	6	2876
chrX	7539334	G	6	2438	4	2068	16	3677	3	1008	13	5499	8	2874
chrX	7539335	G	7	2437	4	2067	10	3683	1	1010	27	5485	5	2878
chrX	7539338	G	18	2426	4	2068	3	3688	2	1008	31	5481	6	2877
chrX	7539343	G	5	2440	3	2067	6	3689	2	1009	15	5499	7	2873
chrX	7539344	G	5	2439	5	2070	6	3685	1	1010	11	5503	4	2878
chrX	7539345	C	2	3097	3	8129	0	3131	0	6926	2	6725	3	12195
chrX	7539347	G	13	2429	4	2071	66	3633	1	1008	127	5385	3	2879
chrX	7539348	C	0	3529	3	9651	2	3628	3	8082	1	8204	4	14396
chrX	7539350	G	23	2421	2	2074	30	3672	1	1011	81	5431	125	2756
chrX	7539352	G	5	2439	5	2065	5	3698	2	1010	19	5497	7	2877
chrX	7539353	C	4	3569	1	9759	1	3663	2	8164	1	8281	5	14511
chrX	7539355	G	40	2393	26	2039	47	3629	93	911	100	5363	35	2833
chrX	7539357	G	13	2432	9	2066	8	3696	1	1011	22	5494	8	2878
chrX	7539359	G	35	2411	4	2077	9	3695	3	1009	10	5507	5	2882
chrX	7539360	G	2	2445	0	2080	4	3701	2	1011	10	5505	7	2880

4	27456	4	17983	0	45	3	16933	6	58168	6	29582
1	1591	1	2286	0	2858	1	1560	1	7938	0	6451
0	1590	0	2283	0	2854	0	1558	2	7931	0	6446
12	26938	13	17707	0	44	5	16635	19	56577	14	28855
0	1591	1	2285	1	2858	0	1559	2	7941	1	6449
3	26662	1	17539	0	44	5	16477	5	56056	0	28554
10	26597	0	17461	0	43	1	16430	8	55891	5	28467
2	26520	1	17402	0	43	3	16390	6	55755	2	28412
0	1586	0	2279	0	2854	1	1551	0	7928	0	6434
6	25935	2	17006	0	42	1	16045	4	54516	6	27793
7	25686	9	16847	0	42	6	15886	8	53997	17	27514
2	2183	0	1879	0	3355	0	961	1	5001	2	2613
0	2240	0	1923	1	3433	0	975	0	5113	0	2660
0	2306	1	1976	0	3511	1	986	2	5241	0	2725
0	2420	0	2057	0	3677	0	1009	0	5481	1	2868
20	2439	1	2068	39	3684	8	1010	13	5508	87	2882
5	2441	5	2070	7	3686	0	1010	1	5507	7	2882
8	2427	3	2058	16	3670	0	1006	69	5488	1	2868
5	2441	1	2064	20	3686	1	1007	11	5500	3	2879
8	2441	2	2069	5	3689	5	1011	5	5511	2	2881
5	2441	7	2071	8	3692	3	1011	14	5509	4	2883
7	2441	9	2072	10	3692	2	1010	11	5509	7	2883
7	2441	3	2070	15	3691	3	1011	16	5509	9	2884
3	2442	2	2072	36	3692	3	1009	3	5513	12	2883
2	2443	2	2070	17	3693	2	1011	8	5512	4	2883
3	2443	3	2071	20	3693	3	1011	12	5513	2	2884
4	2443	4	2071	9	3694	2	1011	15	5513	4	2883
6	2444	2	2071	5	3694	1	1011	19	5512	5	2881
14	2018	4	1712	3	3074	2	856	19	4640	3	2147
10	2428	0	2054	2	3648	2	1010	20	5461	4	2834
1	2444	9	2072	3	3690	1	1011	5	5510	6	2882
6	2444	4	2072	16	3693	3	1011	13	5512	8	2882
7	2444	4	2071	10	3693	1	1011	27	5512	5	2883
18	2444	4	2072	3	3691	2	1010	31	5512	6	2883
5	2445	3	2070	6	3695	2	1011	15	5514	7	2880
5	2444	5	2075	6	3691	1	1011	11	5514	4	2882
2	3099	3	8132	0	3131	0	6926	2	6727	3	12198
13	2442	4	2075	66	3699	1	1009	127	5512	3	2882
0	3529	3	9654	2	3630	3	8085	1	8205	4	14400
23	2444	2	2076	30	3702	1	1012	81	5512	125	2881
5	2444	5	2070	5	3703	2	1012	19	5516	7	2884
4	3573	1	9760	1	3664	2	8166	1	8282	5	14516
40	2433	26	2065	47	3676	93	1004	100	5463	35	2868
13	2445	9	2075	8	3704	1	1012	22	5516	8	2886
35	2446	4	2081	9	3704	3	1012	10	5517	5	2887
2	2447	0	2080	4	3705	2	1013	10	5515	7	2887

chrX	7539361	G	19	2424	2	2076	4	3699	6	1005	10	5500	7	2882
chrX	7539362	C	0	3587	1	9773	1	3670	2	8175	0	8307	5	14566
chrX	7539364	C	2	3593	1	9794	1	3676	3	8194	1	8320	1	14592
chrX	7539365	C	2	3593	1	9795	1	3676	2	8196	1	8321	2	14592
chrX	7539366	C	1	3592	1	9793	0	3677	3	8196	3	8317	4	14590
chrX	7539368	G	3	2443	115	1968	24	3663	2	1010	64	5443	6	2867
chrX	7539370	G	20	1274	1	1120	8	2030	0	561	5	3004	1	1557
chrX	7539371	G	17	2004	2	1683	10	3038	4	799	10	4581	3	2346
chrX	7539372	G	5	2309	6	1939	7	3519	6	924	8	5239	7	2688
chrX	7539374	G	52	2391	6	2071	20	3680	2	1001	82	5426	8	2865
chrX	7539376	G	24	2426	4	2080	71	3636	6	1007	61	5461	93	2796
chrX	7539379	G	7	2442	5	2079	4	3706	1	1011	14	5508	7	2884
chrX	7539383	G	3	2442	3	2074	6	3697	0	1010	10	5506	1	2886
chrX	7539384	G	2	2434	5	2072	6	3689	2	1002	6	5513	3	2876
chrX	7539386	C	12	3590	10	9794	1	3687	10	8193	25	8308	26	14588
chrX	7539388	G	65	2384	145	1937	101	3606	3	1008	108	5417	3	2888
chrX	7539389	G	0	2454	3	2078	11	3705	0	1014	17	5513	5	2891
chrX	7539390	G	4	2442	3	2073	10	3697	1	1006	18	5502	5	2888
chrX	7539394	G	1	1037	0	869	6	1504	0	382	8	2330	1	1313
chrX	7539396	C	7	3597	228	9583	24	3665	1	8205	160	8182	270	14356
chrX	7539398	G	1	262	0	201	1	355	0	59	2	535	2	283
chrX	7539399	C	10	3584	20	9753	17	3666	19	8175	44	8261	21	14556
chrX	7539401	C	9	3597	13	9802	15	3675	175	8037	11	8332	14	14615
chrX	7539402	C	12	3595	14	9801	12	3679	16	8197	19	8327	17	14609
chrX	7539403	C	3	3606	20	9796	6	3685	13	8199	20	8324	17	14612
chrX	7539404	C	11	3598	15	9801	17	3674	11	8202	8	8335	20	14611
chrX	7539405	C	3273	336	8670	1149	3351	339	6135	2077	7452	896	13240	1392
chrX	7539406	G	78	4	57	9	88	22	25	0	157	9	77	8
chrX	7539410	G	0	71	0	65	0	100	0	26	2	153	0	75
chrX	7539411	C	31	3558	90	9649	46	3621	8	8153	43	8256	20	14506
chrX	7539413	C	50	3559	15	9807	95	3600	337	7879	158	8200	384	14255
chrX	7539415	G	0	73	0	62	1	94	0	27	0	151	0	75
chrX	7539416	C	16	3595	8	9815	4	3695	12	8202	18	8339	52	14594
chrX	7539417	C	3273	339	8405	1414	3337	362	6892	1326	7334	1026	12128	2517
chrX	7539418	G	52	5	39	16	82	12	25	2	123	21	63	9
chrX	7539419	G	0	58	0	53	2	95	2	26	1	140	0	71
chrX	7539421	G	2	54	1	50	2	96	0	28	3	132	0	69
chrX	7539423	C	34	3572	245	9563	43	3653	12	8205	162	8195	255	14389
chrX	7539425	G	0	60	0	49	0	98	0	27	2	135	0	67
chrX	7539426	G	0	60	0	50	0	98	0	27	1	137	1	67
chrX	7539427	G	0	60	0	50	0	98	0	27	0	140	0	68
chrX	7539429	G	2	56	8	39	3	92	0	26	6	138	1	66
chrX	7539430	C	3313	301	8894	931	3368	332	7407	811	7615	752	12471	2184
chrX	7539431	G	57	2	43	6	87	12	25	1	132	13	58	8
chrX	7539434	G	1	60	1	48	2	96	3	22	6	142	11	57
chrX	7539435	C	84	3532	14	9806	104	3597	44	8175	156	8213	316	14343

19	2443	2	2078	4	3703	6	1011	10	5510	7	2889
0	3587	1	9774	1	3671	2	8177	0	8307	5	14571
2	3595	1	9795	1	3677	3	8197	1	8321	1	14593
2	3595	1	9796	1	3677	2	8198	1	8322	2	14594
1	3593	1	9794	0	3677	3	8199	3	8320	4	14594
3	2446	115	2083	24	3687	2	1012	64	5507	6	2873
20	1294	1	1121	8	2038	0	561	5	3009	1	1558
17	2021	2	1685	10	3048	4	803	10	4591	3	2349
5	2314	6	1945	7	3526	6	930	8	5247	7	2695
52	2443	6	2077	20	3700	2	1003	82	5508	8	2873
24	2450	4	2084	71	3707	6	1013	61	5522	93	2889
7	2449	5	2084	4	3710	1	1012	14	5522	7	2891
3	2445	3	2077	6	3703	0	1010	10	5516	1	2887
2	2436	5	2077	6	3695	2	1004	6	5519	3	2879
12	3602	10	9804	1	3688	10	8203	25	8333	26	14614
65	2449	145	2082	101	3707	3	1011	108	5525	3	2891
0	2454	3	2081	11	3716	0	1014	17	5530	5	2896
4	2446	3	2076	10	3707	1	1007	18	5520	5	2893
1	1038	0	869	6	1510	0	382	8	2338	1	1314
7	3604	228	9811	24	3689	1	8206	160	8342	270	14626
1	263	0	201	1	356	0	59	2	537	2	285
10	3594	20	9773	17	3683	19	8194	44	8305	21	14577
9	3606	13	9815	15	3690	175	8212	11	8343	14	14629
12	3607	14	9815	12	3691	16	8213	19	8346	17	14626
3	3609	20	9816	6	3691	13	8212	20	8344	17	14629
11	3609	15	9816	17	3691	11	8213	8	8343	20	14631
3273	3609	8670	9819	3351	3690	6135	8212	7452	8348	13240	14632
78	82	57	66	88	110	25	25	157	166	77	85
0	71	0	65	0	100	0	26	2	155	0	75
31	3589	90	9739	46	3667	8	8161	43	8299	20	14526
50	3609	15	9822	95	3695	337	8216	158	8358	384	14639
0	73	0	62	1	95	0	27	0	151	0	75
16	3611	8	9823	4	3699	12	8214	18	8357	52	14646
3273	3612	8405	9819	3337	3699	6892	8218	7334	8360	12128	14645
52	57	39	55	82	94	25	27	123	144	63	72
0	58	0	53	2	97	2	28	1	141	0	71
2	56	1	51	2	98	0	28	3	135	0	69
34	3606	245	9808	43	3696	12	8217	162	8357	255	14644
0	60	0	49	0	98	0	27	2	137	0	67
0	60	0	50	0	98	0	27	1	138	1	68
0	60	0	50	0	98	0	27	0	140	0	68
2	58	8	47	3	95	0	26	6	144	1	67
3313	3614	8894	9825	3368	3700	7407	8218	7615	8367	12471	14655
57	59	43	49	87	99	25	26	132	145	58	66
1	61	1	49	2	98	3	25	6	148	11	68
84	3616	14	9820	104	3701	44	8219	156	8369	316	14659

chrX	7539437	G	0	63	0	48	1	98	0	23	0	149	1	70
chrX	7539438	G	0	62	0	51	0	100	0	23	0	150	1	69
chrX	7539439	C	22	3586	20	9774	34	3660	16	8184	47	8310	20	14608
chrX	7539441	G	0	62	1	50	0	98	0	23	1	151	0	70
chrX	7539443	G	11	52	13	37	8	88	0	22	10	146	7	64
chrX	7539444	C	236	3372	703	9089	263	3428	139	8048	649	7695	352	14246
chrX	7539446	G	0	65	0	50	2	96	0	23	1	159	0	72
chrX	7539448	C	8	3609	16	9815	6	3695	20	8206	33	8342	28	14635
chrX	7539449	C	11	3606	25	9806	8	3696	13	8213	31	8344	30	14634
chrX	7539450	C	7	3609	10	9821	1	3702	14	8211	13	8359	14	14645
chrX	7539451	C	14	3604	24	9809	47	3656	16	8211	47	8330	16	14650
chrX	7539453	G	0	68	0	48	1	97	0	24	0	174	2	75
chrX	7539454	C	3160	462	8059	1767	3265	436	7038	1188	7053	1329	11483	3193
chrX	7539455	G	54	14	40	8	82	15	23	1	153	19	74	3
chrX	7539457	C	16	3606	19	9814	34	3672	267	7965	45	8341	151	14532
chrX	7539459	G	0	67	1	45	3	98	0	24	0	186	3	75
chrX	7539460	G	0	67	3	44	1	101	0	24	0	187	0	79
chrX	7539462	G	0	68	1	49	0	104	2	23	1	182	1	82
chrX	7539463	G	0	70	0	51	0	105	0	25	0	186	1	84
chrX	7539465	C	11	3613	18	9816	13	3696	15	8215	34	8362	29	14660
chrX	7539467	G	1	69	0	54	1	106	0	26	4	191	0	88
chrX	7539468	G	0	72	0	55	0	108	0	26	0	198	0	88
chrX	7539470	C	14	3612	23	9808	9	3702	16	8218	29	8369	24	14704
chrX	7539472	C	73	3555	397	9440	79	3634	9	8226	238	8168	146	14586
chrX	7539474	G	1	73	1	55	0	115	1	25	1	205	0	90
chrX	7539475	C	9	3619	19	9816	12	3700	15	8217	21	8386	26	14697
chrX	7539476	C	3439	188	8307	1530	3520	190	7360	877	7508	896	13062	1632
chrX	7539477	G	74	3	46	9	104	11	25	2	200	17	90	9
chrX	7539478	C	47	3585	78	9763	30	3684	24	8217	87	8325	91	14666
chrX	7539480	C	10	3622	20	9818	19	3696	10	8231	30	8388	30	14728
chrX	7539481	C	3312	321	8477	1366	3382	330	7475	763	7389	1028	11990	2771
chrX	7539482	G	96	6	61	8	115	8	26	2	211	22	95	9
chrX	7539484	C	5	3627	19	9823	9	3706	22	8218	34	8385	36	14727
chrX	7539487	C	39	3594	5	9842	57	3657	8	8233	81	8329	21	14733
chrX	7539490	G	0	107	0	72	2	128	0	27	1	249	0	120
chrX	7539491	G	0	125	0	76	0	160	0	31	1	279	0	140
chrX	7539493	G	13	354	3	184	16	467	1	81	18	754	11	412
chrX	7539495	C	8	3617	17	9816	6	3707	17	8222	27	8387	22	14738
chrX	7539496	C	20	742	18	1883	19	709	138	1390	58	2170	43	3592
chrX	7539499	G	1	554	0	325	0	744	0	134	1	1166	2	635
chrX	7539502	C	211	77	147	90	136	53	179	82	494	225	1045	305
chrX	7539503	G	1998	438	1799	249	2801	889	783	219	4480	1046	1943	908
chrX	7539505	G	10	2462	2	2095	12	3740	2	1017	7	5615	1	2933
chrX	7539508	G	46	2425	0	2097	6	3746	0	1019	36	5585	4	2930
chrX	7539511	G	7	2462	2	2091	7	3737	2	1016	49	5558	1	2930
chrX	7539512	C	3	185	0	156	2	127	1	161	5	482	0	970

0	63	0	48	1	99	0	23	0	149	1	71
0	62	0	51	0	100	0	23	0	150	1	70
22	3608	20	9794	34	3694	16	8200	47	8357	20	14628
0	62	1	51	0	98	0	23	1	152	0	70
11	63	13	50	8	96	0	22	10	156	7	71
236	3608	703	9792	263	3691	139	8187	649	8344	352	14598
0	65	0	50	2	98	0	23	1	160	0	72
8	3617	16	9831	6	3701	20	8226	33	8375	28	14663
11	3617	25	9831	8	3704	13	8226	31	8375	30	14664
7	3616	10	9831	1	3703	14	8225	13	8372	14	14659
14	3618	24	9833	47	3703	16	8227	47	8377	16	14666
0	68	0	48	1	98	0	24	0	174	2	77
3160	3622	8059	9826	3265	3701	7038	8226	7053	8382	11483	14676
54	68	40	48	82	97	23	24	153	172	74	77
16	3622	19	9833	34	3706	267	8232	45	8386	151	14683
0	67	1	46	3	101	0	24	0	186	3	78
0	67	3	47	1	102	0	24	0	187	0	79
0	68	1	50	0	104	2	25	1	183	1	83
0	70	0	51	0	105	0	25	0	186	1	85
11	3624	18	9834	13	3709	15	8230	34	8396	29	14689
1	70	0	54	1	107	0	26	4	195	0	88
0	72	0	55	0	108	0	26	0	198	0	88
14	3626	23	9831	9	3711	16	8234	29	8398	24	14728
73	3628	397	9837	79	3713	9	8235	238	8406	146	14732
1	74	1	56	0	115	1	26	1	206	0	90
9	3628	19	9835	12	3712	15	8232	21	8407	26	14723
3439	3627	8307	9837	3520	3710	7360	8237	7508	8404	13062	14694
74	77	46	55	104	115	25	27	200	217	90	99
47	3632	78	9841	30	3714	24	8241	87	8412	91	14757
10	3632	20	9838	19	3715	10	8241	30	8418	30	14758
3312	3633	8477	9843	3382	3712	7475	8238	7389	8417	11990	14761
96	102	61	69	115	123	26	28	211	233	95	104
5	3632	19	9842	9	3715	22	8240	34	8419	36	14763
39	3633	5	9847	57	3714	8	8241	81	8410	21	14754
0	107	0	72	2	130	0	27	1	250	0	120
0	125	0	76	0	160	0	31	1	280	0	140
13	367	3	187	16	483	1	82	18	772	11	423
8	3625	17	9833	6	3713	17	8239	27	8414	22	14760
20	762	18	1901	19	728	138	1528	58	2228	43	3635
1	555	0	325	0	744	0	134	1	1167	2	637
211	288	147	237	136	189	179	261	494	719	1045	1350
1998	2436	1799	2048	2801	3690	783	1002	4480	5526	1943	2851
10	2472	2	2097	12	3752	2	1019	7	5622	1	2934
46	2471	0	2097	6	3752	0	1019	36	5621	4	2934
7	2469	2	2093	7	3744	2	1018	49	5607	1	2931
3	188	0	156	2	129	1	162	5	487	0	970

chrX	7539514	C	0	264	0	235	0	185	1	233	1	670	0	1290
chrX	7539517	C	2	241	2	221	0	178	3	214	0	625	13	1157
chrX	7539518	C	0	264	2	227	0	186	0	226	3	678	1	1304
chrX	7539519	C	1	265	1	230	2	179	0	228	1	697	5	1307
chrX	7539522	C	1	246	0	214	0	179	0	213	1	653	2	1195
chrX	7539523	C	0	247	0	215	0	178	2	213	2	652	0	1198
chrX	7539525	G	2	2468	64	2031	2	3741	1	1018	15	5597	5	2928
chrX	7539526	G	3	2467	3	2090	5	3741	1	1018	9	5606	4	2928
chrX	7539528	C	1	237	0	212	0	165	1	209	2	612	2	1110
chrX	7539531	C	1	231	0	220	0	161	0	200	0	604	3	1059
chrX	7539532	C	2	215	0	227	0	142	0	201	0	562	0	996
chrX	7539534	G	6	2463	1	2094	4	3739	1	1017	10	5600	9	2924
chrX	7539535	G	7	2462	4	2091	7	3735	3	1014	6	5605	9	2921
chrX	7539536	G	8	2457	17	2073	6	3733	8	1008	12	5590	3	2923
chrX	7539537	C	10	3525	20	9775	7	3635	9	8178	27	8160	26	14232
chrX	7539538	C	4	3530	9	9783	2	3639	14	8162	29	8152	28	14224
chrX	7539540	G	20	2301	1	1962	35	3495	2	922	70	5186	1	2713
chrX	7539541	G	3	2458	4	2081	3	3733	1	1015	6	5592	2	2917
chrX	7539542	G	2	2465	1	2092	3	3739	0	1017	22	5586	3	2925
chrX	7539545	G	4	2462	2	2093	5	3736	2	1015	21	5586	8	2920
chrX	7539546	G	4	2461	6	2089	4	3738	0	1016	15	5590	9	2920
chrX	7539547	G	4	2460	7	2087	8	3734	3	1009	16	5588	2	2927
chrX	7539548	G	3	2461	4	2088	11	3726	1	1011	20	5580	6	2915
chrX	7539549	C	18	3492	11	9773	12	3618	11	8165	27	8066	149	13954
chrX	7539552	C	8	3503	19	9761	7	3623	17	8156	30	8053	32	14076
chrX	7539553	C	6	3490	76	9695	8	3618	14	8138	64	7999	349	13687
chrX	7539555	C	4	3505	6	9776	17	3609	7	8162	9	8074	11	14089
chrX	7539558	G	3	2461	2	2088	4	3735	0	1014	7	5595	4	2921
chrX	7539559	G	7	2455	6	2082	6	3730	3	1009	8	5590	1	2921
chrX	7539560	C	4	3491	11	9762	2	3618	13	8148	17	8017	15	13953
chrX	7539562	G	74	2387	2	2086	54	3681	0	1012	125	5471	135	2786
chrX	7539563	G	3	2460	6	2083	1	3738	2	1011	8	5593	115	2809
chrX	7539564	G	3	2460	8	2081	8	3730	2	1011	11	5591	4	2919
chrX	7539565	G	5	2456	39	2047	9	3722	1	1009	18	5578	9	2912
chrX	7539566	C	7	3471	12	9761	24	3593	11	8139	21	7977	174	13717
chrX	7539568	C	31	3451	9	9762	22	3596	212	7938	64	7937	18	13876
chrX	7539570	C	93	3387	399	9370	81	3536	492	7652	300	7695	182	13700
chrX	7539572	G	8	2456	4	2086	7	3727	2	1010	13	5587	5	2919
chrX	7539575	G	17	2442	1	2088	11	3722	0	1013	18	5580	1	2919
chrX	7539577	G	16	2445	6	2084	5	3729	0	1013	21	5578	2	2922
chrX	7539578	G	9	2449	1	2089	15	3718	4	1009	6	5591	6	2914
chrX	7539579	C	10	3466	96	9670	22	3593	58	8089	34	7949	15	13861
chrX	7539581	G	2	2457	4	2086	5	3726	1	1012	24	5574	4	2918
chrX	7539582	G	0	2460	2	2088	10	3722	1	1011	8	5590	3	2915
chrX	7539583	G	17	2443	4	2086	3	3729	2	1011	8	5590	7	2915
chrX	7539586	G	0	2459	1	2087	6	3725	2	1011	5	5590	57	2863

0	264	0	235	0	185	1	234	1	671	0	1290
2	243	2	223	0	178	3	217	0	625	13	1170
0	264	2	229	0	186	0	226	3	681	1	1305
1	266	1	231	2	181	0	228	1	698	5	1312
1	247	0	214	0	179	0	213	1	654	2	1197
0	247	0	215	0	178	2	215	2	654	0	1198
2	2470	64	2095	2	3743	1	1019	15	5612	5	2933
3	2470	3	2093	5	3746	1	1019	9	5615	4	2932
1	238	0	212	0	165	1	210	2	614	2	1112
1	232	0	220	0	161	0	200	0	604	3	1062
2	217	0	227	0	142	0	201	0	562	0	996
6	2469	1	2095	4	3743	1	1018	10	5610	9	2933
7	2469	4	2095	7	3742	3	1017	6	5611	9	2930
8	2465	17	2090	6	3739	8	1016	12	5602	3	2926
10	3535	20	9795	7	3642	9	8187	27	8187	26	14258
4	3534	9	9792	2	3641	14	8176	29	8181	28	14252
20	2321	1	1963	35	3530	2	924	70	5256	1	2714
3	2461	4	2085	3	3736	1	1016	6	5598	2	2919
2	2467	1	2093	3	3742	0	1017	22	5608	3	2928
4	2466	2	2095	5	3741	2	1017	21	5607	8	2928
4	2465	6	2095	4	3742	0	1016	15	5605	9	2929
4	2464	7	2094	8	3742	3	1012	16	5604	2	2929
3	2464	4	2092	11	3737	1	1012	20	5600	6	2921
18	3510	11	9784	12	3630	11	8176	27	8093	149	14103
8	3511	19	9780	7	3630	17	8173	30	8083	32	14108
6	3496	76	9771	8	3626	14	8152	64	8063	349	14036
4	3509	6	9782	17	3626	7	8169	9	8083	11	14100
3	2464	2	2090	4	3739	0	1014	7	5602	4	2925
7	2462	6	2088	6	3736	3	1012	8	5598	1	2922
4	3495	11	9773	2	3620	13	8161	17	8034	15	13968
74	2461	2	2088	54	3735	0	1012	125	5596	135	2921
3	2463	6	2089	1	3739	2	1013	8	5601	115	2924
3	2463	8	2089	8	3738	2	1013	11	5602	4	2923
5	2461	39	2086	9	3731	1	1010	18	5596	9	2921
7	3478	12	9773	24	3617	11	8150	21	7998	174	13891
31	3482	9	9771	22	3618	212	8150	64	8001	18	13894
93	3480	399	9769	81	3617	492	8144	300	7995	182	13882
8	2464	4	2090	7	3734	2	1012	13	5600	5	2924
17	2459	1	2089	11	3733	0	1013	18	5598	1	2920
16	2461	6	2090	5	3734	0	1013	21	5599	2	2924
9	2458	1	2090	15	3733	4	1013	6	5597	6	2920
10	3476	96	9766	22	3615	58	8147	34	7983	15	13876
2	2459	4	2090	5	3731	1	1013	24	5598	4	2922
0	2460	2	2090	10	3732	1	1012	8	5598	3	2918
17	2460	4	2090	3	3732	2	1013	8	5598	7	2922
0	2459	1	2088	6	3731	2	1013	5	5595	57	2920

chrX	7539588	G	158	2301	319	1770	114	3619	1	1012	299	5297	218	2702
chrX	7539590	G	132	2315	194	1887	114	3602	1	1011	83	5495	231	2677
chrX	7539591	C	14	3458	17	9748	5	3612	181	7961	33	7935	24	13836
chrX	7539592	C	3145	310	9358	368	3344	257	7209	905	7335	602	12332	1484
chrX	7539593	G	2234	224	1851	238	3298	435	827	186	5131	466	2776	144
chrX	7539595	C	11	3460	26	9735	11	3603	13	8127	19	7945	25	13824
chrX	7539598	G	27	2426	136	1950	64	3664	0	1013	89	5496	3	2911
chrX	7539599	C	17	3446	25	9737	7	3601	8	8121	22	7930	30	13808
chrX	7539600	C	10	3453	10	9753	19	3594	14	8122	16	7938	12	13825
chrX	7539604	G	2	2455	3	2084	4	3721	0	1010	3	5583	6	2910
chrX	7539605	G	6	2432	6	2074	11	3692	6	998	15	5536	5	2893
chrX	7539606	C	8	3448	15	9731	9	3603	20	8105	22	7921	211	13616
chrX	7539607	C	4	3451	18	9734	5	3607	12	8112	14	7930	26	13794
chrX	7539608	C	12	3444	12	9745	19	3593	162	7960	18	7925	22	13802
chrX	7539610	G	2	2453	1	2086	6	3717	1	1012	12	5570	9	2906
chrX	7539611	C	17	3438	29	9725	8	3602	4	8117	40	7900	193	13622
chrX	7539614	G	8	2444	3	2084	40	3681	1	1009	23	5560	5	2911
chrX	7539615	C	45	3407	131	9620	35	3575	6	8117	117	7815	335	13479
chrX	7539620	G	7	2445	4	2082	15	3705	0	1009	10	5565	4	2910
chrX	7539621	G	9	2445	7	2078	6	3714	2	1008	16	5558	2	2911
chrX	7539623	G	112	2339	137	1948	175	3543	1	1008	217	5355	2	2908
chrX	7539625	G	4	2449	6	2078	17	3700	4	1006	37	5531	8	2902
chrX	7539626	C	11	3438	19	9716	8	3598	14	8093	22	7876	26	13766
chrX	7539627	C	8	3446	21	9722	10	3598	24	8093	30	7902	37	13766
chrX	7539628	C	5	3448	20	9727	9	3600	20	8097	19	7914	24	13780
chrX	7539629	C	3	3449	19	9722	5	3601	11	8091	30	7898	17	13776
chrX	7539630	C	12	3440	244	9499	10	3598	6	8108	107	7824	19	13779
chrX	7539632	G	0	2426	0	2071	1	3709	1	1008	2	5542	1	2896
chrX	7539633	G	0	2427	0	2071	0	3708	0	1009	3	5543	0	2895
chrX	7539638	C	6	3424	20	9705	6	3587	13	8081	19	7882	25	13676
chrX	7539639	C	5	3432	148	9575	3	3598	5	8090	59	7860	42	13680
chrX	7539640	C	17	3418	181	9513	17	3580	164	7904	116	7797	15	13702
chrX	7539642	C	6	3425	19	9696	4	3587	16	8070	19	7876	22	13673
chrX	7539643	C	41	3388	13	9704	33	3559	14	8074	94	7801	25	13664
chrX	7539647	C	5	3423	11	9705	7	3583	6	8082	37	7852	41	13656
chrX	7539649	G	1	1969	0	1808	0	3083	0	903	0	4606	1	2360
chrX	7539652	G	1	1924	2	1771	0	3006	0	889	1	4495	0	2327
chrX	7539655	C	14	3413	234	9479	38	3553	11	8075	176	7709	31	13646
chrX	7539664	C	0	3423	1	9678	0	3582	0	8072	0	7869	2	13661
chrX	7539666	C	0	3422	2	9671	1	3577	1	8070	0	7871	3	13659
chrX	7539667	C	0	3421	0	9677	0	3581	5	8069	1	7871	2	13657
chrX	7539675	C	0	3420	0	9669	1	3578	1	8067	0	7853	2	13629

158	2459	319	2089	114	3733	1	1013	299	5596	218	2920
132	2447	194	2081	114	3716	1	1012	83	5578	231	2908
14	3472	17	9765	5	3617	181	8142	33	7968	24	13860
3145	3455	9358	9726	3344	3601	7209	8114	7335	7937	12332	13816
2234	2458	1851	2089	3298	3733	827	1013	5131	5597	2776	2920
11	3471	26	9761	11	3614	13	8140	19	7964	25	13849
27	2453	136	2086	64	3728	0	1013	89	5585	3	2914
17	3463	25	9762	7	3608	8	8129	22	7952	30	13838
10	3463	10	9763	19	3613	14	8136	16	7954	12	13837
2	2457	3	2087	4	3725	0	1010	3	5586	6	2916
6	2438	6	2080	11	3703	6	1004	15	5551	5	2898
8	3456	15	9746	9	3612	20	8125	22	7943	211	13827
4	3455	18	9752	5	3612	12	8124	14	7944	26	13820
12	3456	12	9757	19	3612	162	8122	18	7943	22	13824
2	2455	1	2087	6	3723	1	1013	12	5582	9	2915
17	3455	29	9754	8	3610	4	8121	40	7940	193	13815
8	2452	3	2087	40	3721	1	1010	23	5583	5	2916
45	3452	131	9751	35	3610	6	8123	117	7932	335	13814
7	2452	4	2086	15	3720	0	1009	10	5575	4	2914
9	2454	7	2085	6	3720	2	1010	16	5574	2	2913
112	2451	137	2085	175	3718	1	1009	217	5572	2	2910
4	2453	6	2084	17	3717	4	1010	37	5568	8	2910
11	3449	19	9735	8	3606	14	8107	22	7898	26	13792
8	3454	21	9743	10	3608	24	8117	30	7932	37	13803
5	3453	20	9747	9	3609	20	8117	19	7933	24	13804
3	3452	19	9741	5	3606	11	8102	30	7928	17	13793
12	3452	244	9743	10	3608	6	8114	107	7931	19	13798
0	2426	0	2071	1	3710	1	1009	2	5544	1	2897
0	2427	0	2071	0	3708	0	1009	3	5546	0	2895
6	3430	20	9725	6	3593	13	8094	19	7901	25	13701
5	3437	148	9723	3	3601	5	8095	59	7919	42	13722
17	3435	181	9694	17	3597	164	8068	116	7913	15	13717
6	3431	19	9715	4	3591	16	8086	19	7895	22	13695
41	3429	13	9717	33	3592	14	8088	94	7895	25	13689
5	3428	11	9716	7	3590	6	8088	37	7889	41	13697
1	1970	0	1808	0	3083	0	903	0	4606	1	2361
1	1925	2	1773	0	3006	0	889	1	4496	0	2327
14	3427	234	9713	38	3591	11	8086	176	7885	31	13677
0	3423	1	9679	0	3582	0	8072	0	7869	2	13663
0	3422	2	9673	1	3578	1	8071	0	7871	3	13662
0	3421	0	9677	0	3581	5	8074	1	7872	2	13659
0	3420	0	9669	1	3579	1	8068	0	7853	2	13631

Lux estimates

					INDIVIDUAL REPLICATES																	
					v651			v652			v653			t2kd1			t2kd2			t2kd3		
Chro	Position	ID	Nu	Cp	p("C")	p("5m(	p("5hn	p("C")	p("5m(	p("5hn	p("C")	p("5m(	p("5hn	p("C")	p("5m(	p("5hn	p("C")	p("5m(	p("5hn	p("C")	p("5m(	p("5hmC")
chr4	139783857	1373	G	1	0.74	0.17	0.09	0.73	0.05	0.21	0.69	0.20	0.11	0.38	0.62	0.00	0.41	0.59	0.00	0.26	0.58	0.16
chr4	139783863	1376	G	1	0.64	0.17	0.19	0.68	0.05	0.28	0.63	0.25	0.12	0.36	0.48	0.16	0.35	0.65	0.00	0.25	0.65	0.11
chr15	100300108	406	C	1	0.68	0.30	0.01	0.69	0.23	0.07	0.76	0.21	0.03	0.46	0.24	0.30	0.44	0.16	0.39	0.44	0.26	0.29
chr4	139783853	1369	G	1	0.70	0.20	0.09	0.73	0.09	0.18	0.68	0.23	0.10	0.42	0.50	0.08	0.39	0.61	0.00	0.27	0.59	0.14
chr15	61868725	235	C	1	0.52	0.41	0.07	0.52	0.16	0.32	0.47	0.39	0.15	0.32	0.66	0.02	0.31	0.68	0.00	0.29	0.65	0.06
chr4	139783746	1310	G	1	0.55	0.35	0.11	0.54	0.24	0.22	0.59	0.29	0.12	0.28	0.68	0.04	0.32	0.63	0.05	0.28	0.67	0.04
chr4	139783852	1368	C	1	0.64	0.29	0.08	0.62	0.14	0.24	0.64	0.29	0.06	0.36	0.62	0.02	0.30	0.62	0.08	0.32	0.66	0.02
chr17	45706559	762	G	1	0.54	0.37	0.09	0.56	0.15	0.29	0.48	0.37	0.15	0.31	0.46	0.23	0.27	0.73	0.00	0.28	0.63	0.09
chr4	139783862	1375	C	1	0.71	0.29	0.01	0.66	0.16	0.18	0.69	0.26	0.06	0.42	0.53	0.05	0.36	0.50	0.14	0.37	0.58	0.05
chr17	45706723	846	C	1	0.49	0.36	0.15	0.46	0.34	0.20	0.45	0.39	0.15	0.23	0.59	0.18	0.23	0.73	0.04	0.23	0.71	0.07
chr4	139783737	1304	G	1	0.44	0.40	0.15	0.48	0.32	0.20	0.47	0.46	0.07	0.26	0.72	0.01	0.26	0.74	0.00	0.23	0.73	0.05
chr15	61868802	272	G	1	0.51	0.41	0.08	0.47	0.26	0.26	0.50	0.36	0.13	0.29	0.60	0.11	0.26	0.66	0.08	0.29	0.68	0.04
chr15	100300203	442	G	1	0.54	0.42	0.04	0.53	0.27	0.19	0.49	0.38	0.14	0.26	0.60	0.14	0.23	0.73	0.03	0.24	0.64	0.11
chr15	61868758	252	G	1	0.40	0.45	0.15	0.38	0.34	0.27	0.38	0.46	0.16	0.22	0.66	0.12	0.22	0.71	0.07	0.21	0.72	0.07
chr15	61868726	236	G	1	0.54	0.36	0.10	0.48	0.26	0.27	0.51	0.34	0.16	0.33	0.59	0.08	0.27	0.64	0.09	0.30	0.61	0.09
chr11	3525888	167	G	1	0.54	0.44	0.01	0.60	0.27	0.13	0.51	0.48	0.01	0.20	0.75	0.06	0.25	0.66	0.09	0.23	0.70	0.08
chr4	139783856	1372	C	1	0.74	0.26	0.01	0.69	0.11	0.19	0.70	0.28	0.01	0.37	0.61	0.02	0.38	0.52	0.11	0.39	0.55	0.06
chr4	139783755	1315	G	1	0.47	0.37	0.16	0.49	0.35	0.16	0.47	0.43	0.10	0.27	0.72	0.01	0.25	0.74	0.01	0.22	0.76	0.01
chr15	61868834	290	G	1	0.50	0.38	0.12	0.44	0.25	0.31	0.47	0.34	0.19	0.30	0.55	0.15	0.30	0.67	0.03	0.29	0.62	0.09
chr15	61868731	239	C	1	0.32	0.60	0.08	0.31	0.30	0.39	0.27	0.46	0.27	0.18	0.81	0.01	0.20	0.79	0.01	0.17	0.76	0.07
chr15	100300140	421	G	1	0.58	0.32	0.10	0.60	0.29	0.11	0.53	0.37	0.10	0.33	0.58	0.10	0.28	0.59	0.13	0.23	0.56	0.21
chr15	100300109	407	G	1	0.70	0.27	0.03	0.70	0.12	0.18	0.64	0.21	0.15	0.43	0.46	0.12	0.40	0.60	0.01	0.43	0.46	0.11
chr17	45706558	761	C	1	0.58	0.26	0.16	0.53	0.31	0.15	0.57	0.19	0.24	0.34	0.45	0.21	0.34	0.55	0.11	0.34	0.59	0.07
chr4	139783678	1275	G	1	0.36	0.48	0.16	0.38	0.47	0.15	0.38	0.55	0.07	0.16	0.77	0.07	0.16	0.84	0.00	0.14	0.82	0.04
chr15	100300171	432	G	1	0.67	0.31	0.02	0.69	0.15	0.15	0.65	0.31	0.04	0.41	0.50	0.09	0.43	0.49	0.08	0.38	0.51	0.11
chr15	100300292	462	G	1	0.46	0.53	0.00	0.44	0.31	0.25	0.36	0.59	0.06	0.21	0.62	0.16	0.14	0.76	0.10	0.16	0.67	0.17
chr15	100300150	428	G	1	0.58	0.18	0.24	0.63	0.18	0.19	0.64	0.27	0.09	0.40	0.42	0.18	0.42	0.56	0.02	0.38	0.44	0.17
chr15	61868770	259	G	1	0.35	0.56	0.09	0.29	0.39	0.33	0.31	0.49	0.20	0.19	0.71	0.11	0.16	0.81	0.03	0.17	0.78	0.05
chr15	100300198	440	G	1	0.58	0.35	0.07	0.58	0.32	0.10	0.54	0.38	0.08	0.32	0.55	0.13	0.29	0.68	0.03	0.30	0.56	0.14
chr15	61868948	336	G	1	0.42	0.37	0.22	0.32	0.10	0.57	0.42	0.49	0.08	0.29	0.63	0.08	0.21	0.72	0.07	0.22	0.72	0.06
chr17	45706583	777	C	1	0.42	0.49	0.09	0.43	0.37	0.20	0.38	0.40	0.21	0.28	0.64	0.08	0.23	0.68	0.09	0.22	0.65	0.12
chr15	100300063	385	G	1	0.74	0.25	0.01	0.73	0.19	0.08	0.67	0.19	0.14	0.47	0.39	0.14	0.43	0.54	0.02	0.45	0.51	0.04
chr15	61868885	311	C	1	0.42	0.47	0.11	0.44	0.29	0.27	0.37	0.41	0.21	0.25	0.69	0.05	0.28	0.70	0.02	0.25	0.58	0.17
chr8	89376824	1580	g	1	0.49	0.45	0.06	0.54	0.39	0.07	0.40	0.42	0.18	0.23	0.65	0.12	0.29	0.67	0.04	0.22	0.65	0.12
chr17	45706598	786	C	1	0.59	0.33	0.08	0.59	0.34	0.07	0.54	0.37	0.09	0.38	0.57	0.05	0.34	0.65	0.01	0.29	0.70	0.01
chr15	61868801	271	C	1	0.47	0.47	0.06	0.48	0.28	0.25	0.44	0.49	0.07	0.26	0.73	0.01	0.28	0.70	0.03	0.24	0.75	0.01
chr17	45706508	734	C	1	0.62	0.23	0.15	0.61	0.31	0.08	0.59	0.27	0.14	0.44	0.51	0.05	0.44	0.43	0.13	0.44	0.51	0.04
chr4	139783745	1309	C	1	0.46	0.47	0.07	0.53	0.37	0.10	0.52	0.44	0.04	0.25	0.73	0.02	0.26	0.72	0.02	0.28	0.69	0.03

BIOLOGICAL SAMPLES						
v65			t2kd			
p("C")	p("5m(	p("5hn	p("C")	p("5m(	p("5hn	BF
0.79	0.11	0.11	0.36	0.62	0.02	67,776
0.71	0.11	0.17	0.31	0.66	0.03	42,9434
0.75	0.23	0.02	0.46	0.20	0.34	13,4187
0.76	0.14	0.10	0.36	0.61	0.03	7,62389
0.56	0.29	0.15	0.29	0.69	0.02	4,63507
0.60	0.27	0.12	0.29	0.68	0.03	4,09402
0.70	0.21	0.09	0.31	0.66	0.03	3,63642
0.60	0.26	0.15	0.27	0.68	0.05	3,3759
0.76	0.20	0.03	0.38	0.56	0.06	2,42232
0.45	0.38	0.16	0.22	0.72	0.06	2,28838
0.48	0.41	0.11	0.23	0.76	0.01	2,22254
0.57	0.30	0.13	0.25	0.69	0.05	2,18607
0.57	0.35	0.09	0.23	0.72	0.05	2,00014
0.41	0.42	0.17	0.20	0.74	0.07	1,82742
0.56	0.31	0.14	0.29	0.63	0.08	1,75874
0.59	0.39	0.03	0.20	0.75	0.05	1,71528
0.78	0.20	0.03	0.37	0.59	0.04	1,65277
0.50	0.39	0.11	0.23	0.76	0.01	1,58722
0.50	0.32	0.18	0.29	0.65	0.06	1,57142
0.32	0.50	0.18	0.15	0.83	0.02	1,48026
0.59	0.34	0.07	0.27	0.62	0.11	1,39558
0.76	0.16	0.07	0.44	0.52	0.04	1,3247
0.60	0.24	0.16	0.36	0.55	0.09	1,28908
0.36	0.54	0.10	0.13	0.86	0.02	1,24402
0.74	0.23	0.04	0.42	0.51	0.07	1,20602
0.46	0.51	0.03	0.14	0.73	0.12	1,19093
0.68	0.18	0.14	0.42	0.50	0.08	1,17122
0.32	0.49	0.18	0.15	0.81	0.04	1,13087
0.60	0.34	0.06	0.29	0.64	0.07	1,12805
0.43	0.36	0.21	0.23	0.72	0.06	1,05778
0.44	0.41	0.15	0.22	0.70	0.07	0,93964
0.79	0.17	0.04	0.47	0.49	0.04	0,88011
0.43	0.40	0.17	0.26	0.70	0.05	0,81293
0.51	0.41	0.08	0.22	0.71	0.07	0,77545
0.60	0.34	0.06	0.33	0.65	0.02	0,75038
0.47	0.44	0.09	0.23	0.76	0.01	0,74465
0.63	0.25	0.12	0.46	0.49	0.05	0,69996
0.54	0.41	0.05	0.25	0.73	0.02	0,69846

chr1!	100300149	427	C	1	0.56	0.29	0.15	0.69	0.19	0.13	0.58	0.26	0.16	0.36	0.28	0.36	0.37	0.58	0.06	0.40	0.55	0.06
chr1!	61868732	240	G	1	0.42	0.51	0.08	0.37	0.45	0.17	0.38	0.47	0.16	0.24	0.69	0.07	0.21	0.73	0.06	0.20	0.78	0.01
chr1!	61868886	312	G	1	0.49	0.44	0.07	0.47	0.34	0.18	0.49	0.39	0.12	0.36	0.58	0.05	0.28	0.71	0.01	0.31	0.61	0.07
chr4	139783736	1303	C	1	0.39	0.50	0.11	0.32	0.42	0.26	0.30	0.46	0.24	0.20	0.78	0.02	0.20	0.75	0.05	0.21	0.76	0.03
chr11	3525877	161	G	1	0.58	0.41	0.02	0.62	0.26	0.12	0.59	0.40	0.01	0.32	0.62	0.06	0.31	0.68	0.01	0.32	0.62	0.06
chr1(	92644509	609	C	1	0.39	0.39	0.23	0.44	0.33	0.23	0.47	0.44	0.10	0.34	0.61	0.04	0.26	0.52	0.23	0.36	0.62	0.02
chr1!	100300339	482	C	1	0.38	0.53	0.09	0.39	0.46	0.15	0.26	0.37	0.37	0.17	0.45	0.38	0.22	0.61	0.17	0.15	0.84	0.01
chr5	64987442	1460	C	1	0.36	0.53	0.11	0.39	0.39	0.23	0.23	0.47	0.30	0.25	0.72	0.04	0.25	0.62	0.13	0.23	0.69	0.09
chr1;	45706599	787	G	1	0.59	0.33	0.07	0.59	0.37	0.04	0.56	0.39	0.04	0.34	0.54	0.12	0.33	0.61	0.06	0.35	0.61	0.04
chr1;	45706584	778	G	1	0.34	0.58	0.09	0.36	0.39	0.25	0.30	0.57	0.13	0.17	0.82	0.01	0.16	0.83	0.01	0.17	0.76	0.06
chr1;	45706658	816	G	1	0.55	0.39	0.06	0.60	0.34	0.06	0.48	0.41	0.11	0.28	0.52	0.21	0.29	0.54	0.16	0.34	0.57	0.09
chr8	89376869	1607	c	1	0.27	0.59	0.14	0.26	0.55	0.19	0.29	0.55	0.15	0.15	0.72	0.13	0.15	0.72	0.13	0.18	0.81	0.01
chr11	3525867	155	C	1	0.42	0.51	0.07	0.42	0.37	0.21	0.43	0.49	0.08	0.23	0.65	0.12	0.24	0.76	0.00	0.22	0.73	0.06
chr8	89376870	1608	g	1	0.35	0.53	0.12	0.37	0.56	0.08	0.32	0.55	0.13	0.15	0.78	0.07	0.20	0.73	0.07	0.17	0.71	0.12
chr1!	100300139	420	C	1	0.44	0.46	0.10	0.44	0.45	0.10	0.55	0.41	0.04	0.27	0.52	0.21	0.22	0.76	0.02	0.22	0.68	0.10
chr1!	100300211	443	G	1	0.86	0.07	0.07	0.87	0.03	0.11	0.82	0.10	0.07	0.60	0.33	0.07	0.63	0.29	0.08	0.65	0.34	0.00
chr4	139783754	1314	C	1	0.39	0.58	0.03	0.36	0.30	0.34	0.41	0.55	0.05	0.13	0.87	0.00	0.17	0.74	0.10	0.19	0.79	0.01
chr1!	100300283	456	G	0	0.76	0.24	0.00	0.73	0.13	0.14	0.70	0.25	0.06	0.56	0.23	0.22	0.48	0.35	0.17	0.47	0.31	0.22
chr1;	45706600	788	C	1	0.52	0.35	0.12	0.57	0.39	0.05	0.52	0.41	0.07	0.36	0.49	0.15	0.39	0.37	0.24	0.32	0.54	0.14
chr1;	45706657	815	C	1	0.49	0.40	0.11	0.50	0.38	0.12	0.47	0.44	0.08	0.26	0.72	0.03	0.26	0.60	0.14	0.27	0.60	0.13
chr5	64987443	1461	G	1	0.17	0.62	0.22	0.17	0.61	0.21	0.21	0.65	0.14	0.15	0.75	0.10	0.17	0.80	0.03	0.18	0.80	0.02
chr4	139783700	1287	G	1	0.28	0.63	0.10	0.29	0.50	0.22	0.25	0.67	0.08	0.13	0.87	0.00	0.15	0.84	0.02	0.12	0.83	0.05
chr1(	92644555	635	C	1	0.54	0.36	0.10	0.58	0.32	0.10	0.60	0.24	0.16	0.41	0.55	0.04	0.39	0.56	0.05	0.43	0.54	0.03
chr1!	61868734	242	G	1	0.40	0.53	0.07	0.31	0.50	0.19	0.32	0.48	0.20	0.20	0.72	0.08	0.19	0.78	0.03	0.18	0.80	0.01
chr11	3525887	166	C	1	0.53	0.37	0.10	0.58	0.38	0.04	0.55	0.44	0.00	0.28	0.72	0.00	0.29	0.70	0.01	0.26	0.72	0.02
chr1(	92644538	627	G	1	0.46	0.37	0.17	0.56	0.32	0.13	0.53	0.37	0.10	0.38	0.58	0.04	0.38	0.56	0.06	0.38	0.54	0.08
chr11	3525893	170	C	1	0.46	0.45	0.09	0.46	0.37	0.17	0.48	0.48	0.04	0.27	0.71	0.02	0.27	0.62	0.11	0.25	0.74	0.01
chr4	139783677	1274	C	1	0.37	0.58	0.05	0.35	0.53	0.12	0.36	0.52	0.11	0.19	0.80	0.01	0.16	0.82	0.03	0.18	0.73	0.08
chr1!	100300235	449	G	1	0.61	0.34	0.05	0.58	0.25	0.17	0.61	0.33	0.06	0.42	0.48	0.10	0.39	0.59	0.02	0.40	0.53	0.07
chr11	3525644	28	G	1	0.45	0.50	0.05	0.46	0.16	0.38	0.43	0.53	0.04	0.28	0.62	0.10	0.30	0.69	0.00	0.27	0.64	0.08
chr11	3525757	85	G	1	0.55	0.41	0.04	0.59	0.32	0.08	0.53	0.43	0.05	0.30	0.69	0.02	0.34	0.62	0.04	0.30	0.64	0.06
chr1(	92644503	604	G	1	0.42	0.48	0.09	0.43	0.43	0.14	0.43	0.39	0.18	0.29	0.59	0.12	0.27	0.63	0.10	0.27	0.62	0.11
chr1!	100300040	373	G	1	0.90	0.06	0.04	0.87	0.07	0.05	0.86	0.07	0.07	0.71	0.22	0.07	0.67	0.28	0.05	0.68	0.26	0.05
chr4	139783639	1252	G	1	0.30	0.60	0.10	0.32	0.56	0.12	0.30	0.59	0.11	0.19	0.78	0.03	0.19	0.81	0.00	0.17	0.83	0.00
chr1(	92644455	568	C	1	0.66	0.30	0.04	0.70	0.22	0.09	0.65	0.24	0.11	0.45	0.53	0.01	0.47	0.45	0.08	0.49	0.44	0.07
chr1!	100300274	454	G	1	0.68	0.31	0.01	0.66	0.30	0.04	0.58	0.36	0.06	0.37	0.33	0.30	0.31	0.65	0.04	0.33	0.51	0.15
chr1!	100300223	448	G	1	0.76	0.23	0.00	0.77	0.08	0.16	0.73	0.23	0.04	0.53	0.41	0.06	0.53	0.44	0.03	0.55	0.39	0.05
chr1!	100300307	469	C	1	0.30	0.63	0.07	0.27	0.58	0.15	0.24	0.38	0.37	0.17	0.63	0.19	0.18	0.78	0.03	0.19	0.79	0.02
chr1(	92644709	668	C	0	0.59	0.02	0.39	0.58	0.23	0.19	0.58	0.34	0.08	0.47	0.41	0.13	0.39	0.28	0.33	0.70	0.04	0.26
chr1!	100300380	493	C	1	0.29	0.62	0.09	0.39	0.42	0.19	0.34	0.42	0.24	0.24	0.46	0.30	0.20	0.67	0.13	0.16	0.83	0.01
chr11	3525858	147	C	1	0.40	0.48	0.13	0.42	0.53	0.04	0.39	0.52	0.09	0.23	0.77	0.00	0.24	0.76	0.00	0.20	0.80	0.00
chr1!	61868666	208	C	1	0.32	0.58	0.10	0.27	0.33	0.40	0.26	0.59	0.15	0.15	0.83	0.01	0.15	0.79	0.06	0.14	0.78	0.08
chr1;	45706482	720	G	1	0.27	0.65	0.09	0.20	0.51	0.28	0.18	0.61	0.21	0.13	0.78	0.09	0.13	0.79	0.08	0.11	0.83	0.07
chr9	100498367	1851	G	1	0.50	0.40	0.10	0.49	0.33	0.18	0.49	0.35	0.16	0.41	0.51	0.08	0.38	0.51	0.10	0.38	0.53	0.10
chr1(	92644336	520	G	1	0.34	0.54	0.12	0.40	0.41	0.20	0.37	0.53	0.10	0.27	0.59	0.14	0.26	0.70	0.04	0.27	0.68	0.06
chr1!	61868769	258	C	1	0.31	0.64	0.05	0.30	0.42	0.27	0.27	0.57	0.16	0.12	0.88	0.00	0.14	0.85	0.01	0.14	0.79	0.07

0.66	0.22	0.11	0.42	0.49	0.09	0,69568
0.39	0.51	0.10	0.19	0.78	0.03	0,66833
0.50	0.40	0.10	0.29	0.68	0.03	0,65022
0.36	0.48	0.17	0.19	0.79	0.02	0,62965
0.63	0.34	0.03	0.30	0.67	0.03	0,62571
0.47	0.38	0.15	0.32	0.63	0.05	0,61639
0.33	0.52	0.16	0.16	0.75	0.10	0,61278
0.34	0.48	0.18	0.21	0.73	0.06	0,59422
0.62	0.35	0.03	0.34	0.60	0.06	0,59421
0.34	0.52	0.14	0.14	0.84	0.02	0,57372
0.56	0.38	0.05	0.30	0.58	0.12	0,564
0.27	0.60	0.13	0.14	0.82	0.04	0,56255
0.43	0.46	0.10	0.22	0.75	0.03	0,55712
0.36	0.55	0.09	0.15	0.79	0.06	0,55153
0.50	0.45	0.06	0.22	0.72	0.06	0,53981
0.89	0.05	0.06	0.67	0.30	0.03	0,53366
0.42	0.51	0.07	0.13	0.85	0.02	0,53089
0.79	0.18	0.03	0.55	0.28	0.18	0,52663
0.56	0.39	0.05	0.37	0.49	0.14	0,49419
0.50	0.43	0.07	0.25	0.69	0.06	0,49281
0.18	0.65	0.17	0.13	0.83	0.03	0,49209
0.26	0.64	0.10	0.11	0.88	0.01	0,47208
0.62	0.29	0.10	0.42	0.55	0.03	0,46098
0.36	0.53	0.12	0.17	0.80	0.03	0,46038
0.58	0.40	0.02	0.26	0.73	0.01	0,45316
0.54	0.35	0.11	0.37	0.59	0.04	0,44807
0.48	0.46	0.07	0.25	0.73	0.02	0,43385
0.36	0.58	0.07	0.15	0.83	0.02	0,42992
0.65	0.29	0.06	0.40	0.55	0.04	0,4223
0.49	0.41	0.10	0.28	0.69	0.03	0,41396
0.57	0.39	0.04	0.30	0.68	0.02	0,41382
0.42	0.47	0.10	0.26	0.65	0.09	0,40907
0.91	0.05	0.04	0.72	0.23	0.04	0,40757
0.30	0.59	0.10	0.16	0.83	0.01	0,40149
0.73	0.22	0.06	0.48	0.49	0.03	0,39957
0.64	0.33	0.03	0.35	0.56	0.09	0,38798
0.83	0.14	0.03	0.55	0.43	0.03	0,3878
0.26	0.59	0.15	0.16	0.79	0.05	0,38445
0.73	0.12	0.16	0.59	0.18	0.23	0,3815
0.34	0.53	0.13	0.20	0.74	0.06	0,37983
0.38	0.56	0.06	0.20	0.79	0.01	0,3782
0.24	0.58	0.18	0.12	0.85	0.03	0,3749
0.20	0.65	0.15	0.10	0.84	0.06	0,35416
0.53	0.35	0.12	0.39	0.53	0.07	0,35089
0.40	0.49	0.11	0.23	0.71	0.06	0,34142
0.28	0.61	0.11	0.11	0.87	0.01	0,34057

chr17	45706724	847	G	1	0.46	0.53	0.01	0.42	0.43	0.14	0.40	0.43	0.16	0.23	0.66	0.11	0.20	0.70	0.10	0.23	0.75	0.01
chr16	92644670	657	C	1	0.63	0.29	0.08	0.65	0.11	0.24	0.63	0.24	0.13	0.51	0.46	0.03	0.51	0.24	0.25	0.54	0.32	0.14
chr5	64987445	1462	C	1	0.47	0.44	0.09	0.43	0.51	0.06	0.37	0.39	0.24	0.28	0.62	0.10	0.29	0.59	0.12	0.26	0.71	0.03
chr15	100300308	470	G	1	0.28	0.62	0.10	0.21	0.55	0.25	0.15	0.64	0.21	0.12	0.76	0.12	0.07	0.83	0.10	0.07	0.74	0.19
chr5	64987451	1466	C	1	0.33	0.50	0.17	0.38	0.52	0.10	0.30	0.54	0.16	0.21	0.67	0.11	0.20	0.77	0.02	0.18	0.81	0.00
chr16	92644584	650	C	1	0.57	0.32	0.11	0.63	0.27	0.10	0.65	0.23	0.11	0.48	0.38	0.14	0.44	0.51	0.05	0.48	0.48	0.04
chr15	61868833	289	C	1	0.47	0.49	0.04	0.43	0.37	0.20	0.37	0.52	0.11	0.22	0.77	0.01	0.26	0.69	0.05	0.24	0.75	0.01
chr17	45706709	835	C	1	0.58	0.37	0.05	0.57	0.36	0.08	0.51	0.39	0.10	0.39	0.58	0.02	0.37	0.59	0.04	0.30	0.57	0.12
chr11	3525876	160	C	1	0.63	0.34	0.02	0.62	0.29	0.09	0.64	0.35	0.01	0.38	0.61	0.01	0.38	0.62	0.00	0.36	0.53	0.11
chr15	100300291	461	C	1	0.40	0.54	0.06	0.44	0.48	0.08	0.32	0.52	0.17	0.21	0.34	0.45	0.28	0.63	0.09	0.22	0.74	0.04
chr15	100300191	439	G	1	0.59	0.39	0.02	0.60	0.33	0.07	0.56	0.40	0.04	0.33	0.54	0.13	0.31	0.69	0.00	0.32	0.54	0.14
chr11	3525859	148	G	1	0.39	0.59	0.02	0.42	0.50	0.07	0.38	0.61	0.01	0.19	0.72	0.09	0.20	0.72	0.08	0.20	0.77	0.04
chr15	61868757	251	C	1	0.35	0.59	0.06	0.38	0.40	0.22	0.33	0.52	0.15	0.21	0.78	0.01	0.21	0.77	0.01	0.20	0.76	0.04
chr11	3525756	84	C	1	0.54	0.41	0.05	0.53	0.43	0.04	0.55	0.42	0.03	0.31	0.68	0.01	0.32	0.67	0.00	0.29	0.70	0.01
chr16	92644556	636	G	1	0.56	0.35	0.09	0.56	0.26	0.18	0.59	0.34	0.07	0.43	0.49	0.07	0.43	0.54	0.02	0.41	0.48	0.10
chr11	3525782	101	G	1	0.51	0.45	0.04	0.51	0.43	0.06	0.43	0.56	0.01	0.27	0.60	0.13	0.24	0.75	0.01	0.23	0.75	0.02
chr9	100498548	1948	C	1	0.27	0.50	0.23	0.32	0.59	0.09	0.34	0.50	0.17	0.26	0.72	0.02	0.28	0.44	0.28	0.30	0.67	0.03
chr8	89376823	1579	c	1	0.49	0.49	0.02	0.47	0.46	0.08	0.48	0.43	0.09	0.23	0.75	0.02	0.27	0.71	0.02	0.24	0.76	0.00
chr15	100300086	396	C	1	0.56	0.42	0.03	0.60	0.31	0.09	0.58	0.29	0.13	0.38	0.51	0.11	0.45	0.48	0.07	0.42	0.30	0.29
chr16	92644502	603	C	1	0.47	0.47	0.07	0.50	0.37	0.12	0.56	0.32	0.12	0.34	0.63	0.02	0.37	0.38	0.25	0.40	0.59	0.02
chr15	61868812	277	C	1	0.36	0.61	0.03	0.34	0.50	0.16	0.30	0.49	0.21	0.17	0.82	0.00	0.23	0.76	0.01	0.19	0.77	0.04
chr9	100498451	1894	G	1	0.38	0.46	0.16	0.38	0.48	0.14	0.36	0.48	0.16	0.32	0.55	0.12	0.29	0.63	0.09	0.29	0.60	0.11
chr5	64987368	1430	G	1	0.71	0.24	0.05	0.64	0.30	0.06	0.65	0.24	0.11	0.45	0.38	0.16	0.50	0.49	0.01	0.49	0.50	0.01
chr16	92644533	623	C	1	0.47	0.38	0.14	0.56	0.39	0.05	0.59	0.35	0.06	0.33	0.65	0.02	0.39	0.52	0.09	0.39	0.55	0.06
chr4	139783699	1286	C	1	0.20	0.67	0.13	0.21	0.66	0.13	0.20	0.68	0.12	0.11	0.82	0.07	0.10	0.86	0.03	0.12	0.86	0.02
chr5	64987448	1464	C	1	0.38	0.53	0.09	0.38	0.53	0.09	0.30	0.51	0.19	0.27	0.62	0.11	0.25	0.70	0.05	0.21	0.74	0.06
chr11	3525894	171	G	1	0.47	0.52	0.01	0.51	0.36	0.14	0.44	0.55	0.01	0.27	0.61	0.12	0.26	0.72	0.02	0.21	0.76	0.02
chr17	45706632	803	G	1	0.44	0.48	0.08	0.37	0.52	0.11	0.32	0.53	0.15	0.27	0.67	0.06	0.20	0.77	0.03	0.28	0.65	0.07
chr15	61868813	278	G	1	0.39	0.52	0.09	0.40	0.52	0.09	0.39	0.52	0.10	0.27	0.68	0.05	0.21	0.79	0.00	0.24	0.75	0.01
chr5	64987378	1433	G	1	0.71	0.25	0.04	0.68	0.25	0.07	0.66	0.22	0.11	0.51	0.37	0.12	0.53	0.45	0.01	0.52	0.42	0.06
chr15	61868733	241	C	1	0.36	0.56	0.08	0.41	0.40	0.19	0.35	0.56	0.09	0.27	0.72	0.01	0.23	0.76	0.00	0.23	0.68	0.09
chr16	92644537	626	C	1	0.50	0.44	0.05	0.51	0.41	0.08	0.53	0.37	0.10	0.31	0.67	0.02	0.32	0.67	0.02	0.41	0.56	0.03
chr15	100300087	397	G	1	0.61	0.37	0.02	0.62	0.32	0.07	0.54	0.38	0.07	0.39	0.42	0.20	0.36	0.63	0.01	0.37	0.47	0.16
chr5	64987419	1446	C	1	0.46	0.48	0.07	0.44	0.52	0.03	0.41	0.50	0.10	0.30	0.55	0.15	0.30	0.62	0.09	0.25	0.67	0.08
chr15	100300145	424	C	1	0.25	0.67	0.08	0.28	0.62	0.10	0.36	0.57	0.07	0.20	0.55	0.25	0.23	0.69	0.08	0.18	0.65	0.16
chr17	45706720	843	C	1	0.36	0.55	0.09	0.37	0.58	0.05	0.32	0.51	0.16	0.23	0.69	0.08	0.22	0.78	0.01	0.21	0.77	0.02
chr11	3525791	107	G	1	0.44	0.55	0.01	0.48	0.42	0.10	0.42	0.57	0.01	0.19	0.81	0.00	0.22	0.77	0.01	0.21	0.69	0.11
chr11	3525680	44	C	1	0.31	0.62	0.07	0.31	0.39	0.30	0.34	0.63	0.03	0.22	0.78	0.01	0.21	0.70	0.08	0.20	0.74	0.07
chr16	92644506	606	C	1	0.27	0.64	0.09	0.32	0.48	0.20	0.36	0.55	0.09	0.21	0.71	0.08	0.18	0.80	0.02	0.24	0.73	0.03
chr16	92644484	590	C	1	0.47	0.44	0.09	0.49	0.37	0.14	0.51	0.44	0.05	0.31	0.66	0.02	0.36	0.60	0.05	0.40	0.56	0.04
chr16	92644510	610	G	1	0.45	0.51	0.04	0.49	0.34	0.17	0.46	0.44	0.10	0.34	0.57	0.09	0.33	0.62	0.05	0.30	0.61	0.10
chr5	64987420	1447	G	1	0.42	0.50	0.08	0.35	0.57	0.08	0.34	0.58	0.08	0.21	0.73	0.06	0.24	0.75	0.00	0.22	0.77	0.01
chr15	61868789	268	C	1	0.19	0.73	0.08	0.20	0.69	0.11	0.16	0.68	0.16	0.11	0.88	0.01	0.10	0.85	0.05	0.09	0.83	0.09
chr11	3525891	169	G	1	0.75	0.24	0.01	0.77	0.09	0.13	0.72	0.27	0.01	0.52	0.47	0.01	0.55	0.45	0.01	0.58	0.41	0.01
chr16	92644534	624	G	1	0.52	0.44	0.04	0.50	0.36	0.15	0.54	0.36	0.10	0.39	0.55	0.06	0.42	0.56	0.02	0.40	0.56	0.04
chr16	92644456	569	G	1	0.64	0.34	0.02	0.63	0.28	0.10	0.64	0.29	0.07	0.45	0.49	0.06	0.46	0.52	0.02	0.45	0.42	0.13

0.45	0.50	0.05	0.20	0.76	0.04	0,33801
0.74	0.16	0.10	0.59	0.32	0.09	0,33719
0.46	0.47	0.07	0.26	0.68	0.06	0,33306
0.19	0.67	0.15	0.07	0.83	0.11	0,31876
0.34	0.54	0.11	0.18	0.80	0.02	0,3133
0.67	0.24	0.08	0.49	0.46	0.05	0,31281
0.46	0.47	0.07	0.23	0.76	0.01	0,31064
0.58	0.37	0.05	0.36	0.60	0.04	0,3084
0.66	0.31	0.03	0.37	0.62	0.01	0,29589
0.39	0.54	0.07	0.25	0.64	0.11	0,29477
0.59	0.38	0.03	0.32	0.65	0.04	0,29273
0.40	0.58	0.02	0.17	0.78	0.05	0,28594
0.37	0.53	0.10	0.19	0.79	0.02	0,2768
0.56	0.42	0.03	0.29	0.70	0.01	0,27675
0.63	0.28	0.09	0.43	0.52	0.05	0,27562
0.50	0.47	0.03	0.23	0.75	0.02	0,26574
0.29	0.56	0.15	0.30	0.65	0.05	0,2636
0.49	0.47	0.04	0.22	0.77	0.01	0,26295
0.61	0.34	0.05	0.42	0.46	0.11	0,26075
0.48	0.43	0.09	0.38	0.58	0.04	0,26004
0.35	0.57	0.09	0.18	0.81	0.01	0,25545
0.37	0.49	0.13	0.28	0.61	0.11	0,25502
0.69	0.26	0.05	0.50	0.48	0.03	0,24733
0.57	0.37	0.06	0.36	0.60	0.04	0,24622
0.18	0.72	0.10	0.08	0.89	0.03	0,2402
0.35	0.55	0.09	0.22	0.73	0.05	0,23954
0.50	0.47	0.03	0.23	0.74	0.03	0,23727
0.37	0.55	0.08	0.22	0.75	0.03	0,23494
0.39	0.54	0.07	0.22	0.77	0.01	0,23282
0.73	0.22	0.05	0.54	0.42	0.04	0,23139
0.38	0.53	0.09	0.23	0.75	0.02	0,22301
0.55	0.39	0.06	0.33	0.64	0.02	0,21832
0.61	0.35	0.04	0.39	0.55	0.06	0,21734
0.42	0.53	0.05	0.26	0.67	0.07	0,217
0.28	0.66	0.06	0.19	0.68	0.12	0,21612
0.36	0.57	0.07	0.20	0.78	0.02	0,20938
0.45	0.53	0.02	0.18	0.80	0.02	0,20674
0.32	0.59	0.09	0.19	0.79	0.03	0,20662
0.33	0.57	0.10	0.18	0.79	0.03	0,20621
0.51	0.42	0.07	0.34	0.63	0.03	0,20331
0.48	0.45	0.07	0.31	0.63	0.05	0,20264
0.38	0.57	0.05	0.20	0.78	0.02	0,19977
0.17	0.73	0.10	0.08	0.89	0.03	0,19898
0.81	0.16	0.03	0.56	0.43	0.01	0,1984
0.55	0.39	0.07	0.39	0.60	0.01	0,19745
0.67	0.28	0.05	0.47	0.49	0.04	0,1966

chr11	3525675	42	G	1	0.53	0.45	0.02	0.51	0.32	0.16	0.48	0.49	0.04	0.29	0.67	0.04	0.31	0.68	0.01	0.30	0.61	0.09
chr15	100300067	387	C	1	0.88	0.06	0.06	0.83	0.10	0.07	0.84	0.11	0.05	0.62	0.20	0.18	0.69	0.24	0.07	0.70	0.20	0.10
chr9	100498576	1964	C	1	0.21	0.68	0.11	0.20	0.67	0.13	0.20	0.70	0.10	0.18	0.82	0.01	0.19	0.75	0.06	0.19	0.76	0.05
chr5	64987423	1450	G	1	0.19	0.73	0.08	0.15	0.68	0.17	0.17	0.68	0.15	0.13	0.84	0.03	0.13	0.75	0.11	0.14	0.85	0.01
chr16	92644507	607	G	1	0.26	0.62	0.12	0.29	0.51	0.20	0.31	0.59	0.10	0.22	0.69	0.09	0.22	0.73	0.05	0.20	0.70	0.10
chr17	45706710	836	G	1	0.53	0.47	0.00	0.55	0.42	0.02	0.52	0.40	0.08	0.38	0.52	0.11	0.38	0.44	0.19	0.38	0.57	0.04
chr9	100498549	1949	G	1	0.37	0.51	0.12	0.30	0.59	0.11	0.28	0.59	0.13	0.36	0.55	0.09	0.22	0.66	0.12	0.23	0.68	0.09
chr17	45706481	719	C	1	0.37	0.50	0.12	0.34	0.62	0.04	0.36	0.56	0.09	0.23	0.57	0.21	0.23	0.71	0.06	0.19	0.80	0.00
chr16	92644604	654	C	1	0.60	0.35	0.06	0.65	0.29	0.07	0.68	0.29	0.04	0.45	0.37	0.18	0.51	0.44	0.04	0.42	0.52	0.05
chr11	3525842	135	C	1	0.44	0.39	0.17	0.44	0.52	0.03	0.45	0.54	0.01	0.28	0.72	0.00	0.27	0.73	0.00	0.28	0.71	0.01
chr17	45706631	802	C	1	0.43	0.52	0.05	0.42	0.50	0.08	0.38	0.48	0.14	0.30	0.69	0.01	0.27	0.62	0.11	0.30	0.61	0.10
chr5	64987525	1500	C	1	0.43	0.53	0.04	0.40	0.49	0.11	0.32	0.44	0.24	0.31	0.66	0.03	0.32	0.66	0.02	0.30	0.69	0.02
chr5	64987422	1449	C	1	0.44	0.51	0.06	0.45	0.49	0.06	0.39	0.47	0.13	0.36	0.53	0.11	0.33	0.55	0.13	0.29	0.66	0.05
chr11	3525868	156	G	1	0.43	0.55	0.02	0.44	0.53	0.03	0.37	0.62	0.01	0.17	0.82	0.01	0.23	0.75	0.01	0.17	0.73	0.10
chr17	45706601	789	G	1	0.40	0.58	0.02	0.49	0.48	0.03	0.38	0.57	0.05	0.25	0.70	0.05	0.24	0.67	0.09	0.26	0.65	0.09
chr5	64987454	1468	C	1	0.20	0.63	0.18	0.21	0.70	0.09	0.15	0.57	0.28	0.15	0.81	0.04	0.13	0.78	0.09	0.12	0.81	0.06
chr19	24277496	908	c	1	0.38	0.53	0.09	0.39	0.47	0.14	0.42	0.46	0.12	0.40	0.59	0.01	0.45	0.53	0.02	0.44	0.46	0.10
chr5	64987446	1463	G	1	0.33	0.61	0.06	0.43	0.46	0.11	0.38	0.51	0.12	0.23	0.70	0.06	0.28	0.68	0.04	0.27	0.67	0.06
chr9	100498415	1875	C	1	0.26	0.65	0.09	0.23	0.57	0.20	0.22	0.70	0.08	0.23	0.77	0.01	0.22	0.61	0.16	0.22	0.70	0.09
chr11	3525643	27	C	1	0.45	0.55	0.00	0.45	0.27	0.27	0.43	0.46	0.11	0.31	0.55	0.14	0.29	0.53	0.18	0.25	0.75	0.00
chr9	100498490	1917	C	1	0.20	0.70	0.10	0.22	0.56	0.21	0.23	0.70	0.07	0.19	0.81	0.00	0.21	0.72	0.07	0.21	0.74	0.05
chr15	100300062	384	C	1	0.68	0.30	0.02	0.70	0.22	0.08	0.68	0.28	0.04	0.40	0.49	0.11	0.52	0.46	0.01	0.47	0.21	0.32
chr9	100498506	1927	G	1	0.28	0.59	0.14	0.29	0.65	0.07	0.28	0.55	0.17	0.27	0.62	0.11	0.25	0.65	0.10	0.24	0.70	0.06
chr5	64987465	1473	C	1	0.19	0.65	0.15	0.21	0.74	0.06	0.14	0.58	0.28	0.17	0.67	0.17	0.12	0.75	0.13	0.12	0.80	0.08
chr15	61868811	276	G	1	0.31	0.66	0.04	0.26	0.68	0.07	0.27	0.64	0.10	0.14	0.77	0.08	0.12	0.88	0.00	0.13	0.87	0.00
chr17	45706509	735	G	1	0.58	0.42	0.01	0.64	0.32	0.04	0.50	0.38	0.13	0.37	0.59	0.04	0.37	0.62	0.00	0.38	0.62	0.00
chr8	120115794	1805	C	1	0.18	0.70	0.11	0.17	0.64	0.19	0.16	0.75	0.10	0.15	0.84	0.01	0.09	0.78	0.13	0.12	0.80	0.09
chr15	100300146	425	G	1	0.29	0.68	0.03	0.39	0.53	0.08	0.37	0.60	0.03	0.24	0.55	0.21	0.25	0.72	0.03	0.20	0.68	0.12
chr11	3525669	38	G	1	0.37	0.59	0.04	0.40	0.53	0.07	0.35	0.63	0.02	0.19	0.75	0.06	0.22	0.76	0.01	0.19	0.73	0.08
chr15	61868810	275	C	1	0.23	0.71	0.07	0.21	0.52	0.27	0.18	0.75	0.07	0.10	0.90	0.00	0.11	0.83	0.07	0.10	0.76	0.14
chr5	64987367	1429	C	1	0.71	0.27	0.01	0.74	0.21	0.05	0.70	0.17	0.12	0.55	0.21	0.24	0.55	0.44	0.01	0.50	0.32	0.18
chr5	64987449	1465	G	1	0.32	0.59	0.10	0.36	0.54	0.11	0.30	0.65	0.05	0.16	0.75	0.09	0.26	0.70	0.04	0.21	0.76	0.04
chr11	3525717	63	G	1	0.39	0.52	0.09	0.42	0.42	0.17	0.34	0.65	0.01	0.25	0.74	0.01	0.24	0.74	0.02	0.23	0.76	0.01
chr11	3525674	41	C	1	0.52	0.48	0.00	0.53	0.30	0.17	0.52	0.41	0.07	0.34	0.54	0.12	0.34	0.66	0.00	0.34	0.60	0.06
chr9	100498491	1918	G	1	0.21	0.67	0.12	0.20	0.74	0.07	0.19	0.69	0.12	0.20	0.68	0.12	0.16	0.68	0.16	0.18	0.75	0.07
chr16	92644467	577	G	1	0.37	0.54	0.09	0.39	0.49	0.12	0.39	0.53	0.07	0.32	0.64	0.04	0.32	0.65	0.03	0.30	0.57	0.13
chr11	3525823	125	C	1	0.37	0.60	0.03	0.40	0.51	0.09	0.37	0.57	0.06	0.24	0.56	0.20	0.27	0.68	0.05	0.24	0.76	0.00
chr16	92644485	591	G	1	0.43	0.49	0.07	0.47	0.43	0.11	0.43	0.54	0.02	0.33	0.63	0.04	0.29	0.69	0.02	0.32	0.61	0.07
chr5	64987377	1432	C	1	0.73	0.27	0.00	0.72	0.22	0.06	0.68	0.17	0.15	0.61	0.33	0.06	0.59	0.36	0.05	0.56	0.34	0.10
chr15	61868790	269	G	1	0.24	0.73	0.03	0.23	0.66	0.11	0.23	0.70	0.07	0.13	0.85	0.02	0.10	0.89	0.01	0.10	0.85	0.06
chr11	3525781	100	C	1	0.48	0.51	0.01	0.50	0.42	0.08	0.50	0.49	0.01	0.29	0.70	0.01	0.31	0.63	0.07	0.26	0.72	0.02
chr17	45706661	819	G	1	0.25	0.72	0.03	0.29	0.57	0.14	0.24	0.69	0.07	0.13	0.83	0.04	0.13	0.84	0.03	0.17	0.72	0.11
chr5	64987466	1474	G	1	0.26	0.61	0.13	0.27	0.64	0.09	0.29	0.61	0.11	0.24	0.73	0.03	0.31	0.67	0.02	0.27	0.71	0.02
chr11	3525843	136	G	1	0.39	0.59	0.01	0.40	0.36	0.24	0.35	0.63	0.01	0.21	0.77	0.02	0.23	0.76	0.01	0.22	0.77	0.02
chr17	45706660	818	C	1	0.19	0.69	0.12	0.19	0.72	0.09	0.19	0.68	0.13	0.16	0.81	0.03	0.15	0.73	0.12	0.14	0.82	0.04
chr11	3525743	79	G	1	0.27	0.72	0.01	0.28	0.49	0.24	0.22	0.69	0.10	0.13	0.84	0.03	0.22	0.73	0.05	0.15	0.75	0.10

0.53	0.44	0.03	0.31	0.67	0.03	0,19659
0.90	0.06	0.04	0.72	0.19	0.09	0,1926
0.18	0.73	0.10	0.16	0.82	0.02	0,19184
0.16	0.72	0.12	0.11	0.87	0.02	0,19163
0.27	0.62	0.11	0.19	0.75	0.06	0,1915
0.55	0.43	0.02	0.41	0.51	0.08	0,18835
0.30	0.59	0.11	0.24	0.67	0.09	0,18729
0.36	0.58	0.06	0.20	0.76	0.04	0,18503
0.66	0.29	0.05	0.48	0.47	0.06	0,185
0.47	0.50	0.04	0.25	0.75	0.01	0,18324
0.40	0.52	0.07	0.28	0.68	0.04	0,18299
0.39	0.52	0.09	0.29	0.69	0.02	0,18011
0.43	0.50	0.06	0.30	0.62	0.07	0,17764
0.41	0.58	0.01	0.16	0.82	0.02	0,17164
0.42	0.56	0.03	0.22	0.72	0.06	0,17156
0.17	0.70	0.13	0.11	0.84	0.05	0,16829
0.40	0.50	0.10	0.43	0.55	0.02	0,16818
0.37	0.56	0.07	0.24	0.72	0.04	0,16795
0.20	0.71	0.10	0.21	0.75	0.05	0,16206
0.47	0.48	0.05	0.28	0.69	0.03	0,16068
0.20	0.71	0.10	0.18	0.80	0.02	0,15638
0.75	0.22	0.03	0.53	0.39	0.07	0,15331
0.26	0.65	0.09	0.23	0.68	0.08	0,1532
0.16	0.73	0.11	0.11	0.79	0.10	0,14655
0.24	0.70	0.06	0.11	0.88	0.01	0,14176
0.60	0.37	0.03	0.38	0.61	0.01	0,14
0.16	0.73	0.11	0.10	0.86	0.04	0,13999
0.34	0.63	0.03	0.22	0.71	0.07	0,13684
0.37	0.61	0.03	0.18	0.79	0.03	0,13542
0.19	0.72	0.09	0.08	0.89	0.03	0,13501
0.76	0.21	0.03	0.61	0.31	0.07	0,13292
0.32	0.62	0.06	0.18	0.78	0.04	0,13193
0.45	0.51	0.04	0.22	0.77	0.01	0,13094
0.57	0.40	0.04	0.33	0.65	0.02	0,12658
0.17	0.74	0.09	0.16	0.74	0.09	0,12508
0.39	0.54	0.07	0.30	0.66	0.04	0,12393
0.37	0.58	0.05	0.23	0.74	0.03	0,12254
0.44	0.51	0.05	0.30	0.67	0.03	0,1222
0.79	0.19	0.03	0.62	0.33	0.05	0,12207
0.22	0.73	0.05	0.09	0.90	0.02	0,12113
0.50	0.48	0.02	0.27	0.71	0.02	0,12109
0.25	0.70	0.05	0.11	0.85	0.04	0,11885
0.24	0.67	0.09	0.25	0.73	0.02	0,1161
0.41	0.56	0.04	0.20	0.79	0.01	0,11494
0.17	0.74	0.09	0.13	0.84	0.03	0,11338
0.26	0.68	0.06	0.14	0.82	0.04	0,11282

chr16	92644466	576	C		1	0.40	0.58	0.03	0.46	0.42	0.12	0.41	0.50	0.10	0.29	0.70	0.01	0.22	0.77	0.01	0.38	0.60	0.03
chr11	3525668	37	C		1	0.37	0.63	0.00	0.36	0.37	0.26	0.36	0.58	0.07	0.24	0.71	0.05	0.23	0.76	0.01	0.21	0.67	0.12
chr9	100498421	1877	C		1	0.20	0.74	0.06	0.19	0.54	0.27	0.20	0.71	0.09	0.18	0.76	0.06	0.22	0.73	0.05	0.19	0.73	0.08
chr9	100498416	1876	G		1	0.27	0.60	0.13	0.26	0.69	0.05	0.25	0.61	0.14	0.26	0.70	0.04	0.22	0.74	0.04	0.23	0.74	0.04
chr11	3525821	123	C		1	0.37	0.61	0.02	0.37	0.55	0.07	0.36	0.62	0.02	0.22	0.71	0.06	0.23	0.70	0.06	0.21	0.78	0.01
chr9	100498589	1968	C		1	0.29	0.63	0.08	0.31	0.44	0.25	0.32	0.65	0.03	0.23	0.77	0.00	0.27	0.58	0.15	0.28	0.71	0.01
chr9	100498422	1878	G		1	0.24	0.68	0.09	0.20	0.68	0.12	0.20	0.69	0.11	0.22	0.71	0.06	0.21	0.78	0.02	0.20	0.73	0.08
chr17	45706411	697	C		1	0.27	0.68	0.04	0.24	0.57	0.19	0.13	0.68	0.19	0.21	0.78	0.01	0.16	0.71	0.12	0.17	0.74	0.09
chr15	100300345	484	C		1	0.16	0.78	0.05	0.17	0.69	0.14	0.21	0.68	0.10	0.11	0.58	0.31	0.10	0.87	0.03	0.09	0.88	0.03
chr11	3525824	126	G		1	0.38	0.60	0.02	0.37	0.57	0.06	0.35	0.64	0.02	0.19	0.78	0.03	0.22	0.77	0.01	0.19	0.80	0.01
chr9	100498505	1926	C		1	0.32	0.53	0.15	0.35	0.58	0.07	0.35	0.59	0.06	0.32	0.67	0.01	0.30	0.67	0.02	0.32	0.64	0.04
chr4	139783638	1251	C		1	0.34	0.53	0.12	0.33	0.66	0.01	0.34	0.53	0.13	0.19	0.80	0.00	0.24	0.75	0.01	0.25	0.70	0.05
chr8	89376858	1600	g		1	0.26	0.73	0.01	0.28	0.70	0.02	0.22	0.73	0.05	0.12	0.84	0.04	0.18	0.78	0.04	0.14	0.75	0.11
chr8	120115795	1806	G		1	0.30	0.64	0.06	0.31	0.62	0.07	0.34	0.62	0.04	0.25	0.74	0.01	0.25	0.71	0.04	0.28	0.70	0.02
chrX	7539502	2335	C		1	0.21	0.63	0.16	0.22	0.68	0.10	0.30	0.65	0.04	0.27	0.62	0.11	0.27	0.68	0.05	0.25	0.74	0.01
chr11	3525790	106	C		1	0.48	0.52	0.00	0.46	0.41	0.13	0.48	0.51	0.01	0.29	0.70	0.01	0.30	0.69	0.00	0.27	0.68	0.05
chr16	92644386	538	G		1	0.51	0.46	0.04	0.48	0.28	0.24	0.51	0.47	0.02	0.39	0.58	0.03	0.41	0.57	0.02	0.39	0.52	0.09
chr11	3525681	45	G		1	0.34	0.65	0.01	0.31	0.62	0.08	0.29	0.63	0.07	0.16	0.83	0.01	0.22	0.75	0.03	0.18	0.63	0.19
chr11	3525857	146	G		1	0.28	0.70	0.03	0.24	0.62	0.15	0.25	0.74	0.01	0.12	0.85	0.03	0.14	0.82	0.04	0.14	0.76	0.11
chr17	45706519	739	C		1	0.23	0.75	0.02	0.21	0.73	0.06	0.22	0.67	0.11	0.17	0.73	0.10	0.19	0.79	0.02	0.16	0.77	0.07
chr5	64987452	1467	G		1	0.27	0.69	0.04	0.32	0.61	0.07	0.23	0.72	0.05	0.16	0.81	0.03	0.19	0.73	0.08	0.16	0.81	0.03
chr16	92644716	672	C		0	0.71	0.28	0.02	0.73	0.20	0.07	0.86	0.03	0.11	0.84	0.02	0.15	0.86	0.01	0.13	0.83	0.01	0.16
chr15	100300068	388	G		1	0.87	0.10	0.03	0.85	0.12	0.03	0.84	0.10	0.06	0.68	0.32	0.00	0.68	0.23	0.09	0.65	0.14	0.21
chr15	61868649	201	C		1	0.19	0.75	0.05	0.17	0.67	0.16	0.14	0.76	0.10	0.10	0.89	0.00	0.09	0.90	0.01	0.07	0.85	0.07
chrX	7539418	2283	G		1	0.20	0.73	0.07	0.18	0.73	0.10	0.15	0.76	0.09	0.11	0.72	0.18	0.11	0.84	0.05	0.13	0.83	0.04
chr8	120115796	1807	C		1	0.13	0.76	0.11	0.14	0.75	0.11	0.11	0.80	0.09	0.14	0.83	0.03	0.06	0.87	0.07	0.06	0.87	0.07
chr8	89376926	1634	C		1	0.16	0.72	0.11	0.13	0.75	0.12	0.18	0.76	0.06	0.11	0.86	0.03	0.11	0.79	0.11	0.13	0.86	0.01
chr5	64987555	1515	C		1	0.28	0.70	0.03	0.27	0.65	0.08	0.22	0.65	0.13	0.24	0.72	0.04	0.23	0.61	0.16	0.19	0.80	0.01
chr16	92644707	667	C		0	0.75	0.24	0.01	0.61	0.20	0.19	0.85	0.08	0.07	0.73	0.17	0.10	0.69	0.29	0.01	0.72	0.12	0.16
chr15	100300050	376	C		1	0.96	0.01	0.04	0.94	0.02	0.04	0.86	0.12	0.02	0.76	0.16	0.08	0.70	0.29	0.01	0.80	0.19	0.01
chr15	100300026	365	C		1	0.87	0.02	0.11	0.90	0.04	0.06	0.85	0.08	0.08	0.76	0.10	0.14	0.79	0.18	0.03	0.74	0.17	0.09
chr11	3525890	168	C		1	0.72	0.21	0.07	0.74	0.26	0.00	0.75	0.25	0.00	0.60	0.37	0.03	0.60	0.40	0.00	0.57	0.36	0.07
chr19	24277497	909	g		1	0.40	0.54	0.05	0.37	0.55	0.09	0.39	0.59	0.03	0.37	0.60	0.03	0.34	0.51	0.15	0.36	0.61	0.03
chr15	100300353	487	C		1	0.15	0.80	0.05	0.14	0.79	0.07	0.13	0.62	0.26	0.09	0.87	0.03	0.08	0.92	0.01	0.07	0.74	0.19
chr8	89376857	1599	c		1	0.19	0.76	0.05	0.14	0.66	0.19	0.20	0.76	0.05	0.13	0.85	0.01	0.12	0.84	0.04	0.19	0.78	0.03
chr15	100300051	377	G		1	0.94	0.06	0.00	0.93	0.04	0.03	0.91	0.07	0.01	0.79	0.13	0.08	0.77	0.19	0.04	0.76	0.15	0.10
chr17	45706721	844	G		1	0.39	0.61	0.00	0.36	0.54	0.11	0.35	0.56	0.08	0.25	0.68	0.07	0.26	0.74	0.00	0.26	0.72	0.02
chr11	3525822	124	G		1	0.32	0.67	0.01	0.36	0.58	0.06	0.33	0.66	0.01	0.12	0.88	0.00	0.18	0.81	0.01	0.19	0.80	0.01
chr8	120115659	1743	C		1	0.07	0.80	0.13	0.10	0.77	0.12	0.12	0.80	0.08	0.10	0.89	0.01	0.09	0.90	0.02	0.08	0.89	0.03
chr8	89376927	1635	G		1	0.20	0.71	0.08	0.21	0.68	0.11	0.19	0.77	0.04	0.11	0.87	0.01	0.15	0.85	0.00	0.13	0.82	0.05
chr11	3525716	62	C		1	0.46	0.53	0.00	0.47	0.38	0.15	0.49	0.48	0.02	0.35	0.65	0.00	0.37	0.63	0.00	0.34	0.63	0.03
chr8	89376976	1655	C		1	0.28	0.65	0.07	0.26	0.69	0.05	0.30	0.67	0.03	0.24	0.72	0.04	0.22	0.73	0.06	0.23	0.76	0.00
chr8	120115764	1792	C		1	0.28	0.67	0.05	0.28	0.66	0.06	0.25	0.64	0.11	0.26	0.72	0.01	0.21	0.78	0.01	0.27	0.69	0.04
chr5	64987470	1478	G		1	0.10	0.80	0.10	0.14	0.80	0.06	0.22	0.76	0.02	0.17	0.81	0.02	0.23	0.76	0.01	0.23	0.72	0.05
chr19	24277596	959	g		1	0.09	0.77	0.14	0.11	0.81	0.08	0.09	0.82	0.09	0.10	0.88	0.01	0.08	0.88	0.04	0.10	0.90	0.00
chr8	120115745	1784	C		1	0.14	0.79	0.07	0.16	0.78	0.07	0.11	0.76	0.13	0.14	0.77	0.08	0.11	0.86	0.03	0.12	0.85	0.03

0.44	0.51	0.05	0.27	0.71	0.02	0,11275
0.37	0.59	0.04	0.21	0.75	0.03	0,11258
0.19	0.73	0.09	0.17	0.78	0.05	0,11128
0.24	0.68	0.08	0.22	0.76	0.03	0,10616
0.36	0.62	0.03	0.20	0.77	0.03	0,1023
0.32	0.61	0.07	0.26	0.72	0.02	0,10034
0.20	0.72	0.08	0.19	0.78	0.04	0,09774
0.20	0.73	0.07	0.13	0.83	0.04	0,09556
0.16	0.78	0.06	0.08	0.86	0.05	0,09287
0.35	0.63	0.02	0.18	0.81	0.01	0,09244
0.34	0.60	0.07	0.29	0.69	0.02	0,09203
0.32	0.63	0.04	0.21	0.78	0.01	0,08683
0.26	0.73	0.02	0.12	0.84	0.04	0,08482
0.30	0.65	0.05	0.24	0.75	0.02	0,08447
0.23	0.70	0.06	0.25	0.72	0.03	0,08272
0.48	0.50	0.02	0.27	0.71	0.01	0,08255
0.56	0.39	0.04	0.40	0.58	0.02	0,08192
0.30	0.67	0.03	0.17	0.81	0.03	0,0786
0.25	0.73	0.03	0.12	0.84	0.04	0,07264
0.19	0.76	0.04	0.13	0.84	0.04	0,07096
0.27	0.70	0.04	0.15	0.82	0.03	0,07028
0.85	0.10	0.05	0.87	0.02	0.11	0,07009
0.89	0.08	0.03	0.74	0.23	0.03	0,06993
0.16	0.78	0.06	0.07	0.92	0.02	0,06918
0.14	0.80	0.06	0.09	0.87	0.04	0,06906
0.09	0.82	0.09	0.06	0.90	0.04	0,06897
0.12	0.80	0.07	0.09	0.88	0.02	0,06743
0.24	0.71	0.05	0.21	0.75	0.04	0,06729
0.80	0.15	0.05	0.79	0.16	0.05	0,06521
0.95	0.02	0.03	0.80	0.18	0.02	0,06504
0.92	0.03	0.05	0.82	0.12	0.05	0,06301
0.78	0.21	0.01	0.60	0.38	0.02	0,06167
0.40	0.57	0.04	0.36	0.60	0.04	0,06097
0.12	0.81	0.07	0.06	0.91	0.03	0,06079
0.14	0.79	0.07	0.12	0.86	0.02	0,06047
0.95	0.03	0.01	0.82	0.12	0.05	0,05946
0.36	0.61	0.03	0.24	0.74	0.02	0,05702
0.32	0.66	0.02	0.13	0.86	0.01	0,05689
0.07	0.84	0.09	0.06	0.92	0.02	0,05559
0.18	0.76	0.05	0.11	0.88	0.01	0,05503
0.50	0.47	0.02	0.34	0.64	0.01	0,05466
0.21	0.75	0.04	0.21	0.76	0.02	0,05449
0.25	0.70	0.05	0.22	0.76	0.02	0,05291
0.12	0.84	0.04	0.23	0.68	0.08	0,05218
0.07	0.85	0.08	0.07	0.92	0.01	0,05181
0.11	0.83	0.06	0.10	0.88	0.02	0,04819

chr8	120115765	1793	G	1	0.22	0.78	0.00	0.26	0.73	0.01	0.24	0.69	0.07	0.19	0.74	0.07	0.21	0.73	0.06	0.20	0.76	0.04
chrX	7539417	2282	C	1	0.07	0.84	0.10	0.10	0.73	0.17	0.12	0.83	0.05	0.09	0.85	0.06	0.09	0.83	0.07	0.12	0.82	0.06
chr5	64987455	1469	G	1	0.21	0.75	0.04	0.20	0.75	0.06	0.21	0.73	0.05	0.14	0.81	0.04	0.14	0.81	0.05	0.12	0.85	0.03
chr1(	92644348	524	G	1	0.14	0.82	0.04	0.15	0.76	0.09	0.17	0.75	0.08	0.17	0.80	0.03	0.15	0.82	0.03	0.14	0.74	0.12
chrX	7539406	2276	G	1	0.09	0.75	0.16	0.06	0.81	0.13	0.14	0.78	0.08	0.06	0.85	0.09	0.16	0.81	0.04	0.06	0.89	0.06
chr1(	24277537	931	c	1	0.11	0.77	0.12	0.10	0.84	0.07	0.15	0.78	0.07	0.15	0.77	0.08	0.10	0.86	0.04	0.12	0.85	0.02
chr9	100498450	1893	C	1	0.42	0.49	0.09	0.43	0.54	0.02	0.40	0.59	0.00	0.31	0.69	0.00	0.32	0.66	0.01	0.32	0.63	0.06
chr1;	45706520	740	G	1	0.16	0.81	0.03	0.20	0.75	0.06	0.16	0.81	0.04	0.13	0.75	0.12	0.13	0.86	0.01	0.14	0.82	0.04
chr5	64987469	1477	C	1	0.16	0.80	0.04	0.15	0.80	0.05	0.12	0.81	0.07	0.13	0.83	0.04	0.13	0.77	0.11	0.10	0.84	0.06
chr1'	3525855	144	G	1	0.19	0.75	0.06	0.20	0.75	0.06	0.17	0.82	0.01	0.11	0.83	0.06	0.16	0.81	0.03	0.11	0.83	0.06
chr1(	24277535	930	g	1	0.12	0.81	0.07	0.13	0.79	0.08	0.13	0.82	0.05	0.11	0.88	0.01	0.12	0.88	0.01	0.12	0.88	0.00
chr1(	24277550	937	c	1	0.09	0.78	0.13	0.11	0.79	0.10	0.08	0.83	0.09	0.09	0.85	0.07	0.07	0.91	0.02	0.10	0.89	0.01
chr5	64987526	1501	G	1	0.43	0.55	0.02	0.43	0.52	0.05	0.41	0.54	0.05	0.36	0.51	0.13	0.36	0.62	0.02	0.40	0.60	0.01
chrX	7539481	2325	C	1	0.10	0.83	0.07	0.10	0.72	0.18	0.09	0.87	0.04	0.09	0.86	0.06	0.08	0.91	0.01	0.12	0.80	0.08
chr1'	3525854	143	C	1	0.17	0.75	0.08	0.19	0.69	0.12	0.19	0.80	0.01	0.11	0.89	0.00	0.14	0.86	0.01	0.10	0.79	0.11
chr1'	3525742	78	C	1	0.22	0.66	0.12	0.25	0.69	0.05	0.23	0.76	0.01	0.17	0.83	0.00	0.16	0.84	0.00	0.15	0.84	0.01
chr8	120115732	1780	C	1	0.18	0.75	0.07	0.17	0.74	0.09	0.17	0.79	0.04	0.21	0.78	0.01	0.12	0.87	0.01	0.15	0.83	0.02
chr8	89376912	1630	G	1	0.15	0.79	0.06	0.16	0.80	0.04	0.14	0.82	0.04	0.07	0.92	0.00	0.10	0.90	0.00	0.11	0.87	0.02
chr8	120115733	1781	G	1	0.23	0.73	0.04	0.25	0.73	0.02	0.22	0.68	0.10	0.18	0.81	0.00	0.19	0.81	0.00	0.21	0.79	0.00
chr1!	100300039	372	C	1	0.88	0.05	0.07	0.89	0.07	0.04	0.87	0.08	0.04	0.69	0.04	0.27	0.77	0.19	0.04	0.74	0.25	0.01
chr5	64987556	1516	G	1	0.32	0.67	0.01	0.29	0.66	0.06	0.27	0.61	0.13	0.25	0.73	0.02	0.30	0.64	0.06	0.28	0.71	0.00
chr9	100498691	1991	c	1	0.10	0.87	0.03	0.10	0.84	0.06	0.10	0.90	0.01	0.12	0.85	0.03	0.11	0.82	0.07	0.09	0.77	0.14
chrX	7539482	2326	G	1	0.10	0.80	0.10	0.14	0.78	0.09	0.07	0.83	0.09	0.06	0.88	0.06	0.06	0.89	0.05	0.09	0.88	0.03
chr1'	3525856	145	C	1	0.27	0.67	0.06	0.29	0.71	0.00	0.27	0.68	0.05	0.16	0.84	0.00	0.16	0.84	0.00	0.16	0.82	0.02
chr8	120115699	1764	G	1	0.10	0.85	0.06	0.11	0.84	0.05	0.11	0.85	0.04	0.10	0.87	0.03	0.09	0.82	0.10	0.12	0.84	0.04
chr1(	24277521	922	g	1	0.15	0.81	0.04	0.15	0.71	0.13	0.14	0.81	0.04	0.15	0.80	0.06	0.13	0.84	0.03	0.13	0.87	0.00
chr1!	100300027	366	G	1	0.89	0.11	0.00	0.89	0.01	0.10	0.88	0.10	0.02	0.78	0.11	0.11	0.79	0.18	0.03	0.79	0.17	0.04
chr1(	24277534	929	c	1	0.16	0.81	0.04	0.10	0.87	0.04	0.13	0.85	0.02	0.18	0.69	0.13	0.18	0.80	0.02	0.13	0.84	0.02
chrX	7539405	2275	C	1	0.11	0.74	0.15	0.08	0.90	0.02	0.09	0.80	0.11	0.09	0.88	0.03	0.09	0.73	0.18	0.10	0.90	0.00
chr1(	24277560	942	c	1	0.12	0.85	0.03	0.11	0.76	0.13	0.14	0.83	0.03	0.10	0.80	0.10	0.10	0.85	0.05	0.12	0.85	0.02
chr8	120115746	1785	G	1	0.13	0.85	0.01	0.15	0.83	0.02	0.13	0.83	0.04	0.12	0.82	0.06	0.11	0.83	0.06	0.11	0.87	0.01
chr8	89376977	1656	G	1	0.31	0.68	0.01	0.31	0.67	0.01	0.28	0.70	0.02	0.17	0.83	0.01	0.24	0.76	0.00	0.22	0.69	0.09
chr1(	24277520	921	c	1	0.10	0.84	0.06	0.08	0.88	0.04	0.15	0.80	0.06	0.10	0.86	0.04	0.11	0.87	0.02	0.12	0.84	0.04
chrX	7539503	2336	G	1	0.16	0.80	0.04	0.16	0.83	0.01	0.17	0.77	0.06	0.15	0.85	0.00	0.23	0.76	0.01	0.19	0.65	0.16
chr8	120115672	1749	G	1	0.10	0.90	0.01	0.09	0.80	0.11	0.11	0.83	0.06	0.10	0.84	0.06	0.08	0.88	0.03	0.09	0.89	0.02
chr1(	24277563	944	c	1	0.10	0.86	0.04	0.11	0.85	0.04	0.13	0.83	0.04	0.11	0.82	0.07	0.13	0.84	0.03	0.12	0.85	0.03
chrX	7539455	2308	G	1	0.12	0.84	0.04	0.06	0.85	0.09	0.12	0.82	0.06	0.17	0.78	0.04	0.13	0.83	0.04	0.09	0.89	0.02
chrX	7539477	2322	G	1	0.07	0.89	0.04	0.10	0.85	0.06	0.06	0.86	0.08	0.05	0.83	0.12	0.09	0.86	0.06	0.07	0.88	0.04
chr1(	24277538	932	g	1	0.09	0.81	0.10	0.11	0.79	0.10	0.11	0.83	0.06	0.10	0.89	0.01	0.07	0.92	0.00	0.11	0.88	0.00
chr9	100498676	1985	c	1	0.17	0.83	0.01	0.17	0.70	0.13	0.18	0.82	0.00	0.19	0.79	0.02	0.19	0.80	0.01	0.20	0.72	0.08
chr1(	24277529	926	g	1	0.10	0.81	0.09	0.12	0.84	0.03	0.09	0.87	0.04	0.09	0.85	0.06	0.10	0.88	0.03	0.10	0.89	0.01
chrX	7539454	2307	C	1	0.13	0.87	0.01	0.08	0.91	0.00	0.14	0.85	0.01	0.12	0.82	0.06	0.11	0.86	0.03	0.16	0.77	0.07
chrX	7539431	2292	G	1	0.07	0.89	0.04	0.07	0.84	0.09	0.10	0.82	0.07	0.05	0.87	0.09	0.10	0.86	0.04	0.09	0.86	0.06
chr1(	24277690	990	C	1	0.10	0.89	0.01	0.09	0.86	0.05	0.09	0.89	0.01	0.13	0.86	0.01	0.12	0.69	0.19	0.12	0.87	0.01
chr1!	61868761	254	C	0	0.88	0.11	0.01	0.89	0.01	0.11	0.88	0.08	0.04	0.84	0.15	0.00	0.87	0.12	0.00	0.87	0.10	0.03
chr8	120115588	1708	G	1	0.07	0.88	0.05	0.07	0.81	0.12	0.07	0.83	0.09	0.06	0.84	0.10	0.08	0.91	0.00	0.07	0.89	0.03

0.21	0.77	0.02	0.18	0.77	0.04	0,04787
0.07	0.86	0.07	0.08	0.88	0.04	0,04756
0.18	0.79	0.03	0.11	0.86	0.03	0,04674
0.13	0.82	0.05	0.13	0.84	0.03	0,04505
0.07	0.84	0.09	0.06	0.90	0.04	0,04459
0.09	0.85	0.06	0.10	0.87	0.03	0,04417
0.42	0.55	0.02	0.30	0.68	0.01	0,04374
0.16	0.81	0.03	0.09	0.88	0.03	0,04316
0.12	0.84	0.04	0.10	0.86	0.04	0,04281
0.16	0.81	0.02	0.10	0.86	0.04	0,04098
0.11	0.83	0.05	0.09	0.91	0.01	0,03945
0.07	0.86	0.07	0.06	0.92	0.02	0,03917
0.43	0.54	0.03	0.37	0.60	0.02	0,03846
0.08	0.86	0.06	0.07	0.90	0.03	0,03757
0.18	0.79	0.04	0.09	0.90	0.01	0,03653
0.18	0.79	0.04	0.14	0.86	0.01	0,03638
0.16	0.80	0.04	0.13	0.85	0.01	0,03603
0.13	0.83	0.04	0.07	0.92	0.01	0,03522
0.21	0.75	0.04	0.18	0.82	0.01	0,03416
0.92	0.05	0.03	0.85	0.10	0.05	0,03387
0.29	0.68	0.03	0.26	0.72	0.01	0,03302
0.08	0.90	0.02	0.08	0.87	0.05	0,03199
0.08	0.86	0.07	0.04	0.93	0.03	0,03189
0.26	0.72	0.02	0.13	0.86	0.01	0,0316
0.08	0.88	0.04	0.08	0.89	0.03	0,03134
0.13	0.83	0.04	0.11	0.87	0.02	0,03046
0.95	0.04	0.02	0.84	0.12	0.04	0,03027
0.10	0.88	0.02	0.13	0.83	0.03	0,02987
0.07	0.88	0.05	0.08	0.90	0.02	0,02851
0.09	0.87	0.04	0.08	0.89	0.03	0,02754
0.12	0.86	0.02	0.09	0.88	0.03	0,0256
0.29	0.69	0.01	0.18	0.80	0.02	0,02473
0.08	0.88	0.04	0.09	0.89	0.02	0,0243
0.14	0.83	0.03	0.16	0.82	0.02	0,0235
0.08	0.89	0.03	0.07	0.91	0.03	0,02339
0.10	0.87	0.03	0.09	0.88	0.03	0,02298
0.07	0.89	0.04	0.10	0.88	0.02	0,02296
0.06	0.90	0.04	0.05	0.90	0.04	0,0225
0.08	0.86	0.06	0.07	0.92	0.01	0,02204
0.17	0.81	0.02	0.18	0.80	0.02	0,02091
0.09	0.88	0.03	0.07	0.91	0.02	0,02071
0.08	0.91	0.01	0.11	0.86	0.04	0,0194
0.06	0.90	0.04	0.05	0.92	0.04	0,01923
0.07	0.92	0.01	0.10	0.87	0.03	0,01864
0.94	0.04	0.03	0.89	0.10	0.01	0,01842
0.06	0.89	0.05	0.06	0.92	0.02	0,01836

chr8	120115691	1759	G	1	0.11	0.88	0.01	0.15	0.80	0.05	0.15	0.80	0.05	0.12	0.87	0.00	0.14	0.80	0.07	0.15	0.83	0.01
chr8	120115671	1748	C	1	0.07	0.92	0.01	0.09	0.78	0.14	0.07	0.88	0.05	0.07	0.90	0.03	0.06	0.92	0.02	0.07	0.85	0.09
chr1(	24277595	958	c	1	0.09	0.87	0.04	0.07	0.82	0.11	0.09	0.87	0.04	0.09	0.89	0.02	0.09	0.91	0.00	0.10	0.87	0.03
chr1(	24277561	943	g	1	0.12	0.83	0.05	0.11	0.84	0.05	0.11	0.84	0.05	0.10	0.90	0.01	0.10	0.89	0.01	0.11	0.88	0.00
chr8	120115797	1808	G	1	0.15	0.85	0.01	0.16	0.81	0.04	0.15	0.80	0.05	0.14	0.84	0.02	0.15	0.82	0.03	0.13	0.87	0.00
chr1(	24277564	945	g	1	0.10	0.84	0.05	0.11	0.86	0.03	0.13	0.81	0.05	0.13	0.85	0.02	0.11	0.88	0.01	0.12	0.87	0.01
chr1(	24277593	957	g	1	0.09	0.86	0.05	0.11	0.85	0.04	0.12	0.86	0.02	0.11	0.87	0.02	0.08	0.87	0.04	0.10	0.90	0.00
chr8	120115811	1814	C	0	0.90	0.09	0.01	0.87	0.08	0.05	0.84	0.15	0.01	0.87	0.05	0.08	0.79	0.20	0.01	0.84	0.11	0.05
chr1(	24277592	956	c	1	0.06	0.88	0.06	0.06	0.90	0.05	0.06	0.87	0.07	0.05	0.87	0.07	0.05	0.95	0.00	0.07	0.92	0.01
chr1(	24277551	938	g	1	0.08	0.86	0.06	0.09	0.86	0.05	0.10	0.88	0.02	0.10	0.89	0.01	0.08	0.88	0.04	0.11	0.88	0.01
chr4	136547586	1156	G	1	0.11	0.85	0.04	0.09	0.90	0.01	0.10	0.90	0.00	0.12	0.87	0.01	0.11	0.83	0.05	0.11	0.83	0.06
chr1(	24277739	1007	g	1	0.05	0.88	0.07	0.05	0.88	0.07	0.08	0.88	0.04	0.07	0.92	0.01	0.06	0.94	0.00	0.08	0.92	0.00
chr8	120115680	1754	G	1	0.07	0.90	0.03	0.07	0.89	0.04	0.06	0.86	0.07	0.07	0.93	0.00	0.06	0.89	0.05	0.07	0.93	0.00
chr8	120115698	1763	C	1	0.05	0.92	0.03	0.05	0.83	0.12	0.07	0.88	0.05	0.05	0.92	0.03	0.02	0.96	0.02	0.06	0.91	0.04
chr8	120115599	1713	G	1	0.05	0.90	0.05	0.05	0.91	0.04	0.06	0.90	0.05	0.04	0.90	0.06	0.07	0.91	0.03	0.06	0.85	0.09
chr8	120115660	1744	G	1	0.11	0.89	0.00	0.13	0.85	0.02	0.13	0.84	0.02	0.09	0.89	0.02	0.10	0.86	0.04	0.11	0.84	0.04
chr1(	24277528	925	c	1	0.10	0.88	0.01	0.09	0.86	0.05	0.07	0.86	0.07	0.10	0.87	0.03	0.09	0.90	0.01	0.08	0.89	0.03
chr4	136547585	1155	C	1	0.05	0.92	0.02	0.02	0.79	0.19	0.05	0.91	0.03	0.06	0.91	0.03	0.10	0.87	0.03	0.09	0.87	0.04
chr8	89377076	1690	G	1	0.13	0.83	0.05	0.11	0.88	0.01	0.08	0.87	0.05	0.03	0.97	0.00	0.07	0.85	0.07	0.06	0.90	0.04
chr8	120115679	1753	C	1	0.03	0.92	0.05	0.04	0.91	0.04	0.03	0.95	0.02	0.02	0.85	0.13	0.02	0.93	0.05	0.03	0.86	0.11
chr4	136547531	1124	C	1	0.06	0.91	0.03	0.07	0.86	0.07	0.05	0.88	0.07	0.07	0.92	0.01	0.06	0.93	0.01	0.04	0.92	0.04
chrX	7539443	2299	G	0	0.94	0.02	0.04	0.93	0.03	0.04	0.90	0.05	0.05	0.77	0.19	0.04	0.91	0.04	0.05	0.91	0.06	0.03
chr8	120115784	1802	C	0	0.89	0.10	0.01	0.90	0.08	0.02	0.89	0.05	0.06	0.91	0.08	0.01	0.79	0.20	0.01	0.87	0.08	0.05
chrX	7539476	2321	C	1	0.07	0.91	0.02	0.06	0.89	0.04	0.08	0.91	0.00	0.05	0.83	0.12	0.05	0.89	0.06	0.10	0.89	0.01
chr8	89376911	1629	C	1	0.13	0.86	0.02	0.11	0.84	0.05	0.13	0.83	0.04	0.05	0.95	0.00	0.06	0.90	0.04	0.11	0.89	0.01
chr4	136547564	1145	C	1	0.07	0.87	0.06	0.02	0.87	0.11	0.05	0.92	0.03	0.04	0.91	0.05	0.04	0.94	0.02	0.07	0.90	0.03
chr4	136547423	1059	G	0	0.85	0.15	0.00	0.90	0.09	0.01	0.86	0.08	0.06	0.82	0.18	0.00	0.86	0.14	0.00	0.85	0.15	0.00
chr1(	24277590	955	g	1	0.07	0.89	0.04	0.07	0.88	0.05	0.09	0.89	0.03	0.07	0.93	0.00	0.06	0.94	0.00	0.09	0.91	0.00
chr4	136547414	1054	G	0	0.78	0.22	0.00	0.82	0.15	0.04	0.80	0.20	0.01	0.75	0.13	0.11	0.73	0.27	0.00	0.78	0.22	0.00
chr1(	24277691	991	G	1	0.10	0.89	0.00	0.13	0.85	0.02	0.13	0.85	0.03	0.12	0.88	0.01	0.13	0.85	0.02	0.14	0.86	0.00
chr4	136547443	1072	C	1	0.12	0.84	0.04	0.09	0.89	0.02	0.13	0.86	0.01	0.14	0.79	0.07	0.12	0.87	0.00	0.12	0.88	0.00
chr4	136547566	1147	C	1	0.10	0.87	0.03	0.02	0.86	0.12	0.03	0.92	0.05	0.02	0.92	0.06	0.10	0.86	0.05	0.05	0.93	0.02
chr4	136547748	1207	C	0	0.96	0.01	0.03	0.87	0.03	0.10	0.88	0.01	0.11	0.94	0.06	0.00	0.94	0.02	0.04	0.86	0.14	0.00
chr4	136547729	1199	C	1	0.07	0.89	0.05	0.05	0.93	0.03	0.05	0.90	0.04	0.06	0.90	0.04	0.07	0.93	0.00	0.06	0.83	0.11
chrX	7539430	2291	C	1	0.08	0.91	0.00	0.09	0.87	0.04	0.09	0.90	0.01	0.08	0.91	0.01	0.09	0.90	0.01	0.09	0.84	0.07
chr4	136547539	1130	C	1	0.02	0.94	0.04	0.02	0.91	0.07	0.08	0.88	0.04	0.06	0.91	0.03	0.09	0.87	0.05	0.03	0.90	0.07
chr1(	92644703	664	C	0	0.85	0.02	0.13	0.91	0.03	0.06	0.81	0.16	0.03	0.91	0.04	0.05	0.97	0.01	0.01	0.90	0.05	0.05
chr1(	24277511	915	c	0	0.90	0.09	0.01	0.89	0.06	0.04	0.92	0.06	0.02	0.92	0.03	0.05	0.88	0.11	0.01	0.91	0.06	0.04
chr4	136547425	1060	G	0	0.89	0.11	0.00	0.87	0.12	0.01	0.87	0.12	0.01	0.87	0.11	0.02	0.87	0.10	0.03	0.89	0.10	0.01
chr4	136547527	1122	C	1	0.05	0.89	0.06	0.05	0.88	0.07	0.03	0.89	0.08	0.04	0.94	0.01	0.05	0.94	0.01	0.03	0.90	0.08
chrX	7539593	2391	G	1	0.08	0.84	0.08	0.06	0.93	0.01	0.09	0.91	0.00	0.09	0.89	0.03	0.11	0.81	0.07	0.07	0.93	0.00
chr4	136547545	1133	C	1	0.03	0.96	0.01	0.04	0.92	0.04	0.04	0.85	0.10	0.04	0.94	0.02	0.05	0.86	0.09	0.03	0.90	0.07
chr8	120115690	1758	C	1	0.08	0.90	0.02	0.07	0.89	0.04	0.06	0.90	0.04	0.07	0.93	0.01	0.06	0.93	0.01	0.07	0.85	0.08
chr1!	61868823	283	C	0	0.92	0.07	0.01	0.93	0.01	0.06	0.91	0.07	0.02	0.90	0.08	0.01	0.90	0.10	0.00	0.90	0.05	0.05
chr4	136547498	1104	G	1	0.05	0.93	0.01	0.07	0.88	0.05	0.07	0.90	0.02	0.07	0.90	0.03	0.06	0.94	0.00	0.07	0.91	0.02
chr4	136547518	1117	G	1	0.07	0.92	0.01	0.07	0.88	0.05	0.07	0.90	0.03	0.05	0.90	0.05	0.07	0.93	0.00	0.07	0.93	0.01

0.11	0.87	0.02	0.10	0.88	0.01	0,01772
0.06	0.91	0.03	0.04	0.93	0.03	0,01755
0.06	0.90	0.04	0.07	0.92	0.01	0,01727
0.09	0.88	0.03	0.08	0.91	0.01	0,01719
0.13	0.85	0.02	0.12	0.87	0.01	0,01717
0.10	0.87	0.03	0.09	0.89	0.01	0,01684
0.07	0.90	0.03	0.08	0.91	0.01	0,01604
0.90	0.08	0.01	0.89	0.08	0.03	0,01602
0.04	0.91	0.04	0.04	0.95	0.01	0,01587
0.07	0.90	0.03	0.08	0.91	0.02	0,01559
0.08	0.91	0.01	0.09	0.88	0.03	0,01544
0.05	0.91	0.04	0.06	0.94	0.01	0,01481
0.05	0.91	0.03	0.05	0.94	0.01	0,01432
0.04	0.91	0.04	0.04	0.94	0.02	0,01397
0.04	0.93	0.03	0.04	0.92	0.04	0,01391
0.08	0.90	0.01	0.07	0.90	0.02	0,01379
0.06	0.91	0.03	0.06	0.92	0.02	0,01372
0.03	0.93	0.04	0.06	0.92	0.02	0,01352
0.08	0.90	0.02	0.04	0.94	0.02	0,01288
0.02	0.95	0.03	0.02	0.91	0.07	0,01236
0.05	0.92	0.04	0.04	0.94	0.02	0,01205
0.95	0.02	0.03	0.91	0.06	0.03	0,01203
0.93	0.06	0.02	0.90	0.09	0.02	0,01197
0.05	0.94	0.02	0.04	0.92	0.03	0,01188
0.09	0.89	0.02	0.05	0.94	0.01	0,01184
0.03	0.93	0.04	0.04	0.94	0.02	0,01144
0.91	0.07	0.01	0.87	0.13	0.01	0,01112
0.06	0.92	0.03	0.05	0.94	0.01	0,01032
0.83	0.16	0.01	0.79	0.20	0.01	0,01028
0.09	0.89	0.01	0.10	0.89	0.01	0,01013
0.08	0.90	0.02	0.10	0.89	0.01	0,00995
0.03	0.93	0.04	0.03	0.94	0.03	0,00992
0.93	0.01	0.05	0.95	0.04	0.01	0,00985
0.04	0.94	0.03	0.05	0.93	0.02	0,00971
0.05	0.93	0.01	0.06	0.92	0.02	0,00971
0.03	0.94	0.03	0.04	0.93	0.03	0,00949
0.92	0.04	0.04	0.95	0.02	0.03	0,00947
0.93	0.05	0.02	0.94	0.04	0.02	0,00935
0.90	0.09	0.01	0.90	0.08	0.02	0,00928
0.03	0.92	0.05	0.03	0.96	0.02	0,00903
0.06	0.93	0.01	0.07	0.92	0.02	0,00857
0.03	0.95	0.03	0.03	0.94	0.03	0,00828
0.06	0.93	0.02	0.04	0.94	0.02	0,00807
0.95	0.03	0.02	0.93	0.06	0.01	0,00794
0.05	0.93	0.02	0.05	0.94	0.01	0,00785
0.05	0.93	0.02	0.04	0.94	0.01	0,00779

chr4	136547517	1116	C	1	0.05	0.93	0.02	0.07	0.90	0.03	0.04	0.80	0.15	0.03	0.97	0.00	0.05	0.94	0.01	0.03	0.96	0.00
chrX	7539592	2390	C	1	0.08	0.91	0.01	0.08	0.88	0.04	0.05	0.89	0.06	0.05	0.95	0.00	0.07	0.89	0.04	0.07	0.89	0.04
chr4	136547764	1214	C	1	0.05	0.93	0.02	0.05	0.91	0.05	0.04	0.91	0.05	0.02	0.97	0.01	0.06	0.93	0.01	0.02	0.91	0.07
chr1!	24277589	954	c	1	0.07	0.90	0.04	0.05	0.82	0.14	0.04	0.95	0.01	0.05	0.90	0.05	0.05	0.95	0.00	0.07	0.93	0.01
chr8	89376892	1621	c	1	0.04	0.88	0.08	0.03	0.89	0.08	0.05	0.94	0.01	0.06	0.93	0.01	0.04	0.93	0.03	0.04	0.96	0.00
chr8	89376889	1619	g	0	0.92	0.07	0.01	0.91	0.03	0.07	0.90	0.09	0.01	0.87	0.12	0.01	0.89	0.11	0.00	0.90	0.05	0.05
chr4	136547454	1079	C	1	0.05	0.84	0.11	0.07	0.91	0.01	0.04	0.93	0.04	0.04	0.95	0.00	0.08	0.91	0.01	0.05	0.94	0.01
chr8	89376893	1622	g	1	0.08	0.92	0.00	0.07	0.88	0.05	0.07	0.90	0.03	0.03	0.97	0.00	0.07	0.93	0.00	0.07	0.93	0.01
chrX	7539429	2290	G	0	0.90	0.06	0.04	0.89	0.05	0.06	0.90	0.03	0.07	0.89	0.09	0.02	0.95	0.02	0.03	0.95	0.02	0.03
chr4	136547383	1038	G	0	0.91	0.09	0.00	0.95	0.04	0.01	0.92	0.05	0.03	0.92	0.05	0.04	0.91	0.02	0.07	0.92	0.07	0.01
chr4	136547497	1103	C	1	0.06	0.92	0.02	0.06	0.93	0.01	0.08	0.92	0.01	0.07	0.85	0.08	0.04	0.96	0.00	0.10	0.88	0.03
chr4	136547408	1051	C	1	0.07	0.91	0.02	0.06	0.93	0.01	0.04	0.85	0.11	0.06	0.94	0.00	0.06	0.92	0.02	0.05	0.95	0.00
chr5	64987481	1481	G	0	0.90	0.04	0.05	0.92	0.04	0.04	0.94	0.02	0.04	0.93	0.01	0.06	0.94	0.04	0.02	0.94	0.02	0.04
chr4	136547444	1073	G	1	0.04	0.95	0.01	0.06	0.93	0.01	0.06	0.93	0.01	0.06	0.89	0.05	0.05	0.94	0.01	0.06	0.91	0.03
chr1(	92644705	665	C	0	0.95	0.03	0.02	0.95	0.03	0.03	0.90	0.06	0.04	0.93	0.05	0.02	0.95	0.03	0.01	0.90	0.09	0.01
chr4	136547455	1080	G	1	0.03	0.90	0.07	0.06	0.92	0.02	0.05	0.94	0.01	0.06	0.92	0.02	0.04	0.93	0.03	0.05	0.94	0.01
chr4	136547548	1136	C	1	0.03	0.93	0.03	0.01	0.93	0.06	0.02	0.93	0.05	0.05	0.93	0.02	0.07	0.89	0.04	0.03	0.95	0.02
chr8	120115608	1717	G	0	0.93	0.06	0.01	0.93	0.06	0.01	0.91	0.06	0.03	0.94	0.05	0.01	0.92	0.08	0.00	0.92	0.05	0.03
chr4	136547546	1134	G	1	0.04	0.95	0.02	0.04	0.87	0.10	0.04	0.95	0.01	0.03	0.94	0.03	0.03	0.95	0.02	0.04	0.96	0.00
chr1!	24277569	947	c	0	0.99	0.01	0.00	0.98	0.01	0.02	0.97	0.02	0.01	0.96	0.04	0.00	0.98	0.02	0.01	0.97	0.02	0.00
chrX	7539444	2300	C	0	0.95	0.03	0.02	0.94	0.05	0.01	0.94	0.05	0.01	0.93	0.07	0.00	0.93	0.01	0.06	0.92	0.01	0.07
chrX	7539434	2293	G	0	0.92	0.05	0.03	0.89	0.04	0.06	0.95	0.03	0.02	0.96	0.02	0.02	0.94	0.04	0.02	0.91	0.07	0.01
chr8	120115782	1800	C	0	0.93	0.01	0.05	0.94	0.04	0.01	0.93	0.06	0.02	0.86	0.14	0.00	0.93	0.01	0.05	0.95	0.03	0.03
chr1(	92644483	589	G	0	0.95	0.04	0.01	0.94	0.04	0.03	0.92	0.07	0.01	0.92	0.06	0.03	0.94	0.05	0.01	0.93	0.07	0.01
chr1!	24277741	1008	g	1	0.03	0.93	0.04	0.03	0.95	0.02	0.04	0.94	0.02	0.06	0.93	0.01	0.05	0.95	0.00	0.04	0.96	0.00
chr8	120115720	1774	C	0	0.92	0.07	0.01	0.94	0.02	0.04	0.91	0.06	0.03	0.95	0.01	0.04	0.91	0.09	0.01	0.95	0.04	0.01
chr4	136547558	1141	C	0	0.96	0.02	0.02	0.96	0.02	0.02	0.90	0.02	0.08	0.91	0.07	0.02	0.94	0.01	0.05	0.92	0.03	0.05
chr8	89376902	1626	c	0	0.95	0.04	0.01	0.94	0.05	0.01	0.95	0.03	0.02	0.90	0.10	0.00	0.93	0.06	0.01	0.94	0.03	0.03
chr1(	92644706	666	C	0	0.93	0.06	0.01	0.95	0.03	0.02	0.95	0.03	0.02	0.95	0.03	0.03	0.95	0.03	0.01	0.89	0.09	0.02
chr1!	24277644	980	C	0	0.91	0.09	0.01	0.90	0.01	0.09	0.88	0.00	0.12	0.91	0.08	0.01	0.97	0.00	0.03	0.96	0.03	0.01
chr4	136547567	1148	G	1	0.02	0.95	0.03	0.04	0.94	0.03	0.03	0.97	0.00	0.05	0.93	0.02	0.03	0.94	0.03	0.04	0.94	0.03
chr4	136547501	1106	G	0	0.91	0.09	0.00	0.93	0.04	0.02	0.92	0.08	0.01	0.92	0.08	0.01	0.94	0.04	0.03	0.92	0.08	0.00
chr4	136547528	1123	G	1	0.04	0.91	0.05	0.05	0.93	0.02	0.06	0.94	0.00	0.06	0.93	0.01	0.05	0.94	0.01	0.05	0.95	0.00
chr4	136547431	1064	G	0	0.91	0.09	0.01	0.92	0.07	0.01	0.91	0.09	0.01	0.92	0.02	0.07	0.92	0.08	0.00	0.93	0.07	0.00
chr8	120115775	1797	C	0	0.96	0.03	0.01	0.94	0.05	0.01	0.94	0.02	0.05	0.93	0.03	0.04	0.92	0.01	0.07	0.92	0.06	0.02
chr8	89376991	1660	C	0	0.94	0.06	0.00	0.94	0.05	0.01	0.94	0.04	0.01	0.96	0.02	0.02	0.94	0.04	0.01	0.94	0.06	0.00
chr5	64987431	1455	G	0	0.96	0.03	0.01	0.93	0.04	0.02	0.95	0.04	0.01	0.93	0.07	0.00	0.94	0.06	0.01	0.96	0.04	0.01
chr8	120115634	1732	C	0	1.00	0.00	0.00	1.00	0.00	0.00	0.99	0.01	0.00	0.99	0.00	0.00	1.00	0.00	0.00	1.00	0.00	0.00
chr5	64987483	1482	G	0	0.97	0.02	0.01	0.96	0.02	0.03	0.92	0.02	0.06	0.94	0.04	0.02	0.94	0.05	0.02	0.95	0.04	0.01
chr1!	61868872	306	G	0	0.96	0.02	0.03	0.95	0.01	0.04	0.97	0.02	0.01	0.94	0.05	0.01	0.92	0.07	0.00	0.96	0.03	0.00
chr4	136547657	1184	C	0	0.96	0.03	0.00	0.98	0.01	0.02	0.91	0.01	0.08	0.95	0.05	0.00	0.94	0.06	0.00	0.93	0.07	0.00
chr4	136547565	1146	G	1	0.03	0.93	0.04	0.04	0.95	0.01	0.04	0.95	0.02	0.03	0.97	0.00	0.04	0.95	0.01	0.04	0.94	0.02
chr4	136547540	1131	G	1	0.03	0.95	0.02	0.04	0.92	0.05	0.05	0.95	0.00	0.04	0.95	0.02	0.03	0.97	0.00	0.04	0.96	0.00
chr8	120115778	1799	C	0	0.95	0.04	0.00	0.96	0.03	0.01	0.97	0.02	0.01	0.96	0.03	0.02	0.94	0.01	0.04	0.94	0.05	0.01
chrX	7539588	2387	G	0	0.93	0.03	0.04	0.94	0.06	0.00	0.92	0.07	0.01	0.89	0.10	0.00	0.97	0.00	0.03	0.94	0.06	0.00
chr4	136547607	1168	C	0	0.96	0.02	0.01	0.96	0.02	0.03	0.96	0.02	0.02	0.94	0.02	0.04	0.89	0.09	0.01	0.95	0.02	0.04

0.04	0.93	0.03	0.03	0.97	0.01	0,00741
0.05	0.93	0.02	0.05	0.94	0.01	0,00727
0.04	0.94	0.03	0.02	0.96	0.02	0,00698
0.04	0.94	0.03	0.04	0.95	0.01	0,00683
0.03	0.94	0.03	0.03	0.95	0.01	0,0067
0.94	0.04	0.01	0.92	0.07	0.01	0,00662
0.04	0.93	0.03	0.04	0.95	0.01	0,00662
0.06	0.93	0.02	0.03	0.96	0.01	0,00659
0.93	0.03	0.04	0.96	0.02	0.02	0,00643
0.95	0.04	0.01	0.95	0.03	0.03	0,00631
0.05	0.94	0.01	0.05	0.94	0.02	0,00619
0.04	0.93	0.03	0.03	0.96	0.01	0,00607
0.95	0.03	0.03	0.96	0.02	0.02	0,00577
0.04	0.95	0.01	0.04	0.94	0.02	0,00555
0.95	0.03	0.02	0.95	0.04	0.01	0,00548
0.04	0.95	0.02	0.04	0.95	0.01	0,00534
0.02	0.95	0.03	0.03	0.95	0.02	0,00521
0.94	0.04	0.01	0.94	0.05	0.01	0,00485
0.03	0.95	0.02	0.03	0.96	0.01	0,0048
0.98	0.01	0.01	0.61	0.24	0.15	0,00443
0.96	0.03	0.01	0.96	0.02	0.02	0,00441
0.95	0.03	0.02	0.96	0.03	0.01	0,00436
0.96	0.03	0.02	0.95	0.03	0.02	0,0042
0.96	0.03	0.01	0.95	0.04	0.01	0,00413
0.03	0.96	0.02	0.04	0.96	0.01	0,00404
0.95	0.03	0.02	0.96	0.03	0.01	0,004
0.96	0.01	0.02	0.95	0.02	0.03	0,00382
0.96	0.03	0.01	0.94	0.04	0.01	0,0038
0.96	0.03	0.02	0.95	0.03	0.02	0,00378
0.95	0.02	0.04	0.97	0.02	0.01	0,00368
0.02	0.97	0.01	0.03	0.95	0.02	0,00351
0.94	0.05	0.01	0.95	0.04	0.01	0,00351
0.04	0.95	0.01	0.04	0.96	0.01	0,00343
0.93	0.06	0.01	0.96	0.04	0.01	0,00343
0.96	0.02	0.01	0.95	0.02	0.02	0,0034
0.95	0.04	0.01	0.96	0.03	0.01	0,00331
0.96	0.03	0.01	0.95	0.04	0.01	0,00322
0.99	0.00	0.01	0.66	0.16	0.18	0,00304
0.97	0.01	0.02	0.96	0.03	0.01	0,00296
0.97	0.01	0.02	0.96	0.04	0.01	0,00296
0.97	0.01	0.01	0.96	0.04	0.01	0,0027
0.03	0.96	0.01	0.03	0.97	0.01	0,00254
0.03	0.96	0.02	0.03	0.97	0.01	0,00253
0.97	0.02	0.01	0.97	0.01	0.02	0,00245
0.95	0.03	0.01	0.97	0.02	0.01	0,00242
0.97	0.02	0.01	0.96	0.02	0.02	0,00242

chr1(	92644702	663	C	0	0.97	0.03	0.01	0.96	0.02	0.02	0.95	0.02	0.03	0.95	0.03	0.02	0.96	0.03	0.01	0.94	0.03	0.03
chr4	136547549	1137	G	1	0.05	0.95	0.00	0.04	0.95	0.00	0.04	0.96	0.00	0.06	0.89	0.05	0.04	0.95	0.00	0.04	0.96	0.00
chr1;	45706595	784	C	0	0.98	0.00	0.02	0.96	0.01	0.03	0.96	0.01	0.03	0.95	0.01	0.03	0.95	0.03	0.03	0.95	0.01	0.04
chr5	64987440	1459	G	0	0.96	0.03	0.02	0.96	0.02	0.02	0.96	0.03	0.02	0.91	0.04	0.05	0.98	0.01	0.01	0.96	0.01	0.03
chr4	136547396	1047	G	0	0.96	0.03	0.00	0.96	0.04	0.00	0.95	0.04	0.00	0.93	0.05	0.02	0.92	0.07	0.02	0.93	0.07	0.00
chr4	136547532	1125	G	1	0.04	0.96	0.00	0.04	0.94	0.02	0.03	0.90	0.07	0.03	0.93	0.04	0.03	0.97	0.00	0.04	0.95	0.01
chr8	120115805	1811	G	0	0.96	0.03	0.01	0.96	0.04	0.00	0.96	0.03	0.01	0.94	0.06	0.00	0.94	0.06	0.00	0.96	0.04	0.00
chr4	136547469	1088	C	0	0.92	0.01	0.07	0.95	0.01	0.04	0.97	0.02	0.01	0.95	0.00	0.04	0.91	0.09	0.00	0.90	0.10	0.00
chr8	120115645	1736	C	0	0.95	0.01	0.04	0.95	0.01	0.04	0.95	0.05	0.01	0.97	0.00	0.03	0.95	0.02	0.03	0.96	0.01	0.03
chr8	120115783	1801	C	0	0.94	0.04	0.02	0.98	0.01	0.01	0.93	0.07	0.00	0.96	0.03	0.01	0.93	0.07	0.00	0.95	0.04	0.00
chr4	136547435	1066	G	0	0.92	0.08	0.00	0.95	0.05	0.00	0.96	0.02	0.02	0.94	0.04	0.03	0.95	0.05	0.00	0.94	0.06	0.00
chr1!	100300420	501	C	0	0.96	0.01	0.03	0.96	0.02	0.02	0.95	0.01	0.03	0.97	0.01	0.02	0.95	0.01	0.04	0.96	0.00	0.04
chr4	139783720	1296	C	0	0.95	0.01	0.04	0.97	0.01	0.02	0.96	0.02	0.02	0.95	0.04	0.01	0.94	0.04	0.01	0.97	0.02	0.01
chr5	64987342	1418	G	0	0.96	0.04	0.01	0.94	0.06	0.01	0.96	0.03	0.01	0.94	0.05	0.00	0.97	0.00	0.03	0.96	0.01	0.03
chr5	64987484	1483	C	0	0.95	0.03	0.02	0.97	0.01	0.01	0.94	0.04	0.02	0.96	0.02	0.02	0.96	0.03	0.01	0.96	0.02	0.02
chr4	136547409	1052	G	1	0.02	0.97	0.01	0.02	0.95	0.03	0.03	0.96	0.01	0.03	0.96	0.01	0.03	0.97	0.00	0.04	0.96	0.00
chr1(	92644590	651	C	0	0.98	0.01	0.01	0.99	0.01	0.01	0.98	0.01	0.01	0.98	0.01	0.01	0.97	0.01	0.02	0.99	0.00	0.01
chr1(	92644711	669	C	0	0.94	0.05	0.01	0.96	0.02	0.03	0.96	0.02	0.03	0.90	0.08	0.02	0.97	0.01	0.02	0.97	0.02	0.01
chr1(	24277642	979	C	0	0.97	0.00	0.03	0.94	0.03	0.03	0.92	0.04	0.04	0.96	0.00	0.04	0.95	0.05	0.00	0.99	0.01	0.00
chr1!	61868658	205	C	0	0.97	0.03	0.00	0.95	0.04	0.01	0.95	0.04	0.00	0.95	0.05	0.00	0.96	0.02	0.03	0.95	0.04	0.00
chrX	7539493	2331	G	0	0.97	0.03	0.01	0.97	0.02	0.01	0.92	0.02	0.06	0.96	0.02	0.02	0.96	0.02	0.02	0.97	0.02	0.01
chr8	120115758	1789	C	0	0.99	0.01	0.00	0.99	0.01	0.00	0.99	0.00	0.01	0.99	0.01	0.00	0.98	0.00	0.02	0.96	0.03	0.00
chr8	120115777	1798	C	0	0.96	0.02	0.02	0.97	0.02	0.01	0.96	0.03	0.01	0.96	0.02	0.02	0.96	0.02	0.02	0.96	0.04	0.01
chr4	136547589	1158	C	0	0.93	0.03	0.03	0.94	0.03	0.03	0.98	0.01	0.01	0.98	0.01	0.02	0.97	0.01	0.02	0.90	0.09	0.01
chr4	139783776	1327	C	0	0.96	0.01	0.03	0.97	0.01	0.02	0.97	0.03	0.00	0.96	0.03	0.00	0.94	0.01	0.05	0.95	0.01	0.03
chr4	139783696	1284	C	0	0.97	0.00	0.03	0.96	0.03	0.01	0.98	0.00	0.02	0.95	0.05	0.00	0.97	0.03	0.00	0.96	0.02	0.03
chr8	120115718	1773	C	0	0.95	0.04	0.01	0.97	0.01	0.02	0.97	0.02	0.01	0.98	0.00	0.02	0.91	0.09	0.00	0.96	0.03	0.01
chr4	136547742	1205	C	0	0.96	0.03	0.01	0.95	0.01	0.04	0.96	0.04	0.01	0.95	0.01	0.03	0.92	0.07	0.00	0.96	0.04	0.00
chr4	139783694	1283	C	0	0.97	0.02	0.00	0.96	0.04	0.00	0.98	0.02	0.00	0.94	0.06	0.00	0.94	0.05	0.00	0.94	0.05	0.00
chr1!	100300299	465	C	0	0.98	0.01	0.01	0.97	0.01	0.01	0.94	0.01	0.04	0.92	0.06	0.02	0.98	0.01	0.01	0.97	0.01	0.02
chrX	7539421	2285	G	0	0.97	0.01	0.02	0.94	0.03	0.03	0.97	0.01	0.02	0.95	0.02	0.03	0.97	0.01	0.02	0.97	0.01	0.02
chr1!	100300316	473	C	0	0.94	0.05	0.00	0.96	0.01	0.02	0.97	0.01	0.03	0.98	0.01	0.01	0.95	0.01	0.04	0.96	0.00	0.04
chr1;	45706778	865	G	0	0.97	0.01	0.03	0.98	0.01	0.01	0.97	0.02	0.01	0.96	0.04	0.00	0.97	0.03	0.01	0.96	0.04	0.01
chr5	64987632	1543	C	0	0.97	0.03	0.00	0.96	0.03	0.01	0.98	0.00	0.01	0.96	0.02	0.01	0.97	0.01	0.02	0.95	0.01	0.04
chr1!	100300176	435	G	0	0.97	0.02	0.01	0.99	0.00	0.01	0.97	0.01	0.02	0.95	0.03	0.03	0.96	0.03	0.01	0.96	0.01	0.04
chrX	7539459	2310	G	0	0.96	0.01	0.03	0.94	0.03	0.04	0.98	0.01	0.01	0.97	0.02	0.01	0.96	0.02	0.02	0.98	0.01	0.01
chr1!	61868776	263	G	0	0.97	0.01	0.02	0.97	0.02	0.01	0.97	0.02	0.01	0.97	0.02	0.01	0.96	0.03	0.01	0.95	0.03	0.02
chr1(	92644517	615	C	0	0.96	0.02	0.02	0.98	0.01	0.01	0.98	0.01	0.01	0.96	0.02	0.01	0.92	0.07	0.01	0.97	0.01	0.02
chr1(	92644701	662	C	0	0.98	0.01	0.01	0.95	0.02	0.03	0.95	0.02	0.02	0.97	0.01	0.02	0.97	0.01	0.02	0.96	0.01	0.02
chr4	139783732	1302	C	0	0.97	0.01	0.01	0.97	0.01	0.01	0.98	0.01	0.01	0.96	0.02	0.02	0.95	0.03	0.02	0.96	0.04	0.01
chr4	136547385	1039	G	0	0.97	0.03	0.00	0.97	0.01	0.02	0.97	0.02	0.01	0.94	0.06	0.00	0.96	0.04	0.00	0.97	0.02	0.01
chr4	136547478	1093	C	0	0.96	0.00	0.04	0.94	0.03	0.03	0.96	0.04	0.00	0.96	0.00	0.04	0.94	0.02	0.04	0.94	0.06	0.00
chr1(	24277476	897	g	0	1.00	0.00	0.00	1.00	0.00	0.00	1.00	0.00	0.00	1.00	0.00	0.00	1.00	0.00	0.00	1.00	0.00	0.00
chr4	136547624	1173	C	0	0.97	0.01	0.03	0.96	0.02	0.03	0.97	0.01	0.02	0.96	0.02	0.02	0.96	0.01	0.03	0.97	0.01	0.02
chr4	136547626	1174	C	0	0.98	0.01	0.01	0.93	0.03	0.04	0.95	0.03	0.02	0.97	0.01	0.02	0.98	0.01	0.01	0.99	0.00	0.01
chr1;	45706494	727	C	0	0.98	0.01	0.01	0.99	0.01	0.01	0.97	0.03	0.00	0.97	0.01	0.03	0.96	0.02	0.02	0.97	0.02	0.01

0.97	0.02	0.01	0.96	0.02	0.01	0,00228
0.03	0.97	0.01	0.04	0.96	0.01	0,00226
0.97	0.01	0.02	0.97	0.02	0.01	0,00224
0.97	0.02	0.01	0.97	0.01	0.02	0,00222
0.97	0.03	0.01	0.95	0.04	0.01	0,00216
0.03	0.96	0.01	0.02	0.97	0.01	0,00215
0.97	0.03	0.01	0.96	0.04	0.00	0,00214
0.97	0.01	0.02	0.97	0.02	0.01	0,00213
0.97	0.02	0.02	0.97	0.01	0.02	0,00208
0.97	0.02	0.01	0.96	0.03	0.01	0,00206
0.96	0.03	0.01	0.96	0.03	0.01	0,00204
0.97	0.01	0.02	0.97	0.01	0.02	0,00202
0.97	0.01	0.02	0.97	0.03	0.01	0,002
0.96	0.03	0.01	0.98	0.01	0.01	0,00198
0.96	0.02	0.01	0.97	0.02	0.01	0,00197
0.02	0.97	0.01	0.03	0.97	0.01	0,00192
0.98	0.01	0.01	0.80	0.09	0.11	0,0019
0.97	0.02	0.01	0.97	0.02	0.01	0,00188
0.96	0.01	0.03	0.98	0.01	0.01	0,00186
0.97	0.03	0.01	0.97	0.03	0.01	0,00185
0.97	0.02	0.01	0.97	0.01	0.01	0,00185
0.99	0.01	0.01	0.81	0.12	0.07	0,00181
0.97	0.02	0.01	0.97	0.02	0.01	0,00181
0.97	0.01	0.02	0.97	0.01	0.01	0,0018
0.97	0.01	0.01	0.97	0.02	0.02	0,00177
0.98	0.01	0.01	0.97	0.03	0.01	0,00176
0.97	0.02	0.01	0.97	0.02	0.01	0,00173
0.97	0.02	0.01	0.97	0.03	0.01	0,00169
0.97	0.02	0.01	0.95	0.04	0.01	0,00167
0.97	0.01	0.01	0.97	0.01	0.01	0,00157
0.97	0.01	0.01	0.97	0.01	0.02	0,00157
0.97	0.02	0.01	0.97	0.01	0.02	0,00155
0.98	0.01	0.01	0.97	0.02	0.01	0,00154
0.98	0.01	0.01	0.97	0.01	0.02	0,00147
0.98	0.01	0.01	0.97	0.01	0.02	0,00142
0.97	0.01	0.02	0.98	0.01	0.01	0,00139
0.98	0.01	0.01	0.97	0.02	0.01	0,00138
0.98	0.01	0.01	0.97	0.02	0.01	0,00138
0.98	0.01	0.01	0.97	0.01	0.02	0,00138
0.98	0.01	0.01	0.97	0.02	0.01	0,00136
0.98	0.01	0.01	0.97	0.02	0.01	0,00134
0.98	0.01	0.01	0.97	0.01	0.01	0,00132
0.99	0.00	0.00	0.75	0.12	0.13	0,00131
0.97	0.01	0.01	0.97	0.01	0.01	0,00131
0.97	0.02	0.02	0.98	0.01	0.01	0,00129
0.98	0.01	0.01	0.97	0.01	0.02	0,00127

chr1(	92644719	674	C	0	0.99	0.00	0.01	0.98	0.01	0.01	0.98	0.01	0.01	0.98	0.01	0.01	0.98	0.01	0.01	0.98	0.00	0.01
chr1;	45706641	808	G	0	0.98	0.01	0.01	0.96	0.02	0.02	0.97	0.01	0.02	0.97	0.02	0.02	0.98	0.01	0.01	0.98	0.01	0.01
chr4	136547402	1049	G	0	0.94	0.06	0.00	0.97	0.01	0.01	0.97	0.03	0.00	0.96	0.00	0.04	0.95	0.03	0.01	0.97	0.03	0.00
chr1(	92644436	556	G	0	0.98	0.01	0.01	0.96	0.02	0.02	0.96	0.04	0.00	0.98	0.01	0.01	0.97	0.02	0.01	0.97	0.02	0.00
chr4	136547733	1201	C	0	0.98	0.02	0.00	0.95	0.04	0.01	0.97	0.03	0.01	0.98	0.00	0.02	0.98	0.02	0.00	0.95	0.05	0.00
chr1;	45706695	830	G	0	0.97	0.00	0.03	0.98	0.01	0.01	0.97	0.01	0.02	0.96	0.01	0.02	0.95	0.03	0.02	0.96	0.00	0.04
chr1!	24277745	1009	g	0	0.97	0.01	0.01	0.98	0.01	0.01	0.97	0.03	0.01	0.96	0.01	0.03	0.97	0.02	0.01	0.97	0.03	0.00
chr4	136547655	1183	C	0	0.97	0.02	0.01	0.97	0.02	0.01	0.95	0.05	0.01	0.97	0.03	0.00	0.98	0.00	0.02	0.94	0.06	0.00
chr5	64987345	1420	G	0	0.98	0.00	0.02	0.98	0.01	0.01	0.97	0.02	0.01	0.95	0.05	0.00	0.97	0.01	0.02	0.97	0.02	0.01
chr4	136547629	1176	C	0	0.98	0.01	0.01	0.96	0.02	0.02	0.98	0.01	0.01	0.94	0.03	0.03	0.97	0.01	0.02	0.98	0.01	0.02
chr1;	45706574	771	G	0	1.00	0.00	0.00	0.99	0.00	0.00	1.00	0.00	0.00	1.00	0.00	0.00	1.00	0.00	0.00	1.00	0.00	0.00
chr4	136547754	1211	C	0	0.97	0.00	0.03	0.97	0.02	0.01	0.94	0.05	0.00	0.96	0.00	0.04	0.95	0.02	0.04	0.96	0.04	0.00
chr8	120115741	1783	C	0	0.99	0.01	0.00	0.99	0.00	0.00	0.97	0.01	0.02	0.98	0.00	0.02	0.94	0.01	0.05	0.96	0.02	0.02
chr4	139783730	1301	C	0	0.97	0.01	0.03	0.97	0.02	0.01	0.95	0.02	0.03	0.98	0.01	0.01	0.93	0.06	0.02	0.98	0.00	0.01
chrX	7539460	2311	G	0	0.96	0.02	0.02	0.97	0.01	0.01	0.98	0.01	0.01	0.96	0.03	0.01	0.97	0.01	0.01	0.99	0.00	0.01
chr8	89376879	1613	c	0	0.97	0.02	0.01	0.97	0.01	0.02	0.98	0.02	0.00	0.98	0.01	0.01	0.97	0.02	0.01	0.97	0.03	0.00
chr1;	45706636	805	C	0	0.99	0.01	0.00	0.97	0.01	0.02	0.97	0.02	0.00	0.97	0.01	0.01	0.96	0.03	0.00	0.96	0.03	0.01
chrX	7539623	2407	G	0	0.96	0.00	0.04	0.92	0.08	0.00	0.96	0.02	0.02	0.94	0.05	0.00	0.95	0.00	0.04	0.96	0.00	0.04
chr1!	24277553	939	c	0	0.99	0.01	0.01	0.97	0.02	0.01	0.97	0.01	0.01	0.96	0.01	0.03	0.96	0.03	0.01	0.98	0.01	0.01
chr1!	24277707	999	G	0	0.96	0.04	0.00	0.97	0.01	0.01	0.98	0.02	0.00	0.99	0.01	0.01	0.97	0.01	0.01	0.98	0.01	0.01
chrX	7539388	2264	G	0	0.97	0.02	0.00	0.98	0.02	0.00	0.98	0.01	0.02	0.95	0.05	0.00	0.97	0.00	0.03	0.98	0.00	0.02
chrX	7539437	2295	G	0	0.97	0.01	0.02	0.97	0.01	0.02	0.95	0.02	0.03	0.98	0.01	0.01	0.98	0.01	0.01	0.98	0.01	0.01
chr4	136547553	1139	C	0	0.98	0.01	0.01	0.96	0.02	0.02	0.97	0.01	0.02	0.97	0.01	0.02	0.96	0.01	0.03	0.94	0.04	0.02
chr11	3525801	113	C	0	0.97	0.03	0.00	0.96	0.00	0.03	0.97	0.00	0.03	0.96	0.01	0.02	0.94	0.06	0.00	0.96	0.00	0.04
chr9	100498517	1933	C	0	0.95	0.00	0.05	0.97	0.00	0.02	0.97	0.03	0.00	0.96	0.03	0.01	0.97	0.00	0.03	0.95	0.04	0.00
chrX	7539467	2315	G	0	0.96	0.03	0.01	0.97	0.01	0.01	0.98	0.01	0.01	0.97	0.01	0.02	0.98	0.01	0.01	0.98	0.01	0.02
chrX	7539590	2388	G	0	0.97	0.03	0.00	0.98	0.02	0.00	0.96	0.04	0.00	0.93	0.07	0.00	0.97	0.00	0.03	0.96	0.04	0.00
chr1!	61868907	323	G	0	0.98	0.02	0.00	0.98	0.01	0.01	0.97	0.02	0.01	0.97	0.03	0.00	0.96	0.01	0.03	0.97	0.03	0.00
chr4	136547543	1132	C	0	0.99	0.00	0.01	0.98	0.01	0.01	0.95	0.01	0.04	0.97	0.02	0.01	0.99	0.00	0.01	0.98	0.01	0.02
chr1!	61868651	202	C	0	1.00	0.00	0.00	1.00	0.00	0.00	0.99	0.00	0.01	1.00	0.00	0.00	0.99	0.01	0.00	1.00	0.00	0.00
chrX	7539441	2298	G	0	0.98	0.01	0.01	0.96	0.02	0.02	0.95	0.02	0.03	0.97	0.02	0.01	0.98	0.01	0.01	0.98	0.01	0.01
chr5	64987475	1479	C	0	0.98	0.01	0.01	0.95	0.03	0.03	0.98	0.01	0.01	0.96	0.01	0.02	0.98	0.01	0.01	0.98	0.01	0.01
chr4	139783759	1317	G	0	0.98	0.00	0.02	0.98	0.00	0.01	0.98	0.02	0.00	0.96	0.04	0.00	0.97	0.03	0.00	0.97	0.03	0.00
chrX	7539570	2377	C	0	0.98	0.02	0.00	0.95	0.05	0.00	0.98	0.00	0.02	0.96	0.04	0.00	0.95	0.05	0.00	0.96	0.01	0.03
chr1!	61868786	266	C	0	0.98	0.01	0.00	0.99	0.01	0.01	0.97	0.02	0.00	0.96	0.04	0.00	0.97	0.03	0.00	0.97	0.02	0.01
chr4	139783728	1300	C	0	0.97	0.01	0.01	0.98	0.01	0.01	0.99	0.01	0.01	0.95	0.02	0.03	0.97	0.01	0.02	0.98	0.01	0.01
chrX	7539496	2333	C	0	0.99	0.00	0.00	0.98	0.01	0.01	0.98	0.01	0.00	0.97	0.01	0.02	0.93	0.07	0.00	0.97	0.01	0.02
chr1;	45706736	853	G	0	0.97	0.03	0.00	0.97	0.01	0.01	0.97	0.02	0.01	0.96	0.04	0.00	0.98	0.00	0.02	0.96	0.04	0.00
chrX	7539462	2312	G	0	0.98	0.01	0.02	0.97	0.01	0.01	0.97	0.01	0.02	0.97	0.01	0.01	0.97	0.02	0.01	0.98	0.01	0.01
chr1;	45706545	753	C	0	0.98	0.01	0.01	0.98	0.01	0.01	0.97	0.01	0.01	0.97	0.01	0.02	0.98	0.00	0.02	0.97	0.03	0.01
chr1;	45706741	855	G	0	1.00	0.00	0.00	0.99	0.00	0.00	1.00	0.00	0.00	0.99	0.00	0.00	1.00	0.00	0.00	1.00	0.00	0.00
chr1;	45706548	755	C	0	0.98	0.01	0.01	0.98	0.01	0.01	0.98	0.01	0.02	0.96	0.01	0.03	0.98	0.00	0.02	0.96	0.03	0.01
chr1;	45706485	722	G	0	1.00	0.00	0.00	1.00	0.00	0.00	0.99	0.00	0.00	0.98	0.02	0.00	1.00	0.00	0.00	0.99	0.00	0.01
chrX	7539453	2306	G	0	0.98	0.01	0.01	0.95	0.02	0.03	0.97	0.01	0.02	0.98	0.01	0.01	0.97	0.01	0.01	0.98	0.01	0.01
chr11	3525596	3	G	0	1.00	0.00	0.00	1.00	0.00	0.00	1.00	0.00	0.00	1.00	0.00	0.00	1.00	0.00	0.00	1.00	0.00	0.00
chr1!	24277674	985	G	0	0.96	0.02	0.02	0.98	0.01	0.01	0.97	0.00	0.03	0.96	0.03	0.01	0.98	0.00	0.01	0.97	0.02	0.00

0.98	0.01	0.01	0.92	0.03	0.05	0,00127
0.97	0.01	0.01	0.97	0.02	0.01	0,00126
0.97	0.02	0.01	0.98	0.01	0.01	0,00126
0.97	0.01	0.01	0.98	0.02	0.01	0,00124
0.97	0.02	0.01	0.98	0.01	0.01	0,00122
0.98	0.01	0.01	0.97	0.01	0.02	0,00121
0.98	0.01	0.01	0.98	0.01	0.01	0,0012
0.97	0.02	0.01	0.98	0.02	0.01	0,0012
0.98	0.01	0.01	0.97	0.02	0.01	0,00117
0.98	0.01	0.01	0.97	0.01	0.02	0,00117
0.99	0.01	0.01	0.77	0.01	0.22	0,00115
0.98	0.01	0.01	0.98	0.01	0.01	0,00113
0.98	0.01	0.01	0.97	0.01	0.02	0,00111
0.97	0.01	0.01	0.98	0.01	0.01	0,0011
0.97	0.01	0.01	0.98	0.01	0.01	0,00109
0.98	0.01	0.01	0.98	0.02	0.01	0,00108
0.98	0.01	0.01	0.97	0.02	0.01	0,00108
0.97	0.01	0.01	0.98	0.01	0.01	0,00105
0.98	0.01	0.01	0.98	0.01	0.01	0,00104
0.98	0.02	0.01	0.98	0.01	0.01	0,001
0.98	0.02	0.01	0.98	0.01	0.01	0,00099
0.97	0.01	0.01	0.98	0.01	0.01	0,00098
0.98	0.01	0.01	0.97	0.01	0.01	0,00097
0.98	0.01	0.01	0.98	0.01	0.01	0,00097
0.98	0.01	0.01	0.98	0.01	0.01	0,00097
0.98	0.01	0.01	0.98	0.01	0.01	0,00096
0.97	0.02	0.01	0.98	0.01	0.01	0,00096
0.98	0.01	0.01	0.98	0.02	0.01	0,00095
0.98	0.01	0.01	0.98	0.01	0.01	0,00095
0.99	0.00	0.01	0.89	0.04	0.07	0,00095
0.97	0.01	0.01	0.98	0.01	0.01	0,00095
0.98	0.01	0.01	0.98	0.01	0.01	0,00094
0.98	0.01	0.01	0.97	0.02	0.00	0,00094
0.98	0.01	0.01	0.97	0.02	0.01	0,00093
0.98	0.01	0.01	0.97	0.02	0.01	0,00093
0.98	0.01	0.01	0.98	0.01	0.01	0,00092
0.98	0.01	0.01	0.97	0.02	0.01	0,00092
0.98	0.01	0.01	0.98	0.01	0.01	0,00091
0.98	0.01	0.01	0.98	0.01	0.01	0,0009
0.98	0.01	0.01	0.98	0.01	0.01	0,0009
0.99	0.00	0.01	0.83	0.09	0.08	0,0009
0.98	0.01	0.01	0.98	0.01	0.01	0,00089
0.99	0.01	0.00	0.91	0.05	0.05	0,00089
0.98	0.01	0.01	0.98	0.01	0.01	0,00088
0.99	0.00	0.00	0.82	0.10	0.09	0,00088
0.98	0.01	0.01	0.98	0.01	0.01	0,00087

chr4	136547731	1200	C	0	0.97	0.03	0.00	0.97	0.01	0.01	0.94	0.00	0.06	0.95	0.05	0.00	0.96	0.04	0.00	0.96	0.00	0.04
chrX	7539410	2277	G	0	0.97	0.01	0.02	0.98	0.01	0.01	0.95	0.02	0.03	0.98	0.01	0.01	0.98	0.01	0.01	0.98	0.01	0.01
chr1	3525905	176	G	0	0.98	0.01	0.01	0.99	0.01	0.00	0.98	0.00	0.01	0.97	0.02	0.01	0.98	0.02	0.00	0.97	0.03	0.00
chr8	89376891	1620	g	0	0.98	0.02	0.00	0.98	0.02	0.00	0.98	0.02	0.01	0.95	0.05	0.00	0.97	0.03	0.00	0.98	0.01	0.01
chr5	64987604	1531	C	0	0.98	0.00	0.02	0.99	0.00	0.01	0.97	0.00	0.03	0.97	0.03	0.00	0.98	0.01	0.01	0.98	0.00	0.02
chr4	136547740	1204	C	0	0.98	0.00	0.01	0.99	0.00	0.01	0.96	0.00	0.04	0.97	0.03	0.00	0.99	0.01	0.00	0.97	0.00	0.03
chrX	7476506	2076	G	1	0.98	0.01	0.01	0.98	0.01	0.01	0.98	0.01	0.01	0.96	0.01	0.02	0.98	0.01	0.01	0.98	0.01	0.01
chr1	24277524	924	c	0	0.99	0.00	0.01	0.98	0.01	0.01	0.98	0.02	0.01	0.95	0.04	0.01	0.98	0.01	0.01	0.98	0.01	0.00
chrX	7539463	2313	G	0	0.98	0.01	0.01	0.98	0.01	0.01	0.97	0.01	0.02	0.98	0.01	0.01	0.98	0.01	0.01	0.99	0.01	0.00
chrX	7539562	2371	G	0	0.97	0.03	0.00	0.98	0.02	0.00	0.98	0.02	0.00	0.97	0.00	0.03	0.99	0.00	0.01	0.97	0.03	0.00
chr1	92644713	671	C	0	0.99	0.00	0.01	0.96	0.02	0.02	0.96	0.01	0.03	0.95	0.00	0.05	0.98	0.01	0.01	0.98	0.01	0.01
chr8	89376973	1653	G	0	0.98	0.00	0.02	0.99	0.00	0.01	0.98	0.01	0.01	0.97	0.03	0.00	0.99	0.01	0.00	0.99	0.01	0.00
chr4	136547581	1154	C	0	0.98	0.00	0.01	0.98	0.01	0.01	0.98	0.01	0.01	0.98	0.01	0.01	0.93	0.05	0.01	0.97	0.01	0.02
chr4	139783742	1307	C	0	0.97	0.02	0.01	0.98	0.01	0.01	0.99	0.01	0.01	0.99	0.00	0.01	0.96	0.03	0.02	0.97	0.02	0.00
chr8	120115629	1729	G	0	0.98	0.01	0.01	0.98	0.01	0.01	0.98	0.01	0.00	0.98	0.01	0.02	0.97	0.03	0.00	0.98	0.01	0.00
chr4	139783802	1341	C	0	0.99	0.00	0.01	0.98	0.02	0.00	0.99	0.01	0.00	0.96	0.04	0.00	0.97	0.02	0.01	0.95	0.05	0.00
chr4	136547632	1177	C	0	0.97	0.01	0.02	0.97	0.01	0.02	0.98	0.01	0.01	0.97	0.01	0.02	0.97	0.01	0.01	0.99	0.01	0.01
chr8	120115716	1772	C	0	0.98	0.01	0.01	0.98	0.01	0.01	0.97	0.02	0.01	0.99	0.00	0.01	0.91	0.08	0.00	0.98	0.00	0.02
chr4	139783875	1383	G	0	0.97	0.03	0.00	0.98	0.00	0.02	0.97	0.02	0.01	0.97	0.01	0.02	0.96	0.04	0.00	0.96	0.04	0.00
chr1	61868854	299	C	0	0.98	0.02	0.00	0.99	0.00	0.01	0.97	0.02	0.01	0.94	0.06	0.00	0.97	0.03	0.00	0.97	0.00	0.03
chr1	100300279	455	C	0	0.98	0.01	0.01	0.96	0.02	0.02	0.97	0.01	0.02	0.96	0.01	0.02	0.98	0.01	0.01	0.98	0.01	0.01
chr4	136547416	1055	G	0	0.98	0.02	0.00	0.98	0.02	0.00	0.97	0.03	0.00	0.97	0.01	0.02	0.98	0.00	0.02	0.98	0.02	0.00
chr8	120115752	1787	C	0	0.98	0.00	0.02	0.99	0.01	0.00	0.94	0.05	0.00	0.99	0.00	0.01	0.97	0.00	0.03	0.97	0.00	0.03
chr1	92644520	617	C	0	0.97	0.00	0.03	0.99	0.00	0.01	0.97	0.02	0.01	0.97	0.02	0.01	0.97	0.00	0.02	0.98	0.01	0.01
chr8	120115686	1756	C	0	0.97	0.03	0.00	0.98	0.01	0.01	0.99	0.01	0.01	0.98	0.01	0.02	0.98	0.00	0.01	0.98	0.01	0.01
chrX	7476435	2038	G	1	0.99	0.01	0.01	0.98	0.01	0.01	0.99	0.01	0.01	0.96	0.01	0.03	0.98	0.01	0.01	0.97	0.01	0.02
chr1	92644700	661	C	0	0.99	0.01	0.01	0.98	0.01	0.01	0.98	0.01	0.01	0.97	0.01	0.02	0.98	0.00	0.01	0.97	0.01	0.01
chr1	45706547	754	G	0	0.98	0.02	0.00	0.98	0.01	0.01	0.98	0.01	0.01	0.97	0.02	0.00	0.97	0.03	0.00	0.97	0.00	0.03
chr5	64987536	1505	G	0	0.98	0.01	0.00	0.97	0.01	0.01	0.98	0.01	0.01	0.94	0.06	0.00	0.98	0.00	0.02	0.97	0.03	0.00
chr1	92644754	684	C	0	0.97	0.01	0.02	0.97	0.01	0.02	0.98	0.01	0.01	0.99	0.00	0.01	0.98	0.01	0.01	0.98	0.01	0.02
chrX	7539419	2284	G	0	0.98	0.01	0.01	0.97	0.01	0.01	0.98	0.01	0.01	0.98	0.01	0.01	0.95	0.03	0.02	0.98	0.01	0.01
chrX	7539427	2289	G	0	0.98	0.01	0.01	0.96	0.02	0.02	0.97	0.01	0.01	0.98	0.01	0.01	0.98	0.01	0.01	0.99	0.00	0.01
chr9	100498469	1904	G	0	0.98	0.02	0.00	0.98	0.02	0.00	0.98	0.01	0.00	0.97	0.02	0.01	0.98	0.02	0.00	0.98	0.02	0.00
chr8	120115604	1715	G	0	0.99	0.00	0.01	0.98	0.01	0.01	0.98	0.01	0.00	0.98	0.02	0.00	0.99	0.01	0.00	0.99	0.01	0.00
chr1	45706650	812	G	0	0.97	0.02	0.01	0.98	0.01	0.01	0.99	0.00	0.01	0.97	0.01	0.02	0.97	0.02	0.01	0.98	0.00	0.01
chr1	100300289	459	C	0	0.98	0.01	0.01	0.97	0.02	0.02	0.97	0.01	0.02	0.95	0.02	0.04	0.98	0.01	0.01	0.99	0.00	0.01
chrX	7476511	2080	G	0	0.98	0.01	0.01	0.97	0.01	0.01	0.98	0.01	0.01	0.98	0.01	0.02	0.97	0.01	0.01	0.99	0.00	0.01
chrX	7476531	2095	G	1	0.96	0.02	0.02	0.98	0.01	0.01	0.98	0.01	0.01	0.98	0.01	0.01	0.98	0.01	0.01	0.97	0.01	0.02
chr8	89376862	1603	c	0	0.98	0.02	0.00	0.97	0.02	0.00	0.99	0.01	0.01	0.98	0.00	0.02	0.98	0.00	0.01	0.97	0.03	0.00
chr1	45706472	713	C	0	0.99	0.00	0.01	0.98	0.00	0.01	0.99	0.01	0.01	0.97	0.03	0.00	0.98	0.00	0.02	0.98	0.02	0.00
chr1	61868773	261	G	0	1.00	0.00	0.00	1.00	0.00	0.00	0.99	0.00	0.00	0.99	0.00	0.00	0.99	0.01	0.00	0.99	0.00	0.00
chrX	7539474	2319	G	0	0.98	0.01	0.01	0.98	0.01	0.01	0.98	0.01	0.01	0.97	0.02	0.02	0.98	0.01	0.01	0.99	0.01	0.01
chr1	3525611	11	G	0	0.98	0.02	0.00	0.99	0.01	0.01	0.99	0.01	0.01	0.94	0.06	0.00	0.96	0.04	0.00	0.99	0.00	0.00
chr4	136547578	1152	C	0	0.98	0.01	0.01	0.97	0.01	0.02	0.98	0.01	0.01	0.98	0.01	0.01	0.98	0.01	0.01	0.99	0.00	0.01
chrX	7476478	2058	G	1	0.98	0.01	0.01	0.97	0.01	0.02	0.99	0.00	0.01	0.96	0.01	0.03	0.98	0.01	0.01	0.99	0.00	0.01
chr9	100498357	1849	G	0	0.98	0.01	0.01	0.98	0.01	0.00	0.98	0.00	0.01	0.98	0.01	0.01	0.98	0.00	0.02	0.98	0.02	0.00

0.98	0.01	0.01	0.98	0.01	0.01	0,00087
0.98	0.01	0.01	0.98	0.01	0.01	0,00086
0.98	0.01	0.01	0.98	0.02	0.01	0,00086
0.98	0.01	0.01	0.97	0.02	0.01	0,00086
0.98	0.01	0.02	0.98	0.01	0.01	0,00086
0.98	0.01	0.01	0.98	0.01	0.01	0,00085
0.98	0.01	0.01	0.98	0.01	0.01	0,00085
0.98	0.01	0.01	0.98	0.01	0.01	0,00084
0.98	0.01	0.01	0.98	0.01	0.01	0,00084
0.98	0.02	0.00	0.98	0.01	0.01	0,00084
0.98	0.01	0.01	0.98	0.01	0.01	0,00083
0.98	0.01	0.01	0.98	0.01	0.01	0,00083
0.98	0.01	0.01	0.97	0.01	0.01	0,00082
0.98	0.01	0.01	0.98	0.01	0.01	0,00082
0.98	0.01	0.01	0.98	0.01	0.01	0,00082
0.99	0.01	0.01	0.97	0.02	0.01	0,00081
0.98	0.01	0.01	0.98	0.01	0.01	0,00081
0.98	0.01	0.01	0.98	0.01	0.01	0,0008
0.98	0.01	0.01	0.97	0.02	0.01	0,00079
0.98	0.01	0.01	0.98	0.01	0.01	0,00079
0.98	0.01	0.01	0.98	0.01	0.01	0,00079
0.98	0.02	0.01	0.98	0.01	0.01	0,00079
0.98	0.01	0.01	0.98	0.01	0.02	0,00078
0.98	0.01	0.01	0.98	0.01	0.01	0,00077
0.98	0.01	0.01	0.98	0.01	0.01	0,00077
0.98	0.01	0.01	0.98	0.01	0.01	0,00075
0.98	0.01	0.01	0.98	0.01	0.01	0,00075
0.98	0.01	0.01	0.98	0.01	0.01	0,00075
0.98	0.01	0.01	0.98	0.01	0.01	0,00074
0.98	0.01	0.01	0.98	0.01	0.01	0,00074
0.98	0.01	0.01	0.98	0.01	0.01	0,00073
0.98	0.01	0.01	0.98	0.01	0.01	0,00073
0.98	0.01	0.01	0.98	0.02	0.01	0,00072
0.98	0.01	0.01	0.98	0.01	0.00	0,00072
0.98	0.01	0.01	0.98	0.01	0.01	0,00072
0.98	0.01	0.01	0.98	0.01	0.01	0,00071
0.98	0.01	0.01	0.98	0.01	0.01	0,00071
0.98	0.01	0.01	0.98	0.01	0.01	0,00071
0.98	0.01	0.01	0.98	0.01	0.01	0,00071
0.98	0.01	0.01	0.98	0.01	0.01	0,0007
0.94	0.05	0.01	0.99	0.00	0.01	0,0007
0.98	0.01	0.01	0.98	0.01	0.01	0,0007
0.98	0.01	0.01	0.98	0.02	0.00	0,0007
0.98	0.01	0.01	0.98	0.01	0.01	0,0007
0.98	0.01	0.01	0.98	0.01	0.01	0,00069
0.98	0.01	0.01	0.97	0.01	0.02	0,00069

chr15	100300243	450	G	0	1.00	0.00	0.00	1.00	0.00	0.00	1.00	0.00	0.00	1.00	0.00	0.00	0.99	0.01	0.00	1.00	0.00	0.00
chrX	7539425	2287	G	0	0.99	0.01	0.01	0.97	0.01	0.01	0.98	0.01	0.01	0.98	0.01	0.02	0.98	0.01	0.01	0.98	0.01	0.01
chr11	3525935	186	G	0	0.97	0.01	0.02	0.98	0.01	0.01	0.99	0.01	0.01	0.96	0.00	0.03	0.98	0.01	0.01	0.96	0.03	0.00
chr4	136547636	1178	C	0	0.99	0.00	0.00	0.97	0.01	0.01	0.98	0.01	0.02	0.98	0.01	0.01	0.98	0.01	0.01	0.99	0.00	0.01
chr19	24277610	966	G	0	0.97	0.03	0.00	0.97	0.01	0.02	0.97	0.02	0.00	0.98	0.02	0.01	0.98	0.00	0.02	0.97	0.03	0.00
chr9	100498533	1941	G	0	0.98	0.01	0.01	0.99	0.01	0.00	0.98	0.01	0.01	0.98	0.01	0.01	0.98	0.00	0.02	0.98	0.02	0.00
chr4	136547405	1050	G	0	0.96	0.04	0.00	0.98	0.02	0.00	0.98	0.01	0.01	0.98	0.01	0.00	0.99	0.00	0.01	0.98	0.02	0.00
chrX	7476517	2085	G	1	0.98	0.01	0.01	0.97	0.01	0.01	0.99	0.01	0.01	0.98	0.01	0.01	0.98	0.01	0.01	0.99	0.01	0.01
chr4	139783812	1346	G	0	0.99	0.00	0.01	0.99	0.01	0.00	0.99	0.01	0.00	0.98	0.02	0.00	0.97	0.03	0.00	0.98	0.02	0.01
chrX	7539426	2288	G	0	0.98	0.01	0.01	0.97	0.01	0.01	0.98	0.01	0.01	0.98	0.01	0.01	0.98	0.01	0.01	0.98	0.01	0.01
chrX	7476465	2051	G	0	0.98	0.01	0.01	0.98	0.01	0.01	0.99	0.00	0.01	0.97	0.01	0.02	0.97	0.01	0.01	0.99	0.00	0.01
chr4	136547611	1169	C	0	0.99	0.00	0.01	0.97	0.01	0.02	0.97	0.01	0.02	0.97	0.01	0.02	0.97	0.02	0.01	0.99	0.00	0.01
chrX	7476476	2056	G	0	0.98	0.01	0.01	0.98	0.01	0.01	0.98	0.01	0.01	0.98	0.01	0.02	0.98	0.01	0.01	0.99	0.01	0.01
chrX	7476543	2106	G	1	0.98	0.01	0.01	0.98	0.01	0.01	0.99	0.01	0.01	0.98	0.01	0.01	0.97	0.01	0.01	0.97	0.01	0.02
chrX	7539446	2301	G	0	0.98	0.01	0.01	0.97	0.01	0.01	0.98	0.01	0.01	0.98	0.01	0.01	0.97	0.02	0.02	0.98	0.01	0.01
chr17	45706677	824	G	0	0.98	0.02	0.00	1.00	0.00	0.00	0.98	0.01	0.01	0.96	0.04	0.00	0.97	0.01	0.01	0.97	0.03	0.00
chr15	100300290	460	C	0	0.98	0.01	0.01	0.97	0.02	0.02	0.97	0.01	0.02	0.97	0.01	0.02	0.98	0.01	0.01	0.99	0.00	0.01
chrX	7476518	2086	G	0	0.98	0.01	0.01	0.98	0.01	0.01	0.98	0.01	0.01	0.98	0.01	0.02	0.98	0.01	0.01	0.99	0.00	0.00
chrX	7476526	2092	G	0	0.98	0.01	0.01	0.98	0.01	0.01	0.99	0.01	0.01	0.98	0.01	0.01	0.98	0.01	0.01	0.99	0.00	0.01
chr16	92644580	647	C	0	0.98	0.00	0.02	0.98	0.01	0.01	0.99	0.00	0.01	0.98	0.01	0.02	0.96	0.03	0.01	0.99	0.00	0.01
chr16	92644722	677	C	0	0.99	0.00	0.00	0.90	0.07	0.03	0.98	0.01	0.01	0.99	0.00	0.01	0.99	0.00	0.01	0.99	0.00	0.01
chr4	136547577	1151	C	0	0.99	0.00	0.01	0.98	0.01	0.01	0.98	0.01	0.01	0.98	0.01	0.01	0.98	0.01	0.01	0.98	0.01	0.01
chr4	136547560	1142	G	0	0.98	0.01	0.01	0.99	0.00	0.01	0.98	0.02	0.00	0.96	0.04	0.00	0.97	0.03	0.00	0.98	0.02	0.00
chr8	89376930	1637	C	0	0.99	0.01	0.01	0.98	0.00	0.01	0.98	0.01	0.01	0.99	0.00	0.01	0.98	0.02	0.00	0.96	0.04	0.00
chrX	7539398	2269	G	0	1.00	0.00	0.00	0.99	0.00	0.00	0.99	0.00	0.00	0.99	0.00	0.01	0.99	0.00	0.00	0.99	0.00	0.00
chrX	7476494	2068	G	0	0.98	0.01	0.01	0.98	0.01	0.01	0.98	0.01	0.01	0.97	0.01	0.02	0.97	0.01	0.02	0.99	0.01	0.00
chr8	89376859	1601	c	0	0.99	0.00	0.01	0.98	0.01	0.01	0.99	0.01	0.00	0.99	0.01	0.00	0.97	0.03	0.00	0.98	0.01	0.00
chr17	45706717	840	G	0	0.96	0.04	0.00	0.99	0.00	0.01	0.98	0.02	0.00	0.98	0.02	0.00	0.98	0.00	0.02	0.98	0.00	0.01
chr16	92644551	632	C	0	1.00	0.00	0.00	0.98	0.01	0.01	0.99	0.00	0.01	0.86	0.13	0.01	0.98	0.00	0.02	0.99	0.00	0.00
chrX	7476507	2077	G	0	0.98	0.01	0.01	0.97	0.01	0.01	0.98	0.01	0.01	0.97	0.01	0.02	0.98	0.01	0.01	0.99	0.00	0.01
chrX	7476496	2070	G	1	0.98	0.01	0.01	0.97	0.01	0.01	0.98	0.01	0.01	0.97	0.01	0.02	0.98	0.01	0.01	0.99	0.00	0.00
chr16	92644511	611	C	0	0.98	0.01	0.01	0.96	0.03	0.02	0.99	0.00	0.01	0.99	0.01	0.01	0.97	0.00	0.02	0.99	0.00	0.00
chr4	136547587	1157	C	0	0.97	0.01	0.02	0.98	0.01	0.01	0.99	0.01	0.01	0.98	0.01	0.01	0.97	0.01	0.02	0.99	0.00	0.01
chr4	136547605	1167	C	0	0.98	0.01	0.01	0.97	0.01	0.01	0.98	0.01	0.01	0.98	0.01	0.01	0.98	0.01	0.01	0.99	0.00	0.01
chr11	3525708	57	G	0	0.99	0.00	0.01	0.99	0.00	0.00	0.99	0.01	0.01	0.98	0.02	0.00	0.98	0.02	0.00	0.98	0.01	0.00
chr19	24277588	953	g	0	0.98	0.00	0.01	0.98	0.01	0.01	0.99	0.01	0.00	0.98	0.02	0.01	0.98	0.01	0.01	0.98	0.01	0.00
chrX	7476486	2063	G	0	0.98	0.01	0.01	0.98	0.01	0.01	0.99	0.01	0.01	0.98	0.01	0.02	0.98	0.01	0.01	0.99	0.00	0.00
chr8	89377060	1686	G	0	0.99	0.00	0.01	0.98	0.02	0.00	0.98	0.01	0.00	0.95	0.05	0.00	0.98	0.02	0.00	0.98	0.00	0.01
chrX	7476508	2078	G	0	0.98	0.01	0.01	0.98	0.01	0.01	0.99	0.00	0.01	0.97	0.01	0.02	0.98	0.01	0.01	0.99	0.00	0.01
chr5	64987333	1414	G	0	0.98	0.01	0.00	0.99	0.00	0.01	0.98	0.00	0.01	0.98	0.00	0.02	0.97	0.00	0.02	0.96	0.04	0.00
chr8	120115807	1812	G	0	0.99	0.01	0.00	0.98	0.01	0.00	0.98	0.02	0.00	0.98	0.01	0.01	0.98	0.02	0.00	0.97	0.03	0.00
chr9	100498460	1899	G	0	0.97	0.02	0.01	0.98	0.02	0.01	0.98	0.01	0.01	0.97	0.03	0.00	0.97	0.03	0.00	0.98	0.00	0.02
chr11	3525848	139	G	0	0.98	0.02	0.00	0.99	0.00	0.00	0.99	0.01	0.01	0.98	0.02	0.01	0.98	0.01	0.00	0.98	0.01	0.01
chrX	7476521	2089	G	0	0.98	0.01	0.01	0.98	0.01	0.01	0.99	0.00	0.01	0.98	0.01	0.01	0.98	0.01	0.01	0.99	0.00	0.00
chrX	7476513	2082	G	0	0.98	0.01	0.01	0.98	0.01	0.01	0.99	0.00	0.01	0.98	0.01	0.01	0.98	0.01	0.01	0.99	0.00	0.01
chr5	64987438	1458	G	0	0.98	0.01	0.01	0.97	0.02	0.01	0.98	0.01	0.01	0.99	0.01	0.01	0.98	0.01	0.01	0.99	0.00	0.00

0.99	0.00	0.00	0.91	0.04	0.05	0,00069
0.98	0.01	0.01	0.98	0.01	0.01	0,00069
0.98	0.01	0.01	0.98	0.01	0.01	0,00069
0.98	0.01	0.01	0.98	0.01	0.01	0,00069
0.97	0.02	0.01	0.98	0.01	0.01	0,00068
0.98	0.01	0.01	0.98	0.01	0.01	0,00068
0.97	0.02	0.01	0.98	0.01	0.01	0,00068
0.98	0.01	0.01	0.98	0.01	0.01	0,00068
0.99	0.01	0.01	0.98	0.02	0.00	0,00068
0.98	0.01	0.01	0.98	0.01	0.01	0,00068
0.98	0.01	0.01	0.98	0.01	0.01	0,00067
0.98	0.01	0.01	0.98	0.01	0.01	0,00067
0.98	0.01	0.01	0.98	0.01	0.01	0,00067
0.98	0.01	0.01	0.98	0.01	0.01	0,00067
0.98	0.01	0.01	0.98	0.01	0.01	0,00067
0.99	0.01	0.01	0.97	0.02	0.01	0,00067
0.98	0.01	0.01	0.98	0.01	0.01	0,00067
0.98	0.01	0.01	0.98	0.01	0.01	0,00066
0.98	0.01	0.01	0.97	0.01	0.02	0,00066
0.98	0.01	0.01	0.98	0.01	0.01	0,00066
0.98	0.01	0.01	0.99	0.00	0.01	0,00066
0.98	0.01	0.01	0.98	0.01	0.01	0,00066
0.99	0.01	0.01	0.97	0.02	0.00	0,00065
0.98	0.01	0.01	0.98	0.01	0.01	0,00065
0.99	0.00	0.00	0.95	0.03	0.03	0,00065
0.98	0.01	0.01	0.98	0.01	0.01	0,00065
0.98	0.01	0.01	0.98	0.01	0.01	0,00065
0.98	0.01	0.01	0.98	0.01	0.01	0,00064
0.99	0.01	0.01	0.98	0.01	0.01	0,00064
0.98	0.01	0.01	0.98	0.01	0.01	0,00064
0.98	0.01	0.01	0.98	0.01	0.01	0,00064
0.98	0.01	0.01	0.98	0.01	0.01	0,00064
0.98	0.01	0.01	0.98	0.01	0.01	0,00064
0.98	0.01	0.01	0.98	0.01	0.01	0,00064
0.98	0.01	0.01	0.98	0.01	0.01	0,00063
0.98	0.01	0.01	0.98	0.01	0.01	0,00063
0.98	0.01	0.01	0.98	0.01	0.01	0,00063
0.98	0.01	0.01	0.98	0.01	0.01	0,00062
0.98	0.01	0.01	0.98	0.01	0.01	0,00062
0.98	0.01	0.01	0.98	0.01	0.01	0,00062
0.98	0.02	0.01	0.98	0.02	0.00	0,00062
0.98	0.01	0.01	0.98	0.01	0.01	0,00062
0.98	0.01	0.01	0.98	0.01	0.01	0,00062
0.98	0.01	0.01	0.98	0.01	0.01	0,00061
0.98	0.01	0.01	0.98	0.01	0.01	0,00061
0.98	0.01	0.01	0.99	0.01	0.01	0,00061

chrX	7476484	2062	G	0	0.98	0.01	0.01	0.98	0.01	0.01	0.99	0.00	0.01	0.98	0.01	0.02	0.98	0.01	0.01	0.99	0.01	0.00
chr1(	92644712	670	C	0	0.99	0.00	0.01	0.98	0.01	0.01	0.99	0.01	0.01	0.97	0.01	0.02	0.98	0.01	0.01	0.97	0.01	0.01
chr1(	92644476	584	G	0	0.97	0.02	0.01	0.99	0.01	0.01	0.98	0.01	0.00	0.98	0.01	0.01	0.98	0.01	0.01	0.99	0.01	0.00
chr4	136547492	1099	G	0	0.98	0.00	0.02	0.99	0.00	0.01	0.98	0.02	0.00	0.97	0.03	0.00	0.97	0.03	0.00	0.98	0.01	0.01
chr4	136547576	1150	C	0	0.99	0.00	0.01	0.98	0.01	0.01	0.98	0.01	0.01	0.98	0.01	0.01	0.97	0.01	0.02	0.99	0.00	0.01
chr4	136547618	1172	C	0	0.99	0.00	0.01	0.97	0.01	0.02	0.98	0.01	0.01	0.96	0.02	0.02	0.98	0.01	0.01	0.99	0.00	0.01
chr4	136547562	1144	C	0	0.99	0.00	0.01	0.96	0.02	0.02	0.98	0.01	0.01	0.96	0.01	0.03	0.98	0.01	0.01	0.98	0.01	0.01
chr4	136547570	1149	C	0	0.99	0.00	0.01	0.98	0.01	0.01	0.97	0.02	0.02	0.98	0.01	0.01	0.98	0.01	0.01	0.99	0.00	0.01
chrX	7476537	2101	G	1	0.97	0.01	0.01	0.98	0.01	0.01	0.99	0.00	0.01	0.98	0.01	0.01	0.98	0.01	0.01	0.99	0.00	0.01
chr8	120115707	1767	C	0	0.99	0.01	0.00	0.98	0.01	0.01	0.97	0.01	0.02	0.99	0.00	0.01	0.98	0.00	0.01	0.98	0.01	0.01
chrX	7476519	2087	G	0	0.98	0.01	0.01	0.98	0.01	0.01	0.98	0.01	0.01	0.98	0.01	0.02	0.98	0.01	0.01	0.99	0.00	0.00
chr4	136547536	1127	G	0	0.98	0.01	0.02	0.98	0.01	0.01	0.99	0.01	0.00	0.98	0.00	0.02	0.97	0.03	0.00	0.98	0.00	0.02
chr4	139783744	1308	C	0	0.99	0.00	0.00	0.99	0.01	0.01	0.99	0.00	0.01	0.99	0.00	0.01	0.99	0.00	0.01	0.99	0.00	0.00
chr8	120115786	1803	C	0	0.98	0.01	0.01	0.98	0.01	0.01	0.96	0.03	0.01	0.99	0.01	0.00	0.99	0.01	0.00	0.98	0.01	0.01
chrX	7539438	2296	G	0	0.98	0.01	0.01	0.97	0.01	0.01	0.98	0.01	0.01	0.98	0.01	0.01	0.98	0.01	0.01	0.98	0.01	0.01
chrX	7539472	2318	C	0	0.99	0.01	0.00	0.97	0.03	0.00	0.97	0.00	0.02	0.96	0.04	0.00	0.98	0.00	0.02	0.97	0.01	0.02
chr5	64987508	1494	G	0	1.00	0.00	0.00	1.00	0.00	0.00	0.99	0.00	0.00	0.99	0.00	0.01	1.00	0.00	0.00	1.00	0.00	0.00
chrX	7476490	2065	G	0	0.98	0.01	0.01	0.97	0.01	0.01	0.99	0.00	0.01	0.97	0.01	0.02	0.98	0.01	0.01	0.99	0.00	0.00
chr1(	24277600	961	C	0	0.99	0.01	0.01	0.98	0.00	0.01	0.98	0.01	0.01	0.98	0.00	0.02	0.99	0.00	0.01	0.99	0.00	0.01
chr5	64987310	1404	G	0	0.99	0.00	0.01	0.98	0.01	0.01	0.99	0.01	0.01	0.99	0.00	0.01	0.98	0.00	0.02	0.98	0.01	0.00
chrX	7476520	2088	G	0	0.98	0.01	0.01	0.98	0.01	0.01	0.98	0.01	0.01	0.97	0.01	0.02	0.98	0.01	0.01	0.99	0.00	0.00
chr8	120115614	1720	G	0	0.98	0.01	0.00	0.99	0.01	0.00	0.98	0.01	0.01	0.98	0.02	0.00	0.97	0.03	0.00	0.97	0.03	0.00
chr9	100498531	1940	G	0	0.98	0.01	0.00	0.98	0.02	0.01	0.98	0.01	0.01	0.98	0.01	0.01	0.97	0.00	0.03	0.98	0.02	0.00
chrX	7476493	2067	G	0	0.98	0.01	0.01	0.98	0.01	0.01	0.99	0.01	0.01	0.96	0.01	0.03	0.98	0.01	0.01	0.99	0.01	0.01
chr1(	92644353	526	G	0	0.99	0.00	0.00	1.00	0.00	0.00	1.00	0.00	0.00	0.99	0.00	0.00	1.00	0.00	0.00	1.00	0.00	0.00
chrX	7539415	2280	G	0	0.99	0.01	0.01	0.98	0.01	0.01	0.97	0.01	0.02	0.98	0.01	0.01	0.97	0.01	0.01	0.99	0.00	0.01
chrX	7539435	2294	C	0	0.97	0.00	0.03	0.97	0.02	0.01	0.98	0.02	0.00	0.98	0.00	0.02	0.97	0.00	0.03	0.98	0.02	0.00
chrX	7476471	2054	G	1	0.98	0.01	0.01	0.98	0.01	0.01	0.99	0.00	0.01	0.96	0.01	0.03	0.98	0.01	0.01	0.99	0.01	0.00
chr4	136547612	1170	C	0	0.99	0.00	0.01	0.97	0.01	0.02	0.98	0.01	0.01	0.97	0.01	0.02	0.98	0.01	0.01	0.99	0.00	0.01
chr4	136547722	1197	C	0	0.98	0.02	0.00	0.99	0.01	0.00	0.99	0.00	0.01	0.98	0.01	0.01	0.98	0.00	0.02	0.95	0.05	0.00
chrX	7539355	2245	G	0	0.98	0.01	0.01	0.99	0.01	0.00	0.99	0.00	0.01	0.98	0.01	0.01	0.97	0.03	0.00	0.98	0.01	0.01
chr1!	100300129	415	C	0	0.98	0.00	0.01	0.98	0.01	0.01	0.99	0.00	0.00	0.98	0.01	0.01	0.98	0.01	0.02	0.98	0.00	0.02
chr4	136547653	1182	C	0	0.97	0.02	0.00	0.99	0.00	0.01	0.98	0.01	0.01	0.99	0.00	0.01	0.99	0.00	0.00	0.97	0.03	0.00
chrX	7476456	2046	G	0	0.98	0.01	0.01	0.98	0.01	0.01	0.99	0.00	0.01	0.98	0.01	0.02	0.98	0.01	0.01	0.99	0.00	0.00
chrX	7539468	2316	G	0	0.99	0.01	0.01	0.97	0.01	0.02	0.98	0.01	0.01	0.98	0.01	0.01	0.98	0.01	0.01	0.99	0.00	0.01
chr4	139783690	1281	C	0	0.98	0.01	0.01	0.98	0.02	0.00	0.98	0.02	0.00	0.99	0.00	0.01	0.97	0.02	0.01	0.97	0.03	0.00
chr1(	24277627	974	G	0	0.99	0.00	0.00	0.98	0.01	0.01	0.97	0.01	0.02	0.97	0.02	0.01	0.99	0.00	0.01	0.98	0.01	0.01
chr1!	100300296	463	C	0	0.98	0.01	0.01	0.97	0.02	0.02	0.97	0.01	0.02	0.97	0.01	0.02	0.99	0.01	0.01	0.99	0.00	0.00
chrX	7476492	2066	G	0	0.97	0.01	0.02	0.98	0.01	0.01	0.99	0.00	0.01	0.97	0.01	0.02	0.98	0.01	0.01	0.99	0.00	0.01
chr1(	24277705	998	G	0	0.97	0.03	0.00	0.98	0.01	0.01	0.98	0.01	0.01	0.98	0.01	0.00	0.99	0.01	0.00	0.99	0.00	0.01
chr4	139783791	1335	C	0	0.99	0.00	0.01	0.98	0.01	0.00	0.99	0.00	0.01	0.92	0.07	0.00	0.97	0.01	0.01	0.99	0.00	0.01
chr4	136547438	1068	G	0	0.98	0.01	0.01	0.98	0.02	0.00	0.99	0.01	0.00	0.97	0.03	0.00	0.98	0.02	0.00	0.99	0.01	0.00
chr4	139783740	1305	C	0	0.98	0.01	0.01	0.98	0.01	0.01	0.99	0.00	0.01	0.98	0.01	0.01	0.99	0.00	0.01	0.98	0.01	0.01
chr5	64987486	1484	C	0	0.98	0.01	0.01	0.98	0.01	0.01	0.99	0.00	0.01	0.98	0.01	0.02	0.99	0.00	0.01	0.99	0.00	0.01
chr1(	24277636	977	C	0	0.99	0.01	0.00	0.98	0.01	0.01	0.98	0.02	0.00	0.98	0.02	0.00	0.99	0.00	0.00	0.99	0.01	0.00
chrX	7476504	2074	G	0	0.98	0.01	0.01	0.97	0.01	0.01	0.98	0.01	0.01	0.98	0.01	0.01	0.98	0.01	0.01	0.99	0.00	0.00

0.98	0.01	0.01	0.98	0.01	0.01	0,00061
0.98	0.01	0.01	0.98	0.01	0.01	0,0006
0.98	0.01	0.01	0.98	0.01	0.01	0,0006
0.99	0.01	0.01	0.98	0.02	0.01	0,0006
0.99	0.01	0.01	0.98	0.00	0.02	0,0006
0.98	0.01	0.01	0.98	0.01	0.01	0,0006
0.98	0.01	0.01	0.98	0.01	0.01	0,0006
0.98	0.01	0.01	0.98	0.01	0.01	0,0006
0.98	0.01	0.01	0.98	0.01	0.01	0,0006
0.98	0.01	0.01	0.98	0.01	0.01	0,00059
0.98	0.01	0.01	0.98	0.01	0.01	0,00059
0.98	0.01	0.01	0.98	0.01	0.01	0,00059
0.99	0.01	0.01	0.96	0.02	0.02	0,00059
0.98	0.01	0.01	0.99	0.01	0.01	0,00059
0.98	0.01	0.01	0.98	0.01	0.01	0,00059
0.98	0.01	0.01	0.98	0.01	0.01	0,00058
0.99	0.01	0.01	0.93	0.03	0.04	0,00058
0.98	0.01	0.01	0.98	0.01	0.01	0,00058
0.98	0.01	0.01	0.98	0.01	0.01	0,00058
0.98	0.01	0.01	0.98	0.01	0.01	0,00058
0.98	0.01	0.01	0.98	0.01	0.01	0,00058
0.98	0.01	0.01	0.98	0.02	0.00	0,00057
0.98	0.01	0.01	0.98	0.01	0.01	0,00057
0.98	0.01	0.01	0.98	0.01	0.01	0,00057
0.99	0.01	0.01	0.94	0.03	0.03	0,00057
0.98	0.01	0.01	0.98	0.01	0.01	0,00057
0.98	0.01	0.01	0.98	0.01	0.01	0,00057
0.98	0.01	0.01	0.98	0.01	0.01	0,00057
0.98	0.01	0.01	0.98	0.01	0.01	0,00056
0.99	0.01	0.01	0.98	0.01	0.01	0,00056
0.99	0.01	0.01	0.98	0.01	0.01	0,00056
0.99	0.01	0.01	0.98	0.01	0.01	0,00056
0.98	0.01	0.01	0.99	0.01	0.01	0,00056
0.98	0.01	0.01	0.98	0.01	0.01	0,00056
0.98	0.01	0.01	0.98	0.01	0.01	0,00056
0.98	0.01	0.01	0.98	0.01	0.01	0,00056
0.98	0.01	0.01	0.98	0.01	0.01	0,00055
0.98	0.01	0.01	0.98	0.01	0.01	0,00055
0.98	0.01	0.01	0.98	0.01	0.01	0,00055
0.98	0.01	0.01	0.99	0.01	0.01	0,00055
0.99	0.00	0.01	0.98	0.01	0.01	0,00055
0.98	0.01	0.01	0.98	0.01	0.00	0,00055
0.98	0.01	0.01	0.98	0.01	0.01	0,00055
0.98	0.01	0.01	0.98	0.01	0.01	0,00055
0.98	0.01	0.01	0.98	0.01	0.01	0,00054
0.98	0.01	0.01	0.99	0.01	0.01	0,00054

chrX	7476512	2081	G	0	0.98	0.01	0.01	0.98	0.01	0.01	0.98	0.01	0.01	0.98	0.01	0.01	0.98	0.01	0.01	0.99	0.00	0.01
chr1(	92644379	535	G	0	0.98	0.01	0.01	0.98	0.01	0.01	0.99	0.01	0.00	0.98	0.01	0.01	0.98	0.01	0.01	0.98	0.01	0.00
chr4	136547660	1186	C	0	1.00	0.00	0.00	0.99	0.00	0.01	1.00	0.00	0.00	0.99	0.00	0.00	1.00	0.00	0.00	1.00	0.00	0.00
chrX	7476510	2079	G	0	0.98	0.01	0.01	0.98	0.01	0.01	0.99	0.00	0.01	0.97	0.01	0.02	0.98	0.01	0.01	0.99	0.01	0.00
chr1'	3525928	185	G	0	0.98	0.00	0.01	0.98	0.01	0.01	0.98	0.01	0.01	0.98	0.01	0.01	0.98	0.01	0.01	0.99	0.00	0.01
chr9	100498381	1857	G	0	0.98	0.01	0.01	0.98	0.02	0.00	0.99	0.01	0.00	0.99	0.01	0.00	0.98	0.01	0.01	0.98	0.02	0.00
chr4	136547692	1193	C	0	0.98	0.00	0.02	0.96	0.03	0.01	1.00	0.00	0.00	0.98	0.00	0.02	0.96	0.00	0.03	0.99	0.00	0.01
chr8	120115676	1752	G	0	0.98	0.01	0.00	0.99	0.00	0.00	0.99	0.01	0.00	0.98	0.01	0.02	0.99	0.00	0.01	0.98	0.00	0.01
chr1(	92644731	678	C	0	0.99	0.00	0.00	0.98	0.01	0.01	0.99	0.01	0.01	0.98	0.00	0.02	0.98	0.01	0.01	0.98	0.01	0.01
chr1;	45706530	745	C	0	0.99	0.01	0.00	0.99	0.00	0.00	0.99	0.01	0.00	0.98	0.00	0.01	0.97	0.00	0.02	0.98	0.01	0.01
chr1;	45706569	767	C	0	0.98	0.01	0.01	0.98	0.01	0.01	0.99	0.00	0.01	0.98	0.01	0.01	0.98	0.01	0.01	0.98	0.01	0.01
chr4	136547628	1175	C	0	0.99	0.00	0.01	0.97	0.01	0.02	0.98	0.01	0.01	0.97	0.01	0.02	0.98	0.01	0.01	0.99	0.00	0.01
chr5	64987505	1492	C	0	0.99	0.00	0.00	0.98	0.01	0.01	0.98	0.01	0.01	0.97	0.00	0.03	0.99	0.00	0.00	0.98	0.02	0.01
chr1;	45706622	798	G	0	0.99	0.01	0.00	0.98	0.01	0.01	0.98	0.01	0.01	0.98	0.01	0.01	0.99	0.00	0.01	0.99	0.01	0.00
chr4	136547684	1190	C	0	0.98	0.00	0.02	0.97	0.01	0.02	0.99	0.00	0.01	0.97	0.00	0.03	0.98	0.01	0.00	0.99	0.00	0.01
chr1(	24277542	934	C	0	0.99	0.01	0.00	0.99	0.00	0.00	0.98	0.01	0.01	0.98	0.01	0.01	0.99	0.01	0.01	0.98	0.01	0.01
chrX	7539376	2259	G	0	0.97	0.00	0.02	0.99	0.00	0.01	0.98	0.00	0.01	0.99	0.00	0.01	0.98	0.01	0.01	0.98	0.02	0.00
chrX	7539512	2340	C	0	0.99	0.00	0.00	0.99	0.01	0.01	0.99	0.01	0.00	0.98	0.01	0.01	0.98	0.01	0.02	0.99	0.00	0.01
chr8	120115711	1770	C	0	0.99	0.01	0.00	0.98	0.00	0.01	0.97	0.00	0.02	0.99	0.01	0.00	0.99	0.00	0.01	0.99	0.01	0.00
chr1;	45706540	750	C	0	0.99	0.00	0.01	0.99	0.00	0.01	0.99	0.01	0.01	0.98	0.01	0.01	0.99	0.00	0.00	0.99	0.01	0.00
chr1;	45706498	730	C	0	0.99	0.01	0.00	0.98	0.01	0.01	0.98	0.01	0.01	0.98	0.02	0.00	0.98	0.00	0.02	0.98	0.00	0.01
chr4	139783652	1259	G	0	0.99	0.01	0.00	0.99	0.00	0.00	0.99	0.00	0.00	0.99	0.01	0.00	0.99	0.01	0.00	0.99	0.01	0.00
chr5	64987463	1471	G	0	0.98	0.01	0.01	0.98	0.01	0.01	0.98	0.01	0.01	0.99	0.00	0.01	0.98	0.01	0.01	0.99	0.00	0.01
chr4	136547725	1198	C	0	0.99	0.00	0.00	0.98	0.01	0.01	0.97	0.03	0.00	0.99	0.00	0.01	1.00	0.00	0.00	1.00	0.00	0.00
chr4	136547359	1030	G	0	1.00	0.00	0.00	1.00	0.00	0.00	1.00	0.00	0.00	1.00	0.00	0.00	1.00	0.00	0.00	1.00	0.00	0.00
chr9	100498558	1953	C	0	0.99	0.00	0.01	0.99	0.01	0.01	0.99	0.00	0.01	0.98	0.01	0.01	0.99	0.00	0.01	0.99	0.00	0.01
chr1(	92644758	685	C	0	0.99	0.00	0.00	0.98	0.01	0.01	0.98	0.01	0.01	0.98	0.01	0.01	0.99	0.00	0.01	0.99	0.00	0.01
chr1(	92644718	673	C	0	0.99	0.01	0.01	0.98	0.01	0.01	0.99	0.01	0.01	0.99	0.00	0.01	0.98	0.01	0.01	0.99	0.00	0.01
chrX	7476447	2043	G	0	0.98	0.01	0.01	0.98	0.01	0.01	0.99	0.00	0.01	0.98	0.01	0.02	0.98	0.01	0.01	0.99	0.00	0.00
chr1(	92644603	653	C	0	0.98	0.01	0.01	0.98	0.01	0.01	0.99	0.00	0.01	0.98	0.00	0.01	0.98	0.00	0.01	0.99	0.00	0.00
chr1'	3525638	24	G	0	0.99	0.00	0.01	0.99	0.01	0.01	0.99	0.00	0.01	0.98	0.01	0.00	0.98	0.00	0.01	0.97	0.03	0.00
chrX	7476501	2072	G	1	0.98	0.01	0.01	0.98	0.01	0.01	0.99	0.00	0.01	0.97	0.01	0.02	0.98	0.01	0.01	0.99	0.00	0.00
chr1;	45706653	813	C	0	0.99	0.00	0.01	0.99	0.00	0.01	0.98	0.01	0.00	0.98	0.00	0.01	0.97	0.03	0.00	0.98	0.00	0.02
chr5	64987616	1537	C	0	0.99	0.00	0.01	0.98	0.01	0.01	0.98	0.01	0.01	0.99	0.00	0.01	0.99	0.00	0.00	0.98	0.01	0.01
chr5	64987490	1486	G	0	0.99	0.00	0.01	0.98	0.01	0.01	0.99	0.00	0.01	0.98	0.01	0.01	0.99	0.00	0.01	0.99	0.00	0.01
chr9	100498688	1990	c	0	0.99	0.01	0.00	0.99	0.00	0.01	0.99	0.01	0.00	0.99	0.01	0.01	0.98	0.02	0.00	0.99	0.01	0.00
chrX	7476524	2091	G	0	0.98	0.01	0.01	0.98	0.01	0.01	0.99	0.01	0.01	0.98	0.01	0.01	0.97	0.01	0.01	0.99	0.00	0.00
chr5	64987537	1506	C	0	0.99	0.00	0.01	0.99	0.01	0.01	0.98	0.01	0.01	0.99	0.00	0.01	0.98	0.01	0.01	0.99	0.00	0.01
chr4	136547616	1171	C	0	0.99	0.00	0.01	0.97	0.01	0.02	0.98	0.01	0.01	0.97	0.01	0.02	0.98	0.01	0.01	0.99	0.00	0.01
chrX	7476439	2039	G	0	0.98	0.01	0.01	0.99	0.01	0.01	0.99	0.00	0.01	0.97	0.01	0.01	0.98	0.01	0.01	0.99	0.00	0.00
chr1!	100300152	429	G	0	0.99	0.01	0.01	0.98	0.01	0.01	0.99	0.00	0.01	0.99	0.00	0.01	0.99	0.00	0.01	1.00	0.00	0.00
chrX	7476428	2037	G	0	0.99	0.01	0.01	0.98	0.01	0.01	0.99	0.00	0.01	0.98	0.01	0.01	0.98	0.01	0.01	0.99	0.00	0.00
chr4	136547386	1040	G	0	0.99	0.00	0.01	1.00	0.00	0.00	1.00	0.00	0.00	0.99	0.00	0.00	0.99	0.00	0.00	0.99	0.01	0.00
chr4	136547534	1126	C	0	0.99	0.00	0.01	0.98	0.01	0.01	0.98	0.01	0.01	0.99	0.01	0.01	0.99	0.00	0.01	0.99	0.00	0.01
chrX	7476522	2090	G	0	0.98	0.01	0.01	0.98	0.01	0.01	0.99	0.00	0.01	0.98	0.01	0.01	0.98	0.01	0.01	0.99	0.00	0.00
chrX	7476535	2099	G	1	0.98	0.01	0.01	0.98	0.01	0.01	0.99	0.01	0.01	0.98	0.01	0.01	0.98	0.01	0.01	0.97	0.01	0.02

0.98	0.01	0.01	0.98	0.01	0.01	0,00054
0.98	0.01	0.01	0.98	0.01	0.01	0,00054
0.99	0.01	0.01	0.91	0.02	0.07	0,00054
0.98	0.01	0.01	0.98	0.01	0.01	0,00054
0.98	0.01	0.01	0.98	0.01	0.01	0,00053
0.98	0.01	0.01	0.98	0.01	0.01	0,00053
0.98	0.01	0.01	0.98	0.00	0.01	0,00052
0.99	0.01	0.00	0.98	0.00	0.01	0,00052
0.98	0.01	0.01	0.98	0.01	0.01	0,00052
0.99	0.01	0.01	0.98	0.01	0.01	0,00052
0.98	0.01	0.01	0.98	0.01	0.01	0,00052
0.98	0.01	0.01	0.98	0.01	0.01	0,00052
0.98	0.01	0.01	0.98	0.01	0.01	0,00051
0.98	0.01	0.01	0.99	0.01	0.01	0,00051
0.98	0.00	0.01	0.98	0.01	0.01	0,00051
0.99	0.01	0.01	0.98	0.01	0.01	0,0005
0.98	0.01	0.01	0.98	0.01	0.01	0,0005
0.99	0.01	0.01	0.98	0.01	0.01	0,0005
0.98	0.01	0.01	0.98	0.01	0.01	0,0005
0.99	0.01	0.01	0.98	0.01	0.01	0,00049
0.98	0.01	0.01	0.99	0.01	0.01	0,00049
0.99	0.01	0.00	0.92	0.07	0.00	0,00049
0.98	0.01	0.01	0.99	0.01	0.01	0,00049
0.98	0.01	0.01	0.99	0.00	0.01	0,00049
0.99	0.00	0.00	0.98	0.01	0.01	0,00048
0.98	0.01	0.01	0.98	0.01	0.01	0,00048
0.98	0.01	0.01	0.98	0.01	0.01	0,00048
0.98	0.01	0.01	0.99	0.01	0.01	0,00048
0.98	0.01	0.01	0.98	0.01	0.01	0,00048
0.98	0.01	0.01	0.99	0.01	0.01	0,00047
0.99	0.01	0.01	0.98	0.01	0.01	0,00047
0.98	0.01	0.01	0.98	0.01	0.01	0,00047
0.99	0.01	0.01	0.98	0.01	0.01	0,00047
0.98	0.01	0.01	0.99	0.01	0.01	0,00047
0.99	0.01	0.01	0.98	0.01	0.01	0,00047
0.98	0.01	0.01	0.98	0.01	0.01	0,00047
0.98	0.01	0.01	0.98	0.01	0.01	0,00046
0.98	0.01	0.01	0.98	0.01	0.01	0,00046
0.98	0.01	0.01	0.98	0.01	0.01	0,00046
0.98	0.01	0.01	0.98	0.01	0.01	0,00046
0.98	0.01	0.01	0.99	0.01	0.01	0,00046
0.98	0.01	0.01	0.98	0.01	0.01	0,00046
0.99	0.00	0.01	0.96	0.02	0.02	0,00045
0.98	0.01	0.01	0.99	0.01	0.01	0,00045
0.98	0.01	0.01	0.98	0.01	0.01	0,00045
0.98	0.01	0.01	0.98	0.01	0.01	0,00045

chr8	120115722	1775	C	0	0.99	0.01	0.00	0.99	0.00	0.01	0.98	0.00	0.02	0.99	0.00	0.00	0.98	0.00	0.02	0.99	0.00	0.01
chr9	100498338	1842	G	0	0.99	0.01	0.00	0.99	0.00	0.01	0.99	0.01	0.00	0.99	0.01	0.00	0.99	0.01	0.00	0.99	0.01	0.00
chr8	89376964	1647	G	0	0.98	0.01	0.00	0.99	0.00	0.01	0.98	0.01	0.01	0.98	0.01	0.00	0.99	0.01	0.00	0.99	0.00	0.01
chr11	3525647	30	G	0	0.99	0.00	0.01	0.99	0.00	0.01	1.00	0.00	0.00	0.97	0.02	0.00	0.98	0.00	0.01	0.99	0.00	0.01
chrX	7539413	2279	C	0	0.99	0.00	0.01	0.98	0.01	0.01	0.99	0.00	0.01	0.99	0.00	0.01	0.96	0.04	0.00	0.98	0.02	0.00
chr4	139783724	1298	C	0	0.99	0.00	0.00	0.99	0.01	0.01	0.98	0.01	0.01	0.98	0.01	0.02	0.99	0.00	0.01	0.99	0.00	0.00
chrX	7539490	2329	G	0	0.99	0.00	0.01	0.98	0.01	0.01	0.98	0.01	0.01	0.99	0.01	0.01	0.97	0.01	0.02	0.99	0.00	0.01
chr16	92644721	676	C	0	0.99	0.00	0.00	0.98	0.01	0.01	0.98	0.01	0.01	0.99	0.00	0.01	0.98	0.01	0.01	0.98	0.01	0.01
chr16	92644782	692	C	0	0.99	0.00	0.00	0.98	0.01	0.01	0.98	0.01	0.01	0.99	0.00	0.01	0.99	0.00	0.01	0.99	0.00	0.01
chr17	45706483	721	G	0	0.98	0.01	0.01	0.99	0.01	0.01	0.99	0.00	0.01	0.98	0.02	0.00	0.99	0.01	0.00	0.99	0.01	0.00
chr4	136547689	1191	C	0	0.99	0.00	0.01	0.99	0.01	0.01	0.98	0.00	0.02	0.97	0.03	0.00	0.99	0.00	0.00	0.97	0.00	0.03
chrX	7476444	2042	G	0	0.99	0.01	0.01	0.99	0.01	0.01	0.98	0.01	0.01	0.98	0.01	0.02	0.98	0.01	0.01	0.99	0.01	0.01
chr16	92644539	628	G	0	0.98	0.01	0.01	0.99	0.00	0.00	0.99	0.01	0.01	0.99	0.00	0.00	0.99	0.00	0.00	0.98	0.01	0.01
chr4	136547551	1138	C	0	0.99	0.00	0.01	0.98	0.01	0.01	0.98	0.01	0.01	0.98	0.01	0.01	0.98	0.01	0.01	0.99	0.00	0.00
chr15	100300414	498	C	0	0.98	0.00	0.01	0.98	0.01	0.01	0.99	0.00	0.01	0.99	0.00	0.01	0.99	0.00	0.00	1.00	0.00	0.00
chr15	100300143	422	G	0	0.98	0.01	0.01	0.99	0.00	0.01	0.98	0.01	0.01	0.98	0.01	0.01	0.99	0.00	0.01	0.99	0.00	0.00
chr4	139783649	1257	G	0	1.00	0.00	0.00	0.99	0.00	0.00	0.99	0.00	0.00	0.99	0.00	0.00	1.00	0.00	0.00	0.99	0.00	0.01
chr16	92644583	649	C	0	0.98	0.01	0.01	0.99	0.01	0.01	0.99	0.01	0.01	0.98	0.01	0.01	0.98	0.01	0.01	0.99	0.00	0.00
chr8	120115674	1751	C	0	0.99	0.00	0.01	0.99	0.00	0.00	0.98	0.01	0.00	0.99	0.01	0.00	0.99	0.01	0.01	0.99	0.00	0.00
chr19	24277506	912	c	0	0.99	0.00	0.01	0.99	0.00	0.00	0.98	0.01	0.00	0.99	0.01	0.00	0.98	0.00	0.02	0.99	0.01	0.00
chr19	24277555	940	c	0	0.98	0.01	0.01	0.99	0.00	0.00	0.99	0.00	0.00	0.98	0.01	0.01	0.98	0.01	0.02	0.99	0.00	0.01
chr15	61868821	282	C	0	0.98	0.00	0.02	0.99	0.00	0.00	0.98	0.02	0.00	0.98	0.02	0.00	0.98	0.02	0.00	0.99	0.01	0.00
chrX	7476464	2050	G	0	0.98	0.01	0.01	0.98	0.01	0.01	0.99	0.00	0.01	0.98	0.01	0.02	0.98	0.01	0.02	0.99	0.01	0.00
chr16	92644781	691	C	0	0.99	0.00	0.00	0.98	0.01	0.01	0.98	0.01	0.01	0.99	0.00	0.01	0.99	0.00	0.01	0.99	0.00	0.01
chr9	100498370	1853	G	0	0.98	0.02	0.00	0.99	0.01	0.00	0.99	0.01	0.01	0.99	0.01	0.00	0.99	0.01	0.00	0.99	0.01	0.00
chr16	92644488	594	C	0	0.99	0.00	0.00	0.98	0.01	0.01	0.99	0.00	0.01	0.99	0.00	0.01	0.99	0.00	0.01	0.99	0.00	0.01
chr8	120115726	1777	C	0	0.99	0.00	0.01	0.99	0.01	0.01	0.98	0.00	0.02	0.98	0.01	0.00	0.99	0.01	0.00	0.99	0.00	0.00
chr16	92644745	680	C	0	0.99	0.00	0.00	0.98	0.01	0.01	0.98	0.01	0.01	0.98	0.01	0.01	0.98	0.00	0.01	0.99	0.00	0.01
chr15	61868828	286	G	0	1.00	0.00	0.00	0.99	0.00	0.00	0.99	0.00	0.01	0.99	0.00	0.00	0.99	0.00	0.00	0.99	0.00	0.00
chr16	92644720	675	C	0	0.99	0.00	0.00	0.98	0.01	0.01	0.97	0.01	0.02	0.99	0.00	0.01	0.98	0.00	0.01	0.99	0.00	0.01
chrX	7539566	2375	C	0	0.99	0.00	0.01	0.99	0.01	0.00	0.99	0.01	0.00	1.00	0.00	0.00	0.99	0.00	0.01	0.99	0.01	0.00
chr8	89377002	1664	G	0	0.98	0.02	0.00	0.98	0.00	0.01	0.98	0.01	0.01	0.97	0.02	0.00	0.98	0.00	0.01	0.98	0.01	0.00
chrX	7476424	2035	G	1	0.99	0.00	0.01	0.99	0.01	0.01	0.98	0.01	0.01	0.98	0.01	0.02	0.99	0.01	0.01	0.99	0.00	0.01
chr19	24277416	883	c	0	1.00	0.00	0.00	0.99	0.00	0.00	1.00	0.00	0.00	0.99	0.00	0.00	1.00	0.00	0.00	1.00	0.00	0.00
chr19	24277494	907	c	0	0.99	0.00	0.00	0.99	0.01	0.00	0.99	0.00	0.00	0.99	0.00	0.01	1.00	0.00	0.00	0.99	0.00	0.01
chr16	92644591	652	C	0	0.98	0.01	0.01	0.99	0.01	0.01	0.99	0.01	0.01	0.99	0.00	0.01	0.99	0.00	0.01	0.99	0.00	0.01
chr4	139783787	1333	G	0	0.99	0.00	0.00	0.98	0.02	0.00	0.99	0.00	0.01	0.98	0.02	0.00	0.98	0.02	0.00	0.98	0.00	0.01
chr5	64987467	1475	G	0	0.98	0.01	0.01	0.99	0.01	0.01	0.98	0.01	0.01	0.99	0.00	0.01	0.99	0.00	0.01	0.99	0.00	0.00
chr11	3525827	128	G	0	0.99	0.01	0.00	1.00	0.00	0.00	0.99	0.00	0.01	1.00	0.00	0.00	0.99	0.00	0.01	0.98	0.02	0.00
chr16	92644434	555	G	0	0.99	0.00	0.01	0.99	0.00	0.00	0.99	0.01	0.01	0.99	0.01	0.00	0.98	0.01	0.01	0.99	0.01	0.00
chrX	7476514	2083	G	0	0.98	0.01	0.01	0.98	0.01	0.01	0.99	0.01	0.01	0.98	0.01	0.01	0.98	0.01	0.01	0.99	0.00	0.00
chr17	45706775	864	G	0	0.99	0.00	0.01	0.99	0.00	0.01	0.99	0.01	0.00	0.99	0.00	0.01	0.99	0.00	0.00	0.99	0.00	0.01
chr19	24277664	983	G	0	0.98	0.02	0.00	0.99	0.01	0.01	0.98	0.00	0.01	0.99	0.00	0.01	1.00	0.00	0.00	0.99	0.00	0.01
chr4	136547516	1115	C	0	0.99	0.00	0.00	0.99	0.01	0.01	0.99	0.00	0.00	0.99	0.00	0.01	0.99	0.00	0.01	0.99	0.00	0.00
chr4	139783843	1362	C	0	0.98	0.02	0.00	0.99	0.00	0.01	0.99	0.01	0.00	0.99	0.00	0.01	0.98	0.00	0.02	0.98	0.01	0.00
chr16	92644696	660	C	0	0.99	0.00	0.00	0.98	0.01	0.01	0.99	0.01	0.01	0.98	0.00	0.02	0.99	0.00	0.01	0.99	0.00	0.01

0.99	0.01	0.01	0.98	0.01	0.01	0,00045
0.99	0.01	0.01	0.98	0.01	0.00	0,00045
0.98	0.01	0.01	0.99	0.01	0.01	0,00045
0.99	0.01	0.01	0.98	0.01	0.01	0,00045
0.98	0.01	0.01	0.98	0.01	0.01	0,00045
0.98	0.01	0.01	0.99	0.01	0.01	0,00044
0.98	0.01	0.01	0.98	0.01	0.01	0,00044
0.98	0.01	0.01	0.98	0.01	0.01	0,00044
0.98	0.01	0.01	0.98	0.00	0.01	0,00044
0.98	0.01	0.01	0.99	0.01	0.01	0,00044
0.98	0.01	0.01	0.98	0.01	0.01	0,00044
0.98	0.01	0.01	0.98	0.00	0.01	0,00044
0.98	0.01	0.01	0.99	0.01	0.01	0,00044
0.98	0.01	0.01	0.98	0.01	0.01	0,00044
0.98	0.01	0.01	0.99	0.00	0.01	0,00043
0.98	0.01	0.01	0.99	0.01	0.01	0,00043
0.99	0.01	0.01	0.97	0.01	0.02	0,00043
0.98	0.01	0.01	0.98	0.01	0.01	0,00043
0.98	0.01	0.01	0.99	0.01	0.01	0,00043
0.99	0.01	0.01	0.98	0.01	0.01	0,00043
0.99	0.01	0.01	0.98	0.01	0.01	0,00043
0.99	0.01	0.01	0.98	0.01	0.00	0,00043
0.98	0.01	0.01	0.98	0.01	0.01	0,00043
0.98	0.01	0.01	0.99	0.01	0.01	0,00042
0.99	0.01	0.01	0.98	0.01	0.00	0,00042
0.98	0.01	0.01	0.99	0.01	0.01	0,00042
0.98	0.01	0.01	0.99	0.01	0.01	0,00042
0.99	0.01	0.01	0.98	0.01	0.01	0,00041
0.99	0.01	0.01	0.97	0.02	0.01	0,00041
0.99	0.01	0.01	0.98	0.01	0.01	0,00041
0.99	0.01	0.01	0.96	0.01	0.03	0,00041
0.98	0.01	0.01	0.99	0.01	0.01	0,00041
0.98	0.01	0.01	0.98	0.01	0.01	0,00041
0.99	0.01	0.01	0.97	0.02	0.01	0,00041
0.98	0.01	0.01	0.99	0.01	0.01	0,00041
0.99	0.01	0.01	0.99	0.01	0.01	0,00041
0.99	0.01	0.01	0.98	0.01	0.01	0,00041
0.98	0.01	0.01	0.99	0.01	0.01	0,00041
0.99	0.01	0.01	0.99	0.01	0.01	0,0004
0.99	0.01	0.01	0.98	0.01	0.01	0,0004
0.98	0.01	0.01	0.98	0.01	0.01	0,0004
0.98	0.01	0.01	0.99	0.00	0.01	0,0004
0.98	0.01	0.01	0.99	0.00	0.01	0,0004
0.99	0.01	0.01	0.98	0.01	0.01	0,0004
0.99	0.01	0.01	0.98	0.01	0.01	0,0004
0.99	0.01	0.01	0.98	0.01	0.01	0,0004

chr17	45706550	756	G	0	0.99	0.01	0.00	0.98	0.01	0.00	0.99	0.00	0.01	0.98	0.02	0.00	0.99	0.00	0.00	0.99	0.00	0.01
chrX	7539347	2240	G	0	0.99	0.00	0.00	0.97	0.03	0.00	1.00	0.00	0.00	0.99	0.00	0.00	0.98	0.00	0.02	0.98	0.00	0.02
chr11	3525917	182	G	0	0.99	0.00	0.01	0.98	0.01	0.01	0.99	0.00	0.01	0.99	0.01	0.01	0.99	0.00	0.01	0.99	0.01	0.00
chrX	7476502	2073	G	0	0.98	0.01	0.01	0.98	0.01	0.01	0.99	0.00	0.01	0.98	0.01	0.01	0.98	0.01	0.01	0.99	0.00	0.00
chr19	24277585	952	g	0	0.99	0.00	0.01	0.99	0.00	0.00	0.99	0.00	0.00	1.00	0.00	0.00	0.99	0.00	0.00	1.00	0.00	0.00
chr4	139783741	1306	C	0	0.99	0.00	0.00	0.99	0.01	0.01	0.99	0.00	0.01	0.98	0.01	0.02	0.98	0.00	0.01	0.99	0.00	0.01
chr15	61868730	238	C	0	1.00	0.00	0.00	0.99	0.00	0.00	1.00	0.00	0.00	0.99	0.00	0.00	0.99	0.01	0.00	0.99	0.01	0.00
chr15	100300301	466	C	0	0.99	0.00	0.01	0.98	0.01	0.01	0.97	0.01	0.02	0.98	0.01	0.01	0.99	0.00	0.01	1.00	0.00	0.00
chr19	24277579	950	g	0	0.98	0.02	0.00	0.99	0.00	0.01	0.98	0.01	0.01	0.99	0.01	0.00	0.98	0.01	0.00	0.99	0.00	0.01
chr19	24277612	967	C	0	0.99	0.01	0.00	0.99	0.00	0.00	0.97	0.02	0.01	1.00	0.00	0.00	0.99	0.01	0.01	0.99	0.00	0.01
chr4	139783618	1240	G	0	0.99	0.00	0.01	0.99	0.01	0.00	0.99	0.01	0.00	0.98	0.02	0.00	0.98	0.01	0.01	0.98	0.00	0.02
chr8	89376815	1575	g	0	0.99	0.01	0.00	0.99	0.00	0.00	0.99	0.00	0.01	0.97	0.03	0.00	0.99	0.00	0.01	0.99	0.01	0.00
chr11	3525768	91	G	0	0.99	0.00	0.00	0.99	0.00	0.00	0.99	0.00	0.00	0.97	0.02	0.00	0.99	0.00	0.00	0.99	0.00	0.01
chr16	92644339	522	G	0	0.99	0.00	0.01	0.99	0.00	0.00	0.99	0.00	0.01	0.99	0.01	0.00	0.99	0.00	0.00	0.99	0.00	0.00
chr17	45706461	707	C	0	0.99	0.00	0.01	0.99	0.00	0.01	0.99	0.01	0.00	0.99	0.00	0.01	0.98	0.00	0.02	0.98	0.00	0.02
chr16	92644570	644	C	0	1.00	0.00	0.00	0.99	0.00	0.00	0.99	0.00	0.01	0.98	0.01	0.01	0.98	0.00	0.02	0.98	0.01	0.00
chr8	89376875	1611	c	0	0.99	0.01	0.00	1.00	0.00	0.00	0.99	0.01	0.00	0.98	0.01	0.00	0.98	0.01	0.00	0.99	0.00	0.01
chrX	7476427	2036	G	0	0.98	0.01	0.01	0.99	0.00	0.01	0.98	0.01	0.01	0.98	0.01	0.01	0.98	0.01	0.01	0.99	0.00	0.01
chrX	7539540	2357	G	0	0.98	0.02	0.00	0.98	0.02	0.00	0.99	0.00	0.01	0.99	0.00	0.01	0.99	0.00	0.01	0.99	0.00	0.01
chrX	7539598	2393	G	0	0.98	0.02	0.00	0.98	0.00	0.02	0.99	0.00	0.01	0.96	0.04	0.00	0.98	0.00	0.01	0.98	0.00	0.01
chr16	92644439	558	G	0	0.99	0.00	0.00	1.00	0.00	0.00	0.99	0.01	0.00	0.99	0.01	0.01	0.99	0.00	0.01	0.99	0.00	0.01
chr5	64987491	1487	C	0	0.99	0.00	0.01	0.97	0.01	0.02	0.99	0.00	0.00	0.98	0.00	0.02	0.99	0.00	0.00	0.99	0.00	0.00
chr19	24277474	896	c	0	0.99	0.00	0.01	0.98	0.01	0.01	0.99	0.01	0.00	0.99	0.00	0.01	0.99	0.00	0.00	0.99	0.00	0.01
chr8	89376884	1616	c	0	0.98	0.01	0.00	0.99	0.00	0.01	0.99	0.00	0.01	0.98	0.01	0.00	0.99	0.00	0.01	0.98	0.02	0.00
chr11	3525606	8	C	0	1.00	0.00	0.00	1.00	0.00	0.00	1.00	0.00	0.00	1.00	0.00	0.00	1.00	0.00	0.00	1.00	0.00	0.00
chr4	139783722	1297	C	0	0.99	0.00	0.01	0.99	0.00	0.00	0.99	0.01	0.01	0.99	0.00	0.01	0.97	0.01	0.02	0.99	0.00	0.01
chrX	7476442	2041	G	0	0.99	0.01	0.01	0.99	0.01	0.01	0.99	0.00	0.01	0.98	0.01	0.01	0.98	0.01	0.01	0.99	0.00	0.00
chr4	136547399	1048	G	0	0.99	0.01	0.00	0.98	0.00	0.01	0.99	0.01	0.00	0.98	0.01	0.01	0.99	0.01	0.00	0.99	0.01	0.00
chr11	3525831	130	G	0	0.99	0.01	0.00	0.99	0.00	0.01	0.99	0.01	0.00	0.99	0.00	0.01	0.99	0.00	0.01	0.98	0.01	0.00
chr8	120115713	1771	G	0	0.99	0.00	0.00	1.00	0.00	0.00	0.99	0.00	0.00	0.98	0.02	0.00	0.99	0.01	0.00	0.99	0.01	0.00
chr16	92644686	658	C	0	0.99	0.00	0.00	0.98	0.01	0.01	0.99	0.00	0.01	0.98	0.01	0.01	0.99	0.00	0.01	0.99	0.00	0.01
chr17	45706638	806	G	0	0.99	0.00	0.00	0.99	0.01	0.01	0.98	0.01	0.01	0.98	0.01	0.01	0.98	0.01	0.01	1.00	0.00	0.00
chr8	120115563	1700	G	0	1.00	0.00	0.00	1.00	0.00	0.00	1.00	0.00	0.00	1.00	0.00	0.00	1.00	0.00	0.00	1.00	0.00	0.00
chr15	100300383	494	C	0	0.99	0.00	0.00	0.99	0.00	0.00	0.99	0.00	0.00	0.99	0.00	0.00	1.00	0.00	0.00	1.00	0.00	0.00
chrX	7539517	2342	C	0	0.99	0.00	0.00	0.99	0.00	0.01	1.00	0.00	0.00	0.98	0.01	0.01	0.98	0.01	0.01	0.99	0.01	0.00
chr4	136547580	1153	G	0	0.98	0.02	0.00	0.99	0.00	0.01	0.99	0.01	0.00	0.99	0.00	0.01	0.99	0.00	0.01	0.98	0.02	0.00
chrX	7539491	2330	G	0	0.99	0.00	0.00	0.99	0.01	0.01	0.99	0.01	0.01	0.99	0.00	0.01	0.99	0.01	0.01	0.99	0.00	0.01
chr16	92644494	598	C	0	0.99	0.00	0.00	0.99	0.01	0.01	0.99	0.00	0.01	0.99	0.00	0.01	0.99	0.00	0.01	0.99	0.00	0.00
chr9	100498401	1868	G	0	1.00	0.00	0.00	0.99	0.00	0.00	1.00	0.00	0.00	1.00	0.00	0.00	0.99	0.00	0.00	1.00	0.00	0.00
chr9	100498562	1956	C	0	0.99	0.00	0.00	1.00	0.00	0.00	1.00	0.00	0.00	0.98	0.01	0.00	0.99	0.00	0.01	1.00	0.00	0.00
chr17	45706518	738	G	0	0.99	0.01	0.00	0.99	0.00	0.00	0.98	0.01	0.01	0.99	0.00	0.01	0.98	0.00	0.02	0.98	0.01	0.00
chr17	45706668	822	C	0	0.99	0.00	0.01	0.99	0.00	0.00	0.99	0.00	0.01	0.98	0.01	0.01	0.98	0.00	0.01	0.97	0.02	0.00
chr19	24277576	949	g	0	0.99	0.01	0.00	0.98	0.01	0.01	0.99	0.01	0.00	0.99	0.01	0.00	0.99	0.00	0.00	0.99	0.01	0.00
chr5	64987478	1480	G	0	0.99	0.00	0.01	0.98	0.01	0.01	0.98	0.01	0.01	0.99	0.00	0.01	0.99	0.00	0.01	0.99	0.00	0.00
chr9	100498464	1901	C	0	1.00	0.00	0.00	0.98	0.01	0.00	0.99	0.00	0.01	0.99	0.00	0.01	0.99	0.00	0.01	0.99	0.01	0.00
chr9	100498539	1943	C	0	0.99	0.00	0.01	0.99	0.01	0.01	0.99	0.00	0.00	0.99	0.01	0.01	0.99	0.01	0.01	0.99	0.00	0.00

0.98	0.01	0.01	0.99	0.01	0.01	0,0004
0.99	0.01	0.01	0.98	0.00	0.01	0,00039
0.99	0.01	0.01	0.98	0.01	0.01	0,00039
0.99	0.01	0.01	0.99	0.01	0.01	0,00039
0.99	0.00	0.01	0.98	0.01	0.01	0,00039
0.99	0.01	0.01	0.98	0.01	0.01	0,00039
0.99	0.01	0.01	0.97	0.01	0.01	0,00039
0.98	0.01	0.01	0.99	0.01	0.01	0,00039
0.99	0.01	0.01	0.98	0.01	0.01	0,00039
0.98	0.01	0.01	0.99	0.01	0.01	0,00039
0.99	0.01	0.01	0.98	0.01	0.01	0,00039
0.99	0.01	0.01	0.98	0.01	0.01	0,00039
0.99	0.01	0.01	0.98	0.01	0.01	0,00039
0.98	0.01	0.01	0.99	0.01	0.01	0,00039
0.99	0.01	0.01	0.98	0.01	0.01	0,00039
0.99	0.01	0.01	0.98	0.01	0.01	0,00039
0.99	0.01	0.01	0.99	0.01	0.01	0,00039
0.98	0.01	0.01	0.99	0.01	0.01	0,00039
0.98	0.01	0.01	0.99	0.00	0.01	0,00038
0.98	0.01	0.01	0.99	0.01	0.01	0,00038
0.99	0.01	0.01	0.98	0.01	0.01	0,00038
0.98	0.01	0.01	0.99	0.01	0.01	0,00038
0.98	0.01	0.01	0.99	0.01	0.01	0,00038
0.99	0.01	0.01	0.98	0.01	0.01	0,00038
0.99	0.00	0.00	0.93	0.04	0.03	0,00038
0.99	0.01	0.01	0.98	0.01	0.01	0,00038
0.99	0.01	0.01	0.99	0.01	0.01	0,00038
0.99	0.01	0.01	0.98	0.01	0.00	0,00038
0.99	0.01	0.01	0.99	0.01	0.01	0,00038
0.99	0.00	0.01	0.98	0.01	0.01	0,00038
0.99	0.01	0.01	0.98	0.01	0.01	0,00038
0.99	0.01	0.01	0.98	0.01	0.01	0,00038
0.99	0.00	0.00	0.95	0.02	0.03	0,00038
0.99	0.01	0.01	0.98	0.01	0.01	0,00038
0.99	0.01	0.01	0.98	0.01	0.01	0,00037
0.98	0.01	0.01	0.99	0.01	0.01	0,00037
0.99	0.01	0.01	0.99	0.01	0.01	0,00037
0.99	0.01	0.01	0.99	0.01	0.01	0,00037
0.99	0.00	0.00	0.97	0.02	0.01	0,00037
0.99	0.00	0.01	0.98	0.01	0.01	0,00037
0.99	0.01	0.01	0.99	0.01	0.01	0,00037
0.99	0.01	0.01	0.98	0.01	0.01	0,00037
0.98	0.01	0.01	0.99	0.01	0.01	0,00037
0.98	0.01	0.01	0.99	0.01	0.01	0,00037
0.99	0.01	0.01	0.99	0.00	0.01	0,00037
0.99	0.01	0.01	0.99	0.01	0.01	0,00037

chr16	92644748	683	C	0	0.99	0.00	0.00	0.98	0.01	0.01	0.99	0.01	0.01	0.99	0.00	0.01	0.99	0.00	0.01	0.98	0.01	0.01
chrX	7539374	2258	G	0	0.98	0.02	0.00	0.99	0.00	0.00	0.99	0.00	0.00	0.98	0.00	0.02	0.99	0.00	0.00	0.99	0.00	0.01
chr15	61868719	232	G	0	0.99	0.00	0.00	0.99	0.00	0.01	0.99	0.01	0.00	0.99	0.00	0.01	0.98	0.00	0.01	0.97	0.02	0.00
chr17	45706746	859	G	0	1.00	0.00	0.00	0.99	0.01	0.01	0.99	0.00	0.00	0.98	0.02	0.00	0.99	0.00	0.01	0.98	0.02	0.00
chr8	89376894	1623	c	0	0.99	0.01	0.00	0.99	0.01	0.01	0.98	0.02	0.00	0.99	0.00	0.01	0.97	0.03	0.00	0.99	0.01	0.00
chrX	7539370	2255	G	0	0.99	0.01	0.00	0.99	0.01	0.01	0.98	0.01	0.01	0.99	0.00	0.01	1.00	0.00	0.00	1.00	0.00	0.00
chr16	92644761	686	C	0	1.00	0.00	0.00	0.98	0.01	0.01	0.99	0.01	0.01	0.99	0.00	0.01	0.99	0.00	0.01	0.99	0.00	0.01
chr17	45706490	724	G	0	0.99	0.01	0.00	0.99	0.01	0.01	0.98	0.01	0.01	0.99	0.00	0.01	0.99	0.00	0.01	0.98	0.02	0.00
chr4	136547673	1188	C	0	0.99	0.00	0.01	0.99	0.01	0.01	0.98	0.01	0.00	0.99	0.00	0.01	0.98	0.00	0.02	0.99	0.00	0.01
chr11	3525738	77	G	0	0.99	0.00	0.00	1.00	0.00	0.00	0.98	0.01	0.01	0.97	0.02	0.01	0.99	0.00	0.01	0.98	0.01	0.00
chr16	92644508	608	C	0	1.00	0.00	0.00	0.98	0.01	0.01	0.99	0.00	0.00	0.99	0.00	0.01	0.99	0.00	0.01	0.99	0.00	0.01
chr5	64987316	1405	G	0	0.99	0.01	0.00	0.99	0.01	0.01	0.99	0.00	0.00	1.00	0.00	0.00	0.99	0.01	0.00	0.99	0.01	0.00
chr17	45706659	817	G	0	0.99	0.00	0.00	0.99	0.01	0.01	0.99	0.00	0.01	0.99	0.01	0.01	0.99	0.01	0.01	0.99	0.00	0.00
chr5	64987626	1541	C	0	0.99	0.01	0.00	0.99	0.01	0.01	0.99	0.00	0.01	0.99	0.00	0.00	0.99	0.00	0.00	0.99	0.00	0.01
chrX	7539350	2242	G	0	0.99	0.00	0.01	0.97	0.03	0.00	0.98	0.01	0.01	0.99	0.00	0.01	0.99	0.00	0.01	0.98	0.02	0.00
chr17	45706662	820	G	0	0.99	0.01	0.01	0.98	0.01	0.01	0.99	0.00	0.01	0.99	0.00	0.01	0.99	0.00	0.01	1.00	0.00	0.00
chr11	3525765	89	G	0	0.99	0.00	0.00	0.99	0.00	0.00	1.00	0.00	0.00	0.99	0.00	0.00	0.99	0.00	0.00	0.99	0.01	0.00
chr15	100300155	430	G	0	0.99	0.00	0.00	0.99	0.00	0.00	0.98	0.01	0.01	0.99	0.00	0.01	0.99	0.00	0.01	0.99	0.01	0.00
chr8	120115821	1821	C	0	0.99	0.00	0.00	0.98	0.01	0.00	0.98	0.01	0.01	0.99	0.00	0.00	0.98	0.00	0.01	0.99	0.01	0.01
chr16	92644777	690	C	0	0.99	0.00	0.00	0.98	0.01	0.01	0.98	0.01	0.01	0.99	0.00	0.01	0.99	0.00	0.01	0.99	0.00	0.01
chr16	92644772	689	C	0	0.99	0.00	0.00	0.98	0.01	0.01	0.98	0.01	0.01	0.98	0.01	0.01	0.99	0.00	0.01	0.99	0.00	0.01
chr17	45706647	811	G	0	0.99	0.00	0.01	0.99	0.01	0.01	0.99	0.00	0.01	0.98	0.01	0.01	0.99	0.00	0.00	0.99	0.00	0.00
chr8	120115567	1701	G	0	0.99	0.01	0.00	0.98	0.01	0.01	0.98	0.02	0.00	0.99	0.01	0.00	0.98	0.01	0.00	0.98	0.00	0.01
chr9	100498430	1883	G	0	0.99	0.01	0.00	0.99	0.01	0.00	0.99	0.01	0.00	0.99	0.00	0.01	0.99	0.01	0.00	0.99	0.00	0.01
chrX	7539411	2278	C	0	0.99	0.01	0.00	0.99	0.01	0.01	0.99	0.01	0.00	0.99	0.01	0.00	0.99	0.00	0.01	0.99	0.00	0.00
chr15	100300350	486	C	0	0.99	0.01	0.00	0.98	0.01	0.01	0.99	0.01	0.01	0.99	0.00	0.01	0.99	0.00	0.00	0.99	0.00	0.00
chr19	24277614	968	C	0	0.98	0.01	0.00	1.00	0.00	0.00	0.98	0.01	0.01	0.99	0.00	0.01	0.99	0.00	0.01	0.99	0.01	0.00
chr5	64987507	1493	G	0	1.00	0.00	0.00	1.00	0.00	0.00	1.00	0.00	0.00	0.99	0.00	0.00	1.00	0.00	0.00	1.00	0.00	0.00
chr8	89376925	1633	C	0	0.99	0.01	0.00	0.98	0.02	0.00	0.99	0.00	0.00	0.99	0.00	0.00	0.98	0.02	0.00	0.99	0.00	0.00
chr8	120115665	1746	C	0	0.99	0.01	0.00	0.99	0.00	0.01	0.98	0.01	0.01	0.99	0.01	0.00	0.99	0.00	0.01	0.99	0.00	0.00
chr16	92644746	681	C	0	0.99	0.00	0.00	0.98	0.01	0.01	0.98	0.01	0.01	0.99	0.00	0.01	0.99	0.00	0.01	0.99	0.00	0.01
chr4	139783826	1354	G	0	1.00	0.00	0.00	0.98	0.00	0.01	0.99	0.00	0.00	0.99	0.00	0.01	0.97	0.03	0.00	0.98	0.00	0.02
chr17	45706621	797	G	0	0.99	0.00	0.01	0.98	0.01	0.01	0.98	0.01	0.01	0.99	0.00	0.01	0.99	0.00	0.00	0.99	0.00	0.00
chr4	136547503	1107	G	0	0.98	0.02	0.00	0.99	0.00	0.00	0.99	0.01	0.01	0.99	0.01	0.00	0.99	0.01	0.00	0.99	0.01	0.00
chr15	100300136	418	C	0	0.99	0.00	0.01	0.99	0.01	0.01	0.99	0.00	0.01	0.99	0.01	0.01	0.99	0.00	0.00	0.99	0.00	0.00
chr16	92644762	687	C	0	0.99	0.00	0.00	0.98	0.01	0.01	0.98	0.01	0.01	0.99	0.00	0.01	0.99	0.00	0.01	0.99	0.00	0.01
chr19	24277510	914	c	0	0.99	0.00	0.00	0.99	0.00	0.00	0.99	0.00	0.00	0.99	0.01	0.01	0.99	0.00	0.01	0.99	0.00	0.01
chr4	136547393	1045	G	0	1.00	0.00	0.00	0.99	0.01	0.00	1.00	0.00	0.00	1.00	0.00	0.00	0.99	0.00	0.00	0.99	0.00	0.00
chr11	3525803	114	C	0	0.99	0.00	0.01	0.98	0.02	0.00	0.98	0.02	0.00	0.99	0.00	0.01	0.99	0.01	0.00	0.99	0.00	0.01
chr15	100300304	467	C	0	0.99	0.00	0.01	0.98	0.01	0.01	0.98	0.01	0.01	0.98	0.01	0.01	0.99	0.00	0.01	1.00	0.00	0.00
chr16	92644691	659	C	0	0.99	0.00	0.00	0.98	0.01	0.01	0.99	0.00	0.01	0.99	0.00	0.01	0.99	0.00	0.01	0.99	0.00	0.00
chr5	64987488	1485	C	0	0.99	0.00	0.00	0.98	0.01	0.01	0.99	0.00	0.00	0.98	0.00	0.01	0.99	0.00	0.01	0.99	0.00	0.00
chr15	100300147	426	G	0	0.99	0.00	0.01	0.99	0.00	0.00	0.98	0.01	0.01	0.99	0.00	0.01	0.99	0.00	0.00	0.99	0.00	0.00
chrX	7539478	2323	C	0	1.00	0.00	0.00	0.98	0.01	0.01	0.99	0.01	0.01	0.99	0.01	0.01	0.99	0.00	0.01	0.99	0.01	0.01
chr9	100498545	1945	C	0	0.99	0.00	0.00	0.99	0.00	0.01	0.99	0.00	0.00	0.99	0.00	0.01	0.99	0.00	0.00	0.99	0.01	0.00
chr19	24277503	911	c	0	0.99	0.00	0.00	0.99	0.01	0.00	0.99	0.00	0.00	0.98	0.00	0.01	0.99	0.00	0.00	0.99	0.01	0.00

0.98	0.01	0.01	0.99	0.01	0.01	0,00037
0.99	0.01	0.01	0.99	0.01	0.01	0,00036
0.98	0.01	0.01	0.99	0.01	0.01	0,00036
0.99	0.01	0.01	0.99	0.01	0.01	0,00036
0.98	0.01	0.00	0.99	0.01	0.01	0,00036
0.98	0.01	0.01	0.99	0.00	0.01	0,00036
0.99	0.01	0.01	0.99	0.01	0.01	0,00036
0.98	0.01	0.01	0.99	0.01	0.01	0,00036
0.99	0.01	0.01	0.98	0.01	0.01	0,00036
0.99	0.01	0.01	0.98	0.01	0.01	0,00036
0.99	0.01	0.01	0.99	0.01	0.01	0,00036
0.99	0.01	0.01	0.99	0.01	0.00	0,00036
0.99	0.01	0.01	0.99	0.01	0.01	0,00036
0.99	0.01	0.01	0.99	0.01	0.01	0,00036
0.99	0.01	0.01	0.99	0.01	0.01	0,00036
0.98	0.01	0.01	0.99	0.01	0.01	0,00036
0.99	0.01	0.01	0.98	0.01	0.01	0,00036
0.99	0.01	0.01	0.98	0.01	0.01	0,00036
0.99	0.01	0.01	0.99	0.01	0.01	0,00036
0.98	0.01	0.01	0.99	0.00	0.01	0,00035
0.99	0.01	0.01	0.99	0.01	0.01	0,00035
0.99	0.01	0.01	0.99	0.01	0.01	0,00035
0.98	0.01	0.01	0.99	0.01	0.01	0,00035
0.98	0.01	0.01	0.99	0.01	0.01	0,00035
0.98	0.01	0.00	0.99	0.00	0.01	0,00035
0.98	0.01	0.01	0.99	0.01	0.01	0,00035
0.99	0.01	0.01	0.99	0.01	0.01	0,00035
0.99	0.01	0.01	0.98	0.01	0.01	0,00035
0.99	0.01	0.01	0.99	0.01	0.01	0,00035
0.98	0.01	0.01	0.99	0.01	0.01	0,00035
0.99	0.01	0.01	0.99	0.01	0.01	0,00035
0.99	0.01	0.01	0.99	0.01	0.01	0,00034
0.98	0.01	0.01	0.99	0.01	0.01	0,00034
0.98	0.01	0.01	0.99	0.01	0.00	0,00034
0.99	0.01	0.01	0.99	0.01	0.01	0,00034
0.99	0.01	0.01	0.99	0.01	0.01	0,00034
0.99	0.01	0.01	0.99	0.01	0.01	0,00034
0.99	0.01	0.00	0.97	0.01	0.02	0,00034
0.99	0.01	0.01	0.99	0.01	0.01	0,00034
0.99	0.01	0.01	0.99	0.01	0.01	0,00034
0.99	0.01	0.01	0.99	0.01	0.00	0,00034
0.99	0.01	0.01	0.99	0.01	0.01	0,00034
0.99	0.01	0.01	0.99	0.01	0.01	0,00034
0.99	0.01	0.01	0.98	0.01	0.01	0,00034
0.99	0.01	0.01	0.99	0.01	0.01	0,00034
0.99	0.01	0.01	0.99	0.01	0.01	0,00034

chr1;	45706556	759	C	0	0.99	0.01	0.01	0.99	0.01	0.01	0.99	0.00	0.00	0.99	0.01	0.01	0.99	0.00	0.01	1.00	0.00	0.00
chr1(	24277754	1010	g	0	0.99	0.00	0.01	0.99	0.01	0.01	0.98	0.02	0.00	0.99	0.00	0.01	0.99	0.00	0.01	0.99	0.00	0.01
chr1!	100300331	480	C	0	0.99	0.00	0.01	0.99	0.00	0.00	0.99	0.00	0.00	0.98	0.01	0.01	0.99	0.00	0.00	0.98	0.01	0.00
chr8	89376827	1581	g	0	1.00	0.00	0.00	1.00	0.00	0.00	0.99	0.00	0.00	1.00	0.00	0.00	1.00	0.00	0.00	1.00	0.00	0.00
chr9	100498511	1930	C	0	0.99	0.01	0.00	0.99	0.00	0.01	0.99	0.00	0.00	0.99	0.00	0.01	0.98	0.02	0.00	0.99	0.00	0.01
chr1!	100300083	395	C	0	0.99	0.00	0.00	0.99	0.00	0.00	0.99	0.00	0.01	0.99	0.00	0.00	1.00	0.00	0.00	1.00	0.00	0.00
chr8	89376849	1593	g	0	1.00	0.00	0.00	0.99	0.00	0.00	0.99	0.01	0.00	0.99	0.00	0.00	1.00	0.00	0.00	0.99	0.00	0.00
chr9	100498467	1903	C	0	0.99	0.00	0.00	0.99	0.00	0.00	1.00	0.00	0.00	1.00	0.00	0.00	1.00	0.00	0.00	1.00	0.00	0.00
chr4	139783779	1329	C	0	1.00	0.00	0.00	1.00	0.00	0.00	0.99	0.00	0.00	0.98	0.02	0.00	0.98	0.00	0.02	0.98	0.02	0.00
chr4	139783781	1330	C	0	0.99	0.00	0.01	0.98	0.02	0.00	0.99	0.01	0.00	0.98	0.02	0.00	0.99	0.00	0.01	0.99	0.00	0.01
chr5	64987529	1503	C	0	0.99	0.01	0.00	0.99	0.00	0.00	0.99	0.01	0.00	0.98	0.00	0.01	0.99	0.00	0.00	0.99	0.01	0.00
chr9	100498499	1922	G	0	0.99	0.01	0.00	1.00	0.00	0.00	0.99	0.00	0.00	0.99	0.00	0.00	0.99	0.00	0.00	0.99	0.01	0.00
chr1(	92644740	679	C	0	0.99	0.00	0.00	0.98	0.01	0.01	0.99	0.01	0.01	0.99	0.00	0.01	0.98	0.01	0.01	0.99	0.00	0.01
chr1(	24277686	989	G	0	0.99	0.01	0.00	0.98	0.02	0.00	0.99	0.00	0.00	0.99	0.00	0.01	0.99	0.00	0.01	0.99	0.00	0.01
chrX	7476581	2132	C	1	0.99	0.01	0.00	0.99	0.01	0.00	1.00	0.00	0.00	0.99	0.01	0.00	0.95	0.01	0.04	0.98	0.01	0.00
chr1	3525954	189	g	0	0.99	0.00	0.00	0.98	0.01	0.01	0.99	0.01	0.01	0.99	0.00	0.01	0.99	0.00	0.01	1.00	0.00	0.00
chr1(	92644547	631	C	0	0.99	0.00	0.00	0.99	0.00	0.01	0.99	0.00	0.01	0.97	0.01	0.01	0.99	0.00	0.00	0.99	0.00	0.00
chr4	139783876	1384	C	0	0.99	0.01	0.00	0.99	0.00	0.00	0.99	0.00	0.01	0.98	0.00	0.02	0.96	0.04	0.00	0.99	0.00	0.01
chr1(	24277604	962	C	0	1.00	0.00	0.00	0.98	0.00	0.01	0.99	0.01	0.00	0.99	0.00	0.01	0.99	0.00	0.01	0.99	0.00	0.01
chr4	139783768	1322	G	0	0.99	0.00	0.01	0.99	0.01	0.00	1.00	0.00	0.00	0.98	0.02	0.00	0.99	0.00	0.01	0.99	0.01	0.00
chr4	139783847	1364	C	0	0.99	0.01	0.00	0.99	0.00	0.00	0.99	0.01	0.00	0.99	0.01	0.00	0.99	0.00	0.01	0.98	0.00	0.01
chr8	120115771	1796	C	0	0.98	0.02	0.00	0.99	0.00	0.00	0.99	0.00	0.00	0.99	0.01	0.00	0.99	0.01	0.00	0.99	0.00	0.00
chr1	3525950	188	g	0	0.99	0.00	0.01	0.98	0.01	0.01	0.98	0.01	0.01	0.99	0.00	0.01	0.99	0.00	0.01	1.00	0.00	0.00
chrX	7539275	2218	G	0	1.00	0.00	0.00	0.98	0.00	0.01	0.99	0.00	0.01	0.99	0.00	0.01	0.99	0.01	0.00	0.99	0.01	0.00
chr1(	92644420	548	G	0	0.99	0.01	0.00	1.00	0.00	0.00	0.99	0.01	0.00	0.99	0.01	0.00	0.99	0.01	0.00	0.99	0.00	0.01
chr1(	24277617	969	G	0	0.99	0.00	0.01	0.99	0.01	0.01	0.99	0.00	0.01	0.99	0.00	0.01	0.99	0.00	0.00	0.99	0.00	0.01
chr1(	24277757	1011	g	0	1.00	0.00	0.00	0.99	0.01	0.01	1.00	0.00	0.00	1.00	0.00	0.00	1.00	0.00	0.00	0.99	0.00	0.00
chr9	100498446	1891	G	0	0.99	0.00	0.00	0.99	0.00	0.00	0.99	0.00	0.01	0.99	0.01	0.00	0.99	0.00	0.00	0.99	0.00	0.00
chr1(	92644747	682	C	0	0.99	0.00	0.00	0.98	0.01	0.01	0.98	0.01	0.01	0.99	0.00	0.01	0.99	0.00	0.01	0.99	0.00	0.01
chr8	120115728	1778	C	0	0.99	0.00	0.00	0.99	0.00	0.01	0.98	0.00	0.02	1.00	0.00	0.00	0.99	0.00	0.00	0.99	0.00	0.01
chrX	7476555	2115	C	0	1.00	0.00	0.00	1.00	0.00	0.00	1.00	0.00	0.00	1.00	0.00	0.00	0.99	0.00	0.01	1.00	0.00	0.00
chr1(	92644496	599	C	0	0.99	0.00	0.00	0.97	0.01	0.01	0.99	0.00	0.01	0.99	0.00	0.01	0.99	0.00	0.01	0.99	0.01	0.00
chr1!	61868917	329	G	0	0.99	0.00	0.01	0.99	0.01	0.01	0.99	0.00	0.01	0.99	0.00	0.01	0.97	0.03	0.00	0.98	0.00	0.01
chr1;	45706614	794	G	0	0.99	0.00	0.01	0.99	0.01	0.01	0.99	0.00	0.01	0.99	0.00	0.00	1.00	0.00	0.00	0.99	0.00	0.01
chr8	120115593	1710	G	0	0.99	0.00	0.01	0.99	0.00	0.00	0.99	0.00	0.00	0.99	0.01	0.00	0.99	0.01	0.00	0.99	0.00	0.00
chr8	120115655	1741	C	0	0.99	0.00	0.01	0.99	0.00	0.00	0.99	0.01	0.00	1.00	0.00	0.00	0.99	0.00	0.01	0.99	0.00	0.01
chr9	100498470	1905	C	0	0.99	0.00	0.01	0.98	0.02	0.00	0.99	0.01	0.00	0.97	0.03	0.00	0.99	0.00	0.01	0.99	0.01	0.00
chr1(	24277668	984	G	0	1.00	0.00	0.00	0.99	0.01	0.00	0.99	0.01	0.00	0.99	0.01	0.00	0.99	0.00	0.00	0.98	0.02	0.00
chr4	136547426	1061	C	0	0.99	0.01	0.00	0.99	0.00	0.00	0.99	0.01	0.00	0.99	0.00	0.01	0.99	0.00	0.00	0.99	0.00	0.00
chr1(	24277681	988	G	0	0.99	0.01	0.00	0.99	0.01	0.00	0.99	0.00	0.01	0.99	0.00	0.00	0.99	0.00	0.01	0.99	0.01	0.00
chr9	100498514	1931	C	0	0.99	0.01	0.00	0.99	0.00	0.00	0.99	0.00	0.01	0.98	0.02	0.00	0.99	0.00	0.01	0.98	0.02	0.00
chr4	139783640	1253	C	0	1.00	0.00	0.00	0.99	0.00	0.01	0.99	0.00	0.00	0.99	0.01	0.00	0.98	0.02	0.00	0.99	0.00	0.01
chr1!	100300080	393	C	0	0.96	0.04	0.00	0.99	0.00	0.00	0.99	0.00	0.00	0.99	0.00	0.01	0.99	0.00	0.00	0.99	0.00	0.01
chr5	64987391	1439	G	0	0.99	0.00	0.00	0.99	0.00	0.01	0.99	0.00	0.01	0.99	0.00	0.01	0.99	0.00	0.00	0.99	0.00	0.01
chrX	7476586	2136	G	1	0.99	0.01	0.00	1.00	0.00	0.00	1.00	0.00	0.00	0.99	0.00	0.01	1.00	0.00	0.00	0.99	0.00	0.01
chr1(	92644514	613	C	0	0.99	0.00	0.00	0.99	0.01	0.01	0.99	0.01	0.00	0.99	0.00	0.00	0.99	0.00	0.01	1.00	0.00	0.00

0.99	0.01	0.01	0.99	0.01	0.01	0,00033
0.99	0.01	0.01	0.98	0.00	0.01	0,00033
0.99	0.01	0.01	0.99	0.01	0.01	0,00033
0.99	0.00	0.01	0.97	0.02	0.01	0,00033
0.99	0.01	0.01	0.99	0.00	0.01	0,00033
0.99	0.01	0.01	0.99	0.01	0.00	0,00033
0.99	0.01	0.01	0.97	0.02	0.01	0,00033
0.99	0.01	0.00	0.98	0.01	0.01	0,00033
0.99	0.01	0.00	0.99	0.01	0.01	0,00033
0.99	0.01	0.01	0.99	0.01	0.01	0,00033
0.99	0.01	0.01	0.99	0.01	0.01	0,00033
0.99	0.01	0.01	0.99	0.01	0.01	0,00033
0.98	0.01	0.01	0.99	0.01	0.01	0,00033
0.99	0.01	0.00	0.99	0.00	0.01	0,00032
0.99	0.01	0.00	0.98	0.01	0.01	0,00032
0.98	0.01	0.01	0.99	0.01	0.01	0,00032
0.99	0.01	0.01	0.99	0.01	0.01	0,00032
0.99	0.01	0.01	0.99	0.01	0.01	0,00032
0.99	0.01	0.01	0.99	0.00	0.01	0,00032
0.99	0.00	0.01	0.99	0.01	0.01	0,00032
0.99	0.01	0.00	0.99	0.01	0.01	0,00032
0.99	0.01	0.01	0.99	0.01	0.00	0,00032
0.99	0.01	0.01	0.99	0.00	0.01	0,00032
0.99	0.00	0.01	0.99	0.01	0.01	0,00032
0.99	0.01	0.01	0.99	0.01	0.01	0,00032
0.99	0.01	0.01	0.99	0.01	0.01	0,00032
0.99	0.00	0.01	0.98	0.01	0.01	0,00032
0.99	0.01	0.01	0.99	0.01	0.00	0,00032
0.99	0.01	0.01	0.99	0.01	0.01	0,00031
0.98	0.01	0.01	0.99	0.01	0.01	0,00031
0.99	0.00	0.00	0.97	0.02	0.01	0,00031
0.99	0.01	0.01	0.99	0.01	0.01	0,00031
0.98	0.01	0.01	0.99	0.01	0.01	0,00031
0.99	0.01	0.01	0.99	0.00	0.01	0,00031
0.99	0.00	0.01	0.99	0.01	0.00	0,00031
0.99	0.01	0.01	0.99	0.01	0.01	0,00031
0.99	0.01	0.01	0.98	0.01	0.01	0,00031
0.99	0.01	0.01	0.99	0.01	0.01	0,00031
0.99	0.01	0.01	0.99	0.01	0.01	0,00031
0.99	0.01	0.01	0.99	0.01	0.01	0,00031
0.99	0.01	0.01	0.99	0.01	0.01	0,00031
0.99	0.00	0.01	0.98	0.01	0.01	0,00031
0.99	0.01	0.01	0.99	0.01	0.01	0,00031
0.99	0.01	0.01	0.99	0.01	0.01	0,00031
0.99	0.01	0.00	0.99	0.00	0.01	0,00031
0.99	0.01	0.01	0.99	0.01	0.01	0,0003

chr1!	100300306	468	G	0	0.99	0.00	0.01	0.99	0.01	0.00	0.98	0.01	0.00	0.99	0.01	0.00	0.99	0.00	0.01	0.99	0.01	0.00
chr1(	92644501	602	C	0	1.00	0.00	0.00	0.99	0.00	0.01	0.98	0.01	0.01	0.99	0.00	0.01	0.99	0.00	0.01	0.99	0.00	0.00
chr1(	92644573	645	C	0	1.00	0.00	0.00	0.99	0.01	0.01	0.99	0.00	0.01	0.99	0.00	0.01	0.98	0.01	0.01	0.99	0.00	0.00
chr4	136547376	1034	G	0	0.96	0.04	0.00	0.99	0.00	0.00	0.99	0.01	0.00	0.99	0.00	0.01	0.98	0.00	0.02	0.99	0.01	0.00
chr4	136547737	1202	C	0	0.99	0.00	0.01	0.99	0.00	0.00	0.99	0.00	0.00	1.00	0.00	0.00	0.99	0.01	0.00	0.99	0.01	0.00
chr5	64987386	1437	G	0	0.99	0.01	0.00	0.99	0.00	0.00	0.99	0.01	0.00	1.00	0.00	0.00	0.99	0.00	0.01	0.99	0.00	0.01
chr1;	45706554	758	C	0	0.99	0.00	0.01	0.99	0.00	0.00	0.99	0.00	0.00	0.98	0.01	0.01	0.99	0.00	0.01	0.99	0.00	0.00
chr9	100498525	1937	C	0	1.00	0.00	0.00	0.99	0.01	0.00	1.00	0.00	0.00	0.99	0.00	0.00	0.98	0.02	0.00	0.99	0.01	0.00
chr9	100498557	1952	C	0	0.99	0.00	0.00	0.99	0.01	0.01	0.99	0.00	0.01	0.99	0.00	0.01	0.99	0.00	0.01	1.00	0.00	0.00
chrX	7476374	2020	G	1	1.00	0.00	0.00	1.00	0.00	0.00	1.00	0.00	0.00	0.99	0.00	0.01	1.00	0.00	0.00	1.00	0.00	0.00
chr1(	92644474	583	C	0	0.99	0.00	0.01	0.99	0.00	0.01	0.99	0.00	0.00	0.99	0.00	0.00	0.99	0.00	0.01	0.99	0.00	0.00
chr1(	92644639	655	c	0	0.99	0.00	0.00	0.99	0.01	0.01	0.99	0.00	0.01	0.99	0.00	0.01	0.99	0.00	0.01	0.99	0.00	0.00
chr5	64987539	1507	G	0	0.99	0.01	0.00	0.99	0.00	0.00	0.99	0.00	0.01	0.99	0.00	0.01	0.99	0.00	0.01	0.99	0.00	0.01
chr1!	100300144	423	G	0	0.99	0.00	0.01	0.99	0.01	0.01	0.99	0.00	0.00	0.99	0.00	0.01	0.99	0.00	0.01	1.00	0.00	0.00
chr1!	100300314	472	C	0	0.99	0.00	0.01	0.99	0.00	0.00	0.96	0.03	0.01	0.99	0.00	0.00	1.00	0.00	0.00	0.99	0.00	0.00
chr1;	45706551	757	C	0	0.99	0.00	0.01	0.99	0.00	0.00	0.99	0.00	0.00	0.98	0.01	0.01	0.99	0.00	0.01	0.99	0.00	0.00
chr5	64987551	1512	G	0	1.00	0.00	0.00	0.99	0.01	0.01	0.99	0.00	0.01	0.99	0.00	0.01	0.98	0.02	0.00	0.99	0.00	0.01
chr9	100498583	1966	C	0	0.99	0.00	0.01	0.99	0.01	0.00	0.99	0.00	0.00	0.99	0.00	0.01	0.99	0.00	0.01	0.97	0.03	0.00
chr1!	24277517	919	c	0	1.00	0.00	0.00	0.99	0.00	0.01	0.99	0.00	0.00	0.99	0.00	0.01	0.99	0.00	0.00	0.99	0.00	0.00
chr1'	3525948	187	g	0	0.99	0.00	0.00	0.98	0.01	0.01	0.99	0.01	0.01	0.99	0.00	0.01	0.99	0.00	0.00	1.00	0.00	0.00
chr1;	45706476	716	C	0	1.00	0.00	0.00	0.99	0.01	0.00	0.99	0.01	0.00	0.99	0.00	0.00	0.99	0.00	0.01	0.99	0.00	0.01
chrX	7476599	2143	C	1	1.00	0.00	0.00	1.00	0.00	0.00	0.99	0.00	0.01	0.99	0.01	0.00	0.96	0.01	0.03	0.99	0.01	0.00
chr1'	3525958	190	g	0	0.99	0.00	0.00	0.98	0.01	0.01	0.98	0.01	0.01	0.99	0.00	0.01	0.99	0.00	0.01	1.00	0.00	0.00
chr1(	92644493	597	C	0	1.00	0.00	0.00	0.99	0.00	0.01	0.99	0.00	0.00	0.99	0.00	0.00	0.99	0.01	0.01	0.99	0.00	0.01
chr1(	92644525	618	C	0	0.98	0.00	0.01	0.99	0.00	0.01	0.99	0.00	0.01	0.99	0.00	0.00	0.99	0.00	0.00	0.99	0.00	0.01
chr1!	24277516	918	c	0	0.99	0.00	0.00	0.99	0.01	0.01	1.00	0.00	0.00	0.99	0.00	0.00	0.99	0.00	0.01	0.99	0.00	0.00
chr4	136547495	1101	C	0	0.99	0.00	0.00	0.98	0.01	0.01	0.99	0.00	0.00	1.00	0.00	0.00	0.99	0.00	0.00	1.00	0.00	0.00
chrX	7476661	2189	C	1	1.00	0.00	0.00	1.00	0.00	0.00	1.00	0.00	0.00	1.00	0.00	0.00	0.96	0.01	0.03	0.99	0.01	0.00
chr1;	45706626	799	C	0	0.99	0.00	0.00	1.00	0.00	0.00	0.99	0.00	0.01	0.98	0.02	0.00	0.99	0.00	0.01	0.99	0.00	0.01
chrX	7539505	2337	G	0	1.00	0.00	0.00	1.00	0.00	0.00	0.99	0.00	0.00	1.00	0.00	0.00	1.00	0.00	0.00	1.00	0.00	0.00
chr4	136547365	1032	G	0	1.00	0.00	0.00	1.00	0.00	0.00	1.00	0.00	0.00	1.00	0.00	0.00	1.00	0.00	0.00	1.00	0.00	0.00
chr1;	45706458	706	G	0	1.00	0.00	0.00	1.00	0.00	0.00	1.00	0.00	0.00	1.00	0.00	0.00	1.00	0.00	0.00	1.00	0.00	0.00
chr5	64987624	1540	C	0	1.00	0.00	0.00	0.99	0.00	0.00	0.99	0.00	0.01	1.00	0.00	0.00	0.99	0.00	0.00	0.99	0.00	0.00
chr1'	3525968	192	g	0	0.99	0.00	0.01	0.98	0.01	0.01	0.98	0.01	0.01	0.99	0.00	0.01	0.99	0.00	0.01	1.00	0.00	0.00
chr1(	92644559	639	C	0	1.00	0.00	0.00	0.99	0.00	0.01	0.99	0.00	0.00	0.99	0.00	0.01	0.99	0.00	0.01	0.99	0.01	0.00
chr1!	24277441	888	c	0	1.00	0.00	0.00	0.99	0.00	0.00	0.99	0.00	0.01	0.99	0.00	0.00	0.99	0.01	0.00	0.99	0.01	0.00
chr8	89376974	1654	C	0	1.00	0.00	0.00	0.99	0.01	0.00	0.99	0.00	0.00	1.00	0.00	0.00	0.99	0.00	0.01	1.00	0.00	0.00
chr1!	100300394	495	C	0	0.99	0.00	0.01	0.99	0.00	0.00	0.99	0.00	0.01	0.99	0.01	0.01	1.00	0.00	0.00	1.00	0.00	0.00
chr9	100498546	1946	C	0	0.98	0.01	0.01	0.99	0.00	0.00	0.99	0.00	0.00	0.99	0.00	0.01	0.99	0.01	0.01	0.99	0.00	0.00
chrX	7539655	2424	C	0	1.00	0.00	0.00	0.99	0.01	0.00	0.99	0.00	0.01	0.98	0.02	0.00	0.99	0.00	0.01	0.98	0.00	0.02
chr1!	61868870	305	G	0	0.99	0.00	0.00	0.99	0.01	0.01	0.99	0.00	0.00	0.99	0.00	0.00	1.00	0.00	0.00	0.99	0.00	0.01
chr4	136547645	1181	C	0	1.00	0.00	0.00	0.99	0.00	0.00	1.00	0.00	0.00	0.99	0.00	0.01	0.99	0.00	0.01	0.99	0.00	0.01
chr5	64987412	1443	G	0	0.99	0.01	0.00	0.99	0.00	0.00	0.99	0.00	0.01	0.99	0.00	0.01	0.99	0.00	0.01	0.99	0.01	0.00
chr4	136547525	1121	C	0	0.99	0.00	0.01	0.98	0.01	0.01	0.99	0.00	0.01	0.99	0.00	0.01	0.99	0.01	0.00	0.99	0.00	0.00
chr5	64987492	1488	C	0	0.99	0.00	0.00	0.99	0.01	0.01	0.98	0.01	0.01	0.99	0.00	0.01	0.99	0.00	0.00	0.99	0.00	0.00
chr1(	92644352	525	G	0	0.99	0.00	0.00	0.99	0.01	0.01	0.99	0.01	0.00	0.99	0.00	0.00	1.00	0.00	0.00	1.00	0.00	0.00

0.99	0.01	0.01	0.99	0.01	0.00	0,0003
0.99	0.01	0.01	0.99	0.01	0.01	0,0003
0.99	0.01	0.01	0.99	0.01	0.01	0,0003
0.98	0.01	0.00	0.99	0.01	0.01	0,0003
0.99	0.01	0.01	0.99	0.01	0.01	0,0003
0.99	0.01	0.01	0.99	0.01	0.01	0,0003
0.99	0.01	0.01	0.99	0.01	0.01	0,0003
0.99	0.01	0.01	0.99	0.01	0.01	0,0003
0.99	0.01	0.01	0.99	0.01	0.01	0,0003
0.99	0.00	0.00	0.98	0.01	0.01	0,0003
0.99	0.01	0.01	0.99	0.01	0.01	0,0003
0.99	0.01	0.01	0.99	0.01	0.01	0,0003
0.99	0.01	0.01	0.99	0.01	0.01	0,0003
0.99	0.01	0.01	0.99	0.00	0.01	0,0003
0.98	0.01	0.01	0.99	0.01	0.01	0,0003
0.99	0.01	0.01	0.99	0.01	0.01	0,0003
0.99	0.01	0.01	0.99	0.01	0.01	0,0003
0.99	0.01	0.01	0.99	0.01	0.01	0,0003
0.99	0.01	0.01	0.99	0.01	0.01	0,00029
0.98	0.01	0.01	0.99	0.01	0.01	0,00029
0.99	0.01	0.01	0.99	0.00	0.01	0,00029
0.99	0.00	0.01	0.98	0.01	0.01	0,00029
0.99	0.01	0.01	0.99	0.00	0.01	0,00029
0.99	0.01	0.01	0.99	0.01	0.00	0,00029
0.99	0.01	0.01	0.99	0.01	0.01	0,00029
0.99	0.01	0.01	0.99	0.00	0.01	0,00029
0.99	0.01	0.01	0.99	0.00	0.01	0,00029
0.99	0.01	0.00	0.98	0.02	0.01	0,00029
0.99	0.01	0.01	0.98	0.01	0.01	0,00029
0.99	0.00	0.01	0.97	0.01	0.02	0,00029
0.99	0.01	0.00	0.96	0.03	0.01	0,00029
0.99	0.00	0.00	0.96	0.01	0.02	0,00029
0.99	0.01	0.01	0.99	0.00	0.01	0,00029
0.99	0.01	0.01	0.99	0.00	0.01	0,00029
0.99	0.01	0.01	0.99	0.01	0.01	0,00029
0.99	0.00	0.01	0.99	0.01	0.01	0,00029
0.99	0.01	0.01	0.99	0.00	0.01	0,00029
0.99	0.01	0.01	0.99	0.01	0.01	0,00029
0.99	0.01	0.01	0.99	0.01	0.01	0,00029
0.99	0.01	0.01	0.99	0.00	0.01	0,00029
0.99	0.01	0.01	0.99	0.00	0.01	0,00029
0.99	0.01	0.01	0.99	0.00	0.01	0,00029
0.99	0.01	0.01	0.99	0.01	0.01	0,00029
0.99	0.01	0.01	0.99	0.01	0.01	0,00028
0.99	0.01	0.01	0.99	0.00	0.01	0,00028
0.99	0.01	0.01	0.99	0.01	0.00	0,00028

chr8	89376916	1631	G	0	1.00	0.00	0.00	0.99	0.00	0.00	0.99	0.01	0.00	0.99	0.01	0.01	1.00	0.00	0.00	0.99	0.00	0.00
chr1!	100300096	401	C	0	0.99	0.00	0.01	0.99	0.00	0.00	0.99	0.01	0.01	0.99	0.00	0.00	1.00	0.00	0.00	1.00	0.00	0.00
chr1(	92644527	619	C	0	0.99	0.01	0.00	0.99	0.00	0.01	0.99	0.00	0.01	0.99	0.01	0.01	0.99	0.00	0.01	0.99	0.00	0.00
chr1;	45706539	749	G	0	1.00	0.00	0.00	1.00	0.00	0.00	0.98	0.01	0.01	0.99	0.00	0.01	0.99	0.00	0.01	0.99	0.00	0.01
chr5	64987501	1491	C	0	0.99	0.00	0.01	0.99	0.01	0.01	0.99	0.00	0.00	0.99	0.00	0.01	0.99	0.00	0.01	1.00	0.00	0.00
chr8	120115736	1782	C	0	0.99	0.00	0.00	1.00	0.00	0.00	0.98	0.01	0.01	0.99	0.00	0.00	0.99	0.01	0.00	0.99	0.00	0.01
chrX	7539615	2404	C	0	0.99	0.01	0.00	0.99	0.00	0.01	0.99	0.01	0.00	0.99	0.01	0.00	0.99	0.00	0.01	0.98	0.02	0.00
chrX	7539643	2420	C	0	0.98	0.00	0.02	0.99	0.00	0.01	0.98	0.01	0.00	0.99	0.00	0.01	0.99	0.00	0.01	0.99	0.00	0.01
chr1!	61868702	224	C	0	0.99	0.00	0.01	0.99	0.01	0.00	0.99	0.00	0.01	0.99	0.00	0.01	0.99	0.00	0.01	0.99	0.00	0.01
chr1!	61868853	298	G	0	0.99	0.00	0.01	1.00	0.00	0.00	0.99	0.00	0.01	0.98	0.01	0.00	0.99	0.00	0.01	0.99	0.00	0.01
chr1(	92644771	688	C	0	0.99	0.00	0.00	0.98	0.01	0.01	0.99	0.01	0.01	0.99	0.00	0.01	0.99	0.00	0.01	0.99	0.00	0.01
chr9	100498482	1912	G	0	0.99	0.01	0.00	0.99	0.00	0.00	0.99	0.01	0.00	0.99	0.00	0.00	0.99	0.00	0.01	0.99	0.01	0.00
chr1!	100300118	411	C	0	0.99	0.00	0.00	0.99	0.00	0.01	0.99	0.00	0.01	0.99	0.00	0.00	1.00	0.00	0.00	0.99	0.00	0.00
chr1(	92644441	559	G	0	0.99	0.00	0.01	1.00	0.00	0.00	0.99	0.00	0.00	0.99	0.01	0.01	0.99	0.00	0.00	0.99	0.00	0.00
chr1!	100300416	499	C	0	0.99	0.00	0.00	0.99	0.01	0.01	0.97	0.02	0.01	0.99	0.00	0.01	1.00	0.00	0.00	1.00	0.00	0.00
chr1;	45706628	800	G	0	0.99	0.00	0.01	0.99	0.01	0.01	0.99	0.00	0.01	0.99	0.00	0.01	0.99	0.00	0.00	1.00	0.00	0.00
chr8	120115647	1737	G	0	0.99	0.00	0.00	0.99	0.00	0.00	0.99	0.00	0.00	1.00	0.00	0.00	0.99	0.01	0.00	0.99	0.01	0.00
chrX	7476547	2108	G	0	0.99	0.00	0.00	0.99	0.01	0.01	1.00	0.00	0.00	0.98	0.01	0.01	0.99	0.01	0.01	1.00	0.00	0.00
chr9	100498403	1869	G	0	0.99	0.00	0.01	0.99	0.00	0.01	0.99	0.01	0.00	0.99	0.00	0.01	0.99	0.01	0.00	0.99	0.00	0.01
chr1;	45706689	828	C	0	1.00	0.00	0.00	0.99	0.00	0.00	1.00	0.00	0.00	0.99	0.00	0.00	0.98	0.02	0.00	0.99	0.01	0.00
chr1(	92644318	514	G	0	1.00	0.00	0.00	1.00	0.00	0.00	0.99	0.00	0.00	0.98	0.01	0.01	1.00	0.00	0.00	0.99	0.01	0.00
chr8	120115803	1810	G	0	1.00	0.00	0.00	0.99	0.00	0.01	0.99	0.01	0.00	0.99	0.00	0.01	0.99	0.00	0.01	0.99	0.01	0.00
chr1!	100300355	488	C	0	0.99	0.00	0.00	0.99	0.01	0.01	1.00	0.00	0.00	0.99	0.01	0.01	1.00	0.00	0.00	0.99	0.00	0.00
chr8	120115602	1714	G	0	0.99	0.00	0.00	0.99	0.00	0.00	0.99	0.01	0.00	0.99	0.00	0.01	0.99	0.00	0.00	0.99	0.00	0.00
chrX	7476548	2109	C	1	1.00	0.00	0.00	0.99	0.01	0.00	1.00	0.00	0.00	0.99	0.01	0.00	0.97	0.01	0.03	0.99	0.01	0.00
chr1	3525964	191	g	0	0.99	0.00	0.01	0.98	0.01	0.01	0.98	0.01	0.01	0.99	0.00	0.01	0.99	0.00	0.01	1.00	0.00	0.00
chr1!	100300131	416	C	0	0.99	0.00	0.00	0.99	0.00	0.00	0.99	0.00	0.00	0.99	0.00	0.01	1.00	0.00	0.00	0.99	0.00	0.00
chr4	139783711	1293	C	0	0.97	0.03	0.00	0.99	0.00	0.00	0.99	0.01	0.00	0.99	0.00	0.01	0.99	0.00	0.00	0.98	0.01	0.00
chr5	64987614	1535	C	0	0.99	0.01	0.00	1.00	0.00	0.00	1.00	0.00	0.00	1.00	0.00	0.00	1.00	0.00	0.00	1.00	0.00	0.00
chr1!	24277417	884	c	0	1.00	0.00	0.00	1.00	0.00	0.00	0.98	0.00	0.01	0.99	0.01	0.00	0.99	0.00	0.00	1.00	0.00	0.00
chr1!	24277625	973	C	0	0.99	0.00	0.00	1.00	0.00	0.00	0.99	0.00	0.00	0.99	0.00	0.01	0.99	0.00	0.00	0.99	0.00	0.00
chrX	7539396	2268	C	0	0.99	0.00	0.01	0.99	0.00	0.01	0.98	0.00	0.01	0.98	0.02	0.00	0.99	0.00	0.01	0.98	0.02	0.00
chrX	7539568	2376	C	0	0.99	0.00	0.01	0.99	0.00	0.00	0.99	0.00	0.01	0.99	0.00	0.01	0.98	0.02	0.00	0.99	0.00	0.01
chr1	3525774	95	C	0	0.99	0.00	0.01	0.99	0.00	0.00	0.99	0.00	0.01	0.99	0.00	0.01	0.99	0.01	0.00	0.99	0.00	0.00
chr1;	45706613	793	G	0	0.99	0.00	0.01	0.99	0.00	0.01	0.98	0.01	0.01	0.99	0.00	0.00	1.00	0.00	0.00	1.00	0.00	0.00
chr4	136547690	1192	C	0	1.00	0.00	0.00	1.00	0.00	0.00	0.99	0.00	0.00	0.99	0.01	0.00	1.00	0.00	0.00	0.99	0.01	0.00
chr5	64987458	1470	C	0	0.99	0.00	0.00	0.99	0.01	0.01	0.99	0.01	0.00	0.99	0.00	0.01	0.99	0.01	0.00	1.00	0.00	0.00
chr8	120115610	1718	G	0	0.99	0.01	0.00	0.99	0.01	0.00	0.99	0.01	0.00	0.98	0.02	0.00	0.99	0.00	0.01	0.99	0.01	0.00
chr1(	92644480	587	G	0	0.99	0.00	0.00	0.99	0.00	0.00	1.00	0.00	0.00	0.99	0.01	0.00	0.99	0.01	0.00	0.99	0.00	0.00
chr8	120115730	1779	C	0	1.00	0.00	0.00	0.99	0.00	0.01	0.98	0.01	0.01	0.99	0.00	0.00	0.99	0.00	0.00	0.99	0.00	0.00
chr8	120115843	1831	C	0	1.00	0.00	0.00	1.00	0.00	0.00	1.00	0.00	0.00	0.99	0.00	0.00	0.99	0.00	0.01	0.99	0.00	0.01
chr1!	61868656	204	C	0	0.99	0.00	0.01	0.99	0.00	0.00	0.98	0.01	0.00	0.99	0.00	0.01	0.99	0.00	0.00	0.99	0.00	0.00
chr1(	92644361	528	G	0	1.00	0.00	0.00	0.98	0.01	0.01	0.99	0.00	0.00	0.99	0.00	0.00	0.99	0.01	0.00	1.00	0.00	0.00
chr1(	92644535	625	C	0	0.99	0.01	0.01	0.99	0.00	0.01	0.99	0.00	0.00	0.98	0.01	0.01	0.99	0.01	0.00	0.99	0.00	0.00
chr4	136547759	1213	C	0	0.99	0.00	0.01	0.98	0.01	0.01	1.00	0.00	0.00	0.99	0.00	0.00	0.99	0.00	0.01	0.99	0.01	0.00
chr9	100498547	1947	C	0	0.99	0.01	0.01	0.99	0.00	0.00	1.00	0.00	0.00	0.99	0.00	0.01	0.99	0.01	0.01	0.99	0.00	0.00

0.99	0.01	0.01	0.99	0.01	0.01	0,00028
0.99	0.01	0.01	0.99	0.01	0.01	0,00028
0.99	0.01	0.01	0.99	0.01	0.01	0,00028
0.99	0.01	0.01	0.99	0.00	0.01	0,00028
0.99	0.01	0.01	0.99	0.00	0.01	0,00028
0.99	0.01	0.01	0.99	0.01	0.01	0,00028
0.99	0.01	0.01	0.99	0.01	0.00	0,00028
0.99	0.01	0.01	0.99	0.00	0.01	0,00028
0.99	0.00	0.01	0.99	0.00	0.01	0,00028
0.99	0.00	0.01	0.99	0.00	0.01	0,00028
0.99	0.01	0.01	0.99	0.01	0.01	0,00028
0.99	0.01	0.01	0.99	0.01	0.01	0,00028
0.99	0.01	0.01	0.99	0.01	0.01	0,00028
0.99	0.00	0.01	0.99	0.01	0.01	0,00028
0.99	0.01	0.01	0.99	0.01	0.01	0,00028
0.99	0.01	0.01	0.99	0.00	0.01	0,00028
0.99	0.01	0.01	0.99	0.01	0.01	0,00028
0.99	0.01	0.01	0.99	0.01	0.01	0,00028
0.99	0.01	0.01	0.99	0.01	0.01	0,00028
0.99	0.00	0.00	0.99	0.01	0.00	0,00028
0.99	0.01	0.01	0.99	0.01	0.01	0,00028
0.99	0.01	0.01	0.99	0.01	0.01	0,00028
0.99	0.01	0.01	0.99	0.01	0.01	0,00028
0.99	0.01	0.01	0.99	0.00	0.01	0,00028
0.99	0.01	0.00	0.99	0.01	0.00	0,00028
0.98	0.01	0.01	0.99	0.01	0.01	0,00027
0.99	0.01	0.01	0.99	0.01	0.01	0,00027
0.99	0.01	0.01	0.99	0.01	0.01	0,00027
0.99	0.01	0.00	0.99	0.01	0.01	0,00027
0.99	0.01	0.01	0.99	0.01	0.01	0,00027
0.99	0.01	0.01	0.99	0.01	0.01	0,00027
0.99	0.00	0.01	0.99	0.01	0.01	0,00027
0.99	0.00	0.01	0.99	0.01	0.01	0,00027
0.99	0.00	0.01	0.99	0.01	0.01	0,00027
0.99	0.01	0.01	0.99	0.01	0.01	0,00027
0.99	0.01	0.01	0.99	0.01	0.00	0,00027
0.99	0.01	0.01	0.99	0.01	0.01	0,00027
0.99	0.01	0.00	0.99	0.01	0.00	0,00027
0.99	0.01	0.01	0.99	0.01	0.01	0,00027
0.99	0.01	0.01	0.99	0.01	0.01	0,00027
0.99	0.00	0.01	0.99	0.01	0.01	0,00027
0.99	0.01	0.01	0.99	0.00	0.01	0,00027
0.99	0.01	0.01	0.99	0.01	0.00	0,00027
0.99	0.01	0.01	0.99	0.01	0.01	0,00027
0.99	0.00	0.01	0.99	0.01	0.01	0,00027
0.99	0.01	0.01	0.99	0.01	0.01	0,00027

chr1(	92644532	622	C	0	0.99	0.00	0.00	0.99	0.00	0.00	0.99	0.00	0.01	0.99	0.00	0.01	0.99	0.00	0.00	0.99	0.00	0.00
chr1(	92644331	519	G	0	0.99	0.00	0.01	0.99	0.00	0.00	1.00	0.00	0.00	0.99	0.00	0.00	1.00	0.00	0.00	1.00	0.00	0.00
chr9	100498561	1955	C	0	0.99	0.00	0.00	0.99	0.00	0.00	0.99	0.00	0.00	1.00	0.00	0.00	0.99	0.01	0.01	0.99	0.00	0.00
chr1'	3525642	26	G	0	0.99	0.00	0.01	0.99	0.00	0.00	0.99	0.00	0.00	0.99	0.00	0.00	1.00	0.00	0.00	0.99	0.00	0.00
chr8	120115664	1745	G	0	0.99	0.01	0.00	0.99	0.00	0.01	0.99	0.00	0.01	0.99	0.00	0.01	0.99	0.00	0.01	0.99	0.01	0.00
chr9	100498572	1961	C	0	0.99	0.00	0.00	1.00	0.00	0.00	0.99	0.01	0.00	1.00	0.00	0.00	0.99	0.00	0.01	1.00	0.00	0.00
chr1!	61868766	257	G	0	1.00	0.00	0.00	0.99	0.00	0.00	0.99	0.00	0.00	0.96	0.04	0.00	0.99	0.00	0.00	0.98	0.01	0.00
chr1!	61868826	285	C	0	1.00	0.00	0.00	1.00	0.00	0.00	0.99	0.01	0.00	0.99	0.00	0.00	0.99	0.00	0.00	0.99	0.00	0.01
chr1(	92644565	642	C	0	1.00	0.00	0.00	0.99	0.01	0.01	0.99	0.00	0.00	0.99	0.00	0.00	0.99	0.00	0.01	1.00	0.00	0.00
chr8	89376856	1598	c	0	0.99	0.01	0.00	0.99	0.01	0.00	0.99	0.00	0.00	0.99	0.00	0.01	1.00	0.00	0.00	0.99	0.00	0.00
chrX	7476470	2053	C	1	1.00	0.00	0.00	1.00	0.00	0.00	0.99	0.00	0.00	0.99	0.01	0.00	0.97	0.01	0.02	0.99	0.01	0.00
chr4	136547412	1053	C	0	1.00	0.00	0.00	0.99	0.00	0.00	0.99	0.00	0.01	0.99	0.01	0.00	0.99	0.00	0.00	0.99	0.00	0.01
chr8	89376847	1592	c	0	0.99	0.01	0.00	1.00	0.00	0.00	0.99	0.00	0.00	0.99	0.01	0.00	0.99	0.00	0.01	0.99	0.01	0.00
chrX	7476542	2105	C	1	1.00	0.00	0.00	0.99	0.00	0.00	1.00	0.00	0.00	0.99	0.01	0.00	0.97	0.01	0.02	0.99	0.01	0.00
chrX	7539368	2254	G	0	1.00	0.00	0.00	1.00	0.00	0.00	0.99	0.00	0.01	0.97	0.03	0.00	0.99	0.00	0.00	0.99	0.00	0.01
chr1!	61868897	317	G	0	0.99	0.00	0.01	0.99	0.00	0.00	1.00	0.00	0.00	1.00	0.00	0.00	0.99	0.01	0.00	0.99	0.01	0.01
chr5	64987583	1526	C	0	1.00	0.00	0.00	1.00	0.00	0.00	0.99	0.00	0.00	1.00	0.00	0.00	0.99	0.00	0.00	0.99	0.01	0.00
chr8	89376999	1663	G	0	0.99	0.01	0.00	0.99	0.01	0.00	0.99	0.01	0.01	0.99	0.00	0.01	0.99	0.00	0.01	0.99	0.01	0.00
chr1!	100300347	485	C	0	0.99	0.00	0.01	0.99	0.00	0.00	0.99	0.00	0.01	0.99	0.00	0.00	0.99	0.00	0.00	0.99	0.00	0.00
chr1;	45706587	780	G	0	0.99	0.00	0.00	1.00	0.00	0.00	0.99	0.01	0.00	1.00	0.00	0.00	0.99	0.00	0.00	0.99	0.00	0.01
chrX	7476656	2184	G	0	0.99	0.00	0.01	1.00	0.00	0.00	1.00	0.00	0.00	1.00	0.00	0.00	1.00	0.00	0.00	1.00	0.00	0.00
chr1!	61868738	244	G	0	1.00	0.00	0.00	0.99	0.00	0.01	0.99	0.00	0.00	0.99	0.00	0.01	0.98	0.02	0.00	0.99	0.00	0.00
chr1!	100300374	490	C	0	0.99	0.00	0.00	0.99	0.00	0.00	0.95	0.04	0.01	0.99	0.00	0.00	0.99	0.00	0.00	0.99	0.01	0.00
chr1;	45706593	783	C	0	0.99	0.00	0.00	0.99	0.00	0.00	0.99	0.00	0.01	0.99	0.00	0.00	1.00	0.00	0.00	0.98	0.02	0.00
chr1;	45706680	825	G	0	0.99	0.01	0.00	1.00	0.00	0.00	0.99	0.00	0.00	1.00	0.00	0.00	0.99	0.00	0.01	0.99	0.00	0.01
chr1(	24277708	1000	G	0	1.00	0.00	0.00	0.99	0.00	0.00	0.99	0.00	0.01	1.00	0.00	0.00	1.00	0.00	0.00	1.00	0.00	0.00
chr1'	3525619	15	G	0	0.99	0.01	0.00	0.99	0.01	0.00	1.00	0.00	0.00	0.99	0.00	0.00	0.99	0.00	0.00	1.00	0.00	0.00
chr1(	24277566	946	c	0	1.00	0.00	0.00	0.99	0.00	0.00	0.99	0.00	0.00	1.00	0.00	0.00	0.98	0.02	0.00	1.00	0.00	0.00
chr5	64987319	1407	G	0	1.00	0.00	0.00	0.99	0.00	0.00	1.00	0.00	0.00	0.99	0.00	0.00	0.99	0.00	0.00	0.99	0.00	0.01
chr9	100498439	1887	G	0	0.99	0.01	0.00	0.99	0.00	0.00	0.99	0.00	0.00	0.99	0.01	0.00	0.99	0.00	0.01	0.99	0.00	0.00
chrX	7539532	2351	C	0	0.99	0.00	0.01	0.99	0.00	0.00	0.99	0.01	0.00	0.99	0.00	0.01	0.99	0.00	0.01	1.00	0.00	0.00
chr1'	3525729	71	C	0	0.99	0.01	0.00	0.99	0.00	0.01	0.99	0.01	0.00	0.98	0.00	0.01	0.99	0.01	0.00	0.99	0.00	0.01
chr1!	61868842	293	C	0	0.99	0.00	0.01	1.00	0.00	0.00	0.99	0.00	0.00	0.99	0.00	0.01	0.99	0.00	0.01	0.99	0.00	0.01
chr1(	92644463	573	G	0	1.00	0.00	0.00	0.99	0.00	0.00	0.99	0.01	0.00	0.99	0.00	0.01	0.99	0.00	0.00	0.99	0.00	0.00
chr4	136547720	1196	C	0	0.99	0.00	0.01	0.99	0.00	0.00	0.99	0.00	0.00	1.00	0.00	0.00	1.00	0.00	0.00	0.99	0.00	0.00
chr1'	3525615	13	G	0	1.00	0.00	0.00	1.00	0.00	0.00	0.98	0.02	0.00	0.99	0.00	0.00	1.00	0.00	0.00	1.00	0.00	0.00
chr1(	24277472	895	c	0	0.97	0.03	0.00	0.99	0.00	0.00	0.99	0.00	0.00	1.00	0.00	0.00	0.99	0.01	0.00	1.00	0.00	0.00
chr4	136547507	1110	C	0	1.00	0.00	0.00	1.00	0.00	0.00	0.99	0.00	0.00	0.98	0.02	0.00	1.00	0.00	0.00	0.99	0.00	0.01
chr4	136547510	1112	C	0	0.99	0.00	0.01	0.99	0.00	0.01	0.99	0.00	0.00	1.00	0.00	0.00	0.97	0.03	0.00	1.00	0.00	0.00
chrX	7476566	2123	C	1	1.00	0.00	0.00	1.00	0.00	0.00	0.99	0.01	0.00	1.00	0.00	0.00	0.97	0.01	0.02	0.99	0.01	0.00
chr1;	45706542	751	C	0	1.00	0.00	0.00	0.99	0.00	0.00	0.99	0.00	0.00	0.99	0.00	0.00	0.99	0.00	0.00	0.99	0.00	0.01
chr5	64987494	1489	G	0	1.00	0.00	0.00	0.99	0.00	0.00	0.99	0.00	0.00	0.97	0.03	0.00	0.98	0.02	0.00	1.00	0.00	0.00
chr8	120115705	1766	C	0	1.00	0.00	0.00	0.99	0.00	0.00	0.99	0.00	0.00	0.99	0.00	0.00	0.99	0.00	0.01	0.99	0.00	0.01
chrX	7476533	2097	C	0	1.00	0.00	0.00	1.00	0.00	0.00	1.00	0.00	0.00	1.00	0.00	0.00	0.99	0.00	0.01	1.00	0.00	0.00
chr1'	3525759	87	G	0	0.99	0.00	0.00	0.99	0.00	0.00	0.99	0.00	0.00	0.99	0.00	0.01	0.99	0.00	0.00	1.00	0.00	0.00
chr1;	45706575	772	C	0	1.00	0.00	0.00	0.99	0.00	0.00	0.99	0.00	0.00	0.99	0.01	0.01	0.99	0.00	0.01	0.98	0.01	0.00

0.99	0.01	0.01	0.99	0.01	0.01	0,00027
0.99	0.01	0.01	0.99	0.01	0.01	0,00027
0.99	0.01	0.01	0.99	0.01	0.01	0,00027
0.99	0.01	0.01	0.99	0.01	0.00	0,00027
0.99	0.01	0.01	0.99	0.01	0.01	0,00027
0.99	0.01	0.00	0.99	0.01	0.01	0,00027
0.99	0.00	0.01	0.99	0.01	0.01	0,00026
0.99	0.01	0.01	0.99	0.00	0.01	0,00026
0.99	0.01	0.01	0.99	0.00	0.01	0,00026
0.99	0.01	0.00	0.99	0.00	0.01	0,00026
0.99	0.01	0.00	0.98	0.01	0.00	0,00026
0.99	0.00	0.01	0.99	0.01	0.01	0,00026
0.99	0.01	0.00	0.99	0.01	0.01	0,00026
0.99	0.00	0.00	0.98	0.01	0.00	0,00026
0.99	0.00	0.01	0.99	0.01	0.01	0,00026
0.99	0.01	0.01	0.99	0.01	0.01	0,00026
0.99	0.01	0.01	0.99	0.01	0.01	0,00026
0.99	0.01	0.01	0.99	0.01	0.01	0,00026
0.99	0.01	0.01	0.99	0.01	0.01	0,00026
0.99	0.01	0.00	0.99	0.01	0.01	0,00026
0.99	0.01	0.01	0.99	0.00	0.01	0,00026
0.99	0.01	0.01	0.99	0.01	0.01	0,00026
0.99	0.01	0.01	0.99	0.01	0.01	0,00026
0.99	0.00	0.01	0.99	0.01	0.01	0,00026
0.99	0.01	0.00	0.99	0.00	0.01	0,00026
0.99	0.01	0.01	0.99	0.01	0.00	0,00026
0.99	0.01	0.01	0.99	0.01	0.01	0,00026
0.99	0.01	0.01	0.99	0.01	0.01	0,00026
0.99	0.01	0.01	0.99	0.01	0.01	0,00026
0.99	0.01	0.01	0.99	0.01	0.00	0,00026
0.99	0.01	0.01	0.99	0.00	0.01	0,00026
0.99	0.01	0.00	0.99	0.01	0.01	0,00026
0.99	0.01	0.01	0.99	0.00	0.01	0,00026
0.99	0.01	0.01	0.99	0.01	0.01	0,00026
0.99	0.01	0.01	0.99	0.01	0.01	0,00026
0.99	0.01	0.00	0.99	0.01	0.00	0,00026
0.99	0.01	0.01	0.99	0.01	0.01	0,00026
0.99	0.01	0.01	0.99	0.01	0.01	0,00026
0.99	0.01	0.01	0.99	0.01	0.00	0,00026
0.99	0.01	0.00	0.99	0.01	0.01	0,00026
0.99	0.00	0.01	0.99	0.01	0.01	0,00026
0.99	0.01	0.01	0.99	0.01	0.01	0,00026
0.99	0.01	0.01	0.99	0.01	0.01	0,00026
0.99	0.01	0.00	0.98	0.01	0.01	0,00026
0.99	0.01	0.01	0.99	0.01	0.01	0,00026
0.99	0.01	0.01	0.99	0.01	0.01	0,00026

chr17	45706644	810	C	0	0.99	0.00	0.00	0.99	0.00	0.00	0.99	0.01	0.00	0.98	0.01	0.00	1.00	0.00	0.00	0.99	0.00	0.01
chr4	139783830	1356	C	0	0.99	0.00	0.01	1.00	0.00	0.00	0.98	0.01	0.00	0.99	0.00	0.01	0.98	0.02	0.00	0.99	0.01	0.00
chr5	64987563	1520	C	0	1.00	0.00	0.00	0.99	0.01	0.01	0.99	0.00	0.00	0.99	0.00	0.01	0.99	0.00	0.01	0.99	0.01	0.00
chr8	120115770	1795	C	0	0.99	0.00	0.00	1.00	0.00	0.00	0.99	0.01	0.00	0.99	0.00	0.00	1.00	0.00	0.00	0.99	0.00	0.00
chr9	100498340	1843	G	0	1.00	0.00	0.00	1.00	0.00	0.00	0.99	0.00	0.01	0.99	0.01	0.00	1.00	0.00	0.00	0.99	0.00	0.01
chr15	100300038	371	C	0	0.99	0.00	0.01	0.99	0.00	0.00	0.99	0.00	0.00	0.99	0.00	0.00	1.00	0.00	0.00	0.99	0.01	0.00
chrX	7476585	2135	C	1	1.00	0.00	0.00	1.00	0.00	0.00	1.00	0.00	0.00	1.00	0.00	0.00	0.96	0.01	0.03	0.99	0.01	0.00
chrX	7539371	2256	G	0	0.99	0.01	0.00	0.99	0.01	0.00	0.99	0.01	0.00	0.99	0.00	0.01	1.00	0.00	0.00	1.00	0.00	0.00
chr17	45706557	760	C	0	0.99	0.00	0.01	0.99	0.00	0.00	0.99	0.00	0.00	0.99	0.01	0.01	0.99	0.00	0.00	1.00	0.00	0.00
chr4	139783749	1312	C	0	0.99	0.01	0.01	0.99	0.01	0.01	0.99	0.00	0.00	0.99	0.00	0.00	0.99	0.00	0.01	0.99	0.00	0.00
chr8	120115683	1755	G	0	1.00	0.00	0.00	0.99	0.00	0.00	0.99	0.00	0.00	0.99	0.00	0.01	0.99	0.00	0.01	1.00	0.00	0.00
chr9	100498334	1841	G	0	1.00	0.00	0.00	1.00	0.00	0.00	0.99	0.01	0.00	1.00	0.00	0.00	0.99	0.00	0.00	0.99	0.00	0.01
chr11	3525786	103	G	0	0.99	0.00	0.00	1.00	0.00	0.00	1.00	0.00	0.00	0.99	0.00	0.00	0.99	0.00	0.00	0.99	0.01	0.00
chr11	3525793	109	G	0	0.99	0.00	0.00	0.99	0.00	0.00	0.99	0.00	0.00	0.99	0.00	0.01	0.99	0.00	0.00	0.99	0.00	0.00
chr17	45706496	728	G	0	1.00	0.00	0.00	0.99	0.00	0.00	0.99	0.01	0.00	0.98	0.02	0.00	1.00	0.00	0.00	1.00	0.00	0.00
chr5	64987565	1521	G	0	0.99	0.01	0.00	0.99	0.00	0.00	0.99	0.00	0.01	1.00	0.00	0.00	0.99	0.00	0.01	0.99	0.00	0.01
chr9	100498333	1840	G	0	1.00	0.00	0.00	0.99	0.00	0.00	0.99	0.00	0.00	1.00	0.00	0.00	0.99	0.00	0.00	0.98	0.00	0.01
chr11	3525711	59	C	0	0.98	0.02	0.00	0.99	0.01	0.00	0.99	0.00	0.00	0.99	0.01	0.00	0.98	0.02	0.00	0.99	0.00	0.01
chr16	92644544	629	G	0	0.99	0.00	0.00	1.00	0.00	0.00	1.00	0.00	0.00	0.99	0.00	0.00	0.99	0.00	0.00	1.00	0.00	0.00
chr5	64987384	1436	C	0	0.99	0.01	0.00	0.99	0.01	0.01	0.99	0.00	0.00	0.99	0.00	0.01	1.00	0.00	0.00	1.00	0.00	0.00
chr5	64987561	1519	C	0	0.99	0.01	0.00	0.99	0.00	0.00	0.99	0.00	0.00	0.99	0.01	0.00	0.99	0.00	0.01	0.99	0.00	0.00
chrX	7539518	2343	C	0	1.00	0.00	0.00	0.99	0.00	0.00	0.99	0.00	0.00	0.99	0.01	0.00	0.99	0.00	0.00	0.99	0.00	0.00
chr11	3525744	80	C	0	1.00	0.00	0.00	0.99	0.01	0.00	1.00	0.00	0.00	0.99	0.00	0.01	1.00	0.00	0.00	0.99	0.00	0.00
chr11	3525776	96	G	0	1.00	0.00	0.00	1.00	0.00	0.00	1.00	0.00	0.00	0.98	0.02	0.00	0.99	0.00	0.01	0.99	0.00	0.01
chr17	45706532	746	G	0	1.00	0.00	0.00	0.99	0.00	0.00	0.99	0.00	0.01	0.99	0.00	0.00	1.00	0.00	0.00	0.99	0.00	0.00
chr19	24277619	970	C	0	0.99	0.01	0.00	1.00	0.00	0.00	0.99	0.00	0.01	0.99	0.01	0.00	0.99	0.00	0.01	1.00	0.00	0.00
chr4	136547467	1087	C	0	1.00	0.00	0.00	0.99	0.00	0.00	0.99	0.01	0.00	0.99	0.00	0.01	1.00	0.00	0.00	0.99	0.00	0.01
chr15	100300379	492	C	0	0.99	0.00	0.00	0.99	0.00	0.01	0.99	0.00	0.01	0.99	0.00	0.00	1.00	0.00	0.00	0.99	0.00	0.00
chr8	120115816	1818	C	0	1.00	0.00	0.00	0.99	0.01	0.00	0.99	0.00	0.01	0.99	0.00	0.00	1.00	0.00	0.00	0.99	0.00	0.00
chrX	7476662	2190	G	1	0.99	0.00	0.01	0.97	0.02	0.00	1.00	0.00	0.00	0.99	0.00	0.01	1.00	0.00	0.00	0.99	0.00	0.01
chrX	7539423	2286	C	0	1.00	0.00	0.00	0.99	0.00	0.01	0.98	0.01	0.01	0.98	0.02	0.00	0.99	0.00	0.01	0.98	0.02	0.00
chr11	3525897	172	C	0	0.99	0.00	0.01	0.99	0.01	0.00	0.99	0.00	0.01	0.99	0.00	0.01	0.99	0.01	0.00	0.99	0.01	0.00
chr15	61868750	248	C	0	0.99	0.00	0.00	1.00	0.00	0.00	0.99	0.00	0.01	0.99	0.00	0.00	0.99	0.00	0.00	0.99	0.00	0.00
chr15	100299991	348	C	0	0.99	0.00	0.00	0.99	0.00	0.01	0.99	0.00	0.01	0.99	0.00	0.00	1.00	0.00	0.00	1.00	0.00	0.00
chr15	100300218	444	G	0	1.00	0.00	0.00	1.00	0.00	0.00	0.99	0.00	0.00	0.99	0.01	0.00	0.98	0.02	0.00	1.00	0.00	0.00
chr16	92644346	523	G	0	0.99	0.00	0.00	0.99	0.01	0.01	0.99	0.00	0.01	0.99	0.00	0.01	0.99	0.01	0.00	0.99	0.00	0.00
chr17	45706536	748	C	0	1.00	0.00	0.00	1.00	0.00	0.00	1.00	0.00	0.00	0.99	0.00	0.00	1.00	0.00	0.00	1.00	0.00	0.00
chr5	64987615	1536	C	0	1.00	0.00	0.00	0.99	0.00	0.00	0.99	0.00	0.00	0.99	0.00	0.00	1.00	0.00	0.00	1.00	0.00	0.00
chr9	100498497	1921	C	0	0.99	0.01	0.00	0.99	0.00	0.01	0.99	0.01	0.00	0.99	0.00	0.00	0.99	0.00	0.00	0.99	0.00	0.00
chr11	3525758	86	G	0	1.00	0.00	0.00	0.99	0.00	0.00	0.99	0.01	0.01	0.99	0.00	0.01	0.99	0.00	0.00	0.99	0.00	0.00
chr16	92644663	656	C	0	0.99	0.00	0.00	0.98	0.01	0.01	0.99	0.00	0.01	0.99	0.00	0.01	0.99	0.00	0.01	0.99	0.00	0.01
chr17	45706581	776	C	0	0.99	0.00	0.00	0.99	0.01	0.01	1.00	0.00	0.00	0.99	0.00	0.00	1.00	0.00	0.00	0.99	0.00	0.00
chr19	24277654	982	C	0	0.99	0.00	0.00	1.00	0.00	0.00	0.99	0.01	0.00	1.00	0.00	0.00	0.99	0.00	0.00	1.00	0.00	0.00
chr8	89376840	1588	c	0	0.99	0.00	0.00	1.00	0.00	0.00	0.99	0.00	0.00	1.00	0.00	0.00	0.99	0.00	0.00	1.00	0.00	0.00
chr19	24277455	890	c	0	0.99	0.01	0.00	0.99	0.00	0.00	1.00	0.00	0.00	1.00	0.00	0.00	1.00	0.00	0.00	1.00	0.00	0.00
chr19	24277698	995	G	0	0.99	0.01	0.00	1.00	0.00	0.00	0.99	0.00	0.00	0.99	0.00	0.00	1.00	0.00	0.00	1.00	0.00	0.00

0.99	0.01	0.01	0.99	0.01	0.00	0,00026
0.99	0.01	0.01	0.99	0.01	0.01	0,00026
0.99	0.00	0.01	0.99	0.01	0.01	0,00026
0.99	0.01	0.01	0.99	0.01	0.01	0,00026
0.99	0.01	0.01	0.99	0.01	0.01	0,00026
0.99	0.01	0.01	0.99	0.01	0.00	0,00025
0.99	0.01	0.00	0.99	0.01	0.01	0,00025
0.99	0.01	0.00	0.99	0.01	0.01	0,00025
0.99	0.01	0.01	0.99	0.01	0.01	0,00025
0.99	0.01	0.01	0.99	0.01	0.01	0,00025
0.99	0.01	0.01	0.99	0.00	0.01	0,00025
0.99	0.01	0.00	0.99	0.01	0.01	0,00025
0.99	0.01	0.01	0.99	0.01	0.01	0,00025
0.99	0.01	0.01	0.99	0.01	0.01	0,00025
0.99	0.01	0.01	0.99	0.01	0.01	0,00025
0.99	0.01	0.01	0.99	0.01	0.01	0,00025
0.99	0.01	0.01	0.99	0.01	0.01	0,00025
0.99	0.01	0.00	0.99	0.01	0.00	0,00025
0.99	0.01	0.01	0.99	0.01	0.01	0,00025
0.99	0.01	0.01	0.99	0.01	0.00	0,00025
0.99	0.01	0.01	0.99	0.01	0.01	0,00025
0.99	0.01	0.01	0.99	0.01	0.01	0,00025
0.99	0.01	0.00	0.99	0.00	0.01	0,00025
0.99	0.00	0.00	0.99	0.01	0.01	0,00025
0.99	0.01	0.01	0.99	0.01	0.01	0,00025
0.99	0.01	0.01	0.99	0.01	0.01	0,00025
0.99	0.01	0.01	0.99	0.00	0.01	0,00025
0.99	0.01	0.01	0.99	0.01	0.01	0,00025
0.99	0.01	0.01	0.99	0.01	0.01	0,00025
0.99	0.01	0.01	0.99	0.00	0.01	0,00025
0.99	0.01	0.00	0.99	0.00	0.01	0,00025
0.99	0.00	0.01	0.99	0.01	0.01	0,00025
0.99	0.01	0.01	0.99	0.01	0.00	0,00025
0.99	0.01	0.01	0.99	0.01	0.00	0,00025
0.99	0.01	0.01	0.99	0.01	0.00	0,00025
0.99	0.01	0.01	0.99	0.00	0.01	0,00025
0.99	0.00	0.00	0.98	0.01	0.01	0,00025
0.99	0.01	0.01	0.99	0.00	0.01	0,00025
0.99	0.01	0.00	0.99	0.01	0.01	0,00025
0.99	0.01	0.01	0.99	0.01	0.01	0,00025
0.99	0.01	0.01	0.99	0.00	0.01	0,00025
0.99	0.01	0.01	0.99	0.01	0.01	0,00025
0.99	0.01	0.01	0.99	0.00	0.01	0,00025
0.99	0.01	0.01	0.99	0.01	0.01	0,00025
0.99	0.01	0.00	0.98	0.01	0.01	0,00025
0.99	0.01	0.00	0.99	0.01	0.01	0,00025

chr19	24277582	951	g	0	0.99	0.00	0.01	0.99	0.00	0.01	1.00	0.00	0.00	0.99	0.00	0.01	1.00	0.00	0.00	0.99	0.00	0.01
chrX	7539457	2309	C	0	1.00	0.00	0.00	0.99	0.01	0.00	1.00	0.00	0.00	1.00	0.00	0.00	0.97	0.03	0.00	0.99	0.01	0.00
chr16	92644372	531	G	0	0.99	0.01	0.00	0.98	0.01	0.01	1.00	0.00	0.00	0.99	0.00	0.00	0.99	0.00	0.00	1.00	0.00	0.00
chr16	92644416	546	G	0	0.99	0.00	0.00	0.99	0.00	0.00	1.00	0.00	0.00	0.99	0.01	0.01	0.99	0.00	0.00	1.00	0.00	0.00
chr8	120115697	1762	C	0	0.99	0.00	0.00	1.00	0.00	0.00	0.99	0.00	0.00	0.99	0.00	0.00	1.00	0.00	0.00	0.99	0.00	0.01
chr17	45706597	785	C	0	1.00	0.00	0.00	0.99	0.00	0.00	1.00	0.00	0.00	1.00	0.00	0.00	0.99	0.00	0.00	0.99	0.00	0.00
chr19	24277598	960	c	0	0.99	0.01	0.01	1.00	0.00	0.00	0.99	0.01	0.00	1.00	0.00	0.00	0.99	0.00	0.01	0.99	0.00	0.00
chr4	136547641	1179	C	0	1.00	0.00	0.00	0.99	0.00	0.01	1.00	0.00	0.00	1.00	0.00	0.00	1.00	0.00	0.00	1.00	0.00	0.00
chr5	64987363	1428	C	0	1.00	0.00	0.00	0.98	0.01	0.01	1.00	0.00	0.00	1.00	0.00	0.00	1.00	0.00	0.00	0.99	0.01	0.00
chr8	89376865	1604	c	0	0.99	0.00	0.00	0.99	0.01	0.00	1.00	0.00	0.00	1.00	0.00	0.00	0.99	0.00	0.01	0.99	0.00	0.01
chrX	7539553	2366	C	0	0.99	0.01	0.00	1.00	0.00	0.00	0.99	0.00	0.00	0.99	0.01	0.00	1.00	0.00	0.00	0.98	0.02	0.00
chr15	100300028	367	C	0	0.99	0.01	0.01	0.99	0.01	0.00	0.99	0.00	0.01	0.99	0.00	0.00	1.00	0.00	0.00	1.00	0.00	0.00
chr15	100300105	405	C	0	0.99	0.00	0.01	0.99	0.00	0.00	0.99	0.00	0.00	0.99	0.00	0.00	1.00	0.00	0.00	1.00	0.00	0.00
chr16	92644374	533	G	0	0.99	0.00	0.00	0.99	0.00	0.00	0.99	0.01	0.00	1.00	0.00	0.00	0.99	0.00	0.00	1.00	0.00	0.00
chr16	92644454	567	C	0	0.99	0.00	0.00	0.99	0.00	0.00	0.99	0.00	0.00	0.99	0.00	0.00	0.99	0.00	0.00	1.00	0.00	0.00
chr19	24277548	936	g	0	1.00	0.00	0.00	0.99	0.00	0.00	0.99	0.00	0.00	1.00	0.00	0.00	1.00	0.00	0.00	1.00	0.00	0.00
chr19	24277675	986	G	0	1.00	0.00	0.00	0.99	0.00	0.00	0.99	0.00	0.01	0.99	0.00	0.00	1.00	0.00	0.00	0.99	0.00	0.00
chr4	139783748	1311	C	0	0.99	0.00	0.00	0.99	0.00	0.00	0.99	0.00	0.01	0.99	0.00	0.00	0.99	0.01	0.01	1.00	0.00	0.00
chr5	64987618	1538	C	0	1.00	0.00	0.00	0.99	0.00	0.00	0.99	0.01	0.00	0.99	0.00	0.00	1.00	0.00	0.00	1.00	0.00	0.00
chrX	7539522	2345	C	0	0.99	0.00	0.00	0.99	0.00	0.00	1.00	0.00	0.00	0.99	0.00	0.01	0.99	0.00	0.01	1.00	0.00	0.00
chr19	24277696	994	G	0	0.99	0.00	0.00	1.00	0.00	0.00	0.99	0.00	0.01	0.99	0.00	0.00	1.00	0.00	0.00	1.00	0.00	0.00
chr4	139783774	1326	C	0	0.99	0.01	0.00	1.00	0.00	0.00	0.99	0.01	0.00	0.99	0.00	0.01	0.99	0.00	0.01	0.99	0.00	0.01
chrX	7539514	2341	C	0	0.99	0.00	0.00	1.00	0.00	0.00	0.99	0.00	0.00	0.99	0.00	0.00	0.99	0.00	0.01	1.00	0.00	0.00
chr16	92644554	634	C	0	0.99	0.00	0.00	0.99	0.01	0.01	0.99	0.00	0.00	0.99	0.00	0.00	0.99	0.00	0.01	1.00	0.00	0.00
chr4	136547475	1092	C	0	0.99	0.00	0.00	1.00	0.00	0.00	0.99	0.00	0.00	1.00	0.00	0.00	1.00	0.00	0.00	0.98	0.02	0.00
chr4	139783763	1319	G	0	1.00	0.00	0.00	1.00	0.00	0.00	1.00	0.00	0.00	0.99	0.01	0.00	0.99	0.00	0.01	0.99	0.01	0.00
chr16	92644377	534	G	0	0.99	0.00	0.00	0.99	0.01	0.01	1.00	0.00	0.00	0.99	0.00	0.00	0.99	0.00	0.00	1.00	0.00	0.00
chr4	136547390	1043	G	0	0.99	0.00	0.01	0.99	0.01	0.00	0.99	0.01	0.00	0.98	0.00	0.02	0.99	0.00	0.01	0.99	0.01	0.00
chr4	139783727	1299	G	0	0.99	0.00	0.00	0.99	0.00	0.00	0.99	0.00	0.00	0.99	0.00	0.01	0.99	0.01	0.00	0.99	0.00	0.01
chrX	7539286	2220	G	0	1.00	0.00	0.00	0.99	0.01	0.00	1.00	0.00	0.00	1.00	0.00	0.00	1.00	0.00	0.00	0.99	0.00	0.01
chrX	7539451	2305	C	0	1.00	0.00	0.00	1.00	0.00	0.00	1.00	0.00	0.00	1.00	0.00	0.00	0.99	0.00	0.01	0.99	0.00	0.01
chr17	45706580	775	C	0	0.99	0.01	0.00	0.99	0.00	0.00	0.99	0.00	0.00	1.00	0.00	0.00	1.00	0.00	0.00	1.00	0.00	0.00
chr8	120115692	1760	C	0	0.99	0.00	0.01	0.99	0.00	0.00	0.99	0.01	0.00	0.99	0.00	0.01	1.00	0.00	0.00	0.99	0.00	0.01
chr8	120115839	1830	C	0	1.00	0.00	0.00	1.00	0.00	0.00	1.00	0.00	0.00	0.99	0.00	0.00	0.99	0.00	0.01	0.99	0.00	0.00
chr11	3525665	35	G	0	0.99	0.00	0.00	0.99	0.00	0.00	0.99	0.00	0.00	0.99	0.00	0.00	1.00	0.00	0.00	1.00	0.00	0.00
chr11	3525834	131	G	0	1.00	0.00	0.00	1.00	0.00	0.00	1.00	0.00	0.00	0.99	0.00	0.00	1.00	0.00	0.00	1.00	0.00	0.00
chr15	100300189	438	G	0	1.00	0.00	0.00	1.00	0.00	0.00	0.99	0.00	0.00	0.99	0.01	0.00	1.00	0.00	0.00	1.00	0.00	0.00
chr16	92644553	633	C	0	1.00	0.00	0.00	0.99	0.00	0.01	0.99	0.00	0.00	0.99	0.00	0.01	0.99	0.00	0.00	1.00	0.00	0.00
chr9	100498502	1924	C	0	0.99	0.00	0.00	1.00	0.00	0.00	1.00	0.00	0.00	1.00	0.00	0.00	1.00	0.00	0.00	1.00	0.00	0.00
chrX	7539614	2403	G	0	1.00	0.00	0.00	1.00	0.00	0.00	0.99	0.01	0.00	1.00	0.00	0.00	0.99	0.00	0.01	1.00	0.00	0.00
chr5	64987528	1502	G	0	1.00	0.00	0.00	1.00	0.00	0.00	1.00	0.00	0.00	0.99	0.00	0.01	1.00	0.00	0.00	1.00	0.00	0.00
chr19	24277606	963	G	0	1.00	0.00	0.00	1.00	0.00	0.00	1.00	0.00	0.00	0.99	0.00	0.01	0.99	0.00	0.00	1.00	0.00	0.00
chr8	89376932	1638	C	0	0.99	0.01	0.00	1.00	0.00	0.00	1.00	0.00	0.00	0.99	0.00	0.01	0.99	0.00	0.01	0.99	0.00	0.01
chr8	120115756	1788	C	0	1.00	0.00	0.00	0.99	0.00	0.00	0.99	0.01	0.00	0.99	0.00	0.01	0.99	0.00	0.00	1.00	0.00	0.00
chrX	7539639	2417	C	0	1.00	0.00	0.00	0.99	0.01	0.00	0.99	0.01	0.00	0.99	0.01	0.00	1.00	0.00	0.00	0.99	0.00	0.01
chr11	3525639	25	C	0	0.99	0.00	0.01	0.99	0.01	0.00	0.99	0.01	0.00	0.99	0.00	0.01	0.99	0.00	0.01	0.98	0.02	0.00

0.99	0.00	0.01	0.99	0.00	0.01	0,00024
0.99	0.01	0.01	0.99	0.01	0.00	0,00024
0.99	0.01	0.01	0.99	0.01	0.00	0,00024
0.99	0.01	0.01	0.99	0.01	0.01	0,00024
0.99	0.01	0.01	0.99	0.01	0.01	0,00024
0.99	0.01	0.01	0.99	0.01	0.01	0,00024
0.99	0.01	0.01	0.99	0.00	0.01	0,00024
0.99	0.01	0.01	0.99	0.00	0.01	0,00024
0.99	0.01	0.01	0.99	0.01	0.00	0,00024
0.99	0.01	0.01	0.99	0.00	0.01	0,00024
0.99	0.01	0.00	0.99	0.01	0.00	0,00024
0.99	0.01	0.01	0.99	0.01	0.00	0,00024
0.99	0.00	0.01	0.99	0.00	0.01	0,00024
0.99	0.01	0.01	0.99	0.01	0.01	0,00024
0.99	0.01	0.01	0.99	0.00	0.01	0,00024
0.99	0.01	0.01	0.99	0.00	0.01	0,00024
0.99	0.01	0.01	0.99	0.01	0.00	0,00024
0.99	0.01	0.01	0.99	0.01	0.01	0,00024
0.99	0.01	0.01	0.99	0.00	0.01	0,00024
0.99	0.01	0.01	0.99	0.01	0.01	0,00024
0.99	0.01	0.01	0.99	0.00	0.01	0,00024
0.99	0.01	0.00	0.99	0.00	0.01	0,00024
0.99	0.01	0.01	0.99	0.01	0.01	0,00024
0.99	0.01	0.01	0.99	0.00	0.01	0,00024
0.99	0.00	0.01	0.99	0.01	0.01	0,00024
0.99	0.00	0.01	0.99	0.01	0.00	0,00024
0.99	0.01	0.01	0.99	0.01	0.01	0,00024
0.99	0.01	0.01	0.99	0.00	0.01	0,00024
0.99	0.01	0.01	0.99	0.01	0.01	0,00024
0.99	0.01	0.01	0.99	0.00	0.01	0,00024
0.99	0.00	0.01	0.99	0.00	0.01	0,00024
0.99	0.01	0.01	0.99	0.00	0.01	0,00024
0.99	0.01	0.01	0.99	0.00	0.01	0,00024
0.99	0.01	0.01	0.99	0.01	0.01	0,00024
0.99	0.01	0.01	0.99	0.01	0.01	0,00024
0.99	0.01	0.01	0.99	0.01	0.00	0,00024
0.99	0.01	0.00	0.99	0.01	0.00	0,00024
0.99	0.01	0.01	0.99	0.01	0.01	0,00024
0.99	0.01	0.01	0.99	0.01	0.01	0,00024
0.99	0.01	0.00	0.99	0.01	0.01	0,00024
0.99	0.01	0.00	0.99	0.01	0.01	0,00024
0.99	0.01	0.01	0.99	0.01	0.01	0,00023
0.99	0.00	0.01	0.99	0.00	0.01	0,00023
0.99	0.01	0.01	0.99	0.01	0.01	0,00023
0.99	0.01	0.01	0.99	0.01	0.01	0,00023
0.99	0.01	0.01	0.99	0.00	0.01	0,00023

chr11	3525766	90	G	0	1.00	0.00	0.00	0.99	0.00	0.00	1.00	0.00	0.00	0.99	0.00	0.01	0.99	0.00	0.00	1.00	0.00	0.00
chr15	61868900	319	G	0	1.00	0.00	0.00	0.99	0.00	0.00	0.99	0.00	0.00	1.00	0.00	0.00	0.99	0.00	0.01	0.99	0.00	0.00
chr19	24277608	965	G	0	0.99	0.01	0.00	1.00	0.00	0.00	0.99	0.00	0.01	1.00	0.00	0.00	1.00	0.00	0.00	0.99	0.00	0.00
chr4	139783632	1246	G	0	1.00	0.00	0.00	1.00	0.00	0.00	1.00	0.00	0.00	0.99	0.00	0.00	1.00	0.00	0.00	0.99	0.00	0.01
chr4	139783636	1250	G	0	1.00	0.00	0.00	1.00	0.00	0.00	1.00	0.00	0.00	1.00	0.00	0.00	1.00	0.00	0.00	1.00	0.00	0.00
chr5	64987548	1511	C	0	1.00	0.00	0.00	0.99	0.00	0.00	0.99	0.00	0.00	1.00	0.00	0.00	0.99	0.00	0.00	1.00	0.00	0.00
chr8	120115710	1769	C	0	0.99	0.00	0.00	1.00	0.00	0.00	0.99	0.00	0.00	1.00	0.00	0.00	0.99	0.00	0.00	1.00	0.00	0.00
chr11	3525809	117	G	0	0.99	0.00	0.00	1.00	0.00	0.00	0.99	0.00	0.00	0.99	0.00	0.00	1.00	0.00	0.00	0.98	0.02	0.00
chr15	100299982	344	C	0	0.99	0.01	0.01	0.99	0.01	0.01	0.99	0.00	0.01	0.99	0.01	0.01	1.00	0.00	0.00	1.00	0.00	0.00
chr17	45706544	752	G	0	1.00	0.00	0.00	0.99	0.01	0.00	0.99	0.01	0.00	1.00	0.00	0.00	1.00	0.00	0.00	1.00	0.00	0.00
chr4	136547757	1212	C	0	1.00	0.00	0.00	0.98	0.01	0.01	1.00	0.00	0.00	0.99	0.00	0.01	1.00	0.00	0.00	0.99	0.00	0.01
chr5	64987596	1530	C	0	0.99	0.00	0.00	0.99	0.00	0.00	1.00	0.00	0.00	0.99	0.00	0.00	1.00	0.00	0.00	1.00	0.00	0.00
chr8	120115657	1742	C	0	0.99	0.01	0.00	0.99	0.01	0.00	0.99	0.01	0.00	1.00	0.00	0.00	1.00	0.00	0.00	1.00	0.00	0.00
chrX	7476556	2116	C	1	1.00	0.00	0.00	1.00	0.00	0.00	0.99	0.00	0.00	0.99	0.01	0.00	0.96	0.01	0.03	0.98	0.01	0.00
chr11	3525777	97	C	0	0.99	0.01	0.00	0.99	0.00	0.00	0.99	0.00	0.00	0.99	0.01	0.00	0.99	0.00	0.00	0.99	0.00	0.01
chr15	61868880	309	C	0	0.99	0.00	0.00	1.00	0.00	0.00	1.00	0.00	0.00	0.98	0.02	0.00	0.99	0.01	0.00	1.00	0.00	0.00
chr15	100300022	364	C	0	0.99	0.00	0.01	0.99	0.00	0.00	0.99	0.00	0.00	0.99	0.00	0.01	1.00	0.00	0.00	1.00	0.00	0.00
chr9	100498496	1920	G	0	0.99	0.00	0.01	0.99	0.00	0.01	0.99	0.01	0.00	0.99	0.01	0.00	0.99	0.00	0.01	0.99	0.00	0.01
chr15	61868692	220	C	0	1.00	0.00	0.00	1.00	0.00	0.00	1.00	0.00	0.00	0.99	0.00	0.01	0.98	0.02	0.00	0.99	0.00	0.00
chr15	100300138	419	C	0	0.99	0.00	0.00	0.98	0.01	0.01	0.99	0.00	0.01	0.99	0.01	0.01	1.00	0.00	0.00	0.99	0.00	0.00
chr16	92644322	515	G	0	1.00	0.00	0.00	0.99	0.00	0.00	1.00	0.00	0.00	0.99	0.00	0.00	1.00	0.00	0.00	1.00	0.00	0.00
chr4	139783753	1313	G	0	1.00	0.00	0.00	0.99	0.00	0.00	0.99	0.00	0.00	0.99	0.00	0.00	0.99	0.01	0.00	1.00	0.00	0.00
chr8	120115627	1728	C	0	1.00	0.00	0.00	1.00	0.00	0.00	1.00	0.00	0.00	1.00	0.00	0.00	0.99	0.00	0.01	0.98	0.01	0.00
chrX	7539439	2297	C	0	1.00	0.00	0.00	1.00	0.00	0.00	1.00	0.00	0.00	0.99	0.00	0.00	0.99	0.00	0.01	0.99	0.00	0.01
chr15	100300460	508	C	0	0.99	0.00	0.00	0.99	0.01	0.01	0.99	0.00	0.01	0.99	0.00	0.01	1.00	0.00	0.00	1.00	0.00	0.00
chr16	92644473	582	G	0	0.99	0.00	0.00	0.99	0.00	0.00	1.00	0.00	0.00	0.99	0.00	0.00	0.99	0.00	0.00	1.00	0.00	0.00
chr4	139783822	1351	C	0	1.00	0.00	0.00	1.00	0.00	0.00	1.00	0.00	0.00	0.99	0.00	0.01	0.99	0.00	0.01	0.98	0.02	0.00
chr5	64987424	1451	G	0	1.00	0.00	0.00	0.99	0.00	0.00	0.99	0.00	0.01	0.99	0.00	0.01	0.99	0.00	0.00	1.00	0.00	0.00
chr8	120115695	1761	G	0	1.00	0.00	0.00	1.00	0.00	0.00	1.00	0.00	0.00	0.99	0.01	0.00	0.99	0.00	0.00	1.00	0.00	0.00
chrX	7539531	2350	C	0	1.00	0.00	0.00	1.00	0.00	0.00	1.00	0.00	0.00	0.99	0.00	0.01	0.99	0.00	0.01	1.00	0.00	0.00
chr15	100300161	431	G	0	0.99	0.00	0.00	0.99	0.00	0.00	0.99	0.00	0.01	0.99	0.00	0.00	0.99	0.00	0.00	1.00	0.00	0.00
chr16	92644513	612	G	0	0.99	0.00	0.00	0.99	0.00	0.00	1.00	0.00	0.00	0.99	0.00	0.01	1.00	0.00	0.00	1.00	0.00	0.00
chr19	24277420	885	c	0	1.00	0.00	0.00	0.99	0.00	0.00	0.99	0.00	0.00	0.99	0.00	0.00	1.00	0.00	0.00	0.99	0.01	0.00
chr11	3525719	65	G	0	1.00	0.00	0.00	0.99	0.00	0.00	0.99	0.00	0.00	1.00	0.00	0.00	1.00	0.00	0.00	0.99	0.00	0.00
chr15	100300077	392	C	0	1.00	0.00	0.00	0.99	0.00	0.00	0.99	0.00	0.00	0.99	0.00	0.00	1.00	0.00	0.00	1.00	0.00	0.00
chr16	92644389	540	G	0	1.00	0.00	0.00	0.99	0.00	0.01	1.00	0.00	0.00	0.99	0.00	0.00	1.00	0.00	0.00	1.00	0.00	0.00
chr16	92644471	580	G	0	0.99	0.00	0.00	0.99	0.01	0.01	1.00	0.00	0.00	0.99	0.00	0.01	1.00	0.00	0.00	1.00	0.00	0.00
chr17	45706566	766	G	0	0.99	0.00	0.01	0.99	0.00	0.00	0.99	0.00	0.00	0.99	0.00	0.01	0.99	0.00	0.01	1.00	0.00	0.00
chr17	45706617	795	C	0	1.00	0.00	0.00	1.00	0.00	0.00	0.99	0.01	0.00	0.99	0.00	0.01	1.00	0.00	0.00	1.00	0.00	0.00
chr17	45706727	850	G	0	1.00	0.00	0.00	1.00	0.00	0.00	0.99	0.00	0.00	1.00	0.00	0.00	1.00	0.00	0.00	1.00	0.00	0.00
chr5	64987340	1417	G	0	1.00	0.00	0.00	0.99	0.00	0.00	0.99	0.00	0.00	0.99	0.00	0.01	1.00	0.00	0.00	1.00	0.00	0.00
chrX	7476530	2094	C	1	1.00	0.00	0.00	1.00	0.00	0.00	1.00	0.00	0.00	0.99	0.00	0.01	0.99	0.00	0.01	0.99	0.00	0.01
chrX	7476602	2146	C	1	1.00	0.00	0.00	1.00	0.00	0.00	1.00	0.00	0.00	0.99	0.00	0.00	0.98	0.00	0.02	0.99	0.01	0.00
chrX	7539630	2413	C	0	0.99	0.00	0.01	1.00	0.00	0.00	0.99	0.00	0.01	0.98	0.02	0.00	1.00	0.00	0.00	0.99	0.00	0.01
chr15	100300406	497	C	0	0.99	0.00	0.00	0.99	0.00	0.00	0.99	0.00	0.00	0.99	0.00	0.01	1.00	0.00	0.00	1.00	0.00	0.00
chr16	92644417	547	G	0	0.99	0.00	0.00	0.99	0.00	0.00	1.00	0.00	0.00	0.99	0.00	0.01	1.00	0.00	0.00	1.00	0.00	0.00

0.99	0.01	0.01	0.99	0.00	0.01	0,00023
0.99	0.01	0.01	0.99	0.01	0.01	0,00023
0.99	0.01	0.01	0.99	0.00	0.01	0,00023
0.99	0.00	0.01	0.99	0.00	0.01	0,00023
0.99	0.01	0.00	0.97	0.02	0.01	0,00023
0.99	0.01	0.01	0.99	0.01	0.00	0,00023
0.99	0.00	0.01	0.99	0.01	0.01	0,00023
0.99	0.01	0.01	0.99	0.01	0.00	0,00023
0.99	0.01	0.01	0.99	0.00	0.01	0,00023
0.99	0.01	0.01	0.99	0.01	0.00	0,00023
0.99	0.01	0.01	0.99	0.00	0.01	0,00023
0.99	0.00	0.01	0.99	0.00	0.01	0,00023
0.99	0.01	0.01	0.99	0.00	0.00	0,00023
0.99	0.01	0.00	0.98	0.01	0.00	0,00023
0.99	0.01	0.00	0.99	0.01	0.01	0,00023
0.99	0.01	0.01	0.99	0.01	0.00	0,00023
0.99	0.01	0.01	0.99	0.01	0.01	0,00023
0.99	0.00	0.01	0.99	0.00	0.01	0,00023
0.99	0.00	0.01	0.99	0.01	0.01	0,00023
0.99	0.01	0.01	0.99	0.01	0.01	0,00023
0.99	0.00	0.01	0.99	0.01	0.01	0,00023
0.99	0.01	0.01	0.99	0.01	0.01	0,00023
0.99	0.00	0.01	0.99	0.01	0.01	0,00023
0.99	0.00	0.01	0.99	0.00	0.01	0,00023
0.99	0.01	0.01	0.99	0.00	0.01	0,00023
0.99	0.01	0.01	0.99	0.00	0.01	0,00023
0.99	0.00	0.00	0.99	0.01	0.01	0,00023
0.99	0.01	0.01	0.99	0.00	0.01	0,00023
0.99	0.01	0.00	0.99	0.01	0.01	0,00023
0.99	0.01	0.01	0.99	0.01	0.01	0,00023
0.99	0.01	0.01	0.99	0.01	0.01	0,00023
0.99	0.00	0.01	0.99	0.01	0.01	0,00023
0.99	0.01	0.00	0.99	0.01	0.01	0,00023
0.99	0.01	0.01	0.99	0.00	0.01	0,00023
0.99	0.01	0.01	0.99	0.00	0.01	0,00023
0.99	0.01	0.01	0.99	0.00	0.01	0,00023
0.99	0.01	0.01	0.99	0.01	0.00	0,00023
0.99	0.00	0.01	0.99	0.00	0.01	0,00023
0.99	0.01	0.01	0.99	0.00	0.01	0,00023
0.99	0.01	0.01	0.99	0.00	0.00	0,00023
0.99	0.01	0.01	0.99	0.01	0.01	0,00023
0.99	0.00	0.00	0.99	0.00	0.01	0,00023
0.99	0.01	0.00	0.99	0.01	0.01	0,00023
0.99	0.00	0.01	0.99	0.01	0.01	0,00023
0.99	0.01	0.01	0.99	0.01	0.01	0,00023
0.99	0.01	0.01	0.99	0.01	0.01	0,00023

chr11	3525770	93	G	0	1.00	0.00	0.00	0.99	0.00	0.00	1.00	0.00	0.00	0.99	0.00	0.00	1.00	0.00	0.00	1.00	0.00	0.00
chr8	89376935	1639	C	0	1.00	0.00	0.00	1.00	0.00	0.00	0.99	0.00	0.00	0.98	0.02	0.00	0.98	0.02	0.00	1.00	0.00	0.00
chrX	7476658	2186	G	1	1.00	0.00	0.00	1.00	0.00	0.00	1.00	0.00	0.00	1.00	0.00	0.00	1.00	0.00	0.00	0.99	0.00	0.01
chr11	3525726	70	C	0	1.00	0.00	0.00	0.99	0.00	0.00	1.00	0.00	0.00	0.99	0.01	0.00	0.99	0.00	0.01	0.99	0.00	0.00
chr15	61868882	310	G	0	1.00	0.00	0.00	1.00	0.00	0.00	1.00	0.00	0.00	0.99	0.01	0.00	0.99	0.00	0.01	0.99	0.00	0.01
chr15	100300461	509	C	0	0.99	0.00	0.00	0.99	0.01	0.01	0.99	0.00	0.01	0.99	0.01	0.01	1.00	0.00	0.00	1.00	0.00	0.00
chr16	92644391	542	G	0	0.99	0.00	0.00	0.99	0.00	0.01	0.99	0.00	0.00	0.99	0.00	0.00	1.00	0.00	0.00	1.00	0.00	0.00
chr16	92644465	575	G	0	0.99	0.01	0.00	0.99	0.01	0.01	0.99	0.01	0.00	0.99	0.00	0.00	1.00	0.00	0.00	1.00	0.00	0.00
chr17	45706500	731	C	0	1.00	0.00	0.00	0.99	0.00	0.00	1.00	0.00	0.00	0.99	0.00	0.01	1.00	0.00	0.00	0.98	0.02	0.00
chr19	24277523	923	c	0	1.00	0.00	0.00	0.99	0.00	0.00	1.00	0.00	0.00	0.99	0.01	0.01	0.99	0.00	0.00	0.99	0.00	0.01
chr4	139783796	1338	G	0	1.00	0.00	0.00	1.00	0.00	0.00	0.99	0.00	0.00	0.98	0.02	0.00	0.99	0.01	0.00	0.99	0.00	0.01
chr8	89376872	1609	g	0	1.00	0.00	0.00	0.99	0.00	0.01	0.99	0.00	0.00	0.99	0.00	0.00	1.00	0.00	0.00	1.00	0.00	0.00
chr8	89376956	1644	C	0	0.99	0.01	0.00	1.00	0.00	0.00	0.99	0.00	0.00	1.00	0.00	0.00	0.99	0.01	0.00	0.99	0.01	0.00
chr8	120115810	1813	C	0	1.00	0.00	0.00	1.00	0.00	0.00	0.99	0.01	0.00	1.00	0.00	0.00	0.99	0.00	0.00	0.99	0.00	0.01
chrX	7539359	2247	G	0	0.99	0.00	0.00	1.00	0.00	0.00	1.00	0.00	0.00	0.99	0.00	0.01	1.00	0.00	0.00	1.00	0.00	0.00
chr15	100300371	489	C	0	0.99	0.00	0.00	0.99	0.00	0.00	0.99	0.00	0.00	0.99	0.01	0.00	1.00	0.00	0.00	0.99	0.00	0.00
chr16	92644328	518	G	0	0.99	0.00	0.01	0.99	0.00	0.00	1.00	0.00	0.00	1.00	0.00	0.00	1.00	0.00	0.00	1.00	0.00	0.00
chr17	45706469	711	C	0	1.00	0.00	0.00	1.00	0.00	0.00	1.00	0.00	0.00	0.99	0.00	0.00	1.00	0.00	0.00	1.00	0.00	0.00
chr19	24277761	1013	g	0	0.99	0.00	0.01	0.99	0.00	0.00	0.99	0.00	0.00	0.99	0.00	0.01	1.00	0.00	0.00	0.99	0.00	0.00
chr5	64987547	1510	G	0	0.99	0.00	0.00	1.00	0.00	0.00	0.99	0.00	0.00	1.00	0.00	0.00	0.99	0.00	0.00	0.99	0.00	0.01
chr9	100498311	1838	G	0	1.00	0.00	0.00	1.00	0.00	0.00	1.00	0.00	0.00	1.00	0.00	0.00	1.00	0.00	0.00	1.00	0.00	0.00
chrX	7539577	2380	G	0	0.99	0.00	0.01	0.99	0.00	0.01	0.99	0.00	0.00	0.99	0.00	0.00	1.00	0.00	0.00	1.00	0.00	0.00
chr15	100300030	368	C	0	0.99	0.00	0.00	0.99	0.00	0.00	0.99	0.00	0.01	0.99	0.00	0.01	1.00	0.00	0.00	1.00	0.00	0.00
chr15	100300422	502	C	0	0.99	0.00	0.00	0.99	0.00	0.00	0.99	0.00	0.00	0.99	0.00	0.00	1.00	0.00	0.00	1.00	0.00	0.00
chr16	92644500	601	G	0	0.99	0.00	0.01	0.99	0.01	0.01	1.00	0.00	0.00	1.00	0.00	0.00	1.00	0.00	0.00	1.00	0.00	0.00
chr17	45706524	742	G	0	1.00	0.00	0.00	1.00	0.00	0.00	0.99	0.00	0.00	1.00	0.00	0.00	0.99	0.00	0.01	0.99	0.00	0.01
chr4	136547354	1028	G	0	0.99	0.00	0.01	0.99	0.00	0.01	0.99	0.01	0.00	0.97	0.03	0.00	0.99	0.00	0.01	0.99	0.01	0.00
chr4	136547489	1098	C	0	0.99	0.00	0.00	0.99	0.00	0.00	1.00	0.00	0.00	0.99	0.00	0.01	1.00	0.00	0.00	1.00	0.00	0.00
chr8	89376986	1658	C	0	0.99	0.00	0.00	1.00	0.00	0.00	1.00	0.00	0.00	1.00	0.00	0.00	0.99	0.00	0.01	1.00	0.00	0.00
chrX	7476536	2100	C	1	1.00	0.00	0.00	1.00	0.00	0.00	1.00	0.00	0.00	0.99	0.01	0.00	0.99	0.00	0.01	0.99	0.01	0.00
chrX	7539465	2314	C	0	0.99	0.01	0.00	1.00	0.00	0.00	0.99	0.01	0.00	1.00	0.00	0.00	1.00	0.00	0.00	1.00	0.00	0.00
chrX	7539519	2344	C	0	0.99	0.00	0.01	0.99	0.00	0.01	1.00	0.00	0.00	0.99	0.00	0.01	0.98	0.00	0.01	0.99	0.00	0.00
chr15	100300098	402	C	0	0.99	0.00	0.00	0.99	0.00	0.00	1.00	0.00	0.00	0.99	0.00	0.00	1.00	0.00	0.00	1.00	0.00	0.00
chr15	100300174	434	G	0	1.00	0.00	0.00	0.99	0.00	0.01	1.00	0.00	0.00	0.99	0.00	0.00	1.00	0.00	0.00	0.99	0.00	0.00
chr15	100300423	503	C	0	0.99	0.00	0.00	0.99	0.00	0.00	0.99	0.00	0.01	0.99	0.00	0.00	1.00	0.00	0.00	1.00	0.00	0.00
chr17	45706512	736	G	0	1.00	0.00	0.00	0.99	0.01	0.00	0.99	0.01	0.00	0.99	0.00	0.01	0.99	0.01	0.00	0.99	0.00	0.01
chrX	7539591	2389	C	0	1.00	0.00	0.00	0.99	0.00	0.00	0.99	0.00	0.00	1.00	0.00	0.00	0.98	0.02	0.00	1.00	0.00	0.00
chr16	92644402	543	G	0	0.99	0.00	0.00	0.99	0.01	0.01	1.00	0.00	0.00	1.00	0.00	0.00	1.00	0.00	0.00	1.00	0.00	0.00
chr16	92644451	564	G	0	0.99	0.00	0.00	0.99	0.00	0.00	0.99	0.00	0.00	1.00	0.00	0.00	1.00	0.00	0.00	1.00	0.00	0.00
chr16	92644458	570	G	0	1.00	0.00	0.00	0.99	0.00	0.01	1.00	0.00	0.00	0.99	0.00	0.00	1.00	0.00	0.00	1.00	0.00	0.00
chr19	24277502	910	g	0	0.99	0.01	0.00	0.99	0.00	0.00	0.99	0.00	0.00	1.00	0.00	0.00	1.00	0.00	0.00	1.00	0.00	0.00
chr5	64987610	1533	G	0	1.00	0.00	0.00	1.00	0.00	0.00	0.99	0.00	0.00	0.99	0.00	0.00	1.00	0.00	0.00	0.99	0.00	0.00
chr9	100498426	1880	G	0	0.99	0.00	0.00	1.00	0.00	0.00	1.00	0.00	0.00	0.99	0.01	0.00	0.99	0.00	0.01	0.99	0.00	0.00
chr15	100300059	383	C	0	1.00	0.00	0.00	0.99	0.00	0.00	0.99	0.00	0.00	0.99	0.01	0.01	1.00	0.00	0.00	1.00	0.00	0.00
chr15	100300121	412	C	0	1.00	0.00	0.00	0.99	0.00	0.01	1.00	0.00	0.00	0.99	0.00	0.00	1.00	0.00	0.00	0.99	0.00	0.00
chr16	92644424	551	G	0	1.00	0.00	0.00	0.99	0.00	0.00	1.00	0.00	0.00	0.99	0.01	0.00	1.00	0.00	0.00	1.00	0.00	0.00

0.99	0.01	0.01	0.99	0.01	0.01	0,00023
0.99	0.01	0.00	0.99	0.01	0.00	0,00023
0.99	0.00	0.00	0.98	0.01	0.01	0,00023
0.99	0.01	0.01	0.99	0.01	0.01	0,00022
0.99	0.00	0.01	0.99	0.01	0.01	0,00022
0.99	0.01	0.01	0.99	0.01	0.00	0,00022
0.99	0.01	0.01	0.99	0.00	0.01	0,00022
0.99	0.01	0.01	0.99	0.00	0.01	0,00022
0.99	0.01	0.01	0.99	0.01	0.01	0,00022
0.99	0.00	0.00	0.99	0.01	0.01	0,00022
0.99	0.01	0.00	0.99	0.01	0.00	0,00022
0.99	0.01	0.01	0.99	0.01	0.00	0,00022
0.99	0.01	0.00	0.99	0.01	0.00	0,00022
0.99	0.01	0.01	0.99	0.01	0.01	0,00022
0.99	0.01	0.01	0.99	0.01	0.00	0,00022
0.99	0.00	0.01	0.99	0.01	0.01	0,00022
0.99	0.01	0.01	0.99	0.00	0.01	0,00022
0.99	0.01	0.00	0.99	0.01	0.01	0,00022
0.99	0.01	0.01	0.99	0.00	0.01	0,00022
0.99	0.01	0.01	0.99	0.01	0.01	0,00022
0.99	0.00	0.00	0.97	0.02	0.01	0,00022
0.99	0.00	0.01	0.99	0.01	0.01	0,00022
0.99	0.01	0.01	0.99	0.00	0.01	0,00022
0.99	0.01	0.01	0.99	0.01	0.01	0,00022
0.99	0.01	0.01	0.99	0.00	0.00	0,00022
0.99	0.01	0.01	0.99	0.00	0.01	0,00022
0.99	0.01	0.01	0.99	0.01	0.00	0,00022
0.99	0.01	0.01	0.99	0.00	0.01	0,00022
0.99	0.00	0.01	0.99	0.01	0.01	0,00022
0.99	0.01	0.01	0.99	0.01	0.00	0,00022
0.99	0.01	0.00	0.99	0.00	0.01	0,00022
0.99	0.00	0.01	0.99	0.01	0.01	0,00022
0.99	0.01	0.01	0.99	0.01	0.01	0,00022
0.99	0.01	0.01	0.99	0.01	0.00	0,00022
0.99	0.01	0.01	0.99	0.01	0.01	0,00022
0.99	0.01	0.01	0.99	0.01	0.00	0,00022
0.99	0.01	0.01	0.99	0.01	0.01	0,00022
0.99	0.01	0.00	0.99	0.01	0.01	0,00022
0.99	0.01	0.01	0.99	0.01	0.01	0,00022
0.99	0.00	0.01	0.99	0.00	0.01	0,00022
0.99	0.01	0.01	0.99	0.01	0.01	0,00022
0.99	0.00	0.01	0.99	0.00	0.01	0,00022
0.99	0.01	0.00	0.99	0.01	0.01	0,00022
0.99	0.01	0.01	0.99	0.01	0.01	0,00022
0.99	0.00	0.01	0.99	0.01	0.01	0,00022
0.99	0.01	0.00	0.99	0.01	0.00	0,00022

chr16	92644478	586	G	0	1.00	0.00	0.00	0.99	0.00	0.00	1.00	0.00	0.00	0.99	0.01	0.00	0.99	0.00	0.00	1.00	0.00	0.00
chr17	45706442	703	C	0	1.00	0.00	0.00	1.00	0.00	0.00	1.00	0.00	0.00	1.00	0.00	0.00	1.00	0.00	0.00	0.99	0.00	0.00
chr17	45706563	765	G	0	0.99	0.01	0.00	1.00	0.00	0.00	0.99	0.00	0.00	1.00	0.00	0.00	0.99	0.00	0.00	0.99	0.00	0.00
chr17	45706686	827	C	0	1.00	0.00	0.00	1.00	0.00	0.00	1.00	0.00	0.00	0.99	0.01	0.00	1.00	0.00	0.00	0.99	0.00	0.00
chr19	24277486	903	c	0	0.99	0.01	0.00	1.00	0.00	0.00	0.99	0.00	0.00	1.00	0.00	0.00	1.00	0.00	0.00	1.00	0.00	0.00
chr4	139783673	1271	C	0	1.00	0.00	0.00	0.99	0.00	0.01	1.00	0.00	0.00	0.99	0.00	0.00	1.00	0.00	0.00	1.00	0.00	0.00
chr8	89376993	1661	C	0	0.99	0.00	0.00	1.00	0.00	0.00	1.00	0.00	0.00	1.00	0.00	0.00	1.00	0.00	0.00	1.00	0.00	0.00
chr15	100300018	361	C	0	0.99	0.00	0.00	0.99	0.00	0.01	0.99	0.00	0.01	0.99	0.01	0.00	1.00	0.00	0.00	1.00	0.00	0.00
chr17	45706639	807	C	0	1.00	0.00	0.00	1.00	0.00	0.00	0.99	0.00	0.00	0.99	0.00	0.01	0.99	0.00	0.01	0.99	0.00	0.01
chr5	64987581	1525	C	0	1.00	0.00	0.00	1.00	0.00	0.00	0.99	0.00	0.01	0.99	0.00	0.01	0.99	0.00	0.01	0.99	0.01	0.00
chr8	89376845	1591	c	0	1.00	0.00	0.00	1.00	0.00	0.00	0.99	0.00	0.00	1.00	0.00	0.00	1.00	0.00	0.00	0.99	0.00	0.00
chr15	61868715	230	G	0	1.00	0.00	0.00	1.00	0.00	0.00	0.99	0.00	0.01	0.99	0.00	0.01	1.00	0.00	0.00	0.99	0.00	0.01
chr15	61868735	243	C	0	0.99	0.01	0.00	0.99	0.00	0.00	0.99	0.00	0.00	1.00	0.00	0.00	1.00	0.00	0.00	1.00	0.00	0.00
chr19	24277395	879	c	0	1.00	0.00	0.00	1.00	0.00	0.00	0.99	0.00	0.00	1.00	0.00	0.00	0.99	0.00	0.00	1.00	0.00	0.00
chr8	89376861	1602	c	0	1.00	0.00	0.00	1.00	0.00	0.00	0.99	0.00	0.00	1.00	0.00	0.00	1.00	0.00	0.00	1.00	0.00	0.00
chr8	120115619	1722	G	0	0.99	0.01	0.00	1.00	0.00	0.00	0.99	0.00	0.00	1.00	0.00	0.00	1.00	0.00	0.00	0.99	0.00	0.00
chrX	7476582	2133	G	1	1.00	0.00	0.00	1.00	0.00	0.00	1.00	0.00	0.00	0.98	0.00	0.02	1.00	0.00	0.00	0.99	0.00	0.01
chr17	45706634	804	C	0	1.00	0.00	0.00	1.00	0.00	0.00	1.00	0.00	0.00	1.00	0.00	0.00	0.99	0.01	0.00	0.98	0.02	0.00
chr9	100498385	1860	G	0	0.99	0.01	0.00	1.00	0.00	0.00	1.00	0.00	0.00	1.00	0.00	0.00	0.99	0.00	0.00	0.99	0.00	0.00
chr9	100498487	1916	G	0	1.00	0.00	0.00	1.00	0.00	0.00	1.00	0.00	0.00	0.99	0.00	0.00	0.99	0.01	0.00	1.00	0.00	0.00
chr15	100300298	464	G	0	1.00	0.00	0.00	0.99	0.00	0.00	0.99	0.00	0.00	1.00	0.00	0.00	0.99	0.00	0.00	1.00	0.00	0.00
chr16	92644486	592	G	0	0.99	0.01	0.01	0.99	0.00	0.00	0.99	0.00	0.00	0.99	0.01	0.00	1.00	0.00	0.00	1.00	0.00	0.00
chr4	136547738	1203	C	0	1.00	0.00	0.00	0.99	0.00	0.01	0.99	0.00	0.01	1.00	0.00	0.00	1.00	0.00	0.00	0.99	0.00	0.00
chr4	139783706	1291	C	0	1.00	0.00	0.00	0.99	0.01	0.00	1.00	0.00	0.00	0.99	0.00	0.01	0.99	0.00	0.01	0.99	0.01	0.00
chr8	120115819	1819	C	0	1.00	0.00	0.00	1.00	0.00	0.00	0.99	0.01	0.01	1.00	0.00	0.00	1.00	0.00	0.00	0.99	0.00	0.00
chr15	100300074	391	C	0	0.99	0.00	0.00	0.99	0.00	0.00	1.00	0.00	0.00	0.99	0.00	0.00	0.99	0.00	0.00	1.00	0.00	0.00
chr15	100300344	483	C	0	0.99	0.00	0.00	0.99	0.00	0.00	0.99	0.00	0.01	0.99	0.01	0.00	1.00	0.00	0.00	0.99	0.00	0.00
chr4	136547520	1119	G	0	1.00	0.00	0.00	0.99	0.00	0.00	1.00	0.00	0.00	1.00	0.00	0.00	0.99	0.00	0.00	1.00	0.00	0.00
chr8	89376981	1657	C	0	1.00	0.00	0.00	1.00	0.00	0.00	1.00	0.00	0.00	0.99	0.00	0.01	0.99	0.00	0.00	1.00	0.00	0.00
chr9	100498519	1934	C	0	0.99	0.00	0.01	1.00	0.00	0.00	0.99	0.00	0.01	0.99	0.00	0.00	0.99	0.01	0.00	0.99	0.01	0.00
chr9	100498672	1983	c	0	0.99	0.00	0.00	0.99	0.00	0.01	1.00	0.00	0.00	1.00	0.00	0.00	1.00	0.00	0.00	0.99	0.00	0.00
chrX	7539338	2236	G	0	0.99	0.00	0.00	1.00	0.00	0.00	1.00	0.00	0.00	0.99	0.00	0.01	1.00	0.00	0.00	0.99	0.00	0.00
chrX	7539499	2334	G	0	1.00	0.00	0.00	0.99	0.01	0.01	0.99	0.00	0.00	1.00	0.00	0.00	1.00	0.00	0.00	1.00	0.00	0.00
chr15	61868774	262	C	0	0.99	0.00	0.00	1.00	0.00	0.00	1.00	0.00	0.00	0.98	0.01	0.00	1.00	0.00	0.00	0.99	0.01	0.00
chr15	100300285	457	G	0	1.00	0.00	0.00	1.00	0.00	0.00	0.99	0.01	0.00	0.99	0.00	0.01	0.99	0.00	0.00	1.00	0.00	0.00
chr15	100300338	481	C	0	0.99	0.00	0.00	0.99	0.00	0.01	0.99	0.00	0.00	0.99	0.00	0.01	1.00	0.00	0.00	1.00	0.00	0.00
chr17	45706470	712	C	0	0.99	0.00	0.00	1.00	0.00	0.00	0.99	0.00	0.00	1.00	0.00	0.00	1.00	0.00	0.00	0.99	0.00	0.00
chr17	45706526	743	C	0	1.00	0.00	0.00	1.00	0.00	0.00	1.00	0.00	0.00	0.99	0.00	0.00	1.00	0.00	0.00	1.00	0.00	0.00
chr19	24277532	928	g	0	1.00	0.00	0.00	1.00	0.00	0.00	0.99	0.00	0.00	0.99	0.00	0.01	1.00	0.00	0.00	1.00	0.00	0.00
chr4	136547442	1071	C	0	1.00	0.00	0.00	0.99	0.00	0.00	0.99	0.00	0.00	1.00	0.00	0.00	1.00	0.00	0.00	1.00	0.00	0.00
chr4	139783605	1233	c	0	1.00	0.00	0.00	1.00	0.00	0.00	0.99	0.01	0.00	0.99	0.00	0.01	1.00	0.00	0.00	0.99	0.00	0.00
chr4	139783623	1243	C	0	0.99	0.00	0.01	0.99	0.01	0.00	0.99	0.01	0.00	0.98	0.01	0.00	0.99	0.00	0.01	0.99	0.00	0.01
chr4	139783629	1245	G	0	0.99	0.01	0.00	1.00	0.00	0.00	0.99	0.00	0.00	0.99	0.00	0.01	0.99	0.00	0.00	0.99	0.00	0.00
chr4	139783766	1321	C	0	0.99	0.01	0.00	1.00	0.00	0.00	1.00	0.00	0.00	1.00	0.00	0.00	1.00	0.00	0.00	1.00	0.00	0.00
chrX	7539487	2328	C	0	1.00	0.00	0.00	0.99	0.00	0.01	1.00	0.00	0.00	0.99	0.00	0.01	0.99	0.00	0.01	0.99	0.00	0.01
chrX	7539523	2346	C	0	0.99	0.00	0.00	1.00	0.00	0.00	0.99	0.00	0.00	0.99	0.00	0.00	0.99	0.01	0.01	1.00	0.00	0.00

0.99	0.01	0.01	0.99	0.01	0.01	0,00022
0.99	0.01	0.01	0.99	0.01	0.01	0,00022
0.99	0.01	0.00	0.99	0.00	0.01	0,00022
0.99	0.01	0.01	0.99	0.01	0.01	0,00022
0.99	0.01	0.01	0.99	0.00	0.00	0,00022
0.99	0.01	0.01	0.99	0.00	0.01	0,00022
0.99	0.00	0.01	0.99	0.00	0.01	0,00022
0.99	0.01	0.01	0.99	0.01	0.01	0,00022
0.99	0.01	0.01	0.99	0.01	0.01	0,00022
0.99	0.01	0.01	0.99	0.01	0.01	0,00022
0.99	0.00	0.01	0.99	0.00	0.01	0,00022
0.99	0.01	0.00	0.99	0.00	0.01	0,00022
0.99	0.01	0.00	0.99	0.01	0.01	0,00022
0.99	0.01	0.01	0.99	0.01	0.00	0,00022
0.99	0.01	0.00	0.99	0.00	0.01	0,00022
0.99	0.01	0.00	0.99	0.01	0.01	0,00022
0.99	0.01	0.00	0.99	0.01	0.01	0,00022
0.99	0.00	0.01	0.99	0.01	0.01	0,00022
0.99	0.01	0.00	0.99	0.01	0.01	0,00022
0.99	0.01	0.01	0.99	0.01	0.00	0,00022
0.99	0.00	0.01	0.99	0.00	0.01	0,00022
0.99	0.01	0.01	0.99	0.00	0.01	0,00022
0.99	0.01	0.01	0.99	0.00	0.00	0,00022
0.99	0.01	0.00	0.99	0.01	0.01	0,00022
0.99	0.01	0.01	0.99	0.00	0.01	0,00022
0.99	0.01	0.01	0.99	0.01	0.01	0,00021
0.99	0.01	0.01	0.99	0.01	0.01	0,00021
0.99	0.01	0.01	0.99	0.01	0.01	0,00021
0.99	0.01	0.00	0.99	0.00	0.01	0,00021
0.99	0.00	0.01	0.99	0.01	0.01	0,00021
0.99	0.00	0.01	0.99	0.01	0.00	0,00021
0.99	0.01	0.01	0.99	0.00	0.01	0,00021
0.99	0.01	0.01	0.99	0.00	0.00	0,00021
0.99	0.01	0.01	0.99	0.01	0.00	0,00021
0.99	0.01	0.01	0.99	0.01	0.01	0,00021
0.99	0.01	0.01	0.99	0.01	0.01	0,00021
0.99	0.01	0.01	0.99	0.00	0.01	0,00021
0.99	0.01	0.01	0.99	0.00	0.01	0,00021
0.99	0.01	0.01	0.99	0.00	0.01	0,00021
0.99	0.01	0.01	0.99	0.00	0.00	0,00021
0.99	0.01	0.00	0.99	0.00	0.01	0,00021
0.99	0.01	0.00	0.99	0.00	0.01	0,00021
0.99	0.00	0.01	0.99	0.01	0.01	0,00021
0.99	0.01	0.00	0.99	0.01	0.01	0,00021
0.99	0.00	0.01	0.99	0.00	0.01	0,00021
0.99	0.01	0.00	0.99	0.00	0.01	0,00021

chrX	7539647	2421	C	0	0.99	0.00	0.00	1.00	0.00	0.00	1.00	0.00	0.00	1.00	0.00	0.00	1.00	0.00	0.00	1.00	0.00	0.00
chr1!	61868672	210	C	0	1.00	0.00	0.00	1.00	0.00	0.00	1.00	0.00	0.00	0.98	0.02	0.00	1.00	0.00	0.00	1.00	0.00	0.00
chr1!	100299987	347	C	0	0.99	0.01	0.01	0.99	0.00	0.01	0.99	0.00	0.00	0.99	0.00	0.00	1.00	0.00	0.00	1.00	0.00	0.00
chr8	89376967	1650	C	0	0.99	0.00	0.00	1.00	0.00	0.00	0.99	0.00	0.00	1.00	0.00	0.00	1.00	0.00	0.00	0.99	0.00	0.00
chr9	100498368	1852	G	0	1.00	0.00	0.00	0.99	0.00	0.00	1.00	0.00	0.00	1.00	0.00	0.00	1.00	0.00	0.00	1.00	0.00	0.00
chr9	100498639	1977	C	0	0.99	0.01	0.00	0.99	0.00	0.00	0.99	0.00	0.01	1.00	0.00	0.00	0.99	0.01	0.00	0.99	0.00	0.01
chr1!	100300288	458	G	0	1.00	0.00	0.00	1.00	0.00	0.00	0.99	0.00	0.00	1.00	0.00	0.00	0.99	0.01	0.00	1.00	0.00	0.00
chr1;	45706707	834	G	0	1.00	0.00	0.00	0.99	0.00	0.00	1.00	0.00	0.00	1.00	0.00	0.00	1.00	0.00	0.00	1.00	0.00	0.00
chr1!	24277653	981	C	0	0.99	0.01	0.00	1.00	0.00	0.00	1.00	0.00	0.00	0.99	0.00	0.00	1.00	0.00	0.00	1.00	0.00	0.00
chr4	136547358	1029	G	0	1.00	0.00	0.00	1.00	0.00	0.00	0.99	0.00	0.00	0.99	0.00	0.01	1.00	0.00	0.00	0.99	0.01	0.00
chr8	120115652	1738	G	0	0.99	0.01	0.00	0.99	0.00	0.00	0.99	0.01	0.00	0.99	0.00	0.00	1.00	0.00	0.00	1.00	0.00	0.00
chr1	3525882	164	G	0	1.00	0.00	0.00	0.99	0.00	0.00	1.00	0.00	0.00	1.00	0.00	0.00	0.99	0.01	0.00	1.00	0.00	0.00
chr1!	100299986	346	C	0	0.99	0.00	0.00	0.99	0.00	0.01	0.99	0.00	0.00	0.99	0.00	0.01	1.00	0.00	0.00	1.00	0.00	0.00
chr1(	92644423	550	G	0	1.00	0.00	0.00	1.00	0.00	0.00	1.00	0.00	0.00	1.00	0.00	0.00	1.00	0.00	0.00	1.00	0.00	0.00
chr4	136547474	1091	C	0	0.99	0.00	0.01	1.00	0.00	0.00	0.99	0.00	0.00	1.00	0.00	0.00	0.98	0.02	0.00	1.00	0.00	0.00
chr4	139783800	1339	C	0	1.00	0.00	0.00	0.99	0.00	0.00	1.00	0.00	0.00	0.99	0.00	0.01	1.00	0.00	0.00	1.00	0.00	0.00
chrX	7539334	2234	G	0	1.00	0.00	0.00	1.00	0.00	0.00	1.00	0.00	0.00	1.00	0.00	0.00	1.00	0.00	0.00	1.00	0.00	0.00
chr1	3525660	33	G	0	1.00	0.00	0.00	1.00	0.00	0.00	0.98	0.02	0.00	0.98	0.01	0.00	0.99	0.00	0.00	1.00	0.00	0.00
chr1	3525670	39	C	0	1.00	0.00	0.00	0.99	0.00	0.01	1.00	0.00	0.00	0.99	0.00	0.00	1.00	0.00	0.00	1.00	0.00	0.00
chr1	3525771	94	G	0	1.00	0.00	0.00	0.99	0.00	0.00	1.00	0.00	0.00	0.99	0.00	0.01	0.99	0.00	0.01	1.00	0.00	0.00
chr1	3525779	98	C	0	1.00	0.00	0.00	1.00	0.00	0.00	1.00	0.00	0.00	1.00	0.00	0.00	1.00	0.00	0.00	1.00	0.00	0.00
chr1	3525825	127	C	0	1.00	0.00	0.00	1.00	0.00	0.00	0.99	0.01	0.00	0.99	0.01	0.00	0.99	0.00	0.00	0.99	0.01	0.00
chr1(	92644381	537	G	0	0.99	0.00	0.00	0.99	0.00	0.01	0.99	0.00	0.00	1.00	0.00	0.00	1.00	0.00	0.00	1.00	0.00	0.00
chr1(	92644431	553	G	0	0.99	0.00	0.00	0.99	0.00	0.00	0.99	0.00	0.00	0.99	0.00	0.00	1.00	0.00	0.00	1.00	0.00	0.00
chr4	136547561	1143	G	0	1.00	0.00	0.00	1.00	0.00	0.00	0.99	0.00	0.00	1.00	0.00	0.00	1.00	0.00	0.00	0.99	0.00	0.00
chr5	64987435	1457	G	0	1.00	0.00	0.00	0.99	0.00	0.00	0.99	0.00	0.01	0.99	0.00	0.01	0.99	0.00	0.00	0.99	0.01	0.00
chr5	64987514	1497	C	0	1.00	0.00	0.00	0.99	0.00	0.00	1.00	0.00	0.00	1.00	0.00	0.00	0.99	0.00	0.00	0.99	0.00	0.01
chr8	89376904	1627	G	0	0.99	0.01	0.00	1.00	0.00	0.00	1.00	0.00	0.00	0.97	0.03	0.00	1.00	0.00	0.00	1.00	0.00	0.00
chr8	120115815	1817	C	0	1.00	0.00	0.00	0.99	0.00	0.00	1.00	0.00	0.00	1.00	0.00	0.00	1.00	0.00	0.00	0.99	0.00	0.00
chr8	120115820	1820	C	0	1.00	0.00	0.00	1.00	0.00	0.00	1.00	0.00	0.00	0.99	0.00	0.01	1.00	0.00	0.00	1.00	0.00	0.00
chrX	7476560	2119	C	0	1.00	0.00	0.00	1.00	0.00	0.00	1.00	0.00	0.00	1.00	0.00	0.00	0.99	0.00	0.01	1.00	0.00	0.00
chr1	3525635	22	G	0	1.00	0.00	0.00	1.00	0.00	0.00	1.00	0.00	0.00	1.00	0.00	0.00	0.99	0.00	0.00	1.00	0.00	0.00
chr1!	61868695	222	C	0	1.00	0.00	0.00	0.99	0.00	0.01	0.99	0.01	0.00	1.00	0.00	0.00	1.00	0.00	0.00	0.99	0.00	0.01
chr1(	92644516	614	G	0	0.99	0.00	0.00	0.99	0.00	0.00	0.99	0.00	0.00	0.99	0.00	0.00	1.00	0.00	0.00	0.99	0.00	0.00
chr1!	24277558	941	g	0	1.00	0.00	0.00	1.00	0.00	0.00	0.99	0.00	0.01	0.98	0.01	0.00	1.00	0.00	0.00	1.00	0.00	0.00
chr1!	24277702	997	G	0	1.00	0.00	0.00	0.99	0.00	0.00	0.99	0.00	0.00	1.00	0.00	0.00	0.99	0.00	0.01	1.00	0.00	0.00
chr4	136547555	1140	G	0	0.99	0.01	0.00	1.00	0.00	0.00	0.99	0.00	0.00	0.98	0.02	0.00	0.98	0.00	0.02	0.98	0.00	0.02
chrX	7539583	2385	G	0	1.00	0.00	0.00	0.99	0.00	0.00	1.00	0.00	0.00	0.99	0.00	0.01	1.00	0.00	0.00	1.00	0.00	0.00
chr1!	61868865	303	G	0	1.00	0.00	0.00	1.00	0.00	0.00	0.99	0.00	0.00	1.00	0.00	0.00	0.99	0.00	0.01	1.00	0.00	0.00
chr1!	100300376	491	C	0	0.99	0.00	0.00	0.99	0.00	0.00	0.99	0.00	0.00	0.99	0.01	0.01	1.00	0.00	0.00	1.00	0.00	0.00
chr1(	92644430	552	G	0	0.99	0.00	0.00	0.99	0.00	0.00	1.00	0.00	0.00	0.99	0.00	0.01	1.00	0.00	0.00	1.00	0.00	0.00
chr1;	45706506	733	G	0	0.99	0.00	0.01	0.99	0.00	0.00	1.00	0.00	0.00	1.00	0.00	0.00	1.00	0.00	0.00	0.99	0.00	0.01
chr1;	45706571	768	G	0	0.99	0.00	0.00	0.99	0.00	0.00	1.00	0.00	0.00	0.99	0.00	0.01	0.99	0.00	0.01	1.00	0.00	0.00
chr4	136547471	1089	G	0	1.00	0.00	0.00	1.00	0.00	0.00	1.00	0.00	0.00	1.00	0.00	0.00	1.00	0.00	0.00	1.00	0.00	0.00
chr4	136547512	1113	C	0	1.00	0.00	0.00	0.99	0.00	0.00	1.00	0.00	0.00	1.00	0.00	0.00	1.00	0.00	0.00	0.99	0.00	0.00
chr5	64987468	1476	C	0	1.00	0.00	0.00	1.00	0.00	0.00	0.99	0.00	0.00	0.99	0.00	0.00	0.99	0.00	0.00	1.00	0.00	0.00

0.99	0.01	0.01	0.99	0.01	0.00	0,00021
0.99	0.00	0.00	0.99	0.01	0.00	0,00021
0.99	0.01	0.01	0.99	0.01	0.00	0,00021
0.99	0.00	0.01	0.99	0.01	0.01	0,00021
0.99	0.01	0.01	0.99	0.01	0.00	0,00021
0.99	0.00	0.01	0.99	0.00	0.01	0,00021
0.99	0.01	0.00	0.99	0.01	0.00	0,00021
0.99	0.01	0.01	0.99	0.01	0.00	0,00021
0.99	0.01	0.00	0.99	0.01	0.00	0,00021
0.99	0.00	0.01	0.99	0.01	0.00	0,00021
0.99	0.01	0.00	0.99	0.01	0.01	0,00021
0.99	0.01	0.01	0.99	0.01	0.01	0,00021
0.99	0.01	0.01	0.99	0.00	0.00	0,00021
0.99	0.00	0.01	0.99	0.01	0.00	0,00021
0.99	0.00	0.01	0.99	0.01	0.01	0,00021
0.99	0.01	0.01	0.99	0.00	0.01	0,00021
0.99	0.00	0.00	0.99	0.01	0.00	0,00021
0.99	0.01	0.00	0.99	0.01	0.01	0,00021
0.99	0.01	0.00	0.99	0.01	0.01	0,00021
0.99	0.01	0.00	0.99	0.01	0.01	0,00021
0.99	0.01	0.00	0.99	0.01	0.00	0,00021
0.99	0.01	0.00	0.99	0.01	0.00	0,00021
0.99	0.01	0.01	0.99	0.00	0.00	0,00021
0.99	0.01	0.01	0.99	0.01	0.01	0,00021
0.99	0.01	0.01	0.99	0.00	0.00	0,00021
0.99	0.01	0.01	0.99	0.01	0.01	0,00021
0.99	0.00	0.01	0.99	0.00	0.01	0,00021
0.99	0.01	0.00	0.99	0.01	0.01	0,00021
0.99	0.01	0.01	0.99	0.01	0.01	0,00021
0.99	0.00	0.01	0.99	0.00	0.01	0,00021
0.99	0.01	0.00	0.99	0.01	0.01	0,00021
0.99	0.00	0.00	0.99	0.01	0.01	0,00021
0.99	0.01	0.01	0.99	0.00	0.01	0,00021
0.99	0.01	0.01	0.99	0.01	0.01	0,00021
0.99	0.00	0.01	0.99	0.01	0.01	0,00021
0.99	0.01	0.01	0.99	0.00	0.01	0,00021
0.99	0.00	0.00	0.99	0.01	0.01	0,00021
0.99	0.01	0.00	0.99	0.01	0.01	0,00021
0.99	0.00	0.01	0.99	0.00	0.01	0,00021
0.99	0.01	0.01	0.99	0.00	0.01	0,00021
0.99	0.01	0.00	0.99	0.01	0.01	0,00021
0.99	0.00	0.01	0.99	0.00	0.01	0,00021
0.99	0.01	0.01	0.99	0.01	0.01	0,00021
0.99	0.01	0.00	0.99	0.01	0.00	0,00021
0.99	0.00	0.01	0.99	0.01	0.00	0,00021
0.99	0.01	0.00	0.99	0.01	0.01	0,00021

chr8	120115688	1757	C	0	1.00	0.00	0.00	0.99	0.00	0.00	0.99	0.00	0.00	1.00	0.00	0.00	1.00	0.00	0.00	0.99	0.00	0.00
chr9	100498485	1914	G	0	1.00	0.00	0.00	1.00	0.00	0.00	1.00	0.00	0.00	1.00	0.00	0.00	0.99	0.00	0.00	0.99	0.00	0.00
chrX	7476587	2137	C	1	1.00	0.00	0.00	1.00	0.00	0.00	1.00	0.00	0.00	0.99	0.00	0.01	0.96	0.01	0.03	0.98	0.02	0.00
chr11	3525688	47	G	0	0.99	0.00	0.00	0.99	0.00	0.00	1.00	0.00	0.00	1.00	0.00	0.00	0.99	0.00	0.00	0.99	0.01	0.00
chr16	92644453	566	C	0	1.00	0.00	0.00	0.99	0.00	0.00	0.99	0.00	0.00	0.99	0.00	0.00	0.99	0.00	0.00	1.00	0.00	0.00
chr16	92644529	620	G	0	1.00	0.00	0.00	0.99	0.00	0.00	1.00	0.00	0.00	0.99	0.00	0.01	1.00	0.00	0.00	1.00	0.00	0.00
chr17	45706491	725	C	0	0.99	0.00	0.00	1.00	0.00	0.00	1.00	0.00	0.00	0.99	0.00	0.00	0.99	0.00	0.00	1.00	0.00	0.00
chr17	45706608	791	G	0	0.99	0.00	0.00	0.99	0.00	0.00	0.99	0.00	0.00	1.00	0.00	0.00	0.99	0.00	0.00	1.00	0.00	0.00
chr5	64987402	1441	G	0	1.00	0.00	0.00	0.99	0.00	0.00	1.00	0.00	0.00	0.97	0.03	0.00	1.00	0.00	0.00	1.00	0.00	0.00
chr8	120115844	1832	C	0	1.00	0.00	0.00	1.00	0.00	0.00	1.00	0.00	0.00	0.99	0.00	0.00	0.99	0.00	0.01	0.99	0.00	0.01
chr9	100498510	1929	G	0	0.99	0.00	0.00	1.00	0.00	0.00	1.00	0.00	0.00	1.00	0.00	0.00	1.00	0.00	0.00	1.00	0.00	0.00
chrX	7476371	2019	G	1	1.00	0.00	0.00	1.00	0.00	0.00	1.00	0.00	0.00	0.99	0.00	0.01	1.00	0.00	0.00	0.99	0.00	0.00
chrX	7539640	2418	C	0	0.99	0.00	0.00	0.99	0.00	0.01	0.98	0.00	0.02	0.98	0.01	0.00	0.98	0.02	0.00	0.99	0.00	0.01
chr11	3525835	132	G	0	1.00	0.00	0.00	1.00	0.00	0.00	1.00	0.00	0.00	1.00	0.00	0.00	1.00	0.00	0.00	1.00	0.00	0.00
chr15	61868944	335	G	0	1.00	0.00	0.00	1.00	0.00	0.00	1.00	0.00	0.00	1.00	0.00	0.00	1.00	0.00	0.00	1.00	0.00	0.00
chr15	100300066	386	C	0	1.00	0.00	0.00	0.99	0.00	0.00	0.99	0.00	0.00	0.99	0.01	0.01	1.00	0.00	0.00	1.00	0.00	0.00
chr15	100300402	496	C	0	1.00	0.00	0.00	0.99	0.00	0.00	0.99	0.00	0.00	0.99	0.00	0.00	1.00	0.00	0.00	1.00	0.00	0.00
chr8	120115653	1739	G	0	1.00	0.00	0.00	1.00	0.00	0.00	1.00	0.00	0.00	1.00	0.00	0.00	1.00	0.00	0.00	1.00	0.00	0.00
chrX	7476635	2169	C	1	1.00	0.00	0.00	1.00	0.00	0.00	1.00	0.00	0.00	0.99	0.01	0.00	0.99	0.00	0.01	0.99	0.01	0.00
chr15	100299985	345	C	0	0.99	0.00	0.00	0.99	0.00	0.00	0.99	0.00	0.01	0.99	0.00	0.01	1.00	0.00	0.00	1.00	0.00	0.00
chr5	64987414	1444	G	0	1.00	0.00	0.00	0.99	0.01	0.00	1.00	0.00	0.00	1.00	0.00	0.00	1.00	0.00	0.00	0.99	0.00	0.00
chr9	100498522	1936	C	0	0.99	0.00	0.00	1.00	0.00	0.00	0.99	0.00	0.01	0.98	0.02	0.00	1.00	0.00	0.00	1.00	0.00	0.00
chr9	100498563	1957	C	0	1.00	0.00	0.00	0.99	0.00	0.00	1.00	0.00	0.00	0.99	0.00	0.01	0.99	0.00	0.01	0.99	0.00	0.00
chrX	7476495	2069	C	1	1.00	0.00	0.00	1.00	0.00	0.00	1.00	0.00	0.00	0.99	0.01	0.00	0.98	0.00	0.02	1.00	0.00	0.00
chr15	61868805	274	G	0	1.00	0.00	0.00	0.99	0.00	0.00	1.00	0.00	0.00	1.00	0.00	0.00	1.00	0.00	0.00	1.00	0.00	0.00
chr15	100300173	433	G	0	0.99	0.00	0.01	0.99	0.00	0.00	0.99	0.00	0.01	0.99	0.00	0.00	0.99	0.00	0.01	1.00	0.00	0.00
chr15	100300455	507	C	0	0.99	0.00	0.00	0.99	0.00	0.01	0.99	0.00	0.00	0.99	0.00	0.01	1.00	0.00	0.00	1.00	0.00	0.00
chr16	92644327	517	G	0	0.99	0.00	0.00	0.99	0.00	0.00	1.00	0.00	0.00	0.99	0.00	0.00	1.00	0.00	0.00	1.00	0.00	0.00
chr16	92644487	593	G	0	0.99	0.00	0.00	0.99	0.00	0.00	0.99	0.00	0.00	0.99	0.00	0.00	1.00	0.00	0.00	1.00	0.00	0.00
chr19	24277731	1005	G	0	1.00	0.00	0.00	0.99	0.00	0.00	0.99	0.00	0.00	0.99	0.00	0.00	1.00	0.00	0.00	1.00	0.00	0.00
chr4	139783654	1261	G	0	1.00	0.00	0.00	1.00	0.00	0.00	1.00	0.00	0.00	0.99	0.00	0.01	1.00	0.00	0.00	1.00	0.00	0.00
chrX	7476567	2124	G	1	1.00	0.00	0.00	1.00	0.00	0.00	0.99	0.01	0.00	0.99	0.00	0.01	1.00	0.00	0.00	1.00	0.00	0.00
chrX	7476608	2150	G	0	1.00	0.00	0.00	0.99	0.00	0.00	1.00	0.00	0.00	1.00	0.00	0.00	1.00	0.00	0.00	1.00	0.00	0.00
chrX	7476631	2166	G	1	1.00	0.00	0.00	1.00	0.00	0.00	1.00	0.00	0.00	1.00	0.00	0.00	0.99	0.01	0.00	0.99	0.00	0.00
chrX	7476636	2170	G	1	1.00	0.00	0.00	1.00	0.00	0.00	1.00	0.00	0.00	0.99	0.00	0.01	1.00	0.00	0.00	1.00	0.00	0.00
chr11	3525805	115	C	0	1.00	0.00	0.00	1.00	0.00	0.00	1.00	0.00	0.00	0.99	0.00	0.00	1.00	0.00	0.00	1.00	0.00	0.00
chr11	3525817	120	C	0	1.00	0.00	0.00	1.00	0.00	0.00	1.00	0.00	0.00	1.00	0.00	0.00	0.99	0.00	0.01	1.00	0.00	0.00
chr15	61868674	212	C	0	1.00	0.00	0.00	1.00	0.00	0.00	0.99	0.01	0.00	1.00	0.00	0.00	1.00	0.00	0.00	0.99	0.00	0.00
chr16	92644366	529	G	0	0.99	0.00	0.00	0.99	0.00	0.00	1.00	0.00	0.00	1.00	0.00	0.00	1.00	0.00	0.00	1.00	0.00	0.00
chr17	45706715	839	G	0	1.00	0.00	0.00	1.00	0.00	0.00	0.99	0.00	0.00	1.00	0.00	0.00	1.00	0.00	0.00	1.00	0.00	0.00
chr19	24277531	927	g	0	0.99	0.00	0.00	0.99	0.00	0.01	0.99	0.00	0.00	0.99	0.00	0.01	1.00	0.00	0.00	1.00	0.00	0.00
chr19	24277726	1004	G	0	1.00	0.00	0.00	0.99	0.00	0.00	1.00	0.00	0.00	0.99	0.00	0.00	1.00	0.00	0.00	1.00	0.00	0.00
chr5	64987354	1424	G	0	1.00	0.00	0.00	1.00	0.00	0.00	1.00	0.00	0.00	0.98	0.02	0.00	1.00	0.00	0.00	1.00	0.00	0.00
chr9	100498536	1942	G	0	1.00	0.00	0.00	1.00	0.00	0.00	1.00	0.00	0.00	1.00	0.00	0.00	1.00	0.00	0.00	1.00	0.00	0.00
chrX	7539528	2349	C	0	1.00	0.00	0.00	1.00	0.00	0.00	1.00	0.00	0.00	0.99	0.00	0.01	0.99	0.01	0.01	0.99	0.00	0.00
chr11	3525722	67	G	0	0.99	0.01	0.00	1.00	0.00	0.00	1.00	0.00	0.00	0.99	0.01	0.00	0.99	0.01	0.00	0.99	0.00	0.00

0.99	0.01	0.01	0.99	0.00	0.01	0,00021
0.99	0.01	0.01	0.99	0.01	0.00	0,00021
0.99	0.01	0.00	0.99	0.01	0.01	0,00021
0.99	0.01	0.01	0.99	0.01	0.01	0,00021
0.99	0.00	0.01	0.99	0.00	0.01	0,00021
0.99	0.00	0.00	0.99	0.01	0.01	0,00021
0.99	0.00	0.01	0.99	0.01	0.01	0,00021
0.99	0.00	0.01	0.99	0.01	0.00	0,00021
0.99	0.00	0.00	0.99	0.01	0.01	0,00021
0.99	0.01	0.00	0.99	0.01	0.01	0,00021
0.99	0.01	0.00	0.99	0.00	0.01	0,00021
0.99	0.01	0.01	0.99	0.01	0.01	0,00021
0.99	0.01	0.01	0.99	0.01	0.00	0,00021
0.99	0.00	0.01	0.99	0.01	0.01	0,00021
0.99	0.00	0.00	0.99	0.01	0.01	0,00021
0.99	0.01	0.00	0.99	0.01	0.01	0,00021
0.99	0.00	0.01	0.99	0.01	0.00	0,00021
0.99	0.00	0.01	0.99	0.01	0.00	0,00021
0.99	0.00	0.01	0.99	0.01	0.00	0,00021
0.99	0.01	0.01	0.99	0.00	0.00	0,0002
0.99	0.01	0.01	0.99	0.00	0.01	0,0002
0.99	0.01	0.00	0.99	0.01	0.00	0,0002
0.99	0.00	0.01	0.99	0.01	0.01	0,0002
0.99	0.01	0.00	0.99	0.01	0.01	0,0002
0.99	0.00	0.01	0.99	0.01	0.01	0,0002
0.99	0.01	0.01	0.99	0.01	0.01	0,0002
0.99	0.01	0.01	0.99	0.01	0.00	0,0002
0.99	0.00	0.01	0.99	0.01	0.01	0,0002
0.99	0.01	0.01	0.99	0.00	0.00	0,0002
0.99	0.01	0.01	0.99	0.01	0.01	0,0002
0.99	0.00	0.00	0.99	0.00	0.01	0,0002
0.99	0.01	0.00	0.99	0.01	0.00	0,0002
0.99	0.01	0.01	0.99	0.01	0.00	0,0002
0.99	0.00	0.00	0.99	0.01	0.00	0,0002
0.99	0.00	0.01	0.99	0.01	0.01	0,0002
0.99	0.01	0.00	0.99	0.01	0.00	0,0002
0.99	0.01	0.01	0.99	0.01	0.01	0,0002
0.99	0.01	0.00	0.99	0.01	0.00	0,0002
0.99	0.01	0.01	0.99	0.00	0.00	0,0002
0.99	0.00	0.00	0.99	0.01	0.00	0,0002
0.99	0.01	0.01	0.99	0.00	0.01	0,0002
0.99	0.00	0.01	0.99	0.00	0.01	0,0002
0.99	0.01	0.01	0.99	0.01	0.00	0,0002
0.99	0.00	0.00	0.98	0.01	0.01	0,0002
0.99	0.00	0.00	0.99	0.01	0.01	0,0002
0.99	0.00	0.00	0.99	0.01	0.01	0,0002

chr16	92644370	530	G	0	0.99	0.00	0.00	0.99	0.00	0.00	0.99	0.00	0.00	0.99	0.00	0.00	1.00	0.00	0.00	1.00	0.00	0.00
chr16	92644505	605	G	0	0.99	0.00	0.01	0.99	0.01	0.01	1.00	0.00	0.00	1.00	0.00	0.00	1.00	0.00	0.00	1.00	0.00	0.00
chr17	45706479	718	C	0	1.00	0.00	0.00	1.00	0.00	0.00	1.00	0.00	0.00	0.99	0.00	0.00	1.00	0.00	0.00	0.99	0.00	0.00
chr17	45706731	851	G	0	1.00	0.00	0.00	0.99	0.00	0.00	1.00	0.00	0.00	1.00	0.00	0.00	1.00	0.00	0.00	0.99	0.00	0.01
chr5	64987426	1453	G	0	1.00	0.00	0.00	0.99	0.00	0.00	0.99	0.00	0.00	0.99	0.00	0.00	1.00	0.00	0.00	1.00	0.00	0.00
chr8	89377049	1678	G	0	1.00	0.00	0.00	1.00	0.00	0.00	1.00	0.00	0.00	0.97	0.02	0.00	1.00	0.00	0.00	0.99	0.01	0.00
chrX	7539608	2400	C	0	1.00	0.00	0.00	0.99	0.00	0.00	0.99	0.00	0.00	1.00	0.00	0.00	0.98	0.02	0.00	1.00	0.00	0.00
chr11	3525645	29	C	0	1.00	0.00	0.00	1.00	0.00	0.00	1.00	0.00	0.00	1.00	0.00	0.00	1.00	0.00	0.00	1.00	0.00	0.00
chr11	3525703	55	G	0	1.00	0.00	0.00	0.99	0.00	0.00	1.00	0.00	0.00	1.00	0.00	0.00	0.99	0.00	0.00	1.00	0.00	0.00
chr15	61868707	226	C	0	1.00	0.00	0.00	1.00	0.00	0.00	1.00	0.00	0.00	0.99	0.00	0.00	1.00	0.00	0.00	1.00	0.00	0.00
chr15	61868935	333	G	0	1.00	0.00	0.00	0.99	0.00	0.00	1.00	0.00	0.00	1.00	0.00	0.00	1.00	0.00	0.00	1.00	0.00	0.00
chr16	92644452	565	G	0	0.99	0.00	0.00	0.99	0.00	0.00	1.00	0.00	0.00	0.99	0.00	0.00	1.00	0.00	0.00	1.00	0.00	0.00
chr17	45706577	773	G	0	1.00	0.00	0.00	0.99	0.00	0.00	1.00	0.00	0.00	1.00	0.00	0.00	1.00	0.00	0.00	1.00	0.00	0.00
chr19	24277622	971	C	0	0.99	0.01	0.00	1.00	0.00	0.00	0.99	0.00	0.00	1.00	0.00	0.00	1.00	0.00	0.00	1.00	0.00	0.00
chr4	139783645	1255	C	0	1.00	0.00	0.00	1.00	0.00	0.00	0.99	0.01	0.00	0.98	0.02	0.00	1.00	0.00	0.00	0.99	0.01	0.00
chr4	139783869	1379	G	0	0.99	0.00	0.00	1.00	0.00	0.00	1.00	0.00	0.00	0.99	0.01	0.00	1.00	0.00	0.00	0.99	0.01	0.00
chr8	120115584	1707	G	0	1.00	0.00	0.00	0.99	0.00	0.00	1.00	0.00	0.00	1.00	0.00	0.00	1.00	0.00	0.00	1.00	0.00	0.00
chr15	61868764	256	C	0	1.00	0.00	0.00	0.99	0.00	0.00	1.00	0.00	0.00	1.00	0.00	0.00	1.00	0.00	0.00	1.00	0.00	0.00
chr15	100300182	436	G	0	1.00	0.00	0.00	0.99	0.00	0.00	0.99	0.00	0.00	0.99	0.00	0.00	1.00	0.00	0.00	1.00	0.00	0.00
chr16	92644408	544	G	0	0.99	0.00	0.00	0.99	0.00	0.00	1.00	0.00	0.00	1.00	0.00	0.00	1.00	0.00	0.00	1.00	0.00	0.00
chr16	92644460	572	G	0	1.00	0.00	0.00	1.00	0.00	0.00	1.00	0.00	0.00	0.99	0.00	0.01	1.00	0.00	0.00	1.00	0.00	0.00
chr16	92644531	621	G	0	0.99	0.00	0.00	0.99	0.00	0.00	1.00	0.00	0.00	0.99	0.00	0.00	0.99	0.00	0.00	1.00	0.00	0.00
chr17	45706643	809	C	0	1.00	0.00	0.00	1.00	0.00	0.00	1.00	0.00	0.00	0.99	0.00	0.00	1.00	0.00	0.00	1.00	0.00	0.00
chr17	45706734	852	G	0	1.00	0.00	0.00	0.99	0.00	0.00	1.00	0.00	0.00	1.00	0.00	0.00	1.00	0.00	0.00	1.00	0.00	0.00
chr19	24277480	900	c	0	1.00	0.00	0.00	0.99	0.00	0.00	1.00	0.00	0.00	0.99	0.00	0.00	1.00	0.00	0.00	1.00	0.00	0.00
chr4	136547428	1062	G	0	1.00	0.00	0.00	1.00	0.00	0.00	1.00	0.00	0.00	1.00	0.00	0.00	1.00	0.00	0.00	1.00	0.00	0.00
chr4	139783870	1380	C	0	1.00	0.00	0.00	1.00	0.00	0.00	1.00	0.00	0.00	0.97	0.02	0.00	1.00	0.00	0.00	0.99	0.01	0.00
chr5	64987434	1456	G	0	1.00	0.00	0.00	1.00	0.00	0.00	1.00	0.00	0.00	0.99	0.00	0.00	1.00	0.00	0.00	1.00	0.00	0.00
chr5	64987591	1528	G	0	1.00	0.00	0.00	0.99	0.00	0.00	1.00	0.00	0.00	1.00	0.00	0.00	0.99	0.00	0.00	1.00	0.00	0.00
chrX	7476588	2138	G	1	1.00	0.00	0.00	1.00	0.00	0.00	0.99	0.01	0.00	0.99	0.00	0.01	1.00	0.00	0.00	0.99	0.00	0.01
chr11	3525918	183	G	0	1.00	0.00	0.00	0.99	0.00	0.00	1.00	0.00	0.00	0.99	0.01	0.00	1.00	0.00	0.00	1.00	0.00	0.00
chr15	61868866	304	G	0	1.00	0.00	0.00	1.00	0.00	0.00	0.99	0.00	0.00	1.00	0.00	0.00	0.99	0.00	0.01	1.00	0.00	0.00
chr16	92644414	545	G	0	0.99	0.00	0.00	0.99	0.00	0.00	0.99	0.00	0.00	0.99	0.00	0.00	1.00	0.00	0.00	1.00	0.00	0.00
chr16	92644481	588	G	0	0.99	0.00	0.00	0.99	0.01	0.01	1.00	0.00	0.00	1.00	0.00	0.00	1.00	0.00	0.00	1.00	0.00	0.00
chr19	24277394	878	c	0	1.00	0.00	0.00	0.99	0.00	0.00	1.00	0.00	0.00	1.00	0.00	0.00	1.00	0.00	0.00	1.00	0.00	0.00
chr19	24277631	975	C	0	1.00	0.00	0.00	0.99	0.00	0.00	1.00	0.00	0.00	0.99	0.00	0.00	0.99	0.00	0.00	1.00	0.00	0.00
chr19	24277635	976	C	0	1.00	0.00	0.00	0.99	0.00	0.01	1.00	0.00	0.00	0.99	0.00	0.00	0.99	0.00	0.00	1.00	0.00	0.00
chr4	136547677	1189	C	0	0.99	0.00	0.00	1.00	0.00	0.00	0.99	0.00	0.01	1.00	0.00	0.00	1.00	0.00	0.00	1.00	0.00	0.00
chr5	64987511	1495	C	0	1.00	0.00	0.00	0.99	0.01	0.00	1.00	0.00	0.00	0.99	0.00	0.01	0.99	0.00	0.00	1.00	0.00	0.00
chr8	120115702	1765	G	0	0.99	0.00	0.01	1.00	0.00	0.00	1.00	0.00	0.00	1.00	0.00	0.00	0.99	0.00	0.00	1.00	0.00	0.00
chr11	3525713	60	G	0	1.00	0.00	0.00	1.00	0.00	0.00	1.00	0.00	0.00	1.00	0.00	0.00	1.00	0.00	0.00	0.99	0.01	0.00
chr15	61868706	225	C	0	1.00	0.00	0.00	0.99	0.00	0.00	0.99	0.00	0.00	1.00	0.00	0.00	0.99	0.00	0.00	1.00	0.00	0.00
chr15	61868815	279	G	0	1.00	0.00	0.00	1.00	0.00	0.00	0.99	0.00	0.00	0.99	0.00	0.01	0.99	0.00	0.01	1.00	0.00	0.00
chr16	92644323	516	G	0	0.99	0.00	0.00	0.99	0.00	0.00	1.00	0.00	0.00	0.99	0.00	0.00	1.00	0.00	0.00	1.00	0.00	0.00
chr16	92644490	595	G	0	0.99	0.00	0.01	0.98	0.01	0.01	0.99	0.01	0.00	0.98	0.01	0.00	1.00	0.00	0.00	1.00	0.00	0.00
chr17	45706573	770	G	0	1.00	0.00	0.00	1.00	0.00	0.00	0.99	0.00	0.00	1.00	0.00	0.00	1.00	0.00	0.00	1.00	0.00	0.00

0.99	0.01	0.01	0.99	0.01	0.00	0,0002
0.99	0.01	0.01	0.99	0.00	0.01	0,0002
0.99	0.00	0.01	0.99	0.01	0.01	0,0002
0.99	0.00	0.01	0.99	0.01	0.01	0,0002
0.99	0.01	0.01	0.99	0.01	0.00	0,0002
0.99	0.01	0.00	0.99	0.01	0.00	0,0002
0.99	0.01	0.00	0.99	0.01	0.00	0,0002
0.99	0.01	0.01	0.99	0.01	0.00	0,0002
0.99	0.00	0.00	0.99	0.01	0.01	0,0002
0.99	0.00	0.01	0.99	0.00	0.01	0,0002
0.99	0.01	0.01	0.99	0.00	0.01	0,0002
0.99	0.01	0.01	0.99	0.01	0.00	0,0002
0.99	0.01	0.01	0.99	0.01	0.00	0,0002
0.99	0.01	0.00	0.99	0.00	0.01	0,0002
0.99	0.00	0.00	0.99	0.01	0.00	0,0002
0.99	0.01	0.00	0.99	0.01	0.00	0,0002
0.99	0.01	0.01	0.99	0.01	0.00	0,0002
0.99	0.01	0.01	0.99	0.00	0.01	0,0002
0.99	0.01	0.01	0.99	0.01	0.00	0,0002
0.99	0.01	0.00	0.99	0.00	0.00	0,0002
0.99	0.01	0.01	0.99	0.01	0.01	0,0002
0.99	0.01	0.01	0.99	0.01	0.00	0,0002
0.99	0.01	0.00	0.99	0.01	0.01	0,0002
0.99	0.00	0.01	0.99	0.00	0.01	0,0002
0.99	0.01	0.01	0.99	0.00	0.01	0,0002
0.99	0.00	0.01	0.99	0.00	0.01	0,0002
0.99	0.00	0.00	0.99	0.01	0.00	0,0002
0.99	0.01	0.00	0.99	0.00	0.01	0,0002
0.99	0.01	0.01	0.99	0.01	0.01	0,0002
0.99	0.01	0.00	0.99	0.00	0.01	0,0002
0.99	0.00	0.01	0.99	0.01	0.01	0,0002
0.99	0.00	0.01	0.99	0.00	0.01	0,0002
0.99	0.00	0.01	0.99	0.01	0.00	0,0002
0.99	0.01	0.01	0.99	0.00	0.00	0,0002
0.99	0.01	0.01	0.99	0.01	0.00	0,0002
0.99	0.01	0.00	0.99	0.00	0.01	0,0002
0.99	0.00	0.01	0.99	0.01	0.01	0,0002
0.99	0.01	0.01	0.99	0.00	0.01	0,0002
0.99	0.00	0.01	0.99	0.00	0.01	0,0002
0.99	0.00	0.01	0.99	0.00	0.01	0,0002
0.99	0.01	0.01	0.99	0.01	0.01	0,0002
0.99	0.01	0.01	0.99	0.00	0.01	0,0002
0.99	0.00	0.01	0.99	0.00	0.01	0,0002
0.99	0.01	0.01	0.99	0.00	0.01	0,0002
0.99	0.01	0.01	0.99	0.01	0.01	0,0002
0.99	0.01	0.00	0.99	0.01	0.00	0,0002

chr5	64987524	1499	C	0	0.99	0.00	0.00	0.99	0.00	0.00	0.99	0.00	0.00	1.00	0.00	0.00	1.00	0.00	0.00	1.00	0.00	0.00
chrX	7539536	2354	G	0	1.00	0.00	0.00	1.00	0.00	0.00	1.00	0.00	0.00	0.99	0.01	0.00	1.00	0.00	0.00	1.00	0.00	0.00
chr15	61868841	292	G	0	1.00	0.00	0.00	1.00	0.00	0.00	1.00	0.00	0.00	1.00	0.00	0.00	0.99	0.00	0.00	1.00	0.00	0.00
chr19	24277409	882	c	0	1.00	0.00	0.00	1.00	0.00	0.00	1.00	0.00	0.00	1.00	0.00	0.00	1.00	0.00	0.00	1.00	0.00	0.00
chr4	139783622	1242	G	0	0.99	0.00	0.00	1.00	0.00	0.00	0.99	0.00	0.00	1.00	0.00	0.00	1.00	0.00	0.00	1.00	0.00	0.00
chr4	139783647	1256	G	0	1.00	0.00	0.00	1.00	0.00	0.00	0.99	0.01	0.00	0.99	0.00	0.00	1.00	0.00	0.00	0.99	0.00	0.00
chr5	64987512	1496	C	0	0.99	0.01	0.00	0.99	0.00	0.00	1.00	0.00	0.00	1.00	0.00	0.00	0.99	0.00	0.01	1.00	0.00	0.00
chr9	100498602	1970	C	0	1.00	0.00	0.00	1.00	0.00	0.00	1.00	0.00	0.00	1.00	0.00	0.00	1.00	0.00	0.00	1.00	0.00	0.00
chr9	100498638	1976	C	0	1.00	0.00	0.00	1.00	0.00	0.00	1.00	0.00	0.00	1.00	0.00	0.00	1.00	0.00	0.00	0.99	0.01	0.00
chrX	7476650	2179	C	1	1.00	0.00	0.00	1.00	0.00	0.00	0.99	0.00	0.00	0.99	0.01	0.00	0.99	0.00	0.01	0.98	0.02	0.00
chrX	7539600	2395	C	0	0.99	0.01	0.00	0.99	0.00	0.00	0.99	0.01	0.00	1.00	0.00	0.00	0.99	0.00	0.00	1.00	0.00	0.00
chr11	3525696	52	G	0	1.00	0.00	0.00	1.00	0.00	0.00	1.00	0.00	0.00	1.00	0.00	0.00	0.99	0.00	0.00	1.00	0.00	0.00
chr17	45706698	831	C	0	1.00	0.00	0.00	1.00	0.00	0.00	1.00	0.00	0.00	1.00	0.00	0.00	1.00	0.00	0.00	0.99	0.00	0.01
chr19	24277465	891	g	0	1.00	0.00	0.00	1.00	0.00	0.00	1.00	0.00	0.00	1.00	0.00	0.00	1.00	0.00	0.00	1.00	0.00	0.00
chr8	89376844	1590	c	0	1.00	0.00	0.00	0.99	0.00	0.00	1.00	0.00	0.00	1.00	0.00	0.00	1.00	0.00	0.00	1.00	0.00	0.00
chr8	89376962	1646	G	0	1.00	0.00	0.00	1.00	0.00	0.00	1.00	0.00	0.00	1.00	0.00	0.00	1.00	0.00	0.00	1.00	0.00	0.00
chr8	89377062	1687	G	0	0.99	0.01	0.00	1.00	0.00	0.00	0.99	0.00	0.00	0.99	0.01	0.00	0.99	0.01	0.00	1.00	0.00	0.00
chr15	100300020	362	G	0	0.99	0.01	0.00	1.00	0.00	0.00	1.00	0.00	0.00	1.00	0.00	0.00	0.98	0.02	0.00	1.00	0.00	0.00
chr16	92644469	578	G	0	0.99	0.00	0.00	0.99	0.00	0.00	0.99	0.00	0.01	0.99	0.00	0.00	1.00	0.00	0.00	1.00	0.00	0.00
chr19	24277514	917	g	0	1.00	0.00	0.00	1.00	0.00	0.00	0.99	0.00	0.00	0.99	0.00	0.01	1.00	0.00	0.00	1.00	0.00	0.00
chr5	64987464	1472	C	0	1.00	0.00	0.00	0.99	0.00	0.01	1.00	0.00	0.00	1.00	0.00	0.00	1.00	0.00	0.00	0.99	0.01	0.00
chr8	89376852	1596	c	0	1.00	0.00	0.00	1.00	0.00	0.00	0.99	0.00	0.00	0.98	0.02	0.00	0.99	0.00	0.00	0.99	0.00	0.01
chr9	100498423	1879	C	0	1.00	0.00	0.00	1.00	0.00	0.00	1.00	0.00	0.00	1.00	0.00	0.00	1.00	0.00	0.00	1.00	0.00	0.00
chr9	100498453	1895	G	0	1.00	0.00	0.00	1.00	0.00	0.00	1.00	0.00	0.00	1.00	0.00	0.00	0.99	0.01	0.00	1.00	0.00	0.00
chr15	100299998	353	C	0	0.99	0.00	0.00	0.99	0.00	0.00	0.99	0.00	0.01	0.99	0.00	0.00	1.00	0.00	0.00	1.00	0.00	0.00
chr15	100300056	381	G	0	1.00	0.00	0.00	1.00	0.00	0.00	1.00	0.00	0.00	0.99	0.00	0.00	1.00	0.00	0.00	1.00	0.00	0.00
chr16	92644422	549	G	0	0.99	0.01	0.00	0.99	0.00	0.00	1.00	0.00	0.00	0.99	0.00	0.00	1.00	0.00	0.00	1.00	0.00	0.00
chr16	92644519	616	C	0	1.00	0.00	0.00	0.99	0.00	0.01	0.99	0.00	0.00	0.99	0.00	0.01	0.99	0.00	0.00	1.00	0.00	0.00
chr17	45706718	841	C	0	1.00	0.00	0.00	1.00	0.00	0.00	1.00	0.00	0.00	0.99	0.00	0.00	0.99	0.00	0.00	1.00	0.00	0.00
chr11	3525816	119	G	0	1.00	0.00	0.00	1.00	0.00	0.00	1.00	0.00	0.00	0.99	0.00	0.00	1.00	0.00	0.00	0.99	0.00	0.00
chr16	92644337	521	G	0	0.99	0.00	0.00	0.99	0.01	0.01	1.00	0.00	0.00	0.99	0.00	0.00	1.00	0.00	0.00	1.00	0.00	0.00
chr5	64987357	1425	G	0	1.00	0.00	0.00	0.99	0.00	0.00	1.00	0.00	0.00	0.99	0.00	0.00	1.00	0.00	0.00	1.00	0.00	0.00
chr8	89376966	1649	C	0	1.00	0.00	0.00	1.00	0.00	0.00	1.00	0.00	0.00	1.00	0.00	0.00	1.00	0.00	0.00	1.00	0.00	0.00
chr8	120115643	1735	C	0	0.98	0.00	0.01	0.99	0.00	0.00	1.00	0.00	0.00	1.00	0.00	0.00	1.00	0.00	0.00	1.00	0.00	0.00
chr8	120115654	1740	G	0	1.00	0.00	0.00	1.00	0.00	0.00	1.00	0.00	0.00	1.00	0.00	0.00	1.00	0.00	0.00	1.00	0.00	0.00
chr9	100498610	1972	C	0	0.99	0.00	0.01	1.00	0.00	0.00	1.00	0.00	0.00	0.99	0.00	0.01	1.00	0.00	0.00	0.98	0.01	0.00
chrX	7476592	2140	C	0	1.00	0.00	0.00	1.00	0.00	0.00	1.00	0.00	0.00	1.00	0.00	0.00	0.98	0.01	0.02	1.00	0.00	0.00
chr15	61868830	288	C	0	0.99	0.01	0.00	1.00	0.00	0.00	1.00	0.00	0.00	0.99	0.00	0.00	1.00	0.00	0.00	1.00	0.00	0.00
chr4	136547751	1208	C	0	1.00	0.00	0.00	0.99	0.00	0.00	1.00	0.00	0.00	1.00	0.00	0.00	0.99	0.00	0.00	1.00	0.00	0.00
chr5	64987534	1504	C	0	1.00	0.00	0.00	0.99	0.00	0.00	0.99	0.00	0.00	0.99	0.00	0.00	1.00	0.00	0.00	1.00	0.00	0.00
chr8	89376948	1641	C	0	1.00	0.00	0.00	1.00	0.00	0.00	1.00	0.00	0.00	1.00	0.00	0.00	1.00	0.00	0.00	1.00	0.00	0.00
chr9	100498493	1919	C	0	0.99	0.00	0.00	1.00	0.00	0.00	1.00	0.00	0.00	1.00	0.00	0.00	1.00	0.00	0.00	1.00	0.00	0.00
chr11	3525672	40	C	0	0.99	0.00	0.01	1.00	0.00	0.00	1.00	0.00	0.00	0.98	0.01	0.00	1.00	0.00	0.00	1.00	0.00	0.00
chr15	61868846	295	G	0	1.00	0.00	0.00	1.00	0.00	0.00	1.00	0.00	0.00	0.99	0.00	0.00	0.99	0.00	0.00	1.00	0.00	0.00
chr15	61868848	297	C	0	1.00	0.00	0.00	1.00	0.00	0.00	1.00	0.00	0.00	1.00	0.00	0.00	1.00	0.00	0.00	1.00	0.00	0.00
chr17	45706585	779	G	0	1.00	0.00	0.00	1.00	0.00	0.00	1.00	0.00	0.00	1.00	0.00	0.00	1.00	0.00	0.00	1.00	0.00	0.00

0.99	0.01	0.01	0.99	0.00	0.00	0,0002
0.99	0.00	0.01	0.99	0.01	0.00	0,0002
0.99	0.00	0.01	0.99	0.00	0.01	0,0002
0.99	0.00	0.01	0.99	0.00	0.01	0,0002
0.99	0.01	0.00	0.99	0.01	0.00	0,0002
0.99	0.01	0.00	0.99	0.00	0.01	0,0002
0.99	0.01	0.01	0.99	0.00	0.01	0,0002
0.99	0.00	0.00	0.99	0.01	0.01	0,0002
0.99	0.00	0.00	0.99	0.01	0.01	0,0002
0.99	0.01	0.00	0.99	0.01	0.00	0,0002
0.99	0.01	0.00	0.99	0.00	0.01	0,0002
0.99	0.00	0.01	0.99	0.01	0.01	0,0002
0.99	0.00	0.01	0.99	0.01	0.01	0,0002
0.99	0.00	0.00	0.98	0.01	0.01	0,0002
0.99	0.01	0.00	0.99	0.00	0.01	0,0002
0.99	0.00	0.00	0.99	0.01	0.00	0,0002
0.99	0.01	0.00	0.99	0.01	0.01	0,0002
0.99	0.01	0.01	0.99	0.01	0.01	0,00019
0.99	0.01	0.01	0.99	0.00	0.01	0,00019
0.99	0.01	0.00	0.99	0.01	0.01	0,00019
0.99	0.00	0.01	0.99	0.01	0.01	0,00019
0.99	0.01	0.01	0.99	0.01	0.00	0,00019
0.99	0.01	0.00	0.99	0.00	0.01	0,00019
0.99	0.00	0.00	0.99	0.01	0.00	0,00019
0.99	0.01	0.01	0.99	0.01	0.00	0,00019
0.99	0.01	0.00	0.99	0.01	0.01	0,00019
0.99	0.00	0.01	0.99	0.01	0.01	0,00019
0.99	0.00	0.01	0.99	0.01	0.01	0,00019
0.99	0.01	0.00	0.99	0.01	0.00	0,00019
0.99	0.01	0.00	0.99	0.01	0.00	0,00019
0.99	0.01	0.01	0.99	0.01	0.00	0,00019
0.99	0.00	0.01	0.99	0.01	0.01	0,00019
0.99	0.01	0.01	0.99	0.01	0.01	0,00019
0.99	0.00	0.01	0.99	0.00	0.00	0,00019
0.99	0.01	0.00	0.99	0.01	0.01	0,00019
0.99	0.00	0.01	0.99	0.01	0.01	0,00019
0.99	0.01	0.00	0.99	0.01	0.00	0,00019
0.99	0.01	0.01	0.99	0.01	0.01	0,00019
0.99	0.01	0.01	0.99	0.01	0.01	0,00019
0.99	0.00	0.00	0.99	0.01	0.01	0,00019
0.99	0.00	0.01	0.99	0.01	0.01	0,00019
0.99	0.00	0.01	0.99	0.01	0.01	0,00019
0.99	0.01	0.00	0.99	0.01	0.00	0,00019
0.99	0.01	0.00	0.99	0.01	0.01	0,00019
0.99	0.00	0.01	0.99	0.01	0.01	0,00019
0.99	0.01	0.00	0.99	0.00	0.00	0,00019

chr19	24277489	906	c	0	0.99	0.01	0.00	1.00	0.00	0.00	1.00	0.00	0.00	1.00	0.00	0.00	1.00	0.00	0.00	1.00	0.00	0.00
chr8	89376820	1578	c	0	1.00	0.00	0.00	1.00	0.00	0.00	0.99	0.00	0.00	1.00	0.00	0.00	1.00	0.00	0.00	0.99	0.00	0.00
chr8	89377012	1666	G	0	1.00	0.00	0.00	0.99	0.00	0.00	0.99	0.00	0.00	1.00	0.00	0.00	0.99	0.00	0.00	0.99	0.01	0.00
chr9	100498503	1925	C	0	1.00	0.00	0.00	1.00	0.00	0.00	1.00	0.00	0.00	1.00	0.00	0.00	1.00	0.00	0.00	1.00	0.00	0.00
chrX	7476419	2033	G	1	1.00	0.00	0.00	1.00	0.00	0.00	0.99	0.01	0.00	1.00	0.00	0.00	1.00	0.00	0.00	0.99	0.00	0.00
chrX	7476630	2165	C	1	1.00	0.00	0.00	0.99	0.01	0.00	1.00	0.00	0.00	0.99	0.00	0.00	0.99	0.00	0.01	0.99	0.01	0.00
chrX	7476651	2180	G	1	1.00	0.00	0.00	1.00	0.00	0.00	1.00	0.00	0.00	0.99	0.00	0.01	1.00	0.00	0.00	0.99	0.00	0.01
chrX	7539379	2260	G	0	0.99	0.00	0.00	0.99	0.00	0.01	1.00	0.00	0.00	1.00	0.00	0.00	1.00	0.00	0.00	1.00	0.00	0.00
chrX	7539552	2365	C	0	1.00	0.00	0.00	1.00	0.00	0.00	0.99	0.01	0.00	1.00	0.00	0.00	1.00	0.00	0.00	1.00	0.00	0.00
chr11	3525872	158	C	0	0.99	0.00	0.01	0.99	0.01	0.00	0.99	0.01	0.00	0.99	0.00	0.01	0.99	0.01	0.00	0.99	0.00	0.01
chr15	61868747	247	G	0	1.00	0.00	0.00	1.00	0.00	0.00	1.00	0.00	0.00	0.99	0.00	0.00	1.00	0.00	0.00	0.99	0.00	0.00
chr16	92644432	554	C	0	1.00	0.00	0.00	0.99	0.00	0.01	0.99	0.00	0.00	0.99	0.00	0.00	0.99	0.00	0.00	1.00	0.00	0.00
chr17	45706439	701	C	0	1.00	0.00	0.00	1.00	0.00	0.00	1.00	0.00	0.00	0.99	0.00	0.00	1.00	0.00	0.00	0.99	0.00	0.00
chr19	24277641	978	C	0	1.00	0.00	0.00	0.99	0.00	0.00	1.00	0.00	0.00	0.99	0.00	0.00	0.99	0.01	0.00	1.00	0.00	0.00
chr4	139783676	1273	C	0	1.00	0.00	0.00	1.00	0.00	0.00	0.99	0.00	0.00	0.99	0.00	0.01	1.00	0.00	0.00	1.00	0.00	0.00
chr8	89376898	1625	c	0	1.00	0.00	0.00	0.99	0.00	0.00	1.00	0.00	0.00	1.00	0.00	0.00	1.00	0.00	0.00	0.99	0.01	0.00
chr8	89376917	1632	C	0	1.00	0.00	0.00	1.00	0.00	0.00	1.00	0.00	0.00	0.99	0.00	0.00	1.00	0.00	0.00	0.99	0.00	0.00
chr8	120115814	1816	G	0	1.00	0.00	0.00	1.00	0.00	0.00	0.99	0.00	0.00	1.00	0.00	0.00	1.00	0.00	0.00	1.00	0.00	0.00
chr9	100498353	1846	G	0	0.99	0.00	0.00	0.99	0.00	0.01	0.99	0.00	0.00	0.99	0.01	0.00	1.00	0.00	0.00	1.00	0.00	0.00
chrX	7476619	2157	G	1	1.00	0.00	0.00	1.00	0.00	0.00	1.00	0.00	0.00	0.99	0.00	0.01	1.00	0.00	0.00	0.99	0.00	0.01
chr11	3525693	50	C	0	1.00	0.00	0.00	1.00	0.00	0.00	0.99	0.01	0.00	0.99	0.01	0.00	0.99	0.00	0.01	0.99	0.00	0.01
chr15	61868722	233	G	0	0.99	0.00	0.00	1.00	0.00	0.00	1.00	0.00	0.00	1.00	0.00	0.00	1.00	0.00	0.00	0.99	0.00	0.01
chr15	61868804	273	G	0	1.00	0.00	0.00	1.00	0.00	0.00	0.99	0.00	0.00	1.00	0.00	0.00	1.00	0.00	0.00	1.00	0.00	0.00
chr15	61868816	280	C	0	1.00	0.00	0.00	1.00	0.00	0.00	1.00	0.00	0.00	1.00	0.00	0.00	1.00	0.00	0.00	1.00	0.00	0.00
chr15	100300125	413	G	0	1.00	0.00	0.00	1.00	0.00	0.00	1.00	0.00	0.00	1.00	0.00	0.00	1.00	0.00	0.00	1.00	0.00	0.00
chr16	92644448	562	G	0	1.00	0.00	0.00	0.99	0.00	0.01	1.00	0.00	0.00	0.99	0.01	0.01	1.00	0.00	0.00	1.00	0.00	0.00
chr17	45706475	715	C	0	1.00	0.00	0.00	1.00	0.00	0.00	1.00	0.00	0.00	1.00	0.00	0.00	1.00	0.00	0.00	1.00	0.00	0.00
chr17	45706757	860	G	0	1.00	0.00	0.00	0.99	0.00	0.00	0.99	0.01	0.00	1.00	0.00	0.00	1.00	0.00	0.00	1.00	0.00	0.00
chr4	136547508	1111	C	0	0.99	0.00	0.00	0.99	0.01	0.00	1.00	0.00	0.00	0.99	0.00	0.00	1.00	0.00	0.00	1.00	0.00	0.00
chr8	89376873	1610	c	0	0.99	0.00	0.00	1.00	0.00	0.00	1.00	0.00	0.00	0.99	0.01	0.00	0.99	0.00	0.00	1.00	0.00	0.00
chr9	100498431	1884	G	0	1.00	0.00	0.00	1.00	0.00	0.00	1.00	0.00	0.00	1.00	0.00	0.00	1.00	0.00	0.00	1.00	0.00	0.00
chr9	100498687	1989	c	0	1.00	0.00	0.00	1.00	0.00	0.00	0.99	0.00	0.00	0.99	0.01	0.00	0.98	0.02	0.00	1.00	0.00	0.00
chr15	100300008	357	C	0	1.00	0.00	0.00	0.99	0.00	0.00	0.99	0.00	0.00	0.99	0.00	0.00	1.00	0.00	0.00	1.00	0.00	0.00
chr15	100300425	504	C	0	1.00	0.00	0.00	0.99	0.00	0.00	0.99	0.00	0.01	0.99	0.00	0.00	1.00	0.00	0.00	1.00	0.00	0.00
chr4	139783613	1238	c	0	1.00	0.00	0.00	0.99	0.00	0.01	1.00	0.00	0.00	0.99	0.01	0.00	0.99	0.00	0.01	1.00	0.00	0.00
chr9	100498394	1865	G	0	1.00	0.00	0.00	0.99	0.00	0.00	0.99	0.00	0.00	1.00	0.00	0.00	1.00	0.00	0.00	0.99	0.01	0.00
chrX	7539290	2221	G	0	0.99	0.00	0.00	0.99	0.00	0.00	1.00	0.00	0.00	1.00	0.00	0.00	0.99	0.00	0.00	1.00	0.00	0.00
chrX	7539621	2406	G	0	1.00	0.00	0.00	0.99	0.00	0.01	1.00	0.00	0.00	1.00	0.00	0.00	1.00	0.00	0.00	1.00	0.00	0.00
chr11	3525663	34	G	0	0.99	0.00	0.01	0.99	0.00	0.00	0.99	0.00	0.00	0.99	0.00	0.01	0.99	0.00	0.00	1.00	0.00	0.00
chr17	45706706	833	G	0	1.00	0.00	0.00	1.00	0.00	0.00	1.00	0.00	0.00	1.00	0.00	0.00	1.00	0.00	0.00	1.00	0.00	0.00
chr4	136547453	1078	C	0	1.00	0.00	0.00	1.00	0.00	0.00	0.99	0.00	0.00	1.00	0.00	0.00	0.99	0.00	0.00	0.99	0.00	0.01
chr4	139783849	1365	G	0	1.00	0.00	0.00	1.00	0.00	0.00	0.99	0.00	0.00	1.00	0.00	0.00	1.00	0.00	0.00	1.00	0.00	0.00
chr8	89376990	1659	G	0	1.00	0.00	0.00	1.00	0.00	0.00	0.99	0.00	0.00	0.99	0.00	0.00	1.00	0.00	0.00	0.99	0.00	0.00
chr9	100498606	1971	C	0	1.00	0.00	0.00	1.00	0.00	0.00	1.00	0.00	0.00	1.00	0.00	0.00	0.98	0.02	0.00	1.00	0.00	0.00
chrX	7539638	2416	C	0	0.99	0.00	0.01	1.00	0.00	0.00	1.00	0.00	0.00	1.00	0.00	0.00	1.00	0.00	0.00	1.00	0.00	0.00
chr11	3525689	48	G	0	1.00	0.00	0.00	1.00	0.00	0.00	1.00	0.00	0.00	0.99	0.01	0.00	1.00	0.00	0.00	1.00	0.00	0.00

0.99	0.01	0.01	0.99	0.01	0.01	0,00019
0.99	0.00	0.01	0.99	0.00	0.01	0,00019
0.99	0.01	0.00	0.99	0.01	0.00	0,00019
0.99	0.01	0.01	0.99	0.00	0.00	0,00019
0.99	0.01	0.00	0.99	0.00	0.01	0,00019
0.99	0.01	0.00	0.99	0.01	0.01	0,00019
0.99	0.00	0.01	0.99	0.01	0.01	0,00019
0.99	0.01	0.01	0.99	0.00	0.00	0,00019
0.99	0.00	0.01	0.99	0.01	0.00	0,00019
0.99	0.01	0.00	0.99	0.00	0.01	0,00019
0.99	0.01	0.01	0.99	0.01	0.01	0,00019
0.99	0.01	0.01	0.99	0.00	0.01	0,00019
0.99	0.01	0.01	0.99	0.01	0.01	0,00019
0.99	0.00	0.00	0.99	0.01	0.01	0,00019
0.99	0.00	0.01	0.99	0.00	0.01	0,00019
0.99	0.01	0.01	0.99	0.01	0.00	0,00019
0.99	0.00	0.00	0.99	0.01	0.01	0,00019
0.99	0.00	0.01	0.99	0.01	0.00	0,00019
0.99	0.01	0.01	0.99	0.00	0.00	0,00019
0.99	0.00	0.00	0.99	0.01	0.01	0,00019
0.99	0.01	0.01	0.99	0.00	0.01	0,00019
0.99	0.01	0.00	0.99	0.01	0.00	0,00019
0.99	0.00	0.01	0.99	0.00	0.00	0,00019
0.99	0.00	0.01	0.99	0.01	0.01	0,00019
0.99	0.00	0.01	0.99	0.01	0.00	0,00019
0.99	0.01	0.01	0.99	0.01	0.00	0,00019
0.99	0.01	0.00	0.99	0.00	0.01	0,00019
0.99	0.01	0.00	0.99	0.00	0.01	0,00019
0.99	0.01	0.01	0.99	0.00	0.01	0,00019
0.99	0.00	0.00	0.99	0.01	0.01	0,00019
0.99	0.00	0.00	0.99	0.01	0.00	0,00019
0.99	0.00	0.00	0.99	0.01	0.00	0,00019
0.99	0.01	0.01	0.99	0.00	0.01	0,00019
0.99	0.00	0.01	0.99	0.00	0.00	0,00019
0.99	0.00	0.00	0.99	0.01	0.01	0,00019
0.99	0.01	0.01	0.99	0.00	0.01	0,00019
0.99	0.01	0.01	0.99	0.00	0.01	0,00019
0.99	0.00	0.01	0.99	0.00	0.00	0,00019
0.99	0.00	0.01	0.99	0.00	0.01	0,00019
0.99	0.01	0.01	0.99	0.01	0.00	0,00019
0.99	0.00	0.01	0.99	0.00	0.01	0,00019
0.99	0.01	0.01	0.99	0.01	0.00	0,00019
0.99	0.01	0.01	0.99	0.01	0.01	0,00019
0.99	0.00	0.00	0.99	0.01	0.01	0,00019
0.99	0.01	0.01	0.99	0.00	0.00	0,00019
0.99	0.01	0.01	0.99	0.01	0.00	0,00019

chr11	3525828	129	C	0	1.00	0.00	0.00	1.00	0.00	0.00	1.00	0.00	0.00	1.00	0.00	0.00	1.00	0.00	0.00	1.00	0.00	0.00
chr15	61868829	287	C	0	1.00	0.00	0.00	1.00	0.00	0.00	1.00	0.00	0.00	1.00	0.00	0.00	1.00	0.00	0.00	1.00	0.00	0.00
chr4	139783660	1264	C	0	1.00	0.00	0.00	1.00	0.00	0.00	1.00	0.00	0.00	1.00	0.00	0.00	0.99	0.00	0.01	0.99	0.00	0.01
chr4	139783685	1279	C	0	0.99	0.00	0.01	1.00	0.00	0.00	1.00	0.00	0.00	1.00	0.00	0.00	0.99	0.00	0.00	1.00	0.00	0.00
chr4	139783698	1285	C	0	0.99	0.01	0.00	1.00	0.00	0.00	1.00	0.00	0.00	0.99	0.00	0.00	1.00	0.00	0.00	1.00	0.00	0.00
chr4	139783772	1324	G	0	1.00	0.00	0.00	1.00	0.00	0.00	1.00	0.00	0.00	1.00	0.00	0.00	1.00	0.00	0.00	1.00	0.00	0.00
chr5	64987350	1423	C	0	1.00	0.00	0.00	1.00	0.00	0.00	1.00	0.00	0.00	0.99	0.00	0.00	0.99	0.00	0.00	1.00	0.00	0.00
chr9	100498442	1889	C	0	1.00	0.00	0.00	1.00	0.00	0.00	0.99	0.01	0.00	0.99	0.00	0.01	0.99	0.00	0.01	0.99	0.01	0.00
chrX	7539416	2281	C	0	1.00	0.00	0.00	0.99	0.01	0.00	1.00	0.00	0.00	1.00	0.00	0.00	1.00	0.00	0.00	1.00	0.00	0.00
chr11	3525874	159	G	0	1.00	0.00	0.00	0.99	0.00	0.00	0.99	0.01	0.00	1.00	0.00	0.00	1.00	0.00	0.00	1.00	0.00	0.00
chr15	61868817	281	C	0	1.00	0.00	0.00	0.99	0.00	0.00	0.99	0.00	0.00	1.00	0.00	0.00	1.00	0.00	0.00	1.00	0.00	0.00
chr15	61868837	291	C	0	0.99	0.00	0.00	1.00	0.00	0.00	0.99	0.00	0.00	1.00	0.00	0.00	1.00	0.00	0.00	1.00	0.00	0.00
chr16	92644449	563	G	0	1.00	0.00	0.00	0.99	0.00	0.00	1.00	0.00	0.00	0.99	0.01	0.00	1.00	0.00	0.00	1.00	0.00	0.00
chr5	64987425	1452	G	0	0.99	0.01	0.00	0.99	0.00	0.00	1.00	0.00	0.00	1.00	0.00	0.00	1.00	0.00	0.00	1.00	0.00	0.00
chr9	100498679	1987	c	0	0.99	0.01	0.00	1.00	0.00	0.00	1.00	0.00	0.00	1.00	0.00	0.00	1.00	0.00	0.00	1.00	0.00	0.00
chrX	7476387	2023	G	1	1.00	0.00	0.00	1.00	0.00	0.00	1.00	0.00	0.00	0.98	0.00	0.02	1.00	0.00	0.00	0.99	0.00	0.01
chrX	7539404	2274	C	0	1.00	0.00	0.00	1.00	0.00	0.00	0.99	0.01	0.00	1.00	0.00	0.00	1.00	0.00	0.00	1.00	0.00	0.00
chr4	136547456	1081	G	0	1.00	0.00	0.00	0.99	0.01	0.00	0.99	0.01	0.00	0.99	0.01	0.00	0.99	0.00	0.01	0.99	0.01	0.00
chr4	136547746	1206	C	0	0.99	0.00	0.00	1.00	0.00	0.00	0.99	0.00	0.00	0.99	0.00	0.01	0.99	0.00	0.01	1.00	0.00	0.00
chr5	64987317	1406	G	0	1.00	0.00	0.00	1.00	0.00	0.00	1.00	0.00	0.00	1.00	0.00	0.00	1.00	0.00	0.00	1.00	0.00	0.00
chr8	89376850	1594	c	0	0.99	0.01	0.00	1.00	0.00	0.00	1.00	0.00	0.00	1.00	0.00	0.00	0.99	0.00	0.00	0.99	0.00	0.00
chr8	120115767	1794	G	0	1.00	0.00	0.00	1.00	0.00	0.00	0.99	0.00	0.00	0.99	0.01	0.00	1.00	0.00	0.00	1.00	0.00	0.00
chr9	100498433	1885	C	0	1.00	0.00	0.00	1.00	0.00	0.00	0.99	0.01	0.00	1.00	0.00	0.00	1.00	0.00	0.00	0.99	0.01	0.00
chr15	61868887	313	G	0	1.00	0.00	0.00	1.00	0.00	0.00	1.00	0.00	0.00	1.00	0.00	0.00	1.00	0.00	0.00	1.00	0.00	0.00
chr16	92644373	532	G	0	0.99	0.00	0.00	1.00	0.00	0.00	1.00	0.00	0.00	0.99	0.00	0.00	1.00	0.00	0.00	1.00	0.00	0.00
chr4	139783771	1323	G	0	1.00	0.00	0.00	1.00	0.00	0.00	1.00	0.00	0.00	1.00	0.00	0.00	1.00	0.00	0.00	1.00	0.00	0.00
chr8	89376839	1587	c	0	1.00	0.00	0.00	1.00	0.00	0.00	1.00	0.00	0.00	0.99	0.01	0.00	0.99	0.00	0.00	1.00	0.00	0.00
chr8	89376867	1605	c	0	1.00	0.00	0.00	1.00	0.00	0.00	0.99	0.00	0.01	1.00	0.00	0.00	1.00	0.00	0.00	1.00	0.00	0.00
chr8	89377030	1672	G	0	1.00	0.00	0.00	0.99	0.00	0.00	0.99	0.00	0.00	1.00	0.00	0.00	1.00	0.00	0.00	1.00	0.00	0.00
chr9	100498478	1910	C	0	1.00	0.00	0.00	1.00	0.00	0.00	1.00	0.00	0.00	1.00	0.00	0.00	0.99	0.00	0.00	1.00	0.00	0.00
chrX	7476307	2006	G	0	1.00	0.00	0.00	1.00	0.00	0.00	1.00	0.00	0.00	0.99	0.00	0.00	0.99	0.00	0.00	1.00	0.00	0.00
chrX	7476618	2156	C	1	1.00	0.00	0.00	0.99	0.01	0.00	1.00	0.00	0.00	0.99	0.01	0.00	0.99	0.00	0.01	0.98	0.01	0.00
chrX	7539389	2265	G	0	0.99	0.00	0.00	1.00	0.00	0.00	0.99	0.00	0.00	1.00	0.00	0.00	1.00	0.00	0.00	1.00	0.00	0.00
chr11	3525612	12	G	0	1.00	0.00	0.00	0.99	0.00	0.00	1.00	0.00	0.00	0.99	0.00	0.00	0.99	0.00	0.00	1.00	0.00	0.00
chr11	3525666	36	C	0	1.00	0.00	0.00	1.00	0.00	0.00	0.99	0.00	0.00	1.00	0.00	0.00	1.00	0.00	0.00	1.00	0.00	0.00
chr15	61868844	294	G	0	1.00	0.00	0.00	1.00	0.00	0.00	0.99	0.00	0.00	1.00	0.00	0.00	0.99	0.00	0.00	1.00	0.00	0.00
chr15	61868878	307	C	0	0.99	0.00	0.00	1.00	0.00	0.00	1.00	0.00	0.00	1.00	0.00	0.00	1.00	0.00	0.00	1.00	0.00	0.00
chr4	136547436	1067	G	0	1.00	0.00	0.00	1.00	0.00	0.00	1.00	0.00	0.00	0.99	0.01	0.00	0.99	0.00	0.00	1.00	0.00	0.00
chr4	139783625	1244	G	0	1.00	0.00	0.00	1.00	0.00	0.00	0.99	0.00	0.00	0.99	0.01	0.00	1.00	0.00	0.00	0.99	0.00	0.00
chr5	64987587	1527	G	0	1.00	0.00	0.00	1.00	0.00	0.00	1.00	0.00	0.00	1.00	0.00	0.00	1.00	0.00	0.00	0.99	0.00	0.00
chr8	89376829	1582	c	0	1.00	0.00	0.00	1.00	0.00	0.00	0.99	0.00	0.00	1.00	0.00	0.00	1.00	0.00	0.00	1.00	0.00	0.00
chr8	89377027	1670	C	0	0.99	0.01	0.00	1.00	0.00	0.00	1.00	0.00	0.00	1.00	0.00	0.00	1.00	0.00	0.00	1.00	0.00	0.00
chr8	120115589	1709	G	0	1.00	0.00	0.00	1.00	0.00	0.00	1.00	0.00	0.00	1.00	0.00	0.00	1.00	0.00	0.00	1.00	0.00	0.00
chr8	120115751	1786	G	0	1.00	0.00	0.00	1.00	0.00	0.00	0.99	0.01	0.00	0.99	0.00	0.01	0.99	0.00	0.00	0.99	0.01	0.00
chrX	7539312	2227	G	0	0.99	0.00	0.00	1.00	0.00	0.00	1.00	0.00	0.00	1.00	0.00	0.00	0.99	0.00	0.00	1.00	0.00	0.00
chrX	7539470	2317	C	0	1.00	0.00	0.00	1.00	0.00	0.00	0.99	0.00	0.00	1.00	0.00	0.00	1.00	0.00	0.00	1.00	0.00	0.00

0.99	0.00	0.01	0.99	0.01	0.00	0,00019
0.99	0.00	0.00	0.99	0.01	0.01	0,00019
0.99	0.00	0.01	0.99	0.00	0.01	0,00019
0.99	0.00	0.01	0.99	0.00	0.01	0,00019
0.99	0.01	0.00	0.99	0.01	0.01	0,00019
0.99	0.01	0.00	0.99	0.01	0.00	0,00019
0.99	0.00	0.00	0.99	0.01	0.01	0,00019
0.99	0.01	0.00	0.99	0.01	0.01	0,00019
0.99	0.01	0.01	0.99	0.00	0.00	0,00019
0.99	0.01	0.00	0.99	0.00	0.01	0,00018
0.99	0.01	0.00	0.99	0.01	0.01	0,00018
0.99	0.01	0.01	0.99	0.01	0.00	0,00018
0.99	0.01	0.00	0.99	0.00	0.01	0,00018
0.99	0.01	0.00	0.99	0.01	0.00	0,00018
0.99	0.01	0.01	0.99	0.00	0.01	0,00018
0.99	0.00	0.00	0.99	0.01	0.01	0,00018
0.99	0.01	0.00	0.99	0.00	0.00	0,00018
0.99	0.01	0.00	0.99	0.01	0.00	0,00018
0.99	0.00	0.01	0.99	0.00	0.01	0,00018
0.99	0.01	0.00	0.99	0.00	0.01	0,00018
0.99	0.01	0.00	0.99	0.01	0.01	0,00018
0.99	0.00	0.01	0.99	0.01	0.01	0,00018
0.99	0.01	0.00	0.99	0.00	0.01	0,00018
0.99	0.01	0.00	0.99	0.00	0.01	0,00018
0.99	0.01	0.01	0.99	0.01	0.00	0,00018
0.99	0.01	0.00	0.99	0.01	0.00	0,00018
0.99	0.00	0.00	0.99	0.01	0.01	0,00018
0.99	0.01	0.01	0.99	0.00	0.00	0,00018
0.99	0.01	0.00	0.99	0.00	0.00	0,00018
0.99	0.01	0.00	0.99	0.01	0.00	0,00018
0.99	0.00	0.00	0.99	0.01	0.01	0,00018
0.99	0.01	0.00	0.99	0.01	0.00	0,00018
0.99	0.01	0.00	0.99	0.00	0.00	0,00018
0.99	0.00	0.01	0.99	0.01	0.00	0,00018
0.99	0.01	0.00	0.99	0.01	0.00	0,00018
0.99	0.00	0.01	0.99	0.01	0.00	0,00018
0.99	0.01	0.00	0.99	0.01	0.00	0,00018
0.99	0.01	0.00	0.99	0.01	0.01	0,00018
0.99	0.01	0.00	0.99	0.01	0.00	0,00018
0.99	0.00	0.00	0.99	0.01	0.00	0,00018
0.99	0.00	0.01	0.99	0.00	0.00	0,00018
0.99	0.00	0.01	0.99	0.01	0.00	0,00018
0.99	0.01	0.00	0.99	0.00	0.01	0,00018
0.99	0.00	0.01	0.99	0.01	0.01	0,00018
0.99	0.01	0.00	0.99	0.01	0.00	0,00018
0.99	0.01	0.00	0.99	0.01	0.00	0,00018

chrX	7539508	2338	G	0	0.99	0.00	0.00	1.00	0.00	0.00	1.00	0.00	0.00	0.98	0.00	0.02	1.00	0.00	0.00	0.99	0.00	0.01
chr15	61868663	206	C	0	0.99	0.01	0.00	1.00	0.00	0.00	1.00	0.00	0.00	1.00	0.00	0.00	1.00	0.00	0.00	0.99	0.00	0.00
chr15	100300220	446	G	0	1.00	0.00	0.00	1.00	0.00	0.00	0.99	0.00	0.00	1.00	0.00	0.00	1.00	0.00	0.00	1.00	0.00	0.00
chr17	45706414	698	C	0	1.00	0.00	0.00	1.00	0.00	0.00	1.00	0.00	0.00	1.00	0.00	0.00	1.00	0.00	0.00	0.99	0.00	0.01
chr4	139783705	1290	G	0	1.00	0.00	0.00	1.00	0.00	0.00	1.00	0.00	0.00	1.00	0.00	0.00	1.00	0.00	0.00	0.99	0.00	0.00
chr5	64987339	1416	G	0	1.00	0.00	0.00	1.00	0.00	0.00	0.99	0.00	0.00	1.00	0.00	0.00	0.99	0.00	0.00	1.00	0.00	0.00
chr5	64987393	1440	G	0	1.00	0.00	0.00	1.00	0.00	0.00	0.99	0.00	0.00	1.00	0.00	0.00	1.00	0.00	0.00	1.00	0.00	0.00
chr8	120115582	1706	G	0	1.00	0.00	0.00	1.00	0.00	0.00	1.00	0.00	0.00	0.99	0.01	0.00	1.00	0.00	0.00	1.00	0.00	0.00
chr9	100498354	1847	G	0	1.00	0.00	0.00	1.00	0.00	0.00	1.00	0.00	0.00	1.00	0.00	0.00	1.00	0.00	0.00	1.00	0.00	0.00
chr9	100498594	1969	C	0	1.00	0.00	0.00	1.00	0.00	0.00	1.00	0.00	0.00	0.99	0.00	0.01	0.99	0.00	0.01	1.00	0.00	0.00
chrX	7476383	2022	G	0	1.00	0.00	0.00	1.00	0.00	0.00	1.00	0.00	0.00	0.99	0.00	0.01	1.00	0.00	0.00	1.00	0.00	0.00
chrX	7476516	2084	C	1	1.00	0.00	0.00	1.00	0.00	0.00	1.00	0.00	0.00	0.99	0.01	0.00	0.96	0.01	0.03	0.99	0.01	0.00
chrX	7539357	2246	G	0	1.00	0.00	0.00	1.00	0.00	0.00	0.99	0.01	0.00	0.99	0.00	0.00	1.00	0.00	0.00	1.00	0.00	0.00
chrX	7539361	2249	G	0	1.00	0.00	0.00	1.00	0.00	0.00	1.00	0.00	0.00	0.99	0.00	0.01	1.00	0.00	0.00	1.00	0.00	0.00
chr16	92644354	527	G	0	1.00	0.00	0.00	0.99	0.00	0.00	1.00	0.00	0.00	1.00	0.00	0.00	1.00	0.00	0.00	1.00	0.00	0.00
chr4	139783653	1260	G	0	1.00	0.00	0.00	1.00	0.00	0.00	1.00	0.00	0.00	1.00	0.00	0.00	1.00	0.00	0.00	1.00	0.00	0.00
chr8	89376855	1597	c	0	1.00	0.00	0.00	1.00	0.00	0.00	1.00	0.00	0.00	0.99	0.00	0.01	0.99	0.01	0.00	1.00	0.00	0.00
chrX	7539372	2257	G	0	0.99	0.01	0.00	1.00	0.00	0.00	0.99	0.00	0.01	1.00	0.00	0.00	1.00	0.00	0.00	1.00	0.00	0.00
chrX	7539399	2270	C	0	0.99	0.00	0.01	1.00	0.00	0.00	1.00	0.00	0.00	1.00	0.00	0.00	0.99	0.00	0.00	0.99	0.00	0.00
chrX	7539642	2419	C	0	1.00	0.00	0.00	1.00	0.00	0.00	0.99	0.01	0.00	1.00	0.00	0.00	1.00	0.00	0.00	1.00	0.00	0.00
chr15	100300126	414	C	0	0.99	0.00	0.00	0.99	0.00	0.00	0.99	0.00	0.01	0.99	0.01	0.01	1.00	0.00	0.00	1.00	0.00	0.00
chr16	92644470	579	G	0	0.99	0.00	0.00	1.00	0.00	0.00	1.00	0.00	0.00	1.00	0.00	0.00	1.00	0.00	0.00	1.00	0.00	0.00
chr17	45706664	821	C	0	1.00	0.00	0.00	1.00	0.00	0.00	0.99	0.00	0.00	0.99	0.00	0.00	0.99	0.00	0.01	0.99	0.00	0.01
chr4	136547441	1070	G	0	1.00	0.00	0.00	1.00	0.00	0.00	1.00	0.00	0.00	1.00	0.00	0.00	1.00	0.00	0.00	1.00	0.00	0.00
chr5	64987571	1523	G	0	1.00	0.00	0.00	0.99	0.00	0.00	1.00	0.00	0.00	1.00	0.00	0.00	0.99	0.00	0.01	0.99	0.00	0.00
chr8	89376808	1573	c	0	1.00	0.00	0.00	1.00	0.00	0.00	1.00	0.00	0.00	0.99	0.01	0.00	1.00	0.00	0.00	1.00	0.00	0.00
chr8	89377037	1673	G	0	1.00	0.00	0.00	1.00	0.00	0.00	1.00	0.00	0.00	1.00	0.00	0.00	0.99	0.00	0.01	1.00	0.00	0.00
chr9	100498472	1906	G	0	1.00	0.00	0.00	1.00	0.00	0.00	1.00	0.00	0.00	0.99	0.00	0.00	1.00	0.00	0.00	1.00	0.00	0.00
chrX	7476477	2057	C	1	1.00	0.00	0.00	1.00	0.00	0.00	1.00	0.00	0.00	1.00	0.00	0.00	0.99	0.00	0.01	0.99	0.01	0.00
chrX	7476583	2134	C	0	1.00	0.00	0.00	1.00	0.00	0.00	1.00	0.00	0.00	1.00	0.00	0.00	0.99	0.00	0.01	1.00	0.00	0.00
chrX	7476601	2145	G	0	1.00	0.00	0.00	1.00	0.00	0.00	1.00	0.00	0.00	1.00	0.00	0.00	1.00	0.00	0.00	1.00	0.00	0.00
chrX	7476628	2163	C	1	1.00	0.00	0.00	1.00	0.00	0.00	0.99	0.00	0.00	1.00	0.00	0.00	0.99	0.00	0.01	0.99	0.01	0.00
chrX	7476668	2194	G	0	1.00	0.00	0.00	0.99	0.01	0.00	1.00	0.00	0.00	1.00	0.00	0.00	1.00	0.00	0.00	1.00	0.00	0.00
chrX	7539548	2363	G	0	1.00	0.00	0.00	1.00	0.00	0.00	0.99	0.00	0.00	1.00	0.00	0.00	1.00	0.00	0.00	1.00	0.00	0.00
chr11	3525789	105	C	0	1.00	0.00	0.00	1.00	0.00	0.00	1.00	0.00	0.00	1.00	0.00	0.00	1.00	0.00	0.00	1.00	0.00	0.00
chr15	61868863	302	G	0	1.00	0.00	0.00	1.00	0.00	0.00	0.99	0.00	0.00	0.99	0.00	0.00	1.00	0.00	0.00	0.99	0.00	0.00
chr15	100300419	500	C	0	1.00	0.00	0.00	0.99	0.00	0.00	0.99	0.01	0.01	0.99	0.00	0.00	1.00	0.00	0.00	1.00	0.00	0.00
chr19	24277485	902	c	0	1.00	0.00	0.00	0.99	0.00	0.00	0.99	0.01	0.00	0.99	0.00	0.00	1.00	0.00	0.00	1.00	0.00	0.00
chr19	24277513	916	g	0	0.99	0.01	0.00	1.00	0.00	0.00	1.00	0.00	0.00	1.00	0.00	0.00	1.00	0.00	0.00	0.99	0.00	0.00
chr19	24277573	948	g	0	0.99	0.01	0.00	1.00	0.00	0.00	1.00	0.00	0.00	0.99	0.01	0.00	0.99	0.01	0.00	1.00	0.00	0.00
chr4	136547344	1026	G	0	0.99	0.00	0.00	1.00	0.00	0.00	1.00	0.00	0.00	1.00	0.00	0.00	0.99	0.00	0.00	1.00	0.00	0.00
chrX	7539449	2303	C	0	0.99	0.01	0.00	0.99	0.01	0.00	1.00	0.00	0.00	1.00	0.00	0.00	1.00	0.00	0.00	1.00	0.00	0.00
chr11	3525628	19	G	0	0.99	0.00	0.01	1.00	0.00	0.00	1.00	0.00	0.00	0.99	0.00	0.01	1.00	0.00	0.00	1.00	0.00	0.00
chr15	61868729	237	G	0	1.00	0.00	0.00	1.00	0.00	0.00	0.99	0.00	0.00	1.00	0.00	0.00	1.00	0.00	0.00	1.00	0.00	0.00
chr15	61868777	264	G	0	1.00	0.00	0.00	1.00	0.00	0.00	0.99	0.00	0.00	1.00	0.00	0.00	1.00	0.00	0.00	0.99	0.00	0.00
chr15	100300034	370	G	0	1.00	0.00	0.00	1.00	0.00	0.00	0.99	0.01	0.00	0.99	0.01	0.00	1.00	0.00	0.00	1.00	0.00	0.00

0.99	0.00	0.01	0.99	0.00	0.01	0,00018
0.99	0.01	0.00	0.99	0.01	0.00	0,00018
0.99	0.01	0.00	0.99	0.00	0.01	0,00018
0.99	0.01	0.00	0.99	0.01	0.00	0,00018
0.99	0.00	0.00	0.99	0.01	0.01	0,00018
0.99	0.01	0.00	0.99	0.00	0.01	0,00018
0.99	0.01	0.01	0.99	0.01	0.01	0,00018
0.99	0.00	0.00	0.99	0.01	0.00	0,00018
0.99	0.01	0.01	0.99	0.01	0.00	0,00018
0.99	0.00	0.01	0.99	0.00	0.01	0,00018
0.99	0.00	0.01	0.99	0.00	0.01	0,00018
0.99	0.00	0.00	0.99	0.01	0.00	0,00018
0.99	0.00	0.00	0.99	0.01	0.01	0,00018
0.99	0.00	0.01	0.99	0.01	0.00	0,00018
0.99	0.00	0.01	0.99	0.00	0.00	0,00018
0.99	0.01	0.00	0.99	0.01	0.00	0,00018
0.99	0.00	0.01	0.99	0.01	0.00	0,00018
0.99	0.01	0.01	0.99	0.01	0.00	0,00018
0.99	0.00	0.01	0.99	0.00	0.01	0,00018
0.99	0.01	0.00	0.99	0.01	0.00	0,00018
0.99	0.00	0.01	0.99	0.01	0.01	0,00018
0.99	0.01	0.00	0.99	0.01	0.00	0,00018
0.99	0.01	0.01	0.99	0.00	0.01	0,00018
0.99	0.00	0.00	0.99	0.01	0.01	0,00018
0.99	0.00	0.01	0.99	0.00	0.01	0,00018
0.99	0.01	0.00	0.99	0.01	0.00	0,00018
0.99	0.01	0.01	0.99	0.00	0.01	0,00018
0.99	0.01	0.00	0.99	0.01	0.00	0,00018
0.99	0.01	0.00	0.99	0.01	0.00	0,00018
0.99	0.01	0.00	0.99	0.01	0.00	0,00018
0.99	0.01	0.00	0.99	0.00	0.01	0,00018
0.99	0.01	0.00	0.99	0.01	0.00	0,00018
0.99	0.01	0.01	0.99	0.00	0.01	0,00018
0.99	0.01	0.01	0.99	0.00	0.01	0,00018
0.99	0.01	0.00	0.99	0.00	0.00	0,00018
0.99	0.00	0.00	0.99	0.01	0.01	0,00018
0.99	0.01	0.01	0.99	0.00	0.00	0,00018
0.99	0.01	0.00	0.99	0.01	0.00	0,00018
0.99	0.01	0.00	0.99	0.00	0.01	0,00018
0.99	0.01	0.00	0.99	0.01	0.01	0,00018
0.99	0.01	0.00	0.99	0.00	0.01	0,00018
0.99	0.01	0.00	0.99	0.01	0.00	0,00018
0.99	0.00	0.01	0.99	0.00	0.01	0,00018
0.99	0.01	0.00	0.99	0.01	0.00	0,00018
0.99	0.01	0.01	0.99	0.01	0.00	0,00018
0.99	0.01	0.00	0.99	0.01	0.00	0,00018

chr1!	100300435	506	C	0	1.00	0.00	0.00	0.99	0.00	0.00	0.99	0.00	0.01	0.99	0.00	0.00	1.00	0.00	0.00	1.00	0.00	0.00
chr1(	92644492	596	C	0	1.00	0.00	0.00	0.99	0.00	0.01	0.99	0.00	0.01	0.99	0.00	0.01	0.99	0.00	0.00	1.00	0.00	0.00
chr1;	45706503	732	C	0	1.00	0.00	0.00	1.00	0.00	0.00	0.99	0.00	0.00	1.00	0.00	0.00	1.00	0.00	0.00	0.98	0.02	0.00
chr1;	45706521	741	G	0	1.00	0.00	0.00	0.99	0.00	0.00	0.99	0.00	0.00	1.00	0.00	0.00	1.00	0.00	0.00	1.00	0.00	0.00
chr4	136547659	1185	C	0	1.00	0.00	0.00	1.00	0.00	0.00	1.00	0.00	0.00	0.99	0.00	0.00	1.00	0.00	0.00	1.00	0.00	0.00
chr4	139783841	1361	C	0	1.00	0.00	0.00	1.00	0.00	0.00	1.00	0.00	0.00	1.00	0.00	0.00	1.00	0.00	0.00	1.00	0.00	0.00
chr5	64987380	1434	C	0	1.00	0.00	0.00	1.00	0.00	0.00	0.99	0.00	0.00	0.99	0.00	0.00	1.00	0.00	0.00	1.00	0.00	0.00
chr5	64987558	1517	G	0	0.99	0.01	0.00	1.00	0.00	0.00	1.00	0.00	0.00	0.99	0.00	0.01	1.00	0.00	0.00	1.00	0.00	0.00
chr8	89376818	1576	g	0	1.00	0.00	0.00	1.00	0.00	0.00	0.99	0.00	0.00	1.00	0.00	0.00	1.00	0.00	0.00	1.00	0.00	0.00
chr9	100498529	1939	G	0	1.00	0.00	0.00	1.00	0.00	0.00	1.00	0.00	0.00	1.00	0.00	0.00	1.00	0.00	0.00	1.00	0.00	0.00
chrX	7476600	2144	G	1	1.00	0.00	0.00	1.00	0.00	0.00	1.00	0.00	0.00	0.98	0.00	0.02	1.00	0.00	0.00	0.99	0.01	0.00
chrX	7476614	2154	G	0	0.99	0.00	0.00	1.00	0.00	0.00	1.00	0.00	0.00	1.00	0.00	0.00	1.00	0.00	0.00	1.00	0.00	0.00
chr1'	3525846	138	C	0	1.00	0.00	0.00	1.00	0.00	0.00	1.00	0.00	0.00	1.00	0.00	0.00	1.00	0.00	0.00	1.00	0.00	0.00
chr1!	61868723	234	C	0	1.00	0.00	0.00	1.00	0.00	0.00	1.00	0.00	0.00	1.00	0.00	0.00	1.00	0.00	0.00	1.00	0.00	0.00
chr1!	100299993	350	C	0	0.99	0.00	0.00	0.99	0.00	0.01	0.99	0.00	0.00	0.99	0.00	0.00	1.00	0.00	0.00	1.00	0.00	0.00
chr1!	100300132	417	C	0	0.99	0.00	0.00	0.99	0.00	0.00	1.00	0.00	0.00	0.99	0.00	0.01	1.00	0.00	0.00	1.00	0.00	0.00
chr1(	24277481	901	c	0	1.00	0.00	0.00	1.00	0.00	0.00	0.99	0.00	0.00	1.00	0.00	0.00	1.00	0.00	0.00	1.00	0.00	0.00
chr4	136547381	1037	G	0	1.00	0.00	0.00	1.00	0.00	0.00	1.00	0.00	0.00	1.00	0.00	0.00	1.00	0.00	0.00	0.99	0.00	0.00
chr4	136547538	1129	G	0	0.99	0.00	0.00	1.00	0.00	0.00	1.00	0.00	0.00	1.00	0.00	0.00	1.00	0.00	0.00	1.00	0.00	0.00
chr4	139783764	1320	C	0	0.99	0.00	0.00	1.00	0.00	0.00	1.00	0.00	0.00	1.00	0.00	0.00	0.99	0.01	0.00	1.00	0.00	0.00
chr8	89376968	1651	C	0	1.00	0.00	0.00	1.00	0.00	0.00	0.99	0.00	0.00	0.99	0.00	0.01	0.99	0.01	0.00	1.00	0.00	0.00
chr9	100498474	1908	C	0	1.00	0.00	0.00	0.99	0.00	0.01	1.00	0.00	0.00	1.00	0.00	0.00	1.00	0.00	0.00	1.00	0.00	0.00
chr9	100498541	1944	G	0	1.00	0.00	0.00	1.00	0.00	0.00	1.00	0.00	0.00	0.99	0.01	0.00	0.99	0.01	0.00	1.00	0.00	0.00
chrX	7476549	2110	G	1	1.00	0.00	0.00	1.00	0.00	0.00	1.00	0.00	0.00	0.99	0.00	0.01	1.00	0.00	0.00	1.00	0.00	0.00
chrX	7539605	2397	G	0	1.00	0.00	0.00	1.00	0.00	0.00	1.00	0.00	0.00	1.00	0.00	0.00	1.00	0.00	0.00	1.00	0.00	0.00
chr1'	3525865	153	C	0	1.00	0.00	0.00	1.00	0.00	0.00	1.00	0.00	0.00	1.00	0.00	0.00	1.00	0.00	0.00	1.00	0.00	0.00
chr1!	61868644	198	C	0	1.00	0.00	0.00	1.00	0.00	0.00	1.00	0.00	0.00	1.00	0.00	0.00	1.00	0.00	0.00	1.00	0.00	0.00
chr1!	61868763	255	C	0	1.00	0.00	0.00	1.00	0.00	0.00	1.00	0.00	0.00	1.00	0.00	0.00	1.00	0.00	0.00	1.00	0.00	0.00
chr1!	100299992	349	C	0	0.99	0.00	0.00	0.99	0.00	0.00	0.99	0.00	0.00	0.99	0.00	0.01	1.00	0.00	0.00	1.00	0.00	0.00
chr1!	100300221	447	G	0	1.00	0.00	0.00	1.00	0.00	0.00	1.00	0.00	0.00	1.00	0.00	0.00	1.00	0.00	0.00	1.00	0.00	0.00
chr1!	100300320	475	C	0	1.00	0.00	0.00	0.99	0.00	0.00	1.00	0.00	0.00	0.99	0.00	0.00	1.00	0.00	0.00	1.00	0.00	0.00
chr1(	92644545	630	G	0	0.99	0.00	0.00	1.00	0.00	0.00	1.00	0.00	0.00	1.00	0.00	0.00	1.00	0.00	0.00	1.00	0.00	0.00
chr1;	45706683	826	C	0	1.00	0.00	0.00	1.00	0.00	0.00	0.99	0.00	0.00	0.99	0.00	0.00	0.99	0.00	0.00	1.00	0.00	0.00
chr4	136547460	1083	C	0	1.00	0.00	0.00	1.00	0.00	0.00	1.00	0.00	0.00	1.00	0.00	0.00	1.00	0.00	0.00	1.00	0.00	0.00
chr4	136547671	1187	C	0	1.00	0.00	0.00	1.00	0.00	0.00	0.99	0.00	0.01	0.99	0.00	0.00	1.00	0.00	0.00	1.00	0.00	0.00
chr5	64987383	1435	C	0	1.00	0.00	0.00	1.00	0.00	0.00	0.99	0.00	0.00	0.99	0.00	0.00	1.00	0.00	0.00	1.00	0.00	0.00
chr8	89376830	1583	c	0	1.00	0.00	0.00	1.00	0.00	0.00	0.99	0.00	0.00	1.00	0.00	0.00	1.00	0.00	0.00	1.00	0.00	0.00
chrX	7476644	2176	G	1	1.00	0.00	0.00	1.00	0.00	0.00	1.00	0.00	0.00	1.00	0.00	0.00	0.99	0.00	0.00	0.99	0.00	0.01
chrX	7539305	2225	G	0	1.00	0.00	0.00	1.00	0.00	0.00	1.00	0.00	0.00	1.00	0.00	0.00	1.00	0.00	0.00	1.00	0.00	0.00
chrX	7539348	2241	C	0	1.00	0.00	0.00	1.00	0.00	0.00	1.00	0.00	0.00	1.00	0.00	0.00	1.00	0.00	0.00	1.00	0.00	0.00
chrX	7539599	2394	C	0	0.99	0.00	0.01	1.00	0.00	0.00	1.00	0.00	0.00	0.99	0.00	0.00	1.00	0.00	0.00	1.00	0.00	0.00
chr1!	61868683	216	C	0	1.00	0.00	0.00	1.00	0.00	0.00	1.00	0.00	0.00	1.00	0.00	0.00	1.00	0.00	0.00	1.00	0.00	0.00
chr1!	61868746	246	G	0	1.00	0.00	0.00	1.00	0.00	0.00	0.99	0.00	0.00	1.00	0.00	0.00	1.00	0.00	0.00	1.00	0.00	0.00
chr1!	61868915	328	G	0	1.00	0.00	0.00	0.99	0.00	0.00	1.00	0.00	0.00	1.00	0.00	0.00	1.00	0.00	0.00	1.00	0.00	0.00
chr4	139783817	1348	C	0	1.00	0.00	0.00	1.00	0.00	0.00	1.00	0.00	0.00	1.00	0.00	0.00	1.00	0.00	0.00	1.00	0.00	0.00
chr8	89377051	1680	G	0	1.00	0.00	0.00	1.00	0.00	0.00	1.00	0.00	0.00	0.99	0.00	0.00	1.00	0.00	0.00	1.00	0.00	0.00

0.99	0.01	0.01	0.99	0.00	0.00	0,00018
0.99	0.00	0.01	0.99	0.00	0.01	0,00018
0.99	0.00	0.01	0.99	0.01	0.01	0,00018
0.99	0.00	0.01	0.99	0.01	0.00	0,00018
0.99	0.01	0.01	0.99	0.01	0.00	0,00018
0.99	0.01	0.00	0.99	0.01	0.00	0,00018
0.99	0.00	0.01	0.99	0.00	0.01	0,00018
0.99	0.01	0.00	0.99	0.00	0.00	0,00018
0.99	0.01	0.00	0.99	0.01	0.00	0,00018
0.99	0.00	0.00	0.99	0.00	0.00	0,00018
0.99	0.00	0.00	0.99	0.01	0.01	0,00018
0.99	0.01	0.00	0.99	0.01	0.00	0,00018
0.99	0.01	0.00	0.99	0.01	0.01	0,00018
0.99	0.01	0.01	0.99	0.00	0.00	0,00018
0.99	0.01	0.01	0.99	0.00	0.01	0,00018
0.99	0.00	0.01	0.99	0.00	0.00	0,00018
0.99	0.01	0.00	0.99	0.01	0.00	0,00018
0.99	0.00	0.01	0.99	0.01	0.01	0,00018
0.99	0.00	0.00	0.99	0.01	0.01	0,00018
0.99	0.00	0.01	0.99	0.01	0.00	0,00018
0.99	0.00	0.00	0.99	0.00	0.01	0,00018
0.99	0.00	0.01	0.99	0.01	0.00	0,00018
0.99	0.00	0.00	0.99	0.01	0.00	0,00018
0.99	0.00	0.00	0.99	0.01	0.01	0,00018
0.99	0.01	0.00	0.99	0.01	0.00	0,00018
0.99	0.00	0.00	0.99	0.00	0.00	0,00017
0.99	0.00	0.00	0.98	0.01	0.01	0,00017
0.99	0.00	0.01	0.99	0.00	0.00	0,00017
0.99	0.00	0.01	0.99	0.00	0.01	0,00017
0.99	0.01	0.01	0.99	0.00	0.01	0,00017
0.99	0.00	0.01	0.99	0.00	0.01	0,00017
0.99	0.01	0.01	0.99	0.01	0.00	0,00017
0.99	0.01	0.01	0.99	0.01	0.01	0,00017
0.99	0.01	0.00	0.99	0.01	0.00	0,00017
0.99	0.00	0.01	0.99	0.00	0.01	0,00017
0.99	0.00	0.00	0.99	0.00	0.01	0,00017
0.99	0.01	0.00	0.99	0.01	0.00	0,00017
0.99	0.00	0.00	0.99	0.00	0.01	0,00017
0.99	0.00	0.00	0.99	0.01	0.00	0,00017
0.99	0.00	0.00	0.98	0.01	0.01	0,00017
0.99	0.00	0.01	0.99	0.00	0.01	0,00017
0.99	0.00	0.01	0.99	0.01	0.00	0,00017
0.99	0.01	0.01	0.99	0.00	0.01	0,00017
0.99	0.00	0.01	0.99	0.00	0.00	0,00017
0.99	0.00	0.00	0.99	0.01	0.01	0,00017
0.99	0.01	0.00	0.99	0.00	0.01	0,00017

chr9	100498660	1982	c	0	1.00	0.00	0.00	1.00	0.00	0.00	1.00	0.00	0.00	1.00	0.00	0.00	1.00	0.00	0.00	1.00	0.00	0.00
chrX	7476621	2159	G	0	1.00	0.00	0.00	0.99	0.00	0.01	1.00	0.00	0.00	1.00	0.00	0.00	1.00	0.00	0.00	1.00	0.00	0.00
chrX	7476660	2188	G	0	1.00	0.00	0.00	1.00	0.00	0.00	1.00	0.00	0.00	1.00	0.00	0.00	1.00	0.00	0.00	1.00	0.00	0.00
chrX	7539511	2339	G	0	0.99	0.00	0.00	1.00	0.00	0.00	0.99	0.00	0.00	1.00	0.00	0.00	1.00	0.00	0.00	0.99	0.00	0.01
chrX	7539625	2408	G	0	1.00	0.00	0.00	1.00	0.00	0.00	1.00	0.00	0.00	1.00	0.00	0.00	0.99	0.00	0.00	0.99	0.00	0.00
chr11	3525692	49	C	0	1.00	0.00	0.00	1.00	0.00	0.00	0.99	0.00	0.00	1.00	0.00	0.00	1.00	0.00	0.00	1.00	0.00	0.00
chr11	3525837	133	C	0	1.00	0.00	0.00	1.00	0.00	0.00	1.00	0.00	0.00	1.00	0.00	0.00	1.00	0.00	0.00	1.00	0.00	0.00
chr11	3525853	142	G	0	1.00	0.00	0.00	1.00	0.00	0.00	1.00	0.00	0.00	1.00	0.00	0.00	0.99	0.00	0.00	1.00	0.00	0.00
chr15	61868673	211	C	0	1.00	0.00	0.00	1.00	0.00	0.00	1.00	0.00	0.00	0.98	0.02	0.00	1.00	0.00	0.00	1.00	0.00	0.00
chr17	45706589	781	C	0	0.99	0.00	0.00	1.00	0.00	0.00	1.00	0.00	0.00	1.00	0.00	0.00	1.00	0.00	0.00	1.00	0.00	0.00
chr17	45706655	814	C	0	1.00	0.00	0.00	0.99	0.01	0.00	0.99	0.00	0.00	1.00	0.00	0.00	1.00	0.00	0.00	1.00	0.00	0.00
chr17	45706700	832	C	0	1.00	0.00	0.00	1.00	0.00	0.00	1.00	0.00	0.00	1.00	0.00	0.00	0.99	0.00	0.00	1.00	0.00	0.00
chr19	24277518	920	c	0	1.00	0.00	0.00	1.00	0.00	0.00	0.99	0.00	0.00	0.99	0.00	0.00	0.99	0.00	0.00	1.00	0.00	0.00
chr5	64987327	1411	G	0	1.00	0.00	0.00	1.00	0.00	0.00	1.00	0.00	0.00	0.99	0.00	0.01	1.00	0.00	0.00	1.00	0.00	0.00
chr5	64987407	1442	C	0	1.00	0.00	0.00	0.99	0.00	0.00	1.00	0.00	0.00	1.00	0.00	0.00	1.00	0.00	0.00	0.99	0.01	0.00
chr8	120115620	1723	G	0	1.00	0.00	0.00	1.00	0.00	0.00	1.00	0.00	0.00	1.00	0.00	0.00	1.00	0.00	0.00	1.00	0.00	0.00
chr8	120115725	1776	G	0	1.00	0.00	0.00	0.99	0.00	0.00	0.99	0.00	0.00	1.00	0.00	0.00	0.99	0.01	0.00	1.00	0.00	0.00
chrX	7539480	2324	C	0	1.00	0.00	0.00	1.00	0.00	0.00	1.00	0.00	0.00	1.00	0.00	0.00	0.99	0.00	0.00	1.00	0.00	0.00
chr11	3525769	92	G	0	1.00	0.00	0.00	0.99	0.00	0.00	0.99	0.00	0.01	0.99	0.00	0.00	1.00	0.00	0.00	1.00	0.00	0.00
chr11	3525870	157	G	0	1.00	0.00	0.00	1.00	0.00	0.00	0.99	0.00	0.00	1.00	0.00	0.00	1.00	0.00	0.00	1.00	0.00	0.00
chr15	61868713	229	C	0	1.00	0.00	0.00	1.00	0.00	0.00	1.00	0.00	0.00	0.99	0.00	0.00	1.00	0.00	0.00	1.00	0.00	0.00
chr17	45706629	801	C	0	1.00	0.00	0.00	1.00	0.00	0.00	1.00	0.00	0.00	0.99	0.00	0.00	1.00	0.00	0.00	1.00	0.00	0.00
chr17	45706768	861	G	0	1.00	0.00	0.00	1.00	0.00	0.00	0.99	0.00	0.00	1.00	0.00	0.00	1.00	0.00	0.00	1.00	0.00	0.00
chr19	24277677	987	C	0	1.00	0.00	0.00	1.00	0.00	0.00	1.00	0.00	0.00	0.99	0.00	0.00	1.00	0.00	0.00	1.00	0.00	0.00
chr5	64987553	1513	G	0	1.00	0.00	0.00	0.99	0.00	0.00	0.99	0.00	0.00	1.00	0.00	0.00	1.00	0.00	0.00	1.00	0.00	0.00
chr5	64987607	1532	C	0	1.00	0.00	0.00	0.99	0.00	0.00	1.00	0.00	0.00	0.99	0.00	0.00	1.00	0.00	0.00	1.00	0.00	0.00
chr8	89377009	1665	G	0	1.00	0.00	0.00	1.00	0.00	0.00	1.00	0.00	0.00	0.95	0.05	0.00	1.00	0.00	0.00	1.00	0.00	0.00
chr9	100498477	1909	G	0	0.99	0.00	0.00	0.99	0.00	0.00	1.00	0.00	0.00	1.00	0.00	0.00	1.00	0.00	0.00	1.00	0.00	0.00
chr9	100498575	1963	C	0	1.00	0.00	0.00	1.00	0.00	0.00	1.00	0.00	0.00	1.00	0.00	0.00	0.99	0.00	0.00	1.00	0.00	0.00
chrX	7476321	2009	G	0	1.00	0.00	0.00	1.00	0.00	0.00	1.00	0.00	0.00	1.00	0.00	0.00	0.99	0.00	0.00	1.00	0.00	0.00
chrX	7476403	2029	G	0	0.99	0.00	0.00	1.00	0.00	0.00	0.99	0.00	0.00	1.00	0.00	0.00	1.00	0.00	0.00	1.00	0.00	0.00
chrX	7476645	2177	G	0	1.00	0.00	0.00	1.00	0.00	0.00	1.00	0.00	0.00	1.00	0.00	0.00	1.00	0.00	0.00	1.00	0.00	0.00
chrX	7476653	2182	G	0	1.00	0.00	0.00	0.98	0.02	0.00	1.00	0.00	0.00	1.00	0.00	0.00	1.00	0.00	0.00	1.00	0.00	0.00
chr11	3525794	110	C	0	1.00	0.00	0.00	0.99	0.00	0.01	0.99	0.00	0.01	1.00	0.00	0.00	1.00	0.00	0.00	0.99	0.00	0.01
chr15	61868716	231	G	0	0.99	0.00	0.00	1.00	0.00	0.00	0.99	0.00	0.00	1.00	0.00	0.00	1.00	0.00	0.00	1.00	0.00	0.00
chr15	100300434	505	C	0	1.00	0.00	0.00	0.99	0.00	0.00	0.99	0.00	0.00	0.99	0.00	0.00	1.00	0.00	0.00	1.00	0.00	0.00
chr17	45706692	829	C	0	1.00	0.00	0.00	1.00	0.00	0.00	1.00	0.00	0.00	1.00	0.00	0.00	1.00	0.00	0.00	1.00	0.00	0.00
chr19	24277487	904	c	0	1.00	0.00	0.00	1.00	0.00	0.00	1.00	0.00	0.00	1.00	0.00	0.00	1.00	0.00	0.00	0.99	0.01	0.00
chr4	139783851	1367	C	0	1.00	0.00	0.00	1.00	0.00	0.00	1.00	0.00	0.00	1.00	0.00	0.00	1.00	0.00	0.00	1.00	0.00	0.00
chr8	89376807	1572	c	0	1.00	0.00	0.00	1.00	0.00	0.00	1.00	0.00	0.00	1.00	0.00	0.00	1.00	0.00	0.00	1.00	0.00	0.00
chr8	89376886	1617	g	0	1.00	0.00	0.00	1.00	0.00	0.00	1.00	0.00	0.00	0.99	0.00	0.00	1.00	0.00	0.00	1.00	0.00	0.00
chr8	120115669	1747	C	0	1.00	0.00	0.00	1.00	0.00	0.00	0.99	0.00	0.00	1.00	0.00	0.00	1.00	0.00	0.00	1.00	0.00	0.00
chr8	120115823	1822	G	0	1.00	0.00	0.00	1.00	0.00	0.00	1.00	0.00	0.00	1.00	0.00	0.00	1.00	0.00	0.00	1.00	0.00	0.00
chr9	100498351	1845	G	0	1.00	0.00	0.00	1.00	0.00	0.00	1.00	0.00	0.00	0.99	0.00	0.01	0.99	0.00	0.00	1.00	0.00	0.00
chrX	7476337	2012	G	1	1.00	0.00	0.00	1.00	0.00	0.00	1.00	0.00	0.00	1.00	0.00	0.00	1.00	0.00	0.00	0.99	0.00	0.00
chrX	7476421	2034	G	1	1.00	0.00	0.00	1.00	0.00	0.00	1.00	0.00	0.00	1.00	0.00	0.00	1.00	0.00	0.00	1.00	0.00	0.00

0.99	0.00	0.00	0.99	0.01	0.00	0,00017
0.99	0.01	0.01	0.99	0.00	0.00	0,00017
0.99	0.01	0.00	0.99	0.01	0.00	0,00017
0.99	0.00	0.01	0.99	0.00	0.01	0,00017
0.99	0.00	0.00	0.99	0.01	0.01	0,00017
0.99	0.01	0.00	0.99	0.00	0.00	0,00017
0.99	0.00	0.00	0.99	0.00	0.01	0,00017
0.99	0.00	0.01	0.99	0.00	0.01	0,00017
0.99	0.00	0.01	0.99	0.01	0.00	0,00017
0.99	0.00	0.01	0.99	0.00	0.00	0,00017
0.99	0.01	0.01	0.99	0.00	0.01	0,00017
0.99	0.00	0.01	0.99	0.00	0.01	0,00017
0.99	0.00	0.01	0.99	0.00	0.01	0,00017
0.99	0.01	0.01	0.99	0.00	0.01	0,00017
0.99	0.01	0.00	0.99	0.00	0.00	0,00017
0.99	0.01	0.00	0.99	0.01	0.00	0,00017
0.99	0.01	0.01	0.99	0.01	0.00	0,00017
0.99	0.00	0.00	0.99	0.01	0.01	0,00017
0.99	0.00	0.01	0.99	0.00	0.01	0,00017
0.99	0.01	0.00	0.99	0.00	0.01	0,00017
0.99	0.01	0.00	0.99	0.00	0.01	0,00017
0.99	0.00	0.00	0.99	0.01	0.01	0,00017
0.99	0.00	0.00	0.99	0.01	0.01	0,00017
0.99	0.00	0.00	0.99	0.01	0.01	0,00017
0.99	0.00	0.01	0.99	0.00	0.00	0,00017
0.99	0.00	0.01	0.99	0.00	0.00	0,00017
0.99	0.00	0.01	0.99	0.01	0.00	0,00017
0.99	0.01	0.00	0.99	0.00	0.00	0,00017
0.99	0.00	0.00	0.99	0.01	0.00	0,00017
0.99	0.00	0.00	0.99	0.01	0.00	0,00017
0.99	0.00	0.01	0.99	0.01	0.00	0,00017
0.99	0.01	0.00	0.99	0.01	0.00	0,00017
0.99	0.01	0.00	0.99	0.01	0.00	0,00017
0.99	0.00	0.01	0.99	0.00	0.01	0,00017
0.99	0.01	0.01	0.99	0.00	0.00	0,00017
0.99	0.01	0.01	0.99	0.00	0.00	0,00017
0.99	0.01	0.01	0.99	0.00	0.01	0,00017
0.99	0.01	0.00	0.99	0.00	0.01	0,00017
0.99	0.00	0.00	0.99	0.00	0.00	0,00017
0.99	0.00	0.00	0.99	0.01	0.01	0,00017
0.99	0.00	0.00	0.99	0.00	0.01	0,00017
0.99	0.01	0.01	0.99	0.00	0.00	0,00017
0.99	0.01	0.00	0.99	0.00	0.01	0,00017
0.99	0.00	0.00	0.99	0.01	0.00	0,00017
0.99	0.00	0.00	0.99	0.01	0.01	0,00017
0.99	0.01	0.00	0.99	0.00	0.00	0,00017

chrX	7539565	2374	G	0	0.99	0.00	0.00	1.00	0.00	0.00	1.00	0.00	0.00	0.99	0.01	0.00	1.00	0.00	0.00	1.00	0.00	0.00
chr11	3525618	14	G	0	1.00	0.00	0.00	0.99	0.00	0.00	1.00	0.00	0.00	1.00	0.00	0.00	0.99	0.00	0.01	1.00	0.00	0.00
chr16	92644445	561	C	0	1.00	0.00	0.00	0.99	0.00	0.01	0.99	0.00	0.00	0.99	0.00	0.01	0.99	0.00	0.00	1.00	0.00	0.00
chr16	92644459	571	G	0	1.00	0.00	0.00	0.99	0.00	0.01	1.00	0.00	0.00	0.99	0.00	0.00	1.00	0.00	0.00	1.00	0.00	0.00
chr17	45706515	737	C	0	1.00	0.00	0.00	1.00	0.00	0.00	1.00	0.00	0.00	1.00	0.00	0.00	1.00	0.00	0.00	1.00	0.00	0.00
chr19	24277403	881	c	0	1.00	0.00	0.00	1.00	0.00	0.00	1.00	0.00	0.00	1.00	0.00	0.00	1.00	0.00	0.00	1.00	0.00	0.00
chr4	139783824	1353	C	0	1.00	0.00	0.00	1.00	0.00	0.00	1.00	0.00	0.00	1.00	0.00	0.00	1.00	0.00	0.00	1.00	0.00	0.00
chr8	89376819	1577	c	0	1.00	0.00	0.00	1.00	0.00	0.00	0.99	0.00	0.00	1.00	0.00	0.00	1.00	0.00	0.00	1.00	0.00	0.00
chr8	89377028	1671	C	0	1.00	0.00	0.00	1.00	0.00	0.00	0.99	0.01	0.00	1.00	0.00	0.00	1.00	0.00	0.00	0.99	0.01	0.00
chr8	120115824	1823	G	0	1.00	0.00	0.00	1.00	0.00	0.00	1.00	0.00	0.00	0.99	0.00	0.01	0.99	0.00	0.00	1.00	0.00	0.00
chrX	7476633	2168	G	1	1.00	0.00	0.00	1.00	0.00	0.00	1.00	0.00	0.00	1.00	0.00	0.00	0.99	0.00	0.00	0.99	0.00	0.01
chr11	3525695	51	G	0	1.00	0.00	0.00	1.00	0.00	0.00	1.00	0.00	0.00	1.00	0.00	0.00	0.99	0.00	0.00	1.00	0.00	0.00
chr11	3525860	149	G	0	1.00	0.00	0.00	0.99	0.00	0.00	1.00	0.00	0.00	0.99	0.00	0.00	1.00	0.00	0.00	1.00	0.00	0.00
chr15	61868755	250	G	0	1.00	0.00	0.00	1.00	0.00	0.00	1.00	0.00	0.00	1.00	0.00	0.00	1.00	0.00	0.00	0.99	0.00	0.00
chr17	45706440	702	C	0	1.00	0.00	0.00	1.00	0.00	0.00	1.00	0.00	0.00	1.00	0.00	0.00	1.00	0.00	0.00	1.00	0.00	0.00
chr4	136547392	1044	G	0	1.00	0.00	0.00	1.00	0.00	0.00	1.00	0.00	0.00	1.00	0.00	0.00	1.00	0.00	0.00	0.99	0.00	0.00
chr4	139783836	1359	C	0	1.00	0.00	0.00	1.00	0.00	0.00	1.00	0.00	0.00	1.00	0.00	0.00	1.00	0.00	0.00	1.00	0.00	0.00
chr5	64987323	1409	G	0	1.00	0.00	0.00	0.99	0.00	0.01	1.00	0.00	0.00	1.00	0.00	0.00	1.00	0.00	0.00	0.99	0.00	0.00
chr8	89376950	1642	G	0	1.00	0.00	0.00	0.99	0.00	0.00	0.99	0.00	0.01	0.99	0.00	0.01	1.00	0.00	0.00	1.00	0.00	0.00
chr9	100498408	1872	C	0	1.00	0.00	0.00	1.00	0.00	0.00	1.00	0.00	0.00	1.00	0.00	0.00	1.00	0.00	0.00	1.00	0.00	0.00
chr9	100498455	1896	G	0	1.00	0.00	0.00	1.00	0.00	0.00	1.00	0.00	0.00	1.00	0.00	0.00	1.00	0.00	0.00	1.00	0.00	0.00
chr9	100498693	1992	c	0	1.00	0.00	0.00	1.00	0.00	0.00	1.00	0.00	0.00	1.00	0.00	0.00	1.00	0.00	0.00	1.00	0.00	0.00
chrX	7539331	2231	G	0	0.99	0.00	0.01	1.00	0.00	0.00	1.00	0.00	0.00	0.99	0.00	0.01	1.00	0.00	0.00	1.00	0.00	0.00
chrX	7539475	2320	C	0	1.00	0.00	0.00	1.00	0.00	0.00	1.00	0.00	0.00	1.00	0.00	0.00	1.00	0.00	0.00	1.00	0.00	0.00
chrX	7539627	2410	C	0	1.00	0.00	0.00	1.00	0.00	0.00	0.99	0.00	0.00	1.00	0.00	0.00	1.00	0.00	0.00	1.00	0.00	0.00
chr11	3525788	104	G	0	1.00	0.00	0.00	1.00	0.00	0.00	1.00	0.00	0.00	0.99	0.00	0.00	1.00	0.00	0.00	1.00	0.00	0.00
chr4	136547449	1076	C	0	1.00	0.00	0.00	1.00	0.00	0.00	1.00	0.00	0.00	1.00	0.00	0.00	0.99	0.00	0.00	1.00	0.00	0.00
chr4	139783675	1272	G	0	1.00	0.00	0.00	1.00	0.00	0.00	0.99	0.00	0.00	1.00	0.00	0.00	0.99	0.00	0.00	0.99	0.01	0.00
chr5	64987349	1422	C	0	1.00	0.00	0.00	0.99	0.00	0.00	1.00	0.00	0.00	0.99	0.00	0.00	1.00	0.00	0.00	1.00	0.00	0.00
chrX	7539484	2327	C	0	1.00	0.00	0.00	0.99	0.00	0.00	1.00	0.00	0.00	1.00	0.00	0.00	1.00	0.00	0.00	1.00	0.00	0.00
chrX	7539572	2378	G	0	1.00	0.00	0.00	1.00	0.00	0.00	1.00	0.00	0.00	1.00	0.00	0.00	1.00	0.00	0.00	1.00	0.00	0.00
chr15	100300055	380	G	0	1.00	0.00	0.00	1.00	0.00	0.00	1.00	0.00	0.00	1.00	0.00	0.00	1.00	0.00	0.00	1.00	0.00	0.00
chr4	136547480	1094	G	0	1.00	0.00	0.00	0.99	0.00	0.00	1.00	0.00	0.00	1.00	0.00	0.00	0.99	0.01	0.00	1.00	0.00	0.00
chr4	139783794	1337	C	0	1.00	0.00	0.00	1.00	0.00	0.00	1.00	0.00	0.00	1.00	0.00	0.00	1.00	0.00	0.00	1.00	0.00	0.00
chr4	139783829	1355	G	0	1.00	0.00	0.00	1.00	0.00	0.00	1.00	0.00	0.00	1.00	0.00	0.00	1.00	0.00	0.00	1.00	0.00	0.00
chr4	139783864	1377	G	0	1.00	0.00	0.00	1.00	0.00	0.00	1.00	0.00	0.00	1.00	0.00	0.00	1.00	0.00	0.00	1.00	0.00	0.00
chrX	7476532	2096	C	0	1.00	0.00	0.00	1.00	0.00	0.00	1.00	0.00	0.00	1.00	0.00	0.00	0.99	0.00	0.01	1.00	0.00	0.00
chrX	7539626	2409	C	0	1.00	0.00	0.00	1.00	0.00	0.00	1.00	0.00	0.00	1.00	0.00	0.00	1.00	0.00	0.00	1.00	0.00	0.00
chr11	3525732	73	C	0	1.00	0.00	0.00	1.00	0.00	0.00	1.00	0.00	0.00	1.00	0.00	0.00	1.00	0.00	0.00	1.00	0.00	0.00
chr15	100300082	394	C	0	0.99	0.00	0.00	0.99	0.00	0.00	1.00	0.00	0.00	0.99	0.00	0.00	1.00	0.00	0.00	1.00	0.00	0.00
chr16	92644380	536	G	0	0.99	0.00	0.00	1.00	0.00	0.00	1.00	0.00	0.00	0.99	0.00	0.00	1.00	0.00	0.00	1.00	0.00	0.00
chr17	45706611	792	G	0	1.00	0.00	0.00	0.99	0.00	0.00	0.99	0.00	0.00	0.99	0.00	0.00	1.00	0.00	0.00	1.00	0.00	0.00
chr4	136547462	1085	C	0	1.00	0.00	0.00	1.00	0.00	0.00	0.99	0.01	0.00	1.00	0.00	0.00	1.00	0.00	0.00	1.00	0.00	0.00
chr4	139783635	1249	G	0	1.00	0.00	0.00	1.00	0.00	0.00	1.00	0.00	0.00	1.00	0.00	0.00	1.00	0.00	0.00	1.00	0.00	0.00
chr5	64987358	1426	C	0	1.00	0.00	0.00	0.99	0.00	0.00	1.00	0.00	0.00	1.00	0.00	0.00	1.00	0.00	0.00	1.00	0.00	0.00
chr5	64987421	1448	G	0	1.00	0.00	0.00	0.99	0.00	0.00	1.00	0.00	0.00	0.99	0.00	0.00	1.00	0.00	0.00	1.00	0.00	0.00

0.99	0.01	0.00	0.99	0.01	0.00	0,00017
0.99	0.00	0.00	0.99	0.00	0.01	0,00017
0.99	0.01	0.01	0.99	0.01	0.01	0,00017
0.99	0.01	0.01	0.99	0.00	0.00	0,00017
0.99	0.00	0.00	0.99	0.00	0.01	0,00017
0.99	0.01	0.00	0.99	0.00	0.00	0,00017
0.99	0.00	0.00	0.99	0.01	0.01	0,00017
0.99	0.00	0.00	0.99	0.00	0.00	0,00017
0.99	0.00	0.01	0.99	0.01	0.00	0,00017
0.99	0.01	0.00	0.99	0.00	0.01	0,00017
0.99	0.00	0.00	0.99	0.01	0.00	0,00017
0.99	0.00	0.00	0.99	0.00	0.01	0,00017
0.99	0.01	0.01	0.99	0.01	0.00	0,00017
0.99	0.01	0.00	0.99	0.01	0.01	0,00017
0.99	0.00	0.01	0.99	0.01	0.00	0,00017
0.99	0.01	0.00	0.99	0.01	0.00	0,00017
0.99	0.01	0.01	0.99	0.00	0.00	0,00017
0.99	0.00	0.00	0.99	0.00	0.01	0,00017
0.99	0.01	0.01	0.99	0.00	0.01	0,00017
0.99	0.00	0.01	0.99	0.00	0.01	0,00017
0.99	0.01	0.00	0.99	0.01	0.00	0,00017
0.99	0.01	0.00	0.99	0.00	0.00	0,00017
0.99	0.00	0.01	0.99	0.00	0.01	0,00017
0.99	0.01	0.00	0.99	0.00	0.00	0,00017
0.99	0.01	0.00	0.99	0.01	0.00	0,00017
0.99	0.01	0.01	0.99	0.00	0.00	0,00017
0.99	0.00	0.00	0.99	0.01	0.01	0,00017
0.99	0.00	0.00	0.99	0.01	0.01	0,00017
0.99	0.01	0.00	0.99	0.01	0.00	0,00017
0.99	0.00	0.00	0.99	0.01	0.00	0,00017
0.99	0.00	0.01	0.99	0.01	0.00	0,00017
0.99	0.00	0.01	0.99	0.01	0.01	0,00017
0.99	0.01	0.01	0.99	0.01	0.00	0,00017
0.99	0.00	0.01	0.99	0.00	0.01	0,00017
0.99	0.00	0.00	0.99	0.00	0.01	0,00017
0.99	0.01	0.00	0.99	0.00	0.00	0,00017
0.99	0.01	0.00	0.99	0.01	0.00	0,00017
0.99	0.00	0.00	0.99	0.01	0.00	0,00017
0.99	0.01	0.00	0.99	0.01	0.00	0,00017
0.99	0.01	0.01	0.99	0.00	0.01	0,00017
0.99	0.01	0.00	0.99	0.01	0.00	0,00017
0.99	0.00	0.01	0.99	0.00	0.01	0,00017
0.99	0.00	0.01	0.99	0.00	0.01	0,00017
0.99	0.01	0.00	0.99	0.01	0.00	0,00017
0.99	0.00	0.00	0.99	0.00	0.00	0,00017
0.99	0.01	0.00	0.99	0.00	0.01	0,00017

chr8	89377013	1667	C	0	1.00	0.00	0.00	1.00	0.00	0.00	1.00	0.00	0.00	1.00	0.00	0.00	1.00	0.00	0.00	1.00	0.00	0.00
chrX	7476479	2059	C	0	1.00	0.00	0.00	1.00	0.00	0.00	1.00	0.00	0.00	1.00	0.00	0.00	0.98	0.00	0.01	1.00	0.00	0.00
chr11	3525624	18	G	0	1.00	0.00	0.00	1.00	0.00	0.00	1.00	0.00	0.00	0.99	0.00	0.01	1.00	0.00	0.00	1.00	0.00	0.00
chr11	3525862	151	G	0	1.00	0.00	0.00	1.00	0.00	0.00	1.00	0.00	0.00	0.99	0.00	0.00	1.00	0.00	0.00	1.00	0.00	0.00
chr15	61868825	284	C	0	1.00	0.00	0.00	1.00	0.00	0.00	0.99	0.00	0.00	1.00	0.00	0.00	1.00	0.00	0.00	0.99	0.00	0.00
chr15	61868898	318	G	0	1.00	0.00	0.00	1.00	0.00	0.00	1.00	0.00	0.00	0.99	0.00	0.01	1.00	0.00	0.00	1.00	0.00	0.00
chr15	100300088	398	C	0	1.00	0.00	0.00	0.99	0.00	0.00	0.99	0.00	0.00	0.99	0.00	0.00	1.00	0.00	0.00	0.99	0.00	0.00
chr16	92644390	541	G	0	1.00	0.00	0.00	1.00	0.00	0.00	1.00	0.00	0.00	0.99	0.01	0.00	1.00	0.00	0.00	1.00	0.00	0.00
chr19	24277758	1012	g	0	1.00	0.00	0.00	1.00	0.00	0.00	1.00	0.00	0.00	1.00	0.00	0.00	1.00	0.00	0.00	1.00	0.00	0.00
chr5	64987595	1529	G	0	1.00	0.00	0.00	1.00	0.00	0.00	1.00	0.00	0.00	1.00	0.00	0.00	1.00	0.00	0.00	1.00	0.00	0.00
chr8	89376943	1640	G	0	1.00	0.00	0.00	1.00	0.00	0.00	0.98	0.01	0.01	0.99	0.00	0.01	1.00	0.00	0.00	1.00	0.00	0.00
chrX	7476500	2071	C	1	1.00	0.00	0.00	1.00	0.00	0.00	1.00	0.00	0.00	0.99	0.01	0.00	0.99	0.00	0.01	0.99	0.01	0.00
chrX	7539335	2235	G	0	1.00	0.00	0.00	1.00	0.00	0.00	1.00	0.00	0.00	1.00	0.00	0.00	1.00	0.00	0.00	0.99	0.00	0.00
chr11	3525636	23	C	0	1.00	0.00	0.00	1.00	0.00	0.00	1.00	0.00	0.00	1.00	0.00	0.00	1.00	0.00	0.00	1.00	0.00	0.00
chr11	3525657	32	C	0	1.00	0.00	0.00	1.00	0.00	0.00	1.00	0.00	0.00	1.00	0.00	0.00	1.00	0.00	0.00	1.00	0.00	0.00
chr15	61868795	270	G	0	1.00	0.00	0.00	1.00	0.00	0.00	1.00	0.00	0.00	1.00	0.00	0.00	0.99	0.00	0.00	1.00	0.00	0.00
chr15	61868929	331	G	0	1.00	0.00	0.00	1.00	0.00	0.00	0.99	0.00	0.01	1.00	0.00	0.00	1.00	0.00	0.00	1.00	0.00	0.00
chr15	100300058	382	C	0	1.00	0.00	0.00	0.99	0.00	0.00	0.99	0.00	0.00	0.99	0.00	0.00	1.00	0.00	0.00	1.00	0.00	0.00
chr16	92644472	581	G	0	0.99	0.00	0.00	0.99	0.00	0.00	1.00	0.00	0.00	1.00	0.00	0.00	1.00	0.00	0.00	0.99	0.00	0.00
chr19	24277508	913	g	0	1.00	0.00	0.00	0.99	0.00	0.00	1.00	0.00	0.00	0.99	0.00	0.00	1.00	0.00	0.00	1.00	0.00	0.00
chr19	24277623	972	C	0	1.00	0.00	0.00	0.99	0.00	0.00	1.00	0.00	0.00	1.00	0.00	0.00	1.00	0.00	0.00	0.99	0.01	0.00
chr4	136547340	1025	G	0	0.99	0.01	0.00	1.00	0.00	0.00	1.00	0.00	0.00	0.99	0.00	0.01	1.00	0.00	0.00	1.00	0.00	0.00
chr4	136547494	1100	C	0	1.00	0.00	0.00	1.00	0.00	0.00	0.99	0.00	0.00	1.00	0.00	0.00	1.00	0.00	0.00	1.00	0.00	0.00
chr4	139783642	1254	G	0	1.00	0.00	0.00	1.00	0.00	0.00	1.00	0.00	0.00	1.00	0.00	0.00	1.00	0.00	0.00	1.00	0.00	0.00
chr5	64987338	1415	G	0	1.00	0.00	0.00	1.00	0.00	0.00	0.99	0.00	0.00	1.00	0.00	0.00	1.00	0.00	0.00	1.00	0.00	0.00
chr5	64987545	1509	G	0	1.00	0.00	0.00	0.99	0.00	0.00	1.00	0.00	0.00	1.00	0.00	0.00	1.00	0.00	0.00	1.00	0.00	0.00
chr8	89376811	1574	c	0	1.00	0.00	0.00	1.00	0.00	0.00	1.00	0.00	0.00	1.00	0.00	0.00	1.00	0.00	0.00	1.00	0.00	0.00
chr8	89377023	1668	C	0	1.00	0.00	0.00	1.00	0.00	0.00	1.00	0.00	0.00	1.00	0.00	0.00	1.00	0.00	0.00	1.00	0.00	0.00
chr8	120115709	1768	G	0	1.00	0.00	0.00	1.00	0.00	0.00	1.00	0.00	0.00	1.00	0.00	0.00	1.00	0.00	0.00	1.00	0.00	0.00
chr8	120115793	1804	C	0	1.00	0.00	0.00	0.99	0.00	0.00	0.99	0.00	0.01	0.99	0.00	0.00	1.00	0.00	0.00	0.99	0.00	0.00
chrX	7476603	2147	G	1	1.00	0.00	0.00	1.00	0.00	0.00	1.00	0.00	0.00	0.99	0.00	0.01	1.00	0.00	0.00	0.99	0.00	0.01
chrX	7539343	2237	G	0	1.00	0.00	0.00	1.00	0.00	0.00	1.00	0.00	0.00	1.00	0.00	0.00	1.00	0.00	0.00	1.00	0.00	0.00
chrX	7539547	2362	G	0	1.00	0.00	0.00	1.00	0.00	0.00	1.00	0.00	0.00	1.00	0.00	0.00	1.00	0.00	0.00	1.00	0.00	0.00
chr15	61868693	221	C	0	1.00	0.00	0.00	1.00	0.00	0.00	1.00	0.00	0.00	1.00	0.00	0.00	1.00	0.00	0.00	1.00	0.00	0.00
chr15	61868760	253	G	0	1.00	0.00	0.00	1.00	0.00	0.00	1.00	0.00	0.00	1.00	0.00	0.00	0.99	0.01	0.00	1.00	0.00	0.00
chr15	100300015	360	G	0	1.00	0.00	0.00	1.00	0.00	0.00	1.00	0.00	0.00	1.00	0.00	0.00	1.00	0.00	0.00	1.00	0.00	0.00
chr16	92644388	539	G	0	0.99	0.01	0.00	0.99	0.00	0.01	1.00	0.00	0.00	0.99	0.00	0.00	1.00	0.00	0.00	1.00	0.00	0.00
chr17	45706497	729	G	0	1.00	0.00	0.00	0.99	0.00	0.00	1.00	0.00	0.00	1.00	0.00	0.00	1.00	0.00	0.00	1.00	0.00	0.00
chr17	45706770	862	G	0	1.00	0.00	0.00	1.00	0.00	0.00	1.00	0.00	0.00	0.99	0.01	0.00	1.00	0.00	0.00	1.00	0.00	0.00
chr4	136547349	1027	G	0	1.00	0.00	0.00	1.00	0.00	0.00	1.00	0.00	0.00	0.99	0.01	0.00	1.00	0.00	0.00	1.00	0.00	0.00
chr4	139783650	1258	G	0	1.00	0.00	0.00	1.00	0.00	0.00	1.00	0.00	0.00	1.00	0.00	0.00	1.00	0.00	0.00	1.00	0.00	0.00
chr4	139783808	1344	G	0	1.00	0.00	0.00	1.00	0.00	0.00	1.00	0.00	0.00	1.00	0.00	0.00	0.99	0.01	0.00	0.99	0.00	0.00
chrX	7476394	2027	G	0	1.00	0.00	0.00	1.00	0.00	0.00	1.00	0.00	0.00	1.00	0.00	0.00	1.00	0.00	0.00	1.00	0.00	0.00
chrX	7539316	2229	G	0	1.00	0.00	0.00	0.99	0.00	0.01	1.00	0.00	0.00	1.00	0.00	0.00	1.00	0.00	0.00	1.00	0.00	0.00
chrX	7539537	2355	C	0	1.00	0.00	0.00	1.00	0.00	0.00	1.00	0.00	0.00	1.00	0.00	0.00	1.00	0.00	0.00	1.00	0.00	0.00
chr11	3525753	82	C	0	1.00	0.00	0.00	1.00	0.00	0.00	0.99	0.00	0.00	0.98	0.02	0.00	1.00	0.00	0.00	1.00	0.00	0.00

0.99	0.00	0.00	0.99	0.00	0.00	0,00017
0.99	0.01	0.00	0.99	0.01	0.00	0,00017
0.99	0.00	0.01	0.99	0.00	0.01	0,00016
0.99	0.00	0.01	0.99	0.00	0.01	0,00016
0.99	0.01	0.01	0.99	0.01	0.00	0,00016
0.99	0.01	0.00	0.99	0.01	0.01	0,00016
0.99	0.01	0.00	0.99	0.01	0.00	0,00016
0.99	0.00	0.00	0.99	0.01	0.00	0,00016
0.99	0.00	0.00	0.99	0.00	0.00	0,00016
0.99	0.00	0.00	0.99	0.00	0.00	0,00016
0.99	0.01	0.01	0.99	0.00	0.01	0,00016
0.99	0.00	0.00	0.99	0.01	0.00	0,00016
0.99	0.01	0.00	0.99	0.01	0.01	0,00016
0.99	0.01	0.00	0.99	0.01	0.00	0,00016
0.99	0.01	0.00	0.99	0.01	0.00	0,00016
0.99	0.01	0.00	0.99	0.01	0.00	0,00016
0.99	0.01	0.00	0.99	0.01	0.00	0,00016
0.99	0.01	0.01	0.99	0.00	0.00	0,00016
0.99	0.01	0.01	0.99	0.00	0.00	0,00016
0.99	0.00	0.01	0.99	0.01	0.00	0,00016
0.99	0.00	0.00	0.99	0.00	0.00	0,00016
0.99	0.00	0.00	0.99	0.00	0.01	0,00016
0.99	0.00	0.01	0.99	0.01	0.00	0,00016
0.99	0.01	0.00	0.99	0.01	0.00	0,00016
0.99	0.01	0.01	0.99	0.00	0.01	0,00016
0.99	0.00	0.01	0.99	0.00	0.01	0,00016
0.99	0.00	0.01	0.99	0.01	0.01	0,00016
0.99	0.00	0.01	0.99	0.00	0.00	0,00016
0.99	0.00	0.00	0.99	0.01	0.00	0,00016
0.99	0.00	0.01	0.99	0.00	0.01	0,00016
0.99	0.01	0.00	0.99	0.00	0.01	0,00016
0.99	0.00	0.00	0.99	0.00	0.00	0,00016
0.99	0.01	0.00	0.99	0.00	0.00	0,00016
0.99	0.00	0.01	0.99	0.00	0.01	0,00016
0.99	0.00	0.00	0.99	0.01	0.00	0,00016
0.99	0.01	0.00	0.99	0.01	0.00	0,00016
0.99	0.01	0.01	0.99	0.00	0.00	0,00016
0.99	0.01	0.00	0.99	0.01	0.00	0,00016
0.99	0.00	0.00	0.99	0.01	0.01	0,00016
0.99	0.00	0.00	0.99	0.01	0.00	0,00016
0.99	0.00	0.00	0.99	0.01	0.00	0,00016
0.99	0.00	0.01	0.99	0.01	0.01	0,00016
0.99	0.01	0.00	0.99	0.00	0.01	0,00016
0.99	0.01	0.00	0.99	0.01	0.00	0,00016
0.99	0.01	0.00	0.99	0.00	0.01	0,00016
0.99	0.00	0.01	0.99	0.00	0.01	0,00016

chr15	61868740	245	C		0	1.00	0.00	0.00	1.00	0.00	0.00	1.00	0.00	0.00	1.00	0.00	0.00	0.99	0.00	0.00	1.00	0.00	0.00
chr15	61868914	327	G		0	0.99	0.01	0.00	1.00	0.00	0.00	0.99	0.00	0.00	1.00	0.00	0.00	1.00	0.00	0.00	1.00	0.00	0.00
chr15	100300049	375	C		0	1.00	0.00	0.00	0.99	0.00	0.00	1.00	0.00	0.00	0.99	0.00	0.01	1.00	0.00	0.00	1.00	0.00	0.00
chr17	45706602	790	G		0	1.00	0.00	0.00	1.00	0.00	0.00	1.00	0.00	0.00	1.00	0.00	0.00	1.00	0.00	0.00	1.00	0.00	0.00
chr17	45706773	863	G		0	1.00	0.00	0.00	1.00	0.00	0.00	1.00	0.00	0.00	0.99	0.00	0.00	1.00	0.00	0.00	1.00	0.00	0.00
chr19	24277477	898	c		0	0.99	0.00	0.00	1.00	0.00	0.00	1.00	0.00	0.00	1.00	0.00	0.00	1.00	0.00	0.00	1.00	0.00	0.00
chr19	24277540	933	G		0	1.00	0.00	0.00	1.00	0.00	0.00	1.00	0.00	0.00	0.99	0.00	0.00	1.00	0.00	0.00	0.99	0.00	0.00
chr4	139783666	1267	C		0	1.00	0.00	0.00	1.00	0.00	0.00	1.00	0.00	0.00	1.00	0.00	0.00	1.00	0.00	0.00	1.00	0.00	0.00
chr4	139783785	1332	G		0	1.00	0.00	0.00	1.00	0.00	0.00	1.00	0.00	0.00	1.00	0.00	0.00	1.00	0.00	0.00	1.00	0.00	0.00
chr5	64987496	1490	G		0	1.00	0.00	0.00	1.00	0.00	0.00	0.99	0.00	0.00	1.00	0.00	0.00	1.00	0.00	0.00	1.00	0.00	0.00
chr8	89376994	1662	C		0	1.00	0.00	0.00	1.00	0.00	0.00	0.99	0.00	0.00	1.00	0.00	0.00	1.00	0.00	0.00	1.00	0.00	0.00
chr9	100498341	1844	G		0	1.00	0.00	0.00	1.00	0.00	0.00	1.00	0.00	0.00	1.00	0.00	0.00	1.00	0.00	0.00	1.00	0.00	0.00
chr9	100498358	1850	G		0	0.99	0.00	0.00	1.00	0.00	0.00	1.00	0.00	0.00	1.00	0.00	0.00	1.00	0.00	0.00	1.00	0.00	0.00
chr9	100498406	1870	G		0	1.00	0.00	0.00	1.00	0.00	0.00	1.00	0.00	0.00	0.99	0.00	0.01	1.00	0.00	0.00	1.00	0.00	0.00
chrX	7539324	2230	G		0	1.00	0.00	0.00	0.99	0.00	0.01	1.00	0.00	0.00	1.00	0.00	0.00	1.00	0.00	0.00	1.00	0.00	0.00
chrX	7539352	2243	G		0	1.00	0.00	0.00	1.00	0.00	0.00	1.00	0.00	0.00	1.00	0.00	0.00	1.00	0.00	0.00	1.00	0.00	0.00
chrX	7539606	2398	C		0	1.00	0.00	0.00	1.00	0.00	0.00	1.00	0.00	0.00	1.00	0.00	0.00	1.00	0.00	0.00	0.99	0.01	0.00
chr15	61868753	249	G		0	1.00	0.00	0.00	1.00	0.00	0.00	0.99	0.00	0.00	1.00	0.00	0.00	1.00	0.00	0.00	1.00	0.00	0.00
chr15	61868856	300	C		0	1.00	0.00	0.00	1.00	0.00	0.00	0.99	0.00	0.00	0.99	0.00	0.00	1.00	0.00	0.00	0.99	0.01	0.00
chr15	61868893	316	G		0	1.00	0.00	0.00	1.00	0.00	0.00	1.00	0.00	0.00	1.00	0.00	0.00	1.00	0.00	0.00	1.00	0.00	0.00
chr17	45706722	845	C		0	1.00	0.00	0.00	1.00	0.00	0.00	1.00	0.00	0.00	0.99	0.00	0.01	1.00	0.00	0.00	1.00	0.00	0.00
chr4	136547496	1102	C		0	1.00	0.00	0.00	0.98	0.01	0.00	1.00	0.00	0.00	1.00	0.00	0.00	1.00	0.00	0.00	1.00	0.00	0.00
chr4	139783680	1276	C		0	1.00	0.00	0.00	1.00	0.00	0.00	1.00	0.00	0.00	1.00	0.00	0.00	0.98	0.02	0.00	1.00	0.00	0.00
chr4	139783823	1352	C		0	1.00	0.00	0.00	1.00	0.00	0.00	1.00	0.00	0.00	1.00	0.00	0.00	1.00	0.00	0.00	1.00	0.00	0.00
chr8	89376868	1606	c		0	1.00	0.00	0.00	1.00	0.00	0.00	1.00	0.00	0.00	1.00	0.00	0.00	0.99	0.00	0.00	1.00	0.00	0.00
chr9	100498411	1873	C		0	1.00	0.00	0.00	1.00	0.00	0.00	1.00	0.00	0.00	1.00	0.00	0.00	1.00	0.00	0.00	1.00	0.00	0.00
chr9	100498457	1897	G		0	0.99	0.00	0.00	1.00	0.00	0.00	1.00	0.00	0.00	1.00	0.00	0.00	1.00	0.00	0.00	1.00	0.00	0.00
chr9	100498650	1979	C		0	1.00	0.00	0.00	1.00	0.00	0.00	1.00	0.00	0.00	1.00	0.00	0.00	0.99	0.00	0.00	0.99	0.01	0.00
chrX	7539525	2347	G		0	1.00	0.00	0.00	0.99	0.01	0.00	1.00	0.00	0.00	0.98	0.01	0.00	1.00	0.00	0.00	1.00	0.00	0.00
chrX	7539578	2381	G		0	1.00	0.00	0.00	1.00	0.00	0.00	1.00	0.00	0.00	1.00	0.00	0.00	1.00	0.00	0.00	1.00	0.00	0.00
chrX	7539611	2402	C		0	1.00	0.00	0.00	1.00	0.00	0.00	1.00	0.00	0.00	0.99	0.00	0.00	1.00	0.00	0.00	0.99	0.01	0.00
chr11	3525633	21	G		0	1.00	0.00	0.00	1.00	0.00	0.00	1.00	0.00	0.00	1.00	0.00	0.00	1.00	0.00	0.00	1.00	0.00	0.00
chr11	3525723	68	C		0	0.99	0.00	0.01	1.00	0.00	0.00	1.00	0.00	0.00	0.99	0.01	0.00	1.00	0.00	0.00	1.00	0.00	0.00
chr11	3525783	102	G		0	1.00	0.00	0.00	1.00	0.00	0.00	1.00	0.00	0.00	1.00	0.00	0.00	1.00	0.00	0.00	1.00	0.00	0.00
chr15	61868788	267	G		0	1.00	0.00	0.00	0.99	0.01	0.00	1.00	0.00	0.00	1.00	0.00	0.00	0.99	0.00	0.00	1.00	0.00	0.00
chr15	61868904	321	G		0	1.00	0.00	0.00	1.00	0.00	0.00	1.00	0.00	0.00	0.99	0.01	0.00	1.00	0.00	0.00	1.00	0.00	0.00
chr15	100300090	399	G		0	1.00	0.00	0.00	1.00	0.00	0.00	1.00	0.00	0.00	0.99	0.01	0.00	1.00	0.00	0.00	1.00	0.00	0.00
chr15	100300219	445	G		0	1.00	0.00	0.00	1.00	0.00	0.00	1.00	0.00	0.00	1.00	0.00	0.00	1.00	0.00	0.00	1.00	0.00	0.00
chr16	92644477	585	G		0	0.99	0.00	0.00	0.99	0.00	0.00	1.00	0.00	0.00	0.99	0.00	0.00	1.00	0.00	0.00	1.00	0.00	0.00
chr16	92644499	600	G		0	0.99	0.00	0.00	0.99	0.00	0.00	1.00	0.00	0.00	1.00	0.00	0.00	1.00	0.00	0.00	1.00	0.00	0.00
chr17	45706713	837	G		0	1.00	0.00	0.00	1.00	0.00	0.00	0.99	0.00	0.01	1.00	0.00	0.00	1.00	0.00	0.00	1.00	0.00	0.00
chr19	24277433	887	c		0	1.00	0.00	0.00	1.00	0.00	0.00	0.99	0.00	0.00	1.00	0.00	0.00	1.00	0.00	0.00	1.00	0.00	0.00
chr4	136547696	1194	C		0	1.00	0.00	0.00	1.00	0.00	0.00	1.00	0.00	0.00	1.00	0.00	0.00	0.99	0.00	0.00	1.00	0.00	0.00
chr4	139783778	1328	C		0	1.00	0.00	0.00	1.00	0.00	0.00	1.00	0.00	0.00	1.00	0.00	0.00	1.00	0.00	0.00	1.00	0.00	0.00
chr4	139783793	1336	C		0	0.99	0.01	0.00	1.00	0.00	0.00	1.00	0.00	0.00	1.00	0.00	0.00	1.00	0.00	0.00	1.00	0.00	0.00
chr8	89376971	1652	G		0	1.00	0.00	0.00	1.00	0.00	0.00	1.00	0.00	0.00	1.00	0.00	0.00	1.00	0.00	0.00	1.00	0.00	0.00

0.99	0.01	0.00	0.99	0.00	0.01	0,00016
0.99	0.01	0.00	0.99	0.01	0.00	0,00016
0.99	0.01	0.00	0.99	0.01	0.00	0,00016
0.99	0.00	0.01	0.99	0.00	0.00	0,00016
0.99	0.00	0.00	0.99	0.00	0.01	0,00016
0.99	0.01	0.00	0.99	0.00	0.00	0,00016
0.99	0.00	0.00	0.99	0.01	0.00	0,00016
0.99	0.00	0.01	0.99	0.00	0.00	0,00016
0.99	0.00	0.01	0.99	0.00	0.01	0,00016
0.99	0.01	0.01	0.99	0.00	0.01	0,00016
0.99	0.01	0.01	0.99	0.00	0.00	0,00016
0.99	0.01	0.00	0.99	0.01	0.00	0,00016
0.99	0.01	0.00	0.99	0.01	0.00	0,00016
0.99	0.00	0.00	0.99	0.01	0.01	0,00016
0.99	0.00	0.01	0.99	0.00	0.00	0,00016
0.99	0.00	0.01	0.99	0.01	0.00	0,00016
0.99	0.00	0.01	0.99	0.00	0.00	0,00016
0.99	0.00	0.00	0.99	0.00	0.00	0,00016
0.99	0.01	0.00	0.99	0.01	0.01	0,00016
0.99	0.01	0.00	0.99	0.00	0.01	0,00016
0.99	0.01	0.00	0.99	0.01	0.01	0,00016
0.99	0.00	0.00	0.99	0.00	0.00	0,00016
0.99	0.00	0.00	0.99	0.01	0.01	0,00016
0.99	0.00	0.00	0.99	0.00	0.01	0,00016
0.99	0.00	0.01	0.99	0.00	0.01	0,00016
0.99	0.00	0.00	0.99	0.01	0.00	0,00016
0.99	0.01	0.00	0.99	0.01	0.00	0,00016
0.99	0.01	0.00	0.99	0.01	0.00	0,00016
0.99	0.01	0.00	0.99	0.01	0.00	0,00016
0.99	0.01	0.00	0.99	0.01	0.00	0,00016
0.99	0.00	0.00	0.99	0.01	0.00	0,00016
0.99	0.00	0.00	0.99	0.00	0.01	0,00016
0.99	0.01	0.01	0.99	0.01	0.00	0,00016
0.99	0.01	0.00	0.99	0.00	0.01	0,00016
0.99	0.00	0.01	0.99	0.00	0.01	0,00016
0.99	0.00	0.00	0.99	0.01	0.00	0,00016
0.99	0.00	0.00	0.99	0.01	0.00	0,00016
0.99	0.00	0.00	0.99	0.01	0.00	0,00016
0.99	0.01	0.00	0.99	0.00	0.00	0,00016
0.99	0.01	0.00	0.99	0.00	0.01	0,00016
0.99	0.00	0.01	0.99	0.00	0.00	0,00016
0.99	0.00	0.00	0.99	0.00	0.01	0,00016
0.99	0.00	0.00	0.99	0.00	0.01	0,00016
0.99	0.00	0.01	0.99	0.00	0.00	0,00016
0.99	0.01	0.00	0.99	0.01	0.00	0,00016
0.99	0.00	0.01	0.99	0.00	0.01	0,00016

chr9	100498444	1890	C	0	1.00	0.00	0.00	1.00	0.00	0.00	1.00	0.00	0.00	1.00	0.00	0.00	0.99	0.00	0.00	0.99	0.01	0.00
chr9	100498458	1898	C	0	1.00	0.00	0.00	1.00	0.00	0.00	1.00	0.00	0.00	1.00	0.00	0.00	1.00	0.00	0.00	1.00	0.00	0.00
chrX	7476330	2011	G	0	1.00	0.00	0.00	1.00	0.00	0.00	1.00	0.00	0.00	1.00	0.00	0.00	1.00	0.00	0.00	1.00	0.00	0.00
chrX	7476545	2107	C	0	1.00	0.00	0.00	1.00	0.00	0.00	1.00	0.00	0.00	1.00	0.00	0.00	0.99	0.00	0.01	1.00	0.00	0.00
chrX	7476641	2173	G	0	1.00	0.00	0.00	1.00	0.00	0.00	1.00	0.00	0.00	1.00	0.00	0.00	1.00	0.00	0.00	1.00	0.00	0.00
chrX	7539535	2353	G	0	1.00	0.00	0.00	1.00	0.00	0.00	1.00	0.00	0.00	1.00	0.00	0.00	1.00	0.00	0.00	1.00	0.00	0.00
chrX	7539563	2372	G	0	1.00	0.00	0.00	1.00	0.00	0.00	1.00	0.00	0.00	1.00	0.00	0.00	1.00	0.00	0.00	0.99	0.01	0.00
chr11	3525718	64	G	0	1.00	0.00	0.00	1.00	0.00	0.00	0.99	0.00	0.00	1.00	0.00	0.00	0.99	0.00	0.01	1.00	0.00	0.00
chr16	92644464	574	G	0	1.00	0.00	0.00	0.99	0.00	0.00	1.00	0.00	0.00	0.99	0.00	0.00	1.00	0.00	0.00	1.00	0.00	0.00
chr4	136547482	1095	C	0	1.00	0.00	0.00	1.00	0.00	0.00	1.00	0.00	0.00	1.00	0.00	0.00	1.00	0.00	0.00	1.00	0.00	0.00
chr8	89376906	1628	C	0	1.00	0.00	0.00	1.00	0.00	0.00	1.00	0.00	0.00	1.00	0.00	0.00	1.00	0.00	0.00	1.00	0.00	0.00
chr8	120115595	1712	G	0	1.00	0.00	0.00	1.00	0.00	0.00	1.00	0.00	0.00	1.00	0.00	0.00	1.00	0.00	0.00	1.00	0.00	0.00
chr8	120115638	1733	G	0	1.00	0.00	0.00	1.00	0.00	0.00	1.00	0.00	0.00	1.00	0.00	0.00	1.00	0.00	0.00	1.00	0.00	0.00
chr9	100498674	1984	c	0	1.00	0.00	0.00	1.00	0.00	0.00	1.00	0.00	0.00	1.00	0.00	0.00	1.00	0.00	0.00	0.99	0.01	0.00
chr15	100300021	363	G	0	1.00	0.00	0.00	1.00	0.00	0.00	1.00	0.00	0.00	1.00	0.00	0.00	0.99	0.00	0.00	1.00	0.00	0.00
chr15	100300187	437	G	0	1.00	0.00	0.00	0.99	0.01	0.00	1.00	0.00	0.00	1.00	0.00	0.00	1.00	0.00	0.00	1.00	0.00	0.00
chr9	100498617	1973	C	0	1.00	0.00	0.00	0.99	0.01	0.00	1.00	0.00	0.00	1.00	0.00	0.00	1.00	0.00	0.00	1.00	0.00	0.00
chrX	7476505	2075	C	1	1.00	0.00	0.00	1.00	0.00	0.00	1.00	0.00	0.00	1.00	0.00	0.00	0.99	0.00	0.01	0.99	0.01	0.00
chrX	7476554	2114	G	0	1.00	0.00	0.00	1.00	0.00	0.00	1.00	0.00	0.00	1.00	0.00	0.00	1.00	0.00	0.00	1.00	0.00	0.00
chrX	7476557	2117	G	1	1.00	0.00	0.00	1.00	0.00	0.00	1.00	0.00	0.00	0.98	0.00	0.01	1.00	0.00	0.00	0.99	0.00	0.01
chrX	7476620	2158	G	0	1.00	0.00	0.00	1.00	0.00	0.00	1.00	0.00	0.00	1.00	0.00	0.00	1.00	0.00	0.00	1.00	0.00	0.00
chrX	7539402	2272	C	0	1.00	0.00	0.00	1.00	0.00	0.00	1.00	0.00	0.00	1.00	0.00	0.00	1.00	0.00	0.00	1.00	0.00	0.00
chr11	3525714	61	G	0	1.00	0.00	0.00	1.00	0.00	0.00	1.00	0.00	0.00	0.99	0.00	0.00	1.00	0.00	0.00	1.00	0.00	0.00
chr15	100300116	410	G	0	1.00	0.00	0.00	1.00	0.00	0.00	1.00	0.00	0.00	1.00	0.00	0.00	0.99	0.00	0.00	1.00	0.00	0.00
chr16	92644300	511	G	0	0.99	0.00	0.00	0.99	0.01	0.01	1.00	0.00	0.00	0.99	0.00	0.00	1.00	0.00	0.00	1.00	0.00	0.00
chr16	92644442	560	G	0	0.99	0.00	0.00	1.00	0.00	0.00	1.00	0.00	0.00	0.99	0.00	0.00	1.00	0.00	0.00	1.00	0.00	0.00
chr17	45706783	866	G	0	1.00	0.00	0.00	0.99	0.01	0.00	1.00	0.00	0.00	1.00	0.00	0.00	1.00	0.00	0.00	1.00	0.00	0.00
chr4	136547374	1033	G	0	1.00	0.00	0.00	1.00	0.00	0.00	0.99	0.00	0.01	0.99	0.00	0.01	0.98	0.01	0.00	0.99	0.00	0.01
chr4	136547473	1090	C	0	1.00	0.00	0.00	1.00	0.00	0.00	1.00	0.00	0.00	1.00	0.00	0.00	1.00	0.00	0.00	1.00	0.00	0.00
chr4	136547753	1210	C	0	1.00	0.00	0.00	0.99	0.00	0.00	1.00	0.00	0.00	1.00	0.00	0.00	1.00	0.00	0.00	1.00	0.00	0.00
chr4	139783783	1331	C	0	1.00	0.00	0.00	1.00	0.00	0.00	1.00	0.00	0.00	1.00	0.00	0.00	1.00	0.00	0.00	1.00	0.00	0.00
chr4	139783801	1340	C	0	1.00	0.00	0.00	1.00	0.00	0.00	1.00	0.00	0.00	1.00	0.00	0.00	1.00	0.00	0.00	1.00	0.00	0.00
chr5	64987427	1454	G	0	1.00	0.00	0.00	1.00	0.00	0.00	1.00	0.00	0.00	1.00	0.00	0.00	1.00	0.00	0.00	1.00	0.00	0.00
chr8	89377050	1679	G	0	1.00	0.00	0.00	1.00	0.00	0.00	1.00	0.00	0.00	1.00	0.00	0.00	1.00	0.00	0.00	1.00	0.00	0.00
chr8	120115813	1815	G	0	1.00	0.00	0.00	1.00	0.00	0.00	1.00	0.00	0.00	1.00	0.00	0.00	1.00	0.00	0.00	1.00	0.00	0.00
chr9	100498508	1928	C	0	0.99	0.01	0.00	1.00	0.00	0.00	1.00	0.00	0.00	1.00	0.00	0.00	1.00	0.00	0.00	1.00	0.00	0.00
chr9	100498694	1993	c	0	1.00	0.00	0.00	1.00	0.00	0.00	1.00	0.00	0.00	1.00	0.00	0.00	1.00	0.00	0.00	1.00	0.00	0.00
chrX	7476306	2005	G	0	1.00	0.00	0.00	0.99	0.00	0.00	1.00	0.00	0.00	1.00	0.00	0.00	1.00	0.00	0.00	1.00	0.00	0.00
chrX	7476365	2018	G	1	1.00	0.00	0.00	1.00	0.00	0.00	1.00	0.00	0.00	0.99	0.00	0.01	1.00	0.00	0.00	1.00	0.00	0.00
chrX	7476664	2192	G	0	0.99	0.00	0.00	1.00	0.00	0.00	1.00	0.00	0.00	1.00	0.00	0.00	1.00	0.00	0.00	1.00	0.00	0.00
chrX	7539620	2405	G	0	1.00	0.00	0.00	1.00	0.00	0.00	0.99	0.01	0.00	1.00	0.00	0.00	1.00	0.00	0.00	1.00	0.00	0.00
chr11	3525705	56	C	0	0.99	0.00	0.00	0.99	0.01	0.00	1.00	0.00	0.00	1.00	0.00	0.00	1.00	0.00	0.00	1.00	0.00	0.00
chr15	61868676	213	C	0	1.00	0.00	0.00	1.00	0.00	0.00	0.99	0.00	0.00	1.00	0.00	0.00	1.00	0.00	0.00	1.00	0.00	0.00
chr15	100300054	379	G	0	1.00	0.00	0.00	1.00	0.00	0.00	1.00	0.00	0.00	1.00	0.00	0.00	1.00	0.00	0.00	1.00	0.00	0.00
chr15	100300094	400	G	0	1.00	0.00	0.00	1.00	0.00	0.00	1.00	0.00	0.00	1.00	0.00	0.00	1.00	0.00	0.00	1.00	0.00	0.00
chr4	139783611	1236	c	0	1.00	0.00	0.00	0.99	0.01	0.00	1.00	0.00	0.00	1.00	0.00	0.00	1.00	0.00	0.00	1.00	0.00	0.00

0.99	0.00	0.00	0.99	0.01	0.01	0,00016
0.99	0.01	0.00	0.99	0.01	0.00	0,00016
0.99	0.01	0.00	0.99	0.00	0.00	0,00016
0.99	0.01	0.00	0.99	0.00	0.00	0,00016
0.99	0.00	0.01	0.99	0.01	0.00	0,00016
0.99	0.01	0.00	0.99	0.01	0.00	0,00016
0.99	0.01	0.00	0.99	0.01	0.00	0,00016
0.99	0.00	0.01	0.99	0.00	0.01	0,00016
0.99	0.00	0.00	0.99	0.01	0.01	0,00016
0.99	0.00	0.01	0.99	0.00	0.00	0,00016
0.99	0.00	0.00	0.99	0.00	0.01	0,00016
0.99	0.01	0.00	0.99	0.01	0.00	0,00016
0.99	0.01	0.00	0.99	0.01	0.00	0,00016
0.99	0.00	0.00	0.99	0.00	0.00	0,00016
0.99	0.00	0.00	0.99	0.01	0.00	0,00016
0.99	0.01	0.00	0.99	0.00	0.00	0,00016
0.99	0.01	0.00	0.99	0.00	0.00	0,00016
0.99	0.00	0.00	0.99	0.00	0.01	0,00016
0.99	0.01	0.00	0.99	0.00	0.00	0,00016
0.99	0.00	0.00	0.99	0.00	0.01	0,00016
0.99	0.00	0.00	0.99	0.01	0.00	0,00016
0.99	0.00	0.00	0.99	0.01	0.01	0,00016
0.99	0.00	0.00	0.99	0.01	0.00	0,00016
0.99	0.00	0.00	0.99	0.00	0.01	0,00016
0.99	0.00	0.01	0.99	0.00	0.00	0,00016
0.99	0.01	0.01	0.99	0.00	0.01	0,00016
0.99	0.01	0.01	0.99	0.00	0.00	0,00016
0.99	0.00	0.00	0.99	0.01	0.01	0,00016
0.99	0.01	0.00	0.99	0.00	0.00	0,00016
0.99	0.00	0.01	0.99	0.00	0.00	0,00016
0.99	0.00	0.00	0.99	0.01	0.01	0,00016
0.99	0.00	0.00	0.99	0.00	0.00	0,00016
0.99	0.00	0.00	0.99	0.00	0.01	0,00016
0.99	0.00	0.00	0.99	0.01	0.01	0,00016
0.99	0.00	0.01	0.99	0.00	0.00	0,00016
0.99	0.01	0.00	0.99	0.00	0.00	0,00016
0.99	0.00	0.00	0.99	0.01	0.00	0,00016
0.99	0.01	0.00	0.99	0.01	0.00	0,00016
0.99	0.00	0.00	0.99	0.01	0.01	0,00016
0.99	0.00	0.01	0.99	0.00	0.00	0,00016
0.99	0.01	0.00	0.99	0.01	0.00	0,00016
0.99	0.01	0.00	0.99	0.01	0.00	0,00016
0.99	0.00	0.01	0.99	0.01	0.00	0,00016
0.99	0.00	0.01	0.99	0.01	0.00	0,00016
0.99	0.00	0.00	0.99	0.00	0.00	0,00016
0.99	0.01	0.00	0.99	0.00	0.01	0,00016

chr4	139783850	1366	C	0	1.00	0.00	0.00	1.00	0.00	0.00	1.00	0.00	0.00	1.00	0.00	0.00	1.00	0.00	0.00	1.00	0.00	0.00
chr8	89376833	1585	g	0	1.00	0.00	0.00	1.00	0.00	0.00	1.00	0.00	0.00	1.00	0.00	0.00	1.00	0.00	0.00	1.00	0.00	0.00
chr8	120115837	1829	C	0	1.00	0.00	0.00	1.00	0.00	0.00	1.00	0.00	0.00	1.00	0.00	0.00	1.00	0.00	0.00	1.00	0.00	0.00
chr11	3525849	140	C	0	1.00	0.00	0.00	1.00	0.00	0.00	0.99	0.00	0.01	1.00	0.00	0.00	0.99	0.01	0.00	0.99	0.00	0.01
chr11	3525863	152	G	0	1.00	0.00	0.00	1.00	0.00	0.00	1.00	0.00	0.00	1.00	0.00	0.00	1.00	0.00	0.00	1.00	0.00	0.00
chr4	136547338	1024	G	0	1.00	0.00	0.00	1.00	0.00	0.00	1.00	0.00	0.00	1.00	0.00	0.00	1.00	0.00	0.00	1.00	0.00	0.00
chr4	136547505	1109	G	0	1.00	0.00	0.00	1.00	0.00	0.00	1.00	0.00	0.00	1.00	0.00	0.00	1.00	0.00	0.00	1.00	0.00	0.00
chr4	139783665	1266	C	0	1.00	0.00	0.00	1.00	0.00	0.00	1.00	0.00	0.00	0.99	0.00	0.01	1.00	0.00	0.00	1.00	0.00	0.00
chr5	64987376	1431	C	0	1.00	0.00	0.00	1.00	0.00	0.00	1.00	0.00	0.00	1.00	0.00	0.00	1.00	0.00	0.00	1.00	0.00	0.00
chr8	89376831	1584	c	0	1.00	0.00	0.00	1.00	0.00	0.00	1.00	0.00	0.00	1.00	0.00	0.00	1.00	0.00	0.00	1.00	0.00	0.00
chr8	89376882	1615	c	0	0.99	0.01	0.00	0.99	0.00	0.00	0.99	0.00	0.00	1.00	0.00	0.00	1.00	0.00	0.00	1.00	0.00	0.00
chr8	89376887	1618	g	0	1.00	0.00	0.00	1.00	0.00	0.00	1.00	0.00	0.00	1.00	0.00	0.00	1.00	0.00	0.00	1.00	0.00	0.00
chrX	7539302	2222	G	0	1.00	0.00	0.00	1.00	0.00	0.00	1.00	0.00	0.00	1.00	0.00	0.00	1.00	0.00	0.00	1.00	0.00	0.00
chrX	7539332	2232	G	0	0.99	0.00	0.00	1.00	0.00	0.00	1.00	0.00	0.00	1.00	0.00	0.00	1.00	0.00	0.00	1.00	0.00	0.00
chr11	3525844	137	G	0	1.00	0.00	0.00	1.00	0.00	0.00	1.00	0.00	0.00	1.00	0.00	0.00	1.00	0.00	0.00	1.00	0.00	0.00
chr15	61868933	332	G	0	1.00	0.00	0.00	1.00	0.00	0.00	1.00	0.00	0.00	1.00	0.00	0.00	1.00	0.00	0.00	1.00	0.00	0.00
chr17	45706419	700	C	0	1.00	0.00	0.00	1.00	0.00	0.00	1.00	0.00	0.00	1.00	0.00	0.00	1.00	0.00	0.00	1.00	0.00	0.00
chr17	45706572	769	G	0	1.00	0.00	0.00	1.00	0.00	0.00	0.99	0.00	0.00	0.99	0.01	0.00	1.00	0.00	0.00	1.00	0.00	0.00
chr19	24277732	1006	G	0	1.00	0.00	0.00	1.00	0.00	0.00	0.99	0.00	0.01	1.00	0.00	0.00	1.00	0.00	0.00	1.00	0.00	0.00
chr4	136547417	1056	G	0	1.00	0.00	0.00	1.00	0.00	0.00	0.99	0.00	0.00	1.00	0.00	0.00	1.00	0.00	0.00	1.00	0.00	0.00
chr4	139783790	1334	G	0	1.00	0.00	0.00	1.00	0.00	0.00	1.00	0.00	0.00	1.00	0.00	0.00	1.00	0.00	0.00	1.00	0.00	0.00
chr5	64987516	1498	C	0	1.00	0.00	0.00	1.00	0.00	0.00	1.00	0.00	0.00	1.00	0.00	0.00	1.00	0.00	0.00	1.00	0.00	0.00
chr5	64987559	1518	C	0	1.00	0.00	0.00	0.99	0.00	0.00	0.99	0.00	0.00	1.00	0.00	0.00	1.00	0.00	0.00	1.00	0.00	0.00
chr8	120115761	1790	G	0	1.00	0.00	0.00	1.00	0.00	0.00	1.00	0.00	0.00	1.00	0.00	0.00	1.00	0.00	0.00	1.00	0.00	0.00
chr9	100498655	1981	C	0	0.99	0.00	0.01	1.00	0.00	0.00	1.00	0.00	0.00	1.00	0.00	0.00	1.00	0.00	0.00	1.00	0.00	0.00
chrX	7476348	2013	G	0	1.00	0.00	0.00	1.00	0.00	0.00	1.00	0.00	0.00	1.00	0.00	0.00	1.00	0.00	0.00	0.99	0.00	0.00
chrX	7539549	2364	C	0	0.99	0.01	0.00	1.00	0.00	0.00	0.99	0.00	0.00	0.99	0.00	0.00	1.00	0.00	0.00	0.99	0.01	0.00
chrX	7539579	2382	C	0	1.00	0.00	0.00	1.00	0.00	0.00	0.99	0.00	0.01	0.99	0.01	0.00	0.99	0.01	0.00	1.00	0.00	0.00
chrX	7539595	2392	C	0	1.00	0.00	0.00	1.00	0.00	0.00	1.00	0.00	0.00	1.00	0.00	0.00	1.00	0.00	0.00	1.00	0.00	0.00
chr17	45706488	723	G	0	1.00	0.00	0.00	1.00	0.00	0.00	0.99	0.00	0.00	1.00	0.00	0.00	1.00	0.00	0.00	1.00	0.00	0.00
chr4	136547524	1120	G	0	1.00	0.00	0.00	1.00	0.00	0.00	1.00	0.00	0.00	0.99	0.01	0.00	1.00	0.00	0.00	1.00	0.00	0.00
chr4	139783634	1248	G	0	1.00	0.00	0.00	1.00	0.00	0.00	1.00	0.00	0.00	1.00	0.00	0.00	1.00	0.00	0.00	1.00	0.00	0.00
chr4	139783819	1350	C	0	1.00	0.00	0.00	1.00	0.00	0.00	1.00	0.00	0.00	1.00	0.00	0.00	1.00	0.00	0.00	1.00	0.00	0.00
chr5	64987302	1402	G	0	1.00	0.00	0.00	1.00	0.00	0.00	1.00	0.00	0.00	1.00	0.00	0.00	1.00	0.00	0.00	1.00	0.00	0.00
chrX	7476305	2004	G	0	1.00	0.00	0.00	1.00	0.00	0.00	1.00	0.00	0.00	1.00	0.00	0.00	1.00	0.00	0.00	1.00	0.00	0.00
chrX	7476643	2175	C	1	1.00	0.00	0.00	1.00	0.00	0.00	0.99	0.01	0.00	0.99	0.00	0.00	0.99	0.00	0.01	0.99	0.01	0.00
chrX	7539448	2302	C	0	1.00	0.00	0.00	1.00	0.00	0.00	1.00	0.00	0.00	1.00	0.00	0.00	1.00	0.00	0.00	1.00	0.00	0.00
chr11	3525733	74	C	0	1.00	0.00	0.00	1.00	0.00	0.00	1.00	0.00	0.00	1.00	0.00	0.00	1.00	0.00	0.00	1.00	0.00	0.00
chr19	24277545	935	g	0	1.00	0.00	0.00	1.00	0.00	0.00	1.00	0.00	0.00	0.99	0.01	0.00	1.00	0.00	0.00	1.00	0.00	0.00
chr4	139783672	1270	G	0	1.00	0.00	0.00	1.00	0.00	0.00	1.00	0.00	0.00	1.00	0.00	0.00	1.00	0.00	0.00	1.00	0.00	0.00
chr4	139783682	1277	G	0	1.00	0.00	0.00	1.00	0.00	0.00	0.99	0.00	0.00	1.00	0.00	0.00	1.00	0.00	0.00	1.00	0.00	0.00
chr4	139783814	1347	G	0	1.00	0.00	0.00	1.00	0.00	0.00	1.00	0.00	0.00	1.00	0.00	0.00	0.99	0.01	0.00	1.00	0.00	0.00
chr5	64987569	1522	C	0	1.00	0.00	0.00	0.99	0.00	0.00	1.00	0.00	0.00	1.00	0.00	0.00	1.00	0.00	0.00	0.99	0.00	0.00
chrX	7476565	2122	G	0	1.00	0.00	0.00	1.00	0.00	0.00	1.00	0.00	0.00	1.00	0.00	0.00	1.00	0.00	0.00	1.00	0.00	0.00
chrX	7539394	2267	G	0	1.00	0.00	0.00	1.00	0.00	0.00	1.00	0.00	0.00	1.00	0.00	0.00	1.00	0.00	0.00	1.00	0.00	0.00
chr17	45706560	763	G	0	1.00	0.00	0.00	0.99	0.00	0.00	1.00	0.00	0.00	0.98	0.02	0.00	1.00	0.00	0.00	1.00	0.00	0.00

0.99	0.00	0.00	0.99	0.01	0.00	0,00016
0.99	0.01	0.00	0.99	0.00	0.00	0,00016
0.99	0.00	0.00	0.99	0.00	0.01	0,00016
0.99	0.00	0.01	0.99	0.00	0.00	0,00015
0.99	0.00	0.01	0.99	0.01	0.00	0,00015
0.99	0.00	0.00	0.99	0.01	0.00	0,00015
0.99	0.01	0.00	0.99	0.01	0.00	0,00015
0.99	0.01	0.00	0.99	0.00	0.01	0,00015
0.99	0.00	0.01	0.99	0.00	0.00	0,00015
0.99	0.01	0.00	0.99	0.00	0.00	0,00015
0.99	0.01	0.00	0.99	0.00	0.01	0,00015
0.99	0.00	0.00	0.99	0.01	0.00	0,00015
0.99	0.00	0.00	0.99	0.00	0.00	0,00015
0.99	0.01	0.00	0.99	0.01	0.00	0,00015
0.99	0.01	0.00	0.99	0.00	0.01	0,00015
0.99	0.00	0.00	0.99	0.00	0.00	0,00015
0.99	0.00	0.00	0.99	0.01	0.00	0,00015
0.99	0.00	0.00	0.99	0.01	0.01	0,00015
0.99	0.00	0.01	0.99	0.00	0.00	0,00015
0.99	0.00	0.00	0.99	0.01	0.00	0,00015
0.99	0.01	0.00	0.99	0.00	0.00	0,00015
0.99	0.00	0.01	0.99	0.00	0.00	0,00015
0.99	0.00	0.01	0.99	0.00	0.00	0,00015
0.99	0.01	0.00	0.99	0.00	0.00	0,00015
0.99	0.00	0.00	0.99	0.00	0.01	0,00015
0.99	0.00	0.00	0.99	0.00	0.00	0,00015
0.99	0.01	0.00	0.99	0.00	0.00	0,00015
0.99	0.00	0.00	0.99	0.01	0.00	0,00015
0.99	0.01	0.00	0.99	0.01	0.00	0,00015
0.99	0.00	0.01	0.99	0.01	0.00	0,00015
0.99	0.00	0.00	0.99	0.01	0.00	0,00015
0.99	0.01	0.00	0.99	0.00	0.00	0,00015
0.99	0.00	0.00	0.99	0.00	0.01	0,00015
0.99	0.00	0.00	0.99	0.00	0.01	0,00015
0.99	0.01	0.01	0.99	0.00	0.00	0,00015
0.99	0.01	0.00	0.99	0.01	0.01	0,00015
0.99	0.01	0.01	0.99	0.00	0.00	0,00015
0.99	0.01	0.00	0.99	0.00	0.00	0,00015
0.99	0.00	0.01	0.99	0.00	0.00	0,00015
0.99	0.01	0.00	0.99	0.01	0.00	0,00015
0.99	0.00	0.01	0.99	0.01	0.00	0,00015
0.99	0.01	0.00	0.99	0.01	0.00	0,00015
0.99	0.00	0.01	0.99	0.00	0.00	0,00015
0.99	0.01	0.01	0.99	0.00	0.00	0,00015
0.99	0.01	0.00	0.99	0.00	0.01	0,00015
0.99	0.00	0.00	0.99	0.01	0.00	0,00015

chr17	45706714	838	G	0	1.00	0.00	0.00	0.99	0.00	0.00	1.00	0.00	0.00	1.00	0.00	0.00	1.00	0.00	0.00	1.00	0.00	0.00
chr4	136547448	1075	G	0	1.00	0.00	0.00	1.00	0.00	0.00	1.00	0.00	0.00	1.00	0.00	0.00	1.00	0.00	0.00	1.00	0.00	0.00
chr4	136547459	1082	G	0	1.00	0.00	0.00	1.00	0.00	0.00	1.00	0.00	0.00	1.00	0.00	0.00	1.00	0.00	0.00	1.00	0.00	0.00
chr4	139783810	1345	C	0	1.00	0.00	0.00	1.00	0.00	0.00	0.99	0.01	0.00	0.99	0.00	0.01	1.00	0.00	0.00	1.00	0.00	0.00
chr8	120115631	1730	G	0	1.00	0.00	0.00	1.00	0.00	0.00	1.00	0.00	0.00	1.00	0.00	0.00	1.00	0.00	0.00	1.00	0.00	0.00
chr8	120115639	1734	G	0	1.00	0.00	0.00	1.00	0.00	0.00	0.99	0.00	0.00	1.00	0.00	0.00	1.00	0.00	0.00	1.00	0.00	0.00
chrX	7476629	2164	G	1	1.00	0.00	0.00	1.00	0.00	0.00	1.00	0.00	0.00	0.99	0.00	0.00	1.00	0.00	0.00	1.00	0.00	0.00
chrX	7539315	2228	G	0	1.00	0.00	0.00	1.00	0.00	0.00	0.99	0.00	0.01	1.00	0.00	0.00	0.99	0.00	0.00	1.00	0.00	0.00
chrX	7539546	2361	G	0	1.00	0.00	0.00	1.00	0.00	0.00	1.00	0.00	0.00	1.00	0.00	0.00	1.00	0.00	0.00	1.00	0.00	0.00
chr11	3525880	163	C	0	1.00	0.00	0.00	1.00	0.00	0.00	1.00	0.00	0.00	1.00	0.00	0.00	1.00	0.00	0.00	1.00	0.00	0.00
chr15	61868708	227	C	0	1.00	0.00	0.00	1.00	0.00	0.00	1.00	0.00	0.00	0.99	0.01	0.00	1.00	0.00	0.00	1.00	0.00	0.00
chr15	61868847	296	G	0	1.00	0.00	0.00	0.99	0.00	0.00	1.00	0.00	0.00	1.00	0.00	0.00	1.00	0.00	0.00	1.00	0.00	0.00
chr15	100300053	378	G	0	1.00	0.00	0.00	1.00	0.00	0.00	1.00	0.00	0.00	1.00	0.00	0.00	1.00	0.00	0.00	1.00	0.00	0.00
chr17	45706463	708	C	0	1.00	0.00	0.00	1.00	0.00	0.00	1.00	0.00	0.00	1.00	0.00	0.00	1.00	0.00	0.00	1.00	0.00	0.00
chr19	24277466	892	c	0	1.00	0.00	0.00	1.00	0.00	0.00	1.00	0.00	0.00	1.00	0.00	0.00	1.00	0.00	0.00	1.00	0.00	0.00
chr4	139783608	1235	c	0	1.00	0.00	0.00	1.00	0.00	0.00	1.00	0.00	0.00	1.00	0.00	0.00	1.00	0.00	0.00	1.00	0.00	0.00
chr5	64987417	1445	C	0	1.00	0.00	0.00	0.99	0.00	0.00	1.00	0.00	0.00	1.00	0.00	0.00	1.00	0.00	0.00	1.00	0.00	0.00
chr8	89377026	1669	G	0	1.00	0.00	0.00	1.00	0.00	0.00	0.99	0.00	0.00	1.00	0.00	0.00	1.00	0.00	0.00	1.00	0.00	0.00
chr9	100498383	1859	G	0	1.00	0.00	0.00	1.00	0.00	0.00	1.00	0.00	0.00	1.00	0.00	0.00	1.00	0.00	0.00	1.00	0.00	0.00
chr9	100498678	1986	c	0	1.00	0.00	0.00	1.00	0.00	0.00	1.00	0.00	0.00	1.00	0.00	0.00	1.00	0.00	0.00	1.00	0.00	0.00
chrX	7539390	2266	G	0	1.00	0.00	0.00	1.00	0.00	0.00	1.00	0.00	0.00	1.00	0.00	0.00	1.00	0.00	0.00	1.00	0.00	0.00
chrX	7539628	2411	C	0	1.00	0.00	0.00	1.00	0.00	0.00	1.00	0.00	0.00	1.00	0.00	0.00	1.00	0.00	0.00	1.00	0.00	0.00
chr15	61868879	308	C	0	1.00	0.00	0.00	1.00	0.00	0.00	1.00	0.00	0.00	1.00	0.00	0.00	1.00	0.00	0.00	1.00	0.00	0.00
chr15	100300268	453	G	0	1.00	0.00	0.00	1.00	0.00	0.00	1.00	0.00	0.00	1.00	0.00	0.00	1.00	0.00	0.00	1.00	0.00	0.00
chr17	45706592	782	C	0	1.00	0.00	0.00	1.00	0.00	0.00	1.00	0.00	0.00	0.99	0.00	0.00	1.00	0.00	0.00	1.00	0.00	0.00
chr17	45706726	849	G	0	1.00	0.00	0.00	0.99	0.00	0.00	1.00	0.00	0.00	1.00	0.00	0.00	1.00	0.00	0.00	1.00	0.00	0.00
chr19	24277451	889	c	0	0.99	0.00	0.01	1.00	0.00	0.00	1.00	0.00	0.00	1.00	0.00	0.00	0.99	0.01	0.00	1.00	0.00	0.00
chr4	136547440	1069	G	0	0.99	0.01	0.00	1.00	0.00	0.00	1.00	0.00	0.00	1.00	0.00	0.00	1.00	0.00	0.00	1.00	0.00	0.00
chr4	136547642	1180	C	0	1.00	0.00	0.00	1.00	0.00	0.00	1.00	0.00	0.00	1.00	0.00	0.00	1.00	0.00	0.00	1.00	0.00	0.00
chr4	139783773	1325	C	0	1.00	0.00	0.00	1.00	0.00	0.00	1.00	0.00	0.00	1.00	0.00	0.00	1.00	0.00	0.00	1.00	0.00	0.00
chr4	139783866	1378	G	0	1.00	0.00	0.00	1.00	0.00	0.00	1.00	0.00	0.00	1.00	0.00	0.00	1.00	0.00	0.00	1.00	0.00	0.00
chr8	89376851	1595	c	0	1.00	0.00	0.00	1.00	0.00	0.00	0.99	0.00	0.00	1.00	0.00	0.00	1.00	0.00	0.00	1.00	0.00	0.00
chr8	120115762	1791	G	0	1.00	0.00	0.00	1.00	0.00	0.00	1.00	0.00	0.00	1.00	0.00	0.00	1.00	0.00	0.00	1.00	0.00	0.00
chr9	100498521	1935	G	0	1.00	0.00	0.00	1.00	0.00	0.00	1.00	0.00	0.00	1.00	0.00	0.00	1.00	0.00	0.00	1.00	0.00	0.00
chrX	7476637	2171	C	0	1.00	0.00	0.00	1.00	0.00	0.00	1.00	0.00	0.00	1.00	0.00	0.00	0.99	0.00	0.01	1.00	0.00	0.00
chrX	7476639	2172	C	0	1.00	0.00	0.00	1.00	0.00	0.00	1.00	0.00	0.00	1.00	0.00	0.00	0.99	0.00	0.01	1.00	0.00	0.00
chrX	7476655	2183	G	0	1.00	0.00	0.00	1.00	0.00	0.00	1.00	0.00	0.00	1.00	0.00	0.00	1.00	0.00	0.00	1.00	0.00	0.00
chrX	7539304	2224	G	0	1.00	0.00	0.00	0.99	0.01	0.00	1.00	0.00	0.00	1.00	0.00	0.00	1.00	0.00	0.00	1.00	0.00	0.00
chrX	7539401	2271	C	0	1.00	0.00	0.00	1.00	0.00	0.00	1.00	0.00	0.00	1.00	0.00	0.00	0.98	0.02	0.00	1.00	0.00	0.00
chr11	3525746	81	C	0	1.00	0.00	0.00	1.00	0.00	0.00	1.00	0.00	0.00	1.00	0.00	0.00	1.00	0.00	0.00	1.00	0.00	0.00
chr17	45706533	747	G	0	1.00	0.00	0.00	1.00	0.00	0.00	1.00	0.00	0.00	1.00	0.00	0.00	1.00	0.00	0.00	0.99	0.00	0.00
chr4	139783657	1263	C	0	1.00	0.00	0.00	1.00	0.00	0.00	1.00	0.00	0.00	1.00	0.00	0.00	1.00	0.00	0.00	1.00	0.00	0.00
chr4	139783701	1288	C	0	1.00	0.00	0.00	1.00	0.00	0.00	1.00	0.00	0.00	1.00	0.00	0.00	1.00	0.00	0.00	1.00	0.00	0.00
chr8	89376928	1636	G	0	1.00	0.00	0.00	1.00	0.00	0.00	1.00	0.00	0.00	1.00	0.00	0.00	1.00	0.00	0.00	1.00	0.00	0.00
chrX	7476349	2014	G	0	1.00	0.00	0.00	1.00	0.00	0.00	1.00	0.00	0.00	1.00	0.00	0.00	1.00	0.00	0.00	1.00	0.00	0.00
chrX	7539344	2238	G	0	1.00	0.00	0.00	1.00	0.00	0.00	1.00	0.00	0.00	1.00	0.00	0.00	1.00	0.00	0.00	1.00	0.00	0.00

0.99	0.00	0.00	0.99	0.00	0.00	0,00015
0.99	0.00	0.00	0.99	0.00	0.00	0,00015
0.99	0.01	0.00	0.99	0.00	0.00	0,00015
0.99	0.01	0.00	0.99	0.01	0.00	0,00015
0.99	0.00	0.01	0.99	0.01	0.00	0,00015
0.99	0.01	0.00	0.99	0.00	0.00	0,00015
0.99	0.00	0.00	0.99	0.01	0.00	0,00015
0.99	0.00	0.00	0.99	0.00	0.00	0,00015
0.99	0.00	0.00	0.99	0.01	0.00	0,00015
0.99	0.00	0.00	0.99	0.01	0.00	0,00015
0.99	0.00	0.00	0.99	0.01	0.00	0,00015
0.99	0.00	0.00	0.99	0.00	0.00	0,00015
0.99	0.00	0.01	0.99	0.00	0.00	0,00015
0.99	0.00	0.00	0.99	0.00	0.01	0,00015
0.99	0.00	0.00	0.99	0.00	0.00	0,00015
0.99	0.00	0.00	0.99	0.00	0.00	0,00015
0.99	0.00	0.00	0.99	0.00	0.01	0,00015
0.99	0.00	0.00	0.99	0.01	0.00	0,00015
0.99	0.00	0.00	0.99	0.01	0.00	0,00015
0.99	0.00	0.00	0.99	0.00	0.01	0,00015
0.99	0.01	0.00	0.99	0.00	0.00	0,00015
0.99	0.00	0.00	0.99	0.01	0.00	0,00015
0.99	0.00	0.00	0.99	0.00	0.01	0,00015
0.99	0.00	0.00	0.99	0.00	0.00	0,00015
0.99	0.00	0.00	0.99	0.00	0.00	0,00015
0.99	0.00	0.01	0.99	0.00	0.00	0,00015
0.99	0.00	0.01	0.99	0.01	0.00	0,00015
0.99	0.00	0.00	0.99	0.01	0.00	0,00015
0.99	0.00	0.01	0.99	0.00	0.00	0,00015
0.99	0.00	0.00	0.99	0.00	0.00	0,00015
0.99	0.00	0.01	0.99	0.00	0.00	0,00015
0.99	0.00	0.01	0.99	0.00	0.01	0,00015
0.99	0.00	0.00	0.99	0.01	0.00	0,00015
0.99	0.00	0.00	0.99	0.01	0.00	0,00015
0.99	0.01	0.00	0.99	0.01	0.00	0,00015
0.99	0.00	0.00	0.99	0.00	0.01	0,00015
0.99	0.00	0.00	0.99	0.01	0.00	0,00015
0.99	0.01	0.00	0.99	0.00	0.01	0,00015
0.99	0.00	0.00	0.99	0.01	0.00	0,00015
0.99	0.01	0.00	0.99	0.00	0.00	0,00015
0.99	0.00	0.00	0.99	0.01	0.00	0,00015
0.99	0.00	0.00	0.99	0.01	0.00	0,00015
0.99	0.00	0.00	0.99	0.01	0.00	0,00015
0.99	0.00	0.00	0.99	0.00	0.00	0,00015
0.99	0.00	0.01	0.99	0.00	0.00	0,00015
0.99	0.01	0.00	0.99	0.00	0.00	0,00015

chrX	7539575	2379	G	0	1.00	0.00	0.00	1.00	0.00	0.00	1.00	0.00	0.00	0.99	0.00	0.01	1.00	0.00	0.00	1.00	0.00	0.00
chr11	3525807	116	C	0	1.00	0.00	0.00	1.00	0.00	0.00	1.00	0.00	0.00	1.00	0.00	0.00	1.00	0.00	0.00	1.00	0.00	0.00
chr15	100300115	409	G	0	1.00	0.00	0.00	1.00	0.00	0.00	1.00	0.00	0.00	1.00	0.00	0.00	1.00	0.00	0.00	1.00	0.00	0.00
chr4	139783655	1262	G	0	1.00	0.00	0.00	1.00	0.00	0.00	1.00	0.00	0.00	1.00	0.00	0.00	1.00	0.00	0.00	0.99	0.01	0.00
chr4	139783667	1268	C	0	1.00	0.00	0.00	1.00	0.00	0.00	1.00	0.00	0.00	1.00	0.00	0.00	1.00	0.00	0.00	1.00	0.00	0.00
chr9	100498462	1900	G	0	1.00	0.00	0.00	1.00	0.00	0.00	1.00	0.00	0.00	1.00	0.00	0.00	1.00	0.00	0.00	1.00	0.00	0.00
chrX	7476663	2191	G	0	1.00	0.00	0.00	1.00	0.00	0.00	1.00	0.00	0.00	1.00	0.00	0.00	1.00	0.00	0.00	1.00	0.00	0.00
chrX	7539534	2352	G	0	1.00	0.00	0.00	1.00	0.00	0.00	1.00	0.00	0.00	1.00	0.00	0.00	1.00	0.00	0.00	1.00	0.00	0.00
chr15	61868709	228	C	0	1.00	0.00	0.00	1.00	0.00	0.00	1.00	0.00	0.00	1.00	0.00	0.00	1.00	0.00	0.00	1.00	0.00	0.00
chr15	61868910	325	G	0	1.00	0.00	0.00	1.00	0.00	0.00	1.00	0.00	0.00	1.00	0.00	0.00	1.00	0.00	0.00	0.99	0.01	0.00
chr4	136547466	1086	C	0	1.00	0.00	0.00	1.00	0.00	0.00	1.00	0.00	0.00	1.00	0.00	0.00	1.00	0.00	0.00	1.00	0.00	0.00
chr8	89376842	1589	g	0	1.00	0.00	0.00	1.00	0.00	0.00	1.00	0.00	0.00	1.00	0.00	0.00	1.00	0.00	0.00	1.00	0.00	0.00
chr9	100498649	1978	C	0	1.00	0.00	0.00	1.00	0.00	0.00	1.00	0.00	0.00	1.00	0.00	0.00	1.00	0.00	0.00	1.00	0.00	0.00
chr9	100498654	1980	C	0	0.99	0.00	0.01	1.00	0.00	0.00	1.00	0.00	0.00	1.00	0.00	0.00	1.00	0.00	0.00	1.00	0.00	0.00
chrX	7476657	2185	C	1	1.00	0.00	0.00	1.00	0.00	0.00	1.00	0.00	0.00	0.99	0.01	0.00	0.99	0.00	0.01	0.99	0.01	0.00
chrX	7476659	2187	G	0	1.00	0.00	0.00	1.00	0.00	0.00	1.00	0.00	0.00	1.00	0.00	0.00	1.00	0.00	0.00	1.00	0.00	0.00
chrX	7539333	2233	G	0	1.00	0.00	0.00	1.00	0.00	0.00	1.00	0.00	0.00	1.00	0.00	0.00	1.00	0.00	0.00	1.00	0.00	0.00
chrX	7539541	2358	G	0	1.00	0.00	0.00	1.00	0.00	0.00	1.00	0.00	0.00	1.00	0.00	0.00	1.00	0.00	0.00	1.00	0.00	0.00
chrX	7539560	2370	C	0	1.00	0.00	0.00	1.00	0.00	0.00	0.99	0.00	0.00	1.00	0.00	0.00	1.00	0.00	0.00	1.00	0.00	0.00
chr11	3525686	46	C	0	1.00	0.00	0.00	1.00	0.00	0.00	1.00	0.00	0.00	1.00	0.00	0.00	1.00	0.00	0.00	1.00	0.00	0.00
chr11	3525731	72	C	0	1.00	0.00	0.00	1.00	0.00	0.00	1.00	0.00	0.00	1.00	0.00	0.00	1.00	0.00	0.00	1.00	0.00	0.00
chr11	3525762	88	C	0	1.00	0.00	0.00	1.00	0.00	0.00	1.00	0.00	0.00	1.00	0.00	0.00	1.00	0.00	0.00	1.00	0.00	0.00
chr16	92644437	557	G	0	1.00	0.00	0.00	0.99	0.01	0.00	1.00	0.00	0.00	1.00	0.00	0.00	1.00	0.00	0.00	1.00	0.00	0.00
chr4	136547777	1217	C	0	1.00	0.00	0.00	1.00	0.00	0.00	1.00	0.00	0.00	1.00	0.00	0.00	1.00	0.00	0.00	1.00	0.00	0.00
chr4	139783873	1382	G	0	1.00	0.00	0.00	1.00	0.00	0.00	1.00	0.00	0.00	1.00	0.00	0.00	1.00	0.00	0.00	1.00	0.00	0.00
chr5	64987554	1514	G	0	1.00	0.00	0.00	1.00	0.00	0.00	1.00	0.00	0.00	0.99	0.00	0.00	1.00	0.00	0.00	1.00	0.00	0.00
chrX	7476487	2064	C	0	1.00	0.00	0.00	1.00	0.00	0.00	1.00	0.00	0.00	1.00	0.00	0.00	0.99	0.00	0.01	1.00	0.00	0.00
chrX	7476568	2125	C	0	1.00	0.00	0.00	1.00	0.00	0.00	1.00	0.00	0.00	1.00	0.00	0.00	0.98	0.00	0.01	1.00	0.00	0.00
chrX	7476613	2153	G	0	1.00	0.00	0.00	1.00	0.00	0.00	1.00	0.00	0.00	1.00	0.00	0.00	1.00	0.00	0.00	1.00	0.00	0.00
chrX	7476615	2155	C	0	1.00	0.00	0.00	1.00	0.00	0.00	1.00	0.00	0.00	1.00	0.00	0.00	0.99	0.00	0.01	1.00	0.00	0.00
chrX	7539311	2226	G	0	1.00	0.00	0.00	1.00	0.00	0.00	1.00	0.00	0.00	1.00	0.00	0.00	0.99	0.00	0.01	1.00	0.00	0.00
chr15	61868689	219	C	0	1.00	0.00	0.00	1.00	0.00	0.00	1.00	0.00	0.00	1.00	0.00	0.00	1.00	0.00	0.00	1.00	0.00	0.00
chr17	45706725	848	G	0	1.00	0.00	0.00	1.00	0.00	0.00	1.00	0.00	0.00	1.00	0.00	0.00	1.00	0.00	0.00	0.99	0.01	0.00
chr19	24277425	886	c	0	1.00	0.00	0.00	1.00	0.00	0.00	1.00	0.00	0.00	1.00	0.00	0.00	0.99	0.00	0.00	0.99	0.01	0.00
chr4	139783693	1282	G	0	1.00	0.00	0.00	1.00	0.00	0.00	1.00	0.00	0.00	1.00	0.00	0.00	1.00	0.00	0.00	1.00	0.00	0.00
chr4	139783804	1342	C	0	1.00	0.00	0.00	1.00	0.00	0.00	1.00	0.00	0.00	1.00	0.00	0.00	1.00	0.00	0.00	1.00	0.00	0.00
chr8	89377070	1689	G	0	1.00	0.00	0.00	1.00	0.00	0.00	1.00	0.00	0.00	1.00	0.00	0.00	1.00	0.00	0.00	1.00	0.00	0.00
chr8	120115632	1731	G	0	1.00	0.00	0.00	1.00	0.00	0.00	1.00	0.00	0.00	1.00	0.00	0.00	1.00	0.00	0.00	1.00	0.00	0.00
chr9	100498414	1874	C	0	1.00	0.00	0.00	1.00	0.00	0.00	1.00	0.00	0.00	1.00	0.00	0.00	1.00	0.00	0.00	1.00	0.00	0.00
chrX	7476415	2031	G	0	1.00	0.00	0.00	1.00	0.00	0.00	1.00	0.00	0.00	1.00	0.00	0.00	1.00	0.00	0.00	1.00	0.00	0.00
chrX	7476534	2098	C	1	1.00	0.00	0.00	1.00	0.00	0.00	1.00	0.00	0.00	0.99	0.01	0.00	0.99	0.00	0.01	0.99	0.01	0.00
chrX	7476607	2149	G	0	1.00	0.00	0.00	1.00	0.00	0.00	1.00	0.00	0.00	1.00	0.00	0.00	1.00	0.00	0.00	1.00	0.00	0.00
chrX	7476669	2195	G	0	1.00	0.00	0.00	1.00	0.00	0.00	1.00	0.00	0.00	0.99	0.00	0.00	1.00	0.00	0.00	1.00	0.00	0.00
chrX	7539303	2223	G	0	1.00	0.00	0.00	1.00	0.00	0.00	1.00	0.00	0.00	1.00	0.00	0.00	1.00	0.00	0.00	1.00	0.00	0.00
chrX	7539629	2412	C	0	1.00	0.00	0.00	1.00	0.00	0.00	1.00	0.00	0.00	1.00	0.00	0.00	1.00	0.00	0.00	1.00	0.00	0.00
chr15	100300044	374	G	0	1.00	0.00	0.00	1.00	0.00	0.00	1.00	0.00	0.00	1.00	0.00	0.00	1.00	0.00	0.00	1.00	0.00	0.00

0.99	0.00	0.00	0.99	0.00	0.01	0,00015
0.99	0.01	0.00	0.99	0.00	0.00	0,00015
0.99	0.00	0.00	0.99	0.01	0.00	0,00015
0.99	0.01	0.00	0.99	0.01	0.00	0,00015
0.99	0.00	0.01	0.99	0.00	0.00	0,00015
0.99	0.01	0.00	0.99	0.01	0.00	0,00015
0.99	0.00	0.00	0.99	0.00	0.00	0,00015
0.99	0.00	0.00	0.99	0.00	0.00	0,00015
0.99	0.01	0.00	0.99	0.01	0.00	0,00015
0.99	0.00	0.00	0.99	0.01	0.00	0,00015
0.99	0.00	0.00	0.99	0.01	0.00	0,00015
0.99	0.00	0.00	0.99	0.01	0.00	0,00015
0.99	0.00	0.00	0.99	0.01	0.00	0,00015
0.99	0.00	0.00	0.99	0.01	0.00	0,00015
0.99	0.00	0.00	0.99	0.01	0.00	0,00015
0.99	0.00	0.00	0.99	0.01	0.00	0,00015
0.99	0.01	0.00	0.99	0.01	0.00	0,00015
0.99	0.01	0.00	0.99	0.00	0.00	0,00015
0.99	0.00	0.00	0.99	0.00	0.00	0,00015
0.99	0.00	0.00	0.99	0.00	0.00	0,00014
0.99	0.01	0.00	0.99	0.00	0.00	0,00014
0.99	0.00	0.01	0.99	0.00	0.00	0,00014
0.99	0.01	0.01	0.99	0.00	0.00	0,00014
0.99	0.00	0.01	0.99	0.00	0.01	0,00014
0.99	0.01	0.00	0.99	0.01	0.00	0,00014
0.99	0.00	0.00	0.99	0.01	0.00	0,00014
0.99	0.00	0.00	0.99	0.00	0.00	0,00014
0.99	0.00	0.00	0.99	0.01	0.00	0,00014
0.99	0.00	0.00	0.99	0.00	0.01	0,00014
0.99	0.01	0.00	0.99	0.01	0.00	0,00014
0.99	0.00	0.00	0.99	0.01	0.01	0,00014
0.99	0.00	0.00	0.99	0.01	0.00	0,00014
0.99	0.00	0.01	0.99	0.01	0.00	0,00014
0.99	0.00	0.00	0.99	0.01	0.00	0,00014
0.99	0.00	0.00	0.99	0.00	0.00	0,00014
0.99	0.01	0.00	0.99	0.00	0.00	0,00014
0.99	0.00	0.00	0.99	0.00	0.00	0,00014
0.99	0.01	0.00	0.99	0.00	0.00	0,00014
0.99	0.00	0.00	0.99	0.00	0.00	0,00014
0.99	0.01	0.00	0.99	0.00	0.00	0,00014
0.99	0.00	0.00	0.99	0.01	0.01	0,00014
0.99	0.00	0.00	0.99	0.01	0.00	0,00014
0.99	0.01	0.00	0.99	0.01	0.00	0,00014
0.99	0.00	0.00	0.99	0.01	0.00	0,00014
0.99	0.00	0.00	0.99	0.01	0.00	0,00014
0.99	0.00	0.00	0.99	0.00	0.00	0,00014

chr4	139783832	1357	C	0	1.00	0.00	0.00	0.99	0.01	0.00	1.00	0.00	0.00	1.00	0.00	0.00	1.00	0.00	0.00	1.00	0.00	0.00
chr5	64987389	1438	C	0	1.00	0.00	0.00	1.00	0.00	0.00	1.00	0.00	0.00	1.00	0.00	0.00	0.99	0.01	0.00	1.00	0.00	0.00
chr5	64987574	1524	G	0	1.00	0.00	0.00	1.00	0.00	0.00	1.00	0.00	0.00	1.00	0.00	0.00	1.00	0.00	0.00	1.00	0.00	0.00
chr9	100498382	1858	G	0	1.00	0.00	0.00	1.00	0.00	0.00	1.00	0.00	0.00	1.00	0.00	0.00	1.00	0.00	0.00	1.00	0.00	0.00
chrX	7476538	2102	C	0	1.00	0.00	0.00	1.00	0.00	0.00	1.00	0.00	0.00	1.00	0.00	0.00	0.99	0.00	0.01	1.00	0.00	0.00
chr11	3525797	111	C	0	1.00	0.00	0.00	1.00	0.00	0.00	1.00	0.00	0.00	1.00	0.00	0.00	1.00	0.00	0.00	1.00	0.00	0.00
chr11	3525800	112	C	0	1.00	0.00	0.00	1.00	0.00	0.00	1.00	0.00	0.00	1.00	0.00	0.00	1.00	0.00	0.00	1.00	0.00	0.00
chr19	24277365	875	c	0	1.00	0.00	0.00	1.00	0.00	0.00	1.00	0.00	0.00	1.00	0.00	0.00	1.00	0.00	0.00	1.00	0.00	0.00
chr19	24277396	880	c	0	1.00	0.00	0.00	1.00	0.00	0.00	1.00	0.00	0.00	1.00	0.00	0.00	1.00	0.00	0.00	1.00	0.00	0.00
chr4	136547504	1108	G	0	1.00	0.00	0.00	1.00	0.00	0.00	1.00	0.00	0.00	1.00	0.00	0.00	1.00	0.00	0.00	1.00	0.00	0.00
chr8	89376965	1648	G	0	1.00	0.00	0.00	1.00	0.00	0.00	1.00	0.00	0.00	1.00	0.00	0.00	0.99	0.00	0.00	1.00	0.00	0.00
chr8	120115673	1750	G	0	1.00	0.00	0.00	1.00	0.00	0.00	1.00	0.00	0.00	1.00	0.00	0.00	1.00	0.00	0.00	1.00	0.00	0.00
chr9	100498427	1881	G	0	1.00	0.00	0.00	1.00	0.00	0.00	1.00	0.00	0.00	1.00	0.00	0.00	1.00	0.00	0.00	1.00	0.00	0.00
chrX	7476611	2152	C	0	1.00	0.00	0.00	1.00	0.00	0.00	1.00	0.00	0.00	1.00	0.00	0.00	0.99	0.00	0.01	1.00	0.00	0.00
chr11	3525649	31	C	0	1.00	0.00	0.00	1.00	0.00	0.00	1.00	0.00	0.00	1.00	0.00	0.00	1.00	0.00	0.00	1.00	0.00	0.00
chr11	3525702	54	G	0	1.00	0.00	0.00	1.00	0.00	0.00	1.00	0.00	0.00	1.00	0.00	0.00	1.00	0.00	0.00	1.00	0.00	0.00
chr11	3525720	66	C	0	1.00	0.00	0.00	1.00	0.00	0.00	0.99	0.01	0.00	1.00	0.00	0.00	1.00	0.00	0.00	1.00	0.00	0.00
chr11	3525852	141	G	0	1.00	0.00	0.00	1.00	0.00	0.00	0.99	0.01	0.00	1.00	0.00	0.00	1.00	0.00	0.00	1.00	0.00	0.00
chr15	100300032	369	C	0	0.99	0.00	0.00	0.99	0.00	0.00	0.99	0.00	0.01	0.99	0.00	0.00	1.00	0.00	0.00	1.00	0.00	0.00
chr15	100300199	441	G	0	1.00	0.00	0.00	1.00	0.00	0.00	1.00	0.00	0.00	1.00	0.00	0.00	1.00	0.00	0.00	1.00	0.00	0.00
chr17	45706418	699	C	0	1.00	0.00	0.00	1.00	0.00	0.00	1.00	0.00	0.00	1.00	0.00	0.00	1.00	0.00	0.00	1.00	0.00	0.00
chr17	45706464	709	C	0	1.00	0.00	0.00	1.00	0.00	0.00	1.00	0.00	0.00	1.00	0.00	0.00	1.00	0.00	0.00	1.00	0.00	0.00
chr17	45706493	726	C	0	1.00	0.00	0.00	1.00	0.00	0.00	1.00	0.00	0.00	1.00	0.00	0.00	1.00	0.00	0.00	1.00	0.00	0.00
chr19	24277607	964	G	0	1.00	0.00	0.00	1.00	0.00	0.00	1.00	0.00	0.00	1.00	0.00	0.00	1.00	0.00	0.00	1.00	0.00	0.00
chr5	64987611	1534	G	0	1.00	0.00	0.00	1.00	0.00	0.00	1.00	0.00	0.00	0.99	0.00	0.00	1.00	0.00	0.00	1.00	0.00	0.00
chr9	100498396	1866	G	0	1.00	0.00	0.00	0.99	0.00	0.00	1.00	0.00	0.00	1.00	0.00	0.00	1.00	0.00	0.00	1.00	0.00	0.00
chr9	100498428	1882	G	0	0.99	0.00	0.00	1.00	0.00	0.00	1.00	0.00	0.00	1.00	0.00	0.00	0.99	0.01	0.00	1.00	0.00	0.00
chrX	7476625	2161	G	0	1.00	0.00	0.00	1.00	0.00	0.00	1.00	0.00	0.00	1.00	0.00	0.00	1.00	0.00	0.00	1.00	0.00	0.00
chr15	100300103	404	G	0	0.99	0.00	0.00	1.00	0.00	0.00	1.00	0.00	0.00	1.00	0.00	0.00	1.00	0.00	0.00	1.00	0.00	0.00
chr4	136547429	1063	G	0	1.00	0.00	0.00	1.00	0.00	0.00	1.00	0.00	0.00	1.00	0.00	0.00	1.00	0.00	0.00	1.00	0.00	0.00
chr8	89377069	1688	G	0	1.00	0.00	0.00	1.00	0.00	0.00	1.00	0.00	0.00	1.00	0.00	0.00	0.99	0.01	0.00	1.00	0.00	0.00
chr8	120115594	1711	G	0	1.00	0.00	0.00	1.00	0.00	0.00	1.00	0.00	0.00	1.00	0.00	0.00	1.00	0.00	0.00	1.00	0.00	0.00
chr8	120115623	1725	C	0	1.00	0.00	0.00	1.00	0.00	0.00	1.00	0.00	0.00	1.00	0.00	0.00	1.00	0.00	0.00	1.00	0.00	0.00
chr9	100498437	1886	G	0	1.00	0.00	0.00	1.00	0.00	0.00	1.00	0.00	0.00	1.00	0.00	0.00	0.99	0.01	0.00	1.00	0.00	0.00
chrX	7476417	2032	G	0	1.00	0.00	0.00	1.00	0.00	0.00	1.00	0.00	0.00	1.00	0.00	0.00	1.00	0.00	0.00	1.00	0.00	0.00
chrX	7476606	2148	G	0	1.00	0.00	0.00	1.00	0.00	0.00	1.00	0.00	0.00	1.00	0.00	0.00	1.00	0.00	0.00	1.00	0.00	0.00
chrX	7476609	2151	C	0	0.99	0.00	0.00	1.00	0.00	0.00	1.00	0.00	0.00	1.00	0.00	0.00	0.99	0.00	0.01	1.00	0.00	0.00
chrX	7476642	2174	G	0	1.00	0.00	0.00	1.00	0.00	0.00	1.00	0.00	0.00	1.00	0.00	0.00	1.00	0.00	0.00	1.00	0.00	0.00
chrX	7539383	2261	G	0	0.99	0.00	0.01	1.00	0.00	0.00	1.00	0.00	0.00	1.00	0.00	0.00	1.00	0.00	0.00	1.00	0.00	0.00
chrX	7539559	2369	G	0	1.00	0.00	0.00	1.00	0.00	0.00	1.00	0.00	0.00	1.00	0.00	0.00	1.00	0.00	0.00	1.00	0.00	0.00
chr15	100300073	390	G	0	1.00	0.00	0.00	1.00	0.00	0.00	1.00	0.00	0.00	1.00	0.00	0.00	1.00	0.00	0.00	1.00	0.00	0.00
chr4	139783713	1294	G	0	1.00	0.00	0.00	1.00	0.00	0.00	0.99	0.00	0.00	1.00	0.00	0.00	1.00	0.00	0.00	1.00	0.00	0.00
chr8	89376877	1612	g	0	0.99	0.00	0.00	1.00	0.00	0.00	0.99	0.00	0.00	0.99	0.00	0.00	1.00	0.00	0.00	1.00	0.00	0.00
chr8	120115568	1702	G	0	1.00	0.00	0.00	1.00	0.00	0.00	1.00	0.00	0.00	1.00	0.00	0.00	1.00	0.00	0.00	1.00	0.00	0.00
chr8	120115621	1724	C	0	1.00	0.00	0.00	1.00	0.00	0.00	1.00	0.00	0.00	1.00	0.00	0.00	1.00	0.00	0.00	1.00	0.00	0.00
chr8	120115801	1809	G	0	1.00	0.00	0.00	1.00	0.00	0.00	1.00	0.00	0.00	1.00	0.00	0.00	1.00	0.00	0.00	1.00	0.00	0.00

0.99	0.01	0.00	0.99	0.01	0.00	0,00014
0.99	0.00	0.00	0.99	0.00	0.00	0,00014
0.99	0.01	0.00	0.99	0.00	0.00	0,00014
0.99	0.00	0.00	0.99	0.01	0.00	0,00014
0.99	0.01	0.00	0.99	0.00	0.01	0,00014
0.99	0.01	0.00	0.99	0.01	0.00	0,00014
0.99	0.01	0.00	0.99	0.00	0.00	0,00014
0.99	0.00	0.00	0.99	0.00	0.01	0,00014
0.99	0.00	0.00	0.99	0.01	0.00	0,00014
0.99	0.01	0.00	0.99	0.01	0.00	0,00014
0.99	0.00	0.00	0.99	0.00	0.01	0,00014
0.99	0.01	0.00	0.99	0.01	0.00	0,00014
0.99	0.01	0.00	0.99	0.01	0.00	0,00014
0.99	0.00	0.00	0.99	0.00	0.01	0,00014
0.99	0.01	0.00	0.99	0.00	0.01	0,00014
0.99	0.00	0.00	0.99	0.00	0.01	0,00014
0.99	0.01	0.00	0.99	0.00	0.00	0,00014
0.99	0.01	0.00	0.99	0.00	0.01	0,00014
0.99	0.01	0.01	0.99	0.01	0.00	0,00014
0.99	0.00	0.00	0.99	0.00	0.01	0,00014
0.99	0.00	0.00	0.99	0.01	0.00	0,00014
0.99	0.00	0.00	0.99	0.00	0.01	0,00014
0.99	0.00	0.00	0.99	0.00	0.01	0,00014
0.99	0.00	0.00	0.99	0.00	0.00	0,00014
0.99	0.00	0.01	0.99	0.00	0.00	0,00014
0.99	0.01	0.00	0.99	0.00	0.00	0,00014
0.99	0.00	0.00	0.99	0.01	0.00	0,00014
0.99	0.00	0.00	0.99	0.00	0.01	0,00014
0.99	0.01	0.00	0.99	0.00	0.01	0,00014
0.99	0.00	0.01	0.99	0.00	0.00	0,00014
0.99	0.00	0.00	0.99	0.01	0.00	0,00014
0.99	0.00	0.00	0.99	0.00	0.00	0,00014
0.99	0.00	0.00	0.99	0.00	0.00	0,00014
0.99	0.00	0.00	0.99	0.01	0.00	0,00014
0.99	0.00	0.00	0.99	0.00	0.00	0,00014
0.99	0.00	0.00	0.99	0.00	0.00	0,00014
0.99	0.00	0.00	0.99	0.01	0.00	0,00014
0.99	0.00	0.00	0.99	0.00	0.00	0,00014
0.99	0.00	0.01	0.99	0.00	0.00	0,00014
0.99	0.00	0.01	0.99	0.00	0.01	0,00014
0.99	0.00	0.00	0.99	0.00	0.00	0,00014
0.99	0.00	0.00	0.99	0.00	0.01	0,00014
0.99	0.00	0.00	0.99	0.01	0.00	0,00014
0.99	0.00	0.00	0.99	0.01	0.00	0,00014
0.99	0.00	0.00	0.99	0.00	0.00	0,00014
0.99	0.01	0.00	0.99	0.00	0.00	0,00014

chr9	100498473	1907	G	0	1.00	0.00	0.00	1.00	0.00	0.00	1.00	0.00	0.00	1.00	0.00	0.00	1.00	0.00	0.00	1.00	0.00	0.00
chr9	100498479	1911	C	0	1.00	0.00	0.00	1.00	0.00	0.00	1.00	0.00	0.00	1.00	0.00	0.00	1.00	0.00	0.00	1.00	0.00	0.00
chrX	7476558	2118	G	0	1.00	0.00	0.00	1.00	0.00	0.00	1.00	0.00	0.00	1.00	0.00	0.00	1.00	0.00	0.00	1.00	0.00	0.00
chrX	7539276	2219	G	0	1.00	0.00	0.00	1.00	0.00	0.00	1.00	0.00	0.00	1.00	0.00	0.00	1.00	0.00	0.00	1.00	0.00	0.00
chrX	7539607	2399	C	0	1.00	0.00	0.00	1.00	0.00	0.00	1.00	0.00	0.00	1.00	0.00	0.00	1.00	0.00	0.00	1.00	0.00	0.00
chr11	3525755	83	C	0	1.00	0.00	0.00	1.00	0.00	0.00	1.00	0.00	0.00	1.00	0.00	0.00	1.00	0.00	0.00	1.00	0.00	0.00
chr15	61868655	203	C	0	1.00	0.00	0.00	1.00	0.00	0.00	1.00	0.00	0.00	0.99	0.01	0.00	1.00	0.00	0.00	1.00	0.00	0.00
chr15	61868771	260	G	0	1.00	0.00	0.00	1.00	0.00	0.00		1.00	0.00	1.00	0.00	0.00	1.00	0.00	0.00	1.00	0.00	0.00
chr16	92644291	510	G	0	0.99	0.00	0.00	0.99	0.01	0.01	1.00	0.00	0.00	0.99	0.00	0.00	1.00	0.00	0.00	1.00	0.00	0.00
chr4	136547711	1195	C	0	1.00	0.00	0.00	1.00	0.00	0.00	1.00	0.00	0.00	1.00	0.00	0.00	1.00	0.00	0.00	1.00	0.00	0.00
chr4	139783621	1241	G	0	1.00	0.00	0.00	1.00	0.00	0.00	1.00	0.00	0.00	1.00	0.00	0.00	1.00	0.00	0.00	1.00	0.00	0.00
chr9	100498441	1888	C	0	1.00	0.00	0.00	1.00	0.00	0.00	1.00	0.00	0.00	1.00	0.00	0.00	1.00	0.00	0.00	1.00	0.00	0.00
chrX	7476632	2167	C	1	1.00	0.00	0.00	1.00	0.00	0.00	1.00	0.00	0.00	1.00	0.00	0.00	0.99	0.00	0.01	0.99	0.01	0.00
chr11	3525629	20	C	0	1.00	0.00	0.00	1.00	0.00	0.00	1.00	0.00	0.00	1.00	0.00	0.00	1.00	0.00	0.00	1.00	0.00	0.00
chr15	100300112	408	G	0	1.00	0.00	0.00	1.00	0.00	0.00	1.00	0.00	0.00	1.00	0.00	0.00	1.00	0.00	0.00	1.00	0.00	0.00
chr16	92644309	512	G	0	1.00	0.00	0.00	0.99	0.00	0.00	1.00	0.00	0.00	0.99	0.00	0.00	1.00	0.00	0.00	1.00	0.00	0.00
chr19	24277709	1001	G	0	1.00	0.00	0.00	1.00	0.00	0.00	1.00	0.00	0.00	1.00	0.00	0.00	1.00	0.00	0.00	1.00	0.00	0.00
chr4	139783661	1265	C	0	0.99	0.00	0.01	1.00	0.00	0.00	1.00	0.00	0.00	1.00	0.00	0.00	1.00	0.00	0.00	1.00	0.00	0.00
chr5	64987346	1421	G	0	1.00	0.00	0.00	1.00	0.00	0.00	1.00	0.00	0.00	1.00	0.00	0.00	0.99	0.00	0.00	1.00	0.00	0.00
chrX	7476539	2103	C	0	1.00	0.00	0.00	1.00	0.00	0.00	1.00	0.00	0.00	1.00	0.00	0.00	0.99	0.00	0.00	1.00	0.00	0.00
chrX	7476627	2162	C	0	1.00	0.00	0.00	1.00	0.00	0.00	1.00	0.00	0.00	1.00	0.00	0.00	0.99	0.00	0.01	1.00	0.00	0.00
chr11	3525734	75	C	0	1.00	0.00	0.00	1.00	0.00	0.00	1.00	0.00	0.00	1.00	0.00	0.00	1.00	0.00	0.00	1.00	0.00	0.00
chr11	3525879	162	C	0	1.00	0.00	0.00	1.00	0.00	0.00	1.00	0.00	0.00	1.00	0.00	0.00	1.00	0.00	0.00	1.00	0.00	0.00
chr15	61868665	207	C	0	1.00	0.00	0.00	1.00	0.00	0.00	1.00	0.00	0.00	1.00	0.00	0.00	1.00	0.00	0.00	1.00	0.00	0.00
chr15	61868698	223	C	0	1.00	0.00	0.00	1.00	0.00	0.00	1.00	0.00	0.00	1.00	0.00	0.00	0.99	0.00	0.01	1.00	0.00	0.00
chr17	45706527	744	C	0	1.00	0.00	0.00	1.00	0.00	0.00	1.00	0.00	0.00	1.00	0.00	0.00	1.00	0.00	0.00	1.00	0.00	0.00
chr17	45706619	796	C	0	1.00	0.00	0.00	1.00	0.00	0.00	1.00	0.00	0.00	1.00	0.00	0.00	0.99	0.00	0.01	1.00	0.00	0.00
chr4	136547487	1096	G	0	1.00	0.00	0.00	1.00	0.00	0.00	1.00	0.00	0.00	1.00	0.00	0.00	1.00	0.00	0.00	1.00	0.00	0.00
chr4	136547547	1135	G	0	1.00	0.00	0.00	1.00	0.00	0.00	1.00	0.00	0.00	1.00	0.00	0.00	1.00	0.00	0.00	1.00	0.00	0.00
chr4	139783756	1316	G	0	1.00	0.00	0.00	1.00	0.00	0.00	1.00	0.00	0.00	1.00	0.00	0.00	1.00	0.00	0.00	1.00	0.00	0.00
chr4	139783818	1349	C	0	1.00	0.00	0.00	0.99	0.00	0.00	1.00	0.00	0.00	1.00	0.00	0.00	1.00	0.00	0.00	1.00	0.00	0.00
chr4	139783854	1370	G	0	1.00	0.00	0.00	1.00	0.00	0.00	1.00	0.00	0.00	1.00	0.00	0.00	1.00	0.00	0.00	1.00	0.00	0.00
chr8	89376959	1645	G	0	1.00	0.00	0.00	1.00	0.00	0.00	1.00	0.00	0.00	1.00	0.00	0.00	1.00	0.00	0.00	1.00	0.00	0.00
chr8	120115570	1704	G	0	1.00	0.00	0.00	1.00	0.00	0.00	1.00	0.00	0.00	1.00	0.00	0.00	1.00	0.00	0.00	1.00	0.00	0.00
chr9	100498588	1967	C	0	1.00	0.00	0.00	1.00	0.00	0.00	1.00	0.00	0.00	1.00	0.00	0.00	1.00	0.00	0.00	1.00	0.00	0.00
chrX	7539495	2332	C	0	1.00	0.00	0.00	0.99	0.00	0.00	1.00	0.00	0.00	1.00	0.00	0.00	1.00	0.00	0.00	1.00	0.00	0.00
chrX	7539545	2360	G	0	1.00	0.00	0.00	1.00	0.00	0.00	1.00	0.00	0.00	1.00	0.00	0.00	1.00	0.00	0.00	1.00	0.00	0.00
chr17	45706578	774	G	0	1.00	0.00	0.00	1.00	0.00	0.00	0.99	0.00	0.00	1.00	0.00	0.00	1.00	0.00	0.00	1.00	0.00	0.00
chr4	136547432	1065	G	0	1.00	0.00	0.00	1.00	0.00	0.00	1.00	0.00	0.00	1.00	0.00	0.00	1.00	0.00	0.00	1.00	0.00	0.00
chr4	136547752	1209	C	0	1.00	0.00	0.00	1.00	0.00	0.00	0.99	0.00	0.01	1.00	0.00	0.00	1.00	0.00	0.00	1.00	0.00	0.00
chr4	139783710	1292	C	0	1.00	0.00	0.00	1.00	0.00	0.00	1.00	0.00	0.00	1.00	0.00	0.00	1.00	0.00	0.00	1.00	0.00	0.00
chr4	139783872	1381	G	0	1.00	0.00	0.00	1.00	0.00	0.00	1.00	0.00	0.00	1.00	0.00	0.00	1.00	0.00	0.00	1.00	0.00	0.00
chr8	89376897	1624	c	0	1.00	0.00	0.00	1.00	0.00	0.00	0.99	0.00	0.00	1.00	0.00	0.00	1.00	0.00	0.00	1.00	0.00	0.00
chr8	89377038	1674	G	0	1.00	0.00	0.00	1.00	0.00	0.00	1.00	0.00	0.00	1.00	0.00	0.00	1.00	0.00	0.00	1.00	0.00	0.00
chr8	120115575	1705	G	0	1.00	0.00	0.00	1.00	0.00	0.00	1.00	0.00	0.00	1.00	0.00	0.00	1.00	0.00	0.00	1.00	0.00	0.00
chr8	120115611	1719	C	0	1.00	0.00	0.00	1.00	0.00	0.00	1.00	0.00	0.00	1.00	0.00	0.00	1.00	0.00	0.00	1.00	0.00	0.00

0.99	0.00	0.00	0.99	0.00	0.00	0,00014
0.99	0.00	0.00	0.99	0.01	0.00	0,00014
0.99	0.00	0.01	0.99	0.00	0.00	0,00014
0.99	0.00	0.00	0.99	0.00	0.00	0,00014
0.99	0.00	0.00	0.99	0.01	0.00	0,00014
0.99	0.00	0.00	0.99	0.00	0.00	0,00014
0.99	0.00	0.00	0.99	0.00	0.00	0,00014
0.99	0.00	0.00	0.99	0.01	0.00	0,00014
0.99	0.01	0.00	0.99	0.00	0.00	0,00014
0.99	0.00	0.00	0.99	0.00	0.01	0,00014
0.99	0.00	0.00	0.99	0.00	0.00	0,00014
0.99	0.00	0.00	0.99	0.01	0.00	0,00014
0.99	0.00	0.00	0.99	0.01	0.00	0,00014
0.99	0.00	0.00	0.99	0.00	0.01	0,00014
0.99	0.00	0.00	0.99	0.00	0.01	0,00014
0.99	0.00	0.01	0.99	0.00	0.00	0,00014
0.99	0.00	0.00	0.99	0.00	0.00	0,00014
0.99	0.00	0.00	0.99	0.00	0.01	0,00014
0.99	0.00	0.00	0.99	0.00	0.01	0,00014
0.99	0.00	0.00	0.99	0.00	0.00	0,00014
0.99	0.01	0.00	0.99	0.01	0.00	0,00014
0.99	0.01	0.00	0.99	0.01	0.00	0,00014
0.99	0.00	0.00	0.99	0.01	0.00	0,00014
0.99	0.01	0.00	0.99	0.00	0.00	0,00014
0.99	0.00	0.00	0.99	0.00	0.01	0,00014
0.99	0.00	0.00	0.99	0.01	0.01	0,00014
0.99	0.00	0.01	0.99	0.00	0.01	0,00014
0.99	0.00	0.01	0.99	0.00	0.00	0,00014
0.99	0.00	0.00	0.99	0.01	0.00	0,00014
0.99	0.00	0.00	0.99	0.00	0.01	0,00014
0.99	0.00	0.00	0.99	0.00	0.01	0,00014
0.99	0.01	0.00	0.99	0.00	0.00	0,00014
0.99	0.01	0.01	0.99	0.00	0.00	0,00014
0.99	0.00	0.00	0.99	0.01	0.00	0,00014
0.99	0.00	0.00	0.99	0.00	0.00	0,00014
0.99	0.00	0.01	0.99	0.01	0.00	0,00014
0.99	0.00	0.00	0.99	0.00	0.00	0,00014
0.99	0.00	0.01	0.99	0.00	0.00	0,00013
0.99	0.00	0.00	0.99	0.01	0.00	0,00013
0.99	0.00	0.01	0.99	0.00	0.01	0,00013
0.99	0.00	0.00	0.99	0.00	0.00	0,00013
0.99	0.00	0.01	0.99	0.00	0.00	0,00013
0.99	0.00	0.00	0.99	0.00	0.01	0,00013
0.99	0.00	0.00	0.99	0.01	0.00	0,00013
0.99	0.00	0.00	0.99	0.00	0.01	0,00013
0.99	0.00	0.00	0.99	0.00	0.00	0,00013

chr8	120115834	1828	C		0	1.00	0.00	0.00	1.00	0.00	0.00	1.00	0.00	0.00	1.00	0.00	0.00	1.00	0.00	0.00	1.00	0.00	0.00
chrX	7476528	2093	C		0	1.00	0.00	0.00	1.00	0.00	0.00	1.00	0.00	0.00	1.00	0.00	0.00	0.99	0.00	0.01	1.00	0.00	0.00
chrX	7539360	2248	G		0	1.00	0.00	0.00	1.00	0.00	0.00	1.00	0.00	0.00	1.00	0.00	0.00	1.00	0.00	0.00	1.00	0.00	0.00
chr11	3525792	108	G		0	1.00	0.00	0.00	1.00	0.00	0.00	1.00	0.00	0.00	1.00	0.00	0.00	1.00	0.00	0.00	1.00	0.00	0.00
chr11	3525907	177	G		0	1.00	0.00	0.00	1.00	0.00	0.00	1.00	0.00	0.00	1.00	0.00	0.00	1.00	0.00	0.00	1.00	0.00	0.00
chr19	24277488	905	c		0	1.00	0.00	0.00	1.00	0.00	0.00	1.00	0.00	0.00	1.00	0.00	0.00	1.00	0.00	0.00	1.00	0.00	0.00
chr4	136547519	1118	G		0	1.00	0.00	0.00	1.00	0.00	0.00	1.00	0.00	0.00	1.00	0.00	0.00	1.00	0.00	0.00	1.00	0.00	0.00
chr4	139783590	1226	c		0	0.99	0.01	0.00	1.00	0.00	0.00	1.00	0.00	0.00	1.00	0.00	0.00	1.00	0.00	0.00	1.00	0.00	0.00
chr4	139783684	1278	C		0	0.99	0.01	0.00	1.00	0.00	0.00	1.00	0.00	0.00	1.00	0.00	0.00	1.00	0.00	0.00	1.00	0.00	0.00
chr4	139783855	1371	C		0	1.00	0.00	0.00	1.00	0.00	0.00	1.00	0.00	0.00	1.00	0.00	0.00	1.00	0.00	0.00	1.00	0.00	0.00
chr8	89376952	1643	C		0	1.00	0.00	0.00	1.00	0.00	0.00	1.00	0.00	0.00	1.00	0.00	0.00	1.00	0.00	0.00	1.00	0.00	0.00
chrX	7476569	2126	C		0	1.00	0.00	0.00	1.00	0.00	0.00	1.00	0.00	0.00	1.00	0.00	0.00	1.00	0.00	0.00	1.00	0.00	0.00
chr11	3525678	43	C		0	1.00	0.00	0.00	1.00	0.00	0.00	0.99	0.01	0.00	1.00	0.00	0.00	1.00	0.00	0.00	1.00	0.00	0.00
chr4	136547499	1105	G		0	1.00	0.00	0.00	0.99	0.00	0.00	1.00	0.00	0.00	1.00	0.00	0.00	1.00	0.00	0.00	1.00	0.00	0.00
chr4	139783589	1225	c		0	1.00	0.00	0.00	1.00	0.00	0.00	1.00	0.00	0.00	1.00	0.00	0.00	1.00	0.00	0.00	1.00	0.00	0.00
chr4	139783760	1318	G		0	1.00	0.00	0.00	1.00	0.00	0.00	1.00	0.00	0.00	1.00	0.00	0.00	1.00	0.00	0.00	1.00	0.00	0.00
chr5	64987540	1508	C		0	1.00	0.00	0.00	0.99	0.00	0.00	1.00	0.00	0.00	1.00	0.00	0.00	1.00	0.00	0.00	1.00	0.00	0.00
chr9	100498486	1915	G		0	1.00	0.00	0.00	1.00	0.00	0.00	1.00	0.00	0.00	1.00	0.00	0.00	1.00	0.00	0.00	1.00	0.00	0.00
chr9	100498528	1938	G		0	1.00	0.00	0.00	1.00	0.00	0.00	1.00	0.00	0.00	1.00	0.00	0.00	1.00	0.00	0.00	1.00	0.00	0.00
chr9	100498581	1965	C		0	1.00	0.00	0.00	1.00	0.00	0.00	1.00	0.00	0.00	1.00	0.00	0.00	1.00	0.00	0.00	1.00	0.00	0.00
chrX	7476351	2015	G		0	1.00	0.00	0.00	1.00	0.00	0.00	1.00	0.00	0.00	1.00	0.00	0.00	1.00	0.00	0.00	1.00	0.00	0.00
chrX	7476382	2021	G		0	1.00	0.00	0.00	1.00	0.00	0.00	1.00	0.00	0.00	1.00	0.00	0.00	1.00	0.00	0.00	1.00	0.00	0.00
chrX	7476474	2055	C		0	1.00	0.00	0.00	1.00	0.00	0.00	1.00	0.00	0.00	1.00	0.00	0.00	0.99	0.00	0.01	1.00	0.00	0.00
chrX	7476573	2128	G		0	1.00	0.00	0.00	1.00	0.00	0.00	1.00	0.00	0.00	1.00	0.00	0.00	1.00	0.00	0.00	1.00	0.00	0.00
chrX	7476576	2130	G		0	1.00	0.00	0.00	1.00	0.00	0.00	1.00	0.00	0.00	1.00	0.00	0.00	1.00	0.00	0.00	1.00	0.00	0.00
chrX	7539555	2367	C		0	1.00	0.00	0.00	1.00	0.00	0.00	1.00	0.00	0.00	1.00	0.00	0.00	0.99	0.00	0.00	1.00	0.00	0.00
chrX	7539610	2401	G		0	1.00	0.00	0.00	1.00	0.00	0.00	1.00	0.00	0.00	1.00	0.00	0.00	1.00	0.00	0.00	1.00	0.00	0.00
chr17	45706452	705	C		0	1.00	0.00	0.00	1.00	0.00	0.00	1.00	0.00	0.00	1.00	0.00	0.00	1.00	0.00	0.00	1.00	0.00	0.00
chr4	136547515	1114	G		0	1.00	0.00	0.00	1.00	0.00	0.00	1.00	0.00	0.00	1.00	0.00	0.00	1.00	0.00	0.00	1.00	0.00	0.00
chr4	136547537	1128	G		0	1.00	0.00	0.00	1.00	0.00	0.00	1.00	0.00	0.00	1.00	0.00	0.00	1.00	0.00	0.00	1.00	0.00	0.00
chr4	139783805	1343	C		0	1.00	0.00	0.00	1.00	0.00	0.00	1.00	0.00	0.00	1.00	0.00	0.00	1.00	0.00	0.00	1.00	0.00	0.00
chrX	7476596	2142	G		0	1.00	0.00	0.00	1.00	0.00	0.00	1.00	0.00	0.00	1.00	0.00	0.00	1.00	0.00	0.00	1.00	0.00	0.00
chrX	7539558	2368	G		0	1.00	0.00	0.00	1.00	0.00	0.00	1.00	0.00	0.00	1.00	0.00	0.00	1.00	0.00	0.00	1.00	0.00	0.00
chr11	3525598	4	C		0	1.00	0.00	0.00	1.00	0.00	0.00	1.00	0.00	0.00	1.00	0.00	0.00	1.00	0.00	0.00	1.00	0.00	0.00
chr16	92644311	513	G		0	1.00	0.00	0.00	0.99	0.00	0.00	1.00	0.00	0.00	0.99	0.00	0.00	1.00	0.00	0.00	1.00	0.00	0.00
chr4	136547461	1084	C		0	1.00	0.00	0.00	1.00	0.00	0.00	1.00	0.00	0.00	1.00	0.00	0.00	0.99	0.00	0.00	1.00	0.00	0.00
chr5	64987644	1550	G		0	1.00	0.00	0.00	1.00	0.00	0.00	1.00	0.00	0.00	1.00	0.00	0.00	1.00	0.00	0.00	1.00	0.00	0.00
chr8	89376805	1571	g		0	1.00	0.00	0.00	1.00	0.00	0.00	1.00	0.00	0.00	1.00	0.00	0.00	1.00	0.00	0.00	1.00	0.00	0.00
chr9	100498355	1848	G		0	1.00	0.00	0.00	1.00	0.00	0.00	1.00	0.00	0.00	1.00	0.00	0.00	1.00	0.00	0.00	1.00	0.00	0.00
chr9	100498407	1871	C		0	1.00	0.00	0.00	1.00	0.00	0.00	1.00	0.00	0.00	1.00	0.00	0.00	1.00	0.00	0.00	1.00	0.00	0.00
chrX	7476313	2007	G		0	1.00	0.00	0.00	1.00	0.00	0.00	1.00	0.00	0.00	1.00	0.00	0.00	1.00	0.00	0.00	1.00	0.00	0.00
chrX	7476672	2197	G		0	1.00	0.00	0.00	1.00	0.00	0.00	1.00	0.00	0.00	1.00	0.00	0.00	1.00	0.00	0.00	1.00	0.00	0.00
chrX	7539386	2263	C		0	1.00	0.00	0.00	1.00	0.00	0.00	1.00	0.00	0.00	1.00	0.00	0.00	1.00	0.00	0.00	1.00	0.00	0.00
chr11	3525899	173	C		0	1.00	0.00	0.00	1.00	0.00	0.00	1.00	0.00	0.00	1.00	0.00	0.00	1.00	0.00	0.00	1.00	0.00	0.00
chr17	45706449	704	C		0	1.00	0.00	0.00	1.00	0.00	0.00	1.00	0.00	0.00	0.99	0.00	0.00	1.00	0.00	0.00	1.00	0.00	0.00
chr8	89376793	1565	g		0	1.00	0.00	0.00	1.00	0.00	0.00	1.00	0.00	0.00	1.00	0.00	0.00	1.00	0.00	0.00	1.00	0.00	0.00

0.99	0.00	0.00	0.99	0.00	0.00	0,00013
0.99	0.00	0.00	0.99	0.01	0.00	0,00013
0.99	0.00	0.00	0.99	0.00	0.00	0,00013
0.99	0.00	0.01	0.99	0.00	0.00	0,00013
0.99	0.00	0.01	0.99	0.01	0.00	0,00013
0.99	0.00	0.00	0.99	0.00	0.00	0,00013
0.99	0.00	0.00	0.99	0.00	0.00	0,00013
0.99	0.00	0.00	0.99	0.00	0.01	0,00013
0.99	0.00	0.00	0.99	0.00	0.00	0,00013
0.99	0.00	0.00	0.99	0.01	0.00	0,00013
0.99	0.00	0.00	0.99	0.00	0.00	0,00013
0.99	0.01	0.00	0.99	0.00	0.00	0,00013
0.99	0.01	0.00	0.99	0.00	0.01	0,00013
0.99	0.00	0.00	0.99	0.01	0.00	0,00013
0.99	0.01	0.00	0.99	0.00	0.00	0,00013
0.99	0.00	0.00	0.99	0.01	0.00	0,00013
0.99	0.00	0.00	0.99	0.00	0.00	0,00013
0.99	0.00	0.00	0.99	0.01	0.00	0,00013
0.99	0.01	0.00	0.99	0.01	0.00	0,00013
0.99	0.00	0.01	0.99	0.00	0.00	0,00013
0.99	0.00	0.00	0.99	0.01	0.00	0,00013
0.99	0.00	0.00	0.99	0.01	0.00	0,00013
0.99	0.01	0.00	0.99	0.01	0.00	0,00013
0.99	0.00	0.00	0.99	0.00	0.00	0,00013
0.99	0.00	0.00	0.99	0.01	0.00	0,00013
0.99	0.01	0.00	0.99	0.00	0.00	0,00013
0.99	0.01	0.00	0.99	0.00	0.00	0,00013
0.99	0.00	0.00	0.99	0.00	0.00	0,00013
0.99	0.00	0.00	0.99	0.00	0.00	0,00013
0.99	0.00	0.00	0.99	0.00	0.00	0,00013
0.99	0.01	0.00	0.99	0.01	0.00	0,00013
0.99	0.00	0.00	0.99	0.01	0.00	0,00013
0.99	0.01	0.00	0.99	0.01	0.00	0,00013
0.99	0.00	0.00	0.99	0.01	0.00	0,00013
0.99	0.00	0.01	0.99	0.00	0.00	0,00013
0.99	0.00	0.00	0.99	0.00	0.01	0,00013
0.99	0.00	0.00	0.99	0.00	0.00	0,00013
0.99	0.00	0.01	0.99	0.00	0.00	0,00013
0.99	0.00	0.01	0.99	0.01	0.00	0,00013
0.99	0.00	0.00	0.99	0.01	0.00	0,00013
0.99	0.00	0.00	0.99	0.00	0.00	0,00013
0.99	0.00	0.00	0.99	0.00	0.00	0,00013
0.99	0.00	0.00	0.99	0.00	0.00	0,00013
0.99	0.00	0.01	0.99	0.00	0.00	0,00013
0.99	0.00	0.00	0.99	0.00	0.00	0,00013
0.99	0.00	0.00	0.99	0.00	0.01	0,00013

chrX	7476388	2024	G	0	1.00	0.00	0.00	1.00	0.00	0.00	1.00	0.00	0.00	1.00	0.00	0.00	1.00	0.00	0.00	1.00	0.00	0.00
chrX	7539450	2304	C	0	1.00	0.00	0.00	1.00	0.00	0.00	1.00	0.00	0.00	1.00	0.00	0.00	1.00	0.00	0.00	1.00	0.00	0.00
chrX	7539526	2348	G	0	1.00	0.00	0.00	1.00	0.00	0.00	1.00	0.00	0.00	1.00	0.00	0.00	1.00	0.00	0.00	1.00	0.00	0.00
chrX	7539604	2396	G	0	1.00	0.00	0.00	1.00	0.00	0.00	1.00	0.00	0.00	1.00	0.00	0.00	1.00	0.00	0.00	1.00	0.00	0.00
chr1[illegible]	3525623	17	G	0	1.00	0.00	0.00	1.00	0.00	0.00	1.00	0.00	0.00	1.00	0.00	0.00	1.00	0.00	0.00	0.99	0.01	0.00
chr1[illegible]	61868901	320	G	0	1.00	0.00	0.00	1.00	0.00	0.00	1.00	0.00	0.00	1.00	0.00	0.00	1.00	0.00	0.00	1.00	0.00	0.00
chr1[illegible]	100300010	358	G	0	1.00	0.00	0.00	1.00	0.00	0.00	1.00	0.00	0.00	1.00	0.00	0.00	1.00	0.00	0.00	1.00	0.00	0.00
chr4	136547447	1074	G	0	1.00	0.00	0.00	1.00	0.00	0.00	1.00	0.00	0.00	1.00	0.00	0.00	1.00	0.00	0.00	1.00	0.00	0.00
chr8	89376836	1586	c	0	1.00	0.00	0.00	1.00	0.00	0.00	1.00	0.00	0.00	1.00	0.00	0.00	1.00	0.00	0.00	1.00	0.00	0.00
chr9	100498686	1988	c	0	1.00	0.00	0.00	1.00	0.00	0.00	1.00	0.00	0.00	1.00	0.00	0.00	1.00	0.00	0.00	1.00	0.00	0.00
chrX	7476550	2111	C	0	1.00	0.00	0.00	1.00	0.00	0.00	1.00	0.00	0.00	1.00	0.00	0.00	0.99	0.00	0.01	1.00	0.00	0.00
chrX	7476564	2121	G	0	1.00	0.00	0.00	1.00	0.00	0.00	1.00	0.00	0.00	1.00	0.00	0.00	1.00	0.00	0.00	1.00	0.00	0.00
chr4	139783596	1228	c	0	1.00	0.00	0.00	1.00	0.00	0.00	1.00	0.00	0.00	1.00	0.00	0.00	1.00	0.00	0.00	1.00	0.00	0.00
chr4	139783860	1374	C	0	1.00	0.00	0.00	1.00	0.00	0.00	1.00	0.00	0.00	0.99	0.01	0.00	1.00	0.00	0.00	1.00	0.00	0.00
chr8	89376881	1614	c	0	1.00	0.00	0.00	1.00	0.00	0.00	1.00	0.00	0.00	1.00	0.00	0.00	1.00	0.00	0.00	1.00	0.00	0.00
chr9	100498377	1855	G	0	1.00	0.00	0.00	1.00	0.00	0.00	1.00	0.00	0.00	1.00	0.00	0.00	1.00	0.00	0.00	1.00	0.00	0.00
chrX	7476361	2016	G	0	1.00	0.00	0.00	1.00	0.00	0.00	1.00	0.00	0.00	1.00	0.00	0.00	1.00	0.00	0.00	1.00	0.00	0.00
chr1[illegible]	3525820	122	C	0	1.00	0.00	0.00	1.00	0.00	0.00	1.00	0.00	0.00	1.00	0.00	0.00	0.99	0.01	0.00	1.00	0.00	0.00
chr1[illegible]	3525866	154	C	0	1.00	0.00	0.00	1.00	0.00	0.00	1.00	0.00	0.00	1.00	0.00	0.00	1.00	0.00	0.00	1.00	0.00	0.00
chr1[illegible]	100300013	359	G	0	1.00	0.00	0.00	1.00	0.00	0.00	1.00	0.00	0.00	1.00	0.00	0.00	1.00	0.00	0.00	1.00	0.00	0.00
chr1[illegible]	100300267	452	G	0	1.00	0.00	0.00	1.00	0.00	0.00	1.00	0.00	0.00	1.00	0.00	0.00	1.00	0.00	0.00	1.00	0.00	0.00
chr1[illegible]	92644567	643	G	0	1.00	0.00	0.00	0.99	0.00	0.00	1.00	0.00	0.00	1.00	0.00	0.00	1.00	0.00	0.00	1.00	0.00	0.00
chr1[illegible]	45706561	764	G	0	1.00	0.00	0.00	1.00	0.00	0.00	1.00	0.00	0.00	1.00	0.00	0.00	1.00	0.00	0.00	1.00	0.00	0.00
chr4	136547421	1058	G	0	1.00	0.00	0.00	1.00	0.00	0.00	1.00	0.00	0.00	1.00	0.00	0.00	1.00	0.00	0.00	1.00	0.00	0.00
chr9	100498324	1839	G	0	1.00	0.00	0.00	1.00	0.00	0.00	1.00	0.00	0.00	1.00	0.00	0.00	0.99	0.01	0.00	1.00	0.00	0.00
chrX	7476553	2113	G	0	1.00	0.00	0.00	1.00	0.00	0.00	1.00	0.00	0.00	1.00	0.00	0.00	1.00	0.00	0.00	1.00	0.00	0.00
chr1[illegible]	3525861	150	G	0	1.00	0.00	0.00	1.00	0.00	0.00	1.00	0.00	0.00	1.00	0.00	0.00	1.00	0.00	0.00	1.00	0.00	0.00
chr4	136547774	1216	C	0	1.00	0.00	0.00	1.00	0.00	0.00	1.00	0.00	0.00	1.00	0.00	0.00	1.00	0.00	0.00	1.00	0.00	0.00
chr8	120115616	1721	C	0	1.00	0.00	0.00	1.00	0.00	0.00	1.00	0.00	0.00	1.00	0.00	0.00	1.00	0.00	0.00	1.00	0.00	0.00
chr9	100498466	1902	C	0	1.00	0.00	0.00	1.00	0.00	0.00	1.00	0.00	0.00	1.00	0.00	0.00	0.99	0.00	0.00	1.00	0.00	0.00
chrX	7476329	2010	G	0	1.00	0.00	0.00	1.00	0.00	0.00	1.00	0.00	0.00	1.00	0.00	0.00	1.00	0.00	0.00	1.00	0.00	0.00
chr1[illegible]	3525819	121	C	0	1.00	0.00	0.00	1.00	0.00	0.00	1.00	0.00	0.00	1.00	0.00	0.00	1.00	0.00	0.00	1.00	0.00	0.00
chr1[illegible]	61868861	301	G	0	1.00	0.00	0.00	1.00	0.00	0.00	0.99	0.00	0.01	1.00	0.00	0.00	1.00	0.00	0.00	1.00	0.00	0.00
chr4	136547452	1077	G	0	1.00	0.00	0.00	1.00	0.00	0.00	1.00	0.00	0.00	1.00	0.00	0.00	1.00	0.00	0.00	1.00	0.00	0.00
chrX	7476362	2017	G	0	1.00	0.00	0.00	1.00	0.00	0.00	1.00	0.00	0.00	1.00	0.00	0.00	1.00	0.00	0.00	1.00	0.00	0.00
chr1[illegible]	61868781	265	G	0	0.99	0.00	0.01	1.00	0.00	0.00	1.00	0.00	0.00	0.99	0.00	0.01	1.00	0.00	0.00	0.99	0.00	0.00
chrX	7476467	2052	C	0	1.00	0.00	0.00	1.00	0.00	0.00	1.00	0.00	0.00	1.00	0.00	0.00	0.99	0.00	0.01	1.00	0.00	0.00
chr1[illegible]	3525608	9	G	0	1.00	0.00	0.00	1.00	0.00	0.00	1.00	0.00	0.00	1.00	0.00	0.00	1.00	0.00	0.00	1.00	0.00	0.00
chr1[illegible]	3525699	53	C	0	1.00	0.00	0.00	1.00	0.00	0.00	1.00	0.00	0.00	1.00	0.00	0.00	1.00	0.00	0.00	1.00	0.00	0.00
chr1[illegible]	3525725	69	G	0	1.00	0.00	0.00	1.00	0.00	0.00	1.00	0.00	0.00	0.99	0.00	0.00	1.00	0.00	0.00	1.00	0.00	0.00
chr1[illegible]	61868939	334	G	0	1.00	0.00	0.00	1.00	0.00	0.00	1.00	0.00	0.00	1.00	0.00	0.00	1.00	0.00	0.00	1.00	0.00	0.00
chr1[illegible]	100300263	451	G	0	1.00	0.00	0.00	1.00	0.00	0.00	1.00	0.00	0.00	1.00	0.00	0.00	1.00	0.00	0.00	1.00	0.00	0.00
chr4	139783687	1280	C	0	1.00	0.00	0.00	1.00	0.00	0.00	1.00	0.00	0.00	1.00	0.00	0.00	1.00	0.00	0.00	1.00	0.00	0.00
chr4	139783845	1363	G	0	1.00	0.00	0.00	1.00	0.00	0.00	1.00	0.00	0.00	0.99	0.01	0.00	1.00	0.00	0.00	1.00	0.00	0.00
chr5	64987320	1408	C	0	1.00	0.00	0.00	0.99	0.00	0.00	1.00	0.00	0.00	1.00	0.00	0.00	1.00	0.00	0.00	1.00	0.00	0.00
chr4	136547488	1097	G	0	1.00	0.00	0.00	1.00	0.00	0.00	1.00	0.00	0.00	1.00	0.00	0.00	1.00	0.00	0.00	1.00	0.00	0.00

0.99	0.00	0.00	0.99	0.00	0.00	0,00013
0.99	0.00	0.00	0.99	0.00	0.00	0,00013
0.99	0.00	0.00	0.99	0.00	0.00	0,00013
0.99	0.00	0.00	0.99	0.00	0.00	0,00013
0.99	0.00	0.00	0.99	0.00	0.01	0,00013
0.99	0.01	0.00	0.99	0.01	0.00	0,00013
0.99	0.00	0.00	0.99	0.00	0.00	0,00013
0.99	0.00	0.00	0.99	0.00	0.01	0,00013
0.99	0.01	0.00	0.99	0.01	0.00	0,00013
0.99	0.00	0.00	0.99	0.00	0.00	0,00013
0.99	0.00	0.00	0.99	0.00	0.00	0,00013
0.99	0.00	0.00	0.99	0.00	0.00	0,00013
0.99	0.00	0.00	0.99	0.00	0.00	0,00013
0.99	0.00	0.00	0.99	0.01	0.00	0,00013
0.99	0.00	0.00	0.99	0.00	0.00	0,00013
0.99	0.01	0.00	0.99	0.01	0.00	0,00013
0.99	0.00	0.00	0.99	0.00	0.00	0,00013
0.99	0.00	0.00	0.99	0.01	0.00	0,00013
0.99	0.01	0.00	0.99	0.01	0.00	0,00013
0.99	0.00	0.00	0.99	0.00	0.00	0,00013
0.99	0.00	0.00	0.99	0.01	0.00	0,00013
0.99	0.00	0.01	0.99	0.00	0.00	0,00013
0.99	0.00	0.00	0.99	0.00	0.00	0,00013
0.99	0.00	0.00	0.99	0.01	0.00	0,00013
0.99	0.00	0.00	0.99	0.00	0.00	0,00013
0.99	0.00	0.00	0.99	0.00	0.00	0,00013
0.99	0.00	0.00	0.99	0.01	0.00	0,00013
0.99	0.00	0.00	0.99	0.00	0.00	0,00013
0.99	0.00	0.00	0.99	0.00	0.01	0,00013
0.99	0.00	0.00	0.99	0.00	0.00	0,00013
0.99	0.00	0.00	0.99	0.00	0.00	0,00013
0.99	0.00	0.00	0.99	0.01	0.00	0,00012
0.99	0.00	0.00	0.99	0.00	0.00	0,00012
0.99	0.00	0.00	0.99	0.01	0.00	0,00012
0.99	0.00	0.00	0.99	0.00	0.00	0,00012
0.99	0.00	0.01	0.99	0.01	0.01	0,00012
0.99	0.01	0.00	0.99	0.00	0.00	0,00012
0.99	0.00	0.00	0.99	0.00	0.00	0,00012
0.99	0.01	0.00	0.99	0.00	0.00	0,00012
0.99	0.00	0.00	0.99	0.00	0.01	0,00012
0.99	0.00	0.00	0.99	0.00	0.00	0,00012
0.99	0.00	0.01	0.99	0.00	0.00	0,00012
0.99	0.00	0.00	0.99	0.00	0.00	0,00012
0.99	0.00	0.00	0.99	0.01	0.00	0,00012
0.99	0.00	0.00	0.99	0.00	0.00	0,00012
0.99	0.00	0.00	0.99	0.00	0.00	0,00012

chrX	7476393	2026	G	0	1.00	0.00	0.00	1.00	0.00	0.00	1.00	0.00	0.00	0.99	0.00	0.00	1.00	0.00	0.00	1.00	0.00	0.00
chrX	7476575	2129	G	0	1.00	0.00	0.00	1.00	0.00	0.00	1.00	0.00	0.00	1.00	0.00	0.00	1.00	0.00	0.00	1.00	0.00	0.00
chrX	7539586	2386	G	0	1.00	0.00	0.00	0.99	0.01	0.00	1.00	0.00	0.00	1.00	0.00	0.00	1.00	0.00	0.00	0.99	0.01	0.00
chr4	139783612	1237	c	0	1.00	0.00	0.00	1.00	0.00	0.00	1.00	0.00	0.00	1.00	0.00	0.00	1.00	0.00	0.00	1.00	0.00	0.00
chr4	139783702	1289	C	0	1.00	0.00	0.00	1.00	0.00	0.00	1.00	0.00	0.00	1.00	0.00	0.00	1.00	0.00	0.00	1.00	0.00	0.00
chr5	64987343	1419	C	0	1.00	0.00	0.00	1.00	0.00	0.00	1.00	0.00	0.00	1.00	0.00	0.00	1.00	0.00	0.00	1.00	0.00	0.00
chr5	64987656	1553	C	0	1.00	0.00	0.00	1.00	0.00	0.00	1.00	0.00	0.00	1.00	0.00	0.00	1.00	0.00	0.00	1.00	0.00	0.00
chrX	7476402	2028	G	0	1.00	0.00	0.00	1.00	0.00	0.00	1.00	0.00	0.00	1.00	0.00	0.00	1.00	0.00	0.00	1.00	0.00	0.00
chrX	7476407	2030	G	0	1.00	0.00	0.00	1.00	0.00	0.00	1.00	0.00	0.00	1.00	0.00	0.00	1.00	0.00	0.00	1.00	0.00	0.00
chrX	7476665	2193	G	0	1.00	0.00	0.00	1.00	0.00	0.00	1.00	0.00	0.00	1.00	0.00	0.00	1.00	0.00	0.00	1.00	0.00	0.00
chrX	7539384	2262	G	0	1.00	0.00	0.00	1.00	0.00	0.00	1.00	0.00	0.00	1.00	0.00	0.00	1.00	0.00	0.00	1.00	0.00	0.00
chrX	7539538	2356	C	0	1.00	0.00	0.00	1.00	0.00	0.00	1.00	0.00	0.00	1.00	0.00	0.00	1.00	0.00	0.00	1.00	0.00	0.00
chrX	7539542	2359	G	0	1.00	0.00	0.00	1.00	0.00	0.00	1.00	0.00	0.00	1.00	0.00	0.00	1.00	0.00	0.00	1.00	0.00	0.00
chr9	100498501	1923	C	0	1.00	0.00	0.00	1.00	0.00	0.00	1.00	0.00	0.00	1.00	0.00	0.00	1.00	0.00	0.00	1.00	0.00	0.00
chr11	3525840	134	C	0	1.00	0.00	0.00	1.00	0.00	0.00	1.00	0.00	0.00	1.00	0.00	0.00	1.00	0.00	0.00	1.00	0.00	0.00
chr8	120115825	1824	C	0	1.00	0.00	0.00	1.00	0.00	0.00	1.00	0.00	0.00	1.00	0.00	0.00	1.00	0.00	0.00	1.00	0.00	0.00
chr9	100498483	1913	G	0	1.00	0.00	0.00	1.00	0.00	0.00	1.00	0.00	0.00	1.00	0.00	0.00	1.00	0.00	0.00	1.00	0.00	0.00
chrX	7476391	2025	G	0	1.00	0.00	0.00	1.00	0.00	0.00	1.00	0.00	0.00	1.00	0.00	0.00	1.00	0.00	0.00	1.00	0.00	0.00
chrX	7476652	2181	G	0	1.00	0.00	0.00	1.00	0.00	0.00	1.00	0.00	0.00	1.00	0.00	0.00	1.00	0.00	0.00	1.00	0.00	0.00
chrX	7539581	2383	G	0	1.00	0.00	0.00	1.00	0.00	0.00	1.00	0.00	0.00	1.00	0.00	0.00	1.00	0.00	0.00	1.00	0.00	0.00
chr11	3525884	165	C	0	1.00	0.00	0.00	1.00	0.00	0.00	1.00	0.00	0.00	1.00	0.00	0.00	1.00	0.00	0.00	1.00	0.00	0.00
chr16	92644557	637	G	0	1.00	0.00	0.00	0.99	0.00	0.00	1.00	0.00	0.00	1.00	0.00	0.00	1.00	0.00	0.00	1.00	0.00	0.00
chr4	139783719	1295	G	0	1.00	0.00	0.00	0.99	0.00	0.00	1.00	0.00	0.00	1.00	0.00	0.00	1.00	0.00	0.00	1.00	0.00	0.00
chr9	100498624	1974	C	0	1.00	0.00	0.00	1.00	0.00	0.00	1.00	0.00	0.00	1.00	0.00	0.00	1.00	0.00	0.00	1.00	0.00	0.00
chr4	136547387	1041	C	0	1.00	0.00	0.00	1.00	0.00	0.00	1.00	0.00	0.00	1.00	0.00	0.00	1.00	0.00	0.00	1.00	0.00	0.00
chr4	139783840	1360	G	0	1.00	0.00	0.00	1.00	0.00	0.00	1.00	0.00	0.00	1.00	0.00	0.00	1.00	0.00	0.00	1.00	0.00	0.00
chr5	64987654	1552	C	0	1.00	0.00	0.00	1.00	0.00	0.00	1.00	0.00	0.00	1.00	0.00	0.00	1.00	0.00	0.00	1.00	0.00	0.00
chr8	89377042	1675	G	0	1.00	0.00	0.00	1.00	0.00	0.00	1.00	0.00	0.00	1.00	0.00	0.00	1.00	0.00	0.00	1.00	0.00	0.00
chrX	7476551	2112	C	0	1.00	0.00	0.00	1.00	0.00	0.00	1.00	0.00	0.00	1.00	0.00	0.00	0.99	0.00	0.01	1.00	0.00	0.00
chrX	7539564	2373	G	0	0.99	0.00	0.01	1.00	0.00	0.00	1.00	0.00	0.00	1.00	0.00	0.00	1.00	0.00	0.00	1.00	0.00	0.00
chr11	3525812	118	C	0	1.00	0.00	0.00	1.00	0.00	0.00	1.00	0.00	0.00	1.00	0.00	0.00	0.99	0.00	0.01	1.00	0.00	0.00
chr9	100498447	1892	G	0	1.00	0.00	0.00	1.00	0.00	0.00	1.00	0.00	0.00	1.00	0.00	0.00	1.00	0.00	0.00	1.00	0.00	0.00
chr9	100498709	1996	c	0	1.00	0.00	0.00	1.00	0.00	0.00	1.00	0.00	0.00	1.00	0.00	0.00	0.99	0.00	0.00	1.00	0.00	0.00
chrX	7476623	2160	C	0	1.00	0.00	0.00	1.00	0.00	0.00	1.00	0.00	0.00	1.00	0.00	0.00	0.99	0.00	0.01	1.00	0.00	0.00
chrX	7476647	2178	C	0	1.00	0.00	0.00	1.00	0.00	0.00	1.00	0.00	0.00	1.00	0.00	0.00	1.00	0.00	0.00	1.00	0.00	0.00
chrX	7476670	2196	C	0	1.00	0.00	0.00	1.00	0.00	0.00	1.00	0.00	0.00	1.00	0.00	0.00	1.00	0.00	0.00	1.00	0.00	0.00
chr11	3525735	76	C	0	1.00	0.00	0.00	1.00	0.00	0.00	1.00	0.00	0.00	1.00	0.00	0.00	1.00	0.00	0.00	1.00	0.00	0.00
chr16	92644582	648	G	0	1.00	0.00	0.00	1.00	0.00	0.00	1.00	0.00	0.00	1.00	0.00	0.00	1.00	0.00	0.00	1.00	0.00	0.00
chrX	7476302	2003	G	1	1.00	0.00	0.00	1.00	0.00	0.00	1.00	0.00	0.00	1.00	0.00	0.00	1.00	0.00	0.00	1.00	0.00	0.00
chrX	7476541	2104	C	0	1.00	0.00	0.00	1.00	0.00	0.00	1.00	0.00	0.00	1.00	0.00	0.00	0.99	0.00	0.01	1.00	0.00	0.00
chrX	7476591	2139	C	0	1.00	0.00	0.00	1.00	0.00	0.00	1.00	0.00	0.00	1.00	0.00	0.00	0.99	0.00	0.01	1.00	0.00	0.00
chr17	45706719	842	C	0	1.00	0.00	0.00	1.00	0.00	0.00	1.00	0.00	0.00	1.00	0.00	0.00	1.00	0.00	0.00	1.00	0.00	0.00
chr4	139783833	1358	C	0	1.00	0.00	0.00	1.00	0.00	0.00	1.00	0.00	0.00	1.00	0.00	0.00	1.00	0.00	0.00	1.00	0.00	0.00
chr9	100498626	1975	C	0	1.00	0.00	0.00	1.00	0.00	0.00	1.00	0.00	0.00	1.00	0.00	0.00	1.00	0.00	0.00	1.00	0.00	0.00
chr15	61868668	209	G	0	1.00	0.00	0.00	1.00	0.00	0.00	1.00	0.00	0.00	1.00	0.00	0.00	1.00	0.00	0.00	1.00	0.00	0.00
chr15	61868918	330	G	0	1.00	0.00	0.00	1.00	0.00	0.00	1.00	0.00	0.00	1.00	0.00	0.00	1.00	0.00	0.00	1.00	0.00	0.00

0.99	0.00	0.00	0.99	0.00	0.01	0,00012
0.99	0.00	0.00	0.99	0.00	0.00	0,00012
0.99	0.00	0.00	0.99	0.00	0.00	0,00012
0.99	0.01	0.00	0.99	0.00	0.00	0,00012
0.99	0.00	0.00	0.99	0.00	0.01	0,00012
0.99	0.00	0.01	0.99	0.00	0.00	0,00012
0.99	0.00	0.00	0.99	0.00	0.00	0,00012
0.99	0.00	0.00	0.99	0.00	0.00	0,00012
0.99	0.00	0.00	0.99	0.00	0.00	0,00012
0.99	0.00	0.00	0.99	0.01	0.00	0,00012
0.99	0.00	0.00	0.99	0.00	0.01	0,00012
0.99	0.00	0.00	0.99	0.00	0.00	0,00012
0.99	0.00	0.00	0.99	0.01	0.00	0,00012
0.99	0.00	0.01	0.99	0.00	0.01	0,00012
0.99	0.00	0.00	0.99	0.00	0.00	0,00012
0.99	0.00	0.00	0.99	0.00	0.00	0,00012
0.99	0.00	0.00	0.99	0.01	0.00	0,00012
0.99	0.00	0.00	0.99	0.00	0.00	0,00012
0.99	0.00	0.00	0.99	0.00	0.00	0,00012
0.99	0.00	0.00	0.99	0.00	0.01	0,00012
0.99	0.00	0.00	0.99	0.01	0.00	0,00012
0.99	0.00	0.00	0.99	0.00	0.00	0,00012
0.99	0.00	0.00	0.99	0.00	0.01	0,00012
0.99	0.00	0.00	0.99	0.00	0.00	0,00012
0.99	0.00	0.00	0.99	0.00	0.00	0,00012
0.99	0.00	0.00	0.99	0.01	0.00	0,00012
0.99	0.00	0.00	0.99	0.00	0.00	0,00012
0.99	0.00	0.00	0.99	0.01	0.00	0,00012
0.99	0.00	0.00	0.99	0.00	0.01	0,00012
0.99	0.00	0.00	0.99	0.00	0.00	0,00012
0.99	0.00	0.00	0.99	0.01	0.00	0,00012
0.99	0.00	0.00	0.99	0.00	0.00	0,00012
0.99	0.00	0.00	0.99	0.01	0.00	0,00012
0.99	0.00	0.00	0.99	0.00	0.00	0,00012
0.99	0.00	0.00	0.99	0.00	0.00	0,00012
0.99	0.00	0.00	0.99	0.00	0.00	0,00012
0.99	0.00	0.00	0.99	0.00	0.00	0,00011
0.99	0.00	0.00	0.99	0.00	0.00	0,00011
0.99	0.00	0.00	0.99	0.00	0.00	0,00011
0.99	0.01	0.00	0.99	0.01	0.00	0,00011
0.99	0.00	0.00	0.99	0.00	0.00	0,00011
0.99	0.00	0.00	0.99	0.01	0.00	0,00011
0.99	0.00	0.00	0.99	0.00	0.00	0,00011
0.99	0.00	0.00	0.99	0.01	0.00	0,00011
0.99	0.00	0.00	0.99	0.00	0.00	0,00011
0.99	0.00	0.00	0.99	0.00	0.00	0,00011

chr16	92644563	641	G	0	1.00	0.00	0.00	0.99	0.00	0.00	1.00	0.00	0.00	1.00	0.00	0.00	1.00	0.00	0.00	1.00	0.00	0.00
chr4	136547364	1031	G	0	1.00	0.00	0.00	1.00	0.00	0.00	1.00	0.00	0.00	1.00	0.00	0.00	1.00	0.00	0.00	1.00	0.00	0.00
chr5	64987649	1551	C	0	1.00	0.00	0.00	1.00	0.00	0.00	1.00	0.00	0.00	1.00	0.00	0.00	1.00	0.00	0.00	1.00	0.00	0.00
chrX	7476578	2131	G	0	1.00	0.00	0.00	1.00	0.00	0.00	1.00	0.00	0.00	1.00	0.00	0.00	1.00	0.00	0.00	1.00	0.00	0.00
chrX	7539582	2384	G	0	1.00	0.00	0.00	1.00	0.00	0.00	1.00	0.00	0.00	1.00	0.00	0.00	1.00	0.00	0.00	1.00	0.00	0.00
chr11	3525710	58	C	0	1.00	0.00	0.00	1.00	0.00	0.00	1.00	0.00	0.00	1.00	0.00	0.00	1.00	0.00	0.00	1.00	0.00	0.00
chrX	7476480	2060	C	0	1.00	0.00	0.00	1.00	0.00	0.00	1.00	0.00	0.00	1.00	0.00	0.00	0.99	0.00	0.01	1.00	0.00	0.00
chr15	100300072	389	G	0	1.00	0.00	0.00	1.00	0.00	0.00	1.00	0.00	0.00	1.00	0.00	0.00	1.00	0.00	0.00	1.00	0.00	0.00
chr16	92644558	638	G	0	1.00	0.00	0.00	0.99	0.00	0.00	1.00	0.00	0.00	1.00	0.00	0.00	1.00	0.00	0.00	1.00	0.00	0.00
chr4	139783616	1239	g	0	1.00	0.00	0.00	1.00	0.00	0.00	1.00	0.00	0.00	1.00	0.00	0.00	1.00	0.00	0.00	1.00	0.00	0.00
chr4	139783633	1247	G	0	1.00	0.00	0.00	1.00	0.00	0.00	1.00	0.00	0.00	1.00	0.00	0.00	1.00	0.00	0.00	1.00	0.00	0.00
chr9	100498516	1932	C	0	1.00	0.00	0.00	1.00	0.00	0.00	1.00	0.00	0.00	1.00	0.00	0.00	1.00	0.00	0.00	1.00	0.00	0.00
chr11	3525780	99	C	0	1.00	0.00	0.00	1.00	0.00	0.00	1.00	0.00	0.00	1.00	0.00	0.00	1.00	0.00	0.00	1.00	0.00	0.00
chr15	100300102	403	G	0	1.00	0.00	0.00	1.00	0.00	0.00	1.00	0.00	0.00	1.00	0.00	0.00	1.00	0.00	0.00	1.00	0.00	0.00
chr16	92644562	640	G	0	1.00	0.00	0.00	1.00	0.00	0.00	1.00	0.00	0.00	1.00	0.00	0.00	1.00	0.00	0.00	1.00	0.00	0.00
chr17	45706737	854	C	0	1.00	0.00	0.00	1.00	0.00	0.00	1.00	0.00	0.00	1.00	0.00	0.00	1.00	0.00	0.00	1.00	0.00	0.00
chr17	45706742	856	C	0	1.00	0.00	0.00	1.00	0.00	0.00	1.00	0.00	0.00	1.00	0.00	0.00	1.00	0.00	0.00	1.00	0.00	0.00
chr19	24277364	874	c	0	1.00	0.00	0.00	1.00	0.00	0.00	1.00	0.00	0.00	1.00	0.00	0.00	1.00	0.00	0.00	1.00	0.00	0.00
chr5	64987289	1398	G	0	1.00	0.00	0.00	1.00	0.00	0.00	1.00	0.00	0.00	1.00	0.00	0.00	1.00	0.00	0.00	1.00	0.00	0.00
chr5	64987294	1400	G	0	1.00	0.00	0.00	1.00	0.00	0.00	1.00	0.00	0.00	1.00	0.00	0.00	1.00	0.00	0.00	1.00	0.00	0.00
chr4	139783891	1389	C	0	1.00	0.00	0.00	1.00	0.00	0.00	1.00	0.00	0.00	1.00	0.00	0.00	1.00	0.00	0.00	1.00	0.00	0.00
chr5	64987330	1413	C	0	1.00	0.00	0.00	1.00	0.00	0.00	1.00	0.00	0.00	1.00	0.00	0.00	1.00	0.00	0.00	1.00	0.00	0.00
chr16	92644576	646	G	0	1.00	0.00	0.00	0.99	0.00	0.00	1.00	0.00	0.00	1.00	0.00	0.00	1.00	0.00	0.00	1.00	0.00	0.00
chr19	24277358	873	c	0	1.00	0.00	0.00	1.00	0.00	0.00	1.00	0.00	0.00	1.00	0.00	0.00	1.00	0.00	0.00	1.00	0.00	0.00
chr5	64987634	1544	G	0	1.00	0.00	0.00	1.00	0.00	0.00	1.00	0.00	0.00	1.00	0.00	0.00	1.00	0.00	0.00	1.00	0.00	0.00
chr8	89377045	1676	C	0	1.00	0.00	0.00	1.00	0.00	0.00	1.00	0.00	0.00	1.00	0.00	0.00	1.00	0.00	0.00	1.00	0.00	0.00
chr8	89377055	1683	C	0	1.00	0.00	0.00	1.00	0.00	0.00	1.00	0.00	0.00	1.00	0.00	0.00	1.00	0.00	0.00	1.00	0.00	0.00
chr19	24277367	876	c	0	1.00	0.00	0.00	1.00	0.00	0.00	1.00	0.00	0.00	1.00	0.00	0.00	1.00	0.00	0.00	1.00	0.00	0.00
chr4	139783598	1229	c	0	1.00	0.00	0.00	1.00	0.00	0.00	1.00	0.00	0.00	1.00	0.00	0.00	1.00	0.00	0.00	1.00	0.00	0.00
chrX	7539403	2273	C	0	1.00	0.00	0.00	1.00	0.00	0.00	1.00	0.00	0.00	1.00	0.00	0.00	1.00	0.00	0.00	1.00	0.00	0.00
chr19	24277699	996	C	0	1.00	0.00	0.00	1.00	0.00	0.00	1.00	0.00	0.00	1.00	0.00	0.00	1.00	0.00	0.00	1.00	0.00	0.00
chr5	64987329	1412	C	0	1.00	0.00	0.00	1.00	0.00	0.00	1.00	0.00	0.00	1.00	0.00	0.00	1.00	0.00	0.00	1.00	0.00	0.00
chr5	64987643	1549	G	0	1.00	0.00	0.00	1.00	0.00	0.00	1.00	0.00	0.00	1.00	0.00	0.00	1.00	0.00	0.00	1.00	0.00	0.00
chr8	120115606	1716	C	0	1.00	0.00	0.00	1.00	0.00	0.00	1.00	0.00	0.00	1.00	0.00	0.00	1.00	0.00	0.00	1.00	0.00	0.00
chr17	45706386	693	C	0	1.00	0.00	0.00	1.00	0.00	0.00	1.00	0.00	0.00	1.00	0.00	0.00	1.00	0.00	0.00	1.00	0.00	0.00
chr15	61868625	194	C	0	1.00	0.00	0.00	1.00	0.00	0.00	1.00	0.00	0.00	1.00	0.00	0.00	1.00	0.00	0.00	1.00	0.00	0.00
chr4	136547377	1035	C	0	1.00	0.00	0.00	1.00	0.00	0.00	1.00	0.00	0.00	1.00	0.00	0.00	1.00	0.00	0.00	1.00	0.00	0.00
chr4	136547420	1057	G	0	1.00	0.00	0.00	1.00	0.00	0.00	1.00	0.00	0.00	1.00	0.00	0.00	1.00	0.00	0.00	1.00	0.00	0.00
chr4	136547788	1218	C	0	1.00	0.00	0.00	1.00	0.00	0.00	1.00	0.00	0.00	1.00	0.00	0.00	1.00	0.00	0.00	1.00	0.00	0.00
chrX	7476595	2141	G	0	1.00	0.00	0.00	1.00	0.00	0.00	1.00	0.00	0.00	1.00	0.00	0.00	1.00	0.00	0.00	1.00	0.00	0.00
chr15	61868912	326	C	0	1.00	0.00	0.00	1.00	0.00	0.00	1.00	0.00	0.00	1.00	0.00	0.00	1.00	0.00	0.00	1.00	0.00	0.00
chr5	64987307	1403	G	0	1.00	0.00	0.00	1.00	0.00	0.00	1.00	0.00	0.00	1.00	0.00	0.00	1.00	0.00	0.00	1.00	0.00	0.00
chrX	7476314	2008	G	0	1.00	0.00	0.00	1.00	0.00	0.00	1.00	0.00	0.00	1.00	0.00	0.00	1.00	0.00	0.00	1.00	0.00	0.00
chr17	45706410	696	C	0	1.00	0.00	0.00	1.00	0.00	0.00	1.00	0.00	0.00	1.00	0.00	0.00	1.00	0.00	0.00	1.00	0.00	0.00
chr17	45706674	823	G	0	1.00	0.00	0.00	1.00	0.00	0.00	1.00	0.00	0.00	1.00	0.00	0.00	1.00	0.00	0.00	1.00	0.00	0.00
chr5	64987641	1548	C	0	1.00	0.00	0.00	1.00	0.00	0.00	1.00	0.00	0.00	1.00	0.00	0.00	1.00	0.00	0.00	1.00	0.00	0.00

0.99	0.00	0.01	0.99	0.00	0.00	0,00011
0.99	0.00	0.00	0.99	0.01	0.00	0,00011
0.99	0.00	0.00	0.99	0.00	0.00	0,00011
0.99	0.00	0.00	0.99	0.01	0.00	0,00011
0.99	0.00	0.00	0.99	0.00	0.00	0,00011
0.99	0.00	0.00	0.99	0.01	0.00	0,00011
0.99	0.00	0.00	0.99	0.00	0.00	0,00011
0.99	0.00	0.00	0.99	0.00	0.00	0,00011
0.99	0.00	0.00	0.99	0.00	0.00	0,00011
0.99	0.00	0.00	0.99	0.00	0.00	0,00011
0.99	0.00	0.00	0.99	0.00	0.00	0,00011
0.99	0.01	0.00	0.99	0.00	0.00	0,00011
0.99	0.00	0.00	0.99	0.00	0.00	0,00011
0.99	0.00	0.00	0.99	0.00	0.00	0,00011
0.99	0.00	0.00	0.99	0.00	0.00	0,00011
0.99	0.00	0.00	0.99	0.00	0.01	0,00011
0.99	0.00	0.00	0.99	0.00	0.00	0,00011
0.99	0.00	0.00	0.99	0.00	0.00	0,00011
0.99	0.00	0.00	0.99	0.00	0.00	0,00011
0.99	0.00	0.00	0.99	0.00	0.00	0,00011
0.99	0.00	0.00	0.99	0.00	0.00	0,00011
0.99	0.00	0.00	0.99	0.00	0.00	0,00011
0.99	0.00	0.00	0.99	0.00	0.00	0,00011
0.99	0.00	0.00	0.99	0.00	0.00	0,00011
0.99	0.00	0.00	0.99	0.00	0.00	0,00011
0.99	0.00	0.00	0.99	0.00	0.00	0,00011
0.99	0.00	0.00	0.99	0.00	0.00	0,00011
0.99	0.00	0.00	0.99	0.00	0.01	0,00011
0.99	0.00	0.00	0.99	0.00	0.00	0,00011
0.99	0.00	0.00	0.99	0.00	0.00	0,00011
0.99	0.00	0.00	0.99	0.00	0.00	0,0001
0.99	0.00	0.00	0.99	0.00	0.00	0,0001
0.99	0.00	0.00	0.99	0.00	0.00	0,0001
0.99	0.00	0.00	0.99	0.00	0.00	0,0001
0.99	0.00	0.00	0.99	0.00	0.00	0,0001
0.99	0.00	0.00	0.99	0.00	0.00	0,0001
0.99	0.00	0.00	0.99	0.00	0.00	0,0001
0.99	0.00	0.00	0.99	0.00	0.00	0,0001
0.99	0.00	0.00	0.99	0.00	0.00	0,0001
0.99	0.00	0.00	0.99	0.00	0.00	0,0001
0.99	0.00	0.00	0.99	0.01	0.00	0,0001
0.99	0.00	0.00	0.99	0.00	0.00	0,0001
0.99	0.00	0.00	0.99	0.00	0.00	0,0001
0.99	0.00	0.00	0.99	0.00	0.00	0,0001
0.99	0.00	0.00	0.99	0.00	0.00	0,0001
0.99	0.00	0.00	0.99	0.00	0.00	0,0001

chr5	64987657	1554	C	0	1.00	0.00	0.00	1.00	0.00	0.00	1.00	0.00	0.00	1.00	0.00	0.00	1.00	0.00	0.00	1.00	0.00	0.00
chr8	120115625	1727	C	0	1.00	0.00	0.00	1.00	0.00	0.00	1.00	0.00	0.00	1.00	0.00	0.00	1.00	0.00	0.00	1.00	0.00	0.00
chr1(	24277783	1017	g	0	1.00	0.00	0.00	1.00	0.00	0.00	1.00	0.00	0.00	1.00	0.00	0.00	1.00	0.00	0.00	1.00	0.00	0.00
chr4	136547388	1042	C	0	1.00	0.00	0.00	1.00	0.00	0.00	1.00	0.00	0.00	1.00	0.00	0.00	1.00	0.00	0.00	1.00	0.00	0.00
chr4	136547772	1215	C	0	1.00	0.00	0.00	1.00	0.00	0.00	1.00	0.00	0.00	1.00	0.00	0.00	1.00	0.00	0.00	1.00	0.00	0.00
chr1!	61868961	337	G	0	1.00	0.00	0.00	1.00	0.00	0.00	1.00	0.00	0.00	1.00	0.00	0.00	1.00	0.00	0.00	1.00	0.00	0.00
chr1(	24277714	1003	C	0	1.00	0.00	0.00	1.00	0.00	0.00	1.00	0.00	0.00	1.00	0.00	0.00	1.00	0.00	0.00	1.00	0.00	0.00
chr4	139783588	1224	c	0	1.00	0.00	0.00	1.00	0.00	0.00	1.00	0.00	0.00	1.00	0.00	0.00	1.00	0.00	0.00	1.00	0.00	0.00
chr1!	61868685	217	G	0	1.00	0.00	0.00	1.00	0.00	0.00	1.00	0.00	0.00	1.00	0.00	0.00	1.00	0.00	0.00	1.00	0.00	0.00
chr1!	61868972	339	G	0	1.00	0.00	0.00	1.00	0.00	0.00	1.00	0.00	0.00	1.00	0.00	0.00	1.00	0.00	0.00	1.00	0.00	0.00
chr1!	100300326	478	G	0	1.00	0.00	0.00	1.00	0.00	0.00	1.00	0.00	0.00	1.00	0.00	0.00	1.00	0.00	0.00	1.00	0.00	0.00
chr1;	45706743	857	C	0	1.00	0.00	0.00	1.00	0.00	0.00	1.00	0.00	0.00	1.00	0.00	0.00	1.00	0.00	0.00	1.00	0.00	0.00
chr1;	45706813	871	G	0	1.00	0.00	0.00	1.00	0.00	0.00	1.00	0.00	0.00	1.00	0.00	0.00	1.00	0.00	0.00	1.00	0.00	0.00
chr1(	24277779	1016	g	0	1.00	0.00	0.00	1.00	0.00	0.00	1.00	0.00	0.00	1.00	0.00	0.00	1.00	0.00	0.00	1.00	0.00	0.00
chr11	3525609	10	G	0	1.00	0.00	0.00	1.00	0.00	0.00	1.00	0.00	0.00	1.00	0.00	0.00	1.00	0.00	0.00	1.00	0.00	0.00
chr1!	61868889	314	C	0	1.00	0.00	0.00	1.00	0.00	0.00	1.00	0.00	0.00	1.00	0.00	0.00	1.00	0.00	0.00	1.00	0.00	0.00
chr8	89376788	1562	C	0	1.00	0.00	0.00	1.00	0.00	0.00	1.00	0.00	0.00	1.00	0.00	0.00	1.00	0.00	0.00	1.00	0.00	0.00
chr1!	61868624	193	C	0	1.00	0.00	0.00	1.00	0.00	0.00	1.00	0.00	0.00	1.00	0.00	0.00	1.00	0.00	0.00	1.00	0.00	0.00
chr1!	61868647	199	C	0	1.00	0.00	0.00	1.00	0.00	0.00	1.00	0.00	0.00	1.00	0.00	0.00	1.00	0.00	0.00	1.00	0.00	0.00
chr1;	45706744	858	C	0	1.00	0.00	0.00	1.00	0.00	0.00	1.00	0.00	0.00	1.00	0.00	0.00	1.00	0.00	0.00	1.00	0.00	0.00
chr1(	24277713	1002	C	0	1.00	0.00	0.00	1.00	0.00	0.00	1.00	0.00	0.00	1.00	0.00	0.00	1.00	0.00	0.00	1.00	0.00	0.00
chr8	120115569	1703	G	0	1.00	0.00	0.00	1.00	0.00	0.00	1.00	0.00	0.00	1.00	0.00	0.00	1.00	0.00	0.00	1.00	0.00	0.00
chr9	100498718	1997	c	0	1.00	0.00	0.00	1.00	0.00	0.00	1.00	0.00	0.00	1.00	0.00	0.00	1.00	0.00	0.00	1.00	0.00	0.00
chrX	7476281	1999	G	0	1.00	0.00	0.00	1.00	0.00	0.00	1.00	0.00	0.00	1.00	0.00	0.00	1.00	0.00	0.00	1.00	0.00	0.00
chr1!	100299995	351	G	0	1.00	0.00	0.00	1.00	0.00	0.00	1.00	0.00	0.00	1.00	0.00	0.00	1.00	0.00	0.00	1.00	0.00	0.00
chr5	64987658	1555	C	0	1.00	0.00	0.00	1.00	0.00	0.00	1.00	0.00	0.00	1.00	0.00	0.00	1.00	0.00	0.00	1.00	0.00	0.00
chrX	7476572	2127	G	0	1.00	0.00	0.00	1.00	0.00	0.00	1.00	0.00	0.00	1.00	0.00	0.00	1.00	0.00	0.00	1.00	0.00	0.00
chr5	64987359	1427	C	0	1.00	0.00	0.00	1.00	0.00	0.00	1.00	0.00	0.00	1.00	0.00	0.00	1.00	0.00	0.00	1.00	0.00	0.00
chr8	120115624	1726	C	0	1.00	0.00	0.00	1.00	0.00	0.00	1.00	0.00	0.00	1.00	0.00	0.00	1.00	0.00	0.00	1.00	0.00	0.00
chr11	3525594	2	G	0	1.00	0.00	0.00	1.00	0.00	0.00	1.00	0.00	0.00	1.00	0.00	0.00	1.00	0.00	0.00	1.00	0.00	0.00
chr1;	45706405	695	C	0	1.00	0.00	0.00	1.00	0.00	0.00	1.00	0.00	0.00	1.00	0.00	0.00	1.00	0.00	0.00	1.00	0.00	0.00
chr1;	45706474	714	G	0	1.00	0.00	0.00	1.00	0.00	0.00	1.00	0.00	0.00	1.00	0.00	0.00	1.00	0.00	0.00	1.00	0.00	0.00
chr4	139783601	1230	g	0	1.00	0.00	0.00	1.00	0.00	0.00	1.00	0.00	0.00	1.00	0.00	0.00	1.00	0.00	0.00	1.00	0.00	0.00
chr5	64987290	1399	G	0	1.00	0.00	0.00	1.00	0.00	0.00	1.00	0.00	0.00	1.00	0.00	0.00	1.00	0.00	0.00	1.00	0.00	0.00
chrX	7476482	2061	C	0	1.00	0.00	0.00	1.00	0.00	0.00	1.00	0.00	0.00	1.00	0.00	0.00	1.00	0.00	0.00	1.00	0.00	0.00
chrX	7476562	2120	C	0	1.00	0.00	0.00	1.00	0.00	0.00	1.00	0.00	0.00	1.00	0.00	0.00	0.99	0.00	0.00	1.00	0.00	0.00
chr1!	61868635	197	C	0	1.00	0.00	0.00	1.00	0.00	0.00	1.00	0.00	0.00	1.00	0.00	0.00	1.00	0.00	0.00	1.00	0.00	0.00
chr1!	61868648	200	C	0	1.00	0.00	0.00	1.00	0.00	0.00	1.00	0.00	0.00	1.00	0.00	0.00	1.00	0.00	0.00	1.00	0.00	0.00
chr4	139783668	1269	C	0	1.00	0.00	0.00	1.00	0.00	0.00	1.00	0.00	0.00	1.00	0.00	0.00	1.00	0.00	0.00	1.00	0.00	0.00
chr5	64987296	1401	G	0	1.00	0.00	0.00	1.00	0.00	0.00	1.00	0.00	0.00	1.00	0.00	0.00	1.00	0.00	0.00	1.00	0.00	0.00
chr5	64987635	1545	C	0	1.00	0.00	0.00	1.00	0.00	0.00	1.00	0.00	0.00	1.00	0.00	0.00	1.00	0.00	0.00	1.00	0.00	0.00
chr1!	61868908	324	C	0	1.00	0.00	0.00	1.00	0.00	0.00	1.00	0.00	0.00	1.00	0.00	0.00	1.00	0.00	0.00	1.00	0.00	0.00
chr1;	45706803	867	G	0	1.00	0.00	0.00	1.00	0.00	0.00	1.00	0.00	0.00	1.00	0.00	0.00	1.00	0.00	0.00	1.00	0.00	0.00
chr1;	45706804	868	G	0	1.00	0.00	0.00	1.00	0.00	0.00	1.00	0.00	0.00	1.00	0.00	0.00	1.00	0.00	0.00	1.00	0.00	0.00
chr1(	24277479	899	g	0	1.00	0.00	0.00	1.00	0.00	0.00	1.00	0.00	0.00	1.00	0.00	0.00	1.00	0.00	0.00	1.00	0.00	0.00
chr5	64987631	1542	G	0	1.00	0.00	0.00	1.00	0.00	0.00	1.00	0.00	0.00	1.00	0.00	0.00	1.00	0.00	0.00	1.00	0.00	0.00

0.99	0.00	0.00	0.99	0.00	0.00	0,0001
0.99	0.00	0.00	0.99	0.00	0.00	0,0001
0.99	0.00	0.00	0.99	0.00	0.00	9,9E-05
0.99	0.00	0.00	0.99	0.00	0.00	9,9E-05
0.99	0.00	0.00	0.99	0.00	0.00	9,9E-05
0.99	0.00	0.00	0.99	0.00	0.00	9,8E-05
0.99	0.00	0.00	0.99	0.00	0.00	9,8E-05
0.99	0.00	0.00	0.99	0.00	0.00	9,8E-05
0.99	0.00	0.00	0.99	0.00	0.00	9,7E-05
0.99	0.00	0.00	0.99	0.00	0.00	9,7E-05
0.99	0.00	0.00	0.99	0.00	0.00	9,7E-05
0.99	0.00	0.00	0.99	0.00	0.00	9,7E-05
0.99	0.00	0.00	0.99	0.00	0.00	9,7E-05
0.99	0.00	0.00	0.99	0.00	0.00	9,7E-05
0.99	0.00	0.00	0.99	0.00	0.00	9,6E-05
0.99	0.00	0.00	0.99	0.00	0.00	9,6E-05
0.99	0.00	0.00	0.99	0.00	0.00	9,6E-05
0.99	0.00	0.00	0.99	0.00	0.00	9,5E-05
0.99	0.00	0.00	0.99	0.00	0.00	9,5E-05
0.99	0.00	0.00	0.99	0.00	0.00	9,5E-05
0.99	0.00	0.00	0.99	0.00	0.00	9,5E-05
0.99	0.00	0.00	0.99	0.00	0.00	9,5E-05
0.99	0.00	0.00	0.99	0.00	0.00	9,5E-05
0.99	0.00	0.00	0.99	0.00	0.00	9,5E-05
0.99	0.00	0.00	0.99	0.00	0.00	9,4E-05
0.99	0.00	0.00	0.99	0.00	0.00	9,4E-05
0.99	0.00	0.00	0.99	0.00	0.00	9,4E-05
0.99	0.00	0.00	0.99	0.00	0.00	9,3E-05
0.99	0.00	0.00	0.99	0.00	0.00	9,3E-05
0.99	0.00	0.00	0.99	0.00	0.00	9,2E-05
0.99	0.00	0.00	0.99	0.00	0.00	9,2E-05
0.99	0.00	0.00	0.99	0.00	0.00	9,2E-05
0.99	0.00	0.00	0.99	0.00	0.00	9,2E-05
0.99	0.00	0.00	0.99	0.00	0.00	9,2E-05
0.99	0.00	0.00	0.99	0.00	0.00	9,2E-05
0.99	0.00	0.00	0.99	0.00	0.00	9,2E-05
0.99	0.00	0.00	0.99	0.00	0.00	9,1E-05
0.99	0.00	0.00	0.99	0.00	0.00	9,1E-05
0.99	0.00	0.00	0.99	0.00	0.00	9,1E-05
0.99	0.00	0.00	0.99	0.00	0.00	9,1E-05
0.99	0.00	0.00	0.99	0.00	0.00	9,1E-05
0.99	0.00	0.00	0.99	0.00	0.00	0,00009
0.99	0.00	0.00	0.99	0.00	0.00	0,00009
0.99	0.00	0.00	0.99	0.00	0.00	0,00009
0.99	0.00	0.00	0.99	0.00	0.00	0,00009
0.99	0.00	0.00	0.99	0.00	0.00	0,00009

chr5	64987637	1546	C	0	1.00	0.00	0.00	1.00	0.00	0.00	1.00	0.00	0.00	1.00	0.00	0.00	1.00	0.00	0.00	1.00	0.00	0.00
chr1'	3525912	180	C	0	1.00	0.00	0.00	1.00	0.00	0.00	1.00	0.00	0.00	1.00	0.00	0.00	1.00	0.00	0.00	1.00	0.00	0.00
chr1!	61868680	215	G	0	1.00	0.00	0.00	1.00	0.00	0.00	1.00	0.00	0.00	1.00	0.00	0.00	1.00	0.00	0.00	1.00	0.00	0.00
chr1!	61868905	322	C	0	1.00	0.00	0.00	1.00	0.00	0.00	1.00	0.00	0.00	1.00	0.00	0.00	1.00	0.00	0.00	1.00	0.00	0.00
chr8	89376799	1570	c	0	1.00	0.00	0.00	1.00	0.00	0.00	1.00	0.00	0.00	1.00	0.00	0.00	1.00	0.00	0.00	1.00	0.00	0.00
chrX	7539652	2423	G	0	1.00	0.00	0.00	1.00	0.00	0.00	1.00	0.00	0.00	1.00	0.00	0.00	1.00	0.00	0.00	1.00	0.00	0.00
chr1!	61868890	315	C	0	1.00	0.00	0.00	1.00	0.00	0.00	1.00	0.00	0.00	1.00	0.00	0.00	1.00	0.00	0.00	1.00	0.00	0.00
chr1;	45706467	710	G	0	1.00	0.00	0.00	1.00	0.00	0.00	1.00	0.00	0.00	1.00	0.00	0.00	1.00	0.00	0.00	1.00	0.00	0.00
chr5	64987640	1547	C	0	1.00	0.00	0.00	1.00	0.00	0.00	1.00	0.00	0.00	1.00	0.00	0.00	1.00	0.00	0.00	1.00	0.00	0.00
chr8	89376783	1560	G	0	1.00	0.00	0.00	1.00	0.00	0.00	1.00	0.00	0.00	1.00	0.00	0.00	1.00	0.00	0.00	1.00	0.00	0.00
chrX	7476688	2207	G	0	1.00	0.00	0.00	1.00	0.00	0.00	1.00	0.00	0.00	1.00	0.00	0.00	1.00	0.00	0.00	1.00	0.00	0.00
chr1'	3525591	1	G	0	1.00	0.00	0.00	1.00	0.00	0.00	1.00	0.00	0.00	1.00	0.00	0.00	1.00	0.00	0.00	1.00	0.00	0.00
chr1!	61868979	342	G	0	1.00	0.00	0.00	1.00	0.00	0.00	1.00	0.00	0.00	1.00	0.00	0.00	1.00	0.00	0.00	1.00	0.00	0.00
chr1(	24277469	894	g	0	1.00	0.00	0.00	1.00	0.00	0.00	1.00	0.00	0.00	1.00	0.00	0.00	1.00	0.00	0.00	1.00	0.00	0.00
chr4	136547394	1046	C	0	1.00	0.00	0.00	1.00	0.00	0.00	1.00	0.00	0.00	1.00	0.00	0.00	1.00	0.00	0.00	1.00	0.00	0.00
chr5	64987325	1410	C	0	1.00	0.00	0.00	1.00	0.00	0.00	1.00	0.00	0.00	1.00	0.00	0.00	1.00	0.00	0.00	1.00	0.00	0.00
chr8	89376798	1569	g	0	1.00	0.00	0.00	1.00	0.00	0.00	1.00	0.00	0.00	1.00	0.00	0.00	1.00	0.00	0.00	1.00	0.00	0.00
chrX	7476282	2000	G	0	1.00	0.00	0.00	1.00	0.00	0.00	1.00	0.00	0.00	1.00	0.00	0.00	1.00	0.00	0.00	1.00	0.00	0.00
chr8	89377057	1684	C	0	1.00	0.00	0.00	1.00	0.00	0.00	1.00	0.00	0.00	1.00	0.00	0.00	1.00	0.00	0.00	1.00	0.00	0.00
chrX	7539250	2216	G	0	1.00	0.00	0.00	1.00	0.00	0.00	1.00	0.00	0.00	1.00	0.00	0.00	1.00	0.00	0.00	1.00	0.00	0.00
chrX	7539649	2422	G	0	1.00	0.00	0.00	1.00	0.00	0.00	1.00	0.00	0.00	1.00	0.00	0.00	1.00	0.00	0.00	1.00	0.00	0.00
chr1(	24277775	1014	g	0	1.00	0.00	0.00	1.00	0.00	0.00	1.00	0.00	0.00	1.00	0.00	0.00	1.00	0.00	0.00	1.00	0.00	0.00
chr4	139783896	1391	C	0	1.00	0.00	0.00	1.00	0.00	0.00	1.00	0.00	0.00	1.00	0.00	0.00	1.00	0.00	0.00	1.00	0.00	0.00
chr1;	45706403	694	C	0	1.00	0.00	0.00	1.00	0.00	0.00	1.00	0.00	0.00	1.00	0.00	0.00	1.00	0.00	0.00	1.00	0.00	0.00
chr1(	24277468	893	g	0	1.00	0.00	0.00	1.00	0.00	0.00	1.00	0.00	0.00	1.00	0.00	0.00	1.00	0.00	0.00	1.00	0.00	0.00
chr5	64987661	1556	C	0	1.00	0.00	0.00	1.00	0.00	0.00	1.00	0.00	0.00	1.00	0.00	0.00	1.00	0.00	0.00	1.00	0.00	0.00
chrX	7539243	2214	G	0	1.00	0.00	0.00	1.00	0.00	0.00	1.00	0.00	0.00	1.00	0.00	0.00	1.00	0.00	0.00	1.00	0.00	0.00
chr1;	45706820	872	G	0	1.00	0.00	0.00	1.00	0.00	0.00	1.00	0.00	0.00	1.00	0.00	0.00	1.00	0.00	0.00	1.00	0.00	0.00
chr4	136547591	1159	G	0	1.00	0.00	0.00	1.00	0.00	0.00	1.00	0.00	0.00	1.00	0.00	0.00	1.00	0.00	0.00	1.00	0.00	0.00
chr8	89376790	1563	c	0	1.00	0.00	0.00	1.00	0.00	0.00	1.00	0.00	0.00	1.00	0.00	0.00	1.00	0.00	0.00	1.00	0.00	0.00
chr8	89376791	1564	c	0	1.00	0.00	0.00	1.00	0.00	0.00	1.00	0.00	0.00	1.00	0.00	0.00	1.00	0.00	0.00	1.00	0.00	0.00
chr1!	61868634	196	C	0	1.00	0.00	0.00	1.00	0.00	0.00	1.00	0.00	0.00	1.00	0.00	0.00	1.00	0.00	0.00	1.00	0.00	0.00
chr1!	100300318	474	G	0	1.00	0.00	0.00	1.00	0.00	0.00	1.00	0.00	0.00	1.00	0.00	0.00	1.00	0.00	0.00	1.00	0.00	0.00
chr9	100498375	1854	C	0	1.00	0.00	0.00	1.00	0.00	0.00	1.00	0.00	0.00	1.00	0.00	0.00	1.00	0.00	0.00	1.00	0.00	0.00
chr9	100498720	1998	c	0	1.00	0.00	0.00	1.00	0.00	0.00	1.00	0.00	0.00	1.00	0.00	0.00	1.00	0.00	0.00	1.00	0.00	0.00
chrX	7476287	2001	G	0	1.00	0.00	0.00	1.00	0.00	0.00	1.00	0.00	0.00	1.00	0.00	0.00	1.00	0.00	0.00	1.00	0.00	0.00
chrX	7476681	2204	G	0	1.00	0.00	0.00	1.00	0.00	0.00	1.00	0.00	0.00	1.00	0.00	0.00	1.00	0.00	0.00	1.00	0.00	0.00
chrX	7539353	2244	C	0	1.00	0.00	0.00	1.00	0.00	0.00	1.00	0.00	0.00	1.00	0.00	0.00	1.00	0.00	0.00	1.00	0.00	0.00
chr1!	61868975	340	G	0	1.00	0.00	0.00	1.00	0.00	0.00	1.00	0.00	0.00	1.00	0.00	0.00	1.00	0.00	0.00	1.00	0.00	0.00
chr1!	61868976	341	G	0	1.00	0.00	0.00	1.00	0.00	0.00	1.00	0.00	0.00	1.00	0.00	0.00	1.00	0.00	0.00	1.00	0.00	0.00
chr1(	24277368	877	c	0	1.00	0.00	0.00	1.00	0.00	0.00	1.00	0.00	0.00	1.00	0.00	0.00	1.00	0.00	0.00	1.00	0.00	0.00
chr8	89376781	1559	G	0	1.00	0.00	0.00	1.00	0.00	0.00	1.00	0.00	0.00	1.00	0.00	0.00	1.00	0.00	0.00	1.00	0.00	0.00
chr8	89377047	1677	C	0	1.00	0.00	0.00	1.00	0.00	0.00	1.00	0.00	0.00	1.00	0.00	0.00	1.00	0.00	0.00	1.00	0.00	0.00
chr8	120115555	1697	G	0	1.00	0.00	0.00	1.00	0.00	0.00	1.00	0.00	0.00	1.00	0.00	0.00	1.00	0.00	0.00	1.00	0.00	0.00
chr9	100498566	1959	G	0	1.00	0.00	0.00	1.00	0.00	0.00	1.00	0.00	0.00	1.00	0.00	0.00	1.00	0.00	0.00	1.00	0.00	0.00
chrX	7539632	2414	G	0	1.00	0.00	0.00	1.00	0.00	0.00	1.00	0.00	0.00	1.00	0.00	0.00	1.00	0.00	0.00	1.00	0.00	0.00

0.99	0.00	0.00	0.99	0.00	0.00	0,00009
0.99	0.00	0.00	0.99	0.00	0.00	8,9E-05
0.99	0.00	0.00	0.99	0.00	0.00	8,9E-05
0.99	0.00	0.00	0.99	0.00	0.00	8,9E-05
0.99	0.00	0.00	0.99	0.00	0.00	8,9E-05
0.99	0.00	0.00	0.99	0.00	0.00	8,9E-05
0.99	0.00	0.00	0.99	0.00	0.00	8,8E-05
0.99	0.00	0.00	0.99	0.00	0.00	8,8E-05
0.99	0.00	0.00	0.99	0.00	0.00	8,8E-05
0.99	0.00	0.00	0.99	0.00	0.00	8,8E-05
0.99	0.00	0.00	0.99	0.00	0.00	8,8E-05
0.99	0.00	0.00	0.99	0.00	0.00	8,7E-05
0.99	0.00	0.00	0.99	0.00	0.00	8,7E-05
0.99	0.00	0.00	0.99	0.00	0.00	8,7E-05
0.99	0.00	0.00	0.99	0.00	0.00	8,7E-05
0.99	0.00	0.00	0.99	0.00	0.00	8,7E-05
0.99	0.00	0.00	0.99	0.00	0.00	8,7E-05
0.99	0.00	0.00	0.99	0.00	0.00	8,7E-05
0.99	0.00	0.00	0.99	0.00	0.00	8,6E-05
0.99	0.00	0.00	0.99	0.00	0.00	8,6E-05
0.99	0.00	0.00	0.99	0.00	0.00	8,6E-05
0.99	0.00	0.00	0.99	0.00	0.00	8,5E-05
0.99	0.00	0.00	0.99	0.00	0.00	8,5E-05
0.99	0.00	0.00	0.99	0.00	0.00	8,4E-05
0.99	0.00	0.00	0.99	0.00	0.00	8,4E-05
0.99	0.00	0.00	0.99	0.00	0.00	8,3E-05
0.99	0.00	0.00	0.99	0.00	0.00	8,3E-05
0.99	0.00	0.00	0.99	0.00	0.00	8,2E-05
0.99	0.00	0.00	0.99	0.00	0.00	8,2E-05
0.99	0.00	0.00	0.99	0.00	0.00	8,2E-05
0.99	0.00	0.00	0.99	0.00	0.00	8,2E-05
0.99	0.00	0.00	0.99	0.00	0.00	8,1E-05
0.99	0.00	0.00	0.99	0.00	0.00	8,1E-05
0.99	0.00	0.00	0.99	0.00	0.00	8,1E-05
0.99	0.00	0.00	0.99	0.00	0.00	8,1E-05
0.99	0.00	0.00	0.99	0.00	0.00	8,1E-05
0.99	0.00	0.00	0.99	0.00	0.00	8,1E-05
0.99	0.00	0.00	0.99	0.00	0.00	8,1E-05
0.99	0.00	0.00	0.99	0.00	0.00	0,00008
0.99	0.00	0.00	0.99	0.00	0.00	0,00008
0.99	0.00	0.00	0.99	0.00	0.00	0,00008
0.99	0.00	0.00	0.99	0.00	0.00	0,00008
0.99	0.00	0.00	0.99	0.00	0.00	0,00008
0.99	0.00	0.00	0.99	0.00	0.00	0,00008
0.99	0.00	0.00	0.99	0.00	0.00	0,00008
0.99	0.00	0.00	0.99	0.00	0.00	0,00008

chr15	61868969	338	G	0	1.00	0.00	0.00	1.00	0.00	0.00	1.00	0.00	0.00	1.00	0.00	0.00	1.00	0.00	0.00	1.00	0.00	0.00
chr15	100300330	479	G	0	1.00	0.00	0.00	1.00	0.00	0.00	1.00	0.00	0.00	1.00	0.00	0.00	1.00	0.00	0.00	1.00	0.00	0.00
chr9	100498574	1962	G	0	1.00	0.00	0.00	1.00	0.00	0.00	1.00	0.00	0.00	1.00	0.00	0.00	1.00	0.00	0.00	1.00	0.00	0.00
chr15	61868980	343	G	0	1.00	0.00	0.00	1.00	0.00	0.00	1.00	0.00	0.00	1.00	0.00	0.00	1.00	0.00	0.00	1.00	0.00	0.00
chr17	45706478	717	G	0	1.00	0.00	0.00	1.00	0.00	0.00	1.00	0.00	0.00	1.00	0.00	0.00	1.00	0.00	0.00	1.00	0.00	0.00
chr8	89377052	1681	C	0	1.00	0.00	0.00	1.00	0.00	0.00	1.00	0.00	0.00	1.00	0.00	0.00	1.00	0.00	0.00	1.00	0.00	0.00
chrX	7476675	2200	G	0	1.00	0.00	0.00	1.00	0.00	0.00	1.00	0.00	0.00	1.00	0.00	0.00	1.00	0.00	0.00	1.00	0.00	0.00
chr19	24277694	993	C	0	1.00	0.00	0.00	1.00	0.00	0.00	1.00	0.00	0.00	1.00	0.00	0.00	1.00	0.00	0.00	1.00	0.00	0.00
chr19	24277778	1015	g	0	1.00	0.00	0.00	1.00	0.00	0.00	1.00	0.00	0.00	1.00	0.00	0.00	1.00	0.00	0.00	1.00	0.00	0.00
chr4	136547332	1022	G	0	1.00	0.00	0.00	1.00	0.00	0.00	1.00	0.00	0.00	1.00	0.00	0.00	1.00	0.00	0.00	1.00	0.00	0.00
chr8	89376778	1557	G	0	1.00	0.00	0.00	1.00	0.00	0.00	1.00	0.00	0.00	1.00	0.00	0.00	1.00	0.00	0.00	1.00	0.00	0.00
chrX	7539248	2215	G	0	1.00	0.00	0.00	1.00	0.00	0.00	1.00	0.00	0.00	1.00	0.00	0.00	1.00	0.00	0.00	1.00	0.00	0.00
chr15	61868632	195	C	0	1.00	0.00	0.00	1.00	0.00	0.00	1.00	0.00	0.00	1.00	0.00	0.00	1.00	0.00	0.00	1.00	0.00	0.00
chr15	100300322	476	G	0	1.00	0.00	0.00	1.00	0.00	0.00	1.00	0.00	0.00	1.00	0.00	0.00	1.00	0.00	0.00	1.00	0.00	0.00
chr17	45706808	869	G	0	1.00	0.00	0.00	1.00	0.00	0.00	1.00	0.00	0.00	1.00	0.00	0.00	1.00	0.00	0.00	1.00	0.00	0.00
chr19	24277692	992	C	0	1.00	0.00	0.00	1.00	0.00	0.00	1.00	0.00	0.00	1.00	0.00	0.00	1.00	0.00	0.00	1.00	0.00	0.00
chr9	100498387	1861	C	0	1.00	0.00	0.00	1.00	0.00	0.00	1.00	0.00	0.00	1.00	0.00	0.00	1.00	0.00	0.00	1.00	0.00	0.00
chrX	7476289	2002	G	0	1.00	0.00	0.00	1.00	0.00	0.00	1.00	0.00	0.00	1.00	0.00	0.00	1.00	0.00	0.00	1.00	0.00	0.00
chrX	7476678	2202	G	0	1.00	0.00	0.00	1.00	0.00	0.00	1.00	0.00	0.00	1.00	0.00	0.00	1.00	0.00	0.00	1.00	0.00	0.00
chrX	7539258	2217	G	0	1.00	0.00	0.00	1.00	0.00	0.00	1.00	0.00	0.00	1.00	0.00	0.00	1.00	0.00	0.00	1.00	0.00	0.00
chrX	7539633	2415	G	0	1.00	0.00	0.00	1.00	0.00	0.00	1.00	0.00	0.00	1.00	0.00	0.00	1.00	0.00	0.00	1.00	0.00	0.00
chr4	136547321	1020	G	0	1.00	0.00	0.00	1.00	0.00	0.00	1.00	0.00	0.00	1.00	0.00	0.00	1.00	0.00	0.00	1.00	0.00	0.00
chr4	139783578	1221	c	0	1.00	0.00	0.00	1.00	0.00	0.00	1.00	0.00	0.00	1.00	0.00	0.00	1.00	0.00	0.00	1.00	0.00	0.00
chr4	139783898	1393	C	0	1.00	0.00	0.00	1.00	0.00	0.00	1.00	0.00	0.00	1.00	0.00	0.00	1.00	0.00	0.00	1.00	0.00	0.00
chr11	3525910	179	C	0	1.00	0.00	0.00	1.00	0.00	0.00	1.00	0.00	0.00	1.00	0.00	0.00	1.00	0.00	0.00	1.00	0.00	0.00
chr15	61868679	214	G	0	1.00	0.00	0.00	1.00	0.00	0.00	1.00	0.00	0.00	1.00	0.00	0.00	1.00	0.00	0.00	1.00	0.00	0.00
chrX	7476685	2206	C	0	1.00	0.00	0.00	1.00	0.00	0.00	1.00	0.00	0.00	1.00	0.00	0.00	1.00	0.00	0.00	1.00	0.00	0.00
chr15	100300324	477	G	0	1.00	0.00	0.00	1.00	0.00	0.00	1.00	0.00	0.00	1.00	0.00	0.00	1.00	0.00	0.00	1.00	0.00	0.00
chr17	45706809	870	G	0	1.00	0.00	0.00	1.00	0.00	0.00	1.00	0.00	0.00	1.00	0.00	0.00	1.00	0.00	0.00	1.00	0.00	0.00
chr4	139783592	1227	g	0	1.00	0.00	0.00	1.00	0.00	0.00	1.00	0.00	0.00	1.00	0.00	0.00	1.00	0.00	0.00	1.00	0.00	0.00
chr8	89376785	1561	C	0	1.00	0.00	0.00	1.00	0.00	0.00	1.00	0.00	0.00	1.00	0.00	0.00	1.00	0.00	0.00	1.00	0.00	0.00
chr15	100299997	352	G	0	1.00	0.00	0.00	1.00	0.00	0.00	1.00	0.00	0.00	1.00	0.00	0.00	1.00	0.00	0.00	1.00	0.00	0.00
chr15	100300002	355	G	0	1.00	0.00	0.00	1.00	0.00	0.00	1.00	0.00	0.00	1.00	0.00	0.00	1.00	0.00	0.00	1.00	0.00	0.00
chr4	139783897	1392	C	0	1.00	0.00	0.00	1.00	0.00	0.00	1.00	0.00	0.00	1.00	0.00	0.00	1.00	0.00	0.00	1.00	0.00	0.00
chr8	89377053	1682	C	0	1.00	0.00	0.00	1.00	0.00	0.00	1.00	0.00	0.00	1.00	0.00	0.00	1.00	0.00	0.00	1.00	0.00	0.00
chr8	89377081	1691	G	0	1.00	0.00	0.00	1.00	0.00	0.00	1.00	0.00	0.00	1.00	0.00	0.00	1.00	0.00	0.00	1.00	0.00	0.00
chr8	89377093	1693	G	0	1.00	0.00	0.00	1.00	0.00	0.00	1.00	0.00	0.00	1.00	0.00	0.00	1.00	0.00	0.00	1.00	0.00	0.00
chrX	7476694	2211	G	0	1.00	0.00	0.00	1.00	0.00	0.00	1.00	0.00	0.00	1.00	0.00	0.00	1.00	0.00	0.00	1.00	0.00	0.00
chrX	7539362	2250	C	0	1.00	0.00	0.00	1.00	0.00	0.00	1.00	0.00	0.00	1.00	0.00	0.00	1.00	0.00	0.00	1.00	0.00	0.00
chr11	3525599	5	C	0	1.00	0.00	0.00	1.00	0.00	0.00	1.00	0.00	0.00	1.00	0.00	0.00	1.00	0.00	0.00	1.00	0.00	0.00
chr4	139783571	1220	c	0	1.00	0.00	0.00	1.00	0.00	0.00	1.00	0.00	0.00	1.00	0.00	0.00	1.00	0.00	0.00	1.00	0.00	0.00
chr8	89376796	1568	c	0	1.00	0.00	0.00	1.00	0.00	0.00	1.00	0.00	0.00	1.00	0.00	0.00	1.00	0.00	0.00	1.00	0.00	0.00
chr8	89377091	1692	G	0	1.00	0.00	0.00	1.00	0.00	0.00	1.00	0.00	0.00	1.00	0.00	0.00	1.00	0.00	0.00	1.00	0.00	0.00
chr8	89377094	1694	G	0	1.00	0.00	0.00	1.00	0.00	0.00	1.00	0.00	0.00	1.00	0.00	0.00	1.00	0.00	0.00	1.00	0.00	0.00
chr8	120115846	1833	G	0	1.00	0.00	0.00	1.00	0.00	0.00	1.00	0.00	0.00	1.00	0.00	0.00	1.00	0.00	0.00	1.00	0.00	0.00
chrX	7539667	2427	C	0	1.00	0.00	0.00	1.00	0.00	0.00	1.00	0.00	0.00	1.00	0.00	0.00	1.00	0.00	0.00	1.00	0.00	0.00

0.99	0.00	0.00	0.99	0.00	0.00	7,9E-05
0.99	0.00	0.00	0.99	0.00	0.00	7,9E-05
0.99	0.00	0.00	0.99	0.00	0.00	7,9E-05
0.99	0.00	0.00	0.99	0.00	0.00	7,8E-05
0.99	0.00	0.00	0.99	0.00	0.01	7,8E-05
0.99	0.00	0.00	0.99	0.00	0.00	7,8E-05
0.99	0.00	0.00	0.99	0.00	0.00	7,8E-05
0.99	0.00	0.00	0.99	0.00	0.00	7,7E-05
0.99	0.00	0.00	0.99	0.00	0.00	7,7E-05
0.99	0.00	0.00	0.99	0.00	0.00	7,7E-05
0.99	0.00	0.00	0.99	0.00	0.00	7,7E-05
0.99	0.00	0.00	0.99	0.00	0.00	7,7E-05
0.99	0.00	0.00	0.99	0.00	0.00	7,6E-05
0.99	0.00	0.00	0.99	0.00	0.00	7,6E-05
0.99	0.00	0.00	0.99	0.00	0.00	7,6E-05
0.99	0.00	0.00	0.99	0.00	0.00	7,6E-05
0.99	0.00	0.00	0.99	0.00	0.00	7,6E-05
0.99	0.00	0.00	0.99	0.00	0.00	7,6E-05
0.99	0.00	0.00	0.99	0.00	0.00	7,6E-05
0.99	0.00	0.00	0.99	0.00	0.00	7,6E-05
0.99	0.00	0.00	0.99	0.00	0.00	7,6E-05
0.99	0.00	0.00	0.99	0.00	0.00	7,5E-05
0.99	0.00	0.00	0.99	0.00	0.00	7,5E-05
0.99	0.00	0.00	0.99	0.00	0.00	7,5E-05
0.99	0.00	0.00	0.99	0.00	0.00	7,4E-05
0.99	0.00	0.00	0.99	0.00	0.00	7,4E-05
0.99	0.00	0.00	0.99	0.00	0.00	7,4E-05
0.99	0.00	0.00	0.99	0.00	0.00	7,3E-05
0.99	0.00	0.00	0.99	0.00	0.00	7,3E-05
0.99	0.00	0.00	0.99	0.00	0.00	7,3E-05
0.99	0.00	0.00	0.99	0.00	0.00	7,3E-05
0.99	0.00	0.00	0.99	0.00	0.00	7,2E-05
0.99	0.00	0.00	0.99	0.00	0.00	7,2E-05
0.99	0.00	0.00	0.99	0.00	0.00	7,2E-05
0.99	0.00	0.00	0.99	0.00	0.00	7,2E-05
0.99	0.00	0.00	0.99	0.00	0.00	7,2E-05
0.99	0.00	0.00	0.99	0.00	0.00	7,2E-05
0.99	0.00	0.00	0.99	0.00	0.00	7,2E-05
0.99	0.00	0.00	0.99	0.00	0.00	7,2E-05
0.99	0.00	0.00	0.99	0.00	0.00	7,1E-05
0.99	0.00	0.00	0.99	0.00	0.00	7,1E-05
0.99	0.00	0.00	0.99	0.00	0.00	7,1E-05
0.99	0.00	0.00	0.99	0.00	0.00	7,1E-05
0.99	0.00	0.00	0.99	0.00	0.00	7,1E-05
0.99	0.00	0.00	0.99	0.00	0.00	7,1E-05
0.99	0.00	0.00	0.99	0.00	0.00	7,1E-05

chr11	3525909	178	C	0	1.00	0.00	0.00	1.00	0.00	0.00	1.00	0.00	0.00	1.00	0.00	0.00	1.00	0.00	0.00	1.00	0.00	0.00
chr9	100498295	1834	G	0	1.00	0.00	0.00	1.00	0.00	0.00	1.00	0.00	0.00	1.00	0.00	0.00	1.00	0.00	0.00	1.00	0.00	0.00
chr15	100300003	356	G	0	1.00	0.00	0.00	1.00	0.00	0.00	1.00	0.00	0.00	1.00	0.00	0.00	1.00	0.00	0.00	1.00	0.00	0.00
chr15	100300312	471	G	0	1.00	0.00	0.00	1.00	0.00	0.00	1.00	0.00	0.00	1.00	0.00	0.00	1.00	0.00	0.00	1.00	0.00	0.00
chr4	139783884	1386	C	0	1.00	0.00	0.00	1.00	0.00	0.00	1.00	0.00	0.00	1.00	0.00	0.00	1.00	0.00	0.00	1.00	0.00	0.00
chr9	100498560	1954	G	0	1.00	0.00	0.00	1.00	0.00	0.00	1.00	0.00	0.00	1.00	0.00	0.00	1.00	0.00	0.00	1.00	0.00	0.00
chr4	139783904	1397	C	0	1.00	0.00	0.00	1.00	0.00	0.00	1.00	0.00	0.00	1.00	0.00	0.00	1.00	0.00	0.00	1.00	0.00	0.00
chr8	89377058	1685	C	0	1.00	0.00	0.00	1.00	0.00	0.00	1.00	0.00	0.00	1.00	0.00	0.00	1.00	0.00	0.00	1.00	0.00	0.00
chr8	120115562	1699	G	0	1.00	0.00	0.00	1.00	0.00	0.00	1.00	0.00	0.00	1.00	0.00	0.00	1.00	0.00	0.00	1.00	0.00	0.00
chrX	7476673	2198	G	0	1.00	0.00	0.00	1.00	0.00	0.00	1.00	0.00	0.00	1.00	0.00	0.00	1.00	0.00	0.00	1.00	0.00	0.00
chrX	7539365	2252	C	0	1.00	0.00	0.00	1.00	0.00	0.00	1.00	0.00	0.00	1.00	0.00	0.00	1.00	0.00	0.00	1.00	0.00	0.00
chrX	7539366	2253	C	0	1.00	0.00	0.00	1.00	0.00	0.00	1.00	0.00	0.00	1.00	0.00	0.00	1.00	0.00	0.00	1.00	0.00	0.00
chrX	7539666	2426	C	0	1.00	0.00	0.00	1.00	0.00	0.00	1.00	0.00	0.00	1.00	0.00	0.00	1.00	0.00	0.00	1.00	0.00	0.00
chr5	64987623	1539	G	0	1.00	0.00	0.00	1.00	0.00	0.00	1.00	0.00	0.00	1.00	0.00	0.00	1.00	0.00	0.00	1.00	0.00	0.00
chr9	100498398	1867	C	0	1.00	0.00	0.00	1.00	0.00	0.00	1.00	0.00	0.00	1.00	0.00	0.00	1.00	0.00	0.00	1.00	0.00	0.00
chrX	7539345	2239	C	0	1.00	0.00	0.00	1.00	0.00	0.00	1.00	0.00	0.00	1.00	0.00	0.00	1.00	0.00	0.00	1.00	0.00	0.00
chrX	7539364	2251	C	0	1.00	0.00	0.00	1.00	0.00	0.00	1.00	0.00	0.00	1.00	0.00	0.00	1.00	0.00	0.00	1.00	0.00	0.00
chrX	7539664	2425	C	0	1.00	0.00	0.00	1.00	0.00	0.00	1.00	0.00	0.00	1.00	0.00	0.00	1.00	0.00	0.00	1.00	0.00	0.00
chr4	136547319	1018	G	0	1.00	0.00	0.00	1.00	0.00	0.00	1.00	0.00	0.00	1.00	0.00	0.00	1.00	0.00	0.00	1.00	0.00	0.00
chr4	136547320	1019	G	0	1.00	0.00	0.00	1.00	0.00	0.00	1.00	0.00	0.00	1.00	0.00	0.00	1.00	0.00	0.00	1.00	0.00	0.00
chr4	139783570	1219	c	0	1.00	0.00	0.00	1.00	0.00	0.00	1.00	0.00	0.00	1.00	0.00	0.00	1.00	0.00	0.00	1.00	0.00	0.00
chr4	139783585	1222	c	0	1.00	0.00	0.00	1.00	0.00	0.00	1.00	0.00	0.00	1.00	0.00	0.00	1.00	0.00	0.00	1.00	0.00	0.00
chr4	139783587	1223	c	0	1.00	0.00	0.00	1.00	0.00	0.00	1.00	0.00	0.00	1.00	0.00	0.00	1.00	0.00	0.00	1.00	0.00	0.00
chr4	139783603	1232	g	0	1.00	0.00	0.00	1.00	0.00	0.00	1.00	0.00	0.00	1.00	0.00	0.00	1.00	0.00	0.00	1.00	0.00	0.00
chr9	100498392	1864	C	0	1.00	0.00	0.00	1.00	0.00	0.00	1.00	0.00	0.00	1.00	0.00	0.00	1.00	0.00	0.00	1.00	0.00	0.00
chrX	7476440	2040	C	0	1.00	0.00	0.00	1.00	0.00	0.00	1.00	0.00	0.00	1.00	0.00	0.00	1.00	0.00	0.00	1.00	0.00	0.00
chrX	7539675	2428	C	0	1.00	0.00	0.00	1.00	0.00	0.00	1.00	0.00	0.00	1.00	0.00	0.00	1.00	0.00	0.00	1.00	0.00	0.00
chr8	89376794	1566	g	0	1.00	0.00	0.00	1.00	0.00	0.00	1.00	0.00	0.00	1.00	0.00	0.00	1.00	0.00	0.00	1.00	0.00	0.00
chr9	100498551	1950	G	0	1.00	0.00	0.00	1.00	0.00	0.00	1.00	0.00	0.00	1.00	0.00	0.00	1.00	0.00	0.00	1.00	0.00	0.00
chr9	100498565	1958	G	0	1.00	0.00	0.00	1.00	0.00	0.00	1.00	0.00	0.00	1.00	0.00	0.00	1.00	0.00	0.00	1.00	0.00	0.00
chr9	100498697	1994	c	0	1.00	0.00	0.00	1.00	0.00	0.00	1.00	0.00	0.00	1.00	0.00	0.00	1.00	0.00	0.00	1.00	0.00	0.00
chr15	61868687	218	G	0	1.00	0.00	0.00	1.00	0.00	0.00	1.00	0.00	0.00	1.00	0.00	0.00	1.00	0.00	0.00	1.00	0.00	0.00
chr4	139783602	1231	g	0	1.00	0.00	0.00	1.00	0.00	0.00	1.00	0.00	0.00	1.00	0.00	0.00	1.00	0.00	0.00	1.00	0.00	0.00
chr8	120115553	1696	G	0	1.00	0.00	0.00	1.00	0.00	0.00	1.00	0.00	0.00	1.00	0.00	0.00	1.00	0.00	0.00	1.00	0.00	0.00
chr9	100498390	1863	C	0	1.00	0.00	0.00	1.00	0.00	0.00	1.00	0.00	0.00	1.00	0.00	0.00	1.00	0.00	0.00	1.00	0.00	0.00
chr11	3525915	181	C	0	1.00	0.00	0.00	1.00	0.00	0.00	1.00	0.00	0.00	1.00	0.00	0.00	1.00	0.00	0.00	1.00	0.00	0.00
chr4	136547594	1161	G	0	1.00	0.00	0.00	1.00	0.00	0.00	1.00	0.00	0.00	1.00	0.00	0.00	1.00	0.00	0.00	1.00	0.00	0.00
chr8	120115560	1698	G	0	1.00	0.00	0.00	1.00	0.00	0.00	1.00	0.00	0.00	1.00	0.00	0.00	1.00	0.00	0.00	1.00	0.00	0.00
chr9	100498306	1836	G	0	1.00	0.00	0.00	1.00	0.00	0.00	1.00	0.00	0.00	1.00	0.00	0.00	1.00	0.00	0.00	1.00	0.00	0.00
chrX	7476451	2045	C	0	1.00	0.00	0.00	1.00	0.00	0.00	1.00	0.00	0.00	1.00	0.00	0.00	1.00	0.00	0.00	1.00	0.00	0.00
chr4	136547596	1162	G	0	1.00	0.00	0.00	1.00	0.00	0.00	1.00	0.00	0.00	1.00	0.00	0.00	1.00	0.00	0.00	1.00	0.00	0.00
chr4	136547604	1166	G	0	1.00	0.00	0.00	1.00	0.00	0.00	1.00	0.00	0.00	1.00	0.00	0.00	1.00	0.00	0.00	1.00	0.00	0.00
chr4	139783878	1385	G	0	1.00	0.00	0.00	1.00	0.00	0.00	1.00	0.00	0.00	1.00	0.00	0.00	1.00	0.00	0.00	1.00	0.00	0.00
chr8	120115829	1825	G	0	1.00	0.00	0.00	1.00	0.00	0.00	1.00	0.00	0.00	1.00	0.00	0.00	1.00	0.00	0.00	1.00	0.00	0.00
chr9	100498307	1837	G	0	1.00	0.00	0.00	1.00	0.00	0.00	1.00	0.00	0.00	1.00	0.00	0.00	1.00	0.00	0.00	1.00	0.00	0.00
chr9	100498703	1995	c	0	1.00	0.00	0.00	1.00	0.00	0.00	1.00	0.00	0.00	1.00	0.00	0.00	1.00	0.00	0.00	1.00	0.00	0.00

0.99	0.00	0.00	0.99	0.00	0.00	0,00007
0.99	0.00	0.00	0.99	0.00	0.00	0,00007
0.99	0.00	0.00	0.99	0.00	0.00	6,9E-05
0.99	0.00	0.00	0.99	0.00	0.00	6,9E-05
0.99	0.00	0.00	0.99	0.00	0.00	6,9E-05
0.99	0.00	0.00	0.99	0.00	0.00	6,9E-05
0.99	0.00	0.00	0.99	0.00	0.00	6,8E-05
0.99	0.00	0.00	0.99	0.00	0.00	6,8E-05
0.99	0.00	0.00	0.99	0.00	0.00	6,8E-05
0.99	0.00	0.00	0.99	0.00	0.00	6,8E-05
0.99	0.00	0.00	0.99	0.00	0.00	6,8E-05
0.99	0.00	0.00	0.99	0.00	0.00	6,8E-05
0.99	0.00	0.00	0.99	0.00	0.00	6,8E-05
0.99	0.00	0.00	0.99	0.00	0.00	6,7E-05
0.99	0.00	0.00	0.99	0.00	0.00	6,7E-05
0.99	0.00	0.00	0.99	0.00	0.00	6,7E-05
0.99	0.00	0.00	0.99	0.00	0.00	6,7E-05
0.99	0.00	0.00	0.99	0.00	0.00	6,7E-05
0.99	0.00	0.00	0.99	0.00	0.00	6,6E-05
0.99	0.00	0.00	0.99	0.00	0.00	6,6E-05
0.99	0.00	0.00	0.99	0.00	0.00	6,6E-05
0.99	0.00	0.00	0.99	0.00	0.00	6,6E-05
0.99	0.00	0.00	0.99	0.00	0.00	6,6E-05
0.99	0.00	0.00	0.99	0.00	0.00	6,6E-05
0.99	0.00	0.00	0.99	0.00	0.00	6,6E-05
0.99	0.00	0.00	0.99	0.00	0.00	6,6E-05
0.99	0.00	0.00	0.99	0.00	0.00	6,6E-05
0.99	0.00	0.00	0.99	0.00	0.00	6,5E-05
0.99	0.00	0.00	0.99	0.00	0.00	6,5E-05
0.99	0.00	0.00	0.99	0.00	0.00	6,5E-05
0.99	0.00	0.00	0.99	0.00	0.00	6,5E-05
0.99	0.00	0.00	0.99	0.00	0.00	6,4E-05
0.99	0.00	0.00	0.99	0.00	0.00	6,4E-05
0.99	0.00	0.00	0.99	0.00	0.00	6,4E-05
0.99	0.00	0.00	0.99	0.00	0.00	6,4E-05
0.99	0.00	0.00	0.99	0.00	0.00	6,3E-05
0.99	0.00	0.00	0.99	0.00	0.00	6,3E-05
0.99	0.00	0.00	0.99	0.00	0.00	6,3E-05
0.99	0.00	0.00	0.99	0.00	0.00	6,3E-05
0.99	0.00	0.00	0.99	0.00	0.00	6,3E-05
0.99	0.00	0.00	0.99	0.00	0.00	6,2E-05
0.99	0.00	0.00	0.99	0.00	0.00	6,2E-05
0.99	0.00	0.00	0.99	0.00	0.00	6,2E-05
0.99	0.00	0.00	0.99	0.00	0.00	6,2E-05
0.99	0.00	0.00	0.99	0.00	0.00	6,2E-05
0.99	0.00	0.00	0.99	0.00	0.00	6,2E-05

chr11	3525603	6	C	0	1.00	0.00	0.00	1.00	0.00	0.00	1.00	0.00	0.00	1.00	0.00	0.00	1.00	0.00	0.00	1.00	0.00	0.00
chr8	89376779	1558	G	0	1.00	0.00	0.00	1.00	0.00	0.00	1.00	0.00	0.00	1.00	0.00	0.00	1.00	0.00	0.00	1.00	0.00	0.00
chr8	120115832	1827	G	0	1.00	0.00	0.00	1.00	0.00	0.00	1.00	0.00	0.00	1.00	0.00	0.00	1.00	0.00	0.00	1.00	0.00	0.00
chr15	100300001	354	G	0	1.00	0.00	0.00	1.00	0.00	0.00	1.00	0.00	0.00	1.00	0.00	0.00	1.00	0.00	0.00	1.00	0.00	0.00
chr4	136547378	1036	C	0	1.00	0.00	0.00	1.00	0.00	0.00	1.00	0.00	0.00	1.00	0.00	0.00	1.00	0.00	0.00	1.00	0.00	0.00
chr4	139783607	1234	g	0	1.00	0.00	0.00	1.00	0.00	0.00	1.00	0.00	0.00	1.00	0.00	0.00	1.00	0.00	0.00	1.00	0.00	0.00
chr9	100498379	1856	C	0	1.00	0.00	0.00	1.00	0.00	0.00	1.00	0.00	0.00	1.00	0.00	0.00	1.00	0.00	0.00	1.00	0.00	0.00
chr4	139783901	1395	G	0	1.00	0.00	0.00	1.00	0.00	0.00	1.00	0.00	0.00	1.00	0.00	0.00	1.00	0.00	0.00	1.00	0.00	0.00
chr4	139783902	1396	G	0	1.00	0.00	0.00	1.00	0.00	0.00	1.00	0.00	0.00	1.00	0.00	0.00	1.00	0.00	0.00	1.00	0.00	0.00
chrX	7476697	2213	C	0	1.00	0.00	0.00	1.00	0.00	0.00	1.00	0.00	0.00	1.00	0.00	0.00	1.00	0.00	0.00	1.00	0.00	0.00
chr11	3525605	7	C	0	1.00	0.00	0.00	1.00	0.00	0.00	1.00	0.00	0.00	1.00	0.00	0.00	1.00	0.00	0.00	1.00	0.00	0.00
chr4	136547327	1021	G	0	1.00	0.00	0.00	1.00	0.00	0.00	1.00	0.00	0.00	1.00	0.00	0.00	1.00	0.00	0.00	1.00	0.00	0.00
chr4	136547600	1164	G	0	1.00	0.00	0.00	1.00	0.00	0.00	1.00	0.00	0.00	1.00	0.00	0.00	1.00	0.00	0.00	1.00	0.00	0.00
chr9	100498389	1862	C	0	1.00	0.00	0.00	1.00	0.00	0.00	1.00	0.00	0.00	1.00	0.00	0.00	1.00	0.00	0.00	1.00	0.00	0.00
chr4	139783885	1387	C	0	1.00	0.00	0.00	1.00	0.00	0.00	1.00	0.00	0.00	1.00	0.00	0.00	1.00	0.00	0.00	1.00	0.00	0.00
chr8	120115831	1826	G	0	1.00	0.00	0.00	1.00	0.00	0.00	1.00	0.00	0.00	1.00	0.00	0.00	1.00	0.00	0.00	1.00	0.00	0.00
chr4	136547598	1163	G	0	1.00	0.00	0.00	1.00	0.00	0.00	1.00	0.00	0.00	1.00	0.00	0.00	1.00	0.00	0.00	1.00	0.00	0.00
chr4	139783900	1394	G	0	1.00	0.00	0.00	1.00	0.00	0.00	1.00	0.00	0.00	1.00	0.00	0.00	1.00	0.00	0.00	1.00	0.00	0.00
chrX	7476459	2047	C	0	1.00	0.00	0.00	1.00	0.00	0.00	1.00	0.00	0.00	1.00	0.00	0.00	1.00	0.00	0.00	1.00	0.00	0.00
chrX	7476676	2201	C	0	1.00	0.00	0.00	1.00	0.00	0.00	1.00	0.00	0.00	1.00	0.00	0.00	1.00	0.00	0.00	1.00	0.00	0.00
chr11	3525923	184	C	0	1.00	0.00	0.00	1.00	0.00	0.00	1.00	0.00	0.00	1.00	0.00	0.00	1.00	0.00	0.00	1.00	0.00	0.00
chr8	89377095	1695	G	0	1.00	0.00	0.00	1.00	0.00	0.00	1.00	0.00	0.00	1.00	0.00	0.00	1.00	0.00	0.00	1.00	0.00	0.00
chrX	7476674	2199	G	0	1.00	0.00	0.00	1.00	0.00	0.00	1.00	0.00	0.00	1.00	0.00	0.00	1.00	0.00	0.00	1.00	0.00	0.00
chr4	136547333	1023	G	0	1.00	0.00	0.00	1.00	0.00	0.00	1.00	0.00	0.00	1.00	0.00	0.00	1.00	0.00	0.00	1.00	0.00	0.00
chr4	136547602	1165	G	0	1.00	0.00	0.00	1.00	0.00	0.00	1.00	0.00	0.00	1.00	0.00	0.00	1.00	0.00	0.00	1.00	0.00	0.00
chr4	139783888	1388	G	0	1.00	0.00	0.00	1.00	0.00	0.00	1.00	0.00	0.00	1.00	0.00	0.00	1.00	0.00	0.00	1.00	0.00	0.00
chr9	100498553	1951	G	0	1.00	0.00	0.00	1.00	0.00	0.00	1.00	0.00	0.00	1.00	0.00	0.00	1.00	0.00	0.00	1.00	0.00	0.00
chr9	100498569	1960	G	0	1.00	0.00	0.00	1.00	0.00	0.00	1.00	0.00	0.00	1.00	0.00	0.00	1.00	0.00	0.00	1.00	0.00	0.00
chrX	7476461	2049	C	0	1.00	0.00	0.00	1.00	0.00	0.00	1.00	0.00	0.00	1.00	0.00	0.00	1.00	0.00	0.00	1.00	0.00	0.00
chr11	3525903	175	C	0	1.00	0.00	0.00	1.00	0.00	0.00	1.00	0.00	0.00	1.00	0.00	0.00	1.00	0.00	0.00	1.00	0.00	0.00
chr4	136547592	1160	G	0	1.00	0.00	0.00	1.00	0.00	0.00	1.00	0.00	0.00	1.00	0.00	0.00	1.00	0.00	0.00	1.00	0.00	0.00
chrX	7476683	2205	G	0	1.00	0.00	0.00	1.00	0.00	0.00	1.00	0.00	0.00	1.00	0.00	0.00	1.00	0.00	0.00	1.00	0.00	0.00
chr9	100498302	1835	G	0	1.00	0.00	0.00	1.00	0.00	0.00	1.00	0.00	0.00	1.00	0.00	0.00	1.00	0.00	0.00	1.00	0.00	0.00
chrX	7476679	2203	C	0	1.00	0.00	0.00	1.00	0.00	0.00	1.00	0.00	0.00	1.00	0.00	0.00	1.00	0.00	0.00	1.00	0.00	0.00
chrX	7476690	2208	C	0	1.00	0.00	0.00	1.00	0.00	0.00	1.00	0.00	0.00	1.00	0.00	0.00	1.00	0.00	0.00	1.00	0.00	0.00
chrX	7476460	2048	C	0	1.00	0.00	0.00	1.00	0.00	0.00	1.00	0.00	0.00	1.00	0.00	0.00	1.00	0.00	0.00	1.00	0.00	0.00
chrX	7476691	2209	C	0	1.00	0.00	0.00	1.00	0.00	0.00	1.00	0.00	0.00	1.00	0.00	0.00	1.00	0.00	0.00	1.00	0.00	0.00
chrX	7476692	2210	C	0	1.00	0.00	0.00	1.00	0.00	0.00	1.00	0.00	0.00	1.00	0.00	0.00	1.00	0.00	0.00	1.00	0.00	0.00
chrX	7476696	2212	C	0	1.00	0.00	0.00	1.00	0.00	0.00	1.00	0.00	0.00	1.00	0.00	0.00	1.00	0.00	0.00	1.00	0.00	0.00
chr11	3525620	16	C	0	1.00	0.00	0.00	1.00	0.00	0.00	1.00	0.00	0.00	1.00	0.00	0.00	1.00	0.00	0.00	1.00	0.00	0.00
chr11	3525902	174	C	0	1.00	0.00	0.00	1.00	0.00	0.00	1.00	0.00	0.00	1.00	0.00	0.00	1.00	0.00	0.00	1.00	0.00	0.00
chr4	139783894	1390	G	0	1.00	0.00	0.00	1.00	0.00	0.00	1.00	0.00	0.00	1.00	0.00	0.00	1.00	0.00	0.00	1.00	0.00	0.00
chr8	89376795	1567	c	0	1.00	0.00	0.00	1.00	0.00	0.00	1.00	0.00	0.00	1.00	0.00	0.00	1.00	0.00	0.00	1.00	0.00	0.00
chrX	7476450	2044	C	0	1.00	0.00	0.00	1.00	0.00	0.00	1.00	0.00	0.00	1.00	0.00	0.00	1.00	0.00	0.00	1.00	0.00	0.00

0.99	0.00	0.00	0.99	0.00	0.00	6,1E-05
0.99	0.00	0.00	0.99	0.00	0.00	6,1E-05
0.99	0.00	0.00	0.99	0.00	0.00	6,1E-05
0.99	0.00	0.00	0.99	0.00	0.00	0,00006
0.99	0.00	0.00	0.99	0.00	0.00	0,00006
0.99	0.00	0.00	0.99	0.00	0.00	0,00006
0.99	0.00	0.00	0.99	0.00	0.00	0,00006
0.99	0.00	0.00	0.99	0.00	0.00	5,9E-05
0.99	0.00	0.00	0.99	0.00	0.00	5,9E-05
0.99	0.00	0.00	0.99	0.00	0.00	5,9E-05
0.99	0.00	0.00	0.99	0.00	0.00	5,8E-05
0.99	0.00	0.00	0.99	0.00	0.00	5,8E-05
0.99	0.00	0.00	0.99	0.00	0.00	5,8E-05
0.99	0.00	0.00	0.99	0.00	0.00	5,8E-05
0.99	0.00	0.00	0.99	0.00	0.00	5,7E-05
0.99	0.00	0.00	0.99	0.00	0.00	5,7E-05
0.99	0.00	0.00	0.99	0.00	0.00	5,6E-05
0.99	0.00	0.00	0.99	0.00	0.00	5,6E-05
0.99	0.00	0.00	0.99	0.00	0.00	5,6E-05
0.99	0.00	0.00	0.99	0.00	0.00	5,6E-05
0.99	0.00	0.00	0.99	0.00	0.00	5,5E-05
0.99	0.00	0.00	0.99	0.00	0.00	5,5E-05
0.99	0.00	0.00	0.99	0.00	0.00	5,5E-05
0.99	0.00	0.00	0.99	0.00	0.00	5,4E-05
0.99	0.00	0.00	0.99	0.00	0.00	5,4E-05
0.99	0.00	0.00	0.99	0.00	0.00	5,4E-05
0.99	0.00	0.00	0.99	0.00	0.00	5,4E-05
0.99	0.00	0.00	1.00	0.00	0.00	5,4E-05
0.99	0.00	0.00	0.99	0.00	0.00	5,4E-05
0.99	0.00	0.00	0.99	0.00	0.00	5,3E-05
0.99	0.00	0.00	0.99	0.00	0.00	5,3E-05
0.99	0.00	0.00	0.99	0.00	0.00	5,3E-05
0.99	0.00	0.00	0.99	0.00	0.00	5,1E-05
0.99	0.00	0.00	0.99	0.00	0.00	5,1E-05
0.99	0.00	0.00	0.99	0.00	0.00	5,1E-05
0.99	0.00	0.00	0.99	0.00	0.00	0,00005
0.99	0.00	0.00	0.99	0.00	0.00	0,00005
0.99	0.00	0.00	0.99	0.00	0.00	4,8E-05
1.00	0.00	0.00	0.99	0.00	0.00	4,8E-05
0.99	0.00	0.00	0.99	0.00	0.00	4,7E-05
0.99	0.00	0.00	0.99	0.00	0.00	4,7E-05
0.99	0.00	0.00	0.99	0.00	0.00	4,5E-05
0.99	0.00	0.00	0.99	0.00	0.00	4,3E-05
0.99	0.00	0.00	0.99	0.00	0.00	4,2E-05

Pearson's *r*	Booth et al. (2012) estimates and glucMS-qPCR	Lux estimates with ideal parameters and glucMS-qPCR	Lux estimates with non-ideal parameters and glucMS-qPCR
5mC	0.86 ($p<1e-4$)	0.87 ($p<1e-4$)	0.86 ($p<1e-4$)
5hmC	0.54 ($p=0.012$)	0.57 ($p=0.007$)	0.63 ($p=0.002$)

Supplemental Table 4

Supplemental Table 5

Window-based analysis

Chromosome	Start	End	Number of cytosines in Cpg Context	Bayes factor	Methylation in v6.5 p("C")	Methylation in v6.5 p("5mC")	Methylation in v6.5 p("5hmC")	Methylation in Tet2kd p("C")	Methylation in Tet2kd p("5mC")	Methylation in Tet2kd p("5hmC")
chr8	120115570	120115670	4	0,007275	4,40E-02	9,30E-01	2,20E-02	3,60E-02	9,50E-01	1,70E-02
chr8	120115620	120115720	0	0,0029	4,40E-02	9,50E-01	7,10E-03	3,00E-02	9,60E-01	8,50E-03
chr8	120115670	120115770	4	0,002204	8,30E-02	9,10E-01	6,00E-03	6,60E-02	9,30E-01	5,40E-03
chr8	120115720	120115820	0	0,006513	1,70E-01	8,20E-01	1,20E-02	1,40E-01	8,60E-01	7,10E-03
chr8	120115770	120115870	4	0,033401	1,40E-01	8,30E-01	3,40E-02	8,90E-02	8,90E-01	2,20E-02
chr16	92644400	92644500	6	0,084238	5,40E-01	4,40E-01	1,70E-02	3,70E-01	6,20E-01	1,40E-02
chr16	92644450	92644550	6	2,397023	5,10E-01	4,70E-01	2,50E-02	3,50E-01	6,40E-01	9,90E-03
chr16	92644500	92644600	3	6,597622	5,30E-01	4,30E-01	4,40E-02	3,60E-01	6,30E-01	1,40E-02
chr16	92644550	92644650	4	0,178777	6,80E-01	2,80E-01	3,50E-02	4,80E-01	4,90E-01	3,10E-02
chr15	100299971	100300071	0	0,012638	9,60E-01	3,20E-02	5,90E-03	8,90E-01	9,50E-02	1,70E-02
chr4	136547384	136547484	6	0,00106	2,00E-02	9,70E-01	5,40E-03	2,60E-02	9,70E-01	4,40E-03
chr4	136547434	136547534	2	0,000489	2,50E-02	9,70E-01	2,60E-03	2,50E-02	9,70E-01	2,10E-03
chr4	136547484	136547584	8	0,000299	1,10E-02	9,90E-01	1,90E-03	1,90E-02	9,80E-01	1,50E-03
chr4	136547534	136547634	2	0,000379	8,50E-03	9,90E-01	2,60E-03	1,50E-02	9,80E-01	3,10E-03
chr15	100300021	100300121	4	0,013158	9,30E-01	6,50E-02	5,70E-03	8,60E-01	1,20E-01	2,20E-02
chrX	7539347	7539447	6	0,005784	4,60E-02	9,40E-01	1,50E-02	3,40E-02	9,50E-01	1,40E-02
chrX	7539397	7539497	2	0,003301	6,00E-02	9,30E-01	6,20E-03	4,10E-02	9,50E-01	9,20E-03
chrX	7539447	7539547	8	0,00563	6,80E-02	9,20E-01	7,60E-03	5,20E-02	9,40E-01	1,20E-02
chrX	7539497	7539597	4	0,009308	7,60E-02	9,10E-01	1,40E-02	7,50E-02	9,10E-01	1,40E-02
chr15	100300071	100300171	1	0,534905	6,40E-01	3,50E-01	1,10E-02	4,00E-01	5,30E-01	6,80E-02
chrX	7476284	7476384	5	0,000028	1,00E+00	1,80E-03	1,80E-03	1,00E+00	2,00E-03	2,20E-03
chrX	7476334	7476434	8	0,000007	1,00E+00	9,30E-04	9,80E-04	1,00E+00	9,30E-04	9,10E-04
chrX	7476384	7476484	9	0,000006	1,00E+00	7,90E-04	7,60E-04	1,00E+00	9,80E-04	9,50E-04
chrX	7476434	7476534	6	0,000001	1,00E+00	2,90E-04	3,50E-04	1,00E+00	4,30E-04	3,40E-04
chrX	7476484	7476584	4	0	1,00E+00	1,50E-04	1,50E-04	1,00E+00	2,50E-04	1,80E-04
chrX	7476534	7476634	0	0,000048	1,00E+00	8,60E-05	7,80E-05	9,60E-01	2,00E-02	2,00E-02
chrX	7476584	7476684	6	0	1,00E+00	1,00E-04	9,20E-05	1,00E+00	1,50E-04	1,30E-04
chrX	7476634	7476734	0	0,000002	1,00E+00	4,80E-04	5,00E-04	1,00E+00	6,30E-04	6,40E-04
chr15	100300121	100300221	1	11,296367	6,50E-01	3,40E-01	1,80E-02	3,40E-01	6,20E-01	4,20E-02
chr15	100300171	100300271	7	1,248879	7,80E-01	2,10E-01	1,70E-02	4,40E-01	5,30E-01	3,00E-02
chr15	100300221	100300321	7	0,949476	5,30E-01	4,50E-01	2,10E-02	2,20E-01	7,20E-01	6,10E-02
chr15	100300271	100300371	8	0,626458	3,00E-01	6,70E-01	2,70E-02	7,10E-02	8,90E-01	3,60E-02
chr15	100300321	100300421	4	0,138129	2,00E-01	7,30E-01	6,40E-02	7,00E-02	8,90E-01	4,50E-02
chr15	61868640	61868740	8	8,57753	4,10E-01	5,20E-01	7,40E-02	1,80E-01	8,20E-01	8,70E-03
chr15	61868690	61868790	2	2211695693	3,50E-01	5,50E-01	9,50E-02	1,70E-01	8,20E-01	7,60E-03
chr15	61868740	61868840	4	1,42447E+16	3,50E-01	5,80E-01	6,50E-02	1,60E-01	8,30E-01	5,00E-03
chr15	61868790	61868890	1	16,722538	4,10E-01	5,40E-01	5,10E-02	1,90E-01	8,00E-01	5,60E-03

chr11	3525584	3525684	8	0,446713	4,50E-01	5,40E-01	9,60E-03	2,40E-01	7,50E-01	9,00E-03
chr11	3525634	3525734	0	0,897018	4,50E-01	5,40E-01	7,50E-03	2,50E-01	7,40E-01	5,80E-03
chr11	3525684	3525784	8	0,490645	4,30E-01	5,60E-01	6,00E-03	2,40E-01	7,50E-01	4,30E-03
chr11	3525734	3525834	2	2,773189	4,20E-01	5,80E-01	3,50E-03	2,20E-01	7,80E-01	3,10E-03
chr11	3525784	3525884	8	3,3084E+175	3,90E-01	6,10E-01	2,80E-03	2,00E-01	8,00E-01	2,00E-03
chr11	3525834	3525934	8	9,8091E+271	4,70E-01	5,20E-01	3,00E-03	2,40E-01	7,60E-01	2,50E-03
chr11	3525884	3525984	6	0,924184	6,50E-01	3,40E-01	4,60E-03	3,40E-01	6,50E-01	1,10E-02
chr9	100498351	100498451	7	0,051775	3,10E-01	6,50E-01	3,80E-02	2,40E-01	7,30E-01	2,70E-02
chr9	100498401	100498501	8	0,037847	2,50E-01	7,10E-01	4,20E-02	2,10E-01	7,70E-01	2,10E-02
chr9	100498451	100498551	7	0,087052	2,80E-01	6,40E-01	7,40E-02	2,50E-01	7,20E-01	3,70E-02
chr9	100498501	100498601	6	0,069437	2,90E-01	6,50E-01	5,80E-02	2,40E-01	7,40E-01	1,90E-02
chr4	139783712	139783812	6	12,304372	5,20E-01	4,40E-01	4,40E-02	2,20E-01	7,70E-01	6,90E-03
chr4	139783762	139783862	5	211,007453	8,60E-01	1,20E-01	2,50E-02	3,70E-01	6,10E-01	1,70E-02
chr4	139783812	139783912	6	26046585,42	8,70E-01	9,80E-02	2,90E-02	3,80E-01	6,10E-01	1,30E-02
chr4	139783612	139783712	6	0,374594	3,00E-01	6,50E-01	4,60E-02	1,30E-01	8,70E-01	6,60E-03
chr4	139783662	139783762	0	1,98921E+18	4,20E-01	5,20E-01	5,70E-02	1,80E-01	8,20E-01	3,80E-03
chr5	64987313	64987413	4	0,099405	7,90E-01	1,90E-01	2,20E-02	6,10E-01	3,50E-01	4,00E-02
chr5	64987363	64987463	8	0,095645	4,10E-01	5,70E-01	1,50E-02	2,60E-01	7,20E-01	1,10E-02
chr5	64987413	64987513	8	0,104863	2,80E-01	6,90E-01	3,00E-02	1,90E-01	8,00E-01	1,40E-02
chr5	64987463	64987563	8	0,01198	2,40E-01	7,50E-01	1,50E-02	2,10E-01	7,80E-01	1,20E-02
chr5	64987513	64987613	4	0,02884	3,20E-01	6,60E-01	2,40E-02	2,80E-01	7,10E-01	1,60E-02
chr19	24277451	24277551	2	0,003024	1,10E-01	8,80E-01	9,90E-03	1,00E-01	8,90E-01	3,70E-03
chr19	24277501	24277601	0	0,002537	8,00E-02	9,10E-01	1,30E-02	8,40E-02	9,10E-01	1,10E-03
chr19	24277551	24277651	1	0,001568	6,20E-02	9,30E-01	8,90E-03	6,60E-02	9,30E-01	2,10E-03
chr19	24277651	24277751	4	0,003561	3,40E-02	9,60E-01	1,10E-02	4,30E-02	9,50E-01	8,40E-03
chr8	89376795	89376895	8	0,025617	1,70E-01	8,10E-01	2,00E-02	8,70E-02	9,00E-01	1,20E-02
chr8	89376845	89376945	0	0,012902	1,20E-01	8,60E-01	1,60E-02	6,70E-02	9,30E-01	4,90E-03
chr8	89376895	89376995	6	0,013117	1,60E-01	8,30E-01	1,30E-02	9,00E-02	9,00E-01	5,80E-03
chr17	45706451	45706551	6	0,07641	3,40E-01	6,30E-01	2,50E-02	1,90E-01	7,90E-01	1,90E-02
chr17	45706501	45706601	2	0,582026	5,10E-01	4,70E-01	1,80E-02	2,80E-01	7,10E-01	1,60E-02
chr17	45706551	45706651	0	3,640936	5,20E-01	4,40E-01	3,40E-02	2,90E-01	6,90E-01	2,50E-02
chr17	45706601	45706701	7	0,198384	3,90E-01	5,90E-01	2,40E-02	2,10E-01	7,60E-01	3,60E-02
chr17	45706651	45706751	0	0,458075	4,40E-01	5,50E-01	1,70E-02	2,30E-01	7,50E-01	2,50E-02
chr17	45706701	45706801	6	0,228145	4,80E-01	5,00E-01	2,00E-02	2,50E-01	7,20E-01	2,40E-02

Supplemental Table 6

DP controls

Number of C's and T's

			dpbs1		dpoxbs1		dpbs2		dpoxbs2		dpbs3		dpoxbs3	
**Chromosome**	**Position**		**C to C**	**C to T**	**C to C**	**C to T**	**C to C**	**C to T**	**C to C**	**C to T**	**C to C**	**C to T**	**C to C**	**C to T**
Lambda_ctrl	22924	C	3	2377	0	588	2	1490	0	646	1	1485	0	138
Lambda_ctrl	22928	C	0	2370	1	583	2	1484	0	644	0	1481	0	138
Lambda_ctrl	22933	C	0	2383	0	588	0	1496	0	646	0	1487	0	138
Lambda_ctrl	22938	C	0	2382	0	589	1	1497	1	645	1	1485	0	137
Lambda_ctrl	22939	C	2	2376	0	586	0	1496	0	647	1	1483	0	136
Lambda_ctrl	22944	C	1	2377	0	586	2	1492	0	646	0	1486	0	137
Lambda_ctrl	22946	C	1	2385	0	589	0	1503	0	648	0	1491	0	137
Lambda_ctrl	22953	C	1	2385	0	589	1	1506	0	648	2	1493	0	134
Lambda_ctrl	22954	C	0	2385	0	589	1	1506	0	648	0	1495	0	134
Lambda_ctrl	22963	C	3	2383	0	589	0	1512	0	658	0	1503	1	133
Lambda_ctrl	22964	C	1	2386	0	589	0	1514	0	659	0	1504	0	133
Lambda_ctrl	22970	C	0	2379	0	591	4	1510	2	657	2	1502	0	131
Lambda_ctrl	22985	C	0	2387	0	591	1	1514	1	659	1	1508	0	128
Lambda_ctrl	22987	C	0	2383	0	590	0	1516	0	660	0	1507	0	127
Lambda_ctrl	22991	C	2	2386	0	591	0	1514	0	658	1	1503	0	126
Lambda_ctrl	22994	C	3	2381	0	588	0	1508	0	659	2	1491	0	122
Lambda_ctrl	22997	C	2	2383	1	590	0	1509	0	659	2	1497	1	118
Lambda_ctrl	23002	C	1	2384	0	591	1	1513	0	655	1	1499	0	111
Lambda_ctrl	23004	C	3	2384	1	589	2	1511	0	652	0	1499	0	110
Lambda_ctrl	23005	C	1	2386	0	591	0	1512	0	653	0	1497	0	109
Lambda_ctrl	23021	C	0	2385	0	589	0	1511	0	652	1	1496	0	105
Lambda_ctrl	23024	C	4	2379	0	589	2	1508	2	651	0	1492	0	106
Lambda_ctrl	23030	C	1	2376	1	587	0	1506	0	645	2	1482	0	105
Lambda_ctrl	23033	C	1	2381	1	586	0	1506	1	645	1	1480	0	104
Lambda_ctrl	23054	C	1	2374	1	582	1	1495	0	638	0	1469	0	103
Lambda_ctrl	23075	C	0	2341	0	576	0	1459	0	624	1	1439	0	99
Lambda_ctrl	23789	C	293	2613	4	665	217	3093	5	921	235	3098	1	176
Lambda_ctrl	23794	C	163	2751	3	669	154	3176	3	930	164	3194	0	178
Lambda_ctrl	23798	C	201	2722	7	663	227	3106	5	926	228	3131	2	174
Lambda_ctrl	23817	C	215	2716	5	668	205	3139	4	931	225	3154	2	178
Lambda_ctrl	23823	C	199	2736	4	671	192	3157	3	933	229	3153	1	179
Lambda_ctrl	23827	C	221	2694	3	669	181	3159	4	930	195	3172	2	177
Lambda_ctrl	23837	C	210	2729	5	670	226	3131	5	933	254	3141	0	179
Lambda_ctrl	23843	C	217	2724	0	676	213	3148	6	934	229	3173	0	178
Lambda_ctrl	23855	C	228	2714	4	672	224	3138	10	931	221	3191	3	175
Lambda_ctrl	23860	C	255	2659	5	647	282	3040	5	929	273	3100	5	170
Lambda_ctrl	23863	C	247	2678	6	669	273	3082	10	929	248	3152	1	177
Lambda_ctrl	23871	C	218	2692	9	663	244	3095	5	927	278	3107	1	174
Lambda_ctrl	23877	C	217	2722	1	675	214	3145	7	936	220	3184	0	176
Lambda_ctrl	23885	C	95	2846	2	673	82	3276	4	937	90	3309	2	173

Number of C's and total read outs

dpbs1		dpoxbs1		dpbs2		dpoxbs2		dpbs3		dpoxbs3	
C	Total	C	Total	C	Total	C	Total	C	Total	C	Total
3	2380	0	588	2	1492	0	646	1	1486	0	138
0	2370	1	584	2	1486	0	644	0	1481	0	138
0	2383	0	588	0	1496	0	646	0	1487	0	138
0	2382	0	589	1	1498	1	646	1	1486	0	137
2	2378	0	586	0	1496	0	647	1	1484	0	136
1	2378	0	586	2	1494	0	646	0	1486	0	137
1	2386	0	589	0	1503	0	648	0	1491	0	137
1	2386	0	589	1	1507	0	648	2	1495	0	134
0	2385	0	589	1	1507	0	648	0	1495	0	134
3	2386	0	589	0	1512	0	658	0	1503	1	134
1	2387	0	589	0	1514	0	659	0	1504	0	133
0	2379	0	591	4	1514	2	659	2	1504	0	131
0	2387	0	591	1	1515	1	660	1	1509	0	128
0	2383	0	590	0	1516	0	660	0	1507	0	127
2	2388	0	591	0	1514	0	658	1	1504	0	126
3	2384	0	588	0	1508	0	659	2	1493	0	122
2	2385	1	591	0	1509	0	659	2	1499	1	119
1	2385	0	591	1	1514	0	655	1	1500	0	111
3	2387	1	590	2	1513	0	652	0	1499	0	110
1	2387	0	591	0	1512	0	653	0	1497	0	109
0	2385	0	589	0	1511	0	652	1	1497	0	105
4	2383	0	589	2	1510	2	653	0	1492	0	106
1	2377	1	588	0	1506	0	645	2	1484	0	105
1	2382	1	587	0	1506	1	646	1	1481	0	104
1	2375	1	583	1	1496	0	638	0	1469	0	103
0	2341	0	576	0	1459	0	624	1	1440	0	99
293	2906	4	669	217	3310	5	926	235	3333	1	177
163	2914	3	672	154	3330	3	933	164	3358	0	178
201	2923	7	670	227	3333	5	931	228	3359	2	176
215	2931	5	673	205	3344	4	935	225	3379	2	180
199	2935	4	675	192	3349	3	936	229	3382	1	180
221	2915	3	672	181	3340	4	934	195	3367	2	179
210	2939	5	675	226	3357	5	938	254	3395	0	179
217	2941	0	676	213	3361	6	940	229	3402	0	178
228	2942	4	676	224	3362	10	941	221	3412	3	178
255	2914	5	652	282	3322	5	934	273	3373	5	175
247	2925	6	675	273	3355	10	939	248	3400	1	178
218	2910	9	672	244	3339	5	932	278	3385	1	175
217	2939	1	676	214	3359	7	943	220	3404	0	176
95	2941	2	675	82	3358	4	941	90	3399	2	175

Lambda_ctrl	23893	C	149	2696	2	634	153	3081	5	884	159	3118	1	162
Lambda_ctrl	23896	C	135	2806	0	674	148	3205	7	924	134	3252	0	170
Lambda_ctrl	23900	C	135	2799	6	668	131	3220	4	923	142	3240	0	167
Lambda_ctrl	23901	C	119	2805	3	668	130	3218	5	919	139	3230	2	165
Lambda_ctrl	23902	C	186	2738	2	669	204	3136	5	918	220	3154	1	165
Lambda_ctrl	23916	C	225	2701	10	662	258	3074	5	914	293	3071	4	159
Lambda_ctrl	23923	C	136	2784	6	664	130	3198	4	910	152	3200	0	163
Lambda_ctrl	23925	C	89	2823	1	670	86	3239	8	908	108	3229	3	159
Lambda_ctrl	23927	C	171	2745	7	664	166	3160	4	911	209	3133	1	162
Lambda_ctrl	23928	C	215	2703	8	662	238	3086	11	904	277	3066	0	162
Lambda_ctrl	23943	C	317	2585	5	663	332	2977	12	900	355	2974	1	160
Lambda_ctrl	23947	C	192	2713	4	664	219	3093	11	904	212	3124	4	156
Lambda_ctrl	23954	C	108	2785	5	659	101	3195	6	906	134	3187	2	157
Lambda_ctrl	47359	C	27015	146	17322	116	7628	47	14749	119	12485	72	25174	166
Lambda_ctrl	47367	C	27125	314	17410	227	7704	99	14915	178	12603	141	25453	303
Lambda_ctrl	47377	C	26954	409	17326	284	7681	106	14856	223	12580	150	25373	372
Lambda_ctrl	47387	C	27152	339	17480	214	7768	95	15029	179	12706	128	25649	309
Lambda_ctrl	47392	C	27058	431	17423	275	7782	86	15026	194	12699	143	25654	314
Lambda_ctrl	47394	C	27124	361	17437	251	7779	86	15052	172	12693	155	25698	280
Lambda_ctrl	47398	C	27102	410	17439	268	7778	99	15050	189	12686	173	25622	373
Lambda_ctrl	47404	C	27061	453	17431	281	7795	87	15050	196	12739	127	25719	306
Lambda_ctrl	47409	C	27093	395	17440	253	7757	118	14978	263	12682	162	25619	361
Lambda_ctrl	47411	C	27165	352	17443	273	7790	95	15063	193	12714	154	25684	353
Lambda_ctrl	47413	C	27203	314	17481	234	7778	106	15054	204	12691	178	25696	337
Lambda_ctrl	47419	C	27196	316	17430	276	7770	113	15023	237	12661	215	25633	395
Lambda_ctrl	47428	C	27159	356	17455	260	7790	94	15077	185	12737	141	25654	389
Lambda_ctrl	47440	C	27181	335	17497	216	7797	90	15101	163	12741	137	25724	330
Lambda_ctrl	47443	C	27147	365	17463	253	7791	97	15027	234	12707	173	25695	362
Lambda_ctrl	47444	C	27136	378	17441	271	7798	89	15069	195	12734	149	25706	354
Lambda_ctrl	47449	C	27245	263	17535	173	7824	63	15093	167	12779	100	25790	262
Lambda_ctrl	47450	C	27181	336	17503	212	7813	74	15093	171	12762	116	25757	298
Lambda_ctrl	47461	C	27056	364	17404	251	7804	67	15046	188	12686	156	25672	319
Lambda_ctrl	47462	C	27096	401	17433	271	7783	105	15042	220	12706	169	25707	337
Lambda_ctrl	47465	C	27114	394	17433	277	7789	99	15034	233	12709	167	25685	372
Lambda_ctrl	47466	C	27089	411	17461	247	7778	111	15051	215	12709	164	25711	339
Lambda_ctrl	47475	C	27084	409	17439	262	7782	100	15022	232	12686	183	25680	360
Lambda_ctrl	47481	C	27035	450	17401	298	7792	93	15018	235	12717	152	25658	378
Lambda_ctrl	47483	C	27076	415	17452	250	7805	76	15032	215	12706	156	25666	370
Lambda_ctrl	47492	C	26445	428	17071	256	7638	97	14737	202	12476	169	25126	349
Lambda_ctrl	47498	C	27006	454	17421	251	7766	102	14995	226	12654	184	25618	374
Lambda_ctrl	47507	C	27043	416	17412	266	7723	143	14973	250	12640	198	25574	424
Lambda_ctrl	47512	C	27063	348	17403	247	7752	103	14981	222	12643	176	25617	321
Lambda_ctrl	47516	C	27061	385	17417	246	7750	111	14974	240	12654	173	25559	420
Lambda_ctrl	47521	C	27045	387	17392	259	7721	122	14995	214	12614	207	25529	423
Lambda_ctrl	47525	C	27077	320	17391	230	7722	99	14947	234	12578	208	25503	399
Lambda_ctrl	47526	C	27019	349	17352	235	7705	104	14930	216	12587	174	25517	336

149	2845	2	636	153	3234	5	889	159	3277	1	163
135	2941	0	674	148	3353	7	931	134	3386	0	170
135	2934	6	674	131	3351	4	927	142	3382	0	167
119	2924	3	671	130	3348	5	924	139	3369	2	167
186	2924	2	671	204	3340	5	923	220	3374	1	166
225	2926	10	672	258	3332	5	919	293	3364	4	163
136	2920	6	670	130	3328	4	914	152	3352	0	163
89	2912	1	671	86	3325	8	916	108	3337	3	162
171	2916	7	671	166	3326	4	915	209	3342	1	163
215	2918	8	670	238	3324	11	915	277	3343	0	162
317	2902	5	668	332	3309	12	912	355	3329	1	161
192	2905	4	668	219	3312	11	915	212	3336	4	160
108	2893	5	664	101	3296	6	912	134	3321	2	159
27015	27161	17322	17438	7628	7675	14749	14868	12485	12557	25174	25340
27125	27439	17410	17637	7704	7803	14915	15093	12603	12744	25453	25756
26954	27363	17326	17610	7681	7787	14856	15079	12580	12730	25373	25745
27152	27491	17480	17694	7768	7863	15029	15208	12706	12834	25649	25958
27058	27489	17423	17698	7782	7868	15026	15220	12699	12842	25654	25968
27124	27485	17437	17688	7779	7865	15052	15224	12693	12848	25698	25978
27102	27512	17439	17707	7778	7877	15050	15239	12686	12859	25622	25995
27061	27514	17431	17712	7795	7882	15050	15246	12739	12866	25719	26025
27093	27488	17440	17693	7757	7875	14978	15241	12682	12844	25619	25980
27165	27517	17443	17716	7790	7885	15063	15256	12714	12868	25684	26037
27203	27517	17481	17715	7778	7884	15054	15258	12691	12869	25696	26033
27196	27512	17430	17706	7770	7883	15023	15260	12661	12876	25633	26028
27159	27515	17455	17715	7790	7884	15077	15262	12737	12878	25654	26043
27181	27516	17497	17713	7797	7887	15101	15264	12741	12878	25724	26054
27147	27512	17463	17716	7791	7888	15027	15261	12707	12880	25695	26057
27136	27514	17441	17712	7798	7887	15069	15264	12734	12883	25706	26060
27245	27508	17535	17708	7824	7887	15093	15260	12779	12879	25790	26052
27181	27517	17503	17715	7813	7887	15093	15264	12762	12878	25757	26055
27056	27420	17404	17655	7804	7871	15046	15234	12686	12842	25672	25991
27096	27497	17433	17704	7783	7888	15042	15262	12706	12875	25707	26044
27114	27508	17433	17710	7789	7888	15034	15267	12709	12876	25685	26057
27089	27500	17461	17708	7778	7889	15051	15266	12709	12873	25711	26050
27084	27493	17439	17701	7782	7882	15022	15254	12686	12869	25680	26040
27035	27485	17401	17699	7792	7885	15018	15253	12717	12869	25658	26036
27076	27491	17452	17702	7805	7881	15032	15247	12706	12862	25666	26036
26445	26873	17071	17327	7638	7735	14737	14939	12476	12645	25126	25475
27006	27460	17421	17672	7766	7868	14995	15221	12654	12838	25618	25992
27043	27459	17412	17678	7723	7866	14973	15223	12640	12838	25574	25998
27063	27411	17403	17650	7752	7855	14981	15203	12643	12819	25617	25938
27061	27446	17417	17663	7750	7861	14974	15214	12654	12827	25559	25979
27045	27432	17392	17651	7721	7843	14995	15209	12614	12821	25529	25952
27077	27397	17391	17621	7722	7821	14947	15181	12578	12786	25503	25902
27019	27368	17352	17587	7705	7809	14930	15146	12587	12761	25517	25853

Lambda_ctrl	47528	C	26977	403	17322	276	7707	105	14928	238	12561	203	25428	438
Lambda_ctrl	47529	C	26966	372	17339	242	7686	118	14920	228	12560	182	25449	379

26977	27380	17322	17598	7707	7812	14928	15166	12561	12764	25428	25866
26966	27338	17339	17581	7686	7804	14920	15148	12560	12742	25449	25828

CD4 SP controls

Number of C's and T's

			spbs1		spoxbs1		spbs2		spoxbs2		spbs3		spoxbs3	
**Chromosome**	**Position**		**C to C**	**C to T**	**C to C**	**C to T**	**C to C**	**C to T**	**C to C**	**C to T**	**C to C**	**C to T**	**C to C**	**C to T**
Lambda_ctrl	22924	C	4	3136	2	1686	2	3603	2	1693	1	2414	0	1690
Lambda_ctrl	22928	C	1	3130	2	1671	3	3590	2	1682	0	2397	1	1678
Lambda_ctrl	22933	C	1	3139	0	1686	0	3609	0	1695	0	2418	0	1691
Lambda_ctrl	22938	C	0	3145	1	1687	2	3607	1	1697	1	2425	0	1695
Lambda_ctrl	22939	C	1	3133	0	1682	0	3606	2	1693	2	2422	0	1694
Lambda_ctrl	22944	C	1	3138	0	1686	3	3611	0	1693	4	2416	1	1696
Lambda_ctrl	22946	C	0	3144	1	1689	3	3618	0	1703	0	2430	0	1700
Lambda_ctrl	22953	C	0	3145	0	1691	2	3624	0	1705	0	2434	1	1700
Lambda_ctrl	22954	C	1	3143	0	1690	1	3625	0	1706	0	2434	0	1700
Lambda_ctrl	22963	C	1	3140	1	1692	3	3638	0	1709	4	2440	2	1705
Lambda_ctrl	22964	C	1	3147	0	1693	1	3643	0	1713	1	2446	1	1708
Lambda_ctrl	22970	C	1	3146	1	1688	1	3644	0	1707	3	2440	1	1706
Lambda_ctrl	22985	C	2	3142	0	1689	1	3654	0	1712	5	2436	1	1702
Lambda_ctrl	22987	C	1	3146	1	1690	0	3652	0	1711	0	2441	0	1700
Lambda_ctrl	22991	C	0	3147	0	1693	2	3650	0	1711	0	2435	2	1693
Lambda_ctrl	22994	C	4	3130	3	1688	2	3639	5	1701	3	2425	1	1692
Lambda_ctrl	22997	C	3	3137	3	1683	8	3638	1	1704	6	2423	4	1692
Lambda_ctrl	23002	C	2	3145	0	1697	1	3645	1	1703	2	2427	1	1697
Lambda_ctrl	23004	C	0	3147	0	1695	2	3645	0	1704	1	2430	1	1692
Lambda_ctrl	23005	C	2	3146	1	1696	0	3645	0	1705	0	2425	0	1693
Lambda_ctrl	23021	C	3	3143	1	1695	2	3639	1	1704	1	2415	0	1692
Lambda_ctrl	23024	C	3	3143	0	1695	1	3636	3	1697	1	2417	0	1691
Lambda_ctrl	23030	C	0	3144	1	1692	2	3628	0	1697	3	2405	0	1685
Lambda_ctrl	23033	C	2	3144	2	1690	2	3628	2	1696	2	2406	0	1686
Lambda_ctrl	23054	C	0	3133	1	1684	0	3614	1	1692	2	2388	1	1675
Lambda_ctrl	23075	C	0	3086	1	1663	0	3557	0	1659	1	2348	0	1647
Lambda_ctrl	23789	C	271	2586	10	1143	285	3696	6	1295	234	2694	8	1348
Lambda_ctrl	23794	C	164	2699	17	1137	188	3818	21	1289	164	2785	8	1362
Lambda_ctrl	23798	C	232	2631	25	1133	259	3742	17	1294	208	2746	24	1344
Lambda_ctrl	23817	C	213	2664	17	1145	251	3776	13	1301	205	2766	11	1367
Lambda_ctrl	23823	C	206	2669	16	1146	260	3769	8	1305	191	2785	10	1368
Lambda_ctrl	23827	C	212	2660	16	1144	213	3807	9	1299	199	2764	16	1355
Lambda_ctrl	23837	C	210	2671	23	1140	266	3773	16	1299	198	2788	24	1360
Lambda_ctrl	23843	C	227	2658	16	1150	277	3770	12	1301	203	2788	15	1369
Lambda_ctrl	23855	C	239	2648	14	1152	280	3768	15	1301	219	2774	17	1367
Lambda_ctrl	23860	C	267	2585	12	1136	319	3682	20	1278	281	2676	21	1348
Lambda_ctrl	23863	C	266	2609	21	1141	291	3745	13	1298	265	2715	23	1359
Lambda_ctrl	23871	C	238	2623	14	1144	301	3713	11	1292	250	2708	10	1363
Lambda_ctrl	23877	C	224	2658	23	1142	244	3797	27	1288	218	2760	18	1365
Lambda_ctrl	23885	C	116	2768	9	1157	107	3935	10	1301	92	2888	13	1370

Number of C's and total read outs

spbs1		spoxbs1		spbs2		spoxbs2		spbs3		spoxbs3	
**C**	**Total**	**C**	**Total**	**C**	**Total**	**C**	**Total**	**C**	**Total**	**C**	**Total**
4	3140	2	1688	2	3605	2	1695	1	2415	0	1690
1	3131	2	1673	3	3593	2	1684	0	2397	1	1679
1	3140	0	1686	0	3609	0	1695	0	2418	0	1691
0	3145	1	1688	2	3609	1	1698	1	2426	0	1695
1	3134	0	1682	0	3606	2	1695	2	2424	0	1694
1	3139	0	1686	3	3614	0	1693	4	2420	1	1697
0	3144	1	1690	3	3621	0	1703	0	2430	0	1700
0	3145	0	1691	2	3626	0	1705	0	2434	1	1701
1	3144	0	1690	1	3626	0	1706	0	2434	0	1700
1	3141	1	1693	3	3641	0	1709	4	2444	2	1707
1	3148	0	1693	1	3644	0	1713	1	2447	1	1709
1	3147	1	1689	1	3645	0	1707	3	2443	1	1707
2	3144	0	1689	1	3655	0	1712	5	2441	1	1703
1	3147	1	1691	0	3652	0	1711	0	2441	0	1700
0	3147	0	1693	2	3652	0	1711	0	2435	2	1695
4	3134	3	1691	2	3641	5	1706	3	2428	1	1693
3	3140	3	1686	8	3646	1	1705	6	2429	4	1696
2	3147	0	1697	1	3646	1	1704	2	2429	1	1698
0	3147	0	1695	2	3647	0	1704	1	2431	1	1693
2	3148	1	1697	0	3645	0	1705	0	2425	0	1693
3	3146	1	1696	2	3641	1	1705	1	2416	0	1692
3	3146	0	1695	1	3637	3	1700	1	2418	0	1691
0	3144	1	1693	2	3630	0	1697	3	2408	0	1685
2	3146	2	1692	2	3630	2	1698	2	2408	0	1686
0	3133	1	1685	0	3614	1	1693	2	2390	1	1676
0	3086	1	1664	0	3557	0	1659	1	2349	0	1647
271	2857	10	1153	285	3981	6	1301	234	2928	8	1356
164	2863	17	1154	188	4006	21	1310	164	2949	8	1370
232	2863	25	1158	259	4001	17	1311	208	2954	24	1368
213	2877	17	1162	251	4027	13	1314	205	2971	11	1378
206	2875	16	1162	260	4029	8	1313	191	2976	10	1378
212	2872	16	1160	213	4020	9	1308	199	2963	16	1371
210	2881	23	1163	266	4039	16	1315	198	2986	24	1384
227	2885	16	1166	277	4047	12	1313	203	2991	15	1384
239	2887	14	1166	280	4048	15	1316	219	2993	17	1384
267	2852	12	1148	319	4001	20	1298	281	2957	21	1369
266	2875	21	1162	291	4036	13	1311	265	2980	23	1382
238	2861	14	1158	301	4014	11	1303	250	2958	10	1373
224	2882	23	1165	244	4041	27	1315	218	2978	18	1383
116	2884	9	1166	107	4042	10	1311	92	2980	13	1383

Lambda_ctrl	23893	C	149	2620	16	1109	174	3691	14	1253	150	2701	10	1305
Lambda_ctrl	23896	C	157	2721	11	1155	158	3874	20	1291	148	2810	18	1360
Lambda_ctrl	23900	C	127	2750	17	1149	153	3877	17	1292	136	2812	9	1366
Lambda_ctrl	23901	C	122	2749	17	1147	131	3893	8	1299	106	2837	10	1361
Lambda_ctrl	23902	C	214	2657	22	1143	247	3774	18	1288	204	2741	12	1359
Lambda_ctrl	23916	C	262	2601	23	1139	282	3731	22	1286	293	2636	24	1343
Lambda_ctrl	23923	C	135	2721	13	1148	144	3862	6	1299	152	2768	11	1352
Lambda_ctrl	23925	C	98	2755	16	1144	112	3880	10	1293	111	2799	12	1349
Lambda_ctrl	23927	C	180	2680	19	1142	205	3795	18	1286	195	2724	21	1339
Lambda_ctrl	23928	C	214	2646	21	1140	216	3780	23	1277	268	2641	13	1351
Lambda_ctrl	23943	C	311	2541	23	1133	409	3570	22	1271	330	2562	31	1331
Lambda_ctrl	23947	C	190	2656	21	1137	239	3741	18	1278	208	2685	20	1340
Lambda_ctrl	23954	C	104	2732	17	1135	143	3818	12	1274	133	2744	15	1339
Lambda_ctrl	47359	C	32964	191	20959	160	21028	142	12132	88	27345	198	16916	141
Lambda_ctrl	47367	C	33108	409	21124	245	21245	258	12261	147	27647	330	17092	213
Lambda_ctrl	47377	C	32894	525	20991	335	21159	328	12182	205	27519	434	17020	261
Lambda_ctrl	47387	C	33031	541	21167	257	21299	300	12306	161	27751	368	17184	205
Lambda_ctrl	47392	C	32986	592	21096	334	21334	279	12302	175	27722	411	17171	227
Lambda_ctrl	47394	C	33036	519	21128	286	21314	294	12300	171	27728	402	17180	213
Lambda_ctrl	47398	C	32966	624	21107	324	21300	323	12315	168	27718	428	17167	239
Lambda_ctrl	47404	C	32830	756	21116	322	21337	305	12305	184	27749	412	17139	278
Lambda_ctrl	47409	C	33084	467	21074	347	21327	285	12283	186	27745	387	17140	260
Lambda_ctrl	47411	C	33062	540	21131	307	21378	262	12336	153	27839	324	17196	225
Lambda_ctrl	47413	C	33079	521	21121	318	21384	259	12302	182	27832	334	17178	243
Lambda_ctrl	47419	C	33207	388	21124	313	21367	273	12301	186	27814	342	17161	261
Lambda_ctrl	47428	C	33056	548	21098	344	21363	283	12334	155	27799	373	17187	233
Lambda_ctrl	47440	C	33195	413	21195	251	21410	242	12332	157	27826	349	17201	220
Lambda_ctrl	47443	C	33107	500	21149	295	21366	287	12307	185	27789	388	17170	257
Lambda_ctrl	47444	C	33109	494	21095	349	21375	279	12318	170	27792	383	17205	225
Lambda_ctrl	47449	C	33271	325	21206	234	21457	194	12374	108	27919	255	17262	167
Lambda_ctrl	47450	C	33209	393	21176	267	21435	218	12350	140	27842	332	17230	199
Lambda_ctrl	47461	C	32952	543	21084	289	21331	254	12305	146	27763	338	17130	257
Lambda_ctrl	47462	C	33069	507	21121	310	21348	288	12296	193	27738	414	17167	246
Lambda_ctrl	47465	C	33058	532	21095	340	21370	277	12313	168	27743	423	17147	278
Lambda_ctrl	47466	C	33098	479	21109	321	21377	265	12316	166	27816	343	17187	240
Lambda_ctrl	47475	C	33026	555	21119	297	21339	291	12279	198	27741	415	17143	277
Lambda_ctrl	47481	C	32931	627	21060	356	21305	316	12291	184	27714	423	17165	246
Lambda_ctrl	47483	C	33101	469	21138	287	21347	283	12338	144	27765	379	17192	216
Lambda_ctrl	47492	C	32183	574	20640	305	20904	308	12050	187	27160	422	16849	223
Lambda_ctrl	47498	C	33017	508	21074	326	21262	325	12281	182	27639	467	17118	268
Lambda_ctrl	47507	C	32964	558	21083	314	21228	360	12247	216	27660	436	17099	287
Lambda_ctrl	47512	C	32986	482	21089	276	21276	292	12289	157	27658	395	17118	251
Lambda_ctrl	47516	C	32988	521	21061	327	21269	309	12228	227	27669	416	17113	266
Lambda_ctrl	47521	C	33068	425	21074	296	21292	280	12262	191	27680	403	17097	278
Lambda_ctrl	47525	C	33077	370	21065	276	21250	293	12238	191	27680	349	17047	300
Lambda_ctrl	47526	C	32913	477	20996	318	21230	275	12201	192	27574	408	17098	235

149	2769	16	1125	174	3865	14	1267	150	2851	10	1315
157	2878	11	1166	158	4032	20	1311	148	2958	18	1378
127	2877	17	1166	153	4030	17	1309	136	2948	9	1375
122	2871	17	1164	131	4024	8	1307	106	2943	10	1371
214	2871	22	1165	247	4021	18	1306	204	2945	12	1371
262	2863	23	1162	282	4013	22	1308	293	2929	24	1367
135	2856	13	1161	144	4006	6	1305	152	2920	11	1363
98	2853	16	1160	112	3992	10	1303	111	2910	12	1361
180	2860	19	1161	205	4000	18	1304	195	2919	21	1360
214	2860	21	1161	216	3996	23	1300	268	2909	13	1364
311	2852	23	1156	409	3979	22	1293	330	2892	31	1362
190	2846	21	1158	239	3980	18	1296	208	2893	20	1360
104	2836	17	1152	143	3961	12	1286	133	2877	15	1354
32964	33155	20959	21119	21028	21170	12132	12220	27345	27543	16916	17057
33108	33517	21124	21369	21245	21503	12261	12408	27647	27977	17092	17305
32894	33419	20991	21326	21159	21487	12182	12387	27519	27953	17020	17281
33031	33572	21167	21424	21299	21599	12306	12467	27751	28119	17184	17389
32986	33578	21096	21430	21334	21613	12302	12477	27722	28133	17171	17398
33036	33555	21128	21414	21314	21608	12300	12471	27728	28130	17180	17393
32966	33590	21107	21431	21300	21623	12315	12483	27718	28146	17167	17406
32830	33586	21116	21438	21337	21642	12305	12489	27749	28161	17139	17417
33084	33551	21074	21421	21327	21612	12283	12469	27745	28132	17140	17400
33062	33602	21131	21438	21378	21640	12336	12489	27839	28163	17196	17421
33079	33600	21121	21439	21384	21643	12302	12484	27832	28166	17178	17421
33207	33595	21124	21437	21367	21640	12301	12487	27814	28156	17161	17422
33056	33604	21098	21442	21363	21646	12334	12489	27799	28172	17187	17420
33195	33608	21195	21446	21410	21652	12332	12489	27826	28175	17201	17421
33107	33607	21149	21444	21366	21653	12307	12492	27789	28177	17170	17427
33109	33603	21095	21444	21375	21654	12318	12488	27792	28175	17205	17430
33271	33596	21206	21440	21457	21651	12374	12482	27919	28174	17262	17429
33209	33602	21176	21443	21435	21653	12350	12490	27842	28174	17230	17429
32952	33495	21084	21373	21331	21585	12305	12451	27763	28101	17130	17387
33069	33576	21121	21431	21348	21636	12296	12489	27738	28152	17167	17413
33058	33590	21095	21435	21370	21647	12313	12481	27743	28166	17147	17425
33098	33577	21109	21430	21377	21642	12316	12482	27816	28159	17187	17427
33026	33581	21119	21416	21339	21630	12279	12477	27741	28156	17143	17420
32931	33558	21060	21416	21305	21621	12291	12475	27714	28137	17165	17411
33101	33570	21138	21425	21347	21630	12338	12482	27765	28144	17192	17408
32183	32757	20640	20945	20904	21212	12050	12237	27160	27582	16849	17072
33017	33525	21074	21400	21262	21587	12281	12463	27639	28106	17118	17386
32964	33522	21083	21397	21228	21588	12247	12463	27660	28096	17099	17386
32986	33468	21089	21365	21276	21568	12289	12446	27658	28053	17118	17369
32988	33509	21061	21388	21269	21578	12228	12455	27669	28085	17113	17379
33068	33493	21074	21370	21292	21572	12262	12453	27680	28083	17097	17375
33077	33447	21065	21341	21250	21543	12238	12429	27680	28029	17047	17347
32913	33390	20996	21314	21230	21505	12201	12393	27574	27982	17098	17333

Lambda_ctrl	47528	C	32898	544	20947	368	21166	368	12210	214	27588	417	17050	282
Lambda_ctrl	47529	C	32973	439	20987	314	21222	301	12216	191	27601	372	17079	246

32898	33442	20947	21315	21166	21534	12210	12424	27588	28005	17050	17332
32973	33412	20987	21301	21222	21523	12216	12407	27601	27973	17079	17325

CD4 naïve controls

Number of C's and T's

			naivebs1		naiveoxbs1		naivebs2		naiveoxbs2		naivebs3		naiveoxbs3	
**Chromosome**	**Position**		**C to C**	**C to T**	**C to C**	**C to T**	**C to C**	**C to T**	**C to C**	**C to T**	**C to C**	**C to T**	**C to C**	**C to T**
Lambda_ctrl	22924	C	3	4607	0	1576	2	3728	2	2458	2	3909	1	2314
Lambda_ctrl	22928	C	5	4583	3	1569	2	3716	2	2450	4	3891	1	2312
Lambda_ctrl	22933	C	0	4610	0	1577	2	3731	0	2463	1	3917	1	2318
Lambda_ctrl	22938	C	2	4609	2	1575	2	3735	2	2463	2	3922	2	2322
Lambda_ctrl	22939	C	1	4597	0	1573	3	3730	0	2463	1	3919	2	2312
Lambda_ctrl	22944	C	3	4595	0	1571	2	3728	2	2457	1	3920	3	2312
Lambda_ctrl	22946	C	1	4613	0	1577	1	3749	0	2473	0	3938	0	2330
Lambda_ctrl	22953	C	0	4614	1	1577	1	3755	0	2476	2	3938	1	2333
Lambda_ctrl	22954	C	0	4613	0	1578	0	3755	0	2474	0	3937	0	2331
Lambda_ctrl	22963	C	2	4607	1	1579	1	3761	1	2477	4	3950	1	2333
Lambda_ctrl	22964	C	3	4616	0	1580	1	3763	0	2487	1	3957	0	2341
Lambda_ctrl	22970	C	3	4608	2	1578	4	3754	1	2484	4	3958	3	2337
Lambda_ctrl	22985	C	4	4610	0	1579	2	3763	2	2484	5	3963	1	2336
Lambda_ctrl	22987	C	3	4614	0	1581	0	3765	0	2488	0	3971	1	2337
Lambda_ctrl	22991	C	2	4619	0	1580	0	3762	1	2487	2	3969	0	2336
Lambda_ctrl	22994	C	3	4605	3	1569	5	3739	1	2473	2	3949	3	2325
Lambda_ctrl	22997	C	12	4603	3	1574	3	3754	5	2476	1	3964	4	2329
Lambda_ctrl	23002	C	0	4622	2	1578	3	3755	1	2481	0	3965	1	2338
Lambda_ctrl	23004	C	2	4620	2	1577	1	3757	1	2480	2	3960	1	2338
Lambda_ctrl	23005	C	0	4622	1	1578	0	3755	0	2483	1	3962	0	2338
Lambda_ctrl	23021	C	2	4619	0	1572	1	3747	1	2476	3	3956	1	2329
Lambda_ctrl	23024	C	3	4617	0	1572	1	3744	4	2473	2	3954	1	2326
Lambda_ctrl	23030	C	3	4606	2	1567	1	3734	2	2465	4	3935	3	2319
Lambda_ctrl	23033	C	0	4615	2	1567	0	3733	0	2464	1	3941	0	2323
Lambda_ctrl	23054	C	2	4605	0	1565	4	3708	2	2453	1	3922	0	2308
Lambda_ctrl	23075	C	3	4530	1	1540	0	3633	1	2410	1	3833	0	2279
Lambda_ctrl	23789	C	325	3284	24	1731	252	3496	29	3147	332	3742	26	2962
Lambda_ctrl	23794	C	174	3444	22	1750	148	3622	33	3169	189	3916	29	2985
Lambda_ctrl	23798	C	242	3379	29	1742	272	3490	51	3148	280	3830	41	2974
Lambda_ctrl	23817	C	240	3392	18	1762	213	3581	33	3184	226	3907	34	2998
Lambda_ctrl	23823	C	233	3401	17	1765	189	3609	27	3192	247	3891	29	3009
Lambda_ctrl	23827	C	203	3414	21	1745	209	3580	39	3160	225	3898	26	3002
Lambda_ctrl	23837	C	256	3378	28	1760	233	3580	40	3189	231	3920	39	3007
Lambda_ctrl	23843	C	289	3346	10	1780	262	3558	19	3216	271	3885	28	3020
Lambda_ctrl	23855	C	278	3360	23	1766	256	3564	41	3196	288	3874	27	3020
Lambda_ctrl	23860	C	312	3290	27	1738	300	3471	35	3160	322	3797	42	2970
Lambda_ctrl	23863	C	307	3319	25	1756	304	3506	36	3191	320	3837	51	2975
Lambda_ctrl	23871	C	309	3299	23	1755	250	3543	39	3171	300	3842	32	2984
Lambda_ctrl	23877	C	271	3363	25	1761	222	3596	48	3189	236	3929	36	3006
Lambda_ctrl	23885	C	105	3532	14	1770	89	3733	12	3225	102	4063	22	3023

Number of C's and total read outs

naivebs1		naiveoxbs1		naivebs2		naiveoxbs2		naivebs3		naiveoxbs3	
C	Total	C	Total	C	Total	C	Total	C	Total	C	Total
3	4610	0	1576	2	3730	2	2460	2	3911	1	2315
5	4588	3	1572	2	3718	2	2452	4	3895	1	2313
0	4610	0	1577	2	3733	0	2463	1	3918	1	2319
2	4611	2	1577	2	3737	2	2465	2	3924	2	2324
1	4598	0	1573	3	3733	0	2463	1	3920	2	2314
3	4598	0	1571	2	3730	2	2459	1	3921	3	2315
1	4614	0	1577	1	3750	0	2473	0	3938	0	2330
0	4614	1	1578	1	3756	0	2476	2	3940	1	2334
0	4613	0	1578	0	3755	0	2474	0	3937	0	2331
2	4609	1	1580	1	3762	1	2478	4	3954	1	2334
3	4619	0	1580	1	3764	0	2487	1	3958	0	2341
3	4611	2	1580	4	3758	1	2485	4	3962	3	2340
4	4614	0	1579	2	3765	2	2486	5	3968	1	2337
3	4617	0	1581	0	3765	0	2488	0	3971	1	2338
2	4621	0	1580	0	3762	1	2488	2	3971	0	2336
3	4608	3	1572	5	3744	1	2474	2	3951	3	2328
12	4615	3	1577	3	3757	5	2481	1	3965	4	2333
0	4622	2	1580	3	3758	1	2482	0	3965	1	2339
2	4622	2	1579	1	3758	1	2481	2	3962	1	2339
0	4622	1	1579	0	3755	0	2483	1	3963	0	2338
2	4621	0	1572	1	3748	1	2477	3	3959	1	2330
3	4620	0	1572	1	3745	4	2477	2	3956	1	2327
3	4609	2	1569	1	3735	2	2467	4	3939	3	2322
0	4615	2	1569	0	3733	0	2464	1	3942	0	2323
2	4607	0	1565	4	3712	2	2455	1	3923	0	2308
3	4533	1	1541	0	3633	1	2411	1	3834	0	2279
325	3609	24	1755	252	3748	29	3176	332	4074	26	2988
174	3618	22	1772	148	3770	33	3202	189	4105	29	3014
242	3621	29	1771	272	3762	51	3199	280	4110	41	3015
240	3632	18	1780	213	3794	33	3217	226	4133	34	3032
233	3634	17	1782	189	3798	27	3219	247	4138	29	3038
203	3617	21	1766	209	3789	39	3199	225	4123	26	3028
256	3634	28	1788	233	3813	40	3229	231	4151	39	3046
289	3635	10	1790	262	3820	19	3235	271	4156	28	3048
278	3638	23	1789	256	3820	41	3237	288	4162	27	3047
312	3602	27	1765	300	3771	35	3195	322	4119	42	3012
307	3626	25	1781	304	3810	36	3227	320	4157	51	3026
309	3608	23	1778	250	3793	39	3210	300	4142	32	3016
271	3634	25	1786	222	3818	48	3237	236	4165	36	3042
105	3637	14	1784	89	3822	12	3237	102	4165	22	3045

Lambda_ctrl	23893	C	170	3337	18	1694	118	3539	23	3080	175	3840	25	2875
Lambda_ctrl	23896	C	132	3505	22	1764	133	3677	31	3205	158	3994	29	3008
Lambda_ctrl	23900	C	137	3499	19	1767	145	3659	32	3198	140	4007	32	3002
Lambda_ctrl	23901	C	147	3482	23	1761	128	3667	17	3206	131	4002	31	2997
Lambda_ctrl	23902	C	232	3399	29	1752	232	3567	36	3178	255	3884	29	2995
Lambda_ctrl	23916	C	331	3298	27	1753	280	3514	41	3179	298	3826	29	2990
Lambda_ctrl	23923	C	174	3447	27	1749	126	3655	20	3188	148	3963	18	2992
Lambda_ctrl	23925	C	132	3489	16	1758	125	3653	23	3183	115	3982	23	2985
Lambda_ctrl	23927	C	209	3411	23	1755	197	3574	32	3177	215	3897	32	2977
Lambda_ctrl	23928	C	245	3378	34	1743	261	3519	43	3165	277	3833	43	2970
Lambda_ctrl	23943	C	396	3207	28	1743	353	3407	50	3153	419	3670	55	2945
Lambda_ctrl	23947	C	245	3359	30	1738	233	3523	45	3154	264	3824	38	2960
Lambda_ctrl	23954	C	147	3431	19	1741	101	3637	29	3157	144	3917	30	2952
Lambda_ctrl	47359	C	21463	124	28897	192	9833	69	18454	127	11527	76	20256	126
Lambda_ctrl	47367	C	21565	276	29073	397	9933	122	18625	243	11648	129	20383	275
Lambda_ctrl	47377	C	21470	322	28904	486	9890	152	18584	260	11608	140	20324	311
Lambda_ctrl	47387	C	21644	247	29076	439	9978	120	18758	226	11716	122	20537	250
Lambda_ctrl	47392	C	21596	298	29042	469	10001	109	18726	268	11719	134	20471	313
Lambda_ctrl	47394	C	21555	328	29004	473	9996	113	18717	262	11713	142	20528	257
Lambda_ctrl	47398	C	21574	326	29017	508	9995	125	18721	279	11696	163	20484	322
Lambda_ctrl	47404	C	21590	313	28995	532	10014	112	18757	258	11734	135	20504	311
Lambda_ctrl	47409	C	21557	321	29066	445	9956	157	18701	293	11687	175	20484	314
Lambda_ctrl	47411	C	21623	280	29099	429	10018	114	18788	228	11731	145	20564	252
Lambda_ctrl	47413	C	21606	293	29074	453	10017	114	18785	236	11756	124	20548	273
Lambda_ctrl	47419	C	21620	280	29086	441	9998	137	18718	299	11700	178	20479	344
Lambda_ctrl	47428	C	21599	296	29079	450	10020	113	18757	264	11769	110	20510	313
Lambda_ctrl	47440	C	21632	271	29167	367	10006	132	18809	219	11752	131	20551	273
Lambda_ctrl	47443	C	21632	273	29077	450	9995	143	18761	274	11738	146	20548	282
Lambda_ctrl	47444	C	21610	293	29083	445	10006	130	18788	240	11727	152	20547	283
Lambda_ctrl	47449	C	21678	223	29206	312	10050	87	18855	180	11789	96	20649	189
Lambda_ctrl	47450	C	21644	258	29135	397	10010	125	18826	210	11752	133	20598	237
Lambda_ctrl	47461	C	21512	319	28980	441	9965	138	18757	241	11708	145	20540	254
Lambda_ctrl	47462	C	21563	325	29038	470	9999	135	18781	241	11728	160	20536	295
Lambda_ctrl	47465	C	21592	306	29039	477	9973	163	18729	300	11732	155	20530	307
Lambda_ctrl	47466	C	21607	287	29042	471	10011	125	18792	235	11740	146	20538	294
Lambda_ctrl	47475	C	21585	307	29006	487	9986	140	18732	290	11729	155	20536	290
Lambda_ctrl	47481	C	21569	318	29046	452	9998	129	18750	275	11728	157	20527	303
Lambda_ctrl	47483	C	21579	311	29067	428	10005	128	18805	216	11748	135	20582	250
Lambda_ctrl	47492	C	21117	312	28437	429	9861	110	18412	261	11550	152	20165	274
Lambda_ctrl	47498	C	21542	321	29011	451	9960	146	18739	254	11701	158	20490	308
Lambda_ctrl	47507	C	21545	318	28994	465	9963	151	18713	283	11669	199	20478	321
Lambda_ctrl	47512	C	21545	292	29040	393	9978	122	18714	244	11683	158	20477	285
Lambda_ctrl	47516	C	21569	294	28997	459	9941	174	18700	281	11643	212	20430	351
Lambda_ctrl	47521	C	21561	294	29056	392	9946	151	18642	316	11669	178	20460	311
Lambda_ctrl	47525	C	21501	318	29029	383	9920	148	18640	279	11618	185	20425	287
Lambda_ctrl	47526	C	21425	339	28929	433	9935	114	18641	245	11620	158	20372	304

170	3507	18	1712	118	3657	23	3103	175	4015	25	2900
132	3637	22	1786	133	3810	31	3236	158	4152	29	3037
137	3636	19	1786	145	3804	32	3230	140	4147	32	3034
147	3629	23	1784	128	3795	17	3223	131	4133	31	3028
232	3631	29	1781	232	3799	36	3214	255	4139	29	3024
331	3629	27	1780	280	3794	41	3220	298	4124	29	3019
174	3621	27	1776	126	3781	20	3208	148	4111	18	3010
132	3621	16	1774	125	3778	23	3206	115	4097	23	3008
209	3620	23	1778	197	3771	32	3209	215	4112	32	3009
245	3623	34	1777	261	3780	43	3208	277	4110	43	3013
396	3603	28	1771	353	3760	50	3203	419	4089	55	3000
245	3604	30	1768	233	3756	45	3199	264	4088	38	2998
147	3578	19	1760	101	3738	29	3186	144	4061	30	2982
21463	21587	28897	29089	9833	9902	18454	18581	11527	11603	20256	20382
21565	21841	29073	29470	9933	10055	18625	18868	11648	11777	20383	20658
21470	21792	28904	29390	9890	10042	18584	18844	11608	11748	20324	20635
21644	21891	29076	29515	9978	10098	18758	18984	11716	11838	20537	20787
21596	21894	29042	29511	10001	10110	18726	18994	11719	11853	20471	20784
21555	21883	29004	29477	9996	10109	18717	18979	11713	11855	20528	20785
21574	21900	29017	29525	9995	10120	18721	19000	11696	11859	20484	20806
21590	21903	28995	29527	10014	10126	18757	19015	11734	11869	20504	20815
21557	21878	29066	29511	9956	10113	18701	18994	11687	11862	20484	20798
21623	21903	29099	29528	10018	10132	18788	19016	11731	11876	20564	20816
21606	21899	29074	29527	10017	10131	18785	19021	11756	11880	20548	20821
21620	21900	29086	29527	9998	10135	18718	19017	11700	11878	20479	20823
21599	21895	29079	29529	10020	10133	18757	19021	11769	11879	20510	20823
21632	21903	29167	29534	10006	10138	18809	19028	11752	11883	20551	20824
21632	21905	29077	29527	9995	10138	18761	19035	11738	11884	20548	20830
21610	21903	29083	29528	10006	10136	18788	19028	11727	11879	20547	20830
21678	21901	29206	29518	10050	10137	18855	19035	11789	11885	20649	20838
21644	21902	29135	29532	10010	10135	18826	19036	11752	11885	20598	20835
21512	21831	28980	29421	9965	10103	18757	18998	11708	11853	20540	20794
21563	21888	29038	29508	9999	10134	18781	19022	11728	11888	20536	20831
21592	21898	29039	29516	9973	10136	18729	19029	11732	11887	20530	20837
21607	21894	29042	29513	10011	10136	18792	19027	11740	11886	20538	20832
21585	21892	29006	29493	9986	10126	18732	19022	11729	11884	20536	20826
21569	21887	29046	29498	9998	10127	18750	19025	11728	11885	20527	20830
21579	21890	29067	29495	10005	10133	18805	19021	11748	11883	20582	20832
21117	21429	28437	28866	9861	9971	18412	18673	11550	11702	20165	20439
21542	21863	29011	29462	9960	10106	18739	18993	11701	11859	20490	20798
21545	21863	28994	29459	9963	10114	18713	18996	11669	11868	20478	20799
21545	21837	29040	29433	9978	10100	18714	18958	11683	11841	20477	20762
21569	21863	28997	29456	9941	10115	18700	18981	11643	11855	20430	20781
21561	21855	29056	29448	9946	10097	18642	18958	11669	11847	20460	20771
21501	21819	29029	29412	9920	10068	18640	18919	11618	11803	20425	20712
21425	21764	28929	29362	9935	10049	18641	18886	11620	11778	20372	20676

Lambda_ctrl	47528	C	21487	302	28929	481	9892	159	18596	313	11607	181	20366	332
Lambda_ctrl	47529	C	21456	292	28953	429	9893	145	18568	305	11608	164	20331	315

21487	21789	28929	29410	9892	10051	18596	18909	11607	11788	20366	20698
21456	21748	28953	29382	9893	10038	18568	18873	11608	11772	20331	20646

DP wild type cytosines

Number of C's and T's

			dpbs1		dpoxbs1		dpbs2		dpoxbs2		dpbs3		dpoxbs3	
**Chromosome**	**Position**		**C to C**	**C to T**	**C to C**	**C to T**	**C to C**	**C to T**	**C to C**	**C to T**	**C to C**	**C to T**	**C to C**	**C to T**
chr2	11124412	0	9	12502	2	6157	5	7824	4	9983	6	11841	6	11672
chr2	11124413	0	1	12561	1	6172	3	7862	1	10030	2	11910	2	11734
chr2	11124414	0	1	12561	2	6174	0	7870	1	10036	3	11907	1	11738
chr2	11124418	0	6	12493	4	6160	5	7853	4	9997	9	11870	5	11699
chr2	11124422	0	2	12568	0	6182	0	7895	1	10058	1	11935	2	11751
chr2	11124423	0	1	12573	3	6181	1	7892	3	10053	1	11934	0	11757
chr2	11124429	0	29	12558	4	6186	18	7884	5	10068	25	11922	13	11754
chr2	11124431	0	3	12592	1	6190	2	7903	5	10068	4	11944	9	11766
chr2	11124435	0	12	12588	9	6183	13	7893	18	10059	15	11938	9	11777
chr2	11124442	0	9	12526	5	6160	3	7879	5	10060	10	11882	16	11735
chr2	11124443	0	4	12580	1	6196	6	7901	10	10070	14	11934	7	11783
chr2	11124447	0	49	12279	23	6053	26	7746	42	9872	40	11680	30	11536
chr2	11124451	0	19	12547	10	6183	20	7878	14	10082	30	11898	24	11756
chr2	11124454	0	3	12610	2	6206	2	7932	4	10133	8	11982	5	11828
chr2	11124458	0	10	12606	1	6210	3	7931	3	10138	6	11992	13	11829
chr2	11124460	0	4	12614	26	6185	4	7938	60	10086	8	11993	62	11787
chr2	11124463	1	12139	483	5481	735	7596	347	8783	1372	11500	509	10374	1483
chr2	11124467	0	21	12533	10	6179	11	7885	12	10088	20	11924	9	11787
chr2	11124468	0	7	12608	6	6210	10	7914	15	10132	15	11963	17	11819
chr2	11124469	0	39	12593	10	6214	16	7933	25	10137	40	11972	38	11824
chr2	11124470	0	14	12612	4	6218	8	7939	13	10149	11	11998	16	11847
chr2	11124471	0	26	12602	3	6221	15	7931	13	10153	22	11990	13	11853
chr2	11124474	0	33	12566	7	6196	19	7904	25	10125	29	11956	22	11812
chr2	11124479	0	0	18	0	38	0	37	0	155	1	40	0	329
chr2	11124480	0	34	12571	11	6189	17	7914	20	10090	39	11951	25	11803
chr2	11124481	0	9	12629	11	6212	7	7942	17	10154	15	12005	10	11864
chr2	11124483	0	10	12627	4	6219	6	7944	9	10166	10	12009	6	11867
chr2	11124484	0	15	12622	30	6194	12	7933	32	10138	14	12005	43	11830
chr2	11124485	0	32	12604	0	6221	29	7921	6	10167	45	11968	9	11859
chr2	11124488	0	0	25	0	47	0	42	0	176	0	49	5	365
chr2	11124489	0	18	12597	12	6199	3	7930	15	10150	16	11990	23	11844
chr2	11124491	0	16	12618	4	6216	5	7944	9	10166	10	12016	14	11867
chr2	11124492	0	25	12609	4	6218	16	7933	9	10168	28	11995	5	11879
chr2	11124494	0	0	27	0	51	0	47	0	191	0	57	0	400
chr2	11124495	0	0	25	0	46	2	40	2	184	1	52	3	371
chr2	11124496	0	101	12388	28	6140	52	7831	65	10012	74	11820	51	11706
chr2	11124498	1	10279	2323	4383	1825	6588	1341	7167	2981	9906	2088	8752	3097
chr2	11124499	1	19	8	14	37	39	9	44	146	41	17	93	311
chr2	11124502	0	1	24	1	47	0	43	1	173	1	53	0	371
chr2	11124503	0	27	12589	11	6207	28	7909	17	10145	30	11981	29	11839

Number of C's and total read outs

dpbs1		dpoxbs1		dpbs2		dpoxbs2		dpbs3		dpoxbs3	
C	Total	C	Total	C	Total	C	Total	C	Total	C	Total
9	12511	2	6159	5	7829	4	9987	6	11847	6	11678
1	12562	1	6173	3	7865	1	10031	2	11912	2	11736
1	12562	2	6176	0	7870	1	10037	3	11910	1	11739
6	12499	4	6164	5	7858	4	10001	9	11879	5	11704
2	12570	0	6182	0	7895	1	10059	1	11936	2	11753
1	12574	3	6184	1	7893	3	10056	1	11935	0	11757
29	12587	4	6190	18	7902	5	10073	25	11947	13	11767
3	12595	1	6191	2	7905	5	10073	4	11948	9	11775
12	12600	9	6192	13	7906	18	10077	15	11953	9	11786
9	12535	5	6165	3	7882	5	10065	10	11892	16	11751
4	12584	1	6197	6	7907	10	10080	14	11948	7	11790
49	12328	23	6076	26	7772	42	9914	40	11720	30	11566
19	12566	10	6193	20	7898	14	10096	30	11928	24	11780
3	12613	2	6208	2	7934	4	10137	8	11990	5	11833
10	12616	1	6211	3	7934	3	10141	6	11998	13	11842
4	12618	26	6211	4	7942	60	10146	8	12001	62	11849
12139	12622	5481	6216	7596	7943	8783	10155	11500	12009	10374	11857
21	12554	10	6189	11	7896	12	10100	20	11944	9	11796
7	12615	6	6216	10	7924	15	10147	15	11978	17	11836
39	12632	10	6224	16	7949	25	10162	40	12012	38	11862
14	12626	4	6222	8	7947	13	10162	11	12009	16	11863
26	12628	3	6224	15	7946	13	10166	22	12012	13	11866
33	12599	7	6203	19	7923	25	10150	29	11985	22	11834
0	18	0	38	0	37	0	155	1	41	0	329
34	12605	11	6200	17	7931	20	10110	39	11990	25	11828
9	12638	11	6223	7	7949	17	10171	15	12020	10	11874
10	12637	4	6223	6	7950	9	10175	10	12019	6	11873
15	12637	30	6224	12	7945	32	10170	14	12019	43	11873
32	12636	0	6221	29	7950	6	10173	45	12013	9	11868
0	25	0	47	0	42	0	176	0	49	5	370
18	12615	12	6211	3	7933	15	10165	16	12006	23	11867
16	12634	4	6220	5	7949	9	10175	10	12026	14	11881
25	12634	4	6222	16	7949	9	10177	28	12023	5	11884
0	27	0	51	0	47	0	191	0	57	0	400
0	25	0	46	2	42	2	186	1	53	3	374
101	12489	28	6168	52	7883	65	10077	74	11894	51	11757
10279	12602	4383	6208	6588	7929	7167	10148	9906	11994	8752	11849
19	27	14	51	39	48	44	190	41	58	93	404
1	25	1	48	0	43	1	174	1	54	0	371
27	12616	11	6218	28	7937	17	10162	30	12011	29	11868

chr2	11124504	0	46	12581	5	6218	33	7903	7	10166	55	11969	7	11872
chr2	11124507	0	0	24	0	47	0	44	0	178	0	56	2	382
chr2	11124508	0	274	12349	72	6145	189	7735	129	10027	254	11752	133	11723
chr2	11124510	0	40	12579	6	6217	22	7915	11	10151	39	11975	16	11852
chr2	11124511	0	11	12617	3	6219	8	7932	9	10159	4	12014	10	11858
chr2	11124513	0	11	12616	4	6219	10	7930	8	10160	8	12013	8	11861
chr2	11124514	0	12	12614	5	6217	10	7927	10	10159	5	12014	3	11868
chr2	11124516	0	48	12569	14	6206	33	7899	11	10149	58	11955	24	11841
chr2	11124517	1	12219	403	5560	661	7694	238	9057	1102	11616	396	10804	1058
chr2	11124518	1	26	1	36	15	48	1	105	79	56	2	243	156
chr2	11124519	0	26	12590	9	6210	8	7918	22	10129	14	11998	22	11830
chr2	11124520	0	13	12612	30	6190	8	7921	56	10107	11	12005	71	11797
chr2	11124522	0	45	12531	2	6195	27	7873	10	10123	36	11948	24	11822
chr2	11124523	1	12112	506	5732	490	7606	315	9400	752	11544	463	11124	720
chr2	11124524	1	27	0	37	14	50	1	114	79	58	3	259	153
chr2	11124525	0	19	12603	18	6203	12	7913	32	10129	13	11999	44	11814
chr2	11124526	0	12	12573	19	6173	9	7885	21	10097	6	11967	37	11772
chr2	11124528	0	48	12574	5	6215	31	7894	7	10149	28	11983	7	11851
chr2	11124529	1	12269	355	5667	549	7724	198	9214	940	11708	301	10926	930
chr2	11124530	1	4	22	3	49	20	32	12	183	17	47	28	389
chr2	11124531	0	67	12536	12	6201	36	7872	12	10133	59	11937	14	11830
chr2	11124532	0	15	12606	9	6214	6	7915	12	10147	11	11992	9	11842
chr2	11124534	1	12117	496	5350	864	7642	272	8770	1364	11566	412	10402	1440
chr2	11124535	1	26	1	25	27	53	1	74	124	73	1	170	253
chr2	11124536	0	0	27	0	52	0	54	0	198	0	74	0	424
chr2	11124537	0	26	12514	8	6191	11	7864	10	10084	24	11906	12	11756
chr2	11124538	0	23	12594	6	6212	26	7878	9	10138	29	11950	5	11827
chr2	11124540	0	12	12601	2	6218	1	7903	5	10138	7	11975	9	11816
chr2	11124542	0	1	26	0	52	0	54	2	199	0	75	1	423
chr2	11124543	0	32	12581	19	6203	30	7863	19	10121	40	11927	26	11785
chr2	11124544	0	11	12604	11	6210	7	7892	15	10125	12	11958	19	11794
chr2	11124546	1	11699	898	4929	1282	7337	551	8060	2066	11129	822	9567	2213
chr2	11124547	1	23	4	28	24	49	6	88	114	62	13	178	249
chr2	11124548	0	0	27	0	52	0	55	1	201	0	75	1	426
chr2	11124549	0	35	12548	18	6193	17	7851	28	10079	12	11917	28	11725
chr2	11124550	0	13	12581	70	6146	7	7872	104	10013	8	11938	120	11638
chr2	11124552	0	18	12532	16	6168	11	7845	20	10056	20	11885	31	11676
chr2	11124553	1	11881	543	5134	994	7490	287	8465	1532	11287	444	9897	1691
chr2	11124554	1	26	1	26	26	50	5	76	125	74	2	195	230
chr2	11124555	0	19	12553	10	6198	15	7846	11	10073	19	11891	19	11690
chr2	11124556	0	13	12529	68	6131	10	7834	90	9984	18	11876	107	11583
chr2	11124558	0	10	12417	7	6150	11	7750	10	10003	11	11792	8	11621
chr2	11124559	1	10261	2256	3940	2247	6430	1383	6550	3495	9740	2072	7752	3894
chr2	11124560	1	21	6	19	33	46	9	64	140	63	14	145	282
chr2	11124561	0	0	27	1	51	0	54	0	204	0	77	0	426
chr2	11124562	0	0	27	0	52	1	53	2	201	0	76	0	426

46	12627	5	6223	33	7936	7	10173	55	12024	7	11879
0	24	0	47	0	44	0	178	0	56	2	384
274	12623	72	6217	189	7924	129	10156	254	12006	133	11856
40	12619	6	6223	22	7937	11	10162	39	12014	16	11868
11	12628	3	6222	8	7940	9	10168	4	12018	10	11868
11	12627	4	6223	10	7940	8	10168	8	12021	8	11869
12	12626	5	6222	10	7937	10	10169	5	12019	3	11871
48	12617	14	6220	33	7932	11	10160	58	12013	24	11865
12219	12622	5560	6221	7694	7932	9057	10159	11616	12012	10804	11862
26	27	36	51	48	49	105	184	56	58	243	399
26	12616	9	6219	8	7926	22	10151	14	12012	22	11852
13	12625	30	6220	8	7929	56	10163	11	12016	71	11868
45	12576	2	6197	27	7900	10	10133	36	11984	24	11846
12112	12618	5732	6222	7606	7921	9400	10152	11544	12007	11124	11844
27	27	37	51	50	51	114	193	58	61	259	412
19	12622	18	6221	12	7925	32	10161	13	12012	44	11858
12	12585	19	6192	9	7894	21	10118	6	11973	37	11809
48	12622	5	6220	31	7925	7	10156	28	12011	7	11858
12269	12624	5667	6216	7724	7922	9214	10154	11708	12009	10926	11856
4	26	3	52	20	52	12	195	17	64	28	417
67	12603	12	6213	36	7908	12	10145	59	11996	14	11844
15	12621	9	6223	6	7921	12	10159	11	12003	9	11851
12117	12613	5350	6214	7642	7914	8770	10134	11566	11978	10402	11842
26	27	25	52	53	54	74	198	73	74	170	423
0	27	0	52	0	54	0	198	0	74	0	424
26	12540	8	6199	11	7875	10	10094	24	11930	12	11768
23	12617	6	6218	26	7904	9	10147	29	11979	5	11832
12	12613	2	6220	1	7904	5	10143	7	11982	9	11825
1	27	0	52	0	54	2	201	0	75	1	424
32	12613	19	6222	30	7893	19	10140	40	11967	26	11811
11	12615	11	6221	7	7899	15	10140	12	11970	19	11813
11699	12597	4929	6211	7337	7888	8060	10126	11129	11951	9567	11780
23	27	28	52	49	55	88	202	62	75	178	427
0	27	0	52	0	55	1	202	0	75	1	427
35	12583	18	6211	17	7868	28	10107	12	11929	28	11753
13	12594	70	6216	7	7879	104	10117	8	11946	120	11758
18	12550	16	6184	11	7856	20	10076	20	11905	31	11707
11881	12424	5134	6128	7490	7777	8465	9997	11287	11731	9897	11588
26	27	26	52	50	55	76	201	74	76	195	425
19	12572	10	6208	15	7861	11	10084	19	11910	19	11709
13	12542	68	6199	10	7844	90	10074	18	11894	107	11690
10	12427	7	6157	11	7761	10	10013	11	11803	8	11629
10261	12517	3940	6187	6430	7813	6550	10045	9740	11812	7752	11646
21	27	19	52	46	55	64	204	63	77	145	427
0	27	1	52	0	54	0	204	0	77	0	426
0	27	0	52	1	54	2	203	0	76	0	426

chr2	11124563	0	3	12365	0	6144	0	7690	0	9942	0	11600	0	11474
chr2	11124567	0	0	27	0	52	0	54	1	203	1	76	0	428
chr2	11124572	0	0	27	0	52	1	54	0	204	0	77	0	428
chr2	11124576	0	1	12052	0	6011	0	7351	4	9627	4	11143	0	11091
chr2	11124581	0	7	11958	1	5956	2	7285	2	9559	3	11042	1	10895
chr2	11124584	0	0	27	0	52	0	54	0	203	0	77	1	426
chr2	11124586	0	8	11690	2	5843	7	6994	6	9230	6	10583	7	10520
chr2	11124588	0	0	27	0	52	0	54	0	203	0	77	0	428
chr2	11124593	0	0	27	0	52	0	54	0	203	0	77	0	428
chr2	11124597	0	0	27	0	52	0	54	0	203	0	77	0	428
chr2	11124598	0	0	27	0	52	0	54	0	203	0	77	0	428
chr2	11124600	0	0	27	0	51	0	54	0	199	0	77	0	420
chr2	11124609	0	0	27	0	48	0	52	0	194	0	74	0	412
chr3	89184744	0	3	2821	2	1402	0	2247	2	1806	5	3205	0	1347
chr3	89184752	0	0	2876	2	1503	0	805	0	646	1	1025	1	302
chr3	89184753	0	0	2918	1	1449	0	2341	1	1911	1	3363	1	1410
chr3	89184754	0	0	2928	0	1452	0	2346	0	1919	0	3372	0	1417
chr3	89184756	0	0	2902	0	1442	0	2325	0	1911	1	3358	0	1409
chr3	89184758	0	1	2684	1	1384	3	1640	1	1712	1	2115	0	718
chr3	89184759	0	0	2970	1	1543	2	1650	1	1678	1	2133	2	699
chr3	89184760	0	8	2968	4	1539	3	1717	4	1760	4	2217	1	741
chr3	89184762	0	0	2928	0	1453	0	2348	1	1921	0	3383	0	1417
chr3	89184763	0	6	2911	1	1446	3	2345	1	1918	1	3380	0	1417
chr3	89184765	0	1	2986	0	1550	0	1757	0	1805	0	2266	0	754
chr3	89184768	0	3	2983	1	1548	0	1758	1	1805	0	2269	2	752
chr3	89184769	0	1	2986	0	1550	1	1759	1	1808	0	2272	1	754
chr3	89184773	0	0	2991	0	1552	0	1761	0	1810	0	2276	0	754
chr3	89184774	0	2	2933	5	1451	2	2353	2	1929	4	3398	2	1426
chr3	89184776	1	2323	615	1152	305	1865	494	1465	466	2730	678	1081	350
chr3	89184777	1	2331	655	1077	471	1348	413	1226	586	1757	518	560	197
chr3	89184778	0	3	2982	3	1541	4	1741	6	1789	6	2255	3	752
chr3	89184779	0	6	2992	5	1550	4	1756	6	1805	3	2273	1	752
chr3	89184780	0	9	2889	4	1438	5	2326	3	1896	6	3351	2	1417
chr3	89184781	0	4	2937	2	1457	3	2358	3	1930	9	3399	3	1427
chr3	89184782	0	7	2923	2	1446	4	2340	2	1922	4	3382	2	1423
chr3	89184783	0	4	2875	2	1422	6	2319	6	1895	8	3335	1	1406
chr3	89184787	0	2	3006	0	1561	0	1761	1	1814	1	2284	1	757
chr3	89184788	0	10	2895	1	1505	4	1734	1	1801	4	2227	2	740
chr3	89184792	0	19	2886	7	1499	3	1681	6	1750	11	2192	0	722
chr3	89184795	0	3	3006	3	1556	3	1767	2	1819	1	2288	1	760
chr3	89184800	0	3	3003	8	1557	1	1769	1	1823	2	2292	1	761
chr3	89184801	0	2	2934	1	1455	5	2355	1	1933	0	3411	3	1435
chr3	89184805	0	0	2942	1	1460	2	2363	1	1941	1	3422	2	1440
chr3	89184806	1	2552	390	1190	272	2078	288	1594	349	3028	393	1206	236
chr3	89184807	1	2534	486	1293	273	1451	333	1472	352	1895	404	643	125
chr3	89184808	0	2	3011	1	1559	2	1776	0	1823	2	2288	2	762

3	12368	0	6144	0	7690	0	9942	0	11600	0	11474
0	27	0	52	0	54	1	204	1	77	0	428
0	27	0	52	1	55	0	204	0	77	0	428
1	12053	0	6011	0	7351	4	9631	4	11147	0	11091
7	11965	1	5957	2	7287	2	9561	3	11045	1	10896
0	27	0	52	0	54	0	203	0	77	1	427
8	11698	2	5845	7	7001	6	9236	6	10589	7	10527
0	27	0	52	0	54	0	203	0	77	0	428
0	27	0	52	0	54	0	203	0	77	0	428
0	27	0	52	0	54	0	203	0	77	0	428
0	27	0	52	0	54	0	203	0	77	0	428
0	27	0	51	0	54	0	199	0	77	0	420
0	27	0	48	0	52	0	194	0	74	0	412
3	2824	2	1404	0	2247	2	1808	5	3210	0	1347
0	2876	2	1505	0	805	0	646	1	1026	1	303
0	2918	1	1450	0	2341	1	1912	1	3364	1	1411
0	2928	0	1452	0	2346	0	1919	0	3372	0	1417
0	2902	0	1442	0	2325	0	1911	1	3359	0	1409
1	2685	1	1385	3	1643	1	1713	1	2116	0	718
0	2970	1	1544	2	1652	1	1679	1	2134	2	701
8	2976	4	1543	3	1720	4	1764	4	2221	1	742
0	2928	0	1453	0	2348	1	1922	0	3383	0	1417
6	2917	1	1447	3	2348	1	1919	1	3381	0	1417
1	2987	0	1550	0	1757	0	1805	0	2266	0	754
3	2986	1	1549	0	1758	1	1806	0	2269	2	754
1	2987	0	1550	1	1760	1	1809	0	2272	1	755
0	2991	0	1552	0	1761	0	1810	0	2276	0	754
2	2935	5	1456	2	2355	2	1931	4	3402	2	1428
2323	2938	1152	1457	1865	2359	1465	1931	2730	3408	1081	1431
2331	2986	1077	1548	1348	1761	1226	1812	1757	2275	560	757
3	2985	3	1544	4	1745	6	1795	6	2261	3	755
6	2998	5	1555	4	1760	6	1811	3	2276	1	753
9	2898	4	1442	5	2331	3	1899	6	3357	2	1419
4	2941	2	1459	3	2361	3	1933	9	3408	3	1430
7	2930	2	1448	4	2344	2	1924	4	3386	2	1425
4	2879	2	1424	6	2325	6	1901	8	3343	1	1407
2	3008	0	1561	0	1761	1	1815	1	2285	1	758
10	2905	1	1506	4	1738	1	1802	4	2231	2	742
19	2905	7	1506	3	1684	6	1756	11	2203	0	722
3	3009	3	1559	3	1770	2	1821	1	2289	1	761
3	3006	8	1565	1	1770	1	1824	2	2294	1	762
2	2936	1	1456	5	2360	1	1934	0	3411	3	1438
0	2942	1	1461	2	2365	1	1942	1	3423	2	1442
2552	2942	1190	1462	2078	2366	1594	1943	3028	3421	1206	1442
2534	3020	1293	1566	1451	1784	1472	1824	1895	2299	643	768
2	3013	1	1560	2	1778	0	1823	2	2290	2	764

chr3	89184809	0	15	3006	6	1561	5	1774	4	1817	6	2285	1	764
chr3	89184810	0	4	3015	3	1564	6	1778	3	1821	8	2292	3	766
chr3	89184814	0	0	3004	2	1556	2	1772	3	1821	1	2296	0	767
chr3	89184815	1	2706	240	1306	158	2209	162	1732	214	3214	221	1296	150
chr3	89184816	1	2911	112	1453	114	1708	77	1671	155	2209	93	721	46
chr3	89184817	0	5	3018	2	1564	8	1778	6	1821	9	2298	1	771
chr3	89184818	0	11	2933	1	1459	9	2357	2	1944	27	3405	2	1445
chr3	89184820	0	8	2981	3	1544	1	1775	2	1814	3	2304	0	767
chr3	89184821	1	2586	361	1139	324	2103	271	1491	461	3076	364	1136	312
chr3	89184822	1	2514	506	1328	239	1460	313	1548	273	1848	449	649	114
chr3	89184824	1	2594	355	1034	430	2092	283	1352	603	3059	380	1042	408
chr3	89184825	1	2709	276	1280	251	1596	179	1452	356	2042	257	646	111
chr3	89184826	0	10	2864	4	1483	4	1624	9	1657	6	2093	2	718
chr3	89184827	0	6	2804	1	1434	4	1670	4	1731	4	2142	2	723
chr3	89184828	0	1	2891	4	1429	3	2311	3	1910	5	3373	2	1428
chr3	89184831	0	27	2924	1	1464	23	2357	1	1958	43	3407	0	1454
chr3	89184833	0	7	3006	1	1564	4	1761	4	1805	0	2275	3	756
chr3	89184834	0	39	2892	2	1521	17	1756	4	1797	30	2243	0	762
chr3	89184836	1	2507	444	1186	282	2046	336	1594	366	2981	471	1214	239
chr3	89184837	1	2679	345	1229	339	1562	232	1442	385	1982	327	622	149
chr3	89184839	0	6	3004	7	1556	1	1785	2	1815	2	2304	1	766
chr3	89184840	0	6	3021	7	1561	4	1787	4	1821	6	2306	0	771
chr3	89184842	0	2	2870	25	1450	0	1734	19	1781	3	2258	12	748
chr3	89184843	0	6	2990	2	1554	2	1711	3	1736	1	2224	0	736
chr3	89184844	1	2734	215	1343	123	2213	172	1791	168	3225	226	1336	119
chr3	89184845	1	2853	175	1464	107	1678	120	1703	140	2156	173	722	55
chr3	89184846	0	2	3010	5	1553	2	1791	2	1826	4	2317	0	771
chr3	89184848	0	12	3015	1	1568	15	1765	16	1812	18	2292	5	761
chr3	89184849	0	3	3025	0	1570	3	1788	4	1838	1	2328	3	770
chr3	89184850	1	2715	225	1320	146	2226	157	1756	205	3229	224	1320	132
chr3	89184851	1	2800	229	1298	273	1626	167	1503	338	2106	227	658	115
chr3	89184852	0	5	3020	0	1568	7	1785	3	1836	8	2321	4	769
chr3	89184853	1	2750	198	1320	149	2217	166	1780	182	3269	190	1325	129
chr3	89184854	1	2857	171	1313	257	1701	93	1478	362	2182	151	641	134
chr3	89184855	0	4	2799	1	1466	3	1733	5	1795	5	2266	1	754
chr3	89184856	1	2712	232	1283	170	2206	169	1724	222	3197	248	1295	155
chr3	89184857	1	2945	85	1373	197	1743	54	1562	277	2254	80	686	86
chr3	89184858	0	4	3025	3	1568	2	1793	2	1842	2	2334	1	772
chr3	89184861	0	2	3025	3	1567	2	1793	1	1840	3	2334	1	768
chr3	89184862	0	1	3023	13	1553	1	1795	13	1826	2	2333	8	761
chr3	89184864	0	4	3022	1	1570	1	1797	1	1841	2	2335	0	766
chr3	89184865	0	10	3020	3	1569	3	1796	4	1842	4	2332	5	768
chr3	89184866	0	5	3025	3	1569	2	1797	2	1844	4	2334	1	769
chr3	89184867	0	17	3005	5	1559	7	1788	2	1837	12	2322	0	768
chr3	89184868	0	47	2818	9	1401	39	2265	12	1861	31	3318	6	1399
chr3	89184870	0	7	2948	3	1463	4	2384	4	1967	6	3472	2	1458

15	3021	6	1567	5	1779	4	1821	6	2291	1	765
4	3019	3	1567	6	1784	3	1824	8	2300	3	769
0	3004	2	1558	2	1774	3	1824	1	2297	0	767
2706	2946	1306	1464	2209	2371	1732	1946	3214	3435	1296	1446
2911	3023	1453	1567	1708	1785	1671	1826	2209	2302	721	767
5	3023	2	1566	8	1786	6	1827	9	2307	1	772
11	2944	1	1460	9	2366	2	1946	27	3432	2	1447
8	2989	3	1547	1	1776	2	1816	3	2307	0	767
2586	2947	1139	1463	2103	2374	1491	1952	3076	3440	1136	1448
2514	3020	1328	1567	1460	1773	1548	1821	1848	2297	649	763
2594	2949	1034	1464	2092	2375	1352	1955	3059	3439	1042	1450
2709	2985	1280	1531	1596	1775	1452	1808	2042	2299	646	757
10	2874	4	1487	4	1628	9	1666	6	2099	2	720
6	2810	1	1435	4	1674	4	1735	4	2146	2	725
1	2892	4	1433	3	2314	3	1913	5	3378	2	1430
27	2951	1	1465	23	2380	1	1959	43	3450	0	1454
7	3013	1	1565	4	1765	4	1809	0	2275	3	759
39	2931	2	1523	17	1773	4	1801	30	2273	0	762
2507	2951	1186	1468	2046	2382	1594	1960	2981	3452	1214	1453
2679	3024	1229	1568	1562	1794	1442	1827	1982	2309	622	771
6	3010	7	1563	1	1786	2	1817	2	2306	1	767
6	3027	7	1568	4	1791	4	1825	6	2312	0	771
2	2872	25	1475	0	1734	19	1800	3	2261	12	760
6	2996	2	1556	2	1713	3	1739	1	2225	0	736
2734	2949	1343	1466	2213	2385	1791	1959	3225	3451	1336	1455
2853	3028	1464	1571	1678	1798	1703	1843	2156	2329	722	777
2	3012	5	1558	2	1793	2	1828	4	2321	0	771
12	3027	1	1569	15	1780	16	1828	18	2310	5	766
3	3028	0	1570	3	1791	4	1842	1	2329	3	773
2715	2940	1320	1466	2226	2383	1756	1961	3229	3453	1320	1452
2800	3029	1298	1571	1626	1793	1503	1841	2106	2333	658	773
5	3025	0	1568	7	1792	3	1839	8	2329	4	773
2750	2948	1320	1469	2217	2383	1780	1962	3269	3459	1325	1454
2857	3028	1313	1570	1701	1794	1478	1840	2182	2333	641	775
4	2803	1	1467	3	1736	5	1800	5	2271	1	755
2712	2944	1283	1453	2206	2375	1724	1946	3197	3445	1295	1450
2945	3030	1373	1570	1743	1797	1562	1839	2254	2334	686	772
4	3029	3	1571	2	1795	2	1844	2	2336	1	773
2	3027	3	1570	2	1795	1	1841	3	2337	1	769
1	3024	13	1566	1	1796	13	1839	2	2335	8	769
4	3026	1	1571	1	1798	1	1842	2	2337	0	766
10	3030	3	1572	3	1799	4	1846	4	2336	5	773
5	3030	3	1572	2	1799	2	1846	4	2338	1	770
17	3022	5	1564	7	1795	2	1839	12	2334	0	768
47	2865	9	1410	39	2304	12	1873	31	3349	6	1405
7	2955	3	1466	4	2388	4	1971	6	3478	2	1460

chr3	89184871	0	5	2952	2	1465	3	2388	2	1969	10	3475	0	1459
chr3	89184873	0	5	3012	4	1563	2	1789	3	1840	3	2320	2	770
chr3	89184875	0	0	2996	0	1564	1	1780	1	1833	2	2313	0	763
chr3	89184876	0	1	3018	8	1563	4	1787	5	1836	3	2322	3	762
chr3	89184878	0	4	3016	2	1569	2	1791	6	1837	3	2323	1	764
chr3	89184879	0	6	3014	17	1554	1	1791	18	1822	2	2324	10	754
chr3	89184880	0	6	3010	3	1566	6	1783	5	1833	6	2315	3	760
chr3	89184881	0	11	2820	6	1381	8	2274	6	1857	8	3329	3	1401
chr3	89184883	0	2	3004	0	1561	1	1786	1	1836	1	2317	0	759
chr3	89184885	0	10	2953	6	1462	7	2388	10	1966	2	3488	6	1458
chr3	89184888	0	4	2958	1	1467	1	2391	1	1973	1	3487	0	1463
chr3	89184890	0	6	2960	2	1467	4	2390	0	1976	4	3486	1	1464
chr3	89184891	1	1936	1015	820	643	1631	749	1117	846	2373	1103	836	624
chr3	89184892	1	1920	1099	947	621	1167	613	1051	775	1485	827	467	277
chr3	89184895	0	6	3009	3	1561	5	1772	2	1822	7	2303	3	737
chr3	89184896	0	2	3016	2	1562	2	1775	3	1821	4	2308	1	738
chr3	89184897	0	9	3007	2	1561	3	1775	10	1813	8	2303	2	739
chr3	89184898	0	0	2948	3	1459	1	2384	0	1967	2	3473	0	1464
chr3	89184902	0	6	2946	3	1462	5	2381	2	1966	8	3451	4	1457
chr3	89184903	0	0	2950	8	1455	3	2369	17	1941	5	3419	8	1450
chr3	89184905	0	0	3008	2	1559	1	1766	3	1808	4	2295	0	722
chr3	89184907	0	3	3007	3	1558	2	1765	1	1809	1	2301	2	721
chr3	89184908	0	6	2998	4	1555	3	1760	0	1807	3	2293	1	720
chr3	89184911	0	3	3000	0	1559	1	1760	0	1806	0	2294	0	721
chr3	89184913	0	4	3003	11	1549	2	1761	21	1785	6	2284	10	707
chr3	89184914	0	4	2966	4	1527	3	1741	1	1776	10	2250	1	705
chr3	89184915	0	3	2931	2	1447	7	2365	3	1964	9	3442	4	1458
chr3	89184917	0	3	3005	3	1556	1	1760	2	1804	1	2284	1	711
chr3	89184919	0	1	3007	4	1556	2	1759	2	1804	7	2276	0	713
chr3	89184920	0	38	2971	2	1557	27	1730	2	1804	22	2263	0	713
chr3	89184922	0	5	3004	1	1559	1	1758	2	1803	7	2281	2	711
chr3	89184923	0	18	2990	1	1559	10	1749	0	1803	9	2278	0	714
chr3	89184925	0	2	3007	3	1557	3	1756	1	1803	3	2283	0	714
chr3	89184926	0	4	3004	2	1558	4	1754	4	1799	3	2284	0	713
chr3	89184927	0	2	2959	1	1467	4	2385	2	1968	6	3461	4	1458
chr3	89184929	0	6	3003	3	1557	0	1758	3	1799	2	2285	1	712
chr3	89184931	0	2	3007	2	1557	0	1759	1	1801	2	2278	0	712
chr3	89184932	0	10	3000	0	1560	4	1755	1	1800	4	2278	2	711
chr3	89184933	0	2	2911	0	1446	1	2339	4	1937	0	3382	1	1448
chr3	89184938	0	9	2982	4	1549	6	1741	4	1781	7	2266	4	703
chr3	89184939	0	4	2912	2	1450	2	2348	0	1940	1	3384	0	1452
chr3	89184940	0	3	2913	1	1439	3	2339	2	1916	6	3376	0	1434
chr3	89184942	0	4	2867	1	1426	6	2348	1	1941	3	3376	1	1437
chr3	89184943	0	2	2944	1	1459	2	2358	0	1949	0	3409	1	1444
chr3	89184945	0	5	3001	2	1557	3	1754	3	1794	1	2271	0	708
chr3	89184946	0	8	2998	3	1555	3	1752	1	1794	2	2268	0	707

5	2957	2	1467	3	2391	2	1971	10	3485	0	1459
5	3017	4	1567	2	1791	3	1843	3	2323	2	772
0	2996	0	1564	1	1781	1	1834	2	2315	0	763
1	3019	8	1571	4	1791	5	1841	3	2325	3	765
4	3020	2	1571	2	1793	6	1843	3	2326	1	765
6	3020	17	1571	1	1792	18	1840	2	2326	10	764
6	3016	3	1569	6	1789	5	1838	6	2321	3	763
11	2831	6	1387	8	2282	6	1863	8	3337	3	1404
2	3006	0	1561	1	1787	1	1837	1	2318	0	759
10	2963	6	1468	7	2395	10	1976	2	3490	6	1464
4	2962	1	1468	1	2392	1	1974	1	3488	0	1463
6	2966	2	1469	4	2394	0	1976	4	3490	1	1465
1936	2951	820	1463	1631	2380	1117	1963	2373	3476	836	1460
1920	3019	947	1568	1167	1780	1051	1826	1485	2312	467	744
6	3015	3	1564	5	1777	2	1824	7	2310	3	740
2	3018	2	1564	2	1777	3	1824	4	2312	1	739
9	3016	2	1563	3	1778	10	1823	8	2311	2	741
0	2948	3	1462	1	2385	0	1967	2	3475	0	1464
6	2952	3	1465	5	2386	2	1968	8	3459	4	1461
0	2950	8	1463	3	2372	17	1958	5	3424	8	1458
0	3008	2	1561	1	1767	3	1811	4	2299	0	722
3	3010	3	1561	2	1767	1	1810	1	2302	2	723
6	3004	4	1559	3	1763	0	1807	3	2296	1	721
3	3003	0	1559	1	1761	0	1806	0	2294	0	721
4	3007	11	1560	2	1763	21	1806	6	2290	10	717
4	2970	4	1531	3	1744	1	1777	10	2260	1	706
3	2934	2	1449	7	2372	3	1967	9	3451	4	1462
3	3008	3	1559	1	1761	2	1806	1	2285	1	712
1	3008	4	1560	2	1761	2	1806	7	2283	0	713
38	3009	2	1559	27	1757	2	1806	22	2285	0	713
5	3009	1	1560	1	1759	2	1805	7	2288	2	713
18	3008	1	1560	10	1759	0	1803	9	2287	0	714
2	3009	3	1560	3	1759	1	1804	3	2286	0	714
4	3008	2	1560	4	1758	4	1803	3	2287	0	713
2	2961	1	1468	4	2389	2	1970	6	3467	4	1462
6	3009	3	1560	0	1758	3	1802	2	2287	1	713
2	3009	2	1559	0	1759	1	1802	2	2280	0	712
10	3010	0	1560	4	1759	1	1801	4	2282	2	713
2	2913	0	1446	1	2340	4	1941	0	3382	1	1449
9	2991	4	1553	6	1747	4	1785	7	2273	4	707
4	2916	2	1452	2	2350	0	1940	1	3385	0	1452
3	2916	1	1440	3	2342	2	1918	6	3382	0	1434
4	2871	1	1427	6	2354	1	1942	3	3379	1	1438
2	2946	1	1460	2	2360	0	1949	0	3409	1	1445
5	3006	2	1559	3	1757	3	1797	1	2272	0	708
8	3006	3	1558	3	1755	1	1795	2	2270	0	707

chr3	89184949	0	5	2723	2	1347	1	2261	1	1864	5	3311	1	1410
chr3	89184951	0	12	2989	1	1557	8	1745	1	1787	19	2246	0	706
chr3	89184953	1	2685	268	1190	277	2188	201	1597	372	3179	268	1206	254
chr3	89184954	1	2714	286	1281	274	1546	205	1430	354	2014	251	597	108
chr3	89184955	0	4	2997	1	1554	0	1750	1	1785	3	2262	0	706
chr3	89184956	0	5	2994	0	1554	4	1744	3	1782	4	2259	1	705
chr3	89184957	0	6	2939	5	1456	5	2384	3	1962	10	3433	4	1456
chr3	89184959	1	2718	221	1317	137	2209	167	1789	171	3187	230	1317	131
chr3	89184960	1	2797	196	1364	181	1604	135	1564	218	2082	172	613	90
chr3	89184961	0	30	2915	8	1457	20	2369	9	1956	28	3411	8	1449
chr3	89184963	0	9	2918	0	1456	7	2380	5	1952	14	3425	3	1452
chr3	89184965	0	0	2942	0	1462	2	2392	4	1965	0	3437	2	1457
chr3	89184967	0	6	2976	1	1548	4	1727	4	1771	2	2244	1	699
chr3	89184969	0	2	2927	2	1455	3	2382	4	1962	5	3421	0	1449
chr3	89184972	0	14	2920	4	1459	7	2373	5	1956	8	3420	8	1450
chr3	89184973	0	5	2919	2	1459	5	2375	5	1947	4	3414	3	1448
chr3	89184975	0	10	2918	9	1453	5	2362	9	1951	12	3395	10	1438
chr3	89184978	1	2775	162	1329	132	2253	132	1794	162	3271	150	1325	129
chr3	89184979	1	2807	178	1411	135	1606	125	1610	172	2098	156	635	66
chr3	89184980	0	4	2982	1	1546	0	1729	0	1782	2	2250	0	701
chr3	89184981	0	7	2979	0	1545	3	1725	2	1780	4	2247	1	700
chr3	89184982	0	10	2964	2	1537	1	1715	5	1768	4	2234	1	697
chr3	89184983	0	3	2926	2	1455	3	2375	3	1951	9	3402	1	1445
chr3	89184985	0	1	2978	0	1546	0	1724	2	1777	1	2246	2	697
chr3	89184986	0	1	2975	1	1537	1	1719	2	1774	0	2245	0	698
chr3	89184987	0	0	2981	0	1546	2	1722	0	1780	0	2250	0	699
chr3	89184988	0	0	2975	0	1545	0	1724	0	1777	0	2248	0	698
chr3	89184989	0	0	2933	1	1525	0	1685	0	1753	1	2211	1	693
chr3	89184990	0	7	2877	0	1445	4	2366	5	1939	8	3379	1	1442
chr3	89184993	0	2	2901	10	1428	0	2365	12	1934	3	3386	13	1431
chr3	89184994	0	12	2884	3	1437	10	2354	0	1945	13	3373	1	1443
chr3	89184996	0	6	2967	1	1539	0	1716	1	1769	1	2241	1	698
chr3	89184997	0	2	2761	1	1385	1	2286	1	1883	4	3270	1	1390
chr3	89184998	0	1	2758	0	1380	1	2283	0	1883	0	3266	0	1384
chr3	89184999	0	1	2756	2	1376	3	2279	0	1883	0	3274	0	1381
chr3	89185001	0	0	2749	0	1380	1	2275	0	1882	1	3261	1	1379
chr3	89185003	0	2	2877	1	1487	3	1658	2	1718	1	2164	1	673
chr3	89185004	0	1	2747	3	1378	2	2276	0	1882	5	3251	0	1378
chr3	89185005	0	1	2733	2	1362	2	2256	0	1872	0	3207	1	1366
chr3	89185008	0	6	2436	2	1266	4	1238	0	1226	5	1573	0	478
chr3	89185015	0	0	2250	2	1114	1	1742	2	1493	2	2529	3	1070
chr3	89185017	0	0	2214	0	1090	2	1693	0	1442	0	2448	1	1038
chr3	89185018	0	1	2059	2	996	1	1636	0	1424	2	2314	0	1001
chr3	89185022	0	0	2150	1	1067	1	1647	0	1395	0	2403	0	1025
chr3	89679038	0	2	3703	5	13512	0	1763	6	10102	1	1951	0	1991
chr3	89679041	0	3	3889	6	14221	1	1926	9	10762	1	2116	3	2125

5	2728	2	1349	1	2262	1	1865	5	3316	1	1411
12	3001	1	1558	8	1753	1	1788	19	2265	0	706
2685	2953	1190	1467	2188	2389	1597	1969	3179	3447	1206	1460
2714	3000	1281	1555	1546	1751	1430	1784	2014	2265	597	705
4	3001	1	1555	0	1750	1	1786	3	2265	0	706
5	2999	0	1554	4	1748	3	1785	4	2263	1	706
6	2945	5	1461	5	2389	3	1965	10	3443	4	1460
2718	2939	1317	1454	2209	2376	1789	1960	3187	3417	1317	1448
2797	2993	1364	1545	1604	1739	1564	1782	2082	2254	613	703
30	2945	8	1465	20	2389	9	1965	28	3439	8	1457
9	2927	0	1456	7	2387	5	1957	14	3439	3	1455
0	2942	0	1462	2	2394	4	1969	0	3437	2	1459
6	2982	1	1549	4	1731	4	1775	2	2246	1	700
2	2929	2	1457	3	2385	4	1966	5	3426	0	1449
14	2934	4	1463	7	2380	5	1961	8	3428	8	1458
5	2924	2	1461	5	2380	5	1952	4	3418	3	1451
10	2928	9	1462	5	2367	9	1960	12	3407	10	1448
2775	2937	1329	1461	2253	2385	1794	1956	3271	3421	1325	1454
2807	2985	1411	1546	1606	1731	1610	1782	2098	2254	635	701
4	2986	1	1547	0	1729	0	1782	2	2252	0	701
7	2986	0	1545	3	1728	2	1782	4	2251	1	701
10	2974	2	1539	1	1716	5	1773	4	2238	1	698
3	2929	2	1457	3	2378	3	1954	9	3411	1	1446
1	2979	0	1546	0	1724	2	1779	1	2247	2	699
1	2976	1	1538	1	1720	2	1776	0	2245	0	698
0	2981	0	1546	2	1724	0	1780	0	2250	0	699
0	2975	0	1545	0	1724	0	1777	0	2248	0	698
0	2933	1	1526	0	1685	0	1753	1	2212	1	694
7	2884	0	1445	4	2370	5	1944	8	3387	1	1443
2	2903	10	1438	0	2365	12	1946	3	3389	13	1444
12	2896	3	1440	10	2364	0	1945	13	3386	1	1444
6	2973	1	1540	0	1716	1	1770	1	2242	1	699
2	2763	1	1386	1	2287	1	1884	4	3274	1	1391
1	2759	0	1380	1	2284	0	1883	0	3266	0	1384
1	2757	2	1378	3	2282	0	1883	0	3274	0	1381
0	2749	0	1380	1	2276	0	1882	1	3262	1	1380
2	2879	1	1488	3	1661	2	1720	1	2165	1	674
1	2748	3	1381	2	2278	0	1882	5	3256	0	1378
1	2734	2	1364	2	2258	0	1872	0	3207	1	1367
6	2442	2	1268	4	1242	0	1226	5	1578	0	478
0	2250	2	1116	1	1743	2	1495	2	2531	3	1073
0	2214	0	1090	2	1695	0	1442	0	2448	1	1039
1	2060	2	998	1	1637	0	1424	2	2316	0	1001
0	2150	1	1068	1	1648	0	1395	0	2403	0	1025
2	3705	5	13517	0	1763	6	10108	1	1952	0	1991
3	3892	6	14227	1	1927	9	10771	1	2117	3	2128

chr3	89679051	0	7	4039	1	14799	0	2104	3	11644	0	2375	2	2483
chr3	89679054	0	4	4162	14	15252	4	2189	15	12559	2	2495	4	2955
chr3	89679072	1	4925	582	14271	2435	2927	369	14502	2550	3868	380	5644	523
chr3	89679075	0	9	5429	58	16359	6	3255	50	16891	8	4201	17	6122
chr3	89679079	0	6	5532	30	16781	5	3350	42	17408	12	4333	12	6352
chr3	89679081	0	4	5524	49	16729	3	3357	47	17412	6	4345	17	6372
chr3	89679091	0	7	5536	18	16803	3	3373	14	17621	5	4381	7	6524
chr3	89679103	0	8	5604	46	16814	6	3437	54	17738	7	4464	28	6755
chr3	89679107	1	5336	306	14280	2604	3193	272	14852	3011	4228	276	6087	805
chr3	89679108	0	3	5639	17	16873	6	3459	20	17844	11	4495	14	6886
chr3	89679109	0	12	5630	74	16815	3	3461	66	17801	3	4501	19	6883
chr3	89679117	0	19	82314	6	50952	11	77016	12	78848	10	1E+05	15	84219
chr3	89679123	0	5	5638	68	16825	3	3468	72	17849	3	4530	29	6938
chr3	89679126	0	10	83984	6	51607	14	78299	12	79974	17	1E+05	12	85917
chr3	89679132	0	10	83943	6	51548	5	78239	6	79935	10	1E+05	7	85865
chr3	89679136	0	23	5597	30	16780	19	3446	37	17840	26	4504	12	6976
chr3	89679137	0	27	81199	10	50110	16	75977	22	77713	42	1E+05	28	83519
chr3	89679141	0	11	84088	9	51553	11	78417	15	80028	15	1E+05	17	86086
chr3	89679142	0	304	84018	107	51599	354	78318	118	80109	523	1E+05	171	86127
chr3	89679144	0	22	5620	23	16879	9	3484	21	17960	9	4563	6	7059
chr3	89679146	0	195	84160	81	51642	214	78487	116	80165	296	1E+05	149	86231
chr3	89679148	0	3	5638	14	16884	7	3486	24	17961	1	4573	10	7058
chr3	89679149	0	4	5636	25	16861	4	3487	20	17958	4	4558	4	7064
chr3	89679150	0	176	83823	51	51422	189	78194	106	79803	314	1E+05	111	85892
chr3	89679155	1	80187	4210	31666	20063	74505	4227	49372	30917	114446	6714	57165	29229
chr3	89679156	1	5500	142	13067	3823	3410	82	13810	4176	4459	114	5797	1284
chr3	89679158	0	57	84221	115	51537	69	78563	181	80023	129	1E+05	200	86108
chr3	89679162	1	80251	4052	37293	14350	74664	4010	57821	22329	114851	6199	66143	20154
chr3	89679163	1	5491	152	13601	3276	3400	92	14427	3545	4471	101	5993	1089
chr3	89679164	0	164	84042	88	51423	119	78404	114	79831	237	1E+05	163	85851
chr3	89679165	0	481	83877	70	51657	417	78309	108	80183	648	1E+05	147	86272
chr3	89679167	0	213	84122	88	51614	173	78517	129	80130	298	1E+05	176	86197
chr3	89679168	0	548	83791	153	51554	474	78210	239	80015	756	1E+05	307	86079
chr3	89679169	0	1103	83302	1646	50096	1145	77609	2476	77850	1614	1E+05	2891	83565
chr3	89679171	0	125	84295	256	51484	125	78625	383	79942	194	1E+05	444	86013
chr3	89679173	1	76891	6914	30220	21107	71673	6501	46672	32973	110194	10106	54552	31223
chr3	89679174	1	5459	184	13046	3844	3360	127	13698	4272	4448	123	5804	1280
chr3	89679176	0	58	5572	54	16811	28	3451	60	17868	40	4521	23	7043
chr3	89679177	0	890	83380	660	50988	819	77776	1069	79116	1293	1E+05	1153	85169
chr3	89679180	0	258	84120	70	51660	253	78458	139	80143	381	1E+05	166	86233
chr3	89679181	0	157	84246	81	51650	143	78588	102	80208	205	1E+05	127	86302
chr3	89679182	0	353	84060	181	51561	324	78418	273	80050	501	1E+05	318	86138
chr3	89679184	0	21	5612	44	16829	13	3458	39	17838	19	4535	23	7027
chr3	89679186	1	79422	4972	31981	19753	74213	4513	49511	30771	114200	6974	57154	29253
chr3	89679187	1	5457	183	12964	3911	3381	103	13584	4364	4429	136	5685	1392
chr3	89679188	0	202	84070	147	51520	193	78445	197	80024	269	1E+05	221	86112

7	4046	1	14800	0	2104	3	11647	0	2375	2	2485
4	4166	14	15266	4	2193	15	12574	2	2497	4	2959
4925	5507	14271	16706	2927	3296	####	17052	3868	4248	5644	6167
9	5438	58	16417	6	3261	50	16941	8	4209	17	6139
6	5538	30	16811	5	3355	42	17450	12	4345	12	6364
4	5528	49	16778	3	3360	47	17459	6	4351	17	6389
7	5543	18	16821	3	3376	14	17635	5	4386	7	6531
8	5612	46	16860	6	3443	54	17792	7	4471	28	6783
5336	5642	14280	16884	3193	3465	####	17863	4228	4504	6087	6892
3	5642	17	16890	6	3465	20	17864	11	4506	14	6900
12	5642	74	16889	3	3464	66	17867	3	4504	19	6902
19	82333	6	50958	11	77027	12	78860	10	1E+05	15	84234
5	5643	68	16893	3	3471	72	17921	3	4533	29	6967
10	83994	6	51613	14	78313	12	79986	17	1E+05	12	85929
10	83953	6	51554	5	78244	6	79941	10	1E+05	7	85872
23	5620	30	16810	19	3465	37	17877	26	4530	12	6988
27	81226	10	50120	16	75993	22	77735	42	1E+05	28	83547
11	84099	9	51562	11	78428	15	80043	15	1E+05	17	86103
304	84322	107	51706	354	78672	118	80227	523	1E+05	171	86298
22	5642	23	16902	9	3493	21	17981	9	4572	6	7065
195	84355	81	51723	214	78701	116	80281	296	1E+05	149	86380
3	5641	14	16898	7	3493	24	17985	1	4574	10	7068
4	5640	25	16886	4	3491	20	17978	4	4562	4	7068
176	83999	51	51473	189	78383	106	79909	314	1E+05	111	86003
80187	84397	31666	51729	####	78732	####	80289	####	1E+05	57165	86394
5500	5642	13067	16890	3410	3492	####	17986	4459	4573	5797	7081
57	84278	115	51652	69	78632	181	80204	129	1E+05	200	86308
80251	84303	37293	51643	####	78674	####	80150	####	1E+05	66143	86297
5491	5643	13601	16877	3400	3492	####	17972	4471	4572	5993	7082
164	84206	88	51511	119	78523	114	79945	237	1E+05	163	86014
481	84358	70	51727	417	78726	108	80291	648	1E+05	147	86419
213	84335	88	51702	173	78690	129	80259	298	1E+05	176	86373
548	84339	153	51707	474	78684	239	80254	756	1E+05	307	86386
1103	84405	1646	51742	1145	78754	2476	80326	1614	1E+05	2891	86456
125	84420	256	51740	125	78750	383	80325	194	1E+05	444	86457
76891	83805	30220	51327	####	78174	####	79645	####	1E+05	54552	85775
5459	5643	13046	16890	3360	3487	####	17970	4448	4571	5804	7084
58	5630	54	16865	28	3479	60	17928	40	4561	23	7066
890	84270	660	51648	819	78595	1069	80185	1293	1E+05	1153	86322
258	84378	70	51730	253	78711	139	80282	381	1E+05	166	86399
157	84403	81	51731	143	78731	102	80310	205	1E+05	127	86429
353	84413	181	51742	324	78742	273	80323	501	1E+05	318	86456
21	5633	44	16873	13	3471	39	17877	19	4554	23	7050
79422	84394	31981	51734	####	78726	49511	80282	####	1E+05	57154	86407
5457	5640	12964	16875	3381	3484	####	17948	4429	4565	5685	7077
202	84272	147	51667	193	78638	197	80221	269	1E+05	221	86333

chr3	89679190	0	6	5636	27	16859	1	3482	23	17934	8	4560	6	7074
chr3	89679192	0	18	5624	65	16824	10	3475	72	17881	13	4555	34	7042
chr3	89679193	0	19	5615	31	16848	8	3470	18	17917	9	4559	15	7055
chr3	89679194	0	237	84049	160	51518	220	78404	264	79929	327	1E+05	249	86053
chr3	89679196	0	254	84089	212	51505	217	78476	295	79980	429	1E+05	337	86043
chr3	89679198	0	19	5601	31	16809	12	3457	37	17853	16	4543	14	7037
chr3	89679199	0	247	83137	144	50997	214	77546	225	79214	335	1E+05	282	85212
chr3	89679200	1	80241	4151	33209	18515	74794	3916	51261	29040	115063	6086	58958	27436
chr3	89679201	1	5383	229	12759	4064	3331	137	13353	4523	4366	183	5678	1371
chr3	89679202	0	3	5547	14	16648	1	3443	16	17716	5	4500	7	6970
chr3	89679203	0	221	83845	86	51456	175	78271	133	79894	272	1E+05	132	85939
chr3	89679210	0	104	83878	391	51141	90	78302	638	79361	146	1E+05	671	85377
chr3	89679212	0	10	84227	8	51624	13	78544	12	80114	23	1E+05	7	86265
chr3	89679213	0	4	84091	10	51539	5	78443	10	80038	7	1E+05	5	86164
chr3	89679214	0	5	84405	5	51733	7	78715	9	80302	10	1E+05	6	86420
chr3	89679216	0	1	5492	0	16641	1	3362	1	17443	0	4418	0	6885
chr3	89679218	0	9	83989	4	51482	8	78349	6	79956	6	1E+05	8	86044
chr3	89679220	0	3	84313	3	51695	6	78630	8	80217	13	1E+05	5	86336
chr3	89679224	0	20	5139	38	15882	13	3127	51	16518	6	4155	19	6511
chr3	89679226	0	0	5201	7	15956	4	3115	12	16175	0	4107	0	6397
chr3	89679232	0	14	83566	7	51422	9	78120	9	79800	12	1E+05	12	85811
chr4	44628612	0	0	71	0	43	1	1015	0	197	1	441	0	60
chr4	44628613	0	0	73	0	44	0	1019	0	202	0	446	0	62
chr4	44628615	0	0	74	0	44	0	1054	1	206	0	458	0	62
chr4	44628620	0	0	71	0	42	0	1039	0	205	0	453	0	61
chr4	44628623	0	0	74	0	44	0	1064	0	207	0	464	0	62
chr4	44628630	0	0	74	0	43	1	1051	0	206	0	462	0	61
chr4	44628631	0	0	75	0	44	0	1065	0	207	0	465	0	62
chr4	44628632	0	0	75	0	44	2	1059	0	207	0	464	1	61
chr4	44628640	0	0	77	3	41	5	1062	17	191	4	462	3	59
chr4	44628641	0	0	77	0	44	3	1062	0	208	1	465	1	61
chr4	44628642	0	0	77	0	44	0	1068	0	208	0	467	0	62
chr4	44628643	1	59	18	35	9	802	267	166	42	342	124	46	16
chr4	44628646	1	64	13	42	2	926	143	190	18	408	58	56	6
chr4	44628652	0	0	76	0	44	3	1056	1	205	2	459	0	61
chr4	44628657	0	0	77	0	44	1	1061	0	205	0	466	0	62
chr4	44628659	0	0	77	0	43	0	1065	0	207	0	464	0	62
chr4	44628661	0	0	77	0	44	0	1073	0	209	0	469	0	62
chr4	44628663	0	1	79	0	44	2	1073	0	208	2	466	0	62
chr4	44628665	0	0	80	0	44	1	1073	1	207	0	468	0	61
chr4	44628666	0	0	80	0	44	0	1075	0	208	0	469	0	61
chr4	44628667	0	0	80	0	44	1	1075	0	208	1	468	0	61
chr4	44628670	0	0	81	0	44	1	1075	1	207	1	469	0	61
chr4	44628671	0	0	81	0	44	0	1074	0	208	0	469	0	61
chr4	44628679	0	0	81	0	44	1	1070	0	206	3	467	0	60
chr4	44628680	0	0	81	0	44	1	1076	1	207	0	472	0	60

6	5642	27	16886	1	3483	23	17957	8	4568	6	7080
18	5642	65	16889	10	3485	72	17953	13	4568	34	7076
19	5634	31	16879	8	3478	18	17935	9	4568	15	7070
237	84286	160	51678	220	78624	264	80193	327	1E+05	249	86302
254	84343	212	51717	217	78693	295	80275	429	1E+05	337	86380
19	5620	31	16840	12	3469	37	17890	16	4559	14	7051
247	83384	144	51141	214	77760	225	79439	335	1E+05	282	85494
80241	84392	33209	51724	####	78710	####	80301	####	1E+05	58958	86394
5383	5612	12759	16823	3331	3468	####	17876	4366	4549	5678	7049
3	5550	14	16662	1	3444	16	17732	5	4505	7	6977
221	84066	86	51542	175	78446	133	80027	272	1E+05	132	86071
104	83982	391	51532	90	78392	638	79999	146	1E+05	671	86048
10	84237	8	51632	13	78557	12	80126	23	1E+05	7	86272
4	84095	10	51549	5	78448	10	80048	7	1E+05	5	86169
5	84410	5	51738	7	78722	9	80311	10	1E+05	6	86426
1	5493	0	16641	1	3363	1	17444	0	4418	0	6885
9	83998	4	51486	8	78357	6	79962	6	1E+05	8	86052
3	84316	3	51698	6	78636	8	80225	13	1E+05	5	86341
20	5159	38	15920	13	3140	51	16569	6	4161	19	6530
0	5201	7	15963	4	3119	12	16187	0	4107	0	6397
14	83580	7	51429	9	78129	9	79809	12	1E+05	12	85823
0	71	0	43	1	1016	0	197	1	442	0	60
0	73	0	44	0	1019	0	202	0	446	0	62
0	74	0	44	0	1054	1	207	0	458	0	62
0	71	0	42	0	1039	0	205	0	453	0	61
0	74	0	44	0	1064	0	207	0	464	0	62
0	74	0	43	1	1052	0	206	0	462	0	61
0	75	0	44	0	1065	0	207	0	465	0	62
0	75	0	44	2	1061	0	207	0	464	1	62
0	77	3	44	5	1067	17	208	4	466	3	62
0	77	0	44	3	1065	0	208	1	466	1	62
0	77	0	44	0	1068	0	208	0	467	0	62
59	77	35	44	802	1069	166	208	342	466	46	62
64	77	42	44	926	1069	190	208	408	466	56	62
0	76	0	44	3	1059	1	206	2	461	0	61
0	77	0	44	1	1062	0	205	0	466	0	62
0	77	0	43	0	1065	0	207	0	464	0	62
0	77	0	44	0	1073	0	209	0	469	0	62
1	80	0	44	2	1075	0	208	2	468	0	62
0	80	0	44	1	1074	1	208	0	468	0	61
0	80	0	44	0	1075	0	208	0	469	0	61
0	80	0	44	1	1076	0	208	1	469	0	61
0	81	0	44	1	1076	1	208	1	470	0	61
0	81	0	44	0	1074	0	208	0	469	0	61
0	81	0	44	1	1071	0	206	3	470	0	60
0	81	0	44	1	1077	1	208	0	472	0	60

chr4	44628684	1	68	13	37	7	955	123	168	40	420	52	47	13
chr4	44628686	0	0	81	0	44	2	1073	2	207	1	471	0	60
chr4	44628687	0	0	81	1	43	3	1074	0	210	1	471	1	59
chr4	44628690	0	0	81	0	44	2	1076	0	210	2	470	0	59
chr4	44628692	0	1	80	0	44	9	1068	1	208	2	471	0	59
chr4	44628694	0	0	82	0	44	1	1076	0	209	4	468	0	59
chr4	44628695	0	0	82	0	44	3	1074	0	209	4	469	0	59
chr4	44628697	0	0	82	0	43	0	1075	1	206	2	471	0	59
chr4	44628698	0	0	82	0	44	1	1076	0	208	0	473	0	59
chr4	44628700	0	0	84	0	45	1	1077	0	208	0	473	0	58
chr4	44628701	0	0	85	0	44	2	1076	0	208	2	471	1	57
chr4	44628702	0	1	84	0	45	4	1073	0	208	0	473	0	58
chr4	44628703	0	0	86	0	45	0	1075	0	207	1	471	0	56
chr4	44628706	0	0	86	0	45	0	1071	0	208	1	469	0	56
chr4	44628707	0	0	86	0	45	2	1076	0	208	0	472	0	56
chr4	44628708	0	0	86	0	45	0	1076	1	206	2	470	0	55
chr4	44628712	0	0	87	0	45	0	1079	0	208	0	477	0	54
chr4	44628716	0	0	83	0	45	5	1070	1	206	2	472	0	53
chr4	44628717	0	0	88	0	47	3	1075	0	207	0	478	0	53
chr4	44628737	0	0	88	1	46	4	1078	0	205	2	484	0	53
chr4	44628738	0	0	87	0	47	3	1079	2	205	1	487	0	53
chr4	44628740	0	0	86	0	47	4	1081	1	205	0	487	0	53
chr4	44628746	0	3	82	0	47	1	1085	0	204	6	485	0	52
chr4	44628750	0	1	85	0	47	0	1089	0	204	1	493	0	53
chr4	44628751	0	0	85	1	46	4	1080	1	202	0	495	0	53
chr4	44628786	0	0	39	0	14	6	317	1	76	2	182	0	20
chr4	44628787	1	75	15	45	4	1002	88	198	5	457	51	52	1
chr4	44628795	0	0	91	0	48	3	1088	0	197	1	513	0	54
chr4	44628796	0	1	90	0	46	4	1083	1	197	2	510	0	55
chr4	44628797	0	0	87	0	47	0	1075	0	192	0	502	0	55
chr4	44628798	0	0	91	0	47	1	1088	0	200	3	516	1	53
chr4	44628799	1	73	18	38	9	962	134	175	25	447	73	41	14
chr4	44628804	0	0	91	0	45	4	1097	2	197	2	513	0	54
chr4	44628807	0	0	92	0	47	6	1087	1	196	1	516	0	54
chr4	44628819	0	0	93	0	48	0	1105	0	199	0	523	0	53
chr4	44628821	0	0	96	0	48	1	1106	0	198	1	526	0	54
chr4	44628828	0	0	97	0	49	4	1105	0	203	2	530	0	55
chr4	44628832	1	88	7	49	0	1038	74	201	1	477	47	55	0
chr4	44628834	0	2	93	0	49	2	1102	0	201	1	524	0	55
chr4	44628843	0	0	98	0	50	3	1111	0	203	2	527	1	54
chr4	44628845	0	0	98	1	49	2	1102	0	203	1	517	0	55
chr4	44628851	0	0	101	1	49	3	1111	2	198	2	526	0	55
chr4	44628852	0	0	100	1	49	1	1103	0	200	0	519	0	53
chr4	44628856	0	1	98	0	50	0	1086	0	200	1	520	0	53
chr4	44628862	0	0	101	0	48	1	1082	0	195	0	519	0	53
chr4	44628869	0	0	101	0	50	2	1109	1	199	0	526	0	55

68	81	37	44	955	1078	168	208	420	472	47	60
0	81	0	44	2	1075	2	209	1	472	0	60
0	81	1	44	3	1077	0	210	1	472	1	60
0	81	0	44	2	1078	0	210	2	472	0	59
1	81	0	44	9	1077	1	209	2	473	0	59
0	82	0	44	1	1077	0	209	4	472	0	59
0	82	0	44	3	1077	0	209	4	473	0	59
0	82	0	43	0	1075	1	207	2	473	0	59
0	82	0	44	1	1077	0	208	0	473	0	59
0	84	0	45	1	1078	0	208	0	473	0	58
0	85	0	44	2	1078	0	208	2	473	1	58
1	85	0	45	4	1077	0	208	0	473	0	58
0	86	0	45	0	1075	0	207	1	472	0	56
0	86	0	45	0	1071	0	208	1	470	0	56
0	86	0	45	2	1078	0	208	0	472	0	56
0	86	0	45	0	1076	1	207	2	472	0	55
0	87	0	45	0	1079	0	208	0	477	0	54
0	83	0	45	5	1075	1	207	2	474	0	53
0	88	0	47	3	1078	0	207	0	478	0	53
0	88	1	47	4	1082	0	205	2	486	0	53
0	87	0	47	3	1082	2	207	1	488	0	53
0	86	0	47	4	1085	1	206	0	487	0	53
3	85	0	47	1	1086	0	204	6	491	0	52
1	86	0	47	0	1089	0	204	1	494	0	53
0	85	1	47	4	1084	1	203	0	495	0	53
0	39	0	14	6	323	1	77	2	184	0	20
75	90	45	49	1002	1090	198	203	457	508	52	53
0	91	0	48	3	1091	0	197	1	514	0	54
1	91	0	46	4	1087	1	198	2	512	0	55
0	87	0	47	0	1075	0	192	0	502	0	55
0	91	0	47	1	1089	0	200	3	519	1	54
73	91	38	47	962	1096	175	200	447	520	41	55
0	91	0	45	4	1101	2	199	2	515	0	54
0	92	0	47	6	1093	1	197	1	517	0	54
0	93	0	48	0	1105	0	199	0	523	0	53
0	96	0	48	1	1107	0	198	1	527	0	54
0	97	0	49	4	1109	0	203	2	532	0	55
88	95	49	49	1038	1112	201	202	477	524	55	55
2	95	0	49	2	1104	0	201	1	525	0	55
0	98	0	50	3	1114	0	203	2	529	1	55
0	98	1	50	2	1104	0	203	1	518	0	55
0	101	1	50	3	1114	2	200	2	528	0	55
0	100	1	50	1	1104	0	200	0	519	0	53
1	99	0	50	0	1086	0	200	1	521	0	53
0	101	0	48	1	1083	0	195	0	519	0	53
0	101	0	50	2	1111	1	200	0	526	0	55

chr4	44628871	0	0	101	0	50	2	1113	0	201	2	525	0	55
chr4	44628876	0	0	101	0	50	3	1108	2	198	2	523	0	55
chr4	44628882	0	0	101	1	49	4	1107	1	198	3	521	0	55
chr4	44628883	0	0	100	0	50	3	1105	0	200	0	526	0	55
chr4	44628889	0	0	101	0	50	0	1107	2	198	2	522	0	55
chr4	44628890	0	1	100	0	50	4	1101	0	199	3	518	1	54
chr4	44628891	0	0	101	0	50	4	1103	0	200	1	523	0	55
chr4	44628893	0	0	101	0	50	2	1104	0	200	0	522	0	55
chr4	44628898	0	0	100	0	48	4	1091	0	197	1	520	0	55
chr4	44628906	0	1	100	0	50	1	1101	0	199	0	521	0	55
chr4	44628908	1	89	12	45	5	905	199	174	25	435	87	50	5
chr4	44628911	0	0	101	0	49	0	1097	0	195	1	517	0	55
chr4	44628912	0	0	100	0	49	0	1099	0	197	0	521	0	55
chr4	44628918	0	0	101	0	50	0	1100	0	197	0	520	0	55
chr4	44628931	0	0	99	0	49	5	1078	0	194	2	508	0	54

0	101	0	50	2	1115	0	201	2	527	0	55
0	101	0	50	3	1111	2	200	2	525	0	55
0	101	1	50	4	1111	1	199	3	524	0	55
0	100	0	50	3	1108	0	200	0	526	0	55
0	101	0	50	0	1107	2	200	2	524	0	55
1	101	0	50	4	1105	0	199	3	521	1	55
0	101	0	50	4	1107	0	200	1	524	0	55
0	101	0	50	2	1106	0	200	0	522	0	55
0	100	0	48	4	1095	0	197	1	521	0	55
1	101	0	50	1	1102	0	199	0	521	0	55
89	101	45	50	905	1104	174	199	435	522	50	55
0	101	0	49	0	1097	0	195	1	518	0	55
0	100	0	49	0	1099	0	197	0	521	0	55
0	101	0	50	0	1100	0	197	0	520	0	55
0	99	0	49	5	1083	0	194	2	510	0	54

CD4 SP wild type cytosines

Number of C's and T's

			spbs1		spoxbs1		spbs2		spoxbs2		spbs3		spoxbs3	
**Chromosome**	**Position**		**C to C**	**C to T**	**C to C**	**C to T**	**C to C**	**C to T**	**C to C**	**C to T**	**C to C**	**C to T**	**C to C**	**C to T**
chr2	11124412	0	6	7383	10	6831	4	8819	4	8312	11	11080	7	10308
chr2	11124413	0	4	7403	0	6865	2	8852	2	8353	1	11146	0	10385
chr2	11124414	0	3	7407	1	6864	0	8859	2	8358	1	11148	1	10384
chr2	11124418	0	2	7385	6	6838	8	8817	6	8333	7	11115	7	10334
chr2	11124422	0	0	7418	0	6869	0	8880	2	8378	3	11173	0	10401
chr2	11124423	0	1	7418	2	6868	2	8878	3	8375	3	11176	2	10395
chr2	11124429	0	15	7409	6	6865	25	8861	4	8378	20	11184	5	10397
chr2	11124431	0	3	7421	9	6862	2	8888	17	8366	2	11209	11	10402
chr2	11124435	0	14	7413	23	6851	9	8879	31	8354	12	11203	38	10371
chr2	11124442	0	9	7393	7	6838	10	8847	10	8332	21	11167	13	10365
chr2	11124443	0	2	7417	4	6862	10	8875	8	8360	8	11202	12	10386
chr2	11124447	0	25	7246	51	6677	29	8712	71	8185	42	10975	98	10109
chr2	11124451	0	32	7383	15	6847	68	8807	30	8327	81	11123	37	10341
chr2	11124454	0	34	7408	5	6882	36	8887	8	8397	61	11189	6	10431
chr2	11124458	0	5	7436	20	6868	10	8920	32	8377	8	11252	38	10398
chr2	11124460	0	2	7442	52	6836	3	8928	85	8326	5	11257	88	10354
chr2	11124463	1	6734	711	5785	1103	7941	995	7122	1286	10019	1254	8730	1710
chr2	11124467	0	11	7404	18	6836	15	8886	23	8344	13	11188	32	10363
chr2	11124468	0	8	7447	8	6874	10	8911	7	8382	13	11234	16	10415
chr2	11124469	0	5	7458	9	6888	11	8932	13	8403	21	11257	12	10438
chr2	11124470	0	3	7462	12	6881	10	8936	16	8397	18	11257	22	10429
chr2	11124471	0	5	7446	10	6884	11	8935	13	8401	13	11261	9	10444
chr2	11124474	0	16	7435	12	6858	18	8905	24	8372	20	11233	22	10389
chr2	11124479	0	0	40	1	72	0	129	0	148	0	145	0	251
chr2	11124480	0	32	7412	20	6848	51	8870	23	8355	55	11193	39	10378
chr2	11124481	0	9	7461	10	6888	13	8938	4	8413	8	11276	18	10440
chr2	11124483	0	15	7455	10	6887	25	8925	12	8403	16	11268	11	10445
chr2	11124484	0	8	7460	8	6890	9	8941	14	8400	20	11264	11	10445
chr2	11124485	0	3	7461	17	6880	6	8945	18	8396	10	11276	23	10425
chr2	11124488	0	0	56	0	76	0	143	0	180	0	162	0	277
chr2	11124489	0	39	7425	10	6881	44	8892	13	8396	54	11218	13	10424
chr2	11124491	0	7	7465	11	6886	11	8942	19	8398	10	11280	23	10435
chr2	11124492	0	4	7465	7	6890	3	8951	7	8409	11	11281	2	10458
chr2	11124494	0	0	59	0	85	0	152	0	192	0	179	0	299
chr2	11124495	0	0	54	0	81	0	145	0	183	0	164	1	279
chr2	11124496	0	57	7327	41	6771	45	8819	39	8280	62	11139	51	10300
chr2	11124498	1	5010	2438	4423	2455	5919	3008	5399	2989	7433	3829	6735	3685
chr2	11124499	1	23	36	22	63	43	108	34	158	53	126	68	234
chr2	11124502	0	0	56	0	76	1	138	2	183	0	160	1	282
chr2	11124503	0	16	7434	15	6875	16	8927	11	8395	23	11242	18	10430

Number of C's and total read outs

spbs1		spoxbs1		spbs2		spoxbs2		spbs3		spoxbs3	
**C**	**Total**	**C**	**Total**	**C**	**Total**	**C**	**Total**	**C**	**Total**	**C**	**Total**
6	7389	10	6841	4	8823	4	8316	11	11091	7	10315
4	7407	0	6865	2	8854	2	8355	1	11147	0	10385
3	7410	1	6865	0	8859	2	8360	1	11149	1	10385
2	7387	6	6844	8	8825	6	8339	7	11122	7	10341
0	7418	0	6869	0	8880	2	8380	3	11176	0	10401
1	7419	2	6870	2	8880	3	8378	3	11179	2	10397
15	7424	6	6871	25	8886	4	8382	20	11204	5	10402
3	7424	9	6871	2	8890	17	8383	2	11211	11	10413
14	7427	23	6874	9	8888	31	8385	12	11215	38	10409
9	7402	7	6845	10	8857	10	8342	21	11188	13	10378
2	7419	4	6866	10	8885	8	8368	8	11210	12	10398
25	7271	51	6728	29	8741	71	8256	42	11017	98	10207
32	7415	15	6862	68	8875	30	8357	81	11204	37	10378
34	7442	5	6887	36	8923	8	8405	61	11250	6	10437
5	7441	20	6888	10	8930	32	8409	8	11260	38	10436
2	7444	52	6888	3	8931	85	8411	5	11262	88	10442
6734	7445	5785	6888	7941	8936	7122	8408	10019	11273	8730	10440
11	7415	18	6854	15	8901	23	8367	13	11201	32	10395
8	7455	8	6882	10	8921	7	8389	13	11247	16	10431
5	7463	9	6897	11	8943	13	8416	21	11278	12	10450
3	7465	12	6893	10	8946	16	8413	18	11275	22	10451
5	7451	10	6894	11	8946	13	8414	13	11274	9	10453
16	7451	12	6870	18	8923	24	8396	20	11253	22	10411
0	40	1	73	0	129	0	148	0	145	0	251
32	7444	20	6868	51	8921	23	8378	55	11248	39	10417
9	7470	10	6898	13	8951	4	8417	8	11284	18	10458
15	7470	10	6897	25	8950	12	8415	16	11284	11	10456
8	7468	8	6898	9	8950	14	8414	20	11284	11	10456
3	7464	17	6897	6	8951	18	8414	10	11286	23	10448
0	56	0	76	0	143	0	180	0	162	0	277
39	7464	10	6891	44	8936	13	8409	54	11272	13	10437
7	7472	11	6897	11	8953	19	8417	10	11290	23	10458
4	7469	7	6897	3	8954	7	8416	11	11292	2	10460
0	59	0	85	0	152	0	192	0	179	0	299
0	54	0	81	0	145	0	183	0	164	1	280
57	7384	41	6812	45	8864	39	8319	62	11201	51	10351
5010	7448	4423	6878	5919	8927	5399	8388	7433	11262	6735	10420
23	59	22	85	43	151	34	192	53	179	68	302
0	56	0	76	1	139	2	185	0	160	1	283
16	7450	15	6890	16	8943	11	8406	23	11265	18	10448

chr2	11124504	0	8	7461	7	6890	9	8942	4	8411	5	11275	8	10450
chr2	11124507	0	0	57	0	83	2	135	3	182	0	167	0	282
chr2	11124508	0	179	7280	17	6873	202	8737	11	8383	274	10989	29	10408
chr2	11124510	0	24	7437	14	6874	39	8906	10	8399	39	11237	15	10428
chr2	11124511	0	6	7459	10	6880	5	8946	7	8401	10	11269	5	10441
chr2	11124513	0	2	7464	3	6887	8	8940	7	8393	6	11275	1	10442
chr2	11124514	0	56	7410	5	6890	61	8887	5	8396	67	11210	5	10443
chr2	11124516	0	7	7456	10	6882	13	8934	11	8388	10	11261	11	10429
chr2	11124517	1	6947	522	5731	1163	8259	688	6984	1418	10352	917	8703	1736
chr2	11124518	1	46	13	42	39	104	44	87	102	139	38	132	168
chr2	11124519	0	8	7455	8	6883	20	8923	12	8387	17	11253	10	10424
chr2	11124520	0	31	7435	9	6886	51	8896	5	8396	37	11237	8	10433
chr2	11124522	0	14	7429	8	6868	6	8907	12	8367	17	11232	8	10393
chr2	11124523	1	7227	234	6246	644	8624	313	7636	754	10858	407	9415	1014
chr2	11124524	1	50	9	37	48	102	51	80	119	146	41	122	183
chr2	11124525	0	17	7454	15	6880	12	8927	19	8381	25	11241	26	10409
chr2	11124526	0	39	7399	8	6864	48	8869	6	8359	67	11166	11	10375
chr2	11124528	0	4	7465	6	6886	4	8936	8	8387	8	11255	6	10430
chr2	11124529	1	7007	460	5971	922	8316	622	7315	1079	10447	812	9076	1353
chr2	11124530	1	3	56	4	81	10	145	12	189	17	173	10	296
chr2	11124531	0	44	7411	12	6870	58	8869	17	8369	70	11170	19	10397
chr2	11124532	0	3	7465	7	6888	4	8934	6	8387	5	11252	10	10423
chr2	11124534	1	6431	1030	5578	1311	7636	1296	6916	1464	9551	1685	8531	1884
chr2	11124535	1	37	22	23	62	70	89	57	145	95	96	83	229
chr2	11124536	0	0	59	0	85	0	161	1	200	0	191	1	310
chr2	11124537	0	8	7414	16	6843	8	8888	11	8335	17	11167	11	10358
chr2	11124538	0	24	7432	7	6880	34	8896	8	8374	37	11196	6	10416
chr2	11124540	0	6	7446	69	6819	8	8923	93	8294	4	11224	110	10314
chr2	11124542	0	0	59	1	84	0	164	1	206	0	191	0	310
chr2	11124543	0	13	7438	15	6872	19	8894	11	8365	20	11199	21	10391
chr2	11124544	0	5	7447	7	6883	4	8915	5	8377	9	11208	7	10408
chr2	11124546	1	5584	1851	5620	1251	6474	2433	6999	1369	8099	3099	8647	1745
chr2	11124547	1	29	30	18	67	66	99	40	167	74	118	62	250
chr2	11124548	0	0	59	2	83	0	165	3	203	1	191	5	308
chr2	11124549	0	23	7405	22	6852	22	8870	22	8334	32	11152	22	10357
chr2	11124550	0	11	7427	9	6876	22	8873	11	8353	29	11161	3	10390
chr2	11124552	0	12	7405	14	6835	10	8846	14	8311	16	11138	15	10352
chr2	11124553	1	6259	1083	5596	1211	7319	1453	6823	1426	9144	1866	8506	1771
chr2	11124554	1	29	30	17	68	66	99	44	161	87	106	71	237
chr2	11124555	0	10	7413	9	6870	15	8855	13	8326	14	11136	17	10361
chr2	11124556	0	22	7392	10	6853	31	8822	22	8300	37	11097	20	10346
chr2	11124558	0	9	7334	5	6808	11	8754	12	8250	12	11054	11	10308
chr2	11124559	1	5013	2372	4694	2160	5838	2973	5796	2504	7306	3779	7235	3092
chr2	11124560	1	28	31	14	71	60	105	34	173	69	124	68	245
chr2	11124561	0	0	59	0	84	0	165	0	207	0	193	1	312
chr2	11124562	0	0	58	0	84	0	164	1	206	0	192	0	312

8	7469	7	6897	9	8951	4	8415	5	11280	8	10458
0	57	0	83	2	137	3	185	0	167	0	282
179	7459	17	6890	202	8939	11	8394	274	11263	29	10437
24	7461	14	6888	39	8945	10	8409	39	11276	15	10443
6	7465	10	6890	5	8951	7	8408	10	11279	5	10446
2	7466	3	6890	8	8948	7	8400	6	11281	1	10443
56	7466	5	6895	61	8948	5	8401	67	11277	5	10448
7	7463	10	6892	13	8947	11	8399	10	11271	11	10440
6947	7469	5731	6894	8259	8947	6984	8402	10352	11269	8703	10439
46	59	42	81	104	148	87	189	139	177	132	300
8	7463	8	6891	20	8943	12	8399	17	11270	10	10434
31	7466	9	6895	51	8947	5	8401	37	11274	8	10441
14	7443	8	6876	6	8913	12	8379	17	11249	8	10401
7227	7461	6246	6890	8624	8937	7636	8390	10858	11265	9415	10429
50	59	37	85	102	153	80	199	146	187	122	305
17	7471	15	6895	12	8939	19	8400	25	11266	26	10435
39	7438	8	6872	48	8917	6	8365	67	11233	11	10386
4	7469	6	6892	4	8940	8	8395	8	11263	6	10436
7007	7467	5971	6893	8316	8938	7315	8394	10447	11259	9076	10429
3	59	4	85	10	155	12	201	17	190	10	306
44	7455	12	6882	58	8927	17	8386	70	11240	19	10416
3	7468	7	6895	4	8938	6	8393	5	11257	10	10433
6431	7461	5578	6889	7636	8932	6916	8380	9551	11236	8531	10415
37	59	23	85	70	159	57	202	95	191	83	312
0	59	0	85	0	161	1	201	0	191	1	311
8	7422	16	6859	8	8896	11	8346	17	11184	11	10369
24	7456	7	6887	34	8930	8	8382	37	11233	6	10422
6	7452	69	6888	8	8931	93	8387	4	11228	110	10424
0	59	1	85	0	164	1	207	0	191	0	310
13	7451	15	6887	19	8913	11	8376	20	11219	21	10412
5	7452	7	6890	4	8919	5	8382	9	11217	7	10415
5584	7435	5620	6871	6474	8907	6999	8368	8099	11198	8647	10392
29	59	18	85	66	165	40	207	74	192	62	312
0	59	2	85	0	165	3	206	1	192	5	313
23	7428	22	6874	22	8892	22	8356	32	11184	22	10379
11	7438	9	6885	22	8895	11	8364	29	11190	3	10393
12	7417	14	6849	10	8856	14	8325	16	11154	15	10367
6259	7342	5596	6807	7319	8772	6823	8249	9144	11010	8506	10277
29	59	17	85	66	165	44	205	87	193	71	308
10	7423	9	6879	15	8870	13	8339	14	11150	17	10378
22	7414	10	6863	31	8853	22	8322	37	11134	20	10366
9	7343	5	6813	11	8765	12	8262	12	11066	11	10319
5013	7385	4694	6854	5838	8811	5796	8300	7306	11085	7235	10327
28	59	14	85	60	165	34	207	69	193	68	313
0	59	0	84	0	165	0	207	0	193	1	313
0	58	0	84	0	164	1	207	0	192	0	312

chr2	11124563	0	0	7346	4	6784	0	8723	1	8215	1	10962	1	10220
chr2	11124567	0	0	58	0	85	0	165	1	207	0	191	0	313
chr2	11124572	0	0	58	0	85	1	164	0	208	0	193	0	312
chr2	11124576	0	0	7181	1	6633	2	8403	0	7960	0	10587	1	9897
chr2	11124581	0	1	7153	2	6578	1	8320	0	7854	2	10450	4	9766
chr2	11124584	0	0	58	0	84	0	165	0	208	1	190	0	312
chr2	11124586	0	4	7010	5	6422	8	8016	9	7620	4	10107	14	9394
chr2	11124588	0	0	58	0	84	0	165	0	208	0	192	0	310
chr2	11124593	0	0	58	0	84	0	165	0	208	0	192	0	311
chr2	11124597	0	0	58	0	84	0	165	0	208	0	192	0	311
chr2	11124598	0	0	58	0	84	0	165	0	208	0	192	0	311
chr2	11124600	0	0	57	0	83	0	163	0	206	0	192	0	310
chr2	11124609	0	0	55	0	82	0	161	0	199	0	182	0	295
chr3	89184744	0	2	2307	5	1964	1	3195	1	1940	1	3737	0	2340
chr3	89184752	0	1	3267	1	2472	1	1186	2	797	3	1375	3	1006
chr3	89184753	0	3	2390	0	2018	0	3331	2	2035	0	3905	1	2455
chr3	89184754	0	1	2398	0	2025	0	3342	0	2048	0	3921	0	2467
chr3	89184756	0	0	2384	0	2012	0	3321	0	2042	0	3894	0	2451
chr3	89184758	0	3	2994	0	2295	4	2294	1	1834	1	2607	8	2183
chr3	89184759	0	2	3356	0	2527	2	2324	3	1839	0	2611	0	2190
chr3	89184760	0	3	3344	8	2524	1	2417	2	1917	2	2739	4	2306
chr3	89184762	0	0	2401	0	2025	1	3348	0	2054	1	3922	0	2470
chr3	89184763	0	0	2395	1	2020	4	3338	2	2053	4	3908	3	2462
chr3	89184765	0	1	3372	0	2543	2	2450	1	1958	0	2790	3	2331
chr3	89184768	0	0	3372	1	2541	0	2459	1	1962	0	2798	2	2336
chr3	89184769	0	1	3374	0	2545	2	2457	2	1958	2	2797	0	2340
chr3	89184773	0	0	3376	0	2550	2	2456	0	1966	1	2796	2	2339
chr3	89184774	0	5	2407	2	2029	8	3358	1	2065	9	3929	7	2472
chr3	89184776	1	1894	520	1378	654	2692	675	1429	639	3114	824	1740	740
chr3	89184777	1	2524	845	1817	720	1781	679	1423	543	2068	733	1703	637
chr3	89184778	0	4	3358	1	2537	3	2442	2	1948	4	2784	6	2324
chr3	89184779	0	23	3360	4	2543	15	2442	6	1957	21	2778	3	2338
chr3	89184780	0	8	2367	6	1997	12	3319	3	2036	12	3886	5	2454
chr3	89184781	0	4	2409	7	2028	6	3366	4	2066	8	3937	4	2477
chr3	89184782	0	6	2400	5	2017	10	3344	7	2056	16	3911	2	2455
chr3	89184783	0	19	2351	7	1993	22	3294	4	2036	21	3837	10	2432
chr3	89184787	0	2	3393	0	2556	1	2472	2	1969	3	2811	2	2341
chr3	89184788	0	3	3299	4	2471	1	2439	2	1945	1	2749	4	2283
chr3	89184792	0	12	3266	9	2435	5	2386	6	1887	4	2702	4	2241
chr3	89184795	0	40	3359	2	2553	27	2451	3	1968	31	2780	4	2349
chr3	89184800	0	4	3399	3	2559	4	2476	1	1974	4	2812	5	2351
chr3	89184801	0	6	2408	1	2029	7	3365	3	2063	6	3943	3	2479
chr3	89184805	0	0	2421	1	2036	1	3382	0	2073	1	3960	1	2492
chr3	89184806	1	2073	348	1593	444	2950	432	1677	397	3412	550	2015	476
chr3	89184807	1	3056	357	2092	473	2201	281	1612	360	2486	344	1942	416
chr3	89184808	0	9	3399	6	2551	13	2458	5	1964	13	2808	4	2345

0	7346	4	6788	0	8723	1	8216	1	10963	1	10221
0	58	0	85	0	165	1	208	0	191	0	313
0	58	0	85	1	165	0	208	0	193	0	312
0	7181	1	6634	2	8405	0	7960	0	10587	1	9898
1	7154	2	6580	1	8321	0	7854	2	10452	4	9770
0	58	0	84	0	165	0	208	1	191	0	312
4	7014	5	6427	8	8024	9	7629	4	10111	14	9408
0	58	0	84	0	165	0	208	0	192	0	310
0	58	0	84	0	165	0	208	0	192	0	311
0	58	0	84	0	165	0	208	0	192	0	311
0	58	0	84	0	165	0	208	0	192	0	311
0	57	0	83	0	163	0	206	0	192	0	310
0	55	0	82	0	161	0	199	0	182	0	295
2	2309	5	1969	1	3196	1	1941	1	3738	0	2340
1	3268	1	2473	1	1187	2	799	3	1378	3	1009
3	2393	0	2018	0	3331	2	2037	0	3905	1	2456
1	2399	0	2025	0	3342	0	2048	0	3921	0	2467
0	2384	0	2012	0	3321	0	2042	0	3894	0	2451
3	2997	0	2295	4	2298	1	1835	1	2608	8	2191
2	3358	0	2527	2	2326	3	1842	0	2611	0	2190
3	3347	8	2532	1	2418	2	1919	2	2741	4	2310
0	2401	0	2025	1	3349	0	2054	1	3923	0	2470
0	2395	1	2021	4	3342	2	2055	4	3912	3	2465
1	3373	0	2543	2	2452	1	1959	0	2790	3	2334
0	3372	1	2542	0	2459	1	1963	0	2798	2	2338
1	3375	0	2545	2	2459	2	1960	2	2799	0	2340
0	3376	0	2550	2	2458	0	1966	1	2797	2	2341
5	2412	2	2031	8	3366	1	2066	9	3938	7	2479
1894	2414	1378	2032	2692	3367	1429	2068	3114	3938	1740	2480
2524	3369	1817	2537	1781	2460	1423	1966	2068	2801	1703	2340
4	3362	1	2538	3	2445	2	1950	4	2788	6	2330
23	3383	4	2547	15	2457	6	1963	21	2799	3	2341
8	2375	6	2003	12	3331	3	2039	12	3898	5	2459
4	2413	7	2035	6	3372	4	2070	8	3945	4	2481
6	2406	5	2022	10	3354	7	2063	16	3927	2	2457
19	2370	7	2000	22	3316	4	2040	21	3858	10	2442
2	3395	0	2556	1	2473	2	1971	3	2814	2	2343
3	3302	4	2475	1	2440	2	1947	1	2750	4	2287
12	3278	9	2444	5	2391	6	1893	4	2706	4	2245
40	3399	2	2555	27	2478	3	1971	31	2811	4	2353
4	3403	3	2562	4	2480	1	1975	4	2816	5	2356
6	2414	1	2030	7	3372	3	2066	6	3949	3	2482
0	2421	1	2037	1	3383	0	2073	1	3961	1	2493
2073	2421	1593	2037	2950	3382	1677	2074	3412	3962	2015	2491
3056	3413	2092	2565	2201	2482	1612	1972	2486	2830	1942	2358
9	3408	6	2557	13	2471	5	1969	13	2821	4	2349

chr3	89184809	0	13	3407	15	2552	10	2473	8	1966	8	2825	4	2355
chr3	89184810	0	17	3401	5	2562	26	2461	4	1973	20	2811	3	2359
chr3	89184814	0	4	3397	4	2550	4	2472	6	1960	4	2819	2	2361
chr3	89184815	1	2135	286	1744	296	3026	358	1820	254	3529	438	2182	316
chr3	89184816	1	3220	203	2306	261	2316	172	1802	173	2634	200	2164	203
chr3	89184817	0	4	3420	7	2562	6	2481	7	1969	10	2828	9	2361
chr3	89184818	0	5	2414	4	2033	5	3376	4	2068	8	3957	8	2486
chr3	89184820	0	24	3351	4	2524	11	2471	4	1973	14	2803	4	2354
chr3	89184821	1	1921	501	1601	439	2730	658	1716	361	3234	736	2039	459
chr3	89184822	1	2975	440	2110	459	2143	326	1618	345	2452	376	1958	399
chr3	89184824	1	1982	443	1432	610	2828	560	1530	550	3301	672	1832	669
chr3	89184825	1	2837	526	1934	563	2039	419	1547	410	2310	491	1876	460
chr3	89184826	0	3	3254	2	2447	9	2285	6	1814	8	2614	1	2184
chr3	89184827	0	7	3137	4	2381	3	2332	1	1831	13	2642	3	2198
chr3	89184828	0	5	2368	0	2000	4	3313	4	2042	9	3881	2	2461
chr3	89184831	0	1	2425	2	2037	1	3394	1	2085	5	3975	3	2501
chr3	89184833	0	5	3416	5	2562	4	2460	4	1951	5	2787	5	2329
chr3	89184834	0	19	3338	1	2503	30	2425	2	1954	28	2767	4	2344
chr3	89184836	1	1952	474	1583	459	2788	607	1673	416	3315	665	2012	492
chr3	89184837	1	2933	495	1979	596	2130	360	1534	445	2433	404	1865	505
chr3	89184839	0	5	3413	3	2553	5	2484	2	1973	3	2827	1	2362
chr3	89184840	0	5	3422	5	2570	1	2487	5	1973	9	2833	2	2368
chr3	89184842	0	3	3246	6	2448	4	2428	2	1931	3	2778	7	2315
chr3	89184843	0	4	3388	3	2542	7	2415	2	1898	9	2716	4	2269
chr3	89184844	1	2212	212	1740	299	3134	263	1855	234	3677	302	2220	286
chr3	89184845	1	3183	245	2306	272	2296	216	1793	199	2627	247	2150	235
chr3	89184846	0	6	3405	6	2555	5	2494	6	1977	8	2855	4	2372
chr3	89184848	0	3	3424	2	2573	19	2478	17	1958	29	2825	11	2346
chr3	89184849	0	15	3411	7	2569	15	2492	3	1986	17	2858	5	2370
chr3	89184850	1	2105	318	1635	403	3012	384	1754	329	3513	460	2154	353
chr3	89184851	1	3167	262	2253	322	2290	219	1770	219	2675	200	2123	261
chr3	89184852	0	7	3419	5	2567	6	2502	7	1984	6	2870	7	2374
chr3	89184853	1	2175	251	1762	278	3080	317	1881	210	3615	367	2267	242
chr3	89184854	1	3246	180	2234	338	2367	145	1744	247	2738	138	2088	294
chr3	89184855	0	24	3177	3	2426	24	2385	4	1932	32	2753	4	2293
chr3	89184856	1	2155	260	1739	292	3091	298	1868	211	3581	384	2242	259
chr3	89184857	1	3196	234	2191	385	2334	176	1741	251	2694	185	2065	313
chr3	89184858	0	34	3398	4	2574	19	2491	4	1991	29	2853	4	2380
chr3	89184861	0	1	3429	37	2537	2	2501	36	1954	2	2871	40	2339
chr3	89184862	0	8	3418	3	2574	5	2501	5	1988	5	2872	5	2374
chr3	89184864	0	5	3427	5	2575	2	2507	2	1993	4	2880	0	2383
chr3	89184865	0	7	3425	5	2575	6	2505	12	1984	6	2881	7	2376
chr3	89184866	0	1	3431	3	2577	3	2509	2	1995	6	2881	3	2380
chr3	89184867	0	11	3410	3	2572	3	2507	3	1987	5	2871	4	2371
chr3	89184868	0	14	2312	8	1964	26	3231	20	2006	35	3803	12	2413
chr3	89184870	0	3	2433	2	2040	8	3401	3	2104	6	3982	3	2517

13	3420	15	2567	10	2483	8	1974	8	2833	4	2359
17	3418	5	2567	26	2487	4	1977	20	2831	3	2362
4	3401	4	2554	4	2476	6	1966	4	2823	2	2363
2135	2421	1744	2040	3026	3384	1820	2074	3529	3967	2182	2498
3220	3423	2306	2567	2316	2488	1802	1975	2634	2834	2164	2367
4	3424	7	2569	6	2487	7	1976	10	2838	9	2370
5	2419	4	2037	5	3381	4	2072	8	3965	8	2494
24	3375	4	2528	11	2482	4	1977	14	2817	4	2358
1921	2422	1601	2040	2730	3388	1716	2077	3234	3970	2039	2498
2975	3415	2110	2569	2143	2469	1618	1963	2452	2828	1958	2357
1982	2425	1432	2042	2828	3388	1530	2080	3301	3973	1832	2501
2837	3363	1934	2497	2039	2458	1547	1957	2310	2801	1876	2336
3	3257	2	2449	9	2294	6	1820	8	2622	1	2185
7	3144	4	2385	3	2335	1	1832	13	2655	3	2201
5	2373	0	2000	4	3317	4	2046	9	3890	2	2463
1	2426	2	2039	1	3395	1	2086	5	3980	3	2504
5	3421	5	2567	4	2464	4	1955	5	2792	5	2334
19	3357	1	2504	30	2455	2	1956	28	2795	4	2348
1952	2426	1583	2042	2788	3395	1673	2089	3315	3980	2012	2504
2933	3428	1979	2575	2130	2490	1534	1979	2433	2837	1865	2370
5	3418	3	2556	5	2489	2	1975	3	2830	1	2363
5	3427	5	2575	1	2488	5	1978	9	2842	2	2370
3	3249	6	2454	4	2432	2	1933	3	2781	7	2322
4	3392	3	2545	7	2422	2	1900	9	2725	4	2273
2212	2424	1740	2039	3134	3397	1855	2089	3677	3979	2220	2506
3183	3428	2306	2578	2296	2512	1793	1992	2627	2874	2150	2385
6	3411	6	2561	5	2499	6	1983	8	2863	4	2376
3	3427	2	2575	19	2497	17	1975	29	2854	11	2357
15	3426	7	2576	15	2507	3	1989	17	2875	5	2375
2105	2423	1635	2038	3012	3396	1754	2083	3513	3973	2154	2507
3167	3429	2253	2575	2290	2509	1770	1989	2675	2875	2123	2384
7	3426	5	2572	6	2508	7	1991	6	2876	7	2381
2175	2426	1762	2040	3080	3397	1881	2091	3615	3982	2267	2509
3246	3426	2234	2572	2367	2512	1744	1991	2738	2876	2088	2382
24	3201	3	2429	24	2409	4	1936	32	2785	4	2297
2155	2415	1739	2031	3091	3389	1868	2079	3581	3965	2242	2501
3196	3430	2191	2576	2334	2510	1741	1992	2694	2879	2065	2378
34	3432	4	2578	19	2510	4	1995	29	2882	4	2384
1	3430	37	2574	2	2503	36	1990	2	2873	40	2379
8	3426	3	2577	5	2506	5	1993	5	2877	5	2379
5	3432	5	2580	2	2509	2	1995	4	2884	0	2383
7	3432	5	2580	6	2511	12	1996	6	2887	7	2383
1	3432	3	2580	3	2512	2	1997	6	2887	3	2383
11	3421	3	2575	3	2510	3	1990	5	2876	4	2375
14	2326	8	1972	26	3257	20	2026	35	3838	12	2425
3	2436	2	2042	8	3409	3	2107	6	3988	3	2520

chr3	89184871	0	4	2434	5	2037	8	3401	2	2105	7	3987	3	2523
chr3	89184873	0	1	3422	0	2577	3	2502	0	1996	2	2879	4	2373
chr3	89184875	0	24	3395	41	2527	17	2475	23	1961	30	2841	28	2340
chr3	89184876	0	2	3423	4	2573	1	2504	2	1994	2	2877	5	2376
chr3	89184878	0	7	3417	7	2573	6	2501	2	1993	11	2869	3	2378
chr3	89184879	0	3	3422	4	2576	3	2503	4	1993	2	2875	2	2380
chr3	89184880	0	7	3409	6	2566	8	2495	5	1987	3	2870	5	2373
chr3	89184881	0	7	2318	8	1952	7	3228	3	2018	8	3764	6	2404
chr3	89184883	0	4	3406	1	2567	0	2503	0	1982	2	2864	1	2369
chr3	89184885	0	4	2439	2	2042	3	3413	4	2116	3	3994	2	2528
chr3	89184888	0	6	2439	19	2026	8	3407	25	2096	7	3983	28	2504
chr3	89184890	0	17	2430	1	2043	31	3388	5	2117	23	3972	1	2532
chr3	89184891	1	1319	1115	1265	770	1862	1546	1418	693	2239	1740	1715	810
chr3	89184892	1	1944	1476	1361	1212	1461	1029	1064	923	1696	1163	1256	1112
chr3	89184895	0	4	3415	2	2570	1	2482	1	1983	2	2855	1	2365
chr3	89184896	0	3	3416	2	2569	4	2478	1	1983	0	2854	4	2362
chr3	89184897	0	3	3414	12	2559	9	2474	10	1973	3	2851	6	2359
chr3	89184898	0	3	2430	0	2036	3	3395	1	2110	4	3988	3	2524
chr3	89184902	0	8	2434	4	2033	4	3401	5	2097	16	3967	3	2518
chr3	89184903	0	0	2430	3	2029	4	3386	5	2092	1	3944	3	2499
chr3	89184905	0	3	3411	0	2562	1	2473	1	1974	0	2838	1	2355
chr3	89184907	0	1	3416	23	2542	2	2470	19	1955	2	2835	19	2340
chr3	89184908	0	5	3407	4	2554	6	2464	2	1967	6	2827	5	2351
chr3	89184911	0	0	3405	2	2554	1	2463	1	1968	2	2820	3	2342
chr3	89184913	0	4	3399	3	2557	5	2461	3	1967	11	2816	4	2348
chr3	89184914	0	10	3331	6	2511	5	2424	5	1944	5	2768	9	2314
chr3	89184915	0	7	2409	1	2021	8	3380	7	2085	6	3975	7	2520
chr3	89184917	0	42	3363	6	2550	46	2418	10	1958	46	2774	18	2328
chr3	89184919	0	4	3402	1	2555	7	2456	3	1964	3	2818	5	2337
chr3	89184920	0	9	3396	4	2553	3	2460	2	1964	5	2814	6	2336
chr3	89184922	0	4	3401	0	2555	2	2458	4	1961	3	2817	4	2340
chr3	89184923	0	2	3405	1	2554	3	2457	3	1962	1	2818	1	2342
chr3	89184925	0	77	3328	135	2420	49	2409	85	1880	79	2741	120	2222
chr3	89184926	0	8	3397	4	2550	6	2451	1	1962	6	2811	3	2340
chr3	89184927	0	3	2440	5	2034	2	3416	4	2118	7	3999	9	2530
chr3	89184929	0	4	3398	29	2524	6	2451	28	1937	4	2812	26	2318
chr3	89184931	0	8	3395	3	2548	6	2449	0	1962	7	2808	2	2342
chr3	89184932	0	2	3399	2	2549	5	2451	1	1961	6	2809	2	2340
chr3	89184933	0	11	2400	2	2009	11	3326	4	2073	33	3906	2	2493
chr3	89184938	0	13	3369	3	2536	7	2432	4	1949	15	2790	4	2322
chr3	89184939	0	3	2396	5	2005	1	3350	2	2076	0	3933	8	2486
chr3	89184940	0	28	2376	2	2002	28	3308	1	2077	38	3876	5	2476
chr3	89184942	0	8	2345	3	1967	3	3344	2	2100	4	3914	2	2489
chr3	89184943	0	1	2425	2	2025	3	3380	4	2112	3	3955	2	2499
chr3	89184945	0	2	3398	5	2543	2	2444	1	1959	2	2802	4	2335
chr3	89184946	0	7	3392	6	2541	2	2439	2	1955	3	2801	3	2333

4	2438	5	2042	8	3409	2	2107	7	3994	3	2526
1	3423	0	2577	3	2505	0	1996	2	2881	4	2377
24	3419	41	2568	17	2492	23	1984	30	2871	28	2368
2	3425	4	2577	1	2505	2	1996	2	2879	5	2381
7	3424	7	2580	6	2507	2	1995	11	2880	3	2381
3	3425	4	2580	3	2506	4	1997	2	2877	2	2382
7	3416	6	2572	8	2503	5	1992	3	2873	5	2378
7	2325	8	1960	7	3235	3	2021	8	3772	6	2410
4	3410	1	2568	0	2503	0	1982	2	2866	1	2370
4	2443	2	2044	3	3416	4	2120	3	3997	2	2530
6	2445	19	2045	8	3415	25	2121	7	3990	28	2532
17	2447	1	2044	31	3419	5	2122	23	3995	1	2533
1319	2434	1265	2035	1862	3408	1418	2111	2239	3979	1715	2525
1944	3420	1361	2573	1461	2490	1064	1987	1696	2859	1256	2368
4	3419	2	2572	1	2483	1	1984	2	2857	1	2366
3	3419	2	2571	4	2482	1	1984	0	2854	4	2366
3	3417	12	2571	9	2483	10	1983	3	2854	6	2365
3	2433	0	2036	3	3398	1	2111	4	3992	3	2527
8	2442	4	2037	4	3405	5	2102	16	3983	3	2521
0	2430	3	2032	4	3390	5	2097	1	3945	3	2502
3	3414	0	2562	1	2474	1	1975	0	2838	1	2356
1	3417	23	2565	2	2472	19	1974	2	2837	19	2359
5	3412	4	2558	6	2470	2	1969	6	2833	5	2356
0	3405	2	2556	1	2464	1	1969	2	2822	3	2345
4	3403	3	2560	5	2466	3	1970	11	2827	4	2352
10	3341	6	2517	5	2429	5	1949	5	2773	9	2323
7	2416	1	2022	8	3388	7	2092	6	3981	7	2527
42	3405	6	2556	46	2464	10	1968	46	2820	18	2346
4	3406	1	2556	7	2463	3	1967	3	2821	5	2342
9	3405	4	2557	3	2463	2	1966	5	2819	6	2342
4	3405	0	2555	2	2460	4	1965	3	2820	4	2344
2	3407	1	2555	3	2460	3	1965	1	2819	1	2343
77	3405	135	2555	49	2458	85	1965	79	2820	120	2342
8	3405	4	2554	6	2457	1	1963	6	2817	3	2343
3	2443	5	2039	2	3418	4	2122	7	4006	9	2539
4	3402	29	2553	6	2457	28	1965	4	2816	26	2344
8	3403	3	2551	6	2455	0	1962	7	2815	2	2344
2	3401	2	2551	5	2456	1	1962	6	2815	2	2342
11	2411	2	2011	11	3337	4	2077	33	3939	2	2495
13	3382	3	2539	7	2439	4	1953	15	2805	4	2326
3	2399	5	2010	1	3351	2	2078	0	3933	8	2494
28	2404	2	2004	28	3336	1	2078	38	3914	5	2481
8	2353	3	1970	3	3347	2	2102	4	3918	2	2491
1	2426	2	2027	3	3383	4	2116	3	3958	2	2501
2	3400	5	2548	2	2446	1	1960	2	2804	4	2339
7	3399	6	2547	2	2441	2	1957	3	2804	3	2336

chr3	89184949	0	3	2286	0	1862	2	3221	0	2013	4	3808	0	2422
chr3	89184951	0	25	3370	0	2543	21	2418	0	1952	29	2762	3	2329
chr3	89184953	1	2030	410	1643	394	2837	569	1787	341	3339	655	2171	362
chr3	89184954	1	3018	376	1982	561	2104	332	1533	414	2456	331	1809	519
chr3	89184955	0	2	3392	37	2507	3	2434	19	1928	1	2787	27	2303
chr3	89184956	0	6	3386	2	2541	5	2430	2	1946	5	2781	3	2326
chr3	89184957	0	3	2435	9	2024	9	3388	5	2114	7	3980	5	2524
chr3	89184959	1	2170	262	1599	419	3006	373	1697	412	3585	376	2057	467
chr3	89184960	1	3068	311	2158	376	2176	253	1649	288	2524	255	1975	347
chr3	89184961	0	7	2433	3	2028	5	3398	3	2116	5	3986	4	2526
chr3	89184963	0	0	2435	4	2012	4	3389	1	2117	7	3981	3	2521
chr3	89184965	0	21	2419	2	2026	24	3382	4	2118	33	3952	4	2520
chr3	89184967	0	2	3364	4	2522	6	2414	5	1932	6	2762	10	2301
chr3	89184969	0	3	2432	1	2021	4	3391	2	2114	3	3961	0	2516
chr3	89184972	0	8	2424	8	2014	13	3375	12	2106	13	3969	5	2512
chr3	89184973	0	3	2423	3	2011	5	3381	1	2104	6	3967	3	2509
chr3	89184975	0	0	2424	2	2016	2	3379	7	2099	3	3951	2	2498
chr3	89184978	1	2208	219	1805	212	3115	273	1917	200	3641	338	2262	253
chr3	89184979	1	3006	376	2215	320	2120	298	1702	236	2456	324	2005	309
chr3	89184980	0	4	3378	3	2531	0	2418	1	1937	2	2777	3	2312
chr3	89184981	0	23	3358	8	2527	16	2403	5	1930	20	2758	7	2309
chr3	89184982	0	4	3356	3	2521	4	2400	2	1925	4	2755	6	2293
chr3	89184983	0	4	2418	2	2014	9	3377	3	2107	8	3960	6	2506
chr3	89184985	0	0	3375	3	2530	5	2411	1	1936	2	2772	0	2311
chr3	89184986	0	7	3365	0	2523	5	2411	1	1933	2	2767	2	2307
chr3	89184987	0	1	3378	2	2531	0	2418	1	1935	1	2775	0	2314
chr3	89184988	0	1	3374	0	2530	0	2414	0	1934	1	2773	0	2310
chr3	89184989	0	0	3302	1	2470	0	2391	2	1905	2	2719	0	2279
chr3	89184990	0	3	2399	3	1999	5	3356	2	2093	7	3932	3	2487
chr3	89184993	0	3	2392	1	2003	1	3369	0	2103	1	3945	1	2499
chr3	89184994	0	2	2393	2	2000	3	3364	0	2107	3	3938	5	2498
chr3	89184996	0	3	3369	2	2529	6	2401	1	1930	3	2763	4	2306
chr3	89184997	0	6	2257	3	1925	1	3288	1	2071	0	3821	0	2457
chr3	89184998	0	2	2261	0	1921	1	3280	0	2067	2	3815	1	2443
chr3	89184999	0	0	2254	1	1917	2	3280	0	2065	2	3806	0	2454
chr3	89185001	0	0	2251	1	1913	1	3263	2	2057	1	3799	2	2448
chr3	89185003	0	5	3231	1	2440	4	2332	3	1877	3	2668	0	2228
chr3	89185004	0	5	2239	0	1917	6	3262	4	2066	1	3786	5	2441
chr3	89185005	0	1	2243	2	1902	1	3242	1	2043	2	3752	2	2427
chr3	89185008	0	8	2755	3	2075	6	1741	3	1375	4	1952	3	1623
chr3	89185015	0	1	1818	0	1570	0	2601	1	1600	4	2976	0	1937
chr3	89185017	0	1	1766	0	1547	2	2513	0	1537	1	2853	1	1882
chr3	89185018	0	2	1658	1	1440	1	2420	0	1515	1	2714	3	1829
chr3	89185022	0	1	1733	0	1510	1	2482	0	1526	1	2854	0	1847
chr3	89679038	0	1	3722	0	1713	2	4573	2	985	1	3053	2	1138
chr3	89679041	0	0	3912	0	1809	2	4954	0	1100	5	3294	1	1226

3	2289	0	1862	2	3223	0	2013	4	3812	0	2422
25	3395	0	2543	21	2439	0	1952	29	2791	3	2332
2030	2440	1643	2037	2837	3406	1787	2128	3339	3994	2171	2533
3018	3394	1982	2543	2104	2436	1533	1947	2456	2787	1809	2328
2	3394	37	2544	3	2437	19	1947	1	2788	27	2330
6	3392	2	2543	5	2435	2	1948	5	2786	3	2329
3	2438	9	2033	9	3397	5	2119	7	3987	5	2529
2170	2432	1599	2018	3006	3379	1697	2109	3585	3961	2057	2524
3068	3379	2158	2534	2176	2429	1649	1937	2524	2779	1975	2322
7	2440	3	2031	5	3403	3	2119	5	3991	4	2530
0	2435	4	2016	4	3393	1	2118	7	3988	3	2524
21	2440	2	2028	24	3406	4	2122	33	3985	4	2524
2	3366	4	2526	6	2420	5	1937	6	2768	10	2311
3	2435	1	2022	4	3395	2	2116	3	3964	0	2516
8	2432	8	2022	13	3388	12	2118	13	3982	5	2517
3	2426	3	2014	5	3386	1	2105	6	3973	3	2512
0	2424	2	2018	2	3381	7	2106	3	3954	2	2500
2208	2427	1805	2017	3115	3388	1917	2117	3641	3979	2262	2515
3006	3382	2215	2535	2120	2418	1702	1938	2456	2780	2005	2314
4	3382	3	2534	0	2418	1	1938	2	2779	3	2315
23	3381	8	2535	16	2419	5	1935	20	2778	7	2316
4	3360	3	2524	4	2404	2	1927	4	2759	6	2299
4	2422	2	2016	9	3386	3	2110	8	3968	6	2512
0	3375	3	2533	5	2416	1	1937	2	2774	0	2311
7	3372	0	2523	5	2416	1	1934	2	2769	2	2309
1	3379	2	2533	0	2418	1	1936	1	2776	0	2314
1	3375	0	2530	0	2414	0	1934	1	2774	0	2310
0	3302	1	2471	0	2391	2	1907	2	2721	0	2279
3	2402	3	2002	5	3361	2	2095	7	3939	3	2490
3	2395	1	2004	1	3370	0	2103	1	3946	1	2500
2	2395	2	2002	3	3367	0	2107	3	3941	5	2503
3	3372	2	2531	6	2407	1	1931	3	2766	4	2310
6	2263	3	1928	1	3289	1	2072	0	3821	0	2457
2	2263	0	1921	1	3281	0	2067	2	3817	1	2444
0	2254	1	1918	2	3282	0	2065	2	3808	0	2454
0	2251	1	1914	1	3264	2	2059	1	3800	2	2450
5	3236	1	2441	4	2336	3	1880	3	2671	0	2228
5	2244	0	1917	6	3268	4	2070	1	3787	5	2446
1	2244	2	1904	1	3243	1	2044	2	3754	2	2429
8	2763	3	2078	6	1747	3	1378	4	1956	3	1626
1	1819	0	1570	0	2601	1	1601	4	2980	0	1937
1	1767	0	1547	2	2515	0	1537	1	2854	1	1883
2	1660	1	1441	1	2421	0	1515	1	2715	3	1832
1	1734	0	1510	1	2483	0	1526	1	2855	0	1847
1	3723	0	1713	2	4575	2	987	1	3054	2	1140
0	3912	0	1809	2	4956	0	1100	5	3299	1	1227

chr3	89679051	0	0	4075	0	1883	4	5261	0	1246	2	3650	0	1386
chr3	89679054	0	4	4177	1	1916	3	5416	0	1291	1	3820	1	1447
chr3	89679072	1	4625	616	1858	481	6065	1061	1466	353	5047	778	1758	376
chr3	89679075	0	19	5164	12	2297	16	7054	9	1802	13	5762	12	2101
chr3	89679079	0	29	5254	3	2358	40	7201	2	1850	19	5936	4	2178
chr3	89679081	0	30	5243	7	2346	31	7201	5	1851	27	5939	1	2181
chr3	89679091	0	4	5304	1	2369	9	7274	3	1872	6	6019	5	2192
chr3	89679103	0	3	5379	2	2394	7	7356	4	1906	12	6089	3	2236
chr3	89679107	1	4096	1304	1732	669	5196	2187	1348	574	4480	1654	1594	652
chr3	89679108	0	10	5391	4	2397	15	7368	1	1923	12	6124	2	2248
chr3	89679109	0	3	5400	3	2398	6	7378	2	1922	7	6128	2	2248
chr3	89679117	0	6	38348	11	47892	14	76177	13	77181	12	98900	11	97145
chr3	89679123	0	20	5389	2	2401	23	7387	1	1933	27	6142	2	2259
chr3	89679126	0	3	38840	5	48605	6	77111	9	78452	9	1E+05	11	98724
chr3	89679132	0	1	38820	2	48572	5	77055	7	78401	18	1E+05	11	98675
chr3	89679136	0	13	5373	1	2395	19	7367	3	1932	14	6158	2	2253
chr3	89679137	0	8	37825	8	47284	22	75234	21	76756	35	97525	22	96071
chr3	89679141	0	2	38844	7	48635	14	77155	7	78583	11	1E+05	15	98816
chr3	89679142	0	51	38886	66	48649	194	77145	119	78633	253	1E+05	166	98946
chr3	89679144	0	1	5412	2	2404	3	7435	1	1953	1	6208	4	2269
chr3	89679146	0	38	38905	213	48505	108	77238	386	78383	146	1E+05	464	98674
chr3	89679148	0	2	5412	0	2406	2	7443	0	1955	2	6208	1	2274
chr3	89679149	0	6	5405	3	2400	10	7431	1	1953	7	6199	2	2271
chr3	89679150	0	122	38604	143	48351	224	76727	208	78227	283	99798	278	98382
chr3	89679155	1	15037	23899	29497	19229	28171	49183	47017	31760	36503	64107	59583	39574
chr3	89679156	1	2817	2596	1715	689	3447	3993	1385	569	3021	3193	1637	636
chr3	89679158	0	94	38796	64	48614	173	77130	107	78593	237	1E+05	134	98899
chr3	89679162	1	25149	13706	30998	17615	47551	29654	49854	28801	61922	38492	62862	36109
chr3	89679163	1	3110	2294	1782	618	3940	3488	1419	535	3378	2818	1701	568
chr3	89679164	0	87	38755	92	48487	154	77021	145	78469	239	1E+05	195	98733
chr3	89679165	0	76	38864	68	48656	143	77236	108	78676	160	1E+05	147	99039
chr3	89679167	0	17	38908	46	48653	50	77289	57	78708	70	1E+05	77	99051
chr3	89679168	0	122	38809	210	48492	377	76967	272	78504	470	1E+05	394	98732
chr3	89679169	0	1313	37652	639	48105	2573	74829	965	77858	3366	97309	1306	97904
chr3	89679171	0	455	38501	433	48301	914	76486	636	78186	1266	99404	892	98318
chr3	89679173	1	17027	21602	29337	19054	31582	45227	46742	31475	41227	58624	59032	39400
chr3	89679174	1	2785	2627	1754	648	3474	3966	1400	556	3027	3176	1638	635
chr3	89679176	0	52	5352	4	2396	82	7340	0	1947	87	6102	2	2269
chr3	89679177	0	660	38220	220	48427	1278	75988	357	78330	1650	98843	459	98580
chr3	89679180	0	41	38906	69	48655	79	77288	117	78671	107	1E+05	140	99025
chr3	89679181	0	60	38897	188	48542	114	77281	312	78498	151	1E+05	371	98825
chr3	89679182	0	357	38603	143	48590	739	76662	225	78597	910	99755	296	98918
chr3	89679184	0	7	5395	4	2398	11	7388	2	1946	3	6167	2	2265
chr3	89679186	1	18355	20601	30594	18127	34276	43100	49529	29276	44559	56085	62386	36766
chr3	89679187	1	2668	2741	1594	808	3338	4091	1290	665	2910	3281	1545	726
chr3	89679188	0	215	38698	342	48334	395	76919	490	78249	548	1E+05	599	98488

0	4075	0	1883	4	5265	0	1246	2	3652	0	1386
4	4181	1	1917	3	5419	0	1291	1	3821	1	1448
4625	5241	1858	2339	6065	7126	1466	1819	5047	5825	1758	2134
19	5183	12	2309	16	7070	9	1811	13	5775	12	2113
29	5283	3	2361	40	7241	2	1852	19	5955	4	2182
30	5273	7	2353	31	7232	5	1856	27	5966	1	2182
4	5308	1	2370	9	7283	3	1875	6	6025	5	2197
3	5382	2	2396	7	7363	4	1910	12	6101	3	2239
4096	5400	1732	2401	5196	7383	1348	1922	4480	6134	1594	2246
10	5401	4	2401	15	7383	1	1924	12	6136	2	2250
3	5403	3	2401	6	7384	2	1924	7	6135	2	2250
6	38354	11	47903	14	76191	13	77194	12	98912	11	97156
20	5409	2	2403	23	7410	1	1934	27	6169	2	2261
3	38843	5	48610	6	77117	9	78461	9	1E+05	11	98735
1	38821	2	48574	5	77060	7	78408	18	1E+05	11	98686
13	5386	1	2396	19	7386	3	1935	14	6172	2	2255
8	37833	8	47292	22	75256	21	76777	35	97560	22	96093
2	38846	7	48642	14	77169	7	78590	11	1E+05	15	98831
51	38937	66	48715	194	77339	119	78752	253	1E+05	166	99112
1	5413	2	2406	3	7438	1	1954	1	6209	4	2273
38	38943	213	48718	108	77346	386	78769	146	1E+05	464	99138
2	5414	0	2406	2	7445	0	1955	2	6210	1	2275
6	5411	3	2403	10	7441	1	1954	7	6206	2	2273
122	38726	143	48494	224	76951	208	78435	283	1E+05	278	98660
15037	38936	29497	48726	28171	77354	####	78777	36503	1E+05	59583	99157
2817	5413	1715	2404	3447	7440	1385	1954	3021	6214	1637	2273
94	38890	64	48678	173	77303	107	78700	237	1E+05	134	99033
25149	38855	30998	48613	47551	77205	####	78655	61922	1E+05	62862	98971
3110	5404	1782	2400	3940	7428	1419	1954	3378	6196	1701	2269
87	38842	92	48579	154	77175	145	78614	239	1E+05	195	98928
76	38940	68	48724	143	77379	108	78784	160	1E+05	147	99186
17	38925	46	48699	50	77339	57	78765	70	1E+05	77	99128
122	38931	210	48702	377	77344	272	78776	470	1E+05	394	99126
1313	38965	639	48744	2573	77402	965	78823	3366	1E+05	1306	99210
455	38956	433	48734	914	77400	636	78822	1266	1E+05	892	99210
17027	38629	29337	48391	31582	76809	####	78217	41227	99851	59032	98432
2785	5412	1754	2402	3474	7440	1400	1956	3027	6203	1638	2273
52	5404	4	2400	82	7422	0	1947	87	6189	2	2271
660	38880	220	48647	1278	77266	357	78687	1650	1E+05	459	99039
41	38947	69	48724	79	77367	117	78788	107	1E+05	140	99165
60	38957	188	48730	114	77395	312	78810	151	1E+05	371	99196
357	38960	143	48733	739	77401	225	78822	910	1E+05	296	99214
7	5402	4	2402	11	7399	2	1948	3	6170	2	2267
18355	38956	30594	48721	34276	77376	####	78805	44559	1E+05	62386	99152
2668	5409	1594	2402	3338	7429	1290	1955	2910	6191	1545	2271
215	38913	342	48676	395	77314	490	78739	548	1E+05	599	99087

chr3	89679190	0	5	5405	1	2401	15	7419	0	1956	9	6189	2	2270
chr3	89679192	0	20	5392	4	2400	19	7419	1	1955	15	6183	1	2272
chr3	89679193	0	9	5395	4	2398	21	7402	3	1952	21	6164	3	2267
chr3	89679194	0	79	38816	81	48595	129	77169	171	78538	173	1E+05	201	98880
chr3	89679196	0	235	38694	111	48593	358	76997	156	78614	537	1E+05	197	98961
chr3	89679198	0	11	5382	5	2391	15	7375	3	1943	10	6150	9	2261
chr3	89679199	0	113	38418	69	48152	194	76441	92	77898	270	99295	132	97948
chr3	89679200	1	17442	21513	30861	17869	32369	45019	49898	28900	42209	58444	63055	36104
chr3	89679201	1	2662	2733	1750	639	3416	3992	1412	538	2909	3264	1694	569
chr3	89679202	0	3	5305	1	2361	3	7322	0	1936	5	6096	0	2250
chr3	89679203	0	53	38779	138	48436	123	77007	249	78264	139	1E+05	271	98547
chr3	89679210	0	210	38573	342	48211	366	76679	515	77997	505	99724	768	97978
chr3	89679212	0	4	38873	7	48647	16	77235	8	78690	13	1E+05	15	98975
chr3	89679213	0	5	38828	9	48584	7	77122	8	78647	9	1E+05	6	98863
chr3	89679214	0	0	38956	5	48732	9	77381	4	78808	4	1E+05	5	99185
chr3	89679216	0	0	5259	0	2346	0	7218	1	1902	0	5995	1	2205
chr3	89679218	0	3	38791	4	48517	9	77000	7	78498	9	1E+05	7	98704
chr3	89679220	0	4	38922	8	48678	13	77305	5	78747	9	1E+05	10	99090
chr3	89679224	0	14	4939	8	2220	17	6780	1	1808	10	5623	3	2072
chr3	89679226	0	2	4989	0	2247	0	6737	1	1771	0	5551	2	2046
chr3	89679232	0	3	38715	4	48379	11	76990	5	78316	12	1E+05	8	98497
chr4	44628612	0	0	62	0	73	1	1050	0	529	0	401	0	395
chr4	44628613	0	0	63	0	72	0	1074	0	537	1	403	1	397
chr4	44628615	0	0	63	0	73	0	1097	0	549	1	414	0	411
chr4	44628620	0	0	62	1	70	0	1097	0	548	0	408	1	408
chr4	44628623	0	0	63	0	74	0	1115	1	556	0	419	0	415
chr4	44628630	0	0	63	0	73	0	1109	1	554	1	417	0	411
chr4	44628631	0	0	63	0	74	0	1119	0	558	0	420	0	416
chr4	44628632	0	0	63	0	74	3	1115	0	558	1	419	1	411
chr4	44628640	0	0	63	0	73	1	1120	3	555	1	420	1	414
chr4	44628641	0	0	63	0	74	7	1113	3	555	1	421	2	412
chr4	44628642	0	0	63	0	74	2	1118	1	559	1	421	2	413
chr4	44628643	1	45	18	64	10	855	268	480	79	336	86	366	49
chr4	44628646	1	53	10	73	1	1026	98	543	17	387	35	406	11
chr4	44628652	0	0	63	5	68	2	1108	38	520	0	417	32	381
chr4	44628657	0	0	63	0	73	1	1119	1	556	0	422	0	411
chr4	44628659	0	0	63	0	74	0	1120	0	560	0	423	0	414
chr4	44628661	0	0	62	0	75	0	1126	1	560	0	425	0	418
chr4	44628663	0	0	63	0	75	1	1125	0	562	0	425	0	418
chr4	44628665	0	0	63	1	73	0	1122	1	561	0	425	2	416
chr4	44628666	0	0	64	0	74	0	1126	0	562	0	425	0	417
chr4	44628667	0	0	64	0	75	1	1125	0	562	0	425	0	418
chr4	44628670	0	0	64	0	76	0	1127	0	561	1	425	0	418
chr4	44628671	0	0	64	0	76	0	1126	2	559	0	427	0	417
chr4	44628679	0	0	64	0	75	4	1119	1	557	4	423	5	414
chr4	44628680	0	0	64	0	76	2	1126	2	559	0	428	0	421

5	5410	1	2402	15	7434	0	1956	9	6198	2	2272
20	5412	4	2404	19	7438	1	1956	15	6198	1	2273
9	5404	4	2402	21	7423	3	1955	21	6185	3	2270
79	38895	81	48676	129	77298	171	78709	173	1E+05	201	99081
235	38929	111	48704	358	77355	156	78770	537	1E+05	197	99158
11	5393	5	2396	15	7390	3	1946	10	6160	9	2270
113	38531	69	48221	194	76635	92	77990	270	99565	132	98080
17442	38955	30861	48730	32369	77388	####	78798	42209	1E+05	63055	99159
2662	5395	1750	2389	3416	7408	1412	1950	2909	6173	1694	2263
3	5308	1	2362	3	7325	0	1936	5	6101	0	2250
53	38832	138	48574	123	77130	249	78513	139	1E+05	271	98818
210	38783	342	48553	366	77045	515	78512	505	1E+05	768	98746
4	38877	7	48654	16	77251	8	78698	13	1E+05	15	98990
5	38833	9	48593	7	77129	8	78655	9	1E+05	6	98869
0	38956	5	48737	9	77390	4	78812	4	1E+05	5	99190
0	5259	0	2346	0	7218	1	1903	0	5995	1	2206
3	38794	4	48521	9	77009	7	78505	9	1E+05	7	98711
4	38926	8	48686	13	77318	5	78752	9	1E+05	10	99100
14	4953	8	2228	17	6797	1	1809	10	5633	3	2075
2	4991	0	2247	0	6737	1	1772	0	5551	2	2048
3	38718	4	48383	11	77001	5	78321	12	1E+05	8	98505
0	62	0	73	1	1051	0	529	0	401	0	395
0	63	0	72	0	1074	0	537	1	404	1	398
0	63	0	73	0	1097	0	549	1	415	0	411
0	62	1	71	0	1097	0	548	0	408	1	409
0	63	0	74	0	1115	1	557	0	419	0	415
0	63	0	73	0	1109	1	555	1	418	0	411
0	63	0	74	0	1119	0	558	0	420	0	416
0	63	0	74	3	1118	0	558	1	420	1	412
0	63	0	73	1	1121	3	558	1	421	1	415
0	63	0	74	7	1120	3	558	1	422	2	414
0	63	0	74	2	1120	1	560	1	422	2	415
45	63	64	74	855	1123	480	559	336	422	366	415
53	63	73	74	1026	1124	543	560	387	422	406	417
0	63	5	73	2	1110	38	558	0	417	32	413
0	63	0	73	1	1120	1	557	0	422	0	411
0	63	0	74	0	1120	0	560	0	423	0	414
0	62	0	75	0	1126	1	561	0	425	0	418
0	63	0	75	1	1126	0	562	0	425	0	418
0	63	1	74	0	1122	1	562	0	425	2	418
0	64	0	74	0	1126	0	562	0	425	0	417
0	64	0	75	1	1126	0	562	0	425	0	418
0	64	0	76	0	1127	0	561	1	426	0	418
0	64	0	76	0	1126	2	561	0	427	0	417
0	64	0	75	4	1123	1	558	4	427	5	419
0	64	0	76	2	1128	2	561	0	428	0	421

chr4	44628684	1	57	7	70	6	964	166	525	38	371	58	392	29
chr4	44628686	0	0	65	0	76	6	1123	0	563	0	427	0	423
chr4	44628687	0	0	65	0	76	1	1130	6	557	0	427	1	422
chr4	44628690	0	0	65	0	76	4	1127	2	561	1	427	1	421
chr4	44628692	0	0	64	0	76	2	1129	1	562	0	427	0	424
chr4	44628694	0	0	65	1	77	3	1128	0	562	0	428	1	422
chr4	44628695	0	0	64	0	78	1	1131	0	563	0	428	3	421
chr4	44628697	0	0	65	0	78	1	1127	0	561	0	428	1	421
chr4	44628698	0	0	68	0	78	0	1131	0	562	1	426	0	422
chr4	44628700	0	0	71	0	79	1	1133	0	564	0	428	0	422
chr4	44628701	0	0	72	0	79	2	1129	1	563	4	424	3	419
chr4	44628702	0	0	72	0	79	2	1131	3	561	1	427	0	418
chr4	44628703	0	0	72	0	78	1	1130	1	563	3	423	0	419
chr4	44628706	0	0	72	0	79	1	1127	1	558	0	426	1	414
chr4	44628707	0	0	71	0	79	1	1133	0	563	0	423	1	416
chr4	44628708	0	0	72	0	79	1	1131	0	559	1	424	1	416
chr4	44628712	0	0	72	0	79	0	1136	0	564	0	425	0	419
chr4	44628716	0	0	68	0	81	2	1124	0	562	2	416	2	410
chr4	44628717	0	0	72	0	81	1	1135	0	567	1	424	2	416
chr4	44628737	0	0	70	0	83	1	1135	2	574	2	427	0	422
chr4	44628738	0	0	71	0	83	4	1136	0	578	0	431	0	424
chr4	44628740	0	0	70	0	83	1	1139	0	578	2	428	0	422
chr4	44628746	0	0	70	0	84	0	1140	1	579	0	432	4	421
chr4	44628750	0	0	71	0	84	4	1138	1	581	0	430	1	424
chr4	44628751	0	0	71	0	84	3	1141	0	580	3	429	0	424
chr4	44628786	0	0	29	2	22	6	317	0	169	2	165	2	148
chr4	44628787	1	68	4	84	2	1100	55	556	31	424	20	389	30
chr4	44628795	0	0	72	0	86	1	1154	0	595	1	441	1	416
chr4	44628796	0	1	71	0	87	5	1158	1	596	3	440	1	419
chr4	44628797	0	0	70	0	86	2	1133	3	577	2	436	3	408
chr4	44628798	0	0	72	0	87	0	1160	0	599	0	444	0	421
chr4	44628799	1	58	14	85	1	1016	146	573	27	386	57	399	20
chr4	44628804	0	0	71	0	85	2	1158	0	593	1	437	3	409
chr4	44628807	0	0	71	0	86	1	1156	1	589	0	436	1	412
chr4	44628819	0	0	74	0	90	1	1163	0	598	0	442	0	412
chr4	44628821	0	0	78	0	92	2	1155	0	594	0	444	2	411
chr4	44628828	0	0	77	1	92	4	1161	1	597	2	444	1	411
chr4	44628832	1	67	9	89	3	1103	66	594	3	427	18	411	3
chr4	44628834	0	0	75	0	91	4	1156	4	595	1	446	2	410
chr4	44628843	0	0	74	0	90	3	1163	1	598	0	445	1	415
chr4	44628845	0	0	72	0	89	1	1150	0	594	1	434	0	411
chr4	44628851	0	0	72	0	89	5	1158	2	596	2	439	1	412
chr4	44628852	0	0	72	0	88	3	1155	3	590	1	437	3	407
chr4	44628856	0	0	70	1	87	3	1139	1	582	1	424	1	403
chr4	44628862	0	0	70	0	86	2	1130	0	587	0	424	1	406
chr4	44628869	0	1	69	0	89	0	1159	1	596	1	437	1	410

57	64	70	76	964	1130	525	563	371	429	392	421
0	65	0	76	6	1129	0	563	0	427	0	423
0	65	0	76	1	1131	6	563	0	427	1	423
0	65	0	76	4	1131	2	563	1	428	1	422
0	64	0	76	2	1131	1	563	0	427	0	424
0	65	1	78	3	1131	0	562	0	428	1	423
0	64	0	78	1	1132	0	563	0	428	3	424
0	65	0	78	1	1128	0	561	0	428	1	422
0	68	0	78	0	1131	0	562	1	427	0	422
0	71	0	79	1	1134	0	564	0	428	0	422
0	72	0	79	2	1131	1	564	4	428	3	422
0	72	0	79	2	1133	3	564	1	428	0	418
0	72	0	78	1	1131	1	564	3	426	0	419
0	72	0	79	1	1128	1	559	0	426	1	415
0	71	0	79	1	1134	0	563	0	423	1	417
0	72	0	79	1	1132	0	559	1	425	1	417
0	72	0	79	0	1136	0	564	0	425	0	419
0	68	0	81	2	1126	0	562	2	418	2	412
0	72	0	81	1	1136	0	567	1	425	2	418
0	70	0	83	1	1136	2	576	2	429	0	422
0	71	0	83	4	1140	0	578	0	431	0	424
0	70	0	83	1	1140	0	578	2	430	0	422
0	70	0	84	0	1140	1	580	0	432	4	425
0	71	0	84	4	1142	1	582	0	430	1	425
0	71	0	84	3	1144	0	580	3	432	0	424
0	29	2	24	6	323	0	169	2	167	2	150
68	72	84	86	1100	1155	556	587	424	444	389	419
0	72	0	86	1	1155	0	595	1	442	1	417
1	72	0	87	5	1163	1	597	3	443	1	420
0	70	0	86	2	1135	3	580	2	438	3	411
0	72	0	87	0	1160	0	599	0	444	0	421
58	72	85	86	1016	1162	573	600	386	443	399	419
0	71	0	85	2	1160	0	593	1	438	3	412
0	71	0	86	1	1157	1	590	0	436	1	413
0	74	0	90	1	1164	0	598	0	442	0	412
0	78	0	92	2	1157	0	594	0	444	2	413
0	77	1	93	4	1165	1	598	2	446	1	412
67	76	89	92	1103	1169	594	597	427	445	411	414
0	75	0	91	4	1160	4	599	1	447	2	412
0	74	0	90	3	1166	1	599	0	445	1	416
0	72	0	89	1	1151	0	594	1	435	0	411
0	72	0	89	5	1163	2	598	2	441	1	413
0	72	0	88	3	1158	3	593	1	438	3	410
0	70	1	88	3	1142	1	583	1	425	1	404
0	70	0	86	2	1132	0	587	0	424	1	407
1	70	0	89	0	1159	1	597	1	438	1	411

chr4	44628871	0	0	71	1	88	0	1163	1	598	4	434	1	411
chr4	44628876	0	0	71	1	88	6	1156	2	595	1	437	2	409
chr4	44628882	0	0	71	2	87	3	1154	2	595	2	432	0	408
chr4	44628883	0	2	68	0	89	4	1156	2	594	1	436	1	410
chr4	44628889	0	0	70	0	88	2	1154	4	591	2	433	2	409
chr4	44628890	0	0	71	1	88	2	1156	3	591	3	428	1	408
chr4	44628891	0	0	71	1	88	2	1159	0	595	2	430	0	410
chr4	44628893	0	0	71	0	89	1	1155	1	592	1	431	1	408
chr4	44628898	0	0	70	0	86	0	1142	2	583	0	431	2	407
chr4	44628906	0	0	71	0	88	4	1151	1	593	3	429	0	410
chr4	44628908	1	61	10	84	4	943	210	554	40	351	80	391	19
chr4	44628911	0	0	70	0	87	0	1146	0	590	0	429	0	407
chr4	44628912	0	0	71	0	87	1	1150	0	591	0	428	0	406
chr4	44628918	0	0	71	0	88	0	1152	0	591	0	429	0	408
chr4	44628931	0	1	69	0	88	0	1129	0	573	0	417	1	396

0	71	1	89	0	1163	1	599	4	438	1	412
0	71	1	89	6	1162	2	597	1	438	2	411
0	71	2	89	3	1157	2	597	2	434	0	408
2	70	0	89	4	1160	2	596	1	437	1	411
0	70	0	88	2	1156	4	595	2	435	2	411
0	71	1	89	2	1158	3	594	3	431	1	409
0	71	1	89	2	1161	0	595	2	432	0	410
0	71	0	89	1	1156	1	593	1	432	1	409
0	70	0	86	0	1142	2	585	0	431	2	409
0	71	0	88	4	1155	1	594	3	432	0	410
61	71	84	88	943	1153	554	594	351	431	391	410
0	70	0	87	0	1146	0	590	0	429	0	407
0	71	0	87	1	1151	0	591	0	428	0	406
0	71	0	88	0	1152	0	591	0	429	0	408
1	70	0	88	0	1129	0	573	0	417	1	397

CD4 naïve wild type cytosines

Number of C's and T's

			naivebs1		naiveoxbs1		naivebs2		naiveoxbs2		naivebs3		naiveoxbs3	
**Chromosome**	**Position**		**C to C**	**C to T**	**C to C**	**C to T**	**C to C**	**C to T**	**C to C**	**C to T**	**C to C**	**C to T**	**C to C**	**C to T**
chr2	11124412	0	8	8321	5	9088	9	8175	8	10301	4	10085	4	11363
chr2	11124413	0	2	8374	1	9124	2	8223	6	10362	2	10154	2	11417
chr2	11124414	0	0	8379	4	9121	2	8228	0	10366	1	10152	2	11420
chr2	11124418	0	5	8337	5	9092	8	8192	6	10315	8	10112	8	11369
chr2	11124422	0	2	8386	1	9137	3	8246	2	10390	1	10177	6	11448
chr2	11124423	0	2	8385	0	9133	0	8250	4	10390	3	10173	0	11452
chr2	11124429	0	6	8386	6	9135	8	8259	15	10385	11	10185	8	11456
chr2	11124431	0	26	8370	1	9145	34	8241	4	10407	35	10169	3	11460
chr2	11124435	0	7	8394	35	9113	10	8271	42	10372	11	10200	57	11413
chr2	11124442	0	8	8353	6	9106	11	8249	7	10365	15	10175	11	11421
chr2	11124443	0	13	8382	9	9134	30	8250	13	10378	39	10165	10	11432
chr2	11124447	0	37	8164	31	8942	26	8093	34	10185	27	10023	46	11220
chr2	11124451	0	30	8355	15	9095	38	8222	22	10354	51	10152	28	11413
chr2	11124454	0	38	8371	53	9110	38	8279	65	10367	63	10187	88	11418
chr2	11124458	0	5	8403	3	9160	9	8308	7	10428	8	10245	8	11513
chr2	11124460	0	3	8407	4	9160	3	8323	4	10436	6	10250	7	11512
chr2	11124463	1	6285	2126	6304	2857	5888	2439	7092	3349	7323	2942	7839	3676
chr2	11124467	0	13	8343	19	9099	19	8274	15	10368	13	10207	15	11452
chr2	11124468	0	44	8373	8	9151	51	8264	13	10409	69	10178	18	11488
chr2	11124469	0	12	8414	12	9158	14	8320	17	10431	17	10261	23	11512
chr2	11124470	0	10	8421	5	9167	12	8318	15	10433	11	10267	16	11513
chr2	11124471	0	24	8397	13	9162	38	8296	13	10440	49	10230	15	11521
chr2	11124474	0	10	8399	5	9127	9	8312	11	10411	21	10238	18	11490
chr2	11124479	0	0	171	1	189	1	365	2	327	1	375	2	392
chr2	11124480	0	22	8389	21	9140	16	8299	22	10397	24	10224	30	11469
chr2	11124481	0	11	8426	13	9162	13	8335	11	10447	9	10278	22	11518
chr2	11124483	0	9	8426	8	9168	7	8340	9	10454	12	10275	9	11533
chr2	11124484	0	17	8420	12	9164	28	8314	12	10449	45	10240	17	11525
chr2	11124485	0	28	8405	5	9163	24	8317	6	10450	33	10251	6	11530
chr2	11124488	0	0	207	1	203	0	448	1	396	3	444	2	469
chr2	11124489	0	15	8411	12	9152	12	8323	16	10429	13	10264	17	11495
chr2	11124491	0	11	8424	7	9167	15	8331	16	10448	13	10277	21	11523
chr2	11124492	0	39	8396	6	9168	44	8310	9	10456	49	10245	6	11540
chr2	11124494	0	0	221	0	222	0	471	0	430	0	488	1	514
chr2	11124495	0	2	203	1	210	0	454	2	402	3	463	0	490
chr2	11124496	0	91	8251	129	8951	101	8153	150	10210	135	10044	174	11254
chr2	11124498	1	1352	7034	3237	5900	1316	7007	3722	6695	1589	8652	4043	7451
chr2	11124499	1	15	207	18	203	38	433	28	402	31	457	41	478
chr2	11124502	0	1	204	1	204	1	446	1	393	1	452	0	480
chr2	11124503	0	11	8407	18	9135	20	8319	24	10423	18	10261	31	11506

Number of C's and total read outs

naivebs1		naiveoxbs1		naivebs2		naiveoxbs2		naivebs3		naiveoxbs3	
C	Total	C	Total	C	Total	C	Total	C	Total	C	Total
8	8329	5	9093	9	8184	8	10309	4	10089	4	11367
2	8376	1	9125	2	8225	6	10368	2	10156	2	11419
0	8379	4	9125	2	8230	0	10366	1	10153	2	11422
5	8342	5	9097	8	8200	6	10321	8	10120	8	11377
2	8388	1	9138	3	8249	2	10392	1	10178	6	11454
2	8387	0	9133	0	8250	4	10394	3	10176	0	11452
6	8392	6	9141	8	8267	15	10400	11	10196	8	11464
26	8396	1	9146	34	8275	4	10411	35	10204	3	11463
7	8401	35	9148	10	8281	42	10414	11	10211	57	11470
8	8361	6	9112	11	8260	7	10372	15	10190	11	11432
13	8395	9	9143	30	8280	13	10391	39	10204	10	11442
37	8201	31	8973	26	8119	34	10219	27	10050	46	11266
30	8385	15	9110	38	8260	22	10376	51	10203	28	11441
38	8409	53	9163	38	8317	65	10432	63	10250	88	11506
5	8408	3	9163	9	8317	7	10435	8	10253	8	11521
3	8410	4	9164	3	8326	4	10440	6	10256	7	11519
6285	8411	6304	9161	5888	8327	7092	10441	7323	10265	7839	11515
13	8356	19	9118	19	8293	15	10383	13	10220	15	11467
44	8417	8	9159	51	8315	13	10422	69	10247	18	11506
12	8426	12	9170	14	8334	17	10448	17	10278	23	11535
10	8431	5	9172	12	8330	15	10448	11	10278	16	11529
24	8421	13	9175	38	8334	13	10453	49	10279	15	11536
10	8409	5	9132	9	8321	11	10422	21	10259	18	11508
0	171	1	190	1	366	2	329	1	376	2	394
22	8411	21	9161	16	8315	22	10419	24	10248	30	11499
11	8437	13	9175	13	8348	11	10458	9	10287	22	11540
9	8435	8	9176	7	8347	9	10463	12	10287	9	11542
17	8437	12	9176	28	8342	12	10461	45	10285	17	11542
28	8433	5	9168	24	8341	6	10456	33	10284	6	11536
0	207	1	204	0	448	1	397	3	447	2	471
15	8426	12	9164	12	8335	16	10445	13	10277	17	11512
11	8435	7	9174	15	8346	16	10464	13	10290	21	11544
39	8435	6	9174	44	8354	9	10465	49	10294	6	11546
0	221	0	222	0	471	0	430	0	488	1	515
2	205	1	211	0	454	2	404	3	466	0	490
91	8342	129	9080	101	8254	150	10360	135	10179	174	11428
1352	8386	3237	9137	1316	8323	3722	10417	1589	10241	4043	11494
15	222	18	221	38	471	28	430	31	488	41	519
1	205	1	205	1	447	1	394	1	453	0	480
11	8418	18	9153	20	8339	24	10447	18	10279	31	11537

chr2	11124504	0	9	8430	11	9161	11	8343	3	10454	13	10274	8	11533
chr2	11124507	0	2	200	2	204	1	452	0	402	4	455	1	484
chr2	11124508	0	412	8014	251	8910	409	7929	252	10184	501	9769	299	11220
chr2	11124510	0	18	8409	11	9155	26	8326	12	10435	23	10251	10	11512
chr2	11124511	0	55	8380	7	9160	57	8293	9	10450	85	10200	10	11531
chr2	11124513	0	3	8432	11	9160	4	8346	11	10442	9	10272	15	11526
chr2	11124514	0	45	8392	7	9162	40	8310	11	10445	64	10217	11	11529
chr2	11124516	0	5	8430	10	9157	12	8333	12	10437	12	10268	12	11527
chr2	11124517	1	6102	2330	6163	3003	5755	2591	6850	3603	7136	3143	7500	4036
chr2	11124518	1	120	92	102	113	224	230	211	207	246	227	253	262
chr2	11124519	0	19	8410	19	9144	21	8324	14	10433	24	10241	11	11517
chr2	11124520	0	14	8417	53	9117	18	8330	51	10401	13	10266	82	11453
chr2	11124522	0	14	8402	9	9137	9	8317	14	10420	13	10234	17	11486
chr2	11124523	1	6343	2082	6887	2274	5956	2383	7784	2656	7433	2835	8460	3062
chr2	11124524	1	141	80	96	125	244	228	212	222	267	226	226	294
chr2	11124525	0	12	8424	12	9155	13	8330	15	10436	13	10261	17	11515
chr2	11124526	0	49	8349	7	9122	32	8292	7	10412	36	10200	8	11497
chr2	11124528	0	10	8424	8	9155	10	8333	7	10438	7	10265	8	11521
chr2	11124529	1	5316	3113	6775	2390	5057	3280	7687	2757	6331	3936	8482	3048
chr2	11124530	1	3	217	7	214	7	466	7	427	7	493	9	517
chr2	11124531	0	13	8406	11	9146	16	8314	16	10408	20	10232	25	11492
chr2	11124532	0	13	8416	7	9160	3	8338	9	10437	10	10257	3	11525
chr2	11124534	1	3138	5256	4811	4334	2851	5468	5352	5054	3640	6596	5841	5656
chr2	11124535	1	37	186	40	183	73	402	93	348	92	411	95	434
chr2	11124536	0	1	222	0	223	3	473	0	440	0	502	0	527
chr2	11124537	0	16	8369	13	9087	15	8273	27	10348	22	10166	18	11443
chr2	11124538	0	9	8418	6	9151	3	8323	11	10416	13	10240	11	11496
chr2	11124540	0	10	8414	11	9148	9	8317	8	10414	11	10243	14	11495
chr2	11124542	0	3	218	1	222	0	475	1	441	2	500	0	527
chr2	11124543	0	16	8409	11	9141	15	8306	12	10396	20	10225	15	11487
chr2	11124544	0	29	8396	5	9151	24	8296	2	10415	32	10219	10	11498
chr2	11124546	1	1652	6770	4676	4471	1581	6735	5268	5131	1939	8296	5755	5729
chr2	11124547	1	24	199	34	189	29	450	76	370	51	454	85	444
chr2	11124548	0	0	223	0	220	4	472	0	444	2	498	2	524
chr2	11124549	0	15	8396	19	9116	12	8293	24	10362	28	10193	30	11455
chr2	11124550	0	46	8375	26	9122	50	8262	41	10366	49	10190	33	11458
chr2	11124552	0	9	8384	34	9087	11	8257	29	10337	13	10192	42	11399
chr2	11124553	1	3136	5230	5275	3776	2978	5287	5873	4420	3649	6520	6483	4879
chr2	11124554	1	21	202	27	194	42	431	83	361	62	437	101	425
chr2	11124555	0	18	8391	11	9118	10	8285	15	10370	14	10197	14	11446
chr2	11124556	0	12	8374	10	9111	10	8268	13	10362	22	10171	14	11425
chr2	11124558	0	5	8329	14	9067	9	8210	14	10304	7	10153	16	11372
chr2	11124559	1	1730	6637	3717	5379	1558	6698	4216	6122	1986	8173	4490	6911
chr2	11124560	1	18	205	23	200	34	445	72	374	42	462	62	465
chr2	11124561	0	1	222	0	222	0	475	0	445	1	503	0	526
chr2	11124562	0	0	223	0	222	1	476	1	441	4	498	0	525

9	8439	11	9172	11	8354	3	10457	13	10287	8	11541
2	202	2	206	1	453	0	402	4	459	1	485
412	8426	251	9161	409	8338	252	10436	501	10270	299	11519
18	8427	11	9166	26	8352	12	10447	23	10274	10	11522
55	8435	7	9167	57	8350	9	10459	85	10285	10	11541
3	8435	11	9171	4	8350	11	10453	9	10281	15	11541
45	8437	7	9169	40	8350	11	10456	64	10281	11	11540
5	8435	10	9167	12	8345	12	10449	12	10280	12	11539
6102	8432	6163	9166	5755	8346	6850	10453	7136	10279	7500	11536
120	212	102	215	224	454	211	418	246	473	253	515
19	8429	19	9163	21	8345	14	10447	24	10265	11	11528
14	8431	53	9170	18	8348	51	10452	13	10279	82	11535
14	8416	9	9146	9	8326	14	10434	13	10247	17	11503
6343	8425	6887	9161	5956	8339	7784	10440	7433	10268	8460	11522
141	221	96	221	244	472	212	434	267	493	226	520
12	8436	12	9167	13	8343	15	10451	13	10274	17	11532
49	8398	7	9129	32	8324	7	10419	36	10236	8	11505
10	8434	8	9163	10	8343	7	10445	7	10272	8	11529
5316	8429	6775	9165	5057	8337	7687	10444	6331	10267	8482	11530
3	220	7	221	7	473	7	434	7	500	9	526
13	8419	11	9157	16	8330	16	10424	20	10252	25	11517
13	8429	7	9167	3	8341	9	10446	10	10267	3	11528
3138	8394	4811	9145	2851	8319	5352	10406	3640	10236	5841	11497
37	223	40	223	73	475	93	441	92	503	95	529
1	223	0	223	3	476	0	440	0	502	0	527
16	8385	13	9100	15	8288	27	10375	22	10188	18	11461
9	8427	6	9157	3	8326	11	10427	13	10253	11	11507
10	8424	11	9159	9	8326	8	10422	11	10254	14	11509
3	221	1	223	0	475	1	442	2	502	0	527
16	8425	11	9152	15	8321	12	10408	20	10245	15	11502
29	8425	5	9156	24	8320	2	10417	32	10251	10	11508
1652	8422	4676	9147	1581	8316	5268	10399	1939	10235	5755	11484
24	223	34	223	29	479	76	446	51	505	85	529
0	223	0	220	4	476	0	444	2	500	2	526
15	8411	19	9135	12	8305	24	10386	28	10221	30	11485
46	8421	26	9148	50	8312	41	10407	49	10239	33	11491
9	8393	34	9121	11	8268	29	10366	13	10205	42	11441
3136	8366	5275	9051	2978	8265	5873	10293	3649	10169	6483	11362
21	223	27	221	42	473	83	444	62	499	101	526
18	8409	11	9129	10	8295	15	10385	14	10211	14	11460
12	8386	10	9121	10	8278	13	10375	22	10193	14	11439
5	8334	14	9081	9	8219	14	10318	7	10160	16	11388
1730	8367	3717	9096	1558	8256	4216	10338	1986	10159	4490	11401
18	223	23	223	34	479	72	446	42	504	62	527
1	223	0	222	0	475	0	445	1	504	0	526
0	223	0	222	1	477	1	442	4	502	0	525

chr2	11124563	0	0	8341	2	9051	1	8215	1	10266	2	10134	0	11323
chr2	11124567	0	0	223	0	223	0	479	0	444	0	503	0	528
chr2	11124572	0	1	222	0	223	2	477	0	446	1	503	0	527
chr2	11124576	0	0	8142	0	8903	1	7943	0	9996	0	9864	2	11053
chr2	11124581	0	5	8097	0	8807	1	7880	4	9922	1	9712	3	10876
chr2	11124584	0	0	221	0	222	1	475	0	445	0	504	0	527
chr2	11124586	0	15	7935	5	8624	4	7540	5	9525	5	9341	9	10408
chr2	11124588	0	0	221	0	220	0	477	0	445	0	504	0	525
chr2	11124593	0	0	221	0	219	1	478	0	444	0	504	0	523
chr2	11124597	0	0	221	0	219	1	478	0	444	0	503	0	524
chr2	11124598	0	0	221	0	219	0	479	0	444	1	502	0	523
chr2	11124600	0	0	219	0	213	1	467	2	437	1	499	1	517
chr2	11124609	0	0	212	0	213	0	458	0	425	2	482	1	501
chr3	89184744	0	0	1801	2	2026	1	2090	1	2078	3	2290	5	2294
chr3	89184752	0	3	2419	0	1455	2	1049	1	763	3	1296	0	751
chr3	89184753	0	3	1830	3	2077	6	2197	1	2210	1	2408	0	2421
chr3	89184754	0	1	1842	0	2087	0	2210	0	2226	0	2422	0	2433
chr3	89184756	0	0	1827	0	2071	0	2200	1	2210	0	2409	1	2417
chr3	89184758	0	0	2202	0	1316	4	2302	5	2612	4	2652	7	2539
chr3	89184759	0	1	2452	0	1479	1	2290	3	2493	0	2653	1	2479
chr3	89184760	0	3	2453	2	1477	10	2398	2	2644	7	2757	2	2600
chr3	89184762	0	0	1844	0	2089	0	2214	1	2230	2	2426	1	2435
chr3	89184763	0	0	1836	0	2080	1	2210	1	2226	1	2424	0	2431
chr3	89184765	0	0	2461	1	1480	0	2448	1	2702	2	2816	3	2664
chr3	89184768	0	1	2458	2	1481	1	2457	1	2711	2	2822	1	2675
chr3	89184769	0	0	2458	0	1482	4	2455	1	2713	3	2824	1	2676
chr3	89184773	0	1	2460	1	1481	1	2461	0	2714	1	2825	1	2678
chr3	89184774	0	3	1846	4	2088	4	2213	3	2238	5	2433	2	2452
chr3	89184776	1	1141	709	1128	964	1366	858	1221	1020	1557	885	1338	1120
chr3	89184777	1	1391	1056	928	551	1272	1188	1618	1095	1480	1347	1626	1054
chr3	89184778	0	7	2442	3	1472	7	2433	4	2698	3	2808	6	2661
chr3	89184779	0	3	2458	2	1483	8	2455	6	2707	5	2825	4	2673
chr3	89184780	0	2	1823	5	2056	4	2184	5	2207	7	2407	4	2406
chr3	89184781	0	3	1846	3	2090	10	2215	4	2239	4	2443	6	2452
chr3	89184782	0	3	1841	6	2072	4	2208	6	2228	4	2431	6	2436
chr3	89184783	0	4	1812	13	2032	6	2188	18	2194	2	2400	16	2410
chr3	89184787	0	3	2460	1	1482	2	2463	2	2718	1	2834	1	2687
chr3	89184788	0	3	2378	3	1436	2	2429	6	2688	4	2771	4	2653
chr3	89184792	0	12	2334	3	1411	16	2347	3	2606	21	2694	7	2560
chr3	89184795	0	3	2463	4	1482	3	2476	6	2723	5	2835	4	2692
chr3	89184800	0	4	2460	2	1480	3	2477	9	2721	7	2833	2	2693
chr3	89184801	0	2	1846	1	2095	3	2227	1	2240	3	2446	6	2451
chr3	89184805	0	0	1857	2	2100	0	2235	2	2249	1	2457	2	2469
chr3	89184806	1	1465	392	1627	475	1776	458	1748	503	1981	475	1920	551
chr3	89184807	1	1865	603	1194	295	1801	685	2125	608	2107	742	2084	602
chr3	89184808	0	1	2464	2	1488	2	2476	1	2719	7	2831	6	2691

0	8341	2	9053	1	8216	1	10267	2	10136	0	11323
0	223	0	223	0	479	0	444	0	503	0	528
1	223	0	223	2	479	0	446	1	504	0	527
0	8142	0	8903	1	7944	0	9996	0	9864	2	11055
5	8102	0	8807	1	7881	4	9926	1	9713	3	10879
0	221	0	222	1	476	0	445	0	504	0	527
15	7950	5	8629	4	7544	5	9530	5	9346	9	10417
0	221	0	220	0	477	0	445	0	504	0	525
0	221	0	219	1	479	0	444	0	504	0	523
0	221	0	219	1	479	0	444	0	503	0	524
0	221	0	219	0	479	0	444	1	503	0	523
0	219	0	213	1	468	2	439	1	500	1	518
0	212	0	213	0	458	0	425	2	484	1	502
0	1801	2	2028	1	2091	1	2079	3	2293	5	2299
3	2422	0	1455	2	1051	1	764	3	1299	0	751
3	1833	3	2080	6	2203	1	2211	1	2409	0	2421
1	1843	0	2087	0	2210	0	2226	0	2422	0	2433
0	1827	0	2071	0	2200	1	2211	0	2409	1	2418
0	2202	0	1316	4	2306	5	2617	4	2656	7	2546
1	2453	0	1479	1	2291	3	2496	0	2653	1	2480
3	2456	2	1479	10	2408	2	2646	7	2764	2	2602
0	1844	0	2089	0	2214	1	2231	2	2428	1	2436
0	1836	0	2080	1	2211	1	2227	1	2425	0	2431
0	2461	1	1481	0	2448	1	2703	2	2818	3	2667
1	2459	2	1483	1	2458	1	2712	2	2824	1	2676
0	2458	0	1482	4	2459	1	2714	3	2827	1	2677
1	2461	1	1482	1	2462	0	2714	1	2826	1	2679
3	1849	4	2092	4	2217	3	2241	5	2438	2	2454
1141	1850	1128	2092	1366	2224	1221	2241	1557	2442	1338	2458
1391	2447	928	1479	1272	2460	1618	2713	1480	2827	1626	2680
7	2449	3	1475	7	2440	4	2702	3	2811	6	2667
3	2461	2	1485	8	2463	6	2713	5	2830	4	2677
2	1825	5	2061	4	2188	5	2212	7	2414	4	2410
3	1849	3	2093	10	2225	4	2243	4	2447	6	2458
3	1844	6	2078	4	2212	6	2234	4	2435	6	2442
4	1816	13	2045	6	2194	18	2212	2	2402	16	2426
3	2463	1	1483	2	2465	2	2720	1	2835	1	2688
3	2381	3	1439	2	2431	6	2694	4	2775	4	2657
12	2346	3	1414	16	2363	3	2609	21	2715	7	2567
3	2466	4	1486	3	2479	6	2729	5	2840	4	2696
4	2464	2	1482	3	2480	9	2730	7	2840	2	2695
2	1848	1	2096	3	2230	1	2241	3	2449	6	2457
0	1857	2	2102	0	2235	2	2251	1	2458	2	2471
1465	1857	1627	2102	1776	2234	1748	2251	1981	2456	1920	2471
1865	2468	1194	1489	1801	2486	2125	2733	2107	2849	2084	2686
1	2465	2	1490	2	2478	1	2720	7	2838	6	2697

chr3	89184809	0	9	2466	1	1492	9	2478	6	2722	10	2836	7	2681
chr3	89184810	0	1	2470	2	1490	9	2481	12	2721	4	2849	11	2688
chr3	89184814	0	2	2463	0	1489	2	2486	4	2725	1	2845	1	2696
chr3	89184815	1	1568	292	1697	408	1902	339	1801	452	2116	351	1982	493
chr3	89184816	1	2127	351	1239	254	2055	433	2242	496	2401	452	2251	444
chr3	89184817	0	3	2472	3	1490	8	2484	13	2728	7	2850	8	2689
chr3	89184818	0	2	1855	4	2092	4	2231	2	2251	2	2467	9	2462
chr3	89184820	0	4	2435	2	1474	4	2474	7	2725	2	2842	1	2697
chr3	89184821	1	1380	480	1348	758	1682	558	1426	829	1905	569	1594	880
chr3	89184822	1	1946	527	1173	319	1876	605	2028	695	2163	681	2011	680
chr3	89184824	1	1246	616	1381	726	1469	772	1503	756	1685	790	1638	840
chr3	89184825	1	1799	626	1236	233	1687	775	2201	514	2005	806	2180	493
chr3	89184826	0	10	2334	3	1417	8	2300	5	2497	5	2625	6	2439
chr3	89184827	0	6	2289	7	1354	3	2347	6	2596	6	2669	10	2538
chr3	89184828	0	2	1821	3	2072	3	2206	0	2221	1	2430	6	2430
chr3	89184831	0	1	1862	5	2105	3	2239	2	2259	2	2472	6	2480
chr3	89184833	0	4	2465	3	1482	2	2488	7	2695	9	2804	6	2628
chr3	89184834	0	2	2423	4	1455	5	2463	3	2714	2	2815	4	2666
chr3	89184836	1	1446	417	1473	638	1715	529	1591	672	1940	533	1763	721
chr3	89184837	1	1817	659	1114	377	1774	734	2016	736	2025	840	2002	700
chr3	89184839	0	3	2468	3	1485	1	2505	5	2741	6	2855	5	2695
chr3	89184840	0	5	2472	2	1489	6	2507	6	2737	6	2862	4	2697
chr3	89184842	0	0	2352	6	1417	3	2472	10	2701	1	2801	20	2648
chr3	89184843	0	9	2441	11	1471	7	2423	34	2587	8	2750	33	2533
chr3	89184844	1	1604	254	1715	393	1949	296	1851	412	2176	291	2038	449
chr3	89184845	1	2163	313	1098	395	2125	400	1998	768	2422	468	1969	750
chr3	89184846	0	10	2452	4	1481	4	2506	5	2744	7	2869	2	2701
chr3	89184848	0	3	2470	16	1476	17	2487	64	2675	19	2851	45	2646
chr3	89184849	0	3	2469	1	1491	3	2516	6	2751	3	2883	9	2701
chr3	89184850	1	1579	281	1627	475	1896	345	1748	511	2097	359	1953	532
chr3	89184851	1	2093	381	1258	236	2062	463	2253	512	2369	523	2212	502
chr3	89184852	0	28	2447	3	1490	29	2495	8	2754	44	2849	8	2702
chr3	89184853	1	1595	268	1643	470	1916	331	1731	525	2135	330	1982	510
chr3	89184854	1	2059	411	1303	189	2056	462	2382	386	2342	549	2328	388
chr3	89184855	0	10	2306	0	1412	3	2435	5	2703	3	2804	4	2653
chr3	89184856	1	1618	237	1694	406	1936	298	1816	433	2152	309	2022	457
chr3	89184857	1	2121	353	1278	216	2086	433	2321	443	2383	496	2258	447
chr3	89184858	0	2	2474	4	1490	6	2515	8	2762	2	2885	4	2714
chr3	89184861	0	3	2472	0	1491	5	2518	2	2764	4	2878	4	2706
chr3	89184862	0	5	2467	2	1486	10	2509	9	2761	10	2876	10	2699
chr3	89184864	0	4	2473	2	1489	1	2522	3	2767	5	2883	3	2712
chr3	89184865	0	7	2469	3	1489	6	2517	9	2765	7	2882	9	2710
chr3	89184866	0	1	2473	1	1490	5	2517	2	2771	3	2888	5	2712
chr3	89184867	0	4	2466	2	1488	4	2516	5	2759	7	2877	7	2698
chr3	89184868	0	12	1784	18	2002	22	2139	17	2143	9	2357	8	2367
chr3	89184870	0	1	1864	9	2107	3	2251	2	2274	2	2471	2	2501

9	2475	1	1493	9	2487	6	2728	10	2846	7	2688
1	2471	2	1492	9	2490	12	2733	4	2853	11	2699
2	2465	0	1489	2	2488	4	2729	1	2846	1	2697
1568	1860	1697	2105	1902	2241	1801	2253	2116	2467	1982	2475
2127	2478	1239	1493	2055	2488	2242	2738	2401	2853	2251	2695
3	2475	3	1493	8	2492	13	2741	7	2857	8	2697
2	1857	4	2096	4	2235	2	2253	2	2469	9	2471
4	2439	2	1476	4	2478	7	2732	2	2844	1	2698
1380	1860	1348	2106	1682	2240	1426	2255	1905	2474	1594	2474
1946	2473	1173	1492	1876	2481	2028	2723	2163	2844	2011	2691
1246	1862	1381	2107	1469	2241	1503	2259	1685	2475	1638	2478
1799	2425	1236	1469	1687	2462	2201	2715	2005	2811	2180	2673
10	2344	3	1420	8	2308	5	2502	5	2630	6	2445
6	2295	7	1361	3	2350	6	2602	6	2675	10	2548
2	1823	3	2075	3	2209	0	2221	1	2431	6	2436
1	1863	5	2110	3	2242	2	2261	2	2474	6	2486
4	2469	3	1485	2	2490	7	2702	9	2813	6	2634
2	2425	4	1459	5	2468	3	2717	2	2817	4	2670
1446	1863	1473	2111	1715	2244	1591	2263	1940	2473	1763	2484
1817	2476	1114	1491	1774	2508	2016	2752	2025	2865	2002	2702
3	2471	3	1488	1	2506	5	2746	6	2861	5	2700
5	2477	2	1491	6	2513	6	2743	6	2868	4	2701
0	2352	6	1423	3	2475	10	2711	1	2802	20	2668
9	2450	11	1482	7	2430	34	2621	8	2758	33	2566
1604	1858	1715	2108	1949	2245	1851	2263	2176	2467	2038	2487
2163	2476	1098	1493	2125	2525	1998	2766	2422	2890	1969	2719
10	2462	4	1485	4	2510	5	2749	7	2876	2	2703
3	2473	16	1492	17	2504	64	2739	19	2870	45	2691
3	2472	1	1492	3	2519	6	2757	3	2886	9	2710
1579	1860	1627	2102	1896	2241	1748	2259	2097	2456	1953	2485
2093	2474	1258	1494	2062	2525	2253	2765	2369	2892	2212	2714
28	2475	3	1493	29	2524	8	2762	44	2893	8	2710
1595	1863	1643	2113	1916	2247	1731	2256	2135	2465	1982	2492
2059	2470	1303	1492	2056	2518	2382	2768	2342	2891	2328	2716
10	2316	0	1412	3	2438	5	2708	3	2807	4	2657
1618	1855	1694	2100	1936	2234	1816	2249	2152	2461	2022	2479
2121	2474	1278	1494	2086	2519	2321	2764	2383	2879	2258	2705
2	2476	4	1494	6	2521	8	2770	2	2887	4	2718
3	2475	0	1491	5	2523	2	2766	4	2882	4	2710
5	2472	2	1488	10	2519	9	2770	10	2886	10	2709
4	2477	2	1491	1	2523	3	2770	5	2888	3	2715
7	2476	3	1492	6	2523	9	2774	7	2889	9	2719
1	2474	1	1491	5	2522	2	2773	3	2891	5	2717
4	2470	2	1490	4	2520	5	2764	7	2884	7	2705
12	1796	18	2020	22	2161	17	2160	9	2366	8	2375
1	1865	9	2116	3	2254	2	2276	2	2473	2	2503

chr3	89184871	0	6	1860	43	2072	5	2246	59	2213	6	2471	48	2457
chr3	89184873	0	2	2467	0	1489	2	2515	2	2763	2	2877	2	2711
chr3	89184875	0	2	2461	3	1483	5	2497	1	2748	2	2869	1	2702
chr3	89184876	0	10	2459	1	1492	16	2496	4	2762	12	2867	3	2709
chr3	89184878	0	5	2465	2	1490	5	2510	2	2762	8	2874	3	2713
chr3	89184879	0	7	2462	2	1491	0	2516	6	2761	2	2879	2	2711
chr3	89184880	0	2	2467	5	1487	2	2507	14	2745	7	2869	7	2707
chr3	89184881	0	9	1741	4	1997	4	2136	5	2133	7	2351	9	2349
chr3	89184883	0	2	2456	0	1484	0	2509	3	2760	4	2868	3	2704
chr3	89184885	0	2	1859	4	2113	2	2256	5	2281	5	2477	1	2508
chr3	89184888	0	7	1855	3	2113	4	2255	2	2281	7	2473	1	2506
chr3	89184890	0	2	1861	3	2113	3	2257	5	2279	1	2483	4	2504
chr3	89184891	1	799	1060	921	1191	986	1273	979	1295	1054	1424	1092	1405
chr3	89184892	1	1265	1204	813	681	1152	1352	1447	1306	1381	1478	1413	1283
chr3	89184895	0	1	2465	7	1485	1	2493	8	2741	4	2850	15	2671
chr3	89184896	0	3	2464	2	1491	5	2493	7	2742	1	2855	1	2686
chr3	89184897	0	4	2457	2	1489	4	2491	6	2739	3	2852	3	2683
chr3	89184898	0	7	1843	2	2103	11	2234	2	2267	10	2462	3	2491
chr3	89184902	0	1	1852	1	2107	4	2240	2	2270	5	2447	6	2477
chr3	89184903	0	5	1845	1	2097	2	2234	4	2249	3	2440	4	2456
chr3	89184905	0	1	2464	0	1490	1	2481	0	2739	4	2836	3	2664
chr3	89184907	0	1	2464	0	1493	1	2478	6	2734	3	2832	6	2662
chr3	89184908	0	13	2452	1	1491	14	2468	4	2733	17	2817	3	2662
chr3	89184911	0	2	2461	1	1491	1	2468	2	2725	1	2827	2	2658
chr3	89184913	0	1	2460	6	1485	2	2473	3	2730	3	2823	1	2660
chr3	89184914	0	8	2401	5	1457	10	2431	9	2673	10	2792	10	2614
chr3	89184915	0	6	1837	2	2089	10	2235	6	2256	5	2453	3	2497
chr3	89184917	0	36	2427	3	1487	29	2439	5	2727	39	2782	7	2645
chr3	89184919	0	3	2461	3	1487	3	2462	5	2725	5	2816	3	2646
chr3	89184920	0	2	2460	1	1492	1	2461	5	2726	6	2815	9	2640
chr3	89184922	0	2	2461	49	1442	4	2458	84	2644	3	2818	85	2560
chr3	89184923	0	2	2461	2	1490	3	2461	3	2725	4	2817	2	2644
chr3	89184925	0	44	2419	2	1487	41	2423	2	2722	40	2780	3	2643
chr3	89184926	0	4	2455	2	1488	6	2456	1	2721	3	2815	5	2641
chr3	89184927	0	11	1847	17	2093	9	2252	28	2244	12	2456	23	2487
chr3	89184929	0	6	2456	3	1487	5	2460	4	2717	3	2817	1	2642
chr3	89184931	0	2	2456	2	1487	0	2459	1	2720	0	2819	2	2637
chr3	89184932	0	2	2457	11	1478	3	2458	27	2693	7	2807	15	2625
chr3	89184933	0	2	1842	1	2082	1	2205	3	2227	1	2416	5	2436
chr3	89184938	0	4	2444	8	1472	15	2437	18	2687	11	2794	12	2620
chr3	89184939	0	12	1814	3	2071	19	2186	0	2232	24	2412	3	2451
chr3	89184940	0	1	1834	5	2076	2	2203	6	2217	6	2406	2	2450
chr3	89184942	0	3	1795	3	2048	4	2208	0	2232	0	2431	0	2455
chr3	89184943	0	1	1845	3	2098	2	2234	1	2240	2	2428	1	2464
chr3	89184945	0	3	2455	0	1489	1	2455	4	2703	4	2803	1	2637
chr3	89184946	0	4	2446	3	1484	5	2448	3	2700	1	2800	2	2632

6	1866	43	2115	5	2251	59	2272	6	2477	48	2505
2	2469	0	1489	2	2517	2	2765	2	2879	2	2713
2	2463	3	1486	5	2502	1	2749	2	2871	1	2703
10	2469	1	1493	16	2512	4	2766	12	2879	3	2712
5	2470	2	1492	5	2515	2	2764	8	2882	3	2716
7	2469	2	1493	0	2516	6	2767	2	2881	2	2713
2	2469	5	1492	2	2509	14	2759	7	2876	7	2714
9	1750	4	2001	4	2140	5	2138	7	2358	9	2358
2	2458	0	1484	0	2509	3	2763	4	2872	3	2707
2	1861	4	2117	2	2258	5	2286	5	2482	1	2509
7	1862	3	2116	4	2259	2	2283	7	2480	1	2507
2	1863	3	2116	3	2260	5	2284	1	2484	4	2508
799	1859	921	2112	986	2259	979	2274	1054	2478	1092	2497
1265	2469	813	1494	1152	2504	1447	2753	1381	2859	1413	2696
1	2466	7	1492	1	2494	8	2749	4	2854	15	2686
3	2467	2	1493	5	2498	7	2749	1	2856	1	2687
4	2461	2	1491	4	2495	6	2745	3	2855	3	2686
7	1850	2	2105	11	2245	2	2269	10	2472	3	2494
1	1853	1	2108	4	2244	2	2272	5	2452	6	2483
5	1850	1	2098	2	2236	4	2253	3	2443	4	2460
1	2465	0	1490	1	2482	0	2739	4	2840	3	2667
1	2465	0	1493	1	2479	6	2740	3	2835	6	2668
13	2465	1	1492	14	2482	4	2737	17	2834	3	2665
2	2463	1	1492	1	2469	2	2727	1	2828	2	2660
1	2461	6	1491	2	2475	3	2733	3	2826	1	2661
8	2409	5	1462	10	2441	9	2682	10	2802	10	2624
6	1843	2	2091	10	2245	6	2262	5	2458	3	2500
36	2463	3	1490	29	2468	5	2732	39	2821	7	2652
3	2464	3	1490	3	2465	5	2730	5	2821	3	2649
2	2462	1	1493	1	2462	5	2731	6	2821	9	2649
2	2463	49	1491	4	2462	84	2728	3	2821	85	2645
2	2463	2	1492	3	2464	3	2728	4	2821	2	2646
44	2463	2	1489	41	2464	2	2724	40	2820	3	2646
4	2459	2	1490	6	2462	1	2722	3	2818	5	2646
11	1858	17	2110	9	2261	28	2272	12	2468	23	2510
6	2462	3	1490	5	2465	4	2721	3	2820	1	2643
2	2458	2	1489	0	2459	1	2721	0	2819	2	2639
2	2459	11	1489	3	2461	27	2720	7	2814	15	2640
2	1844	1	2083	1	2206	3	2230	1	2417	5	2441
4	2448	8	1480	15	2452	18	2705	11	2805	12	2632
12	1826	3	2074	19	2205	0	2232	24	2436	3	2454
1	1835	5	2081	2	2205	6	2223	6	2412	2	2452
3	1798	3	2051	4	2212	0	2232	0	2431	0	2455
1	1846	3	2101	2	2236	1	2241	2	2430	1	2465
3	2458	0	1489	1	2456	4	2707	4	2807	1	2638
4	2450	3	1487	5	2453	3	2703	1	2801	2	2634

chr3	89184949	0	12	1698	4	1976	21	2114	2	2127	20	2347	2	2376
chr3	89184951	0	3	2409	1	1474	1	2443	3	2696	4	2787	5	2619
chr3	89184953	1	1421	431	1319	784	1724	516	1436	836	1975	489	1565	934
chr3	89184954	1	1423	988	1019	455	1399	1043	1754	938	1638	1152	1707	915
chr3	89184955	0	14	2396	2	1472	26	2417	1	2693	16	2774	2	2621
chr3	89184956	0	7	2403	4	1471	11	2430	7	2686	18	2770	4	2620
chr3	89184957	0	1	1847	4	2096	5	2232	5	2262	7	2452	3	2492
chr3	89184959	1	1571	273	1680	417	1889	333	1798	459	2054	385	2009	475
chr3	89184960	1	1883	515	1066	399	1842	582	1981	695	2140	632	1884	714
chr3	89184961	0	10	1840	2	2100	6	2232	3	2262	10	2446	9	2478
chr3	89184963	0	2	1838	0	2098	4	2230	3	2261	1	2453	1	2488
chr3	89184965	0	8	1840	2	2100	14	2226	1	2267	13	2441	2	2485
chr3	89184967	0	4	2387	1	1458	3	2422	3	2670	2	2762	3	2593
chr3	89184969	0	2	1834	1	2091	2	2232	2	2263	2	2441	2	2472
chr3	89184972	0	13	1828	10	2089	11	2225	11	2248	13	2432	7	2479
chr3	89184973	0	1	1839	6	2079	1	2225	5	2257	3	2432	11	2470
chr3	89184975	0	0	1844	2	2083	3	2228	1	2252	5	2416	3	2470
chr3	89184978	1	1657	188	1770	326	1969	265	1883	375	2181	259	2094	387
chr3	89184979	1	1992	414	1151	317	1931	497	2110	565	2225	551	2026	573
chr3	89184980	0	1	2404	1	1467	3	2426	4	2673	2	2774	3	2596
chr3	89184981	0	8	2398	2	1466	7	2422	8	2667	6	2767	8	2591
chr3	89184982	0	3	2389	2	1456	4	2406	5	2653	6	2747	1	2579
chr3	89184983	0	1	1836	6	2088	1	2226	5	2249	3	2431	8	2472
chr3	89184985	0	0	2403	0	1468	2	2422	2	2671	3	2760	0	2593
chr3	89184986	0	1	2397	3	1464	3	2419	2	2666	2	2765	5	2585
chr3	89184987	0	0	2404	0	1468	0	2428	1	2676	0	2772	0	2597
chr3	89184988	0	0	2401	0	1465	0	2426	0	2672	1	2767	0	2592
chr3	89184989	0	0	2355	1	1433	0	2390	0	2643	0	2737	0	2546
chr3	89184990	0	1	1813	30	2044	3	2209	31	2211	3	2407	46	2422
chr3	89184993	0	4	1810	0	2069	2	2204	2	2249	5	2405	3	2473
chr3	89184994	0	1	1812	3	2063	0	2209	2	2247	1	2408	1	2470
chr3	89184996	0	1	2402	0	1466	1	2412	4	2659	2	2751	4	2578
chr3	89184997	0	2	1743	1	1957	3	2135	0	2200	1	2310	1	2410
chr3	89184998	0	1	1735	1	1951	0	2135	1	2193	0	2300	2	2406
chr3	89184999	0	2	1737	0	1956	2	2136	0	2200	0	2310	0	2406
chr3	89185001	0	2	1731	0	1956	1	2127	1	2193	0	2302	1	2401
chr3	89185003	0	6	2320	2	1425	2	2353	6	2583	2	2678	3	2490
chr3	89185004	0	5	1727	1	1946	1	2130	0	2191	4	2296	2	2393
chr3	89185005	0	0	1726	1	1936	2	2097	3	2172	1	2269	3	2379
chr3	89185008	0	7	1971	5	1199	2	1682	3	1797	2	1912	3	1778
chr3	89185015	0	0	1412	1	1594	3	1608	1	1762	2	1724	2	1926
chr3	89185017	0	1	1381	0	1571	0	1559	1	1708	2	1667	1	1861
chr3	89185018	0	0	1265	1	1443	1	1519	1	1676	0	1604	0	1810
chr3	89185022	0	0	1344	1	1535	0	1518	0	1659	1	1635	1	1805
chr3	89679038	0	4	2819	0	4473	3	2291	2	6331	2	2418	3	5683
chr3	89679041	0	1	2970	4	4668	1	2519	3	6868	1	2632	1	6146

12	1710	4	1980	21	2135	2	2129	20	2367	2	2378
3	2412	1	1475	1	2444	3	2699	4	2791	5	2624
1421	1852	1319	2103	1724	2240	1436	2272	1975	2464	1565	2499
1423	2411	1019	1474	1399	2442	1754	2692	1638	2790	1707	2622
14	2410	2	1474	26	2443	1	2694	16	2790	2	2623
7	2410	4	1475	11	2441	7	2693	18	2788	4	2624
1	1848	4	2100	5	2237	5	2267	7	2459	3	2495
1571	1844	1680	2097	1889	2222	1798	2257	2054	2439	2009	2484
1883	2398	1066	1465	1842	2424	1981	2676	2140	2772	1884	2598
10	1850	2	2102	6	2238	3	2265	10	2456	9	2487
2	1840	0	2098	4	2234	3	2264	1	2454	1	2489
8	1848	2	2102	14	2240	1	2268	13	2454	2	2487
4	2391	1	1459	3	2425	3	2673	2	2764	3	2596
2	1836	1	2092	2	2234	2	2265	2	2443	2	2474
13	1841	10	2099	11	2236	11	2259	13	2445	7	2486
1	1840	6	2085	1	2226	5	2262	3	2435	11	2481
0	1844	2	2085	3	2231	1	2253	5	2421	3	2473
1657	1845	1770	2096	1969	2234	1883	2258	2181	2440	2094	2481
1992	2406	1151	1468	1931	2428	2110	2675	2225	2776	2026	2599
1	2405	1	1468	3	2429	4	2677	2	2776	3	2599
8	2406	2	1468	7	2429	8	2675	6	2773	8	2599
3	2392	2	1458	4	2410	5	2658	6	2753	1	2580
1	1837	6	2094	1	2227	5	2254	3	2434	8	2480
0	2403	0	1468	2	2424	2	2673	3	2763	0	2593
1	2398	3	1467	3	2422	2	2668	2	2767	5	2590
0	2404	0	1468	0	2428	1	2677	0	2772	0	2597
0	2401	0	1465	0	2426	0	2672	1	2768	0	2592
0	2355	1	1434	0	2390	0	2643	0	2737	0	2546
1	1814	30	2074	3	2212	31	2242	3	2410	46	2468
4	1814	0	2069	2	2206	2	2251	5	2410	3	2476
1	1813	3	2066	0	2209	2	2249	1	2409	1	2471
1	2403	0	1466	1	2413	4	2663	2	2753	4	2582
2	1745	1	1958	3	2138	0	2200	1	2311	1	2411
1	1736	1	1952	0	2135	1	2194	0	2300	2	2408
2	1739	0	1956	2	2138	0	2200	0	2310	0	2406
2	1733	0	1956	1	2128	1	2194	0	2302	1	2402
6	2326	2	1427	2	2355	6	2589	2	2680	3	2493
5	1732	1	1947	1	2131	0	2191	4	2300	2	2395
0	1726	1	1937	2	2099	3	2175	1	2270	3	2382
7	1978	5	1204	2	1684	3	1800	2	1914	3	1781
0	1412	1	1595	3	1611	1	1763	2	1726	2	1928
1	1382	0	1571	0	1559	1	1709	2	1669	1	1862
0	1265	1	1444	1	1520	1	1677	0	1604	0	1810
0	1344	1	1536	0	1518	0	1659	1	1636	1	1806
4	2823	0	4473	3	2294	2	6333	2	2420	3	5686
1	2971	4	4672	1	2520	3	6871	1	2633	1	6147

chr3	89679051	0	2	3076	0	4833	1	2751	3	7272	0	2870	1	6513
chr3	89679054	0	3	3099	7	4876	2	2798	6	7381	3	2928	3	6627
chr3	89679072	1	1449	1919	3139	2381	1437	1861	4403	4118	1579	1968	4190	3675
chr3	89679075	0	2	3322	26	5424	7	3277	34	8407	11	3490	41	7723
chr3	89679079	0	9	3388	23	5534	6	3359	48	8597	11	3585	35	7957
chr3	89679081	0	5	3383	35	5516	3	3365	55	8596	2	3605	55	7939
chr3	89679091	0	12	3397	6	5571	25	3363	8	8693	32	3596	4	8033
chr3	89679103	0	4	3433	8	5627	7	3437	19	8771	9	3676	14	8097
chr3	89679107	1	625	2817	2118	3534	675	2786	3112	5706	765	2931	3029	5110
chr3	89679108	0	3	3439	6	5647	5	3459	12	8805	2	3696	10	8125
chr3	89679109	0	2	3440	12	5643	2	3463	11	8809	1	3701	11	8126
chr3	89679117	0	0	72	7	60126	9	77263	30	1E+05	10	91477	20	1E+05
chr3	89679123	0	1	3445	3	5656	2	3480	7	8838	6	3723	9	8161
chr3	89679126	0	0	73	13	60603	9	77826	15	1E+05	12	92102	20	1E+05
chr3	89679132	0	0	73	5	60564	7	77777	12	1E+05	10	91981	12	1E+05
chr3	89679136	0	4	3437	8	5629	6	3484	17	8809	3	3733	7	8165
chr3	89679137	0	0	70	20	59046	19	76064	28	1E+05	10	90311	27	1E+05
chr3	89679141	0	0	73	11	60552	8	77802	16	1E+05	6	92056	13	1E+05
chr3	89679142	0	0	73	255	60426	163	77774	606	1E+05	184	92031	754	1E+05
chr3	89679144	0	1	3451	7	5657	2	3516	15	8858	5	3766	7	8198
chr3	89679146	0	0	73	64	60611	118	77811	168	1E+05	133	92081	154	1E+05
chr3	89679148	0	1	3453	1	5665	1	3518	6	8867	2	3768	6	8200
chr3	89679149	0	6	3445	8	5651	2	3515	17	8854	5	3763	16	8190
chr3	89679150	0	0	73	443	59852	226	77190	889	1E+05	260	91422	983	1E+05
chr3	89679155	1	28	45	10997	49662	1158	76702	21339	91493	1494	90659	23382	1E+05
chr3	89679156	1	56	3399	1456	4201	65	3454	2109	6761	80	3692	2037	6169
chr3	89679158	0	1	72	24	60575	166	77666	70	1E+05	167	91958	98	1E+05
chr3	89679162	1	37	35	20522	39960	9773	67818	39084	73415	11797	80064	43447	82326
chr3	89679163	1	173	3267	1633	4011	161	3345	2262	6576	203	3554	2255	5922
chr3	89679164	0	0	73	101	60408	211	77453	182	1E+05	289	91642	205	1E+05
chr3	89679165	0	0	73	55	60633	160	77778	106	1E+05	185	92043	154	1E+05
chr3	89679167	0	0	73	76	60578	55	77830	145	1E+05	67	92108	160	1E+05
chr3	89679168	0	1	72	322	60331	114	77792	549	1E+05	127	92064	605	1E+05
chr3	89679169	0	3	70	1004	59689	551	77406	1866	1E+05	608	91641	2033	1E+05
chr3	89679171	0	0	73	53	60644	437	77519	117	1E+05	486	91757	117	1E+05
chr3	89679173	1	26	46	13784	46397	1079	76204	25327	86587	1453	90034	27883	97301
chr3	89679174	1	62	3391	1290	4372	45	3469	1719	7151	74	3694	1696	6502
chr3	89679176	0	39	3406	17	5634	51	3458	20	8824	59	3703	28	8138
chr3	89679177	0	0	73	409	60159	266	77536	848	1E+05	291	91821	873	1E+05
chr3	89679180	0	0	73	101	60568	122	77788	189	1E+05	166	92043	164	1E+05
chr3	89679181	0	0	73	219	60468	167	77781	424	1E+05	215	92021	457	1E+05
chr3	89679182	0	0	73	94	60602	159	77785	168	1E+05	162	92078	199	1E+05
chr3	89679184	0	17	3432	11	5646	9	3485	14	8787	15	3738	19	8112
chr3	89679186	1	26	47	14177	46478	1896	76018	26412	86467	2376	89839	28922	97249
chr3	89679187	1	53	3396	1084	4565	46	3464	1494	7352	64	3692	1431	6739
chr3	89679188	0	0	73	248	60375	182	77689	490	1E+05	200	91978	541	1E+05

2	3078	0	4833	1	2752	3	7275	0	2870	1	6514
3	3102	7	4883	2	2800	6	7387	3	2931	3	6630
1449	3368	3139	5520	1437	3298	4403	8521	1579	3547	4190	7865
2	3324	26	5450	7	3284	34	8441	11	3501	41	7764
9	3397	23	5557	6	3365	48	8645	11	3596	35	7992
5	3388	35	5551	3	3368	55	8651	2	3607	55	7994
12	3409	6	5577	25	3388	8	8701	32	3628	4	8037
4	3437	8	5635	7	3444	19	8790	9	3685	14	8111
625	3442	2118	5652	675	3461	3112	8818	765	3696	3029	8139
3	3442	6	5653	5	3464	12	8817	2	3698	10	8135
2	3442	12	5655	2	3465	11	8820	1	3702	11	8137
0	72	7	60133	9	77272	30	1E+05	10	91487	20	1E+05
1	3446	3	5659	2	3482	7	8845	6	3729	9	8170
0	73	13	60616	9	77835	15	1E+05	12	92114	20	1E+05
0	73	5	60569	7	77784	12	1E+05	10	91991	12	1E+05
4	3441	8	5637	6	3490	17	8826	3	3736	7	8172
0	70	20	59066	19	76083	28	1E+05	10	90321	27	1E+05
0	73	11	60563	8	77810	16	1E+05	6	92062	13	1E+05
0	73	255	60681	163	77937	606	1E+05	184	92215	754	1E+05
1	3452	7	5664	2	3518	15	8873	5	3771	7	8205
0	73	64	60675	118	77929	168	1E+05	133	92214	154	1E+05
1	3454	1	5666	1	3519	6	8873	2	3770	6	8206
6	3451	8	5659	2	3517	17	8871	5	3768	16	8206
0	73	443	60295	226	77416	889	1E+05	260	91682	983	1E+05
28	73	10997	60659	1158	77860	####	1E+05	1494	92153	23382	1E+05
56	3455	1456	5657	65	3519	2109	8870	80	3772	2037	8206
1	73	24	60599	166	77832	70	1E+05	167	92125	98	1E+05
37	72	20522	60482	9773	77591	####	1E+05	11797	91861	43447	1E+05
173	3440	1633	5644	161	3506	2262	8838	203	3757	2255	8177
0	73	101	60509	211	77664	182	1E+05	289	91931	205	1E+05
0	73	55	60688	160	77938	106	1E+05	185	92228	154	1E+05
0	73	76	60654	55	77885	145	1E+05	67	92175	160	1E+05
1	73	322	60653	114	77906	549	1E+05	127	92191	605	1E+05
3	73	1004	60693	551	77957	1866	1E+05	608	92249	2033	1E+05
0	73	53	60697	437	77956	117	1E+05	486	92243	117	1E+05
26	72	13784	60181	1079	77283	####	1E+05	1453	91487	27883	1E+05
62	3453	1290	5662	45	3514	1719	8870	74	3768	1696	8198
39	3445	17	5651	51	3509	20	8844	59	3762	28	8166
0	73	409	60568	266	77802	848	1E+05	291	92112	873	1E+05
0	73	101	60669	122	77910	189	1E+05	166	92209	164	1E+05
0	73	219	60687	167	77948	424	1E+05	215	92236	457	1E+05
0	73	94	60696	159	77944	168	1E+05	162	92240	199	1E+05
17	3449	11	5657	9	3494	14	8801	15	3753	19	8131
26	73	14177	60655	1896	77914	####	1E+05	2376	92215	28922	1E+05
53	3449	1084	5649	46	3510	1494	8846	64	3756	1431	8170
0	73	248	60623	182	77871	490	1E+05	200	92178	541	1E+05

chr3	89679190	0	3	3451	4	5659	3	3513	7	8857	6	3760	4	8183
chr3	89679192	0	4	3451	10	5653	1	3516	7	8857	4	3763	12	8177
chr3	89679193	0	10	3443	12	5651	10	3506	14	8841	6	3762	9	8175
chr3	89679194	0	0	73	163	60449	121	77749	313	1E+05	131	92022	358	1E+05
chr3	89679196	0	0	73	254	60406	61	77840	434	1E+05	88	92121	477	1E+05
chr3	89679198	0	6	3427	9	5641	10	3500	10	8809	9	3743	14	8142
chr3	89679199	0	0	73	152	59922	153	77098	288	1E+05	161	91335	324	1E+05
chr3	89679200	1	26	47	15369	45308	1306	76626	28340	84550	1683	90538	31306	94892
chr3	89679201	1	46	3396	1260	4387	70	3436	1787	7045	84	3676	1761	6400
chr3	89679202	0	2	3397	6	5562	1	3466	6	8765	4	3720	10	8061
chr3	89679203	0	0	73	93	60371	113	77589	139	1E+05	126	91854	175	1E+05
chr3	89679210	0	0	73	169	60270	53	77618	290	1E+05	46	91798	285	1E+05
chr3	89679212	0	0	73	7	60575	14	77776	19	1E+05	14	92074	19	1E+05
chr3	89679213	0	0	73	6	60566	5	77700	14	1E+05	5	92035	11	1E+05
chr3	89679214	0	0	73	3	60688	5	77931	10	1E+05	8	92212	7	1E+05
chr3	89679216	0	0	3354	0	5532	1	3407	1	8653	0	3650	3	7957
chr3	89679218	0	0	72	7	60474	9	77625	9	1E+05	11	91817	14	1E+05
chr3	89679220	0	0	73	6	60637	8	77862	14	1E+05	3	92162	11	1E+05
chr3	89679224	0	5	3148	12	5256	9	3193	21	8287	12	3424	9	7579
chr3	89679226	0	1	3163	2	5294	2	3100	5	7981	3	3346	6	7309
chr3	89679232	0	0	73	4	60497	8	77729	11	1E+05	7	92031	13	1E+05
chr4	44628612	0	0	99	1	225	0	1368	2	1329	0	1078	2	1069
chr4	44628613	0	0	98	0	223	3	1392	2	1359	0	1087	0	1124
chr4	44628615	0	0	100	0	230	1	1430	1	1400	1	1110	1	1145
chr4	44628620	0	0	100	0	227	4	1403	1	1378	2	1105	2	1130
chr4	44628623	0	0	101	0	230	0	1452	0	1417	1	1127	1	1160
chr4	44628630	0	0	102	0	227	2	1443	1	1407	1	1128	1	1150
chr4	44628631	0	0	104	0	230	1	1452	0	1417	0	1133	0	1160
chr4	44628632	0	0	104	1	229	2	1446	4	1410	1	1130	1	1153
chr4	44628640	0	0	104	0	230	2	1453	5	1416	2	1137	4	1158
chr4	44628641	0	1	103	1	229	2	1453	4	1412	2	1136	5	1156
chr4	44628642	0	0	104	0	230	0	1457	1	1421	2	1138	1	1162
chr4	44628643	1	76	28	178	52	1105	352	1070	352	849	288	897	266
chr4	44628646	1	95	9	223	7	1323	136	1342	80	1027	110	1107	56
chr4	44628652	0	0	104	0	228	1	1436	5	1400	1	1130	2	1149
chr4	44628657	0	0	104	0	229	0	1452	2	1412	0	1141	0	1161
chr4	44628659	0	1	102	0	228	1	1454	2	1407	1	1139	3	1158
chr4	44628661	0	0	107	0	230	0	1463	1	1420	2	1145	0	1165
chr4	44628663	0	0	108	2	228	1	1466	1	1422	2	1148	1	1163
chr4	44628665	0	1	107	1	229	2	1464	3	1420	1	1148	1	1162
chr4	44628666	0	0	109	0	231	1	1465	3	1419	0	1152	0	1165
chr4	44628667	0	0	109	0	231	0	1467	1	1421	0	1152	1	1165
chr4	44628670	0	1	110	0	231	2	1466	1	1416	1	1153	1	1165
chr4	44628671	0	0	110	0	231	2	1465	2	1415	1	1151	2	1163
chr4	44628679	0	0	109	0	230	9	1457	2	1416	6	1146	4	1161
chr4	44628680	0	0	111	1	230	1	1467	1	1423	3	1154	4	1164

3	3454	4	5663	3	3516	7	8864	6	3766	4	8187
4	3455	10	5663	1	3517	7	8864	4	3767	12	8189
10	3453	12	5663	10	3516	14	8855	6	3768	9	8184
0	73	163	60612	121	77870	313	1E+05	131	92153	358	1E+05
0	73	254	60660	61	77901	434	1E+05	88	92209	477	1E+05
6	3433	9	5650	10	3510	10	8819	9	3752	14	8156
0	73	152	60074	153	77251	288	1E+05	161	91496	324	1E+05
26	73	15369	60677	1306	77932	####	1E+05	1683	92221	31306	1E+05
46	3442	1260	5647	70	3506	1787	8832	84	3760	1761	8161
2	3399	6	5568	1	3467	6	8771	4	3724	10	8071
0	73	93	60464	113	77702	139	1E+05	126	91980	175	1E+05
0	73	169	60439	53	77671	290	1E+05	46	91844	285	1E+05
0	73	7	60582	14	77790	19	1E+05	14	92088	19	1E+05
0	73	6	60572	5	77705	14	1E+05	5	92040	11	1E+05
0	73	3	60691	5	77936	10	1E+05	8	92220	7	1E+05
0	3354	0	5532	1	3408	1	8654	0	3650	3	7960
0	72	7	60481	9	77634	9	1E+05	11	91828	14	1E+05
0	73	6	60643	8	77870	14	1E+05	3	92165	11	1E+05
5	3153	12	5268	9	3202	21	8308	12	3436	9	7588
1	3164	2	5296	2	3102	5	7986	3	3349	6	7315
0	73	4	60501	8	77737	11	1E+05	7	92038	13	1E+05
0	99	1	226	0	1368	2	1331	0	1078	2	1071
0	98	0	223	3	1395	2	1361	0	1087	0	1124
0	100	0	230	1	1431	1	1401	1	1111	1	1146
0	100	0	227	4	1407	1	1379	2	1107	2	1132
0	101	0	230	0	1452	0	1417	1	1128	1	1161
0	102	0	227	2	1445	1	1408	1	1129	1	1151
0	104	0	230	1	1453	0	1417	0	1133	0	1160
0	104	1	230	2	1448	4	1414	1	1131	1	1154
0	104	0	230	2	1455	5	1421	2	1139	4	1162
1	104	1	230	2	1455	4	1416	2	1138	5	1161
0	104	0	230	0	1457	1	1422	2	1140	1	1163
76	104	178	230	1105	1457	1070	1422	849	1137	897	1163
95	104	223	230	1323	1459	1342	1422	1027	1137	1107	1163
0	104	0	228	1	1437	5	1405	1	1131	2	1151
0	104	0	229	0	1452	2	1414	0	1141	0	1161
1	103	0	228	1	1455	2	1409	1	1140	3	1161
0	107	0	230	0	1463	1	1421	2	1147	0	1165
0	108	2	230	1	1467	1	1423	2	1150	1	1164
1	108	1	230	2	1466	3	1423	1	1149	1	1163
0	109	0	231	1	1466	3	1422	0	1152	0	1165
0	109	0	231	0	1467	1	1422	0	1152	1	1166
1	111	0	231	2	1468	1	1417	1	1154	1	1166
0	110	0	231	2	1467	2	1417	1	1152	2	1165
0	109	0	230	9	1466	2	1418	6	1152	4	1165
0	111	1	231	1	1468	1	1424	3	1157	4	1168

chr4	44628684	1	99	12	201	30	1286	186	1244	182	1015	145	1034	136
chr4	44628686	0	0	111	1	230	3	1467	4	1421	1	1157	2	1168
chr4	44628687	0	0	111	1	230	5	1467	3	1421	4	1155	2	1169
chr4	44628690	0	0	111	1	230	3	1470	3	1422	3	1156	2	1170
chr4	44628692	0	0	110	0	231	2	1471	1	1426	1	1158	0	1173
chr4	44628694	0	0	112	0	231	3	1473	1	1424	5	1155	0	1172
chr4	44628695	0	0	112	1	229	3	1472	2	1424	1	1159	1	1172
chr4	44628697	0	1	111	1	230	2	1472	0	1423	1	1157	1	1170
chr4	44628698	0	0	112	0	232	1	1476	1	1425	1	1156	1	1170
chr4	44628700	0	0	113	0	232	3	1474	1	1425	1	1158	1	1170
chr4	44628701	0	0	113	0	232	4	1474	8	1418	3	1156	3	1167
chr4	44628702	0	0	113	0	232	0	1477	3	1422	2	1155	1	1169
chr4	44628703	0	0	121	2	230	1	1472	1	1423	0	1154	1	1168
chr4	44628706	0	0	121	0	231	2	1470	1	1419	1	1157	3	1167
chr4	44628707	0	0	121	0	232	4	1475	1	1425	1	1158	4	1169
chr4	44628708	0	1	120	0	232	6	1469	1	1421	4	1154	0	1169
chr4	44628712	0	0	122	0	232	1	1481	2	1426	0	1164	1	1174
chr4	44628716	0	1	127	1	230	3	1467	1	1418	1	1154	1	1166
chr4	44628717	0	0	129	0	234	1	1486	1	1431	0	1167	0	1174
chr4	44628737	0	0	131	1	233	9	1480	4	1430	3	1182	4	1172
chr4	44628738	0	0	131	0	234	1	1497	3	1434	0	1187	0	1178
chr4	44628740	0	0	132	0	234	1	1497	0	1436	0	1186	0	1180
chr4	44628746	0	1	130	1	234	5	1494	4	1434	1	1186	2	1181
chr4	44628750	0	0	133	3	232	6	1495	12	1426	5	1182	3	1179
chr4	44628751	0	0	132	0	234	6	1491	2	1433	2	1185	1	1183
chr4	44628786	0	0	74	1	42	4	424	3	495	6	369	4	391
chr4	44628787	1	129	27	202	30	1411	107	1280	164	1102	102	1064	130
chr4	44628795	0	0	155	0	234	5	1510	2	1441	1	1203	2	1199
chr4	44628796	0	1	154	1	233	3	1514	5	1429	3	1195	5	1196
chr4	44628797	0	0	159	0	233	6	1487	4	1424	1	1183	0	1183
chr4	44628798	0	0	160	0	235	1	1523	1	1436	2	1208	2	1199
chr4	44628799	1	136	24	209	26	1387	141	1343	105	1090	122	1123	81
chr4	44628804	0	0	159	0	232	2	1513	4	1446	4	1205	4	1191
chr4	44628807	0	0	159	0	235	5	1506	3	1447	1	1202	2	1197
chr4	44628819	0	0	163	0	236	2	1529	2	1458	1	1206	1	1206
chr4	44628821	0	0	172	0	236	2	1532	4	1458	4	1198	0	1204
chr4	44628828	0	1	170	0	236	3	1540	5	1456	2	1211	4	1204
chr4	44628832	1	155	16	215	21	1475	60	1315	150	1161	53	1099	114
chr4	44628834	0	2	169	0	233	5	1524	7	1452	3	1205	2	1206
chr4	44628843	0	0	173	0	236	3	1534	1	1465	4	1213	1	1212
chr4	44628845	0	0	171	0	235	1	1526	9	1445	1	1204	6	1193
chr4	44628851	0	0	174	0	236	4	1525	2	1465	2	1209	2	1209
chr4	44628852	0	2	172	4	228	4	1510	7	1448	2	1199	10	1195
chr4	44628856	0	5	167	0	234	27	1470	0	1447	19	1158	4	1189
chr4	44628862	0	0	170	0	232	6	1479	6	1424	1	1179	0	1177
chr4	44628869	0	0	172	1	233	2	1526	5	1457	8	1201	0	1209

99	111	201	231	1286	1472	1244	1426	1015	1160	1034	1170
0	111	1	231	3	1470	4	1425	1	1158	2	1170
0	111	1	231	5	1472	3	1424	4	1159	2	1171
0	111	1	231	3	1473	3	1425	3	1159	2	1172
0	110	0	231	2	1473	1	1427	1	1159	0	1173
0	112	0	231	3	1476	1	1425	5	1160	0	1172
0	112	1	230	3	1475	2	1426	1	1160	1	1173
1	112	1	231	2	1474	0	1423	1	1158	1	1171
0	112	0	232	1	1477	1	1426	1	1157	1	1171
0	113	0	232	3	1477	1	1426	1	1159	1	1171
0	113	0	232	4	1478	8	1426	3	1159	3	1170
0	113	0	232	0	1477	3	1425	2	1157	1	1170
0	121	2	232	1	1473	1	1424	0	1154	1	1169
0	121	0	231	2	1472	1	1420	1	1158	3	1170
0	121	0	232	4	1479	1	1426	1	1159	4	1173
1	121	0	232	6	1475	1	1422	4	1158	0	1169
0	122	0	232	1	1482	2	1428	0	1164	1	1175
1	128	1	231	3	1470	1	1419	1	1155	1	1167
0	129	0	234	1	1487	1	1432	0	1167	0	1174
0	131	1	234	9	1489	4	1434	3	1185	4	1176
0	131	0	234	1	1498	3	1437	0	1187	0	1178
0	132	0	234	1	1498	0	1436	0	1186	0	1180
1	131	1	235	5	1499	4	1438	1	1187	2	1183
0	133	3	235	6	1501	12	1438	5	1187	3	1182
0	132	0	234	6	1497	2	1435	2	1187	1	1184
0	74	1	43	4	428	3	498	6	375	4	395
129	156	202	232	1411	1518	1280	1444	1102	1204	1064	1194
0	155	0	234	5	1515	2	1443	1	1204	2	1201
1	155	1	234	3	1517	5	1434	3	1198	5	1201
0	159	0	233	6	1493	4	1428	1	1184	0	1183
0	160	0	235	1	1524	1	1437	2	1210	2	1201
136	160	209	235	1387	1528	1343	1448	1090	1212	1123	1204
0	159	0	232	2	1515	4	1450	4	1209	4	1195
0	159	0	235	5	1511	3	1450	1	1203	2	1199
0	163	0	236	2	1531	2	1460	1	1207	1	1207
0	172	0	236	2	1534	4	1462	4	1202	0	1204
1	171	0	236	3	1543	5	1461	2	1213	4	1208
155	171	215	236	1475	1535	1315	1465	1161	1214	1099	1213
2	171	0	233	5	1529	7	1459	3	1208	2	1208
0	173	0	236	3	1537	1	1466	4	1217	1	1213
0	171	0	235	1	1527	9	1454	1	1205	6	1199
0	174	0	236	4	1529	2	1467	2	1211	2	1211
2	174	4	232	4	1514	7	1455	2	1201	10	1205
5	172	0	234	27	1497	0	1447	19	1177	4	1193
0	170	0	232	6	1485	6	1430	1	1180	0	1177
0	172	1	234	2	1528	5	1462	8	1209	0	1209

chr4	44628871	0	1	173	1	233	2	1529	2	1465	3	1207	3	1208
chr4	44628876	0	0	172	0	233	2	1523	2	1463	2	1200	7	1205
chr4	44628882	0	1	171	0	232	6	1518	5	1460	6	1197	0	1210
chr4	44628883	0	1	171	0	232	5	1514	7	1456	1	1201	3	1210
chr4	44628889	0	2	169	1	231	5	1512	6	1456	4	1194	2	1211
chr4	44628890	0	1	171	1	231	2	1520	4	1459	1	1194	3	1206
chr4	44628891	0	0	172	0	232	1	1517	2	1462	0	1194	1	1208
chr4	44628893	0	1	170	0	232	2	1514	5	1454	2	1192	2	1208
chr4	44628898	0	0	168	0	229	0	1502	0	1446	1	1191	0	1205
chr4	44628906	0	1	170	0	231	0	1513	1	1456	2	1193	2	1204
chr4	44628908	1	161	9	215	16	1367	147	1219	238	1081	109	1015	191
chr4	44628911	0	0	170	0	230	0	1499	0	1448	0	1178	1	1202
chr4	44628912	0	0	170	0	228	0	1504	0	1448	1	1179	0	1201
chr4	44628918	0	0	170	0	231	1	1503	0	1453	0	1181	1	1201
chr4	44628931	0	0	166	1	224	1	1457	0	1424	1	1141	1	1166

1	174	1	234	2	1531	2	1467	3	1210	3	1211
0	172	0	233	2	1525	2	1465	2	1202	7	1212
1	172	0	232	6	1524	5	1465	6	1203	0	1210
1	172	0	232	5	1519	7	1463	1	1202	3	1213
2	171	1	232	5	1517	6	1462	4	1198	2	1213
1	172	1	232	2	1522	4	1463	1	1195	3	1209
0	172	0	232	1	1518	2	1464	0	1194	1	1209
1	171	0	232	2	1516	5	1459	2	1194	2	1210
0	168	0	229	0	1502	0	1446	1	1192	0	1205
1	171	0	231	0	1513	1	1457	2	1195	2	1206
161	170	215	231	1367	1514	1219	1457	1081	1190	1015	1206
0	170	0	230	0	1499	0	1448	0	1178	1	1203
0	170	0	228	0	1504	0	1448	1	1180	0	1201
0	170	0	231	1	1504	0	1453	0	1181	1	1202
0	166	1	225	1	1458	0	1424	1	1142	1	1167

Lux estimates

					INDIVIDUAL REPLICATES																										
					dp1			dp2			dp3			sp1			sp2			sp3			naïve1			naïve2			naïve3		
Chrom	Position	ID	Nuc	CpG	p("C"	p("5n	p("5h	p("C"	p("5n	p("5h	p("C"	p("5n	p("5h	p("C"	p("5n	p("5h	p("C"	p("5n	p("5h	p("C"	p("5n	p("5h	p("C"	p("5n	p("5h	p("C"	p("5n	p("5h	p("C"	p("5n	p("5hmC")
chr2	11124412	1	C	0	1	0	0	1	0	0	1	0	0	1	0	0	1	0	0	1	0	0	1	0	0	1	0	0	1	0	0
chr2	11124413	2	C	0	1	0	0	1	0	0	1	0	0	1	0	0	1	0	0	1	0	0	1	0	0	1	0	0	1	0	0
chr2	11124414	3	C	0	1	0	0	1	0	0	1	0	0	1	0	0	1	0	0	1	0	0	1	0	0	1	0	0	1	0	0
chr2	11124418	4	C	0	1	0	0	1	0	0	1	0	0	1	0	0	1	0	0	1	0	0	1	0	0	1	0	0	1	0	0
chr2	11124422	5	C	0	1	0	0	1	0	0	1	0	0	1	0	0	1	0	0	1	0	0	1	0	0	1	0	0	1	0	0
chr2	11124423	6	C	0	1	0	0	1	0	0	1	0	0	1	0	0	1	0	0	1	0	0	1	0	0	1	0	0	1	0	0
chr2	11124429	7	C	0	1	0	0	1	0	0	1	0	0	1	0	0	1	0	0	1	0	0	1	0	0	1	0	0	1	0	0
chr2	11124431	8	C	0	1	0	0	1	0	0	1	0	0	1	0	0	1	0	0	1	0	0	1	0	0	1	0	0	1	0	0
chr2	11124435	9	C	0	1	0	0	1	0	0	1	0	0	1	0	0	1	0	0	1	0	0	1	0	0	1	0	0	1	0	0
chr2	11124442	10	C	0	1	0	0	1	0	0	1	0	0	1	0	0	1	0	0	1	0	0	1	0	0	1	0	0	1	0	0
chr2	11124443	11	C	0	1	0	0	1	0	0	1	0	0	1	0	0	1	0	0	1	0	0	1	0	0	1	0	0	1	0	0
chr2	11124447	12	C	0	1	0	0	1	0	0	1	0	0	0,99	0,01	0	0,99	0,01	0	0,99	0,01	0	1	0	0	1	0	0	1	0	0
chr2	11124451	13	C	0	1	0	0	1	0	0	1	0	0	1	0	0	0,99	0	0	0,99	0	0	1	0	0	1	0	0	0,99	0	0
chr2	11124454	14	C	0	1	0	0	1	0	0	1	0	0	1	0	0	1	0	0	0,99	0	0,01	0,99	0,01	0	0,99	0,01	0	0,99	0,01	0
chr2	11124458	15	C	0	1	0	0	1	0	0	1	0	0	1	0	0	1	0	0	1	0	0	1	0	0	1	0	0	1	0	0
chr2	11124460	16	C	0	1	0	0	1	0	0	1	0	0	1	0	0	0,99	0,01	0	1	0	0	1	0	0	1	0	0	1	0	0
chr2	11124463	17	C	1	0,03	0,89	0,09	0,03	0,87	0,1	0,03	0,88	0,09	0,09	0,84	0,08	0,1	0,85	0,05	0,1	0,84	0,06	0,24	0,69	0,07	0,28	0,68	0,03	0,28	0,68	0,04
chr2	11124467	18	C	0	1	0	0	1	0	0	1	0	0	1	0	0	1	0	0	1	0	0	1	0	0	1	0	0	1	0	0
chr2	11124468	19	C	0	1	0	0	1	0	0	1	0	0	1	0	0	1	0	0	1	0	0	0,99	0	0	0,99	0	0,01	0,99	0	0,01
chr2	11124469	20	C	0	1	0	0	1	0	0	1	0	0	1	0	0	1	0	0	1	0	0	1	0	0	1	0	0	1	0	0
chr2	11124470	21	C	0	1	0	0	1	0	0	1	0	0	1	0	0	1	0	0	1	0	0	1	0	0	1	0	0	1	0	0
chr2	11124471	22	C	0	1	0	0	1	0	0	1	0	0	1	0	0	1	0	0	1	0	0	1	0	0	1	0	0	1	0	0
chr2	11124474	23	C	0	1	0	0	1	0	0	1	0	0	1	0	0	1	0	0	1	0	0	1	0	0	1	0	0	1	0	0
chr2	11124479	24	G	0	0,95	0,01	0,03	0,97	0,01	0,02	0,97	0	0,02	0,97	0,01	0,02	0,99	0	0,01	0,99	0	0,01	0,99	0,01	0,01	0,99	0	0	0,99	0	0
chr2	11124480	25	C	0	1	0	0	1	0	0	1	0	0	1	0	0	0,99	0	0	0,99	0	0	1	0	0	1	0	0	1	0	0
chr2	11124481	26	C	0	1	0	0	1	0	0	1	0	0	1	0	0	1	0	0	1	0	0	1	0	0	1	0	0	1	0	0
chr2	11124483	27	C	0	1	0	0	1	0	0	1	0	0	1	0	0	1	0	0	1	0	0	1	0	0	1	0	0	1	0	0
chr2	11124484	28	C	0	1	0	0	1	0	0	1	0	0	1	0	0	1	0	0	1	0	0	1	0	0	1	0	0	1	0	0
chr2	11124485	29	C	0	1	0	0	1	0	0	1	0	0	1	0	0	1	0	0	1	0	0	1	0	0	1	0	0	1	0	0
chr2	11124488	30	G	0	0,96	0,01	0,03	0,98	0	0,02	0,97	0,01	0,02	0,98	0,01	0,01	0,99	0	0,01	0,99	0	0,01	0,99	0	0,01	1	0	0	0,99	0	0,01
chr2	11124489	31	C	0	1	0	0	1	0	0	1	0	0	0,99	0	0	0,99	0	0	1	0	0	1	0	0	1	0	0	1	0	0
chr2	11124491	32	C	0	1	0	0	1	0	0	1	0	0	1	0	0	1	0	0	1	0	0	1	0	0	1	0	0	1	0	0
chr2	11124492	33	C	0	1	0	0	1	0	0	1	0	0	1	0	0	1	0	0	1	0	0	1	0	0	0,99	0	0	1	0	0
chr2	11124494	34	G	0	0,96	0,01	0,02	0,98	0	0,01	0,99	0	0,01	0,98	0,01	0,01	0,99	0	0,01	0,99	0	0,01	0,99	0	0	1	0	0	1	0	0
chr2	11124495	35	G	0	0,96	0,01	0,03	0,94	0,01	0,04	0,97	0,01	0,03	0,98	0,01	0,01	0,99	0	0,01	0,99	0	0,01	0,98	0,01	0,01	0,99	0	0	0,99	0	0,01
chr2	11124496	36	C	0	0,99	0	0	0,99	0,01	0	0,99	0	0	0,99	0,01	0	0,99	0	0	0,99	0	0	0,99	0,01	0	0,99	0,01	0	0,99	0,01	0
chr2	11124498	37	C	1	0,18	0,71	0,12	0,16	0,71	0,13	0,17	0,74	0,1	0,32	0,65	0,03	0,33	0,65	0,02	0,33	0,65	0,02	0,73	0,26	0	0,73	0,27	0	0,74	0,26	0
chr2	11124499	38	G	1	0,28	0,24	0,48	0,19	0,2	0,61	0,29	0,18	0,53	0,61	0,24	0,14	0,71	0,17	0,12	0,7	0,22	0,08	0,91	0,07	0,02	0,92	0,06	0,02	0,92	0,07	0,01

chr2	11124502	39	G	0	0,93	0,03	0,04	0,97	0,01	0,02	0,98	0	0,02	0,98	0,01	0,01	0,98	0,01	0,01	0,99	0	0,01	0,99	0,01	0,01	0,99	0	0	1	0	0
chr2	11124503	40	C	0	1	0	0	1	0	0	1	0	0	1	0	0	1	0	0	1	0	0	1	0	0	1	0	0	1	0	0
chr2	11124504	41	C	0	1	0	0	1	0	0	1	0	0	1	0	0	1	0	0	1	0	0	1	0	0	1	0	0	1	0	0
chr2	11124507	42	G	0	0,96	0,01	0,03	0,98	0	0,02	0,98	0,01	0,01	0,98	0,01	0,01	0,97	0,01	0,01	0,99	0	0,01	0,98	0,01	0,01	0,99	0	0	0,99	0	0,01
chr2	11124508	43	C	0	0,98	0,01	0,01	0,98	0,01	0,01	0,98	0,01	0,01	0,98	0	0,02	0,98	0	0,02	0,98	0	0,02	0,95	0,03	0,03	0,95	0,02	0,03	0,95	0,02	0,03
chr2	11124510	44	C	0	1	0	0	1	0	0	1	0	0	1	0	0	1	0	0	1	0	0	1	0	0	1	0	0	1	0	0
chr2	11124511	45	C	0	1	0	0	1	0	0	1	0	0	1	0	0	1	0	0	1	0	0	0,99	0	0,01	0,99	0	0,01	0,99	0	0,01
chr2	11124513	46	C	0	1	0	0	1	0	0	1	0	0	1	0	0	1	0	0	1	0	0	1	0	0	1	0	0	1	0	0
chr2	11124514	47	C	0	1	0	0	1	0	0	1	0	0	0,99	0	0,01	0,99	0	0,01	0,99	0	0,01	0,99	0	0,01	1	0	0	0,99	0	0,01
chr2	11124516	48	C	0	1	0	0	1	0	0	1	0	0	1	0	0	1	0	0	1	0	0	1	0	0	1	0	0	1	0	0
chr2	11124517	49	C	1	0,02	0,9	0,08	0,02	0,9	0,09	0,02	0,91	0,06	0,06	0,82	0,12	0,07	0,83	0,11	0,07	0,83	0,1	0,27	0,67	0,06	0,3	0,66	0,04	0,3	0,65	0,05
chr2	11124518	50	G	1	0,05	0,68	0,28	0,03	0,55	0,41	0,04	0,58	0,38	0,22	0,48	0,3	0,29	0,43	0,28	0,21	0,41	0,38	0,43	0,47	0,09	0,48	0,49	0,03	0,47	0,49	0,04
chr2	11124519	51	C	0	1	0	0	1	0	0	1	0	0	1	0	0	1	0	0	1	0	0	1	0	0	1	0	0	1	0	0
chr2	11124520	52	C	0	1	0	0	1	0	0	1	0	0	1	0	0	0,99	0	0,01	1	0	0	1	0	0	1	0	0	1	0	0
chr2	11124522	53	C	0	1	0	0	1	0	0	1	0	0	1	0	0	1	0	0	1	0	0	1	0	0	1	0	0	1	0	0
chr2	11124523	54	C	1	0,03	0,93	0,04	0,03	0,93	0,04	0,03	0,95	0,03	0,02	0,91	0,07	0,02	0,91	0,06	0,03	0,91	0,07	0,24	0,76	0,01	0,26	0,74	0	0,26	0,74	0
chr2	11124524	55	G	1	0,03	0,7	0,27	0,03	0,58	0,39	0,05	0,6	0,35	0,16	0,39	0,45	0,32	0,36	0,32	0,21	0,36	0,43	0,36	0,42	0,22	0,47	0,48	0,05	0,45	0,43	0,12
chr2	11124525	56	C	0	1	0	0	1	0	0	1	0	0	1	0	0	1	0	0	1	0	0	1	0	0	1	0	0	1	0	0
chr2	11124526	57	C	0	1	0	0	1	0	0	1	0	0	0,99	0	0	0,99	0	0,01	0,99	0	0,01	0,99	0	0,01	1	0	0	1	0	0
chr2	11124528	58	C	0	1	0	0	1	0	0	1	0	0	1	0	0	1	0	0	1	0	0	1	0	0	1	0	0	1	0	0
chr2	11124529	59	C	1	0,02	0,92	0,06	0,01	0,91	0,07	0,01	0,93	0,06	0,05	0,86	0,09	0,06	0,87	0,07	0,06	0,87	0,07	0,3	0,7	0	0,31	0,69	0	0,31	0,69	0
chr2	11124530	60	G	1	0,79	0,06	0,15	0,63	0,05	0,33	0,73	0,05	0,22	0,92	0,04	0,03	0,92	0,05	0,03	0,91	0,03	0,06	0,97	0,02	0,01	0,98	0,01	0,01	0,98	0,01	0,01
chr2	11124531	61	C	0	0,99	0	0	1	0	0	0,99	0	0	0,99	0	0	0,99	0	0,01	0,99	0	0,01	1	0	0	1	0	0	1	0	0
chr2	11124532	62	C	0	1	0	0	1	0	0	1	0	0	1	0	0	1	0	0	1	0	0	1	0	0	1	0	0	1	0	0
chr2	11124534	63	C	1	0,03	0,86	0,11	0,02	0,87	0,11	0,02	0,88	0,1	0,13	0,81	0,06	0,14	0,83	0,04	0,14	0,83	0,03	0,54	0,46	0	0,56	0,44	0	0,56	0,44	0
chr2	11124535	64	G	1	0,05	0,44	0,51	0,03	0,34	0,63	0,02	0,34	0,63	0,38	0,23	0,39	0,54	0,26	0,2	0,5	0,24	0,26	0,81	0,16	0,03	0,81	0,18	0,01	0,81	0,17	0,02
chr2	11124536	65	G	0	0,96	0,01	0,02	0,98	0	0,01	0,99	0	0,01	0,98	0,01	0,01	0,99	0,01	0,01	0,99	0	0,01	0,99	0	0,01	0,99	0	0,01	1	0	0
chr2	11124537	66	C	0	1	0	0	1	0	0	1	0	0	1	0	0	1	0	0	1	0	0	1	0	0	1	0	0	1	0	0
chr2	11124538	67	C	0	1	0	0	1	0	0	1	0	0	1	0	0	1	0	0	1	0	0	1	0	0	1	0	0	1	0	0
chr2	11124540	68	C	0	1	0	0	1	0	0	1	0	0	0,99	0,01	0	0,99	0,01	0	0,99	0,01	0	1	0	0	1	0	0	1	0	0
chr2	11124542	69	G	0	0,94	0,01	0,04	0,97	0,01	0,02	0,98	0	0,01	0,97	0,01	0,01	0,99	0,01	0,01	0,99	0	0,01	0,98	0,01	0,01	1	0	0	0,99	0	0
chr2	11124543	70	C	0	1	0	0	1	0	0	1	0	0	1	0	0	1	0	0	1	0	0	1	0	0	1	0	0	1	0	0
chr2	11124544	71	C	0	1	0	0	1	0	0	1	0	0	1	0	0	1	0	0	1	0	0	1	0	0	1	0	0	1	0	0
chr2	11124546	72	C	1	0,06	0,79	0,14	0,06	0,8	0,15	0,06	0,81	0,13	0,21	0,79	0	0,21	0,79	0	0,22	0,78	0	0,64	0,36	0	0,63	0,37	0	0,64	0,36	0
chr2	11124547	73	G	1	0,14	0,51	0,35	0,11	0,41	0,47	0,17	0,38	0,45	0,52	0,18	0,3	0,6	0,17	0,24	0,61	0,18	0,21	0,86	0,13	0,01	0,88	0,11	0	0,86	0,13	0,01
chr2	11124548	74	G	0	0,96	0,01	0,03	0,98	0,01	0,02	0,99	0	0,01	0,97	0,02	0,02	0,98	0,01	0,01	0,98	0,01	0,01	0,99	0	0	0,99	0	0,01	0,99	0	0
chr2	11124549	75	C	0	1	0	0	1	0	0	1	0	0	1	0	0	1	0	0	1	0	0	1	0	0	1	0	0	1	0	0
chr2	11124550	76	C	0	1	0	0	0,99	0,01	0	0,99	0,01	0	1	0	0	1	0	0	1	0	0	0,99	0	0	0,99	0	0	1	0	0
chr2	11124552	77	C	0	1	0	0	1	0	0	1	0	0	1	0	0	1	0	0	1	0	0	1	0	0	1	0	0	1	0	0
chr2	11124553	78	C	1	0,03	0,84	0,13	0,03	0,85	0,13	0,03	0,85	0,12	0,14	0,83	0,04	0,16	0,83	0,01	0,16	0,84	0,01	0,51	0,49	0	0,52	0,48	0	0,52	0,48	0
chr2	11124554	79	G	1	0,05	0,47	0,48	0,09	0,35	0,57	0,03	0,41	0,56	0,52	0,17	0,31	0,59	0,19	0,22	0,54	0,21	0,25	0,88	0,1	0,02	0,86	0,14	0,01	0,83	0,16	0,01
chr2	11124555	80	C	0	1	0	0	1	0	0	1	0	0	1	0	0	1	0	0	1	0	0	1	0	0	1	0	0	1	0	0
chr2	11124556	81	C	0	1	0	0	0,99	0,01	0	0,99	0,01	0	1	0	0	1	0	0	1	0	0	1	0	0	1	0	0	1	0	0
chr2	11124558	82	C	0	1	0	0	1	0	0	1	0	0	1	0	0	1	0	0	1	0	0	1	0	0	1	0	0	1	0	0
chr2	11124559	83	C	1	0,17	0,63	0,2	0,17	0,65	0,18	0,17	0,65	0,18	0,31	0,69	0,01	0,31	0,69	0	0,31	0,69	0	0,68	0,32	0	0,69	0,31	0	0,7	0,3	0
chr2	11124560	84	G	1	0,21	0,34	0,45	0,17	0,28	0,55	0,18	0,29	0,53	0,54	0,14	0,33	0,63	0,14	0,23	0,63	0,2	0,17	0,9	0,09	0,02	0,88	0,11	0,01	0,89	0,1	0,01

chr2	11124561	85	G	0	0,95	0,02	0,03	0,98	0	0,01	0,99	0	0,01	0,98	0,01	0,01	0,99	0	0,01	0,99	0	0,01	0,99	0	0,01	1	0	0	1	0	0
chr2	11124562	86	G	0	0,96	0,01	0,03	0,96	0,01	0,03	0,99	0	0,01	0,98	0,01	0,01	0,99	0	0,01	0,99	0	0,01	0,99	0	0	0,99	0	0	0,99	0	0,01
chr2	11124563	87	C	0	1	0	0	1	0	0	1	0	0	1	0	0	1	0	0	1	0	0	1	0	0	1	0	0	1	0	0
chr2	11124567	88	G	0	0,96	0,01	0,03	0,98	0,01	0,02	0,98	0	0,01	0,98	0,01	0,01	0,99	0,01	0,01	0,99	0	0	0,99	0	0	1	0	0	1	0	0
chr2	11124572	89	G	0	0,96	0,01	0,02	0,97	0	0,02	0,99	0	0,01	0,98	0,01	0,01	0,99	0	0,01	0,99	0	0	0,99	0	0,01	0,99	0	0	1	0	0
chr2	11124576	90	C	0	1	0	0	1	0	0	1	0	0	1	0	0	1	0	0	1	0	0	1	0	0	1	0	0	1	0	0
chr2	11124581	91	C	0	1	0	0	1	0	0	1	0	0	1	0	0	1	0	0	1	0	0	1	0	0	1	0	0	1	0	0
chr2	11124584	92	G	0	0,96	0,01	0,03	0,98	0	0,01	0,99	0	0,01	0,98	0,01	0,01	0,99	0	0,01	0,99	0	0,01	0,99	0	0	1	0	0	1	0	0
chr2	11124586	93	C	0	1	0	0	1	0	0	1	0	0	1	0	0	1	0	0	1	0	0	1	0	0	1	0	0	1	0	0
chr2	11124588	94	G	0	0,96	0,01	0,03	0,98	0	0,01	0,99	0	0,01	0,98	0,01	0,01	0,99	0	0,01	0,99	0	0	0,99	0	0	1	0	0	1	0	0
chr2	11124593	95	G	0	0,96	0,01	0,02	0,98	0	0,01	0,99	0	0,01	0,98	0,01	0,01	0,99	0	0,01	0,99	0	0	0,99	0	0	1	0	0	1	0	0
chr2	11124597	96	G	0	0,96	0,01	0,02	0,98	0	0,01	0,99	0	0,01	0,98	0,01	0,01	0,99	0	0	0,99	0	0	0,99	0	0	1	0	0	1	0	0
chr2	11124598	97	G	0	0,96	0,01	0,03	0,98	0	0,01	0,99	0	0,01	0,98	0,01	0,01	0,99	0	0,01	0,99	0	0,01	0,99	0	0	1	0	0	1	0	0
chr2	11124600	98	G	0	0,96	0,01	0,03	0,98	0	0,01	0,99	0	0,01	0,98	0,01	0,01	0,99	0	0,01	0,99	0	0	0,99	0	0	0,99	0	0	0,99	0	0
chr2	11124609	99	G	0	0,96	0,01	0,02	0,98	0	0,01	0,99	0	0,01	0,98	0,01	0,01	0,99	0	0,01	0,99	0	0	0,99	0	0	1	0	0	0,99	0	0
chr3	89184744	100	C	0	1	0	0	1	0	0	1	0	0	1	0	0	1	0	0	1	0	0	1	0	0	1	0	0	1	0	0
chr3	89184752	101	G	0	1	0	0	1	0	0	1	0	0	1	0	0	1	0	0	1	0	0	1	0	0	1	0	0	1	0	0
chr3	89184753	102	C	0	1	0	0	1	0	0	1	0	0	1	0	0	1	0	0	1	0	0	1	0	0	1	0	0	1	0	0
chr3	89184754	103	C	0	1	0	0	1	0	0	1	0	0	1	0	0	1	0	0	1	0	0	1	0	0	1	0	0	1	0	0
chr3	89184756	104	C	0	1	0	0	1	0	0	1	0	0	1	0	0	1	0	0	1	0	0	1	0	0	1	0	0	1	0	0
chr3	89184758	105	G	0	1	0	0	1	0	0	1	0	0	1	0	0	1	0	0	1	0	0	1	0	0	1	0	0	1	0	0
chr3	89184759	106	G	0	1	0	0	1	0	0	1	0	0	1	0	0	1	0	0	1	0	0	1	0	0	1	0	0	1	0	0
chr3	89184760	107	G	0	1	0	0	1	0	0	1	0	0	1	0	0	1	0	0	1	0	0	1	0	0	1	0	0	1	0	0
chr3	89184762	108	C	0	1	0	0	1	0	0	1	0	0	1	0	0	1	0	0	1	0	0	1	0	0	1	0	0	1	0	0
chr3	89184763	109	C	0	1	0	0	1	0	0	1	0	0	1	0	0	1	0	0	1	0	0	1	0	0	1	0	0	1	0	0
chr3	89184765	110	G	0	1	0	0	1	0	0	1	0	0	1	0	0	1	0	0	1	0	0	1	0	0	1	0	0	1	0	0
chr3	89184768	111	G	0	1	0	0	1	0	0	1	0	0	1	0	0	1	0	0	1	0	0	1	0	0	1	0	0	1	0	0
chr3	89184769	112	G	0	1	0	0	1	0	0	1	0	0	1	0	0	1	0	0	1	0	0	1	0	0	1	0	0	1	0	0
chr3	89184773	113	G	0	1	0	0	1	0	0	1	0	0	1	0	0	1	0	0	1	0	0	1	0	0	1	0	0	1	0	0
chr3	89184774	114	C	0	1	0	0	1	0	0	1	0	0	1	0	0	1	0	0	1	0	0	1	0	0	1	0	0	1	0	0
chr3	89184776	115	C	1	0,2	0,79	0,01	0,2	0,76	0,04	0,19	0,76	0,05	0,21	0,67	0,12	0,19	0,68	0,12	0,2	0,7	0,1	0,38	0,53	0,09	0,38	0,54	0,08	0,36	0,54	0,11
chr3	89184777	116	G	1	0,21	0,7	0,09	0,23	0,68	0,1	0,22	0,74	0,04	0,24	0,72	0,04	0,26	0,72	0,01	0,25	0,73	0,02	0,4	0,59	0	0,43	0,56	0	0,43	0,57	0
chr3	89184778	117	G	0	1	0	0	1	0	0	1	0	0	1	0	0	1	0	0	1	0	0	1	0	0	1	0	0	1	0	0
chr3	89184779	118	G	0	1	0	0	1	0	0	1	0	0	0,99	0	0,01	0,99	0	0	0,99	0	0,01	1	0	0	1	0	0	1	0	0
chr3	89184780	119	C	0	1	0	0	1	0	0	1	0	0	1	0	0	1	0	0	1	0	0	1	0	0	1	0	0	1	0	0
chr3	89184781	120	C	0	1	0	0	1	0	0	1	0	0	1	0	0	1	0	0	1	0	0	1	0	0	0,99	0	0	1	0	0
chr3	89184782	121	C	0	1	0	0	1	0	0	1	0	0	1	0	0	1	0	0	1	0	0	1	0	0	1	0	0	1	0	0
chr3	89184783	122	C	0	1	0	0	1	0	0	1	0	0	0,99	0	0,01	0,99	0	0,01	0,99	0	0	0,99	0	0	0,99	0,01	0	1	0	0
chr3	89184787	123	G	0	1	0	0	1	0	0	1	0	0	1	0	0	1	0	0	1	0	0	1	0	0	1	0	0	1	0	0
chr3	89184788	124	G	0	1	0	0	1	0	0	1	0	0	1	0	0	1	0	0	1	0	0	1	0	0	1	0	0	1	0	0
chr3	89184792	125	G	0	0,99	0	0	1	0	0	0,99	0	0	1	0	0	1	0	0	1	0	0	0,99	0	0	0,99	0	0,01	0,99	0	0,01
chr3	89184795	126	G	0	1	0	0	1	0	0	1	0	0	0,99	0	0,01	0,99	0	0,01	0,99	0	0,01	1	0	0	1	0	0	1	0	0
chr3	89184800	127	G	0	1	0	0	1	0	0	1	0	0	1	0	0	1	0	0	1	0	0	1	0	0	1	0	0	1	0	0
chr3	89184801	128	C	0	1	0	0	1	0	0	1	0	0	1	0	0	1	0	0	1	0	0	1	0	0	1	0	0	1	0	0
chr3	89184805	129	C	0	1	0	0	1	0	0	1	0	0	1	0	0	1	0	0	1	0	0	1	0	0	1	0	0	1	0	0
chr3	89184806	130	C	1	0,12	0,82	0,06	0,11	0,83	0,06	0,11	0,84	0,05	0,13	0,78	0,09	0,12	0,81	0,07	0,13	0,81	0,06	0,2	0,78	0,02	0,2	0,78	0,02	0,18	0,78	0,03

chr3	89184807	131	G	1	0,15	0,83	0,02	0,17	0,81	0,01	0,16	0,83	0,01	0,1	0,81	0,09	0,1	0,82	0,08	0,11	0,83	0,06	0,22	0,78	0	0,24	0,76	0	0,23	0,76	0
chr3	89184808	132	G	0	1	0	0	1	0	0	1	0	0	1	0	0	0,99	0	0	1	0	0	1	0	0	1	0	0	1	0	0
chr3	89184809	133	G	0	0,99	0	0	1	0	0	1	0	0	0,99	0	0	0,99	0	0	1	0	0	1	0	0	1	0	0	1	0	0
chr3	89184810	134	G	0	1	0	0	1	0	0	1	0	0	0,99	0	0	0,99	0	0,01	0,99	0	0,01	1	0	0	0,99	0	0	1	0	0
chr3	89184814	135	G	0	1	0	0	1	0	0	1	0	0	1	0	0	1	0	0	1	0	0	1	0	0	1	0	0	1	0	0
chr3	89184815	136	C	1	0,07	0,9	0,03	0,06	0,9	0,04	0,06	0,9	0,04	0,11	0,86	0,03	0,1	0,88	0,02	0,1	0,88	0,02	0,15	0,81	0,04	0,14	0,8	0,06	0,13	0,8	0,07
chr3	89184816	137	G	1	0,03	0,94	0,04	0,03	0,92	0,04	0,03	0,95	0,02	0,05	0,9	0,05	0,06	0,92	0,02	0,06	0,92	0,02	0,13	0,84	0,03	0,16	0,82	0,01	0,15	0,84	0,01
chr3	89184817	138	G	0	1	0	0	0,99	0	0	1	0	0	1	0	0	1	0	0	1	0	0	1	0	0	0,99	0	0	1	0	0
chr3	89184818	139	C	0	1	0	0	1	0	0	0,99	0	0,01	1	0	0	1	0	0	1	0	0	1	0	0	1	0	0	1	0	0
chr3	89184820	140	G	0	1	0	0	1	0	0	1	0	0	0,99	0	0,01	0,99	0	0	0,99	0	0	1	0	0	1	0	0	1	0	0
chr3	89184821	141	C	1	0,11	0,78	0,11	0,1	0,76	0,13	0,1	0,78	0,12	0,2	0,79	0,01	0,18	0,82	0,01	0,17	0,82	0,01	0,25	0,63	0,12	0,24	0,62	0,13	0,22	0,63	0,14
chr3	89184822	142	G	1	0,15	0,84	0,01	0,15	0,84	0	0,17	0,82	0,01	0,12	0,82	0,06	0,12	0,83	0,05	0,12	0,84	0,04	0,2	0,79	0,01	0,23	0,75	0,02	0,23	0,75	0,02
chr3	89184824	143	C	1	0,11	0,7	0,19	0,11	0,69	0,2	0,1	0,71	0,19	0,17	0,69	0,13	0,16	0,73	0,11	0,16	0,73	0,11	0,32	0,66	0,02	0,33	0,66	0,01	0,31	0,66	0,02
chr3	89184825	144	G	1	0,08	0,84	0,08	0,09	0,81	0,1	0,1	0,86	0,04	0,15	0,77	0,08	0,16	0,79	0,05	0,17	0,81	0,03	0,21	0,79	0	0,24	0,76	0	0,23	0,77	0
chr3	89184826	145	G	0	1	0	0	0,99	0	0	1	0	0	1	0	0	0,99	0	0	1	0	0	1	0	0	1	0	0	1	0	0
chr3	89184827	146	G	0	1	0	0	1	0	0	1	0	0	1	0	0	1	0	0	0,99	0	0	1	0	0	1	0	0	1	0	0
chr3	89184828	147	C	0	1	0	0	1	0	0	1	0	0	1	0	0	1	0	0	1	0	0	1	0	0	1	0	0	1	0	0
chr3	89184831	148	C	0	0,99	0	0,01	0,99	0	0,01	0,99	0	0,01	1	0	0	1	0	0	1	0	0	1	0	0	1	0	0	1	0	0
chr3	89184833	149	G	0	1	0	0	1	0	0	1	0	0	1	0	0	1	0	0	1	0	0	1	0	0	1	0	0	1	0	0
chr3	89184834	150	G	0	0,99	0	0,01	0,99	0	0,01	0,99	0	0,01	0,99	0	0,01	0,99	0	0,01	0,99	0	0,01	1	0	0	1	0	0	1	0	0
chr3	89184836	151	C	1	0,14	0,81	0,04	0,13	0,82	0,05	0,13	0,84	0,03	0,19	0,78	0,04	0,17	0,81	0,03	0,16	0,81	0,03	0,21	0,7	0,09	0,23	0,7	0,07	0,21	0,71	0,09
chr3	89184837	152	G	1	0,1	0,79	0,11	0,12	0,79	0,09	0,13	0,81	0,06	0,14	0,76	0,1	0,13	0,77	0,09	0,13	0,79	0,08	0,25	0,74	0,01	0,27	0,73	0,01	0,27	0,73	0
chr3	89184839	153	G	0	1	0	0	1	0	0	1	0	0	1	0	0	1	0	0	1	0	0	1	0	0	1	0	0	1	0	0
chr3	89184840	154	G	0	1	0	0	1	0	0	1	0	0	1	0	0	1	0	0	1	0	0	1	0	0	1	0	0	1	0	0
chr3	89184842	155	G	0	0,99	0,01	0	0,99	0,01	0	0,99	0,01	0	1	0	0	1	0	0	1	0	0	1	0	0	1	0	0	1	0	0
chr3	89184843	156	G	0	1	0	0	1	0	0	1	0	0	1	0	0	1	0	0	1	0	0	0,99	0	0	0,99	0,01	0	0,99	0,01	0
chr3	89184844	157	C	1	0,06	0,92	0,01	0,06	0,92	0,02	0,06	0,93	0,02	0,08	0,85	0,07	0,07	0,89	0,04	0,07	0,89	0,04	0,13	0,82	0,06	0,12	0,82	0,06	0,11	0,82	0,07
chr3	89184845	158	G	1	0,05	0,94	0,01	0,06	0,93	0,01	0,06	0,93	0,01	0,06	0,9	0,04	0,07	0,91	0,02	0,07	0,91	0,01	0,12	0,72	0,16	0,15	0,71	0,14	0,15	0,72	0,13
chr3	89184846	159	G	0	1	0	0	1	0	0	1	0	0	1	0	0	1	0	0	1	0	0	1	0	0	1	0	0	1	0	0
chr3	89184848	160	G	0	1	0	0	0,99	0,01	0	0,99	0,01	0	1	0	0	0,99	0,01	0	0,99	0	0,01	0,99	0,01	0	0,98	0,02	0	0,99	0,01	0
chr3	89184849	161	G	0	1	0	0	1	0	0	1	0	0	1	0	0	0,99	0	0	0,99	0	0	1	0	0	1	0	0	1	0	0
chr3	89184850	162	C	1	0,07	0,91	0,03	0,06	0,9	0,04	0,06	0,92	0,03	0,12	0,8	0,08	0,1	0,85	0,05	0,11	0,87	0,03	0,14	0,77	0,09	0,14	0,77	0,08	0,14	0,79	0,08
chr3	89184851	163	G	1	0,07	0,83	0,1	0,08	0,82	0,1	0,09	0,85	0,06	0,07	0,88	0,06	0,08	0,9	0,03	0,06	0,9	0,04	0,14	0,85	0,01	0,17	0,82	0,01	0,17	0,82	0,01
chr3	89184852	164	G	0	1	0	0	1	0	0	1	0	0	1	0	0	1	0	0	1	0	0	0,99	0	0,01	0,99	0	0,01	0,98	0	0,01
chr3	89184853	165	C	1	0,06	0,91	0,04	0,06	0,92	0,03	0,04	0,92	0,04	0,09	0,87	0,04	0,08	0,91	0,01	0,08	0,91	0,01	0,13	0,78	0,09	0,14	0,76	0,1	0,12	0,79	0,08
chr3	89184854	166	G	1	0,05	0,84	0,12	0,04	0,8	0,16	0,06	0,82	0,12	0,04	0,87	0,09	0,05	0,88	0,08	0,04	0,88	0,09	0,14	0,86	0	0,15	0,85	0	0,16	0,84	0
chr3	89184855	167	G	0	1	0	0	1	0	0	1	0	0	0,99	0	0,01	0,99	0	0,01	0,99	0	0,01	1	0	0	1	0	0	1	0	0
chr3	89184856	168	C	1	0,07	0,89	0,04	0,06	0,89	0,05	0,06	0,9	0,04	0,1	0,86	0,04	0,08	0,91	0,02	0,09	0,9	0,01	0,12	0,81	0,07	0,12	0,81	0,07	0,12	0,82	0,07
chr3	89184857	169	G	1	0,02	0,88	0,1	0,02	0,85	0,13	0,02	0,89	0,09	0,06	0,85	0,09	0,06	0,88	0,07	0,05	0,87	0,08	0,13	0,86	0,01	0,15	0,84	0,01	0,16	0,84	0,01
chr3	89184858	170	G	0	1	0	0	1	0	0	1	0	0	0,99	0	0,01	0,99	0	0,01	0,99	0	0,01	1	0	0	1	0	0	1	0	0
chr3	89184861	171	G	0	1	0	0	1	0	0	1	0	0	0,99	0,01	0	0,99	0,01	0	0,99	0,01	0	1	0	0	1	0	0	1	0	0
chr3	89184862	172	G	0	1	0	0	1	0	0	1	0	0	1	0	0	1	0	0	1	0	0	1	0	0	0,99	0	0	1	0	0
chr3	89184864	173	G	0	1	0	0	1	0	0	1	0	0	1	0	0	1	0	0	1	0	0	1	0	0	1	0	0	1	0	0
chr3	89184865	174	G	0	1	0	0	1	0	0	1	0	0	1	0	0	1	0	0	1	0	0	1	0	0	1	0	0	1	0	0
chr3	89184866	175	G	0	1	0	0	1	0	0	1	0	0	1	0	0	1	0	0	1	0	0	1	0	0	1	0	0	1	0	0
chr3	89184867	176	G	0	0,99	0	0	1	0	0	0,99	0	0	1	0	0	1	0	0	1	0	0	1	0	0	1	0	0	1	0	0

chr3	89184868	177	C	0	0,98	0,01	0,01	0,98	0,01	0,01	0,99	0	0,01	0,99	0	0	0,99	0,01	0	0,99	0	0	0,99	0,01	0	0,99	0,01	0	1	0	0
chr3	89184870	178	C	0	1	0	0	1	0	0	1	0	0	1	0	0	1	0	0	1	0	0	1	0	0	1	0	0	1	0	0
chr3	89184871	179	C	0	1	0	0	1	0	0	1	0	0	1	0	0	1	0	0	1	0	0	0,99	0,01	0	0,99	0,01	0	0,99	0,01	0
chr3	89184873	180	G	0	1	0	0	1	0	0	1	0	0	1	0	0	1	0	0	1	0	0	1	0	0	1	0	0	1	0	0
chr3	89184875	181	G	0	1	0	0	1	0	0	1	0	0	0,99	0,01	0	0,99	0,01	0	0,99	0,01	0	1	0	0	1	0	0	1	0	0
chr3	89184876	182	G	0	1	0	0	1	0	0	1	0	0	1	0	0	1	0	0	1	0	0	1	0	0	0,99	0	0,01	1	0	0
chr3	89184878	183	G	0	1	0	0	1	0	0	1	0	0	1	0	0	1	0	0	1	0	0	1	0	0	1	0	0	1	0	0
chr3	89184879	184	G	0	0,99	0,01	0	0,99	0,01	0	1	0	0	1	0	0	1	0	0	1	0	0	1	0	0	1	0	0	1	0	0
chr3	89184880	185	G	0	1	0	0	1	0	0	1	0	0	1	0	0	1	0	0	1	0	0	1	0	0	1	0	0	1	0	0
chr3	89184881	186	C	0	0,99	0	0	1	0	0	1	0	0	1	0	0	1	0	0	1	0	0	0,99	0	0	1	0	0	1	0	0
chr3	89184883	187	G	0	1	0	0	1	0	0	1	0	0	1	0	0	1	0	0	1	0	0	1	0	0	1	0	0	1	0	0
chr3	89184885	188	C	0	1	0	0	1	0	0	1	0	0	1	0	0	1	0	0	1	0	0	1	0	0	1	0	0	1	0	0
chr3	89184888	189	C	0	1	0	0	1	0	0	1	0	0	0,99	0,01	0	0,99	0,01	0	0,99	0,01	0	1	0	0	1	0	0	1	0	0
chr3	89184890	190	C	0	1	0	0	1	0	0	1	0	0	0,99	0	0,01	0,99	0	0,01	0,99	0	0,01	1	0	0	1	0	0	1	0	0
chr3	89184891	191	C	1	0,34	0,56	0,1	0,31	0,57	0,13	0,31	0,57	0,12	0,41	0,58	0	0,4	0,6	0	0,38	0,61	0	0,56	0,43	0,01	0,55	0,43	0,01	0,56	0,43	0,01
chr3	89184892	192	G	1	0,36	0,61	0,04	0,34	0,58	0,09	0,35	0,62	0,03	0,43	0,53	0,04	0,41	0,53	0,06	0,4	0,53	0,07	0,47	0,53	0,01	0,5	0,5	0	0,49	0,51	0
chr3	89184895	193	G	0	1	0	0	1	0	0	1	0	0	1	0	0	1	0	0	1	0	0	1	0	0	1	0	0	1	0	0
chr3	89184896	194	G	0	1	0	0	1	0	0	1	0	0	1	0	0	1	0	0	1	0	0	1	0	0	1	0	0	1	0	0
chr3	89184897	195	G	0	1	0	0	1	0	0	1	0	0	1	0	0	0,99	0	0	1	0	0	1	0	0	1	0	0	1	0	0
chr3	89184898	196	C	0	1	0	0	1	0	0	1	0	0	1	0	0	1	0	0	1	0	0	1	0	0	0,99	0	0	1	0	0
chr3	89184902	197	C	0	1	0	0	1	0	0	1	0	0	1	0	0	1	0	0	1	0	0	1	0	0	1	0	0	1	0	0
chr3	89184903	198	C	0	1	0	0	0,99	0	0	1	0	0	1	0	0	1	0	0	1	0	0	1	0	0	1	0	0	1	0	0
chr3	89184905	199	G	0	1	0	0	1	0	0	1	0	0	1	0	0	1	0	0	1	0	0	1	0	0	1	0	0	1	0	0
chr3	89184907	200	G	0	1	0	0	1	0	0	1	0	0	1	0	0	0,99	0	0	1	0	0	1	0	0	1	0	0	1	0	0
chr3	89184908	201	G	0	1	0	0	1	0	0	1	0	0	1	0	0	1	0	0	1	0	0	0,99	0	0	0,99	0	0	0,99	0	0,01
chr3	89184911	202	G	0	1	0	0	1	0	0	1	0	0	1	0	0	1	0	0	1	0	0	1	0	0	1	0	0	1	0	0
chr3	89184913	203	G	0	1	0	0	0,99	0,01	0	0,99	0,01	0	1	0	0	1	0	0	1	0	0	1	0	0	1	0	0	1	0	0
chr3	89184914	204	G	0	1	0	0	1	0	0	0,99	0	0	1	0	0	1	0	0	1	0	0	1	0	0	0,99	0	0	1	0	0
chr3	89184915	205	C	0	1	0	0	1	0	0	1	0	0	1	0	0	1	0	0	1	0	0	1	0	0	0,99	0	0	1	0	0
chr3	89184917	206	G	0	1	0	0	1	0	0	1	0	0	0,99	0	0,01	0,98	0	0,01	0,98	0,01	0,01	0,99	0	0,01	0,99	0	0,01	0,99	0	0,01
chr3	89184919	207	G	0	1	0	0	1	0	0	1	0	0	1	0	0	1	0	0	1	0	0	1	0	0	1	0	0	1	0	0
chr3	89184920	208	G	0	0,99	0	0,01	0,98	0	0,01	0,99	0	0,01	1	0	0	1	0	0	1	0	0	1	0	0	1	0	0	1	0	0
chr3	89184922	209	G	0	1	0	0	1	0	0	1	0	0	1	0	0	1	0	0	1	0	0	0,99	0,01	0	0,98	0,02	0	0,98	0,02	0
chr3	89184923	210	G	0	0,99	0	0,01	0,99	0	0,01	1	0	0	1	0	0	1	0	0	1	0	0	1	0	0	1	0	0	1	0	0
chr3	89184925	211	G	0	1	0	0	1	0	0	1	0	0	0,96	0,04	0	0,97	0,03	0	0,96	0,04	0	0,98	0	0,02	0,98	0	0,01	0,99	0	0,01
chr3	89184926	212	G	0	1	0	0	1	0	0	1	0	0	1	0	0	1	0	0	1	0	0	1	0	0	1	0	0	1	0	0
chr3	89184927	213	C	0	1	0	0	1	0	0	1	0	0	1	0	0	1	0	0	1	0	0	0,99	0,01	0	0,99	0,01	0	0,99	0,01	0
chr3	89184929	214	G	0	1	0	0	1	0	0	1	0	0	0,99	0,01	0	0,99	0,01	0	0,99	0,01	0	1	0	0	1	0	0	1	0	0
chr3	89184931	215	G	0	1	0	0	1	0	0	1	0	0	1	0	0	1	0	0	1	0	0	1	0	0	1	0	0	1	0	0
chr3	89184932	216	G	0	1	0	0	1	0	0	1	0	0	1	0	0	1	0	0	1	0	0	1	0	0	0,99	0,01	0	1	0	0
chr3	89184933	217	C	0	1	0	0	1	0	0	1	0	0	0,99	0	0	1	0	0	0,99	0	0,01	1	0	0	1	0	0	1	0	0
chr3	89184938	218	G	0	1	0	0	1	0	0	1	0	0	1	0	0	1	0	0	0,99	0	0	1	0	0	0,99	0,01	0	0,99	0	0
chr3	89184939	219	C	0	1	0	0	1	0	0	1	0	0	1	0	0	1	0	0	1	0	0	0,99	0	0,01	0,99	0	0,01	0,99	0	0,01
chr3	89184940	220	C	0	1	0	0	1	0	0	1	0	0	0,99	0	0,01	0,99	0	0,01	0,99	0	0,01	1	0	0	1	0	0	1	0	0
chr3	89184942	221	C	0	1	0	0	1	0	0	1	0	0	1	0	0	1	0	0	1	0	0	1	0	0	1	0	0	1	0	0
chr3	89184943	222	C	0	1	0	0	1	0	0	1	0	0	1	0	0	1	0	0	1	0	0	1	0	0	1	0	0	1	0	0

chr3	89184945	223	G	0	1	0	0	1	0	0	1	0	0	1	0	0	1	0	0	1	0	0	1	0	0	1	0	0	1	0	0
chr3	89184946	224	G	0	1	0	0	1	0	0	1	0	0	1	0	0	1	0	0	1	0	0	1	0	0	1	0	0	1	0	0
chr3	89184949	225	C	0	1	0	0	1	0	0	1	0	0	1	0	0	1	0	0	1	0	0	0,99	0	0,01	0,99	0	0,01	0,99	0	0,01
chr3	89184951	226	G	0	1	0	0	0,99	0	0	0,99	0	0,01	0,99	0	0,01	0,99	0	0,01	0,99	0	0,01	1	0	0	1	0	0	1	0	0
chr3	89184953	227	C	1	0,08	0,81	0,1	0,07	0,81	0,11	0,07	0,82	0,11	0,16	0,81	0,03	0,15	0,84	0,01	0,14	0,85	0	0,22	0,61	0,16	0,22	0,62	0,16	0,19	0,61	0,2
chr3	89184954	228	G	1	0,09	0,83	0,09	0,11	0,8	0,09	0,1	0,85	0,05	0,1	0,77	0,13	0,13	0,78	0,09	0,11	0,77	0,12	0,36	0,63	0	0,38	0,62	0	0,37	0,62	0
chr3	89184955	229	G	0	1	0	0	1	0	0	1	0	0	0,99	0,01	0	0,99	0,01	0	0,99	0,01	0	0,99	0	0	0,99	0	0,01	0,99	0	0,01
chr3	89184956	230	G	0	1	0	0	1	0	0	1	0	0	1	0	0	1	0	0	1	0	0	1	0	0	0,99	0	0	0,99	0	0,01
chr3	89184957	231	C	0	1	0	0	1	0	0	1	0	0	1	0	0	1	0	0	1	0	0	1	0	0	1	0	0	1	0	0
chr3	89184959	232	C	1	0,07	0,91	0,02	0,06	0,92	0,02	0,06	0,92	0,03	0,1	0,78	0,12	0,1	0,8	0,1	0,09	0,81	0,1	0,14	0,8	0,06	0,14	0,8	0,06	0,15	0,81	0,04
chr3	89184960	233	G	1	0,06	0,89	0,06	0,07	0,88	0,05	0,07	0,87	0,06	0,08	0,85	0,07	0,09	0,85	0,05	0,08	0,85	0,07	0,21	0,73	0,06	0,23	0,74	0,03	0,22	0,73	0,05
chr3	89184961	234	C	0	0,99	0,01	0,01	0,99	0	0	0,99	0,01	0	1	0	0	1	0	0	1	0	0	0,99	0	0	1	0	0	0,99	0	0
chr3	89184963	235	C	0	1	0	0	1	0	0	1	0	0	1	0	0	1	0	0	1	0	0	1	0	0	1	0	0	1	0	0
chr3	89184965	236	C	0	1	0	0	1	0	0	1	0	0	0,99	0	0,01	0,99	0	0,01	0,99	0	0,01	1	0	0	0,99	0	0,01	0,99	0	0
chr3	89184967	237	G	0	1	0	0	1	0	0	1	0	0	1	0	0	1	0	0	1	0	0	1	0	0	1	0	0	1	0	0
chr3	89184969	238	C	0	1	0	0	1	0	0	1	0	0	1	0	0	1	0	0	1	0	0	1	0	0	1	0	0	1	0	0
chr3	89184972	239	C	0	0,99	0	0	1	0	0	1	0	0	1	0	0	0,99	0	0	1	0	0	0,99	0	0	0,99	0	0	0,99	0	0
chr3	89184973	240	C	0	1	0	0	1	0	0	1	0	0	1	0	0	1	0	0	1	0	0	1	0	0	1	0	0	1	0	0
chr3	89184975	241	C	0	0,99	0	0	1	0	0	0,99	0	0	1	0	0	1	0	0	1	0	0	1	0	0	1	0	0	1	0	0
chr3	89184978	242	C	1	0,04	0,92	0,04	0,04	0,93	0,03	0,03	0,92	0,05	0,08	0,9	0,02	0,07	0,91	0,02	0,07	0,91	0,02	0,09	0,85	0,06	0,11	0,84	0,06	0,1	0,85	0,06
chr3	89184979	243	G	1	0,05	0,92	0,03	0,06	0,91	0,03	0,06	0,91	0,03	0,1	0,88	0,01	0,11	0,88	0,01	0,11	0,87	0,02	0,16	0,79	0,05	0,19	0,79	0,01	0,19	0,78	0,03
chr3	89184980	244	G	0	1	0	0	1	0	0	1	0	0	1	0	0	1	0	0	1	0	0	1	0	0	1	0	0	1	0	0
chr3	89184981	245	G	0	1	0	0	1	0	0	1	0	0	0,99	0	0	0,99	0	0	0,99	0	0	1	0	0	1	0	0	1	0	0
chr3	89184982	246	G	0	1	0	0	1	0	0	1	0	0	1	0	0	1	0	0	1	0	0	1	0	0	1	0	0	1	0	0
chr3	89184983	247	C	0	1	0	0	1	0	0	1	0	0	1	0	0	1	0	0	1	0	0	1	0	0	1	0	0	1	0	0
chr3	89184985	248	G	0	1	0	0	1	0	0	1	0	0	1	0	0	1	0	0	1	0	0	1	0	0	1	0	0	1	0	0
chr3	89184986	249	G	0	1	0	0	1	0	0	1	0	0	1	0	0	1	0	0	1	0	0	1	0	0	1	0	0	1	0	0
chr3	89184987	250	G	0	1	0	0	1	0	0	1	0	0	1	0	0	1	0	0	1	0	0	1	0	0	1	0	0	1	0	0
chr3	89184988	251	G	0	1	0	0	1	0	0	1	0	0	1	0	0	1	0	0	1	0	0	1	0	0	1	0	0	1	0	0
chr3	89184989	252	G	0	1	0	0	1	0	0	1	0	0	1	0	0	1	0	0	1	0	0	1	0	0	1	0	0	1	0	0
chr3	89184990	253	C	0	1	0	0	1	0	0	1	0	0	1	0	0	1	0	0	1	0	0	0,99	0,01	0	0,99	0,01	0	0,99	0,01	0
chr3	89184993	254	C	0	1	0	0	1	0	0	1	0	0	1	0	0	1	0	0	1	0	0	1	0	0	1	0	0	1	0	0
chr3	89184994	255	C	0	1	0	0	1	0	0	1	0	0	1	0	0	1	0	0	1	0	0	1	0	0	1	0	0	1	0	0
chr3	89184996	256	G	0	1	0	0	1	0	0	1	0	0	1	0	0	1	0	0	1	0	0	1	0	0	1	0	0	1	0	0
chr3	89184997	257	C	0	1	0	0	1	0	0	1	0	0	1	0	0	1	0	0	1	0	0	1	0	0	1	0	0	1	0	0
chr3	89184998	258	C	0	1	0	0	1	0	0	1	0	0	1	0	0	1	0	0	1	0	0	1	0	0	1	0	0	1	0	0
chr3	89184999	259	C	0	1	0	0	1	0	0	1	0	0	1	0	0	1	0	0	1	0	0	1	0	0	1	0	0	1	0	0
chr3	89185001	260	C	0	1	0	0	1	0	0	1	0	0	1	0	0	1	0	0	1	0	0	1	0	0	1	0	0	1	0	0
chr3	89185003	261	G	0	1	0	0	1	0	0	1	0	0	1	0	0	1	0	0	1	0	0	1	0	0	1	0	0	1	0	0
chr3	89185004	262	C	0	1	0	0	1	0	0	1	0	0	1	0	0	1	0	0	1	0	0	1	0	0	1	0	0	1	0	0
chr3	89185005	263	C	0	1	0	0	1	0	0	1	0	0	1	0	0	1	0	0	1	0	0	1	0	0	1	0	0	1	0	0
chr3	89185008	264	G	0	1	0	0	1	0	0	1	0	0	1	0	0	1	0	0	1	0	0	0,99	0	0	1	0	0	1	0	0
chr3	89185015	265	C	0	1	0	0	1	0	0	1	0	0	1	0	0	1	0	0	1	0	0	1	0	0	1	0	0	1	0	0
chr3	89185017	266	C	0	1	0	0	1	0	0	1	0	0	1	0	0	1	0	0	1	0	0	1	0	0	1	0	0	1	0	0
chr3	89185018	267	C	0	1	0	0	1	0	0	1	0	0	1	0	0	1	0	0	1	0	0	1	0	0	1	0	0	1	0	0
chr3	89185022	268	C	0	1	0	0	1	0	0	1	0	0	1	0	0	1	0	0	1	0	0	1	0	0	1	0	0	1	0	0

chr3	89679038	269	G	0	1	0	0	1	0	0	1	0	0	1	0	0	1	0	0	1	0	0	1	0	0	1	0	0	1	0	0
chr3	89679041	270	G	0	1	0	0	1	0	0	1	0	0	1	0	0	1	0	0	1	0	0	1	0	0	1	0	0	1	0	0
chr3	89679051	271	G	0	1	0	0	1	0	0	1	0	0	1	0	0	1	0	0	1	0	0	1	0	0	1	0	0	1	0	0
chr3	89679054	272	G	0	1	0	0	1	0	0	1	0	0	1	0	0	1	0	0	1	0	0	1	0	0	1	0	0	1	0	0
chr3	89679072	273	G	1	0,1	0,86	0,04	0,1	0,86	0,04	0,07	0,92	0	0,11	0,79	0,1	0,14	0,81	0,05	0,12	0,83	0,05	0,48	0,52	0	0,5	0,5	0	0,49	0,51	0
chr3	89679075	274	G	0	1	0	0	1	0	0	1	0	0	1	0	0	1	0	0	1	0	0	1	0	0	1	0	0	0,99	0	0
chr3	89679079	275	G	0	1	0	0	1	0	0	1	0	0	0,99	0	0	0,99	0	0	1	0	0	1	0	0	0,99	0	0	1	0	0
chr3	89679081	276	G	0	1	0	0	1	0	0	1	0	0	0,99	0	0	1	0	0	1	0	0	0,99	0	0	0,99	0	0	0,99	0,01	0
chr3	89679091	277	G	0	1	0	0	1	0	0	1	0	0	1	0	0	1	0	0	1	0	0	1	0	0	0,99	0	0,01	0,99	0	0,01
chr3	89679103	278	G	0	1	0	0	1	0	0	1	0	0	1	0	0	1	0	0	1	0	0	1	0	0	1	0	0	1	0	0
chr3	89679107	279	G	1	0,04	0,85	0,11	0,07	0,83	0,1	0,05	0,89	0,06	0,23	0,72	0,04	0,29	0,7	0,01	0,26	0,71	0,02	0,69	0,31	0	0,69	0,31	0	0,68	0,32	0
chr3	89679108	280	G	0	1	0	0	1	0	0	1	0	0	1	0	0	1	0	0	1	0	0	1	0	0	1	0	0	1	0	0
chr3	89679109	281	G	0	1	0	0	1	0	0	1	0	0	1	0	0	1	0	0	1	0	0	1	0	0	1	0	0	1	0	0
chr3	89679117	282	C	0	1	0	0	1	0	0	1	0	0	1	0	0	1	0	0	1	0	0	1	0	0	1	0	0	1	0	0
chr3	89679123	283	G	0	1	0	0	1	0	0	1	0	0	1	0	0	1	0	0	1	0	0	1	0	0	1	0	0	1	0	0
chr3	89679126	284	C	0	1	0	0	1	0	0	1	0	0	1	0	0	1	0	0	1	0	0	1	0	0	1	0	0	1	0	0
chr3	89679132	285	C	0	1	0	0	1	0	0	1	0	0	1	0	0	1	0	0	1	0	0	1	0	0	1	0	0	1	0	0
chr3	89679136	286	G	0	1	0	0	0,99	0	0	0,99	0	0	1	0	0	1	0	0	1	0	0	1	0	0	1	0	0	1	0	0
chr3	89679137	287	C	0	1	0	0	1	0	0	1	0	0	1	0	0	1	0	0	1	0	0	1	0	0	1	0	0	1	0	0
chr3	89679141	288	C	0	1	0	0	1	0	0	1	0	0	1	0	0	1	0	0	1	0	0	1	0	0	1	0	0	1	0	0
chr3	89679142	289	C	0	1	0	0	1	0	0	1	0	0	1	0	0	1	0	0	1	0	0	0,99	0	0,01	1	0	0	1	0	0
chr3	89679144	290	G	0	1	0	0	1	0	0	1	0	0	1	0	0	1	0	0	1	0	0	1	0	0	1	0	0	1	0	0
chr3	89679146	291	C	0	1	0	0	1	0	0	1	0	0	1	0	0	1	0	0	1	0	0	1	0	0	1	0	0	1	0	0
chr3	89679148	292	G	0	1	0	0	1	0	0	1	0	0	1	0	0	1	0	0	1	0	0	1	0	0	1	0	0	1	0	0
chr3	89679149	293	G	0	1	0	0	1	0	0	1	0	0	1	0	0	1	0	0	1	0	0	1	0	0	1	0	0	1	0	0
chr3	89679150	294	C	0	1	0	0	1	0	0	1	0	0	1	0	0	1	0	0	1	0	0	0,98	0,01	0,01	0,99	0,01	0	0,99	0,01	0
chr3	89679155	295	C	1	0,04	0,6	0,36	0,04	0,6	0,36	0,04	0,64	0,32	0,49	0,51	0	0,51	0,49	0	0,51	0,49	0	0,64	0,16	0,2	0,88	0,12	0	0,88	0,12	0
chr3	89679156	296	G	1	0,01	0,77	0,22	0,01	0,76	0,23	0,01	0,81	0,18	0,41	0,59	0	0,48	0,52	0	0,45	0,55	0	0,83	0,17	0	0,82	0,18	0	0,82	0,18	0
chr3	89679158	297	C	0	1	0	0	1	0	0	1	0	0	1	0	0	1	0	0	1	0	0	1	0	0	1	0	0	1	0	0
chr3	89679162	298	C	1	0,04	0,72	0,25	0,04	0,71	0,25	0,04	0,75	0,2	0,35	0,64	0,01	0,37	0,63	0	0,37	0,63	0	0,52	0,32	0,16	0,74	0,26	0	0,74	0,26	0
chr3	89679163	299	G	1	0,02	0,81	0,18	0,02	0,8	0,18	0,01	0,84	0,15	0,37	0,63	0	0,42	0,58	0	0,39	0,61	0	0,8	0,2	0	0,8	0,2	0	0,79	0,21	0
chr3	89679164	300	C	0	1	0	0	1	0	0	1	0	0	1	0	0	1	0	0	1	0	0	0,99	0	0,01	1	0	0	1	0	0
chr3	89679165	301	C	0	0,99	0	0	0,99	0	0	0,99	0	0	1	0	0	1	0	0	1	0	0	1	0	0	1	0	0	1	0	0
chr3	89679167	302	C	0	1	0	0	1	0	0	1	0	0	1	0	0	1	0	0	1	0	0	0,99	0	0	1	0	0	1	0	0
chr3	89679168	303	C	0	0,99	0	0	0,99	0	0	0,99	0	0	1	0	0	1	0	0	1	0	0	0,98	0	0,02	1	0	0	1	0	0
chr3	89679169	304	C	0	0,98	0,02	0	0,98	0,02	0	0,98	0,02	0	0,97	0,01	0,02	0,97	0,01	0,02	0,97	0,01	0,02	0,95	0,01	0,03	0,99	0,01	0	0,99	0,01	0
chr3	89679171	305	C	0	1	0	0	1	0	0	1	0	0	0,99	0,01	0	0,99	0,01	0	0,99	0,01	0	1	0	0	0,99	0	0,01	0,99	0	0,01
chr3	89679173	306	C	1	0,07	0,58	0,35	0,07	0,57	0,36	0,07	0,61	0,31	0,46	0,54	0	0,49	0,51	0	0,49	0,51	0	0,66	0,22	0,12	0,86	0,14	0	0,86	0,14	0
chr3	89679174	307	G	1	0,02	0,77	0,21	0,03	0,76	0,22	0,02	0,81	0,17	0,41	0,59	0	0,48	0,52	0	0,44	0,56	0	0,85	0,15	0	0,86	0,14	0	0,85	0,15	0
chr3	89679176	308	G	0	0,99	0	0,01	0,99	0	0,01	0,99	0	0,01	0,99	0	0,01	0,99	0	0,01	0,99	0	0,01	0,99	0	0,01	0,99	0	0,01	0,98	0	0,01
chr3	89679177	309	C	0	0,99	0,01	0	0,99	0,01	0	0,99	0,01	0	0,98	0	0,01	0,98	0	0,01	0,98	0	0,01	0,98	0,01	0,01	0,99	0,01	0	0,99	0,01	0
chr3	89679180	310	C	0	1	0	0	1	0	0	1	0	0	1	0	0	1	0	0	1	0	0	0,99	0	0,01	1	0	0	1	0	0
chr3	89679181	311	C	0	1	0	0	1	0	0	1	0	0	1	0	0	1	0	0	1	0	0	0,99	0	0,01	1	0	0	1	0	0
chr3	89679182	312	C	0	1	0	0	1	0	0	1	0	0	0,99	0	0,01	0,99	0	0,01	0,99	0	0,01	0,99	0	0,01	1	0	0	1	0	0
chr3	89679184	313	G	0	1	0	0	1	0	0	1	0	0	1	0	0	1	0	0	1	0	0	0,99	0	0	1	0	0	1	0	0
chr3	89679186	314	C	1	0,05	0,61	0,35	0,05	0,6	0,35	0,05	0,64	0,32	0,44	0,56	0	0,46	0,54	0	0,46	0,54	0	0,67	0,22	0,11	0,85	0,15	0	0,86	0,14	0

chr3	89679187	315	G	1	0,02	0,77	0,21	0,02	0,75	0,23	0,02	0,79	0,19	0,45	0,55	0	0,5	0,5	0	0,47	0,53	0	0,87	0,13	0	0,87	0,13	0	0,87	0,13	0
chr3	89679188	316	C	0	1	0	0	1	0	0	1	0	0	0,99	0,01	0	0,99	0,01	0	0,99	0,01	0	0,99	0	0,01	1	0	0	1	0	0
chr3	89679190	317	G	0	1	0	0	1	0	0	1	0	0	1	0	0	1	0	0	1	0	0	1	0	0	1	0	0	1	0	0
chr3	89679192	318	G	0	1	0	0	1	0	0	1	0	0	1	0	0	1	0	0	1	0	0	1	0	0	1	0	0	1	0	0
chr3	89679193	319	G	0	1	0	0	1	0	0	1	0	0	1	0	0	1	0	0	1	0	0	1	0	0	1	0	0	1	0	0
chr3	89679194	320	C	0	1	0	0	1	0	0	1	0	0	1	0	0	1	0	0	1	0	0	0,99	0	0,01	1	0	0	1	0	0
chr3	89679196	321	C	0	1	0	0	1	0	0	1	0	0	0,99	0	0	1	0	0	0,99	0	0	0,99	0	0,01	1	0	0	1	0	0
chr3	89679198	322	G	0	1	0	0	1	0	0	1	0	0	1	0	0	1	0	0	1	0	0	1	0	0	1	0	0	1	0	0
chr3	89679199	323	C	0	1	0	0	1	0	0	1	0	0	1	0	0	1	0	0	1	0	0	0,99	0	0,01	1	0	0	1	0	0
chr3	89679200	324	C	1	0,04	0,63	0,33	0,04	0,62	0,34	0,04	0,66	0,3	0,44	0,56	0	0,47	0,53	0	0,47	0,53	0	0,66	0,24	0,1	0,84	0,16	0	0,85	0,15	0
chr3	89679201	325	G	1	0,03	0,76	0,21	0,03	0,74	0,23	0,03	0,8	0,17	0,43	0,57	0	0,48	0,52	0	0,45	0,55	0	0,85	0,15	0	0,85	0,15	0	0,84	0,16	0
chr3	89679202	326	G	0	1	0	0	1	0	0	1	0	0	1	0	0	1	0	0	1	0	0	1	0	0	1	0	0	1	0	0
chr3	89679203	327	C	0	1	0	0	1	0	0	1	0	0	1	0	0	1	0	0	1	0	0	0,99	0	0,01	1	0	0	1	0	0
chr3	89679210	328	C	0	1	0	0	1	0	0	1	0	0	0,99	0,01	0	0,99	0,01	0	0,99	0,01	0	0,99	0	0,01	1	0	0	1	0	0
chr3	89679212	329	C	0	1	0	0	1	0	0	1	0	0	1	0	0	1	0	0	1	0	0	1	0	0	1	0	0	1	0	0
chr3	89679213	330	C	0	1	0	0	1	0	0	1	0	0	1	0	0	1	0	0	1	0	0	1	0	0	1	0	0	1	0	0
chr3	89679214	331	C	0	1	0	0	1	0	0	1	0	0	1	0	0	1	0	0	1	0	0	1	0	0	1	0	0	1	0	0
chr3	89679216	332	G	0	1	0	0	1	0	0	1	0	0	1	0	0	1	0	0	1	0	0	1	0	0	1	0	0	1	0	0
chr3	89679218	333	C	0	1	0	0	1	0	0	1	0	0	1	0	0	1	0	0	1	0	0	1	0	0	1	0	0	1	0	0
chr3	89679220	334	C	0	1	0	0	1	0	0	1	0	0	1	0	0	1	0	0	1	0	0	1	0	0	1	0	0	1	0	0
chr3	89679224	335	G	0	1	0	0	1	0	0	1	0	0	1	0	0	1	0	0	1	0	0	1	0	0	1	0	0	1	0	0
chr3	89679226	336	G	0	1	0	0	1	0	0	1	0	0	1	0	0	1	0	0	1	0	0	1	0	0	1	0	0	1	0	0
chr3	89679232	337	C	0	1	0	0	1	0	0	1	0	0	1	0	0	1	0	0	1	0	0	1	0	0	1	0	0	1	0	0
chr4	44628612	338	C	0	0,98	0,01	0,01	1	0	0	0,99	0	0	0,98	0,01	0,01	1	0	0	1	0	0	0,99	0,01	0,01	1	0	0	1	0	0
chr4	44628613	339	C	0	0,98	0,01	0,01	1	0	0	1	0	0	0,98	0,01	0,01	1	0	0	0,99	0	0	0,99	0	0,01	1	0	0	1	0	0
chr4	44628615	340	C	0	0,98	0,01	0,01	1	0	0	1	0	0	0,98	0,01	0,01	1	0	0	0,99	0	0	0,99	0	0,01	1	0	0	1	0	0
chr4	44628620	341	C	0	0,98	0,01	0,01	1	0	0	1	0	0	0,97	0,01	0,01	1	0	0	0,99	0	0	0,99	0	0,01	1	0	0	1	0	0
chr4	44628623	342	C	0	0,98	0,01	0,01	1	0	0	1	0	0	0,98	0,01	0,01	1	0	0	1	0	0	0,99	0	0,01	1	0	0	1	0	0
chr4	44628630	343	C	0	0,98	0,01	0,01	1	0	0	1	0	0	0,98	0,01	0,01	1	0	0	0,99	0	0	0,99	0	0,01	1	0	0	1	0	0
chr4	44628631	344	C	0	0,98	0,01	0,01	1	0	0	1	0	0	0,98	0,01	0,01	1	0	0	1	0	0	0,99	0	0,01	1	0	0	1	0	0
chr4	44628632	345	C	0	0,98	0,01	0,01	1	0	0	0,99	0	0	0,98	0,01	0,01	1	0	0	0,99	0	0	0,99	0,01	0,01	1	0	0	1	0	0
chr4	44628640	346	C	0	0,96	0,03	0,01	0,98	0,02	0	0,98	0,01	0	0,98	0,01	0,01	1	0	0	0,99	0	0	0,99	0	0,01	1	0	0	1	0	0
chr4	44628641	347	C	0	0,98	0,01	0,01	1	0	0	0,99	0	0	0,98	0,01	0,01	0,99	0	0	0,99	0	0	0,98	0,01	0,01	1	0	0	1	0	0
chr4	44628642	348	C	0	0,98	0,01	0,01	1	0	0	1	0	0	0,98	0,01	0,01	1	0	0	0,99	0	0	0,99	0	0,01	1	0	0	1	0	0
chr4	44628643	349	C	1	0,21	0,75	0,05	0,23	0,75	0,02	0,25	0,7	0,04	0,19	0,79	0,03	0,2	0,8	0,01	0,15	0,84	0,01	0,22	0,76	0,03	0,23	0,75	0,02	0,23	0,76	0,01
chr4	44628646	350	C	1	0,11	0,86	0,03	0,12	0,87	0,01	0,11	0,86	0,03	0,07	0,92	0,01	0,06	0,94	0	0,04	0,95	0	0,04	0,95	0,01	0,06	0,93	0	0,06	0,94	0
chr4	44628652	351	C	0	0,98	0,01	0,01	0,99	0	0	0,99	0	0	0,95	0,04	0,01	0,97	0,02	0	0,96	0,04	0	0,99	0	0,01	1	0	0	1	0	0
chr4	44628657	352	C	0	0,98	0,01	0,01	1	0	0	1	0	0	0,98	0,01	0,01	1	0	0	1	0	0	0,99	0	0,01	1	0	0	1	0	0
chr4	44628659	353	C	0	0,98	0,01	0,01	1	0	0	1	0	0	0,98	0,01	0,01	1	0	0	1	0	0	0,98	0	0,01	1	0	0	1	0	0
chr4	44628661	354	C	0	0,98	0,01	0,01	1	0	0	1	0	0	0,98	0,01	0,01	1	0	0	1	0	0	0,99	0	0,01	1	0	0	1	0	0
chr4	44628663	355	C	0	0,97	0,01	0,02	1	0	0	0,99	0	0	0,98	0,01	0,01	1	0	0	1	0	0	0,98	0,01	0,01	1	0	0	1	0	0
chr4	44628665	356	C	0	0,98	0,01	0,01	1	0	0	1	0	0	0,97	0,01	0,01	1	0	0	0,99	0	0	0,98	0,01	0,01	1	0	0	1	0	0
chr4	44628666	357	C	0	0,98	0,01	0,01	1	0	0	1	0	0	0,98	0,01	0,01	1	0	0	1	0	0	0,99	0	0,01	1	0	0	1	0	0
chr4	44628667	358	C	0	0,98	0,01	0,01	1	0	0	0,99	0	0	0,98	0,01	0,01	1	0	0	1	0	0	0,99	0	0,01	1	0	0	1	0	0
chr4	44628670	359	C	0	0,98	0,01	0,01	1	0	0	0,99	0	0	0,98	0,01	0,01	1	0	0	0,99	0	0	0,98	0	0,01	1	0	0	1	0	0
chr4	44628671	360	C	0	0,98	0,01	0,01	1	0	0	1	0	0	0,98	0,01	0,01	1	0	0	1	0	0	0,99	0	0,01	1	0	0	1	0	0

chr4	44628679	361	C	0	0,98	0,01	0,01	1	0	0	0,99	0	0,01	0,98	0,01	0,01	0,99	0	0	0,98	0,01	0,01	0,99	0	0,01	0,99	0	0,01	0,99	0	0
chr4	44628680	362	C	0	0,98	0,01	0,01	1	0	0	1	0	0	0,98	0,01	0,01	1	0	0	1	0	0	0,99	0,01	0,01	1	0	0	1	0	0
chr4	44628684	363	C	1	0,14	0,8	0,06	0,1	0,81	0,08	0,1	0,79	0,11	0,08	0,89	0,03	0,11	0,88	0	0,09	0,9	0,01	0,09	0,87	0,04	0,11	0,88	0,01	0,11	0,88	0,01
chr4	44628686	364	C	0	0,98	0,01	0,01	0,99	0	0	0,99	0	0	0,98	0,01	0,01	0,99	0	0	1	0	0	0,99	0,01	0,01	1	0	0	1	0	0
chr4	44628687	365	C	0	0,97	0,01	0,01	1	0	0	0,99	0	0	0,98	0,01	0,01	0,99	0	0	1	0	0	0,99	0,01	0,01	1	0	0	1	0	0
chr4	44628690	366	C	0	0,98	0,01	0,01	1	0	0	0,99	0	0	0,98	0,01	0,01	0,99	0	0	0,99	0	0	0,99	0,01	0,01	1	0	0	1	0	0
chr4	44628692	367	C	0	0,97	0,01	0,02	0,99	0,01	0,01	0,99	0	0	0,98	0,01	0,01	1	0	0	1	0	0	0,99	0	0,01	1	0	0	1	0	0
chr4	44628694	368	C	0	0,98	0,01	0,01	1	0	0	0,99	0,01	0,01	0,97	0,01	0,01	1	0	0	1	0	0	0,99	0	0,01	1	0	0	1	0	0
chr4	44628695	369	C	0	0,98	0,01	0,01	1	0	0	0,99	0,01	0,01	0,98	0,01	0,01	1	0	0	0,99	0	0	0,99	0,01	0,01	1	0	0	1	0	0
chr4	44628697	370	C	0	0,98	0,01	0,01	1	0	0	0,99	0	0	0,98	0,01	0,01	1	0	0	1	0	0	0,98	0,01	0,01	1	0	0	1	0	0
chr4	44628698	371	C	0	0,98	0,01	0,01	1	0	0	1	0	0	0,98	0,01	0,01	1	0	0	0,99	0	0	0,99	0	0,01	1	0	0	1	0	0
chr4	44628700	372	C	0	0,98	0,01	0,01	1	0	0	1	0	0	0,98	0,01	0,01	1	0	0	1	0	0	0,99	0	0,01	1	0	0	1	0	0
chr4	44628701	373	C	0	0,98	0,01	0,01	1	0	0	0,99	0,01	0	0,98	0,01	0,01	1	0	0	0,99	0,01	0,01	0,99	0	0,01	0,99	0	0	1	0	0
chr4	44628702	374	C	0	0,97	0,01	0,02	0,99	0	0	1	0	0	0,98	0,01	0,01	1	0	0	1	0	0	0,99	0	0,01	1	0	0	1	0	0
chr4	44628703	375	C	0	0,98	0,01	0,01	1	0	0	0,99	0	0	0,98	0,01	0,01	1	0	0	0,99	0	0,01	0,98	0,01	0,01	1	0	0	1	0	0
chr4	44628706	376	C	0	0,98	0,01	0,01	1	0	0	0,99	0	0	0,98	0,01	0,01	1	0	0	1	0	0	0,99	0	0,01	1	0	0	1	0	0
chr4	44628707	377	C	0	0,98	0,01	0,01	1	0	0	1	0	0	0,98	0	0,01	1	0	0	0,99	0	0	0,99	0	0,01	1	0	0	1	0	0
chr4	44628708	378	C	0	0,98	0,01	0,01	1	0	0	0,99	0	0	0,99	0,01	0	1	0	0	0,99	0	0	0,98	0	0,01	1	0	0	1	0	0
chr4	44628712	379	C	0	0,98	0,01	0,01	1	0	0	1	0	0	0,98	0,01	0,01	1	0	0	1	0	0	0,99	0	0,01	1	0	0	1	0	0
chr4	44628716	380	C	0	0,98	0,01	0,01	0,99	0	0	0,99	0	0	0,98	0,01	0,01	1	0	0	0,99	0	0	0,98	0,01	0,01	1	0	0	1	0	0
chr4	44628717	381	C	0	0,98	0,01	0,01	1	0	0	1	0	0	0,98	0,01	0,01	1	0	0	0,99	0	0	0,99	0	0,01	1	0	0	1	0	0
chr4	44628737	382	C	0	0,98	0,01	0,01	0,99	0	0	0,99	0	0	0,98	0,01	0,01	1	0	0	0,99	0	0,01	0,99	0,01	0,01	0,99	0	0	1	0	0
chr4	44628738	383	C	0	0,98	0,01	0,01	0,99	0	0	0,99	0	0	0,98	0,01	0,01	1	0	0	1	0	0	0,99	0	0,01	1	0	0	1	0	0
chr4	44628740	384	C	0	0,98	0,01	0,01	0,99	0	0	1	0	0	0,98	0,01	0,01	1	0	0	0,99	0	0,01	0,99	0	0,01	1	0	0	1	0	0
chr4	44628746	385	C	0	0,96	0,01	0,03	1	0	0	0,98	0,01	0,01	0,98	0,01	0,01	1	0	0	0,99	0,01	0	0,98	0,01	0,01	1	0	0	1	0	0
chr4	44628750	386	C	0	0,97	0,01	0,01	1	0	0	0,99	0	0	0,98	0,01	0,01	1	0	0	1	0	0	0,98	0,01	0,01	0,99	0,01	0	0,99	0	0
chr4	44628751	387	C	0	0,98	0,01	0,01	0,99	0	0	1	0	0	0,98	0,01	0,01	1	0	0	0,99	0	0,01	0,99	0	0,01	1	0	0	1	0	0
chr4	44628786	388	C	0	0,96	0,02	0,02	0,98	0,01	0,01	0,98	0,01	0,01	0,93	0,04	0,03	0,98	0,01	0,02	0,98	0,01	0,01	0,97	0,01	0,01	0,99	0,01	0,01	0,98	0,01	0,01
chr4	44628787	389	C	1	0,12	0,85	0,03	0,06	0,93	0,01	0,08	0,9	0,02	0,03	0,95	0,02	0,04	0,95	0,01	0,04	0,93	0,03	0,13	0,85	0,02	0,06	0,89	0,05	0,07	0,9	0,03
chr4	44628795	390	C	0	0,98	0,01	0,01	1	0	0	0,99	0	0	0,98	0,01	0,01	1	0	0	0,99	0	0	0,99	0	0,01	1	0	0	1	0	0
chr4	44628796	391	C	0	0,97	0,01	0,02	0,99	0	0	0,99	0	0	0,98	0,01	0,01	0,99	0	0	0,99	0	0,01	0,98	0,01	0,01	1	0	0	0,99	0	0
chr4	44628797	392	C	0	0,98	0,01	0,01	1	0	0	1	0	0	0,98	0,01	0,01	1	0	0	0,99	0,01	0	0,99	0	0,01	0,99	0	0	1	0	0
chr4	44628798	393	C	0	0,98	0,01	0,01	1	0	0	0,99	0,01	0	0,98	0,01	0,01	1	0	0	1	0	0	0,99	0	0,01	1	0	0	1	0	0
chr4	44628799	394	C	1	0,17	0,77	0,06	0,11	0,86	0,03	0,13	0,76	0,11	0,09	0,9	0,01	0,09	0,91	0	0,08	0,92	0	0,11	0,87	0,02	0,07	0,92	0	0,07	0,92	0
chr4	44628804	395	C	0	0,98	0,01	0,01	0,99	0	0	0,99	0	0	0,98	0,01	0,01	1	0	0	0,99	0,01	0	0,99	0	0,01	1	0	0	0,99	0	0
chr4	44628807	396	C	0	0,98	0,01	0,01	0,99	0	0	0,99	0	0	0,98	0,01	0,01	1	0	0	1	0	0	0,99	0	0,01	1	0	0	1	0	0
chr4	44628819	397	C	0	0,98	0,01	0,01	1	0	0	1	0	0	0,98	0,01	0,01	1	0	0	1	0	0	0,99	0	0,01	1	0	0	1	0	0
chr4	44628821	398	C	0	0,98	0,01	0,01	1	0	0	0,99	0	0	0,99	0,01	0,01	1	0	0	0,99	0	0	0,99	0	0	1	0	0	1	0	0
chr4	44628828	399	C	0	0,98	0,01	0,01	0,99	0	0	0,99	0	0	0,98	0,01	0,01	1	0	0	0,99	0	0	0,99	0	0,01	1	0	0	1	0	0
chr4	44628832	400	C	1	0,04	0,94	0,02	0,05	0,95	0,01	0,07	0,91	0,02	0,06	0,92	0,02	0,03	0,97	0	0,01	0,98	0	0,07	0,9	0,03	0,03	0,9	0,07	0,03	0,91	0,06
chr4	44628834	401	C	0	0,97	0,01	0,02	1	0	0	0,99	0	0	0,98	0,01	0,01	0,99	0	0	0,99	0	0	0,98	0	0,01	0,99	0	0	1	0	0
chr4	44628843	402	C	0	0,98	0,01	0,01	1	0	0	0,99	0,01	0	0,98	0,01	0,01	1	0	0	1	0	0	0,99	0	0,01	1	0	0	1	0	0
chr4	44628845	403	C	0	0,98	0,01	0,01	1	0	0	0,99	0	0	0,98	0	0,01	1	0	0	0,99	0	0	0,99	0	0	1	0	0	1	0	0
chr4	44628851	404	C	0	0,98	0,01	0,01	0,99	0	0	0,99	0	0	0,98	0,01	0,01	0,99	0	0	0,99	0	0,01	0,99	0	0	1	0	0	1	0	0
chr4	44628852	405	C	0	0,98	0,01	0,01	1	0	0	1	0	0	0,98	0,01	0,01	0,99	0	0	0,99	0	0	0,98	0,01	0,01	0,99	0	0	0,99	0,01	0
chr4	44628856	406	C	0	0,98	0,01	0,01	1	0	0	0,99	0	0	0,98	0,01	0,01	1	0	0	0,99	0	0	0,97	0	0,03	0,98	0	0,02	0,98	0	0,01

chr4	44628862	407	C	0	0,98	0,01	0,01	1	0	0	1	0	0	0,98	0,01	0,01	1	0	0	0,99	0	0	0,99	0	0	0,99	0	0	1	0	0
chr4	44628869	408	C	0	0,98	0,01	0,01	1	0	0	1	0	0	0,97	0,01	0,02	1	0	0	0,99	0	0	0,99	0	0,01	1	0	0	0,99	0	0,01
chr4	44628871	409	C	0	0,98	0,01	0,01	1	0	0	0,99	0	0	0,98	0,01	0,01	1	0	0	0,99	0	0,01	0,99	0,01	0,01	1	0	0	1	0	0
chr4	44628876	410	C	0	0,98	0,01	0,01	0,99	0	0	0,99	0	0	0,98	0,01	0,01	0,99	0	0	0,99	0	0	0,99	0	0,01	1	0	0	0,99	0	0
chr4	44628882	411	C	0	0,98	0,01	0,01	0,99	0	0	0,99	0	0	0,97	0,02	0,01	1	0	0	0,99	0	0	0,99	0	0,01	0,99	0	0	0,99	0	0
chr4	44628883	412	C	0	0,98	0,01	0,01	1	0	0	1	0	0	0,96	0,01	0,03	0,99	0	0	0,99	0	0	0,99	0	0,01	0,99	0	0	1	0	0
chr4	44628889	413	C	0	0,98	0,01	0,01	1	0	0	0,99	0	0	0,98	0,01	0,01	0,99	0	0	0,99	0	0,01	0,98	0,01	0,01	0,99	0	0	1	0	0
chr4	44628890	414	C	0	0,98	0,01	0,01	0,99	0	0	0,99	0,01	0	0,98	0,01	0,01	1	0	0	0,99	0	0,01	0,99	0,01	0,01	1	0	0	1	0	0
chr4	44628891	415	C	0	0,98	0,01	0,01	0,99	0	0	0,99	0	0	0,98	0,01	0,01	1	0	0	0,99	0	0	0,99	0	0	1	0	0	1	0	0
chr4	44628893	416	C	0	0,98	0,01	0,01	1	0	0	1	0	0	0,98	0,01	0,01	1	0	0	0,99	0	0	0,99	0	0,01	1	0	0	1	0	0
chr4	44628898	417	C	0	0,98	0,01	0,01	0,99	0	0	0,99	0	0	0,98	0,01	0,01	1	0	0	0,99	0	0	0,99	0	0,01	1	0	0	1	0	0
chr4	44628906	418	C	0	0,98	0,01	0,01	1	0	0	1	0	0	0,98	0,01	0,01	0,99	0	0	0,99	0	0,01	0,99	0	0,01	1	0	0	1	0	0
chr4	44628908	419	C	1	0,1	0,86	0,04	0,16	0,83	0,01	0,15	0,82	0,03	0,08	0,9	0,02	0,13	0,86	0	0,11	0,89	0	0,04	0,93	0,03	0,09	0,84	0,07	0,08	0,84	0,08
chr4	44628911	420	C	0	0,98	0,01	0,01	1	0	0	0,99	0	0	0,98	0,01	0,01	1	0	0	1	0	0	0,99	0	0	1	0	0	1	0	0
chr4	44628912	421	C	0	0,98	0,01	0,01	1	0	0	1	0	0	0,98	0,01	0,01	1	0	0	1	0	0	0,99	0	0	1	0	0	1	0	0
chr4	44628918	422	C	0	0,98	0,01	0,01	1	0	0	1	0	0	0,98	0,01	0,01	1	0	0	1	0	0	0,99	0	0	1	0	0	1	0	0
chr4	44628931	423	C	0	0,98	0,01	0,01	0,99	0	0	0,99	0	0	0,97	0,01	0,02	1	0	0	1	0	0	0,99	0	0,01	1	0	0	1	0	0

Lux estimates

					BIOLOGICAL SAMPLES											
					dp			sp			naïve					
Chrom	Position	ID	Nuc	CpG	p("C")	p("5m(	p("5hn	p("C")	p("5m(	p("5hn	p("C")	p("5m(	p("5hn	BF (DP/ SP)	BF (DP/ naïve)	BF (SP/ naïve)
chr2	11124412	1	C	0	0,99	0	0	0,99	0	0	0,99	0	0	0,000097	0,000093	0,000096
chr2	11124413	2	C	0	0,99	0	0	0,99	0	0,01	0,99	0	0	0,000058	0,000065	0,000065
chr2	11124414	3	C	0	0,99	0	0	0,99	0	0	0,99	0	0	0,000063	0,000051	0,000059
chr2	11124418	4	C	0	0,99	0	0	0,99	0	0	0,99	0	0	0,000096	0,000091	0,000093
chr2	11124422	5	C	0	0,99	0	0	0,99	0	0	0,99	0	0	0,000052	0,000054	0,000049
chr2	11124423	6	C	0	0,99	0	0	0,99	0	0	0,99	0	0	0,000068	0,000062	0,00007
chr2	11124429	7	C	0	0,99	0	0,01	0,99	0	0	0,99	0	0	0,000142	0,000116	0,000118
chr2	11124431	8	C	0	0,99	0	0	0,99	0,01	0,01	0,99	0	0,01	0,000081	0,000129	0,00015
chr2	11124435	9	C	0	0,99	0	0	0,99	0,01	0	0,99	0,01	0	0,000129	0,000149	0,00015
chr2	11124442	10	C	0	0,99	0	0	0,99	0,01	0	0,99	0	0	0,000106	0,003578	0,003684
chr2	11124443	11	C	0	0,99	0	0	0,99	0,01	0	0,99	0	0,01	0,000103	0,000123	0,00013
chr2	11124447	12	C	0	0,99	0,01	0	0,99	0,01	0	0,99	0,01	0	0,000178	0,00019	0,000176
chr2	11124451	13	C	0	0,99	0,01	0	0,99	0,01	0,01	0,99	0,01	0,01	0,000207	0,000241	0,000306
chr2	11124454	14	C	0	0,99	0	0	0,99	0	0,01	0,99	0,01	0	0,000127	0,000165	0,000232
chr2	11124458	15	C	0	0,99	0	0	0,99	0,01	0	0,99	0	0	0,000103	0,000086	0,000116
chr2	11124460	16	C	0	0,99	0,01	0	0,99	0,01	0	0,99	0	0	0,000143	0,000748	0,000846
chr2	11124463	17	C	1	0,02	0,9	0,07	0,06	0,89	0,05	0,25	0,71	0,03	0,039787	1,014601	0,241115
chr2	11124467	18	C	0	0,99	0	0	0,99	0,01	0	0,99	0,01	0	0,00015	0,000108	0,000125
chr2	11124468	19	C	0	0,99	0	0	0,99	0,01	0	0,99	0	0,01	0,000114	0,000152	0,000152
chr2	11124469	20	C	0	0,99	0,01	0	0,99	0	0	0,99	0	0	0,000129	0,002096	0,002003
chr2	11124470	21	C	0	0,99	0	0	0,99	0,01	0	0,99	0	0	0,000115	0,000133	0,000137
chr2	11124471	22	C	0	0,99	0	0	0,99	0	0	0,99	0,01	0,01	0,000117	0,000341	0,0003
chr2	11124474	23	C	0	0,99	0,01	0	0,99	0,01	0	0,99	0	0	0,000144	0,000177	0,000172
chr2	11124479	24	G	0	0,98	0,01	0,02	0,98	0,01	0,01	0,99	0,01	0,01	0,000776	0,000709	0,000424
chr2	11124480	25	C	0	0,99	0,01	0	0,99	0,01	0,01	0,99	0,01	0	0,0002	0,000142	0,000174
chr2	11124481	26	C	0	0,99	0	0	0,99	0	0	0,99	0	0	0,000113	0,000108	0,000112
chr2	11124483	27	C	0	0,99	0	0	0,99	0,01	0	0,99	0	0	0,000127	0,00009	0,00012
chr2	11124484	28	C	0	0,99	0,01	0	0,99	0	0	0,99	0	0,01	0,000133	0,000181	0,000161
chr2	11124485	29	C	0	0,99	0	0,01	0,99	0,01	0	0,99	0	0,01	0,000141	0,000158	0,000125
chr2	11124488	30	G	0	0,98	0,01	0,01	0,98	0,01	0,01	0,99	0,01	0,01	0,000662	0,000605	0,000295
chr2	11124489	31	C	0	0,99	0	0	0,99	0	0,01	0,99	0,01	0	0,000166	0,000118	0,000168
chr2	11124491	32	C	0	0,99	0	0	0,99	0	0	0,99	0	0	0,000101	0,00012	0,000126
chr2	11124492	33	C	0	0,99	0	0	0,99	0	0	0,99	0	0,01	0,000112	0,000451	0,000401
chr2	11124494	34	G	0	0,98	0,01	0,01	0,98	0,01	0,01	0,99	0,01	0,01	0,000485	0,000365	0,00028
chr2	11124495	35	G	0	0,97	0,01	0,02	0,98	0,01	0,01	0,99	0,01	0,01	0,00112	0,001009	0,000373
chr2	11124496	36	C	0	0,99	0,01	0,01	0,99	0,01	0	0,98	0,01	0	0,000293	0,000398	0,000353
chr2	11124498	37	C	1	0,15	0,76	0,09	0,3	0,68	0,02	0,75	0,25	0	0,348552	19979,90686	6,337035
chr2	11124499	38	G	1	0,23	0,18	0,58	0,73	0,18	0,09	0,94	0,05	0,01	41,222765	4,89689E+24	0,168643

chr2	11124502	39	G	0	0,97	0,01	0,02	0,98	0,01	0,01	0,99	0,01	0,01	0,000918	0,000751	0,000378
chr2	11124503	40	C	0	0,99	0,01	0	0,99	0,01	0	0,99	0,01	0	0,000165	0,000144	0,000135
chr2	11124504	41	C	0	0,99	0	0,01	0,99	0	0	0,99	0	0	0,000152	0,000148	0,000113
chr2	11124507	42	G	0	0,98	0,01	0,01	0,98	0,01	0,01	0,99	0,01	0,01	0,000705	0,000567	0,00047
chr2	11124508	43	C	0	0,98	0,01	0,01	0,98	0	0,02	0,96	0,02	0,02	0,000866	0,002736	0,002892
chr2	11124510	44	C	0	0,99	0	0,01	0,99	0	0,01	0,99	0	0,01	0,000171	0,000181	0,000181
chr2	11124511	45	C	0	0,99	0	0	0,99	0	0	0,99	0	0,01	0,0001	0,000162	0,000161
chr2	11124513	46	C	0	0,99	0	0	0,99	0	0	0,99	0	0	0,000086	0,000086	0,000076
chr2	11124514	47	C	0	0,99	0	0	0,99	0	0,01	0,99	0	0,01	0,000138	0,000137	0,000186
chr2	11124516	48	C	0	0,99	0,01	0,01	0,99	0	0	0,99	0	0	0,000172	0,000161	0,000093
chr2	11124517	49	C	1	0,02	0,93	0,05	0,05	0,87	0,08	0,28	0,69	0,04	0,030088	2,24856	0,694344
chr2	11124518	50	G	1	0,03	0,63	0,34	0,25	0,44	0,31	0,47	0,5	0,04	1,083883	39,818593	5,781928
chr2	11124519	51	C	0	0,99	0	0	0,96	0,02	0,01	0,99	0,01	0	0,000124	0,000148	0,000155
chr2	11124520	52	C	0	0,99	0,01	0	0,99	0	0,01	0,99	0,01	0	0,000181	0,000141	0,000174
chr2	11124522	53	C	0	0,99	0	0,01	0,87	0,09	0,05	0,99	0	0	0,000155	0,000155	0,000138
chr2	11124523	54	C	1	0,02	0,95	0,03	0,02	0,93	0,05	0,23	0,76	0,01	0,009217	0,129368	0,358444
chr2	11124524	55	G	1	0,03	0,64	0,33	0,2	0,37	0,44	0,45	0,46	0,09	1,099814	3,190168	5,400096
chr2	11124525	56	C	0	0,99	0,01	0	0,99	0,01	0	0,99	0,01	0	0,000134	0,000262	0,000253
chr2	11124526	57	C	0	0,99	0,01	0	0,99	0	0,01	0,99	0	0,01	0,000182	0,000165	0,000192
chr2	11124528	58	C	0	0,99	0	0,01	0,99	0	0	0,99	0	0	0,000134	0,000133	0,000095
chr2	11124529	59	C	1	0,01	0,93	0,05	0,04	0,9	0,06	0,3	0,7	0,01	0,01797	5,30856	0,903562
chr2	11124530	60	G	1	0,77	0,04	0,19	0,94	0,03	0,03	0,98	0,01	0,01	0,117929	0,124907	0,003826
chr2	11124531	61	C	0	0,99	0	0,01	0,99	0,01	0	0,99	0	0	0,000246	0,000188	0,000195
chr2	11124532	62	C	0	0,99	0	0	0,99	0	0	0,99	0	0	0,000097	0,000098	0,000092
chr2	11124534	63	C	1	0,02	0,9	0,08	0,11	0,86	0,04	0,55	0,45	0	0,098413	20,107965	1,293616
chr2	11124535	64	G	1	0,02	0,37	0,61	0,5	0,22	0,28	0,83	0,15	0,02	24,697697	inf	2,100284
chr2	11124536	65	G	0	0,98	0,01	0,01	0,98	0,01	0,01	0,99	0,01	0,01	0,000493	0,000367	0,000345
chr2	11124537	66	C	0	0,99	0	0	0,99	0	0	0,99	0	0	0,000129	0,000376	0,000353
chr2	11124538	67	C	0	0,99	0	0,01	0,99	0	0,01	0,99	0	0	0,000152	0,00013	0,000136
chr2	11124540	68	C	0	0,99	0	0	0,99	0,01	0	0,99	0	0	0,000144	0,000094	0,000154
chr2	11124542	69	G	0	0,98	0,01	0,01	0,98	0,01	0,01	0,99	0,01	0,01	0,000668	0,000495	0,000328
chr2	11124543	70	C	0	0,99	0,01	0	0,99	0,01	0	0,99	0	0	0,000159	0,000154	0,000134
chr2	11124544	71	C	0	0,99	0,01	0	0,99	0	0	0,99	0	0,01	0,000103	0,000155	0,000128
chr2	11124546	72	C	1	0,05	0,83	0,12	0,2	0,8	0,01	0,65	0,35	0	3,398559	6,12256E+51	1,58882
chr2	11124547	73	G	1	0,12	0,43	0,45	0,63	0,14	0,23	0,89	0,1	0,01	21,205066	425090,7832	0,535652
chr2	11124548	74	G	0	0,98	0,01	0,01	0,98	0,01	0,01	0,99	0,01	0,01	0,000854	0,001795	0,001918
chr2	11124549	75	C	0	0,99	0,01	0	0,99	0,01	0	0,99	0,01	0	0,000158	0,000156	0,000161
chr2	11124550	76	C	0	0,99	0,01	0	0,99	0	0	0,99	0,01	0,01	0,000169	0,000956	0,000829
chr2	11124552	77	C	0	0,99	0	0	0,99	0	0	0,99	0,01	0	0,000115	0,003942	0,003966
chr2	11124553	78	C	1	0,02	0,88	0,1	0,12	0,86	0,01	0,52	0,47	0	0,197299	inf	5,622532
chr2	11124554	79	G	1	0,04	0,4	0,56	0,58	0,17	0,25	0,89	0,1	0,01	25,308818	inf	0,633201
chr2	11124555	80	C	0	0,99	0	0	0,99	0	0	0,99	0	0	0,000131	0,000139	0,000145
chr2	11124556	81	C	0	0,99	0,01	0	0,99	0,01	0	0,99	0	0	0,000172	0,000298	0,000317
chr2	11124558	82	C	0	0,99	0	0	0,99	0	0	0,99	0	0	0,000102	0,0001	0,000094
chr2	11124559	83	C	1	0,14	0,69	0,16	0,3	0,7	0,01	0,7	0,29	0	0,932981	289,737106	2,371698
chr2	11124560	84	G	1	0,16	0,3	0,54	0,65	0,13	0,23	0,91	0,08	0,01	10,460712	1504,254198	0,287376

chr2	11124561	85	G	0	0,98	0,01	0,01	0,99	0,01	0,01	0,99	0,01	0,01	0,000504	0,000435	0,000262
chr2	11124562	86	G	0	0,98	0,01	0,01	0,99	0,01	0,01	0,99	0,01	0,01	0,000546	0,00053	0,000369
chr2	11124563	87	C	0	0,99	0	0	0,99	0	0	0,99	0	0	0,000053	0,000449	0,000453
chr2	11124567	88	G	0	0,98	0,01	0,01	0,98	0,01	0,01	0,99	0,01	0,01	0,000551	0,000402	0,000264
chr2	11124572	89	G	0	0,98	0,01	0,01	0,99	0,01	0,01	0,99	0,01	0,01	0,000532	0,000457	0,000322
chr2	11124576	90	C	0	0,99	0	0	0,99	0	0	0,99	0	0	0,000054	0,00053	0,00005
chr2	11124581	91	C	0	0,99	0	0	0,99	0	0	0,99	0	0	0,000072	0,000073	0,000066
chr2	11124584	92	G	0	0,98	0,01	0,01	0,98	0,01	0,01	0,99	0	0,01	0,000498	0,000368	0,000262
chr2	11124586	93	C	0	0,99	0	0	0,99	0,01	0	0,99	0	0	0,000092	0,000289	0,000295
chr2	11124588	94	G	0	0,98	0,01	0,01	0,99	0,01	0,01	0,99	0	0,01	0,000422	0,000326	0,000237
chr2	11124593	95	G	0	0,98	0,01	0,01	0,99	0,01	0,01	0,99	0	0,01	0,000445	0,000319	0,00027
chr2	11124597	96	G	0	0,98	0,01	0,01	0,99	0,01	0,01	0,99	0	0,01	0,000377	0,000271	0,00022
chr2	11124598	97	G	0	0,98	0,01	0,01	0,88	0,06	0,06	0,99	0	0,01	0,000432	0,000525	0,000402
chr2	11124600	98	G	0	0,98	0,01	0,01	0,99	0,01	0,01	0,99	0,01	0,01	0,000436	0,000394	0,000284
chr2	11124609	99	G	0	0,98	0,01	0,01	0,99	0,01	0,01	0,99	0	0,01	0,000466	0,000319	0,000272
chr3	89184744	100	C	0	0,99	0	0	0,99	0	0	0,99	0	0	0,00011	0,000159	0,000143
chr3	89184752	101	G	0	0,99	0,01	0,01	0,99	0	0	0,99	0	0	0,000145	0,000158	0,000137
chr3	89184753	102	C	0	0,99	0	0	0,99	0	0	0,99	0	0	0,000084	0,000121	0,000128
chr3	89184754	103	C	0	0,99	0	0	0,99	0	0	0,99	0	0	0,000068	0,000071	0,000073
chr3	89184756	104	C	0	0,99	0	0	0,99	0	0	0,99	0	0	0,000064	0,000082	0,000081
chr3	89184758	105	G	0	0,99	0	0	0,95	0,03	0,03	0,99	0	0	0,000112	0,000863	0,000845
chr3	89184759	106	G	0	0,99	0	0	0,99	0	0	0,99	0	0	0,000093	0,000094	0,000078
chr3	89184760	107	G	0	0,99	0,01	0,01	0,99	0,01	0	0,99	0	0,01	0,000157	0,000169	0,000132
chr3	89184762	108	C	0	0,99	0	0	0,99	0	0	0,99	0	0	0,000074	0,000088	0,000085
chr3	89184763	109	C	0	0,99	0	0	0,99	0	0	0,99	0	0	0,000118	0,000106	0,000099
chr3	89184765	110	G	0	0,99	0	0	0,99	0	0	0,99	0	0	0,000086	0,000183	0,000186
chr3	89184768	111	G	0	0,99	0	0	0,99	0	0	0,99	0	0	0,0001	0,000088	0,000074
chr3	89184769	112	G	0	0,99	0	0	0,99	0	0	0,99	0	0	0,000094	0,000114	0,000113
chr3	89184773	113	G	0	0,99	0	0	0,99	0	0	0,99	0	0	0,000078	0,000076	0,000081
chr3	89184774	114	C	0	0,99	0	0	0,99	0	0,01	0,99	0	0	0,000141	0,000568	0,000572
chr3	89184776	115	C	1	0,17	0,8	0,02	0,16	0,73	0,1	0,37	0,56	0,07	0,151685	0,459911	0,466219
chr3	89184777	116	G	1	0,2	0,75	0,06	0,23	0,75	0,02	0,41	0,58	0,01	0,064331	0,258897	0,06027
chr3	89184778	117	G	0	0,99	0,01	0	0,99	0	0	0,99	0	0	0,000165	0,00041	0,000362
chr3	89184779	118	G	0	0,99	0,01	0	0,99	0	0,01	0,99	0,01	0	0,000225	0,000146	0,000205
chr3	89184780	119	C	0	0,99	0,01	0	0,99	0	0,01	0,99	0,01	0	0,0002	0,000174	0,000201
chr3	89184781	120	C	0	0,99	0	0	0,99	0,01	0	0,99	0,01	0	0,000149	0,000698	0,000691
chr3	89184782	121	C	0	0,99	0	0	0,99	0,01	0,01	0,99	0,01	0	0,000158	0,000141	0,000164
chr3	89184783	122	C	0	0,99	0	0	0,99	0,01	0,01	0,99	0,01	0	0,000212	0,000367	0,00056
chr3	89184787	123	G	0	0,99	0	0	0,99	0	0	0,99	0	0	0,0001	0,000107	0,000113
chr3	89184788	124	G	0	0,99	0	0,01	0,99	0,01	0	0,99	0,01	0	0,000132	0,000172	0,000136
chr3	89184792	125	G	0	0,99	0,01	0,01	0,8	0,1	0,1	0,99	0	0,01	0,000186	0,000263	0,000234
chr3	89184795	126	G	0	0,99	0	0	0,99	0	0,01	0,99	0,01	0	0,000217	0,000205	0,000329
chr3	89184800	127	G	0	0,99	0	0	0,99	0	0	0,99	0	0	0,000126	0,00013	0,00012
chr3	89184801	128	C	0	0,99	0	0	0,99	0	0,01	0,99	0	0	0,000127	0,000224	0,000248
chr3	89184805	129	C	0	0,99	0	0	0,99	0	0	0,99	0	0	0,000086	0,0001	0,000088
chr3	89184806	130	C	1	0,09	0,87	0,04	0,1	0,84	0,06	0,18	0,8	0,02	0,047226	0,046535	0,057965

chr3	89184807	131	G	1	0,13	0,85	0,01	0,07	0,87	0,05	0,2	0,79	0,01	0,057434	0,028832	0,191881
chr3	89184808	132	G	0	0,99	0	0	0,99	0,01	0	0,99	0	0	0,000174	0,000112	0,000177
chr3	89184809	133	G	0	0,99	0,01	0,01	0,98	0,01	0,01	0,99	0,01	0,01	0,000203	0,000352	0,00033
chr3	89184810	134	G	0	0,99	0,01	0	0,99	0,01	0,01	0,99	0,01	0	0,000272	0,000186	0,000256
chr3	89184814	135	G	0	0,99	0	0	0,99	0,01	0	0,99	0	0	0,000127	0,000119	0,000131
chr3	89184815	136	C	1	0,04	0,93	0,03	0,08	0,9	0,02	0,12	0,84	0,04	0,017221	0,028211	0,024104
chr3	89184816	137	G	1	0,02	0,95	0,02	0,05	0,93	0,02	0,12	0,87	0,02	0,007883	0,042818	0,027074
chr3	89184817	138	G	0	0,99	0,01	0,01	0,99	0,01	0	0,99	0,01	0	0,000172	0,000217	0,000198
chr3	89184818	139	C	0	0,99	0	0,01	0,99	0,01	0	0,99	0	0	0,000181	0,000215	0,00017
chr3	89184820	140	G	0	0,99	0	0	0,99	0	0,01	0,99	0	0	0,000184	0,000132	0,000193
chr3	89184821	141	C	1	0,08	0,82	0,1	0,17	0,82	0,01	0,22	0,67	0,11	0,200176	0,262842	0,367279
chr3	89184822	142	G	1	0,13	0,86	0,01	0,1	0,88	0,03	0,2	0,79	0,02	0,02954	0,021329	0,06673
chr3	89184824	143	C	1	0,09	0,74	0,17	0,12	0,78	0,11	0,32	0,67	0,02	0,178736	2,267969	0,416038
chr3	89184825	144	G	1	0,08	0,87	0,05	0,14	0,83	0,03	0,2	0,79	0,01	0,055621	0,0947	0,032618
chr3	89184826	145	G	0	0,99	0,01	0	0,99	0	0	0,99	0,01	0,01	0,000195	0,000198	0,000164
chr3	89184827	146	G	0	0,99	0	0	0,99	0	0	0,99	0,01	0	0,000159	0,000181	0,00018
chr3	89184828	147	C	0	0,99	0	0	0,99	0	0,01	0,99	0	0	0,000147	0,000155	0,000141
chr3	89184831	148	C	0	0,99	0	0,01	0,99	0	0	0,99	0	0	0,000183	0,000844	0,000578
chr3	89184833	149	G	0	0,99	0	0	0,99	0	0,01	0,99	0,01	0	0,000143	0,000141	0,000146
chr3	89184834	150	G	0	0,98	0,01	0,01	0,99	0	0,01	0,99	0	0	0,000329	0,000234	0,000223
chr3	89184836	151	C	1	0,11	0,86	0,03	0,19	0,78	0,04	0,2	0,74	0,06	0,029061	0,103387	0,073742
chr3	89184837	152	G	1	0,1	0,84	0,06	0,11	0,82	0,07	0,24	0,75	0,01	0,071773	0,150464	0,135065
chr3	89184839	153	G	0	0,99	0	0	0,99	0	0	0,99	0	0	0,000127	0,000132	0,000118
chr3	89184840	154	G	0	0,99	0,01	0	0,99	0	0	0,99	0,01	0	0,000156	0,000556	0,000532
chr3	89184842	155	G	0	0,99	0,01	0	0,99	0,01	0	0,99	0,01	0	0,000175	0,000212	0,00017
chr3	89184843	156	G	0	0,99	0	0	0,99	0	0,01	0,99	0,01	0	0,000134	0,000292	0,000318
chr3	89184844	157	C	1	0,04	0,94	0,01	0,05	0,91	0,04	0,09	0,86	0,05	0,015295	0,199473	0,065729
chr3	89184845	158	G	1	0,04	0,95	0,01	0,06	0,92	0,02	0,12	0,77	0,11	0,006624	0,075089	0,078766
chr3	89184846	159	G	0	0,99	0	0	0,99	0,01	0	0,99	0	0,01	0,000138	0,000138	0,000149
chr3	89184848	160	G	0	0,99	0,01	0,01	0,99	0,01	0,01	0,99	0,01	0	0,00027	0,000318	0,000286
chr3	89184849	161	G	0	0,99	0	0	0,99	0,01	0,01	0,99	0	0	0,000202	0,000213	0,000337
chr3	89184850	162	C	1	0,04	0,93	0,03	0,09	0,88	0,03	0,12	0,82	0,06	0,026569	0,047384	0,05528
chr3	89184851	163	G	1	0,06	0,87	0,07	0,05	0,91	0,03	0,14	0,85	0,01	0,029854	0,067965	0,03066
chr3	89184852	164	G	0	0,99	0	0,01	0,99	0,01	0	0,98	0,01	0,01	0,00017	0,000344	0,00035
chr3	89184853	165	C	1	0,04	0,94	0,02	0,06	0,92	0,01	0,11	0,82	0,07	0,012067	0,053864	0,050413
chr3	89184854	166	G	1	0,04	0,86	0,11	0,03	0,89	0,08	0,12	0,87	0,01	0,034467	0,241	0,064297
chr3	89184855	167	G	0	0,99	0	0	0,99	0,01	0,01	0,99	0	0	0,000257	0,000143	0,000235
chr3	89184856	168	C	1	0,05	0,92	0,03	0,06	0,92	0,02	0,1	0,85	0,05	0,015175	0,048277	0,048116
chr3	89184857	169	G	1	0,02	0,9	0,08	0,04	0,9	0,06	0,12	0,87	0,01	0,028212	0,163055	0,076554
chr3	89184858	170	G	0	0,99	0	0	0,99	0	0,01	0,99	0,01	0	0,000215	0,00011	0,000206
chr3	89184861	171	G	0	0,99	0	0	0,99	0,01	0	0,99	0	0	0,000182	0,000125	0,000189
chr3	89184862	172	G	0	0,99	0,01	0	0,99	0	0	0,99	0,01	0,01	0,000181	0,000205	0,000185
chr3	89184864	173	G	0	0,99	0	0	0,99	0	0,01	0,99	0	0	0,000115	0,000083	0,000088
chr3	89184865	174	G	0	0,99	0,01	0	0,99	0,01	0	0,99	0,01	0	0,000166	0,000196	0,000193
chr3	89184866	175	G	0	0,99	0,01	0	0,99	0	0	0,99	0	0	0,000136	0,000151	0,000147
chr3	89184867	176	G	0	0,99	0,01	0,01	0,99	0	0,01	0,99	0,01	0	0,000195	0,000523	0,000416

chr3	89184868	177	C	0	0,98	0,01	0,01	0,99	0,01	0,01	0,99	0,01	0,01	0,000448	0,000415	0,000363
chr3	89184870	178	C	0	0,99	0,01	0	0,99	0	0	0,99	0	0	0,000135	0,000112	0,000107
chr3	89184871	179	C	0	0,99	0	0,01	0,99	0	0,01	0,98	0,01	0	0,000143	0,000315	0,000309
chr3	89184873	180	G	0	0,99	0,01	0	0,99	0	0,01	0,99	0	0	0,000117	0,00014	0,000116
chr3	89184875	181	G	0	0,99	0	0	0,99	0,01	0	0,99	0	0	0,000211	0,00011	0,000269
chr3	89184876	182	G	0	0,99	0,01	0	0,99	0	0	0,99	0	0,01	0,000134	0,000186	0,000157
chr3	89184878	183	G	0	0,99	0	0	0,99	0	0	0,99	0	0	0,00016	0,00015	0,000153
chr3	89184879	184	G	0	0,99	0,01	0	0,96	0,02	0,02	0,99	0	0	0,00015	0,00017	0,000113
chr3	89184880	185	G	0	0,99	0,01	0	0,99	0,01	0	0,99	0,01	0	0,000168	0,000166	0,000169
chr3	89184881	186	C	0	0,99	0,01	0	0,99	0,01	0	0,99	0,01	0,01	0,000183	0,000267	0,000266
chr3	89184883	187	G	0	0,99	0	0	0,99	0	0	0,99	0	0	0,000108	0,000437	0,000428
chr3	89184885	188	C	0	0,99	0,01	0	0,99	0,01	0	0,99	0	0	0,000142	0,000165	0,000115
chr3	89184888	189	C	0	0,99	0	0	0,99	0,01	0	0,99	0	0,01	0,000142	0,000113	0,000175
chr3	89184890	190	C	0	0,99	0	0	0,99	0	0,01	0,99	0,01	0	0,000179	0,000309	0,000404
chr3	89184891	191	C	1	0,31	0,59	0,1	0,4	0,6	0,01	0,57	0,42	0,01	0,197435	0,36744	0,026778
chr3	89184892	192	G	1	0,34	0,62	0,04	0,4	0,54	0,05	0,49	0,5	0,01	0,079375	0,050031	0,052668
chr3	89184895	193	G	0	0,99	0,01	0	0,99	0	0,01	0,99	0,01	0	0,000146	0,00016	0,000126
chr3	89184896	194	G	0	0,99	0	0	0,99	0	0	0,99	0	0	0,00012	0,000132	0,00012
chr3	89184897	195	G	0	0,99	0,01	0	0,99	0,01	0	0,99	0,01	0	0,000175	0,000218	0,000207
chr3	89184898	196	C	0	0,99	0	0	0,99	0	0	0,99	0	0,01	0,000104	0,000334	0,000326
chr3	89184902	197	C	0	0,99	0,01	0	0,99	0,01	0,01	0,99	0	0	0,000173	0,000269	0,000277
chr3	89184903	198	C	0	0,99	0,01	0	0,99	0	0	0,99	0	0	0,000147	0,000167	0,000127
chr3	89184905	199	G	0	0,99	0	0	0,99	0	0	0,99	0	0	0,000098	0,000127	0,000102
chr3	89184907	200	G	0	0,99	0	0	0,99	0,01	0	0,99	0	0	0,000165	0,000125	0,000156
chr3	89184908	201	G	0	0,99	0	0	0,99	0	0	0,99	0	0,01	0,000153	0,000164	0,000178
chr3	89184911	202	G	0	0,99	0	0	0,99	0	0,01	0,99	0	0	0,000087	0,000086	0,000089
chr3	89184913	203	G	0	0,99	0,01	0	0,99	0	0,01	0,99	0	0	0,000179	0,000182	0,000159
chr3	89184914	204	G	0	0,99	0	0	0,99	0,01	0	0,99	0,01	0,01	0,000161	0,000181	0,000203
chr3	89184915	205	C	0	0,99	0,01	0	0,99	0	0,01	0,99	0,01	0,01	0,000161	0,000142	0,000129
chr3	89184917	206	G	0	0,99	0	0	0,98	0,01	0,01	0,98	0,01	0,01	0,0003	0,000428	0,000711
chr3	89184919	207	G	0	0,99	0	0	0,99	0	0	0,99	0	0	0,000138	0,000151	0,00015
chr3	89184920	208	G	0	0,98	0	0,01	0,99	0	0	0,99	0	0	0,000279	0,00062	0,000449
chr3	89184922	209	G	0	0,99	0	0	0,99	0	0	0,98	0,01	0	0,000141	0,000323	0,000312
chr3	89184923	210	G	0	0,99	0	0,01	0,99	0	0	0,99	0	0	0,000146	0,000142	0,000106
chr3	89184925	211	G	0	0,99	0	0	0,97	0,03	0	0,98	0	0,01	0,000544	0,000391	0,001812
chr3	89184926	212	G	0	0,99	0	0	0,99	0,01	0	0,99	0	0	0,000148	0,00016	0,000168
chr3	89184927	213	C	0	0,99	0	0	0,99	0	0	0,99	0,01	0	0,000129	0,000207	0,0002
chr3	89184929	214	G	0	0,99	0,01	0	0,99	0,01	0	0,99	0	0	0,000201	0,000136	0,000212
chr3	89184931	215	G	0	0,99	0	0	0,99	0	0	0,99	0	0	0,00013	0,000111	0,000127
chr3	89184932	216	G	0	0,99	0	0,01	0,99	0	0	0,99	0,01	0	0,000155	0,00028	0,000258
chr3	89184933	217	C	0	0,99	0	0	0,99	0	0,01	0,99	0	0	0,000137	0,000273	0,000367
chr3	89184938	218	G	0	0,99	0,01	0	0,99	0	0,01	0,99	0,01	0	0,000225	0,00026	0,000274
chr3	89184939	219	C	0	0,99	0	0	0,99	0	0	0,99	0	0,01	0,000104	0,000202	0,000221
chr3	89184940	220	C	0	0,99	0	0	0,99	0	0,01	0,99	0	0	0,000198	0,000133	0,000241
chr3	89184942	221	C	0	0,99	0	0	0,99	0	0	0,99	0	0	0,000154	0,000108	0,000112
chr3	89184943	222	C	0	0,99	0	0	0,99	0	0	0,99	0	0	0,000103	0,000104	0,000117

chr3	89184945	223	G	0	0,99	0	0	0,99	0,01	0	0,99	0	0	0,000118	0,000145	0,000141
chr3	89184946	224	G	0	0,99	0	0	0,99	0,01	0	0,99	0	0	0,000148	0,000167	0,000158
chr3	89184949	225	C	0	0,99	0	0	0,99	0	0	0,99	0	0,01	0,000107	0,000185	0,000175
chr3	89184951	226	G	0	0,99	0	0,01	0,99	0	0,01	0,99	0	0	0,000254	0,000189	0,000236
chr3	89184953	227	C	1	0,06	0,86	0,08	0,13	0,85	0,01	0,2	0,65	0,15	0,092558	0,540625	1,441423
chr3	89184954	228	G	1	0,08	0,87	0,06	0,09	0,83	0,08	0,36	0,64	0,01	0,074798	1,832916	1,309632
chr3	89184955	229	G	0	0,99	0	0	0,99	0,01	0	0,99	0	0,01	0,000168	0,000177	0,000255
chr3	89184956	230	G	0	0,99	0	0	0,99	0,01	0	0,99	0,01	0,01	0,000142	0,000295	0,000272
chr3	89184957	231	C	0	0,99	0,01	0	0,99	0,01	0	0,99	0,01	0	0,000168	0,000218	0,000205
chr3	89184959	232	C	1	0,04	0,94	0,02	0,07	0,85	0,08	0,12	0,84	0,04	0,040303	0,023592	0,063635
chr3	89184960	233	G	1	0,05	0,91	0,04	0,07	0,88	0,05	0,2	0,77	0,03	0,02762	0,141742	0,127921
chr3	89184961	234	C	0	0,98	0,01	0,01	0,99	0	0	0,99	0	0,01	0,000252	0,000315	0,000225
chr3	89184963	235	C	0	0,99	0,01	0,01	0,99	0	0	0,99	0	0	0,000149	0,000153	0,000112
chr3	89184965	236	C	0	0,99	0	0	0,98	0,01	0,01	0,99	0	0,01	0,000164	0,000144	0,00025
chr3	89184967	237	G	0	0,99	0	0,01	0,99	0,01	0	0,99	0	0	0,000146	0,000102	0,000105
chr3	89184969	238	C	0	0,99	0	0	0,99	0	0	0,99	0	0	0,000109	0,00012	0,000109
chr3	89184972	239	C	0	0,99	0,01	0	0,99	0,01	0	0,99	0,01	0,01	0,000211	0,000264	0,00026
chr3	89184973	240	C	0	0,99	0	0	0,99	0	0	0,99	0,01	0	0,000141	0,000149	0,000149
chr3	89184975	241	C	0	0,99	0,01	0	0,99	0	0	0,99	0,01	0,01	0,000156	0,000172	0,000122
chr3	89184978	242	C	1	0,03	0,95	0,03	0,05	0,93	0,02	0,08	0,88	0,04	0,009196	0,02832	0,025196
chr3	89184979	243	G	1	0,04	0,93	0,02	0,08	0,9	0,01	0,16	0,82	0,02	0,013635	0,044135	0,022289
chr3	89184980	244	G	0	0,99	0	0	0,99	0	0	0,99	0	0	0,000106	0,000115	0,000091
chr3	89184981	245	G	0	0,99	0	0,01	0,99	0,01	0,01	0,99	0,01	0	0,000229	0,000195	0,000272
chr3	89184982	246	G	0	0,99	0,01	0	0,99	0	0	0,99	0	0	0,00015	0,000151	0,000137
chr3	89184983	247	C	0	0,99	0	0	0,99	0,01	0	0,99	0,01	0	0,000134	0,000142	0,000155
chr3	89184985	248	G	0	0,99	0	0	0,99	0	0	0,99	0	0	0,000115	0,000236	0,000233
chr3	89184986	249	G	0	0,99	0	0	0,99	0	0,01	0,99	0	0	0,0001	0,000105	0,000108
chr3	89184987	250	G	0	0,99	0	0	0,99	0	0	0,99	0	0	0,000078	0,000079	0,000078
chr3	89184988	251	G	0	0,99	0	0	0,99	0	0	0,99	0	0	0,000071	0,000069	0,000071
chr3	89184989	252	G	0	0,99	0	0	0,99	0	0	0,99	0	0	0,000079	0,000069	0,000082
chr3	89184990	253	C	0	0,99	0	0	0,99	0	0	0,99	0,01	0	0,000135	0,000259	0,000228
chr3	89184993	254	C	0	0,99	0,01	0	0,99	0	0	0,99	0	0	0,000124	0,000186	0,00014
chr3	89184994	255	C	0	0,99	0	0,01	0,99	0	0	0,99	0	0	0,000154	0,000143	0,000107
chr3	89184996	256	G	0	0,99	0	0	0,99	0	0	0,99	0	0	0,0001	0,000106	0,000106
chr3	89184997	257	C	0	0,99	0	0	0,99	0	0	0,99	0	0	0,000101	0,000134	0,000123
chr3	89184998	258	C	0	0,99	0	0	0,99	0	0,01	0,99	0	0	0,000081	0,000096	0,0001
chr3	89184999	259	C	0	0,99	0	0	0,99	0	0	0,99	0	0	0,000095	0,000094	0,000091
chr3	89185001	260	C	0	0,99	0	0	0,99	0	0	0,99	0	0	0,00009	0,00009	0,000093
chr3	89185003	261	G	0	0,99	0	0	0,99	0	0	0,99	0	0	0,000121	0,000144	0,000138
chr3	89185004	262	C	0	0,99	0	0	0,99	0	0,01	0,99	0	0	0,000126	0,000125	0,000128
chr3	89185005	263	C	0	0,99	0	0	0,99	0	0,01	0,99	0	0	0,000102	0,000106	0,000119
chr3	89185008	264	G	0	0,99	0	0,01	0,99	0,01	0,01	0,99	0,01	0	0,000177	0,000163	0,000169
chr3	89185015	265	C	0	0,99	0	0	0,99	0	0	0,99	0	0	0,000105	0,000131	0,000118
chr3	89185017	266	C	0	0,99	0	0	0,99	0	0	0,99	0	0	0,0001	0,000084	0,000096
chr3	89185018	267	C	0	0,99	0	0	0,99	0	0	0,99	0	0	0,000105	0,000082	0,000093
chr3	89185022	268	C	0	0,99	0	0	0,99	0,01	0,01	0,99	0	0	0,000087	0,000077	0,000086

chr3	89679038	269	G	0	0,99	0	0	0,99	0	0	0,99	0	0	0,000096	0,0001	0,000094
chr3	89679041	270	G	0	0,99	0	0	0,99	0	0	0,99	0	0	0,0001	0,000106	0,000089
chr3	89679051	271	G	0	0,99	0	0	0,99	0	0	0,99	0	0	0,000082	0,000075	0,000075
chr3	89679054	272	G	0	0,99	0	0	0,99	0	0	0,99	0	0	0,000112	0,00013	0,000116
chr3	89679072	273	G	1	0,07	0,92	0,02	0,1	0,85	0,05	0,49	0,51	0,01	0,035189	28,511059	3,729104
chr3	89679075	274	G	0	0,99	0,01	0	0,99	0,01	0	0,99	0,01	0	0,000178	0,000181	0,000192
chr3	89679079	275	G	0	0,99	0,01	0	0,99	0	0,01	0,99	0,01	0	0,000189	0,000172	0,000201
chr3	89679081	276	G	0	0,99	0,01	0	0,99	0,01	0,01	0,99	0,01	0	0,000178	0,00014	0,000165
chr3	89679091	277	G	0	0,99	0,01	0	0,99	0,01	0	0,99	0	0,01	0,000124	0,000456	0,000429
chr3	89679103	278	G	0	0,99	0,01	0	0,99	0	0	0,99	0,01	0	0,000121	0,000137	0,000119
chr3	89679107	279	G	1	0,04	0,9	0,06	0,27	0,71	0,02	0,7	0,29	0,01	0,514081	inf	586,723162
chr3	89679108	280	G	0	0,99	0	0	0,99	0	0,01	0,99	0	0	0,000144	0,000137	0,000161
chr3	89679109	281	G	0	0,99	0,01	0	0,99	0	0	0,99	0	0	0,000133	0,000222	0,000196
chr3	89679117	282	C	0	0,99	0	0	0,99	0	0	0,99	0	0	0,00005	0,000049	0,000056
chr3	89679123	283	G	0	0,99	0,01	0	0,99	0	0,01	0,99	0	0	0,000174	0,000139	0,000161
chr3	89679126	284	C	0	0,99	0	0	0,99	0	0	0,99	0	0	0,000046	0,000046	0,000045
chr3	89679132	285	C	0	0,99	0	0	1	0	0	0,99	0	0	0,000042	0,000057	0,000059
chr3	89679136	286	G	0	0,99	0,01	0,01	0,99	0	0,01	0,99	0	0	0,000217	0,000195	0,000159
chr3	89679137	287	C	0	0,99	0	0	0,99	0	0	0,99	0	0	0,000064	0,000062	0,000059
chr3	89679141	288	C	0	0,99	0	0	0,99	0	0	0,99	0	0	0,000042	0,000048	0,000047
chr3	89679142	289	C	0	0,99	0,01	0,01	0,99	0	0	0,99	0,01	0	0,00018	0,000179	0,00013
chr3	89679144	290	G	0	0,99	0	0,01	0,99	0	0	0,99	0	0	0,000127	0,00017	0,000122
chr3	89679146	291	C	0	0,99	0,01	0	0,99	0,01	0	0,99	0	0	0,000154	0,000242	0,000221
chr3	89679148	292	G	0	0,99	0	0	0,99	0	0	0,99	0	0	0,0001	0,000098	0,000078
chr3	89679149	293	G	0	0,99	0	0	0,99	0	0	0,99	0	0	0,000112	0,000126	0,000119
chr3	89679150	294	C	0	0,99	0	0	0,99	0,01	0	0,99	0,01	0	0,000156	0,000325	0,000353
chr3	89679155	295	C	1	0,04	0,63	0,34	0,5	0,49	0	0,88	0,11	0,01	2621,460021	7,68682E+59	0,206502
chr3	89679156	296	G	1	0,01	0,79	0,19	0,44	0,56	0,01	0,85	0,15	0	1,22958E+18	inf	0,862736
chr3	89679158	297	C	0	0,99	0,01	0	0,99	0	0,01	0,99	0	0	0,000108	0,000106	0,000128
chr3	89679162	298	C	1	0,03	0,75	0,22	0,34	0,65	0,01	0,72	0,27	0,01	35,135826	1108421,096	0,278169
chr3	89679163	299	G	1	0,02	0,84	0,15	0,39	0,6	0,01	0,81	0,19	0,01	13,429348	inf	1,72039
chr3	89679164	300	C	0	0,99	0,01	0	0,99	0,01	0	0,99	0	0,01	0,000139	0,000164	0,000178
chr3	89679165	301	C	0	0,99	0	0,01	0,99	0	0	0,99	0	0	0,000156	0,000156	0,000126
chr3	89679167	302	C	0	0,99	0,01	0	0,99	0	0	0,99	0	0	0,000098	0,000111	0,000088
chr3	89679168	303	C	0	0,99	0,01	0,01	0,99	0,01	0	0,99	0,01	0	0,000289	0,000303	0,000183
chr3	89679169	304	C	0	0,98	0,02	0	0,97	0,01	0,02	0,98	0,01	0	0,001524	0,000362	0,001444
chr3	89679171	305	C	0	0,99	0,01	0	0,98	0,01	0,01	0,99	0	0,01	0,000318	0,000157	0,000385
chr3	89679173	306	C	1	0,06	0,61	0,33	0,47	0,52	0	0,85	0,14	0,01	9522,864806	2,64925E+79	0,381374
chr3	89679174	307	G	1	0,02	0,8	0,18	0,44	0,55	0,01	0,87	0,13	0	1391838,805	inf	7,575595
chr3	89679176	308	G	0	0,99	0,01	0,01	0,98	0	0,01	0,98	0,01	0,01	0,000388	0,000494	0,000474
chr3	89679177	309	C	0	0,99	0,01	0	0,98	0,01	0,01	0,99	0,01	0	0,000658	0,000244	0,000538
chr3	89679180	310	C	0	0,99	0,01	0,01	0,99	0,01	0	0,99	0	0	0,000133	0,000158	0,000108
chr3	89679181	311	C	0	0,99	0,01	0	0,99	0,01	0	0,99	0,01	0	0,00011	0,000104	0,000109
chr3	89679182	312	C	0	0,99	0,01	0	0,99	0,01	0,01	0,99	0,01	0	0,00031	0,000139	0,000239
chr3	89679184	313	G	0	0,99	0,01	0	0,99	0	0	0,99	0,01	0,01	0,000151	0,000241	0,00021
chr3	89679186	314	C	1	0,04	0,63	0,33	0,44	0,56	0	0,85	0,15	0,01	641875,373	4,391484	1,941617

chr3	89679187	315	G	1	0,02	0,8	0,18	0,46	0,53	0,01	0,89	0,1	0	16112,76669	inf		8,903093
chr3	89679188	316	C	0	0,99	0,01	0	0,99	0,01	0	0,99	0,01	0	0,000142		0,000131	0,000163
chr3	89679190	317	G	0	0,99	0	0	0,99	0	0	0,99	0	0	0,000121		0,000119	0,000111
chr3	89679192	318	G	0	0,99	0,01	0	0,99	0	0,01	0,99	0	0	0,000175		0,000158	0,000149
chr3	89679193	319	G	0	0,99	0,01	0	0,99	0	0,01	0,99	0,01	0	0,00019		0,000169	0,000177
chr3	89679194	320	C	0	0,99	0,01	0	0,99	0,01	0	0,99	0,01	0	0,000121		0,00021	0,000202
chr3	89679196	321	C	0	0,99	0,01	0	0,99	0,01	0,01	0,99	0,01	0	0,000213		0,000131	0,000196
chr3	89679198	322	G	0	0,99	0,01	0,01	0,99	0,01	0	0,99	0	0	0,000184		0,00018	0,00016
chr3	89679199	323	C	0	0,99	0,01	0	0,93	0,03	0,04	0,99	0,01	0	0,000155		0,000129	0,000144
chr3	89679200	324	C	1	0,03	0,66	0,31	0,48	0,51	0,01	0,84	0,16	0,01	99822,90122	inf		0,307934
chr3	89679201	325	G	1	0,03	0,79	0,18	0,45	0,54	0,01	0,87	0,12	0	30,00719	inf		3,23159
chr3	89679202	326	G	0	0,99	0	0	0,88	0,05	0,07	0,99	0	0	0,000099		0,000109	0,000091
chr3	89679203	327	C	0	0,99	0,01	0	0,96	0,03	0,01	0,99	0	0	0,000127		0,000149	0,000102
chr3	89679210	328	C	0	0,99	0,01	0	0,99	0,01	0	0,99	0,01	0	0,000136		0,000403	0,00045
chr3	89679212	329	C	0	0,99	0	0	0,99	0	0	0,99	0	0	0,000049		0,000053	0,000052
chr3	89679213	330	C	0	1	0	0	1	0	0	0,99	0	0	0,00005		0,000054	0,000068
chr3	89679214	331	C	0	0,99	0	0	0,99	0	0	0,99	0	0	0,000041		0,000044	0,000044
chr3	89679216	332	G	0	0,99	0	0	0,99	0	0	0,99	0	0	0,00006		0,000063	0,000071
chr3	89679218	333	C	0	0,99	0	0	0,99	0	0	0,99	0	0	0,000049		0,000061	0,000059
chr3	89679220	334	C	0	1	0	0	0,99	0	0	0,99	0	0	0,000046		0,000047	0,00005
chr3	89679224	335	G	0	0,99	0,01	0	0,99	0,01	0	0,99	0,01	0	0,000154		0,000154	0,000152
chr3	89679226	336	G	0	0,99	0	0	0,99	0	0	0,99	0	0	0,000073		0,00009	0,000087
chr3	89679232	337	C	0	0,99	0	0	0,99	0	0	0,99	0	0	0,000049		0,000047	0,000047
chr4	44628612	338	C	0	0,99	0,01	0,01	0,99	0,01	0,01	0,99	0,01	0,01	0,00024		0,000221	0,000197
chr4	44628613	339	C	0	0,99	0,01	0,01	0,99	0,01	0	0,99	0,01	0,01	0,000214		0,000168	0,000191
chr4	44628615	340	C	0	0,99	0,01	0,01	0,99	0,01	0,01	0,99	0	0,01	0,000204		0,000197	0,000184
chr4	44628620	341	C	0	0,99	0,01	0,01	0,99	0,01	0,01	0,99	0,01	0,01	0,000241		0,00023	0,000254
chr4	44628623	342	C	0	0,99	0,01	0,01	0,99	0,01	0,01	0,99	0	0,01	0,000204		0,000177	0,000165
chr4	44628630	343	C	0	0,99	0,01	0,01	0,98	0,01	0,01	0,99	0	0,01	0,000248		0,000223	0,000227
chr4	44628631	344	C	0	0,99	0,01	0,01	0,98	0,01	0,01	0,99	0	0,01	0,000206		0,000389	0,000376
chr4	44628632	345	C	0	0,99	0,01	0,01	0,99	0,01	0,01	0,99	0,01	0,01	0,000287		0,003675	0,003732
chr4	44628640	346	C	0	0,98	0,01	0,01	0,98	0,01	0,01	0,99	0,01	0,01	0,000529		0,000407	0,000203
chr4	44628641	347	C	0	0,99	0,01	0,01	0,99	0,01	0,01	0,99	0,01	0,01	0,000334		0,000288	0,000281
chr4	44628642	348	C	0	0,99	0,01	0,01	0,99	0,01	0,01	0,99	0	0,01	0,000248		0,000175	0,000212
chr4	44628643	349	C	1	0,21	0,76	0,02	0,15	0,84	0,01	0,21	0,78	0,02	0,02379		0,025478	0,018421
chr4	44628646	350	C	1	0,09	0,89	0,02	0,04	0,95	0,01	0,04	0,96	0,01	0,009887		0,009411	0,002879
chr4	44628652	351	C	0	0,99	0,01	0,01	0,97	0,02	0,01	0,99	0,01	0,01	0,00084		0,000261	0,000662
chr4	44628657	352	C	0	0,99	0,01	0,01	0,99	0,01	0,01	0,99	0	0,01	0,00024		0,000166	0,000172
chr4	44628659	353	C	0	0,99	0,01	0,01	0,99	0	0,01	0,99	0,01	0,01	0,000237		0,000265	0,00023
chr4	44628661	354	C	0	0,99	0,01	0,01	0,99	0	0,01	0,99	0	0,01	0,000206		0,000171	0,000183
chr4	44628663	355	C	0	0,99	0,01	0,01	0,99	0,01	0,01	0,99	0,01	0,01	0,000277		0,00025	0,000189
chr4	44628665	356	C	0	0,99	0,01	0,01	0,99	0,01	0,01	0,99	0,01	0,01	0,000254		0,000246	0,000268
chr4	44628666	357	C	0	0,99	0,01	0,01	0,99	0,01	0,01	0,99	0	0,01	0,000209		0,000178	0,00016
chr4	44628667	358	C	0	0,99	0,01	0,01	0,99	0	0,01	0,99	0	0	0,000237		0,000203	0,000188
chr4	44628670	359	C	0	0,99	0,01	0,01	0,99	0	0,01	0,99	0	0,01	0,000227		0,000223	0,00018
chr4	44628671	360	C	0	0,99	0,01	0,01	0,99	0,01	0,01	0,99	0	0,01	0,000208		0,000548	0,000593

chr4	44628679	361	C	0	0,99	0,01	0,01	0,98	0,01	0,01	0,99	0,01	0,01	0,000342	0,000443	0,000544
chr4	44628680	362	C	0	0,99	0,01	0,01	0,99	0,01	0,01	0,99	0,01	0,01	0,000248	0,000383	0,000394
chr4	44628684	363	C	1	0,09	0,86	0,06	0,07	0,91	0,02	0,08	0,9	0,01	0,027503	0,025917	0,006428
chr4	44628686	364	C	0	0,99	0,01	0,01	0,99	0	0,01	0,99	0,01	0,01	0,000262	0,000266	0,000229
chr4	44628687	365	C	0	0,99	0,01	0,01	0,99	0,01	0,01	0,99	0,01	0,01	0,000317	0,000305	0,000283
chr4	44628690	366	C	0	0,99	0,01	0,01	0,99	0,01	0,01	0,99	0,01	0,01	0,000299	0,000288	0,000294
chr4	44628692	367	C	0	0,98	0,01	0,01	0,99	0,01	0,01	0,99	0	0,01	0,000336	0,00029	0,000205
chr4	44628694	368	C	0	0,99	0,01	0,01	0,99	0,01	0,01	0,99	0	0,01	0,000298	0,000259	0,000228
chr4	44628695	369	C	0	0,99	0,01	0,01	0,99	0,01	0,01	0,99	0,01	0,01	0,000279	0,00024	0,000204
chr4	44628697	370	C	0	0,99	0,01	0,01	0,99	0,01	0,01	0,99	0,01	0,01	0,00025	0,000414	0,000365
chr4	44628698	371	C	0	0,99	0,01	0,01	0,99	0,01	0	0,99	0	0,01	0,00021	0,000201	0,000194
chr4	44628700	372	C	0	0,99	0,01	0,01	0,99	0,01	0,01	0,99	0	0,01	0,000221	0,000203	0,000184
chr4	44628701	373	C	0	0,99	0,01	0,01	0,99	0,01	0,01	0,99	0,01	0,01	0,000333	0,000306	0,000358
chr4	44628702	374	C	0	0,99	0,01	0,01	0,99	0,01	0,01	0,99	0	0,01	0,000291	0,000705	0,000673
chr4	44628703	375	C	0	0,99	0,01	0,01	0,99	0,01	0,01	0,99	0,01	0,01	0,000279	0,000199	0,000253
chr4	44628706	376	C	0	0,99	0,01	0,01	0,99	0,01	0,01	0,99	0	0,01	0,000216	0,0002	0,000221
chr4	44628707	377	C	0	0,99	0,01	0,01	0,99	0	0,01	0,99	0,01	0,01	0,000224	0,000219	0,000219
chr4	44628708	378	C	0	0,99	0,01	0,01	0,99	0,01	0	0,99	0	0,01	0,000254	0,000238	0,000208
chr4	44628712	379	C	0	0,99	0,01	0,01	0,99	0,01	0,01	0,99	0	0,01	0,000199	0,000201	0,000185
chr4	44628716	380	C	0	0,99	0,01	0,01	0,99	0,01	0,01	0,99	0,01	0,01	0,0003	0,000266	0,000244
chr4	44628717	381	C	0	0,99	0,01	0,01	0,99	0	0,01	0,99	0	0	0,000246	0,000194	0,000182
chr4	44628737	382	C	0	0,99	0,01	0,01	0,99	0,01	0,01	0,99	0,01	0,01	0,000309	0,000293	0,000282
chr4	44628738	383	C	0	0,99	0,01	0,01	0,99	0,01	0,01	0,99	0	0	0,000254	0,000219	0,000178
chr4	44628740	384	C	0	0,99	0,01	0,01	0,99	0	0,01	0,99	0	0	0,000262	0,001089	0,001041
chr4	44628746	385	C	0	0,98	0,01	0,01	0,99	0,01	0,01	0,99	0,01	0,01	0,00033	0,000335	0,000222
chr4	44628750	386	C	0	0,99	0,01	0,01	0,99	0	0	0,99	0,01	0,01	0,000275	0,000305	0,000288
chr4	44628751	387	C	0	0,99	0,01	0,01	0,99	0,01	0,01	0,99	0,01	0,01	0,000306	0,000596	0,000571
chr4	44628786	388	C	0	0,98	0,01	0,01	0,97	0,01	0,01	0,98	0,01	0,01	0,00112	0,000861	0,000965
chr4	44628787	389	C	1	0,07	0,92	0,01	0,03	0,96	0,01	0,06	0,92	0,03	0,007397	0,010718	0,008837
chr4	44628795	390	C	0	0,99	0,01	0,01	0,99	0,01	0,01	0,99	0,01	0,01	0,000241	0,000305	0,000288
chr4	44628796	391	C	0	0,99	0,01	0,01	0,99	0,01	0	0,99	0,01	0,01	0,000368	0,00031	0,00031
chr4	44628797	392	C	0	0,99	0,01	0,01	0,99	0,01	0,01	0,99	0	0,01	0,000251	0,000185	0,000252
chr4	44628798	393	C	0	0,99	0,01	0,01	0,99	0	0,01	0,99	0	0,01	0,000242	0,000274	0,000223
chr4	44628799	394	C	1	0,11	0,85	0,04	0,07	0,92	0,01	0,06	0,93	0,01	0,024365	0,026215	0,007416
chr4	44628804	395	C	0	0,99	0,01	0,01	0,99	0,01	0,01	0,99	0,01	0	0,000362	0,000284	0,00033
chr4	44628807	396	C	0	0,99	0,01	0,01	0,99	0,01	0,01	0,99	0,01	0,01	0,000281	0,000213	0,000202
chr4	44628819	397	C	0	0,99	0	0,01	0,99	0	0,01	0,99	0	0,01	0,000191	0,000181	0,000187
chr4	44628821	398	C	0	0,99	0,01	0,01	0,99	0,01	0,01	0,99	0	0,01	0,000229	0,000214	0,000206
chr4	44628828	399	C	0	0,99	0,01	0,01	0,98	0	0,01	0,99	0,01	0,01	0,000335	0,00028	0,000327
chr4	44628832	400	C	1	0,04	0,95	0,01	0,02	0,97	0,01	0,03	0,93	0,04	0,002303	0,009128	0,00623
chr4	44628834	401	C	0	0,99	0,01	0,01	0,99	0,01	0,01	0,99	0,01	0,01	0,000328	0,000295	0,000304
chr4	44628843	402	C	0	0,99	0,01	0,01	0,99	0,01	0,01	0,99	0	0,01	0,000293	0,000256	0,000224
chr4	44628845	403	C	0	0,99	0,01	0,01	0,99	0	0,01	0,99	0,01	0	0,000264	0,000218	0,000184
chr4	44628851	404	C	0	0,99	0,01	0,01	0,99	0,01	0,01	0,99	0,01	0,01	0,000333	0,000256	0,000252
chr4	44628852	405	C	0	0,99	0,01	0,01	0,99	0,01	0,01	0,99	0,01	0,01	0,000284	0,000285	0,000337
chr4	44628856	406	C	0	0,99	0,01	0,01	0,99	0,01	0,01	0,98	0,01	0,01	0,000292	0,000392	0,000453

chr4	44628862	407	C	0	0,99	0,01	0,01	0,99	0,01	0	0,99	0,01	0,01	0,000245	0,000201	0,000229
chr4	44628869	408	C	0	0,99	0,01	0,01	0,98	0,01	0,01	0,99	0,01	0,01	0,000268	0,000269	0,0003
chr4	44628871	409	C	0	0,99	0,01	0,01	0,99	0,01	0,01	0,99	0,01	0,01	0,000249	0,000261	0,000301
chr4	44628876	410	C	0	0,99	0,01	0,01	0,99	0,01	0,01	0,99	0,01	0,01	0,000357	0,000277	0,00026
chr4	44628882	411	C	0	0,99	0,01	0,01	0,99	0,01	0,01	0,99	0,01	0,01	0,000345	0,000314	0,000337
chr4	44628883	412	C	0	0,99	0,01	0,01	0,98	0,01	0,01	0,99	0,01	0,01	0,000302	0,000387	0,00046
chr4	44628889	413	C	0	0,99	0,01	0,01	0,99	0,01	0,01	0,99	0,01	0,01	0,000305	0,000267	0,000297
chr4	44628890	414	C	0	0,99	0,01	0,01	0,99	0,01	0,01	0,99	0,01	0,01	0,000403	0,000301	0,000263
chr4	44628891	415	C	0	0,99	0,01	0,01	0,99	0,01	0,01	0,99	0	0	0,000287	0,000245	0,000229
chr4	44628893	416	C	0	0,99	0,01	0,01	0,99	0,01	0,01	0,99	0,01	0,01	0,000264	0,000241	0,000261
chr4	44628898	417	C	0	0,99	0,01	0,01	0,99	0,01	0,01	0,99	0	0	0,000279	0,000252	0,000257
chr4	44628906	418	C	0	0,99	0,01	0,01	0,99	0,01	0,01	0,99	0	0,01	0,00028	0,0002	0,000229
chr4	44628908	419	C	1	0,11	0,87	0,02	0,07	0,92	0,01	0,05	0,91	0,04	0,010881	0,022284	0,01216
chr4	44628911	420	C	0	0,99	0,01	0,01	0,99	0	0,01	0,99	0	0	0,000211	0,000201	0,000182
chr4	44628912	421	C	0	0,99	0	0,01	0,98	0,01	0,01	0,99	0	0	0,000209	0,00014	0,000165
chr4	44628918	422	C	0	0,99	0,01	0,01	0,99	0	0,01	0,99	0	0	0,000192	0,000155	0,00016
chr4	44628931	423	C	0	0,99	0,01	0,01	0,99	0,01	0,01	0,99	0	0,01	0,000258	0,000216	0,000196

		Posterior mean ± standard deviation		
		BS_{eff}	BS^*_{eff}	seq_{err}
DP	Replicate 1	1.0e+0 ± 1.9e-6	1.1e-2 ± 2.4e-4	1.1e-6 ± 2.1e-6
	Replicate 2	1.0e+0 ± 1.9e-6	1.1e-2 ± 2.1e-4	1.0e-6 ± 1.8e-6
	Replicate 3	1.0e+0 ± 1.5e-6	1.1e-2 ± 2.2e-4	9.2e-7 ± 1.8e-6
CD4 SP	Replicate 1	1.0e+0 ± 2.0e-6	1.1e-2 ± 2.3e-4	1.1e-6 ± 2.1e-6
	Replicate 2	1.0e+0 ± 1.6e-6	1.1e-2 ± 2.2e-4	8.8e-7 ± 1.6e-6
	Replicate 3	1.0e+0 ± 2.2e-6	1.2e-2 ± 2.3e-4	1.1e-6 ± 2.2e-6
naïve CD4	Replicate 1	1.0e+0 ± 1.5e-6	1.2e-2 ± 2.5e-4	6.8e-7 ± 1.4e-6
	Replicate 2	1.0e+0 ± 1.8e-6	1.1e-2 ± 2.1e-4	9.2e-7 ± 1.9e-6
	Replicate 3	1.0e+0 ± 1.6e-6	1.1e-2 ± 2.2e-4	8.7e-7 ± 1.7e-6

Supplemental Table 7
